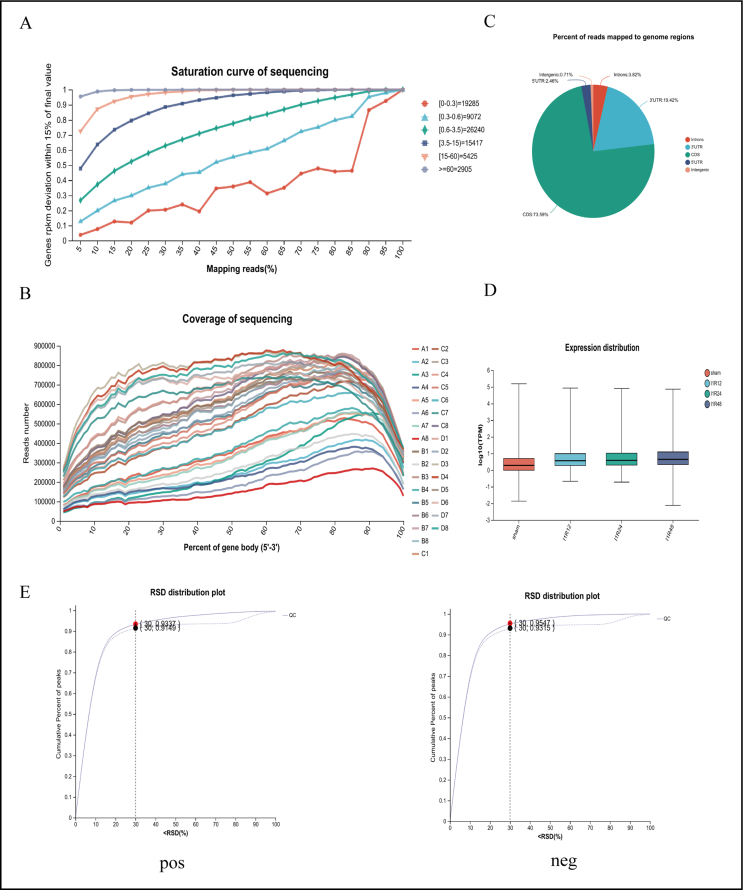# Multi-time point transcriptomics and metabolomics reveal key transcription and metabolic features of hepatic ischemia-reperfusion injury in mice

**DOI:** 10.1016/j.gendis.2024.101465

**Published:** 2024-11-17

**Authors:** Qi Li, Xiaoyan Qin, Liangxu Wang, Dingheng Hu, Rui Liao, Huarong Yu, Zhongjun Wu, Yanyao Liu

**Affiliations:** a Department of Hepatobiliary Surgery, The First Affiliated Hospital of Chongqing Medical University, Chongqing 400016, China; b Department of General Surgery and Trauma Surgery, Children’s Hospital of Chongqing Medical University, Ministry of Education Key Laboratory of Child Development and Disorders, National Clinical Research Center for Child Health and Disorders, China International Science and Technology Cooperation Base of Child Development and Critical Disorders, Chongqing Key Laboratory of Pediatrics, Chongqing 400014, China; c Research Center of Neuroscience, School of Basic Medical Sciences, Chongqing Medical University, Chongqing 400016, China

**Keywords:** Inflammation, Liver ischemia-reperfusion injury, Metabolic remodeling, Metabolomics, Transcriptomics

## Abstract

Hepatic ischemia-reperfusion injury is an unavoidable surgical complication of liver transplantation and the leading cause of poor graft function and increased mortality post-transplantation. Multiple mechanisms have been implicated in ischemia-reperfusion injury; however, the characteristic changes at the transcriptional and metabolic levels in the early, intermediate, and late phases of ischemia-reperfusion injury remain unclear. In the study, mice underwent laparotomy following anesthesia, and the blood vessels of the liver were clipped using a vascular clamp to form 70% warm ischemia of the liver. Mouse liver sections and serum samples were collected and divided into the Sham, I1R12, I1R24, and I1R48 groups. Transcriptomics and metabolomics analyses were performed to study characteristic alterations during the early, intermediate, and late phases of ischemia-reperfusion injury. Quantitative real-time PCR was used to validate the critical differentially expressed genes. The differentially expressed genes and metabolites were identified by transcriptomics and metabolomics analyses. Moreover, GO and KEGG enrichment analyses indicated that glucose metabolism remodeling, inflammatory response activation, and lipid metabolism remodeling were characteristic changes in the early, intermediate, and late phases of ischemia-reperfusion injury, respectively. In summary, our study revealed the importance of glucolipid metabolism in ischemia-reperfusion injury and provided potential therapeutic intervention targets and a new perspective to explore the underlying mechanisms of ischemia-reperfusion injury.

## Introduction

Liver transplantation is an effective treatment for end-stage liver disease.^1^ However, ischemia-reperfusion injury (IRI) is a common and unavoidable surgical complication of liver transplantation, which can result in impaired liver function and even post-transplant liver failure.^2^ Previous studies have shown that the inflammatory cascade response and cytokines play vital roles in the mechanism of hepatic IRI, and metabolic stress following metabolic homeostasis disruption in the liver influences the pathogenesis and pathological process of hepatic IRI by stimulating reactive oxygen species overproduction and sterile inflammation.^3^^,^^4^ However, the intrahepatic inflammatory microenvironment and metabolite changes caused by IRI are still undefined; thus, the characteristic variations in the transcription and metabolite levels in the early, intermediate, and late phases of hepatic IRI require further research.

Transcriptomics technology is used as a medical tool to explore the underlying mechanisms in IRI research. For example, through transcriptomics, hepatic metabolic remodeling, including lipid/fatty acid and 5-aminolevulinate (5-ALA) metabolisms, has shown its significance in IRI and could be a targeted therapeutic intervention.^5^ Tripartite motif-containing 27 (TRIM27), a critical mediator of inflammation, has been revealed by transcriptomics to negatively regulate inflammation via suppressing the NF-κB and MAPK signaling pathways during hepatic IRI and is expected to be a promising treatment to attenuate hepatic IRI.^6^

Moreover, metabolomic technology has also been employed to study the mechanism of hepatic IRI. Metabolomics was used to investigate the impact of glucose metabolism-related genes on hepatic IRI and showed that insulin-induced gene 2 (INSIG2) could reduce hepatic IRI by triggering the downstream pentose phosphate pathway to reprogram glucose metabolism.^7^ A recent study applied metabolomics to demonstrate that oxidized lipid metabolites markedly increased during hepatic IRI and lipid peroxidation, partially caused by nicotinamide adenine dinucleotide deprivation, and could aggravate hepatic IRI.^8^

Here, we established mouse models of liver IRI and investigated the characteristic alterations of transcriptome and metabolome levels of mouse liver in the early, intermediate, and late phases of IRI with transcriptomics and metabolomics. Additionally, we explored the effects of these changes on hepatic IRI during different periods. Finally, our study offers a novel perspective for exploring the occurrence and development of hepatic IRI by combining transcriptomics and metabolomics analyses.

## Materials and methods

### Ethical approval

All animal procedures and experiments were approved by the Institutional Animal Care and Use Committee of Chongqing Medical University. Food and water were provided *ad libitum*, and a normative environment with standard temperature and humidity was maintained.

### Animal model

Following anesthesia, the mice underwent laparotomy, and the blood vessels of the left and middle liver lobes were clipped with a vascular clamp to form 70% warm ischemia of the liver. After ischemia for 1 h, the clamp was removed and kept for reperfusion for 12, 24, and 48 h^9^^,^^10^. Mice in the Sham group were subjected to the same procedure, but the blood vessels were not clipped. All 32 mouse liver samples were obtained and divided into the Sham, I1R12, I1R24, and I1R48 groups (*n* = 8 per group).

### Hematoxylin-eosin staining

After the mice were sacrificed, liver samples were sectioned and fixed with paraformaldehyde. Subsequently, the liver sections were dehydrated using a gradient series of alcohol and embedded in paraffin wax. Liver sections of mice from the four groups were stained with hematoxylin and eosin and then observed under a 200X or 400X light microscope.

### Serum levels of hepatic enzyme

Blood samples were collected from the four groups of mice, and an ELISA kit (Nanjing Jiancheng, Nanjing, China) was used to measure the serum concentrations of aspartate aminotransferase and alanine aminotransferase. IRI in mouse liver samples was evaluated by calculating Suzuki’s score in a blinded manner.^11^

### Quantitative real-time PCR

Mouse liver tissue samples were preserved at −80 °C until mRNA was extracted. Furthermore, quantitative real-time PCR with cDNA as a template and β-actin as an internal reference was conducted to analyze the relative mRNA expression of several biomarkers we selected. The primer sequences are listed in Table 1.

**Table 1:** The sequences.

The primer	The sequence (5′-3′)	The sequence (3′-5′)
PKG1	CCACAGAAGGCTGGTGGATT	GTCTGCAACTTTAGCGCCTC
GcK	CCCAGTCGTTGACTCTGGTAG	CTTCTGAGCCTTCTGGGGTG
LDHA	AACTTGGCGCTCTACTTGCT	GGACTTTGAATCTTTTGAGACCTTG
PI3K	CCACCTCTTTGCCCTGAT	TCGGTTCTTTCCCGTTAG
AKT1	CCGCCTGATCAAGTTCTCCT	GATGATCCATGCGGGGCTT
4E-BP1	ACTCACCTGTGGCCAAAACA	TTGTGACTCTTCACCGCCTG
ALDOA	AACCCAGCTGAATAGGCTGC	CATGGGTCACCTTGCCTGG
TIMP-1	AGCCTGGAGGCAGTGATTTC	GGCCATCATGGTATCTGCTCT
STAT3	TACACCAAGCAGCAGCTGAA	TACGGGGCAGCACTACCT
PLA2	AACACCTCCGCTAAGAACCC	GCAGCCGTAGAAGCCATAGT
PLAAT3	GGAGAAAAGGAGCCAGGGG	GCTTGGGTTCTGGTATGGGT

### Cell culture and hypoxia/reoxygenation model

AML12 cells were purchased from Procell (Wuhan, China) and cultured in DMEM/F12 (Gibco, USA) with 10% fetal bovine serum (Procell), 40 ng/mL dexamethasone and 0.5% insulin-transferrin-selenium (Procell, PB180429). The AML12 cells were incubated in a tri-gas incubator to hypoxia for 12 h (94% N_2_, 5% CO_2_, and 1% O_2_) and transferred to 5% CO_2_ typical incubator for reoxygenation for 12, 24, and 48 h in medium without fetal bovine serum.

### Western blots

The proteins of the tissues and cells were extracted by lysis buffer and the protein concentration was measured using a BCA protein assay kit. The proteins were separated by SDS-PAGE and transferred to PVDF membranes, which then were blocked with NcmBlot blocking buffer for 30 min. The membranes were incubated overnight at 4 °C with primary antibodies and 1 h at room temperature with horseradish peroxidase-conjugated goat-anti-rabbit or goat-anti-mouse antibodies. The blots were visualized by the FUSION Solo S system. The primary antibodies were used in this study included PGK1 (1:1000; Beyotime, China; AF1825), GCK (1:1000; Beyotime; AF6973), LDHA (1:1000; Beyotime; AF0216), PI3K (1:1000; CST, USA; 4249T), AKT1 (1:1000; Beyotime; AF1777), 4EBP1 (1:1000; Beyotime; AF5159), ALDOA (1:1000; Beyotime; AF6189), TIMP1 (1:1000; Beyotime; AF8163), STAT3 (1:1000; Beyotime; AF1492), P-AKT (1:1000; CST; 9271T), P-PI3K (1:1000; ImmunoWay, UK; YP0224), mTOR (1:1000; CST; 2983T), β-actin (1:1500; Servicebio, China; GB15003-100).

### Detection of differentially expressed metabolites (DEMs)

ELISA assays (RUIXIN, China) were used to calculate the concentration of prostaglandin F1 alpha (PGF1α) and the free fatty acid content in the cell culture supernatant of mouse liver tissue samples according to the manufacturer’s instructions. The hydroxyproline content of mouse liver tissue samples was detected by a hydroxyproline content assay kit (Solarbio, BC0255) according to the manufacturer’s instructions.

### Differential expression and functional enrichment analysis

Differentially expressed genes (DEGs) between two samples were identified by calculating the expression level based on the transcripts per million. Furthermore, we quantified gene abundances using RNA-seq by expectation maximization.^12^ Differential expression analysis was conducted via DESeq2.^13^ Significant DEGs were identified depending on the criteria |log_2_fold change| ≥ 1 and false discovery rate <0.05. The Kyoto Encyclopedia of Genes and Genomes (KEGG) and Gene Ontology (GO) enrichment analyses were conducted by Goatools and Python SciPy, respectively, for function and pathway enrichment analysis of DEGs. The above data were analyzed on the Majorbio Cloud Platform.

### Liquid chromatography coupled with mass spectrometry and function enrichment analysis

The analysis of mouse liver samples with liquid chromatography coupled with mass spectrometry was conducted on a Thermo UHPLC-Q Exactive HF-X system. After the addition of 6 mm diameter grinding beads, 50 mg of solid samples were ground and then centrifuged at 13,000 *g* and 4 °C for 15 min. The supernatant was transferred for analysis with liquid chromatography coupled with mass spectrometry. Progenesis QI software pretreated the raw data. The metabolites were identified by the Human Metabolome Database (HMDB), Metlin, and Majorbio databases. The data matrix from the search database was uploaded to the Majorbio Cloud platform for analysis. Significant DEMs were identified based on variable importance in projection >1 and *P* < 0.05. DEMs were sorted into the corresponding biochemical pathways via the KEGG pathway enrichment analysis. Metabolic compound identification was performed using the HMDB and KEGG compound databases. Simultaneously, integrated pathway analysis was conducted using the iPath database version 3.

### Statistical analysis

All data were analyzed using SPSS 22.0 software and presented as mean ± standard error of the mean. The study employed the student’s *t*-test to analyze significant differences between two groups, while one-way analysis of variance (ANOVA) was used for the analysis among three or more groups. Statistical significance was determined at *P* values < 0.05.

## Results

### Hepatic IRI model

**Figure 1:**
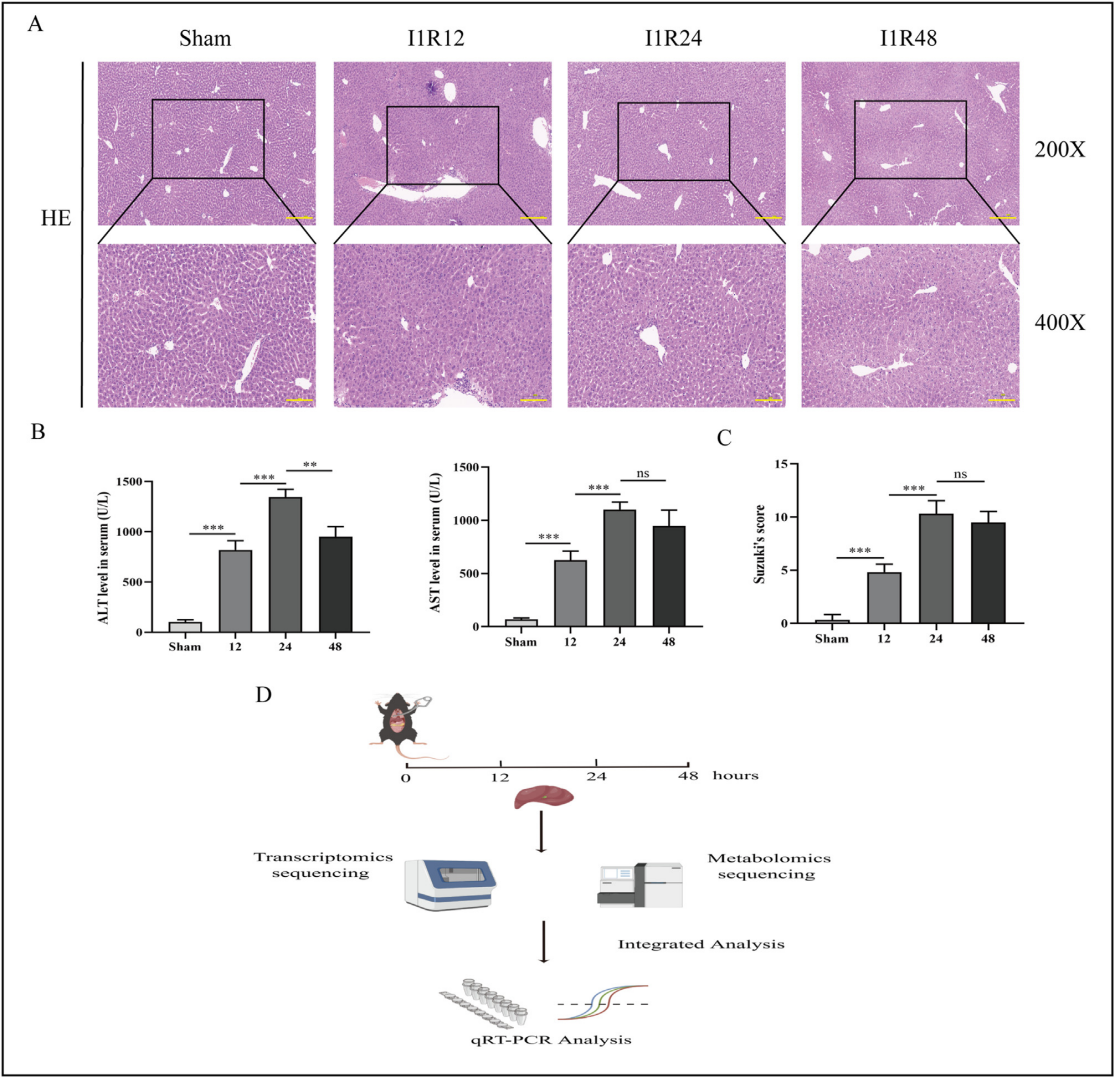
Establishment and validation of the hepatic ischemia-reperfusion injury model. **(A)** Hematoxylin-eosin staining of liver tissue samples of the Sham, I1R12, I1R24, and I1R48 groups (magnification, × 200/ × 400; scale bar, 100 mm). **(B)** Serum concentrations of aspartate aminotransferase (AST) and alanine aminotransferase (ALT). **(C)** Suzuki’s scores of the Sham, I1R12, I1R24, and I1R48 groups. **(D)** Schematic of the research process. *n* = 8; ∗*P* < 0.05, ∗∗*P* < 0.01, ∗∗∗*P* < 0.001; ns, no significance. I1R12, ischemia for 1 h and reperfusion for 12 h; I1R24, ischemia for 1 h and reperfusion for 24 h; I1R48, ischemia for 1 h and reperfusion for 48 h.

In each group, liver tissue samples were stained with hematoxylin and eosin (Fig. 1A). Hepatic IRI extent was assessed by Suzuki’s score (Fig. 1C). The degree of liver damage increased significantly with the extension of post-reperfusion time, and the serum concentrations of aspartate aminotransferase and alanine aminotransferase corroborated this result, peaking at 24 h of reperfusion (Fig. 1B). To investigate the characteristic changes in liver IRI over time, we collected mouse liver tissue samples from the I1R12, I1R24, I1R48, and Sham groups, metabolomics and transcriptomics analyses were performed, and the findings were validated by quantitative real-time PCR (Fig. 1D). The transcriptome and metabolome quality control results indicated that the data were fit to be used for further analysis (Fig. S1).

### Identification of DEGs

**Figure 2:**
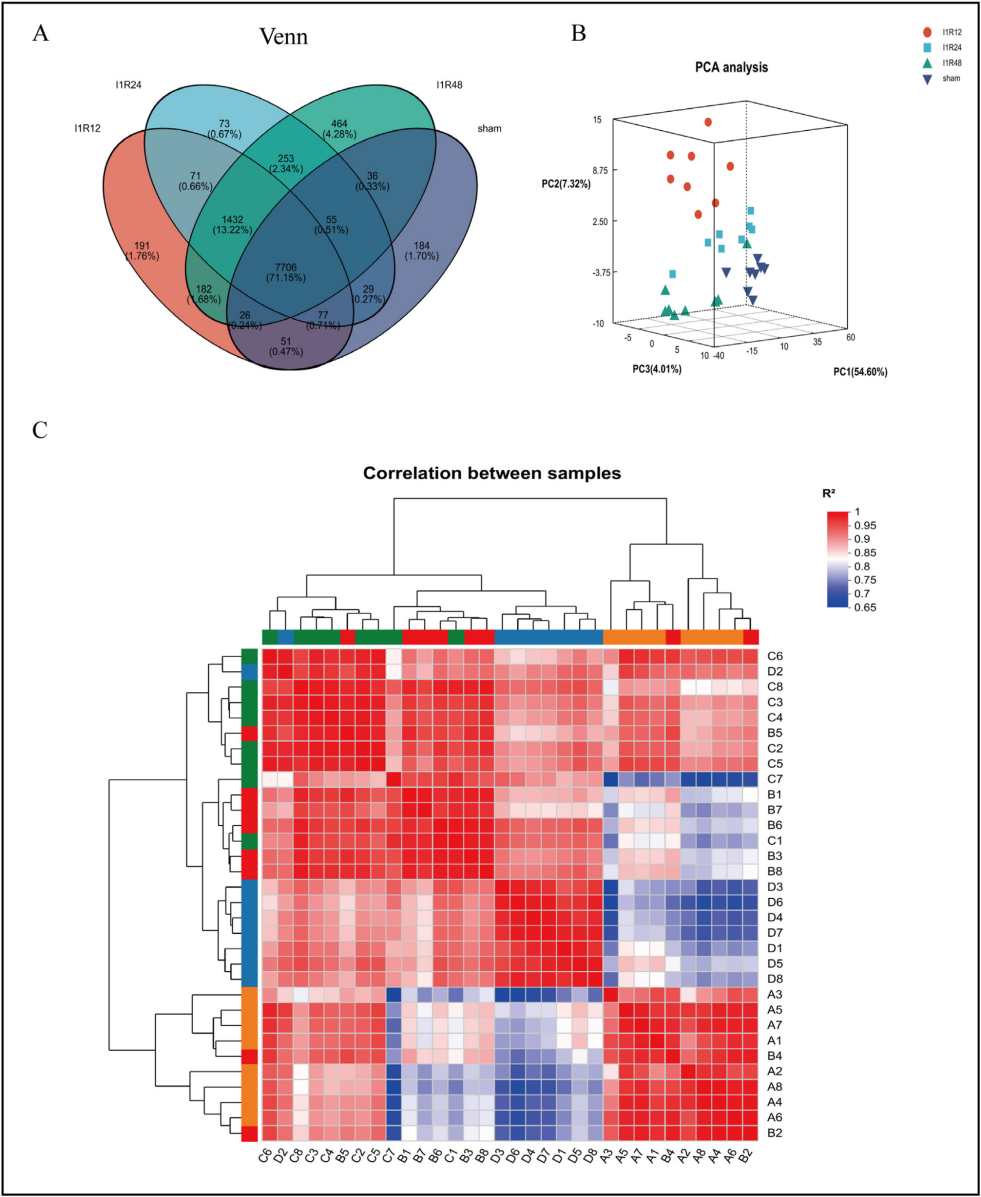
Correlation analysis of liver tissue samples. **(A)** Venn diagram analysis of the genes among the Sham, I1R12, I1R24, and I1R48 groups. **(B)** Principal component analysis of the Sham, I1R12, I1R24, and I1R48 groups. **(C)** Correlation heatmap of the Sham and IR groups. IR, ischemia and reperfusion; I1R12, ischemia for 1 h and reperfusion for 12 h; I1R24, ischemia for 1 h and reperfusion for 24 h; I1R48, ischemia for 1 h and reperfusion for 48 h.

**Figure 3:**
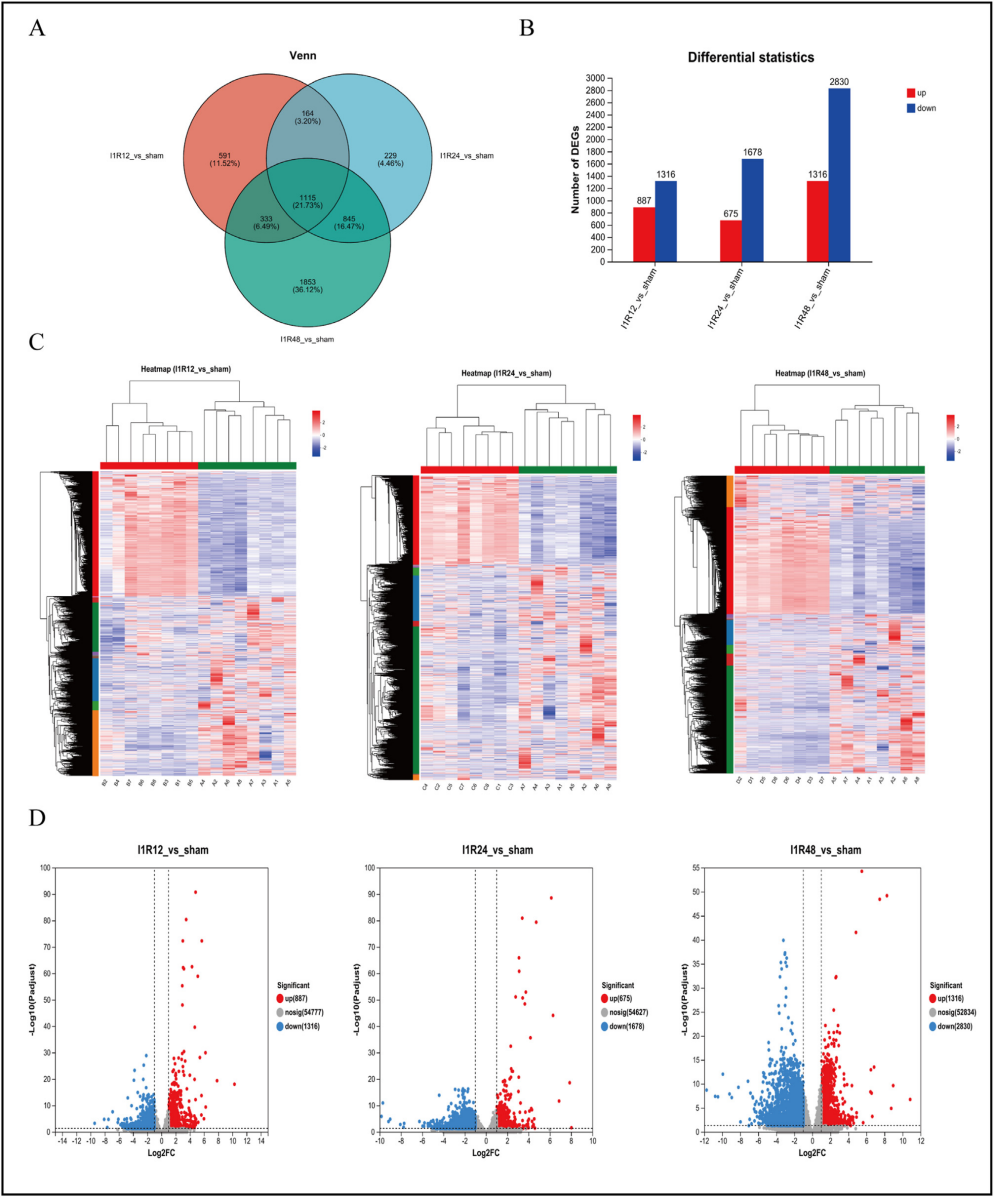
Hepatic ischemia-reperfusion injury involves transcriptional reprogramming. **(A)** Venn analysis of DEGs in the Sham/I1R12 groups, Sham/I1R24 groups, and Sham/I1R48 groups. **(B)** Histogram of the DEG number of the Sham/I1R12 groups, Sham/I1R24 groups, and Sham/I1R48 groups. **(C)** Hierarchical clustering heatmap of DEGs in the Sham/I1R12 groups, Sham/I1R24 groups, and Sham/I1R48 groups. **(D)** Volcano plots of DEGs in the Sham/I1R12 groups, Sham/I1R24 groups, and Sham/I1R48 groups. Blue denotes down-regulated genes, and red represents up-regulated genes. I1R12, ischemia for 1 h and reperfusion for 12 h; I1R24, ischemia for 1 h and reperfusion for 24 h; I1R48, ischemia for 1 h and reperfusion for 48 h; DEG, differentially expressed gene.

The co-expressed and specifically expressed genes among the four groups were indicated in a Venn diagram (Fig. 2A). Principal component analysis and correlation heatmaps revealed significant between-group differences (Fig. 2B, C), proving the rationality of liver tissue samples. Through transcriptome data analysis, based on the criteria |log_2_fold change| ≥ 1 and *P* < 0.05, 2203 DEGs (887 up-regulated and 1316 down-regulated) (Table S1A) were identified in the I1R12 group versus the Sham group. Furthermore, 2353 DEGs (675 up-regulated and 1678 down-regulated) (Table S1B) were identified in the I1R24 group versus the Sham group, and 4146 DEGs (1316 up-regulated and 2830 down-regulated) (Table S1C) were identified in the I1R48 group versus the Sham group (Fig. 3B). The corresponding heatmaps and volcano plots are shown in detail in Figure 3C and D. Based on the above DEG data, the Venn diagram showing the co-expressed genes of three comparison groups yielded 1115 DEGs (Fig. 3A).

### Function enrichment analysis of DEGs

**Figure 4:**
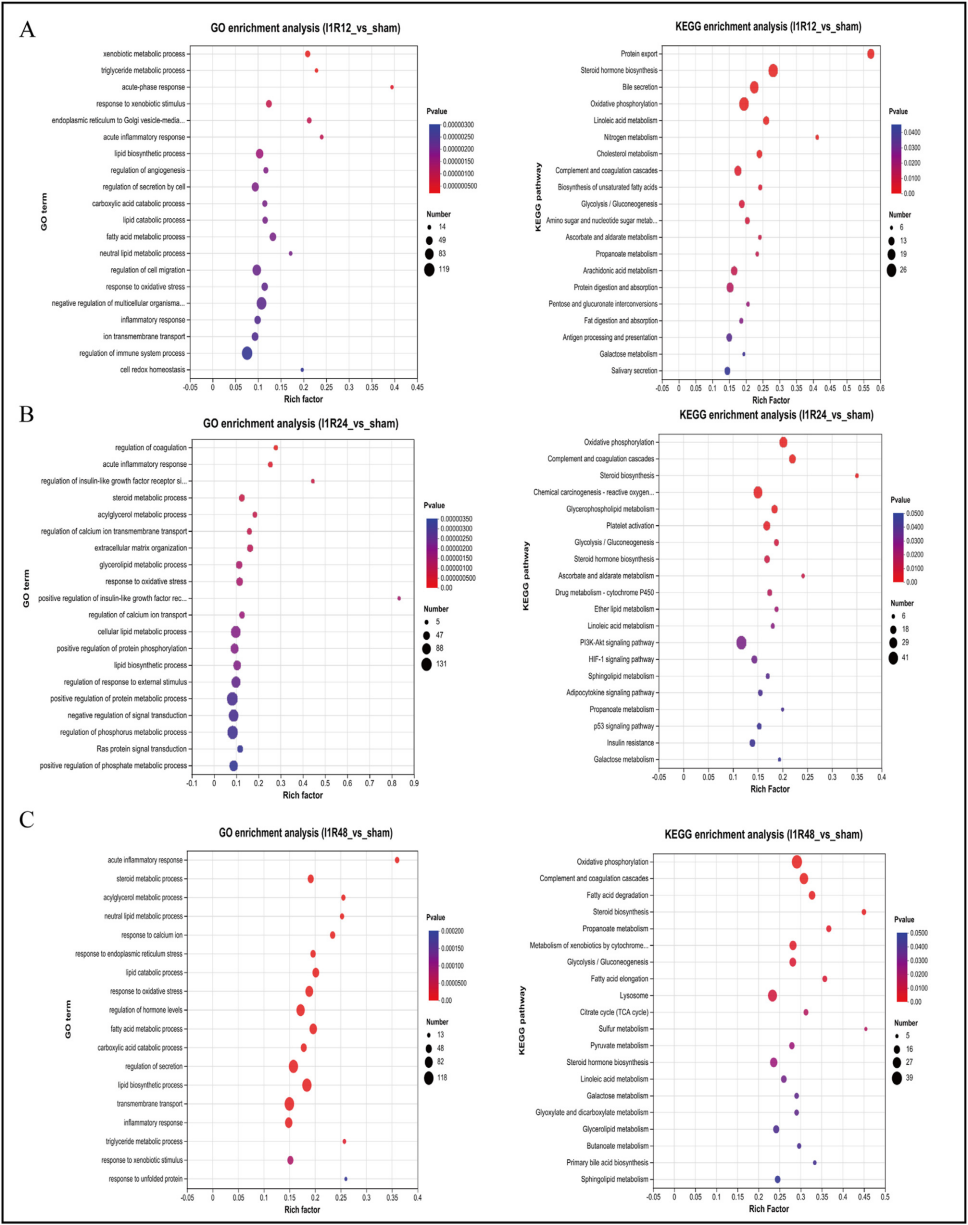
GO function and KEGG pathway enrichment analyses of the DEGs. **(A)** GO and KEGG enrichment analyses of the Sham and I1R12 groups. **(B)** GO and KEGG enrichment analyses of the Sham and I1R24 groups. **(C)** GO and KEGG enrichment analyses of the Sham and I1R48 groups. All GO function and KEGG pathway enrichment analyses of the DEGs revealed the top 20 functional terms and pathways. DEG, differentially expressed gene; KEGG, Kyoto encyclopedia of genes and genomes; GO, Gene Ontology; I1R12, ischemia for 1 h and reperfusion for 12 h; I1R24, ischemia for 1 h and reperfusion for 24 h; I1R48, ischemia for 1 h and reperfusion for 48 h; DEG, differentially expressed gene.

GO function (Tables S2A–C) and KEGG pathway enrichment (Tables S3A–C) analyses were performed to analyze the characteristic changes in biological functions and involved pathways in the early, intermediate, and late phases of IRI. In the early phase of hepatic IRI, KEGG analysis revealed four significant enrichment pathways: glycolysis/gluconeogenesis, galactose metabolism, biosynthesis of unsaturated fatty acids, and pentose and glucuronate interconversions, whereas lipid biosynthetic process and response to oxidative stress processes were enriched in GO analysis (Fig. 4A). Thus, glucose and carbohydrate metabolism were characteristic changes in the early phase of IRI. Regarding the intermediate phase of hepatic IRI, GO enrichment analysis was enriched in the cellular lipid metabolic process and acute inflammatory response. In contrast, KEGG analysis was enriched in the glycolysis/gluconeogenesis and insulin resistance pathways, and PI3K-AKT, HIF-1, and adipocytokine signaling pathways (Fig. 4B), involving glucose and lipid metabolism and activation of the inflammatory pathway. In addition, KEGG analysis was enriched in the fatty acid degradation, fatty acid elongation, and linoleic acid metabolism pathways in the late phase of IRI, and GO enrichment analysis was enriched in lipid catabolic, neutral lipid metabolic, fatty acid metabolic, lipid biosynthetic, and triglyceride metabolic processes (Fig. 4C). This result demonstrated that lipid metabolism was the main characteristic change during the late phase of IRI.

**Figure 5:**
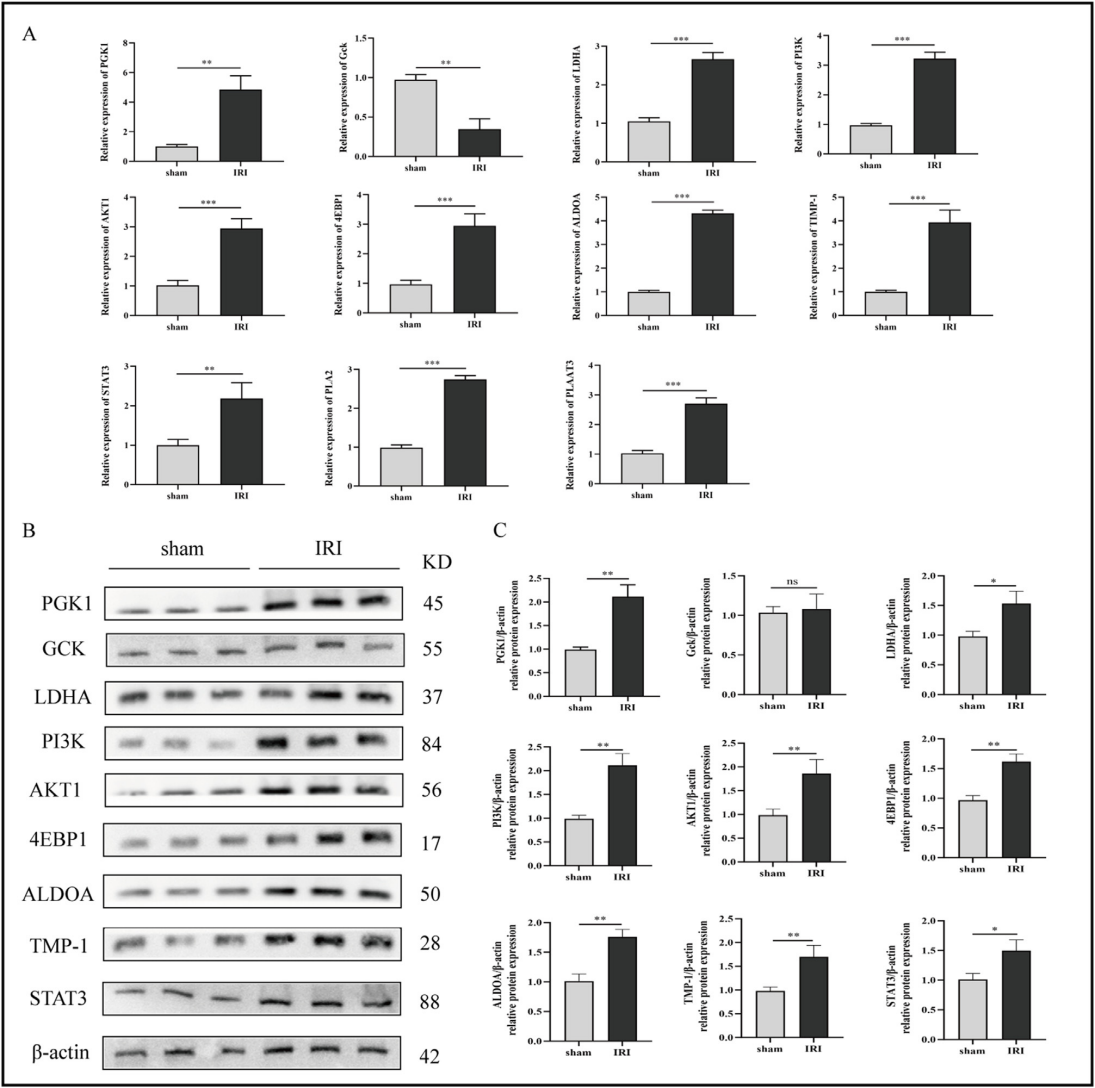
The relative mRNA and protein expression levels of 11 biomarkers in the Sham and IRI groups. **(A)** The relative mRNA levels of 11 biomarkers in the Sham and IRI groups measured by quantitative real-time PCR. **(B, C)** The protein levels of 11 biomarkers detected by western blotting. *n* = 4; ∗*P* < 0.05, ∗∗*P* < 0.01, ∗∗∗*P* < 0.001. IRI, ischemia-reperfusion injury.

**Figure 6:**
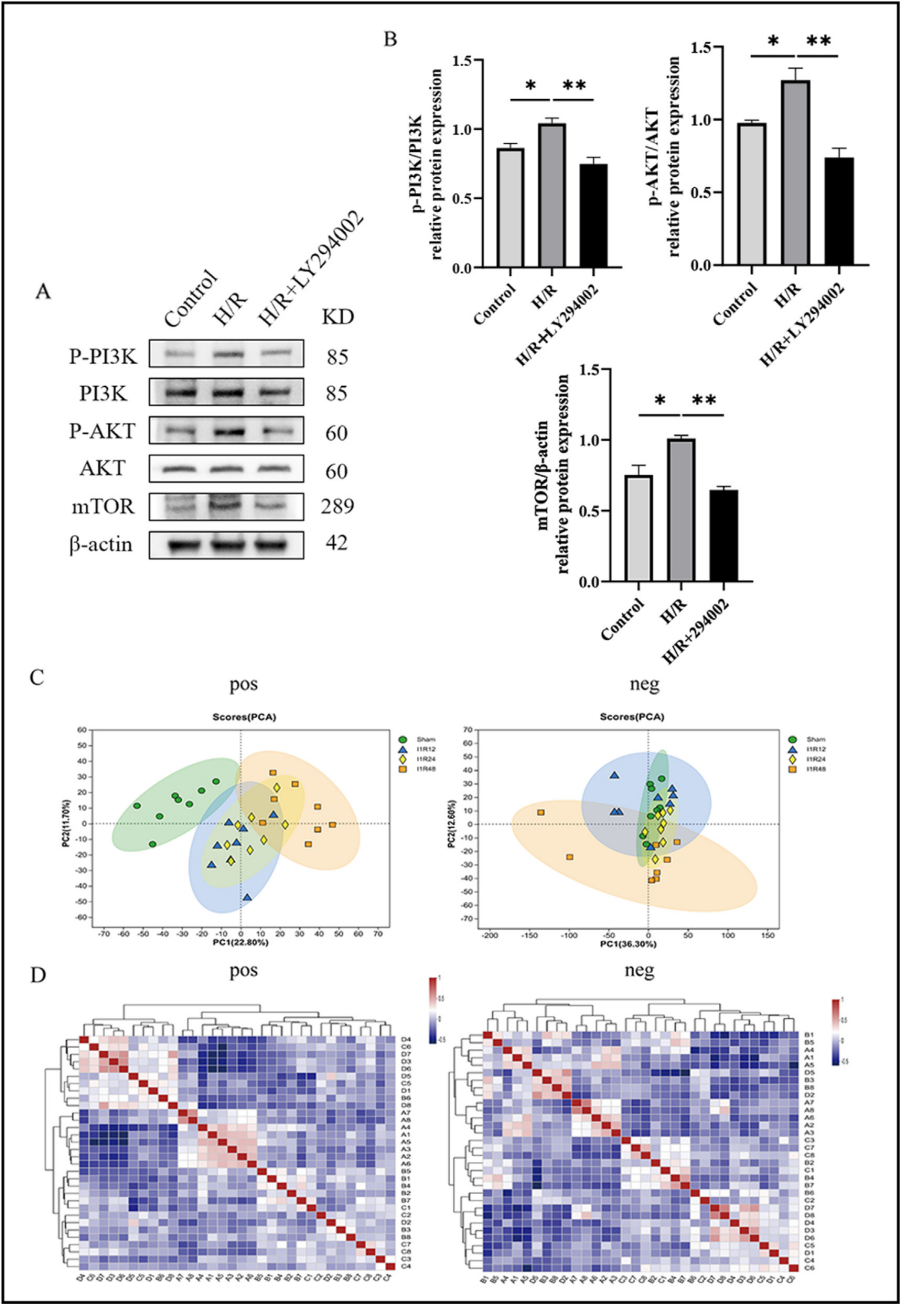
The activation of PI3K/AKT/mTOR pathway and hepatic ischemia-reperfusion injury involves the metabolic reprogramming. **(A, B)** The protein levels of P13K/AKT/mTOR pathway detected by western blotting. **(C)** Principal component analysis of the Sham, I1R12, I1R24, and I1R48 groups in the positive and negative ionization modes. **(D)** Correlation heatmaps of the Sham, I1R12, I1R24, and I1R48 groups in the positive and negative ionization modes. ∗*P* < 0.05, ∗∗*P* < 0.01, ∗∗∗*P* < 0.001. I1R12, ischemia for 1 h and reperfusion for 12 h; I1R24, ischemia for 1 h and reperfusion for 24 h; I1R48, ischemia for 1 h and reperfusion for 48 h; DEG, differentially expressed gene.

To validate the results of GO and KEGG analyses, western blotting and quantitative real-time PCR were applied to measure the relative mRNA and protein expression levels of several biomarker-related pathways selected from KEGG analysis. Compared with the Sham group, the relative mRNA expression of PGK1 and LDHA increased, while the mRNA expression of GCK decreased, indicating up-regulation of the glycolysis pathway. The relative mRNA expression levels of PI3K, AKT1, 4EBP1, ALDOA, TIMP-1, and STAT3 were elevated, suggesting the up-regulation of the HIF-1 and PI3K-AKT pathways. In addition, the increased expression of PLA2 and PLAAT3 indicated up-regulation of the linoleic acid metabolism pathway (Fig. 5A). At the same time, we confirmed that the protein expression of PGK1, LDHA, PI3K, AKT, 4EBP1, ALDOA, TNP-1 and STAT3 in the liver tissue were increased, which were consistent with the results of quantitative real-time PCR (Fig. 5B, C). Though hypoxia/reoxygenation model *in vitro*, we used LY294002 (PI3K inhibitor) to confirm the activation of PI3K/AKT/mTOR pathway and found obviously increased protein expression of phosphorylation of PI3K (p-PI3K) and phosphorylation of AKT (p-AKT) in the hypoxia/reoxygenation-treated group. Meanwhile, the group treated with hypoxia/reoxygenation and LY294002 showed a reduction of the expression of p-PI3K and p-AKT, indicating that LY294002 suppressed the PI3K/AKT/mTOR pathway successfully (Fig. 6A, B). These results were consistent with the GO and KEGG analyses of transcriptomics.

### Identification of DEMs

**Figure 7:**
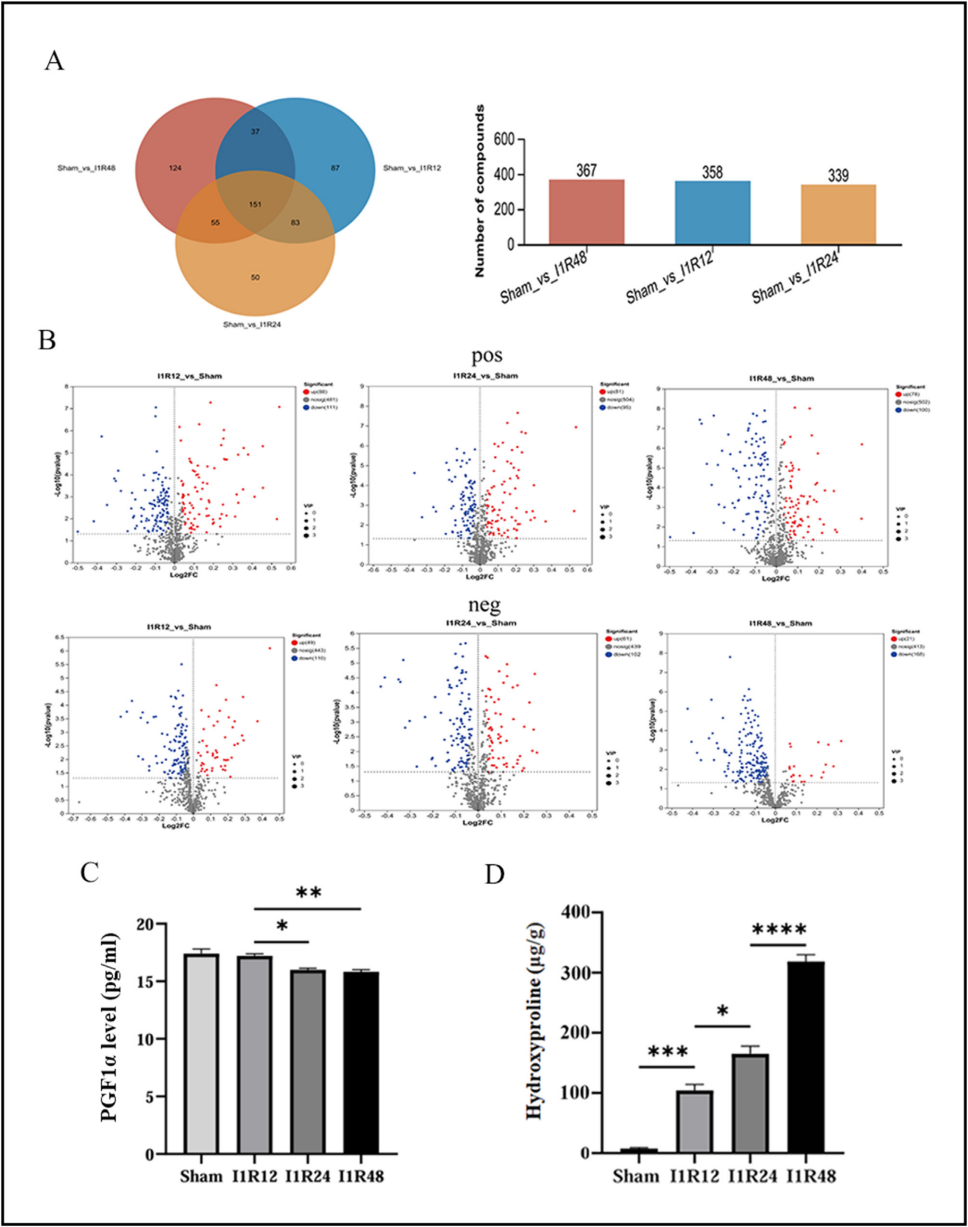
Hepatic ischemia-reperfusion injury involves the metabolic reprogramming. **(A)** Venn diagram (left) and histogram (right) of DEMs in the Sham/I1R12 groups, Sham/I1R24 groups, and Sham/I1R48 groups. **(B)** Positive and negative ionization modes volcano plots of DEMs in the Sham/I1R12 groups, Sham/I1R24 groups, and Sham/I1R48 groups. The blue dots denote uptake of metabolites, and the red dots indicate release of metabolites. **(C)** The level of PGF1α in cell culture media measured by ELISA. **(D)** The level of hydroxyproline in mouse liver samples measured with hydroxyproline content assay kit. *n* = 3; ∗*P* < 0.05, ∗∗*P* < 0.01, ∗∗∗*P* < 0.001. I1R12, ischemia for 1 h and reperfusion for 12 h; I1R24, ischemia for 1 h and reperfusion for 24 h; I1R48, ischemia for 1 h and reperfusion for 48 h; DEG, differentially expressed gene; DEM, differentially expressed metabolite.

Principal component analysis and correlation heatmaps of the positive and negative ionization modes showed apparent separation differences between the samples of each group (Fig. 6C, D). Principal component analysis plots showed that the separation between the I1R48 and Sham groups was the most obvious. Furthermore, based on the criteria (variable importance in projection >1, and *P* < 0.05), 358 DEMs (137 up-regulated, 221 down-regulated) (Table S4A) were identified in the I1R12 and Sham groups. A total of 339 DEMs (142 up-regulated, 197 down-regulated) (Table S4B) were identified in the I1R24 and Sham groups, and 367 DEMs (99 up-regulated, 268 down-regulated) (Table S4C) in the I1R48 and Sham groups. The corresponding volcano plots are shown in Figure 7B. The Venn diagram for analyzing the co-expressed DEMs among the three groups yielded 151 metabolites (Fig. 7A), and we selected two metabolites for validation. We determined the concentration of cell culture supernatant PGF1α was decreased and the hydroxyproline content of mouse liver tissue samples was increased following the extent of reperfusion, consistent with metabolomics results (Fig. 7C, D).

### Function enrichment analysis of DEMs

**Figure 8:**
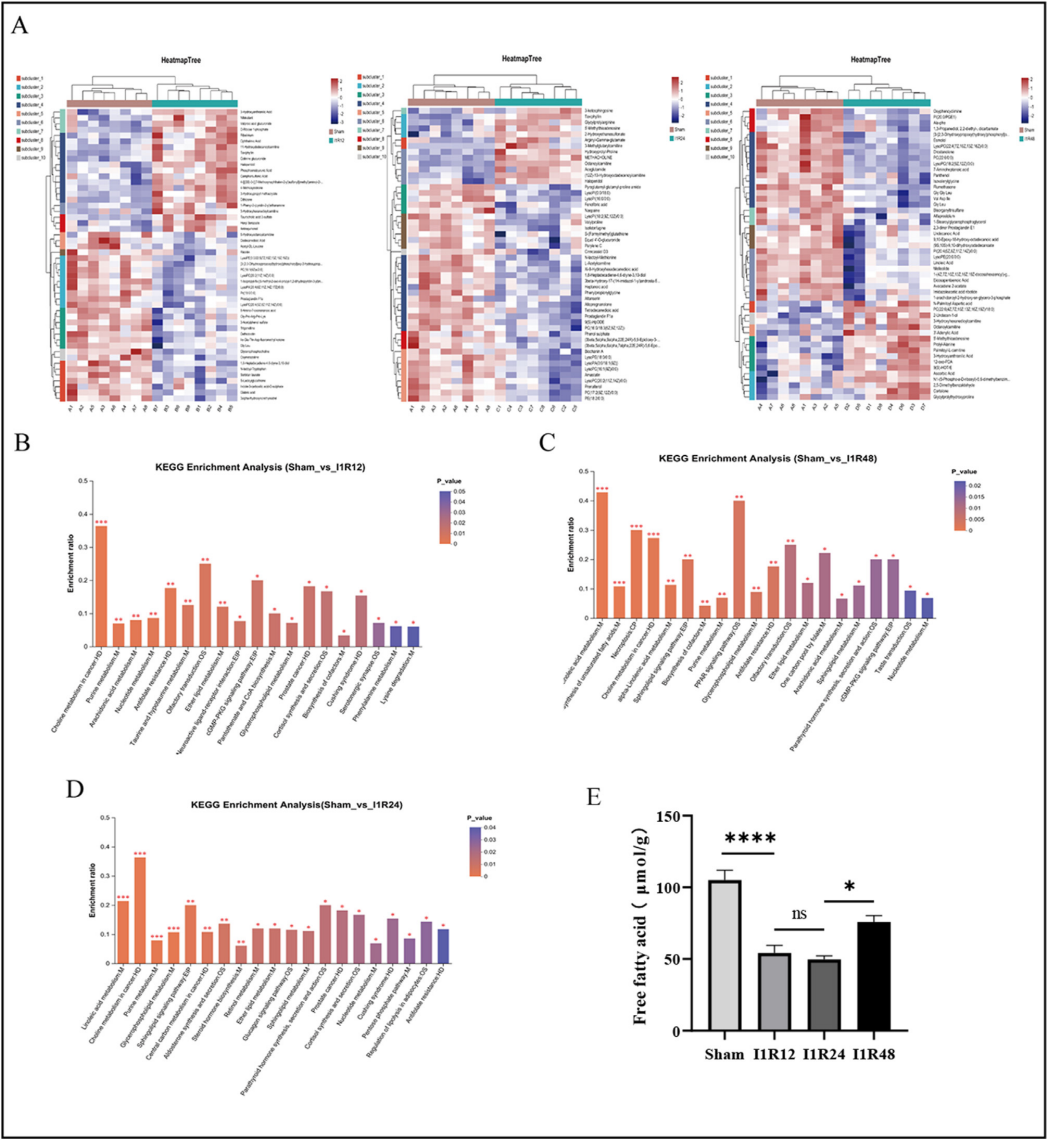
Hierarchical clustering heatmap and KEGG pathway enrichment analysis of the DEMs. **(A)** Hierarchical clustering heatmap of DEMs in the Sham and IR groups. **(B)** KEGG analysis of the DEMs in the Sham and I1R12 groups. **(C)** KEGG analysis of the DEMs in the Sham and I1R24 groups. **(D)** KEGG analysis of the DEMs in the Sham and I1R48 groups. **(E)** The level of free fatty acid (FFA) measured by ELISA. All KEGG pathway enrichment analyses revealed the top 20 pathways. *n* = 3; ∗*P* < 0.05, ∗∗*P* < 0.01, ∗∗∗*P* < 0.001. DEM, differentially expressed metabolite; KEGG, Kyoto encyclopedia of genes and genomes; IR, ischemia and reperfusion; I1R12, ischemia for 1 h and reperfusion for 12 h; I1R24, ischemia for 1 h and reperfusion for 24 h; I1R48, ischemia for 1 h and reperfusion for 48 h.

To analyze the differences in metabolites between groups, we performed clustering heatmaps of the I1R12, I1R24, and I1R48 groups versus the Sham group (Fig. 8A). Clear separations were demonstrated in the heatmaps, showing that the mouse liver reperfusion models underwent significant metabolic recombination following ischemia and reperfusion, consistent with the principal component analysis and correlated heatmaps. Subsequently, KEGG analysis of the DEMs was conducted (Tables S5A–C). Analysis of metabolites showed that the following metabolic pathways were enriched in the I1R12 and Sham groups: arachidonic acid, glycerophospholipid, and ether lipid metabolism (Fig. 8B). In the I1R24 and Sham groups, the significantly differential metabolic pathways were linoleic acid metabolism, glycerophospholipid metabolism, regulation of lipolysis in adipocytes, sphingolipid signaling pathway, and glucagon signaling pathway (Fig. 8D). In the I1R48 and Sham groups, KEGG analysis was enriched in arachidonic acid metabolism, PPAR signaling pathway, alpha-linolenic acid metabolism, and biosynthesis of unsaturated fatty acids (Fig. 8C). Meanwhile, we detected the free fatty acid content of mouse liver tissue samples and showed that the free fatty acid levels in the I1R12 and I1R24 groups were significantly decreased, indicating the existence of lipid metabolism disorder. Our findings demonstrated that the primary metabolic characteristics of lipid metabolism were altered in the early, intermediate, and late phases of IRI.

### Component analysis of DEMs

**Figure 9:**
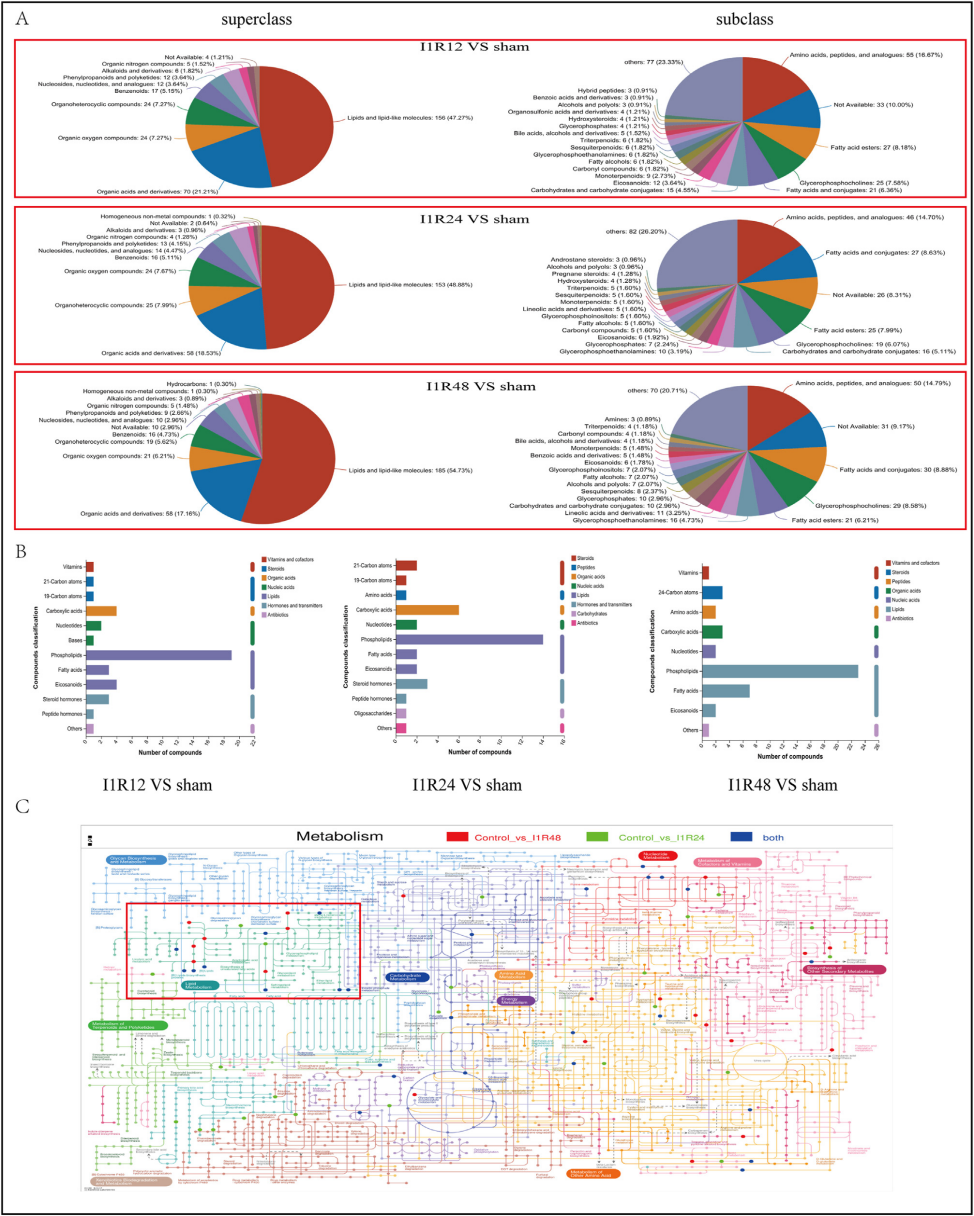
The identified metabolites were classified based on the HMDB and KEGG compound databases. **(A)** Pie chart of the identified metabolites based on the HMDB database. **(B)** Histogram of the identified metabolites based on the KEGG compound database. **(C)** Integrated pathway analysis of DEMs in the Sham/I1R24 groups and Sham/I1R48 groups. The rectangle circled by red line indicated that most DEMs were linked to lipid metabolism. DEM, differentially expressed metabolite; I1R24, ischemia for 1 h and reperfusion for 24 h; I1R48, ischemia for 1 h and reperfusion for 48 h.

To analyze the specific components of DEMs, 358 DEMs in the I1R12 and Sham groups were assigned to the HMDB database; 330 metabolites were classified into 10 HMDB superclasses and 21 HMDB subclasses; 156 metabolites were included in the “lipids and lipid-like molecules” superclass, and 77 metabolites were included in the “others” subclass, which were the first class in superclass and subclass, respectively (Fig. 9A). Similarly, 339 DEMs in the I1R24 and Sham groups were assigned to the HMDB database; 313 metabolites were classified into 11 superclasses and 21 subclasses; “lipids and lipid-like molecules” superclass contained 153 metabolites, and the “others” subclass contained 82 metabolites, both of which are the first class (Fig. 9A). In the I1R48 and Sham groups, 367 DEMs were assigned to the HMDB database; 338 metabolites were classified into 12 superclasses and 21 subclasses; 185 metabolites were included in the first superclass, “lipids and lipid-like molecules”, and 70 metabolites were included in the first subclass, “others” (Fig. 9A). It was evident from the results of the HMDB database that the proportion of “lipids and lipid-like molecules” increased with prolonged reperfusion time, indicating that the lipid metabolism was significantly altered. In addition, the KEGG compound classification results indicated that the number of fatty acids increased markedly with an increase in reperfusion time (Fig. 9B), consistent with the HMDB database results and KEGG analysis. Notably, our findings showed that most DEMs in the three groups were linked to lipid metabolism, and the integrated pathway analysis also proved that (Fig. 9C).

## Discussion

Liver IRI is an unavoidable consequence of liver transplantation and partial hepatectomy that involves multiple pathological mechanisms.^14^^,^^15^ Most liver IRI research focuses on the inflammatory response and cell death; however, metabolism and detoxification are important functions of the liver. Thus, a study suggested ischemia-reperfusion primarily disrupts metabolic homeostasis, followed by an inflammatory response and hepatic damage.^5^ Previous studies have indicated that glucolipid metabolism regulated by INSIG2, and lipid metabolic reprogramming, including arachidonate 12-lipoxygenase (ALOX12) and its downstream metabolites, influence hepatic IRI through the release of damage-associated molecular patterns (DAMPs) and oxidative stress.^7^^,^^16^ However, alterations in signaling pathways and metabolic profiles in the early, intermediate, and late phases of hepatic IRI remain undefined.

Transcriptomics and metabolomics have been employed in medical research to investigate the mechanisms underlying various disorders.^17^ In a recent study, transcriptomics was used to provide a deeper explanation of the molecular mechanism of IRI.^18^ Additionally, metabolomics is to reveal certain pathophysiological processes by detecting the level of changes in metabolites in organisms and as an effective method has been used in research on the molecular mechanisms of IRI in metabolic remodeling.^8^ This study investigated the pathogenesis of hepatic IRI from a new perspective by combining transcriptomics and metabolomics in the early, intermediate, and late phases of hepatic IRI.

In the initial phase of hepatic IRI, ischemia leads to an insufficient oxygen supply to hepatic cells and impairs them via exposure to glucose consumption, pH changes, and ATP depletion, resulting in disturbances in cellular metabolism and inflammation.^3^^,^^19^ Meanwhile, glycolysis is a major energy source, and accelerated glycolysis and ATP depletion increase the accumulation of acidic metabolites, impairing signaling interactions, cellular homeostasis, and hepatocytes, and triggering mitochondrial dysfunction and inflammatory responses.^7^^,^^20^ In this study, KEGG analysis showed that the glycolysis/gluconeogenesis pathway was altered exclusively during the early phase of IRI when glycolytic flux increased to satisfy the energy requirement in the state of hypoxia. The outcomes of our study illustrated that glycose metabolism reprogramming is critical in the early phase of IRI and could be a metabolic intervention treatment to reduce the subsequent inflammatory response. Several studies have reported that glycolysis interference treatments significantly inhibited glycolysis and the release of inflammatory cytokines, improving the acidic microenvironment and acidosis and attenuating hepatic cellular damage.^21^, ^22^, ^23^

Liver IRI involves two interconnected stages: local ischemia injury and reperfusion injury caused by sterile inflammation.^19^ The findings of this study confirmed that in the intermediate phase of IRI, inflammatory responses were triggered and became more intense. The intermediate phase of IRI is characterized by inflammatory disorder, triggered by the overproduction of reactive oxygen species and the release of DAMPs and pro-inflammatory cytokines, aggravating apoptosis and hepatocyte damage.^24^^,^^25^ Based on the KEGG analysis of the intermediate phase, this study found that inflammation-related signaling pathways such as PI3K-AKT and HIF-1 were markedly regulated, taking part in anti-inflammatory and adaptive hypoxia responses during IRI, providing a potential therapeutic intervention in regulating anaerobic glycolysis and inflammatory response to improve IRI.^26^, ^27^, ^28^ Our experiments *in vitro* cell model of hypoxia/reoxygenation also indicated that the activation of the PI3K/AKT pathway during IRI was obviously suppressed by PI3K inhibitors. Meanwhile, some studies have demonstrated the P13K-AKT pathway has the potential to serve as a therapeutic intervention target to mitigate IRI by reducing reactive oxygen species production and pro-apoptotic signals.^29^^,^^30^

Moreover, KEGG analysis, compound classification, and integrated pathway analysis found that lipid metabolism remodeling was the characteristic alteration in the late phase of IRI. The liver is an important organ for lipid metabolism, and essential fatty acids play a vital role in hepatic IRI; for example, lipids are one of the main targets of reactive oxygen species in oxidative stress, contributing to IRI through the concentration of fatty acids and lipid peroxidation, forming cytotoxic lipid aldehydes and lipid hydroperoxides.^31^ Previous studies have reported that lipid metabolic disorders during IRI induce oxidative stress, inflammation, apoptosis, and ferroptosis by modulating interrelated transduction signaling pathways and suppressing antioxidant capacity, which could aggravate lipid metabolic reprogramming.^32^^,^^33^ Meanwhile, previous clinical research demonstrated that lipid biosynthesis was the major change during IRI, severe steatosis was associated with a higher incidence of graft failure after liver transplantation, and some metabolites had the potential to be biomarkers of lipid-related damage of IRI.^24^^,^^34^ These findings and our results indicate that lipid metabolic reprogramming plays a key role in hepatic IRI and aggravates IRI. Consequently, we present a new perspective on IRI therapeutic intervention: intervening in the major metabolic reprogramming at each stage through clinical means could effectively control the subsequent inflammatory response, and even predict the prognosis of liver transplantation through the concentrations of mainly different metabolites at each stage. It has been reported that regulating lipid metabolism response and mediators could ameliorate the pathological damage from ischemia-reperfusion by reducing mitochondrial damage and liver macrophage pyroptosis.^5^^,^^35^^,^^36^ This study illustrated the importance of metabolic reprogramming in hepatic IRI and its potential as a therapeutic intervention target.

The above findings were derived from animal experiments but not verified in clinical samples. We simply validated some of the pathways and metabolites through *in vivo* and *in vitro* hypoxia/reoxygenation models, but we did not deeply explore the specific mechanisms of different metabolites.

In summary, by combining transcriptomics and metabolomics, our study first revealed characteristic changes in signaling pathways and metabolism in the early, intermediate, and late phases of hepatic IRI. Lipid metabolism, precisely regulated by the liver through biochemical, signaling, and cellular pathways, plays a non-negligible role in the occurrence and development of hepatic IRI. This represents a potential therapeutic intervention to treat hepatic IRI and strengthens the understanding of the pathogenesis and pathological process of IRI and its molecular mechanism.

## Funding

This study was funded by the 10.13039/100014717National Natural Science Foundation of China (No. 82300745 to Yanyao Liu), 10.13039/501100010008China Postdoctoral Science Foundation (No. 2023M730442 to Yanyao Liu), 10.13039/501100010008Chongqing Postdoctoral Science Foundation of China (No. CSTB2023NSCQ-BHX1003 to Yanyao Liu), Postdoctoral Cultivation Project of the First Affiliated Hospital of Chongqing Medical University (No.CYYY-BSHPYXM-202301 to Yanyao Liu), and Chongqing Postdoctoral Innovation Talents Support Program (Chongqing, China) (No. 2309013437264551 to Yanyao Liu).

## Author contributions

**Qi Li:** Writing – original draft, Data curation, Investigation, Methodology, Project administration. **Xiaoyan Qin:** Data curation, Investigation, Methodology, Project administration, Resources, Writing – original draft. **Liangxu Wang:** Data curation, Investigation. **Dingheng Hu:** Investigation, Project administration. **Rui Liao:** Project administration, Resources. **Zhongjun Wu:** Data curation, Investigation, Project administration, Resources, Supervision, Writing – original draft, Writing – review & editing. **Huarong Yu:** Project administration, Methodology, Resources, Writing – original draft, Writing – review & editing. **Yanyao Liu:** Funding acquisition, Supervision, Writing – original draft, Writing – review & editing, Data curation, Investigation, Project administration, Resources.

## Data availability

All data generated or analyzed during this study are included in this published article and supplementary material.

## Conflict of interests

The authors declared no conflict of interests.

## Figures and Tables

**figs1:**