# Supplementary material for: Multi-time point transcriptomics and metabolomics reveal key transcription and metabolic features of hepatic ischemia-reperfusion injury in mice
Source: Genes Dis. 2024 Nov 17;12(2):101465. doi: 10.1016/j.gendis.2024.101465 (PMC11697123; doi:10.1016/j.gendis.2024.101465)
Supplement: Multimedia component 1 [file mmc1.docx]

**Table S1A.** Identified differentially expressed genes between Sham and I1R12 groups.

| **Gene ID** | **Gene name** | **Log2FC** | **P-value** | **Regulate** |
| --- | --- | --- | --- | --- |
| ENSMUSG00000120050 | - | -1.97157 | 0.01216 | down |
| ENSMUSG00000086657 | Stamos | 1.722334 | 0.01215 | up |
| ENSMUSG00000086894 | Gm15708 | -2.01496 | 0.01214 | down |
| ENSMUSG00000025888 | Casp1 | -1.22757 | 0.01211 | down |
| ENSMUSG00000085667 | Gm12992 | -1.25943 | 0.01211 | down |
| ENSMUSG00000024730 | Ms4a8a | 1.951016 | 0.01209 | up |
| ENSMUSG00000028884 | Rpa2 | 1.191438 | 0.01203 | up |
| ENSMUSG00000100586 | Vmn1r90 | -2.24559 | 0.01203 | down |
| ENSMUSG00000117490 | Gm35031 | -1.85877 | 0.01203 | down |
| ENSMUSG00000072244 | Trim6 | -1.72864 | 0.01201 | down |
| ENSMUSG00000103580 | Gm10417 | -2.33912 | 0.01199 | down |
| ENSMUSG00000091572 | Vmn2r3 | -2.81279 | 0.01197 | down |
| ENSMUSG00000107117 | Gm43842 | -1.74652 | 0.01195 | down |
| ENSMUSG00000019808 | Adat2 | 1.746453 | 0.01188 | up |
| ENSMUSG00000025877 | Hk3 | -1.22408 | 0.01186 | down |
| ENSMUSG00000038304 | Cd160 | -1.70208 | 0.01184 | down |
| ENSMUSG00000047977 | Synb | -3.15954 | 0.01183 | down |
| ENSMUSG00000104626 | Gm42675 | -4.01033 | 0.01181 | down |
| ENSMUSG00000039316 | Rftn1 | -1.35809 | 0.01178 | down |
| ENSMUSG00000118506 | Cfap141 | -1.38098 | 0.01178 | down |
| ENSMUSG00000025003 | Cyp2c39 | 1.813941 | 0.01172 | up |
| ENSMUSG00000042909 | Olfr648 | -2.5796 | 0.01164 | down |
| ENSMUSG00000084923 | Gm15611 | 3.773717 | 0.01164 | up |
| ENSMUSG00000087132 | A930001C03Rik | -1.31009 | 0.01163 | down |
| ENSMUSG00000029372 | Ppbp | -1.75094 | 0.01158 | down |
| ENSMUSG00000031659 | Adcy7 | -1.04312 | 0.01157 | down |
| ENSMUSG00000120414 | Gm32261 | -2.66916 | 0.01157 | down |
| ENSMUSG00000098789 | Jmjd7 | 2.256988 | 0.01156 | up |
| ENSMUSG00000045225 | Olfr1152 | -2.76607 | 0.01152 | down |
| ENSMUSG00000040987 | Mill2 | -1.36245 | 0.01150 | down |
| ENSMUSG00000037363 | Letm2 | -1.13815 | 0.01149 | down |
| ENSMUSG00000060503 | Olfr715 | -1.33743 | 0.01148 | down |
| ENSMUSG00000053310 | Nrgn | -1.38394 | 0.01147 | down |
| ENSMUSG00000037337 | Map4k1 | -1.42042 | 0.01143 | down |
| ENSMUSG00000121016 | - | -4.67204 | 0.01138 | down |
| ENSMUSG00000041538 | H2-Ob | -1.96724 | 0.01137 | down |
| ENSMUSG00000049685 | Cyp2g1 | -3.18071 | 0.01137 | down |
| ENSMUSG00000043448 | Gjc2 | -1.84572 | 0.01136 | down |
| ENSMUSG00000045744 | Bricd5 | -4.23144 | 0.01136 | down |
| ENSMUSG00000074813 | Morrbid | -1.53118 | 0.01136 | down |
| ENSMUSG00000120212 | - | -1.8821 | 0.01136 | down |
| ENSMUSG00000103220 | Gm37728 | -3.6409 | 0.01133 | down |
| ENSMUSG00000107511 | Gm44440 | -1.24599 | 0.01130 | down |
| ENSMUSG00000036882 | Arhgap33 | -1.87309 | 0.01129 | down |
| ENSMUSG00000038085 | Cnbd2 | 1.192949 | 0.01128 | up |
| ENSMUSG00000074215 | Gm10643 | 1.641463 | 0.01128 | up |
| ENSMUSG00000043300 | B3galnt1 | 2.162498 | 0.01123 | up |
| ENSMUSG00000120369 | - | -1.23022 | 0.01123 | down |
| ENSMUSG00000037624 | Kcnk2 | -2.31647 | 0.01122 | down |
| ENSMUSG00000106408 | Gm43321 | -1.09554 | 0.01115 | down |
| ENSMUSG00000014158 | Trpv4 | -1.98772 | 0.01112 | down |
| ENSMUSG00000023067 | Cdkn1a | 1.914447 | 0.01108 | up |
| ENSMUSG00000097365 | C030034L19Rik | -4.13181 | 0.01100 | down |
| ENSMUSG00000037860 | Aim2 | -1.46973 | 0.01091 | down |
| ENSMUSG00000019992 | Mtfr2 | -3.67502 | 0.01080 | down |
| ENSMUSG00000052909 | Gm9894 | -2.97938 | 0.01074 | down |
| ENSMUSG00000079610 | Ankrd39 | 1.301268 | 0.01071 | up |
| ENSMUSG00000121134 | - | 3.189251 | 0.01071 | up |
| ENSMUSG00000120856 | - | -2.02433 | 0.01066 | down |
| ENSMUSG00000021180 | Rps6ka5 | -1.43387 | 0.01065 | down |
| ENSMUSG00000096006 | Gm21596 | -1.14746 | 0.01065 | down |
| ENSMUSG00000104145 | D130019J16Rik | -4.73864 | 0.01063 | down |
| ENSMUSG00000091945 | Vmn2r114 | -1.00854 | 0.01062 | down |
| ENSMUSG00000120944 | - | -3.49052 | 0.01060 | down |
| ENSMUSG00000105454 | Gm43830 | -1.62465 | 0.01058 | down |
| ENSMUSG00000112833 | Gm36595 | -3.48751 | 0.01058 | down |
| ENSMUSG00000047238 | Mageh1 | 1.914917 | 0.01057 | up |
| ENSMUSG00000074622 | Mafb | -1.01729 | 0.01054 | down |
| ENSMUSG00000091680 | Klhdc7b | -3.19328 | 0.01053 | down |
| ENSMUSG00000053303 | Slc22a26 | -1.5247 | 0.01047 | down |
| ENSMUSG00000028738 | Tas1r2 | -4.69855 | 0.01046 | down |
| ENSMUSG00000021952 | Xpo4 | -1.43445 | 0.01045 | down |
| ENSMUSG00000023274 | Cd4 | -1.52527 | 0.01043 | down |
| ENSMUSG00000031253 | Srpx2 | -1.74 | 0.01042 | down |
| ENSMUSG00000031740 | Mmp2 | -1.40657 | 0.01042 | down |
| ENSMUSG00000105429 | Gm43692 | -1.83654 | 0.01041 | down |
| ENSMUSG00000104937 | Gm43057 | -3.06261 | 0.01040 | down |
| ENSMUSG00000106024 | A530083M17Rik | -1.835 | 0.01039 | down |
| ENSMUSG00000057363 | Uxs1 | 1.013969 | 0.01038 | up |
| ENSMUSG00000113200 | Gm48632 | -1.49451 | 0.01037 | down |
| ENSMUSG00000103364 | Gm38157 | -2.29832 | 0.01036 | down |
| ENSMUSG00000112806 | Gm48146 | -2.28652 | 0.01033 | down |
| ENSMUSG00000115329 | Gm49272 | -1.68092 | 0.01033 | down |
| ENSMUSG00000028633 | Ctps | 1.157096 | 0.01032 | up |
| ENSMUSG00000023755 | Rhebl1 | -2.14415 | 0.01031 | down |
| ENSMUSG00000112855 | Gm47842 | -1.58865 | 0.01031 | down |
| ENSMUSG00000039485 | Tspyl4 | -1.7893 | 0.01030 | down |
| ENSMUSG00000056468 | 5730596B20Rik | -3.44205 | 0.01029 | down |
| ENSMUSG00000087589 | D430040D24Rik | -2.43939 | 0.01028 | down |
| ENSMUSG00000046727 | Cystm1 | 1.044544 | 0.01027 | up |
| ENSMUSG00000121349 | Speer6-ps1 | -1.96811 | 0.01027 | down |
| ENSMUSG00000025372 | Baiap2 | -1.28433 | 0.01026 | down |
| ENSMUSG00000099632 | 2900093K20Rik | -1.16926 | 0.01007 | down |
| ENSMUSG00000119132 | Gm24407 | -3.82328 | 0.01005 | down |
| ENSMUSG00000112739 | Gm20597 | -2.49693 | 0.01000 | down |
| ENSMUSG00000115368 | Gm48942 | -1.06731 | 0.00998 | down |
| ENSMUSG00000025933 | Tmem14a | -1.15657 | 0.00996 | down |
| ENSMUSG00000023915 | Tnfrsf21 | 1.267497 | 0.00995 | up |
| ENSMUSG00000003573 | Homer3 | -1.30226 | 0.00991 | down |
| ENSMUSG00000111892 | Gm47641 | -3.42639 | 0.00991 | down |
| ENSMUSG00000053178 | Mterf1b | -1.27573 | 0.00989 | down |
| ENSMUSG00000023467 | Tulp2 | -3.9954 | 0.00982 | down |
| ENSMUSG00000028871 | Rspo1 | -2.89909 | 0.00981 | down |
| ENSMUSG00000044702 | Palb2 | -2.03844 | 0.00980 | down |
| ENSMUSG00000108162 | Gm20589 | -2.40676 | 0.00979 | down |
| ENSMUSG00000074676 | Foxs1 | -2.37571 | 0.00971 | down |
| ENSMUSG00000038354 | Ankrd35 | -4.81773 | 0.00967 | down |
| ENSMUSG00000103009 | Gm56350 | 2.417977 | 0.00961 | up |
| ENSMUSG00000103070 | Gm37903 | -1.35555 | 0.00961 | down |
| ENSMUSG00000028177 | 1810013D15Rik | -1.75284 | 0.00954 | down |
| ENSMUSG00000075289 | Carns1 | -1.29 | 0.00938 | down |
| ENSMUSG00000073274 | Gm14636 | -1.20778 | 0.00935 | down |
| ENSMUSG00000027605 | Acss2 | -1.05066 | 0.00933 | down |
| ENSMUSG00000020895 | Tmem107 | 1.728038 | 0.00928 | up |
| ENSMUSG00000055323 | Gm9967 | -2.18351 | 0.00927 | down |
| ENSMUSG00000062545 | Tlr12 | 1.587195 | 0.00924 | up |
| ENSMUSG00000105224 | Gm3364 | -2.32937 | 0.00921 | down |
| ENSMUSG00000037020 | Wdr62 | -1.33971 | 0.00915 | down |
| ENSMUSG00000061186 | Sfmbt2 | -4.95276 | 0.00888 | down |
| ENSMUSG00000040084 | Bub1b | -2.06049 | 0.00883 | down |
| ENSMUSG00000092035 | Peg10 | -1.70055 | 0.00883 | down |
| ENSMUSG00000087050 | Dhrs13os | -3.03025 | 0.00882 | down |
| ENSMUSG00000042985 | Upk3b | -2.58961 | 0.00880 | down |
| ENSMUSG00000094396 | Vmn2r124 | -1.12266 | 0.00880 | down |
| ENSMUSG00000081948 | Olfr1191 | -2.73149 | 0.00877 | down |
| ENSMUSG00000039646 | Vasn | 1.048321 | 0.00875 | up |
| ENSMUSG00000107726 | Gm44037 | -2.83224 | 0.00875 | down |
| ENSMUSG00000113523 | Gm48366 | -1.29258 | 0.00875 | down |
| ENSMUSG00000118796 | n-R5s138 | -4.59905 | 0.00875 | down |
| ENSMUSG00000097730 | Gm26588 | 1.334028 | 0.00867 | up |
| ENSMUSG00000062944 | 9130023H24Rik | 1.054203 | 0.00865 | up |
| ENSMUSG00000078496 | Zfp982 | -2.58186 | 0.00864 | down |
| ENSMUSG00000040253 | Gbp7 | -1.08403 | 0.00863 | down |
| ENSMUSG00000035595 | Fam174c | 1.018387 | 0.00861 | up |
| ENSMUSG00000003992 | Ssbp2 | -1.27812 | 0.00859 | down |
| ENSMUSG00000089829 | Gm16565 | -3.1463 | 0.00859 | down |
| ENSMUSG00000032565 | Nudt16 | 1.162649 | 0.00857 | up |
| ENSMUSG00000031433 | Rbm41 | 3.133888 | 0.00855 | up |
| ENSMUSG00000063698 | Sfxn4 | -2.02441 | 0.00854 | down |
| ENSMUSG00000056665 | Them6 | 1.04846 | 0.00851 | up |
| ENSMUSG00000114102 | Gm48048 | -4.90859 | 0.00851 | down |
| ENSMUSG00000118061 | Rbfaos | -1.94736 | 0.00849 | down |
| ENSMUSG00000086468 | Etaa1os | -1.84911 | 0.00847 | down |
| ENSMUSG00000066362 | Rps13-ps1 | 2.227813 | 0.00846 | up |
| ENSMUSG00000086054 | Hnf1aos1 | -1.08898 | 0.00845 | down |
| ENSMUSG00000097691 | 9030616G12Rik | -1.16315 | 0.00842 | down |
| ENSMUSG00000120276 | - | 3.022498 | 0.00840 | up |
| ENSMUSG00000002033 | Cd3g | -1.59455 | 0.00828 | down |
| ENSMUSG00000085873 | Ttc39aos1 | 1.5867 | 0.00828 | up |
| ENSMUSG00000115509 | Gm49012 | -2.83452 | 0.00827 | down |
| ENSMUSG00000036815 | Dpp10 | -1.48771 | 0.00826 | down |
| ENSMUSG00000032171 | Pin1 | 1.061426 | 0.00825 | up |
| ENSMUSG00000026102 | Inpp1 | -1.0005 | 0.00824 | down |
| ENSMUSG00000030030 | 1700003E16Rik | -1.3507 | 0.00823 | down |
| ENSMUSG00000040528 | Milr1 | -1.68748 | 0.00822 | down |
| ENSMUSG00000107331 | Gm42732 | -1.24004 | 0.00822 | down |
| ENSMUSG00000072847 | A530017D24Rik | -1.04128 | 0.00821 | down |
| ENSMUSG00000063388 | BC023105 | -2.06272 | 0.00815 | down |
| ENSMUSG00000037035 | Inhbb | 1.575063 | 0.00814 | up |
| ENSMUSG00000032278 | Paqr5 | 4.156239 | 0.00811 | up |
| ENSMUSG00000028949 | Smarcd3 | 1.087712 | 0.00809 | up |
| ENSMUSG00000121382 | - | -1.22557 | 0.00805 | down |
| ENSMUSG00000066804 | Vmn1r83 | -3.3812 | 0.00804 | down |
| ENSMUSG00000041559 | Fmod | -1.09898 | 0.00802 | down |
| ENSMUSG00000044367 | Slc16a13 | 1.541407 | 0.00802 | up |
| ENSMUSG00000026604 | Ptpn14 | -1.08374 | 0.00795 | down |
| ENSMUSG00000030664 | Sox6os | 1.460577 | 0.00794 | up |
| ENSMUSG00000038538 | Ubn2 | -1.37312 | 0.00791 | down |
| ENSMUSG00000066178 | 6030445D17Rik | -3.46834 | 0.00790 | down |
| ENSMUSG00000102353 | Gm38345 | 1.082966 | 0.00785 | up |
| ENSMUSG00000086859 | Snhg20 | -1.10412 | 0.00780 | down |
| ENSMUSG00000038888 | Ctu1 | 1.198242 | 0.00779 | up |
| ENSMUSG00000004951 | Hspb1 | -1.04449 | 0.00776 | down |
| ENSMUSG00000032311 | Nrg4 | 1.437648 | 0.00774 | up |
| ENSMUSG00000036931 | Nfkbid | -2.20496 | 0.00772 | down |
| ENSMUSG00000042705 | Commd10 | 1.161684 | 0.00772 | up |
| ENSMUSG00000031574 | Star | -1.4069 | 0.00769 | down |
| ENSMUSG00000105931 | Gm43014 | -1.44626 | 0.00769 | down |
| ENSMUSG00000023971 | Rrp36 | 1.124812 | 0.00768 | up |
| ENSMUSG00000089712 | Gm15889 | -1.65709 | 0.00767 | down |
| ENSMUSG00000086607 | 4930511M06Rik | -1.49905 | 0.00765 | down |
| ENSMUSG00000051705 | Senp8 | 1.416454 | 0.00763 | up |
| ENSMUSG00000070604 | Vsig10l | -1.40394 | 0.00758 | down |
| ENSMUSG00000108483 | Gm45184 | -2.85347 | 0.00758 | down |
| ENSMUSG00000074922 | Fam122a | 1.419289 | 0.00755 | up |
| ENSMUSG00000115575 | Gm49024 | -1.87205 | 0.00753 | down |
| ENSMUSG00000031343 | Gabra3 | -1.41188 | 0.00752 | down |
| ENSMUSG00000068227 | Il2rb | -1.10771 | 0.00748 | down |
| ENSMUSG00000037752 | Xkr8 | 1.037765 | 0.00746 | up |
| ENSMUSG00000044938 | Klhl31 | -2.55917 | 0.00746 | down |
| ENSMUSG00000111274 | Gm47409 | -2.32361 | 0.00746 | down |
| ENSMUSG00000084960 | B430010I23Rik | -2.06523 | 0.00744 | down |
| ENSMUSG00000115317 | Gm32618 | -4.28414 | 0.00744 | down |
| ENSMUSG00000060600 | Eno3 | -1.11125 | 0.00741 | down |
| ENSMUSG00000114961 | A930002C04Rik | 3.073398 | 0.00741 | up |
| ENSMUSG00000111325 | Gm47140 | -1.70367 | 0.00740 | down |
| ENSMUSG00000020649 | Rrm2 | -1.67011 | 0.00739 | down |
| ENSMUSG00000061577 | Adgrg5 | -2.19129 | 0.00739 | down |
| ENSMUSG00000111521 | Gm48529 | -1.04415 | 0.00739 | down |
| ENSMUSG00000058656 | Samd12 | -1.90099 | 0.00738 | down |
| ENSMUSG00000110902 | Gm33104 | -4.14856 | 0.00738 | down |
| ENSMUSG00000115210 | Gm49308 | -3.63548 | 0.00738 | down |
| ENSMUSG00000086754 | Gm16098 | 2.704565 | 0.00737 | up |
| ENSMUSG00000114462 | Gm8371 | 1.541804 | 0.00736 | up |
| ENSMUSG00000021182 | Ccdc88c | -1.13584 | 0.00734 | down |
| ENSMUSG00000083822 | Hmgb1-ps5 | -4.96067 | 0.00733 | down |
| ENSMUSG00000002688 | Prkd1 | -3.71754 | 0.00732 | down |
| ENSMUSG00000091867 | Cyp2a22 | -1.63866 | 0.00732 | down |
| ENSMUSG00000109781 | Gm45509 | -2.13541 | 0.00732 | down |
| ENSMUSG00000033361 | Prrg3 | -1.66425 | 0.00730 | down |
| ENSMUSG00000048620 | Olfr1336 | -1.57539 | 0.00730 | down |
| ENSMUSG00000057329 | Bcl2 | -1.09563 | 0.00730 | down |
| ENSMUSG00000118382 | Gm8373 | -1.87643 | 0.00730 | down |
| ENSMUSG00000028008 | Asic5 | 1.004919 | 0.00728 | up |
| ENSMUSG00000110047 | A230085B16Rik | -2.52695 | 0.00728 | down |
| ENSMUSG00000047502 | Mroh7 | -2.15592 | 0.00726 | down |
| ENSMUSG00000103215 | Gm38388 | 1.970835 | 0.00726 | up |
| ENSMUSG00000027684 | Mecom | -1.62966 | 0.00723 | down |
| ENSMUSG00000120095 | - | -1.71168 | 0.00723 | down |
| ENSMUSG00000003062 | Stard3nl | 1.125756 | 0.00722 | up |
| ENSMUSG00000060416 | Gm839 | 3.500459 | 0.00721 | up |
| ENSMUSG00000038827 | Abitram | 1.139527 | 0.00718 | up |
| ENSMUSG00000090362 | Vmn2r79 | -1.33478 | 0.00717 | down |
| ENSMUSG00000084283 | Gm14914 | -3.71829 | 0.00715 | down |
| ENSMUSG00000108802 | Gm44769 | -3.64108 | 0.00711 | down |
| ENSMUSG00000111605 | 4632418H02Rik | -2.12327 | 0.00711 | down |
| ENSMUSG00000112354 | Gm33843 | -2.31215 | 0.00709 | down |
| ENSMUSG00000111583 | Gm47962 | 3.895233 | 0.00707 | up |
| ENSMUSG00000113548 | Gm34047 | 3.121714 | 0.00707 | up |
| ENSMUSG00000089995 | Gm15716 | -2.62849 | 0.00705 | down |
| ENSMUSG00000020319 | Wdpcp | 1.692314 | 0.00699 | up |
| ENSMUSG00000073491 | Ifi213 | -1.64229 | 0.00699 | down |
| ENSMUSG00000117912 | Gm50383 | -3.51668 | 0.00698 | down |
| ENSMUSG00000031450 | Grk1 | -1.78238 | 0.00693 | down |
| ENSMUSG00000094156 | Sult2a7 | 2.287745 | 0.00692 | up |
| ENSMUSG00000102748 | Pcdhgb2 | 2.707611 | 0.00692 | up |
| ENSMUSG00000097439 | Gm16754 | -1.16726 | 0.00688 | down |
| ENSMUSG00000100937 | Nscme3l | -4.26742 | 0.00686 | down |
| ENSMUSG00000028497 | Hacd4 | -1.44755 | 0.00685 | down |
| ENSMUSG00000074024 | 4632427E13Rik | -1.34399 | 0.00684 | down |
| ENSMUSG00000018648 | Dusp14 | -1.38169 | 0.00681 | down |
| ENSMUSG00000022194 | Pabpn1 | -1.5001 | 0.00675 | down |
| ENSMUSG00000094950 | Vmn2r66 | -3.92557 | 0.00675 | down |
| ENSMUSG00000018486 | Wnt9b | -1.04595 | 0.00672 | down |
| ENSMUSG00000022032 | Scara5 | 2.122856 | 0.00671 | up |
| ENSMUSG00000121227 | - | 1.706009 | 0.00665 | up |
| ENSMUSG00000031489 | Adrb3 | -1.07763 | 0.00664 | down |
| ENSMUSG00000020546 | Stxbp4 | -1.05385 | 0.00663 | down |
| ENSMUSG00000031209 | Heph | -1.63808 | 0.00662 | down |
| ENSMUSG00000022508 | Bcl6 | -1.01328 | 0.00661 | down |
| ENSMUSG00000089978 | Crb1-ps | -2.7185 | 0.00659 | down |
| ENSMUSG00000104434 | Gm37421 | 3.464865 | 0.00659 | up |
| ENSMUSG00000031647 | Mfap3l | -2.66506 | 0.00651 | down |
| ENSMUSG00000042109 | Csdc2 | -3.10228 | 0.00645 | down |
| ENSMUSG00000062526 | Mppe1 | 1.01734 | 0.00644 | up |
| ENSMUSG00000085887 | Arhgap27os3 | -3.85366 | 0.00644 | down |
| ENSMUSG00000100706 | Gm19744 | 3.752434 | 0.00639 | up |
| ENSMUSG00000120872 | - | -2.25824 | 0.00639 | down |
| ENSMUSG00000049929 | Lpar4 | -1.84491 | 0.00636 | down |
| ENSMUSG00000056608 | Chd9 | 1.602474 | 0.00635 | up |
| ENSMUSG00000083929 | Gm10600 | -4.90726 | 0.00633 | down |
| ENSMUSG00000049130 | C5ar1 | 1.283166 | 0.00632 | up |
| ENSMUSG00000085189 | Gm11963 | 1.065661 | 0.00632 | up |
| ENSMUSG00000027327 | 1700037H04Rik | 1.000156 | 0.00631 | up |
| ENSMUSG00000056643 | Chst13 | 1.25395 | 0.00628 | up |
| ENSMUSG00000078956 | Gm14221 | -1.87937 | 0.00626 | down |
| ENSMUSG00000078886 | Gm2026 | -1.77832 | 0.00623 | down |
| ENSMUSG00000093668 | Pou5f2 | -4.5166 | 0.00623 | down |
| ENSMUSG00000066538 | Gm6254 | -1.36198 | 0.00618 | down |
| ENSMUSG00000115520 | Gm41335 | -4.19584 | 0.00616 | down |
| ENSMUSG00000028195 | Ccn1 | -1.19807 | 0.00611 | down |
| ENSMUSG00000051190 | Olfr1356 | -1.76806 | 0.00609 | down |
| ENSMUSG00000047517 | Dmbt1 | 3.959924 | 0.00607 | up |
| ENSMUSG00000092563 | Gm3617 | -3.92427 | 0.00606 | down |
| ENSMUSG00000023052 | Npff | -1.59042 | 0.00605 | down |
| ENSMUSG00000039264 | Gimap3 | -1.05463 | 0.00605 | down |
| ENSMUSG00000020038 | Cry1 | -1.13102 | 0.00598 | down |
| ENSMUSG00000075025 | Gm10804 | -3.34882 | 0.00598 | down |
| ENSMUSG00000107577 | Gm44103 | -1.19713 | 0.00597 | down |
| ENSMUSG00000004661 | Arid3b | -1.00825 | 0.00596 | down |
| ENSMUSG00000028044 | Cks1b | 1.319046 | 0.00594 | up |
| ENSMUSG00000082676 | Gm11843 | 2.351364 | 0.00591 | up |
| ENSMUSG00000117079 | Gm41611 | -2.0167 | 0.00590 | down |
| ENSMUSG00000096463 | Gm21750 | -1.34536 | 0.00587 | down |
| ENSMUSG00000103085 | Gm38120 | -2.31387 | 0.00586 | down |
| ENSMUSG00000035401 | Emsy | -1.11808 | 0.00585 | down |
| ENSMUSG00000063060 | Sox7 | -1.785 | 0.00585 | down |
| ENSMUSG00000110588 | Gm45774 | 2.999531 | 0.00585 | up |
| ENSMUSG00000105160 | A530030E21Rik | -2.15739 | 0.00582 | down |
| ENSMUSG00000028717 | Tal1 | -1.12104 | 0.00580 | down |
| ENSMUSG00000117390 | Gm50080 | -1.94011 | 0.00580 | down |
| ENSMUSG00000112639 | A730063M14Rik | -1.09243 | 0.00579 | down |
| ENSMUSG00000000317 | Bcl6b | -1.27308 | 0.00578 | down |
| ENSMUSG00000118672 | Muc4 | 4.626989 | 0.00578 | up |
| ENSMUSG00000039193 | Nlrc4 | -1.04325 | 0.00577 | down |
| ENSMUSG00000032690 | Oas2 | -1.0393 | 0.00575 | down |
| ENSMUSG00000060467 | Gm10080 | -3.44189 | 0.00572 | down |
| ENSMUSG00000026042 | Col5a2 | -1.19158 | 0.00563 | down |
| ENSMUSG00000067038 | Rps12-ps3 | -1.46136 | 0.00561 | down |
| ENSMUSG00000002625 | Akap8l | -1.01981 | 0.00557 | down |
| ENSMUSG00000033420 | Antxr1 | -1.55053 | 0.00557 | down |
| ENSMUSG00000058297 | Spock2 | -2.86514 | 0.00557 | down |
| ENSMUSG00000120442 | - | -1.5727 | 0.00557 | down |
| ENSMUSG00000033882 | Rbm46 | -5.02998 | 0.00551 | down |
| ENSMUSG00000029442 | Wdr66 | -1.05793 | 0.00550 | down |
| ENSMUSG00000048826 | Dact2 | 1.245168 | 0.00549 | up |
| ENSMUSG00000086189 | Gm15462 | -1.97201 | 0.00548 | down |
| ENSMUSG00000120574 | - | -2.38793 | 0.00545 | down |
| ENSMUSG00000023913 | Pla2g7 | 1.115907 | 0.00544 | up |
| ENSMUSG00000047022 | Mipol1 | -1.66457 | 0.00544 | down |
| ENSMUSG00000087179 | 5730471H19Rik | -1.75624 | 0.00543 | down |
| ENSMUSG00000120121 | - | -3.37287 | 0.00541 | down |
| ENSMUSG00000051166 | Eml5 | -1.26265 | 0.00538 | down |
| ENSMUSG00000054808 | Actn4 | -1.01021 | 0.00537 | down |
| ENSMUSG00000053693 | Mast1 | -2.20704 | 0.00535 | down |
| ENSMUSG00000026639 | Lamb3 | 1.215098 | 0.00534 | up |
| ENSMUSG00000084416 | Rpl10a-ps1 | 1.903261 | 0.00534 | up |
| ENSMUSG00000087143 | A830082K12Rik | -3.1374 | 0.00530 | down |
| ENSMUSG00000110156 | Gm42067 | -1.09401 | 0.00528 | down |
| ENSMUSG00000023087 | Noct | -1.05279 | 0.00526 | down |
| ENSMUSG00000027199 | Gatm | -1.11358 | 0.00526 | down |
| ENSMUSG00000052783 | Grk4 | -1.12509 | 0.00526 | down |
| ENSMUSG00000004018 | Fancl | 1.274044 | 0.00524 | up |
| ENSMUSG00000019235 | Rps6kl1 | -1.13946 | 0.00524 | down |
| ENSMUSG00000057068 | Fam47e | 1.226549 | 0.00524 | up |
| ENSMUSG00000115026 | Gm49041 | 3.690512 | 0.00524 | up |
| ENSMUSG00000029417 | Cxcl9 | -1.29244 | 0.00519 | down |
| ENSMUSG00000035228 | Ccdc106 | -4.24977 | 0.00514 | down |
| ENSMUSG00000020205 | Phlda1 | -1.39593 | 0.00513 | down |
| ENSMUSG00000106706 | C530043K16Rik | -1.6911 | 0.00512 | down |
| ENSMUSG00000051314 | Ffar2 | 1.958304 | 0.00510 | up |
| ENSMUSG00000085399 | Foxd2os | -2.21286 | 0.00508 | down |
| ENSMUSG00000045680 | Tcf21 | -1.11958 | 0.00504 | down |
| ENSMUSG00000005338 | Cadm3 | -1.01713 | 0.00503 | down |
| ENSMUSG00000027670 | Ocstamp | 1.832986 | 0.00502 | up |
| ENSMUSG00000040121 | Rep15 | -1.58678 | 0.00501 | down |
| ENSMUSG00000120092 | - | -1.41793 | 0.00501 | down |
| ENSMUSG00000091239 | Vmn2r76 | -2.65614 | 0.00500 | down |
| ENSMUSG00000100005 | B130024G19Rik | -1.40647 | 0.00500 | down |
| ENSMUSG00000120422 | - | -3.41679 | 0.00498 | down |
| ENSMUSG00000073680 | Tmem88b | -1.40611 | 0.00497 | down |
| ENSMUSG00000044359 | P2ry4 | -2.24703 | 0.00492 | down |
| ENSMUSG00000121490 | C920006O11Rik | -1.01222 | 0.00492 | down |
| ENSMUSG00000023906 | Cldn6 | -1.87175 | 0.00491 | down |
| ENSMUSG00000071551 | Akr1c19 | -1.28069 | 0.00488 | down |
| ENSMUSG00000102326 | Gm37788 | -1.57787 | 0.00488 | down |
| ENSMUSG00000106507 | Gm43056 | -3.19542 | 0.00486 | down |
| ENSMUSG00000035373 | Ccl7 | 4.527947 | 0.00485 | up |
| ENSMUSG00000105201 | Gm43362 | -2.32035 | 0.00485 | down |
| ENSMUSG00000078453 | Abracl | 1.14415 | 0.00483 | up |
| ENSMUSG00000106992 | Gm43167 | -1.57349 | 0.00481 | down |
| ENSMUSG00000089417 | Gm22009 | -1.09517 | 0.00480 | down |
| ENSMUSG00000032415 | Ube2cbp | 2.204077 | 0.00477 | up |
| ENSMUSG00000072572 | Slc39a2 | -1.33389 | 0.00477 | down |
| ENSMUSG00000120630 | - | -1.8273 | 0.00477 | down |
| ENSMUSG00000022360 | Atad2 | -1.23063 | 0.00473 | down |
| ENSMUSG00000035042 | Ccl5 | -1.15568 | 0.00473 | down |
| ENSMUSG00000051279 | Gdf6 | -2.4146 | 0.00473 | down |
| ENSMUSG00000033031 | Cip2a | -1.61507 | 0.00468 | down |
| ENSMUSG00000079507 | H2-Q1 | -1.2241 | 0.00468 | down |
| ENSMUSG00000041889 | Shisa4 | -2.28319 | 0.00467 | down |
| ENSMUSG00000097891 | Gm3650 | -1.03044 | 0.00467 | down |
| ENSMUSG00000075307 | Klhl41 | -2.34327 | 0.00465 | down |
| ENSMUSG00000025537 | Phkg1 | -2.18524 | 0.00464 | down |
| ENSMUSG00000053158 | Fes | -1.17521 | 0.00464 | down |
| ENSMUSG00000091076 | Vmn2r115 | -1.39263 | 0.00464 | down |
| ENSMUSG00000023918 | Adgrf4 | -3.32839 | 0.00463 | down |
| ENSMUSG00000120031 | - | 1.821087 | 0.00463 | up |
| ENSMUSG00000108494 | Gm45203 | -1.45692 | 0.00462 | down |
| ENSMUSG00000064360 | mt-Nd3 | -1.73708 | 0.00457 | down |
| ENSMUSG00000068923 | Syt11 | -1.06206 | 0.00456 | down |
| ENSMUSG00000097055 | Gm4419 | -1.91296 | 0.00454 | down |
| ENSMUSG00000078234 | Klhdc7a | 1.084894 | 0.00453 | up |
| ENSMUSG00000112576 | Gm47621 | -1.47587 | 0.00450 | down |
| ENSMUSG00000113921 | Gm48054 | -1.44205 | 0.00450 | down |
| ENSMUSG00000051008 | 4930412M03Rik | 2.622775 | 0.00447 | up |
| ENSMUSG00000115184 | Gm49197 | -2.79166 | 0.00443 | down |
| ENSMUSG00000058331 | Zfp85 | -1.13143 | 0.00442 | down |
| ENSMUSG00000113722 | Snhg10 | -1.78411 | 0.00441 | down |
| ENSMUSG00000056987 | Garin2 | -3.62297 | 0.00440 | down |
| ENSMUSG00000051184 | Zfp524 | 1.09038 | 0.00439 | up |
| ENSMUSG00000068606 | Gm4841 | -2.69261 | 0.00439 | down |
| ENSMUSG00000027577 | Chrna4 | -3.88607 | 0.00438 | down |
| ENSMUSG00000031896 | Ctrl | -3.14969 | 0.00433 | down |
| ENSMUSG00000048583 | Igf2 | -1.45589 | 0.00432 | down |
| ENSMUSG00000041608 | Entpd3 | 3.687268 | 0.00426 | up |
| ENSMUSG00000091119 | Ccdc152 | -2.47574 | 0.00425 | down |
| ENSMUSG00000062939 | Stat4 | -2.35391 | 0.00423 | down |
| ENSMUSG00000038208 | Pgap3 | 1.7491 | 0.00422 | up |
| ENSMUSG00000097167 | Gm16740 | 1.002285 | 0.00420 | up |
| ENSMUSG00000083087 | Gm11249 | 3.666282 | 0.00418 | up |
| ENSMUSG00000039813 | Tbc1d2 | 1.770269 | 0.00416 | up |
| ENSMUSG00000027404 | Snrpb | 1.058556 | 0.00415 | up |
| ENSMUSG00000120967 | - | 1.823958 | 0.00415 | up |
| ENSMUSG00000078919 | Dpm1 | 1.134228 | 0.00413 | up |
| ENSMUSG00000030905 | Crym | 3.286948 | 0.00409 | up |
| ENSMUSG00000044737 | Klk14 | -2.28646 | 0.00408 | down |
| ENSMUSG00000103039 | Gm37123 | -1.6565 | 0.00408 | down |
| ENSMUSG00000109877 | Gm45609 | -2.91391 | 0.00408 | down |
| ENSMUSG00000038599 | Capn8 | 1.705397 | 0.00407 | up |
| ENSMUSG00000048997 | Atxn7l2 | -1.27884 | 0.00405 | down |
| ENSMUSG00000036718 | Micall2 | -1.16329 | 0.00404 | down |
| ENSMUSG00000022025 | Cnmd | -2.21163 | 0.00403 | down |
| ENSMUSG00000097343 | 9030407P20Rik | 2.014276 | 0.00403 | up |
| ENSMUSG00000111938 | 2900045O20Rik | -3.23583 | 0.00403 | down |
| ENSMUSG00000114875 | Gm30363 | -3.76335 | 0.00401 | down |
| ENSMUSG00000020623 | Map2k6 | -1.25172 | 0.00400 | down |
| ENSMUSG00000029544 | Cabp1 | -2.5773 | 0.00400 | down |
| ENSMUSG00000016496 | Cd274 | -1.27562 | 0.00398 | down |
| ENSMUSG00000025092 | Hspa12a | -1.28723 | 0.00397 | down |
| ENSMUSG00000028133 | Rwdd3 | -1.08914 | 0.00397 | down |
| ENSMUSG00000059060 | Rad51b | 1.711065 | 0.00397 | up |
| ENSMUSG00000076498 | Trbc2 | -1.39405 | 0.00397 | down |
| ENSMUSG00000000204 | Slfn4 | 2.542228 | 0.00396 | up |
| ENSMUSG00000025014 | Dntt | 4.15533 | 0.00396 | up |
| ENSMUSG00000112461 | Gm47625 | -3.00406 | 0.00396 | down |
| ENSMUSG00000104867 | Gm43728 | -3.04724 | 0.00390 | down |
| ENSMUSG00000030643 | Rab30 | 1.002982 | 0.00388 | up |
| ENSMUSG00000057722 | Lepr | 1.61321 | 0.00388 | up |
| ENSMUSG00000030079 | Ruvbl1 | 1.000501 | 0.00383 | up |
| ENSMUSG00000022708 | Zbtb20 | -1.38793 | 0.00382 | down |
| ENSMUSG00000111771 | Gm47465 | 1.494329 | 0.00382 | up |
| ENSMUSG00000066122 | Olfr45 | -1.99052 | 0.00381 | down |
| ENSMUSG00000096929 | A330023F24Rik | -1.05325 | 0.00381 | down |
| ENSMUSG00000031403 | Dkc1 | -1.05965 | 0.00380 | down |
| ENSMUSG00000096351 | Samd11 | -5.09201 | 0.00380 | down |
| ENSMUSG00000040183 | Ankrd6 | -3.12648 | 0.00379 | down |
| ENSMUSG00000028194 | Ddah1 | -1.03097 | 0.00376 | down |
| ENSMUSG00000110266 | Gm32742 | -2.1591 | 0.00376 | down |
| ENSMUSG00000026637 | Traf5 | -1.13344 | 0.00374 | down |
| ENSMUSG00000043079 | Synpo | 1.125148 | 0.00374 | up |
| ENSMUSG00000030413 | Pglyrp1 | 2.562159 | 0.00373 | up |
| ENSMUSG00000027628 | Aar2 | 1.127581 | 0.00372 | up |
| ENSMUSG00000024029 | Tff3 | 1.738176 | 0.00370 | up |
| ENSMUSG00000111828 | D830035M03Rik | 2.597391 | 0.00370 | up |
| ENSMUSG00000040061 | Plcb2 | -1.14337 | 0.00369 | down |
| ENSMUSG00000029376 | Mthfd2l | -1.18154 | 0.00366 | down |
| ENSMUSG00000115276 | 9930017N22Rik | -1.31716 | 0.00362 | down |
| ENSMUSG00000105095 | 8430422M14Rik | -1.42661 | 0.00361 | down |
| ENSMUSG00000049411 | Tmem241 | 1.827561 | 0.00360 | up |
| ENSMUSG00000029778 | Adcyap1r1 | -1.39095 | 0.00356 | down |
| ENSMUSG00000075204 | Olfr1039 | -2.16316 | 0.00355 | down |
| ENSMUSG00000109162 | 2900027M19Rik | -1.32837 | 0.00355 | down |
| ENSMUSG00000108431 | Gm42375 | -3.9964 | 0.00351 | down |
| ENSMUSG00000026023 | Cdk15 | -1.76083 | 0.00350 | down |
| ENSMUSG00000060950 | Trmt61a | 1.186502 | 0.00350 | up |
| ENSMUSG00000100862 | Gm10925 | -1.39523 | 0.00350 | down |
| ENSMUSG00000104388 | Gm37033 | -2.33271 | 0.00350 | down |
| ENSMUSG00000107306 | Gm42577 | -1.26893 | 0.00350 | down |
| ENSMUSG00000032098 | Treh | 2.581701 | 0.00349 | up |
| ENSMUSG00000054764 | Mtnr1a | 2.562809 | 0.00348 | up |
| ENSMUSG00000111544 | 4930534H03Rik | -1.77179 | 0.00348 | down |
| ENSMUSG00000028007 | Snx7 | 1.055132 | 0.00347 | up |
| ENSMUSG00000031534 | Smim19 | 1.329718 | 0.00347 | up |
| ENSMUSG00000062933 | Gm10123 | 1.20442 | 0.00347 | up |
| ENSMUSG00000105207 | Gm42927 | -1.16778 | 0.00347 | down |
| ENSMUSG00000117634 | Gm50069 | -1.44306 | 0.00344 | down |
| ENSMUSG00000113924 | Gm48493 | -1.25409 | 0.00343 | down |
| ENSMUSG00000030124 | Lag3 | -2.20047 | 0.00341 | down |
| ENSMUSG00000108046 | Gm43924 | -4.90822 | 0.00341 | down |
| ENSMUSG00000019960 | Dusp6 | -1.02871 | 0.00339 | down |
| ENSMUSG00000046417 | Lrrc75a | 1.583344 | 0.00339 | up |
| ENSMUSG00000113961 | Gm48498 | -1.14128 | 0.00339 | down |
| ENSMUSG00000076613 | Ighg2b | -1.53467 | 0.00338 | down |
| ENSMUSG00000032796 | Lama1 | -1.12729 | 0.00335 | down |
| ENSMUSG00000038768 | 9130409I23Rik | -2.54986 | 0.00334 | down |
| ENSMUSG00000107451 | Gm44421 | -1.87465 | 0.00332 | down |
| ENSMUSG00000106164 | 9430085M18Rik | 1.727051 | 0.00331 | up |
| ENSMUSG00000067736 | Gm10222 | -1.75546 | 0.00330 | down |
| ENSMUSG00000020604 | Arsg | 1.172202 | 0.00328 | up |
| ENSMUSG00000028463 | Car9 | 4.312119 | 0.00328 | up |
| ENSMUSG00000104713 | Gbp6 | -1.03585 | 0.00328 | down |
| ENSMUSG00000029470 | P2rx4 | -1.05375 | 0.00327 | down |
| ENSMUSG00000113095 | Gm7969 | -7.65825 | 0.00325 | down |
| ENSMUSG00000025867 | Cplx2 | -1.3533 | 0.00322 | down |
| ENSMUSG00000034780 | B3galt1 | 1.111519 | 0.00319 | up |
| ENSMUSG00000022816 | Fstl1 | -1.20029 | 0.00318 | down |
| ENSMUSG00000039617 | Gm7488 | 4.626653 | 0.00318 | up |
| ENSMUSG00000050555 | Hyls1 | -1.05955 | 0.00318 | down |
| ENSMUSG00000059429 | Olfr365 | -1.09198 | 0.00318 | down |
| ENSMUSG00000067916 | Zfp991 | -1.44937 | 0.00318 | down |
| ENSMUSG00000029499 | Pxmp2 | 1.018878 | 0.00317 | up |
| ENSMUSG00000028476 | Reck | -1.30447 | 0.00316 | down |
| ENSMUSG00000043822 | Adamtsl5 | -1.47445 | 0.00314 | down |
| ENSMUSG00000064367 | mt-Nd5 | -1.62192 | 0.00314 | down |
| ENSMUSG00000115856 | Gm18095 | -5.23678 | 0.00314 | down |
| ENSMUSG00000052417 | Olfr720 | -3.01907 | 0.00313 | down |
| ENSMUSG00000120260 | - | 2.18023 | 0.00313 | up |
| ENSMUSG00000021552 | Gkap1 | 1.01423 | 0.00311 | up |
| ENSMUSG00000042104 | Uggt2 | 1.430851 | 0.00311 | up |
| ENSMUSG00000039043 | Arpin | 1.349918 | 0.00309 | up |
| ENSMUSG00000108521 | Gm44639 | -2.20459 | 0.00309 | down |
| ENSMUSG00000112304 | Gm35206 | -4.50774 | 0.00307 | down |
| ENSMUSG00000026083 | Eif5b | -1.11724 | 0.00305 | down |
| ENSMUSG00000079457 | Gm7609 | -1.85959 | 0.00305 | down |
| ENSMUSG00000050538 | B230217C12Rik | 2.004492 | 0.00304 | up |
| ENSMUSG00000041974 | Spidr | 2.348558 | 0.00301 | up |
| ENSMUSG00000100257 | C4bp-ps1 | -2.09289 | 0.00301 | down |
| ENSMUSG00000101059 | Gm4017 | -1.87577 | 0.00300 | down |
| ENSMUSG00000012187 | Mogat1 | 3.217664 | 0.00298 | up |
| ENSMUSG00000026073 | Il1r2 | 3.897604 | 0.00298 | up |
| ENSMUSG00000033799 | Tasor2 | -1.04837 | 0.00298 | down |
| ENSMUSG00000025978 | Rftn2 | -1.26336 | 0.00297 | down |
| ENSMUSG00000090799 | Klhl33 | -2.02215 | 0.00296 | down |
| ENSMUSG00000120025 | - | -3.81152 | 0.00294 | down |
| ENSMUSG00000033857 | Engase | 1.041401 | 0.00291 | up |
| ENSMUSG00000108750 | Gm44750 | -3.50275 | 0.00291 | down |
| ENSMUSG00000114989 | Gm49303 | -4.04637 | 0.00290 | down |
| ENSMUSG00000035506 | Slc12a8 | -1.79774 | 0.00289 | down |
| ENSMUSG00000025597 | Klhl4 | -2.56704 | 0.00287 | down |
| ENSMUSG00000050350 | Gpr18 | -3.35541 | 0.00287 | down |
| ENSMUSG00000097787 | 2700046G09Rik | 1.581968 | 0.00287 | up |
| ENSMUSG00000003228 | Grk5 | -1.13043 | 0.00285 | down |
| ENSMUSG00000106634 | Gm43042 | -1.15056 | 0.00285 | down |
| ENSMUSG00000031355 | Arhgap6 | -1.28869 | 0.00282 | down |
| ENSMUSG00000043923 | Ccdc84 | -1.03583 | 0.00282 | down |
| ENSMUSG00000044951 | Mylk4 | -1.95071 | 0.00281 | down |
| ENSMUSG00000112542 | Gm47840 | -1.98033 | 0.00280 | down |
| ENSMUSG00000060675 | Plaat3 | 1.129011 | 0.00279 | up |
| ENSMUSG00000103098 | Gm37559 | -4.42703 | 0.00275 | down |
| ENSMUSG00000107624 | Gm44005 | -1.86555 | 0.00274 | down |
| ENSMUSG00000040621 | Gemin8 | 1.487118 | 0.00273 | up |
| ENSMUSG00000107605 | Gm44117 | -2.27972 | 0.00272 | down |
| ENSMUSG00000108573 | Gm44986 | -4.51612 | 0.00272 | down |
| ENSMUSG00000020437 | Myo1g | -1.40189 | 0.00271 | down |
| ENSMUSG00000079669 | Gm17396 | -2.03007 | 0.00269 | down |
| ENSMUSG00000118215 | Vmn1r55 | -1.54574 | 0.00269 | down |
| ENSMUSG00000001227 | Sema6b | 1.08907 | 0.00268 | up |
| ENSMUSG00000038092 | Hsd3b5 | -1.67406 | 0.00268 | down |
| ENSMUSG00000111132 | Gm48142 | -3.42067 | 0.00267 | down |
| ENSMUSG00000041189 | Chrnb1 | -1.54182 | 0.00266 | down |
| ENSMUSG00000107225 | Gm43637 | -1.24158 | 0.00263 | down |
| ENSMUSG00000028295 | Smim8 | 1.474926 | 0.00262 | up |
| ENSMUSG00000104291 | A130071D04Rik | -1.43771 | 0.00261 | down |
| ENSMUSG00000107068 | Gm42742 | 1.059114 | 0.00256 | up |
| ENSMUSG00000028525 | Pde4b | -1.20317 | 0.00254 | down |
| ENSMUSG00000108779 | Gm45691 | -2.45418 | 0.00254 | down |
| ENSMUSG00000085525 | Gm13166 | -2.48595 | 0.00252 | down |
| ENSMUSG00000001467 | Cyp51 | 1.172944 | 0.00251 | up |
| ENSMUSG00000028803 | Nipal3 | 1.096717 | 0.00251 | up |
| ENSMUSG00000062410 | Hsd3b3 | -1.12001 | 0.00251 | down |
| ENSMUSG00000021087 | Rtn1 | -1.74698 | 0.00250 | down |
| ENSMUSG00000064370 | mt-Cytb | -1.51089 | 0.00250 | down |
| ENSMUSG00000015467 | Egfl8 | -1.61608 | 0.00249 | down |
| ENSMUSG00000121374 | - | 1.499637 | 0.00247 | up |
| ENSMUSG00000005763 | Cd247 | -2.83155 | 0.00246 | down |
| ENSMUSG00000064363 | mt-Nd4 | -1.52785 | 0.00246 | down |
| ENSMUSG00000106628 | Gm43558 | -3.85182 | 0.00246 | down |
| ENSMUSG00000087165 | 2010001A14Rik | 1.51066 | 0.00245 | up |
| ENSMUSG00000031429 | Psmd10 | 1.154085 | 0.00244 | up |
| ENSMUSG00000045349 | Sh2d5 | -3.37464 | 0.00244 | down |
| ENSMUSG00000028037 | Ifi44 | -1.9277 | 0.00242 | down |
| ENSMUSG00000034209 | Rasl10a | -4.14829 | 0.00242 | down |
| ENSMUSG00000037617 | Spag1 | -1.34213 | 0.00242 | down |
| ENSMUSG00000107529 | Gm44291 | -1.59826 | 0.00242 | down |
| ENSMUSG00000115946 | Mirt2 | 2.292853 | 0.00242 | up |
| ENSMUSG00000103285 | Gm37274 | -2.32249 | 0.00241 | down |
| ENSMUSG00000085433 | Gm16001 | -1.09089 | 0.00240 | down |
| ENSMUSG00000030871 | Ears2 | 1.016735 | 0.00238 | up |
| ENSMUSG00000005583 | Mef2c | -1.26533 | 0.00237 | down |
| ENSMUSG00000027133 | Nop10 | 1.023121 | 0.00237 | up |
| ENSMUSG00000029561 | Oasl2 | -1.03655 | 0.00237 | down |
| ENSMUSG00000104211 | Gm37985 | -1.26662 | 0.00237 | down |
| ENSMUSG00000022661 | Cd200 | -1.23088 | 0.00236 | down |
| ENSMUSG00000074052 | BC048644 | -1.62324 | 0.00236 | down |
| ENSMUSG00000104377 | Gm37515 | -1.27263 | 0.00236 | down |
| ENSMUSG00000111375 | Btbd8 | -1.36308 | 0.00236 | down |
| ENSMUSG00000027583 | Zbtb46 | -1.07806 | 0.00233 | down |
| ENSMUSG00000042066 | Tmcc2 | -1.08688 | 0.00233 | down |
| ENSMUSG00000026657 | Frmd4a | -1.06766 | 0.00232 | down |
| ENSMUSG00000033618 | Map3k13 | -1.04014 | 0.00232 | down |
| ENSMUSG00000069668 | Sult3a1 | 3.903138 | 0.00232 | up |
| ENSMUSG00000106863 | Gm42109 | -2.3043 | 0.00231 | down |
| ENSMUSG00000044646 | Zbtb7c | 2.172831 | 0.00230 | up |
| ENSMUSG00000045094 | Arhgef37 | -1.21165 | 0.00230 | down |
| ENSMUSG00000051998 | Lax1 | -1.8006 | 0.00229 | down |
| ENSMUSG00000120337 | - | -3.5793 | 0.00227 | down |
| ENSMUSG00000046782 | Ttc6 | -1.68816 | 0.00224 | down |
| ENSMUSG00000102070 | Gm28661 | -1.55936 | 0.00224 | down |
| ENSMUSG00000005952 | Trpv1 | -2.68673 | 0.00222 | down |
| ENSMUSG00000052920 | Prkg1 | -1.71206 | 0.00222 | down |
| ENSMUSG00000059939 | 9430015G10Rik | -1.07673 | 0.00222 | down |
| ENSMUSG00000033502 | Cdc14a | -1.10961 | 0.00221 | down |
| ENSMUSG00000034880 | Mrpl34 | 1.069072 | 0.00221 | up |
| ENSMUSG00000004730 | Adgre1 | -1.51349 | 0.00220 | down |
| ENSMUSG00000059775 | Rps26-ps1 | 2.410398 | 0.00220 | up |
| ENSMUSG00000059136 | Olfr539 | -1.32686 | 0.00219 | down |
| ENSMUSG00000002043 | Trappc6a | 1.040651 | 0.00218 | up |
| ENSMUSG00000006395 | Hyi | -1.07788 | 0.00218 | down |
| ENSMUSG00000043398 | Gpr135 | -1.43059 | 0.00217 | down |
| ENSMUSG00000109094 | Gm44587 | -2.5969 | 0.00217 | down |
| ENSMUSG00000040264 | Gbp2b | -2.26367 | 0.00216 | down |
| ENSMUSG00000024145 | Pigf | 1.149411 | 0.00215 | up |
| ENSMUSG00000023333 | Gcm1 | -2.48966 | 0.00214 | down |
| ENSMUSG00000051499 | Zfp786 | 3.108298 | 0.00214 | up |
| ENSMUSG00000037849 | Ifi206 | -2.24528 | 0.00213 | down |
| ENSMUSG00000089665 | Fcor | 2.345632 | 0.00213 | up |
| ENSMUSG00000015452 | Ager | -2.15882 | 0.00211 | down |
| ENSMUSG00000069805 | Fbp1 | 1.094454 | 0.00210 | up |
| ENSMUSG00000112572 | Gm7530 | 4.494837 | 0.00208 | up |
| ENSMUSG00000002028 | Kmt2a | -1.03701 | 0.00207 | down |
| ENSMUSG00000029605 | Oas1b | -1.59274 | 0.00207 | down |
| ENSMUSG00000043050 | Tnp2 | -5.18786 | 0.00207 | down |
| ENSMUSG00000097318 | 1700007L15Rik | 1.796435 | 0.00205 | up |
| ENSMUSG00000118106 | Gm54639 | 2.408235 | 0.00205 | up |
| ENSMUSG00000037890 | Wdr19 | -1.03235 | 0.00202 | down |
| ENSMUSG00000076937 | Iglc2 | -1.88011 | 0.00202 | down |
| ENSMUSG00000028403 | Zdhhc21 | -1.00446 | 0.00201 | down |
| ENSMUSG00000068394 | Cep152 | -1.12325 | 0.00201 | down |
| ENSMUSG00000031976 | Urb2 | 1.227836 | 0.00200 | up |
| ENSMUSG00000034205 | Loxl2 | -1.1154 | 0.00199 | down |
| ENSMUSG00000050270 | Tmem220 | 1.200324 | 0.00198 | up |
| ENSMUSG00000114886 | Gm48432 | 1.953752 | 0.00198 | up |
| ENSMUSG00000052160 | Pld4 | -1.00209 | 0.00196 | down |
| ENSMUSG00000078768 | Zfp566 | -1.4501 | 0.00195 | down |
| ENSMUSG00000118501 | Gm53048 | -3.73122 | 0.00194 | down |
| ENSMUSG00000058934 | Igf1os | -1.63456 | 0.00193 | down |
| ENSMUSG00000064351 | mt-Co1 | -1.61132 | 0.00193 | down |
| ENSMUSG00000060317 | Acnat2 | 1.034402 | 0.00192 | up |
| ENSMUSG00000108249 | Gm43960 | -2.87228 | 0.00192 | down |
| ENSMUSG00000074219 | Gm10644 | -1.25999 | 0.00190 | down |
| ENSMUSG00000087213 | 2810408I11Rik | -1.43329 | 0.00190 | down |
| ENSMUSG00000038608 | Dock10 | -1.2289 | 0.00189 | down |
| ENSMUSG00000065947 | mt-Nd4l | -1.99391 | 0.00189 | down |
| ENSMUSG00000015944 | Castor2 | -1.05802 | 0.00185 | down |
| ENSMUSG00000120314 | - | -1.47015 | 0.00185 | down |
| ENSMUSG00000090778 | Gm3235 | -1.74219 | 0.00183 | down |
| ENSMUSG00000120258 | - | -2.5894 | 0.00183 | down |
| ENSMUSG00000085028 | Slc2a4rg-ps | -1.23867 | 0.00182 | down |
| ENSMUSG00000046245 | Pilra | -1.17272 | 0.00181 | down |
| ENSMUSG00000048521 | Cxcr6 | -3.31078 | 0.00180 | down |
| ENSMUSG00000115186 | Gm49417 | -1.47598 | 0.00179 | down |
| ENSMUSG00000019863 | Qrsl1 | 1.227791 | 0.00178 | up |
| ENSMUSG00000025507 | Pidd1 | -1.04183 | 0.00178 | down |
| ENSMUSG00000103539 | Gm37834 | -3.14106 | 0.00178 | down |
| ENSMUSG00000121373 | Cyp2c53-ps | -4.1544 | 0.00178 | down |
| ENSMUSG00000028680 | Plk3 | -1.29232 | 0.00177 | down |
| ENSMUSG00000084910 | C630043F03Rik | -1.12323 | 0.00177 | down |
| ENSMUSG00000091227 | Gm3755 | -3.74703 | 0.00177 | down |
| ENSMUSG00000049922 | Slc35c1 | 1.099516 | 0.00176 | up |
| ENSMUSG00000068101 | Cenpm | 1.313871 | 0.00175 | up |
| ENSMUSG00000041037 | Irgq | 1.120674 | 0.00174 | up |
| ENSMUSG00000054414 | Slc30a7 | 1.294559 | 0.00172 | up |
| ENSMUSG00000091151 | Vmn1r224 | -2.63285 | 0.00172 | down |
| ENSMUSG00000022432 | Smc1b | -3.87022 | 0.00171 | down |
| ENSMUSG00000028643 | Svbp | 1.051109 | 0.00170 | up |
| ENSMUSG00000109787 | Gm45286 | -1.82205 | 0.00170 | down |
| ENSMUSG00000028238 | Atp6v0d2 | 1.55551 | 0.00168 | up |
| ENSMUSG00000030827 | Fgf21 | 2.949111 | 0.00168 | up |
| ENSMUSG00000024067 | Dpy30 | 1.016964 | 0.00167 | up |
| ENSMUSG00000075012 | Fjx1 | 3.149789 | 0.00166 | up |
| ENSMUSG00000019775 | Rgs17 | -2.45315 | 0.00164 | down |
| ENSMUSG00000025902 | Sox17 | -1.08382 | 0.00164 | down |
| ENSMUSG00000026333 | Gin1 | -1.07785 | 0.00162 | down |
| ENSMUSG00000028549 | Itgb3bp | 2.077878 | 0.00161 | up |
| ENSMUSG00000031860 | Pbx4 | -2.97425 | 0.00160 | down |
| ENSMUSG00000043241 | Upf2 | -1.02702 | 0.00160 | down |
| ENSMUSG00000052544 | St6galnac3 | -1.5809 | 0.00160 | down |
| ENSMUSG00000035202 | Lars2 | -1.11226 | 0.00158 | down |
| ENSMUSG00000032449 | Slc25a36 | -1.01326 | 0.00157 | down |
| ENSMUSG00000120947 | - | -4.5022 | 0.00156 | down |
| ENSMUSG00000066037 | Hnrnpr | -1.14386 | 0.00155 | down |
| ENSMUSG00000113161 | Gm47457 | -1.48733 | 0.00155 | down |
| ENSMUSG00000043943 | Naalad2 | -2.30439 | 0.00154 | down |
| ENSMUSG00000043310 | Olfr571 | -1.54337 | 0.00153 | down |
| ENSMUSG00000040829 | Zmynd15 | -1.16472 | 0.00151 | down |
| ENSMUSG00000056055 | Sag | -3.53281 | 0.00151 | down |
| ENSMUSG00000031604 | Msmo1 | 1.277611 | 0.00150 | up |
| ENSMUSG00000066361 | Serpina3c | 2.26542 | 0.00150 | up |
| ENSMUSG00000097451 | Rian | -1.63407 | 0.00150 | down |
| ENSMUSG00000019876 | Pkib | -1.37171 | 0.00149 | down |
| ENSMUSG00000102593 | Gm38384 | -3.67829 | 0.00148 | down |
| ENSMUSG00000030887 | Pdzd9 | -2.25052 | 0.00147 | down |
| ENSMUSG00000029167 | Ppargc1a | -1.06209 | 0.00146 | down |
| ENSMUSG00000031373 | Car5b | -1.23469 | 0.00146 | down |
| ENSMUSG00000037151 | Lrrc20 | 1.177319 | 0.00146 | up |
| ENSMUSG00000110684 | Gm45866 | -1.69443 | 0.00146 | down |
| ENSMUSG00000113427 | Gm46378 | -1.56905 | 0.00146 | down |
| ENSMUSG00000000563 | Atp5pb | 1.019139 | 0.00143 | up |
| ENSMUSG00000027716 | Trpc3 | -2.22336 | 0.00143 | down |
| ENSMUSG00000018927 | Ccl6 | 1.106736 | 0.00142 | up |
| ENSMUSG00000106073 | Gm42892 | -1.86783 | 0.00142 | down |
| ENSMUSG00000111517 | Olfr1238 | -2.44262 | 0.00142 | down |
| ENSMUSG00000120636 | - | -1.24032 | 0.00141 | down |
| ENSMUSG00000074398 | Gm15441 | 3.218434 | 0.00140 | up |
| ENSMUSG00000089940 | Gm4117 | -1.33928 | 0.00140 | down |
| ENSMUSG00000117853 | Vmn1r88 | -1.94644 | 0.00140 | down |
| ENSMUSG00000120368 | - | -1.86418 | 0.00140 | down |
| ENSMUSG00000041012 | Cmtm8 | -1.66967 | 0.00139 | down |
| ENSMUSG00000026691 | Fmo3 | -5.33521 | 0.00138 | down |
| ENSMUSG00000097047 | 1110020A21Rik | -1.18487 | 0.00138 | down |
| ENSMUSG00000116284 | Gm3787 | -1.07282 | 0.00138 | down |
| ENSMUSG00000079550 | Mpp4 | -1.06326 | 0.00137 | down |
| ENSMUSG00000106446 | Gm42970 | -2.39156 | 0.00134 | down |
| ENSMUSG00000036957 | Lrfn3 | 1.072557 | 0.00133 | up |
| ENSMUSG00000038623 | Tm6sf1 | -1.19394 | 0.00133 | down |
| ENSMUSG00000023033 | Scn8a | 2.519513 | 0.00132 | up |
| ENSMUSG00000040170 | Fmo2 | -1.17001 | 0.00132 | down |
| ENSMUSG00000005533 | Igf1r | -1.05726 | 0.00131 | down |
| ENSMUSG00000049173 | Myoz3 | -2.68286 | 0.00130 | down |
| ENSMUSG00000099608 | 4933411E06Rik | -1.5864 | 0.00129 | down |
| ENSMUSG00000101111 | Gm28437 | -1.5897 | 0.00129 | down |
| ENSMUSG00000107659 | Gm44170 | 1.067237 | 0.00129 | up |
| ENSMUSG00000108435 | Gm45051 | -1.30912 | 0.00129 | down |
| ENSMUSG00000097061 | 9330151L19Rik | -1.24455 | 0.00128 | down |
| ENSMUSG00000035142 | Nubpl | 2.147224 | 0.00127 | up |
| ENSMUSG00000041912 | Tdrkh | 1.909286 | 0.00127 | up |
| ENSMUSG00000082585 | Gm15387 | -1.14579 | 0.00126 | down |
| ENSMUSG00000021390 | Ogn | -2.15336 | 0.00125 | down |
| ENSMUSG00000025001 | Hells | -2.21465 | 0.00125 | down |
| ENSMUSG00000052131 | Akr1b7 | 2.471532 | 0.00125 | up |
| ENSMUSG00000018623 | Mmp7 | 3.219284 | 0.00121 | up |
| ENSMUSG00000022351 | Sqle | 1.301004 | 0.00121 | up |
| ENSMUSG00000031271 | Serpina7 | 1.222982 | 0.00121 | up |
| ENSMUSG00000093954 | Gm16867 | 3.613147 | 0.00121 | up |
| ENSMUSG00000102275 | Gm37144 | -1.63416 | 0.00121 | down |
| ENSMUSG00000106107 | Gm43190 | -1.26835 | 0.00121 | down |
| ENSMUSG00000068893 | Sprr2a2 | 3.701747 | 0.00120 | up |
| ENSMUSG00000087299 | Gm12953 | -1.56494 | 0.00120 | down |
| ENSMUSG00000035258 | Abi3bp | -1.29176 | 0.00119 | down |
| ENSMUSG00000040624 | Plekhg1 | -1.08106 | 0.00119 | down |
| ENSMUSG00000069045 | Ddx3y | -1.53167 | 0.00118 | down |
| ENSMUSG00000041468 | Gpr12 | -1.93358 | 0.00117 | down |
| ENSMUSG00000078427 | Sarnp | 1.193523 | 0.00117 | up |
| ENSMUSG00000019066 | Rab3d | 1.293271 | 0.00116 | up |
| ENSMUSG00000111923 | Gm34777 | -1.9708 | 0.00115 | down |
| ENSMUSG00000025479 | Cyp2e1 | 1.137597 | 0.00113 | up |
| ENSMUSG00000025134 | Alyref | 1.264257 | 0.00112 | up |
| ENSMUSG00000027505 | Fam209 | -3.55415 | 0.00112 | down |
| ENSMUSG00000045854 | Lyrm2 | 1.031395 | 0.00112 | up |
| ENSMUSG00000064368 | mt-Nd6 | -1.50034 | 0.00112 | down |
| ENSMUSG00000094856 | Gm21962 | -1.4832 | 0.00111 | down |
| ENSMUSG00000099681 | 1700052K11Rik | 1.158154 | 0.00111 | up |
| ENSMUSG00000026820 | Ptges2 | 1.062699 | 0.00110 | up |
| ENSMUSG00000030468 | Siglecg | -1.92163 | 0.00110 | down |
| ENSMUSG00000052934 | Fbxo31 | 1.005956 | 0.00110 | up |
| ENSMUSG00000117148 | Vmn1r229 | -1.14459 | 0.00110 | down |
| ENSMUSG00000001741 | Il16 | -1.01997 | 0.00109 | down |
| ENSMUSG00000028617 | Lrrc42 | 1.122866 | 0.00109 | up |
| ENSMUSG00000070891 | Gm12689 | -2.35926 | 0.00108 | down |
| ENSMUSG00000027824 | Vmn2r1 | -1.2868 | 0.00106 | down |
| ENSMUSG00000038155 | Gstp2 | -2.76409 | 0.00106 | down |
| ENSMUSG00000040415 | Dtx3 | -1.02081 | 0.00106 | down |
| ENSMUSG00000046229 | Scand1 | 2.730464 | 0.00106 | up |
| ENSMUSG00000114836 | Gm18517 | -1.54649 | 0.00106 | down |
| ENSMUSG00000001103 | Sebox | 1.941836 | 0.00105 | up |
| ENSMUSG00000091721 | Gimd1 | 2.335696 | 0.00105 | up |
| ENSMUSG00000009108 | Gnat2 | 1.77158 | 0.00104 | up |
| ENSMUSG00000024843 | Chka | -1.76509 | 0.00104 | down |
| ENSMUSG00000079110 | Capn3 | -2.59375 | 0.00104 | down |
| ENSMUSG00000025650 | Col7a1 | -2.68175 | 0.00103 | down |
| ENSMUSG00000055413 | H2-Q5 | -1.42953 | 0.00101 | down |
| ENSMUSG00000064358 | mt-Co3 | -1.90408 | 0.00101 | down |
| ENSMUSG00000107304 | Gm43775 | -2.57122 | 0.00101 | down |
| ENSMUSG00000064341 | mt-Nd1 | -1.65688 | 0.00100 | down |
| ENSMUSG00000027276 | Jag1 | -1.12766 | 0.00099 | down |
| ENSMUSG00000095199 | Zfp967 | -1.37474 | 0.00099 | down |
| ENSMUSG00000087433 | Gm14167 | 3.182513 | 0.00098 | up |
| ENSMUSG00000110225 | Gm45528 | -1.89032 | 0.00098 | down |
| ENSMUSG00000014030 | Pax5 | -1.9334 | 0.00097 | down |
| ENSMUSG00000038872 | Zfhx3 | -1.20743 | 0.00097 | down |
| ENSMUSG00000069305 | H4c18 | 3.209966 | 0.00097 | up |
| ENSMUSG00000074896 | Ifit3 | -1.4548 | 0.00097 | down |
| ENSMUSG00000083306 | Gm13868 | -2.6252 | 0.00097 | down |
| ENSMUSG00000020334 | Slc22a4 | -1.8507 | 0.00095 | down |
| ENSMUSG00000044950 | Pwwp2a | -1.03906 | 0.00095 | down |
| ENSMUSG00000048406 | B330016D10Rik | -1.52841 | 0.00095 | down |
| ENSMUSG00000117084 | Gm22146 | -1.51526 | 0.00094 | down |
| ENSMUSG00000021537 | Cetn3 | 1.255195 | 0.00093 | up |
| ENSMUSG00000045991 | Onecut2 | -1.04266 | 0.00093 | down |
| ENSMUSG00000071456 | 1110002L01Rik | 1.87868 | 0.00093 | up |
| ENSMUSG00000113795 | Gm48119 | -1.53264 | 0.00093 | down |
| ENSMUSG00000116656 | Gm49708 | 1.947546 | 0.00093 | up |
| ENSMUSG00000120996 | - | 2.338633 | 0.00093 | up |
| ENSMUSG00000007944 | Ttc9b | -4.48508 | 0.00092 | down |
| ENSMUSG00000044165 | Bcl2l15 | -4.12661 | 0.00092 | down |
| ENSMUSG00000062683 | Atp5g2 | 2.726891 | 0.00092 | up |
| ENSMUSG00000002059 | Rab34 | 1.341001 | 0.00091 | up |
| ENSMUSG00000019326 | Aoc3 | -2.04565 | 0.00089 | down |
| ENSMUSG00000079108 | Srp54c | 1.166907 | 0.00089 | up |
| ENSMUSG00000104520 | Gm37336 | -2.43901 | 0.00089 | down |
| ENSMUSG00000007987 | Ift22 | 1.230133 | 0.00088 | up |
| ENSMUSG00000022151 | Ttc33 | 1.208699 | 0.00087 | up |
| ENSMUSG00000032315 | Cyp1a1 | -1.81273 | 0.00087 | down |
| ENSMUSG00000047050 | Olfr914 | -3.42927 | 0.00087 | down |
| ENSMUSG00000038351 | Sgsm2 | -1.04219 | 0.00086 | down |
| ENSMUSG00000055072 | Gm9964 | -5.40233 | 0.00086 | down |
| ENSMUSG00000097911 | Gm26691 | -1.85692 | 0.00086 | down |
| ENSMUSG00000108456 | 4732496C06Rik | 3.230245 | 0.00085 | up |
| ENSMUSG00000112972 | Gm48417 | -2.51818 | 0.00085 | down |
| ENSMUSG00000118607 | Gm7592 | -1.58945 | 0.00085 | down |
| ENSMUSG00000025371 | Chmp6 | 1.22674 | 0.00084 | up |
| ENSMUSG00000050222 | Il17d | -5.5473 | 0.00084 | down |
| ENSMUSG00000105247 | Gm42519 | -1.94258 | 0.00084 | down |
| ENSMUSG00000026229 | Psmd1 | -1.21413 | 0.00083 | down |
| ENSMUSG00000032580 | Rbm5 | -1.15712 | 0.00083 | down |
| ENSMUSG00000000686 | Abhd15 | 1.182737 | 0.00082 | up |
| ENSMUSG00000019823 | Mical1 | -1.27077 | 0.00082 | down |
| ENSMUSG00000039086 | Ss18l1 | -1.07673 | 0.00082 | down |
| ENSMUSG00000097357 | Gm16793 | -2.28425 | 0.00082 | down |
| ENSMUSG00000020458 | Rtn4 | 1.003292 | 0.00081 | up |
| ENSMUSG00000024742 | Fen1 | 2.608879 | 0.00081 | up |
| ENSMUSG00000085417 | Gm13919 | -2.50223 | 0.00081 | down |
| ENSMUSG00000085923 | Gm12781 | 1.207652 | 0.00081 | up |
| ENSMUSG00000018378 | Cuedc1 | -1.07678 | 0.00080 | down |
| ENSMUSG00000022613 | Miox | 3.480274 | 0.00080 | up |
| ENSMUSG00000043068 | Fam89a | 1.510983 | 0.00080 | up |
| ENSMUSG00000064345 | mt-Nd2 | -1.67272 | 0.00080 | down |
| ENSMUSG00002075453 | Snord3b3 | -4.59655 | 0.00080 | down |
| ENSMUSG00000038241 | Cep250 | -1.0102 | 0.00079 | down |
| ENSMUSG00000095959 | Gm10845 | -1.69435 | 0.00079 | down |
| ENSMUSG00000108129 | 4930417O13Rik | -3.3763 | 0.00078 | down |
| ENSMUSG00000110519 | Olfr839 | -1.35655 | 0.00078 | down |
| ENSMUSG00000006398 | Cdc20 | -3.32753 | 0.00077 | down |
| ENSMUSG00000113601 | Gm48735 | -2.87006 | 0.00077 | down |
| ENSMUSG00000110384 | Gm45301 | -3.85574 | 0.00076 | down |
| ENSMUSG00000027530 | Fabp12 | 4.414759 | 0.00075 | up |
| ENSMUSG00000037007 | Zfp113 | -1.17439 | 0.00075 | down |
| ENSMUSG00000053825 | Ppfia2 | -2.60364 | 0.00075 | down |
| ENSMUSG00000102153 | Gm37474 | -2.00812 | 0.00075 | down |
| ENSMUSG00000037411 | Serpine1 | 2.669313 | 0.00074 | up |
| ENSMUSG00000097224 | Gm26716 | -1.71752 | 0.00074 | down |
| ENSMUSG00000117599 | Gm49971 | -1.59344 | 0.00074 | down |
| ENSMUSG00000120012 | - | -1.26236 | 0.00074 | down |
| ENSMUSG00000088252 | Snord13 | -4.24235 | 0.00073 | down |
| ENSMUSG00000097048 | 1600020E01Rik | -1.27201 | 0.00073 | down |
| ENSMUSG00000097673 | Gm26608 | -1.17163 | 0.00073 | down |
| ENSMUSG00000108216 | Gm44153 | -1.92668 | 0.00073 | down |
| ENSMUSG00000026483 | Niban1 | -1.11702 | 0.00071 | down |
| ENSMUSG00000106743 | Gm42847 | -2.45098 | 0.00071 | down |
| ENSMUSG00000018821 | Avpi1 | 1.047457 | 0.00070 | up |
| ENSMUSG00000108105 | Gm5340 | 3.805997 | 0.00070 | up |
| ENSMUSG00000032477 | Cdc25a | 1.008394 | 0.00069 | up |
| ENSMUSG00000041623 | D11Wsu47e | 1.142405 | 0.00069 | up |
| ENSMUSG00000043801 | Oaz1-ps | 1.656594 | 0.00069 | up |
| ENSMUSG00000054702 | Ap1s3 | -1.96024 | 0.00069 | down |
| ENSMUSG00000102432 | Gm29856 | 2.98818 | 0.00069 | up |
| ENSMUSG00000121158 | Gm35162 | -2.9388 | 0.00069 | down |
| ENSMUSG00000021835 | Bmp4 | -1.23979 | 0.00068 | down |
| ENSMUSG00000037685 | Atp8a1 | -1.07173 | 0.00068 | down |
| ENSMUSG00000104394 | Gm37254 | -1.51349 | 0.00068 | down |
| ENSMUSG00000013076 | Amotl1 | -1.37476 | 0.00067 | down |
| ENSMUSG00000034898 | Filip1 | -2.17123 | 0.00067 | down |
| ENSMUSG00000037613 | Tnfrsf23 | 2.337816 | 0.00067 | up |
| ENSMUSG00000027820 | Mme | -1.00725 | 0.00066 | down |
| ENSMUSG00000028837 | Psmb2 | 1.041551 | 0.00066 | up |
| ENSMUSG00000004347 | Pde1c | -2.80186 | 0.00065 | down |
| ENSMUSG00000040441 | Slc26a10 | -1.1272 | 0.00065 | down |
| ENSMUSG00000071550 | Cfap44 | -5.44421 | 0.00065 | down |
| ENSMUSG00000121201 | - | -2.7184 | 0.00065 | down |
| ENSMUSG00000026981 | Il1rn | 2.066158 | 0.00064 | up |
| ENSMUSG00000041483 | Zfp281 | -1.62218 | 0.00064 | down |
| ENSMUSG00000070282 | 3000002C10Rik | 1.005277 | 0.00064 | up |
| ENSMUSG00000112255 | Gm47594 | -2.21683 | 0.00064 | down |
| ENSMUSG00000027239 | Mdk | -1.82688 | 0.00063 | down |
| ENSMUSG00000057375 | Yipf1 | 1.160167 | 0.00063 | up |
| ENSMUSG00000104682 | Gm42636 | -1.88549 | 0.00062 | down |
| ENSMUSG00000032363 | Adamts7 | -1.14777 | 0.00061 | down |
| ENSMUSG00000034659 | Tmem109 | 1.024077 | 0.00061 | up |
| ENSMUSG00000037606 | Osbpl5 | -1.40406 | 0.00061 | down |
| ENSMUSG00000050855 | Zfp940 | -1.68756 | 0.00061 | down |
| ENSMUSG00000112319 | Gm47221 | -1.02311 | 0.00061 | down |
| ENSMUSG00000022961 | Son | -1.06767 | 0.00060 | down |
| ENSMUSG00000108037 | Gm44597 | -1.52668 | 0.00060 | down |
| ENSMUSG00000115105 | Gm49238 | -2.48955 | 0.00060 | down |
| ENSMUSG00000026142 | Rhbdd1 | 1.063145 | 0.00059 | up |
| ENSMUSG00000052331 | Ankrd44 | -1.26592 | 0.00059 | down |
| ENSMUSG00000091019 | Gm7502 | -4.25192 | 0.00059 | down |
| ENSMUSG00000101906 | Mrgprc2-ps | -1.92973 | 0.00059 | down |
| ENSMUSG00000119994 | - | -1.26824 | 0.00059 | down |
| ENSMUSG00000021068 | Nin | -1.3089 | 0.00058 | down |
| ENSMUSG00000039542 | Ncam1 | -1.84214 | 0.00058 | down |
| ENSMUSG00000052707 | Tnrc6a | -1.17046 | 0.00058 | down |
| ENSMUSG00000090236 | Car15 | -2.39907 | 0.00058 | down |
| ENSMUSG00000021057 | Akap5 | -2.48814 | 0.00057 | down |
| ENSMUSG00000022868 | Ahsg | 1.022003 | 0.00057 | up |
| ENSMUSG00000059729 | Olfr1385 | -1.15556 | 0.00057 | down |
| ENSMUSG00000090222 | Ifi203-ps | -2.22348 | 0.00057 | down |
| ENSMUSG00000031637 | Lrp2bp | -1.65207 | 0.00055 | down |
| ENSMUSG00000036168 | Ccdc38 | -2.4687 | 0.00055 | down |
| ENSMUSG00000048280 | Zfp738 | -1.39079 | 0.00055 | down |
| ENSMUSG00000085696 | Hoxaas3 | -3.34097 | 0.00055 | down |
| ENSMUSG00000037278 | Tmem97 | 1.073472 | 0.00054 | up |
| ENSMUSG00000052188 | Gm14964 | -2.22124 | 0.00054 | down |
| ENSMUSG00000057594 | Arl16 | 1.423432 | 0.00053 | up |
| ENSMUSG00000109807 | Gm45244 | -2.21859 | 0.00053 | down |
| ENSMUSG00000017830 | Dhx58 | -1.2715 | 0.00052 | down |
| ENSMUSG00000031380 | Vegfd | -1.65595 | 0.00052 | down |
| ENSMUSG00000032577 | Mapkapk3 | -1.07982 | 0.00052 | down |
| ENSMUSG00000025351 | Cd63 | 1.11561 | 0.00051 | up |
| ENSMUSG00000086443 | 4933421A08Rik | 1.683907 | 0.00051 | up |
| ENSMUSG00000114458 | Gm47551 | -3.08526 | 0.00051 | down |
| ENSMUSG00000022610 | Mapk12 | -1.25694 | 0.00050 | down |
| ENSMUSG00000041439 | Mfsd6 | 1.192204 | 0.00050 | up |
| ENSMUSG00000046532 | Ar | -1.61634 | 0.00050 | down |
| ENSMUSG00000046982 | Tshz1 | -1.03657 | 0.00050 | down |
| ENSMUSG00000058260 | Serpina9 | -3.42824 | 0.00050 | down |
| ENSMUSG00000096688 | Mup17 | -1.36403 | 0.00050 | down |
| ENSMUSG00000106838 | 1810017P11Rik | -1.47984 | 0.00050 | down |
| ENSMUSG00000108884 | Gm45792 | -1.66669 | 0.00050 | down |
| ENSMUSG00000024427 | Spry4 | -1.56861 | 0.00049 | down |
| ENSMUSG00000030967 | Zranb1 | -1.136 | 0.00049 | down |
| ENSMUSG00000047822 | Angptl8 | -1.95324 | 0.00049 | down |
| ENSMUSG00000102758 | Naaladl2 | -2.17785 | 0.00049 | down |
| ENSMUSG00000103475 | Gm37697 | -1.37968 | 0.00049 | down |
| ENSMUSG00000120250 | - | -1.26986 | 0.00049 | down |
| ENSMUSG00000121304 | - | -2.03651 | 0.00049 | down |
| ENSMUSG00000031447 | Lamp1 | 1.034422 | 0.00048 | up |
| ENSMUSG00000050772 | Olfr1124 | -2.27969 | 0.00048 | down |
| ENSMUSG00000053714 | 4732471J01Rik | -1.25352 | 0.00048 | down |
| ENSMUSG00000113684 | Gm48418 | -1.99999 | 0.00048 | down |
| ENSMUSG00000020056 | Washc3 | 1.252722 | 0.00047 | up |
| ENSMUSG00000029797 | Sspo | -2.28498 | 0.00047 | down |
| ENSMUSG00000064339 | mt-Rnr2 | -1.80762 | 0.00047 | down |
| ENSMUSG00000078624 | Olfr613 | -1.04034 | 0.00047 | down |
| ENSMUSG00000081648 | Gm13423 | -2.95957 | 0.00047 | down |
| ENSMUSG00000090655 | Vmn2r120 | -2.08001 | 0.00047 | down |
| ENSMUSG00000097583 | 6430590A07Rik | -2.60533 | 0.00047 | down |
| ENSMUSG00000121029 | - | -2.66644 | 0.00047 | down |
| ENSMUSG00000002900 | Lamb1 | -1.05079 | 0.00046 | down |
| ENSMUSG00000008575 | Nfib | -1.73918 | 0.00046 | down |
| ENSMUSG00000021969 | Zdhhc20 | -1.03672 | 0.00046 | down |
| ENSMUSG00000027217 | Tspan18 | -1.47866 | 0.00046 | down |
| ENSMUSG00000029632 | Ndufa4 | 1.175032 | 0.00046 | up |
| ENSMUSG00000015759 | Cnih1 | 1.142508 | 0.00045 | up |
| ENSMUSG00000029538 | Srsf9 | 1.103728 | 0.00045 | up |
| ENSMUSG00000041654 | Slc39a11 | 1.116879 | 0.00044 | up |
| ENSMUSG00000020519 | Sap30l | 1.002717 | 0.00043 | up |
| ENSMUSG00000037958 | Nsrp1 | -1.20924 | 0.00043 | down |
| ENSMUSG00000038503 | Mesd | 1.023451 | 0.00043 | up |
| ENSMUSG00000069601 | Ank3 | -1.18969 | 0.00043 | down |
| ENSMUSG00000075044 | Slc22a29 | -3.11221 | 0.00043 | down |
| ENSMUSG00000023885 | Thbs2 | -1.18625 | 0.00042 | down |
| ENSMUSG00000028369 | Svep1 | -1.97603 | 0.00042 | down |
| ENSMUSG00000057359 | Gm17494 | -1.50839 | 0.00042 | down |
| ENSMUSG00000074240 | Cib3 | -2.02031 | 0.00042 | down |
| ENSMUSG00000085008 | Dbhos | -1.87335 | 0.00042 | down |
| ENSMUSG00000020774 | Aspa | -1.00789 | 0.00041 | down |
| ENSMUSG00000024642 | Tle4 | -1.07918 | 0.00041 | down |
| ENSMUSG00000027405 | Nop56 | -1.01011 | 0.00041 | down |
| ENSMUSG00000029765 | Plxna4 | -1.22953 | 0.00041 | down |
| ENSMUSG00000062580 | Timm17a | 1.067814 | 0.00041 | up |
| ENSMUSG00000096971 | 4930556M19Rik | 1.089171 | 0.00041 | up |
| ENSMUSG00000108092 | Gm44189 | -1.79894 | 0.00041 | down |
| ENSMUSG00000108155 | Gm44443 | -2.36894 | 0.00041 | down |
| ENSMUSG00000113683 | Gm47123 | 3.072017 | 0.00041 | up |
| ENSMUSG00000028601 | Echdc2 | -1.00767 | 0.00040 | down |
| ENSMUSG00000032679 | Cd59a | -1.07868 | 0.00040 | down |
| ENSMUSG00000041479 | Syt15 | -2.02004 | 0.00040 | down |
| ENSMUSG00000050332 | Amer1 | -1.4058 | 0.00040 | down |
| ENSMUSG00000100510 | Hand2os1 | -1.27846 | 0.00040 | down |
| ENSMUSG00000106044 | Gm42860 | 1.033042 | 0.00040 | up |
| ENSMUSG00000109461 | Gm44848 | -2.43072 | 0.00040 | down |
| ENSMUSG00000008206 | Cers4 | -1.32995 | 0.00039 | down |
| ENSMUSG00000062488 | Ifit3b | -1.91716 | 0.00039 | down |
| ENSMUSG00000081471 | Gm14735 | -1.79101 | 0.00039 | down |
| ENSMUSG00000021815 | Mss51 | -1.9246 | 0.00038 | down |
| ENSMUSG00000027690 | Slc2a2 | -1.00553 | 0.00038 | down |
| ENSMUSG00000057346 | Apol9a | -1.34363 | 0.00038 | down |
| ENSMUSG00000067931 | Zfp948 | -1.18742 | 0.00038 | down |
| ENSMUSG00000111917 | Gm48793 | -2.20845 | 0.00038 | down |
| ENSMUSG00000019647 | Sema6a | -1.11944 | 0.00037 | down |
| ENSMUSG00000024640 | Psat1 | 2.11521 | 0.00037 | up |
| ENSMUSG00000083563 | Gm13340 | -2.55957 | 0.00037 | down |
| ENSMUSG00000103656 | Gm37205 | -5.51904 | 0.00037 | down |
| ENSMUSG00000016541 | Atxn10 | 1.129968 | 0.00036 | up |
| ENSMUSG00000083863 | Gm13341 | -2.12344 | 0.00036 | down |
| ENSMUSG00000111118 | Gm6545 | -2.31695 | 0.00036 | down |
| ENSMUSG00000017747 | Ghdc | -1.40965 | 0.00035 | down |
| ENSMUSG00000019433 | Gipc1 | 1.200055 | 0.00035 | up |
| ENSMUSG00000020638 | Cmpk2 | -1.56126 | 0.00035 | down |
| ENSMUSG00000030086 | Chchd6 | 1.788726 | 0.00035 | up |
| ENSMUSG00000030364 | Clec2h | -2.20333 | 0.00035 | down |
| ENSMUSG00000036158 | Prickle1 | -1.51559 | 0.00035 | down |
| ENSMUSG00000044453 | Ffar1 | -3.49283 | 0.00035 | down |
| ENSMUSG00000066477 | Gm16551 | -1.49833 | 0.00035 | down |
| ENSMUSG00000091382 | Vmn1r18 | -1.68852 | 0.00035 | down |
| ENSMUSG00000107743 | Gm44087 | -1.43639 | 0.00035 | down |
| ENSMUSG00000061829 | Vmn1r214 | -4.48379 | 0.00034 | down |
| ENSMUSG00000085180 | AI838599 | -2.14414 | 0.00034 | down |
| ENSMUSG00000000184 | Ccnd2 | -1.35295 | 0.00033 | down |
| ENSMUSG00000032076 | Cadm1 | -1.01419 | 0.00033 | down |
| ENSMUSG00000054142 | Vmn1r236 | -2.76591 | 0.00033 | down |
| ENSMUSG00000103322 | Gm37404 | -4.91064 | 0.00033 | down |
| ENSMUSG00000018537 | Pcgf2 | -1.04736 | 0.00032 | down |
| ENSMUSG00000037851 | Iars | 1.314341 | 0.00032 | up |
| ENSMUSG00000042369 | Rbm45 | 1.894177 | 0.00032 | up |
| ENSMUSG00000000088 | Cox5a | 1.172226 | 0.00031 | up |
| ENSMUSG00000024462 | Gabbr1 | -1.22973 | 0.00031 | down |
| ENSMUSG00000026494 | Kif26b | 2.290842 | 0.00031 | up |
| ENSMUSG00000041840 | Haus1 | -1.07549 | 0.00031 | down |
| ENSMUSG00000068083 | Cyp2d40 | 1.049874 | 0.00031 | up |
| ENSMUSG00000072884 | Gm10433 | 2.513121 | 0.00031 | up |
| ENSMUSG00000098055 | Gm26947 | -2.3188 | 0.00031 | down |
| ENSMUSG00000103318 | Gm38356 | -1.24615 | 0.00031 | down |
| ENSMUSG00000014349 | Ube2z | 1.042409 | 0.00030 | up |
| ENSMUSG00000105868 | Gm43766 | -1.90483 | 0.00030 | down |
| ENSMUSG00000106040 | Cyp3a63-ps | -2.68656 | 0.00030 | down |
| ENSMUSG00000113630 | 4930404H11Rik | -2.40791 | 0.00030 | down |
| ENSMUSG00000120616 | - | -4.74676 | 0.00030 | down |
| ENSMUSG00000121503 | H2-K2 | -1.27982 | 0.00030 | down |
| ENSMUSG00000024502 | Jakmip2 | -1.3712 | 0.00029 | down |
| ENSMUSG00000036553 | Sh3tc1 | -1.00477 | 0.00029 | down |
| ENSMUSG00000040481 | Bptf | -1.24882 | 0.00029 | down |
| ENSMUSG00000044636 | Csrnp2 | -1.49601 | 0.00029 | down |
| ENSMUSG00000079020 | Slc45a4 | 1.012924 | 0.00029 | up |
| ENSMUSG00000080877 | Rpl22-ps1 | 2.729023 | 0.00029 | up |
| ENSMUSG00000107276 | Gm42858 | -1.70908 | 0.00029 | down |
| ENSMUSG00000114467 | Gm48430 | -2.50665 | 0.00029 | down |
| ENSMUSG00000023826 | Prkn | -1.89157 | 0.00028 | down |
| ENSMUSG00000024487 | Yipf5 | 1.170375 | 0.00028 | up |
| ENSMUSG00000029298 | Gbp9 | -1.17971 | 0.00028 | down |
| ENSMUSG00000079355 | Ackr4 | -1.87547 | 0.00028 | down |
| ENSMUSG00000105855 | Gm42681 | -1.95277 | 0.00028 | down |
| ENSMUSG00000106019 | Gm43672 | 1.69081 | 0.00028 | up |
| ENSMUSG00000111361 | Gm47445 | -2.24487 | 0.00028 | down |
| ENSMUSG00000115882 | Gm5481 | -4.01436 | 0.00028 | down |
| ENSMUSG00000118642 | AY036118 | -1.27168 | 0.00028 | down |
| ENSMUSG00000022504 | Ciita | -1.66688 | 0.00027 | down |
| ENSMUSG00000093942 | Olfr46 | -1.71251 | 0.00027 | down |
| ENSMUSG00000105832 | Gm43841 | -3.08323 | 0.00027 | down |
| ENSMUSG00000112580 | Gm47673 | -1.30669 | 0.00027 | down |
| ENSMUSG00000005054 | Cstb | 1.411011 | 0.00026 | up |
| ENSMUSG00000030761 | Myo7a | -1.01227 | 0.00026 | down |
| ENSMUSG00000031220 | Awat2 | -1.93294 | 0.00026 | down |
| ENSMUSG00000045671 | Spred2 | -1.14776 | 0.00026 | down |
| ENSMUSG00000048445 | Ccdc57 | -1.6205 | 0.00026 | down |
| ENSMUSG00000052738 | Suclg1 | 1.267 | 0.00026 | up |
| ENSMUSG00000028691 | Prdx1 | 1.0846 | 0.00025 | up |
| ENSMUSG00000049804 | Armcx4 | 1.418607 | 0.00025 | up |
| ENSMUSG00000053477 | Tcf4 | -1.00959 | 0.00025 | down |
| ENSMUSG00000092075 | Serpina4-ps1 | -2.0709 | 0.00025 | down |
| ENSMUSG00000095079 | Igha | -1.1493 | 0.00025 | down |
| ENSMUSG00000120149 | - | -2.23678 | 0.00025 | down |
| ENSMUSG00000002897 | Il17ra | 1.097499 | 0.00024 | up |
| ENSMUSG00000019179 | Mdh2 | 1.152166 | 0.00024 | up |
| ENSMUSG00000056148 | Rdh9 | 1.334391 | 0.00024 | up |
| ENSMUSG00000064356 | mt-Atp8 | -2.3256 | 0.00024 | down |
| ENSMUSG00000070605 | Zfp992 | -1.20477 | 0.00024 | down |
| ENSMUSG00000083594 | Gm13722 | -1.31947 | 0.00024 | down |
| ENSMUSG00000104721 | Gm42696 | -2.80828 | 0.00024 | down |
| ENSMUSG00000023905 | Tnfrsf12a | 1.052088 | 0.00023 | up |
| ENSMUSG00000026162 | Nhej1 | -1.49298 | 0.00023 | down |
| ENSMUSG00000035919 | Bbs9 | 1.534478 | 0.00023 | up |
| ENSMUSG00000051111 | Sv2c | -3.15616 | 0.00023 | down |
| ENSMUSG00000052534 | Pbx1 | -1.24457 | 0.00023 | down |
| ENSMUSG00000054598 | 9130230L23Rik | -2.70323 | 0.00023 | down |
| ENSMUSG00000100039 | Gm28959 | -1.17548 | 0.00023 | down |
| ENSMUSG00000100967 | Gm29666 | -1.8655 | 0.00023 | down |
| ENSMUSG00000108633 | Gm44694 | -4.36708 | 0.00023 | down |
| ENSMUSG00000120681 | - | -1.08835 | 0.00023 | down |
| ENSMUSG00000034459 | Ifit1 | -1.58807 | 0.00022 | down |
| ENSMUSG00000037731 | Themis2 | -1.3052 | 0.00022 | down |
| ENSMUSG00000038692 | Hoxb4 | -1.70627 | 0.00022 | down |
| ENSMUSG00000054728 | Phactr1 | -1.86593 | 0.00022 | down |
| ENSMUSG00000071047 | Ces1a | -5.12455 | 0.00022 | down |
| ENSMUSG00000087054 | Gm12405 | -4.23361 | 0.00022 | down |
| ENSMUSG00000121084 | - | 2.227718 | 0.00022 | up |
| ENSMUSG00000000605 | Clcn4 | -1.23447 | 0.00021 | down |
| ENSMUSG00000019737 | Syne4 | 1.5481 | 0.00021 | up |
| ENSMUSG00000024079 | Eif2ak2 | -1.23899 | 0.00021 | down |
| ENSMUSG00000024304 | Cdh2 | -1.31414 | 0.00021 | down |
| ENSMUSG00000026035 | Ppil3 | 1.153307 | 0.00021 | up |
| ENSMUSG00000027698 | Nceh1 | 1.661563 | 0.00021 | up |
| ENSMUSG00000040681 | Hmgn1 | 1.040598 | 0.00021 | up |
| ENSMUSG00000042115 | Klhdc8a | 1.89841 | 0.00021 | up |
| ENSMUSG00000046721 | Rpl14-ps1 | 1.069831 | 0.00021 | up |
| ENSMUSG00000049287 | Iba57 | 1.406415 | 0.00021 | up |
| ENSMUSG00000072623 | Zfp9 | 1.392174 | 0.00021 | up |
| ENSMUSG00000079165 | Sap25 | -2.06931 | 0.00021 | down |
| ENSMUSG00000112505 | Gm48610 | -2.75872 | 0.00021 | down |
| ENSMUSG00000114722 | Gm31392 | -1.96904 | 0.00021 | down |
| ENSMUSG00000023243 | Kcnk5 | -1.10919 | 0.00020 | down |
| ENSMUSG00000026149 | Tm4sf20 | 4.041032 | 0.00020 | up |
| ENSMUSG00000033767 | Tmem131l | -1.1602 | 0.00020 | down |
| ENSMUSG00000034435 | Tmem30b | 1.045408 | 0.00020 | up |
| ENSMUSG00000086370 | Ftx | -1.22152 | 0.00020 | down |
| ENSMUSG00000030559 | Rab38 | 3.634452 | 0.00019 | up |
| ENSMUSG00000031532 | Saraf | 1.004042 | 0.00019 | up |
| ENSMUSG00000036502 | Tmem255a | -1.68714 | 0.00019 | down |
| ENSMUSG00000041482 | Piezo2 | -1.51561 | 0.00019 | down |
| ENSMUSG00000064357 | mt-Atp6 | -1.85656 | 0.00019 | down |
| ENSMUSG00000066071 | Cyp4a12a | -1.14409 | 0.00019 | down |
| ENSMUSG00000068762 | Gstm6 | -1.03557 | 0.00019 | down |
| ENSMUSG00000029516 | Cit | -2.12504 | 0.00018 | down |
| ENSMUSG00000030089 | Slc41a3 | 2.620731 | 0.00018 | up |
| ENSMUSG00000038074 | Fkbp14 | -1.07294 | 0.00018 | down |
| ENSMUSG00000041147 | Brca2 | -1.04646 | 0.00018 | down |
| ENSMUSG00000059146 | Ntrk3 | -2.13712 | 0.00018 | down |
| ENSMUSG00000071356 | Reg3b | 4.789324 | 0.00018 | up |
| ENSMUSG00000085001 | Rapgef4os2 | -1.26689 | 0.00018 | down |
| ENSMUSG00000085665 | Gm12059 | 3.474104 | 0.00018 | up |
| ENSMUSG00000100147 | 1700047M11Rik | -1.96124 | 0.00018 | down |
| ENSMUSG00000104413 | Gm37065 | -3.99572 | 0.00018 | down |
| ENSMUSG00000013629 | Cad | 1.07883 | 0.00017 | up |
| ENSMUSG00000021701 | Plk2 | -1.14303 | 0.00017 | down |
| ENSMUSG00000022840 | Adcy5 | -1.47005 | 0.00017 | down |
| ENSMUSG00000039628 | Hs3st6 | 2.597336 | 0.00017 | up |
| ENSMUSG00000102752 | Gm7694 | 1.370736 | 0.00017 | up |
| ENSMUSG00000103432 | 6720464F23Rik | -2.62843 | 0.00017 | down |
| ENSMUSG00000108500 | Gm45033 | -1.71502 | 0.00017 | down |
| ENSMUSG00000008892 | Vdac3 | 1.006462 | 0.00016 | up |
| ENSMUSG00000015289 | Lage3 | 1.250147 | 0.00016 | up |
| ENSMUSG00000019899 | Lama2 | -1.53494 | 0.00016 | down |
| ENSMUSG00000026200 | Glb1l | -1.20334 | 0.00016 | down |
| ENSMUSG00000028179 | Cth | -1.29848 | 0.00016 | down |
| ENSMUSG00000033373 | Fntb | 1.943898 | 0.00016 | up |
| ENSMUSG00000033871 | Ppargc1b | -1.80024 | 0.00016 | down |
| ENSMUSG00000034758 | Tle6 | -1.78047 | 0.00016 | down |
| ENSMUSG00000035697 | Arhgap45 | -1.19189 | 0.00016 | down |
| ENSMUSG00000035863 | Palm | -1.24037 | 0.00016 | down |
| ENSMUSG00000036504 | Phpt1 | 1.315247 | 0.00016 | up |
| ENSMUSG00000059447 | Hadhb | 1.106367 | 0.00016 | up |
| ENSMUSG00000086527 | Gm15856 | -5.7886 | 0.00016 | down |
| ENSMUSG00000112774 | Gm36041 | -1.99776 | 0.00016 | down |
| ENSMUSG00000115007 | 5830448L01Rik | -1.2648 | 0.00016 | down |
| ENSMUSG00000117573 | Gm41668 | -5.71868 | 0.00016 | down |
| ENSMUSG00000022401 | Xpnpep3 | 1.19966 | 0.00015 | up |
| ENSMUSG00000026798 | Coq4 | 1.127546 | 0.00015 | up |
| ENSMUSG00000028199 | Cryz | 1.156306 | 0.00015 | up |
| ENSMUSG00000029993 | Nfu1 | 1.200968 | 0.00015 | up |
| ENSMUSG00000033209 | Ttc28 | -1.57716 | 0.00015 | down |
| ENSMUSG00000039233 | Tbce | -1.10387 | 0.00015 | down |
| ENSMUSG00000055435 | Maf | -1.38416 | 0.00015 | down |
| ENSMUSG00000063087 | Gm10125 | -1.36438 | 0.00015 | down |
| ENSMUSG00000104060 | Gm37954 | -1.74362 | 0.00015 | down |
| ENSMUSG00000107143 | Gm6598 | -1.59127 | 0.00015 | down |
| ENSMUSG00000022048 | Dpysl2 | -1.05505 | 0.00014 | down |
| ENSMUSG00000022911 | Arl13b | -1.17728 | 0.00014 | down |
| ENSMUSG00000032601 | Prkar2a | -1.16582 | 0.00014 | down |
| ENSMUSG00000036136 | Fam110c | 2.302598 | 0.00014 | up |
| ENSMUSG00000052013 | Btla | -2.32723 | 0.00014 | down |
| ENSMUSG00000052837 | Junb | 2.284812 | 0.00014 | up |
| ENSMUSG00000062110 | Scfd2 | 1.513247 | 0.00014 | up |
| ENSMUSG00000064220 | H2ac18 | 2.657784 | 0.00014 | up |
| ENSMUSG00000097482 | Gm17634 | -1.55753 | 0.00014 | down |
| ENSMUSG00000104149 | Gm37138 | -1.77815 | 0.00014 | down |
| ENSMUSG00000111045 | Gm47598 | -3.19165 | 0.00014 | down |
| ENSMUSG00000113388 | Gm48111 | -1.21585 | 0.00014 | down |
| ENSMUSG00000030724 | Cd19 | -2.56289 | 0.00013 | down |
| ENSMUSG00000033705 | Stard9 | -1.40319 | 0.00013 | down |
| ENSMUSG00000044309 | Apol7c | -4.48868 | 0.00013 | down |
| ENSMUSG00000046567 | 4930430F08Rik | -1.05888 | 0.00013 | down |
| ENSMUSG00000046794 | Ppp1r3b | -1.13697 | 0.00013 | down |
| ENSMUSG00000075383 | Olfr351 | -1.42737 | 0.00013 | down |
| ENSMUSG00000096950 | Gm9530 | -4.09819 | 0.00013 | down |
| ENSMUSG00000103932 | Gm36963 | -2.72799 | 0.00013 | down |
| ENSMUSG00000020134 | Peli1 | -1.3848 | 0.00012 | down |
| ENSMUSG00000026648 | Dclre1c | -1.04724 | 0.00012 | down |
| ENSMUSG00000033735 | Spr | 1.144316 | 0.00012 | up |
| ENSMUSG00000034858 | Fam214a | -1.05986 | 0.00012 | down |
| ENSMUSG00000037344 | Slc12a9 | -1.11781 | 0.00012 | down |
| ENSMUSG00000038233 | Gask1a | -2.15565 | 0.00012 | down |
| ENSMUSG00000052921 | Arhgef15 | -1.17495 | 0.00012 | down |
| ENSMUSG00000056054 | S100a8 | 2.287705 | 0.00012 | up |
| ENSMUSG00000057933 | Gsta2 | -1.22604 | 0.00012 | down |
| ENSMUSG00000059355 | Wdr83os | 1.186297 | 0.00012 | up |
| ENSMUSG00000063972 | Nr6a1 | -1.20483 | 0.00012 | down |
| ENSMUSG00000080058 | Gm11175 | -5.67375 | 0.00012 | down |
| ENSMUSG00000092626 | 9130230N09Rik | -1.5907 | 0.00012 | down |
| ENSMUSG00000102428 | Pcdhga12 | -1.77181 | 0.00012 | down |
| ENSMUSG00000106924 | Gm42857 | -1.86972 | 0.00012 | down |
| ENSMUSG00000121426 | - | -1.48727 | 0.00012 | down |
| ENSMUSG00000013833 | Med16 | 1.158546 | 0.00011 | up |
| ENSMUSG00000021716 | Srek1ip1 | -1.48699 | 0.00011 | down |
| ENSMUSG00000024168 | Tmem204 | -1.22062 | 0.00011 | down |
| ENSMUSG00000025355 | Mmp19 | 1.081169 | 0.00011 | up |
| ENSMUSG00000028359 | Orm3 | 2.650992 | 0.00011 | up |
| ENSMUSG00000035378 | Shq1 | 1.199599 | 0.00011 | up |
| ENSMUSG00000042874 | D930007J09Rik | 4.446945 | 0.00011 | up |
| ENSMUSG00000061650 | Med9 | 1.101405 | 0.00011 | up |
| ENSMUSG00000084790 | Gm15879 | -2.19991 | 0.00011 | down |
| ENSMUSG00000106568 | Gm42814 | -3.84787 | 0.00011 | down |
| ENSMUSG00000107785 | Gm45083 | 1.812102 | 0.00011 | up |
| ENSMUSG00000112038 | Gm47056 | -1.76912 | 0.00011 | down |
| ENSMUSG00000119972 | - | -1.45167 | 0.00011 | down |
| ENSMUSG00002076650 | Snord3b1 | -5.30781 | 0.00011 | down |
| ENSMUSG00000000560 | Gabra2 | -2.46571 | 0.00010 | down |
| ENSMUSG00000017740 | Slc12a5 | -2.10409 | 0.00010 | down |
| ENSMUSG00000018102 | H2bc4 | 1.290347 | 0.00010 | up |
| ENSMUSG00000022237 | Ankrd33b | -1.08832 | 0.00010 | down |
| ENSMUSG00000026209 | Dnpep | 1.099118 | 0.00010 | up |
| ENSMUSG00000032377 | Plscr4 | -1.62932 | 0.00010 | down |
| ENSMUSG00000059974 | Ntm | -1.67689 | 0.00010 | down |
| ENSMUSG00000068732 | Tmem167b | 1.251793 | 0.00010 | up |
| ENSMUSG00000094520 | Olfr635 | -1.9295 | 0.00010 | down |
| ENSMUSG00000120918 | - | -2.1602 | 0.00010 | down |
| ENSMUSG00000019122 | Ccl9 | 1.06293 | 0.00009 | up |
| ENSMUSG00000020467 | Efemp1 | -1.34399 | 0.00009 | down |
| ENSMUSG00000028982 | Slc25a33 | 1.052714 | 0.00009 | up |
| ENSMUSG00000029270 | Dipk1a | -1.02538 | 0.00009 | down |
| ENSMUSG00000030067 | Foxp1 | -1.52749 | 0.00009 | down |
| ENSMUSG00000031441 | Atp11a | 1.248977 | 0.00009 | up |
| ENSMUSG00000031901 | Dus2 | 1.590662 | 0.00009 | up |
| ENSMUSG00000036840 | Siah1a | -1.09662 | 0.00009 | down |
| ENSMUSG00000050541 | Adra1b | -1.10707 | 0.00009 | down |
| ENSMUSG00000090386 | Mir99ahg | -1.56742 | 0.00009 | down |
| ENSMUSG00000091509 | Gm17066 | -1.59312 | 0.00009 | down |
| ENSMUSG00000113948 | Rpl17-ps3 | -3.73131 | 0.00009 | down |
| ENSMUSG00000120006 | - | -1.79476 | 0.00009 | down |
| ENSMUSG00000003559 | As3mt | 1.000338 | 0.00008 | up |
| ENSMUSG00000007035 | Msh5 | -2.39338 | 0.00008 | down |
| ENSMUSG00000019301 | Hsd17b1 | -3.56659 | 0.00008 | down |
| ENSMUSG00000021660 | Btf3 | 1.005807 | 0.00008 | up |
| ENSMUSG00000023965 | Fbxl17 | 1.291189 | 0.00008 | up |
| ENSMUSG00000026248 | Mrpl44 | 1.221868 | 0.00008 | up |
| ENSMUSG00000029198 | Grpel1 | 1.25037 | 0.00008 | up |
| ENSMUSG00000032112 | Trappc4 | 1.559836 | 0.00008 | up |
| ENSMUSG00000049721 | Gal3st1 | 1.903716 | 0.00008 | up |
| ENSMUSG00000062070 | Pgk1 | 1.095773 | 0.00008 | up |
| ENSMUSG00000062563 | Cys1 | -1.66827 | 0.00008 | down |
| ENSMUSG00000069892 | 9930111J21Rik2 | -1.35091 | 0.00008 | down |
| ENSMUSG00000071637 | Cebpd | 1.889496 | 0.00008 | up |
| ENSMUSG00000074513 | Arfip1 | 1.013212 | 0.00008 | up |
| ENSMUSG00000089942 | Pira2 | 3.40169 | 0.00008 | up |
| ENSMUSG00000097042 | Gm17491 | -1.38066 | 0.00008 | down |
| ENSMUSG00000105287 | Gm43577 | -1.9844 | 0.00008 | down |
| ENSMUSG00000107690 | Gm44044 | -2.66396 | 0.00008 | down |
| ENSMUSG00000114138 | Gm36423 | -3.26004 | 0.00008 | down |
| ENSMUSG00000020044 | Timp3 | -1.45282 | 0.00007 | down |
| ENSMUSG00000021576 | Pdcd6 | 1.042613 | 0.00007 | up |
| ENSMUSG00000022218 | Tgm1 | 1.194618 | 0.00007 | up |
| ENSMUSG00000025521 | Tmem192 | 1.305608 | 0.00007 | up |
| ENSMUSG00000026970 | Rbms1 | -1.4241 | 0.00007 | down |
| ENSMUSG00000028167 | Bdh2 | -1.42372 | 0.00007 | down |
| ENSMUSG00000028189 | Ctbs | 1.441669 | 0.00007 | up |
| ENSMUSG00000031925 | Maml2 | -1.84773 | 0.00007 | down |
| ENSMUSG00000032561 | Acpp | 2.662093 | 0.00007 | up |
| ENSMUSG00000032596 | Uba7 | -1.18534 | 0.00007 | down |
| ENSMUSG00000037440 | Vnn1 | 1.048544 | 0.00007 | up |
| ENSMUSG00000038648 | Creb3l2 | 1.097102 | 0.00007 | up |
| ENSMUSG00000051065 | Mb21d2 | 1.032606 | 0.00007 | up |
| ENSMUSG00000085774 | Gm13055 | -5.24363 | 0.00007 | down |
| ENSMUSG00000097163 | BC051077 | -3.32497 | 0.00007 | down |
| ENSMUSG00000017057 | Il13ra1 | 1.724719 | 0.00006 | up |
| ENSMUSG00000017286 | Glod4 | 1.136144 | 0.00006 | up |
| ENSMUSG00000017446 | C1qtnf1 | 1.975998 | 0.00006 | up |
| ENSMUSG00000021149 | Gtpbp4 | -1.51412 | 0.00006 | down |
| ENSMUSG00000022856 | Tmem41a | 1.79304 | 0.00006 | up |
| ENSMUSG00000024411 | Aqp4 | 1.68943 | 0.00006 | up |
| ENSMUSG00000025421 | Hdhd2 | 1.129653 | 0.00006 | up |
| ENSMUSG00000027195 | Hsd17b12 | 1.069962 | 0.00006 | up |
| ENSMUSG00000028567 | Txndc12 | 1.174914 | 0.00006 | up |
| ENSMUSG00000030786 | Itgam | 1.956079 | 0.00006 | up |
| ENSMUSG00000034912 | Mdga2 | -3.08792 | 0.00006 | down |
| ENSMUSG00000042599 | Kdm7a | -1.4859 | 0.00006 | down |
| ENSMUSG00000054793 | Cadm4 | 1.073118 | 0.00006 | up |
| ENSMUSG00000085404 | Gm12909 | -1.53543 | 0.00006 | down |
| ENSMUSG00000092008 | Cyp2c69 | -2.79025 | 0.00006 | down |
| ENSMUSG00000094822 | Olfr243 | -3.45821 | 0.00006 | down |
| ENSMUSG00000102562 | Gm37694 | -3.31549 | 0.00006 | down |
| ENSMUSG00000112622 | Gm47164 | -1.55256 | 0.00006 | down |
| ENSMUSG00000113517 | Gm47905 | -2.00512 | 0.00006 | down |
| ENSMUSG00000117238 | - | -2.19034 | 0.00006 | down |
| ENSMUSG00000121162 | - | -4.35091 | 0.00006 | down |
| ENSMUSG00000018752 | Tnfsfm13 | 2.385533 | 0.00005 | up |
| ENSMUSG00000021010 | Npas3 | -5.44506 | 0.00005 | down |
| ENSMUSG00000021131 | Erh | 1.107253 | 0.00005 | up |
| ENSMUSG00000021248 | Tmed10 | 1.130832 | 0.00005 | up |
| ENSMUSG00000021719 | Rgs7bp | -1.48054 | 0.00005 | down |
| ENSMUSG00000022436 | Sh3bp1 | -1.94616 | 0.00005 | down |
| ENSMUSG00000025175 | Fn3k | -1.04317 | 0.00005 | down |
| ENSMUSG00000032816 | Igdcc4 | 3.258342 | 0.00005 | up |
| ENSMUSG00000036083 | Slc17a3 | -1.03288 | 0.00005 | down |
| ENSMUSG00000037994 | Slc9b2 | -2.11087 | 0.00005 | down |
| ENSMUSG00000044906 | 4930503L19Rik | -1.16024 | 0.00005 | down |
| ENSMUSG00000054404 | Slfn5 | -1.13709 | 0.00005 | down |
| ENSMUSG00000057561 | Eif1a | 1.055953 | 0.00005 | up |
| ENSMUSG00000057649 | Brd9 | -1.11783 | 0.00005 | down |
| ENSMUSG00000057982 | Zfp809 | -1.38478 | 0.00005 | down |
| ENSMUSG00000064337 | mt-Rnr1 | -1.95694 | 0.00005 | down |
| ENSMUSG00000081769 | Gm12216 | -2.29064 | 0.00005 | down |
| ENSMUSG00000084934 | Gm16035 | -1.22973 | 0.00005 | down |
| ENSMUSG00000086844 | B230206H07Rik | -2.00362 | 0.00005 | down |
| ENSMUSG00000087120 | Gm12279 | -1.69945 | 0.00005 | down |
| ENSMUSG00000100600 | A230077H06Rik | -3.87298 | 0.00005 | down |
| ENSMUSG00000101249 | Gm29216 | -2.48773 | 0.00005 | down |
| ENSMUSG00000102145 | Gm38056 | -5.60602 | 0.00005 | down |
| ENSMUSG00000104159 | Gm38099 | -1.82619 | 0.00005 | down |
| ENSMUSG00000121179 | - | -1.293 | 0.00005 | down |
| ENSMUSG00000004187 | Kifc2 | -1.61435 | 0.00004 | down |
| ENSMUSG00000013033 | Adgrl1 | -1.32524 | 0.00004 | down |
| ENSMUSG00000015013 | Trappc2l | 1.22918 | 0.00004 | up |
| ENSMUSG00000020778 | Ten1 | 1.880654 | 0.00004 | up |
| ENSMUSG00000021508 | Cxcl14 | 2.099266 | 0.00004 | up |
| ENSMUSG00000025192 | Entpd7 | 2.095843 | 0.00004 | up |
| ENSMUSG00000025208 | Mrpl43 | 1.02579 | 0.00004 | up |
| ENSMUSG00000026082 | Rev1 | -1.02949 | 0.00004 | down |
| ENSMUSG00000026785 | Pkn3 | -1.95902 | 0.00004 | down |
| ENSMUSG00000029553 | Tfec | -1.40574 | 0.00004 | down |
| ENSMUSG00000034371 | Tkfc | -1.2131 | 0.00004 | down |
| ENSMUSG00000034854 | Mfsd12 | 1.374285 | 0.00004 | up |
| ENSMUSG00000036545 | Adamts2 | -1.14527 | 0.00004 | down |
| ENSMUSG00000038121 | Fam210a | -1.08989 | 0.00004 | down |
| ENSMUSG00000038569 | Rad9b | -1.24103 | 0.00004 | down |
| ENSMUSG00000040181 | Fmo1 | -1.12902 | 0.00004 | down |
| ENSMUSG00000042496 | Prdm10 | -1.04496 | 0.00004 | down |
| ENSMUSG00000044595 | Dnd1 | -1.92526 | 0.00004 | down |
| ENSMUSG00000045875 | Adra1a | 1.457532 | 0.00004 | up |
| ENSMUSG00000051439 | Cd14 | 2.527379 | 0.00004 | up |
| ENSMUSG00000063787 | Chchd1 | 1.15484 | 0.00004 | up |
| ENSMUSG00000064354 | mt-Co2 | -2.07637 | 0.00004 | down |
| ENSMUSG00000064899 | Snord118 | -4.05379 | 0.00004 | down |
| ENSMUSG00000074882 | Cyp2c68 | -1.07579 | 0.00004 | down |
| ENSMUSG00000076617 | Ighm | -1.37769 | 0.00004 | down |
| ENSMUSG00000097772 | 5430416N02Rik | -1.29321 | 0.00004 | down |
| ENSMUSG00000101892 | 9130401M01Rik | 1.011982 | 0.00004 | up |
| ENSMUSG00000109179 | Gm35339 | -1.62908 | 0.00004 | down |
| ENSMUSG00000115219 | Eef1akmt4 | 1.159057 | 0.00004 | up |
| ENSMUSG00000115869 | Gm31814 | -2.63979 | 0.00004 | down |
| ENSMUSG00000004105 | Angptl2 | -1.16813 | 0.00003 | down |
| ENSMUSG00000008200 | Fnbp4 | -1.18819 | 0.00003 | down |
| ENSMUSG00000012017 | Scarf2 | -1.29091 | 0.00003 | down |
| ENSMUSG00000015711 | Prune1 | 1.350005 | 0.00003 | up |
| ENSMUSG00000020027 | Socs2 | -2.51582 | 0.00003 | down |
| ENSMUSG00000021951 | Eef1akmt1 | 2.445553 | 0.00003 | up |
| ENSMUSG00000024943 | Smc5 | -1.05707 | 0.00003 | down |
| ENSMUSG00000025898 | Cwf19l2 | -1.08067 | 0.00003 | down |
| ENSMUSG00000026417 | Pigr | -1.3745 | 0.00003 | down |
| ENSMUSG00000028671 | Gale | 1.261598 | 0.00003 | up |
| ENSMUSG00000029557 | Mrm2 | 1.231618 | 0.00003 | up |
| ENSMUSG00000030245 | Golt1b | 1.376168 | 0.00003 | up |
| ENSMUSG00000031158 | Timm17b | 1.077529 | 0.00003 | up |
| ENSMUSG00000031712 | Il15 | -2.19126 | 0.00003 | down |
| ENSMUSG00000031775 | Pllp | 1.198728 | 0.00003 | up |
| ENSMUSG00000036155 | Mgat5 | 1.155166 | 0.00003 | up |
| ENSMUSG00000037085 | Trmt12 | 1.654735 | 0.00003 | up |
| ENSMUSG00000039195 | Bbln | 1.211451 | 0.00003 | up |
| ENSMUSG00000045672 | Col27a1 | -2.41547 | 0.00003 | down |
| ENSMUSG00000046352 | Gjb2 | 1.008424 | 0.00003 | up |
| ENSMUSG00000047735 | Samd9l | -1.15199 | 0.00003 | down |
| ENSMUSG00000049580 | Tsku | -2.18493 | 0.00003 | down |
| ENSMUSG00000057098 | Ebf1 | -1.93669 | 0.00003 | down |
| ENSMUSG00000063445 | Nmral1 | 1.195559 | 0.00003 | up |
| ENSMUSG00000063882 | Uqcrh | 1.03589 | 0.00003 | up |
| ENSMUSG00000073684 | Faap20 | 1.199552 | 0.00003 | up |
| ENSMUSG00000080921 | Rpl38-ps2 | -9.44365 | 0.00003 | down |
| ENSMUSG00000097589 | Dleu2 | -1.1597 | 0.00003 | down |
| ENSMUSG00000108218 | Olfr1372 | -4.09493 | 0.00003 | down |
| ENSMUSG00000111928 | Gm48082 | -1.39194 | 0.00003 | down |
| ENSMUSG00000115020 | Vmn1r218 | -1.64513 | 0.00003 | down |
| ENSMUSG00000115431 | Gm3219 | -1.36864 | 0.00003 | down |
| ENSMUSG00000117292 | E330032C10Rik | -2.1152 | 0.00003 | down |
| ENSMUSG00000121473 | Adh6-ps1 | -1.61774 | 0.00003 | down |
| ENSMUSG00000003038 | Hmgn2 | 1.05422 | 0.00002 | up |
| ENSMUSG00000004098 | Col5a3 | -1.57512 | 0.00002 | down |
| ENSMUSG00000004500 | Zfp324 | 1.648149 | 0.00002 | up |
| ENSMUSG00000008140 | Emc10 | 1.013103 | 0.00002 | up |
| ENSMUSG00000018567 | Gabarap | 1.181094 | 0.00002 | up |
| ENSMUSG00000020364 | Zfp354a | -1.31532 | 0.00002 | down |
| ENSMUSG00000020650 | Bcap29 | 1.346474 | 0.00002 | up |
| ENSMUSG00000024299 | Adamts10 | -1.18831 | 0.00002 | down |
| ENSMUSG00000024902 | Mrpl11 | 1.097826 | 0.00002 | up |
| ENSMUSG00000025934 | Gsta3 | -1.22998 | 0.00002 | down |
| ENSMUSG00000025995 | Wdr75 | -1.01925 | 0.00002 | down |
| ENSMUSG00000026473 | Glul | 1.044861 | 0.00002 | up |
| ENSMUSG00000026766 | Mmadhc | 1.152617 | 0.00002 | up |
| ENSMUSG00000027318 | Adam33 | -2.22873 | 0.00002 | down |
| ENSMUSG00000027706 | Sec62 | -1.56775 | 0.00002 | down |
| ENSMUSG00000027801 | Tm4sf4 | 1.130353 | 0.00002 | up |
| ENSMUSG00000028150 | Rorc | -1.36395 | 0.00002 | down |
| ENSMUSG00000028555 | Ttc39a | 3.109169 | 0.00002 | up |
| ENSMUSG00000029066 | Mrpl20 | 1.088486 | 0.00002 | up |
| ENSMUSG00000029314 | Gpat3 | 1.6979 | 0.00002 | up |
| ENSMUSG00000029426 | Scarb2 | 1.127844 | 0.00002 | up |
| ENSMUSG00000031133 | Arhgef6 | -1.37574 | 0.00002 | down |
| ENSMUSG00000031150 | Ccdc120 | 1.613222 | 0.00002 | up |
| ENSMUSG00000035960 | Apex1 | 1.132822 | 0.00002 | up |
| ENSMUSG00000036430 | Tbcc | 1.61358 | 0.00002 | up |
| ENSMUSG00000037797 | Adh4 | 1.035468 | 0.00002 | up |
| ENSMUSG00000039182 | AW209491 | 1.189664 | 0.00002 | up |
| ENSMUSG00000040724 | Kcna2 | -1.38926 | 0.00002 | down |
| ENSMUSG00000041237 | Pklr | -1.63968 | 0.00002 | down |
| ENSMUSG00000042138 | Msantd2 | -1.10326 | 0.00002 | down |
| ENSMUSG00000046688 | Tifa | 1.235846 | 0.00002 | up |
| ENSMUSG00000048126 | Col6a3 | -1.398 | 0.00002 | down |
| ENSMUSG00000048234 | Rnf149 | 1.687877 | 0.00002 | up |
| ENSMUSG00000057315 | Arhgap24 | -1.3692 | 0.00002 | down |
| ENSMUSG00000057425 | Ugt2b37 | -1.83948 | 0.00002 | down |
| ENSMUSG00000060149 | BC002059 | -1.28523 | 0.00002 | down |
| ENSMUSG00000061273 | Mmgt1 | 1.26575 | 0.00002 | up |
| ENSMUSG00000063856 | Gpx1 | 1.403197 | 0.00002 | up |
| ENSMUSG00000064294 | Aox3 | -1.14608 | 0.00002 | down |
| ENSMUSG00000064326 | Siva1 | 2.044662 | 0.00002 | up |
| ENSMUSG00000066196 | Spag8 | -2.93501 | 0.00002 | down |
| ENSMUSG00000073838 | Tufm | 1.197175 | 0.00002 | up |
| ENSMUSG00000074469 | Gm15348 | 4.853293 | 0.00002 | up |
| ENSMUSG00000089774 | Slc5a3 | -1.23974 | 0.00002 | down |
| ENSMUSG00000096002 | Vmn2r53 | -1.62288 | 0.00002 | down |
| ENSMUSG00000096054 | Syne1 | -1.08883 | 0.00002 | down |
| ENSMUSG00000102858 | Gm37086 | -1.42561 | 0.00002 | down |
| ENSMUSG00000107771 | Gm8956 | -3.28517 | 0.00002 | down |
| ENSMUSG00000109157 | Gm44829 | -2.41131 | 0.00002 | down |
| ENSMUSG00000112226 | Gm48786 | -1.81827 | 0.00002 | down |
| ENSMUSG00000112947 | Gm47493 | -1.44587 | 0.00002 | down |
| ENSMUSG00000113069 | Gm48541 | -1.61923 | 0.00002 | down |
| ENSMUSG00000113290 | A530058O07Rik | -2.31964 | 0.00002 | down |
| ENSMUSG00000115124 | Gm49201 | -1.18836 | 0.00002 | down |
| ENSMUSG00000002032 | Tmem25 | -1.38197 | 0.00001 | down |
| ENSMUSG00000002250 | Ppard | -1.95818 | 0.00001 | down |
| ENSMUSG00000006057 | Atp5g1 | 1.027967 | 0.00001 | up |
| ENSMUSG00000009418 | Nav1 | -1.10322 | 0.00001 | down |
| ENSMUSG00000009549 | Srp14 | 1.165641 | 0.00001 | up |
| ENSMUSG00000009646 | Pla2g12b | 1.068691 | 0.00001 | up |
| ENSMUSG00000014778 | Fhod1 | -1.14878 | 0.00001 | down |
| ENSMUSG00000017713 | Tha1 | 1.224332 | 0.00001 | up |
| ENSMUSG00000019487 | Trip10 | -1.20924 | 0.00001 | down |
| ENSMUSG00000019756 | Prl8a1 | -2.34981 | 0.00001 | down |
| ENSMUSG00000019796 | Lrp11 | 4.628263 | 0.00001 | up |
| ENSMUSG00000019806 | Aig1 | 1.593157 | 0.00001 | up |
| ENSMUSG00000020072 | Pbld2 | -1.00505 | 0.00001 | down |
| ENSMUSG00000020448 | Rnf185 | 1.058107 | 0.00001 | up |
| ENSMUSG00000020469 | Myl7 | -6.17205 | 0.00001 | down |
| ENSMUSG00000022651 | Retnlg | 4.277944 | 0.00001 | up |
| ENSMUSG00000024421 | Lama3 | -1.71454 | 0.00001 | down |
| ENSMUSG00000024451 | Arap3 | -1.14978 | 0.00001 | down |
| ENSMUSG00000024694 | Keg1 | -2.54965 | 0.00001 | down |
| ENSMUSG00000025790 | Slco3a1 | -1.74173 | 0.00001 | down |
| ENSMUSG00000026185 | Igfbp5 | -1.44129 | 0.00001 | down |
| ENSMUSG00000026343 | Gpr39 | 1.719505 | 0.00001 | up |
| ENSMUSG00000026463 | Atp2b4 | -1.39963 | 0.00001 | down |
| ENSMUSG00000026941 | Mamdc4 | -1.96782 | 0.00001 | down |
| ENSMUSG00000027165 | Iftap | 1.319511 | 0.00001 | up |
| ENSMUSG00000028073 | Pear1 | -1.37267 | 0.00001 | down |
| ENSMUSG00000028161 | Ppp3ca | -1.39852 | 0.00001 | down |
| ENSMUSG00000028798 | Eif3i | 1.037251 | 0.00001 | up |
| ENSMUSG00000029003 | Mad2l2 | 1.18773 | 0.00001 | up |
| ENSMUSG00000029648 | Flt1 | -1.28934 | 0.00001 | down |
| ENSMUSG00000029675 | Eln | -1.29485 | 0.00001 | down |
| ENSMUSG00000031389 | Arhgap4 | -1.38379 | 0.00001 | down |
| ENSMUSG00000031858 | Mau2 | -1.14248 | 0.00001 | down |
| ENSMUSG00000032078 | Zpr1 | -1.29282 | 0.00001 | down |
| ENSMUSG00000032253 | Phip | -1.01374 | 0.00001 | down |
| ENSMUSG00000032265 | Tent5a | -1.63141 | 0.00001 | down |
| ENSMUSG00000032743 | Katnip | -1.14198 | 0.00001 | down |
| ENSMUSG00000034258 | Flvcr2 | 1.931083 | 0.00001 | up |
| ENSMUSG00000035107 | Dcbld2 | -1.81277 | 0.00001 | down |
| ENSMUSG00000035836 | Ugt2b1 | -1.10758 | 0.00001 | down |
| ENSMUSG00000035941 | Ibtk | 1.218197 | 0.00001 | up |
| ENSMUSG00000035992 | Fnip1 | -1.21877 | 0.00001 | down |
| ENSMUSG00000037190 | Cyb561d2 | 1.578172 | 0.00001 | up |
| ENSMUSG00000038722 | Bud31 | 1.27204 | 0.00001 | up |
| ENSMUSG00000039033 | Tasp1 | 1.816147 | 0.00001 | up |
| ENSMUSG00000041220 | Elovl6 | -1.64938 | 0.00001 | down |
| ENSMUSG00000041445 | Mmrn2 | -1.01328 | 0.00001 | down |
| ENSMUSG00000041798 | Gck | -1.7652 | 0.00001 | down |
| ENSMUSG00000042156 | Dzip1 | -2.37782 | 0.00001 | down |
| ENSMUSG00000042895 | Abra | -4.88252 | 0.00001 | down |
| ENSMUSG00000045211 | Nudt18 | 1.568032 | 0.00001 | up |
| ENSMUSG00000045257 | Morn2 | 1.243561 | 0.00001 | up |
| ENSMUSG00000046826 | Fam187b | 2.24932 | 0.00001 | up |
| ENSMUSG00000047180 | Neurl3 | -1.1969 | 0.00001 | down |
| ENSMUSG00000048865 | Arhgap30 | -1.036 | 0.00001 | down |
| ENSMUSG00000051225 | Fam83a | 4.167952 | 0.00001 | up |
| ENSMUSG00000051234 | Rnf7 | 1.145575 | 0.00001 | up |
| ENSMUSG00000052407 | Ccdc171 | -1.71992 | 0.00001 | down |
| ENSMUSG00000055216 | 9430025C20Rik | -2.40504 | 0.00001 | down |
| ENSMUSG00000055401 | Fbxo6 | 1.314795 | 0.00001 | up |
| ENSMUSG00000056763 | Cspp1 | -1.02183 | 0.00001 | down |
| ENSMUSG00000057913 | Gm10032 | 4.611855 | 0.00001 | up |
| ENSMUSG00000059142 | Zfp945 | -1.05182 | 0.00001 | down |
| ENSMUSG00000060441 | Trim5 | -1.76761 | 0.00001 | down |
| ENSMUSG00000061111 | Mcrip1 | 2.208934 | 0.00001 | up |
| ENSMUSG00000061825 | Ces2c | -1.15231 | 0.00001 | down |
| ENSMUSG00000061906 | Ugt2b38 | -1.47969 | 0.00001 | down |
| ENSMUSG00000063590 | Slc22a28 | -2.70785 | 0.00001 | down |
| ENSMUSG00000064373 | Selenop | 1.697769 | 0.00001 | up |
| ENSMUSG00000069972 | Rps13-ps2 | 1.225023 | 0.00001 | up |
| ENSMUSG00000072501 | Phf20l1 | -1.12139 | 0.00001 | down |
| ENSMUSG00000072893 | 4933439C10Rik | -1.47639 | 0.00001 | down |
| ENSMUSG00000073643 | Wdfy1 | -1.12626 | 0.00001 | down |
| ENSMUSG00000074340 | Ovgp1 | -2.34665 | 0.00001 | down |
| ENSMUSG00000074461 | Gm10699 | 5.147592 | 0.00001 | up |
| ENSMUSG00000078688 | Mup2 | -1.61742 | 0.00001 | down |
| ENSMUSG00000078713 | Tomm5 | 1.019923 | 0.00001 | up |
| ENSMUSG00000081485 | Gm12338 | -2.28146 | 0.00001 | down |
| ENSMUSG00000085334 | Gm12940 | -1.76395 | 0.00001 | down |
| ENSMUSG00000086141 | 9030622O22Rik | -1.16775 | 0.00001 | down |
| ENSMUSG00000087404 | Gm11752 | 4.754327 | 0.00001 | up |
| ENSMUSG00000090272 | Mndal | -1.43868 | 0.00001 | down |
| ENSMUSG00000097415 | AU020206 | -1.41439 | 0.00001 | down |
| ENSMUSG00000098014 | Gm26967 | -1.43587 | 0.00001 | down |
| ENSMUSG00000098332 | Pigbos1 | 1.171409 | 0.00001 | up |
| ENSMUSG00000101939 | Gm28438 | -1.56465 | 0.00001 | down |
| ENSMUSG00000101970 | Chaserr | -1.04643 | 0.00001 | down |
| ENSMUSG00000103672 | Gm37621 | -1.98288 | 0.00001 | down |
| ENSMUSG00000104011 | Gm32391 | -1.72318 | 0.00001 | down |
| ENSMUSG00000106352 | 5033403H07Rik | -1.07015 | 0.00001 | down |
| ENSMUSG00000108857 | Gm44578 | -3.16882 | 0.00001 | down |
| ENSMUSG00000110344 | Smim36 | 2.111449 | 0.00001 | up |
| ENSMUSG00000111269 | Gm47933 | -2.16754 | 0.00001 | down |
| ENSMUSG00000112013 | Gm47967 | -2.41124 | 0.00001 | down |
| ENSMUSG00000112880 | Gm20337 | -2.2929 | 0.00001 | down |
| ENSMUSG00000113831 | Gm49602 | -1.76306 | 0.00001 | down |
| ENSMUSG00000114114 | Gm48499 | -3.6486 | 0.00001 | down |
| ENSMUSG00000120725 | - | -1.75855 | 0.00001 | down |
| ENSMUSG00000120763 | - | 4.613157 | 0.00001 | up |
| ENSMUSG00000120776 | Gm34235 | -1.95546 | 0.00001 | down |
| ENSMUSG00000121083 | - | -1.16002 | 0.00001 | down |
| ENSMUSG00000000340 | Dbt | -1.60774 | <0.00001 | down |
| ENSMUSG00000000693 | Loxl3 | -1.82636 | <0.00001 | down |
| ENSMUSG00000000739 | Sult5a1 | -1.69831 | <0.00001 | down |
| ENSMUSG00000000957 | Mmp14 | 1.653575 | <0.00001 | up |
| ENSMUSG00000001095 | Slc13a2 | -4.23502 | <0.00001 | down |
| ENSMUSG00000001131 | Timp1 | 4.143702 | <0.00001 | up |
| ENSMUSG00000001155 | Ftcd | -1.01448 | <0.00001 | down |
| ENSMUSG00000001380 | Hars | 1.128088 | <0.00001 | up |
| ENSMUSG00000001416 | Cct3 | 1.175333 | <0.00001 | up |
| ENSMUSG00000001588 | Acap1 | -1.79253 | <0.00001 | down |
| ENSMUSG00000001663 | Gstt1 | -1.01192 | <0.00001 | down |
| ENSMUSG00000002014 | Ssr4 | 1.253375 | <0.00001 | up |
| ENSMUSG00000002102 | Psmc3 | 1.043082 | <0.00001 | up |
| ENSMUSG00000002778 | Kdelr1 | 1.309939 | <0.00001 | up |
| ENSMUSG00000002944 | Cd36 | 1.327737 | <0.00001 | up |
| ENSMUSG00000003279 | Dlgap1 | -2.69189 | <0.00001 | down |
| ENSMUSG00000003355 | Fkbp11 | 1.609073 | <0.00001 | up |
| ENSMUSG00000003402 | Prkcsh | 1.138389 | <0.00001 | up |
| ENSMUSG00000003477 | Inmt | -1.49444 | <0.00001 | down |
| ENSMUSG00000003526 | Prodh | -1.32527 | <0.00001 | down |
| ENSMUSG00000003604 | Aven | 1.475632 | <0.00001 | up |
| ENSMUSG00000003762 | Coq8b | 2.174992 | <0.00001 | up |
| ENSMUSG00000003814 | Calr | 1.216419 | <0.00001 | up |
| ENSMUSG00000004038 | Gstm3 | -2.43877 | <0.00001 | down |
| ENSMUSG00000004040 | Stat3 | 1.2042 | <0.00001 | up |
| ENSMUSG00000004069 | Dnaja3 | 1.12251 | <0.00001 | up |
| ENSMUSG00000004394 | Tmed4 | 1.311796 | <0.00001 | up |
| ENSMUSG00000004460 | Dnajb11 | 2.087617 | <0.00001 | up |
| ENSMUSG00000005373 | Mlxipl | -1.79252 | <0.00001 | down |
| ENSMUSG00000005547 | Cyp2a5 | -2.1755 | <0.00001 | down |
| ENSMUSG00000005779 | Psmb4 | 1.490467 | <0.00001 | up |
| ENSMUSG00000006014 | Prg4 | 2.818687 | <0.00001 | up |
| ENSMUSG00000006095 | Tbcb | 1.458974 | <0.00001 | up |
| ENSMUSG00000006315 | Tmem147 | 1.217682 | <0.00001 | up |
| ENSMUSG00000006333 | Rps9 | 1.029206 | <0.00001 | up |
| ENSMUSG00000006442 | Srm | 1.495299 | <0.00001 | up |
| ENSMUSG00000006522 | Itih3 | 1.58005 | <0.00001 | up |
| ENSMUSG00000006526 | Stimate | 1.153395 | <0.00001 | up |
| ENSMUSG00000006763 | Saal1 | 1.24714 | <0.00001 | up |
| ENSMUSG00000007097 | Atp1a2 | -1.53243 | <0.00001 | down |
| ENSMUSG00000007594 | Hapln4 | 1.735333 | <0.00001 | up |
| ENSMUSG00000007739 | Cct4 | 1.026242 | <0.00001 | up |
| ENSMUSG00000007892 | Rplp1 | 1.402524 | <0.00001 | up |
| ENSMUSG00000008305 | Tle1 | -1.32842 | <0.00001 | down |
| ENSMUSG00000008683 | Rps15a | 1.09907 | <0.00001 | up |
| ENSMUSG00000009013 | Dynll1 | 1.00435 | <0.00001 | up |
| ENSMUSG00000009092 | Derl3 | 3.615283 | <0.00001 | up |
| ENSMUSG00000009292 | Trpm2 | -1.7507 | <0.00001 | down |
| ENSMUSG00000009293 | Ube2g2 | 1.261922 | <0.00001 | up |
| ENSMUSG00000009741 | Ubp1 | -1.03796 | <0.00001 | down |
| ENSMUSG00000010122 | Slc47a1 | -1.28054 | <0.00001 | down |
| ENSMUSG00000011114 | Tbrg1 | 1.531259 | <0.00001 | up |
| ENSMUSG00000011305 | Plin5 | 1.455023 | <0.00001 | up |
| ENSMUSG00000012117 | Dhdds | 1.730707 | <0.00001 | up |
| ENSMUSG00000012422 | Tmem167 | 1.180938 | <0.00001 | up |
| ENSMUSG00000012428 | Steap4 | 3.036407 | <0.00001 | up |
| ENSMUSG00000012848 | Rps5 | 1.165211 | <0.00001 | up |
| ENSMUSG00000014504 | Srp19 | 1.094146 | <0.00001 | up |
| ENSMUSG00000014850 | Msh3 | -1.03917 | <0.00001 | down |
| ENSMUSG00000014867 | Surf4 | 1.549114 | <0.00001 | up |
| ENSMUSG00000014905 | Dnajb9 | 1.688485 | <0.00001 | up |
| ENSMUSG00000015016 | Acsf3 | 1.537884 | <0.00001 | up |
| ENSMUSG00000015090 | Ptgds | -3.49863 | <0.00001 | down |
| ENSMUSG00000015224 | Cyp2j9 | -2.6974 | <0.00001 | down |
| ENSMUSG00000015305 | Sash1 | -1.035 | <0.00001 | down |
| ENSMUSG00000015363 | Trabd | 1.068476 | <0.00001 | up |
| ENSMUSG00000015575 | Atp6v0e | 1.393962 | <0.00001 | up |
| ENSMUSG00000015942 | Gtf2ird2 | -1.44229 | <0.00001 | down |
| ENSMUSG00000016024 | Lbp | 1.934359 | <0.00001 | up |
| ENSMUSG00000016194 | Hsd11b1 | -1.1883 | <0.00001 | down |
| ENSMUSG00000017167 | Cntnap1 | 2.120513 | <0.00001 | up |
| ENSMUSG00000017453 | Pipox | -1.15159 | <0.00001 | down |
| ENSMUSG00000017718 | Afmid | -1.60253 | <0.00001 | down |
| ENSMUSG00000017721 | Pigt | 1.466505 | <0.00001 | up |
| ENSMUSG00000017843 | Ppp2r5c | 1.145307 | <0.00001 | up |
| ENSMUSG00000018411 | Mapt | -2.16456 | <0.00001 | down |
| ENSMUSG00000018565 | Elp5 | 1.02665 | <0.00001 | up |
| ENSMUSG00000018574 | Acadvl | 1.012101 | <0.00001 | up |
| ENSMUSG00000018659 | Pnpo | 1.164679 | <0.00001 | up |
| ENSMUSG00000018669 | Cdk5rap3 | 1.507678 | <0.00001 | up |
| ENSMUSG00000018770 | Atp5g3 | 1.604464 | <0.00001 | up |
| ENSMUSG00000019139 | Isyna1 | 2.307729 | <0.00001 | up |
| ENSMUSG00000019232 | Etnppl | -2.99798 | <0.00001 | down |
| ENSMUSG00000019478 | Rab4a | 1.153154 | <0.00001 | up |
| ENSMUSG00000019494 | Cops6 | 1.826058 | <0.00001 | up |
| ENSMUSG00000019505 | Ubb | 1.383207 | <0.00001 | up |
| ENSMUSG00000019590 | Cyb561 | 3.970654 | <0.00001 | up |
| ENSMUSG00000019731 | Slc35e1 | 1.367864 | <0.00001 | up |
| ENSMUSG00000019935 | Slc17a8 | -1.81123 | <0.00001 | down |
| ENSMUSG00000019969 | Psen1 | 1.000208 | <0.00001 | up |
| ENSMUSG00000019978 | Epb41l2 | -1.16084 | <0.00001 | down |
| ENSMUSG00000020048 | Hsp90b1 | 2.253585 | <0.00001 | up |
| ENSMUSG00000020051 | Pah | -1.26555 | <0.00001 | down |
| ENSMUSG00000020089 | Ppa1 | 2.176976 | <0.00001 | up |
| ENSMUSG00000020372 | Rack1 | 1.018674 | <0.00001 | up |
| ENSMUSG00000020444 | Guk1 | 1.299707 | <0.00001 | up |
| ENSMUSG00000020486 | Septin4 | -1.35084 | <0.00001 | down |
| ENSMUSG00000020538 | Srebf1 | -1.47483 | <0.00001 | down |
| ENSMUSG00000020571 | Pdia6 | 2.186346 | <0.00001 | up |
| ENSMUSG00000020576 | Nbas | 1.105908 | <0.00001 | up |
| ENSMUSG00000020580 | Rock2 | -1.11685 | <0.00001 | down |
| ENSMUSG00000020593 | Lpin1 | -1.98887 | <0.00001 | down |
| ENSMUSG00000020668 | Kif3c | -1.8529 | <0.00001 | down |
| ENSMUSG00000020766 | Galk1 | 1.643706 | <0.00001 | up |
| ENSMUSG00000020777 | Acox1 | 1.704887 | <0.00001 | up |
| ENSMUSG00000020805 | Slc13a5 | 3.866017 | <0.00001 | up |
| ENSMUSG00000020869 | Lrrc59 | 2.429609 | <0.00001 | up |
| ENSMUSG00000020873 | Slc35b1 | 1.516269 | <0.00001 | up |
| ENSMUSG00000020946 | Gosr2 | 1.161194 | <0.00001 | up |
| ENSMUSG00000021069 | Pygl | -1.60469 | <0.00001 | down |
| ENSMUSG00000021091 | Serpina3n | 4.781749 | <0.00001 | up |
| ENSMUSG00000021124 | Vti1b | 1.089451 | <0.00001 | up |
| ENSMUSG00000021186 | Fbln5 | -1.47783 | <0.00001 | down |
| ENSMUSG00000021190 | Lgmn | 1.016134 | <0.00001 | up |
| ENSMUSG00000021268 | Meg3 | -2.19212 | <0.00001 | down |
| ENSMUSG00000021336 | Slc17a4 | -2.01505 | <0.00001 | down |
| ENSMUSG00000021338 | Carmil1 | -1.73082 | <0.00001 | down |
| ENSMUSG00000021477 | Ctsl | 2.267569 | <0.00001 | up |
| ENSMUSG00000021484 | Lman2 | 1.009708 | <0.00001 | up |
| ENSMUSG00000021610 | Clptm1l | 1.571473 | <0.00001 | up |
| ENSMUSG00000021615 | Xrcc4 | 1.987577 | <0.00001 | up |
| ENSMUSG00000021877 | Arf4 | 1.826349 | <0.00001 | up |
| ENSMUSG00000021905 | Dph3 | 1.009367 | <0.00001 | up |
| ENSMUSG00000021917 | Spcs1 | 1.911262 | <0.00001 | up |
| ENSMUSG00000021922 | Itih4 | 3.137002 | <0.00001 | up |
| ENSMUSG00000021928 | Ebpl | 1.202557 | <0.00001 | up |
| ENSMUSG00000021999 | Cpb2 | 2.1093 | <0.00001 | up |
| ENSMUSG00000022037 | Clu | 1.50985 | <0.00001 | up |
| ENSMUSG00000022094 | Slc39a14 | 1.614199 | <0.00001 | up |
| ENSMUSG00000022136 | Dnajc3 | 1.493728 | <0.00001 | up |
| ENSMUSG00000022139 | Mbnl2 | -1.19471 | <0.00001 | down |
| ENSMUSG00000022174 | Dad1 | 1.589552 | <0.00001 | up |
| ENSMUSG00000022323 | Rida | -1.31794 | <0.00001 | down |
| ENSMUSG00000022365 | Derl1 | 1.454296 | <0.00001 | up |
| ENSMUSG00000022371 | Col14a1 | -1.41588 | <0.00001 | down |
| ENSMUSG00000022419 | Deptor | -1.08766 | <0.00001 | down |
| ENSMUSG00000022466 | Rpap3 | 1.774213 | <0.00001 | up |
| ENSMUSG00000022500 | Litaf | 1.645599 | <0.00001 | up |
| ENSMUSG00000022503 | Nubp1 | 1.078502 | <0.00001 | up |
| ENSMUSG00000022505 | Emp2 | -1.67967 | <0.00001 | down |
| ENSMUSG00000022769 | Sdf2l1 | 2.73021 | <0.00001 | up |
| ENSMUSG00000022820 | Ndufb4 | -1.02407 | <0.00001 | down |
| ENSMUSG00000022844 | Pdia5 | 1.472087 | <0.00001 | up |
| ENSMUSG00000022853 | Ehhadh | 1.700567 | <0.00001 | up |
| ENSMUSG00000022894 | Adamts5 | -2.12045 | <0.00001 | down |
| ENSMUSG00000022912 | Pros1 | 1.11114 | <0.00001 | up |
| ENSMUSG00000023043 | Krt18 | 1.119479 | <0.00001 | up |
| ENSMUSG00000023057 | Fabp2 | 1.709097 | <0.00001 | up |
| ENSMUSG00000023073 | Slc10a2 | 1.498855 | <0.00001 | up |
| ENSMUSG00000023104 | Rfc2 | 1.065553 | <0.00001 | up |
| ENSMUSG00000023106 | Denr | 1.219055 | <0.00001 | up |
| ENSMUSG00000023176 | Cpn2 | 1.121138 | <0.00001 | up |
| ENSMUSG00000023224 | Serping1 | 1.161763 | <0.00001 | up |
| ENSMUSG00000023232 | Serinc2 | 2.194908 | <0.00001 | up |
| ENSMUSG00000023272 | Creld2 | 2.495117 | <0.00001 | up |
| ENSMUSG00000023456 | Tpi1 | 1.47268 | <0.00001 | up |
| ENSMUSG00000023988 | Bysl | 1.053131 | <0.00001 | up |
| ENSMUSG00000024018 | Ccdc167 | 1.343399 | <0.00001 | up |
| ENSMUSG00000024109 | Nrxn1 | -1.86296 | <0.00001 | down |
| ENSMUSG00000024131 | Slc3a1 | 1.410522 | <0.00001 | up |
| ENSMUSG00000024150 | Mcfd2 | 1.480331 | <0.00001 | up |
| ENSMUSG00000024164 | C3 | 1.483637 | <0.00001 | up |
| ENSMUSG00000024165 | Jpt2 | 1.684163 | <0.00001 | up |
| ENSMUSG00000024174 | Pot1b | -1.20486 | <0.00001 | down |
| ENSMUSG00000024181 | Mrpl28 | 1.451822 | <0.00001 | up |
| ENSMUSG00000024190 | Dusp1 | -1.92256 | <0.00001 | down |
| ENSMUSG00000024197 | Plin3 | 1.04278 | <0.00001 | up |
| ENSMUSG00000024217 | Snrpc | 1.423613 | <0.00001 | up |
| ENSMUSG00000024248 | Cox7a2l | 1.13085 | <0.00001 | up |
| ENSMUSG00000024292 | Cyp4f14 | -1.22384 | <0.00001 | down |
| ENSMUSG00000024327 | Slc39a7 | 1.215236 | <0.00001 | up |
| ENSMUSG00000024369 | Nelfe | 1.084768 | <0.00001 | up |
| ENSMUSG00000024424 | Ttc39c | -1.74019 | <0.00001 | down |
| ENSMUSG00000024430 | Cabyr | -2.35055 | <0.00001 | down |
| ENSMUSG00000024436 | Mrps18b | 1.627561 | <0.00001 | up |
| ENSMUSG00000024516 | Sec11c | 1.0559 | <0.00001 | up |
| ENSMUSG00000024587 | Nars | 1.240763 | <0.00001 | up |
| ENSMUSG00000024678 | Ms4a4d | -1.7434 | <0.00001 | down |
| ENSMUSG00000024778 | Fas | 1.111763 | <0.00001 | up |
| ENSMUSG00000024875 | Yif1a | 1.642738 | <0.00001 | up |
| ENSMUSG00000024899 | Papss2 | -1.44689 | <0.00001 | down |
| ENSMUSG00000025004 | Cyp2c40 | -2.06283 | <0.00001 | down |
| ENSMUSG00000025089 | Gfra1 | 1.579259 | <0.00001 | up |
| ENSMUSG00000025102 | 3110040N11Rik | 2.203437 | <0.00001 | up |
| ENSMUSG00000025130 | P4hb | 1.808765 | <0.00001 | up |
| ENSMUSG00000025172 | Ankrd2 | 3.826005 | <0.00001 | up |
| ENSMUSG00000025350 | Rdh5 | 1.222105 | <0.00001 | up |
| ENSMUSG00000025353 | Ormdl2 | 1.237992 | <0.00001 | up |
| ENSMUSG00000025381 | Cnpy2 | 1.170499 | <0.00001 | up |
| ENSMUSG00000025396 | Hsd17b6 | -1.70779 | <0.00001 | down |
| ENSMUSG00000025481 | Urah | 1.470479 | <0.00001 | up |
| ENSMUSG00000025487 | Psmd13 | 2.074659 | <0.00001 | up |
| ENSMUSG00000025508 | Rplp2 | 1.378543 | <0.00001 | up |
| ENSMUSG00000025511 | Tspan4 | 1.367185 | <0.00001 | up |
| ENSMUSG00000025512 | Chid1 | 1.073913 | <0.00001 | up |
| ENSMUSG00000025574 | Tk1 | -2.66992 | <0.00001 | down |
| ENSMUSG00000025724 | Sec11a | 1.230834 | <0.00001 | up |
| ENSMUSG00000025815 | Dhtkd1 | -1.09306 | <0.00001 | down |
| ENSMUSG00000025816 | Sec61a2 | -1.63384 | <0.00001 | down |
| ENSMUSG00000025823 | Pdia4 | 1.967764 | <0.00001 | up |
| ENSMUSG00000025858 | Get4 | 1.046214 | <0.00001 | up |
| ENSMUSG00000025967 | Eef1b2 | 1.61314 | <0.00001 | up |
| ENSMUSG00000026004 | Kansl1l | -1.32221 | <0.00001 | down |
| ENSMUSG00000026043 | Col3a1 | -1.5857 | <0.00001 | down |
| ENSMUSG00000026048 | Ercc5 | -1.23319 | <0.00001 | down |
| ENSMUSG00000026193 | Fn1 | 1.995872 | <0.00001 | up |
| ENSMUSG00000026222 | Sp100 | -1.24324 | <0.00001 | down |
| ENSMUSG00000026223 | Itm2c | 1.841162 | <0.00001 | up |
| ENSMUSG00000026259 | Ngef | -1.55588 | <0.00001 | down |
| ENSMUSG00000026275 | Ppp1r7 | 1.225486 | <0.00001 | up |
| ENSMUSG00000026342 | Slc35f5 | 1.934256 | <0.00001 | up |
| ENSMUSG00000026348 | Acmsd | -2.07214 | <0.00001 | down |
| ENSMUSG00000026365 | Cfh | 1.115139 | <0.00001 | up |
| ENSMUSG00000026390 | Marco | 1.834741 | <0.00001 | up |
| ENSMUSG00000026405 | C4bp | 1.315193 | <0.00001 | up |
| ENSMUSG00000026427 | Eif2d | 1.287951 | <0.00001 | up |
| ENSMUSG00000026542 | Apcs | 4.665689 | <0.00001 | up |
| ENSMUSG00000026558 | Uck2 | 1.275218 | <0.00001 | up |
| ENSMUSG00000026623 | Lpgat1 | 1.991208 | <0.00001 | up |
| ENSMUSG00000026688 | Mgst3 | -1.31654 | <0.00001 | down |
| ENSMUSG00000026822 | Lcn2 | 8.925879 | <0.00001 | up |
| ENSMUSG00000026839 | Upp2 | -3.81088 | <0.00001 | down |
| ENSMUSG00000026864 | Hspa5 | 1.66765 | <0.00001 | up |
| ENSMUSG00000026872 | Zeb2 | -1.40036 | <0.00001 | down |
| ENSMUSG00000026887 | Mrrf | 1.052191 | <0.00001 | up |
| ENSMUSG00000026956 | Uap1l1 | 1.815909 | <0.00001 | up |
| ENSMUSG00000027006 | Dnajc10 | 1.594746 | <0.00001 | up |
| ENSMUSG00000027012 | Dync1i2 | -1.04765 | <0.00001 | down |
| ENSMUSG00000027048 | Abcb11 | -1.10454 | <0.00001 | down |
| ENSMUSG00000027086 | Fastkd1 | -1.09324 | <0.00001 | down |
| ENSMUSG00000027248 | Pdia3 | 1.481394 | <0.00001 | up |
| ENSMUSG00000027313 | Chac1 | 2.958255 | <0.00001 | up |
| ENSMUSG00000027430 | Dtd1 | 1.403605 | <0.00001 | up |
| ENSMUSG00000027506 | Tpd52 | 1.09735 | <0.00001 | up |
| ENSMUSG00000027533 | Fabp5 | 2.82636 | <0.00001 | up |
| ENSMUSG00000027540 | Ptpn1 | 1.105996 | <0.00001 | up |
| ENSMUSG00000027546 | Atp9a | 1.184309 | <0.00001 | up |
| ENSMUSG00000027556 | Car1 | -2.57094 | <0.00001 | down |
| ENSMUSG00000027559 | Car3 | -2.51764 | <0.00001 | down |
| ENSMUSG00000027613 | Eif6 | 2.275025 | <0.00001 | up |
| ENSMUSG00000027763 | Mbnl1 | -1.10813 | <0.00001 | down |
| ENSMUSG00000027828 | Ssr3 | 1.667268 | <0.00001 | up |
| ENSMUSG00000027879 | Sec22b | 1.406374 | <0.00001 | up |
| ENSMUSG00000027942 | 4933434E20Rik | 1.522322 | <0.00001 | up |
| ENSMUSG00000027999 | Pla2g12a | 1.42783 | <0.00001 | up |
| ENSMUSG00000028001 | Fga | 3.312529 | <0.00001 | up |
| ENSMUSG00000028051 | Hcn3 | -2.99055 | <0.00001 | down |
| ENSMUSG00000028063 | Lmna | 1.238324 | <0.00001 | up |
| ENSMUSG00000028081 | Rps3a1 | 1.325228 | <0.00001 | up |
| ENSMUSG00000028223 | Decr1 | 1.156206 | <0.00001 | up |
| ENSMUSG00000028248 | Pnisr | -1.05982 | <0.00001 | down |
| ENSMUSG00000028268 | Gbp3 | -2.15842 | <0.00001 | down |
| ENSMUSG00000028334 | Nans | 1.543289 | <0.00001 | up |
| ENSMUSG00000028339 | Col15a1 | 1.340268 | <0.00001 | up |
| ENSMUSG00000028356 | Ambp | 1.177591 | <0.00001 | up |
| ENSMUSG00000028399 | Ptprd | -1.09474 | <0.00001 | down |
| ENSMUSG00000028419 | Chmp5 | 1.309335 | <0.00001 | up |
| ENSMUSG00000028467 | Gba2 | -1.16673 | <0.00001 | down |
| ENSMUSG00000028494 | Plin2 | 2.084472 | <0.00001 | up |
| ENSMUSG00000028553 | Angptl3 | -2.15925 | <0.00001 | down |
| ENSMUSG00000028641 | P3h1 | 1.036059 | <0.00001 | up |
| ENSMUSG00000028757 | Ddost | 1.430027 | <0.00001 | up |
| ENSMUSG00000028849 | Map7d1 | 1.340503 | <0.00001 | up |
| ENSMUSG00000028980 | H6pd | 1.113476 | <0.00001 | up |
| ENSMUSG00000029038 | Ssu72 | 1.803043 | <0.00001 | up |
| ENSMUSG00000029073 | Cptp | 1.401161 | <0.00001 | up |
| ENSMUSG00000029161 | Cgref1 | 2.446638 | <0.00001 | up |
| ENSMUSG00000029185 | Fam114a1 | 1.401625 | <0.00001 | up |
| ENSMUSG00000029260 | Ugt2b34 | -1.21604 | <0.00001 | down |
| ENSMUSG00000029272 | Sult1e1 | 4.055296 | <0.00001 | up |
| ENSMUSG00000029273 | Sult1d1 | -1.7074 | <0.00001 | down |
| ENSMUSG00000029352 | Crybb3 | 5.385 | <0.00001 | up |
| ENSMUSG00000029380 | Cxcl1 | 4.773689 | <0.00001 | up |
| ENSMUSG00000029390 | Tmed2 | 1.466947 | <0.00001 | up |
| ENSMUSG00000029407 | Uso1 | 1.146142 | <0.00001 | up |
| ENSMUSG00000029430 | Ran | 1.37334 | <0.00001 | up |
| ENSMUSG00000029545 | Acads | 1.37707 | <0.00001 | up |
| ENSMUSG00000029552 | Tes | 1.618829 | <0.00001 | up |
| ENSMUSG00000029591 | Ung | 1.896561 | <0.00001 | up |
| ENSMUSG00000029596 | Sdsl | 2.034131 | <0.00001 | up |
| ENSMUSG00000029599 | Ddx54 | 1.134602 | <0.00001 | up |
| ENSMUSG00000029610 | Aimp2 | 1.997493 | <0.00001 | up |
| ENSMUSG00000029616 | Erp29 | 1.190498 | <0.00001 | up |
| ENSMUSG00000029630 | Cyp3a25 | -1.83759 | <0.00001 | down |
| ENSMUSG00000029647 | Pan3 | -1.06197 | <0.00001 | down |
| ENSMUSG00000029727 | Cyp3a13 | 1.834531 | <0.00001 | up |
| ENSMUSG00000029752 | Asns | 3.154838 | <0.00001 | up |
| ENSMUSG00000029767 | Calu | 1.64884 | <0.00001 | up |
| ENSMUSG00000029777 | Gars | 1.124187 | <0.00001 | up |
| ENSMUSG00000030062 | Rpn1 | 1.806628 | <0.00001 | up |
| ENSMUSG00000030082 | Sec61a1 | 1.431553 | <0.00001 | up |
| ENSMUSG00000030111 | A2m | 10.26354 | <0.00001 | up |
| ENSMUSG00000030122 | Ptms | -1.44715 | <0.00001 | down |
| ENSMUSG00000030126 | Tmcc1 | -1.09926 | <0.00001 | down |
| ENSMUSG00000030237 | Slco1a4 | -2.23244 | <0.00001 | down |
| ENSMUSG00000030278 | Cidec | 5.61527 | <0.00001 | up |
| ENSMUSG00000030287 | Itpr2 | -1.85212 | <0.00001 | down |
| ENSMUSG00000030298 | Sec13 | 1.26023 | <0.00001 | up |
| ENSMUSG00000030339 | Ltbr | 1.144173 | <0.00001 | up |
| ENSMUSG00000030341 | Tnfrsf1a | 1.625463 | <0.00001 | up |
| ENSMUSG00000030359 | Pzp | 1.393804 | <0.00001 | up |
| ENSMUSG00000030378 | Sult2a8 | -1.15902 | <0.00001 | down |
| ENSMUSG00000030591 | Psmd8 | 1.235817 | <0.00001 | up |
| ENSMUSG00000030612 | Mrpl46 | 1.405042 | <0.00001 | up |
| ENSMUSG00000030652 | Coq7 | 1.550101 | <0.00001 | up |
| ENSMUSG00000030659 | Nucb2 | 2.98312 | <0.00001 | up |
| ENSMUSG00000030681 | Mvp | 1.68423 | <0.00001 | up |
| ENSMUSG00000030731 | Syt3 | -2.55653 | <0.00001 | down |
| ENSMUSG00000030750 | Nsmce1 | 1.746677 | <0.00001 | up |
| ENSMUSG00000030852 | Tacc2 | 2.334012 | <0.00001 | up |
| ENSMUSG00000030879 | Mrpl17 | 1.852896 | <0.00001 | up |
| ENSMUSG00000030895 | Hpx | 2.924438 | <0.00001 | up |
| ENSMUSG00000030968 | Pdilt | -1.78837 | <0.00001 | down |
| ENSMUSG00000030972 | Acsm5 | -1.01086 | <0.00001 | down |
| ENSMUSG00000031029 | Eif3f | 1.590155 | <0.00001 | up |
| ENSMUSG00000031167 | Rbm3 | 1.438292 | <0.00001 | up |
| ENSMUSG00000031173 | Otc | -1.022 | <0.00001 | down |
| ENSMUSG00000031242 | 2610002M06Rik | 1.088528 | <0.00001 | up |
| ENSMUSG00000031245 | Hmgn5 | -1.25063 | <0.00001 | down |
| ENSMUSG00000031320 | Rps4x | 1.286152 | <0.00001 | up |
| ENSMUSG00000031422 | Morf4l2 | 1.073997 | <0.00001 | up |
| ENSMUSG00000031451 | Gas6 | 1.693335 | <0.00001 | up |
| ENSMUSG00000031490 | Eif4ebp1 | 1.223608 | <0.00001 | up |
| ENSMUSG00000031570 | Plpp5 | 2.388004 | <0.00001 | up |
| ENSMUSG00000031594 | Fgl1 | 2.89671 | <0.00001 | up |
| ENSMUSG00000031595 | Pdgfrl | -4.76867 | <0.00001 | down |
| ENSMUSG00000031634 | Ufsp2 | 1.080771 | <0.00001 | up |
| ENSMUSG00000031722 | Hp | 3.027162 | <0.00001 | up |
| ENSMUSG00000031725 | Ces1f | -1.52121 | <0.00001 | down |
| ENSMUSG00000031762 | Mt2 | 4.19912 | <0.00001 | up |
| ENSMUSG00000031765 | Mt1 | 4.139176 | <0.00001 | up |
| ENSMUSG00000031805 | Jak3 | 1.918609 | <0.00001 | up |
| ENSMUSG00000031812 | Map1lc3b | 1.319391 | <0.00001 | up |
| ENSMUSG00000031824 | 6430548M08Rik | 1.248871 | <0.00001 | up |
| ENSMUSG00000031842 | Pde4c | -1.53778 | <0.00001 | down |
| ENSMUSG00000031848 | Lsm4 | 1.745967 | <0.00001 | up |
| ENSMUSG00000031906 | Smpd3 | 3.20744 | <0.00001 | up |
| ENSMUSG00000032010 | Usp2 | -1.97036 | <0.00001 | down |
| ENSMUSG00000032026 | Rexo2 | 1.221068 | <0.00001 | up |
| ENSMUSG00000032042 | Srpr | 1.528455 | <0.00001 | up |
| ENSMUSG00000032050 | Rdx | -1.00881 | <0.00001 | down |
| ENSMUSG00000032077 | Bud13 | -1.64891 | <0.00001 | down |
| ENSMUSG00000032080 | Apoa4 | 2.702979 | <0.00001 | up |
| ENSMUSG00000032115 | Hyou1 | 1.73821 | <0.00001 | up |
| ENSMUSG00000032116 | Stt3a | 1.270521 | <0.00001 | up |
| ENSMUSG00000032123 | Dpagt1 | 1.039314 | <0.00001 | up |
| ENSMUSG00000032125 | Robo4 | -1.48807 | <0.00001 | down |
| ENSMUSG00000032126 | Hmbs | 1.13156 | <0.00001 | up |
| ENSMUSG00000032177 | Pde4a | -1.93313 | <0.00001 | down |
| ENSMUSG00000032198 | Dock6 | -1.59713 | <0.00001 | down |
| ENSMUSG00000032207 | Lipc | -1.11819 | <0.00001 | down |
| ENSMUSG00000032271 | Nnmt | 2.690332 | <0.00001 | up |
| ENSMUSG00000032350 | Gclc | -1.4993 | <0.00001 | down |
| ENSMUSG00000032353 | Tmed3 | 2.032898 | <0.00001 | up |
| ENSMUSG00000032383 | Ppib | 1.643458 | <0.00001 | up |
| ENSMUSG00000032388 | Spg21 | 1.028492 | <0.00001 | up |
| ENSMUSG00000032399 | Rpl4 | 1.408357 | <0.00001 | up |
| ENSMUSG00000032437 | Stt3b | 1.309222 | <0.00001 | up |
| ENSMUSG00000032458 | Copb2 | 1.381232 | <0.00001 | up |
| ENSMUSG00000032478 | Nme6 | 1.990699 | <0.00001 | up |
| ENSMUSG00000032553 | Srprb | 1.529187 | <0.00001 | up |
| ENSMUSG00000032575 | Manf | 2.068179 | <0.00001 | up |
| ENSMUSG00000032744 | Heyl | -1.71726 | <0.00001 | down |
| ENSMUSG00000032802 | Srxn1 | 1.17671 | <0.00001 | up |
| ENSMUSG00000032845 | Alpk2 | -2.67519 | <0.00001 | down |
| ENSMUSG00000032902 | Slc16a1 | 1.38127 | <0.00001 | up |
| ENSMUSG00000032932 | Hspa13 | 1.948991 | <0.00001 | up |
| ENSMUSG00000032966 | Fkbp1a | 1.105381 | <0.00001 | up |
| ENSMUSG00000033107 | Rnf125 | -1.85404 | <0.00001 | down |
| ENSMUSG00000033161 | Atp1a1 | 1.187259 | <0.00001 | up |
| ENSMUSG00000033326 | Kdm4a | 1.052045 | <0.00001 | up |
| ENSMUSG00000033327 | Tnxb | -1.5475 | <0.00001 | down |
| ENSMUSG00000033355 | Rtp4 | -1.2334 | <0.00001 | down |
| ENSMUSG00000033577 | Myo6 | -1.06386 | <0.00001 | down |
| ENSMUSG00000033629 | Hacd3 | 1.583312 | <0.00001 | up |
| ENSMUSG00000033634 | Nat8f2 | 1.116776 | <0.00001 | up |
| ENSMUSG00000033684 | Qsox1 | 1.881612 | <0.00001 | up |
| ENSMUSG00000033713 | Foxn3 | -1.45465 | <0.00001 | down |
| ENSMUSG00000033715 | Akr1c14 | -1.36405 | <0.00001 | down |
| ENSMUSG00000033831 | Fgb | 2.996213 | <0.00001 | up |
| ENSMUSG00000033860 | Fgg | 3.149411 | <0.00001 | up |
| ENSMUSG00000033917 | Gde1 | 1.27607 | <0.00001 | up |
| ENSMUSG00000033943 | Mga | -1.00476 | <0.00001 | down |
| ENSMUSG00000034037 | Fgd5 | -1.19074 | <0.00001 | down |
| ENSMUSG00000034438 | Gbp8 | -1.76728 | <0.00001 | down |
| ENSMUSG00000034456 | Uroc1 | -1.21814 | <0.00001 | down |
| ENSMUSG00000034591 | Slc41a2 | 2.579696 | <0.00001 | up |
| ENSMUSG00000034634 | Ly6d | 3.909653 | <0.00001 | up |
| ENSMUSG00000034708 | Grn | 1.463251 | <0.00001 | up |
| ENSMUSG00000034771 | Tle2 | -1.7449 | <0.00001 | down |
| ENSMUSG00000034807 | Colgalt1 | 1.026281 | <0.00001 | up |
| ENSMUSG00000034837 | Gnat1 | 3.133073 | <0.00001 | up |
| ENSMUSG00000034926 | Dhcr24 | 1.809687 | <0.00001 | up |
| ENSMUSG00000034974 | Dapk3 | 1.238906 | <0.00001 | up |
| ENSMUSG00000034994 | Eef2 | 1.142043 | <0.00001 | up |
| ENSMUSG00000035112 | Wnk4 | -2.23676 | <0.00001 | down |
| ENSMUSG00000035227 | Spcs2 | 1.265622 | <0.00001 | up |
| ENSMUSG00000035268 | Pkig | 1.129901 | <0.00001 | up |
| ENSMUSG00000035357 | Pdzrn3 | 1.627778 | <0.00001 | up |
| ENSMUSG00000035413 | Tmem98 | 1.671191 | <0.00001 | up |
| ENSMUSG00000035597 | Prpf39 | -1.08497 | <0.00001 | down |
| ENSMUSG00000035637 | Grhpr | 1.670259 | <0.00001 | up |
| ENSMUSG00000035666 | Gtf3c4 | 1.15305 | <0.00001 | up |
| ENSMUSG00000035686 | Thrsp | -2.64787 | <0.00001 | down |
| ENSMUSG00000035845 | Alg12 | 1.188441 | <0.00001 | up |
| ENSMUSG00000036138 | Acaa1a | 1.014025 | <0.00001 | up |
| ENSMUSG00000036181 | H1f2 | 1.720625 | <0.00001 | up |
| ENSMUSG00000036372 | Tmem258 | 1.115201 | <0.00001 | up |
| ENSMUSG00000036446 | Lum | -1.5413 | <0.00001 | down |
| ENSMUSG00000036764 | Dnajc12 | 3.551043 | <0.00001 | up |
| ENSMUSG00000036966 | Spryd3 | 1.443935 | <0.00001 | up |
| ENSMUSG00000037071 | Scd1 | -1.37103 | <0.00001 | down |
| ENSMUSG00000037072 | Selenof | 1.204784 | <0.00001 | up |
| ENSMUSG00000037095 | Lrg1 | 4.274347 | <0.00001 | up |
| ENSMUSG00000037287 | Tbcel | -1.01516 | <0.00001 | down |
| ENSMUSG00000037563 | Rps16 | 1.064864 | <0.00001 | up |
| ENSMUSG00000037679 | Inf2 | 1.056366 | <0.00001 | up |
| ENSMUSG00000037798 | Mat1a | 1.119609 | <0.00001 | up |
| ENSMUSG00000037820 | Tgm2 | 1.351764 | <0.00001 | up |
| ENSMUSG00000037847 | Nmrk1 | -1.65743 | <0.00001 | down |
| ENSMUSG00000037942 | Crp | 1.358079 | <0.00001 | up |
| ENSMUSG00000038188 | Scarf1 | -1.61105 | <0.00001 | down |
| ENSMUSG00000038213 | Tapbpl | 1.051165 | <0.00001 | up |
| ENSMUSG00000038295 | Atg9b | 6.209115 | <0.00001 | up |
| ENSMUSG00000038301 | Snx10 | 1.621124 | <0.00001 | up |
| ENSMUSG00000038312 | Edem2 | 1.056223 | <0.00001 | up |
| ENSMUSG00000038372 | Gmds | 4.009243 | <0.00001 | up |
| ENSMUSG00000038467 | Chmp4b | 1.215698 | <0.00001 | up |
| ENSMUSG00000038526 | Car14 | -1.69202 | <0.00001 | down |
| ENSMUSG00000038539 | Atf5 | 1.843301 | <0.00001 | up |
| ENSMUSG00000038552 | Fndc4 | 1.028179 | <0.00001 | up |
| ENSMUSG00000038600 | Atp6v0a4 | 3.884476 | <0.00001 | up |
| ENSMUSG00000038641 | Akr1d1 | -2.09933 | <0.00001 | down |
| ENSMUSG00000038754 | Elovl3 | -3.03098 | <0.00001 | down |
| ENSMUSG00000038871 | Bpgm | 1.061481 | <0.00001 | up |
| ENSMUSG00000038917 | 3930402G23Rik | 2.90879 | <0.00001 | up |
| ENSMUSG00000039018 | Mtg1 | 1.656436 | <0.00001 | up |
| ENSMUSG00000039065 | Atpsckmt | 1.091927 | <0.00001 | up |
| ENSMUSG00000039156 | Stim2 | -1.10198 | <0.00001 | down |
| ENSMUSG00000039196 | Orm1 | 3.454935 | <0.00001 | up |
| ENSMUSG00000039234 | Sec24d | 1.281881 | <0.00001 | up |
| ENSMUSG00000039286 | Fndc3b | 1.39492 | <0.00001 | up |
| ENSMUSG00000039474 | Wfs1 | 1.909883 | <0.00001 | up |
| ENSMUSG00000039601 | Rcan2 | 2.084452 | <0.00001 | up |
| ENSMUSG00000039886 | Tmem120a | 1.704389 | <0.00001 | up |
| ENSMUSG00000039997 | Ifi203 | -1.54092 | <0.00001 | down |
| ENSMUSG00000040017 | Saa4 | 1.479387 | <0.00001 | up |
| ENSMUSG00000040026 | Saa3 | 5.09319 | <0.00001 | up |
| ENSMUSG00000040488 | Ltbp4 | -1.13944 | <0.00001 | down |
| ENSMUSG00000040505 | Abcg5 | -1.32677 | <0.00001 | down |
| ENSMUSG00000040612 | Ildr2 | 2.313422 | <0.00001 | up |
| ENSMUSG00000040809 | Chil3 | 4.732427 | <0.00001 | up |
| ENSMUSG00000040904 | Gm21988 | 2.147793 | <0.00001 | up |
| ENSMUSG00000041044 | Lrit1 | -1.83974 | <0.00001 | down |
| ENSMUSG00000041084 | Ostc | 1.856361 | <0.00001 | up |
| ENSMUSG00000041355 | Ssr2 | 1.658291 | <0.00001 | up |
| ENSMUSG00000041698 | Slco1a1 | -1.40579 | <0.00001 | down |
| ENSMUSG00000041736 | Tspo | 1.78694 | <0.00001 | up |
| ENSMUSG00000041828 | Abca8a | -2.13852 | <0.00001 | down |
| ENSMUSG00000041959 | S100a10 | 1.289524 | <0.00001 | up |
| ENSMUSG00000042041 | 2010003K11Rik | 2.686636 | <0.00001 | up |
| ENSMUSG00000042248 | Cyp2c37 | -1.58072 | <0.00001 | down |
| ENSMUSG00000042389 | Tsen2 | 2.25662 | <0.00001 | up |
| ENSMUSG00000042453 | Reln | -1.4845 | <0.00001 | down |
| ENSMUSG00000042638 | Gucy2c | 4.607825 | <0.00001 | up |
| ENSMUSG00000042834 | Nrep | -1.7425 | <0.00001 | down |
| ENSMUSG00000043183 | Simc1 | -1.63657 | <0.00001 | down |
| ENSMUSG00000043286 | Pnpla1 | -1.72127 | <0.00001 | down |
| ENSMUSG00000043418 | Lrit2 | -2.447 | <0.00001 | down |
| ENSMUSG00000044005 | Gls2 | -1.42232 | <0.00001 | down |
| ENSMUSG00000045503 | Sys1 | 1.193434 | <0.00001 | up |
| ENSMUSG00000045538 | Ddx28 | 2.570838 | <0.00001 | up |
| ENSMUSG00000045790 | Ccdc149 | 2.605938 | <0.00001 | up |
| ENSMUSG00000045948 | Mrps12 | 1.496507 | <0.00001 | up |
| ENSMUSG00000046312 | Myorg | 1.601066 | <0.00001 | up |
| ENSMUSG00000046324 | Ermp1 | 1.304168 | <0.00001 | up |
| ENSMUSG00000046338 | Gpat2 | -2.36816 | <0.00001 | down |
| ENSMUSG00000046811 | Gltpd2 | 1.54575 | <0.00001 | up |
| ENSMUSG00000047123 | Ticam1 | 1.27224 | <0.00001 | up |
| ENSMUSG00000047228 | A2ml1 | 1.479359 | <0.00001 | up |
| ENSMUSG00000047230 | Cldn2 | 1.886195 | <0.00001 | up |
| ENSMUSG00000047492 | Inhbe | 1.809065 | <0.00001 | up |
| ENSMUSG00000047547 | Cltb | 1.414957 | <0.00001 | up |
| ENSMUSG00000047617 | Paxx | -2.05595 | <0.00001 | down |
| ENSMUSG00000047793 | Sned1 | -1.606 | <0.00001 | down |
| ENSMUSG00000048379 | Socs4 | -1.27299 | <0.00001 | down |
| ENSMUSG00000048578 | Mlec | 1.300878 | <0.00001 | up |
| ENSMUSG00000048755 | Mcat | 1.564643 | <0.00001 | up |
| ENSMUSG00000048938 | Nr1h5 | -2.04555 | <0.00001 | down |
| ENSMUSG00000049044 | Rapgef4 | -1.3275 | <0.00001 | down |
| ENSMUSG00000049047 | Armcx3 | 1.456119 | <0.00001 | up |
| ENSMUSG00000049303 | Syt12 | 4.640524 | <0.00001 | up |
| ENSMUSG00000049382 | Krt8 | 1.499279 | <0.00001 | up |
| ENSMUSG00000049404 | Rarres1 | 2.480889 | <0.00001 | up |
| ENSMUSG00000049517 | Rps23 | 1.371236 | <0.00001 | up |
| ENSMUSG00000049555 | Tmie | -1.4871 | <0.00001 | down |
| ENSMUSG00000049600 | Zbtb45 | -1.92044 | <0.00001 | down |
| ENSMUSG00000049971 | Glt1d1 | -1.48971 | <0.00001 | down |
| ENSMUSG00000050240 | Hic2 | -1.77932 | <0.00001 | down |
| ENSMUSG00000050299 | Gm9843 | -8.09558 | <0.00001 | down |
| ENSMUSG00000051238 | Swsap1 | 1.796502 | <0.00001 | up |
| ENSMUSG00000051716 | Apon | 1.207612 | <0.00001 | up |
| ENSMUSG00000051748 | Wfdc21 | 1.961929 | <0.00001 | up |
| ENSMUSG00000051790 | Nlgn2 | -1.74671 | <0.00001 | down |
| ENSMUSG00000052151 | Plpp2 | 1.394645 | <0.00001 | up |
| ENSMUSG00000052271 | Bhlha15 | 3.889041 | <0.00001 | up |
| ENSMUSG00000052310 | Slc39a1 | 1.284689 | <0.00001 | up |
| ENSMUSG00000052560 | Cpne8 | 2.656009 | <0.00001 | up |
| ENSMUSG00000052562 | Slc22a30 | -2.05122 | <0.00001 | down |
| ENSMUSG00000052726 | Kcnt2 | 3.542024 | <0.00001 | up |
| ENSMUSG00000053094 | Tmem248 | 1.57175 | <0.00001 | up |
| ENSMUSG00000053113 | Socs3 | 2.724042 | <0.00001 | up |
| ENSMUSG00000053128 | Rnf26 | 1.337112 | <0.00001 | up |
| ENSMUSG00000053175 | Bcl3 | 1.384302 | <0.00001 | up |
| ENSMUSG00000053317 | Sec61b | 2.134512 | <0.00001 | up |
| ENSMUSG00000053334 | Ficd | 1.235279 | <0.00001 | up |
| ENSMUSG00000053898 | Ech1 | 1.030488 | <0.00001 | up |
| ENSMUSG00000054252 | Fgfr3 | -1.28352 | <0.00001 | down |
| ENSMUSG00000054263 | Lifr | -1.5303 | <0.00001 | down |
| ENSMUSG00000054277 | Arfgap3 | 1.595133 | <0.00001 | up |
| ENSMUSG00000054408 | Spcs3 | 1.494335 | <0.00001 | up |
| ENSMUSG00000054580 | Pla2r1 | -2.34525 | <0.00001 | down |
| ENSMUSG00000054630 | Ugt2b5 | -1.3576 | <0.00001 | down |
| ENSMUSG00000054827 | Cyp2c50 | -1.42195 | <0.00001 | down |
| ENSMUSG00000055027 | Smyd1 | 2.911369 | <0.00001 | up |
| ENSMUSG00000055240 | Zfp101 | -1.23924 | <0.00001 | down |
| ENSMUSG00000055312 | Them7 | 1.309176 | <0.00001 | up |
| ENSMUSG00000055681 | Cope | 1.075422 | <0.00001 | up |
| ENSMUSG00000056071 | S100a9 | 2.776531 | <0.00001 | up |
| ENSMUSG00000056131 | Pgm3 | 1.471208 | <0.00001 | up |
| ENSMUSG00000056313 | Tcim | -1.39534 | <0.00001 | down |
| ENSMUSG00000056492 | Adgrf5 | -1.032 | <0.00001 | down |
| ENSMUSG00000056978 | Hamp2 | -3.36219 | <0.00001 | down |
| ENSMUSG00000057036 | Gm7536 | 1.136776 | <0.00001 | up |
| ENSMUSG00000057465 | Saa2 | 11.62473 | <0.00001 | up |
| ENSMUSG00000057729 | Prtn3 | 6.178813 | <0.00001 | up |
| ENSMUSG00000058135 | Gstm1 | -1.31294 | <0.00001 | down |
| ENSMUSG00000058173 | Smco4 | 1.669303 | <0.00001 | up |
| ENSMUSG00000058207 | Serpina3k | 1.249674 | <0.00001 | up |
| ENSMUSG00000058486 | Wdr91 | -2.06066 | <0.00001 | down |
| ENSMUSG00000058569 | Tmed9 | 1.542972 | <0.00001 | up |
| ENSMUSG00000059040 | Eno1b | -7.49172 | <0.00001 | down |
| ENSMUSG00000059316 | Slc27a4 | 1.388554 | <0.00001 | up |
| ENSMUSG00000059434 | Gckr | -1.34735 | <0.00001 | down |
| ENSMUSG00000059714 | Flot1 | 1.852544 | <0.00001 | up |
| ENSMUSG00000060036 | Rpl3 | 1.549082 | <0.00001 | up |
| ENSMUSG00000060419 | Rps16-ps2 | 1.408577 | <0.00001 | up |
| ENSMUSG00000060487 | Samd5 | -1.99244 | <0.00001 | down |
| ENSMUSG00000060591 | Ifitm2 | 1.802359 | <0.00001 | up |
| ENSMUSG00000060703 | Cd302 | 1.526932 | <0.00001 | up |
| ENSMUSG00000060802 | B2m | -1.22053 | <0.00001 | down |
| ENSMUSG00000060807 | Serpina6 | -2.08751 | <0.00001 | down |
| ENSMUSG00000060961 | Slc4a4 | 2.329756 | <0.00001 | up |
| ENSMUSG00000061292 | Cyp3a59 | -1.98738 | <0.00001 | down |
| ENSMUSG00000061306 | Slc38a10 | 1.146555 | <0.00001 | up |
| ENSMUSG00000061477 | Rps7 | 1.22327 | <0.00001 | up |
| ENSMUSG00000061540 | Orm2 | 5.657053 | <0.00001 | up |
| ENSMUSG00000061947 | Serpina10 | 2.904016 | <0.00001 | up |
| ENSMUSG00000061981 | Flot2 | 1.0977 | <0.00001 | up |
| ENSMUSG00000061983 | Rps12 | 1.244648 | <0.00001 | up |
| ENSMUSG00000062181 | Ces3b | -1.12015 | <0.00001 | down |
| ENSMUSG00000062515 | Fabp4 | 2.281426 | <0.00001 | up |
| ENSMUSG00000062619 | 2310039H08Rik | 1.296999 | <0.00001 | up |
| ENSMUSG00000062825 | Actg1 | 1.822291 | <0.00001 | up |
| ENSMUSG00000062963 | Ufc1 | 1.077129 | <0.00001 | up |
| ENSMUSG00000063229 | Ldha | 3.158675 | <0.00001 | up |
| ENSMUSG00000063450 | Syne2 | -1.02449 | <0.00001 | down |
| ENSMUSG00000063558 | Aox1 | -1.06057 | <0.00001 | down |
| ENSMUSG00000063730 | Hsd3b2 | -2.54526 | <0.00001 | down |
| ENSMUSG00000064225 | Paqr9 | 1.059457 | <0.00001 | up |
| ENSMUSG00000064246 | Chil1 | 7.806866 | <0.00001 | up |
| ENSMUSG00000066150 | Slc31a1 | 1.031547 | <0.00001 | up |
| ENSMUSG00000066258 | Trim12a | -1.45161 | <0.00001 | down |
| ENSMUSG00000066263 | Olfr639 | -2.02806 | <0.00001 | down |
| ENSMUSG00000067149 | Jchain | -1.71647 | <0.00001 | down |
| ENSMUSG00000067225 | Cyp2c54 | -1.98849 | <0.00001 | down |
| ENSMUSG00000067274 | Rplp0 | 1.408418 | <0.00001 | up |
| ENSMUSG00000068011 | Mkrn2os | 1.451043 | <0.00001 | up |
| ENSMUSG00000068245 | Phf11d | -1.49128 | <0.00001 | down |
| ENSMUSG00000068246 | Apol9b | -1.78534 | <0.00001 | down |
| ENSMUSG00000068263 | Efcc1 | -2.00381 | <0.00001 | down |
| ENSMUSG00000068749 | Psma5 | 1.458942 | <0.00001 | up |
| ENSMUSG00000068877 | Selenbp2 | -2.49057 | <0.00001 | down |
| ENSMUSG00000070284 | Gmppb | 1.755526 | <0.00001 | up |
| ENSMUSG00000070427 | Il18bp | 1.519331 | <0.00001 | up |
| ENSMUSG00000071176 | Arhgef10 | -1.40532 | <0.00001 | down |
| ENSMUSG00000071415 | Rpl23 | 1.073867 | <0.00001 | up |
| ENSMUSG00000071644 | Eef1g | 1.18465 | <0.00001 | up |
| ENSMUSG00000071708 | Sms | -1.56313 | <0.00001 | down |
| ENSMUSG00000071856 | Mcc | -1.02233 | <0.00001 | down |
| ENSMUSG00000072115 | Ang | 2.036618 | <0.00001 | up |
| ENSMUSG00000073079 | Srp54a | 1.136126 | <0.00001 | up |
| ENSMUSG00000073409 | H2-Q6 | -1.93103 | <0.00001 | down |
| ENSMUSG00000073758 | Sh3d21 | -1.78861 | <0.00001 | down |
| ENSMUSG00000073888 | Ccl27a | -1.48414 | <0.00001 | down |
| ENSMUSG00000074115 | Saa1 | 9.597358 | <0.00001 | up |
| ENSMUSG00000074211 | Sdhaf1 | 1.089333 | <0.00001 | up |
| ENSMUSG00000074649 | BC029722 | 1.027449 | <0.00001 | up |
| ENSMUSG00000075256 | Cerkl | 2.490696 | <0.00001 | up |
| ENSMUSG00000075701 | Selenos | 2.175835 | <0.00001 | up |
| ENSMUSG00000076431 | Sox4 | -2.33796 | <0.00001 | down |
| ENSMUSG00000076609 | Igkc | -1.85223 | <0.00001 | down |
| ENSMUSG00000078348 | Sf3b5 | 1.68028 | <0.00001 | up |
| ENSMUSG00000078570 | 1110065P20Rik | 1.862709 | <0.00001 | up |
| ENSMUSG00000078650 | G6pc | -1.77293 | <0.00001 | down |
| ENSMUSG00000078687 | Mup8 | -1.47399 | <0.00001 | down |
| ENSMUSG00000078817 | Nlrp12 | 1.629612 | <0.00001 | up |
| ENSMUSG00000078866 | Zfp970 | -1.50402 | <0.00001 | down |
| ENSMUSG00000078931 | Pdf | 1.785303 | <0.00001 | up |
| ENSMUSG00000078964 | Ces1b | -1.52338 | <0.00001 | down |
| ENSMUSG00000079012 | Serpina3m | 2.685579 | <0.00001 | up |
| ENSMUSG00000079111 | Kdelr2 | 1.075764 | <0.00001 | up |
| ENSMUSG00000079263 | Gm6614 | -6.09865 | <0.00001 | down |
| ENSMUSG00000079363 | Gbp4 | -1.50753 | <0.00001 | down |
| ENSMUSG00000079494 | Nat8f5 | -1.7015 | <0.00001 | down |
| ENSMUSG00000079523 | Tmsb10 | -1.2805 | <0.00001 | down |
| ENSMUSG00000079555 | Haus3 | -1.50123 | <0.00001 | down |
| ENSMUSG00000080709 | Gm14324 | -1.95297 | <0.00001 | down |
| ENSMUSG00000080985 | Gm13559 | -4.28162 | <0.00001 | down |
| ENSMUSG00000081058 | H3c15 | 4.008088 | <0.00001 | up |
| ENSMUSG00000081207 | Gm13775 | -2.61395 | <0.00001 | down |
| ENSMUSG00000081406 | Rps6-ps4 | 1.911858 | <0.00001 | up |
| ENSMUSG00000081534 | Slc48a1 | 1.186295 | <0.00001 | up |
| ENSMUSG00000082127 | Gm13577 | -6.90631 | <0.00001 | down |
| ENSMUSG00000084983 | Gm11789 | 1.034416 | <0.00001 | up |
| ENSMUSG00000085786 | Gm15987 | -1.73959 | <0.00001 | down |
| ENSMUSG00000085995 | Gm2788 | 1.174328 | <0.00001 | up |
| ENSMUSG00000086253 | Gm13773 | -2.16216 | <0.00001 | down |
| ENSMUSG00000086583 | Gm15500 | 1.786501 | <0.00001 | up |
| ENSMUSG00000086877 | A230072C01Rik | -1.60717 | <0.00001 | down |
| ENSMUSG00000087412 | Gm15501 | 2.56897 | <0.00001 | up |
| ENSMUSG00000087470 | A630031M04Rik | -1.65006 | <0.00001 | down |
| ENSMUSG00000087516 | Tbx3os1 | -2.04868 | <0.00001 | down |
| ENSMUSG00000087579 | Hectd2os | -2.6763 | <0.00001 | down |
| ENSMUSG00000087684 | 1200007C13Rik | 5.962019 | <0.00001 | up |
| ENSMUSG00000089704 | Galnt2 | 1.015987 | <0.00001 | up |
| ENSMUSG00000090086 | AI480526 | -1.47754 | <0.00001 | down |
| ENSMUSG00000090171 | Ugt1a2 | 22.55819 | <0.00001 | up |
| ENSMUSG00000090622 | A930033H14Rik | -1.70803 | <0.00001 | down |
| ENSMUSG00000090877 | Hspa1b | 1.952962 | <0.00001 | up |
| ENSMUSG00000092021 | Gbp11 | -1.78101 | <0.00001 | down |
| ENSMUSG00000092395 | Gm20463 | -1.73319 | <0.00001 | down |
| ENSMUSG00000093485 | Gm20708 | -2.58235 | <0.00001 | down |
| ENSMUSG00000093916 | Gm379 | 2.394899 | <0.00001 | up |
| ENSMUSG00000094145 | Vmn2r20 | -3.3158 | <0.00001 | down |
| ENSMUSG00000094747 | Olfr1307 | -2.30589 | <0.00001 | down |
| ENSMUSG00000094786 | Gm14403 | -2.30348 | <0.00001 | down |
| ENSMUSG00000095061 | E030018B13Rik | 3.344599 | <0.00001 | up |
| ENSMUSG00000095123 | Gm21781 | -1.51346 | <0.00001 | down |
| ENSMUSG00000097124 | A530020G20Rik | -2.70574 | <0.00001 | down |
| ENSMUSG00000097148 | Gm3839 | -1.5314 | <0.00001 | down |
| ENSMUSG00000097392 | Thoc2l | -1.20144 | <0.00001 | down |
| ENSMUSG00000097893 | 1700034P13Rik | -2.07098 | <0.00001 | down |
| ENSMUSG00000099034 | 2810039B14Rik | -1.25712 | <0.00001 | down |
| ENSMUSG00000099707 | Gm8883 | 2.696399 | <0.00001 | up |
| ENSMUSG00000101599 | Gm20342 | -1.94561 | <0.00001 | down |
| ENSMUSG00000102591 | Gm38383 | -2.3148 | <0.00001 | down |
| ENSMUSG00000103421 | Golt1a | 2.461904 | <0.00001 | up |
| ENSMUSG00000105315 | Gm18635 | -2.28726 | <0.00001 | down |
| ENSMUSG00000106030 | Gm43611 | -2.59423 | <0.00001 | down |
| ENSMUSG00000106577 | Gm31026 | -2.94942 | <0.00001 | down |
| ENSMUSG00000106943 | Dancr | -1.39955 | <0.00001 | down |
| ENSMUSG00000106948 | Gm42785 | -1.91048 | <0.00001 | down |
| ENSMUSG00000107050 | C030017G13Rik | -3.08079 | <0.00001 | down |
| ENSMUSG00000107096 | Gm43597 | -2.03298 | <0.00001 | down |
| ENSMUSG00000107336 | Gm43461 | -3.28993 | <0.00001 | down |
| ENSMUSG00000107516 | Gm30784 | -1.36145 | <0.00001 | down |
| ENSMUSG00000107838 | Gm45769 | -1.28399 | <0.00001 | down |
| ENSMUSG00000109196 | Gm44715 | -1.99094 | <0.00001 | down |
| ENSMUSG00000109628 | BC024386 | -1.30913 | <0.00001 | down |
| ENSMUSG00000109764 | Klkb1 | -1.475 | <0.00001 | down |
| ENSMUSG00000110206 | Flt3l | -1.32565 | <0.00001 | down |
| ENSMUSG00000110234 | Gm45799 | 2.296074 | <0.00001 | up |
| ENSMUSG00000110755 | BC049987 | 2.494549 | <0.00001 | up |
| ENSMUSG00000112043 | Gm48774 | -1.7589 | <0.00001 | down |
| ENSMUSG00000112342 | Gm47031 | -1.90121 | <0.00001 | down |
| ENSMUSG00000112557 | Gm47626 | -1.63739 | <0.00001 | down |
| ENSMUSG00000112788 | Gm47821 | -2.35219 | <0.00001 | down |
| ENSMUSG00000112794 | Gm48878 | -1.62158 | <0.00001 | down |
| ENSMUSG00000113262 | Gm48551 | -4.90462 | <0.00001 | down |
| ENSMUSG00000113476 | Gm48309 | -1.90377 | <0.00001 | down |
| ENSMUSG00000113543 | Gm36264 | -2.84337 | <0.00001 | down |
| ENSMUSG00000113788 | Gm47904 | -4.37315 | <0.00001 | down |
| ENSMUSG00000113949 | Scamp4 | 1.02504 | <0.00001 | up |
| ENSMUSG00000115338 | Pnp | 1.368557 | <0.00001 | up |
| ENSMUSG00000115846 | Gm41144 | 5.599146 | <0.00001 | up |
| ENSMUSG00000115919 | Gm31583 | -1.21889 | <0.00001 | down |
| ENSMUSG00000116450 | Gm49534 | -2.01606 | <0.00001 | down |
| ENSMUSG00000116858 | Gm49797 | -1.53014 | <0.00001 | down |
| ENSMUSG00000116903 | Gm19522 | -2.45494 | <0.00001 | down |
| ENSMUSG00000117098 | Gm49909 | 1.798749 | <0.00001 | up |
| ENSMUSG00000117780 | Gm3734 | -2.03418 | <0.00001 | down |
| ENSMUSG00000117990 | Gm32027 | -2.2165 | <0.00001 | down |
| ENSMUSG00000118295 | Gm8437 | 4.836265 | <0.00001 | up |
| ENSMUSG00000118631 | Gm53019 | -1.47225 | <0.00001 | down |
| ENSMUSG00000120078 | - | 3.413606 | <0.00001 | up |
| ENSMUSG00000120294 | - | -2.55872 | <0.00001 | down |
| ENSMUSG00000120336 | - | -2.26594 | <0.00001 | down |
| ENSMUSG00000120390 | - | -1.22269 | <0.00001 | down |
| ENSMUSG00000120473 | - | 2.914148 | <0.00001 | up |
| ENSMUSG00000120850 | - | 4.04356 | <0.00001 | up |
| ENSMUSG00000120919 | - | -5.23547 | <0.00001 | down |
| ENSMUSG00000121242 | - | 3.480967 | <0.00001 | up |
| ENSMUSG00000121505 | Gm4956 | -3.90105 | <0.00001 | down |
