# Supplementary material for: Multi-time point transcriptomics and metabolomics reveal key transcription and metabolic features of hepatic ischemia-reperfusion injury in mice
Source: Genes Dis. 2024 Nov 17;12(2):101465. doi: 10.1016/j.gendis.2024.101465 (PMC11697123; doi:10.1016/j.gendis.2024.101465)
Supplement: Multimedia component 2 [file mmc2.docx]

**Table S1B.** Identified differentially expressed genes between Sham and I1R24 groups.

| **Gene ID** | **Gene name** | **Log2FC** | **P-value** | **Regulate** |
| --- | --- | --- | --- | --- |
| ENSMUSG00000033467 | Crlf2 | 1.137487 | 0.01345 | up |
| ENSMUSG00000042616 | Oscp1 | -1.88585 | 0.01344 | down |
| ENSMUSG00000085696 | Hoxaas3 | -2.52397 | 0.01344 | down |
| ENSMUSG00000028139 | Riiad1 | -4.59792 | 0.01344 | down |
| ENSMUSG00000004347 | Pde1c | -2.02447 | 0.01342 | down |
| ENSMUSG00000103625 | Gm37357 | -2.16319 | 0.01342 | down |
| ENSMUSG00000102746 | Gm37601 | -1.41604 | 0.01327 | down |
| ENSMUSG00000091228 | Gm20390 | 1.952934 | 0.01325 | up |
| ENSMUSG00000120150 | - | -3.89334 | 0.01324 | down |
| ENSMUSG00000032740 | Ccdc88a | -1.17121 | 0.01322 | down |
| ENSMUSG00000105347 | Gm43503 | -2.23231 | 0.01319 | down |
| ENSMUSG00000087484 | 2900089D17Rik | -2.13654 | 0.01315 | down |
| ENSMUSG00000047216 | Cdh19 | -3.43454 | 0.01311 | down |
| ENSMUSG00000120919 | - | -1.92439 | 0.01309 | down |
| ENSMUSG00000059366 | Olfr959 | -3.71893 | 0.01307 | down |
| ENSMUSG00000085936 | 2610307P16Rik | -2.6461 | 0.01307 | down |
| ENSMUSG00000026586 | Prrx1 | -1.96066 | 0.01289 | down |
| ENSMUSG00000119521 | n-R5s123 | -4.29523 | 0.01289 | down |
| ENSMUSG00000093668 | Pou5f2 | -3.23582 | 0.01288 | down |
| ENSMUSG00000021007 | Spata7 | -1.04135 | 0.01276 | down |
| ENSMUSG00000054598 | 9130230L23Rik | -1.59749 | 0.01275 | down |
| ENSMUSG00000087371 | Gm15541 | -2.00477 | 0.01270 | down |
| ENSMUSG00000052291 | 5330438D12Rik | -1.06742 | 0.01267 | down |
| ENSMUSG00000023191 | P3h3 | -1.10786 | 0.01261 | down |
| ENSMUSG00000086296 | D030055H07Rik | 2.460913 | 0.01261 | up |
| ENSMUSG00000101797 | Gm29266 | -2.18631 | 0.01260 | down |
| ENSMUSG00000079592 | C1qtnf5 | -1.20857 | 0.01254 | down |
| ENSMUSG00000043448 | Gjc2 | -1.8995 | 0.01248 | down |
| ENSMUSG00000111329 | A830035O19Rik | 2.67258 | 0.01246 | up |
| ENSMUSG00000079610 | Ankrd39 | 1.150487 | 0.01243 | up |
| ENSMUSG00000027550 | Lrrcc1 | -1.44544 | 0.01242 | down |
| ENSMUSG00000108129 | 4930417O13Rik | -2.51544 | 0.01241 | down |
| ENSMUSG00000061048 | Cdh3 | -1.62542 | 0.01238 | down |
| ENSMUSG00000021811 | Dnajc9 | 1.067437 | 0.01236 | up |
| ENSMUSG00000097536 | 2610037D02Rik | -1.62677 | 0.01233 | down |
| ENSMUSG00000079457 | Gm7609 | -1.564 | 0.01220 | down |
| ENSMUSG00000085078 | C030013C21Rik | -2.68688 | 0.01213 | down |
| ENSMUSG00000009092 | Derl3 | 1.875912 | 0.01213 | up |
| ENSMUSG00000117013 | Gm30531 | 3.442803 | 0.01211 | up |
| ENSMUSG00000084836 | Gm16274 | -4.26078 | 0.01208 | down |
| ENSMUSG00000120309 | - | -3.44658 | 0.01206 | down |
| ENSMUSG00000097493 | 9930014A18Rik | -2.41726 | 0.01204 | down |
| ENSMUSG00000035458 | Tnni3 | -2.58198 | 0.01203 | down |
| ENSMUSG00000002688 | Prkd1 | -3.16204 | 0.01193 | down |
| ENSMUSG00000029086 | Prom1 | -1.3991 | 0.01186 | down |
| ENSMUSG00000104213 | Ighd | -1.81725 | 0.01182 | down |
| ENSMUSG00000022651 | Retnlg | 2.399169 | 0.01181 | up |
| ENSMUSG00000062861 | Zfp28 | -1.08361 | 0.01181 | down |
| ENSMUSG00000039137 | Whrn | -2.00554 | 0.01180 | down |
| ENSMUSG00000081769 | Gm12216 | -1.13571 | 0.01177 | down |
| ENSMUSG00000020641 | Rsad2 | -1.45743 | 0.01176 | down |
| ENSMUSG00000044244 | Il20rb | -1.14241 | 0.01174 | down |
| ENSMUSG00000097316 | Gm10516 | 1.961057 | 0.01172 | up |
| ENSMUSG00000087404 | Gm11752 | 3.759241 | 0.01171 | up |
| ENSMUSG00000114875 | Gm30363 | -3.21339 | 0.01171 | down |
| ENSMUSG00000086481 | Gm11707 | -1.5991 | 0.01168 | down |
| ENSMUSG00000103656 | Gm37205 | -3.76623 | 0.01164 | down |
| ENSMUSG00000046782 | Ttc6 | -1.30956 | 0.01164 | down |
| ENSMUSG00000074218 | Cox7a1 | -1.16116 | 0.01164 | down |
| ENSMUSG00000112126 | Gm34544 | -4.67223 | 0.01163 | down |
| ENSMUSG00000052726 | Kcnt2 | 1.68899 | 0.01160 | up |
| ENSMUSG00000103207 | Gm9874 | -3.50585 | 0.01157 | down |
| ENSMUSG00000021716 | Srek1ip1 | -1.16951 | 0.01154 | down |
| ENSMUSG00000039742 | Garin1b | -4.68062 | 0.01147 | down |
| ENSMUSG00000025020 | Slit1 | -3.22051 | 0.01147 | down |
| ENSMUSG00000006398 | Cdc20 | -2.0336 | 0.01144 | down |
| ENSMUSG00000037784 | Dzip1l | -1.55594 | 0.01136 | down |
| ENSMUSG00000041012 | Cmtm8 | -1.30153 | 0.01134 | down |
| ENSMUSG00000120382 | - | -1.11876 | 0.01130 | down |
| ENSMUSG00000044702 | Palb2 | -1.93498 | 0.01128 | down |
| ENSMUSG00000121370 | - | -1.82547 | 0.01123 | down |
| ENSMUSG00000035486 | Plk5 | -1.44025 | 0.01122 | down |
| ENSMUSG00000030089 | Slc41a3 | 1.840202 | 0.01118 | up |
| ENSMUSG00000103839 | Gm37607 | -1.29574 | 0.01113 | down |
| ENSMUSG00000096967 | Gm26621 | -1.18636 | 0.01111 | down |
| ENSMUSG00000007646 | Rad51c | -1.31325 | 0.01109 | down |
| ENSMUSG00000031262 | Cenpi | -2.33307 | 0.01105 | down |
| ENSMUSG00000110750 | Gm48702 | 1.862221 | 0.01104 | up |
| ENSMUSG00000076937 | Iglc2 | -1.33924 | 0.01102 | down |
| ENSMUSG00000090081 | Gm16587 | -1.79267 | 0.01096 | down |
| ENSMUSG00000032649 | Colgalt2 | -2.12455 | 0.01094 | down |
| ENSMUSG00000097785 | B230217O12Rik | -1.10357 | 0.01092 | down |
| ENSMUSG00000042712 | Tceal9 | 1.015423 | 0.01089 | up |
| ENSMUSG00000089647 | Gm2245 | -2.82424 | 0.01086 | down |
| ENSMUSG00000090338 | Gm17081 | -1.01835 | 0.01079 | down |
| ENSMUSG00000055323 | Gm9967 | -2.06177 | 0.01077 | down |
| ENSMUSG00000084319 | Tpt1-ps3 | 1.411365 | 0.01072 | up |
| ENSMUSG00000048387 | Osr1 | -4.06627 | 0.01069 | down |
| ENSMUSG00000094588 | Olfr898 | -3.47889 | 0.01067 | down |
| ENSMUSG00000083246 | Gm11839 | -2.42823 | 0.01061 | down |
| ENSMUSG00000121255 | - | -3.40347 | 0.01061 | down |
| ENSMUSG00000117105 | Dreh | 1.598897 | 0.01059 | up |
| ENSMUSG00000111892 | Gm47641 | -3.15731 | 0.01059 | down |
| ENSMUSG00000097385 | Gm26814 | -2.15816 | 0.01057 | down |
| ENSMUSG00000103039 | Gm37123 | -1.27548 | 0.01050 | down |
| ENSMUSG00000044390 | Tigd3 | -2.03458 | 0.01046 | down |
| ENSMUSG00000006542 | Prkag3 | -3.78146 | 0.01045 | down |
| ENSMUSG00000038295 | Atg9b | 3.392893 | 0.01044 | up |
| ENSMUSG00000120414 | Gm32261 | -2.79181 | 0.01043 | down |
| ENSMUSG00000071064 | Zfp827 | -1.32284 | 0.01037 | down |
| ENSMUSG00000061577 | Adgrg5 | -2.35771 | 0.01037 | down |
| ENSMUSG00000114937 | Gm30054 | -2.0271 | 0.01037 | down |
| ENSMUSG00000092075 | Serpina4-ps1 | -1.29766 | 0.01034 | down |
| ENSMUSG00000040809 | Chil3 | 2.810118 | 0.01027 | up |
| ENSMUSG00000091945 | Vmn2r114 | -1.01692 | 0.01025 | down |
| ENSMUSG00000108446 | Gm44997 | -1.71762 | 0.01025 | down |
| ENSMUSG00000085091 | Egfros | -2.08849 | 0.01023 | down |
| ENSMUSG00000061111 | Mcrip1 | 1.27384 | 0.01018 | up |
| ENSMUSG00000027408 | Cpxm1 | -1.51415 | 0.01016 | down |
| ENSMUSG00000104793 | Gm43756 | -3.85987 | 0.01012 | down |
| ENSMUSG00000072109 | A530040E14Rik | -3.90581 | 0.01009 | down |
| ENSMUSG00000091019 | Gm7502 | -2.52267 | 0.01007 | down |
| ENSMUSG00000048583 | Igf2 | -1.15731 | 0.01004 | down |
| ENSMUSG00000030703 | Gdpd3 | -1.83357 | 0.01002 | down |
| ENSMUSG00000031965 | Tbx20 | -1.05011 | 0.01000 | down |
| ENSMUSG00000101578 | Vmn1r206 | -1.54495 | 0.00998 | down |
| ENSMUSG00000104121 | Gm37485 | -2.00929 | 0.00998 | down |
| ENSMUSG00000073768 | Olfr1330 | -2.1551 | 0.00994 | down |
| ENSMUSG00000040631 | Dok4 | -1.37722 | 0.00994 | down |
| ENSMUSG00000058579 | Cela2a | -1.94618 | 0.00993 | down |
| ENSMUSG00000121349 | Speer6-ps1 | -1.72725 | 0.00985 | down |
| ENSMUSG00000008136 | Fhl2 | 3.156332 | 0.00983 | up |
| ENSMUSG00000121485 | - | -1.16848 | 0.00973 | down |
| ENSMUSG00000045038 | Prkce | -1.1526 | 0.00967 | down |
| ENSMUSG00000032507 | Fbxl2 | -3.38565 | 0.00963 | down |
| ENSMUSG00000033342 | Plppr5 | -2.72672 | 0.00958 | down |
| ENSMUSG00000006313 | Upk1a | -4.13831 | 0.00956 | down |
| ENSMUSG00000078490 | Cfap74 | -2.49056 | 0.00955 | down |
| ENSMUSG00000025597 | Klhl4 | -1.86339 | 0.00952 | down |
| ENSMUSG00000102204 | Gm36958 | -1.04604 | 0.00949 | down |
| ENSMUSG00000107495 | Gm44215 | -1.19943 | 0.00948 | down |
| ENSMUSG00000085337 | Gm15964 | -1.41927 | 0.00943 | down |
| ENSMUSG00000014158 | Trpv4 | -1.47241 | 0.00942 | down |
| ENSMUSG00000053553 | 3110082I17Rik | 1.526365 | 0.00939 | up |
| ENSMUSG00000087662 | Gm11373 | -1.29499 | 0.00936 | down |
| ENSMUSG00000107205 | Gm42576 | -4.32994 | 0.00936 | down |
| ENSMUSG00000075289 | Carns1 | -1.23114 | 0.00935 | down |
| ENSMUSG00000021217 | Tshz3 | -1.49226 | 0.00932 | down |
| ENSMUSG00000090173 | Fbxw10 | -4.7507 | 0.00931 | down |
| ENSMUSG00000023046 | Igfbp6 | -1.81696 | 0.00927 | down |
| ENSMUSG00000009108 | Gnat2 | 1.376763 | 0.00926 | up |
| ENSMUSG00000056174 | Col8a2 | -3.10189 | 0.00924 | down |
| ENSMUSG00000019467 | Arhgef25 | -1.17511 | 0.00922 | down |
| ENSMUSG00000110057 | Gm2225 | -4.41896 | 0.00920 | down |
| ENSMUSG00000000247 | Lhx2 | -1.02555 | 0.00920 | down |
| ENSMUSG00000034570 | Inpp5j | -4.32333 | 0.00918 | down |
| ENSMUSG00000112615 | Gm18337 | 2.456022 | 0.00912 | up |
| ENSMUSG00000044453 | Ffar1 | -1.74133 | 0.00908 | down |
| ENSMUSG00000096950 | Gm9530 | -2.38087 | 0.00908 | down |
| ENSMUSG00000019863 | Qrsl1 | 1.025735 | 0.00907 | up |
| ENSMUSG00000097615 | Gm2061 | -1.46992 | 0.00904 | down |
| ENSMUSG00000051998 | Lax1 | -1.37828 | 0.00902 | down |
| ENSMUSG00000048612 | Myof | -1.03078 | 0.00902 | down |
| ENSMUSG00000069763 | Tmem100 | -2.13059 | 0.00900 | down |
| ENSMUSG00000112652 | 4921516A02Rik | 1.089956 | 0.00899 | up |
| ENSMUSG00000024742 | Fen1 | 2.091029 | 0.00896 | up |
| ENSMUSG00000019986 | Ahi1 | -1.61518 | 0.00895 | down |
| ENSMUSG00000102918 | Pcdhgc3 | -1.14459 | 0.00891 | down |
| ENSMUSG00000120342 | - | -2.08215 | 0.00888 | down |
| ENSMUSG00000081143 | Gm15823 | -2.16406 | 0.00886 | down |
| ENSMUSG00000027706 | Sec62 | -1.19988 | 0.00884 | down |
| ENSMUSG00000102282 | A930032L01Rik | -2.35285 | 0.00883 | down |
| ENSMUSG00000085990 | Gm16731 | -1.45079 | 0.00882 | down |
| ENSMUSG00000107331 | Gm42732 | -1.16543 | 0.00868 | down |
| ENSMUSG00000040339 | Fam102b | -1.07807 | 0.00860 | down |
| ENSMUSG00000027217 | Tspan18 | -1.29664 | 0.00859 | down |
| ENSMUSG00000025650 | Col7a1 | -2.11945 | 0.00858 | down |
| ENSMUSG00000025262 | Fam120c | 2.666082 | 0.00858 | up |
| ENSMUSG00000097910 | Hdnr | -1.87799 | 0.00857 | down |
| ENSMUSG00000028871 | Rspo1 | -2.69125 | 0.00857 | down |
| ENSMUSG00000072571 | Tmem253 | -2.9255 | 0.00856 | down |
| ENSMUSG00000116796 | Gm49784 | -4.07559 | 0.00850 | down |
| ENSMUSG00000017740 | Slc12a5 | -1.15658 | 0.00849 | down |
| ENSMUSG00000039153 | Runx2 | -1.73338 | 0.00847 | down |
| ENSMUSG00000028634 | Hivep3 | -1.25213 | 0.00845 | down |
| ENSMUSG00000021108 | Prkch | -1.1612 | 0.00843 | down |
| ENSMUSG00000023912 | Slc25a27 | -1.1034 | 0.00840 | down |
| ENSMUSG00000047155 | Cyp4x1 | -1.75694 | 0.00839 | down |
| ENSMUSG00000091119 | Ccdc152 | -2.131 | 0.00838 | down |
| ENSMUSG00000092008 | Cyp2c69 | -1.7028 | 0.00835 | down |
| ENSMUSG00000039617 | Gm7488 | 4.577562 | 0.00835 | up |
| ENSMUSG00000056055 | Sag | -2.64874 | 0.00834 | down |
| ENSMUSG00000027570 | Col9a3 | -2.53403 | 0.00828 | down |
| ENSMUSG00000058174 | Gm5148 | 2.767498 | 0.00827 | up |
| ENSMUSG00000097416 | Gm26670 | -2.18582 | 0.00826 | down |
| ENSMUSG00000066456 | Hmgn3 | -1.40553 | 0.00826 | down |
| ENSMUSG00000004018 | Fancl | 1.160173 | 0.00826 | up |
| ENSMUSG00000091805 | Vmn2r108 | -1.28069 | 0.00825 | down |
| ENSMUSG00000097974 | Gm10605 | -1.55724 | 0.00821 | down |
| ENSMUSG00000025092 | Hspa12a | -1.08822 | 0.00820 | down |
| ENSMUSG00000108953 | Gm45129 | -2.31393 | 0.00819 | down |
| ENSMUSG00000112990 | Gm47372 | -1.9614 | 0.00811 | down |
| ENSMUSG00000022793 | B4galt4 | -1.12554 | 0.00810 | down |
| ENSMUSG00000116525 | Gm30371 | -2.48166 | 0.00810 | down |
| ENSMUSG00000082894 | Gm6480 | -1.58153 | 0.00808 | down |
| ENSMUSG00000118049 | Gm50103 | 1.087916 | 0.00807 | up |
| ENSMUSG00000121408 | - | 2.830748 | 0.00805 | up |
| ENSMUSG00000018169 | Mfng | -1.46593 | 0.00804 | down |
| ENSMUSG00000120807 | - | -4.77457 | 0.00802 | down |
| ENSMUSG00000025001 | Hells | -1.67245 | 0.00795 | down |
| ENSMUSG00000025185 | Loxl4 | -1.77984 | 0.00793 | down |
| ENSMUSG00000071661 | Zbtb3 | -2.39393 | 0.00791 | down |
| ENSMUSG00000022440 | C1qtnf6 | -1.47033 | 0.00787 | down |
| ENSMUSG00000026556 | Vangl2 | -1.27704 | 0.00787 | down |
| ENSMUSG00000075271 | Ttc30a1 | 2.225203 | 0.00784 | up |
| ENSMUSG00000108866 | Gm35082 | -2.99634 | 0.00782 | down |
| ENSMUSG00000055022 | Cntn1 | -2.11508 | 0.00778 | down |
| ENSMUSG00000061829 | Vmn1r214 | -3.04867 | 0.00777 | down |
| ENSMUSG00000009185 | Ccl8 | -3.85384 | 0.00776 | down |
| ENSMUSG00000059060 | Rad51b | 1.530658 | 0.00775 | up |
| ENSMUSG00000028212 | Ccne2 | -1.4563 | 0.00774 | down |
| ENSMUSG00000022613 | Miox | 3.020624 | 0.00773 | up |
| ENSMUSG00000074912 | Gm14207 | -2.22456 | 0.00770 | down |
| ENSMUSG00000032549 | Rab6b | -1.25881 | 0.00769 | down |
| ENSMUSG00000115329 | Gm49272 | -1.45515 | 0.00769 | down |
| ENSMUSG00000087475 | 4933406I18Rik | -2.1142 | 0.00765 | down |
| ENSMUSG00000024268 | Celf4 | -1.67026 | 0.00764 | down |
| ENSMUSG00000044309 | Apol7c | -2.61063 | 0.00764 | down |
| ENSMUSG00000069072 | Slc7a14 | -3.51254 | 0.00762 | down |
| ENSMUSG00000105601 | Gm42725 | -4.14482 | 0.00758 | down |
| ENSMUSG00000118040 | Gm36602 | -4.40698 | 0.00757 | down |
| ENSMUSG00000121319 | - | 1.692812 | 0.00754 | up |
| ENSMUSG00000121210 | - | -3.67395 | 0.00753 | down |
| ENSMUSG00000071604 | Fam189a2 | -2.08562 | 0.00752 | down |
| ENSMUSG00000027716 | Trpc3 | -1.98222 | 0.00752 | down |
| ENSMUSG00000051008 | 4930412M03Rik | 2.60138 | 0.00751 | up |
| ENSMUSG00000028278 | Rragd | -1.52103 | 0.00750 | down |
| ENSMUSG00000078674 | Mup18 | 1.414862 | 0.00749 | up |
| ENSMUSG00000031349 | Nsdhl | 1.195099 | 0.00749 | up |
| ENSMUSG00000036295 | Lrrn3 | -2.1689 | 0.00747 | down |
| ENSMUSG00000118491 | Gm44505 | -3.77624 | 0.00747 | down |
| ENSMUSG00000030878 | Cdr2 | -2.13295 | 0.00746 | down |
| ENSMUSG00000014030 | Pax5 | -1.46834 | 0.00743 | down |
| ENSMUSG00000040751 | Lat2 | -2.44961 | 0.00743 | down |
| ENSMUSG00000102009 | 4933400F21Rik | -2.60759 | 0.00742 | down |
| ENSMUSG00000069305 | H4c18 | 2.522968 | 0.00738 | up |
| ENSMUSG00000114771 | Gm49064 | -4.2011 | 0.00737 | down |
| ENSMUSG00000043943 | Naalad2 | -1.69492 | 0.00736 | down |
| ENSMUSG00000041482 | Piezo2 | -1.24093 | 0.00735 | down |
| ENSMUSG00000041468 | Gpr12 | -1.49668 | 0.00732 | down |
| ENSMUSG00000024136 | Dnase1l2 | -1.42067 | 0.00730 | down |
| ENSMUSG00000087575 | Gm12976 | -1.88648 | 0.00724 | down |
| ENSMUSG00000034303 | Ccdc15 | -2.37934 | 0.00722 | down |
| ENSMUSG00000023021 | Cers5 | -1.06033 | 0.00721 | down |
| ENSMUSG00000055027 | Smyd1 | 1.255889 | 0.00720 | up |
| ENSMUSG00000038280 | Ostm1 | 1.353369 | 0.00719 | up |
| ENSMUSG00000106992 | Gm43167 | -1.71804 | 0.00713 | down |
| ENSMUSG00000070883 | Ccdc173 | -2.79631 | 0.00712 | down |
| ENSMUSG00000037363 | Letm2 | -1.01907 | 0.00710 | down |
| ENSMUSG00000025889 | Snca | -1.77153 | 0.00702 | down |
| ENSMUSG00000090619 | Vmn2r60 | -4.28893 | 0.00701 | down |
| ENSMUSG00000105471 | A430073D23Rik | -2.6724 | 0.00701 | down |
| ENSMUSG00000000120 | Ngfr | -1.36126 | 0.00696 | down |
| ENSMUSG00000119670 | Gm24305 | -4.05276 | 0.00692 | down |
| ENSMUSG00000027577 | Chrna4 | -3.32032 | 0.00691 | down |
| ENSMUSG00000090222 | Ifi203-ps | -1.57308 | 0.00687 | down |
| ENSMUSG00000025008 | Tctn3 | -1.25968 | 0.00687 | down |
| ENSMUSG00000121309 | - | -1.85916 | 0.00686 | down |
| ENSMUSG00000087579 | Hectd2os | -1.31718 | 0.00684 | down |
| ENSMUSG00000038304 | Cd160 | -1.80462 | 0.00684 | down |
| ENSMUSG00000039209 | Rpl39l | -4.21516 | 0.00679 | down |
| ENSMUSG00000108059 | Gm44369 | -3.32218 | 0.00676 | down |
| ENSMUSG00000031740 | Mmp2 | -1.44311 | 0.00676 | down |
| ENSMUSG00000039629 | Strip2 | -3.19856 | 0.00674 | down |
| ENSMUSG00000087050 | Dhrs13os | -3.01419 | 0.00674 | down |
| ENSMUSG00000100017 | 2410022M11Rik | 1.525262 | 0.00671 | up |
| ENSMUSG00000014776 | Nol3 | 1.590162 | 0.00670 | up |
| ENSMUSG00000050370 | Ch25h | -4.03054 | 0.00669 | down |
| ENSMUSG00000090994 | Gm6153 | -2.28649 | 0.00666 | down |
| ENSMUSG00000041809 | Efhc1 | -3.27965 | 0.00663 | down |
| ENSMUSG00000049173 | Myoz3 | -2.20683 | 0.00658 | down |
| ENSMUSG00000034634 | Ly6d | 1.823445 | 0.00656 | up |
| ENSMUSG00000023966 | Rsph9 | -1.60651 | 0.00655 | down |
| ENSMUSG00000107876 | Gm43936 | -3.45288 | 0.00653 | down |
| ENSMUSG00000027499 | Pkia | -1.97056 | 0.00648 | down |
| ENSMUSG00000022856 | Tmem41a | 1.279335 | 0.00643 | up |
| ENSMUSG00000097787 | 2700046G09Rik | 1.378055 | 0.00639 | up |
| ENSMUSG00000121227 | - | 1.413268 | 0.00635 | up |
| ENSMUSG00000039304 | Tnfsf10 | -1.01601 | 0.00634 | down |
| ENSMUSG00000052407 | Ccdc171 | -1.01966 | 0.00630 | down |
| ENSMUSG00000093445 | Lrch4 | 1.302754 | 0.00628 | up |
| ENSMUSG00000022197 | Pdzd2 | -1.49468 | 0.00626 | down |
| ENSMUSG00000038146 | Notch3 | -1.06284 | 0.00621 | down |
| ENSMUSG00000120258 | - | -2.45043 | 0.00620 | down |
| ENSMUSG00000057914 | Cacnb2 | -4.80935 | 0.00613 | down |
| ENSMUSG00000103436 | Gm36995 | -1.51195 | 0.00611 | down |
| ENSMUSG00000109279 | Gm45220 | -4.96673 | 0.00609 | down |
| ENSMUSG00000031659 | Adcy7 | -1.14246 | 0.00609 | down |
| ENSMUSG00000044551 | 9930012K11Rik | -2.36782 | 0.00608 | down |
| ENSMUSG00000033949 | Trim36 | -3.01209 | 0.00607 | down |
| ENSMUSG00000107529 | Gm44291 | -1.76203 | 0.00607 | down |
| ENSMUSG00000109297 | Gm31522 | -2.03624 | 0.00600 | down |
| ENSMUSG00000049907 | Rasl11b | -1.13159 | 0.00598 | down |
| ENSMUSG00000027478 | Dnmt3b | -1.10233 | 0.00591 | down |
| ENSMUSG00000000416 | Cttnbp2 | -1.97021 | 0.00590 | down |
| ENSMUSG00000021010 | Npas3 | -3.05622 | 0.00588 | down |
| ENSMUSG00000084923 | Gm15611 | 3.98424 | 0.00587 | up |
| ENSMUSG00000037406 | Htra4 | -2.55198 | 0.00585 | down |
| ENSMUSG00000029122 | Evc | -1.01942 | 0.00585 | down |
| ENSMUSG00000030724 | Cd19 | -1.80219 | 0.00585 | down |
| ENSMUSG00000103273 | Gm37913 | -1.69468 | 0.00579 | down |
| ENSMUSG00000061186 | Sfmbt2 | -5.05036 | 0.00577 | down |
| ENSMUSG00000109656 | Gm45548 | -2.64474 | 0.00576 | down |
| ENSMUSG00000039994 | Timeless | -1.10386 | 0.00576 | down |
| ENSMUSG00000060467 | Gm10080 | -2.71073 | 0.00574 | down |
| ENSMUSG00000055320 | Tead1 | -1.01637 | 0.00574 | down |
| ENSMUSG00000118796 | n-R5s138 | -4.69611 | 0.00573 | down |
| ENSMUSG00000113847 | Gm48799 | -1.44606 | 0.00570 | down |
| ENSMUSG00000120841 | - | 1.897927 | 0.00569 | up |
| ENSMUSG00000038252 | Ncapd2 | -1.10531 | 0.00569 | down |
| ENSMUSG00000097365 | C030034L19Rik | -4.9588 | 0.00569 | down |
| ENSMUSG00000104324 | Gm37320 | -2.09409 | 0.00567 | down |
| ENSMUSG00000070458 | Vmn2r73 | -1.86797 | 0.00565 | down |
| ENSMUSG00000050623 | Catsperz | -4.22827 | 0.00562 | down |
| ENSMUSG00000114430 | Gm40264 | -1.15992 | 0.00561 | down |
| ENSMUSG00000097222 | Gata6os | -1.28572 | 0.00557 | down |
| ENSMUSG00000072612 | Gm10382 | 2.576736 | 0.00556 | up |
| ENSMUSG00000102647 | Gm38024 | -1.9328 | 0.00549 | down |
| ENSMUSG00000031561 | Tenm3 | 1.177418 | 0.00546 | up |
| ENSMUSG00000070354 | Evi2 | -2.01355 | 0.00546 | down |
| ENSMUSG00000037337 | Map4k1 | -1.67517 | 0.00545 | down |
| ENSMUSG00000090171 | Ugt1a2 | 8.01117 | 0.00542 | up |
| ENSMUSG00000036815 | Dpp10 | -1.5087 | 0.00539 | down |
| ENSMUSG00000019990 | Pde7b | -1.70615 | 0.00537 | down |
| ENSMUSG00000043251 | Exoc3l | -1.18632 | 0.00534 | down |
| ENSMUSG00000109357 | Gm45236 | -2.68395 | 0.00532 | down |
| ENSMUSG00000111403 | Gm47544 | -3.97848 | 0.00531 | down |
| ENSMUSG00000010044 | Zmynd10 | -2.09658 | 0.00529 | down |
| ENSMUSG00000030022 | Adamts9 | -1.14281 | 0.00527 | down |
| ENSMUSG00000037085 | Trmt12 | 1.139522 | 0.00527 | up |
| ENSMUSG00000090623 | Cfhr3 | 2.712932 | 0.00523 | up |
| ENSMUSG00000108394 | Gm45477 | -2.48712 | 0.00522 | down |
| ENSMUSG00000102712 | Gm37758 | -1.4191 | 0.00518 | down |
| ENSMUSG00000021414 | Fam217a | -3.42307 | 0.00514 | down |
| ENSMUSG00000037020 | Wdr62 | -1.33696 | 0.00512 | down |
| ENSMUSG00000108027 | Gm44271 | -3.9354 | 0.00511 | down |
| ENSMUSG00000040084 | Bub1b | -2.34838 | 0.00509 | down |
| ENSMUSG00000035852 | Misp | -2.65886 | 0.00508 | down |
| ENSMUSG00000055254 | Ntrk2 | -1.89646 | 0.00507 | down |
| ENSMUSG00000106743 | Gm42847 | -1.86237 | 0.00506 | down |
| ENSMUSG00000031283 | Chrdl1 | -2.31724 | 0.00506 | down |
| ENSMUSG00000112433 | Gm30122 | -1.28887 | 0.00505 | down |
| ENSMUSG00000019278 | Dpep1 | -1.52655 | 0.00503 | down |
| ENSMUSG00000052632 | Asap2 | -1.03704 | 0.00502 | down |
| ENSMUSG00000114267 | Gm48600 | -1.62673 | 0.00500 | down |
| ENSMUSG00000087179 | 5730471H19Rik | -1.50586 | 0.00499 | down |
| ENSMUSG00000039521 | Foxp3 | -3.35011 | 0.00494 | down |
| ENSMUSG00000033082 | Clec1a | -1.56976 | 0.00492 | down |
| ENSMUSG00000121201 | - | -2.16837 | 0.00490 | down |
| ENSMUSG00000032355 | Mlip | -2.65029 | 0.00488 | down |
| ENSMUSG00000085702 | Mecomos | -3.72143 | 0.00487 | down |
| ENSMUSG00000120616 | - | -3.58653 | 0.00487 | down |
| ENSMUSG00000046210 | Olfr735 | -1.25951 | 0.00485 | down |
| ENSMUSG00000028532 | Cachd1 | -1.62272 | 0.00484 | down |
| ENSMUSG00000026083 | Eif5b | -1.20419 | 0.00484 | down |
| ENSMUSG00000079173 | Zan | -1.59456 | 0.00483 | down |
| ENSMUSG00002075453 | Snord3b3 | -3.30332 | 0.00483 | down |
| ENSMUSG00000045284 | Dcaf12l1 | 1.361433 | 0.00481 | up |
| ENSMUSG00000112855 | Gm47842 | -1.47368 | 0.00480 | down |
| ENSMUSG00000062093 | Gm10110 | -1.26859 | 0.00478 | down |
| ENSMUSG00000078122 | F630028O10Rik | -1.63375 | 0.00478 | down |
| ENSMUSG00000119132 | Gm24407 | -4.27791 | 0.00478 | down |
| ENSMUSG00000035818 | Plekhs1 | -2.11567 | 0.00478 | down |
| ENSMUSG00000091227 | Gm3755 | -3.19395 | 0.00475 | down |
| ENSMUSG00000039765 | Cc2d2a | -1.02809 | 0.00475 | down |
| ENSMUSG00000071847 | Apcdd1 | -1.54509 | 0.00474 | down |
| ENSMUSG00000111375 | Btbd8 | -1.27723 | 0.00473 | down |
| ENSMUSG00000110588 | Gm45774 | 3.068328 | 0.00472 | up |
| ENSMUSG00000111656 | Gm47232 | -2.86996 | 0.00470 | down |
| ENSMUSG00000066804 | Vmn1r83 | -2.99377 | 0.00470 | down |
| ENSMUSG00000022335 | Zfat | -1.14993 | 0.00468 | down |
| ENSMUSG00000085433 | Gm16001 | -1.0358 | 0.00468 | down |
| ENSMUSG00000083718 | Ccnb2-ps | -1.73329 | 0.00467 | down |
| ENSMUSG00000057363 | Uxs1 | 1.107718 | 0.00467 | up |
| ENSMUSG00000110047 | A230085B16Rik | -2.34054 | 0.00462 | down |
| ENSMUSG00000017830 | Dhx58 | -1.03185 | 0.00459 | down |
| ENSMUSG00000104606 | Gm43409 | -1.68965 | 0.00457 | down |
| ENSMUSG00000020638 | Cmpk2 | -1.20711 | 0.00454 | down |
| ENSMUSG00000054414 | Slc30a7 | 1.080539 | 0.00453 | up |
| ENSMUSG00000016624 | Phf21b | -1.93862 | 0.00452 | down |
| ENSMUSG00000104660 | Gm43601 | -1.9608 | 0.00452 | down |
| ENSMUSG00000049928 | Glp2r | -3.15434 | 0.00451 | down |
| ENSMUSG00000075592 | Nynrin | -1.51704 | 0.00451 | down |
| ENSMUSG00000120121 | - | -2.58526 | 0.00444 | down |
| ENSMUSG00000102374 | Gm38387 | -3.06407 | 0.00442 | down |
| ENSMUSG00000058470 | Gm8369 | -3.44033 | 0.00436 | down |
| ENSMUSG00000107726 | Gm44037 | -3.01796 | 0.00435 | down |
| ENSMUSG00000043822 | Adamtsl5 | -1.41716 | 0.00434 | down |
| ENSMUSG00000028427 | Aqp7 | -2.07989 | 0.00432 | down |
| ENSMUSG00000108521 | Gm44639 | -1.74871 | 0.00431 | down |
| ENSMUSG00000069805 | Fbp1 | 1.246442 | 0.00430 | up |
| ENSMUSG00000115027 | Vmn1r81 | -1.1862 | 0.00426 | down |
| ENSMUSG00000039959 | Hip1 | -1.01423 | 0.00422 | down |
| ENSMUSG00000040740 | Slc25a34 | 1.003673 | 0.00421 | up |
| ENSMUSG00000117123 | Gm49890 | -1.43199 | 0.00419 | down |
| ENSMUSG00000035165 | Kcne3 | -1.1637 | 0.00407 | down |
| ENSMUSG00000083364 | Llph-ps2 | 1.58155 | 0.00402 | up |
| ENSMUSG00000053310 | Nrgn | -1.32196 | 0.00401 | down |
| ENSMUSG00000037851 | Iars | 1.060718 | 0.00400 | up |
| ENSMUSG00000038534 | Osbpl7 | -1.1731 | 0.00400 | down |
| ENSMUSG00000043263 | Ifi209 | -1.3445 | 0.00398 | down |
| ENSMUSG00000055072 | Gm9964 | -4.12879 | 0.00398 | down |
| ENSMUSG00000037617 | Spag1 | -1.14919 | 0.00395 | down |
| ENSMUSG00000121382 | - | -1.31738 | 0.00394 | down |
| ENSMUSG00000113200 | Gm48632 | -1.42404 | 0.00392 | down |
| ENSMUSG00000083929 | Gm10600 | -5.00411 | 0.00391 | down |
| ENSMUSG00000107785 | Gm45083 | 1.46679 | 0.00389 | up |
| ENSMUSG00000049871 | Nlrc3 | -1.68094 | 0.00389 | down |
| ENSMUSG00000013419 | Zfp651 | -1.15068 | 0.00388 | down |
| ENSMUSG00000115186 | Gm49417 | -1.13038 | 0.00388 | down |
| ENSMUSG00000037499 | Nenf | 1.019401 | 0.00387 | up |
| ENSMUSG00000028717 | Tal1 | -1.15687 | 0.00387 | down |
| ENSMUSG00000032561 | Acpp | 2.006381 | 0.00385 | up |
| ENSMUSG00000046532 | Ar | -1.22799 | 0.00384 | down |
| ENSMUSG00000076498 | Trbc2 | -1.53212 | 0.00382 | down |
| ENSMUSG00000078722 | Gm12394 | -5.05128 | 0.00380 | down |
| ENSMUSG00000034121 | Mks1 | -1.25563 | 0.00380 | down |
| ENSMUSG00000104548 | Gm43857 | -1.67661 | 0.00380 | down |
| ENSMUSG00000103132 | Gm37978 | -4.55777 | 0.00379 | down |
| ENSMUSG00000120918 | - | -1.51722 | 0.00379 | down |
| ENSMUSG00000022758 | P2rx6 | -3.26266 | 0.00378 | down |
| ENSMUSG00000105832 | Gm43841 | -2.18813 | 0.00378 | down |
| ENSMUSG00000028555 | Ttc39a | 2.348113 | 0.00378 | up |
| ENSMUSG00000000486 | Septin1 | -1.20581 | 0.00378 | down |
| ENSMUSG00000036687 | Tmem184a | 1.092958 | 0.00377 | up |
| ENSMUSG00000037979 | Ccdc92 | -2.61601 | 0.00377 | down |
| ENSMUSG00000029206 | Nsun7 | -2.92705 | 0.00376 | down |
| ENSMUSG00000120944 | - | -3.88901 | 0.00374 | down |
| ENSMUSG00000078486 | Perm1 | -2.79376 | 0.00374 | down |
| ENSMUSG00000052188 | Gm14964 | -2.02505 | 0.00372 | down |
| ENSMUSG00000083111 | Gm14421 | 1.061906 | 0.00369 | up |
| ENSMUSG00000028289 | Epha7 | -1.57729 | 0.00367 | down |
| ENSMUSG00000085972 | 1110028F11Rik | 1.2207 | 0.00367 | up |
| ENSMUSG00000024232 | Bambi | -1.17161 | 0.00365 | down |
| ENSMUSG00000108199 | Gm44249 | -2.40718 | 0.00365 | down |
| ENSMUSG00000078881 | Gm14434 | 2.741803 | 0.00362 | up |
| ENSMUSG00000111847 | Gm8899 | -3.72694 | 0.00361 | down |
| ENSMUSG00000120250 | - | -1.12008 | 0.00361 | down |
| ENSMUSG00000001943 | Vsig2 | -1.83435 | 0.00355 | down |
| ENSMUSG00000049280 | Olfr509 | -1.1451 | 0.00354 | down |
| ENSMUSG00000030553 | Pgpep1l | 3.570149 | 0.00354 | up |
| ENSMUSG00000000276 | Dgke | -1.0216 | 0.00351 | down |
| ENSMUSG00000031637 | Lrp2bp | -1.19384 | 0.00349 | down |
| ENSMUSG00000015843 | Rxrg | 1.061695 | 0.00347 | up |
| ENSMUSG00000109536 | 9330162G02Rik | -1.3119 | 0.00346 | down |
| ENSMUSG00000084283 | Gm14914 | -4.18121 | 0.00346 | down |
| ENSMUSG00000117079 | Gm41611 | -1.80198 | 0.00344 | down |
| ENSMUSG00000025648 | Pfkfb4 | -1.39896 | 0.00344 | down |
| ENSMUSG00000025172 | Ankrd2 | 1.916984 | 0.00344 | up |
| ENSMUSG00000103070 | Gm37903 | -1.42667 | 0.00343 | down |
| ENSMUSG00000037361 | Sf3b6 | 1.265052 | 0.00342 | up |
| ENSMUSG00000073460 | Pnldc1 | -1.68101 | 0.00342 | down |
| ENSMUSG00000056665 | Them6 | 1.047584 | 0.00339 | up |
| ENSMUSG00000045349 | Sh2d5 | -2.97429 | 0.00337 | down |
| ENSMUSG00000121162 | - | -3.15034 | 0.00337 | down |
| ENSMUSG00000042894 | Olfr1260 | -1.18154 | 0.00336 | down |
| ENSMUSG00000100937 | Nscme3l | -5.08722 | 0.00335 | down |
| ENSMUSG00000034774 | Dsg1c | 1.106646 | 0.00335 | up |
| ENSMUSG00000050921 | P2ry10 | -2.98562 | 0.00333 | down |
| ENSMUSG00000063590 | Slc22a28 | -1.08379 | 0.00327 | down |
| ENSMUSG00000032006 | Pdgfd | -1.52828 | 0.00326 | down |
| ENSMUSG00000033502 | Cdc14a | -1.26507 | 0.00325 | down |
| ENSMUSG00000031425 | Plp1 | -1.11956 | 0.00323 | down |
| ENSMUSG00000097617 | Gm10687 | -1.3053 | 0.00322 | down |
| ENSMUSG00000037139 | Myom3 | -3.03784 | 0.00322 | down |
| ENSMUSG00000025887 | Casp12 | -1.25089 | 0.00322 | down |
| ENSMUSG00000109368 | Gm45015 | -3.50587 | 0.00318 | down |
| ENSMUSG00000044461 | Shisa2 | -3.14689 | 0.00318 | down |
| ENSMUSG00000041120 | Nbl1 | -2.66195 | 0.00316 | down |
| ENSMUSG00000114226 | Gm48412 | -2.49603 | 0.00316 | down |
| ENSMUSG00000108511 | Gm44987 | -3.18523 | 0.00314 | down |
| ENSMUSG00000035067 | Xkr6 | -1.42388 | 0.00313 | down |
| ENSMUSG00000072949 | Acot1 | -1.44293 | 0.00313 | down |
| ENSMUSG00000031429 | Psmd10 | 1.044218 | 0.00312 | up |
| ENSMUSG00000024501 | Dpysl3 | -1.11154 | 0.00308 | down |
| ENSMUSG00000112576 | Gm47621 | -1.61221 | 0.00307 | down |
| ENSMUSG00000037772 | Mrpl23 | 1.971699 | 0.00306 | up |
| ENSMUSG00000082938 | Gm2810 | -3.64758 | 0.00306 | down |
| ENSMUSG00000034898 | Filip1 | -2.03145 | 0.00302 | down |
| ENSMUSG00000032717 | Mdfi | -1.94883 | 0.00300 | down |
| ENSMUSG00000117573 | Gm41668 | -4.03428 | 0.00299 | down |
| ENSMUSG00000033544 | Angptl1 | -1.84072 | 0.00298 | down |
| ENSMUSG00000062661 | Ncs1 | -2.56868 | 0.00297 | down |
| ENSMUSG00000045680 | Tcf21 | -1.01174 | 0.00296 | down |
| ENSMUSG00000084031 | Gm7416 | -2.86241 | 0.00295 | down |
| ENSMUSG00000030772 | Dkk3 | -1.3591 | 0.00293 | down |
| ENSMUSG00000114096 | Gm48641 | -3.34951 | 0.00292 | down |
| ENSMUSG00000060416 | Gm839 | 3.610585 | 0.00291 | up |
| ENSMUSG00000025083 | Afap1l2 | -1.56274 | 0.00291 | down |
| ENSMUSG00000001281 | Itgb7 | -1.17093 | 0.00291 | down |
| ENSMUSG00000099632 | 2900093K20Rik | -1.25115 | 0.00288 | down |
| ENSMUSG00000113095 | Gm7969 | -7.76004 | 0.00288 | down |
| ENSMUSG00000094488 | Olfr393 | -1.78911 | 0.00287 | down |
| ENSMUSG00000018752 | Tnfsfm13 | 2.078611 | 0.00286 | up |
| ENSMUSG00000109787 | Gm45286 | -1.8211 | 0.00286 | down |
| ENSMUSG00000107549 | Gm43961 | -4.09805 | 0.00282 | down |
| ENSMUSG00000095687 | Rnaset2a | 1.503782 | 0.00282 | up |
| ENSMUSG00000073771 | Btbd19 | -1.42497 | 0.00282 | down |
| ENSMUSG00000022658 | Tagln3 | -2.18663 | 0.00282 | down |
| ENSMUSG00000021951 | Eef1akmt1 | 1.69032 | 0.00281 | up |
| ENSMUSG00000021815 | Mss51 | -1.32441 | 0.00279 | down |
| ENSMUSG00000021553 | Slc28a3 | -4.31143 | 0.00277 | down |
| ENSMUSG00000056718 | Gm13199 | -2.37727 | 0.00277 | down |
| ENSMUSG00000078919 | Dpm1 | 1.131627 | 0.00274 | up |
| ENSMUSG00000061331 | Gm17132 | -1.13018 | 0.00273 | down |
| ENSMUSG00000041134 | Cyyr1 | -1.08877 | 0.00270 | down |
| ENSMUSG00000035711 | Dok3 | -1.25635 | 0.00269 | down |
| ENSMUSG00000097061 | 9330151L19Rik | -1.20395 | 0.00268 | down |
| ENSMUSG00000040429 | Mterf1a | -1.00937 | 0.00267 | down |
| ENSMUSG00000027858 | Tspan2 | -1.62427 | 0.00265 | down |
| ENSMUSG00000026077 | Npas2 | -3.70253 | 0.00265 | down |
| ENSMUSG00000037362 | Ccn3 | -4.15274 | 0.00263 | down |
| ENSMUSG00000075028 | Prdm11 | -1.33134 | 0.00263 | down |
| ENSMUSG00000046101 | Mcmdc2 | -1.9547 | 0.00263 | down |
| ENSMUSG00000027412 | Lpin3 | -1.35369 | 0.00262 | down |
| ENSMUSG00000025816 | Sec61a2 | -1.12309 | 0.00262 | down |
| ENSMUSG00000062210 | Tnfaip8 | -1.42871 | 0.00261 | down |
| ENSMUSG00000112229 | Gm48086 | -2.0779 | 0.00260 | down |
| ENSMUSG00000020593 | Lpin1 | -1.05349 | 0.00256 | down |
| ENSMUSG00000048960 | Prex2 | -1.29744 | 0.00256 | down |
| ENSMUSG00000031257 | Nox1 | -3.11562 | 0.00255 | down |
| ENSMUSG00000049561 | Olfr95 | -4.70193 | 0.00254 | down |
| ENSMUSG00000102241 | Gm37716 | -3.22382 | 0.00254 | down |
| ENSMUSG00000063600 | Egfem1 | -4.28747 | 0.00254 | down |
| ENSMUSG00000115200 | Gm46516 | -1.62406 | 0.00253 | down |
| ENSMUSG00000110344 | Smim36 | 1.320614 | 0.00249 | up |
| ENSMUSG00000115232 | Gm49378 | -4.95577 | 0.00249 | down |
| ENSMUSG00000097891 | Gm3650 | -1.06008 | 0.00249 | down |
| ENSMUSG00000110899 | Gm48840 | -1.66707 | 0.00248 | down |
| ENSMUSG00000108779 | Gm45691 | -2.11123 | 0.00247 | down |
| ENSMUSG00000105931 | Gm43014 | -1.83395 | 0.00245 | down |
| ENSMUSG00000049265 | Kcnk3 | -1.77989 | 0.00245 | down |
| ENSMUSG00000030322 | Mbd4 | -1.55305 | 0.00243 | down |
| ENSMUSG00000107481 | 4833403J16Rik | -2.59748 | 0.00243 | down |
| ENSMUSG00000051855 | Mest | -1.6386 | 0.00243 | down |
| ENSMUSG00000108218 | Olfr1372 | -2.41184 | 0.00242 | down |
| ENSMUSG00000031832 | Taf1c | -1.13939 | 0.00242 | down |
| ENSMUSG00000003849 | Nqo1 | 1.157512 | 0.00242 | up |
| ENSMUSG00000033855 | Ston1 | -1.43001 | 0.00242 | down |
| ENSMUSG00000041889 | Shisa4 | -2.34383 | 0.00241 | down |
| ENSMUSG00000121275 | - | -2.14312 | 0.00240 | down |
| ENSMUSG00000115184 | Gm49197 | -2.83547 | 0.00240 | down |
| ENSMUSG00000111326 | Gm3953 | -1.93308 | 0.00237 | down |
| ENSMUSG00000062683 | Atp5g2 | 2.53726 | 0.00237 | up |
| ENSMUSG00000121158 | Gm35162 | -2.77537 | 0.00236 | down |
| ENSMUSG00000036466 | Megf11 | -1.96293 | 0.00236 | down |
| ENSMUSG00000069294 | Vmn1r197 | -1.69309 | 0.00235 | down |
| ENSMUSG00000000440 | Pparg | 1.360529 | 0.00234 | up |
| ENSMUSG00000056987 | Garin2 | -3.58232 | 0.00234 | down |
| ENSMUSG00000023467 | Tulp2 | -4.4269 | 0.00232 | down |
| ENSMUSG00000026637 | Traf5 | -1.17343 | 0.00230 | down |
| ENSMUSG00000111938 | 2900045O20Rik | -3.32152 | 0.00230 | down |
| ENSMUSG00000027360 | Hdc | -1.35173 | 0.00230 | down |
| ENSMUSG00000115509 | Gm49012 | -3.10205 | 0.00229 | down |
| ENSMUSG00000022526 | Zfp251 | -1.30997 | 0.00228 | down |
| ENSMUSG00000029913 | Prdm5 | -3.63936 | 0.00228 | down |
| ENSMUSG00000108827 | Olfr1310 | -3.57174 | 0.00228 | down |
| ENSMUSG00000003062 | Stard3nl | 1.053225 | 0.00228 | up |
| ENSMUSG00000028177 | 1810013D15Rik | -1.78929 | 0.00226 | down |
| ENSMUSG00000036181 | H1f2 | 1.003073 | 0.00226 | up |
| ENSMUSG00000023274 | Cd4 | -1.65881 | 0.00222 | down |
| ENSMUSG00000045327 | 6330549D23Rik | -1.81438 | 0.00221 | down |
| ENSMUSG00000085665 | Gm12059 | 2.913397 | 0.00220 | up |
| ENSMUSG00000110273 | Gm41231 | 3.937535 | 0.00218 | up |
| ENSMUSG00000040170 | Fmo2 | -1.05956 | 0.00217 | down |
| ENSMUSG00000032098 | Treh | 2.870655 | 0.00216 | up |
| ENSMUSG00000107335 | Gm43372 | 3.718903 | 0.00216 | up |
| ENSMUSG00000023064 | Sncg | -2.08425 | 0.00215 | down |
| ENSMUSG00000031712 | Il15 | -1.424 | 0.00214 | down |
| ENSMUSG00000106838 | 1810017P11Rik | -1.59044 | 0.00213 | down |
| ENSMUSG00000047977 | Synb | -3.95338 | 0.00213 | down |
| ENSMUSG00000046818 | Ddit4l | -1.36907 | 0.00212 | down |
| ENSMUSG00000104682 | Gm42636 | -1.42888 | 0.00211 | down |
| ENSMUSG00000037624 | Kcnk2 | -2.14667 | 0.00211 | down |
| ENSMUSG00000066829 | Zfp810 | -1.07152 | 0.00211 | down |
| ENSMUSG00000003680 | Taf6l | 1.888756 | 0.00211 | up |
| ENSMUSG00000030787 | Lyve1 | -1.17322 | 0.00210 | down |
| ENSMUSG00000119946 | - | -2.30848 | 0.00210 | down |
| ENSMUSG00000051279 | Gdf6 | -2.80783 | 0.00210 | down |
| ENSMUSG00000097163 | BC051077 | -2.6299 | 0.00209 | down |
| ENSMUSG00000079036 | Alkbh1 | -1.20468 | 0.00209 | down |
| ENSMUSG00000115210 | Gm49308 | -4.46227 | 0.00209 | down |
| ENSMUSG00000104937 | Gm43057 | -3.41304 | 0.00209 | down |
| ENSMUSG00000094396 | Vmn2r124 | -1.38228 | 0.00207 | down |
| ENSMUSG00000032077 | Bud13 | -1.06623 | 0.00206 | down |
| ENSMUSG00000110151 | Gm38416 | -1.25146 | 0.00204 | down |
| ENSMUSG00000030263 | Irag2 | -1.24496 | 0.00204 | down |
| ENSMUSG00000052534 | Pbx1 | -1.02805 | 0.00202 | down |
| ENSMUSG00000118295 | Gm8437 | 3.463561 | 0.00202 | up |
| ENSMUSG00000091243 | Vgll3 | -2.8815 | 0.00201 | down |
| ENSMUSG00000003452 | Bicd1 | -1.89978 | 0.00201 | down |
| ENSMUSG00000120926 | - | -2.07301 | 0.00200 | down |
| ENSMUSG00000015316 | Slamf1 | -3.03669 | 0.00200 | down |
| ENSMUSG00000032690 | Oas2 | -1.03233 | 0.00200 | down |
| ENSMUSG00000034413 | Neurl1b | -1.89711 | 0.00199 | down |
| ENSMUSG00000102593 | Gm38384 | -3.03902 | 0.00199 | down |
| ENSMUSG00000021087 | Rtn1 | -1.61421 | 0.00199 | down |
| ENSMUSG00000112639 | A730063M14Rik | -1.17032 | 0.00199 | down |
| ENSMUSG00000028655 | Mfsd2a | 1.156081 | 0.00199 | up |
| ENSMUSG00000111517 | Olfr1238 | -2.06741 | 0.00199 | down |
| ENSMUSG00000026939 | Tmem141 | 1.081177 | 0.00198 | up |
| ENSMUSG00000028359 | Orm3 | 2.093144 | 0.00197 | up |
| ENSMUSG00000053825 | Ppfia2 | -2.43088 | 0.00196 | down |
| ENSMUSG00000024043 | Arhgap28 | -2.41106 | 0.00196 | down |
| ENSMUSG00000100768 | Gm29055 | -1.59026 | 0.00196 | down |
| ENSMUSG00000107117 | Gm43842 | -1.90321 | 0.00195 | down |
| ENSMUSG00000020589 | Cyria | -1.10424 | 0.00193 | down |
| ENSMUSG00000031684 | Slc10a7 | 1.006688 | 0.00192 | up |
| ENSMUSG00000042389 | Tsen2 | 1.518066 | 0.00191 | up |
| ENSMUSG00000020086 | Macroh2a2 | -2.09952 | 0.00190 | down |
| ENSMUSG00000106107 | Gm43190 | -1.07277 | 0.00190 | down |
| ENSMUSG00000084824 | Gm16344 | -1.80669 | 0.00189 | down |
| ENSMUSG00000057329 | Bcl2 | -1.36955 | 0.00188 | down |
| ENSMUSG00000121353 | - | -1.26387 | 0.00187 | down |
| ENSMUSG00000039018 | Mtg1 | 1.189793 | 0.00187 | up |
| ENSMUSG00000115575 | Gm49024 | -1.84993 | 0.00186 | down |
| ENSMUSG00000102460 | Gm38197 | -3.16904 | 0.00185 | down |
| ENSMUSG00000105691 | Gm42876 | -1.34355 | 0.00183 | down |
| ENSMUSG00000070417 | Olfr2 | -1.91226 | 0.00181 | down |
| ENSMUSG00000087273 | Gm13203 | 2.944466 | 0.00180 | up |
| ENSMUSG00000031373 | Car5b | -1.01988 | 0.00179 | down |
| ENSMUSG00000110547 | Gm29773 | -2.67714 | 0.00178 | down |
| ENSMUSG00000035142 | Nubpl | 2.104468 | 0.00178 | up |
| ENSMUSG00000017747 | Ghdc | -1.34975 | 0.00177 | down |
| ENSMUSG00000096356 | Olfr889 | -3.06174 | 0.00176 | down |
| ENSMUSG00000105224 | Gm3364 | -2.28576 | 0.00175 | down |
| ENSMUSG00000019756 | Prl8a1 | -1.39552 | 0.00174 | down |
| ENSMUSG00000015766 | Eps8 | -1.20294 | 0.00174 | down |
| ENSMUSG00000114584 | Gm47694 | -1.68158 | 0.00173 | down |
| ENSMUSG00000085887 | Arhgap27os3 | -4.31051 | 0.00173 | down |
| ENSMUSG00000089995 | Gm15716 | -3.13831 | 0.00173 | down |
| ENSMUSG00000032860 | P2ry2 | 1.015219 | 0.00172 | up |
| ENSMUSG00000013155 | Enkd1 | -1.80324 | 0.00172 | down |
| ENSMUSG00000118061 | Rbfaos | -2.06452 | 0.00172 | down |
| ENSMUSG00000087165 | 2010001A14Rik | 1.422793 | 0.00170 | up |
| ENSMUSG00000035208 | Slfn8 | -1.22187 | 0.00169 | down |
| ENSMUSG00000052544 | St6galnac3 | -1.74161 | 0.00168 | down |
| ENSMUSG00000028497 | Hacd4 | -1.14034 | 0.00168 | down |
| ENSMUSG00000078789 | Dph1 | 1.332457 | 0.00167 | up |
| ENSMUSG00000052658 | 5830454E08Rik | -1.60112 | 0.00167 | down |
| ENSMUSG00000098488 | Pla2g4b | -1.46944 | 0.00165 | down |
| ENSMUSG00000054582 | Pabpc1l | -4.67385 | 0.00164 | down |
| ENSMUSG00000020805 | Slc13a5 | 1.781112 | 0.00163 | up |
| ENSMUSG00000118053 | Gm50244 | 1.45089 | 0.00163 | up |
| ENSMUSG00000034573 | Ptpn13 | -1.96161 | 0.00163 | down |
| ENSMUSG00000087054 | Gm12405 | -2.70218 | 0.00162 | down |
| ENSMUSG00000026840 | Lamc3 | -1.0279 | 0.00162 | down |
| ENSMUSG00000039264 | Gimap3 | -1.1896 | 0.00161 | down |
| ENSMUSG00000105207 | Gm42927 | -1.30124 | 0.00161 | down |
| ENSMUSG00000108494 | Gm45203 | -1.64589 | 0.00160 | down |
| ENSMUSG00000111521 | Gm48529 | -1.11114 | 0.00160 | down |
| ENSMUSG00000097204 | Gm17690 | -1.02128 | 0.00159 | down |
| ENSMUSG00000100257 | C4bp-ps1 | -2.15372 | 0.00157 | down |
| ENSMUSG00000108802 | Gm44769 | -4.00247 | 0.00157 | down |
| ENSMUSG00000036036 | Zfp57 | -1.92139 | 0.00156 | down |
| ENSMUSG00000121374 | - | 1.428067 | 0.00156 | up |
| ENSMUSG00000106717 | Gm42798 | -1.92989 | 0.00155 | down |
| ENSMUSG00000094786 | Gm14403 | -1.01349 | 0.00155 | down |
| ENSMUSG00000109108 | Gm36371 | -3.36808 | 0.00153 | down |
| ENSMUSG00000057594 | Arl16 | 1.279818 | 0.00153 | up |
| ENSMUSG00000105519 | E430021H15Rik | -2.31348 | 0.00152 | down |
| ENSMUSG00000106924 | Gm42857 | -1.60103 | 0.00151 | down |
| ENSMUSG00000118661 | Muc6 | -1.9016 | 0.00150 | down |
| ENSMUSG00000019823 | Mical1 | -1.07016 | 0.00149 | down |
| ENSMUSG00000038903 | Ccdc68 | -1.3385 | 0.00149 | down |
| ENSMUSG00000111132 | Gm48142 | -3.9222 | 0.00149 | down |
| ENSMUSG00000007987 | Ift22 | 1.110883 | 0.00149 | up |
| ENSMUSG00000018648 | Dusp14 | -1.89432 | 0.00148 | down |
| ENSMUSG00000060149 | BC002059 | -1.02424 | 0.00148 | down |
| ENSMUSG00000074922 | Fam122a | 1.549921 | 0.00148 | up |
| ENSMUSG00000067149 | Jchain | -1.18694 | 0.00148 | down |
| ENSMUSG00000056515 | Rab31 | -1.06228 | 0.00147 | down |
| ENSMUSG00000073973 | Olfr552 | -1.09634 | 0.00147 | down |
| ENSMUSG00000028443 | Nudt2 | 1.314316 | 0.00147 | up |
| ENSMUSG00000032648 | Pygm | -2.09722 | 0.00146 | down |
| ENSMUSG00000108037 | Gm44597 | -1.25389 | 0.00144 | down |
| ENSMUSG00000086877 | A230072C01Rik | -1.02662 | 0.00144 | down |
| ENSMUSG00000031534 | Smim19 | 1.367574 | 0.00143 | up |
| ENSMUSG00000117853 | Vmn1r88 | -1.72043 | 0.00143 | down |
| ENSMUSG00000071356 | Reg3b | 4.349534 | 0.00143 | up |
| ENSMUSG00000109168 | Gm44709 | -1.15091 | 0.00143 | down |
| ENSMUSG00000020668 | Kif3c | -1.65962 | 0.00143 | down |
| ENSMUSG00000081471 | Gm14735 | -1.34225 | 0.00143 | down |
| ENSMUSG00000019842 | Traf3ip2 | -1.03333 | 0.00143 | down |
| ENSMUSG00000111828 | D830035M03Rik | 2.47757 | 0.00142 | up |
| ENSMUSG00000030887 | Pdzd9 | -2.26371 | 0.00142 | down |
| ENSMUSG00000044165 | Bcl2l15 | -3.78858 | 0.00141 | down |
| ENSMUSG00000121141 | - | -1.46759 | 0.00140 | down |
| ENSMUSG00000027628 | Aar2 | 1.063432 | 0.00139 | up |
| ENSMUSG00000040663 | Clcf1 | -1.15783 | 0.00138 | down |
| ENSMUSG00000111269 | Gm47933 | -1.38199 | 0.00138 | down |
| ENSMUSG00000081948 | Olfr1191 | -3.66204 | 0.00137 | down |
| ENSMUSG00000028617 | Lrrc42 | 1.112893 | 0.00137 | up |
| ENSMUSG00000103835 | Gm37612 | -4.2367 | 0.00136 | down |
| ENSMUSG00000029231 | Pdgfra | -1.02204 | 0.00136 | down |
| ENSMUSG00000019906 | Lin7a | 1.115731 | 0.00136 | up |
| ENSMUSG00000049493 | Pls1 | 1.838023 | 0.00135 | up |
| ENSMUSG00000035239 | Neu3 | -1.89781 | 0.00134 | down |
| ENSMUSG00000115882 | Gm5481 | -3.05065 | 0.00134 | down |
| ENSMUSG00000030401 | Rtn2 | -1.9748 | 0.00133 | down |
| ENSMUSG00000057207 | Olfr1028 | -2.54382 | 0.00133 | down |
| ENSMUSG00000050772 | Olfr1124 | -2.16044 | 0.00133 | down |
| ENSMUSG00000103932 | Gm36963 | -2.37315 | 0.00133 | down |
| ENSMUSG00000081058 | H3c15 | 2.195552 | 0.00132 | up |
| ENSMUSG00000021028 | Mbip | -1.06101 | 0.00132 | down |
| ENSMUSG00000030664 | Sox6os | 1.739148 | 0.00132 | up |
| ENSMUSG00000091239 | Vmn2r76 | -2.97991 | 0.00132 | down |
| ENSMUSG00000070532 | Ccdc190 | -5.12097 | 0.00131 | down |
| ENSMUSG00000120050 | - | -2.27127 | 0.00131 | down |
| ENSMUSG00000119953 | - | -1.60247 | 0.00130 | down |
| ENSMUSG00000024087 | Cyp1b1 | -1.74029 | 0.00130 | down |
| ENSMUSG00000064326 | Siva1 | 1.547922 | 0.00130 | up |
| ENSMUSG00000045328 | Cenpe | -4.15157 | 0.00129 | down |
| ENSMUSG00000036864 | Proser3 | -1.27934 | 0.00129 | down |
| ENSMUSG00000027993 | Trim2 | -1.41097 | 0.00128 | down |
| ENSMUSG00000114516 | Gm46440 | -2.53631 | 0.00128 | down |
| ENSMUSG00000027684 | Mecom | -1.7494 | 0.00128 | down |
| ENSMUSG00000019775 | Rgs17 | -2.48954 | 0.00128 | down |
| ENSMUSG00000081485 | Gm12338 | -1.57511 | 0.00128 | down |
| ENSMUSG00000114886 | Gm48432 | 1.950951 | 0.00127 | up |
| ENSMUSG00000091151 | Vmn1r224 | -2.34597 | 0.00126 | down |
| ENSMUSG00000020437 | Myo1g | -1.46738 | 0.00126 | down |
| ENSMUSG00000041559 | Fmod | -1.1954 | 0.00126 | down |
| ENSMUSG00000103364 | Gm38157 | -2.46639 | 0.00125 | down |
| ENSMUSG00000113159 | Gm48771 | -1.61641 | 0.00125 | down |
| ENSMUSG00000017057 | Il13ra1 | 1.669969 | 0.00124 | up |
| ENSMUSG00000080727 | C920021L13Rik | -1.38175 | 0.00123 | down |
| ENSMUSG00000000686 | Abhd15 | 1.188286 | 0.00122 | up |
| ENSMUSG00000078937 | Cpt1b | -2.05559 | 0.00121 | down |
| ENSMUSG00000032334 | Loxl1 | -1.066 | 0.00120 | down |
| ENSMUSG00000051319 | Mtln | 1.118277 | 0.00119 | up |
| ENSMUSG00000112354 | Gm33843 | -2.98141 | 0.00118 | down |
| ENSMUSG00000029605 | Oas1b | -1.65782 | 0.00118 | down |
| ENSMUSG00000113543 | Gm36264 | -1.46459 | 0.00118 | down |
| ENSMUSG00000111923 | Gm34777 | -1.97956 | 0.00118 | down |
| ENSMUSG00000063556 | Gm10132 | -3.98089 | 0.00117 | down |
| ENSMUSG00000016494 | Cd34 | -1.24263 | 0.00117 | down |
| ENSMUSG00000029632 | Ndufa4 | 1.024884 | 0.00117 | up |
| ENSMUSG00000051504 | Siglech | -2.23297 | 0.00116 | down |
| ENSMUSG00000048603 | Gm9828 | -1.04195 | 0.00115 | down |
| ENSMUSG00000034854 | Mfsd12 | 1.05546 | 0.00115 | up |
| ENSMUSG00000121029 | - | -1.93659 | 0.00115 | down |
| ENSMUSG00000121304 | - | -1.83472 | 0.00115 | down |
| ENSMUSG00000029521 | Chek2 | -1.70293 | 0.00114 | down |
| ENSMUSG00000023333 | Gcm1 | -2.41602 | 0.00113 | down |
| ENSMUSG00000018378 | Cuedc1 | -1.04966 | 0.00113 | down |
| ENSMUSG00000049103 | Ccr2 | -1.43323 | 0.00112 | down |
| ENSMUSG00000020886 | Dlg4 | -1.34704 | 0.00112 | down |
| ENSMUSG00000029778 | Adcyap1r1 | -1.56406 | 0.00112 | down |
| ENSMUSG00000026480 | Ncf2 | -1.2478 | 0.00111 | down |
| ENSMUSG00000097583 | 6430590A07Rik | -2.1117 | 0.00111 | down |
| ENSMUSG00000115520 | Gm41335 | -4.47876 | 0.00110 | down |
| ENSMUSG00000019235 | Rps6kl1 | -1.33926 | 0.00109 | down |
| ENSMUSG00000113388 | Gm48111 | -1.00442 | 0.00109 | down |
| ENSMUSG00000005583 | Mef2c | -1.46164 | 0.00109 | down |
| ENSMUSG00000121373 | Cyp2c53-ps | -5.83773 | 0.00108 | down |
| ENSMUSG00000039620 | Trmt9b | -1.17566 | 0.00107 | down |
| ENSMUSG00000027505 | Fam209 | -3.54057 | 0.00107 | down |
| ENSMUSG00000026229 | Psmd1 | -1.16135 | 0.00107 | down |
| ENSMUSG00000057802 | Gm10030 | -1.70946 | 0.00106 | down |
| ENSMUSG00000052331 | Ankrd44 | -1.12872 | 0.00106 | down |
| ENSMUSG00000055923 | Aasdh | -1.02659 | 0.00105 | down |
| ENSMUSG00000052013 | Btla | -1.79445 | 0.00104 | down |
| ENSMUSG00000110529 | Gm45694 | -2.11084 | 0.00104 | down |
| ENSMUSG00000097639 | Platr4 | -1.59939 | 0.00104 | down |
| ENSMUSG00000048997 | Atxn7l2 | -1.35592 | 0.00104 | down |
| ENSMUSG00000107690 | Gm44044 | -2.19342 | 0.00103 | down |
| ENSMUSG00000120574 | - | -2.25153 | 0.00103 | down |
| ENSMUSG00000098404 | Mrip-ps | 2.309407 | 0.00102 | up |
| ENSMUSG00000086527 | Gm15856 | -4.80334 | 0.00101 | down |
| ENSMUSG00000026343 | Gpr39 | 1.192776 | 0.00101 | up |
| ENSMUSG00000032315 | Cyp1a1 | -1.55452 | 0.00101 | down |
| ENSMUSG00000115422 | 4930452G13Rik | -2.7811 | 0.00100 | down |
| ENSMUSG00000029797 | Sspo | -2.00461 | 0.00100 | down |
| ENSMUSG00000062488 | Ifit3b | -1.69837 | 0.00098 | down |
| ENSMUSG00000067916 | Zfp991 | -1.57477 | 0.00098 | down |
| ENSMUSG00000006360 | Crip1 | -1.3679 | 0.00097 | down |
| ENSMUSG00000034837 | Gnat1 | 1.665541 | 0.00097 | up |
| ENSMUSG00002076161 | Rn7sk | -1.3147 | 0.00097 | down |
| ENSMUSG00000002043 | Trappc6a | 1.116944 | 0.00096 | up |
| ENSMUSG00000061322 | Dnai1 | 1.948389 | 0.00096 | up |
| ENSMUSG00000086477 | Gm15506 | -1.59479 | 0.00095 | down |
| ENSMUSG00000042638 | Gucy2c | 3.030364 | 0.00095 | up |
| ENSMUSG00000110365 | Gm38947 | -1.82978 | 0.00095 | down |
| ENSMUSG00000118298 | Vmn1r60 | -3.0797 | 0.00094 | down |
| ENSMUSG00000026417 | Pigr | -1.07122 | 0.00094 | down |
| ENSMUSG00000025888 | Casp1 | -1.41258 | 0.00094 | down |
| ENSMUSG00000036390 | Gadd45a | 1.398568 | 0.00093 | up |
| ENSMUSG00000019066 | Rab3d | 1.30831 | 0.00092 | up |
| ENSMUSG00000113262 | Gm48551 | -2.27616 | 0.00091 | down |
| ENSMUSG00000023906 | Cldn6 | -2.08747 | 0.00090 | down |
| ENSMUSG00000049287 | Iba57 | 1.209518 | 0.00090 | up |
| ENSMUSG00000026675 | Hsd17b7 | 1.030341 | 0.00090 | up |
| ENSMUSG00000087684 | 1200007C13Rik | 3.931282 | 0.00090 | up |
| ENSMUSG00000000340 | Dbt | -1.15717 | 0.00089 | down |
| ENSMUSG00000107743 | Gm44087 | -1.33669 | 0.00089 | down |
| ENSMUSG00000114247 | Gm32063 | -1.36104 | 0.00089 | down |
| ENSMUSG00000084796 | Mir142hg | -1.1711 | 0.00089 | down |
| ENSMUSG00000022840 | Adcy5 | -1.57992 | 0.00089 | down |
| ENSMUSG00000120294 | - | -1.64644 | 0.00088 | down |
| ENSMUSG00000030067 | Foxp1 | -1.53995 | 0.00088 | down |
| ENSMUSG00000040312 | Cchcr1 | -1.10499 | 0.00087 | down |
| ENSMUSG00000099590 | C330022C24Rik | -1.40916 | 0.00087 | down |
| ENSMUSG00000070605 | Zfp992 | -1.08729 | 0.00087 | down |
| ENSMUSG00000078427 | Sarnp | 1.068573 | 0.00087 | up |
| ENSMUSG00000103475 | Gm37697 | -1.26584 | 0.00087 | down |
| ENSMUSG00000025432 | Avil | -2.38677 | 0.00086 | down |
| ENSMUSG00000022436 | Sh3bp1 | -1.45182 | 0.00086 | down |
| ENSMUSG00000056124 | B4galt6 | -1.12162 | 0.00086 | down |
| ENSMUSG00000028005 | Gucy1b1 | -1.14487 | 0.00086 | down |
| ENSMUSG00000025571 | Tnrc6c | -1.02258 | 0.00085 | down |
| ENSMUSG00000107143 | Gm6598 | -1.46338 | 0.00084 | down |
| ENSMUSG00000109016 | Gm44647 | -5.46215 | 0.00084 | down |
| ENSMUSG00000091076 | Vmn2r115 | -1.57827 | 0.00084 | down |
| ENSMUSG00000109336 | Samd4b | -1.18919 | 0.00084 | down |
| ENSMUSG00000027204 | Fbn1 | -1.36916 | 0.00083 | down |
| ENSMUSG00000035202 | Lars2 | -1.40387 | 0.00083 | down |
| ENSMUSG00000074469 | Gm15348 | 3.588528 | 0.00082 | up |
| ENSMUSG00000042942 | Greb1l | -2.13076 | 0.00082 | down |
| ENSMUSG00000031506 | Ptpn7 | -1.32023 | 0.00080 | down |
| ENSMUSG00000081205 | Gm5940 | -5.96659 | 0.00080 | down |
| ENSMUSG00000107306 | Gm42577 | -1.15885 | 0.00079 | down |
| ENSMUSG00000109038 | Gm45120 | -2.53585 | 0.00079 | down |
| ENSMUSG00000097360 | 9430065F17Rik | -2.27234 | 0.00079 | down |
| ENSMUSG00000121501 | - | -1.08451 | 0.00079 | down |
| ENSMUSG00000085180 | AI838599 | -2.00713 | 0.00078 | down |
| ENSMUSG00000057219 | Armc7 | -1.09066 | 0.00078 | down |
| ENSMUSG00000087632 | Gm6058 | -3.38527 | 0.00078 | down |
| ENSMUSG00000035722 | Abca7 | -1.00968 | 0.00078 | down |
| ENSMUSG00000039193 | Nlrc4 | -1.04174 | 0.00078 | down |
| ENSMUSG00000033361 | Prrg3 | -2.93796 | 0.00077 | down |
| ENSMUSG00000045237 | Eola1 | 1.278547 | 0.00077 | up |
| ENSMUSG00000066705 | Fxyd6 | -2.47058 | 0.00077 | down |
| ENSMUSG00000035378 | Shq1 | 1.07296 | 0.00077 | up |
| ENSMUSG00000119210 | n-R5s117 | -5.13894 | 0.00076 | down |
| ENSMUSG00000116004 | Gm49539 | -4.74992 | 0.00076 | down |
| ENSMUSG00000096056 | Gm21986 | -4.24759 | 0.00076 | down |
| ENSMUSG00000034205 | Loxl2 | -1.15717 | 0.00076 | down |
| ENSMUSG00000097055 | Gm4419 | -2.39768 | 0.00075 | down |
| ENSMUSG00000015812 | Gnrh1 | -2.85096 | 0.00075 | down |
| ENSMUSG00000039474 | Wfs1 | 1.183611 | 0.00075 | up |
| ENSMUSG00000035184 | Fam124a | -1.15221 | 0.00075 | down |
| ENSMUSG00000112461 | Gm47625 | -3.16852 | 0.00074 | down |
| ENSMUSG00000031145 | Prickle3 | -1.06693 | 0.00073 | down |
| ENSMUSG00000034209 | Rasl10a | -4.27911 | 0.00073 | down |
| ENSMUSG00000105160 | A530030E21Rik | -2.0071 | 0.00073 | down |
| ENSMUSG00000021306 | Gpr137b | -1.39018 | 0.00072 | down |
| ENSMUSG00000020601 | Trib2 | -1.29347 | 0.00072 | down |
| ENSMUSG00000038872 | Zfhx3 | -1.24414 | 0.00072 | down |
| ENSMUSG00000113889 | Gm48501 | -1.27581 | 0.00071 | down |
| ENSMUSG00002076650 | Snord3b1 | -4.01124 | 0.00071 | down |
| ENSMUSG00000026111 | Unc50 | 1.189003 | 0.00071 | up |
| ENSMUSG00000001034 | Mapk7 | -1.13817 | 0.00071 | down |
| ENSMUSG00000029499 | Pxmp2 | 1.02228 | 0.00070 | up |
| ENSMUSG00000038070 | Cntln | -1.52503 | 0.00070 | down |
| ENSMUSG00000053091 | Lins1 | -1.03152 | 0.00069 | down |
| ENSMUSG00000052675 | Zfp112 | -1.24741 | 0.00068 | down |
| ENSMUSG00000096220 | Olfr775 | -1.74316 | 0.00068 | down |
| ENSMUSG00000046318 | Ccbe1 | -1.10189 | 0.00068 | down |
| ENSMUSG00000043794 | D830025C05Rik | -2.83229 | 0.00068 | down |
| ENSMUSG00000028549 | Itgb3bp | 1.954661 | 0.00068 | up |
| ENSMUSG00000051185 | Fam174a | 1.035695 | 0.00067 | up |
| ENSMUSG00000073940 | Hbb-bt | -1.16222 | 0.00067 | down |
| ENSMUSG00000051246 | Msantd1 | -2.20776 | 0.00066 | down |
| ENSMUSG00000094156 | Sult2a7 | 2.512527 | 0.00065 | up |
| ENSMUSG00000045589 | Frrs1l | -3.32907 | 0.00065 | down |
| ENSMUSG00000118698 | Gm25890 | -4.15917 | 0.00065 | down |
| ENSMUSG00000118936 | Gm22614 | -4.15917 | 0.00065 | down |
| ENSMUSG00000000579 | Dynlt1c | -1.02531 | 0.00065 | down |
| ENSMUSG00000120252 | - | -4.27402 | 0.00065 | down |
| ENSMUSG00000063954 | H2ac19 | 1.010844 | 0.00065 | up |
| ENSMUSG00000063698 | Sfxn4 | -3.04621 | 0.00065 | down |
| ENSMUSG00000025959 | Klf7 | -1.2589 | 0.00064 | down |
| ENSMUSG00000090799 | Klhl33 | -2.28093 | 0.00064 | down |
| ENSMUSG00000039962 | Olfr906 | -4.71789 | 0.00064 | down |
| ENSMUSG00000048865 | Arhgap30 | -1.13316 | 0.00064 | down |
| ENSMUSG00000102868 | Gm37633 | -3.6334 | 0.00064 | down |
| ENSMUSG00000021707 | Dhfr | 1.013327 | 0.00064 | up |
| ENSMUSG00000112110 | Gm15608 | -1.03642 | 0.00064 | down |
| ENSMUSG00000083306 | Gm13868 | -2.70797 | 0.00063 | down |
| ENSMUSG00000048445 | Ccdc57 | -1.34741 | 0.00063 | down |
| ENSMUSG00000030905 | Crym | 3.444473 | 0.00063 | up |
| ENSMUSG00000106943 | Dancr | -1.35179 | 0.00063 | down |
| ENSMUSG00000108780 | 5430434F05Rik | -3.00275 | 0.00062 | down |
| ENSMUSG00000003573 | Homer3 | -1.64645 | 0.00062 | down |
| ENSMUSG00000038538 | Ubn2 | -1.69546 | 0.00062 | down |
| ENSMUSG00000032322 | Pstpip1 | -1.93294 | 0.00062 | down |
| ENSMUSG00000001131 | Timp1 | 2.724499 | 0.00062 | up |
| ENSMUSG00000085923 | Gm12781 | 1.161149 | 0.00061 | up |
| ENSMUSG00000049580 | Tsku | -1.76079 | 0.00061 | down |
| ENSMUSG00000112319 | Gm47221 | -1.16908 | 0.00061 | down |
| ENSMUSG00000056071 | S100a9 | 1.335632 | 0.00060 | up |
| ENSMUSG00000015850 | Adamtsl4 | -1.12447 | 0.00060 | down |
| ENSMUSG00000036880 | Acaa2 | 1.034954 | 0.00060 | up |
| ENSMUSG00000032359 | Ctsh | 1.05004 | 0.00060 | up |
| ENSMUSG00000028189 | Ctbs | 1.124197 | 0.00059 | up |
| ENSMUSG00000047502 | Mroh7 | -2.10627 | 0.00059 | down |
| ENSMUSG00000089978 | Crb1-ps | -4.7067 | 0.00059 | down |
| ENSMUSG00000002020 | Ltbp2 | -2.25949 | 0.00059 | down |
| ENSMUSG00000054545 | Ugt1a6a | 1.317792 | 0.00059 | up |
| ENSMUSG00000041483 | Zfp281 | -1.64925 | 0.00059 | down |
| ENSMUSG00000033788 | Dysf | -1.09868 | 0.00059 | down |
| ENSMUSG00000021208 | Ifi27l2b | 2.492081 | 0.00058 | up |
| ENSMUSG00000086859 | Snhg20 | -1.21392 | 0.00058 | down |
| ENSMUSG00000021775 | Nr1d2 | 1.233933 | 0.00058 | up |
| ENSMUSG00000028068 | Iqgap3 | -3.31629 | 0.00057 | down |
| ENSMUSG00000050621 | Rps27rt | -3.78052 | 0.00057 | down |
| ENSMUSG00000043015 | Nemp2 | -1.49799 | 0.00056 | down |
| ENSMUSG00000020056 | Washc3 | 1.144861 | 0.00055 | up |
| ENSMUSG00000006362 | Cbfa2t3 | -1.27297 | 0.00054 | down |
| ENSMUSG00000096795 | Zfp433 | -1.61976 | 0.00054 | down |
| ENSMUSG00000054728 | Phactr1 | -1.65476 | 0.00054 | down |
| ENSMUSG00000040274 | Cdk6 | -1.23128 | 0.00053 | down |
| ENSMUSG00000037344 | Slc12a9 | -1.04183 | 0.00053 | down |
| ENSMUSG00000097124 | A530020G20Rik | -1.40941 | 0.00053 | down |
| ENSMUSG00000045991 | Onecut2 | -1.04511 | 0.00053 | down |
| ENSMUSG00000015759 | Cnih1 | 1.003749 | 0.00053 | up |
| ENSMUSG00000057359 | Gm17494 | -1.48396 | 0.00053 | down |
| ENSMUSG00000093916 | Gm379 | 1.699819 | 0.00052 | up |
| ENSMUSG00000087042 | Gm11611 | -5.31636 | 0.00052 | down |
| ENSMUSG00000028268 | Gbp3 | -1.19088 | 0.00052 | down |
| ENSMUSG00000030882 | Dnhd1 | -1.13883 | 0.00052 | down |
| ENSMUSG00000034459 | Ifit1 | -1.47584 | 0.00052 | down |
| ENSMUSG00000096852 | Cyp2d12 | -1.19286 | 0.00051 | down |
| ENSMUSG00000050855 | Zfp940 | -1.62167 | 0.00051 | down |
| ENSMUSG00000022621 | Rabl2 | -1.2238 | 0.00051 | down |
| ENSMUSG00000027665 | Pik3ca | -1.07547 | 0.00051 | down |
| ENSMUSG00000030278 | Cidec | 3.011694 | 0.00051 | up |
| ENSMUSG00000111361 | Gm47445 | -1.97088 | 0.00050 | down |
| ENSMUSG00000047342 | Zfp286 | -4.75478 | 0.00050 | down |
| ENSMUSG00000033420 | Antxr1 | -1.76246 | 0.00050 | down |
| ENSMUSG00000039542 | Ncam1 | -1.89375 | 0.00050 | down |
| ENSMUSG00000114828 | AI463229 | -1.61311 | 0.00050 | down |
| ENSMUSG00000035168 | Tanc1 | -1.03746 | 0.00050 | down |
| ENSMUSG00000036826 | Igflr1 | -2.33742 | 0.00049 | down |
| ENSMUSG00000091577 | Gm6211 | -2.95794 | 0.00049 | down |
| ENSMUSG00000022505 | Emp2 | -1.24818 | 0.00049 | down |
| ENSMUSG00000105476 | Gm35439 | -1.29632 | 0.00048 | down |
| ENSMUSG00000049107 | Ntf3 | 1.643083 | 0.00048 | up |
| ENSMUSG00000094708 | Gm10359 | 1.018341 | 0.00048 | up |
| ENSMUSG00000024079 | Eif2ak2 | -1.14796 | 0.00048 | down |
| ENSMUSG00000117872 | A530088E08Rik | -2.28648 | 0.00048 | down |
| ENSMUSG00000035948 | Acss3 | 1.42962 | 0.00048 | up |
| ENSMUSG00000029470 | P2rx4 | -1.02843 | 0.00047 | down |
| ENSMUSG00000120095 | - | -1.99876 | 0.00047 | down |
| ENSMUSG00000049420 | Tmem200a | -3.56639 | 0.00047 | down |
| ENSMUSG00000028643 | Svbp | 1.040861 | 0.00047 | up |
| ENSMUSG00000106150 | 4921527H02Rik | -2.6829 | 0.00047 | down |
| ENSMUSG00000029516 | Cit | -2.06143 | 0.00047 | down |
| ENSMUSG00000028803 | Nipal3 | 1.108335 | 0.00046 | up |
| ENSMUSG00000091144 | Phf11c | -1.1305 | 0.00046 | down |
| ENSMUSG00000086848 | Lce6a | -5.24983 | 0.00045 | down |
| ENSMUSG00000021615 | Xrcc4 | 1.343735 | 0.00045 | up |
| ENSMUSG00000097673 | Gm26608 | -1.2731 | 0.00045 | down |
| ENSMUSG00000103085 | Gm38120 | -3.27422 | 0.00045 | down |
| ENSMUSG00000027133 | Nop10 | 1.090219 | 0.00045 | up |
| ENSMUSG00000114458 | Gm47551 | -2.35775 | 0.00045 | down |
| ENSMUSG00000104413 | Gm37065 | -2.95508 | 0.00045 | down |
| ENSMUSG00000018486 | Wnt9b | -1.47888 | 0.00045 | down |
| ENSMUSG00000016028 | Celsr1 | -1.03447 | 0.00045 | down |
| ENSMUSG00000040528 | Milr1 | -2.01606 | 0.00044 | down |
| ENSMUSG00000068877 | Selenbp2 | -1.59845 | 0.00044 | down |
| ENSMUSG00000039199 | Zdhhc1 | -1.14309 | 0.00044 | down |
| ENSMUSG00000115279 | Gm49273 | -1.63508 | 0.00044 | down |
| ENSMUSG00000032374 | Plod2 | -1.52765 | 0.00044 | down |
| ENSMUSG00000107659 | Gm44170 | 1.044452 | 0.00044 | up |
| ENSMUSG00000113924 | Gm48493 | -1.24482 | 0.00044 | down |
| ENSMUSG00000085867 | Gm5834 | -1.75965 | 0.00044 | down |
| ENSMUSG00000052271 | Bhlha15 | 2.651162 | 0.00043 | up |
| ENSMUSG00000035967 | Ints6l | -1.01432 | 0.00043 | down |
| ENSMUSG00000034793 | G6pc3 | -1.11642 | 0.00043 | down |
| ENSMUSG00000073434 | Wdr90 | -1.09325 | 0.00043 | down |
| ENSMUSG00000083594 | Gm13722 | -1.17739 | 0.00043 | down |
| ENSMUSG00000033910 | Gucy1a1 | -1.08299 | 0.00042 | down |
| ENSMUSG00000030750 | Nsmce1 | 1.236325 | 0.00042 | up |
| ENSMUSG00000034401 | Spata6 | -1.4983 | 0.00042 | down |
| ENSMUSG00000015880 | Ncapg | -4.6831 | 0.00042 | down |
| ENSMUSG00000019899 | Lama2 | -1.40372 | 0.00042 | down |
| ENSMUSG00000000958 | Slc7a7 | -1.10778 | 0.00042 | down |
| ENSMUSG00000027559 | Car3 | -1.04279 | 0.00042 | down |
| ENSMUSG00000118087 | 4833438C02Rik | -1.21804 | 0.00041 | down |
| ENSMUSG00000038173 | Enpp6 | -2.2608 | 0.00041 | down |
| ENSMUSG00000052557 | Gan | -1.23222 | 0.00041 | down |
| ENSMUSG00000008575 | Nfib | -1.63669 | 0.00041 | down |
| ENSMUSG00000060487 | Samd5 | -1.94207 | 0.00040 | down |
| ENSMUSG00000070867 | Trabd2b | -1.51596 | 0.00040 | down |
| ENSMUSG00000028536 | 2610528J11Rik | 1.44477 | 0.00040 | up |
| ENSMUSG00000027583 | Zbtb46 | -1.14946 | 0.00040 | down |
| ENSMUSG00000050332 | Amer1 | -1.30997 | 0.00039 | down |
| ENSMUSG00000036158 | Prickle1 | -1.18098 | 0.00039 | down |
| ENSMUSG00000035713 | Usp35 | -1.14807 | 0.00039 | down |
| ENSMUSG00000078687 | Mup8 | -1.67762 | 0.00038 | down |
| ENSMUSG00000043872 | Zmym1 | -1.28373 | 0.00038 | down |
| ENSMUSG00000061718 | Ppp1r1b | -1.06926 | 0.00038 | down |
| ENSMUSG00000105095 | 8430422M14Rik | -1.46233 | 0.00037 | down |
| ENSMUSG00000074024 | 4632427E13Rik | -1.75091 | 0.00037 | down |
| ENSMUSG00000107771 | Gm8956 | -2.58224 | 0.00037 | down |
| ENSMUSG00000045826 | Ptprcap | -2.65992 | 0.00037 | down |
| ENSMUSG00000101906 | Mrgprc2-ps | -2.16867 | 0.00037 | down |
| ENSMUSG00000117990 | Gm32027 | -1.53566 | 0.00036 | down |
| ENSMUSG00000058331 | Zfp85 | -1.11396 | 0.00036 | down |
| ENSMUSG00000035401 | Emsy | -1.52164 | 0.00036 | down |
| ENSMUSG00000026657 | Frmd4a | -1.1431 | 0.00036 | down |
| ENSMUSG00000095098 | Ccdc85b | 1.193658 | 0.00036 | up |
| ENSMUSG00000038400 | Pmepa1 | -1.27752 | 0.00036 | down |
| ENSMUSG00000020231 | Dip2a | -1.22733 | 0.00036 | down |
| ENSMUSG00000037845 | Fdxacb1 | -1.04948 | 0.00035 | down |
| ENSMUSG00000024871 | Doc2g | -1.46222 | 0.00035 | down |
| ENSMUSG00000020059 | Sycp3 | -5.42794 | 0.00035 | down |
| ENSMUSG00000041235 | Chd7 | -1.23514 | 0.00035 | down |
| ENSMUSG00000051238 | Swsap1 | 1.32355 | 0.00035 | up |
| ENSMUSG00000068184 | Ndufaf2 | 1.011839 | 0.00035 | up |
| ENSMUSG00000004842 | Pou1f1 | -2.54762 | 0.00034 | down |
| ENSMUSG00000022885 | St6gal1 | 1.093849 | 0.00034 | up |
| ENSMUSG00000113722 | Snhg10 | -2.06334 | 0.00034 | down |
| ENSMUSG00000036330 | Slc18a1 | 1.289084 | 0.00034 | up |
| ENSMUSG00000052738 | Suclg1 | 1.159645 | 0.00034 | up |
| ENSMUSG00000004100 | Ppan | -1.09023 | 0.00034 | down |
| ENSMUSG00000116858 | Gm49797 | -1.1518 | 0.00033 | down |
| ENSMUSG00000022475 | Hdac7 | -1.01897 | 0.00033 | down |
| ENSMUSG00000113101 | Gm33424 | -3.41882 | 0.00033 | down |
| ENSMUSG00000036526 | Card11 | -1.77131 | 0.00032 | down |
| ENSMUSG00000024678 | Ms4a4d | -1.38067 | 0.00032 | down |
| ENSMUSG00000063406 | Tmed5 | -1.69441 | 0.00032 | down |
| ENSMUSG00000053604 | Rpia | -1.0987 | 0.00032 | down |
| ENSMUSG00000032218 | Ccnb2 | -2.66258 | 0.00032 | down |
| ENSMUSG00000006638 | Abhd1 | -1.18653 | 0.00032 | down |
| ENSMUSG00000029641 | Rasl11a | -1.93139 | 0.00032 | down |
| ENSMUSG00000048406 | B330016D10Rik | -1.59784 | 0.00031 | down |
| ENSMUSG00000001036 | Epn2 | -1.10237 | 0.00031 | down |
| ENSMUSG00000106547 | B230303O12Rik | -2.48999 | 0.00031 | down |
| ENSMUSG00000021539 | Lect2 | 1.116231 | 0.00031 | up |
| ENSMUSG00000038418 | Egr1 | 2.362399 | 0.00030 | up |
| ENSMUSG00000087611 | 4930458D05Rik | 4.457494 | 0.00030 | up |
| ENSMUSG00000021252 | Erg28 | 1.820757 | 0.00030 | up |
| ENSMUSG00000005483 | Dnajb1 | 1.060185 | 0.00030 | up |
| ENSMUSG00000031860 | Pbx4 | -3.37593 | 0.00030 | down |
| ENSMUSG00000108847 | B830042I05Rik | -1.55587 | 0.00030 | down |
| ENSMUSG00000021725 | Parp8 | -1.50758 | 0.00029 | down |
| ENSMUSG00000050605 | Zfp61 | -2.09874 | 0.00029 | down |
| ENSMUSG00000013076 | Amotl1 | -1.74515 | 0.00029 | down |
| ENSMUSG00000103928 | Gm37893 | -1.66185 | 0.00029 | down |
| ENSMUSG00000025978 | Rftn2 | -1.60844 | 0.00029 | down |
| ENSMUSG00000035021 | Baz1a | -1.17084 | 0.00029 | down |
| ENSMUSG00000120850 | - | 3.022463 | 0.00029 | up |
| ENSMUSG00000014444 | Piezo1 | -1.27516 | 0.00029 | down |
| ENSMUSG00000025867 | Cplx2 | -1.48853 | 0.00029 | down |
| ENSMUSG00000029765 | Plxna4 | -1.4962 | 0.00029 | down |
| ENSMUSG00000116835 | Gm49594 | -1.76756 | 0.00028 | down |
| ENSMUSG00000100600 | A230077H06Rik | -3.83055 | 0.00028 | down |
| ENSMUSG00000002250 | Ppard | -1.58589 | 0.00028 | down |
| ENSMUSG00000051149 | Adnp | -5.26489 | 0.00027 | down |
| ENSMUSG00000042369 | Rbm45 | 1.768137 | 0.00027 | up |
| ENSMUSG00000105287 | Gm43577 | -1.63644 | 0.00027 | down |
| ENSMUSG00000116692 | Gm49795 | -2.31886 | 0.00027 | down |
| ENSMUSG00000101462 | Gm3052 | -1.71494 | 0.00027 | down |
| ENSMUSG00000079597 | Cstdc4 | -2.58141 | 0.00027 | down |
| ENSMUSG00000061825 | Ces2c | -1.28312 | 0.00027 | down |
| ENSMUSG00000068227 | Il2rb | -1.27953 | 0.00027 | down |
| ENSMUSG00000078650 | G6pc | -1.09772 | 0.00027 | down |
| ENSMUSG00000052417 | Olfr720 | -3.67738 | 0.00027 | down |
| ENSMUSG00000107191 | Gm43579 | -1.6706 | 0.00026 | down |
| ENSMUSG00000006576 | Slc4a3 | -1.78728 | 0.00026 | down |
| ENSMUSG00000082762 | Gm12366 | -3.33993 | 0.00026 | down |
| ENSMUSG00000117358 | Gm31645 | -1.47465 | 0.00026 | down |
| ENSMUSG00000069804 | Gm10277 | -3.19119 | 0.00025 | down |
| ENSMUSG00000102153 | Gm37474 | -1.99292 | 0.00025 | down |
| ENSMUSG00000028322 | Exosc3 | 1.078645 | 0.00025 | up |
| ENSMUSG00000042099 | Kank3 | -1.07977 | 0.00025 | down |
| ENSMUSG00000030309 | Caprin2 | -1.74413 | 0.00025 | down |
| ENSMUSG00000097224 | Gm26716 | -1.74595 | 0.00025 | down |
| ENSMUSG00000028476 | Reck | -1.68569 | 0.00025 | down |
| ENSMUSG00000037890 | Wdr19 | -1.21043 | 0.00025 | down |
| ENSMUSG00000044906 | 4930503L19Rik | -1.05594 | 0.00024 | down |
| ENSMUSG00000064137 | Rhox8 | -2.30889 | 0.00024 | down |
| ENSMUSG00000095325 | Zfp870 | -1.01037 | 0.00024 | down |
| ENSMUSG00000115431 | Gm3219 | -1.08418 | 0.00024 | down |
| ENSMUSG00000111878 | Gm47777 | -3.82115 | 0.00024 | down |
| ENSMUSG00000110520 | Gm45776 | -3.50916 | 0.00023 | down |
| ENSMUSG00000026023 | Cdk15 | -2.08399 | 0.00023 | down |
| ENSMUSG00000051190 | Olfr1356 | -1.80383 | 0.00023 | down |
| ENSMUSG00000009733 | Tfcp2 | -1.0127 | 0.00023 | down |
| ENSMUSG00000116898 | Gm49785 | -1.94402 | 0.00023 | down |
| ENSMUSG00000019210 | Atp6v1e1 | 1.04914 | 0.00023 | up |
| ENSMUSG00000046962 | Zbtb21 | -1.10118 | 0.00023 | down |
| ENSMUSG00000019997 | Ccn2 | -1.2525 | 0.00022 | down |
| ENSMUSG00000031266 | Gla | 1.285307 | 0.00022 | up |
| ENSMUSG00000023052 | Npff | -1.62474 | 0.00022 | down |
| ENSMUSG00000073415 | Gm10501 | -2.56909 | 0.00022 | down |
| ENSMUSG00000079669 | Gm17396 | -2.69446 | 0.00022 | down |
| ENSMUSG00000032172 | Olfm2 | 3.142605 | 0.00022 | up |
| ENSMUSG00000075284 | Wipf1 | -1.06267 | 0.00022 | down |
| ENSMUSG00000000088 | Cox5a | 1.132371 | 0.00022 | up |
| ENSMUSG00000028059 | Arhgef2 | -1.02355 | 0.00022 | down |
| ENSMUSG00000062456 | Rpl9-ps6 | -4.35543 | 0.00022 | down |
| ENSMUSG00000074813 | Morrbid | -1.7475 | 0.00021 | down |
| ENSMUSG00000044595 | Dnd1 | -1.5079 | 0.00021 | down |
| ENSMUSG00000044197 | Gpr146 | 1.08318 | 0.00021 | up |
| ENSMUSG00000029188 | Slc34a2 | -2.64656 | 0.00021 | down |
| ENSMUSG00000108162 | Gm20589 | -3.44166 | 0.00021 | down |
| ENSMUSG00000054938 | Olfr1346 | -4.44677 | 0.00021 | down |
| ENSMUSG00000031220 | Awat2 | -1.44142 | 0.00021 | down |
| ENSMUSG00000031604 | Msmo1 | 1.683838 | 0.00021 | up |
| ENSMUSG00000026185 | Igfbp5 | -1.13726 | 0.00021 | down |
| ENSMUSG00000073274 | Gm14636 | -1.53871 | 0.00021 | down |
| ENSMUSG00000020458 | Rtn4 | 1.066562 | 0.00020 | up |
| ENSMUSG00000040139 | 9430038I01Rik | -1.05511 | 0.00020 | down |
| ENSMUSG00000097254 | C430042M11Rik | -2.27336 | 0.00020 | down |
| ENSMUSG00000033705 | Stard9 | -1.19888 | 0.00020 | down |
| ENSMUSG00000026941 | Mamdc4 | -1.68822 | 0.00020 | down |
| ENSMUSG00000028370 | Pappa | -2.84113 | 0.00020 | down |
| ENSMUSG00000071456 | 1110002L01Rik | 1.897338 | 0.00020 | up |
| ENSMUSG00000069045 | Ddx3y | -1.68859 | 0.00020 | down |
| ENSMUSG00000110755 | BC049987 | 1.072623 | 0.00020 | up |
| ENSMUSG00000104159 | Gm38099 | -1.7716 | 0.00020 | down |
| ENSMUSG00000039601 | Rcan2 | 1.163512 | 0.00020 | up |
| ENSMUSG00000114836 | Gm18517 | -1.83058 | 0.00019 | down |
| ENSMUSG00000104621 | Gm43185 | -2.58673 | 0.00019 | down |
| ENSMUSG00000089942 | Pira2 | 3.794595 | 0.00019 | up |
| ENSMUSG00000056600 | Olfr90 | -1.35671 | 0.00019 | down |
| ENSMUSG00000024330 | Col11a2 | -1.61058 | 0.00019 | down |
| ENSMUSG00000112542 | Gm47840 | -2.35974 | 0.00019 | down |
| ENSMUSG00000056978 | Hamp2 | -2.57958 | 0.00019 | down |
| ENSMUSG00000042436 | Mfap4 | -1.83155 | 0.00019 | down |
| ENSMUSG00000023087 | Noct | -1.01698 | 0.00019 | down |
| ENSMUSG00000034917 | Tjp3 | -1.01587 | 0.00019 | down |
| ENSMUSG00000091898 | Tnnc1 | -2.23227 | 0.00019 | down |
| ENSMUSG00000035863 | Palm | -1.24064 | 0.00018 | down |
| ENSMUSG00000026604 | Ptpn14 | -1.37862 | 0.00018 | down |
| ENSMUSG00000044938 | Klhl31 | -2.72117 | 0.00018 | down |
| ENSMUSG00000040852 | Plekhh2 | -1.85539 | 0.00018 | down |
| ENSMUSG00000085622 | 3110056K07Rik | -1.35679 | 0.00018 | down |
| ENSMUSG00000113098 | Gm2912 | -2.59956 | 0.00018 | down |
| ENSMUSG00000038692 | Hoxb4 | -1.64782 | 0.00018 | down |
| ENSMUSG00000044937 | Ttc41 | -1.15261 | 0.00018 | down |
| ENSMUSG00000033790 | Tubgcp5 | -1.08462 | 0.00017 | down |
| ENSMUSG00000104149 | Gm37138 | -1.72757 | 0.00017 | down |
| ENSMUSG00000105810 | Gm43435 | -2.63296 | 0.00017 | down |
| ENSMUSG00000075010 | AW112010 | 1.268747 | 0.00017 | up |
| ENSMUSG00000028567 | Txndc12 | 1.042024 | 0.00017 | up |
| ENSMUSG00000021094 | Dhrs7 | 1.189735 | 0.00017 | up |
| ENSMUSG00000113601 | Gm48735 | -3.42346 | 0.00017 | down |
| ENSMUSG00000022623 | Shank3 | -1.13665 | 0.00017 | down |
| ENSMUSG00000041992 | Rapgef5 | -1.0373 | 0.00017 | down |
| ENSMUSG00000118043 | Gm50206 | -2.56327 | 0.00017 | down |
| ENSMUSG00000063506 | Arhgap22 | -3.6172 | 0.00016 | down |
| ENSMUSG00000059429 | Olfr365 | -1.18688 | 0.00016 | down |
| ENSMUSG00000025372 | Baiap2 | -1.76026 | 0.00016 | down |
| ENSMUSG00000025004 | Cyp2c40 | -1.97213 | 0.00016 | down |
| ENSMUSG00000112294 | Gm4129 | 2.768781 | 0.00016 | up |
| ENSMUSG00000026435 | Slc45a3 | -1.03341 | 0.00016 | down |
| ENSMUSG00000100967 | Gm29666 | -1.7887 | 0.00016 | down |
| ENSMUSG00000025537 | Phkg1 | -2.54105 | 0.00016 | down |
| ENSMUSG00000019880 | Rspo3 | -1.27596 | 0.00016 | down |
| ENSMUSG00000024653 | Scgb1a1 | -5.30036 | 0.00016 | down |
| ENSMUSG00000100147 | 1700047M11Rik | -1.9682 | 0.00015 | down |
| ENSMUSG00000120775 | - | -2.28803 | 0.00015 | down |
| ENSMUSG00000038042 | Ptpdc1 | -1.42757 | 0.00015 | down |
| ENSMUSG00000105868 | Gm43766 | -2.33141 | 0.00015 | down |
| ENSMUSG00000104388 | Gm37033 | -2.66911 | 0.00015 | down |
| ENSMUSG00000000605 | Clcn4 | -1.22724 | 0.00015 | down |
| ENSMUSG00000072244 | Trim6 | -2.99677 | 0.00015 | down |
| ENSMUSG00000041974 | Spidr | 2.642853 | 0.00015 | up |
| ENSMUSG00000034758 | Tle6 | -1.56723 | 0.00015 | down |
| ENSMUSG00000031591 | Asah1 | 1.076102 | 0.00015 | up |
| ENSMUSG00000112824 | Gm47917 | -1.3759 | 0.00015 | down |
| ENSMUSG00000092563 | Gm3617 | -8.06541 | 0.00014 | down |
| ENSMUSG00000027082 | Tfpi | -1.06386 | 0.00014 | down |
| ENSMUSG00000029094 | Afap1 | -1.5078 | 0.00014 | down |
| ENSMUSG00000026970 | Rbms1 | -1.35358 | 0.00014 | down |
| ENSMUSG00000030930 | Chst15 | -1.08649 | 0.00014 | down |
| ENSMUSG00000083863 | Gm13341 | -1.79679 | 0.00014 | down |
| ENSMUSG00000115846 | Gm41144 | 4.445538 | 0.00014 | up |
| ENSMUSG00000074240 | Cib3 | -2.11418 | 0.00014 | down |
| ENSMUSG00000086189 | Gm15462 | -2.778 | 0.00014 | down |
| ENSMUSG00000094822 | Olfr243 | -2.93332 | 0.00014 | down |
| ENSMUSG00000119994 | - | -1.27558 | 0.00014 | down |
| ENSMUSG00000062580 | Timm17a | 1.04246 | 0.00014 | up |
| ENSMUSG00000086544 | Chn1os3 | -2.08356 | 0.00013 | down |
| ENSMUSG00000070661 | Rnf186 | 1.570241 | 0.00013 | up |
| ENSMUSG00000021428 | Riok1 | -1.00377 | 0.00013 | down |
| ENSMUSG00000038485 | Socs7 | -1.01169 | 0.00013 | down |
| ENSMUSG00000102591 | Gm38383 | -1.52044 | 0.00013 | down |
| ENSMUSG00000032601 | Prkar2a | -1.15885 | 0.00013 | down |
| ENSMUSG00000046567 | 4930430F08Rik | -1.06017 | 0.00013 | down |
| ENSMUSG00000027165 | Iftap | 1.114055 | 0.00013 | up |
| ENSMUSG00000047050 | Olfr914 | -3.03548 | 0.00013 | down |
| ENSMUSG00000000563 | Atp5pb | 1.060778 | 0.00013 | up |
| ENSMUSG00000021273 | Fdft1 | 1.085306 | 0.00013 | up |
| ENSMUSG00000113684 | Gm48418 | -2.51558 | 0.00012 | down |
| ENSMUSG00000021135 | Slc10a1 | 1.033537 | 0.00012 | up |
| ENSMUSG00000027824 | Vmn2r1 | -1.64101 | 0.00012 | down |
| ENSMUSG00000117780 | Gm3734 | -1.87336 | 0.00012 | down |
| ENSMUSG00000089774 | Slc5a3 | -1.02689 | 0.00012 | down |
| ENSMUSG00000030652 | Coq7 | 1.111258 | 0.00012 | up |
| ENSMUSG00000057098 | Ebf1 | -1.55522 | 0.00012 | down |
| ENSMUSG00000003279 | Dlgap1 | -1.91098 | 0.00012 | down |
| ENSMUSG00000108483 | Gm45184 | -4.8367 | 0.00012 | down |
| ENSMUSG00000039086 | Ss18l1 | -1.14313 | 0.00012 | down |
| ENSMUSG00000024411 | Aqp4 | 1.606244 | 0.00012 | up |
| ENSMUSG00000066538 | Gm6254 | -1.3587 | 0.00012 | down |
| ENSMUSG00000106073 | Gm42892 | -2.04297 | 0.00012 | down |
| ENSMUSG00000047409 | Ctdspl | -1.04501 | 0.00012 | down |
| ENSMUSG00000047420 | Fam180a | -2.40799 | 0.00012 | down |
| ENSMUSG00000113290 | A530058O07Rik | -2.13319 | 0.00012 | down |
| ENSMUSG00000046808 | Atp10d | -1.42049 | 0.00011 | down |
| ENSMUSG00000029553 | Tfec | -1.45556 | 0.00011 | down |
| ENSMUSG00000120473 | - | 1.982761 | 0.00011 | up |
| ENSMUSG00000035595 | Fam174c | 1.249178 | 0.00011 | up |
| ENSMUSG00000057614 | Gnai1 | -1.9542 | 0.00011 | down |
| ENSMUSG00000001918 | Slc1a5 | -1.28291 | 0.00011 | down |
| ENSMUSG00000025384 | Faap100 | -1.06521 | 0.00011 | down |
| ENSMUSG00000031906 | Smpd3 | 2.200312 | 0.00011 | up |
| ENSMUSG00000107050 | C030017G13Rik | -2.68351 | 0.00011 | down |
| ENSMUSG00000115368 | Gm48942 | -1.46966 | 0.00011 | down |
| ENSMUSG00000050270 | Tmem220 | 1.307171 | 0.00011 | up |
| ENSMUSG00000068011 | Mkrn2os | 1.022188 | 0.00011 | up |
| ENSMUSG00000053714 | 4732471J01Rik | -1.31558 | 0.00011 | down |
| ENSMUSG00000031706 | Rfx1 | -1.0844 | 0.00011 | down |
| ENSMUSG00000105454 | Gm43830 | -2.12904 | 0.00011 | down |
| ENSMUSG00000021286 | Zfyve21 | 1.004767 | 0.00010 | up |
| ENSMUSG00000002944 | Cd36 | 1.236297 | 0.00010 | up |
| ENSMUSG00000009654 | Oit3 | -1.13814 | 0.00010 | down |
| ENSMUSG00000026981 | Il1rn | 2.153593 | 0.00010 | up |
| ENSMUSG00000096929 | A330023F24Rik | -1.48961 | 0.00010 | down |
| ENSMUSG00000032264 | Zw10 | -1.09653 | 0.00010 | down |
| ENSMUSG00000090145 | Ugt1a6b | 1.00107 | 0.00010 | up |
| ENSMUSG00000019179 | Mdh2 | 1.145801 | 0.00010 | up |
| ENSMUSG00000032815 | Fanca | -1.81978 | 0.00010 | down |
| ENSMUSG00000021196 | Pfkp | -1.69675 | 0.00010 | down |
| ENSMUSG00000042404 | Dennd4b | -1.0638 | 0.00010 | down |
| ENSMUSG00000043384 | Gprasp1 | -1.23615 | 0.00009 | down |
| ENSMUSG00000036840 | Siah1a | -1.02134 | 0.00009 | down |
| ENSMUSG00000117879 | 2310015A16Rik | 1.377677 | 0.00009 | up |
| ENSMUSG00000060419 | Rps16-ps2 | 1.072119 | 0.00009 | up |
| ENSMUSG00000087470 | A630031M04Rik | -1.49376 | 0.00009 | down |
| ENSMUSG00000044258 | Ctla2a | -1.41615 | 0.00009 | down |
| ENSMUSG00000102428 | Pcdhga12 | -2.0566 | 0.00009 | down |
| ENSMUSG00000031728 | Zfp821 | -1.44529 | 0.00009 | down |
| ENSMUSG00000000560 | Gabra2 | -2.19514 | 0.00009 | down |
| ENSMUSG00000031845 | Bco1 | -1.46551 | 0.00009 | down |
| ENSMUSG00000078234 | Klhdc7a | 1.33311 | 0.00009 | up |
| ENSMUSG00000030287 | Itpr2 | -1.23797 | 0.00009 | down |
| ENSMUSG00000027239 | Mdk | -2.1393 | 0.00009 | down |
| ENSMUSG00000117634 | Gm50069 | -2.31986 | 0.00009 | down |
| ENSMUSG00000052305 | Hbb-bs | -1.30922 | 0.00009 | down |
| ENSMUSG00000018102 | H2bc4 | 1.160562 | 0.00009 | up |
| ENSMUSG00000037211 | Spry1 | -1.34446 | 0.00008 | down |
| ENSMUSG00000120872 | - | -2.63219 | 0.00008 | down |
| ENSMUSG00000058173 | Smco4 | 1.123567 | 0.00008 | up |
| ENSMUSG00000102562 | Gm37694 | -2.96006 | 0.00008 | down |
| ENSMUSG00000102858 | Gm37086 | -1.31105 | 0.00008 | down |
| ENSMUSG00000114407 | Gm48765 | -1.07333 | 0.00008 | down |
| ENSMUSG00000028150 | Rorc | -1.22935 | 0.00008 | down |
| ENSMUSG00000036430 | Tbcc | 1.466191 | 0.00008 | up |
| ENSMUSG00000029993 | Nfu1 | 1.124527 | 0.00008 | up |
| ENSMUSG00000104520 | Gm37336 | -3.41327 | 0.00008 | down |
| ENSMUSG00000109807 | Gm45244 | -2.26045 | 0.00008 | down |
| ENSMUSG00000027514 | Zbp1 | 1.660197 | 0.00008 | up |
| ENSMUSG00000110266 | Gm32742 | -2.60985 | 0.00008 | down |
| ENSMUSG00000021576 | Pdcd6 | 1.036684 | 0.00008 | up |
| ENSMUSG00000030222 | Rerg | -1.56683 | 0.00008 | down |
| ENSMUSG00000029752 | Asns | 2.691235 | 0.00008 | up |
| ENSMUSG00000007836 | Hnrnpa0 | 1.168017 | 0.00008 | up |
| ENSMUSG00000054142 | Vmn1r236 | -3.1937 | 0.00008 | down |
| ENSMUSG00000111544 | 4930534H03Rik | -2.0162 | 0.00008 | down |
| ENSMUSG00000036533 | Cdc42ep3 | -2.19469 | 0.00008 | down |
| ENSMUSG00000118215 | Vmn1r55 | -1.81891 | 0.00008 | down |
| ENSMUSG00000114369 | Gm41077 | 3.923193 | 0.00008 | up |
| ENSMUSG00000031450 | Grk1 | -2.52617 | 0.00008 | down |
| ENSMUSG00000028403 | Zdhhc21 | -1.10335 | 0.00008 | down |
| ENSMUSG00000012017 | Scarf2 | -1.29769 | 0.00007 | down |
| ENSMUSG00000114114 | Gm48499 | -2.77442 | 0.00007 | down |
| ENSMUSG00000016541 | Atxn10 | 1.012643 | 0.00007 | up |
| ENSMUSG00000090077 | Lime1 | -1.1432 | 0.00007 | down |
| ENSMUSG00000036136 | Fam110c | 2.142672 | 0.00007 | up |
| ENSMUSG00000029179 | Zcchc4 | -1.08746 | 0.00007 | down |
| ENSMUSG00000045790 | Ccdc149 | 1.536683 | 0.00007 | up |
| ENSMUSG00000097318 | 1700007L15Rik | 1.996979 | 0.00007 | up |
| ENSMUSG00000106863 | Gm42109 | -2.82591 | 0.00007 | down |
| ENSMUSG00000036246 | Gmip | -1.32356 | 0.00007 | down |
| ENSMUSG00000024029 | Tff3 | 2.145237 | 0.00007 | up |
| ENSMUSG00000087516 | Tbx3os1 | -1.32115 | 0.00007 | down |
| ENSMUSG00000028840 | Zfp593 | 1.162402 | 0.00007 | up |
| ENSMUSG00000031805 | Jak3 | 1.365348 | 0.00007 | up |
| ENSMUSG00000000317 | Bcl6b | -1.63642 | 0.00007 | down |
| ENSMUSG00000039646 | Vasn | 1.5131 | 0.00007 | up |
| ENSMUSG00000028713 | Cyp4b1 | -1.35821 | 0.00007 | down |
| ENSMUSG00000024902 | Mrpl11 | 1.003268 | 0.00007 | up |
| ENSMUSG00000019577 | Pdk4 | -1.55532 | 0.00007 | down |
| ENSMUSG00000033545 | Znrf1 | -1.04288 | 0.00007 | down |
| ENSMUSG00000031556 | Tm2d2 | 1.091838 | 0.00007 | up |
| ENSMUSG00000087120 | Gm12279 | -1.58616 | 0.00006 | down |
| ENSMUSG00000115124 | Gm49201 | -1.09547 | 0.00006 | down |
| ENSMUSG00000085774 | Gm13055 | -4.40319 | 0.00006 | down |
| ENSMUSG00000044709 | Gemin7 | 1.047902 | 0.00006 | up |
| ENSMUSG00000060441 | Trim5 | -1.5343 | 0.00006 | down |
| ENSMUSG00000078440 | Dohh | 1.178518 | 0.00006 | up |
| ENSMUSG00000095928 | Olfr204 | -1.44545 | 0.00006 | down |
| ENSMUSG00000112505 | Gm48610 | -3.06209 | 0.00006 | down |
| ENSMUSG00000013033 | Adgrl1 | -1.22842 | 0.00006 | down |
| ENSMUSG00000038895 | Zfp653 | -1.1774 | 0.00006 | down |
| ENSMUSG00000038286 | Bphl | 1.019461 | 0.00006 | up |
| ENSMUSG00000076617 | Ighm | -1.33947 | 0.00006 | down |
| ENSMUSG00000043241 | Upf2 | -1.31621 | 0.00006 | down |
| ENSMUSG00000053158 | Fes | -1.14818 | 0.00006 | down |
| ENSMUSG00000022504 | Ciita | -1.90637 | 0.00006 | down |
| ENSMUSG00000040712 | Camta2 | -1.03065 | 0.00006 | down |
| ENSMUSG00000033439 | Trmt13 | -1.03699 | 0.00006 | down |
| ENSMUSG00000112739 | Gm20597 | -3.63249 | 0.00006 | down |
| ENSMUSG00000036054 | Sugp2 | -1.05618 | 0.00006 | down |
| ENSMUSG00000102326 | Gm37788 | -1.74986 | 0.00006 | down |
| ENSMUSG00000035112 | Wnk4 | -1.84318 | 0.00006 | down |
| ENSMUSG00000032786 | Alas1 | 1.223081 | 0.00006 | up |
| ENSMUSG00000107225 | Gm43637 | -1.42955 | 0.00006 | down |
| ENSMUSG00000029591 | Ung | 1.292385 | 0.00006 | up |
| ENSMUSG00000058656 | Samd12 | -3.21899 | 0.00006 | down |
| ENSMUSG00000068950 | Olfr338 | -1.38232 | 0.00006 | down |
| ENSMUSG00000025003 | Cyp2c39 | 2.239951 | 0.00006 | up |
| ENSMUSG00000021213 | Akr1c13 | 1.142216 | 0.00006 | up |
| ENSMUSG00000091542 | Gm17167 | -1.39918 | 0.00006 | down |
| ENSMUSG00000090086 | AI480526 | -1.16856 | 0.00005 | down |
| ENSMUSG00000059040 | Eno1b | -5.4806 | 0.00005 | down |
| ENSMUSG00000118607 | Gm7592 | -1.82142 | 0.00005 | down |
| ENSMUSG00000095975 | Cphx1 | -1.41948 | 0.00005 | down |
| ENSMUSG00000121473 | Adh6-ps1 | -1.82644 | 0.00005 | down |
| ENSMUSG00000059146 | Ntrk3 | -2.08748 | 0.00005 | down |
| ENSMUSG00000108216 | Gm44153 | -2.33042 | 0.00005 | down |
| ENSMUSG00000075307 | Klhl41 | -2.96321 | 0.00005 | down |
| ENSMUSG00000041220 | Elovl6 | -1.78324 | 0.00005 | down |
| ENSMUSG00000075602 | Ly6a | -1.0402 | 0.00005 | down |
| ENSMUSG00000045854 | Lyrm2 | 1.173628 | 0.00005 | up |
| ENSMUSG00000110519 | Olfr839 | -1.26263 | 0.00005 | down |
| ENSMUSG00000108884 | Gm45792 | -1.70846 | 0.00005 | down |
| ENSMUSG00000021701 | Plk2 | -1.18185 | 0.00005 | down |
| ENSMUSG00000092526 | Gm17907 | -2.28317 | 0.00005 | down |
| ENSMUSG00000042500 | Ago4 | -1.15992 | 0.00005 | down |
| ENSMUSG00000040624 | Plekhg1 | -1.37683 | 0.00005 | down |
| ENSMUSG00000057375 | Yipf1 | 1.246598 | 0.00005 | up |
| ENSMUSG00000108633 | Gm44694 | -4.36881 | 0.00005 | down |
| ENSMUSG00000068732 | Tmem167b | 1.112697 | 0.00005 | up |
| ENSMUSG00000015575 | Atp6v0e | 1.140557 | 0.00005 | up |
| ENSMUSG00000041189 | Chrnb1 | -1.6933 | 0.00005 | down |
| ENSMUSG00000067951 | Vmn1r227 | -1.78452 | 0.00005 | down |
| ENSMUSG00000052005 | Gm9864 | -1.87231 | 0.00005 | down |
| ENSMUSG00000116021 | Gm49474 | -2.16796 | 0.00005 | down |
| ENSMUSG00000011263 | Exoc3l2 | -1.08832 | 0.00005 | down |
| ENSMUSG00000052560 | Cpne8 | 1.569819 | 0.00005 | up |
| ENSMUSG00000112096 | A430103D13Rik | -1.91328 | 0.00005 | down |
| ENSMUSG00000075543 | Urad | 1.115877 | 0.00005 | up |
| ENSMUSG00000060216 | Arrb2 | -1.03249 | 0.00005 | down |
| ENSMUSG00000025902 | Sox17 | -1.37858 | 0.00005 | down |
| ENSMUSG00000031661 | Nkd1 | -1.06973 | 0.00004 | down |
| ENSMUSG00000114452 | A530001N23Rik | -3.60057 | 0.00004 | down |
| ENSMUSG00000056313 | Tcim | -1.04796 | 0.00004 | down |
| ENSMUSG00000049721 | Gal3st1 | 1.964595 | 0.00004 | up |
| ENSMUSG00000020844 | Nxn | -1.53291 | 0.00004 | down |
| ENSMUSG00000106446 | Gm42970 | -2.29753 | 0.00004 | down |
| ENSMUSG00000028161 | Ppp3ca | -1.3083 | 0.00004 | down |
| ENSMUSG00000033713 | Foxn3 | -1.13878 | 0.00004 | down |
| ENSMUSG00000020134 | Peli1 | -1.37983 | 0.00004 | down |
| ENSMUSG00000030595 | Nfkbib | 1.117351 | 0.00004 | up |
| ENSMUSG00000109881 | Gm45507 | -7.7048 | 0.00004 | down |
| ENSMUSG00000090655 | Vmn2r120 | -2.15358 | 0.00004 | down |
| ENSMUSG00000052794 | 1700030K09Rik | -1.17923 | 0.00004 | down |
| ENSMUSG00000013833 | Med16 | 1.181038 | 0.00004 | up |
| ENSMUSG00000027613 | Eif6 | 1.220659 | 0.00004 | up |
| ENSMUSG00000111274 | Gm47409 | -3.36056 | 0.00004 | down |
| ENSMUSG00000085786 | Gm15987 | -1.44327 | 0.00004 | down |
| ENSMUSG00000040118 | Cacna2d1 | -1.6731 | 0.00004 | down |
| ENSMUSG00000022961 | Son | -1.20124 | 0.00004 | down |
| ENSMUSG00000092274 | Neat1 | -1.84003 | 0.00004 | down |
| ENSMUSG00000120012 | - | -1.50463 | 0.00004 | down |
| ENSMUSG00000073758 | Sh3d21 | -1.56642 | 0.00004 | down |
| ENSMUSG00000049303 | Syt12 | 1.901873 | 0.00004 | up |
| ENSMUSG00000022218 | Tgm1 | 1.306416 | 0.00004 | up |
| ENSMUSG00000068923 | Syt11 | -1.42519 | 0.00004 | down |
| ENSMUSG00000030612 | Mrpl46 | 1.207046 | 0.00004 | up |
| ENSMUSG00000032265 | Tent5a | -1.54747 | 0.00004 | down |
| ENSMUSG00000003228 | Grk5 | -1.49157 | 0.00004 | down |
| ENSMUSG00000031447 | Lamp1 | 1.189002 | 0.00004 | up |
| ENSMUSG00000058258 | Idi1 | 1.804166 | 0.00004 | up |
| ENSMUSG00000007944 | Ttc9b | -5.30042 | 0.00004 | down |
| ENSMUSG00000075204 | Olfr1039 | -2.68699 | 0.00004 | down |
| ENSMUSG00000020829 | Slc46a1 | 1.046218 | 0.00004 | up |
| ENSMUSG00000021917 | Spcs1 | 1.685942 | 0.00004 | up |
| ENSMUSG00000018909 | Arrb1 | -1.00349 | 0.00004 | down |
| ENSMUSG00000113161 | Gm47457 | -1.57891 | 0.00004 | down |
| ENSMUSG00000084790 | Gm15879 | -2.62438 | 0.00004 | down |
| ENSMUSG00000089715 | Cbx6 | -1.18153 | 0.00004 | down |
| ENSMUSG00000032554 | Trf | 1.008614 | 0.00004 | up |
| ENSMUSG00000001741 | Il16 | -1.29717 | 0.00004 | down |
| ENSMUSG00000003762 | Coq8b | 1.223134 | 0.00004 | up |
| ENSMUSG00000003352 | Cacnb3 | -1.65368 | 0.00003 | down |
| ENSMUSG00000085404 | Gm12909 | -1.60618 | 0.00003 | down |
| ENSMUSG00000029649 | Pomp | 1.007168 | 0.00003 | up |
| ENSMUSG00000041187 | Prkd2 | -1.27224 | 0.00003 | down |
| ENSMUSG00000040415 | Dtx3 | -1.29975 | 0.00003 | down |
| ENSMUSG00000021149 | Gtpbp4 | -1.43356 | 0.00003 | down |
| ENSMUSG00000098332 | Pigbos1 | 1.031822 | 0.00003 | up |
| ENSMUSG00000008206 | Cers4 | -1.52872 | 0.00003 | down |
| ENSMUSG00000028444 | Cntfr | -1.4683 | 0.00003 | down |
| ENSMUSG00000041567 | Serpina12 | -1.55192 | 0.00003 | down |
| ENSMUSG00000022194 | Pabpn1 | -1.98142 | 0.00003 | down |
| ENSMUSG00000094856 | Gm21962 | -1.67731 | 0.00003 | down |
| ENSMUSG00000019494 | Cops6 | 1.309195 | 0.00003 | up |
| ENSMUSG00000045282 | Tmem86b | 1.066213 | 0.00003 | up |
| ENSMUSG00000003992 | Ssbp2 | -1.96397 | 0.00003 | down |
| ENSMUSG00000072572 | Slc39a2 | -1.82427 | 0.00003 | down |
| ENSMUSG00000078624 | Olfr613 | -1.25463 | 0.00003 | down |
| ENSMUSG00000072770 | Acrbp | -1.52774 | 0.00003 | down |
| ENSMUSG00000035258 | Abi3bp | -1.59249 | 0.00003 | down |
| ENSMUSG00000038722 | Bud31 | 1.125857 | 0.00003 | up |
| ENSMUSG00000034435 | Tmem30b | 1.034278 | 0.00003 | up |
| ENSMUSG00000040121 | Rep15 | -2.59093 | 0.00003 | down |
| ENSMUSG00000091509 | Gm17066 | -1.958 | 0.00003 | down |
| ENSMUSG00000112366 | C730027H18Rik | -1.13537 | 0.00003 | down |
| ENSMUSG00000047767 | Atg16l2 | -1.31871 | 0.00003 | down |
| ENSMUSG00000057068 | Fam47e | 1.54745 | 0.00003 | up |
| ENSMUSG00000121179 | - | -1.18323 | 0.00003 | down |
| ENSMUSG00000105315 | Gm18635 | -1.47819 | 0.00003 | down |
| ENSMUSG00000094520 | Olfr635 | -1.81555 | 0.00003 | down |
| ENSMUSG00000085826 | Gm15638 | -1.9268 | 0.00003 | down |
| ENSMUSG00000120725 | - | -1.78926 | 0.00003 | down |
| ENSMUSG00000070315 | 4930581F22Rik | -1.02962 | 0.00003 | down |
| ENSMUSG00000032377 | Plscr4 | -1.49503 | 0.00003 | down |
| ENSMUSG00000105855 | Gm42681 | -2.12872 | 0.00003 | down |
| ENSMUSG00000084960 | B430010I23Rik | -3.00194 | 0.00003 | down |
| ENSMUSG00000036721 | Zscan12 | -1.05108 | 0.00003 | down |
| ENSMUSG00000030161 | Gabarapl1 | 1.197523 | 0.00003 | up |
| ENSMUSG00000038074 | Fkbp14 | -1.1608 | 0.00003 | down |
| ENSMUSG00000091382 | Vmn1r18 | -1.55828 | 0.00003 | down |
| ENSMUSG00000057933 | Gsta2 | -1.37428 | 0.00003 | down |
| ENSMUSG00000115919 | Gm31583 | -1.49843 | 0.00003 | down |
| ENSMUSG00000043801 | Oaz1-ps | 1.811694 | 0.00003 | up |
| ENSMUSG00000021210 | Akr1c6 | 1.150406 | 0.00003 | up |
| ENSMUSG00000054435 | Gimap4 | -1.01198 | 0.00003 | down |
| ENSMUSG00000031380 | Vegfd | -1.5613 | 0.00003 | down |
| ENSMUSG00000113019 | Gm47467 | -1.06798 | 0.00003 | down |
| ENSMUSG00000042293 | Gm5617 | 1.222702 | 0.00003 | up |
| ENSMUSG00000121242 | - | 2.425095 | 0.00002 | up |
| ENSMUSG00000059974 | Ntm | -1.83859 | 0.00002 | down |
| ENSMUSG00000024049 | Myom1 | -2.05943 | 0.00002 | down |
| ENSMUSG00000088252 | Snord13 | -5.79383 | 0.00002 | down |
| ENSMUSG00000039943 | Plcb4 | -1.62741 | 0.00002 | down |
| ENSMUSG00000106706 | C530043K16Rik | -2.41512 | 0.00002 | down |
| ENSMUSG00000034974 | Dapk3 | 1.108498 | 0.00002 | up |
| ENSMUSG00000079363 | Gbp4 | -1.53827 | 0.00002 | down |
| ENSMUSG00000114138 | Gm36423 | -3.44052 | 0.00002 | down |
| ENSMUSG00000041650 | Pcca | 1.079758 | 0.00002 | up |
| ENSMUSG00000120336 | - | -2.51919 | 0.00002 | down |
| ENSMUSG00000036718 | Micall2 | -1.42741 | 0.00002 | down |
| ENSMUSG00000042599 | Kdm7a | -1.44622 | 0.00002 | down |
| ENSMUSG00000020018 | Snrpf | 1.150302 | 0.00002 | up |
| ENSMUSG00000029426 | Scarb2 | 1.170954 | 0.00002 | up |
| ENSMUSG00000095959 | Gm10845 | -1.81994 | 0.00002 | down |
| ENSMUSG00000042895 | Abra | -4.60865 | 0.00002 | down |
| ENSMUSG00000068245 | Phf11d | -1.3436 | 0.00002 | down |
| ENSMUSG00000024109 | Nrxn1 | -1.61908 | 0.00002 | down |
| ENSMUSG00000090778 | Gm3235 | -2.11835 | 0.00002 | down |
| ENSMUSG00000068856 | Sf3b4 | 1.076737 | 0.00002 | up |
| ENSMUSG00000072893 | 4933439C10Rik | -1.2477 | 0.00002 | down |
| ENSMUSG00000029298 | Gbp9 | -1.13952 | 0.00002 | down |
| ENSMUSG00000029442 | Wdr66 | -1.57479 | 0.00002 | down |
| ENSMUSG00000032737 | Inppl1 | -1.01745 | 0.00002 | down |
| ENSMUSG00000026411 | Tmem9 | 1.177946 | 0.00002 | up |
| ENSMUSG00000051111 | Sv2c | -2.88472 | 0.00002 | down |
| ENSMUSG00000106024 | A530083M17Rik | -3.83153 | 0.00002 | down |
| ENSMUSG00000052906 | Ubxn8 | 1.000167 | 0.00002 | up |
| ENSMUSG00000030468 | Siglecg | -2.49075 | 0.00002 | down |
| ENSMUSG00000038736 | Nudcd1 | -1.17064 | 0.00002 | down |
| ENSMUSG00000079003 | Samd1 | -1.2506 | 0.00002 | down |
| ENSMUSG00000054702 | Ap1s3 | -2.25477 | 0.00002 | down |
| ENSMUSG00000035836 | Ugt2b1 | -1.04425 | 0.00002 | down |
| ENSMUSG00000053477 | Tcf4 | -1.17206 | 0.00002 | down |
| ENSMUSG00000026162 | Nhej1 | -1.5729 | 0.00002 | down |
| ENSMUSG00000020423 | Btg2 | 1.909345 | 0.00002 | up |
| ENSMUSG00000005054 | Cstb | 1.41352 | 0.00002 | up |
| ENSMUSG00000048620 | Olfr1336 | -2.07348 | 0.00002 | down |
| ENSMUSG00000020171 | Yeats4 | 1.151378 | 0.00002 | up |
| ENSMUSG00000006307 | Kmt2b | -1.04065 | 0.00002 | down |
| ENSMUSG00000019232 | Etnppl | -1.22309 | 0.00002 | down |
| ENSMUSG00000023232 | Serinc2 | 1.634569 | 0.00002 | up |
| ENSMUSG00000042138 | Msantd2 | -1.07141 | 0.00002 | down |
| ENSMUSG00000029038 | Ssu72 | 1.305046 | 0.00002 | up |
| ENSMUSG00000027318 | Adam33 | -1.76042 | 0.00002 | down |
| ENSMUSG00000021068 | Nin | -1.54068 | 0.00002 | down |
| ENSMUSG00000037007 | Zfp113 | -1.32533 | 0.00002 | down |
| ENSMUSG00000104291 | A130071D04Rik | -1.87275 | 0.00002 | down |
| ENSMUSG00000029192 | Tbc1d14 | -1.18269 | 0.00002 | down |
| ENSMUSG00000103432 | 6720464F23Rik | -2.90681 | 0.00002 | down |
| ENSMUSG00000018001 | Cyth3 | -1.05051 | 0.00002 | down |
| ENSMUSG00000094145 | Vmn2r20 | -2.35321 | 0.00002 | down |
| ENSMUSG00000040283 | Btnl9 | -1.15652 | 0.00002 | down |
| ENSMUSG00000019762 | Iyd | 1.167468 | 0.00002 | up |
| ENSMUSG00000117098 | Gm49909 | 1.703192 | 0.00002 | up |
| ENSMUSG00000040260 | Daam2 | -1.35554 | 0.00002 | down |
| ENSMUSG00000021969 | Zdhhc20 | -1.24282 | 0.00002 | down |
| ENSMUSG00000112255 | Gm47594 | -2.64604 | 0.00002 | down |
| ENSMUSG00000035585 | Tsen34 | 1.301421 | 0.00002 | up |
| ENSMUSG00000021537 | Cetn3 | 1.192392 | 0.00002 | up |
| ENSMUSG00000104721 | Gm42696 | -2.82044 | 0.00002 | down |
| ENSMUSG00000034258 | Flvcr2 | 1.78955 | 0.00002 | up |
| ENSMUSG00000106040 | Cyp3a63-ps | -3.37734 | 0.00002 | down |
| ENSMUSG00000021211 | Akr1c12 | 1.018472 | 0.00002 | up |
| ENSMUSG00000086784 | Isoc2a | 1.015512 | 0.00002 | up |
| ENSMUSG00000095937 | Gm12671 | 1.162288 | 0.00002 | up |
| ENSMUSG00000032932 | Hspa13 | 1.354004 | 0.00002 | up |
| ENSMUSG00000015647 | Lama5 | -1.26132 | 0.00002 | down |
| ENSMUSG00000026272 | Agxt | 1.008565 | 0.00002 | up |
| ENSMUSG00000021270 | Hsp90aa1 | -1.28651 | 0.00002 | down |
| ENSMUSG00000041479 | Syt15 | -1.88285 | 0.00002 | down |
| ENSMUSG00000030281 | Il17rc | 1.209199 | 0.00002 | up |
| ENSMUSG00000112580 | Gm47673 | -1.55706 | 0.00002 | down |
| ENSMUSG00000064899 | Snord118 | -4.12247 | 0.00001 | down |
| ENSMUSG00000118382 | Gm8373 | -3.1632 | 0.00001 | down |
| ENSMUSG00000118017 | Gm29966 | -3.00874 | 0.00001 | down |
| ENSMUSG00000025486 | Sirt3 | -1.95725 | 0.00001 | down |
| ENSMUSG00000039804 | Ncoa5 | -1.12156 | 0.00001 | down |
| ENSMUSG00000071637 | Cebpd | 1.735721 | 0.00001 | up |
| ENSMUSG00000090236 | Car15 | -2.54871 | 0.00001 | down |
| ENSMUSG00000017286 | Glod4 | 1.253095 | 0.00001 | up |
| ENSMUSG00000027405 | Nop56 | -1.0571 | 0.00001 | down |
| ENSMUSG00000017390 | Aldoc | 1.755351 | 0.00001 | up |
| ENSMUSG00000057193 | Slc44a2 | -1.03325 | 0.00001 | down |
| ENSMUSG00000111045 | Gm47598 | -3.11707 | 0.00001 | down |
| ENSMUSG00000073490 | Ifi207 | -1.22152 | 0.00001 | down |
| ENSMUSG00000097911 | Gm26691 | -2.2441 | 0.00001 | down |
| ENSMUSG00000109089 | 4833411C07Rik | -1.40273 | 0.00001 | down |
| ENSMUSG00000049929 | Lpar4 | -3.28516 | 0.00001 | down |
| ENSMUSG00000024981 | Acsl5 | 1.107389 | 0.00001 | up |
| ENSMUSG00000025355 | Mmp19 | 1.141255 | 0.00001 | up |
| ENSMUSG00000047867 | Gimap6 | -1.01237 | 0.00001 | down |
| ENSMUSG00000032449 | Slc25a36 | -1.22269 | 0.00001 | down |
| ENSMUSG00000024620 | Pdgfrb | -1.06861 | 0.00001 | down |
| ENSMUSG00000032398 | Snapc5 | 1.17582 | 0.00001 | up |
| ENSMUSG00000020546 | Stxbp4 | -1.6162 | 0.00001 | down |
| ENSMUSG00000024462 | Gabbr1 | -1.31429 | 0.00001 | down |
| ENSMUSG00000024424 | Ttc39c | -1.04608 | 0.00001 | down |
| ENSMUSG00000071711 | Mpst | 1.051862 | 0.00001 | up |
| ENSMUSG00000031532 | Saraf | 1.029767 | 0.00001 | up |
| ENSMUSG00000037754 | Ppp1r16b | -1.18823 | 0.00001 | down |
| ENSMUSG00000027078 | Ube2l6 | 1.062679 | 0.00001 | up |
| ENSMUSG00000021509 | Slc25a48 | 1.059781 | 0.00001 | up |
| ENSMUSG00000040033 | Stat2 | -1.08803 | 0.00001 | down |
| ENSMUSG00000054404 | Slfn5 | -1.28558 | 0.00001 | down |
| ENSMUSG00000034708 | Grn | 1.184051 | 0.00001 | up |
| ENSMUSG00000032399 | Rpl4 | 1.047103 | 0.00001 | up |
| ENSMUSG00000006395 | Hyi | -1.19229 | 0.00001 | down |
| ENSMUSG00000118506 | Cfap141 | -1.99942 | 0.00001 | down |
| ENSMUSG00000031153 | Gripap1 | -1.14863 | 0.00001 | down |
| ENSMUSG00000031841 | Cdh13 | -1.63069 | 0.00001 | down |
| ENSMUSG00000045948 | Mrps12 | 1.075883 | 0.00001 | up |
| ENSMUSG00000047793 | Sned1 | -1.01012 | 0.00001 | down |
| ENSMUSG00000106408 | Gm43321 | -1.58491 | 0.00001 | down |
| ENSMUSG00000063856 | Gpx1 | 1.37407 | 0.00001 | up |
| ENSMUSG00000019487 | Trip10 | -1.12343 | 0.00001 | down |
| ENSMUSG00000113523 | Gm48366 | -1.89503 | 0.00001 | down |
| ENSMUSG00000047492 | Inhbe | 1.181291 | 0.00001 | up |
| ENSMUSG00000093485 | Gm20708 | -1.57675 | 0.00001 | down |
| ENSMUSG00000035476 | Tab3 | -1.16809 | 0.00001 | down |
| ENSMUSG00000098055 | Gm26947 | -2.23345 | 0.00001 | down |
| ENSMUSG00000026956 | Uap1l1 | 1.401762 | 0.00001 | up |
| ENSMUSG00000104211 | Gm37985 | -1.81272 | 0.00001 | down |
| ENSMUSG00000001569 | Nom1 | -1.03417 | 0.00001 | down |
| ENSMUSG00000004394 | Tmed4 | 1.019377 | 0.00001 | up |
| ENSMUSG00000020889 | Nr1d1 | 2.676897 | 0.00001 | up |
| ENSMUSG00000095041 | - | -1.10034 | 0.00001 | down |
| ENSMUSG00000052512 | Nav2 | -1.42249 | 0.00001 | down |
| ENSMUSG00000114148 | Gm47701 | -2.9762 | 0.00001 | down |
| ENSMUSG00000046598 | Bdh1 | 1.132734 | 0.00001 | up |
| ENSMUSG00000120390 | - | -1.0129 | 0.00001 | down |
| ENSMUSG00000087412 | Gm15501 | 2.099997 | 0.00001 | up |
| ENSMUSG00000029648 | Flt1 | -1.3898 | 0.00001 | down |
| ENSMUSG00000004187 | Kifc2 | -1.31976 | 0.00001 | down |
| ENSMUSG00000026821 | Ralgds | -1.45951 | 0.00001 | down |
| ENSMUSG00000097415 | AU020206 | -1.33825 | 0.00001 | down |
| ENSMUSG00000056917 | Sipa1 | -1.16566 | 0.00001 | down |
| ENSMUSG00000026574 | Dpt | -1.49341 | 0.00001 | down |
| ENSMUSG00000056268 | Dennd1b | -1.25022 | 0.00001 | down |
| ENSMUSG00000028601 | Echdc2 | -1.00262 | 0.00001 | down |
| ENSMUSG00000081406 | Rps6-ps4 | 1.952195 | 0.00001 | up |
| ENSMUSG00000121503 | H2-K2 | -1.3817 | 0.00001 | down |
| ENSMUSG00000118155 | Gm50136 | -2.47495 | 0.00001 | down |
| ENSMUSG00000086844 | B230206H07Rik | -1.78646 | 0.00001 | down |
| ENSMUSG00000037071 | Scd1 | -1.26773 | 0.00001 | down |
| ENSMUSG00000056035 | Cyp3a11 | -1.25313 | 0.00001 | down |
| ENSMUSG00000096822 | Olfr344 | -1.73937 | 0.00001 | down |
| ENSMUSG00000097462 | 9530026P05Rik | -3.26478 | 0.00001 | down |
| ENSMUSG00000034591 | Slc41a2 | 2.259274 | 0.00001 | up |
| ENSMUSG00000002668 | Dennd1c | -1.81491 | 0.00001 | down |
| ENSMUSG00000047180 | Neurl3 | -1.16474 | 0.00001 | down |
| ENSMUSG00000075081 | Olfr1247 | -1.38542 | 0.00001 | down |
| ENSMUSG00000028982 | Slc25a33 | 1.071643 | 0.00001 | up |
| ENSMUSG00000031925 | Maml2 | -1.89746 | 0.00001 | down |
| ENSMUSG00000078964 | Ces1b | -1.17797 | 0.00001 | down |
| ENSMUSG00000014778 | Fhod1 | -1.14335 | 0.00001 | down |
| ENSMUSG00000069917 | Hba-a2 | -1.35309 | 0.00001 | down |
| ENSMUSG00000015247 | Nipsnap3b | 1.018482 | 0.00001 | up |
| ENSMUSG00000106636 | Gm43813 | -2.59128 | 0.00001 | down |
| ENSMUSG00000062933 | Gm10123 | 1.566241 | 0.00001 | up |
| ENSMUSG00000024430 | Cabyr | -1.61414 | 0.00001 | down |
| ENSMUSG00000040481 | Bptf | -1.41859 | 0.00001 | down |
| ENSMUSG00000005533 | Igf1r | -1.54368 | 0.00001 | down |
| ENSMUSG00000024747 | Aldh1a7 | 1.112355 | 0.00001 | up |
| ENSMUSG00000040936 | Ulk4 | -4.95158 | 0.00001 | down |
| ENSMUSG00000086825 | Gm15675 | -1.34232 | 0.00001 | down |
| ENSMUSG00000041654 | Slc39a11 | 1.449368 | 0.00001 | up |
| ENSMUSG00000104394 | Gm37254 | -1.73258 | 0.00001 | down |
| ENSMUSG00000117148 | Vmn1r229 | -1.30206 | 0.00001 | down |
| ENSMUSG00000046338 | Gpat2 | -1.81981 | 0.00001 | down |
| ENSMUSG00000107994 | D830050J10Rik | 2.096709 | 0.00001 | up |
| ENSMUSG00000119972 | - | -1.80766 | 0.00001 | down |
| ENSMUSG00000041268 | Dmxl2 | -1.03182 | 0.00001 | down |
| ENSMUSG00000048280 | Zfp738 | -1.62934 | 0.00001 | down |
| ENSMUSG00000076609 | Igkc | -1.13237 | 0.00001 | down |
| ENSMUSG00000121083 | - | -1.56675 | 0.00001 | down |
| ENSMUSG00000027942 | 4933434E20Rik | 1.094792 | 0.00001 | up |
| ENSMUSG00000028760 | Eif4g3 | -1.39587 | 0.00001 | down |
| ENSMUSG00000038342 | Mlxip | -1.30876 | 0.00001 | down |
| ENSMUSG00000046402 | Rbp1 | 1.200643 | 0.00001 | up |
| ENSMUSG00000029610 | Aimp2 | 1.508653 | 0.00001 | up |
| ENSMUSG00000000301 | Pemt | 1.135482 | 0.00001 | up |
| ENSMUSG00000026785 | Pkn3 | -1.94771 | 0.00001 | down |
| ENSMUSG00000112557 | Gm47626 | -1.51344 | 0.00001 | down |
| ENSMUSG00000027848 | Olfml3 | -1.12939 | 0.00001 | down |
| ENSMUSG00000059729 | Olfr1385 | -1.45885 | 0.00001 | down |
| ENSMUSG00000025791 | Pgm1 | 1.014583 | 0.00001 | up |
| ENSMUSG00000066724 | Gm10175 | 1.137141 | 0.00001 | up |
| ENSMUSG00000059540 | Tcea2 | -2.06221 | 0.00001 | down |
| ENSMUSG00000069972 | Rps13-ps2 | 1.090423 | 0.00001 | up |
| ENSMUSG00000061894 | Zscan20 | -1.17814 | 0.00001 | down |
| ENSMUSG00000040128 | Pnrc1 | -1.16884 | 0.00001 | down |
| ENSMUSG00000053985 | Zfp14 | -1.65545 | 0.00001 | down |
| ENSMUSG00000103672 | Gm37621 | -1.85742 | 0.00001 | down |
| ENSMUSG00000080921 | Rpl38-ps2 | -9.17482 | 0.00001 | down |
| ENSMUSG00000028199 | Cryz | 1.30704 | 0.00001 | up |
| ENSMUSG00000098014 | Gm26967 | -1.51015 | 0.00001 | down |
| ENSMUSG00000078713 | Tomm5 | 1.005112 | 0.00001 | up |
| ENSMUSG00000044951 | Mylk4 | -2.7175 | 0.00001 | down |
| ENSMUSG00000021687 | Scamp1 | 1.038275 | 0.00001 | up |
| ENSMUSG00000110384 | Gm45301 | -5.17094 | 0.00001 | down |
| ENSMUSG00000059355 | Wdr83os | 1.204826 | 0.00001 | up |
| ENSMUSG00000019791 | Hint3 | 1.123976 | 0.00001 | up |
| ENSMUSG00000003604 | Aven | 1.20623 | 0.00001 | up |
| ENSMUSG00000097048 | 1600020E01Rik | -1.38275 | 0.00001 | down |
| ENSMUSG00000121426 | - | -1.52891 | 0.00001 | down |
| ENSMUSG00000010608 | Rbm25 | -1.04222 | 0.00001 | down |
| ENSMUSG00000101609 | Kcnq1ot1 | -1.63626 | 0.00001 | down |
| ENSMUSG00000040681 | Hmgn1 | 1.191629 | 0.00001 | up |
| ENSMUSG00000033209 | Ttc28 | -1.66967 | 0.00001 | down |
| ENSMUSG00000031245 | Hmgn5 | -1.08968 | 0.00001 | down |
| ENSMUSG00000113069 | Gm48541 | -1.52714 | 0.00001 | down |
| ENSMUSG00000020469 | Myl7 | -6.27101 | 0.00001 | down |
| ENSMUSG00000027931 | Npr1 | -1.27792 | <0.00001 | down |
| ENSMUSG00000082127 | Gm13577 | -4.34227 | <0.00001 | down |
| ENSMUSG00000002486 | Tchp | -1.40119 | <0.00001 | down |
| ENSMUSG00000022351 | Sqle | 1.631809 | <0.00001 | up |
| ENSMUSG00000036620 | Mgat4b | 1.058021 | <0.00001 | up |
| ENSMUSG00000063087 | Gm10125 | -1.3667 | <0.00001 | down |
| ENSMUSG00000001930 | Vwf | -1.00231 | <0.00001 | down |
| ENSMUSG00000027533 | Fabp5 | 1.813242 | <0.00001 | up |
| ENSMUSG00000055435 | Maf | -1.62731 | <0.00001 | down |
| ENSMUSG00000028369 | Svep1 | -2.67885 | <0.00001 | down |
| ENSMUSG00000050777 | Tmem37 | 1.018256 | <0.00001 | up |
| ENSMUSG00000039202 | Abhd2 | 1.079703 | <0.00001 | up |
| ENSMUSG00000030431 | Tmem238 | 2.303729 | <0.00001 | up |
| ENSMUSG00000037818 | Abhd18 | -1.02157 | <0.00001 | down |
| ENSMUSG00000027230 | Creb3l1 | -2.15128 | <0.00001 | down |
| ENSMUSG00000027249 | F2 | 1.100854 | <0.00001 | up |
| ENSMUSG00000025041 | Nt5c2 | -1.00626 | <0.00001 | down |
| ENSMUSG00000052837 | Junb | 2.580569 | <0.00001 | up |
| ENSMUSG00000028013 | Ppa2 | 1.165642 | <0.00001 | up |
| ENSMUSG00000028743 | Akr7a5 | 1.189033 | <0.00001 | up |
| ENSMUSG00000110949 | Nudt8 | 1.155382 | <0.00001 | up |
| ENSMUSG00000020308 | Tpgs1 | 1.082152 | <0.00001 | up |
| ENSMUSG00000020038 | Cry1 | -1.88349 | <0.00001 | down |
| ENSMUSG00000021495 | Fam193b | -1.17607 | <0.00001 | down |
| ENSMUSG00000019796 | Lrp11 | 4.309473 | <0.00001 | up |
| ENSMUSG00000100005 | B130024G19Rik | -1.8695 | <0.00001 | down |
| ENSMUSG00000021835 | Bmp4 | -1.44702 | <0.00001 | down |
| ENSMUSG00000107096 | Gm43597 | -1.77208 | <0.00001 | down |
| ENSMUSG00000048668 | Rhno1 | -1.00513 | <0.00001 | down |
| ENSMUSG00000022466 | Rpap3 | 1.476363 | <0.00001 | up |
| ENSMUSG00000026879 | Gsn | -1.04842 | <0.00001 | down |
| ENSMUSG00000066196 | Spag8 | -3.17613 | <0.00001 | down |
| ENSMUSG00000038754 | Elovl3 | -2.77442 | <0.00001 | down |
| ENSMUSG00000109157 | Gm44829 | -2.93036 | <0.00001 | down |
| ENSMUSG00000066621 | Tecpr1 | -1.27364 | <0.00001 | down |
| ENSMUSG00000032026 | Rexo2 | 1.06829 | <0.00001 | up |
| ENSMUSG00000015016 | Acsf3 | 1.224065 | <0.00001 | up |
| ENSMUSG00000062825 | Actg1 | 1.133552 | <0.00001 | up |
| ENSMUSG00000112038 | Gm47056 | -2.03012 | <0.00001 | down |
| ENSMUSG00000079355 | Ackr4 | -2.66913 | <0.00001 | down |
| ENSMUSG00000074207 | Adh1 | 1.001283 | <0.00001 | up |
| ENSMUSG00000034880 | Mrpl34 | 1.478821 | <0.00001 | up |
| ENSMUSG00000078931 | Pdf | 1.664138 | <0.00001 | up |
| ENSMUSG00000074676 | Foxs1 | -5.02883 | <0.00001 | down |
| ENSMUSG00000052752 | Traf7 | -1.04002 | <0.00001 | down |
| ENSMUSG00000110234 | Gm45799 | 2.057843 | <0.00001 | up |
| ENSMUSG00000070473 | Cldn3 | 1.009734 | <0.00001 | up |
| ENSMUSG00000025421 | Hdhd2 | 1.126354 | <0.00001 | up |
| ENSMUSG00000055912 | Tmem150a | -1.04035 | <0.00001 | down |
| ENSMUSG00000073838 | Tufm | 1.238037 | <0.00001 | up |
| ENSMUSG00000021719 | Rgs7bp | -1.65401 | <0.00001 | down |
| ENSMUSG00000026102 | Inpp1 | -1.48812 | <0.00001 | down |
| ENSMUSG00000055302 | Mrfap1 | 1.0011 | <0.00001 | up |
| ENSMUSG00000097589 | Dleu2 | -1.14561 | <0.00001 | down |
| ENSMUSG00000008540 | Mgst1 | 1.076228 | <0.00001 | up |
| ENSMUSG00000020444 | Guk1 | 1.05507 | <0.00001 | up |
| ENSMUSG00000038884 | Shfl | -1.31413 | <0.00001 | down |
| ENSMUSG00000078201 | Tmem203 | 1.074082 | <0.00001 | up |
| ENSMUSG00000104011 | Gm32391 | -1.72729 | <0.00001 | down |
| ENSMUSG00000026064 | Ptp4a1 | 3.92562 | <0.00001 | up |
| ENSMUSG00000015357 | Clpx | -1.24484 | <0.00001 | down |
| ENSMUSG00000037787 | Coa8 | 1.037314 | <0.00001 | up |
| ENSMUSG00000039233 | Tbce | -1.1112 | <0.00001 | down |
| ENSMUSG00000019734 | Tmc4 | -1.55335 | <0.00001 | down |
| ENSMUSG00000097893 | 1700034P13Rik | -1.7802 | <0.00001 | down |
| ENSMUSG00000027248 | Pdia3 | 1.052586 | <0.00001 | up |
| ENSMUSG00000058952 | Cfi | 1.061456 | <0.00001 | up |
| ENSMUSG00000112734 | Gm47644 | -2.25569 | <0.00001 | down |
| ENSMUSG00000022610 | Mapk12 | -1.18973 | <0.00001 | down |
| ENSMUSG00000013495 | Tmem175 | -1.23961 | <0.00001 | down |
| ENSMUSG00000023965 | Fbxl17 | 1.258833 | <0.00001 | up |
| ENSMUSG00000022010 | Tsc22d1 | -1.22405 | <0.00001 | down |
| ENSMUSG00000043923 | Ccdc84 | -1.17873 | <0.00001 | down |
| ENSMUSG00000056724 | Nbeal2 | -1.23446 | <0.00001 | down |
| ENSMUSG00000036446 | Lum | -1.35801 | <0.00001 | down |
| ENSMUSG00000081648 | Gm13423 | -2.61552 | <0.00001 | down |
| ENSMUSG00000102275 | Gm37144 | -2.02777 | <0.00001 | down |
| ENSMUSG00000085687 | Gm16153 | -2.38232 | <0.00001 | down |
| ENSMUSG00000032827 | Ppp1r9a | -1.52253 | <0.00001 | down |
| ENSMUSG00000093942 | Olfr46 | -1.98312 | <0.00001 | down |
| ENSMUSG00000051095 | Olfr986 | -1.33503 | <0.00001 | down |
| ENSMUSG00000034912 | Mdga2 | -3.11401 | <0.00001 | down |
| ENSMUSG00000035910 | Dcdc2a | -1.55305 | <0.00001 | down |
| ENSMUSG00000020451 | Limk2 | -1.01246 | <0.00001 | down |
| ENSMUSG00000035459 | Stab2 | -1.0626 | <0.00001 | down |
| ENSMUSG00000043008 | Klhl6 | -1.74386 | <0.00001 | down |
| ENSMUSG00000051166 | Eml5 | -1.85861 | <0.00001 | down |
| ENSMUSG00000035561 | Aldh1b1 | 1.411261 | <0.00001 | up |
| ENSMUSG00000097464 | Gm26736 | -2.74712 | <0.00001 | down |
| ENSMUSG00000097336 | Fendrr | -1.71436 | <0.00001 | down |
| ENSMUSG00000002064 | Sdf2 | 1.109996 | <0.00001 | up |
| ENSMUSG00000038372 | Gmds | 3.414066 | <0.00001 | up |
| ENSMUSG00000026728 | Vim | -1.17596 | <0.00001 | down |
| ENSMUSG00000038387 | Rras | 1.061114 | <0.00001 | up |
| ENSMUSG00000038010 | Ccdc138 | -1.44585 | <0.00001 | down |
| ENSMUSG00000069919 | Hba-a1 | -1.35423 | <0.00001 | down |
| ENSMUSG00000018821 | Avpi1 | 1.22603 | <0.00001 | up |
| ENSMUSG00000040269 | Mrps28 | 1.09368 | <0.00001 | up |
| ENSMUSG00000106634 | Gm43042 | -1.77804 | <0.00001 | down |
| ENSMUSG00000037243 | Zfp692 | -1.48612 | <0.00001 | down |
| ENSMUSG00000025487 | Psmd13 | 1.640471 | <0.00001 | up |
| ENSMUSG00000089940 | Gm4117 | -1.84216 | <0.00001 | down |
| ENSMUSG00000111928 | Gm48082 | -1.54217 | <0.00001 | down |
| ENSMUSG00000019647 | Sema6a | -1.5766 | <0.00001 | down |
| ENSMUSG00000104060 | Gm37954 | -1.80982 | <0.00001 | down |
| ENSMUSG00000024502 | Jakmip2 | -1.6789 | <0.00001 | down |
| ENSMUSG00000023010 | Tmbim6 | 1.061019 | <0.00001 | up |
| ENSMUSG00000104867 | Gm43728 | -4.98129 | <0.00001 | down |
| ENSMUSG00000024165 | Jpt2 | 1.185097 | <0.00001 | up |
| ENSMUSG00000028469 | Npr2 | -1.07431 | <0.00001 | down |
| ENSMUSG00000057036 | Gm7536 | 1.049156 | <0.00001 | up |
| ENSMUSG00000029314 | Gpat3 | 1.665647 | <0.00001 | up |
| ENSMUSG00000037797 | Adh4 | 1.01428 | <0.00001 | up |
| ENSMUSG00000024217 | Snrpc | 1.327298 | <0.00001 | up |
| ENSMUSG00000116590 | Gm53028 | -2.08335 | <0.00001 | down |
| ENSMUSG00000020561 | Polr1f | -1.15139 | <0.00001 | down |
| ENSMUSG00000067279 | Ppp1r3c | -1.30096 | <0.00001 | down |
| ENSMUSG00000019978 | Epb41l2 | -1.09818 | <0.00001 | down |
| ENSMUSG00000120368 | - | -2.84579 | <0.00001 | down |
| ENSMUSG00000109162 | 2900027M19Rik | -1.82797 | <0.00001 | down |
| ENSMUSG00000023067 | Cdkn1a | -2.59199 | <0.00001 | down |
| ENSMUSG00000026463 | Atp2b4 | -1.52377 | <0.00001 | down |
| ENSMUSG00000105201 | Gm43362 | -3.29652 | <0.00001 | down |
| ENSMUSG00000029198 | Grpel1 | 1.395849 | <0.00001 | up |
| ENSMUSG00000034957 | Cebpa | 1.140735 | <0.00001 | up |
| ENSMUSG00000048126 | Col6a3 | -1.54484 | <0.00001 | down |
| ENSMUSG00000068263 | Efcc1 | -1.80576 | <0.00001 | down |
| ENSMUSG00000022537 | Tmem44 | -1.39177 | <0.00001 | down |
| ENSMUSG00000019806 | Aig1 | 1.490511 | <0.00001 | up |
| ENSMUSG00000080985 | Gm13559 | -3.33867 | <0.00001 | down |
| ENSMUSG00000018537 | Pcgf2 | -1.40051 | <0.00001 | down |
| ENSMUSG00000113788 | Gm47904 | -4.11308 | <0.00001 | down |
| ENSMUSG00000053128 | Rnf26 | 1.178975 | <0.00001 | up |
| ENSMUSG00000022236 | Ropn1l | 1.235095 | <0.00001 | up |
| ENSMUSG00000036504 | Phpt1 | 1.522461 | <0.00001 | up |
| ENSMUSG00000015745 | Plekho1 | -1.01901 | <0.00001 | down |
| ENSMUSG00000036281 | Snapc4 | -1.2849 | <0.00001 | down |
| ENSMUSG00000046352 | Gjb2 | 1.101866 | <0.00001 | up |
| ENSMUSG00000026074 | Map4k4 | -1.09785 | <0.00001 | down |
| ENSMUSG00000046688 | Tifa | 1.032728 | <0.00001 | up |
| ENSMUSG00000079523 | Tmsb10 | -1.33719 | <0.00001 | down |
| ENSMUSG00000027474 | Ccm2l | -1.32947 | <0.00001 | down |
| ENSMUSG00000103421 | Golt1a | 2.081557 | <0.00001 | up |
| ENSMUSG00000029096 | Htra3 | -1.5773 | <0.00001 | down |
| ENSMUSG00000024824 | Rad9a | -1.1888 | <0.00001 | down |
| ENSMUSG00000031146 | Plp2 | -1.15887 | <0.00001 | down |
| ENSMUSG00000110195 | Pde2a | -1.3401 | <0.00001 | down |
| ENSMUSG00000051790 | Nlgn2 | -1.49506 | <0.00001 | down |
| ENSMUSG00000033327 | Tnxb | -1.1744 | <0.00001 | down |
| ENSMUSG00000032353 | Tmed3 | 1.105259 | <0.00001 | up |
| ENSMUSG00000044139 | Prss53 | -1.35178 | <0.00001 | down |
| ENSMUSG00000025198 | Erlin1 | 1.128693 | <0.00001 | up |
| ENSMUSG00000117912 | Gm50383 | -4.62799 | <0.00001 | down |
| ENSMUSG00000034449 | Dhrs11 | 1.039658 | <0.00001 | up |
| ENSMUSG00000029545 | Acads | 1.31536 | <0.00001 | up |
| ENSMUSG00000109781 | Gm45509 | -2.90389 | <0.00001 | down |
| ENSMUSG00000043467 | Zbtb37 | -1.20572 | <0.00001 | down |
| ENSMUSG00000026837 | Col5a1 | -1.08798 | <0.00001 | down |
| ENSMUSG00000117390 | Gm50080 | -3.31475 | <0.00001 | down |
| ENSMUSG00000057388 | Mrpl18 | -1.0209 | <0.00001 | down |
| ENSMUSG00000087299 | Gm12953 | -2.02406 | <0.00001 | down |
| ENSMUSG00000028398 | Dmac1 | 1.287336 | <0.00001 | up |
| ENSMUSG00000052707 | Tnrc6a | -1.43331 | <0.00001 | down |
| ENSMUSG00000028691 | Prdx1 | 1.2777 | <0.00001 | up |
| ENSMUSG00000121283 | - | -1.50448 | <0.00001 | down |
| ENSMUSG00000030967 | Zranb1 | -1.38516 | <0.00001 | down |
| ENSMUSG00000026273 | Mterf4 | -1.04572 | <0.00001 | down |
| ENSMUSG00000020717 | Pecam1 | -1.14909 | <0.00001 | down |
| ENSMUSG00000041354 | Rgl2 | -1.0304 | <0.00001 | down |
| ENSMUSG00000035107 | Dcbld2 | -1.85005 | <0.00001 | down |
| ENSMUSG00000038738 | Shank1 | -2.33309 | <0.00001 | down |
| ENSMUSG00000109461 | Gm44848 | -2.86321 | <0.00001 | down |
| ENSMUSG00000104377 | Gm37515 | -1.5198 | <0.00001 | down |
| ENSMUSG00000062515 | Fabp4 | 1.778348 | <0.00001 | up |
| ENSMUSG00000071042 | Rasgrp3 | -1.28859 | <0.00001 | down |
| ENSMUSG00000107276 | Gm42858 | -2.34405 | <0.00001 | down |
| ENSMUSG00000025507 | Pidd1 | -1.67905 | <0.00001 | down |
| ENSMUSG00000108500 | Gm45033 | -2.63672 | <0.00001 | down |
| ENSMUSG00000032959 | Pebp1 | 1.077298 | <0.00001 | up |
| ENSMUSG00000037942 | Crp | 1.284686 | <0.00001 | up |
| ENSMUSG00000029273 | Sult1d1 | -1.21422 | <0.00001 | down |
| ENSMUSG00000037958 | Nsrp1 | -1.52892 | <0.00001 | down |
| ENSMUSG00000037278 | Tmem97 | 1.544643 | <0.00001 | up |
| ENSMUSG00000071076 | Jund | 1.613384 | <0.00001 | up |
| ENSMUSG00000040350 | Trim7 | -1.58202 | <0.00001 | down |
| ENSMUSG00000024150 | Mcfd2 | 1.017991 | <0.00001 | up |
| ENSMUSG00000113630 | 4930404H11Rik | -3.11628 | <0.00001 | down |
| ENSMUSG00000025790 | Slco3a1 | -1.797 | <0.00001 | down |
| ENSMUSG00000038301 | Snx10 | 1.19472 | <0.00001 | up |
| ENSMUSG00000006386 | Tek | -1.3048 | <0.00001 | down |
| ENSMUSG00000057894 | Zfp329 | -1.53967 | <0.00001 | down |
| ENSMUSG00000022360 | Atad2 | -1.84604 | <0.00001 | down |
| ENSMUSG00000108155 | Gm44443 | -3.04429 | <0.00001 | down |
| ENSMUSG00000059022 | Kcp | -1.64248 | <0.00001 | down |
| ENSMUSG00000037780 | Mbl1 | 1.043806 | <0.00001 | up |
| ENSMUSG00000034911 | Ushbp1 | -1.20537 | <0.00001 | down |
| ENSMUSG00000054737 | Zfp182 | -1.10999 | <0.00001 | down |
| ENSMUSG00000030541 | Idh2 | 1.279029 | <0.00001 | up |
| ENSMUSG00000109196 | Gm44715 | -2.02339 | <0.00001 | down |
| ENSMUSG00000106577 | Gm31026 | -2.12212 | <0.00001 | down |
| ENSMUSG00000033960 | Jcad | -1.39047 | <0.00001 | down |
| ENSMUSG00000019843 | Fyn | -1.22862 | <0.00001 | down |
| ENSMUSG00000029675 | Eln | -1.78943 | <0.00001 | down |
| ENSMUSG00000061306 | Slc38a10 | 1.038965 | <0.00001 | up |
| ENSMUSG00000048938 | Nr1h5 | -1.76173 | <0.00001 | down |
| ENSMUSG00000026154 | Sdhaf4 | 1.175624 | <0.00001 | up |
| ENSMUSG00000054716 | Zfp771 | 2.735948 | <0.00001 | up |
| ENSMUSG00000078768 | Zfp566 | -2.06828 | <0.00001 | down |
| ENSMUSG00000104434 | Gm37421 | 4.008956 | <0.00001 | up |
| ENSMUSG00000113831 | Gm49602 | -1.81788 | <0.00001 | down |
| ENSMUSG00000079563 | Pglyrp2 | 1.047998 | <0.00001 | up |
| ENSMUSG00000049600 | Zbtb45 | -1.94256 | <0.00001 | down |
| ENSMUSG00000018770 | Atp5g3 | 1.337514 | <0.00001 | up |
| ENSMUSG00000045538 | Ddx28 | 1.581248 | <0.00001 | up |
| ENSMUSG00000078348 | Sf3b5 | 1.602897 | <0.00001 | up |
| ENSMUSG00000046721 | Rpl14-ps1 | 1.397307 | <0.00001 | up |
| ENSMUSG00000115276 | 9930017N22Rik | -2.03417 | <0.00001 | down |
| ENSMUSG00000053754 | Chd8 | -1.0138 | <0.00001 | down |
| ENSMUSG00000057649 | Brd9 | -1.23006 | <0.00001 | down |
| ENSMUSG00000037994 | Slc9b2 | -2.18121 | <0.00001 | down |
| ENSMUSG00000078193 | Gm2000 | -9.02018 | <0.00001 | down |
| ENSMUSG00000023456 | Tpi1 | 1.211177 | <0.00001 | up |
| ENSMUSG00000032079 | Apoa5 | 1.079981 | <0.00001 | up |
| ENSMUSG00000020154 | Ptprb | -1.13347 | <0.00001 | down |
| ENSMUSG00000018567 | Gabarap | 1.207301 | <0.00001 | up |
| ENSMUSG00000025350 | Rdh5 | 1.201882 | <0.00001 | up |
| ENSMUSG00000028214 | Gem | -1.73846 | <0.00001 | down |
| ENSMUSG00000063232 | Serpina11 | 1.030869 | <0.00001 | up |
| ENSMUSG00000000739 | Sult5a1 | -1.71639 | <0.00001 | down |
| ENSMUSG00000031029 | Eif3f | 1.396013 | <0.00001 | up |
| ENSMUSG00000038803 | Ost4 | 1.099531 | <0.00001 | up |
| ENSMUSG00000000693 | Loxl3 | -1.79781 | <0.00001 | down |
| ENSMUSG00000015467 | Egfl8 | -2.28817 | <0.00001 | down |
| ENSMUSG00000007097 | Atp1a2 | -1.64584 | <0.00001 | down |
| ENSMUSG00000017721 | Pigt | 1.529022 | <0.00001 | up |
| ENSMUSG00000004633 | Chn2 | 1.123306 | <0.00001 | up |
| ENSMUSG00000028073 | Pear1 | -1.29631 | <0.00001 | down |
| ENSMUSG00000032271 | Nnmt | 1.550679 | <0.00001 | up |
| ENSMUSG00000046229 | Scand1 | 4.518197 | <0.00001 | up |
| ENSMUSG00000019122 | Ccl9 | 1.356054 | <0.00001 | up |
| ENSMUSG00000024875 | Yif1a | 1.230073 | <0.00001 | up |
| ENSMUSG00000019876 | Pkib | -1.86024 | <0.00001 | down |
| ENSMUSG00000024422 | Dhx16 | -1.04957 | <0.00001 | down |
| ENSMUSG00000086370 | Ftx | -1.56969 | <0.00001 | down |
| ENSMUSG00000066037 | Hnrnpr | -1.65416 | <0.00001 | down |
| ENSMUSG00000020467 | Efemp1 | -1.44779 | <0.00001 | down |
| ENSMUSG00000024843 | Chka | -2.4877 | <0.00001 | down |
| ENSMUSG00000031158 | Timm17b | 1.058933 | <0.00001 | up |
| ENSMUSG00000113795 | Gm48119 | -2.02956 | <0.00001 | down |
| ENSMUSG00000019432 | Ddx39b | -1.0539 | <0.00001 | down |
| ENSMUSG00000022868 | Ahsg | 1.361445 | <0.00001 | up |
| ENSMUSG00000020427 | Igfbp3 | -1.18478 | <0.00001 | down |
| ENSMUSG00000001119 | Col6a1 | -1.05064 | <0.00001 | down |
| ENSMUSG00000053898 | Ech1 | 1.06669 | <0.00001 | up |
| ENSMUSG00000073155 | 1810058I24Rik | 1.088904 | <0.00001 | up |
| ENSMUSG00000071337 | Tia1 | -1.23458 | <0.00001 | down |
| ENSMUSG00000020364 | Zfp354a | -1.67072 | <0.00001 | down |
| ENSMUSG00000105748 | Gm43088 | -2.7784 | <0.00001 | down |
| ENSMUSG00000114722 | Gm31392 | -2.48529 | <0.00001 | down |
| ENSMUSG00000025479 | Cyp2e1 | 1.696788 | <0.00001 | up |
| ENSMUSG00000112226 | Gm48786 | -1.98144 | <0.00001 | down |
| ENSMUSG00000002778 | Kdelr1 | 1.196582 | <0.00001 | up |
| ENSMUSG00000002032 | Tmem25 | -1.35577 | <0.00001 | down |
| ENSMUSG00000032035 | Ets1 | -1.14111 | <0.00001 | down |
| ENSMUSG00000067653 | Ankrd23 | -1.63262 | <0.00001 | down |
| ENSMUSG00000119895 | Gm22513 | -2.67638 | <0.00001 | down |
| ENSMUSG00000040111 | Gramd1b | -1.67021 | <0.00001 | down |
| ENSMUSG00000042156 | Dzip1 | -2.63049 | <0.00001 | down |
| ENSMUSG00000033735 | Spr | 1.376488 | <0.00001 | up |
| ENSMUSG00000025481 | Urah | 1.368784 | <0.00001 | up |
| ENSMUSG00000026199 | Ankzf1 | -1.27204 | <0.00001 | down |
| ENSMUSG00000113961 | Gm48498 | -1.85001 | <0.00001 | down |
| ENSMUSG00000108173 | Gm44231 | -2.10145 | <0.00001 | down |
| ENSMUSG00000113517 | Gm47905 | -2.05078 | <0.00001 | down |
| ENSMUSG00000026656 | Fcgr2b | -1.55067 | <0.00001 | down |
| ENSMUSG00000031858 | Mau2 | -1.07546 | <0.00001 | down |
| ENSMUSG00000002885 | Adgre5 | -1.21302 | <0.00001 | down |
| ENSMUSG00000032177 | Pde4a | -1.74232 | <0.00001 | down |
| ENSMUSG00000041084 | Ostc | 1.410332 | <0.00001 | up |
| ENSMUSG00000106030 | Gm43611 | -2.96634 | <0.00001 | down |
| ENSMUSG00000015090 | Ptgds | -2.73573 | <0.00001 | down |
| ENSMUSG00000017493 | Igfbp4 | 1.238965 | <0.00001 | up |
| ENSMUSG00000108092 | Gm44189 | -2.86395 | <0.00001 | down |
| ENSMUSG00000001755 | Coasy | 1.233588 | <0.00001 | up |
| ENSMUSG00000038213 | Tapbpl | 1.064564 | <0.00001 | up |
| ENSMUSG00000008140 | Emc10 | 1.356838 | <0.00001 | up |
| ENSMUSG00000009549 | Srp14 | 1.102929 | <0.00001 | up |
| ENSMUSG00000116238 | Gm49413 | -3.97876 | <0.00001 | down |
| ENSMUSG00000031133 | Arhgef6 | -1.41284 | <0.00001 | down |
| ENSMUSG00000032744 | Heyl | -1.62872 | <0.00001 | down |
| ENSMUSG00000038009 | Dnajc22 | 1.039553 | <0.00001 | up |
| ENSMUSG00000026222 | Sp100 | -1.22033 | <0.00001 | down |
| ENSMUSG00000022816 | Fstl1 | -1.77405 | <0.00001 | down |
| ENSMUSG00000021186 | Fbln5 | -1.45621 | <0.00001 | down |
| ENSMUSG00000001376 | Vps50 | -1.06487 | <0.00001 | down |
| ENSMUSG00000034771 | Tle2 | -1.24128 | <0.00001 | down |
| ENSMUSG00000061477 | Rps7 | 1.088939 | <0.00001 | up |
| ENSMUSG00000002767 | Mrpl2 | 1.073253 | <0.00001 | up |
| ENSMUSG00000029767 | Calu | 1.12919 | <0.00001 | up |
| ENSMUSG00000023073 | Slc10a2 | 1.328158 | <0.00001 | up |
| ENSMUSG00000036813 | Entpd8 | -1.04128 | <0.00001 | down |
| ENSMUSG00000052921 | Arhgef15 | -1.51148 | <0.00001 | down |
| ENSMUSG00000031807 | Pgls | 1.117377 | <0.00001 | up |
| ENSMUSG00000002625 | Akap8l | -1.38838 | <0.00001 | down |
| ENSMUSG00000066150 | Slc31a1 | 1.014131 | <0.00001 | up |
| ENSMUSG00000048234 | Rnf149 | 1.959065 | <0.00001 | up |
| ENSMUSG00000041775 | Mapk1ip1 | -1.43986 | <0.00001 | down |
| ENSMUSG00000059136 | Olfr539 | -1.8144 | <0.00001 | down |
| ENSMUSG00000059434 | Gckr | -1.06996 | <0.00001 | down |
| ENSMUSG00000100510 | Hand2os1 | -1.57543 | <0.00001 | down |
| ENSMUSG00000002332 | Dhrs1 | 1.19545 | <0.00001 | up |
| ENSMUSG00000042308 | Setd1a | -1.2102 | <0.00001 | down |
| ENSMUSG00000036553 | Sh3tc1 | -1.6329 | <0.00001 | down |
| ENSMUSG00000029417 | Cxcl9 | -1.80009 | <0.00001 | down |
| ENSMUSG00000045257 | Morn2 | 1.431459 | <0.00001 | up |
| ENSMUSG00000047617 | Paxx | -1.80333 | <0.00001 | down |
| ENSMUSG00000056148 | Rdh9 | 1.689147 | <0.00001 | up |
| ENSMUSG00000103693 | Gm37529 | -2.44542 | <0.00001 | down |
| ENSMUSG00000085008 | Dbhos | -3.07195 | <0.00001 | down |
| ENSMUSG00000004460 | Dnajb11 | 1.058285 | <0.00001 | up |
| ENSMUSG00000029238 | Clock | -1.36275 | <0.00001 | down |
| ENSMUSG00000019935 | Slc17a8 | -1.85982 | <0.00001 | down |
| ENSMUSG00000044037 | Als2cl | -1.23413 | <0.00001 | down |
| ENSMUSG00000066258 | Trim12a | -1.31959 | <0.00001 | down |
| ENSMUSG00000034037 | Fgd5 | -1.29578 | <0.00001 | down |
| ENSMUSG00000008398 | Elk3 | -1.29739 | <0.00001 | down |
| ENSMUSG00000055216 | 9430025C20Rik | -2.51028 | <0.00001 | down |
| ENSMUSG00000020884 | Asgr1 | 1.12837 | <0.00001 | up |
| ENSMUSG00000001588 | Acap1 | -1.81239 | <0.00001 | down |
| ENSMUSG00000024045 | Akap8 | -1.0619 | <0.00001 | down |
| ENSMUSG00000015944 | Castor2 | -1.63591 | <0.00001 | down |
| ENSMUSG00000073643 | Wdfy1 | -1.07626 | <0.00001 | down |
| ENSMUSG00000022974 | Paxbp1 | -1.28582 | <0.00001 | down |
| ENSMUSG00000026872 | Zeb2 | -1.47015 | <0.00001 | down |
| ENSMUSG00000002320 | Tm9sf1 | 1.096184 | <0.00001 | up |
| ENSMUSG00000041355 | Ssr2 | 1.164435 | <0.00001 | up |
| ENSMUSG00000023885 | Thbs2 | -1.95539 | <0.00001 | down |
| ENSMUSG00000037686 | Aspg | -1.30865 | <0.00001 | down |
| ENSMUSG00000009418 | Nav1 | -1.52455 | <0.00001 | down |
| ENSMUSG00000021248 | Tmed10 | 1.262377 | <0.00001 | up |
| ENSMUSG00000022799 | Arhgap31 | -1.26637 | <0.00001 | down |
| ENSMUSG00000054580 | Pla2r1 | -2.23937 | <0.00001 | down |
| ENSMUSG00000014905 | Dnajb9 | 1.359828 | <0.00001 | up |
| ENSMUSG00000002580 | Mien1 | 1.082193 | <0.00001 | up |
| ENSMUSG00000101599 | Gm20342 | -2.01814 | <0.00001 | down |
| ENSMUSG00000113427 | Gm46378 | -2.19062 | <0.00001 | down |
| ENSMUSG00000008200 | Fnbp4 | -1.128 | <0.00001 | down |
| ENSMUSG00000083563 | Gm13340 | -2.96993 | <0.00001 | down |
| ENSMUSG00000029727 | Cyp3a13 | 1.613077 | <0.00001 | up |
| ENSMUSG00000047631 | Apof | 1.237311 | <0.00001 | up |
| ENSMUSG00000028238 | Atp6v0d2 | 2.676278 | <0.00001 | up |
| ENSMUSG00000024516 | Sec11c | 1.026018 | <0.00001 | up |
| ENSMUSG00000097042 | Gm17491 | -1.47542 | <0.00001 | down |
| ENSMUSG00000107336 | Gm43461 | -2.99693 | <0.00001 | down |
| ENSMUSG00000009646 | Pla2g12b | 1.152002 | <0.00001 | up |
| ENSMUSG00000032580 | Rbm5 | -1.49204 | <0.00001 | down |
| ENSMUSG00000024421 | Lama3 | -1.67386 | <0.00001 | down |
| ENSMUSG00000032497 | Lrrfip2 | -1.12353 | <0.00001 | down |
| ENSMUSG00000041697 | Cox6a1 | 1.224835 | <0.00001 | up |
| ENSMUSG00000026223 | Itm2c | 1.741583 | <0.00001 | up |
| ENSMUSG00000041736 | Tspo | 1.736066 | <0.00001 | up |
| ENSMUSG00000074340 | Ovgp1 | -2.8486 | <0.00001 | down |
| ENSMUSG00000068101 | Cenpm | 2.14664 | <0.00001 | up |
| ENSMUSG00000053964 | Lgals4 | -1.60351 | <0.00001 | down |
| ENSMUSG00000025508 | Rplp2 | 1.1737 | <0.00001 | up |
| ENSMUSG00000028051 | Hcn3 | -2.28879 | <0.00001 | down |
| ENSMUSG00000027556 | Car1 | -1.59079 | <0.00001 | down |
| ENSMUSG00000002900 | Lamb1 | -1.44579 | <0.00001 | down |
| ENSMUSG00000001100 | Poldip2 | 1.029229 | <0.00001 | up |
| ENSMUSG00000052562 | Slc22a30 | -1.10944 | <0.00001 | down |
| ENSMUSG00000024018 | Ccdc167 | 1.223331 | <0.00001 | up |
| ENSMUSG00000112972 | Gm48417 | -3.10743 | <0.00001 | down |
| ENSMUSG00000079165 | Sap25 | -2.40995 | <0.00001 | down |
| ENSMUSG00000074219 | Gm10644 | -1.60367 | <0.00001 | down |
| ENSMUSG00000020089 | Ppa1 | 1.547695 | <0.00001 | up |
| ENSMUSG00000003380 | Rabac1 | 1.12538 | <0.00001 | up |
| ENSMUSG00000022174 | Dad1 | 1.571264 | <0.00001 | up |
| ENSMUSG00000060036 | Rpl3 | 1.041277 | <0.00001 | up |
| ENSMUSG00000040963 | Asgr2 | 1.00139 | <0.00001 | up |
| ENSMUSG00000096145 | Vkorc1 | 1.137367 | <0.00001 | up |
| ENSMUSG00000112239 | Gm17823 | -2.45155 | <0.00001 | down |
| ENSMUSG00000018669 | Cdk5rap3 | 1.077597 | <0.00001 | up |
| ENSMUSG00000001467 | Cyp51 | 1.950431 | <0.00001 | up |
| ENSMUSG00000032316 | Clk3 | -1.14055 | <0.00001 | down |
| ENSMUSG00000097392 | Thoc2l | -1.32687 | <0.00001 | down |
| ENSMUSG00000081207 | Gm13775 | -2.12514 | <0.00001 | down |
| ENSMUSG00000022877 | Hrg | 1.185713 | <0.00001 | up |
| ENSMUSG00000036040 | Adamtsl2 | -1.48879 | <0.00001 | down |
| ENSMUSG00000005373 | Mlxipl | -1.12569 | <0.00001 | down |
| ENSMUSG00000031595 | Pdgfrl | -2.89884 | <0.00001 | down |
| ENSMUSG00000031842 | Pde4c | -1.42507 | <0.00001 | down |
| ENSMUSG00000095597 | Rps7-ps3 | -9.8403 | <0.00001 | down |
| ENSMUSG00000110631 | Gm42047 | -1.76701 | <0.00001 | down |
| ENSMUSG00000009292 | Trpm2 | -1.76987 | <0.00001 | down |
| ENSMUSG00000070891 | Gm12689 | -3.44642 | <0.00001 | down |
| ENSMUSG00000112794 | Gm48878 | -1.4604 | <0.00001 | down |
| ENSMUSG00000045672 | Col27a1 | -2.70368 | <0.00001 | down |
| ENSMUSG00000099034 | 2810039B14Rik | -1.25575 | <0.00001 | down |
| ENSMUSG00000111917 | Gm48793 | -2.84876 | <0.00001 | down |
| ENSMUSG00000068876 | Cgn | -1.23557 | <0.00001 | down |
| ENSMUSG00000025967 | Eef1b2 | 1.132561 | <0.00001 | up |
| ENSMUSG00000035885 | Cox8a | 1.201485 | <0.00001 | up |
| ENSMUSG00000038312 | Edem2 | 1.111667 | <0.00001 | up |
| ENSMUSG00000041445 | Mmrn2 | -1.31182 | <0.00001 | down |
| ENSMUSG00000022894 | Adamts5 | -1.97537 | <0.00001 | down |
| ENSMUSG00000003402 | Prkcsh | 1.104523 | <0.00001 | up |
| ENSMUSG00000043418 | Lrit2 | -1.79581 | <0.00001 | down |
| ENSMUSG00000040829 | Zmynd15 | -1.5662 | <0.00001 | down |
| ENSMUSG00000112880 | Gm20337 | -2.63417 | <0.00001 | down |
| ENSMUSG00000105247 | Gm42519 | -2.82274 | <0.00001 | down |
| ENSMUSG00000111118 | Gm6545 | -3.05346 | <0.00001 | down |
| ENSMUSG00000027999 | Pla2g12a | 1.207302 | <0.00001 | up |
| ENSMUSG00000042286 | Stab1 | -1.2368 | <0.00001 | down |
| ENSMUSG00000032198 | Dock6 | -1.71059 | <0.00001 | down |
| ENSMUSG00000032845 | Alpk2 | -2.41231 | <0.00001 | down |
| ENSMUSG00000038695 | Josd2 | -1.4484 | <0.00001 | down |
| ENSMUSG00000030591 | Psmd8 | 1.034044 | <0.00001 | up |
| ENSMUSG00000076431 | Sox4 | -2.28442 | <0.00001 | down |
| ENSMUSG00000029701 | Rbm28 | -1.03836 | <0.00001 | down |
| ENSMUSG00000004565 | Pnpla6 | -1.31589 | <0.00001 | down |
| ENSMUSG00000003363 | Pld3 | 1.132848 | <0.00001 | up |
| ENSMUSG00000050240 | Hic2 | -2.00997 | <0.00001 | down |
| ENSMUSG00000107624 | Gm44005 | -3.17866 | <0.00001 | down |
| ENSMUSG00000024065 | Ehd3 | -1.08629 | <0.00001 | down |
| ENSMUSG00000036083 | Slc17a3 | -1.20838 | <0.00001 | down |
| ENSMUSG00000039438 | Ttc36 | 1.139461 | <0.00001 | up |
| ENSMUSG00000057425 | Ugt2b37 | -1.88292 | <0.00001 | down |
| ENSMUSG00000079012 | Serpina3m | 1.768285 | <0.00001 | up |
| ENSMUSG00000021268 | Meg3 | -2.21375 | <0.00001 | down |
| ENSMUSG00000079555 | Haus3 | -1.59603 | <0.00001 | down |
| ENSMUSG00000026342 | Slc35f5 | 2.007646 | <0.00001 | up |
| ENSMUSG00000052031 | Tagap1 | -1.20235 | <0.00001 | down |
| ENSMUSG00000085028 | Slc2a4rg-ps | -1.72576 | <0.00001 | down |
| ENSMUSG00000045954 | Cavin2 | -1.35319 | <0.00001 | down |
| ENSMUSG00000027820 | Mme | -1.68887 | <0.00001 | down |
| ENSMUSG00000099707 | Gm8883 | 1.791576 | <0.00001 | up |
| ENSMUSG00000037966 | Ninj1 | 1.096371 | <0.00001 | up |
| ENSMUSG00000026750 | Psmb7 | 1.268234 | <0.00001 | up |
| ENSMUSG00000023057 | Fabp2 | 1.75307 | <0.00001 | up |
| ENSMUSG00000023367 | Tmem176a | 1.197534 | <0.00001 | up |
| ENSMUSG00000026511 | Srp9 | 1.059838 | <0.00001 | up |
| ENSMUSG00000022037 | Clu | 1.625443 | <0.00001 | up |
| ENSMUSG00000068749 | Psma5 | 1.442166 | <0.00001 | up |
| ENSMUSG00000021820 | Camk2g | -1.03784 | <0.00001 | down |
| ENSMUSG00000022617 | Chkb | -1.07219 | <0.00001 | down |
| ENSMUSG00000018411 | Mapt | -1.92013 | <0.00001 | down |
| ENSMUSG00000058793 | Cds2 | 1.006986 | <0.00001 | up |
| ENSMUSG00000024451 | Arap3 | -1.33743 | <0.00001 | down |
| ENSMUSG00000053175 | Bcl3 | 1.24068 | <0.00001 | up |
| ENSMUSG00000001095 | Slc13a2 | -3.07813 | <0.00001 | down |
| ENSMUSG00000109179 | Gm35339 | -1.98439 | <0.00001 | down |
| ENSMUSG00000028479 | Gne | 1.192788 | <0.00001 | up |
| ENSMUSG00000066263 | Olfr639 | -1.82752 | <0.00001 | down |
| ENSMUSG00000021928 | Ebpl | 1.319981 | <0.00001 | up |
| ENSMUSG00000025196 | Cpn1 | 1.047469 | <0.00001 | up |
| ENSMUSG00000033107 | Rnf125 | -2.03884 | <0.00001 | down |
| ENSMUSG00000027430 | Dtd1 | 1.232484 | <0.00001 | up |
| ENSMUSG00000004040 | Stat3 | 1.169407 | <0.00001 | up |
| ENSMUSG00000043183 | Simc1 | -1.65139 | <0.00001 | down |
| ENSMUSG00000039062 | Anpep | 1.112867 | <0.00001 | up |
| ENSMUSG00000031379 | Pir | 1.35969 | <0.00001 | up |
| ENSMUSG00000032042 | Srpr | 1.212475 | <0.00001 | up |
| ENSMUSG00000047230 | Cldn2 | 1.728366 | <0.00001 | up |
| ENSMUSG00000060600 | Eno3 | -1.90707 | <0.00001 | down |
| ENSMUSG00000060459 | Kng2 | 1.120937 | <0.00001 | up |
| ENSMUSG00000031570 | Plpp5 | 1.378661 | <0.00001 | up |
| ENSMUSG00000018459 | Slc13a3 | 1.213758 | <0.00001 | up |
| ENSMUSG00000021952 | Xpo4 | -2.2735 | <0.00001 | down |
| ENSMUSG00000029119 | Man2b2 | 1.392995 | <0.00001 | up |
| ENSMUSG00000059714 | Flot1 | 1.464782 | <0.00001 | up |
| ENSMUSG00000020486 | Septin4 | -1.45296 | <0.00001 | down |
| ENSMUSG00000020869 | Lrrc59 | 1.169156 | <0.00001 | up |
| ENSMUSG00000085334 | Gm12940 | -1.93141 | <0.00001 | down |
| ENSMUSG00000031389 | Arhgap4 | -1.4274 | <0.00001 | down |
| ENSMUSG00000042066 | Tmcc2 | -1.9838 | <0.00001 | down |
| ENSMUSG00000053094 | Tmem248 | 1.157406 | <0.00001 | up |
| ENSMUSG00000106948 | Gm42785 | -2.21608 | <0.00001 | down |
| ENSMUSG00000022912 | Pros1 | 1.016984 | <0.00001 | up |
| ENSMUSG00000072501 | Phf20l1 | -1.29643 | <0.00001 | down |
| ENSMUSG00000045466 | Zfp956 | -1.53033 | <0.00001 | down |
| ENSMUSG00000051716 | Apon | 1.052116 | <0.00001 | up |
| ENSMUSG00000065947 | mt-Nd4l | -2.67774 | <0.00001 | down |
| ENSMUSG00000038539 | Atf5 | 1.226531 | <0.00001 | up |
| ENSMUSG00000057110 | Cntrl | -1.24035 | <0.00001 | down |
| ENSMUSG00000042770 | Hebp1 | 1.051505 | <0.00001 | up |
| ENSMUSG00000021336 | Slc17a4 | -1.48748 | <0.00001 | down |
| ENSMUSG00000078570 | 1110065P20Rik | 1.881726 | <0.00001 | up |
| ENSMUSG00000027006 | Dnajc10 | 1.127917 | <0.00001 | up |
| ENSMUSG00000032051 | Fdx1 | 1.190032 | <0.00001 | up |
| ENSMUSG00000026193 | Fn1 | 1.071774 | <0.00001 | up |
| ENSMUSG00000006522 | Itih3 | 1.108621 | <0.00001 | up |
| ENSMUSG00000001942 | Siae | 1.047751 | <0.00001 | up |
| ENSMUSG00000090386 | Mir99ahg | -2.25438 | <0.00001 | down |
| ENSMUSG00000049517 | Rps23 | 1.164693 | <0.00001 | up |
| ENSMUSG00000029616 | Erp29 | 1.067549 | <0.00001 | up |
| ENSMUSG00000038188 | Scarf1 | -1.61203 | <0.00001 | down |
| ENSMUSG00000005682 | Pan2 | -1.37059 | <0.00001 | down |
| ENSMUSG00000055312 | Them7 | 1.535274 | <0.00001 | up |
| ENSMUSG00000073409 | H2-Q6 | -1.73818 | <0.00001 | down |
| ENSMUSG00000112622 | Gm47164 | -1.88163 | <0.00001 | down |
| ENSMUSG00000015790 | Surf1 | 1.079467 | <0.00001 | up |
| ENSMUSG00000031490 | Eif4ebp1 | 1.467984 | <0.00001 | up |
| ENSMUSG00000035697 | Arhgap45 | -1.3682 | <0.00001 | down |
| ENSMUSG00000020766 | Galk1 | 1.691359 | <0.00001 | up |
| ENSMUSG00000055116 | Arntl | -2.88272 | <0.00001 | down |
| ENSMUSG00000025381 | Cnpy2 | 1.143571 | <0.00001 | up |
| ENSMUSG00000117292 | E330032C10Rik | -2.71247 | <0.00001 | down |
| ENSMUSG00000054676 | 1600014C10Rik | 1.131618 | <0.00001 | up |
| ENSMUSG00000027313 | Chac1 | 2.607527 | <0.00001 | up |
| ENSMUSG00000064373 | Selenop | 1.952328 | <0.00001 | up |
| ENSMUSG00000041044 | Lrit1 | -1.40104 | <0.00001 | down |
| ENSMUSG00000026614 | Slc30a10 | -2.04727 | <0.00001 | down |
| ENSMUSG00000032125 | Robo4 | -1.77447 | <0.00001 | down |
| ENSMUSG00000025511 | Tspan4 | 1.41568 | <0.00001 | up |
| ENSMUSG00000024299 | Adamts10 | -1.43703 | <0.00001 | down |
| ENSMUSG00000117599 | Gm49971 | -2.08362 | <0.00001 | down |
| ENSMUSG00000053113 | Socs3 | 2.086701 | <0.00001 | up |
| ENSMUSG00000032743 | Katnip | -1.32829 | <0.00001 | down |
| ENSMUSG00000029009 | Mthfr | -1.6878 | <0.00001 | down |
| ENSMUSG00000035413 | Tmem98 | 1.72355 | <0.00001 | up |
| ENSMUSG00000113476 | Gm48309 | -2.33187 | <0.00001 | down |
| ENSMUSG00000036545 | Adamts2 | -1.47438 | <0.00001 | down |
| ENSMUSG00000007892 | Rplp1 | 1.156483 | <0.00001 | up |
| ENSMUSG00000063229 | Ldha | 1.843815 | <0.00001 | up |
| ENSMUSG00000038224 | Serpinf2 | 1.011177 | <0.00001 | up |
| ENSMUSG00000039195 | Bbln | 1.578972 | <0.00001 | up |
| ENSMUSG00000005779 | Psmb4 | 1.316825 | <0.00001 | up |
| ENSMUSG00000030681 | Mvp | 1.21576 | <0.00001 | up |
| ENSMUSG00000060961 | Slc4a4 | 1.327698 | <0.00001 | up |
| ENSMUSG00000022371 | Col14a1 | -1.46521 | <0.00001 | down |
| ENSMUSG00000030062 | Rpn1 | 1.311494 | <0.00001 | up |
| ENSMUSG00000112013 | Gm47967 | -3.1356 | <0.00001 | down |
| ENSMUSG00000074862 | BC025920 | -2.8951 | <0.00001 | down |
| ENSMUSG00000094747 | Olfr1307 | -2.3402 | <0.00001 | down |
| ENSMUSG00000115018 | Ndor1 | -1.81663 | <0.00001 | down |
| ENSMUSG00000029352 | Crybb3 | 3.163497 | <0.00001 | up |
| ENSMUSG00000117238 | - | -3.09831 | <0.00001 | down |
| ENSMUSG00000044636 | Csrnp2 | -2.22887 | <0.00001 | down |
| ENSMUSG00000040488 | Ltbp4 | -1.23554 | <0.00001 | down |
| ENSMUSG00000040904 | Gm21988 | 2.114145 | <0.00001 | up |
| ENSMUSG00000033191 | Tie1 | -1.28202 | <0.00001 | down |
| ENSMUSG00000064220 | H2ac18 | 3.681567 | <0.00001 | up |
| ENSMUSG00000038233 | Gask1a | -3.14059 | <0.00001 | down |
| ENSMUSG00000096002 | Vmn2r53 | -1.92755 | <0.00001 | down |
| ENSMUSG00000001155 | Ftcd | -1.09668 | <0.00001 | down |
| ENSMUSG00000042797 | Aqp11 | 1.367824 | <0.00001 | up |
| ENSMUSG00000021477 | Ctsl | 1.721559 | <0.00001 | up |
| ENSMUSG00000090272 | Mndal | -1.55093 | <0.00001 | down |
| ENSMUSG00000037190 | Cyb561d2 | 1.60828 | <0.00001 | up |
| ENSMUSG00000001750 | Tcirg1 | -1.00669 | <0.00001 | down |
| ENSMUSG00000049404 | Rarres1 | 1.989191 | <0.00001 | up |
| ENSMUSG00000022094 | Slc39a14 | 1.100638 | <0.00001 | up |
| ENSMUSG00000020674 | Pxdn | -1.63568 | <0.00001 | down |
| ENSMUSG00000025357 | Dgka | -1.24136 | <0.00001 | down |
| ENSMUSG00000026365 | Cfh | 1.114521 | <0.00001 | up |
| ENSMUSG00000031451 | Gas6 | 1.102874 | <0.00001 | up |
| ENSMUSG00000039982 | Dtx4 | -1.7303 | <0.00001 | down |
| ENSMUSG00000070427 | Il18bp | 1.731758 | <0.00001 | up |
| ENSMUSG00000026390 | Marco | 1.660736 | <0.00001 | up |
| ENSMUSG00000025102 | 3110040N11Rik | 2.059678 | <0.00001 | up |
| ENSMUSG00000031765 | Mt1 | 4.061129 | <0.00001 | up |
| ENSMUSG00000056492 | Adgrf5 | -1.33246 | <0.00001 | down |
| ENSMUSG00000090877 | Hspa1b | 2.193148 | <0.00001 | up |
| ENSMUSG00000086253 | Gm13773 | -2.05203 | <0.00001 | down |
| ENSMUSG00000067274 | Rplp0 | 1.12176 | <0.00001 | up |
| ENSMUSG00000047547 | Cltb | 1.139067 | <0.00001 | up |
| ENSMUSG00000101939 | Gm28438 | -1.8938 | <0.00001 | down |
| ENSMUSG00000059481 | Plg | 1.044582 | <0.00001 | up |
| ENSMUSG00000030879 | Mrpl17 | 1.322223 | <0.00001 | up |
| ENSMUSG00000051748 | Wfdc21 | 2.091072 | <0.00001 | up |
| ENSMUSG00000022844 | Pdia5 | 1.155418 | <0.00001 | up |
| ENSMUSG00000022365 | Derl1 | 1.201341 | <0.00001 | up |
| ENSMUSG00000081534 | Slc48a1 | 1.50264 | <0.00001 | up |
| ENSMUSG00000031320 | Rps4x | 1.110394 | <0.00001 | up |
| ENSMUSG00000038880 | Mrps34 | 1.169414 | <0.00001 | up |
| ENSMUSG00000023224 | Serping1 | 1.504033 | <0.00001 | up |
| ENSMUSG00000079494 | Nat8f5 | -2.29817 | <0.00001 | down |
| ENSMUSG00000030082 | Sec61a1 | 1.195995 | <0.00001 | up |
| ENSMUSG00000027801 | Tm4sf4 | 1.427765 | <0.00001 | up |
| ENSMUSG00000031762 | Mt2 | 4.297498 | <0.00001 | up |
| ENSMUSG00000108857 | Gm44578 | -3.56058 | <0.00001 | down |
| ENSMUSG00000017344 | Vtn | 1.176878 | <0.00001 | up |
| ENSMUSG00000029390 | Tmed2 | 1.185777 | <0.00001 | up |
| ENSMUSG00000029661 | Col1a2 | -1.3508 | <0.00001 | down |
| ENSMUSG00000039997 | Ifi203 | -1.47378 | <0.00001 | down |
| ENSMUSG00000024181 | Mrpl28 | 1.493006 | <0.00001 | up |
| ENSMUSG00000046312 | Myorg | 1.321866 | <0.00001 | up |
| ENSMUSG00000071176 | Arhgef10 | -1.84179 | <0.00001 | down |
| ENSMUSG00000042073 | Abhd14b | 1.31839 | <0.00001 | up |
| ENSMUSG00000060470 | Adgrg3 | -1.65739 | <0.00001 | down |
| ENSMUSG00000037072 | Selenof | 1.486073 | <0.00001 | up |
| ENSMUSG00000030761 | Myo7a | -1.53946 | <0.00001 | down |
| ENSMUSG00000029380 | Cxcl1 | 4.2355 | <0.00001 | up |
| ENSMUSG00000086583 | Gm15500 | 1.28306 | <0.00001 | up |
| ENSMUSG00000049382 | Krt8 | 1.572958 | <0.00001 | up |
| ENSMUSG00000112788 | Gm47821 | -2.72565 | <0.00001 | down |
| ENSMUSG00000040017 | Saa4 | 1.368902 | <0.00001 | up |
| ENSMUSG00000067736 | Gm10222 | -2.01115 | <0.00001 | down |
| ENSMUSG00000075701 | Selenos | 1.725258 | <0.00001 | up |
| ENSMUSG00000061983 | Rps12 | 1.128656 | <0.00001 | up |
| ENSMUSG00000027642 | Rpn2 | 1.206157 | <0.00001 | up |
| ENSMUSG00000021999 | Cpb2 | 1.950453 | <0.00001 | up |
| ENSMUSG00000028757 | Ddost | 1.414497 | <0.00001 | up |
| ENSMUSG00000015289 | Lage3 | 1.408304 | <0.00001 | up |
| ENSMUSG00000015224 | Cyp2j9 | -2.10758 | <0.00001 | down |
| ENSMUSG00000025512 | Chid1 | 1.197238 | <0.00001 | up |
| ENSMUSG00000034926 | Dhcr24 | 1.928459 | <0.00001 | up |
| ENSMUSG00000053329 | Gatd3a | 1.403512 | <0.00001 | up |
| ENSMUSG00000026405 | C4bp | 1.595716 | <0.00001 | up |
| ENSMUSG00000064356 | mt-Atp8 | -2.49808 | <0.00001 | down |
| ENSMUSG00000027828 | Ssr3 | 1.387218 | <0.00001 | up |
| ENSMUSG00000064339 | mt-Rnr2 | -2.00646 | <0.00001 | down |
| ENSMUSG00000060703 | Cd302 | 1.723754 | <0.00001 | up |
| ENSMUSG00000028356 | Ambp | 1.514979 | <0.00001 | up |
| ENSMUSG00000031848 | Lsm4 | 1.747219 | <0.00001 | up |
| ENSMUSG00000116903 | Gm19522 | -3.52591 | <0.00001 | down |
| ENSMUSG00000021610 | Clptm1l | 1.007765 | <0.00001 | up |
| ENSMUSG00000052151 | Plpp2 | 1.274698 | <0.00001 | up |
| ENSMUSG00000029810 | Tmem176b | 1.5449 | <0.00001 | up |
| ENSMUSG00000116450 | Gm49534 | -2.85986 | <0.00001 | down |
| ENSMUSG00000032383 | Ppib | 1.522863 | <0.00001 | up |
| ENSMUSG00000030359 | Pzp | 1.619043 | <0.00001 | up |
| ENSMUSG00000030341 | Tnfrsf1a | 1.331983 | <0.00001 | up |
| ENSMUSG00000021877 | Arf4 | 1.230597 | <0.00001 | up |
| ENSMUSG00000121505 | Gm4956 | -2.21567 | <0.00001 | down |
| ENSMUSG00000014867 | Surf4 | 1.271098 | <0.00001 | up |
| ENSMUSG00000020733 | Slc9a3r1 | 1.368118 | <0.00001 | up |
| ENSMUSG00000022769 | Sdf2l1 | 1.757161 | <0.00001 | up |
| ENSMUSG00000100862 | Gm10925 | -2.07673 | <0.00001 | down |
| ENSMUSG00000101249 | Gm29216 | -3.28455 | <0.00001 | down |
| ENSMUSG00000019590 | Cyb561 | 3.225365 | <0.00001 | up |
| ENSMUSG00000064368 | mt-Nd6 | -1.82896 | <0.00001 | down |
| ENSMUSG00000091192 | Sardhos | -9.70866 | <0.00001 | down |
| ENSMUSG00000054408 | Spcs3 | 1.285709 | <0.00001 | up |
| ENSMUSG00000028108 | Ecm1 | -1.19413 | <0.00001 | down |
| ENSMUSG00000064358 | mt-Co3 | -2.43553 | <0.00001 | down |
| ENSMUSG00000023176 | Cpn2 | 1.447243 | <0.00001 | up |
| ENSMUSG00000064337 | mt-Rnr1 | -2.04458 | <0.00001 | down |
| ENSMUSG00000042453 | Reln | -1.58749 | <0.00001 | down |
| ENSMUSG00000024943 | Smc5 | -1.55868 | <0.00001 | down |
| ENSMUSG00000026043 | Col3a1 | -1.58397 | <0.00001 | down |
| ENSMUSG00000030111 | A2m | 6.848734 | <0.00001 | up |
| ENSMUSG00000040612 | Ildr2 | 2.123758 | <0.00001 | up |
| ENSMUSG00000046811 | Gltpd2 | 1.832344 | <0.00001 | up |
| ENSMUSG00000064367 | mt-Nd5 | -2.0481 | <0.00001 | down |
| ENSMUSG00000064351 | mt-Co1 | -2.15054 | <0.00001 | down |
| ENSMUSG00000031958 | Ldhd | 1.10534 | <0.00001 | up |
| ENSMUSG00000064345 | mt-Nd2 | -2.03365 | <0.00001 | down |
| ENSMUSG00000024131 | Slc3a1 | 2.264276 | <0.00001 | up |
| ENSMUSG00000020571 | Pdia6 | 1.792966 | <0.00001 | up |
| ENSMUSG00000064360 | mt-Nd3 | -2.60012 | <0.00001 | down |
| ENSMUSG00000038155 | Gstp2 | -4.41171 | <0.00001 | down |
| ENSMUSG00000052310 | Slc39a1 | 1.050855 | <0.00001 | up |
| ENSMUSG00000047228 | A2ml1 | 2.008721 | <0.00001 | up |
| ENSMUSG00000101111 | Gm28437 | -2.22175 | <0.00001 | down |
| ENSMUSG00000033831 | Fgb | 2.084692 | <0.00001 | up |
| ENSMUSG00000028001 | Fga | 2.324758 | <0.00001 | up |
| ENSMUSG00000002014 | Ssr4 | 1.330991 | <0.00001 | up |
| ENSMUSG00000064363 | mt-Nd4 | -2.15427 | <0.00001 | down |
| ENSMUSG00000064370 | mt-Cytb | -2.15116 | <0.00001 | down |
| ENSMUSG00000091780 | Sco2 | -1.73842 | <0.00001 | down |
| ENSMUSG00000036764 | Dnajc12 | 3.028415 | <0.00001 | up |
| ENSMUSG00000030968 | Pdilt | -2.21287 | <0.00001 | down |
| ENSMUSG00000019139 | Isyna1 | 1.888861 | <0.00001 | up |
| ENSMUSG00000021876 | Rnase4 | 1.701676 | <0.00001 | up |
| ENSMUSG00000064341 | mt-Nd1 | -2.23817 | <0.00001 | down |
| ENSMUSG00000058486 | Wdr91 | -2.01313 | <0.00001 | down |
| ENSMUSG00000033684 | Qsox1 | 1.622585 | <0.00001 | up |
| ENSMUSG00000064357 | mt-Atp6 | -2.58568 | <0.00001 | down |
| ENSMUSG00000064354 | mt-Co2 | -2.91394 | <0.00001 | down |
| ENSMUSG00000027074 | Slc43a3 | -1.68935 | <0.00001 | down |
| ENSMUSG00000102070 | Gm28661 | -2.17908 | <0.00001 | down |
| ENSMUSG00000053317 | Sec61b | 1.908486 | <0.00001 | up |
| ENSMUSG00000016024 | Lbp | 1.736422 | <0.00001 | up |
| ENSMUSG00000064246 | Chil1 | 7.845297 | <0.00001 | up |
| ENSMUSG00000024164 | C3 | 1.583293 | <0.00001 | up |
| ENSMUSG00000060591 | Ifitm2 | 1.775795 | <0.00001 | up |
| ENSMUSG00000025130 | P4hb | 1.731576 | <0.00001 | up |
| ENSMUSG00000030659 | Nucb2 | 2.206801 | <0.00001 | up |
| ENSMUSG00000032080 | Apoa4 | 3.058239 | <0.00001 | up |
| ENSMUSG00000033860 | Fgg | 2.3234 | <0.00001 | up |
| ENSMUSG00000006014 | Prg4 | 2.499685 | <0.00001 | up |
| ENSMUSG00000012428 | Steap4 | 2.416742 | <0.00001 | up |
| ENSMUSG00000072115 | Ang | 2.350208 | <0.00001 | up |
| ENSMUSG00000061947 | Serpina10 | 2.305481 | <0.00001 | up |
| ENSMUSG00000026542 | Apcs | 4.159607 | <0.00001 | up |
| ENSMUSG00000057729 | Prtn3 | 6.283251 | <0.00001 | up |
| ENSMUSG00000021091 | Serpina3n | 3.629084 | <0.00001 | up |
| ENSMUSG00000031594 | Fgl1 | 3.425031 | <0.00001 | up |
| ENSMUSG00000031722 | Hp | 2.766503 | <0.00001 | up |
| ENSMUSG00000037095 | Lrg1 | 3.724875 | <0.00001 | up |
| ENSMUSG00000030895 | Hpx | 3.098932 | <0.00001 | up |
| ENSMUSG00000021922 | Itih4 | 3.080887 | <0.00001 | up |
| ENSMUSG00000040026 | Saa3 | 4.699311 | <0.00001 | up |
| ENSMUSG00000039196 | Orm1 | 3.388251 | <0.00001 | up |
| ENSMUSG00000061540 | Orm2 | 6.121768 | <0.00001 | up |
| ENSMUSG00000026822 | Lcn2 | 8.395078 | <0.00001 | up |
| ENSMUSG00000057465 | Saa2 | 11.22827 | <0.00001 | up |
| ENSMUSG00000074115 | Saa1 | 9.400317 | <0.00001 | up |
