# Supplementary material for: Multi-time point transcriptomics and metabolomics reveal key transcription and metabolic features of hepatic ischemia-reperfusion injury in mice
Source: Genes Dis. 2024 Nov 17;12(2):101465. doi: 10.1016/j.gendis.2024.101465 (PMC11697123; doi:10.1016/j.gendis.2024.101465)
Supplement: Multimedia component 3 [file mmc3.docx]

**Table S1C.** Identified differentially expressed genes between Sham and I1R48 groups.

| **Gene ID** | **Gene name** | **Log2FC** | **P-value** | **Regulate** |
| --- | --- | --- | --- | --- |
| ENSMUSG00000055003 | Lrtm2 | 3.157471 | 0.01931 | up |
| ENSMUSG00000074796 | Slc4a11 | -4.46068 | 0.01927 | down |
| ENSMUSG00000037362 | Ccn3 | -2.61821 | 0.01920 | down |
| ENSMUSG00000024087 | Cyp1b1 | -1.13377 | 0.01918 | down |
| ENSMUSG00000069806 | Cacng7 | -1.99426 | 0.01883 | down |
| ENSMUSG00000016206 | H2-M3 | 1.3602 | 0.01883 | up |
| ENSMUSG00000099608 | 4933411E06Rik | -1.25528 | 0.01882 | down |
| ENSMUSG00000020059 | Sycp3 | -2.7773 | 0.01877 | down |
| ENSMUSG00000025610 | Map3k7cl | -3.73189 | 0.01871 | down |
| ENSMUSG00000005410 | Mcm5 | 1.476548 | 0.01863 | up |
| ENSMUSG00000049882 | Vcpkmt | 1.110477 | 0.01854 | up |
| ENSMUSG00000105655 | Gm42659 | -2.04467 | 0.01853 | down |
| ENSMUSG00000072778 | Vmn2r27 | -3.44837 | 0.01852 | down |
| ENSMUSG00000033031 | Cip2a | -1.18192 | 0.01849 | down |
| ENSMUSG00000037849 | Ifi206 | -1.34478 | 0.01847 | down |
| ENSMUSG00000035640 | Cbarp | -1.18065 | 0.01837 | down |
| ENSMUSG00000104851 | E030026E10Rik | -3.53093 | 0.01834 | down |
| ENSMUSG00000060416 | Gm839 | 3.109109 | 0.01834 | up |
| ENSMUSG00000085950 | Gm13589 | -2.93058 | 0.01829 | down |
| ENSMUSG00000023906 | Cldn6 | -1.21187 | 0.01818 | down |
| ENSMUSG00000109864 | Eid3 | -4.20386 | 0.01815 | down |
| ENSMUSG00000113214 | Gm48010 | -2.71537 | 0.01811 | down |
| ENSMUSG00000097385 | Gm26814 | -1.87556 | 0.01810 | down |
| ENSMUSG00000107499 | Ccdc142 | 1.683412 | 0.01809 | up |
| ENSMUSG00000005360 | Slc1a3 | -3.27384 | 0.01807 | down |
| ENSMUSG00000103651 | Gm37206 | 2.81361 | 0.01804 | up |
| ENSMUSG00000026090 | Cracdl | -2.68807 | 0.01797 | down |
| ENSMUSG00000042804 | Gpr153 | -1.09818 | 0.01794 | down |
| ENSMUSG00000062939 | Stat4 | -1.79659 | 0.01792 | down |
| ENSMUSG00000026825 | Dnm1 | -2.4406 | 0.01788 | down |
| ENSMUSG00000031290 | Lrch2 | -3.06457 | 0.01786 | down |
| ENSMUSG00000112627 | 4933412E12Rik | -1.59018 | 0.01782 | down |
| ENSMUSG00000109941 | Exosc6 | -7.05771 | 0.01779 | down |
| ENSMUSG00000033597 | Caskin1 | -2.12753 | 0.01772 | down |
| ENSMUSG00000042029 | Ncapg2 | 2.083288 | 0.01770 | up |
| ENSMUSG00000097755 | 2010110K18Rik | -3.5054 | 0.01761 | down |
| ENSMUSG00000014158 | Trpv4 | -1.45717 | 0.01759 | down |
| ENSMUSG00000028687 | Mutyh | -2.07949 | 0.01758 | down |
| ENSMUSG00000040264 | Gbp2b | -1.4798 | 0.01756 | down |
| ENSMUSG00000035105 | Egln3 | 1.073732 | 0.01754 | up |
| ENSMUSG00000111378 | Gm47230 | -4.18159 | 0.01754 | down |
| ENSMUSG00000042564 | Fam227a | -1.52548 | 0.01753 | down |
| ENSMUSG00000036743 | Psma8 | 1.949538 | 0.01753 | up |
| ENSMUSG00000002688 | Prkd1 | -2.81964 | 0.01750 | down |
| ENSMUSG00000119670 | Gm24305 | -3.00836 | 0.01749 | down |
| ENSMUSG00000104156 | Gm38102 | 2.248872 | 0.01747 | up |
| ENSMUSG00000049103 | Ccr2 | -1.05134 | 0.01742 | down |
| ENSMUSG00000027636 | Sla2 | -1.63263 | 0.01736 | down |
| ENSMUSG00000085781 | Gm15640 | -3.06921 | 0.01734 | down |
| ENSMUSG00000003153 | Slc2a3 | -1.70044 | 0.01724 | down |
| ENSMUSG00000046818 | Ddit4l | -1.02125 | 0.01724 | down |
| ENSMUSG00000073768 | Olfr1330 | -1.90788 | 0.01716 | down |
| ENSMUSG00000023411 | Nfatc4 | -1.57081 | 0.01712 | down |
| ENSMUSG00000084819 | Gm11967 | -1.53031 | 0.01712 | down |
| ENSMUSG00000043346 | Gm6741 | 4.237965 | 0.01708 | up |
| ENSMUSG00000037568 | Vash2 | -3.52322 | 0.01705 | down |
| ENSMUSG00000103009 | Gm56350 | 2.012961 | 0.01704 | up |
| ENSMUSG00000057246 | BC051142 | -3.07011 | 0.01700 | down |
| ENSMUSG00000084836 | Gm16274 | -3.76512 | 0.01696 | down |
| ENSMUSG00000078881 | Gm14434 | 2.549532 | 0.01692 | up |
| ENSMUSG00000118626 | Gm53040 | -4.2468 | 0.01692 | down |
| ENSMUSG00000059910 | Olfr1265 | -2.33296 | 0.01692 | down |
| ENSMUSG00000121483 | Gm20257 | -1.19562 | 0.01689 | down |
| ENSMUSG00000029283 | Cdc7 | -1.99491 | 0.01682 | down |
| ENSMUSG00000060176 | Kif27 | -3.51033 | 0.01680 | down |
| ENSMUSG00000116347 | Gm49416 | -4.53201 | 0.01675 | down |
| ENSMUSG00000112858 | Rnf212b | -4.9164 | 0.01666 | down |
| ENSMUSG00000037636 | Slc25a43 | -3.90543 | 0.01664 | down |
| ENSMUSG00000086322 | E130218I03Rik | -4.4631 | 0.01654 | down |
| ENSMUSG00000102139 | Gm37109 | -4.20345 | 0.01646 | down |
| ENSMUSG00000086561 | Gm15540 | -1.84716 | 0.01645 | down |
| ENSMUSG00000062470 | Fbxl12os | -1.93276 | 0.01644 | down |
| ENSMUSG00000071047 | Ces1a | -2.48103 | 0.01639 | down |
| ENSMUSG00000047517 | Dmbt1 | -4.18459 | 0.01637 | down |
| ENSMUSG00000120959 | - | -3.46542 | 0.01631 | down |
| ENSMUSG00000114516 | Gm46440 | -1.87957 | 0.01631 | down |
| ENSMUSG00000028950 | Tas1r1 | -3.65085 | 0.01627 | down |
| ENSMUSG00000056174 | Col8a2 | -2.13011 | 0.01627 | down |
| ENSMUSG00000002617 | Zfp40 | -1.28939 | 0.01625 | down |
| ENSMUSG00000052137 | Rbm12b2 | -1.10835 | 0.01624 | down |
| ENSMUSG00000121484 | Serpina3h | -1.69322 | 0.01622 | down |
| ENSMUSG00000118138 | Gm50322 | -2.76054 | 0.01621 | down |
| ENSMUSG00000070390 | Nlrp1b | -1.8518 | 0.01620 | down |
| ENSMUSG00000099724 | 4930554C24Rik | -2.31029 | 0.01619 | down |
| ENSMUSG00000117070 | Gm41541 | 3.154623 | 0.01618 | up |
| ENSMUSG00000103258 | Gm37518 | -2.88645 | 0.01615 | down |
| ENSMUSG00000028278 | Rragd | -1.14163 | 0.01612 | down |
| ENSMUSG00000098505 | Gm14496 | -2.57779 | 0.01611 | down |
| ENSMUSG00000022768 | Ccdc116 | -3.47528 | 0.01609 | down |
| ENSMUSG00000104876 | Trdc | -2.59754 | 0.01608 | down |
| ENSMUSG00000084154 | Gm15644 | -4.56889 | 0.01606 | down |
| ENSMUSG00000028885 | Smpdl3b | 2.272452 | 0.01606 | up |
| ENSMUSG00000103220 | Gm37728 | -3.2949 | 0.01599 | down |
| ENSMUSG00000020218 | Wif1 | 3.399983 | 0.01598 | up |
| ENSMUSG00000106197 | Gm42717 | -2.60478 | 0.01596 | down |
| ENSMUSG00000039153 | Runx2 | -1.6223 | 0.01596 | down |
| ENSMUSG00000111709 | Gm3776 | 2.392827 | 0.01594 | up |
| ENSMUSG00000027115 | Kif18a | -1.69943 | 0.01589 | down |
| ENSMUSG00000085218 | BB218582 | -3.59678 | 0.01579 | down |
| ENSMUSG00000068231 | Vmn1r43 | -2.68074 | 0.01570 | down |
| ENSMUSG00000019971 | Cep290 | -1.21849 | 0.01569 | down |
| ENSMUSG00000055202 | Zfp811 | -3.1111 | 0.01569 | down |
| ENSMUSG00000121255 | - | -2.79595 | 0.01569 | down |
| ENSMUSG00000097534 | Gm16675 | 1.56412 | 0.01567 | up |
| ENSMUSG00000110902 | Gm33104 | -2.94799 | 0.01555 | down |
| ENSMUSG00000073931 | Olfr646 | -3.89482 | 0.01553 | down |
| ENSMUSG00000020758 | Itgb4 | -1.12589 | 0.01543 | down |
| ENSMUSG00000084844 | Hoxb3os | -1.6187 | 0.01543 | down |
| ENSMUSG00000115647 | Gm49265 | -3.21856 | 0.01539 | down |
| ENSMUSG00000072955 | Tmsb15l | 2.488379 | 0.01535 | up |
| ENSMUSG00000121222 | - | -3.91217 | 0.01525 | down |
| ENSMUSG00000104806 | Gm42566 | -1.41003 | 0.01525 | down |
| ENSMUSG00000097316 | Gm10516 | 1.514369 | 0.01522 | up |
| ENSMUSG00000120267 | - | -4.14411 | 0.01522 | down |
| ENSMUSG00000017146 | Brca1 | 1.742714 | 0.01520 | up |
| ENSMUSG00000067147 | Rpl7a-ps11 | -2.67911 | 0.01501 | down |
| ENSMUSG00000112122 | Gm47628 | -1.89368 | 0.01494 | down |
| ENSMUSG00000047786 | Lix1 | -1.55082 | 0.01494 | down |
| ENSMUSG00000098975 | Gm27177 | -1.62003 | 0.01490 | down |
| ENSMUSG00000112859 | Gm48522 | -3.54448 | 0.01488 | down |
| ENSMUSG00000097656 | Gm26712 | -1.68259 | 0.01483 | down |
| ENSMUSG00000038550 | Ciart | 1.524026 | 0.01471 | up |
| ENSMUSG00000066952 | Myo1h | -3.91295 | 0.01465 | down |
| ENSMUSG00000020032 | Nuak1 | -1.12392 | 0.01464 | down |
| ENSMUSG00000025163 | Cd7 | -1.68539 | 0.01463 | down |
| ENSMUSG00000018924 | Alox15 | -3.92411 | 0.01457 | down |
| ENSMUSG00000104664 | Idi1-ps2 | 2.607829 | 0.01455 | up |
| ENSMUSG00000033467 | Crlf2 | 1.105457 | 0.01443 | up |
| ENSMUSG00000048520 | Fbxl13 | -4.57143 | 0.01439 | down |
| ENSMUSG00000104706 | Gm43421 | -4.20695 | 0.01436 | down |
| ENSMUSG00000031907 | Zfp90 | -1.05101 | 0.01430 | down |
| ENSMUSG00000021234 | Fam161b | -1.79692 | 0.01429 | down |
| ENSMUSG00000033949 | Trim36 | -2.44961 | 0.01426 | down |
| ENSMUSG00000006360 | Crip1 | -1.003 | 0.01421 | down |
| ENSMUSG00000111892 | Gm47641 | -3.04878 | 0.01420 | down |
| ENSMUSG00000107091 | Gm43343 | -4.47283 | 0.01418 | down |
| ENSMUSG00000001983 | Taco1 | 1.106955 | 0.01417 | up |
| ENSMUSG00000031548 | Sfrp1 | -1.53481 | 0.01413 | down |
| ENSMUSG00000057156 | Homez | 1.242153 | 0.01413 | up |
| ENSMUSG00000101959 | Ldhal6b | -4.2281 | 0.01411 | down |
| ENSMUSG00000120758 | - | -3.9519 | 0.01405 | down |
| ENSMUSG00000104324 | Gm37320 | -1.95542 | 0.01402 | down |
| ENSMUSG00000086782 | E130102H24Rik | 1.132751 | 0.01401 | up |
| ENSMUSG00000103030 | E330011M16Rik | -1.74295 | 0.01384 | down |
| ENSMUSG00000051687 | Vmn1r73 | -4.60184 | 0.01381 | down |
| ENSMUSG00000024793 | Tnfrsf25 | -2.29609 | 0.01376 | down |
| ENSMUSG00000108356 | 9130221F21Rik | -4.21908 | 0.01376 | down |
| ENSMUSG00000050921 | P2ry10 | -1.91193 | 0.01372 | down |
| ENSMUSG00000064032 | Gm10143 | -3.04672 | 0.01371 | down |
| ENSMUSG00000021390 | Ogn | -1.29699 | 0.01366 | down |
| ENSMUSG00000120319 | - | -5.04578 | 0.01363 | down |
| ENSMUSG00000029769 | Ccdc136 | -3.05096 | 0.01363 | down |
| ENSMUSG00000106205 | C230096K16Rik | -1.51458 | 0.01360 | down |
| ENSMUSG00000037860 | Aim2 | -1.43193 | 0.01359 | down |
| ENSMUSG00002075045 | Gm54751 | -4.04197 | 0.01359 | down |
| ENSMUSG00002076697 | Gm54987 | -4.04197 | 0.01359 | down |
| ENSMUSG00000020335 | Zfp354b | -1.35831 | 0.01356 | down |
| ENSMUSG00000031377 | Bmx | -2.46975 | 0.01355 | down |
| ENSMUSG00000048895 | Cdk5r1 | -1.6311 | 0.01351 | down |
| ENSMUSG00000021974 | Fgf9 | -2.3135 | 0.01342 | down |
| ENSMUSG00000003411 | Rab3b | 1.701109 | 0.01340 | up |
| ENSMUSG00000007207 | Stx1a | -1.66693 | 0.01340 | down |
| ENSMUSG00000044702 | Palb2 | -1.77823 | 0.01339 | down |
| ENSMUSG00000093598 | A730085K08Rik | -3.68599 | 0.01339 | down |
| ENSMUSG00000114882 | Gm48581 | -4.01183 | 0.01338 | down |
| ENSMUSG00000039137 | Whrn | -1.80089 | 0.01337 | down |
| ENSMUSG00000037784 | Dzip1l | -1.26055 | 0.01330 | down |
| ENSMUSG00000107335 | Gm43372 | 3.179814 | 0.01327 | up |
| ENSMUSG00000049303 | Syt12 | 1.463076 | 0.01324 | up |
| ENSMUSG00000007030 | Vwa7 | -2.04234 | 0.01323 | down |
| ENSMUSG00000120914 | - | -2.59509 | 0.01321 | down |
| ENSMUSG00000032322 | Pstpip1 | -1.52256 | 0.01320 | down |
| ENSMUSG00000037085 | Trmt12 | 1.056466 | 0.01319 | up |
| ENSMUSG00000043770 | Gm12481 | -1.41436 | 0.01319 | down |
| ENSMUSG00000031257 | Nox1 | -2.84706 | 0.01313 | down |
| ENSMUSG00000039043 | Arpin | 1.091631 | 0.01309 | up |
| ENSMUSG00000018238 | Gdf9 | -2.49809 | 0.01307 | down |
| ENSMUSG00000032609 | Klhdc8b | -1.07164 | 0.01305 | down |
| ENSMUSG00000070287 | Slc35g2 | -1.37599 | 0.01301 | down |
| ENSMUSG00000028696 | Ipp | 1.027986 | 0.01300 | up |
| ENSMUSG00000112112 | Gm48508 | -1.94747 | 0.01298 | down |
| ENSMUSG00000043931 | Gimap7 | -1.65247 | 0.01296 | down |
| ENSMUSG00000114133 | Btf3l4b | -3.20227 | 0.01291 | down |
| ENSMUSG00000091243 | Vgll3 | -1.79561 | 0.01291 | down |
| ENSMUSG00000097785 | B230217O12Rik | -1.02885 | 0.01287 | down |
| ENSMUSG00000052131 | Akr1b7 | 1.774984 | 0.01286 | up |
| ENSMUSG00000056671 | Prelid2 | 3.004224 | 0.01286 | up |
| ENSMUSG00000081557 | Gm5697 | -3.04033 | 0.01284 | down |
| ENSMUSG00000109125 | Gm45159 | -2.05053 | 0.01276 | down |
| ENSMUSG00000117290 | Gm34883 | -4.34034 | 0.01272 | down |
| ENSMUSG00000107835 | Gm43999 | -2.36564 | 0.01271 | down |
| ENSMUSG00000108394 | Gm45477 | -2.04685 | 0.01270 | down |
| ENSMUSG00000061186 | Sfmbt2 | -3.85728 | 0.01268 | down |
| ENSMUSG00000100775 | Gm29107 | -1.71189 | 0.01265 | down |
| ENSMUSG00000113065 | Gm48870 | -3.63958 | 0.01265 | down |
| ENSMUSG00000082465 | Rps6-ps3 | -2.533 | 0.01261 | down |
| ENSMUSG00000013921 | Clip3 | -1.95293 | 0.01257 | down |
| ENSMUSG00000112571 | Gm48207 | -3.32889 | 0.01257 | down |
| ENSMUSG00000121088 | - | 1.245435 | 0.01254 | up |
| ENSMUSG00000090936 | Gm17705 | -1.86638 | 0.01254 | down |
| ENSMUSG00000050605 | Zfp61 | -1.46306 | 0.01253 | down |
| ENSMUSG00000057594 | Arl16 | 1.027529 | 0.01251 | up |
| ENSMUSG00000023968 | Crip3 | -2.62739 | 0.01250 | down |
| ENSMUSG00000023393 | Slc17a9 | -1.08326 | 0.01249 | down |
| ENSMUSG00000032254 | Kif23 | 2.057795 | 0.01247 | up |
| ENSMUSG00000103822 | 6030460B20Rik | -3.12485 | 0.01244 | down |
| ENSMUSG00000042826 | Fgf11 | -1.25689 | 0.01243 | down |
| ENSMUSG00000102718 | Gm37761 | -3.0135 | 0.01243 | down |
| ENSMUSG00000025489 | Ifitm5 | -2.79521 | 0.01240 | down |
| ENSMUSG00000085887 | Arhgap27os3 | -2.82938 | 0.01240 | down |
| ENSMUSG00000000531 | Tamalin | -1.43213 | 0.01238 | down |
| ENSMUSG00000038152 | 5033430I15Rik | -1.71639 | 0.01235 | down |
| ENSMUSG00000048012 | Zfp473 | -2.64933 | 0.01232 | down |
| ENSMUSG00000120196 | - | -2.58602 | 0.01231 | down |
| ENSMUSG00000106705 | Gm2602 | -1.65893 | 0.01226 | down |
| ENSMUSG00000043153 | Crppa | 1.686204 | 0.01226 | up |
| ENSMUSG00000054942 | Miga1 | -1.25473 | 0.01225 | down |
| ENSMUSG00000112454 | Gm48619 | -2.78553 | 0.01220 | down |
| ENSMUSG00000052920 | Prkg1 | -1.24733 | 0.01219 | down |
| ENSMUSG00000034110 | Kctd7 | 1.129233 | 0.01219 | up |
| ENSMUSG00000003849 | Nqo1 | 1.011681 | 0.01215 | up |
| ENSMUSG00000030353 | Tead4 | -2.497 | 0.01214 | down |
| ENSMUSG00000121008 | - | -1.83495 | 0.01211 | down |
| ENSMUSG00000102307 | Gm38194 | 1.577571 | 0.01208 | up |
| ENSMUSG00000071073 | Lrrc73 | -2.48884 | 0.01207 | down |
| ENSMUSG00000116581 | Gm46565 | -1.75841 | 0.01205 | down |
| ENSMUSG00000118106 | Gm54639 | 2.013514 | 0.01205 | up |
| ENSMUSG00000041372 | B4galnt3 | 2.038441 | 0.01202 | up |
| ENSMUSG00000050195 | Scd4 | -3.31814 | 0.01201 | down |
| ENSMUSG00000068964 | Gm6361 | 4.144505 | 0.01198 | up |
| ENSMUSG00000032549 | Rab6b | -1.07467 | 0.01193 | down |
| ENSMUSG00000019990 | Pde7b | -1.44841 | 0.01192 | down |
| ENSMUSG00000104503 | Gm37738 | -1.46555 | 0.01191 | down |
| ENSMUSG00000020691 | Mettl2 | 1.206819 | 0.01189 | up |
| ENSMUSG00000033368 | Trim69 | -4.31858 | 0.01185 | down |
| ENSMUSG00000039521 | Foxp3 | -2.52358 | 0.01183 | down |
| ENSMUSG00000038248 | Sobp | -2.11486 | 0.01182 | down |
| ENSMUSG00000074576 | Mocs3 | 1.067336 | 0.01180 | up |
| ENSMUSG00000052673 | Gm9887 | 2.786951 | 0.01179 | up |
| ENSMUSG00000103065 | Gm20236 | -2.84333 | 0.01174 | down |
| ENSMUSG00000049588 | Ccdc69 | -1.86964 | 0.01169 | down |
| ENSMUSG00000093416 | Gm18294 | -4.74243 | 0.01166 | down |
| ENSMUSG00000020303 | Stc2 | -1.75853 | 0.01165 | down |
| ENSMUSG00000087026 | A230103J11Rik | 2.547176 | 0.01162 | up |
| ENSMUSG00000026303 | Mlph | -1.34647 | 0.01161 | down |
| ENSMUSG00000042874 | D930007J09Rik | 3.3611 | 0.01161 | up |
| ENSMUSG00000051279 | Gdf6 | -1.6443 | 0.01158 | down |
| ENSMUSG00000028018 | Gstcd | 1.28672 | 0.01153 | up |
| ENSMUSG00000039904 | Gpr37 | -4.65605 | 0.01152 | down |
| ENSMUSG00000024353 | Mzb1 | -2.77419 | 0.01149 | down |
| ENSMUSG00000108852 | Gm44911 | -4.33907 | 0.01147 | down |
| ENSMUSG00000097761 | 4930534D22Rik | -4.30235 | 0.01139 | down |
| ENSMUSG00000112860 | Gm47439 | -4.0625 | 0.01138 | down |
| ENSMUSG00000085603 | Gm11346 | -2.61694 | 0.01138 | down |
| ENSMUSG00000086012 | Gm15902 | -2.77341 | 0.01136 | down |
| ENSMUSG00000048728 | Zfp454 | -1.17732 | 0.01134 | down |
| ENSMUSG00000032017 | Grik4 | -3.50216 | 0.01133 | down |
| ENSMUSG00000120201 | - | -4.05066 | 0.01117 | down |
| ENSMUSG00000022262 | Dnah5 | 3.394873 | 0.01114 | up |
| ENSMUSG00000055872 | Gm9985 | -3.46114 | 0.01110 | down |
| ENSMUSG00000074824 | Rslcan18 | -1.3047 | 0.01108 | down |
| ENSMUSG00000051341 | Zfp52 | -2.21553 | 0.01106 | down |
| ENSMUSG00000022469 | Rapgef3 | -1.1472 | 0.01105 | down |
| ENSMUSG00000040907 | Atp1a3 | 3.651723 | 0.01105 | up |
| ENSMUSG00000112654 | 4930455C13Rik | -4.67612 | 0.01102 | down |
| ENSMUSG00000102856 | Gm37084 | -3.67125 | 0.01096 | down |
| ENSMUSG00000110317 | Gm45250 | -1.1438 | 0.01096 | down |
| ENSMUSG00000028357 | Kif12 | -1.63585 | 0.01094 | down |
| ENSMUSG00000049357 | Brd8dc | -3.16279 | 0.01093 | down |
| ENSMUSG00000027843 | Ptpn22 | -1.81462 | 0.01089 | down |
| ENSMUSG00000022032 | Scara5 | 1.956045 | 0.01086 | up |
| ENSMUSG00000086279 | Gm15634 | 2.296729 | 0.01082 | up |
| ENSMUSG00000121103 | - | -4.14334 | 0.01081 | down |
| ENSMUSG00000056995 | Olfr1178 | -4.36946 | 0.01079 | down |
| ENSMUSG00000104660 | Gm43601 | -3.82512 | 0.01078 | down |
| ENSMUSG00000038888 | Ctu1 | 1.099272 | 0.01077 | up |
| ENSMUSG00000120013 | - | 2.686246 | 0.01075 | up |
| ENSMUSG00000112304 | Gm35206 | -3.37996 | 0.01074 | down |
| ENSMUSG00000074607 | Tox2 | -1.26008 | 0.01071 | down |
| ENSMUSG00000104708 | Gm42503 | -2.14196 | 0.01071 | down |
| ENSMUSG00000097149 | G630030J09Rik | -4.0853 | 0.01067 | down |
| ENSMUSG00000027698 | Nceh1 | 1.036602 | 0.01065 | up |
| ENSMUSG00000102476 | Gm37245 | -2.57415 | 0.01064 | down |
| ENSMUSG00000111715 | Olfr1246 | -3.32075 | 0.01064 | down |
| ENSMUSG00000028295 | Smim8 | 1.25289 | 0.01061 | up |
| ENSMUSG00000108059 | Gm44369 | -2.62534 | 0.01059 | down |
| ENSMUSG00000009092 | Derl3 | -1.99475 | 0.01059 | down |
| ENSMUSG00000054304 | D130007C19Rik | -3.16483 | 0.01058 | down |
| ENSMUSG00000105161 | Gm42595 | -1.54109 | 0.01054 | down |
| ENSMUSG00000022489 | Pde1b | -1.48517 | 0.01051 | down |
| ENSMUSG00000099746 | Ppnr | -2.77736 | 0.01044 | down |
| ENSMUSG00000024743 | Syt7 | -1.09667 | 0.01041 | down |
| ENSMUSG00000026077 | Npas2 | -2.3473 | 0.01039 | down |
| ENSMUSG00000040183 | Ankrd6 | -2.79812 | 0.01038 | down |
| ENSMUSG00000109118 | Gm32031 | -1.19722 | 0.01037 | down |
| ENSMUSG00000005980 | Dnase1 | -1.71893 | 0.01034 | down |
| ENSMUSG00000112142 | Gm47022 | -2.80269 | 0.01029 | down |
| ENSMUSG00000116810 | Gm32624 | 1.242347 | 0.01028 | up |
| ENSMUSG00000049871 | Nlrc3 | -1.48172 | 0.01027 | down |
| ENSMUSG00000030401 | Rtn2 | -1.41537 | 0.01021 | down |
| ENSMUSG00000119989 | Gm39213 | -1.01701 | 0.01020 | down |
| ENSMUSG00000108095 | Gm44067 | -3.40709 | 0.01015 | down |
| ENSMUSG00000083246 | Gm11839 | -2.57296 | 0.01014 | down |
| ENSMUSG00000113921 | Gm48054 | -1.39273 | 0.01013 | down |
| ENSMUSG00000060962 | Dmkn | 2.833673 | 0.01010 | up |
| ENSMUSG00000069378 | Prdm6 | -2.57528 | 0.01010 | down |
| ENSMUSG00000055254 | Ntrk2 | -1.72114 | 0.01009 | down |
| ENSMUSG00000119132 | Gm24407 | -3.00825 | 0.01004 | down |
| ENSMUSG00000113137 | Gm7511 | -3.36038 | 0.00998 | down |
| ENSMUSG00000119946 | - | -1.95223 | 0.00995 | down |
| ENSMUSG00000106676 | Gm42895 | -4.77658 | 0.00994 | down |
| ENSMUSG00000106628 | Gm43558 | -2.93823 | 0.00993 | down |
| ENSMUSG00000079444 | Gm21981 | -2.14976 | 0.00992 | down |
| ENSMUSG00000040663 | Clcf1 | -1.2874 | 0.00991 | down |
| ENSMUSG00000028476 | Reck | -1.07323 | 0.00984 | down |
| ENSMUSG00000043263 | Ifi209 | -1.16652 | 0.00982 | down |
| ENSMUSG00000031283 | Chrdl1 | -2.10862 | 0.00979 | down |
| ENSMUSG00000114937 | Gm30054 | -2.29723 | 0.00978 | down |
| ENSMUSG00000097006 | 9530082P21Rik | -1.12818 | 0.00976 | down |
| ENSMUSG00000033855 | Ston1 | -1.21261 | 0.00975 | down |
| ENSMUSG00000104069 | Gm37198 | -3.4548 | 0.00974 | down |
| ENSMUSG00000093954 | Gm16867 | 3.620142 | 0.00973 | up |
| ENSMUSG00000022676 | Snai2 | 1.080289 | 0.00973 | up |
| ENSMUSG00000032171 | Pin1 | 1.047348 | 0.00970 | up |
| ENSMUSG00000038233 | Gask1a | -1.18606 | 0.00970 | down |
| ENSMUSG00000103835 | Gm37612 | -2.82296 | 0.00968 | down |
| ENSMUSG00000028005 | Gucy1b1 | -1.08685 | 0.00967 | down |
| ENSMUSG00000039994 | Timeless | -1.01182 | 0.00967 | down |
| ENSMUSG00000056978 | Hamp2 | -1.28108 | 0.00967 | down |
| ENSMUSG00000042351 | Grap2 | -1.52518 | 0.00965 | down |
| ENSMUSG00000105547 | Iglc3 | -2.07467 | 0.00960 | down |
| ENSMUSG00000062783 | Csprs | -2.23736 | 0.00958 | down |
| ENSMUSG00000117725 | Gm50240 | -1.1806 | 0.00958 | down |
| ENSMUSG00000087968 | Gm25395 | -3.43011 | 0.00952 | down |
| ENSMUSG00000040875 | Osbpl10 | -2.98558 | 0.00952 | down |
| ENSMUSG00000060594 | Layn | -1.84153 | 0.00946 | down |
| ENSMUSG00000040187 | Arntl2 | -1.24488 | 0.00941 | down |
| ENSMUSG00000082724 | Gm14416 | 1.625323 | 0.00939 | up |
| ENSMUSG00000020083 | Fam241b | 1.657406 | 0.00936 | up |
| ENSMUSG00000114103 | Gm8557 | -4.07767 | 0.00934 | down |
| ENSMUSG00000111585 | Gm47355 | -1.29808 | 0.00933 | down |
| ENSMUSG00000027656 | Ccn5 | -1.97903 | 0.00932 | down |
| ENSMUSG00000121207 | - | -3.30448 | 0.00931 | down |
| ENSMUSG00000075062 | Olfr1271 | -2.59974 | 0.00923 | down |
| ENSMUSG00000085735 | Mettl5os | -3.30003 | 0.00919 | down |
| ENSMUSG00000109697 | Gm34030 | -2.37021 | 0.00919 | down |
| ENSMUSG00000022526 | Zfp251 | -1.04589 | 0.00914 | down |
| ENSMUSG00000091831 | Gm4707 | -2.46867 | 0.00909 | down |
| ENSMUSG00000037375 | Hhat | 1.845637 | 0.00905 | up |
| ENSMUSG00000039853 | Trim14 | 1.065621 | 0.00903 | up |
| ENSMUSG00000112126 | Gm34544 | -4.53787 | 0.00901 | down |
| ENSMUSG00000112294 | Gm4129 | 2.123252 | 0.00900 | up |
| ENSMUSG00000004864 | Mapk13 | -1.5063 | 0.00899 | down |
| ENSMUSG00000022223 | Sdr39u1 | 1.73946 | 0.00899 | up |
| ENSMUSG00000022960 | Donson | -1.00874 | 0.00898 | down |
| ENSMUSG00000102577 | Gm37969 | -3.2122 | 0.00898 | down |
| ENSMUSG00000028068 | Iqgap3 | -2.20171 | 0.00897 | down |
| ENSMUSG00000030889 | Vwa3a | -4.15548 | 0.00897 | down |
| ENSMUSG00000092569 | Gm20544 | -1.11881 | 0.00895 | down |
| ENSMUSG00000043300 | B3galnt1 | 2.126393 | 0.00894 | up |
| ENSMUSG00000028654 | Mycl | 1.051871 | 0.00888 | up |
| ENSMUSG00000047369 | Dnah14 | -2.16851 | 0.00887 | down |
| ENSMUSG00000120158 | - | -3.79297 | 0.00886 | down |
| ENSMUSG00000030703 | Gdpd3 | -2.59305 | 0.00885 | down |
| ENSMUSG00000014303 | Glis2 | -1.01498 | 0.00883 | down |
| ENSMUSG00000092323 | BB365896 | -3.67365 | 0.00882 | down |
| ENSMUSG00000052736 | Klrc2 | -2.8623 | 0.00881 | down |
| ENSMUSG00000091867 | Cyp2a22 | -1.83399 | 0.00879 | down |
| ENSMUSG00000004038 | Gstm3 | 1.306645 | 0.00879 | up |
| ENSMUSG00000120461 | - | -2.54584 | 0.00878 | down |
| ENSMUSG00000031197 | Vbp1 | 1.139697 | 0.00875 | up |
| ENSMUSG00000112101 | Gm47924 | -4.8926 | 0.00871 | down |
| ENSMUSG00000003452 | Bicd1 | -1.56561 | 0.00871 | down |
| ENSMUSG00000106229 | Gm19409 | -4.47331 | 0.00867 | down |
| ENSMUSG00000028289 | Epha7 | -1.40892 | 0.00867 | down |
| ENSMUSG00000097536 | 2610037D02Rik | -1.53072 | 0.00865 | down |
| ENSMUSG00000034910 | Pygo1 | -1.74927 | 0.00864 | down |
| ENSMUSG00000103748 | Gm38243 | -2.53086 | 0.00864 | down |
| ENSMUSG00000107094 | Gm43294 | -1.47109 | 0.00862 | down |
| ENSMUSG00000085169 | Gm10785 | 3.094128 | 0.00861 | up |
| ENSMUSG00000120574 | - | -1.9437 | 0.00861 | down |
| ENSMUSG00000000386 | Mx1 | -1.57494 | 0.00857 | down |
| ENSMUSG00000040432 | Ltb4r2 | 2.501229 | 0.00855 | up |
| ENSMUSG00000031489 | Adrb3 | -1.02661 | 0.00852 | down |
| ENSMUSG00000000031 | H19 | 1.893288 | 0.00851 | up |
| ENSMUSG00000084166 | Gm6451 | 3.086924 | 0.00850 | up |
| ENSMUSG00000022074 | Tnfrsf10b | -1.88271 | 0.00850 | down |
| ENSMUSG00000034645 | Zyg11a | 1.987211 | 0.00847 | up |
| ENSMUSG00000062661 | Ncs1 | -2.05657 | 0.00846 | down |
| ENSMUSG00000031227 | Magee1 | 1.653493 | 0.00845 | up |
| ENSMUSG00000026525 | Opn3 | -1.1538 | 0.00839 | down |
| ENSMUSG00000049561 | Olfr95 | -3.89149 | 0.00839 | down |
| ENSMUSG00000120092 | - | -1.51282 | 0.00836 | down |
| ENSMUSG00000048732 | Klhl11 | 3.113092 | 0.00835 | up |
| ENSMUSG00000039264 | Gimap3 | -1.12578 | 0.00835 | down |
| ENSMUSG00000121319 | - | 1.503908 | 0.00832 | up |
| ENSMUSG00000110781 | Gm31992 | 1.695883 | 0.00831 | up |
| ENSMUSG00000120242 | - | -2.40847 | 0.00830 | down |
| ENSMUSG00000083822 | Hmgb1-ps5 | -4.19767 | 0.00826 | down |
| ENSMUSG00000062210 | Tnfaip8 | -1.22749 | 0.00822 | down |
| ENSMUSG00000031478 | Nek3 | -1.13043 | 0.00821 | down |
| ENSMUSG00000023915 | Tnfrsf21 | 1.191 | 0.00820 | up |
| ENSMUSG00000028990 | Lzic | 1.01827 | 0.00816 | up |
| ENSMUSG00000038208 | Pgap3 | 1.409453 | 0.00815 | up |
| ENSMUSG00000104713 | Gbp6 | -1.11819 | 0.00815 | down |
| ENSMUSG00000039110 | Mycbpap | -3.04519 | 0.00813 | down |
| ENSMUSG00000032413 | Rasa2 | -1.03822 | 0.00810 | down |
| ENSMUSG00000000791 | Il12rb1 | 1.851067 | 0.00809 | up |
| ENSMUSG00000041939 | Mvk | 1.485734 | 0.00806 | up |
| ENSMUSG00000115389 | Gm48936 | -3.77734 | 0.00806 | down |
| ENSMUSG00000039116 | Adgrg6 | -1.31232 | 0.00804 | down |
| ENSMUSG00000094306 | Gm24924 | -3.52426 | 0.00804 | down |
| ENSMUSG00000118796 | n-R5s138 | -4.19805 | 0.00801 | down |
| ENSMUSG00000079355 | Ackr4 | -1.08517 | 0.00800 | down |
| ENSMUSG00000085936 | 2610307P16Rik | -2.4211 | 0.00795 | down |
| ENSMUSG00000063234 | Gpr84 | 3.181086 | 0.00784 | up |
| ENSMUSG00000114102 | Gm48048 | -4.50809 | 0.00783 | down |
| ENSMUSG00000112449 | Srp54b | 2.328816 | 0.00780 | up |
| ENSMUSG00000036533 | Cdc42ep3 | -1.25036 | 0.00777 | down |
| ENSMUSG00000121023 | - | -1.74163 | 0.00777 | down |
| ENSMUSG00000085250 | Gm15234 | -2.55607 | 0.00775 | down |
| ENSMUSG00000085445 | Gm16348 | -1.06928 | 0.00773 | down |
| ENSMUSG00000027570 | Col9a3 | -2.482 | 0.00771 | down |
| ENSMUSG00000103160 | C130012C08Rik | -2.34648 | 0.00770 | down |
| ENSMUSG00000042155 | Klhl23 | -1.69438 | 0.00765 | down |
| ENSMUSG00000024403 | Atp6v1g2 | -1.04328 | 0.00763 | down |
| ENSMUSG00000062312 | Erbb2 | -1.18262 | 0.00759 | down |
| ENSMUSG00000115575 | Gm49024 | -1.54785 | 0.00757 | down |
| ENSMUSG00000002458 | Rgs19 | -1.00432 | 0.00753 | down |
| ENSMUSG00000039209 | Rpl39l | -4.44917 | 0.00753 | down |
| ENSMUSG00000043671 | Dpy19l3 | 1.101001 | 0.00752 | up |
| ENSMUSG00000047898 | Ccr4 | -3.05232 | 0.00752 | down |
| ENSMUSG00000034041 | Lyl1 | -1.57485 | 0.00745 | down |
| ENSMUSG00000042942 | Greb1l | -1.57787 | 0.00745 | down |
| ENSMUSG00000120442 | - | -1.65735 | 0.00743 | down |
| ENSMUSG00000120414 | Gm32261 | -2.92337 | 0.00737 | down |
| ENSMUSG00000114598 | D130062J10Rik | -2.63804 | 0.00736 | down |
| ENSMUSG00000056004 | Elapor2 | 3.044046 | 0.00727 | up |
| ENSMUSG00000073682 | Gm10563 | -4.62735 | 0.00726 | down |
| ENSMUSG00000044231 | Nhlrc1 | 1.813097 | 0.00722 | up |
| ENSMUSG00000068606 | Gm4841 | -2.08016 | 0.00721 | down |
| ENSMUSG00000031258 | Xkrx | -2.69266 | 0.00721 | down |
| ENSMUSG00000017861 | Mybl2 | -1.83829 | 0.00720 | down |
| ENSMUSG00000060771 | Tsga10 | -1.26079 | 0.00719 | down |
| ENSMUSG00000050621 | Rps27rt | -3.13546 | 0.00714 | down |
| ENSMUSG00000000567 | Sox9 | -1.065 | 0.00713 | down |
| ENSMUSG00000100750 | Gm29084 | 1.128441 | 0.00713 | up |
| ENSMUSG00000091021 | Gm17300 | -1.0672 | 0.00713 | down |
| ENSMUSG00000115816 | Gm34589 | -3.69273 | 0.00712 | down |
| ENSMUSG00000020027 | Socs2 | -1.65278 | 0.00711 | down |
| ENSMUSG00000040751 | Lat2 | -2.39461 | 0.00711 | down |
| ENSMUSG00000034570 | Inpp5j | -4.18831 | 0.00711 | down |
| ENSMUSG00000043664 | Tmem221 | -2.6335 | 0.00709 | down |
| ENSMUSG00000088252 | Snord13 | -3.30521 | 0.00707 | down |
| ENSMUSG00000107876 | Gm43936 | -3.85751 | 0.00706 | down |
| ENSMUSG00000039629 | Strip2 | -3.13442 | 0.00703 | down |
| ENSMUSG00000019278 | Dpep1 | -1.65005 | 0.00701 | down |
| ENSMUSG00000058163 | Gm5431 | -1.85421 | 0.00697 | down |
| ENSMUSG00000121038 | - | -3.68684 | 0.00697 | down |
| ENSMUSG00000034209 | Rasl10a | -3.31689 | 0.00696 | down |
| ENSMUSG00000052525 | Spdya | -1.84061 | 0.00695 | down |
| ENSMUSG00000051984 | Sec31b | -1.06188 | 0.00694 | down |
| ENSMUSG00000032776 | Mctp2 | 1.07306 | 0.00694 | up |
| ENSMUSG00000029916 | Agk | 1.773855 | 0.00691 | up |
| ENSMUSG00000107306 | Gm42577 | -1.00301 | 0.00690 | down |
| ENSMUSG00000033342 | Plppr5 | -2.5481 | 0.00689 | down |
| ENSMUSG00000030483 | Cyp2b10 | 2.213746 | 0.00688 | up |
| ENSMUSG00000108931 | Olfr1284 | -2.45234 | 0.00686 | down |
| ENSMUSG00000020038 | Cry1 | -1.14464 | 0.00686 | down |
| ENSMUSG00000066191 | Anks6 | -2.14079 | 0.00685 | down |
| ENSMUSG00000021047 | Nova1 | -4.22037 | 0.00682 | down |
| ENSMUSG00000041134 | Cyyr1 | -1.00302 | 0.00678 | down |
| ENSMUSG00000111365 | Gm47772 | -1.65421 | 0.00677 | down |
| ENSMUSG00000015452 | Ager | -2.13048 | 0.00676 | down |
| ENSMUSG00000120811 | - | 3.275241 | 0.00675 | up |
| ENSMUSG00000059659 | Gm10069 | -2.04883 | 0.00673 | down |
| ENSMUSG00000002771 | Grin2d | -2.64695 | 0.00673 | down |
| ENSMUSG00000038793 | Lefty1 | 2.97281 | 0.00673 | up |
| ENSMUSG00000085628 | Appbp2os | 3.370469 | 0.00672 | up |
| ENSMUSG00000072258 | Taf1a | -1.06372 | 0.00671 | down |
| ENSMUSG00000031732 | Phlpp2 | -1.0029 | 0.00666 | down |
| ENSMUSG00000047854 | Stx19 | -4.91857 | 0.00665 | down |
| ENSMUSG00000027860 | Vangl1 | 1.065312 | 0.00664 | up |
| ENSMUSG00000107549 | Gm43961 | -5.83507 | 0.00660 | down |
| ENSMUSG00000097494 | 4933406C10Rik | -3.20515 | 0.00658 | down |
| ENSMUSG00000030222 | Rerg | -1.06037 | 0.00658 | down |
| ENSMUSG00000021208 | Ifi27l2b | 1.89867 | 0.00658 | up |
| ENSMUSG00000087575 | Gm12976 | -1.93185 | 0.00656 | down |
| ENSMUSG00000049721 | Gal3st1 | 1.472172 | 0.00652 | up |
| ENSMUSG00000021217 | Tshz3 | -1.57598 | 0.00649 | down |
| ENSMUSG00000113978 | Gm48260 | -1.94092 | 0.00647 | down |
| ENSMUSG00000097416 | Gm26670 | -1.92262 | 0.00646 | down |
| ENSMUSG00000027737 | Slc7a11 | -2.97362 | 0.00646 | down |
| ENSMUSG00000031637 | Lrp2bp | -1.1193 | 0.00645 | down |
| ENSMUSG00000083672 | Kpna2-ps | 3.327784 | 0.00644 | up |
| ENSMUSG00000073538 | E330020D12Rik | -2.3603 | 0.00642 | down |
| ENSMUSG00000025916 | Ppp1r42 | 2.332798 | 0.00641 | up |
| ENSMUSG00000030276 | Ttll3 | -2.26185 | 0.00639 | down |
| ENSMUSG00000035864 | Syt1 | 1.45478 | 0.00639 | up |
| ENSMUSG00000072647 | Adam1a | -1.40395 | 0.00633 | down |
| ENSMUSG00000108866 | Gm35082 | -2.93874 | 0.00633 | down |
| ENSMUSG00000117322 | 6330415G19Rik | -1.79343 | 0.00632 | down |
| ENSMUSG00002076443 | Gm54922 | -4.98335 | 0.00631 | down |
| ENSMUSG00000109061 | Gm49320 | 3.947185 | 0.00630 | up |
| ENSMUSG00000039620 | Trmt9b | -1.00662 | 0.00626 | down |
| ENSMUSG00000089829 | Gm16565 | -6.75604 | 0.00624 | down |
| ENSMUSG00000057182 | Scn3a | -2.88899 | 0.00624 | down |
| ENSMUSG00000120686 | - | -1.2425 | 0.00622 | down |
| ENSMUSG00000070368 | Prok1 | 2.42936 | 0.00616 | up |
| ENSMUSG00000078862 | Gm14326 | 1.505091 | 0.00616 | up |
| ENSMUSG00000030263 | Irag2 | -1.00512 | 0.00616 | down |
| ENSMUSG00000104299 | Gm9924 | -4.57808 | 0.00614 | down |
| ENSMUSG00000120221 | - | -3.83581 | 0.00613 | down |
| ENSMUSG00000082361 | Btc | 1.919253 | 0.00610 | up |
| ENSMUSG00000113924 | Gm48493 | -1.29306 | 0.00609 | down |
| ENSMUSG00000102649 | Gm38021 | -2.57979 | 0.00607 | down |
| ENSMUSG00000103070 | Gm37903 | -1.33125 | 0.00605 | down |
| ENSMUSG00000039628 | Hs3st6 | 2.128177 | 0.00604 | up |
| ENSMUSG00000096351 | Samd11 | -3.94226 | 0.00603 | down |
| ENSMUSG00000028976 | Slc2a5 | 1.396919 | 0.00601 | up |
| ENSMUSG00000072972 | Adam4 | -2.19924 | 0.00600 | down |
| ENSMUSG00000032714 | Syde1 | -1.1344 | 0.00600 | down |
| ENSMUSG00000021700 | Rab3c | -3.26185 | 0.00598 | down |
| ENSMUSG00000096056 | Gm21986 | -3.4043 | 0.00596 | down |
| ENSMUSG00000038593 | Tctn1 | -1.05985 | 0.00595 | down |
| ENSMUSG00000043050 | Tnp2 | -4.39756 | 0.00594 | down |
| ENSMUSG00000020882 | Cacnb1 | -2.20535 | 0.00592 | down |
| ENSMUSG00000109841 | E330011O21Rik | 1.490021 | 0.00589 | up |
| ENSMUSG00000035285 | Nat14 | -1.84574 | 0.00588 | down |
| ENSMUSG00000116295 | Gm32885 | -3.20754 | 0.00587 | down |
| ENSMUSG00000023961 | Enpp4 | 1.016167 | 0.00585 | up |
| ENSMUSG00000054752 | Fsd1l | -1.73643 | 0.00584 | down |
| ENSMUSG00000091721 | Gimd1 | 2.156108 | 0.00580 | up |
| ENSMUSG00000004347 | Pde1c | -2.29897 | 0.00579 | down |
| ENSMUSG00000044461 | Shisa2 | -2.56676 | 0.00579 | down |
| ENSMUSG00000086127 | Gm11934 | -5.01909 | 0.00578 | down |
| ENSMUSG00000092035 | Peg10 | -1.5596 | 0.00577 | down |
| ENSMUSG00000029482 | Aacs | 1.047887 | 0.00576 | up |
| ENSMUSG00000016356 | Col20a1 | -1.53041 | 0.00573 | down |
| ENSMUSG00000021007 | Spata7 | -1.01716 | 0.00572 | down |
| ENSMUSG00000107168 | Gm42507 | -3.08055 | 0.00572 | down |
| ENSMUSG00000120809 | - | -3.32645 | 0.00571 | down |
| ENSMUSG00000095362 | Gm14325 | 1.251074 | 0.00569 | up |
| ENSMUSG00000113622 | Gm49749 | -1.02461 | 0.00568 | down |
| ENSMUSG00000044229 | Nxpe4 | -1.97076 | 0.00565 | down |
| ENSMUSG00000022102 | Dok2 | -1.05604 | 0.00564 | down |
| ENSMUSG00000072809 | 9330160F10Rik | -2.05033 | 0.00563 | down |
| ENSMUSG00000030206 | Gsg1 | -4.42406 | 0.00561 | down |
| ENSMUSG00000093769 | H3c14 | -1.62036 | 0.00560 | down |
| ENSMUSG00000036086 | Zranb3 | -1.00544 | 0.00559 | down |
| ENSMUSG00000115869 | Gm31814 | -1.50864 | 0.00558 | down |
| ENSMUSG00000093668 | Pou5f2 | -4.45554 | 0.00555 | down |
| ENSMUSG00000041798 | Gck | -1.03503 | 0.00550 | down |
| ENSMUSG00000121016 | - | -4.63985 | 0.00549 | down |
| ENSMUSG00000044390 | Tigd3 | -2.21982 | 0.00549 | down |
| ENSMUSG00000093916 | Gm379 | 1.411177 | 0.00549 | up |
| ENSMUSG00000105347 | Gm43503 | -2.50431 | 0.00548 | down |
| ENSMUSG00000101952 | Gm10550 | -5.09152 | 0.00547 | down |
| ENSMUSG00000108446 | Gm44997 | -1.96042 | 0.00546 | down |
| ENSMUSG00000120992 | - | 1.319817 | 0.00546 | up |
| ENSMUSG00000019577 | Pdk4 | -1.14119 | 0.00546 | down |
| ENSMUSG00000051616 | C230029F24Rik | -3.4319 | 0.00545 | down |
| ENSMUSG00000098708 | Gm27252 | -1.17 | 0.00545 | down |
| ENSMUSG00000039103 | Nexn | -1.57752 | 0.00543 | down |
| ENSMUSG00000116987 | Gm49625 | -3.23397 | 0.00542 | down |
| ENSMUSG00000105519 | E430021H15Rik | -2.54188 | 0.00541 | down |
| ENSMUSG00000045284 | Dcaf12l1 | 1.320658 | 0.00539 | up |
| ENSMUSG00000097445 | Gm26631 | -1.52968 | 0.00537 | down |
| ENSMUSG00000112090 | Gm48231 | -3.49028 | 0.00535 | down |
| ENSMUSG00000056055 | Sag | -2.79094 | 0.00534 | down |
| ENSMUSG00000111737 | Gm47248 | -2.07083 | 0.00533 | down |
| ENSMUSG00000028359 | Orm3 | 1.933504 | 0.00531 | up |
| ENSMUSG00000103098 | Gm37559 | -4.07711 | 0.00530 | down |
| ENSMUSG00000107577 | Gm44103 | -1.15729 | 0.00529 | down |
| ENSMUSG00000091680 | Klhdc7b | -3.0257 | 0.00529 | down |
| ENSMUSG00000054191 | Klf1 | 1.477078 | 0.00528 | up |
| ENSMUSG00000105832 | Gm43841 | -2.20431 | 0.00526 | down |
| ENSMUSG00000036882 | Arhgap33 | -2.21273 | 0.00525 | down |
| ENSMUSG00000021414 | Fam217a | -3.65568 | 0.00524 | down |
| ENSMUSG00000027890 | Gstm4 | 1.434069 | 0.00523 | up |
| ENSMUSG00000121213 | - | 1.774014 | 0.00523 | up |
| ENSMUSG00000111681 | Gm47640 | 2.943907 | 0.00522 | up |
| ENSMUSG00000050640 | Tmem150c | -3.31752 | 0.00522 | down |
| ENSMUSG00000085733 | Arhgap27os1 | -3.98529 | 0.00520 | down |
| ENSMUSG00000020681 | Ace | -1.83911 | 0.00517 | down |
| ENSMUSG00000029206 | Nsun7 | -2.56425 | 0.00517 | down |
| ENSMUSG00000025427 | Rnf165 | -2.66358 | 0.00516 | down |
| ENSMUSG00000108199 | Gm44249 | -2.18851 | 0.00515 | down |
| ENSMUSG00000028240 | Cyp7a1 | 1.041572 | 0.00513 | up |
| ENSMUSG00000110520 | Gm45776 | -2.63421 | 0.00505 | down |
| ENSMUSG00000118664 | Tusc3 | 1.14876 | 0.00504 | up |
| ENSMUSG00000089989 | Gm45713 | 4.004369 | 0.00503 | up |
| ENSMUSG00000055022 | Cntn1 | -2.13433 | 0.00496 | down |
| ENSMUSG00000120050 | - | -1.96621 | 0.00495 | down |
| ENSMUSG00000029086 | Prom1 | -1.43539 | 0.00493 | down |
| ENSMUSG00000079669 | Gm17396 | -1.91748 | 0.00491 | down |
| ENSMUSG00000116883 | Gm49700 | -2.85974 | 0.00491 | down |
| ENSMUSG00000038608 | Dock10 | -1.05258 | 0.00491 | down |
| ENSMUSG00000103409 | Lsmem2 | -2.53626 | 0.00490 | down |
| ENSMUSG00000051314 | Ffar2 | -2.02055 | 0.00488 | down |
| ENSMUSG00000108372 | Gm45124 | -3.85531 | 0.00486 | down |
| ENSMUSG00002076650 | Snord3b1 | -5.53594 | 0.00482 | down |
| ENSMUSG00000048047 | Zbtb33 | 1.093289 | 0.00479 | up |
| ENSMUSG00000008028 | 1700008O03Rik | -3.83096 | 0.00476 | down |
| ENSMUSG00000109751 | Gm45479 | -2.73646 | 0.00475 | down |
| ENSMUSG00000024008 | Cpne5 | -4.05916 | 0.00472 | down |
| ENSMUSG00000120290 | - | -1.92554 | 0.00470 | down |
| ENSMUSG00000035186 | Ubd | 2.569602 | 0.00470 | up |
| ENSMUSG00000035835 | Plppr3 | 1.900322 | 0.00469 | up |
| ENSMUSG00000074825 | Itpripl1 | 1.088705 | 0.00469 | up |
| ENSMUSG00000090785 | Gm17116 | -2.61602 | 0.00469 | down |
| ENSMUSG00000117440 | Gm50087 | -3.80863 | 0.00468 | down |
| ENSMUSG00000105601 | Gm42725 | -4.37096 | 0.00468 | down |
| ENSMUSG00000007646 | Rad51c | -1.60962 | 0.00464 | down |
| ENSMUSG00000085001 | Rapgef4os2 | -1.19956 | 0.00463 | down |
| ENSMUSG00000104168 | Gm38250 | 2.94383 | 0.00463 | up |
| ENSMUSG00000103696 | Gm37531 | -4.77193 | 0.00461 | down |
| ENSMUSG00000031647 | Mfap3l | -2.72501 | 0.00457 | down |
| ENSMUSG00000062456 | Rpl9-ps6 | -2.82265 | 0.00457 | down |
| ENSMUSG00000028031 | Dkk2 | -2.94061 | 0.00456 | down |
| ENSMUSG00000052783 | Grk4 | -1.13673 | 0.00454 | down |
| ENSMUSG00000113750 | Gm5628 | -5.13205 | 0.00453 | down |
| ENSMUSG00000061132 | Blnk | 1.713782 | 0.00450 | up |
| ENSMUSG00000063698 | Sfxn4 | -2.41996 | 0.00450 | down |
| ENSMUSG00000071037 | Camkmt | 1.73168 | 0.00449 | up |
| ENSMUSG00000051998 | Lax1 | -1.60932 | 0.00448 | down |
| ENSMUSG00000050357 | Carmil2 | -2.15213 | 0.00448 | down |
| ENSMUSG00000074415 | Mir100hg | -1.51973 | 0.00447 | down |
| ENSMUSG00000118040 | Gm36602 | -4.9886 | 0.00445 | down |
| ENSMUSG00000036687 | Tmem184a | 1.064024 | 0.00445 | up |
| ENSMUSG00000097729 | 2310015A10Rik | -1.38925 | 0.00443 | down |
| ENSMUSG00000104214 | Gm36638 | -2.76216 | 0.00441 | down |
| ENSMUSG00000027175 | Tcp11l1 | 1.292222 | 0.00440 | up |
| ENSMUSG00000109669 | Gm45472 | -3.25302 | 0.00440 | down |
| ENSMUSG00000044309 | Apol7c | -2.57621 | 0.00439 | down |
| ENSMUSG00000110225 | Gm45528 | -1.77494 | 0.00439 | down |
| ENSMUSG00000110750 | Gm48702 | 1.925355 | 0.00438 | up |
| ENSMUSG00000025887 | Casp12 | -1.20248 | 0.00437 | down |
| ENSMUSG00000105711 | Gm42598 | -4.05323 | 0.00436 | down |
| ENSMUSG00000050069 | Grem2 | 1.087132 | 0.00436 | up |
| ENSMUSG00000021904 | Sema3g | -1.4738 | 0.00436 | down |
| ENSMUSG00000104563 | Gm43041 | 3.513298 | 0.00434 | up |
| ENSMUSG00000116145 | 5730521K06Rik | -3.34503 | 0.00433 | down |
| ENSMUSG00000094145 | Vmn2r20 | -1.6809 | 0.00432 | down |
| ENSMUSG00000007379 | Dennd2c | -1.8228 | 0.00432 | down |
| ENSMUSG00000104174 | Gm37701 | -1.01002 | 0.00430 | down |
| ENSMUSG00000040061 | Plcb2 | -1.15566 | 0.00430 | down |
| ENSMUSG00000103332 | Pcdhga2 | 4.562113 | 0.00429 | up |
| ENSMUSG00000087484 | 2900089D17Rik | -2.4586 | 0.00428 | down |
| ENSMUSG00000105245 | Gm31305 | -3.26618 | 0.00427 | down |
| ENSMUSG00000069808 | Tlcd3a | 1.07921 | 0.00426 | up |
| ENSMUSG00000034898 | Filip1 | -1.9054 | 0.00426 | down |
| ENSMUSG00000032006 | Pdgfd | -1.45744 | 0.00425 | down |
| ENSMUSG00000092563 | Gm3617 | -5.98052 | 0.00424 | down |
| ENSMUSG00000001281 | Itgb7 | -1.22164 | 0.00424 | down |
| ENSMUSG00000113621 | 2900060N12Rik | -4.68283 | 0.00423 | down |
| ENSMUSG00000120919 | - | -2.62356 | 0.00422 | down |
| ENSMUSG00000021950 | Anxa8 | 2.500016 | 0.00421 | up |
| ENSMUSG00000074604 | Mgst2 | -3.46584 | 0.00420 | down |
| ENSMUSG00000098022 | Zfp82 | -2.36937 | 0.00420 | down |
| ENSMUSG00000037772 | Mrpl23 | 1.998201 | 0.00415 | up |
| ENSMUSG00000115447 | Gm48964 | -1.42515 | 0.00415 | down |
| ENSMUSG00000073805 | Insyn2a | -3.80638 | 0.00414 | down |
| ENSMUSG00000028738 | Tas1r2 | -5.02251 | 0.00413 | down |
| ENSMUSG00000094588 | Olfr898 | -3.40802 | 0.00406 | down |
| ENSMUSG00000043029 | Trpv3 | -3.77162 | 0.00404 | down |
| ENSMUSG00000093606 | B130034C11Rik | -1.4264 | 0.00404 | down |
| ENSMUSG00000028391 | Wdr31 | -1.68458 | 0.00401 | down |
| ENSMUSG00000042834 | Nrep | 1.142408 | 0.00399 | up |
| ENSMUSG00000063739 | Gm4963 | -1.36464 | 0.00399 | down |
| ENSMUSG00000091228 | Gm20390 | 2.247302 | 0.00398 | up |
| ENSMUSG00000027160 | Ccdc34 | -1.23261 | 0.00398 | down |
| ENSMUSG00000104121 | Gm37485 | -2.42632 | 0.00395 | down |
| ENSMUSG00000110772 | Gm47856 | -3.73938 | 0.00394 | down |
| ENSMUSG00000035208 | Slfn8 | -1.47195 | 0.00394 | down |
| ENSMUSG00000112990 | Gm47372 | -1.9709 | 0.00393 | down |
| ENSMUSG00000014301 | Pam16 | -2.1792 | 0.00389 | down |
| ENSMUSG00000109498 | Gm45222 | -2.6113 | 0.00386 | down |
| ENSMUSG00000034413 | Neurl1b | -1.60799 | 0.00384 | down |
| ENSMUSG00000035818 | Plekhs1 | -2.04511 | 0.00383 | down |
| ENSMUSG00000086265 | Marcksl1-ps4 | 4.155108 | 0.00382 | up |
| ENSMUSG00000097365 | C030034L19Rik | -4.82211 | 0.00382 | down |
| ENSMUSG00000025648 | Pfkfb4 | -1.40928 | 0.00382 | down |
| ENSMUSG00000036492 | Rnf39 | 1.197306 | 0.00381 | up |
| ENSMUSG00000111132 | Gm48142 | -2.98441 | 0.00381 | down |
| ENSMUSG00000058084 | Olfr825 | -2.48174 | 0.00380 | down |
| ENSMUSG00000031349 | Nsdhl | 1.070706 | 0.00378 | up |
| ENSMUSG00000041624 | Gucy1a2 | -3.24648 | 0.00378 | down |
| ENSMUSG00000086735 | Gm13977 | -3.59922 | 0.00375 | down |
| ENSMUSG00000103473 | Gm37696 | -4.42475 | 0.00372 | down |
| ENSMUSG00000103509 | Gm38372 | -3.07002 | 0.00372 | down |
| ENSMUSG00000035042 | Ccl5 | -1.1247 | 0.00371 | down |
| ENSMUSG00000102659 | Gm37077 | -4.50077 | 0.00370 | down |
| ENSMUSG00000038754 | Elovl3 | -1.60997 | 0.00369 | down |
| ENSMUSG00000105802 | Gm43012 | 3.175244 | 0.00368 | up |
| ENSMUSG00000091514 | Gm17484 | -1.48595 | 0.00368 | down |
| ENSMUSG00000039842 | Mcph1 | 1.255436 | 0.00367 | up |
| ENSMUSG00000120095 | - | -1.55132 | 0.00367 | down |
| ENSMUSG00000044551 | 9930012K11Rik | -2.33886 | 0.00367 | down |
| ENSMUSG00000031561 | Tenm3 | 1.199133 | 0.00365 | up |
| ENSMUSG00000114430 | Gm40264 | -1.27396 | 0.00362 | down |
| ENSMUSG00000093622 | Gm20703 | 2.801639 | 0.00361 | up |
| ENSMUSG00000038352 | Arl5c | -2.09309 | 0.00359 | down |
| ENSMUSG00000070883 | Ccdc173 | -3.35738 | 0.00356 | down |
| ENSMUSG00000034127 | Tspan8 | 2.3975 | 0.00356 | up |
| ENSMUSG00000074469 | Gm15348 | 3.382842 | 0.00353 | up |
| ENSMUSG00000108827 | Olfr1310 | -3.01494 | 0.00352 | down |
| ENSMUSG00000028678 | Kif2c | 3.407192 | 0.00351 | up |
| ENSMUSG00000021811 | Dnajc9 | 1.131485 | 0.00343 | up |
| ENSMUSG00000102964 | 9430034N14Rik | -4.06259 | 0.00342 | down |
| ENSMUSG00000113467 | Gm46350 | -3.00166 | 0.00341 | down |
| ENSMUSG00000079597 | Cstdc4 | -2.49994 | 0.00341 | down |
| ENSMUSG00000031765 | Mt1 | 2.050301 | 0.00340 | up |
| ENSMUSG00000114989 | Gm49303 | -3.71076 | 0.00339 | down |
| ENSMUSG00000068014 | Gm14648 | 5.613174 | 0.00339 | up |
| ENSMUSG00000115381 | Gm49266 | -3.28044 | 0.00337 | down |
| ENSMUSG00000051550 | Zfp579 | -1.07651 | 0.00337 | down |
| ENSMUSG00000049580 | Tsku | -1.67703 | 0.00337 | down |
| ENSMUSG00000112549 | Gm35818 | -4.59383 | 0.00337 | down |
| ENSMUSG00000056643 | Chst13 | 1.351705 | 0.00335 | up |
| ENSMUSG00000084416 | Rpl10a-ps1 | 2.009909 | 0.00335 | up |
| ENSMUSG00000108231 | Gm4045 | 2.178792 | 0.00333 | up |
| ENSMUSG00000040836 | Gpr161 | -3.86277 | 0.00331 | down |
| ENSMUSG00000036036 | Zfp57 | -2.0698 | 0.00329 | down |
| ENSMUSG00000060678 | H4c3 | -1.74805 | 0.00329 | down |
| ENSMUSG00000036172 | Cd200r3 | -2.23717 | 0.00327 | down |
| ENSMUSG00000019066 | Rab3d | 1.122906 | 0.00326 | up |
| ENSMUSG00000091945 | Vmn2r114 | -1.03969 | 0.00325 | down |
| ENSMUSG00000022658 | Tagln3 | -2.31111 | 0.00325 | down |
| ENSMUSG00000029636 | Wasf3 | -3.26571 | 0.00324 | down |
| ENSMUSG00000018169 | Mfng | -1.57004 | 0.00324 | down |
| ENSMUSG00000055704 | Gm9978 | -1.80308 | 0.00324 | down |
| ENSMUSG00000013155 | Enkd1 | -1.84333 | 0.00322 | down |
| ENSMUSG00000097466 | D430036J16Rik | -1.71359 | 0.00321 | down |
| ENSMUSG00000027932 | Slc27a3 | -1.56288 | 0.00319 | down |
| ENSMUSG00000107750 | Gm44013 | -4.03731 | 0.00318 | down |
| ENSMUSG00000104606 | Gm43409 | -1.91364 | 0.00317 | down |
| ENSMUSG00000049265 | Kcnk3 | -1.9125 | 0.00316 | down |
| ENSMUSG00000087611 | 4930458D05Rik | 3.841361 | 0.00313 | up |
| ENSMUSG00000097262 | 4933416M07Rik | -5.21462 | 0.00313 | down |
| ENSMUSG00000117959 | D330050I16Rik | -1.46987 | 0.00311 | down |
| ENSMUSG00000082585 | Gm15387 | -1.0058 | 0.00311 | down |
| ENSMUSG00000022335 | Zfat | -1.07113 | 0.00309 | down |
| ENSMUSG00000037151 | Lrrc20 | 1.052914 | 0.00307 | up |
| ENSMUSG00000025243 | Slc6a20b | -2.85336 | 0.00306 | down |
| ENSMUSG00000029673 | Auts2 | -1.73151 | 0.00306 | down |
| ENSMUSG00000007783 | Cpt1c | -2.13169 | 0.00306 | down |
| ENSMUSG00000020641 | Rsad2 | -1.34153 | 0.00305 | down |
| ENSMUSG00000097318 | 1700007L15Rik | 1.605846 | 0.00305 | up |
| ENSMUSG00000029122 | Evc | -1.00261 | 0.00304 | down |
| ENSMUSG00000001521 | Tulp3 | -1.30711 | 0.00303 | down |
| ENSMUSG00000003228 | Grk5 | -1.08186 | 0.00302 | down |
| ENSMUSG00000053113 | Socs3 | 1.187755 | 0.00301 | up |
| ENSMUSG00000084904 | Gm14827 | -4.57859 | 0.00301 | down |
| ENSMUSG00000108473 | Gm44739 | -3.35299 | 0.00299 | down |
| ENSMUSG00000086154 | Gm16196 | -3.81632 | 0.00299 | down |
| ENSMUSG00000039246 | Lyplal1 | 1.286009 | 0.00297 | up |
| ENSMUSG00000120900 | - | -1.08988 | 0.00297 | down |
| ENSMUSG00000063810 | Alms1 | -1.39751 | 0.00297 | down |
| ENSMUSG00000036526 | Card11 | -1.56387 | 0.00297 | down |
| ENSMUSG00000038156 | Spon1 | -1.30279 | 0.00297 | down |
| ENSMUSG00000115810 | Gm49066 | -2.31151 | 0.00296 | down |
| ENSMUSG00000056973 | Ces1d | 1.010846 | 0.00296 | up |
| ENSMUSG00000072244 | Trim6 | -1.86613 | 0.00295 | down |
| ENSMUSG00000049928 | Glp2r | -2.72164 | 0.00295 | down |
| ENSMUSG00000005983 | 1700037C18Rik | -1.9463 | 0.00295 | down |
| ENSMUSG00000031896 | Ctrl | -2.91339 | 0.00292 | down |
| ENSMUSG00000105107 | Gm43412 | -2.68507 | 0.00291 | down |
| ENSMUSG00000116525 | Gm30371 | -2.74901 | 0.00289 | down |
| ENSMUSG00000100954 | Gm10138 | -1.50575 | 0.00289 | down |
| ENSMUSG00000087371 | Gm15541 | -3.1069 | 0.00287 | down |
| ENSMUSG00000063522 | Ly6m | -2.25074 | 0.00287 | down |
| ENSMUSG00000075588 | Hoxb2 | -2.11464 | 0.00284 | down |
| ENSMUSG00000049420 | Tmem200a | -3.00554 | 0.00283 | down |
| ENSMUSG00000069920 | B3gnt9 | -2.70406 | 0.00282 | down |
| ENSMUSG00000001948 | Spa17 | -1.43078 | 0.00282 | down |
| ENSMUSG00000105789 | Gm42997 | -3.36466 | 0.00281 | down |
| ENSMUSG00000051727 | Kctd14 | -2.71475 | 0.00280 | down |
| ENSMUSG00000107000 | Gm43481 | 2.845478 | 0.00280 | up |
| ENSMUSG00000040658 | Dnph1 | 1.214035 | 0.00280 | up |
| ENSMUSG00000097448 | Platr22 | -2.10133 | 0.00279 | down |
| ENSMUSG00000019863 | Qrsl1 | 1.140697 | 0.00273 | up |
| ENSMUSG00000032172 | Olfm2 | 2.66758 | 0.00273 | up |
| ENSMUSG00000030878 | Cdr2 | -2.08652 | 0.00271 | down |
| ENSMUSG00000035239 | Neu3 | -1.6952 | 0.00270 | down |
| ENSMUSG00000027204 | Fbn1 | -1.11907 | 0.00270 | down |
| ENSMUSG00000066233 | Tmem42 | 1.283531 | 0.00270 | up |
| ENSMUSG00000049676 | Catsperg1 | -3.03054 | 0.00270 | down |
| ENSMUSG00000119584 | Rn18s-rs5 | -1.06594 | 0.00270 | down |
| ENSMUSG00000031262 | Cenpi | -2.76258 | 0.00269 | down |
| ENSMUSG00000104795 | Gm42783 | -3.40332 | 0.00269 | down |
| ENSMUSG00000112667 | Gm48270 | -1.49177 | 0.00267 | down |
| ENSMUSG00000050555 | Hyls1 | -1.17428 | 0.00266 | down |
| ENSMUSG00000021306 | Gpr137b | -1.22267 | 0.00266 | down |
| ENSMUSG00000120006 | - | -1.28032 | 0.00265 | down |
| ENSMUSG00000038252 | Ncapd2 | -1.21948 | 0.00263 | down |
| ENSMUSG00000015316 | Slamf1 | -2.90938 | 0.00261 | down |
| ENSMUSG00000031748 | Gnao1 | -2.15408 | 0.00259 | down |
| ENSMUSG00000069919 | Hba-a1 | -1.05453 | 0.00259 | down |
| ENSMUSG00000115846 | Gm41144 | 3.662274 | 0.00259 | up |
| ENSMUSG00000110161 | Gm45494 | -3.05991 | 0.00259 | down |
| ENSMUSG00000110841 | Gpx4-ps2 | -1.41743 | 0.00259 | down |
| ENSMUSG00000106568 | Gm42814 | -2.7535 | 0.00258 | down |
| ENSMUSG00000030699 | Tbx6 | -2.11216 | 0.00256 | down |
| ENSMUSG00000030030 | 1700003E16Rik | -1.45139 | 0.00255 | down |
| ENSMUSG00000044453 | Ffar1 | -2.14991 | 0.00255 | down |
| ENSMUSG00000120419 | - | -3.63047 | 0.00253 | down |
| ENSMUSG00000020389 | Cdkl3 | -2.36949 | 0.00252 | down |
| ENSMUSG00000034303 | Ccdc15 | -2.14681 | 0.00252 | down |
| ENSMUSG00000019808 | Adat2 | 1.988006 | 0.00250 | up |
| ENSMUSG00000106928 | Gm43860 | -1.85141 | 0.00250 | down |
| ENSMUSG00000105134 | Gm42923 | 2.857403 | 0.00249 | up |
| ENSMUSG00000105224 | Gm3364 | -3.26051 | 0.00249 | down |
| ENSMUSG00000078173 | Lenep | -3.31756 | 0.00249 | down |
| ENSMUSG00000105762 | Gm43605 | -4.55891 | 0.00249 | down |
| ENSMUSG00000049922 | Slc35c1 | 1.079783 | 0.00248 | up |
| ENSMUSG00000086296 | D030055H07Rik | 2.45818 | 0.00248 | up |
| ENSMUSG00000073491 | Ifi213 | -1.37821 | 0.00246 | down |
| ENSMUSG00000084934 | Gm16035 | -1.00916 | 0.00245 | down |
| ENSMUSG00000022661 | Cd200 | -1.25264 | 0.00245 | down |
| ENSMUSG00000094156 | Sult2a7 | 2.32113 | 0.00243 | up |
| ENSMUSG00000020836 | Coro6 | -4.15515 | 0.00243 | down |
| ENSMUSG00000000276 | Dgke | -1.06703 | 0.00242 | down |
| ENSMUSG00000054666 | Olfr63 | -2.58987 | 0.00242 | down |
| ENSMUSG00000047420 | Fam180a | -1.71039 | 0.00241 | down |
| ENSMUSG00000029544 | Cabp1 | -2.73161 | 0.00240 | down |
| ENSMUSG00000042616 | Oscp1 | -2.69893 | 0.00240 | down |
| ENSMUSG00000070604 | Vsig10l | -1.54396 | 0.00238 | down |
| ENSMUSG00000028212 | Ccne2 | -1.67632 | 0.00238 | down |
| ENSMUSG00000035228 | Ccdc106 | -4.56169 | 0.00238 | down |
| ENSMUSG00000085741 | 5430405H02Rik | -1.16674 | 0.00237 | down |
| ENSMUSG00000021684 | Pde8b | -1.66177 | 0.00236 | down |
| ENSMUSG00000022070 | Bora | -1.31234 | 0.00236 | down |
| ENSMUSG00000105370 | Gm42718 | -2.07746 | 0.00235 | down |
| ENSMUSG00000085151 | 1110018N20Rik | -1.53448 | 0.00235 | down |
| ENSMUSG00000021388 | Aspn | -2.52933 | 0.00234 | down |
| ENSMUSG00000105008 | Gm43652 | -3.60781 | 0.00234 | down |
| ENSMUSG00000084824 | Gm16344 | -1.87568 | 0.00233 | down |
| ENSMUSG00000030034 | Ino80b | 1.384811 | 0.00233 | up |
| ENSMUSG00000038534 | Osbpl7 | -1.52591 | 0.00231 | down |
| ENSMUSG00000121504 | Ppp4r1l-ps | -1.0267 | 0.00231 | down |
| ENSMUSG00000102151 | Gm37472 | -3.12321 | 0.00230 | down |
| ENSMUSG00000096373 | Vmn2r31 | -3.62323 | 0.00230 | down |
| ENSMUSG00000026017 | Carf | -1.15427 | 0.00230 | down |
| ENSMUSG00000107143 | Gm6598 | -1.43554 | 0.00228 | down |
| ENSMUSG00000110620 | Gm45733 | -2.43908 | 0.00226 | down |
| ENSMUSG00000026981 | Il1rn | 1.904384 | 0.00226 | up |
| ENSMUSG00000030380 | Mzf1 | -2.71144 | 0.00225 | down |
| ENSMUSG00000052658 | 5830454E08Rik | -1.53386 | 0.00225 | down |
| ENSMUSG00000108211 | Gm44130 | -3.30407 | 0.00225 | down |
| ENSMUSG00000028909 | Ptpru | -2.19706 | 0.00224 | down |
| ENSMUSG00000020429 | Igfbp1 | 1.713602 | 0.00222 | up |
| ENSMUSG00000068101 | Cenpm | 1.207944 | 0.00219 | up |
| ENSMUSG00000018809 | Smyd4 | -1.29495 | 0.00218 | down |
| ENSMUSG00000100937 | Nscme3l | -4.94872 | 0.00217 | down |
| ENSMUSG00000006313 | Upk1a | -5.45528 | 0.00217 | down |
| ENSMUSG00000113095 | Gm7969 | -7.98358 | 0.00216 | down |
| ENSMUSG00000080877 | Rpl22-ps1 | 2.462293 | 0.00216 | up |
| ENSMUSG00000045751 | Mms22l | 2.636725 | 0.00215 | up |
| ENSMUSG00000045211 | Nudt18 | 1.067397 | 0.00213 | up |
| ENSMUSG00000037907 | Ankrd13b | -1.23422 | 0.00212 | down |
| ENSMUSG00000072949 | Acot1 | -1.51839 | 0.00211 | down |
| ENSMUSG00000052833 | Sae1 | 1.105229 | 0.00210 | up |
| ENSMUSG00000106715 | Tmem265 | -2.5748 | 0.00209 | down |
| ENSMUSG00000115186 | Gm49417 | -1.34006 | 0.00209 | down |
| ENSMUSG00000120153 | - | -2.23296 | 0.00208 | down |
| ENSMUSG00000054568 | Usp17la | -3.35816 | 0.00208 | down |
| ENSMUSG00000040339 | Fam102b | -1.13211 | 0.00207 | down |
| ENSMUSG00000028568 | Btf3l4 | 1.218431 | 0.00206 | up |
| ENSMUSG00000120918 | - | -1.72126 | 0.00206 | down |
| ENSMUSG00000113660 | Gm5626 | -1.09134 | 0.00206 | down |
| ENSMUSG00000105553 | Gm42514 | -4.58313 | 0.00205 | down |
| ENSMUSG00000118458 | Gm10599 | -2.22948 | 0.00204 | down |
| ENSMUSG00000056919 | Cep162 | -1.12321 | 0.00204 | down |
| ENSMUSG00000066362 | Rps13-ps1 | 2.608654 | 0.00203 | up |
| ENSMUSG00000029376 | Mthfd2l | -1.22086 | 0.00201 | down |
| ENSMUSG00000084383 | Gm13370 | 1.928647 | 0.00201 | up |
| ENSMUSG00000071550 | Cfap44 | -4.67021 | 0.00201 | down |
| ENSMUSG00000035506 | Slc12a8 | -1.6995 | 0.00200 | down |
| ENSMUSG00000117013 | Gm30531 | 3.718936 | 0.00199 | up |
| ENSMUSG00000105909 | Gm43189 | -1.97654 | 0.00199 | down |
| ENSMUSG00000024678 | Ms4a4d | -1.1801 | 0.00198 | down |
| ENSMUSG00000031574 | Star | -1.58486 | 0.00197 | down |
| ENSMUSG00000032374 | Plod2 | -1.30527 | 0.00197 | down |
| ENSMUSG00000043822 | Adamtsl5 | -1.42658 | 0.00196 | down |
| ENSMUSG00000076498 | Trbc2 | -1.77972 | 0.00196 | down |
| ENSMUSG00000078300 | Gm2606 | 1.825927 | 0.00196 | up |
| ENSMUSG00000023050 | Map3k12 | -1.37951 | 0.00196 | down |
| ENSMUSG00000103957 | Gm10766 | -3.03487 | 0.00196 | down |
| ENSMUSG00000025650 | Col7a1 | -2.80267 | 0.00195 | down |
| ENSMUSG00000114836 | Gm18517 | -1.4326 | 0.00195 | down |
| ENSMUSG00000014444 | Piezo1 | -1.08542 | 0.00195 | down |
| ENSMUSG00000121370 | - | -2.35579 | 0.00194 | down |
| ENSMUSG00000038280 | Ostm1 | 1.493299 | 0.00194 | up |
| ENSMUSG00000054641 | Mmrn1 | -2.05897 | 0.00190 | down |
| ENSMUSG00000027134 | Lpcat4 | -1.50096 | 0.00190 | down |
| ENSMUSG00000019986 | Ahi1 | -2.59096 | 0.00190 | down |
| ENSMUSG00000030515 | Tarsl2 | 1.074173 | 0.00190 | up |
| ENSMUSG00000096006 | Gm21596 | -1.38328 | 0.00189 | down |
| ENSMUSG00000016494 | Cd34 | -1.15849 | 0.00188 | down |
| ENSMUSG00000087632 | Gm6058 | -3.49605 | 0.00188 | down |
| ENSMUSG00000040584 | Abcb1a | 1.368825 | 0.00186 | up |
| ENSMUSG00000028017 | Egf | -1.69034 | 0.00186 | down |
| ENSMUSG00000032740 | Ccdc88a | -1.16904 | 0.00185 | down |
| ENSMUSG00000027438 | Napb | -1.00603 | 0.00185 | down |
| ENSMUSG00000097057 | Gm17638 | -2.75591 | 0.00184 | down |
| ENSMUSG00000093661 | Eif4e3 | -1.09904 | 0.00183 | down |
| ENSMUSG00000028369 | Svep1 | -1.87716 | 0.00182 | down |
| ENSMUSG00000040809 | Chil3 | 3.178868 | 0.00182 | up |
| ENSMUSG00000104626 | Gm42675 | -5.05109 | 0.00182 | down |
| ENSMUSG00000109115 | Gm44669 | -1.98382 | 0.00181 | down |
| ENSMUSG00000031659 | Adcy7 | -1.2197 | 0.00181 | down |
| ENSMUSG00000033032 | Afap1l1 | -1.00116 | 0.00180 | down |
| ENSMUSG00000103739 | Gm37653 | -5.31513 | 0.00179 | down |
| ENSMUSG00000036672 | Cenpt | -1.164 | 0.00179 | down |
| ENSMUSG00000028555 | Ttc39a | 2.675804 | 0.00178 | up |
| ENSMUSG00000026117 | Zap70 | 1.498753 | 0.00178 | up |
| ENSMUSG00000062110 | Scfd2 | 1.295549 | 0.00177 | up |
| ENSMUSG00000018486 | Wnt9b | -1.28501 | 0.00175 | down |
| ENSMUSG00000103761 | Gm37859 | -4.19675 | 0.00175 | down |
| ENSMUSG00000087354 | 4930404I05Rik | -1.90183 | 0.00174 | down |
| ENSMUSG00000037731 | Themis2 | -1.0157 | 0.00173 | down |
| ENSMUSG00000109548 | Gm45066 | -3.27957 | 0.00171 | down |
| ENSMUSG00000020589 | Cyria | -1.0132 | 0.00171 | down |
| ENSMUSG00000030519 | Apba2 | -2.90705 | 0.00170 | down |
| ENSMUSG00000110693 | Gm45899 | -1.51988 | 0.00170 | down |
| ENSMUSG00000084136 | Vmn1r207 | -1.73938 | 0.00169 | down |
| ENSMUSG00000085241 | Snhg3 | -1.0596 | 0.00168 | down |
| ENSMUSG00000075511 | 1700001L05Rik | 1.525462 | 0.00168 | up |
| ENSMUSG00000049184 | Purg | -1.34495 | 0.00167 | down |
| ENSMUSG00000104435 | Gm37422 | -1.63086 | 0.00167 | down |
| ENSMUSG00000030559 | Rab38 | 2.856616 | 0.00167 | up |
| ENSMUSG00000026904 | Slc4a10 | -2.82852 | 0.00167 | down |
| ENSMUSG00000041481 | Serpina3g | -1.03031 | 0.00166 | down |
| ENSMUSG00000079173 | Zan | -2.18409 | 0.00165 | down |
| ENSMUSG00000022432 | Smc1b | -2.66996 | 0.00164 | down |
| ENSMUSG00000002006 | Pdzd4 | -2.18505 | 0.00163 | down |
| ENSMUSG00000030322 | Mbd4 | -1.46063 | 0.00163 | down |
| ENSMUSG00000026567 | Adcy10 | -5.42195 | 0.00163 | down |
| ENSMUSG00000041696 | Rasl12 | -2.74895 | 0.00162 | down |
| ENSMUSG00000090353 | Gm17555 | -1.72085 | 0.00161 | down |
| ENSMUSG00000051238 | Swsap1 | 1.218101 | 0.00160 | up |
| ENSMUSG00000121109 | - | -2.02274 | 0.00159 | down |
| ENSMUSG00000109597 | Gm45492 | -5.13172 | 0.00158 | down |
| ENSMUSG00000112229 | Gm48086 | -2.17887 | 0.00158 | down |
| ENSMUSG00000035495 | Tstd2 | 1.115302 | 0.00157 | up |
| ENSMUSG00000116885 | 4930420G21Rik | -1.80542 | 0.00157 | down |
| ENSMUSG00000029372 | Ppbp | -2.12684 | 0.00157 | down |
| ENSMUSG00000023966 | Rsph9 | -1.77129 | 0.00156 | down |
| ENSMUSG00000021597 | Slf1 | -1.08463 | 0.00156 | down |
| ENSMUSG00000094778 | Olfr143 | -2.46702 | 0.00156 | down |
| ENSMUSG00000097836 | Gm26903 | -3.30677 | 0.00154 | down |
| ENSMUSG00000011257 | Pabpc4 | -1.51724 | 0.00154 | down |
| ENSMUSG00000121215 | - | -2.18505 | 0.00154 | down |
| ENSMUSG00000025001 | Hells | -2.07373 | 0.00153 | down |
| ENSMUSG00000030124 | Lag3 | -1.98757 | 0.00152 | down |
| ENSMUSG00000079467 | Gm14966 | -1.58202 | 0.00152 | down |
| ENSMUSG00000099338 | 2810030D12Rik | -1.20447 | 0.00151 | down |
| ENSMUSG00000041707 | Tmem273 | -1.90209 | 0.00151 | down |
| ENSMUSG00000042249 | Grk3 | -1.1328 | 0.00149 | down |
| ENSMUSG00000052909 | Gm9894 | -3.88429 | 0.00149 | down |
| ENSMUSG00000120342 | - | -2.37717 | 0.00149 | down |
| ENSMUSG00000084283 | Gm14914 | -4.40579 | 0.00149 | down |
| ENSMUSG00000112833 | Gm36595 | -4.54223 | 0.00148 | down |
| ENSMUSG00000073415 | Gm10501 | -2.40209 | 0.00148 | down |
| ENSMUSG00000109695 | Gm31166 | -1.09187 | 0.00147 | down |
| ENSMUSG00000111605 | 4632418H02Rik | -2.85117 | 0.00146 | down |
| ENSMUSG00000050382 | Kif7 | -2.34779 | 0.00146 | down |
| ENSMUSG00000035258 | Abi3bp | -1.18011 | 0.00146 | down |
| ENSMUSG00000113669 | Gm36723 | -2.49437 | 0.00146 | down |
| ENSMUSG00000025008 | Tctn3 | -1.70271 | 0.00146 | down |
| ENSMUSG00000121275 | - | -2.41959 | 0.00146 | down |
| ENSMUSG00000037752 | Xkr8 | 1.204403 | 0.00145 | up |
| ENSMUSG00000115422 | 4930452G13Rik | -2.22159 | 0.00145 | down |
| ENSMUSG00000039452 | Snx22 | -2.18166 | 0.00145 | down |
| ENSMUSG00000020474 | Polm | -1.08363 | 0.00144 | down |
| ENSMUSG00000092283 | Gm20412 | 3.965346 | 0.00144 | up |
| ENSMUSG00000072847 | A530017D24Rik | -1.21754 | 0.00144 | down |
| ENSMUSG00000078588 | Ccdc24 | -2.51199 | 0.00143 | down |
| ENSMUSG00000058174 | Gm5148 | 3.411736 | 0.00143 | up |
| ENSMUSG00000114886 | Gm48432 | 1.849377 | 0.00143 | up |
| ENSMUSG00000034731 | Dgkh | -1.60444 | 0.00142 | down |
| ENSMUSG00000087128 | Gm12655 | -1.83017 | 0.00142 | down |
| ENSMUSG00000037363 | Letm2 | -1.22901 | 0.00140 | down |
| ENSMUSG00000027384 | Ndufaf5 | 1.084188 | 0.00140 | up |
| ENSMUSG00000041716 | Gm20604 | 1.642295 | 0.00140 | up |
| ENSMUSG00000111692 | Gm49373 | -2.33049 | 0.00139 | down |
| ENSMUSG00000074676 | Foxs1 | -3.05621 | 0.00139 | down |
| ENSMUSG00000109097 | Gm29683 | -2.15995 | 0.00139 | down |
| ENSMUSG00000115316 | Gm49096 | -3.23007 | 0.00139 | down |
| ENSMUSG00000055493 | Epm2a | 1.153056 | 0.00138 | up |
| ENSMUSG00000091019 | Gm7502 | -3.0131 | 0.00138 | down |
| ENSMUSG00000120258 | - | -3.2766 | 0.00138 | down |
| ENSMUSG00000036430 | Tbcc | 1.233869 | 0.00136 | up |
| ENSMUSG00000078087 | Rps12l1 | -2.95158 | 0.00135 | down |
| ENSMUSG00000047342 | Zfp286 | -3.85289 | 0.00135 | down |
| ENSMUSG00000038236 | Hoxa7 | -3.97652 | 0.00135 | down |
| ENSMUSG00000109674 | Gm45470 | 1.566176 | 0.00134 | up |
| ENSMUSG00000047216 | Cdh19 | -4.75017 | 0.00134 | down |
| ENSMUSG00000032018 | Sc5d | 1.139954 | 0.00134 | up |
| ENSMUSG00000114729 | C330022B21Rik | -2.6762 | 0.00133 | down |
| ENSMUSG00000070661 | Rnf186 | 1.453794 | 0.00132 | up |
| ENSMUSG00000048621 | Gm6377 | -1.77395 | 0.00132 | down |
| ENSMUSG00000072612 | Gm10382 | 3.058412 | 0.00131 | up |
| ENSMUSG00000102956 | Gm37017 | -4.17791 | 0.00130 | down |
| ENSMUSG00000032320 | Rcn2 | 1.021278 | 0.00128 | up |
| ENSMUSG00000036136 | Fam110c | 1.866502 | 0.00128 | up |
| ENSMUSG00000035279 | Ssc5d | -2.84559 | 0.00128 | down |
| ENSMUSG00000113847 | Gm48799 | -1.70051 | 0.00128 | down |
| ENSMUSG00000015451 | C4a | -1.26723 | 0.00128 | down |
| ENSMUSG00000114452 | A530001N23Rik | -2.14094 | 0.00127 | down |
| ENSMUSG00000040528 | Milr1 | -1.96948 | 0.00127 | down |
| ENSMUSG00000090877 | Hspa1b | 1.194206 | 0.00127 | up |
| ENSMUSG00000073987 | Ggh | 1.264773 | 0.00126 | up |
| ENSMUSG00000118491 | Gm44505 | -6.42642 | 0.00126 | down |
| ENSMUSG00000080888 | Gm14387 | 1.657441 | 0.00125 | up |
| ENSMUSG00000082644 | Mup-ps19 | 1.889578 | 0.00125 | up |
| ENSMUSG00000022793 | B4galt4 | -1.19535 | 0.00124 | down |
| ENSMUSG00000105429 | Gm43692 | -2.49206 | 0.00124 | down |
| ENSMUSG00000109656 | Gm45548 | -3.25812 | 0.00124 | down |
| ENSMUSG00000028427 | Aqp7 | -2.7416 | 0.00124 | down |
| ENSMUSG00000119210 | n-R5s117 | -4.64202 | 0.00123 | down |
| ENSMUSG00000106634 | Gm43042 | -1.36613 | 0.00123 | down |
| ENSMUSG00000021611 | Tert | -1.01076 | 0.00123 | down |
| ENSMUSG00000080316 | Spaca6 | -1.62833 | 0.00122 | down |
| ENSMUSG00000053310 | Nrgn | -1.6385 | 0.00122 | down |
| ENSMUSG00000117105 | Dreh | 1.936558 | 0.00121 | up |
| ENSMUSG00000088185 | Scarna2 | -1.51141 | 0.00121 | down |
| ENSMUSG00000045827 | Serpinb9 | 1.722648 | 0.00121 | up |
| ENSMUSG00000002825 | Qtrt1 | -1.22847 | 0.00121 | down |
| ENSMUSG00000117079 | Gm41611 | -1.98956 | 0.00120 | down |
| ENSMUSG00000107994 | D830050J10Rik | 1.556138 | 0.00119 | up |
| ENSMUSG00000030409 | Dmpk | -1.01175 | 0.00119 | down |
| ENSMUSG00000082765 | Gm14411 | 3.509872 | 0.00118 | up |
| ENSMUSG00000091572 | Vmn2r3 | -4.12298 | 0.00116 | down |
| ENSMUSG00000035948 | Acss3 | 1.30341 | 0.00116 | up |
| ENSMUSG00000057329 | Bcl2 | -1.30119 | 0.00116 | down |
| ENSMUSG00000115700 | Gm7517 | 2.741921 | 0.00116 | up |
| ENSMUSG00000113683 | Gm47123 | 3.058964 | 0.00116 | up |
| ENSMUSG00000078486 | Perm1 | -2.64741 | 0.00115 | down |
| ENSMUSG00000054855 | Rnd1 | 1.116479 | 0.00115 | up |
| ENSMUSG00000022519 | Srl | 3.576957 | 0.00114 | up |
| ENSMUSG00000030166 | Rad52 | -1.05908 | 0.00114 | down |
| ENSMUSG00000043251 | Exoc3l | -1.37614 | 0.00114 | down |
| ENSMUSG00000105931 | Gm43014 | -1.92917 | 0.00113 | down |
| ENSMUSG00000025092 | Hspa12a | -1.30008 | 0.00113 | down |
| ENSMUSG00000032122 | Slc37a2 | -1.39566 | 0.00113 | down |
| ENSMUSG00000022758 | P2rx6 | -4.28337 | 0.00112 | down |
| ENSMUSG00000049670 | Morn4 | -2.206 | 0.00112 | down |
| ENSMUSG00000029641 | Rasl11a | -1.9215 | 0.00112 | down |
| ENSMUSG00000075289 | Carns1 | -1.54556 | 0.00111 | down |
| ENSMUSG00000040987 | Mill2 | -1.42686 | 0.00110 | down |
| ENSMUSG00000090623 | Cfhr3 | 2.781845 | 0.00110 | up |
| ENSMUSG00000091957 | Rps2-ps10 | -1.26102 | 0.00109 | down |
| ENSMUSG00000108218 | Olfr1372 | -2.37989 | 0.00109 | down |
| ENSMUSG00000022504 | Ciita | -1.53743 | 0.00109 | down |
| ENSMUSG00000081724 | Olfr129 | -3.13195 | 0.00109 | down |
| ENSMUSG00000022687 | Boc | -2.32979 | 0.00108 | down |
| ENSMUSG00000033526 | Ppip5k1 | -1.15771 | 0.00108 | down |
| ENSMUSG00000076490 | Trbc1 | -2.52478 | 0.00108 | down |
| ENSMUSG00000103103 | 4833445I07Rik | -1.96992 | 0.00107 | down |
| ENSMUSG00000020844 | Nxn | -1.22578 | 0.00107 | down |
| ENSMUSG00000120142 | - | -1.13041 | 0.00106 | down |
| ENSMUSG00000025212 | Sfxn3 | -1.072 | 0.00105 | down |
| ENSMUSG00000023057 | Fabp2 | 1.000186 | 0.00105 | up |
| ENSMUSG00000093673 | Gm20644 | -4.06391 | 0.00105 | down |
| ENSMUSG00000007987 | Ift22 | 1.031178 | 0.00105 | up |
| ENSMUSG00000097327 | E030030I06Rik | -1.52063 | 0.00104 | down |
| ENSMUSG00000079225 | Gm9531 | 4.025761 | 0.00104 | up |
| ENSMUSG00000108268 | Gm44187 | -3.99207 | 0.00103 | down |
| ENSMUSG00000097891 | Gm3650 | -1.05765 | 0.00103 | down |
| ENSMUSG00000108494 | Gm45203 | -3.23257 | 0.00103 | down |
| ENSMUSG00000104662 | Gm43267 | -2.33632 | 0.00103 | down |
| ENSMUSG00000109916 | 2410039M03Rik | -1.96814 | 0.00103 | down |
| ENSMUSG00000039096 | Rsad1 | -1.07559 | 0.00102 | down |
| ENSMUSG00000121210 | - | -4.68055 | 0.00102 | down |
| ENSMUSG00000037979 | Ccdc92 | -2.49341 | 0.00102 | down |
| ENSMUSG00000022148 | Fyb | -1.9187 | 0.00101 | down |
| ENSMUSG00000022401 | Xpnpep3 | 1.06016 | 0.00101 | up |
| ENSMUSG00000033752 | Mnd1 | 1.223048 | 0.00100 | up |
| ENSMUSG00000114369 | Gm41077 | 3.562557 | 0.00100 | up |
| ENSMUSG00000108533 | Gm44792 | -1.22101 | 0.00099 | down |
| ENSMUSG00000043331 | Olfr975 | -2.32752 | 0.00099 | down |
| ENSMUSG00000021508 | Cxcl14 | 1.647479 | 0.00098 | up |
| ENSMUSG00000023266 | Frs3 | -1.40505 | 0.00098 | down |
| ENSMUSG00000035266 | Helq | -1.02562 | 0.00098 | down |
| ENSMUSG00000094380 | Olfr904 | -2.82779 | 0.00098 | down |
| ENSMUSG00000090081 | Gm16587 | -2.33623 | 0.00097 | down |
| ENSMUSG00000085696 | Hoxaas3 | -3.36208 | 0.00097 | down |
| ENSMUSG00000081988 | Gm15746 | -1.20642 | 0.00096 | down |
| ENSMUSG00000074461 | Gm10699 | 4.054091 | 0.00096 | up |
| ENSMUSG00000038982 | Bloc1s5 | 1.113971 | 0.00096 | up |
| ENSMUSG00000079019 | Insl3 | -2.46689 | 0.00095 | down |
| ENSMUSG00000049439 | Cyp20a1 | 1.195887 | 0.00095 | up |
| ENSMUSG00000075078 | Olfr1250 | -1.12123 | 0.00095 | down |
| ENSMUSG00000053137 | Mapk11 | -1.12007 | 0.00094 | down |
| ENSMUSG00000046605 | B3gntl1 | -1.08042 | 0.00094 | down |
| ENSMUSG00000045838 | Ccdc9b | -1.03239 | 0.00093 | down |
| ENSMUSG00000120409 | - | -2.4307 | 0.00093 | down |
| ENSMUSG00000033361 | Prrg3 | -2.08022 | 0.00093 | down |
| ENSMUSG00000047155 | Cyp4x1 | -2.26749 | 0.00092 | down |
| ENSMUSG00000071456 | 1110002L01Rik | 1.668876 | 0.00092 | up |
| ENSMUSG00000074922 | Fam122a | 1.604106 | 0.00091 | up |
| ENSMUSG00000052407 | Ccdc171 | -1.13394 | 0.00091 | down |
| ENSMUSG00000079243 | Xirp1 | 2.211749 | 0.00091 | up |
| ENSMUSG00000100768 | Gm29055 | -1.84944 | 0.00090 | down |
| ENSMUSG00000040811 | Eml2 | 1.045321 | 0.00090 | up |
| ENSMUSG00000053091 | Lins1 | -1.01286 | 0.00090 | down |
| ENSMUSG00000114096 | Gm48641 | -3.44297 | 0.00090 | down |
| ENSMUSG00000072849 | Serpina1e | 1.264576 | 0.00089 | up |
| ENSMUSG00000119206 | Gm22317 | -4.42198 | 0.00088 | down |
| ENSMUSG00000118501 | Gm53048 | -3.88736 | 0.00088 | down |
| ENSMUSG00000043924 | Ncmap | -1.97072 | 0.00088 | down |
| ENSMUSG00000111857 | 1190001M18Rik | -5.14466 | 0.00088 | down |
| ENSMUSG00000049411 | Tmem241 | 1.886747 | 0.00087 | up |
| ENSMUSG00000047594 | Olfr1122 | -2.52171 | 0.00087 | down |
| ENSMUSG00000118295 | Gm8437 | 3.587663 | 0.00087 | up |
| ENSMUSG00000043794 | D830025C05Rik | -3.95049 | 0.00087 | down |
| ENSMUSG00000102009 | 4933400F21Rik | -3.57653 | 0.00086 | down |
| ENSMUSG00000050103 | Agmo | 1.151854 | 0.00085 | up |
| ENSMUSG00000085527 | Gm15535 | -2.29295 | 0.00085 | down |
| ENSMUSG00000018845 | Unc45b | -1.33177 | 0.00084 | down |
| ENSMUSG00000026556 | Vangl2 | -1.56484 | 0.00084 | down |
| ENSMUSG00000061411 | Nol4l | -1.37494 | 0.00084 | down |
| ENSMUSG00000039533 | Mmd2 | 1.264487 | 0.00084 | up |
| ENSMUSG00000055114 | Anxa13 | -1.15867 | 0.00083 | down |
| ENSMUSG00000072571 | Tmem253 | -3.27194 | 0.00083 | down |
| ENSMUSG00000102712 | Gm37758 | -1.918 | 0.00083 | down |
| ENSMUSG00000070532 | Ccdc190 | -4.98475 | 0.00082 | down |
| ENSMUSG00000048782 | Insc | 1.229293 | 0.00082 | up |
| ENSMUSG00000054417 | Cyp3a44 | 3.484642 | 0.00082 | up |
| ENSMUSG00000111656 | Gm47232 | -3.89013 | 0.00082 | down |
| ENSMUSG00000024742 | Fen1 | 2.354912 | 0.00082 | up |
| ENSMUSG00000031433 | Rbm41 | 3.421607 | 0.00081 | up |
| ENSMUSG00000120121 | - | -3.66454 | 0.00081 | down |
| ENSMUSG00000049515 | Espnl | -4.02634 | 0.00081 | down |
| ENSMUSG00000030613 | Ccdc90b | 1.080283 | 0.00081 | up |
| ENSMUSG00000039033 | Tasp1 | 1.36753 | 0.00081 | up |
| ENSMUSG00000111923 | Gm34777 | -1.93727 | 0.00081 | down |
| ENSMUSG00000120841 | - | 2.021251 | 0.00081 | up |
| ENSMUSG00000109015 | Gm31024 | -2.06425 | 0.00080 | down |
| ENSMUSG00000046782 | Ttc6 | -1.97188 | 0.00080 | down |
| ENSMUSG00000002083 | Bbc3 | 1.323955 | 0.00080 | up |
| ENSMUSG00000021749 | Fam3d | -4.24801 | 0.00080 | down |
| ENSMUSG00000114123 | Gm48416 | -1.30446 | 0.00080 | down |
| ENSMUSG00000000416 | Cttnbp2 | -2.31042 | 0.00080 | down |
| ENSMUSG00000109887 | Gm28756 | -1.21454 | 0.00080 | down |
| ENSMUSG00002075453 | Snord3b3 | -4.8524 | 0.00079 | down |
| ENSMUSG00000085287 | 4833418N02Rik | -1.20399 | 0.00078 | down |
| ENSMUSG00000047473 | Zfp30 | -1.03058 | 0.00078 | down |
| ENSMUSG00000055072 | Gm9964 | -5.37021 | 0.00078 | down |
| ENSMUSG00000078921 | Tgtp2 | -1.68425 | 0.00078 | down |
| ENSMUSG00000111133 | Gm5831 | 4.466603 | 0.00078 | up |
| ENSMUSG00000098050 | Gm5345 | 2.836787 | 0.00077 | up |
| ENSMUSG00000026840 | Lamc3 | -1.0616 | 0.00077 | down |
| ENSMUSG00000106437 | Gm35667 | -4.35031 | 0.00077 | down |
| ENSMUSG00000116004 | Gm49539 | -4.98056 | 0.00077 | down |
| ENSMUSG00000073771 | Btbd19 | -1.76627 | 0.00076 | down |
| ENSMUSG00000067038 | Rps12-ps3 | -2.82919 | 0.00076 | down |
| ENSMUSG00000055323 | Gm9967 | -3.291 | 0.00076 | down |
| ENSMUSG00000024965 | Fermt3 | -1.06738 | 0.00076 | down |
| ENSMUSG00000038930 | Rccd1 | -1.2214 | 0.00076 | down |
| ENSMUSG00000087179 | 5730471H19Rik | -1.64524 | 0.00076 | down |
| ENSMUSG00000031015 | Swap70 | -1.02899 | 0.00076 | down |
| ENSMUSG00000102145 | Gm38056 | -3.91112 | 0.00075 | down |
| ENSMUSG00000109422 | Gm45174 | -3.86712 | 0.00075 | down |
| ENSMUSG00000110151 | Gm38416 | -1.29912 | 0.00075 | down |
| ENSMUSG00000024503 | Spink1 | 4.250605 | 0.00074 | up |
| ENSMUSG00000073700 | Klhl21 | 1.107293 | 0.00074 | up |
| ENSMUSG00000092595 | Gm20427 | -2.68067 | 0.00074 | down |
| ENSMUSG00000086189 | Gm15462 | -2.00251 | 0.00074 | down |
| ENSMUSG00000115007 | 5830448L01Rik | -1.10046 | 0.00074 | down |
| ENSMUSG00000037020 | Wdr62 | -1.6132 | 0.00074 | down |
| ENSMUSG00000043008 | Klhl6 | -1.32857 | 0.00073 | down |
| ENSMUSG00000064899 | Snord118 | -4.33395 | 0.00073 | down |
| ENSMUSG00000025083 | Afap1l2 | -1.62195 | 0.00073 | down |
| ENSMUSG00000066122 | Olfr45 | -2.15162 | 0.00072 | down |
| ENSMUSG00000106040 | Cyp3a63-ps | -1.90233 | 0.00072 | down |
| ENSMUSG00000010205 | Raver1 | 1.061759 | 0.00072 | up |
| ENSMUSG00000042156 | Dzip1 | -1.97523 | 0.00072 | down |
| ENSMUSG00000022621 | Rabl2 | -1.15961 | 0.00071 | down |
| ENSMUSG00000097910 | Hdnr | -2.23751 | 0.00070 | down |
| ENSMUSG00000106671 | Gm42900 | -2.86491 | 0.00070 | down |
| ENSMUSG00000015342 | Xk | 1.579479 | 0.00070 | up |
| ENSMUSG00000030882 | Dnhd1 | -1.20951 | 0.00069 | down |
| ENSMUSG00000121177 | - | -2.11746 | 0.00069 | down |
| ENSMUSG00000041189 | Chrnb1 | -1.28167 | 0.00069 | down |
| ENSMUSG00000032773 | Chrm1 | 3.96468 | 0.00068 | up |
| ENSMUSG00000107304 | Gm43775 | -2.23504 | 0.00068 | down |
| ENSMUSG00000111485 | Gm48443 | -1.00776 | 0.00068 | down |
| ENSMUSG00000045822 | Zswim3 | -1.39182 | 0.00067 | down |
| ENSMUSG00000074912 | Gm14207 | -2.94128 | 0.00067 | down |
| ENSMUSG00000086054 | Hnf1aos1 | -1.10776 | 0.00067 | down |
| ENSMUSG00000066804 | Vmn1r83 | -4.54474 | 0.00067 | down |
| ENSMUSG00000108750 | Gm44750 | -4.13995 | 0.00066 | down |
| ENSMUSG00000026657 | Frmd4a | -1.0602 | 0.00066 | down |
| ENSMUSG00000093565 | Rab26os | -2.40663 | 0.00066 | down |
| ENSMUSG00000048096 | Lmod1 | -2.37062 | 0.00065 | down |
| ENSMUSG00000097974 | Gm10605 | -1.78849 | 0.00065 | down |
| ENSMUSG00000081948 | Olfr1191 | -3.46991 | 0.00064 | down |
| ENSMUSG00000079108 | Srp54c | 1.11143 | 0.00064 | up |
| ENSMUSG00000113170 | Gm36839 | -2.98431 | 0.00064 | down |
| ENSMUSG00000115520 | Gm41335 | -4.69241 | 0.00064 | down |
| ENSMUSG00000107785 | Gm45083 | 1.677741 | 0.00064 | up |
| ENSMUSG00000106889 | Gm7463 | 1.740658 | 0.00063 | up |
| ENSMUSG00000021259 | Cyp46a1 | -1.7273 | 0.00063 | down |
| ENSMUSG00000023935 | Spats1 | -5.37 | 0.00063 | down |
| ENSMUSG00000116262 | Gm49544 | 2.253199 | 0.00063 | up |
| ENSMUSG00000039646 | Vasn | 1.230881 | 0.00062 | up |
| ENSMUSG00000068114 | Ccdc134 | -1.35452 | 0.00062 | down |
| ENSMUSG00000022146 | Osmr | -1.18871 | 0.00061 | down |
| ENSMUSG00000092274 | Neat1 | -1.90869 | 0.00061 | down |
| ENSMUSG00000108249 | Gm43960 | -2.94537 | 0.00061 | down |
| ENSMUSG00000118053 | Gm50244 | 1.702421 | 0.00061 | up |
| ENSMUSG00000061882 | Ccdc62 | -1.01089 | 0.00061 | down |
| ENSMUSG00000020638 | Cmpk2 | -1.40577 | 0.00060 | down |
| ENSMUSG00000024013 | Fgd2 | -1.2607 | 0.00060 | down |
| ENSMUSG00000099519 | Gm29253 | -1.50393 | 0.00060 | down |
| ENSMUSG00000029352 | Crybb3 | 2.123169 | 0.00060 | up |
| ENSMUSG00000109693 | Gm45483 | -2.94074 | 0.00060 | down |
| ENSMUSG00000042099 | Kank3 | -1.00292 | 0.00059 | down |
| ENSMUSG00000097360 | 9430065F17Rik | -2.14958 | 0.00059 | down |
| ENSMUSG00000053825 | Ppfia2 | -2.69484 | 0.00059 | down |
| ENSMUSG00000043015 | Nemp2 | -1.7786 | 0.00059 | down |
| ENSMUSG00000028051 | Hcn3 | -1.58428 | 0.00059 | down |
| ENSMUSG00000108953 | Gm45129 | -3.13967 | 0.00058 | down |
| ENSMUSG00000022020 | Naa16 | -1.21238 | 0.00058 | down |
| ENSMUSG00000025262 | Fam120c | 3.115985 | 0.00058 | up |
| ENSMUSG00000019813 | Cep57l1 | -1.00848 | 0.00058 | down |
| ENSMUSG00000055033 | Olfr420 | -2.9888 | 0.00058 | down |
| ENSMUSG00000086443 | 4933421A08Rik | -1.77151 | 0.00058 | down |
| ENSMUSG00000120789 | - | 3.566867 | 0.00058 | up |
| ENSMUSG00000105345 | BC030343 | -3.23845 | 0.00057 | down |
| ENSMUSG00000037685 | Atp8a1 | -1.03093 | 0.00057 | down |
| ENSMUSG00000096950 | Gm9530 | -3.48196 | 0.00057 | down |
| ENSMUSG00000108483 | Gm45184 | -4.26308 | 0.00057 | down |
| ENSMUSG00000118181 | Gm53015 | -1.03527 | 0.00057 | down |
| ENSMUSG00000040428 | Plekha4 | -2.35609 | 0.00056 | down |
| ENSMUSG00000103436 | Gm36995 | -2.16007 | 0.00056 | down |
| ENSMUSG00000030724 | Cd19 | -1.74818 | 0.00056 | down |
| ENSMUSG00000085417 | Gm13919 | -2.15489 | 0.00056 | down |
| ENSMUSG00000014030 | Pax5 | -1.80044 | 0.00056 | down |
| ENSMUSG00000029314 | Gpat3 | 1.163888 | 0.00056 | up |
| ENSMUSG00000110631 | Gm42047 | -1.85518 | 0.00056 | down |
| ENSMUSG00000030782 | Tgfb1i1 | -1.32445 | 0.00055 | down |
| ENSMUSG00000033697 | Arhgap39 | -1.05449 | 0.00055 | down |
| ENSMUSG00000031684 | Slc10a7 | 1.060396 | 0.00055 | up |
| ENSMUSG00000117490 | Gm35031 | -2.05959 | 0.00054 | down |
| ENSMUSG00000000339 | Rtca | 1.286278 | 0.00054 | up |
| ENSMUSG00000109016 | Gm44647 | -5.32756 | 0.00053 | down |
| ENSMUSG00000078234 | Klhdc7a | 1.163674 | 0.00053 | up |
| ENSMUSG00000086050 | Gm16045 | -3.14439 | 0.00053 | down |
| ENSMUSG00000102571 | Gm37967 | -2.71076 | 0.00053 | down |
| ENSMUSG00000103539 | Gm37834 | -3.66115 | 0.00053 | down |
| ENSMUSG00000021196 | Pfkp | -1.26736 | 0.00052 | down |
| ENSMUSG00000034854 | Mfsd12 | 1.095944 | 0.00052 | up |
| ENSMUSG00000032717 | Mdfi | -1.91098 | 0.00052 | down |
| ENSMUSG00000120986 | - | -3.95923 | 0.00052 | down |
| ENSMUSG00000027082 | Tfpi | -1.04237 | 0.00052 | down |
| ENSMUSG00000105549 | Gm43540 | -1.4108 | 0.00052 | down |
| ENSMUSG00000121476 | - | -2.54134 | 0.00052 | down |
| ENSMUSG00000109108 | Gm36371 | -3.92507 | 0.00052 | down |
| ENSMUSG00000049287 | Iba57 | 1.16059 | 0.00052 | up |
| ENSMUSG00000080921 | Rpl38-ps2 | -6.96608 | 0.00052 | down |
| ENSMUSG00000073915 | Olfr676 | -5.22141 | 0.00052 | down |
| ENSMUSG00000025978 | Rftn2 | -1.83657 | 0.00051 | down |
| ENSMUSG00000107362 | Gm40309 | -4.61024 | 0.00051 | down |
| ENSMUSG00000091577 | Gm6211 | -3.11588 | 0.00051 | down |
| ENSMUSG00000063972 | Nr6a1 | -1.01488 | 0.00051 | down |
| ENSMUSG00000031373 | Car5b | -1.18145 | 0.00051 | down |
| ENSMUSG00000118061 | Rbfaos | -3.42199 | 0.00051 | down |
| ENSMUSG00000108368 | Gm45053 | -1.97469 | 0.00051 | down |
| ENSMUSG00000072919 | Noxred1 | -3.05759 | 0.00051 | down |
| ENSMUSG00000042787 | Exog | -1.3453 | 0.00050 | down |
| ENSMUSG00000107331 | Gm42732 | -1.48786 | 0.00050 | down |
| ENSMUSG00000121154 | - | -4.41339 | 0.00049 | down |
| ENSMUSG00000078317 | F8a | 1.399278 | 0.00049 | up |
| ENSMUSG00000069294 | Vmn1r197 | -2.1044 | 0.00049 | down |
| ENSMUSG00000038453 | Srcin1 | -3.31779 | 0.00049 | down |
| ENSMUSG00000115184 | Gm49197 | -3.21385 | 0.00048 | down |
| ENSMUSG00000020937 | Plcd3 | -1.5766 | 0.00048 | down |
| ENSMUSG00000097224 | Gm26716 | -2.06495 | 0.00048 | down |
| ENSMUSG00000036466 | Megf11 | -2.64772 | 0.00048 | down |
| ENSMUSG00000071637 | Cebpd | 1.434055 | 0.00048 | up |
| ENSMUSG00000057207 | Olfr1028 | -2.65198 | 0.00047 | down |
| ENSMUSG00000118298 | Vmn1r60 | -3.67913 | 0.00047 | down |
| ENSMUSG00000085037 | 4933421O10Rik | -1.10448 | 0.00047 | down |
| ENSMUSG00000101578 | Vmn1r206 | -2.62328 | 0.00047 | down |
| ENSMUSG00000025006 | Sorbs1 | -1.25852 | 0.00047 | down |
| ENSMUSG00000035711 | Dok3 | -1.531 | 0.00047 | down |
| ENSMUSG00000087396 | 4933407K13Rik | -1.81368 | 0.00047 | down |
| ENSMUSG00000113800 | 2210039B01Rik | -1.35219 | 0.00047 | down |
| ENSMUSG00000102241 | Gm37716 | -3.93192 | 0.00047 | down |
| ENSMUSG00000118936 | Gm22614 | -4.02155 | 0.00046 | down |
| ENSMUSG00000066072 | Cyp4a10 | -1.56699 | 0.00046 | down |
| ENSMUSG00000042500 | Ago4 | -1.03678 | 0.00046 | down |
| ENSMUSG00000067279 | Ppp1r3c | -1.09101 | 0.00046 | down |
| ENSMUSG00000028443 | Nudt2 | 1.403609 | 0.00046 | up |
| ENSMUSG00000054469 | Lclat1 | 1.013056 | 0.00046 | up |
| ENSMUSG00000027514 | Zbp1 | 1.575715 | 0.00045 | up |
| ENSMUSG00000028860 | Sytl1 | -1.0523 | 0.00045 | down |
| ENSMUSG00000047090 | Tmem198b | -1.44318 | 0.00045 | down |
| ENSMUSG00000043059 | Zfp513 | -1.04885 | 0.00045 | down |
| ENSMUSG00000014039 | Prdm15 | -1.10443 | 0.00044 | down |
| ENSMUSG00000037443 | Cep85 | -1.66288 | 0.00044 | down |
| ENSMUSG00000111619 | Gm48348 | 2.661446 | 0.00044 | up |
| ENSMUSG00000024304 | Cdh2 | -1.21416 | 0.00044 | down |
| ENSMUSG00000028933 | Xrcc2 | 2.322489 | 0.00044 | up |
| ENSMUSG00000052676 | Zmat1 | -1.48054 | 0.00043 | down |
| ENSMUSG00000031021 | Tmem9b | 1.001367 | 0.00043 | up |
| ENSMUSG00000016624 | Phf21b | -2.37415 | 0.00043 | down |
| ENSMUSG00000102460 | Gm38197 | -4.2701 | 0.00043 | down |
| ENSMUSG00000050623 | Catsperz | -5.55587 | 0.00042 | down |
| ENSMUSG00000055116 | Arntl | -1.85201 | 0.00042 | down |
| ENSMUSG00000031770 | Herpud1 | 1.276692 | 0.00041 | up |
| ENSMUSG00000097222 | Gata6os | -1.53029 | 0.00041 | down |
| ENSMUSG00000083773 | Gm13394 | -1.3295 | 0.00041 | down |
| ENSMUSG00000091227 | Gm3755 | -4.12355 | 0.00041 | down |
| ENSMUSG00000014503 | Pkd2l2 | -2.13808 | 0.00041 | down |
| ENSMUSG00000058883 | Zfp708 | -1.41701 | 0.00040 | down |
| ENSMUSG00000120623 | - | -2.72799 | 0.00040 | down |
| ENSMUSG00000034438 | Gbp8 | -1.60552 | 0.00040 | down |
| ENSMUSG00000112880 | Gm20337 | -1.91128 | 0.00040 | down |
| ENSMUSG00000120872 | - | -2.91524 | 0.00040 | down |
| ENSMUSG00000038425 | Poli | -1.01514 | 0.00039 | down |
| ENSMUSG00000090110 | Cmc4 | -1.37571 | 0.00039 | down |
| ENSMUSG00000104378 | Gm37510 | -2.83887 | 0.00039 | down |
| ENSMUSG00000021932 | Rnaseh2b | 1.12002 | 0.00039 | up |
| ENSMUSG00000037126 | Psd | -2.12975 | 0.00039 | down |
| ENSMUSG00000095098 | Ccdc85b | 1.1097 | 0.00039 | up |
| ENSMUSG00000009633 | G0s2 | -1.40851 | 0.00039 | down |
| ENSMUSG00000110488 | Gm45724 | -1.69687 | 0.00038 | down |
| ENSMUSG00000027809 | Etfdh | 1.009438 | 0.00038 | up |
| ENSMUSG00000060183 | Cxcl11 | -1.46005 | 0.00038 | down |
| ENSMUSG00000121127 | - | -1.07493 | 0.00038 | down |
| ENSMUSG00000026820 | Ptges2 | 1.022513 | 0.00038 | up |
| ENSMUSG00000040841 | Six5 | -1.27308 | 0.00038 | down |
| ENSMUSG00000028177 | 1810013D15Rik | -2.06731 | 0.00038 | down |
| ENSMUSG00000108521 | Gm44639 | -2.5702 | 0.00037 | down |
| ENSMUSG00000047379 | B4gat1 | 1.651362 | 0.00037 | up |
| ENSMUSG00000102204 | Gm36958 | -1.39674 | 0.00037 | down |
| ENSMUSG00000121162 | - | -3.34925 | 0.00037 | down |
| ENSMUSG00000024653 | Scgb1a1 | -4.80582 | 0.00037 | down |
| ENSMUSG00000094076 | Gm4767 | -4.14231 | 0.00036 | down |
| ENSMUSG00000026442 | Nfasc | 2.867875 | 0.00036 | up |
| ENSMUSG00000027599 | Armc1 | 1.055404 | 0.00036 | up |
| ENSMUSG00000047909 | Ankrd16 | -1.0905 | 0.00036 | down |
| ENSMUSG00000066268 | Olfr586 | -5.57783 | 0.00036 | down |
| ENSMUSG00000106826 | Gm42583 | -2.7857 | 0.00036 | down |
| ENSMUSG00000052013 | Btla | -2.03891 | 0.00035 | down |
| ENSMUSG00000042328 | Hps4 | -1.08021 | 0.00035 | down |
| ENSMUSG00000070572 | Trmt112-ps2 | 2.635963 | 0.00035 | up |
| ENSMUSG00000029778 | Adcyap1r1 | -1.8823 | 0.00035 | down |
| ENSMUSG00000051041 | Olfml1 | 1.559808 | 0.00035 | up |
| ENSMUSG00000042389 | Tsen2 | 1.842158 | 0.00035 | up |
| ENSMUSG00000111274 | Gm47409 | -3.29943 | 0.00035 | down |
| ENSMUSG00000110504 | Gm45797 | -5.64795 | 0.00034 | down |
| ENSMUSG00000035458 | Tnni3 | -3.82839 | 0.00034 | down |
| ENSMUSG00000027961 | Lrrc39 | -2.02566 | 0.00034 | down |
| ENSMUSG00000114573 | Gm47732 | -3.69257 | 0.00034 | down |
| ENSMUSG00000118642 | AY036118 | -1.31331 | 0.00034 | down |
| ENSMUSG00000056718 | Gm13199 | -3.3815 | 0.00033 | down |
| ENSMUSG00000091239 | Vmn2r76 | -3.09803 | 0.00033 | down |
| ENSMUSG00000064326 | Siva1 | 1.75478 | 0.00033 | up |
| ENSMUSG00000020952 | Scfd1 | 1.17595 | 0.00033 | up |
| ENSMUSG00000041482 | Piezo2 | -1.49684 | 0.00033 | down |
| ENSMUSG00000040441 | Slc26a10 | -1.3306 | 0.00033 | down |
| ENSMUSG00000038304 | Cd160 | -2.5006 | 0.00033 | down |
| ENSMUSG00000036815 | Dpp10 | -1.81734 | 0.00033 | down |
| ENSMUSG00000039738 | Slx4 | -1.07184 | 0.00032 | down |
| ENSMUSG00000026173 | Plcd4 | -3.79324 | 0.00032 | down |
| ENSMUSG00000028845 | Tekt2 | -2.87878 | 0.00032 | down |
| ENSMUSG00000023021 | Cers5 | -1.38829 | 0.00032 | down |
| ENSMUSG00000085977 | Gm5970 | 2.494698 | 0.00032 | up |
| ENSMUSG00000109357 | Gm45236 | -4.37514 | 0.00032 | down |
| ENSMUSG00000118159 | Gm50402 | -3.36687 | 0.00031 | down |
| ENSMUSG00000087050 | Dhrs13os | -3.86496 | 0.00031 | down |
| ENSMUSG00000085412 | Halr1 | -4.00569 | 0.00031 | down |
| ENSMUSG00000097055 | Gm4419 | -2.58336 | 0.00031 | down |
| ENSMUSG00000022651 | Retnlg | 3.155782 | 0.00031 | up |
| ENSMUSG00000078440 | Dohh | 1.073346 | 0.00031 | up |
| ENSMUSG00000105471 | A430073D23Rik | -3.06693 | 0.00031 | down |
| ENSMUSG00000121142 | - | 1.123074 | 0.00031 | up |
| ENSMUSG00000079177 | Fam228a | -3.14871 | 0.00030 | down |
| ENSMUSG00000100039 | Gm28959 | -1.04963 | 0.00030 | down |
| ENSMUSG00000087042 | Gm11611 | -5.54487 | 0.00030 | down |
| ENSMUSG00000034427 | Myo15b | -2.49319 | 0.00030 | down |
| ENSMUSG00000001034 | Mapk7 | -1.30394 | 0.00030 | down |
| ENSMUSG00000042638 | Gucy2c | 3.563444 | 0.00030 | up |
| ENSMUSG00000025134 | Alyref | 1.365999 | 0.00030 | up |
| ENSMUSG00000121501 | - | -1.33864 | 0.00030 | down |
| ENSMUSG00000031642 | Sh3rf1 | -1.02444 | 0.00030 | down |
| ENSMUSG00000017002 | Slpi | 2.364473 | 0.00030 | up |
| ENSMUSG00000090015 | Gm15446 | -1.32369 | 0.00030 | down |
| ENSMUSG00000020973 | Dnaaf2 | 1.443106 | 0.00030 | up |
| ENSMUSG00000101013 | A630072M18Rik | 4.433611 | 0.00030 | up |
| ENSMUSG00000047735 | Samd9l | -1.02167 | 0.00030 | down |
| ENSMUSG00000104937 | Gm43057 | -3.89022 | 0.00029 | down |
| ENSMUSG00000056071 | S100a9 | 1.912077 | 0.00029 | up |
| ENSMUSG00000069117 | Rps18-ps6 | -2.04928 | 0.00029 | down |
| ENSMUSG00000001036 | Epn2 | -1.06067 | 0.00029 | down |
| ENSMUSG00000033763 | Mtss2 | -1.03477 | 0.00029 | down |
| ENSMUSG00000110755 | BC049987 | 1.185375 | 0.00029 | up |
| ENSMUSG00000110297 | Gm45647 | -4.34086 | 0.00028 | down |
| ENSMUSG00000121349 | Speer6-ps1 | -2.66676 | 0.00028 | down |
| ENSMUSG00000019235 | Rps6kl1 | -1.53144 | 0.00028 | down |
| ENSMUSG00000055150 | Zfp78 | -1.4977 | 0.00028 | down |
| ENSMUSG00000061322 | Dnai1 | 2.383763 | 0.00028 | up |
| ENSMUSG00000031636 | Pdlim3 | -2.28152 | 0.00028 | down |
| ENSMUSG00000029027 | Dffb | -1.14031 | 0.00028 | down |
| ENSMUSG00000120175 | - | -1.23473 | 0.00028 | down |
| ENSMUSG00000115020 | Vmn1r218 | -1.40185 | 0.00028 | down |
| ENSMUSG00000102374 | Gm38387 | -3.465 | 0.00028 | down |
| ENSMUSG00000032011 | Thy1 | -1.51584 | 0.00028 | down |
| ENSMUSG00000092006 | Gm17139 | -1.42265 | 0.00028 | down |
| ENSMUSG00000017167 | Cntnap1 | -1.55954 | 0.00027 | down |
| ENSMUSG00000073409 | H2-Q6 | -1.09915 | 0.00027 | down |
| ENSMUSG00000024983 | Vti1a | 1.373958 | 0.00027 | up |
| ENSMUSG00000103149 | AA914427 | -2.73765 | 0.00027 | down |
| ENSMUSG00000046808 | Atp10d | -1.42691 | 0.00027 | down |
| ENSMUSG00000120379 | - | -3.38737 | 0.00027 | down |
| ENSMUSG00000039962 | Olfr906 | -4.94381 | 0.00027 | down |
| ENSMUSG00000050222 | Il17d | -5.86919 | 0.00027 | down |
| ENSMUSG00000021707 | Dhfr | 1.02251 | 0.00027 | up |
| ENSMUSG00000024896 | Minpp1 | 1.013333 | 0.00027 | up |
| ENSMUSG00000030004 | Nat8 | 1.700945 | 0.00027 | up |
| ENSMUSG00000036158 | Prickle1 | -1.23489 | 0.00027 | down |
| ENSMUSG00000014177 | Tvp23b | 1.202374 | 0.00026 | up |
| ENSMUSG00000102593 | Gm38384 | -4.62997 | 0.00026 | down |
| ENSMUSG00000038370 | Pcp4l1 | -1.17295 | 0.00026 | down |
| ENSMUSG00000112043 | Gm48774 | -1.14058 | 0.00026 | down |
| ENSMUSG00000041992 | Rapgef5 | -1.05188 | 0.00026 | down |
| ENSMUSG00000038903 | Ccdc68 | -1.47394 | 0.00026 | down |
| ENSMUSG00000112332 | 4930466K18Rik | -1.88287 | 0.00026 | down |
| ENSMUSG00000051735 | Rinl | -1.42575 | 0.00026 | down |
| ENSMUSG00000103475 | Gm37697 | -1.24382 | 0.00026 | down |
| ENSMUSG00000120616 | - | -5.07523 | 0.00025 | down |
| ENSMUSG00000034641 | Cd300ld | -1.15365 | 0.00025 | down |
| ENSMUSG00000066829 | Zfp810 | -1.29822 | 0.00025 | down |
| ENSMUSG00000075307 | Klhl41 | -2.83236 | 0.00025 | down |
| ENSMUSG00000061577 | Adgrg5 | -3.11097 | 0.00025 | down |
| ENSMUSG00000056025 | Clca3a1 | -1.00682 | 0.00025 | down |
| ENSMUSG00000030074 | Gxylt2 | -3.30266 | 0.00025 | down |
| ENSMUSG00000079263 | Gm6614 | -4.29509 | 0.00025 | down |
| ENSMUSG00000070713 | Hmgn2-ps | 4.389077 | 0.00025 | up |
| ENSMUSG00000086848 | Lce6a | -5.11512 | 0.00025 | down |
| ENSMUSG00000114138 | Gm36423 | -5.30305 | 0.00024 | down |
| ENSMUSG00000110195 | Pde2a | -1.40291 | 0.00024 | down |
| ENSMUSG00000021539 | Lect2 | 1.055758 | 0.00024 | up |
| ENSMUSG00000030137 | Tuba8 | -1.05873 | 0.00024 | down |
| ENSMUSG00000038070 | Cntln | -1.65667 | 0.00024 | down |
| ENSMUSG00000024851 | Pitpnm1 | -1.18659 | 0.00024 | down |
| ENSMUSG00000114584 | Gm47694 | -2.16721 | 0.00024 | down |
| ENSMUSG00000042203 | Tbc1d22b | -1.0182 | 0.00024 | down |
| ENSMUSG00000092341 | Malat1 | -1.3197 | 0.00024 | down |
| ENSMUSG00000120693 | - | -2.00467 | 0.00024 | down |
| ENSMUSG00000112393 | Gm48655 | -4.34663 | 0.00024 | down |
| ENSMUSG00000114970 | Gm49069 | -2.4746 | 0.00024 | down |
| ENSMUSG00000113101 | Gm33424 | -3.01297 | 0.00024 | down |
| ENSMUSG00000121066 | - | -1.28122 | 0.00024 | down |
| ENSMUSG00000106190 | Gm20768 | -3.79634 | 0.00024 | down |
| ENSMUSG00000041559 | Fmod | -1.36665 | 0.00024 | down |
| ENSMUSG00000030303 | Far2 | -2.03872 | 0.00023 | down |
| ENSMUSG00000022973 | Synj1 | -1.00332 | 0.00023 | down |
| ENSMUSG00000047238 | Mageh1 | 2.120075 | 0.00023 | up |
| ENSMUSG00000110529 | Gm45694 | -2.65665 | 0.00023 | down |
| ENSMUSG00000036168 | Ccdc38 | -3.1016 | 0.00023 | down |
| ENSMUSG00000041187 | Prkd2 | -1.02351 | 0.00023 | down |
| ENSMUSG00000097163 | BC051077 | -3.28416 | 0.00023 | down |
| ENSMUSG00000027997 | Casp6 | 1.078281 | 0.00023 | up |
| ENSMUSG00000113948 | Rpl17-ps3 | -4.45438 | 0.00023 | down |
| ENSMUSG00000107690 | Gm44044 | -2.69395 | 0.00023 | down |
| ENSMUSG00000111517 | Olfr1238 | -2.43469 | 0.00023 | down |
| ENSMUSG00000106032 | Gm42463 | -4.89492 | 0.00022 | down |
| ENSMUSG00000020723 | Cacng4 | -2.75273 | 0.00022 | down |
| ENSMUSG00000056073 | Grik2 | -4.29081 | 0.00022 | down |
| ENSMUSG00000121304 | - | -2.07236 | 0.00022 | down |
| ENSMUSG00000084796 | Mir142hg | -1.58785 | 0.00022 | down |
| ENSMUSG00000117634 | Gm50069 | -2.0496 | 0.00022 | down |
| ENSMUSG00000102289 | Gm31258 | -4.47147 | 0.00022 | down |
| ENSMUSG00000090338 | Gm17081 | -1.41751 | 0.00022 | down |
| ENSMUSG00000108802 | Gm44769 | -4.95582 | 0.00022 | down |
| ENSMUSG00000087579 | Hectd2os | -1.51356 | 0.00022 | down |
| ENSMUSG00000028141 | Oaz3 | -2.9276 | 0.00022 | down |
| ENSMUSG00000027694 | Gm8325 | -1.11853 | 0.00021 | down |
| ENSMUSG00000118698 | Gm25890 | -4.38458 | 0.00021 | down |
| ENSMUSG00000087165 | 2010001A14Rik | 1.640139 | 0.00021 | up |
| ENSMUSG00000118841 | Rn7s2 | -1.97922 | 0.00021 | down |
| ENSMUSG00000118866 | Rn7s1 | -1.97922 | 0.00021 | down |
| ENSMUSG00000028803 | Nipal3 | 1.176994 | 0.00021 | up |
| ENSMUSG00000044122 | Proca1 | -1.04601 | 0.00021 | down |
| ENSMUSG00000038217 | Tlcd2 | 1.129941 | 0.00021 | up |
| ENSMUSG00000031886 | Ces2e | 1.280056 | 0.00021 | up |
| ENSMUSG00000104520 | Gm37336 | -2.77915 | 0.00021 | down |
| ENSMUSG00000026604 | Ptpn14 | -1.21369 | 0.00021 | down |
| ENSMUSG00000085399 | Foxd2os | -3.24097 | 0.00021 | down |
| ENSMUSG00000032763 | Ilvbl | 1.064804 | 0.00021 | up |
| ENSMUSG00000035165 | Kcne3 | -1.32108 | 0.00021 | down |
| ENSMUSG00000031712 | Il15 | -1.53979 | 0.00020 | down |
| ENSMUSG00000036027 | Pou2af2 | 2.544733 | 0.00020 | up |
| ENSMUSG00000052837 | Junb | 2.310559 | 0.00020 | up |
| ENSMUSG00000072494 | Ppp1r3e | -1.13476 | 0.00020 | down |
| ENSMUSG00000082894 | Gm6480 | -2.22204 | 0.00020 | down |
| ENSMUSG00000021010 | Npas3 | -4.22906 | 0.00020 | down |
| ENSMUSG00000047248 | C2cd3 | -1.06216 | 0.00020 | down |
| ENSMUSG00000089417 | Gm22009 | -1.3211 | 0.00020 | down |
| ENSMUSG00000029591 | Ung | 1.223856 | 0.00020 | up |
| ENSMUSG00000066442 | Mthfs | 1.025419 | 0.00020 | up |
| ENSMUSG00000097204 | Gm17690 | -1.20031 | 0.00020 | down |
| ENSMUSG00000001670 | Tat | 1.157957 | 0.00020 | up |
| ENSMUSG00000053862 | Slc51b | 2.279327 | 0.00020 | up |
| ENSMUSG00000001496 | Nkx2-1 | -4.72463 | 0.00019 | down |
| ENSMUSG00000071064 | Zfp827 | -2.01119 | 0.00019 | down |
| ENSMUSG00000030098 | Grip2 | -4.92186 | 0.00019 | down |
| ENSMUSG00000121158 | Gm35162 | -3.0228 | 0.00019 | down |
| ENSMUSG00000093803 | Ppp2r3d | -1.2476 | 0.00019 | down |
| ENSMUSG00000000184 | Ccnd2 | -1.35799 | 0.00019 | down |
| ENSMUSG00000050350 | Gpr18 | -4.04812 | 0.00019 | down |
| ENSMUSG00000049728 | Zfp668 | -1.24595 | 0.00019 | down |
| ENSMUSG00000101906 | Mrgprc2-ps | -2.60253 | 0.00019 | down |
| ENSMUSG00000028188 | Spata1 | -2.09951 | 0.00019 | down |
| ENSMUSG00000010044 | Zmynd10 | -3.08541 | 0.00019 | down |
| ENSMUSG00000018398 | Septin8 | -1.1536 | 0.00019 | down |
| ENSMUSG00000111497 | Gm38431 | -2.9053 | 0.00019 | down |
| ENSMUSG00000042436 | Mfap4 | -1.61719 | 0.00019 | down |
| ENSMUSG00000007035 | Msh5 | -2.37263 | 0.00019 | down |
| ENSMUSG00000091491 | Vmn2r97 | -1.48173 | 0.00019 | down |
| ENSMUSG00000039199 | Zdhhc1 | -1.36788 | 0.00019 | down |
| ENSMUSG00000015812 | Gnrh1 | -3.59084 | 0.00019 | down |
| ENSMUSG00000034659 | Tmem109 | 1.005664 | 0.00018 | up |
| ENSMUSG00000087662 | Gm11373 | -1.97305 | 0.00018 | down |
| ENSMUSG00000003477 | Inmt | 1.086288 | 0.00018 | up |
| ENSMUSG00000009406 | Elk1 | 1.028328 | 0.00018 | up |
| ENSMUSG00000044150 | Bclaf3 | -1.36338 | 0.00018 | down |
| ENSMUSG00000027342 | Pcna | 1.033553 | 0.00018 | up |
| ENSMUSG00000020650 | Bcap29 | 1.14058 | 0.00018 | up |
| ENSMUSG00000018830 | Myh11 | -1.5072 | 0.00018 | down |
| ENSMUSG00000055782 | Abcd2 | 1.230294 | 0.00018 | up |
| ENSMUSG00000026648 | Dclre1c | -1.0605 | 0.00018 | down |
| ENSMUSG00000032725 | Folr2 | 1.22716 | 0.00018 | up |
| ENSMUSG00000031762 | Mt2 | 2.690419 | 0.00018 | up |
| ENSMUSG00000118087 | 4833438C02Rik | -1.29962 | 0.00018 | down |
| ENSMUSG00000108443 | Gm44510 | -2.5866 | 0.00018 | down |
| ENSMUSG00000044244 | Il20rb | -1.57011 | 0.00018 | down |
| ENSMUSG00000091844 | Gm8251 | -4.06856 | 0.00018 | down |
| ENSMUSG00000078651 | Aoc2 | -2.19112 | 0.00018 | down |
| ENSMUSG00000030887 | Pdzd9 | -2.93261 | 0.00018 | down |
| ENSMUSG00000035069 | Oma1 | 1.208796 | 0.00018 | up |
| ENSMUSG00000032860 | P2ry2 | 1.131095 | 0.00018 | up |
| ENSMUSG00000042801 | Olfr769 | -3.60909 | 0.00018 | down |
| ENSMUSG00000038034 | Igsf8 | 1.065345 | 0.00018 | up |
| ENSMUSG00000044258 | Ctla2a | -1.51637 | 0.00018 | down |
| ENSMUSG00000051504 | Siglech | -2.39187 | 0.00018 | down |
| ENSMUSG00000031976 | Urb2 | 1.387464 | 0.00018 | up |
| ENSMUSG00000087470 | A630031M04Rik | -1.18021 | 0.00017 | down |
| ENSMUSG00000073731 | AI507597 | -5.26869 | 0.00017 | down |
| ENSMUSG00000025764 | Jade1 | -1.1024 | 0.00017 | down |
| ENSMUSG00000085774 | Gm13055 | -4.19289 | 0.00017 | down |
| ENSMUSG00000112576 | Gm47621 | -1.92228 | 0.00017 | down |
| ENSMUSG00000032565 | Nudt16 | 1.383575 | 0.00017 | up |
| ENSMUSG00000026207 | Speg | -1.65789 | 0.00017 | down |
| ENSMUSG00000086607 | 4930511M06Rik | -2.00849 | 0.00017 | down |
| ENSMUSG00000022792 | Yars2 | 1.345744 | 0.00017 | up |
| ENSMUSG00000013419 | Zfp651 | -1.5222 | 0.00017 | down |
| ENSMUSG00000025597 | Klhl4 | -3.06949 | 0.00017 | down |
| ENSMUSG00000096967 | Gm26621 | -1.8604 | 0.00016 | down |
| ENSMUSG00000054414 | Slc30a7 | 1.429667 | 0.00016 | up |
| ENSMUSG00000103207 | Gm9874 | -5.83614 | 0.00016 | down |
| ENSMUSG00000045589 | Frrs1l | -3.80968 | 0.00016 | down |
| ENSMUSG00000087404 | Gm11752 | 4.501846 | 0.00016 | up |
| ENSMUSG00000083111 | Gm14421 | 1.383895 | 0.00016 | up |
| ENSMUSG00000006638 | Abhd1 | -1.43189 | 0.00016 | down |
| ENSMUSG00000108378 | Gm44641 | 4.130277 | 0.00016 | up |
| ENSMUSG00000075383 | Olfr351 | -1.38688 | 0.00016 | down |
| ENSMUSG00000061111 | Mcrip1 | 1.877898 | 0.00016 | up |
| ENSMUSG00000021947 | Cryl1 | 1.000594 | 0.00016 | up |
| ENSMUSG00000114469 | C730002L08Rik | -2.4444 | 0.00016 | down |
| ENSMUSG00000106831 | Ube2n-ps1 | 2.633957 | 0.00015 | up |
| ENSMUSG00000053553 | 3110082I17Rik | 1.990474 | 0.00015 | up |
| ENSMUSG00000029128 | Rab28 | 1.054571 | 0.00015 | up |
| ENSMUSG00000033799 | Tasor2 | -1.30477 | 0.00015 | down |
| ENSMUSG00000024268 | Celf4 | -2.10858 | 0.00015 | down |
| ENSMUSG00000118124 | Gm50139 | 4.875378 | 0.00015 | up |
| ENSMUSG00000074577 | Ripor3 | -1.48285 | 0.00015 | down |
| ENSMUSG00000111997 | Gm5176 | -5.19452 | 0.00015 | down |
| ENSMUSG00000023912 | Slc25a27 | -1.95004 | 0.00015 | down |
| ENSMUSG00000033705 | Stard9 | -1.34148 | 0.00015 | down |
| ENSMUSG00000116836 | Gm49727 | -4.0934 | 0.00015 | down |
| ENSMUSG00000044916 | 1700029I15Rik | -1.44821 | 0.00014 | down |
| ENSMUSG00000030772 | Dkk3 | -1.82921 | 0.00014 | down |
| ENSMUSG00000071847 | Apcdd1 | -2.12545 | 0.00014 | down |
| ENSMUSG00000042712 | Tceal9 | 1.424849 | 0.00014 | up |
| ENSMUSG00000052544 | St6galnac3 | -1.80663 | 0.00014 | down |
| ENSMUSG00000121067 | - | -1.91795 | 0.00014 | down |
| ENSMUSG00000107726 | Gm44037 | -4.93624 | 0.00014 | down |
| ENSMUSG00000114501 | Gm48582 | -2.35213 | 0.00014 | down |
| ENSMUSG00000072693 | Gm10401 | -1.07118 | 0.00014 | down |
| ENSMUSG00000025020 | Slit1 | -4.76054 | 0.00014 | down |
| ENSMUSG00000044645 | Gm7334 | 4.388108 | 0.00014 | up |
| ENSMUSG00000081205 | Gm5940 | -4.87716 | 0.00014 | down |
| ENSMUSG00000061829 | Vmn1r214 | -4.44539 | 0.00014 | down |
| ENSMUSG00000003062 | Stard3nl | 1.486757 | 0.00014 | up |
| ENSMUSG00000096852 | Cyp2d12 | -1.26714 | 0.00014 | down |
| ENSMUSG00000003573 | Homer3 | -1.80557 | 0.00014 | down |
| ENSMUSG00000044471 | Lncpint | -2.37918 | 0.00014 | down |
| ENSMUSG00000025500 | Lmntd2 | -1.76512 | 0.00013 | down |
| ENSMUSG00000026082 | Rev1 | -1.02907 | 0.00013 | down |
| ENSMUSG00000058331 | Zfp85 | -1.34935 | 0.00013 | down |
| ENSMUSG00000097617 | Gm10687 | -1.66109 | 0.00013 | down |
| ENSMUSG00000004018 | Fancl | 1.542521 | 0.00013 | up |
| ENSMUSG00000097743 | Gm16973 | -1.44712 | 0.00013 | down |
| ENSMUSG00000102282 | A930032L01Rik | -4.31475 | 0.00013 | down |
| ENSMUSG00000100658 | F730311O21Rik | -1.911 | 0.00013 | down |
| ENSMUSG00000015468 | Notch4 | -1.12055 | 0.00013 | down |
| ENSMUSG00000028643 | Svbp | 1.086228 | 0.00013 | up |
| ENSMUSG00000120173 | - | 2.025705 | 0.00013 | up |
| ENSMUSG00000020495 | Smg8 | 1.110457 | 0.00013 | up |
| ENSMUSG00000036856 | Wnt4 | -1.16786 | 0.00013 | down |
| ENSMUSG00000040855 | Reps2 | 1.266543 | 0.00013 | up |
| ENSMUSG00000037890 | Wdr19 | -1.14901 | 0.00013 | down |
| ENSMUSG00000085702 | Mecomos | -5.60771 | 0.00013 | down |
| ENSMUSG00000016758 | Bik | -1.19697 | 0.00013 | down |
| ENSMUSG00000083718 | Ccnb2-ps | -2.28878 | 0.00013 | down |
| ENSMUSG00000104159 | Gm38099 | -1.80725 | 0.00013 | down |
| ENSMUSG00000113663 | Gm48504 | -3.17574 | 0.00013 | down |
| ENSMUSG00000100213 | Gm28151 | -2.19888 | 0.00013 | down |
| ENSMUSG00000092399 | 4930556N13Rik | -3.8 | 0.00012 | down |
| ENSMUSG00000037661 | Gpr160 | -1.77128 | 0.00012 | down |
| ENSMUSG00000079610 | Ankrd39 | 1.676331 | 0.00012 | up |
| ENSMUSG00000021884 | Hacl1 | 1.055199 | 0.00012 | up |
| ENSMUSG00000051255 | Gm6563 | -5.21563 | 0.00012 | down |
| ENSMUSG00000052188 | Gm14964 | -2.23759 | 0.00012 | down |
| ENSMUSG00000095675 | Ccl21b | -2.15519 | 0.00012 | down |
| ENSMUSG00000096596 | Gm10591 | -2.15519 | 0.00012 | down |
| ENSMUSG00000042705 | Commd10 | 1.509803 | 0.00012 | up |
| ENSMUSG00000000579 | Dynlt1c | -1.21726 | 0.00012 | down |
| ENSMUSG00000112149 | Gm47611 | -1.2935 | 0.00012 | down |
| ENSMUSG00000043310 | Olfr571 | -1.82983 | 0.00012 | down |
| ENSMUSG00000112461 | Gm47625 | -4.63673 | 0.00012 | down |
| ENSMUSG00000026930 | Gpsm1 | -2.07771 | 0.00012 | down |
| ENSMUSG00000031832 | Taf1c | -1.41394 | 0.00012 | down |
| ENSMUSG00000102591 | Gm38383 | -1.60442 | 0.00012 | down |
| ENSMUSG00000051185 | Fam174a | 1.134973 | 0.00012 | up |
| ENSMUSG00000025371 | Chmp6 | 1.162893 | 0.00012 | up |
| ENSMUSG00000035863 | Palm | -1.38405 | 0.00011 | down |
| ENSMUSG00000111883 | Gm31182 | -1.78936 | 0.00011 | down |
| ENSMUSG00000035168 | Tanc1 | -1.117 | 0.00011 | down |
| ENSMUSG00000035873 | Pawr | 2.071082 | 0.00011 | up |
| ENSMUSG00000020889 | Nr1d1 | 2.430716 | 0.00011 | up |
| ENSMUSG00000091478 | Gm10039 | -6.14198 | 0.00011 | down |
| ENSMUSG00000041567 | Serpina12 | -1.60982 | 0.00011 | down |
| ENSMUSG00000091898 | Tnnc1 | -2.60393 | 0.00011 | down |
| ENSMUSG00000103839 | Gm37607 | -1.71888 | 0.00011 | down |
| ENSMUSG00000101447 | Gm28826 | -4.98805 | 0.00011 | down |
| ENSMUSG00000034258 | Flvcr2 | 1.623667 | 0.00011 | up |
| ENSMUSG00000106924 | Gm42857 | -1.80654 | 0.00011 | down |
| ENSMUSG00000116617 | Gm49767 | -2.45628 | 0.00011 | down |
| ENSMUSG00000049957 | Ccdc137 | -1.01697 | 0.00011 | down |
| ENSMUSG00000112806 | Gm48146 | -3.2545 | 0.00011 | down |
| ENSMUSG00000046718 | Bst2 | 1.060968 | 0.00011 | up |
| ENSMUSG00000069324 | Gm5096 | 4.094702 | 0.00011 | up |
| ENSMUSG00000030942 | Thumpd1 | 1.192808 | 0.00011 | up |
| ENSMUSG00000120169 | - | -1.72617 | 0.00011 | down |
| ENSMUSG00000015619 | Gata3 | -2.29353 | 0.00011 | down |
| ENSMUSG00000032355 | Mlip | -4.04858 | 0.00011 | down |
| ENSMUSG00000110547 | Gm29773 | -3.22855 | 0.00011 | down |
| ENSMUSG00000026854 | Usp20 | -1.14256 | 0.00011 | down |
| ENSMUSG00000021497 | Txndc15 | 1.202689 | 0.00011 | up |
| ENSMUSG00000053253 | Ndfip2 | 1.105098 | 0.00011 | up |
| ENSMUSG00000026558 | Uck2 | 1.077796 | 0.00011 | up |
| ENSMUSG00000115368 | Gm48942 | -2.1567 | 0.00011 | down |
| ENSMUSG00000120630 | - | -2.36063 | 0.00011 | down |
| ENSMUSG00000014349 | Ube2z | 1.061634 | 0.00010 | up |
| ENSMUSG00000003680 | Taf6l | 2.124526 | 0.00010 | up |
| ENSMUSG00002076083 | Klhl17 | -1.06256 | 0.00010 | down |
| ENSMUSG00000092511 | Gm20547 | 6.624492 | 0.00010 | up |
| ENSMUSG00000035559 | Mpv17l2 | 1.381214 | 0.00010 | up |
| ENSMUSG00000031109 | Enox2 | 1.067282 | 0.00010 | up |
| ENSMUSG00000044165 | Bcl2l15 | -4.68838 | 0.00010 | down |
| ENSMUSG00000029605 | Oas1b | -2.01163 | 0.00010 | down |
| ENSMUSG00000026209 | Dnpep | 1.093999 | 0.00010 | up |
| ENSMUSG00000103656 | Gm37205 | -5.84653 | 0.00010 | down |
| ENSMUSG00000062604 | Srpk2 | -1.0004 | 0.00010 | down |
| ENSMUSG00000039182 | AW209491 | 1.073085 | 0.00010 | up |
| ENSMUSG00000022814 | Umps | 1.094343 | 0.00010 | up |
| ENSMUSG00000031220 | Awat2 | -1.70043 | 0.00010 | down |
| ENSMUSG00000006154 | Eps8l1 | -1.66534 | 0.00010 | down |
| ENSMUSG00000073274 | Gm14636 | -1.89296 | 0.00009 | down |
| ENSMUSG00000022856 | Tmem41a | 1.710322 | 0.00009 | up |
| ENSMUSG00000018362 | Kpna2 | 1.514562 | 0.00009 | up |
| ENSMUSG00000030750 | Nsmce1 | 1.284179 | 0.00009 | up |
| ENSMUSG00000103085 | Gm38120 | -3.91531 | 0.00009 | down |
| ENSMUSG00000032827 | Ppp1r9a | -1.29292 | 0.00009 | down |
| ENSMUSG00000120082 | - | -2.29903 | 0.00009 | down |
| ENSMUSG00000019775 | Rgs17 | -2.89214 | 0.00009 | down |
| ENSMUSG00000114226 | Gm48412 | -4.16058 | 0.00009 | down |
| ENSMUSG00000058656 | Samd12 | -3.51754 | 0.00009 | down |
| ENSMUSG00000032737 | Inppl1 | -1.00677 | 0.00009 | down |
| ENSMUSG00000089942 | Pira2 | 3.147762 | 0.00009 | up |
| ENSMUSG00000035722 | Abca7 | -1.21116 | 0.00009 | down |
| ENSMUSG00000036435 | Exoc1 | -1.01328 | 0.00009 | down |
| ENSMUSG00000000296 | Tpd52l1 | 1.459298 | 0.00009 | up |
| ENSMUSG00000074813 | Morrbid | -2.11175 | 0.00009 | down |
| ENSMUSG00000046101 | Mcmdc2 | -2.25856 | 0.00009 | down |
| ENSMUSG00000029033 | Acap3 | -1.21252 | 0.00009 | down |
| ENSMUSG00000046711 | Hmga1 | 1.159287 | 0.00009 | up |
| ENSMUSG00000117853 | Vmn1r88 | -2.01627 | 0.00009 | down |
| ENSMUSG00000054938 | Olfr1346 | -4.63293 | 0.00009 | down |
| ENSMUSG00000026821 | Ralgds | -1.22295 | 0.00009 | down |
| ENSMUSG00000024391 | Apom | 1.105762 | 0.00009 | up |
| ENSMUSG00000030309 | Caprin2 | -1.46443 | 0.00009 | down |
| ENSMUSG00000057948 | Unc13d | -1.66547 | 0.00009 | down |
| ENSMUSG00000121201 | - | -3.37879 | 0.00009 | down |
| ENSMUSG00000038500 | Prr3 | -1.00017 | 0.00009 | down |
| ENSMUSG00000049488 | Tmem67 | -1.21282 | 0.00009 | down |
| ENSMUSG00000115509 | Gm49012 | -3.34589 | 0.00009 | down |
| ENSMUSG00000016495 | Plgrkt | -1.06004 | 0.00009 | down |
| ENSMUSG00000116962 | Gm19000 | -4.57369 | 0.00008 | down |
| ENSMUSG00000115105 | Gm49238 | -2.43321 | 0.00008 | down |
| ENSMUSG00000040118 | Cacna2d1 | -1.71255 | 0.00008 | down |
| ENSMUSG00000090362 | Vmn2r79 | -2.01681 | 0.00008 | down |
| ENSMUSG00000018861 | Fdxr | -1.06567 | 0.00008 | down |
| ENSMUSG00000120369 | - | -1.66075 | 0.00008 | down |
| ENSMUSG00000110344 | Smim36 | 1.549954 | 0.00008 | up |
| ENSMUSG00000009145 | Dqx1 | -1.22712 | 0.00008 | down |
| ENSMUSG00000024425 | Ndfip1 | 1.033361 | 0.00008 | up |
| ENSMUSG00000069631 | Strada | -1.27561 | 0.00008 | down |
| ENSMUSG00000120250 | - | -1.34567 | 0.00008 | down |
| ENSMUSG00000026333 | Gin1 | -1.16047 | 0.00008 | down |
| ENSMUSG00000052331 | Ankrd44 | -1.33905 | 0.00008 | down |
| ENSMUSG00000057219 | Armc7 | -1.0729 | 0.00008 | down |
| ENSMUSG00000120681 | - | -1.06809 | 0.00008 | down |
| ENSMUSG00000020018 | Snrpf | 1.044764 | 0.00008 | up |
| ENSMUSG00000107742 | 9530085L11Rik | -3.55635 | 0.00008 | down |
| ENSMUSG00000046688 | Tifa | 1.063767 | 0.00008 | up |
| ENSMUSG00000024590 | Lmnb1 | 1.519931 | 0.00008 | up |
| ENSMUSG00000017057 | Il13ra1 | 2.070673 | 0.00008 | up |
| ENSMUSG00000030905 | Crym | 3.613652 | 0.00008 | up |
| ENSMUSG00000033538 | Casp4 | -1.88805 | 0.00008 | down |
| ENSMUSG00000038244 | Mical2 | -1.16005 | 0.00008 | down |
| ENSMUSG00000094488 | Olfr393 | -2.42644 | 0.00008 | down |
| ENSMUSG00000061273 | Mmgt1 | 1.027466 | 0.00008 | up |
| ENSMUSG00000048546 | Tob2 | 1.01632 | 0.00008 | up |
| ENSMUSG00000099590 | C330022C24Rik | -2.29859 | 0.00008 | down |
| ENSMUSG00000041757 | Plekha6 | -1.03287 | 0.00008 | down |
| ENSMUSG00000007880 | Arid1a | -1.00813 | 0.00007 | down |
| ENSMUSG00000036667 | Tcaf1 | -1.10699 | 0.00007 | down |
| ENSMUSG00000109291 | Gm2814 | -1.39624 | 0.00007 | down |
| ENSMUSG00000113200 | Gm48632 | -2.35909 | 0.00007 | down |
| ENSMUSG00000020231 | Dip2a | -1.22472 | 0.00007 | down |
| ENSMUSG00000032561 | Acpp | 2.596999 | 0.00007 | up |
| ENSMUSG00000029094 | Afap1 | -1.39757 | 0.00007 | down |
| ENSMUSG00000104523 | Gm37335 | -1.30675 | 0.00007 | down |
| ENSMUSG00000020156 | Pwwp3a | -1.02182 | 0.00007 | down |
| ENSMUSG00000024029 | Tff3 | 2.154 | 0.00007 | up |
| ENSMUSG00000120209 | - | -3.09079 | 0.00007 | down |
| ENSMUSG00000051149 | Adnp | -5.6419 | 0.00007 | down |
| ENSMUSG00000020886 | Dlg4 | -1.68403 | 0.00007 | down |
| ENSMUSG00000020721 | Helz | -1.03665 | 0.00007 | down |
| ENSMUSG00000091985 | Gm17354 | -1.11855 | 0.00007 | down |
| ENSMUSG00000096795 | Zfp433 | -2.09646 | 0.00007 | down |
| ENSMUSG00000022840 | Adcy5 | -1.54767 | 0.00007 | down |
| ENSMUSG00000102211 | Gm37490 | -2.97282 | 0.00007 | down |
| ENSMUSG00000040312 | Cchcr1 | -1.27201 | 0.00007 | down |
| ENSMUSG00000076749 | Trgc1 | -4.18117 | 0.00007 | down |
| ENSMUSG00000071661 | Zbtb3 | -4.00865 | 0.00007 | down |
| ENSMUSG00000083364 | Llph-ps2 | 2.058961 | 0.00007 | up |
| ENSMUSG00000094686 | Ccl21a | -2.01213 | 0.00007 | down |
| ENSMUSG00000030499 | Kctd15 | -1.11244 | 0.00007 | down |
| ENSMUSG00000040121 | Rep15 | -2.25671 | 0.00007 | down |
| ENSMUSG00000067149 | Jchain | -1.22022 | 0.00007 | down |
| ENSMUSG00000092837 | Rpph1 | -2.30251 | 0.00007 | down |
| ENSMUSG00000049173 | Myoz3 | -3.53823 | 0.00007 | down |
| ENSMUSG00000048826 | Dact2 | 1.49429 | 0.00007 | up |
| ENSMUSG00000015013 | Trappc2l | 1.051963 | 0.00007 | up |
| ENSMUSG00000085385 | Snhg17 | -1.02354 | 0.00006 | down |
| ENSMUSG00000066071 | Cyp4a12a | -1.12302 | 0.00006 | down |
| ENSMUSG00000009630 | Ppp2cb | 1.070608 | 0.00006 | up |
| ENSMUSG00000029438 | Bcl7a | -1.11644 | 0.00006 | down |
| ENSMUSG00000086429 | Gt(ROSA)26Sor | -1.50522 | 0.00006 | down |
| ENSMUSG00000038271 | Iffo1 | -1.22351 | 0.00006 | down |
| ENSMUSG00000112855 | Gm47842 | -2.12758 | 0.00006 | down |
| ENSMUSG00000109536 | 9330162G02Rik | -2.04066 | 0.00006 | down |
| ENSMUSG00000030431 | Tmem238 | 1.741415 | 0.00006 | up |
| ENSMUSG00000022218 | Tgm1 | 1.324839 | 0.00006 | up |
| ENSMUSG00000028444 | Cntfr | -1.44557 | 0.00006 | down |
| ENSMUSG00000048603 | Gm9828 | -1.7348 | 0.00006 | down |
| ENSMUSG00000057359 | Gm17494 | -1.62852 | 0.00006 | down |
| ENSMUSG00000059851 | Kmt5c | -1.07887 | 0.00006 | down |
| ENSMUSG00000036054 | Sugp2 | -1.01218 | 0.00006 | down |
| ENSMUSG00000039206 | Daglb | 1.213219 | 0.00006 | up |
| ENSMUSG00000117358 | Gm31645 | -1.8091 | 0.00006 | down |
| ENSMUSG00000054836 | Elp6 | -1.01819 | 0.00006 | down |
| ENSMUSG00000120926 | - | -3.5699 | 0.00006 | down |
| ENSMUSG00000069939 | Gm12070 | -1.25041 | 0.00006 | down |
| ENSMUSG00000035699 | Slc51a | -1.68443 | 0.00006 | down |
| ENSMUSG00000113098 | Gm2912 | -2.93122 | 0.00006 | down |
| ENSMUSG00000005952 | Trpv1 | -4.35925 | 0.00006 | down |
| ENSMUSG00000031278 | Acsl4 | 1.050473 | 0.00006 | up |
| ENSMUSG00000105207 | Gm42927 | -1.62304 | 0.00006 | down |
| ENSMUSG00000037624 | Kcnk2 | -3.12316 | 0.00006 | down |
| ENSMUSG00000026414 | Tnnt2 | -1.92028 | 0.00006 | down |
| ENSMUSG00000058402 | Zfp420 | -1.46019 | 0.00006 | down |
| ENSMUSG00000113262 | Gm48551 | -2.92863 | 0.00006 | down |
| ENSMUSG00000104413 | Gm37065 | -4.23066 | 0.00006 | down |
| ENSMUSG00000046080 | Clec9a | -1.77735 | 0.00006 | down |
| ENSMUSG00000120850 | - | 3.146511 | 0.00006 | up |
| ENSMUSG00000118607 | Gm7592 | -1.71643 | 0.00006 | down |
| ENSMUSG00000062785 | Kcnc3 | -1.42926 | 0.00006 | down |
| ENSMUSG00000029149 | Krtcap3 | -1.86896 | 0.00006 | down |
| ENSMUSG00000028454 | Pigo | -1.01021 | 0.00006 | down |
| ENSMUSG00000115329 | Gm49272 | -2.92953 | 0.00006 | down |
| ENSMUSG00000025888 | Casp1 | -1.64263 | 0.00006 | down |
| ENSMUSG00000036241 | Ube2r2 | -1.28131 | 0.00006 | down |
| ENSMUSG00000026385 | Dbi | 1.126123 | 0.00006 | up |
| ENSMUSG00000105740 | Gm42685 | -5.28732 | 0.00005 | down |
| ENSMUSG00000081485 | Gm12338 | -2.15778 | 0.00005 | down |
| ENSMUSG00000028251 | Tstd3 | 1.016572 | 0.00005 | up |
| ENSMUSG00000114401 | Gm38604 | -3.91219 | 0.00005 | down |
| ENSMUSG00000038042 | Ptpdc1 | -1.44848 | 0.00005 | down |
| ENSMUSG00000023064 | Sncg | -2.93147 | 0.00005 | down |
| ENSMUSG00000022151 | Ttc33 | 1.298783 | 0.00005 | up |
| ENSMUSG00000107743 | Gm44087 | -1.56652 | 0.00005 | down |
| ENSMUSG00000015377 | Dennd6b | -1.14251 | 0.00005 | down |
| ENSMUSG00000038602 | Slc35f1 | -4.00522 | 0.00005 | down |
| ENSMUSG00000085078 | C030013C21Rik | -4.65946 | 0.00005 | down |
| ENSMUSG00000034401 | Spata6 | -1.66735 | 0.00005 | down |
| ENSMUSG00000027859 | Ngf | -1.0122 | 0.00005 | down |
| ENSMUSG00000032112 | Trappc4 | 1.360453 | 0.00005 | up |
| ENSMUSG00000111269 | Gm47933 | -1.71814 | 0.00005 | down |
| ENSMUSG00000030588 | Yif1b | 1.205213 | 0.00005 | up |
| ENSMUSG00000058360 | Gm10040 | -2.38688 | 0.00005 | down |
| ENSMUSG00000105192 | Gm42796 | 3.94958 | 0.00005 | up |
| ENSMUSG00000110899 | Gm48840 | -1.8148 | 0.00005 | down |
| ENSMUSG00000026409 | Pfkfb2 | -1.23855 | 0.00005 | down |
| ENSMUSG00000046962 | Zbtb21 | -1.22617 | 0.00005 | down |
| ENSMUSG00000019846 | Lama4 | -1.19336 | 0.00005 | down |
| ENSMUSG00000001131 | Timp1 | 2.78388 | 0.00005 | up |
| ENSMUSG00000103273 | Gm37913 | -2.9152 | 0.00005 | down |
| ENSMUSG00000032786 | Alas1 | 1.264407 | 0.00005 | up |
| ENSMUSG00000034111 | Tmed8 | -1.1871 | 0.00005 | down |
| ENSMUSG00000002831 | Plin4 | -2.04005 | 0.00005 | down |
| ENSMUSG00000022999 | Lmbr1l | -1.00151 | 0.00005 | down |
| ENSMUSG00000022769 | Sdf2l1 | 1.225221 | 0.00005 | up |
| ENSMUSG00000042404 | Dennd4b | -1.42083 | 0.00005 | down |
| ENSMUSG00000027478 | Dnmt3b | -1.50816 | 0.00005 | down |
| ENSMUSG00000021027 | Ralgapa1 | -1.07945 | 0.00005 | down |
| ENSMUSG00000056148 | Rdh9 | 1.400028 | 0.00005 | up |
| ENSMUSG00000034810 | Scn7a | -2.15062 | 0.00004 | down |
| ENSMUSG00000040852 | Plekhh2 | -2.08264 | 0.00004 | down |
| ENSMUSG00000096220 | Olfr775 | -2.2314 | 0.00004 | down |
| ENSMUSG00000084846 | A730011C13Rik | -2.10075 | 0.00004 | down |
| ENSMUSG00000112757 | Gm10743 | -4.83759 | 0.00004 | down |
| ENSMUSG00000120725 | - | -1.55373 | 0.00004 | down |
| ENSMUSG00000020311 | Erlec1 | 1.233284 | 0.00004 | up |
| ENSMUSG00000046650 | Olfr1440 | -3.62401 | 0.00004 | down |
| ENSMUSG00000092008 | Cyp2c69 | -2.52356 | 0.00004 | down |
| ENSMUSG00000038485 | Socs7 | -1.05861 | 0.00004 | down |
| ENSMUSG00000041762 | Gpr155 | 1.168576 | 0.00004 | up |
| ENSMUSG00000053693 | Mast1 | -3.29744 | 0.00004 | down |
| ENSMUSG00000022885 | St6gal1 | 1.340597 | 0.00004 | up |
| ENSMUSG00000054598 | 9130230L23Rik | -4.27737 | 0.00004 | down |
| ENSMUSG00000086150 | Bach2os | -2.6338 | 0.00004 | down |
| ENSMUSG00000045790 | Ccdc149 | 1.637933 | 0.00004 | up |
| ENSMUSG00000005533 | Igf1r | -1.264 | 0.00004 | down |
| ENSMUSG00000025521 | Tmem192 | 1.318169 | 0.00004 | up |
| ENSMUSG00000073460 | Pnldc1 | -2.57986 | 0.00004 | down |
| ENSMUSG00000032405 | Pias1 | -1.00124 | 0.00004 | down |
| ENSMUSG00000018378 | Cuedc1 | -1.28444 | 0.00004 | down |
| ENSMUSG00000041974 | Spidr | 2.883678 | 0.00004 | up |
| ENSMUSG00000097328 | Tnfsf12 | 1.013596 | 0.00004 | up |
| ENSMUSG00000033669 | Zfp7 | -1.29913 | 0.00004 | down |
| ENSMUSG00000001930 | Vwf | -1.09386 | 0.00004 | down |
| ENSMUSG00000110047 | A230085B16Rik | -2.93695 | 0.00004 | down |
| ENSMUSG00000028248 | Pnisr | -1.00384 | 0.00004 | down |
| ENSMUSG00000103039 | Gm37123 | -1.92795 | 0.00004 | down |
| ENSMUSG00000106863 | Gm42109 | -3.16725 | 0.00004 | down |
| ENSMUSG00000109368 | Gm45015 | -5.46129 | 0.00004 | down |
| ENSMUSG00000032098 | Treh | 3.155138 | 0.00004 | up |
| ENSMUSG00000005514 | Por | 1.256736 | 0.00004 | up |
| ENSMUSG00000021670 | Hmgcr | 1.169567 | 0.00004 | up |
| ENSMUSG00000002015 | Bcap31 | 1.1103 | 0.00004 | up |
| ENSMUSG00000034837 | Gnat1 | 2.194473 | 0.00004 | up |
| ENSMUSG00000102647 | Gm38024 | -3.09417 | 0.00004 | down |
| ENSMUSG00000026162 | Nhej1 | -1.42479 | 0.00004 | down |
| ENSMUSG00000023232 | Serinc2 | 1.341933 | 0.00004 | up |
| ENSMUSG00000030089 | Slc41a3 | 2.730895 | 0.00004 | up |
| ENSMUSG00000044737 | Klk14 | -3.19293 | 0.00004 | down |
| ENSMUSG00000028634 | Hivep3 | -1.3909 | 0.00004 | down |
| ENSMUSG00000090290 | Tarbp1 | -1.10736 | 0.00004 | down |
| ENSMUSG00000036639 | Nudt1 | 1.295746 | 0.00004 | up |
| ENSMUSG00000032932 | Hspa13 | 1.423061 | 0.00004 | up |
| ENSMUSG00000074965 | Olfr1277 | -4.15543 | 0.00003 | down |
| ENSMUSG00000059325 | Hopx | 1.342698 | 0.00003 | up |
| ENSMUSG00000110266 | Gm32742 | -3.17556 | 0.00003 | down |
| ENSMUSG00000020544 | Cox11 | 1.695497 | 0.00003 | up |
| ENSMUSG00000015087 | Rabl6 | -1.15482 | 0.00003 | down |
| ENSMUSG00000108037 | Gm44597 | -1.86957 | 0.00003 | down |
| ENSMUSG00000051246 | Msantd1 | -2.86493 | 0.00003 | down |
| ENSMUSG00000111844 | Gm47097 | -4.24141 | 0.00003 | down |
| ENSMUSG00000030513 | Pcsk6 | 1.19283 | 0.00003 | up |
| ENSMUSG00000020917 | Acly | 1.165024 | 0.00003 | up |
| ENSMUSG00000029797 | Sspo | -2.68305 | 0.00003 | down |
| ENSMUSG00000021509 | Slc25a48 | 1.046855 | 0.00003 | up |
| ENSMUSG00000034774 | Dsg1c | 1.600557 | 0.00003 | up |
| ENSMUSG00000019945 | Cabcoco1 | 2.512246 | 0.00003 | up |
| ENSMUSG00000112947 | Gm47493 | -1.39945 | 0.00003 | down |
| ENSMUSG00000019467 | Arhgef25 | -1.92963 | 0.00003 | down |
| ENSMUSG00000027952 | Pmvk | 1.846808 | 0.00003 | up |
| ENSMUSG00000108633 | Gm44694 | -4.20095 | 0.00003 | down |
| ENSMUSG00000035297 | Cops4 | 1.167736 | 0.00003 | up |
| ENSMUSG00000118661 | Muc6 | -2.6177 | 0.00003 | down |
| ENSMUSG00000038400 | Pmepa1 | -1.2677 | 0.00003 | down |
| ENSMUSG00000026972 | Arrdc1 | -1.08713 | 0.00003 | down |
| ENSMUSG00000121485 | - | -1.96113 | 0.00003 | down |
| ENSMUSG00000031146 | Plp2 | -1.20159 | 0.00003 | down |
| ENSMUSG00000045349 | Sh2d5 | -4.27244 | 0.00003 | down |
| ENSMUSG00000020740 | Gga3 | -1.18497 | 0.00003 | down |
| ENSMUSG00000044197 | Gpr146 | 1.148047 | 0.00003 | up |
| ENSMUSG00000067818 | Myl9 | -1.29365 | 0.00003 | down |
| ENSMUSG00000070436 | Serpinh1 | -1.01351 | 0.00003 | down |
| ENSMUSG00000070348 | Ccnd1 | 1.364667 | 0.00003 | up |
| ENSMUSG00000116835 | Gm49594 | -2.71582 | 0.00003 | down |
| ENSMUSG00000015568 | Lpl | -1.34067 | 0.00003 | down |
| ENSMUSG00000089995 | Gm15716 | -4.93421 | 0.00003 | down |
| ENSMUSG00000029516 | Cit | -2.1085 | 0.00003 | down |
| ENSMUSG00000091144 | Phf11c | -1.67651 | 0.00003 | down |
| ENSMUSG00000052291 | 5330438D12Rik | -1.9854 | 0.00003 | down |
| ENSMUSG00000024365 | Cyp21a1 | -2.25256 | 0.00003 | down |
| ENSMUSG00000020805 | Slc13a5 | 2.391209 | 0.00003 | up |
| ENSMUSG00000053178 | Mterf1b | -1.75159 | 0.00003 | down |
| ENSMUSG00000121494 | 9030619P08Rik | 1.571038 | 0.00003 | up |
| ENSMUSG00000091542 | Gm17167 | -1.62963 | 0.00003 | down |
| ENSMUSG00000053080 | Zfta | -1.15204 | 0.00003 | down |
| ENSMUSG00000021252 | Erg28 | 2.013565 | 0.00003 | up |
| ENSMUSG00000037361 | Sf3b6 | 1.823427 | 0.00003 | up |
| ENSMUSG00000033486 | Catsper2 | -1.37047 | 0.00003 | down |
| ENSMUSG00000028189 | Ctbs | 1.341183 | 0.00003 | up |
| ENSMUSG00000113204 | Gm46430 | -1.1311 | 0.00003 | down |
| ENSMUSG00000036246 | Gmip | -1.5176 | 0.00003 | down |
| ENSMUSG00000036167 | Pphln1 | 1.063115 | 0.00003 | up |
| ENSMUSG00000036880 | Acaa2 | 1.207477 | 0.00003 | up |
| ENSMUSG00000054142 | Vmn1r236 | -3.15836 | 0.00003 | down |
| ENSMUSG00000026035 | Ppil3 | 1.216783 | 0.00003 | up |
| ENSMUSG00000032667 | Pon2 | 1.218521 | 0.00003 | up |
| ENSMUSG00000062563 | Cys1 | -1.61261 | 0.00003 | down |
| ENSMUSG00000078453 | Abracl | 1.516269 | 0.00003 | up |
| ENSMUSG00000035299 | Mid1 | -1.55666 | 0.00003 | down |
| ENSMUSG00000071645 | Tut1 | -1.01287 | 0.00003 | down |
| ENSMUSG00000057363 | Uxs1 | 1.482746 | 0.00003 | up |
| ENSMUSG00000021650 | Ptcd2 | 1.218806 | 0.00002 | up |
| ENSMUSG00000066838 | Zfp772 | -1.55422 | 0.00002 | down |
| ENSMUSG00000121463 | Ccl19-ps2 | -2.29051 | 0.00002 | down |
| ENSMUSG00000024900 | Cpt1a | 1.10484 | 0.00002 | up |
| ENSMUSG00000029752 | Asns | 2.726861 | 0.00002 | up |
| ENSMUSG00000063445 | Nmral1 | 1.107115 | 0.00002 | up |
| ENSMUSG00000005583 | Mef2c | -1.63685 | 0.00002 | down |
| ENSMUSG00000025208 | Mrpl43 | 1.019196 | 0.00002 | up |
| ENSMUSG00000032575 | Manf | 1.301604 | 0.00002 | up |
| ENSMUSG00000106743 | Gm42847 | -3.67638 | 0.00002 | down |
| ENSMUSG00000055923 | Aasdh | -1.31337 | 0.00002 | down |
| ENSMUSG00000031534 | Smim19 | 1.697826 | 0.00002 | up |
| ENSMUSG00000104524 | Gm37333 | -5.65905 | 0.00002 | down |
| ENSMUSG00000085433 | Gm16001 | -1.60817 | 0.00002 | down |
| ENSMUSG00000006576 | Slc4a3 | -2.23892 | 0.00002 | down |
| ENSMUSG00000108500 | Gm45033 | -2.2844 | 0.00002 | down |
| ENSMUSG00000109249 | Gm44610 | -2.02757 | 0.00002 | down |
| ENSMUSG00000074166 | AW146154 | -1.04175 | 0.00002 | down |
| ENSMUSG00000050855 | Zfp940 | -2.03137 | 0.00002 | down |
| ENSMUSG00000062729 | Ppox | -1.04328 | 0.00002 | down |
| ENSMUSG00000048583 | Igf2 | -2.06356 | 0.00002 | down |
| ENSMUSG00000020432 | Tcn2 | 1.056106 | 0.00002 | up |
| ENSMUSG00000044676 | Zfp612 | -1.12265 | 0.00002 | down |
| ENSMUSG00000102868 | Gm37633 | -4.83387 | 0.00002 | down |
| ENSMUSG00000019301 | Hsd17b1 | -3.31332 | 0.00002 | down |
| ENSMUSG00000066538 | Gm6254 | -2.20527 | 0.00002 | down |
| ENSMUSG00000087054 | Gm12405 | -3.57508 | 0.00002 | down |
| ENSMUSG00000002250 | Ppard | -1.85396 | 0.00002 | down |
| ENSMUSG00000028322 | Exosc3 | 1.288106 | 0.00002 | up |
| ENSMUSG00000078762 | Haus5 | -1.15287 | 0.00002 | down |
| ENSMUSG00000082938 | Gm2810 | -5.11739 | 0.00002 | down |
| ENSMUSG00000023084 | Lrrc71 | -1.09322 | 0.00002 | down |
| ENSMUSG00000112354 | Gm33843 | -4.42608 | 0.00002 | down |
| ENSMUSG00000032415 | Ube2cbp | 2.341807 | 0.00002 | up |
| ENSMUSG00000020773 | Trim47 | -1.20975 | 0.00002 | down |
| ENSMUSG00000027284 | Cdan1 | -1.08635 | 0.00002 | down |
| ENSMUSG00000039474 | Wfs1 | 1.510335 | 0.00002 | up |
| ENSMUSG00000111521 | Gm48529 | -1.58325 | 0.00002 | down |
| ENSMUSG00000097571 | Jpx | -1.0668 | 0.00002 | down |
| ENSMUSG00000070458 | Vmn2r73 | -3.1197 | 0.00002 | down |
| ENSMUSG00000028717 | Tal1 | -1.84906 | 0.00002 | down |
| ENSMUSG00000001665 | Gstt3 | 1.406743 | 0.00002 | up |
| ENSMUSG00000003161 | Sri | 1.121841 | 0.00002 | up |
| ENSMUSG00000104621 | Gm43185 | -3.57336 | 0.00002 | down |
| ENSMUSG00000043964 | Orai3 | 1.18232 | 0.00002 | up |
| ENSMUSG00000112505 | Gm48610 | -3.6042 | 0.00002 | down |
| ENSMUSG00000027533 | Fabp5 | 2.304871 | 0.00002 | up |
| ENSMUSG00000030595 | Nfkbib | 1.172345 | 0.00002 | up |
| ENSMUSG00000018567 | Gabarap | 1.030279 | 0.00002 | up |
| ENSMUSG00000027716 | Trpc3 | -2.88143 | 0.00002 | down |
| ENSMUSG00000031767 | Nudt7 | 1.092355 | 0.00002 | up |
| ENSMUSG00000086290 | Snhg12 | -1.16804 | 0.00002 | down |
| ENSMUSG00000021725 | Parp8 | -1.74573 | 0.00002 | down |
| ENSMUSG00000028194 | Ddah1 | 1.056554 | 0.00002 | up |
| ENSMUSG00000000916 | Nsun5 | -1.15696 | 0.00002 | down |
| ENSMUSG00000109781 | Gm45509 | -3.26899 | 0.00002 | down |
| ENSMUSG00000041840 | Haus1 | -1.0598 | 0.00002 | down |
| ENSMUSG00000001248 | Gramd1a | -1.01644 | 0.00002 | down |
| ENSMUSG00000033054 | Npat | -1.17503 | 0.00002 | down |
| ENSMUSG00000048731 | Ggnbp1 | -1.06314 | 0.00002 | down |
| ENSMUSG00000054252 | Fgfr3 | -1.18704 | 0.00002 | down |
| ENSMUSG00000034793 | G6pc3 | -1.39946 | 0.00002 | down |
| ENSMUSG00000010376 | Nedd8 | 1.016861 | 0.00002 | up |
| ENSMUSG00000060512 | 0610040J01Rik | 1.033284 | 0.00002 | up |
| ENSMUSG00000078680 | Mup10 | -2.00824 | 0.00002 | down |
| ENSMUSG00000025169 | Ogfod3 | 1.027574 | 0.00002 | up |
| ENSMUSG00000027628 | Aar2 | 1.442689 | 0.00002 | up |
| ENSMUSG00000031555 | Adam9 | 1.078288 | 0.00002 | up |
| ENSMUSG00000040111 | Gramd1b | -1.21899 | 0.00002 | down |
| ENSMUSG00000073490 | Ifi207 | -1.40104 | 0.00002 | down |
| ENSMUSG00000102746 | Gm37601 | -2.33154 | 0.00002 | down |
| ENSMUSG00000016833 | Mrps18c | 1.212057 | 0.00002 | up |
| ENSMUSG00000020701 | Tmem132e | -1.24549 | 0.00002 | down |
| ENSMUSG00000020738 | Sumo2 | 1.008226 | 0.00002 | up |
| ENSMUSG00000109168 | Gm44709 | -1.88409 | 0.00002 | down |
| ENSMUSG00000092626 | 9130230N09Rik | -2.19879 | 0.00002 | down |
| ENSMUSG00000023341 | Mx2 | -1.33419 | 0.00002 | down |
| ENSMUSG00000017740 | Slc12a5 | -2.02973 | 0.00002 | down |
| ENSMUSG00000014905 | Dnajb9 | 1.214474 | 0.00002 | up |
| ENSMUSG00000043398 | Gpr135 | -1.94906 | 0.00002 | down |
| ENSMUSG00000023764 | Sfi1 | -1.06137 | 0.00002 | down |
| ENSMUSG00000038342 | Mlxip | -1.37618 | 0.00002 | down |
| ENSMUSG00000034858 | Fam214a | -1.06852 | 0.00002 | down |
| ENSMUSG00000096356 | Olfr889 | -4.2934 | 0.00002 | down |
| ENSMUSG00000114267 | Gm48600 | -2.90105 | 0.00002 | down |
| ENSMUSG00000105095 | 8430422M14Rik | -2.26845 | 0.00002 | down |
| ENSMUSG00000058503 | Fam133b | -1.01396 | 0.00002 | down |
| ENSMUSG00000025429 | Pstpip2 | 2.302414 | 0.00002 | up |
| ENSMUSG00000097639 | Platr4 | -1.76367 | 0.00002 | down |
| ENSMUSG00000038298 | Pdzk1 | 1.025267 | 0.00002 | up |
| ENSMUSG00000087273 | Gm13203 | 3.429489 | 0.00002 | up |
| ENSMUSG00000062906 | Hdac10 | -1.03361 | 0.00002 | down |
| ENSMUSG00000106150 | 4921527H02Rik | -3.67894 | 0.00001 | down |
| ENSMUSG00000029534 | St7 | 1.104573 | 0.00001 | up |
| ENSMUSG00000061898 | Rbak | -1.26149 | 0.00001 | down |
| ENSMUSG00000026675 | Hsd17b7 | 1.308954 | 0.00001 | up |
| ENSMUSG00000047767 | Atg16l2 | -1.44229 | 0.00001 | down |
| ENSMUSG00000058258 | Idi1 | 1.619188 | 0.00001 | up |
| ENSMUSG00000094856 | Gm21962 | -2.24022 | 0.00001 | down |
| ENSMUSG00000035342 | Lzts2 | -1.20746 | 0.00001 | down |
| ENSMUSG00000006362 | Cbfa2t3 | -1.44273 | 0.00001 | down |
| ENSMUSG00000028689 | Ccdc163 | -1.37159 | 0.00001 | down |
| ENSMUSG00000019487 | Trip10 | -1.12702 | 0.00001 | down |
| ENSMUSG00000078789 | Dph1 | 1.68892 | 0.00001 | up |
| ENSMUSG00000026574 | Dpt | -1.31515 | 0.00001 | down |
| ENSMUSG00000087168 | Gm15983 | -3.01295 | 0.00001 | down |
| ENSMUSG00000058603 | Rpl28-ps1 | -1.12323 | 0.00001 | down |
| ENSMUSG00000043110 | Lrrn4 | 4.882904 | 0.00001 | up |
| ENSMUSG00000035686 | Thrsp | 1.469984 | 0.00001 | up |
| ENSMUSG00000007944 | Ttc9b | -5.52467 | 0.00001 | down |
| ENSMUSG00000114247 | Gm32063 | -1.43844 | 0.00001 | down |
| ENSMUSG00000003279 | Dlgap1 | -2.0026 | 0.00001 | down |
| ENSMUSG00000003992 | Ssbp2 | -1.99978 | 0.00001 | down |
| ENSMUSG00000089875 | Etohd2 | -1.30169 | 0.00001 | down |
| ENSMUSG00000025898 | Cwf19l2 | -1.11027 | 0.00001 | down |
| ENSMUSG00000028011 | Tdo2 | 1.067049 | 0.00001 | up |
| ENSMUSG00000026740 | Dnajc1 | -1.11535 | 0.00001 | down |
| ENSMUSG00000056342 | Usp34 | -1.20385 | 0.00001 | down |
| ENSMUSG00000045757 | Zfp764 | -1.30099 | 0.00001 | down |
| ENSMUSG00000109094 | Gm44587 | -4.25197 | 0.00001 | down |
| ENSMUSG00000102700 | Gm38312 | -1.48573 | 0.00001 | down |
| ENSMUSG00000046598 | Bdh1 | 1.107095 | 0.00001 | up |
| ENSMUSG00000031145 | Prickle3 | -1.29653 | 0.00001 | down |
| ENSMUSG00000104348 | Gm37691 | -2.59283 | 0.00001 | down |
| ENSMUSG00000113161 | Gm47457 | -2.01573 | 0.00001 | down |
| ENSMUSG00000115200 | Gm46516 | -2.57257 | 0.00001 | down |
| ENSMUSG00000027394 | Ttl | -1.01164 | 0.00001 | down |
| ENSMUSG00000045503 | Sys1 | 1.051068 | 0.00001 | up |
| ENSMUSG00000048668 | Rhno1 | -1.00701 | 0.00001 | down |
| ENSMUSG00000053158 | Fes | -1.57603 | 0.00001 | down |
| ENSMUSG00000027239 | Mdk | -2.5389 | 0.00001 | down |
| ENSMUSG00000081143 | Gm15823 | -3.83078 | 0.00001 | down |
| ENSMUSG00000056987 | Garin2 | -5.83027 | 0.00001 | down |
| ENSMUSG00000063179 | Pstk | 1.103413 | 0.00001 | up |
| ENSMUSG00000034118 | Tpst1 | 1.030171 | 0.00001 | up |
| ENSMUSG00000060467 | Gm10080 | -5.86354 | 0.00001 | down |
| ENSMUSG00000057614 | Gnai1 | -1.82367 | 0.00001 | down |
| ENSMUSG00000120382 | - | -2.00194 | 0.00001 | down |
| ENSMUSG00000022257 | Laptm4b | 1.323787 | 0.00001 | up |
| ENSMUSG00000026343 | Gpr39 | 1.40891 | 0.00001 | up |
| ENSMUSG00000000568 | Hnrnpd | 1.038881 | 0.00001 | up |
| ENSMUSG00000019791 | Hint3 | 1.00303 | 0.00001 | up |
| ENSMUSG00000036459 | Wtip | -1.34511 | 0.00001 | down |
| ENSMUSG00000019899 | Lama2 | -1.70186 | 0.00001 | down |
| ENSMUSG00000049760 | Micos13 | 1.19511 | 0.00001 | up |
| ENSMUSG00000111847 | Gm8899 | -5.70199 | 0.00001 | down |
| ENSMUSG00000115124 | Gm49201 | -1.11316 | 0.00001 | down |
| ENSMUSG00000049493 | Pls1 | 2.341997 | 0.00001 | up |
| ENSMUSG00000032500 | Dclk3 | 1.30988 | 0.00001 | up |
| ENSMUSG00000006958 | Chrd | -1.11177 | 0.00001 | down |
| ENSMUSG00000084031 | Gm7416 | -4.77316 | 0.00001 | down |
| ENSMUSG00000052417 | Olfr720 | -5.11004 | 0.00001 | down |
| ENSMUSG00000028549 | Itgb3bp | 2.223386 | 0.00001 | up |
| ENSMUSG00000112095 | Sros1 | -1.11615 | 0.00001 | down |
| ENSMUSG00000033502 | Cdc14a | -1.4833 | 0.00001 | down |
| ENSMUSG00000029179 | Zcchc4 | -1.23829 | 0.00001 | down |
| ENSMUSG00000016496 | Cd274 | -1.92665 | 0.00001 | down |
| ENSMUSG00000001666 | Ddt | 1.017942 | 0.00001 | up |
| ENSMUSG00000029269 | Sult1b1 | 1.115728 | 0.00001 | up |
| ENSMUSG00000081221 | Gm14760 | 3.423515 | 0.00001 | up |
| ENSMUSG00000001943 | Vsig2 | -2.69435 | 0.00001 | down |
| ENSMUSG00000110365 | Gm38947 | -2.46694 | 0.00001 | down |
| ENSMUSG00000048174 | Tmem81 | -1.2597 | 0.00001 | down |
| ENSMUSG00000028536 | 2610528J11Rik | 1.805054 | 0.00001 | up |
| ENSMUSG00000036545 | Adamts2 | -1.10407 | 0.00001 | down |
| ENSMUSG00000023723 | Mrps23 | 1.178331 | 0.00001 | up |
| ENSMUSG00000120252 | - | -6.23952 | 0.00001 | down |
| ENSMUSG00000062203 | Gspt1 | 1.003898 | 0.00001 | up |
| ENSMUSG00000108129 | 4930417O13Rik | -4.73558 | 0.00001 | down |
| ENSMUSG00000035047 | Kri1 | -1.11591 | 0.00001 | down |
| ENSMUSG00000047617 | Paxx | -1.32472 | 0.00001 | down |
| ENSMUSG00000118071 | Gm50455 | -2.15296 | 0.00001 | down |
| ENSMUSG00000019883 | Echdc1 | 1.309939 | 0.00001 | up |
| ENSMUSG00000024136 | Dnase1l2 | -2.44733 | 0.00001 | down |
| ENSMUSG00000074865 | Zfp934 | -1.20895 | 0.00001 | down |
| ENSMUSG00000071203 | Naip5 | -1.76146 | 0.00001 | down |
| ENSMUSG00000019951 | Uhrf1bp1l | -1.0609 | 0.00001 | down |
| ENSMUSG00000023882 | Zfp54 | -1.4256 | 0.00001 | down |
| ENSMUSG00000029649 | Pomp | 1.010899 | 0.00001 | up |
| ENSMUSG00000035783 | Acta2 | -1.43899 | 0.00001 | down |
| ENSMUSG00000042496 | Prdm10 | -1.05173 | 0.00001 | down |
| ENSMUSG00000035142 | Nubpl | 2.711413 | 0.00001 | up |
| ENSMUSG00000071711 | Mpst | 1.124477 | 0.00001 | up |
| ENSMUSG00000045193 | Cirbp | 1.071391 | 0.00001 | up |
| ENSMUSG00000107511 | Gm44440 | -2.00085 | 0.00001 | down |
| ENSMUSG00000087382 | Ctcflos | -1.39823 | 0.00001 | down |
| ENSMUSG00000102428 | Pcdhga12 | -2.83257 | 0.00001 | down |
| ENSMUSG00000078485 | Plekhn1 | -2.41331 | 0.00001 | down |
| ENSMUSG00000031641 | Cbr4 | 1.009299 | 0.00001 | up |
| ENSMUSG00000056185 | Snx32 | -1.33931 | 0.00001 | down |
| ENSMUSG00000057143 | Trim12c | -1.1271 | 0.00001 | down |
| ENSMUSG00000113684 | Gm48418 | -2.70616 | 0.00001 | down |
| ENSMUSG00000066724 | Gm10175 | 1.072727 | 0.00001 | up |
| ENSMUSG00000032077 | Bud13 | -1.45853 | 0.00001 | down |
| ENSMUSG00000044534 | Ackr2 | -1.53868 | 0.00001 | down |
| ENSMUSG00000097061 | 9330151L19Rik | -1.9351 | 0.00001 | down |
| ENSMUSG00000115279 | Gm49273 | -1.82417 | 0.00001 | down |
| ENSMUSG00000033191 | Tie1 | -1.05191 | 0.00001 | down |
| ENSMUSG00000119953 | - | -2.10395 | 0.00001 | down |
| ENSMUSG00000074158 | Zfp976 | -1.50259 | 0.00001 | down |
| ENSMUSG00000021242 | Npc2 | 1.301432 | 0.00001 | up |
| ENSMUSG00000017718 | Afmid | 1.055992 | 0.00001 | up |
| ENSMUSG00000027651 | Rprd1b | 1.160737 | 0.00001 | up |
| ENSMUSG00000027762 | Sucnr1 | 1.737191 | 0.00001 | up |
| ENSMUSG00000089978 | Crb1-ps | -4.44426 | 0.00001 | down |
| ENSMUSG00000121374 | - | 1.954451 | 0.00001 | up |
| ENSMUSG00000063188 | Olfr107 | -2.09241 | 0.00001 | down |
| ENSMUSG00000103364 | Gm38157 | -4.03546 | 0.00001 | down |
| ENSMUSG00000003378 | Grik5 | -1.67387 | 0.00001 | down |
| ENSMUSG00000090150 | Acad11 | 1.160002 | 0.00001 | up |
| ENSMUSG00000071103 | 1700029J07Rik | -2.26352 | 0.00001 | down |
| ENSMUSG00000035024 | Ncapd3 | -1.01053 | 0.00001 | down |
| ENSMUSG00000029417 | Cxcl9 | -1.31681 | 0.00001 | down |
| ENSMUSG00000036826 | Igflr1 | -3.77033 | 0.00001 | down |
| ENSMUSG00000060149 | BC002059 | -1.52456 | 0.00001 | down |
| ENSMUSG00000047822 | Angptl8 | -2.3483 | 0.00001 | down |
| ENSMUSG00000028837 | Psmb2 | 1.169067 | 0.00001 | up |
| ENSMUSG00000041220 | Elovl6 | -2.02545 | 0.00001 | down |
| ENSMUSG00000034800 | Zfp661 | -1.24636 | 0.00001 | down |
| ENSMUSG00000070867 | Trabd2b | -1.89223 | 0.00001 | down |
| ENSMUSG00000059040 | Eno1b | -6.37414 | 0.00001 | down |
| ENSMUSG00000021072 | Tmx1 | 1.223387 | 0.00001 | up |
| ENSMUSG00000028445 | Enho | 1.324733 | 0.00001 | up |
| ENSMUSG00000116021 | Gm49474 | -3.96344 | 0.00001 | down |
| ENSMUSG00000012017 | Scarf2 | -1.47657 | 0.00001 | down |
| ENSMUSG00000040701 | Ap1g2 | -1.25871 | 0.00001 | down |
| ENSMUSG00000027230 | Creb3l1 | -1.8258 | 0.00001 | down |
| ENSMUSG00000023333 | Gcm1 | -4.56972 | 0.00001 | down |
| ENSMUSG00000110537 | Gm4316 | -1.28227 | 0.00001 | down |
| ENSMUSG00000112557 | Gm47626 | -1.66977 | 0.00001 | down |
| ENSMUSG00000059022 | Kcp | -1.42368 | 0.00001 | down |
| ENSMUSG00000039478 | Micu3 | -1.49604 | 0.00001 | down |
| ENSMUSG00000030315 | Vgll4 | 1.192086 | 0.00001 | up |
| ENSMUSG00000093942 | Olfr46 | -1.89548 | 0.00001 | down |
| ENSMUSG00000066892 | Fbxl12 | -1.44999 | 0.00001 | down |
| ENSMUSG00000019823 | Mical1 | -1.52809 | 0.00001 | down |
| ENSMUSG00000016181 | Utp25 | -1.04289 | 0.00001 | down |
| ENSMUSG00000005677 | Nr1i3 | -1.34749 | 0.00001 | down |
| ENSMUSG00000031604 | Msmo1 | 1.648961 | 0.00001 | up |
| ENSMUSG00000040563 | Plppr2 | -1.87962 | 0.00001 | down |
| ENSMUSG00000111938 | 2900045O20Rik | -4.74592 | 0.00001 | down |
| ENSMUSG00000038372 | Gmds | 2.90308 | 0.00001 | up |
| ENSMUSG00000035245 | Eogt | -1.02804 | 0.00001 | down |
| ENSMUSG00000079487 | Med12 | -1.02481 | 0.00001 | down |
| ENSMUSG00000105065 | Gm42513 | -1.57121 | 0.00001 | down |
| ENSMUSG00000112433 | Gm30122 | -2.43013 | 0.00001 | down |
| ENSMUSG00000038344 | Txlng | -1.03364 | 0.00001 | down |
| ENSMUSG00000090674 | Gm17082 | -3.15678 | 0.00001 | down |
| ENSMUSG00000033880 | Lgals3bp | 1.105655 | 0.00001 | up |
| ENSMUSG00000032264 | Zw10 | -1.20848 | 0.00001 | down |
| ENSMUSG00000028907 | Utp11 | -1.02803 | 0.00001 | down |
| ENSMUSG00000026390 | Marco | 1.603874 | 0.00001 | up |
| ENSMUSG00000073468 | Sft2d1 | 1.120227 | 0.00001 | up |
| ENSMUSG00000027505 | Fam209 | -5.78543 | 0.00001 | down |
| ENSMUSG00000031825 | Crispld2 | -1.82696 | 0.00001 | down |
| ENSMUSG00000028007 | Snx7 | 1.381307 | 0.00001 | up |
| ENSMUSG00000035051 | Dhx57 | -1.19858 | 0.00001 | down |
| ENSMUSG00000037720 | Tmem33 | 1.050334 | 0.00001 | up |
| ENSMUSG00000032078 | Zpr1 | -1.26386 | 0.00001 | down |
| ENSMUSG00000052962 | Mrpl35 | 1.008165 | 0.00001 | up |
| ENSMUSG00000059602 | Syn3 | -1.59022 | 0.00001 | down |
| ENSMUSG00000026956 | Uap1l1 | 1.500296 | 0.00001 | up |
| ENSMUSG00000085990 | Gm16731 | -2.39426 | 0.00001 | down |
| ENSMUSG00000114458 | Gm47551 | -3.21759 | 0.00001 | down |
| ENSMUSG00000029765 | Plxna4 | -1.61431 | 0.00001 | down |
| ENSMUSG00000021792 | Prxl2a | 1.494346 | 0.00001 | up |
| ENSMUSG00000025002 | Cyp2c55 | 2.090079 | 0.00001 | up |
| ENSMUSG00000056035 | Cyp3a11 | 1.213842 | 0.00001 | up |
| ENSMUSG00000051977 | Prdm9 | -1.21263 | 0.00001 | down |
| ENSMUSG00000015748 | Prpf3 | -1.03023 | 0.00001 | down |
| ENSMUSG00000041660 | Bbox1 | 1.04845 | 0.00001 | up |
| ENSMUSG00000044475 | Ascc1 | 1.006728 | <0.00001 | up |
| ENSMUSG00000093445 | Lrch4 | 2.00175 | <0.00001 | up |
| ENSMUSG00000048755 | Mcat | 1.351629 | <0.00001 | up |
| ENSMUSG00000117084 | Gm22146 | -2.10675 | <0.00001 | down |
| ENSMUSG00000026113 | Inpp4a | -1.09207 | <0.00001 | down |
| ENSMUSG00000051652 | Lrrc3 | 1.30704 | <0.00001 | up |
| ENSMUSG00000108884 | Gm45792 | -2.22509 | <0.00001 | down |
| ENSMUSG00000034591 | Slc41a2 | 2.588552 | <0.00001 | up |
| ENSMUSG00000028370 | Pappa | -4.33724 | <0.00001 | down |
| ENSMUSG00000004460 | Dnajb11 | 1.061169 | <0.00001 | up |
| ENSMUSG00000028080 | Lrba | 1.032492 | <0.00001 | up |
| ENSMUSG00000100967 | Gm29666 | -2.24186 | <0.00001 | down |
| ENSMUSG00000071662 | Polr2g | 1.252587 | <0.00001 | up |
| ENSMUSG00000121505 | Gm4956 | -1.45695 | <0.00001 | down |
| ENSMUSG00000025159 | Mms19 | -1.10523 | <0.00001 | down |
| ENSMUSG00000002668 | Dennd1c | -1.99321 | <0.00001 | down |
| ENSMUSG00000040724 | Kcna2 | -1.58146 | <0.00001 | down |
| ENSMUSG00000020776 | Fbf1 | -1.5014 | <0.00001 | down |
| ENSMUSG00000029632 | Ndufa4 | 1.352766 | <0.00001 | up |
| ENSMUSG00000038146 | Notch3 | -1.83267 | <0.00001 | down |
| ENSMUSG00000022679 | Mpv17l | 1.201473 | <0.00001 | up |
| ENSMUSG00000025027 | Xpnpep1 | 1.142575 | <0.00001 | up |
| ENSMUSG00000074733 | Zfp950 | -1.27915 | <0.00001 | down |
| ENSMUSG00000028657 | Ppt1 | 1.087781 | <0.00001 | up |
| ENSMUSG00000087516 | Tbx3os1 | -1.88473 | <0.00001 | down |
| ENSMUSG00000059714 | Flot1 | 1.192538 | <0.00001 | up |
| ENSMUSG00000019843 | Fyn | -1.10971 | <0.00001 | down |
| ENSMUSG00000035476 | Tab3 | -1.28229 | <0.00001 | down |
| ENSMUSG00000076437 | Selenoh | 1.525226 | <0.00001 | up |
| ENSMUSG00000023965 | Fbxl17 | 1.348773 | <0.00001 | up |
| ENSMUSG00000000278 | Scpep1 | 1.026313 | <0.00001 | up |
| ENSMUSG00000097772 | 5430416N02Rik | -1.13064 | <0.00001 | down |
| ENSMUSG00000022972 | Cfap298 | 1.037153 | <0.00001 | up |
| ENSMUSG00000108780 | 5430434F05Rik | -4.25726 | <0.00001 | down |
| ENSMUSG00000028134 | Ptbp2 | -1.19944 | <0.00001 | down |
| ENSMUSG00000023075 | Akirin1 | 1.203113 | <0.00001 | up |
| ENSMUSG00000022185 | Acin1 | -1.02634 | <0.00001 | down |
| ENSMUSG00000115497 | Gm49207 | -3.79874 | <0.00001 | down |
| ENSMUSG00000040940 | Arhgef1 | -1.09203 | <0.00001 | down |
| ENSMUSG00000015647 | Lama5 | -1.38594 | <0.00001 | down |
| ENSMUSG00000047866 | Lonp2 | 1.192327 | <0.00001 | up |
| ENSMUSG00000041468 | Gpr12 | -2.35545 | <0.00001 | down |
| ENSMUSG00000062198 | 2700097O09Rik | -1.30248 | <0.00001 | down |
| ENSMUSG00000039887 | Alg14 | 1.092426 | <0.00001 | up |
| ENSMUSG00000034349 | Smc4 | -1.09643 | <0.00001 | down |
| ENSMUSG00000031425 | Plp1 | -2.11082 | <0.00001 | down |
| ENSMUSG00000069844 | Sco1 | 1.092372 | <0.00001 | up |
| ENSMUSG00000054381 | Zfp747 | -1.16763 | <0.00001 | down |
| ENSMUSG00000106411 | Gm42445 | -1.32977 | <0.00001 | down |
| ENSMUSG00000115919 | Gm31583 | -1.1598 | <0.00001 | down |
| ENSMUSG00000069729 | Arid1b | -1.193 | <0.00001 | down |
| ENSMUSG00000078674 | Mup18 | 2.421448 | <0.00001 | up |
| ENSMUSG00000029597 | Sds | 1.287718 | <0.00001 | up |
| ENSMUSG00000036948 | Trappc14 | -1.13107 | <0.00001 | down |
| ENSMUSG00000054199 | Gon4l | -1.00513 | <0.00001 | down |
| ENSMUSG00000057580 | Cox7c-ps1 | -3.54098 | <0.00001 | down |
| ENSMUSG00000001095 | Slc13a2 | -2.43011 | <0.00001 | down |
| ENSMUSG00000033909 | Usp36 | -1.08342 | <0.00001 | down |
| ENSMUSG00000019756 | Prl8a1 | -2.36071 | <0.00001 | down |
| ENSMUSG00000040139 | 9430038I01Rik | -1.36174 | <0.00001 | down |
| ENSMUSG00000058934 | Igf1os | -2.52741 | <0.00001 | down |
| ENSMUSG00000085008 | Dbhos | -2.54612 | <0.00001 | down |
| ENSMUSG00000111878 | Gm47777 | -5.25123 | <0.00001 | down |
| ENSMUSG00000027984 | Hadh | 1.008378 | <0.00001 | up |
| ENSMUSG00000030717 | Nupr1 | -2.01484 | <0.00001 | down |
| ENSMUSG00000030225 | Dera | 1.011879 | <0.00001 | up |
| ENSMUSG00000001119 | Col6a1 | -1.09038 | <0.00001 | down |
| ENSMUSG00000078941 | Ak6 | 1.157914 | <0.00001 | up |
| ENSMUSG00000002778 | Kdelr1 | 1.073974 | <0.00001 | up |
| ENSMUSG00000086253 | Gm13773 | -1.38871 | <0.00001 | down |
| ENSMUSG00000028703 | Lrrc41 | 1.211175 | <0.00001 | up |
| ENSMUSG00000028618 | Tmem59 | 1.171709 | <0.00001 | up |
| ENSMUSG00000026737 | Pip4k2a | -1.07149 | <0.00001 | down |
| ENSMUSG00000054716 | Zfp771 | 2.21179 | <0.00001 | up |
| ENSMUSG00000002814 | Top3a | -1.09663 | <0.00001 | down |
| ENSMUSG00000045538 | Ddx28 | 1.426663 | <0.00001 | up |
| ENSMUSG00000026127 | Imp4 | -1.03235 | <0.00001 | down |
| ENSMUSG00000011158 | Brf1 | -1.3063 | <0.00001 | down |
| ENSMUSG00000073973 | Olfr552 | -1.56151 | <0.00001 | down |
| ENSMUSG00000023980 | Taf8 | -1.20988 | <0.00001 | down |
| ENSMUSG00000033790 | Tubgcp5 | -1.28518 | <0.00001 | down |
| ENSMUSG00000018001 | Cyth3 | -1.16147 | <0.00001 | down |
| ENSMUSG00000037552 | Plekhg2 | -1.32055 | <0.00001 | down |
| ENSMUSG00000100257 | C4bp-ps1 | -4.1497 | <0.00001 | down |
| ENSMUSG00000027684 | Mecom | -2.30096 | <0.00001 | down |
| ENSMUSG00000094708 | Gm10359 | 1.398018 | <0.00001 | up |
| ENSMUSG00000038811 | Gngt2 | -1.31114 | <0.00001 | down |
| ENSMUSG00000049404 | Rarres1 | 1.124841 | <0.00001 | up |
| ENSMUSG00000000317 | Bcl6b | -2.01954 | <0.00001 | down |
| ENSMUSG00000038949 | Cnst | -1.05513 | <0.00001 | down |
| ENSMUSG00000039943 | Plcb4 | -1.69772 | <0.00001 | down |
| ENSMUSG00000026879 | Gsn | -1.01817 | <0.00001 | down |
| ENSMUSG00000042197 | Zfp451 | -1.12386 | <0.00001 | down |
| ENSMUSG00000037894 | H2az1 | 1.220184 | <0.00001 | up |
| ENSMUSG00000045624 | Esf1 | -1.10278 | <0.00001 | down |
| ENSMUSG00000022337 | Emc2 | 1.230672 | <0.00001 | up |
| ENSMUSG00000054843 | Atrnl1 | 1.198989 | <0.00001 | up |
| ENSMUSG00000110384 | Gm45301 | -5.38381 | <0.00001 | down |
| ENSMUSG00000022837 | Iqcb1 | -1.23922 | <0.00001 | down |
| ENSMUSG00000034758 | Tle6 | -1.88425 | <0.00001 | down |
| ENSMUSG00000090799 | Klhl33 | -3.44263 | <0.00001 | down |
| ENSMUSG00000025580 | Eif4a3 | 1.012505 | <0.00001 | up |
| ENSMUSG00000118043 | Gm50206 | -3.41733 | <0.00001 | down |
| ENSMUSG00000031842 | Pde4c | -1.41012 | <0.00001 | down |
| ENSMUSG00000101059 | Gm4017 | -3.28441 | <0.00001 | down |
| ENSMUSG00000113601 | Gm48735 | -4.09257 | <0.00001 | down |
| ENSMUSG00000095123 | Gm21781 | -1.39469 | <0.00001 | down |
| ENSMUSG00000085337 | Gm15964 | -2.83518 | <0.00001 | down |
| ENSMUSG00000110206 | Flt3l | -1.2086 | <0.00001 | down |
| ENSMUSG00000105315 | Gm18635 | -1.79317 | <0.00001 | down |
| ENSMUSG00000031242 | 2610002M06Rik | 1.159323 | <0.00001 | up |
| ENSMUSG00000056209 | Npm3 | 1.170456 | <0.00001 | up |
| ENSMUSG00000021951 | Eef1akmt1 | 2.469679 | <0.00001 | up |
| ENSMUSG00000103285 | Gm37274 | -4.74202 | <0.00001 | down |
| ENSMUSG00000026918 | Brd3 | -1.00207 | <0.00001 | down |
| ENSMUSG00000081207 | Gm13775 | -1.53145 | <0.00001 | down |
| ENSMUSG00000026389 | Steap3 | 1.075327 | <0.00001 | up |
| ENSMUSG00000030800 | Prss8 | 1.642139 | <0.00001 | up |
| ENSMUSG00000019849 | Prep | 1.383066 | <0.00001 | up |
| ENSMUSG00000037754 | Ppp1r16b | -1.26991 | <0.00001 | down |
| ENSMUSG00000023170 | Gps2 | -1.01593 | <0.00001 | down |
| ENSMUSG00000097673 | Gm26608 | -1.51586 | <0.00001 | down |
| ENSMUSG00000037916 | Ndufv1 | 1.015314 | <0.00001 | up |
| ENSMUSG00000095325 | Zfp870 | -1.34249 | <0.00001 | down |
| ENSMUSG00000020171 | Yeats4 | 1.30541 | <0.00001 | up |
| ENSMUSG00000023495 | Pcbp4 | -1.41386 | <0.00001 | down |
| ENSMUSG00000028268 | Gbp3 | -1.64468 | <0.00001 | down |
| ENSMUSG00000015850 | Adamtsl4 | -1.29578 | <0.00001 | down |
| ENSMUSG00000035199 | Arl6ip5 | 1.075333 | <0.00001 | up |
| ENSMUSG00000046352 | Gjb2 | 1.167147 | <0.00001 | up |
| ENSMUSG00000025902 | Sox17 | -1.85481 | <0.00001 | down |
| ENSMUSG00000019872 | Smpdl3a | 1.448644 | <0.00001 | up |
| ENSMUSG00000032459 | Mrps22 | 1.208205 | <0.00001 | up |
| ENSMUSG00000097615 | Gm2061 | -2.40798 | <0.00001 | down |
| ENSMUSG00000025509 | Pnpla2 | 1.022504 | <0.00001 | up |
| ENSMUSG00000068856 | Sf3b4 | 1.265701 | <0.00001 | up |
| ENSMUSG00000002910 | Arrdc2 | -1.25894 | <0.00001 | down |
| ENSMUSG00000086859 | Snhg20 | -1.9569 | <0.00001 | down |
| ENSMUSG00000118815 | Gm22068 | -5.42787 | <0.00001 | down |
| ENSMUSG00000033808 | Tmem87a | -1.17809 | <0.00001 | down |
| ENSMUSG00000025486 | Sirt3 | -2.11725 | <0.00001 | down |
| ENSMUSG00000116898 | Gm49785 | -2.34124 | <0.00001 | down |
| ENSMUSG00000008496 | Pou2f2 | -1.91818 | <0.00001 | down |
| ENSMUSG00000026925 | Inpp5e | -1.42043 | <0.00001 | down |
| ENSMUSG00000091119 | Ccdc152 | -4.16894 | <0.00001 | down |
| ENSMUSG00000105160 | A530030E21Rik | -3.78609 | <0.00001 | down |
| ENSMUSG00000035112 | Wnk4 | -2.21806 | <0.00001 | down |
| ENSMUSG00000076609 | Igkc | -1.40259 | <0.00001 | down |
| ENSMUSG00000041268 | Dmxl2 | -1.1084 | <0.00001 | down |
| ENSMUSG00000098488 | Pla2g4b | -2.26348 | <0.00001 | down |
| ENSMUSG00000071856 | Mcc | -1.00337 | <0.00001 | down |
| ENSMUSG00000025545 | Clybl | 1.092325 | <0.00001 | up |
| ENSMUSG00000027615 | Hps3 | -1.01134 | <0.00001 | down |
| ENSMUSG00000022556 | Hsf1 | -1.15303 | <0.00001 | down |
| ENSMUSG00000021240 | Abcd4 | -1.20759 | <0.00001 | down |
| ENSMUSG00000037851 | Iars | 1.725484 | <0.00001 | up |
| ENSMUSG00000040017 | Saa4 | 1.27655 | <0.00001 | up |
| ENSMUSG00000001783 | Rtcb | 1.237333 | <0.00001 | up |
| ENSMUSG00000022822 | Abcc5 | -1.19695 | <0.00001 | down |
| ENSMUSG00000031506 | Ptpn7 | -1.96665 | <0.00001 | down |
| ENSMUSG00000026142 | Rhbdd1 | 1.33424 | <0.00001 | up |
| ENSMUSG00000023918 | Adgrf4 | -6.47164 | <0.00001 | down |
| ENSMUSG00000024137 | E4f1 | -1.18974 | <0.00001 | down |
| ENSMUSG00000048406 | B330016D10Rik | -2.11978 | <0.00001 | down |
| ENSMUSG00000040269 | Mrps28 | 1.05003 | <0.00001 | up |
| ENSMUSG00000113831 | Gm49602 | -1.74211 | <0.00001 | down |
| ENSMUSG00000003352 | Cacnb3 | -1.83646 | <0.00001 | down |
| ENSMUSG00000033170 | Card10 | -1.11083 | <0.00001 | down |
| ENSMUSG00000068732 | Tmem167b | 1.290595 | <0.00001 | up |
| ENSMUSG00000101397 | Mug-ps1 | -1.41933 | <0.00001 | down |
| ENSMUSG00000031266 | Gla | 1.512467 | <0.00001 | up |
| ENSMUSG00000027207 | Galk2 | 1.216495 | <0.00001 | up |
| ENSMUSG00000071074 | Yipf3 | 1.133897 | <0.00001 | up |
| ENSMUSG00000110234 | Gm45799 | 2.11933 | <0.00001 | up |
| ENSMUSG00000039968 | Rsbn1l | -1.0888 | <0.00001 | down |
| ENSMUSG00000019796 | Lrp11 | 4.327838 | <0.00001 | up |
| ENSMUSG00000020448 | Rnf185 | 1.009064 | <0.00001 | up |
| ENSMUSG00000074024 | 4632427E13Rik | -2.44047 | <0.00001 | down |
| ENSMUSG00000021577 | Sdha | 1.192232 | <0.00001 | up |
| ENSMUSG00000086390 | 1810019D21Rik | -1.34996 | <0.00001 | down |
| ENSMUSG00000100005 | B130024G19Rik | -1.77011 | <0.00001 | down |
| ENSMUSG00000055900 | Tmem69 | 1.06854 | <0.00001 | up |
| ENSMUSG00000028059 | Arhgef2 | -1.22473 | <0.00001 | down |
| ENSMUSG00000020451 | Limk2 | -1.12007 | <0.00001 | down |
| ENSMUSG00000039952 | Dag1 | 1.419205 | <0.00001 | up |
| ENSMUSG00000036721 | Zscan12 | -1.22805 | <0.00001 | down |
| ENSMUSG00000035910 | Dcdc2a | -1.42206 | <0.00001 | down |
| ENSMUSG00000049339 | Retreg2 | 1.019591 | <0.00001 | up |
| ENSMUSG00000107117 | Gm43842 | -3.67053 | <0.00001 | down |
| ENSMUSG00000026811 | St6galnac6 | 1.246806 | <0.00001 | up |
| ENSMUSG00000022010 | Tsc22d1 | -1.22344 | <0.00001 | down |
| ENSMUSG00000097124 | A530020G20Rik | -2.10206 | <0.00001 | down |
| ENSMUSG00000035067 | Xkr6 | -2.25318 | <0.00001 | down |
| ENSMUSG00000029245 | Epha5 | -4.44306 | <0.00001 | down |
| ENSMUSG00000107191 | Gm43579 | -3.08594 | <0.00001 | down |
| ENSMUSG00000029462 | Vps29 | 1.030368 | <0.00001 | up |
| ENSMUSG00000024613 | Tcof1 | -1.24963 | <0.00001 | down |
| ENSMUSG00000004394 | Tmed4 | 1.097055 | <0.00001 | up |
| ENSMUSG00000031903 | Pla2g15 | 1.011734 | <0.00001 | up |
| ENSMUSG00000029563 | Foxp2 | -1.81338 | <0.00001 | down |
| ENSMUSG00000106107 | Gm43190 | -1.84972 | <0.00001 | down |
| ENSMUSG00000032875 | Arhgef17 | -1.42662 | <0.00001 | down |
| ENSMUSG00000121029 | - | -3.47132 | <0.00001 | down |
| ENSMUSG00000042670 | Immp1l | 1.122366 | <0.00001 | up |
| ENSMUSG00000084960 | B430010I23Rik | -3.80372 | <0.00001 | down |
| ENSMUSG00000059540 | Tcea2 | -2.23693 | <0.00001 | down |
| ENSMUSG00000035242 | Oaz1 | 1.05073 | <0.00001 | up |
| ENSMUSG00000031174 | Rpgr | -1.7151 | <0.00001 | down |
| ENSMUSG00000062761 | Zfp512 | -1.21971 | <0.00001 | down |
| ENSMUSG00000078964 | Ces1b | -1.1872 | <0.00001 | down |
| ENSMUSG00000081604 | Gm11518 | -1.14712 | <0.00001 | down |
| ENSMUSG00000044595 | Dnd1 | -2.25405 | <0.00001 | down |
| ENSMUSG00000117123 | Gm49890 | -2.33638 | <0.00001 | down |
| ENSMUSG00000022220 | Adcy4 | -1.31399 | <0.00001 | down |
| ENSMUSG00000022205 | Sub1 | 1.206001 | <0.00001 | up |
| ENSMUSG00000092075 | Serpina4-ps1 | -2.75784 | <0.00001 | down |
| ENSMUSG00000025353 | Ormdl2 | 1.201848 | <0.00001 | up |
| ENSMUSG00000085105 | Gm12758 | -1.18923 | <0.00001 | down |
| ENSMUSG00000115027 | Vmn1r81 | -1.94511 | <0.00001 | down |
| ENSMUSG00000026192 | Atic | 2.107681 | <0.00001 | up |
| ENSMUSG00000116378 | Gcat | 8.722171 | <0.00001 | up |
| ENSMUSG00000024049 | Myom1 | -2.78578 | <0.00001 | down |
| ENSMUSG00000024002 | Brd4 | -1.07974 | <0.00001 | down |
| ENSMUSG00000116735 | Gm49555 | -3.94627 | <0.00001 | down |
| ENSMUSG00000062683 | Atp5g2 | 2.828816 | <0.00001 | up |
| ENSMUSG00000005628 | Tmod4 | -4.60539 | <0.00001 | down |
| ENSMUSG00000104548 | Gm43857 | -2.24387 | <0.00001 | down |
| ENSMUSG00000055818 | A230083G16Rik | -2.30518 | <0.00001 | down |
| ENSMUSG00000060216 | Arrb2 | -1.26431 | <0.00001 | down |
| ENSMUSG00000028467 | Gba2 | -1.05961 | <0.00001 | down |
| ENSMUSG00000032002 | Dcun1d5 | 1.170209 | <0.00001 | up |
| ENSMUSG00000047180 | Neurl3 | -1.2019 | <0.00001 | down |
| ENSMUSG00000108162 | Gm20589 | -5.23404 | <0.00001 | down |
| ENSMUSG00000060450 | Rnf14 | 1.172421 | <0.00001 | up |
| ENSMUSG00000102858 | Gm37086 | -1.56234 | <0.00001 | down |
| ENSMUSG00000036057 | Ptpn23 | -1.16848 | <0.00001 | down |
| ENSMUSG00000029022 | Miip | -1.30697 | <0.00001 | down |
| ENSMUSG00000032076 | Cadm1 | -1.41918 | <0.00001 | down |
| ENSMUSG00000061028 | Clasrp | -1.44969 | <0.00001 | down |
| ENSMUSG00000030177 | Ccdc77 | -1.40101 | <0.00001 | down |
| ENSMUSG00000058173 | Smco4 | 1.387056 | <0.00001 | up |
| ENSMUSG00000091586 | Cyp4f17 | -1.05528 | <0.00001 | down |
| ENSMUSG00000040350 | Trim7 | -1.63212 | <0.00001 | down |
| ENSMUSG00000068457 | Uty | -1.02385 | <0.00001 | down |
| ENSMUSG00000118631 | Gm53019 | -1.27288 | <0.00001 | down |
| ENSMUSG00000045327 | 6330549D23Rik | -3.34694 | <0.00001 | down |
| ENSMUSG00000043872 | Zmym1 | -1.90858 | <0.00001 | down |
| ENSMUSG00000031613 | Hpgd | 1.082247 | <0.00001 | up |
| ENSMUSG00000059316 | Slc27a4 | 1.034316 | <0.00001 | up |
| ENSMUSG00000035184 | Fam124a | -1.62972 | <0.00001 | down |
| ENSMUSG00000036840 | Siah1a | -1.26106 | <0.00001 | down |
| ENSMUSG00000048126 | Col6a3 | -1.39326 | <0.00001 | down |
| ENSMUSG00000005148 | Klf5 | -2.55376 | <0.00001 | down |
| ENSMUSG00000062963 | Ufc1 | 1.078755 | <0.00001 | up |
| ENSMUSG00000036864 | Proser3 | -1.92677 | <0.00001 | down |
| ENSMUSG00000034880 | Mrpl34 | 1.548116 | <0.00001 | up |
| ENSMUSG00000024483 | Ankhd1 | -1.50837 | <0.00001 | down |
| ENSMUSG00000029675 | Eln | -1.52183 | <0.00001 | down |
| ENSMUSG00000110949 | Nudt8 | 1.225868 | <0.00001 | up |
| ENSMUSG00000070417 | Olfr2 | -3.80385 | <0.00001 | down |
| ENSMUSG00000042625 | Safb2 | -1.48867 | <0.00001 | down |
| ENSMUSG00000066196 | Spag8 | -3.4496 | <0.00001 | down |
| ENSMUSG00000100017 | 2410022M11Rik | 2.021777 | <0.00001 | up |
| ENSMUSG00000114605 | C130051F05Rik | -2.66613 | <0.00001 | down |
| ENSMUSG00000061887 | Ssbp3 | -1.21953 | <0.00001 | down |
| ENSMUSG00000036594 | H2-Aa | -1.10765 | <0.00001 | down |
| ENSMUSG00000031591 | Asah1 | 1.326003 | <0.00001 | up |
| ENSMUSG00000004951 | Hspb1 | -1.77673 | <0.00001 | down |
| ENSMUSG00000023832 | Acat2 | 1.367658 | <0.00001 | up |
| ENSMUSG00000117990 | Gm32027 | -2.29154 | <0.00001 | down |
| ENSMUSG00000111917 | Gm48793 | -3.58663 | <0.00001 | down |
| ENSMUSG00000021916 | Glt8d1 | -1.30316 | <0.00001 | down |
| ENSMUSG00000045092 | S1pr1 | 1.407448 | <0.00001 | up |
| ENSMUSG00002076161 | Rn7sk | -2.121 | <0.00001 | down |
| ENSMUSG00000029661 | Col1a2 | -1.04689 | <0.00001 | down |
| ENSMUSG00000063558 | Aox1 | -1.019 | <0.00001 | down |
| ENSMUSG00000042271 | Nxt2 | 1.115755 | <0.00001 | up |
| ENSMUSG00000060470 | Adgrg3 | -1.32192 | <0.00001 | down |
| ENSMUSG00000057068 | Fam47e | 1.790446 | <0.00001 | up |
| ENSMUSG00000033107 | Rnf125 | -1.64174 | <0.00001 | down |
| ENSMUSG00000025510 | Cd151 | 1.303856 | <0.00001 | up |
| ENSMUSG00000005615 | Pcyt1a | 1.203725 | <0.00001 | up |
| ENSMUSG00000081471 | Gm14735 | -2.10304 | <0.00001 | down |
| ENSMUSG00000018171 | Vmp1 | -1.09242 | <0.00001 | down |
| ENSMUSG00000084790 | Gm15879 | -3.45038 | <0.00001 | down |
| ENSMUSG00000028672 | Hmgcl | 1.285495 | <0.00001 | up |
| ENSMUSG00000039253 | Fn3krp | 1.149046 | <0.00001 | up |
| ENSMUSG00000107771 | Gm8956 | -3.88186 | <0.00001 | down |
| ENSMUSG00000025408 | Ddit3 | -1.70445 | <0.00001 | down |
| ENSMUSG00000026623 | Lpgat1 | 1.054795 | <0.00001 | up |
| ENSMUSG00000052974 | Cyp2f2 | 1.333688 | <0.00001 | up |
| ENSMUSG00000001632 | Brpf1 | -1.28001 | <0.00001 | down |
| ENSMUSG00000078427 | Sarnp | 1.638894 | <0.00001 | up |
| ENSMUSG00000024863 | Mbl2 | 1.232988 | <0.00001 | up |
| ENSMUSG00000004098 | Col5a3 | -1.8529 | <0.00001 | down |
| ENSMUSG00000025991 | Cps1 | 1.228395 | <0.00001 | up |
| ENSMUSG00000055322 | Tns1 | -1.06301 | <0.00001 | down |
| ENSMUSG00000009894 | Snap47 | 1.01498 | <0.00001 | up |
| ENSMUSG00000021116 | Eif2s1 | 1.010773 | <0.00001 | up |
| ENSMUSG00000044951 | Mylk4 | -4.16719 | <0.00001 | down |
| ENSMUSG00000094870 | Zfp131 | -1.17134 | <0.00001 | down |
| ENSMUSG00000111045 | Gm47598 | -3.35847 | <0.00001 | down |
| ENSMUSG00000023007 | Prpf40b | -1.41432 | <0.00001 | down |
| ENSMUSG00000028757 | Ddost | 1.039685 | <0.00001 | up |
| ENSMUSG00000049929 | Lpar4 | -3.19673 | <0.00001 | down |
| ENSMUSG00000023885 | Thbs2 | -2.05641 | <0.00001 | down |
| ENSMUSG00000063849 | Ppcdc | -1.1759 | <0.00001 | down |
| ENSMUSG00000045854 | Lyrm2 | 1.320554 | <0.00001 | up |
| ENSMUSG00000024502 | Jakmip2 | -1.63425 | <0.00001 | down |
| ENSMUSG00000035413 | Tmem98 | 1.253976 | <0.00001 | up |
| ENSMUSG00000018405 | Mrm1 | -1.50653 | <0.00001 | down |
| ENSMUSG00000027774 | Gfm1 | 1.193287 | <0.00001 | up |
| ENSMUSG00000019935 | Slc17a8 | -2.03363 | <0.00001 | down |
| ENSMUSG00000113050 | Gm8016 | -1.84432 | <0.00001 | down |
| ENSMUSG00000018339 | Gpx3 | -1.34078 | <0.00001 | down |
| ENSMUSG00000034435 | Tmem30b | 1.200404 | <0.00001 | up |
| ENSMUSG00000036504 | Phpt1 | 1.541332 | <0.00001 | up |
| ENSMUSG00000113722 | Snhg10 | -2.99122 | <0.00001 | down |
| ENSMUSG00000086544 | Chn1os3 | -3.12404 | <0.00001 | down |
| ENSMUSG00000030281 | Il17rc | 1.311943 | <0.00001 | up |
| ENSMUSG00000112824 | Gm47917 | -3.20855 | <0.00001 | down |
| ENSMUSG00000003812 | Dnase2a | -1.19156 | <0.00001 | down |
| ENSMUSG00000118382 | Gm8373 | -3.59712 | <0.00001 | down |
| ENSMUSG00000049940 | Pgrmc2 | 1.115102 | <0.00001 | up |
| ENSMUSG00000022125 | Cln5 | 1.237644 | <0.00001 | up |
| ENSMUSG00000021701 | Plk2 | -1.40566 | <0.00001 | down |
| ENSMUSG00000114114 | Gm48499 | -3.45761 | <0.00001 | down |
| ENSMUSG00000041044 | Lrit1 | -1.30046 | <0.00001 | down |
| ENSMUSG00000060613 | Cyp2c70 | 1.511213 | <0.00001 | up |
| ENSMUSG00000031229 | Atrx | -1.12512 | <0.00001 | down |
| ENSMUSG00000024987 | Cyp26a1 | 1.041682 | <0.00001 | up |
| ENSMUSG00000003810 | Mast2 | -1.13982 | <0.00001 | down |
| ENSMUSG00000027165 | Iftap | 1.275731 | <0.00001 | up |
| ENSMUSG00000025731 | Mettl26 | 1.090246 | <0.00001 | up |
| ENSMUSG00000022505 | Emp2 | -1.73799 | <0.00001 | down |
| ENSMUSG00000040738 | Ints8 | -1.08578 | <0.00001 | down |
| ENSMUSG00000023087 | Noct | -1.49283 | <0.00001 | down |
| ENSMUSG00000027329 | Spef1 | -1.98694 | <0.00001 | down |
| ENSMUSG00000095959 | Gm10845 | -2.30419 | <0.00001 | down |
| ENSMUSG00000035469 | Rcbtb1 | 1.024603 | <0.00001 | up |
| ENSMUSG00000046111 | Cep295 | -1.41054 | <0.00001 | down |
| ENSMUSG00000027404 | Snrpb | 1.747795 | <0.00001 | up |
| ENSMUSG00000106547 | B230303O12Rik | -4.0124 | <0.00001 | down |
| ENSMUSG00000025911 | Adhfe1 | 1.080121 | <0.00001 | up |
| ENSMUSG00000034254 | Agpat1 | -1.22142 | <0.00001 | down |
| ENSMUSG00000020289 | Nprl3 | -1.13421 | <0.00001 | down |
| ENSMUSG00000045594 | Glb1 | 1.139093 | <0.00001 | up |
| ENSMUSG00000109038 | Gm45120 | -4.90645 | <0.00001 | down |
| ENSMUSG00000032690 | Oas2 | -1.83742 | <0.00001 | down |
| ENSMUSG00000028108 | Ecm1 | -1.05728 | <0.00001 | down |
| ENSMUSG00000001467 | Cyp51 | 1.702654 | <0.00001 | up |
| ENSMUSG00000121092 | - | -1.23806 | <0.00001 | down |
| ENSMUSG00000020108 | Ddit4 | 1.594255 | <0.00001 | up |
| ENSMUSG00000119972 | - | -2.00187 | <0.00001 | down |
| ENSMUSG00000046338 | Gpat2 | -1.84445 | <0.00001 | down |
| ENSMUSG00000019987 | Arg1 | 1.130774 | <0.00001 | up |
| ENSMUSG00000032271 | Nnmt | 1.476421 | <0.00001 | up |
| ENSMUSG00000093467 | Gm20659 | -1.53705 | <0.00001 | down |
| ENSMUSG00000035868 | Zfp983 | -1.14384 | <0.00001 | down |
| ENSMUSG00000067931 | Zfp948 | -1.57939 | <0.00001 | down |
| ENSMUSG00000020546 | Stxbp4 | -1.9196 | <0.00001 | down |
| ENSMUSG00000024462 | Gabbr1 | -1.63657 | <0.00001 | down |
| ENSMUSG00000062093 | Gm10110 | -2.58977 | <0.00001 | down |
| ENSMUSG00000018537 | Pcgf2 | -1.51166 | <0.00001 | down |
| ENSMUSG00000030929 | Eri2 | -1.51918 | <0.00001 | down |
| ENSMUSG00000000686 | Abhd15 | 1.59171 | <0.00001 | up |
| ENSMUSG00000021764 | Ndufs4 | 1.133336 | <0.00001 | up |
| ENSMUSG00000026766 | Mmadhc | 1.096941 | <0.00001 | up |
| ENSMUSG00000027824 | Vmn2r1 | -2.15781 | <0.00001 | down |
| ENSMUSG00000035206 | Sppl2b | -1.23293 | <0.00001 | down |
| ENSMUSG00000046721 | Rpl14-ps1 | 1.380805 | <0.00001 | up |
| ENSMUSG00000038611 | Phrf1 | -1.11552 | <0.00001 | down |
| ENSMUSG00000025537 | Phkg1 | -3.41317 | <0.00001 | down |
| ENSMUSG00000112255 | Gm47594 | -3.17908 | <0.00001 | down |
| ENSMUSG00000090942 | F830016B08Rik | 1.241676 | <0.00001 | up |
| ENSMUSG00000089862 | Umad1 | 1.280377 | <0.00001 | up |
| ENSMUSG00000078624 | Olfr613 | -1.60807 | <0.00001 | down |
| ENSMUSG00000121503 | H2-K2 | -1.67038 | <0.00001 | down |
| ENSMUSG00000117942 | Maskbp3 | -1.26994 | <0.00001 | down |
| ENSMUSG00000103928 | Gm37893 | -2.66271 | <0.00001 | down |
| ENSMUSG00000064137 | Rhox8 | -3.57078 | <0.00001 | down |
| ENSMUSG00000090210 | Itga10 | -2.47774 | <0.00001 | down |
| ENSMUSG00000028975 | Pex14 | 1.183428 | <0.00001 | up |
| ENSMUSG00000031521 | Aga | 1.445827 | <0.00001 | up |
| ENSMUSG00000086477 | Gm15506 | -2.73466 | <0.00001 | down |
| ENSMUSG00000024150 | Mcfd2 | 1.120299 | <0.00001 | up |
| ENSMUSG00000042066 | Tmcc2 | -1.74128 | <0.00001 | down |
| ENSMUSG00000031429 | Psmd10 | 1.630149 | <0.00001 | up |
| ENSMUSG00000025432 | Avil | -4.65298 | <0.00001 | down |
| ENSMUSG00000109336 | Samd4b | -1.7139 | <0.00001 | down |
| ENSMUSG00000045954 | Cavin2 | -1.21032 | <0.00001 | down |
| ENSMUSG00000033327 | Tnxb | -1.40043 | <0.00001 | down |
| ENSMUSG00000028378 | Ptgr1 | 1.662488 | <0.00001 | up |
| ENSMUSG00000032855 | Pkd1 | -1.1852 | <0.00001 | down |
| ENSMUSG00000015016 | Acsf3 | 1.29046 | <0.00001 | up |
| ENSMUSG00000039323 | Igfbp2 | 1.31366 | <0.00001 | up |
| ENSMUSG00000029199 | Lias | -1.33339 | <0.00001 | down |
| ENSMUSG00000072893 | 4933439C10Rik | -2.00495 | <0.00001 | down |
| ENSMUSG00000117292 | E330032C10Rik | -2.61277 | <0.00001 | down |
| ENSMUSG00000025899 | Alkbh8 | -1.10708 | <0.00001 | down |
| ENSMUSG00000009646 | Pla2g12b | 1.037514 | <0.00001 | up |
| ENSMUSG00000031706 | Rfx1 | -1.25042 | <0.00001 | down |
| ENSMUSG00000028952 | Zbtb48 | -1.18422 | <0.00001 | down |
| ENSMUSG00000015942 | Gtf2ird2 | -1.05505 | <0.00001 | down |
| ENSMUSG00000004100 | Ppan | -1.62854 | <0.00001 | down |
| ENSMUSG00000094396 | Vmn2r124 | -2.06542 | <0.00001 | down |
| ENSMUSG00000055044 | Pdlim1 | 1.329927 | <0.00001 | up |
| ENSMUSG00000044408 | Sptssa | 1.029105 | <0.00001 | up |
| ENSMUSG00000026365 | Cfh | 1.067398 | <0.00001 | up |
| ENSMUSG00000110519 | Olfr839 | -1.67777 | <0.00001 | down |
| ENSMUSG00000061436 | Hipk2 | -1.59861 | <0.00001 | down |
| ENSMUSG00000051043 | Gprc5c | 1.097758 | <0.00001 | up |
| ENSMUSG00000047649 | Cd3eap | -1.33274 | <0.00001 | down |
| ENSMUSG00000019726 | Lyst | -1.06901 | <0.00001 | down |
| ENSMUSG00000070985 | Acnat1 | -1.0038 | <0.00001 | down |
| ENSMUSG00000019179 | Mdh2 | 1.440675 | <0.00001 | up |
| ENSMUSG00000026097 | Ormdl1 | 1.369928 | <0.00001 | up |
| ENSMUSG00000041921 | Metap1d | -1.3139 | <0.00001 | down |
| ENSMUSG00000097415 | AU020206 | -1.61122 | <0.00001 | down |
| ENSMUSG00000019997 | Ccn2 | -1.31256 | <0.00001 | down |
| ENSMUSG00000041697 | Cox6a1 | 1.16947 | <0.00001 | up |
| ENSMUSG00000003813 | Rad23a | 1.448185 | <0.00001 | up |
| ENSMUSG00000019326 | Aoc3 | -3.31676 | <0.00001 | down |
| ENSMUSG00000029587 | Zfp12 | -1.06588 | <0.00001 | down |
| ENSMUSG00000041012 | Cmtm8 | -2.47206 | <0.00001 | down |
| ENSMUSG00000036985 | Zdhhc9 | 1.204471 | <0.00001 | up |
| ENSMUSG00000063882 | Uqcrh | 1.111651 | <0.00001 | up |
| ENSMUSG00000029439 | Sfswap | -1.41263 | <0.00001 | down |
| ENSMUSG00000078688 | Mup2 | -2.52756 | <0.00001 | down |
| ENSMUSG00000057110 | Cntrl | -1.24394 | <0.00001 | down |
| ENSMUSG00000032026 | Rexo2 | 1.140436 | <0.00001 | up |
| ENSMUSG00000108621 | Gm33989 | -1.12011 | <0.00001 | down |
| ENSMUSG00000030168 | Adipor2 | 1.211481 | <0.00001 | up |
| ENSMUSG00000026023 | Cdk15 | -3.2997 | <0.00001 | down |
| ENSMUSG00000111326 | Gm3953 | -3.55011 | <0.00001 | down |
| ENSMUSG00000091805 | Vmn2r108 | -2.22811 | <0.00001 | down |
| ENSMUSG00000050503 | Fbxl22 | -1.91839 | <0.00001 | down |
| ENSMUSG00000078570 | 1110065P20Rik | 1.580585 | <0.00001 | up |
| ENSMUSG00000056204 | Pgpep1 | 1.098533 | <0.00001 | up |
| ENSMUSG00000029703 | Lrwd1 | -1.28666 | <0.00001 | down |
| ENSMUSG00000057425 | Ugt2b37 | -1.98911 | <0.00001 | down |
| ENSMUSG00000039016 | Timm8b | 1.063192 | <0.00001 | up |
| ENSMUSG00000025453 | Nnt | 1.137924 | <0.00001 | up |
| ENSMUSG00000034450 | Gulo | 1.065546 | <0.00001 | up |
| ENSMUSG00000113388 | Gm48111 | -1.51388 | <0.00001 | down |
| ENSMUSG00000044938 | Klhl31 | -3.94642 | <0.00001 | down |
| ENSMUSG00000117879 | 2310015A16Rik | 1.730743 | <0.00001 | up |
| ENSMUSG00000022707 | Gbe1 | 1.617762 | <0.00001 | up |
| ENSMUSG00000028840 | Zfp593 | 1.312658 | <0.00001 | up |
| ENSMUSG00000079363 | Gbp4 | -1.64065 | <0.00001 | down |
| ENSMUSG00000004562 | Arhgef40 | -1.31853 | <0.00001 | down |
| ENSMUSG00000057133 | Chd6 | -1.23966 | <0.00001 | down |
| ENSMUSG00000025145 | Lrrc45 | -1.38727 | <0.00001 | down |
| ENSMUSG00000100510 | Hand2os1 | -1.72922 | <0.00001 | down |
| ENSMUSG00000054051 | Ercc6 | -1.01296 | <0.00001 | down |
| ENSMUSG00000053279 | Aldh1a1 | 1.278344 | <0.00001 | up |
| ENSMUSG00000075204 | Olfr1039 | -3.50285 | <0.00001 | down |
| ENSMUSG00000042506 | Usp22 | 1.122813 | <0.00001 | up |
| ENSMUSG00000025494 | Sigirr | 1.005518 | <0.00001 | up |
| ENSMUSG00000121347 | Gm2011 | -1.60074 | <0.00001 | down |
| ENSMUSG00000116903 | Gm19522 | -2.07928 | <0.00001 | down |
| ENSMUSG00000106943 | Dancr | -1.75328 | <0.00001 | down |
| ENSMUSG00000033910 | Gucy1a1 | -1.30613 | <0.00001 | down |
| ENSMUSG00000113630 | 4930404H11Rik | -3.03108 | <0.00001 | down |
| ENSMUSG00000026939 | Tmem141 | 1.30764 | <0.00001 | up |
| ENSMUSG00000003559 | As3mt | 1.21531 | <0.00001 | up |
| ENSMUSG00000018752 | Tnfsfm13 | 2.69619 | <0.00001 | up |
| ENSMUSG00000112366 | C730027H18Rik | -1.47153 | <0.00001 | down |
| ENSMUSG00000112739 | Gm20597 | -4.94024 | <0.00001 | down |
| ENSMUSG00000022234 | Cct5 | 1.06359 | <0.00001 | up |
| ENSMUSG00000105748 | Gm43088 | -4.75883 | <0.00001 | down |
| ENSMUSG00000021738 | Atxn7 | -1.13073 | <0.00001 | down |
| ENSMUSG00000027133 | Nop10 | 1.474347 | <0.00001 | up |
| ENSMUSG00000091151 | Vmn1r224 | -4.48574 | <0.00001 | down |
| ENSMUSG00000017677 | Wsb1 | -1.05338 | <0.00001 | down |
| ENSMUSG00000100600 | A230077H06Rik | -5.56878 | <0.00001 | down |
| ENSMUSG00000059248 | Septin9 | 1.221571 | <0.00001 | up |
| ENSMUSG00000039982 | Dtx4 | -1.43752 | <0.00001 | down |
| ENSMUSG00000039195 | Bbln | 1.334529 | <0.00001 | up |
| ENSMUSG00000032353 | Tmed3 | 1.43216 | <0.00001 | up |
| ENSMUSG00000003762 | Coq8b | 1.46986 | <0.00001 | up |
| ENSMUSG00000112038 | Gm47056 | -2.19615 | <0.00001 | down |
| ENSMUSG00000032040 | Dcps | 1.199786 | <0.00001 | up |
| ENSMUSG00000001065 | Zfp276 | -1.07094 | <0.00001 | down |
| ENSMUSG00000117284 | Gm7072 | -1.15699 | <0.00001 | down |
| ENSMUSG00000024610 | Cd74 | -1.01889 | <0.00001 | down |
| ENSMUSG00000051190 | Olfr1356 | -3.30884 | <0.00001 | down |
| ENSMUSG00000027176 | Cstf3 | -1.05351 | <0.00001 | down |
| ENSMUSG00000043418 | Lrit2 | -1.67069 | <0.00001 | down |
| ENSMUSG00000083594 | Gm13722 | -1.68067 | <0.00001 | down |
| ENSMUSG00000045237 | Eola1 | 1.74883 | <0.00001 | up |
| ENSMUSG00000026986 | Hnmt | 1.548058 | <0.00001 | up |
| ENSMUSG00000052428 | Tmco1 | 1.102063 | <0.00001 | up |
| ENSMUSG00000120473 | - | 2.439918 | <0.00001 | up |
| ENSMUSG00000090086 | AI480526 | -1.74855 | <0.00001 | down |
| ENSMUSG00000028403 | Zdhhc21 | -1.41514 | <0.00001 | down |
| ENSMUSG00000036552 | Ermard | -1.28533 | <0.00001 | down |
| ENSMUSG00000014606 | Slc25a11 | 1.054222 | <0.00001 | up |
| ENSMUSG00000061306 | Slc38a10 | 1.07845 | <0.00001 | up |
| ENSMUSG00000030082 | Sec61a1 | 1.008248 | <0.00001 | up |
| ENSMUSG00000020484 | Xbp1 | 1.237735 | <0.00001 | up |
| ENSMUSG00000079523 | Tmsb10 | -1.39106 | <0.00001 | down |
| ENSMUSG00000040429 | Mterf1a | -1.73918 | <0.00001 | down |
| ENSMUSG00000048537 | Phldb1 | -1.30217 | <0.00001 | down |
| ENSMUSG00000044519 | Zfp488 | -1.38495 | <0.00001 | down |
| ENSMUSG00000048490 | Nrip1 | -1.19386 | <0.00001 | down |
| ENSMUSG00000015759 | Cnih1 | 1.419534 | <0.00001 | up |
| ENSMUSG00000116692 | Gm49795 | -3.79184 | <0.00001 | down |
| ENSMUSG00000109787 | Gm45286 | -3.22379 | <0.00001 | down |
| ENSMUSG00000022471 | Xrcc6 | 1.018369 | <0.00001 | up |
| ENSMUSG00000022475 | Hdac7 | -1.37815 | <0.00001 | down |
| ENSMUSG00000057880 | Abat | 1.311794 | <0.00001 | up |
| ENSMUSG00000049152 | Ugt3a2 | 1.175121 | <0.00001 | up |
| ENSMUSG00000022821 | Hgd | 1.025836 | <0.00001 | up |
| ENSMUSG00000031877 | Ces2g | 1.105264 | <0.00001 | up |
| ENSMUSG00000019518 | Ap4m1 | -1.14654 | <0.00001 | down |
| ENSMUSG00000073434 | Wdr90 | -1.9329 | <0.00001 | down |
| ENSMUSG00000029267 | Mtf2 | -1.17846 | <0.00001 | down |
| ENSMUSG00000069804 | Gm10277 | -5.08867 | <0.00001 | down |
| ENSMUSG00000021273 | Fdft1 | 1.357563 | <0.00001 | up |
| ENSMUSG00000113795 | Gm48119 | -2.15402 | <0.00001 | down |
| ENSMUSG00000028527 | Ak4 | 1.000095 | <0.00001 | up |
| ENSMUSG00000026656 | Fcgr2b | -1.44147 | <0.00001 | down |
| ENSMUSG00000054387 | Mdm4 | -1.28272 | <0.00001 | down |
| ENSMUSG00000056124 | B4galt6 | -1.69734 | <0.00001 | down |
| ENSMUSG00000035585 | Tsen34 | 1.592607 | <0.00001 | up |
| ENSMUSG00000040471 | Ggt6 | 1.162098 | <0.00001 | up |
| ENSMUSG00000041126 | H2az2 | 1.106801 | <0.00001 | up |
| ENSMUSG00000053714 | 4732471J01Rik | -2.04835 | <0.00001 | down |
| ENSMUSG00000087177 | E130307A14Rik | -1.81836 | <0.00001 | down |
| ENSMUSG00000040165 | Cd209c | -2.69104 | <0.00001 | down |
| ENSMUSG00000036718 | Micall2 | -1.68476 | <0.00001 | down |
| ENSMUSG00000043286 | Pnpla1 | -1.35232 | <0.00001 | down |
| ENSMUSG00000020056 | Washc3 | 1.57203 | <0.00001 | up |
| ENSMUSG00000026946 | Nmi | 1.234406 | <0.00001 | up |
| ENSMUSG00000024902 | Mrpl11 | 1.140759 | <0.00001 | up |
| ENSMUSG00000100147 | 1700047M11Rik | -2.51617 | <0.00001 | down |
| ENSMUSG00000063087 | Gm10125 | -1.94243 | <0.00001 | down |
| ENSMUSG00000032842 | Abcc10 | -1.67261 | <0.00001 | down |
| ENSMUSG00000120775 | - | -3.81303 | <0.00001 | down |
| ENSMUSG00000025868 | Higd2a | 1.132581 | <0.00001 | up |
| ENSMUSG00000033386 | Frrs1 | -1.15279 | <0.00001 | down |
| ENSMUSG00000008206 | Cers4 | -1.91374 | <0.00001 | down |
| ENSMUSG00000112342 | Gm47031 | -2.32991 | <0.00001 | down |
| ENSMUSG00000004945 | Tmem242 | 1.10592 | <0.00001 | up |
| ENSMUSG00000028238 | Atp6v0d2 | 2.905049 | <0.00001 | up |
| ENSMUSG00000020477 | Mrps24 | 1.39026 | <0.00001 | up |
| ENSMUSG00000086877 | A230072C01Rik | -1.74534 | <0.00001 | down |
| ENSMUSG00000035378 | Shq1 | 1.54842 | <0.00001 | up |
| ENSMUSG00000025190 | Got1 | 1.572118 | <0.00001 | up |
| ENSMUSG00000054580 | Pla2r1 | -1.96442 | <0.00001 | down |
| ENSMUSG00000108511 | Gm44987 | -6.37213 | <0.00001 | down |
| ENSMUSG00000031532 | Saraf | 1.245177 | <0.00001 | up |
| ENSMUSG00000107050 | C030017G13Rik | -3.87626 | <0.00001 | down |
| ENSMUSG00000044792 | Isca1 | 1.134922 | <0.00001 | up |
| ENSMUSG00000108847 | B830042I05Rik | -2.42852 | <0.00001 | down |
| ENSMUSG00000071291 | Zfp58 | -1.07461 | <0.00001 | down |
| ENSMUSG00000015027 | Galns | 1.297015 | <0.00001 | up |
| ENSMUSG00000099707 | Gm8883 | 1.710646 | <0.00001 | up |
| ENSMUSG00000047793 | Sned1 | -1.16731 | <0.00001 | down |
| ENSMUSG00000119994 | - | -1.91269 | <0.00001 | down |
| ENSMUSG00000048997 | Atxn7l2 | -2.2451 | <0.00001 | down |
| ENSMUSG00000050772 | Olfr1124 | -3.46825 | <0.00001 | down |
| ENSMUSG00000022752 | Tomm70a | 1.013654 | <0.00001 | up |
| ENSMUSG00000022236 | Ropn1l | 1.25452 | <0.00001 | up |
| ENSMUSG00000004842 | Pou1f1 | -3.8861 | <0.00001 | down |
| ENSMUSG00000106073 | Gm42892 | -3.84133 | <0.00001 | down |
| ENSMUSG00000067916 | Zfp991 | -2.38589 | <0.00001 | down |
| ENSMUSG00000013833 | Med16 | 1.466452 | <0.00001 | up |
| ENSMUSG00000107411 | Gm19040 | -3.34802 | <0.00001 | down |
| ENSMUSG00000055943 | Emc7 | 1.002743 | <0.00001 | up |
| ENSMUSG00000039193 | Nlrc4 | -2.0652 | <0.00001 | down |
| ENSMUSG00000056600 | Olfr90 | -2.09135 | <0.00001 | down |
| ENSMUSG00000110996 | Gm36251 | -1.55211 | <0.00001 | down |
| ENSMUSG00000033096 | Apmap | 1.233492 | <0.00001 | up |
| ENSMUSG00000020122 | Egfr | 1.352388 | <0.00001 | up |
| ENSMUSG00000081534 | Slc48a1 | 1.151838 | <0.00001 | up |
| ENSMUSG00000032478 | Nme6 | 1.086206 | <0.00001 | up |
| ENSMUSG00000001300 | Efnb2 | -1.58092 | <0.00001 | down |
| ENSMUSG00000032199 | Polr2m | 1.409496 | <0.00001 | up |
| ENSMUSG00000024871 | Doc2g | -3.027 | <0.00001 | down |
| ENSMUSG00000083863 | Gm13341 | -2.66912 | <0.00001 | down |
| ENSMUSG00000032398 | Snapc5 | 1.36322 | <0.00001 | up |
| ENSMUSG00000042895 | Abra | -5.93163 | <0.00001 | down |
| ENSMUSG00000052906 | Ubxn8 | 1.206646 | <0.00001 | up |
| ENSMUSG00000060862 | Zbtb40 | -1.28196 | <0.00001 | down |
| ENSMUSG00000039018 | Mtg1 | 1.758283 | <0.00001 | up |
| ENSMUSG00000020585 | Laptm4a | 1.078815 | <0.00001 | up |
| ENSMUSG00000034820 | Cpsf7 | -1.09728 | <0.00001 | down |
| ENSMUSG00000050270 | Tmem220 | 1.61521 | <0.00001 | up |
| ENSMUSG00000121506 | - | -1.01474 | <0.00001 | down |
| ENSMUSG00000085786 | Gm15987 | -1.89122 | <0.00001 | down |
| ENSMUSG00000079036 | Alkbh1 | -2.15605 | <0.00001 | down |
| ENSMUSG00000047409 | Ctdspl | -1.18641 | <0.00001 | down |
| ENSMUSG00000044906 | 4930503L19Rik | -1.85038 | <0.00001 | down |
| ENSMUSG00000029380 | Cxcl1 | 3.367008 | <0.00001 | up |
| ENSMUSG00000031728 | Zfp821 | -1.90525 | <0.00001 | down |
| ENSMUSG00000025255 | Zfhx4 | -1.18028 | <0.00001 | down |
| ENSMUSG00000121353 | - | -2.24309 | <0.00001 | down |
| ENSMUSG00000068876 | Cgn | -1.23636 | <0.00001 | down |
| ENSMUSG00000104060 | Gm37954 | -2.20436 | <0.00001 | down |
| ENSMUSG00000030468 | Siglecg | -2.93554 | <0.00001 | down |
| ENSMUSG00000026074 | Map4k4 | -1.3398 | <0.00001 | down |
| ENSMUSG00000015474 | Ppt2 | 1.329008 | <0.00001 | up |
| ENSMUSG00000107838 | Gm45769 | -1.28954 | <0.00001 | down |
| ENSMUSG00000057388 | Mrpl18 | -1.17033 | <0.00001 | down |
| ENSMUSG00000058076 | Sdhc | 1.094835 | <0.00001 | up |
| ENSMUSG00000087299 | Gm12953 | -2.41652 | <0.00001 | down |
| ENSMUSG00000028567 | Txndc12 | 1.509518 | <0.00001 | up |
| ENSMUSG00000042408 | Zmym6 | -1.42519 | <0.00001 | down |
| ENSMUSG00000040415 | Dtx3 | -1.79754 | <0.00001 | down |
| ENSMUSG00000023791 | Pigx | 1.09637 | <0.00001 | up |
| ENSMUSG00000037942 | Crp | 1.524875 | <0.00001 | up |
| ENSMUSG00000078348 | Sf3b5 | 1.708441 | <0.00001 | up |
| ENSMUSG00000037331 | Larp1 | -1.34045 | <0.00001 | down |
| ENSMUSG00000097583 | 6430590A07Rik | -4.53663 | <0.00001 | down |
| ENSMUSG00000030062 | Rpn1 | 1.197184 | <0.00001 | up |
| ENSMUSG00000068184 | Ndufaf2 | 1.364938 | <0.00001 | up |
| ENSMUSG00000035713 | Usp35 | -1.88643 | <0.00001 | down |
| ENSMUSG00000041920 | Slc16a6 | -1.19117 | <0.00001 | down |
| ENSMUSG00000022604 | Cep97 | -1.50833 | <0.00001 | down |
| ENSMUSG00000078768 | Zfp566 | -2.39171 | <0.00001 | down |
| ENSMUSG00000097482 | Gm17634 | -1.88907 | <0.00001 | down |
| ENSMUSG00000018372 | Cep95 | -1.41727 | <0.00001 | down |
| ENSMUSG00000019916 | P4ha1 | -1.2866 | <0.00001 | down |
| ENSMUSG00000034957 | Cebpa | 1.13599 | <0.00001 | up |
| ENSMUSG00000104721 | Gm42696 | -4.19352 | <0.00001 | down |
| ENSMUSG00000056592 | Zfp658 | -1.96134 | <0.00001 | down |
| ENSMUSG00000056493 | Foxk1 | 1.130602 | <0.00001 | up |
| ENSMUSG00000105855 | Gm42681 | -2.73979 | <0.00001 | down |
| ENSMUSG00000029250 | Polr2b | 1.429041 | <0.00001 | up |
| ENSMUSG00000029076 | Sdf4 | 1.139573 | <0.00001 | up |
| ENSMUSG00000021428 | Riok1 | -1.40405 | <0.00001 | down |
| ENSMUSG00000099632 | 2900093K20Rik | -2.36039 | <0.00001 | down |
| ENSMUSG00000029426 | Scarb2 | 1.401281 | <0.00001 | up |
| ENSMUSG00000104682 | Gm42636 | -2.74879 | <0.00001 | down |
| ENSMUSG00000022623 | Shank3 | -1.6176 | <0.00001 | down |
| ENSMUSG00000029192 | Tbc1d14 | -1.33561 | <0.00001 | down |
| ENSMUSG00000047150 | 1700001C19Rik | 1.180758 | <0.00001 | up |
| ENSMUSG00000015745 | Plekho1 | -1.34211 | <0.00001 | down |
| ENSMUSG00000112096 | A430103D13Rik | -2.68803 | <0.00001 | down |
| ENSMUSG00000022477 | Aco2 | 1.084846 | <0.00001 | up |
| ENSMUSG00000039168 | Dap | 1.008113 | <0.00001 | up |
| ENSMUSG00000043065 | Spice1 | -1.80726 | <0.00001 | down |
| ENSMUSG00000094822 | Olfr243 | -5.03794 | <0.00001 | down |
| ENSMUSG00000107624 | Gm44005 | -5.64069 | <0.00001 | down |
| ENSMUSG00000022684 | Bfar | -1.34804 | <0.00001 | down |
| ENSMUSG00000032377 | Plscr4 | -2.01861 | <0.00001 | down |
| ENSMUSG00000068394 | Cep152 | -1.80583 | <0.00001 | down |
| ENSMUSG00000085687 | Gm16153 | -2.87869 | <0.00001 | down |
| ENSMUSG00000038736 | Nudcd1 | -1.39028 | <0.00001 | down |
| ENSMUSG00000085622 | 3110056K07Rik | -2.06629 | <0.00001 | down |
| ENSMUSG00000004070 | Hmox2 | 1.008893 | <0.00001 | up |
| ENSMUSG00000095975 | Cphx1 | -2.16856 | <0.00001 | down |
| ENSMUSG00000043801 | Oaz1-ps | 2.26402 | <0.00001 | up |
| ENSMUSG00000024096 | Ralbp1 | -1.0024 | <0.00001 | down |
| ENSMUSG00000020523 | Fam114a2 | -1.01691 | <0.00001 | down |
| ENSMUSG00000113290 | A530058O07Rik | -2.91303 | <0.00001 | down |
| ENSMUSG00000032458 | Copb2 | 1.12745 | <0.00001 | up |
| ENSMUSG00000036553 | Sh3tc1 | -1.53455 | <0.00001 | down |
| ENSMUSG00000001588 | Acap1 | -2.16313 | <0.00001 | down |
| ENSMUSG00000056211 | R3hdm1 | -1.03641 | <0.00001 | down |
| ENSMUSG00000025959 | Klf7 | -1.88972 | <0.00001 | down |
| ENSMUSG00000024875 | Yif1a | 1.335685 | <0.00001 | up |
| ENSMUSG00000028563 | Tm2d1 | 1.474469 | <0.00001 | up |
| ENSMUSG00000030161 | Gabarapl1 | 1.282354 | <0.00001 | up |
| ENSMUSG00000036898 | Zfp157 | -1.4861 | <0.00001 | down |
| ENSMUSG00000087120 | Gm12279 | -2.25536 | <0.00001 | down |
| ENSMUSG00000034245 | Hdac11 | 1.150425 | <0.00001 | up |
| ENSMUSG00000034708 | Grn | 1.433697 | <0.00001 | up |
| ENSMUSG00000107336 | Gm43461 | -2.9912 | <0.00001 | down |
| ENSMUSG00000030079 | Ruvbl1 | 1.440075 | <0.00001 | up |
| ENSMUSG00000049354 | Dcaf7 | 1.145173 | <0.00001 | up |
| ENSMUSG00000079012 | Serpina3m | 1.879833 | <0.00001 | up |
| ENSMUSG00000000560 | Gabra2 | -3.21403 | <0.00001 | down |
| ENSMUSG00000071005 | Ccl19 | -1.68232 | <0.00001 | down |
| ENSMUSG00000070427 | Il18bp | 1.489195 | <0.00001 | up |
| ENSMUSG00000059981 | Taok2 | -1.17593 | <0.00001 | down |
| ENSMUSG00000004896 | Rrnad1 | -1.46596 | <0.00001 | down |
| ENSMUSG00000022139 | Mbnl2 | -1.24343 | <0.00001 | down |
| ENSMUSG00000054737 | Zfp182 | -1.78828 | <0.00001 | down |
| ENSMUSG00000020385 | Clk4 | -1.22534 | <0.00001 | down |
| ENSMUSG00000005621 | Zfp592 | -1.01492 | <0.00001 | down |
| ENSMUSG00000007892 | Rplp1 | 1.003487 | <0.00001 | up |
| ENSMUSG00000024985 | Tcf7l2 | -1.0196 | <0.00001 | down |
| ENSMUSG00000026229 | Psmd1 | -1.88937 | <0.00001 | down |
| ENSMUSG00000073676 | Hspe1 | 1.134813 | <0.00001 | up |
| ENSMUSG00000042605 | Atxn2 | -1.06316 | <0.00001 | down |
| ENSMUSG00000030652 | Coq7 | 1.441092 | <0.00001 | up |
| ENSMUSG00000024238 | Zeb1 | -1.12786 | <0.00001 | down |
| ENSMUSG00000052005 | Gm9864 | -2.44018 | <0.00001 | down |
| ENSMUSG00000025317 | Car5a | 1.635063 | <0.00001 | up |
| ENSMUSG00000119895 | Gm22513 | -3.27532 | <0.00001 | down |
| ENSMUSG00000020376 | Rnf130 | 1.234313 | <0.00001 | up |
| ENSMUSG00000060459 | Kng2 | 1.052648 | <0.00001 | up |
| ENSMUSG00000110156 | Gm42067 | -2.14604 | <0.00001 | down |
| ENSMUSG00000031672 | Got2 | 1.178377 | <0.00001 | up |
| ENSMUSG00000041354 | Rgl2 | -1.42378 | <0.00001 | down |
| ENSMUSG00000031379 | Pir | 1.217839 | <0.00001 | up |
| ENSMUSG00000029672 | Fam3c | 1.168622 | <0.00001 | up |
| ENSMUSG00000036502 | Tmem255a | -2.38683 | <0.00001 | down |
| ENSMUSG00000025782 | Taf3 | -1.49502 | <0.00001 | down |
| ENSMUSG00000022174 | Dad1 | 1.594179 | <0.00001 | up |
| ENSMUSG00000057335 | Cep170 | -1.11715 | <0.00001 | down |
| ENSMUSG00000085867 | Gm5834 | -2.24024 | <0.00001 | down |
| ENSMUSG00000025326 | Ube3a | -1.09815 | <0.00001 | down |
| ENSMUSG00000025816 | Sec61a2 | -2.0987 | <0.00001 | down |
| ENSMUSG00000020017 | Hal | 1.167552 | <0.00001 | up |
| ENSMUSG00000052520 | Cyp2j5 | 1.366073 | <0.00001 | up |
| ENSMUSG00000106717 | Gm42798 | -3.74816 | <0.00001 | down |
| ENSMUSG00000047502 | Mroh7 | -3.27706 | <0.00001 | down |
| ENSMUSG00000026272 | Agxt | 1.403763 | <0.00001 | up |
| ENSMUSG00000041654 | Slc39a11 | 1.629333 | <0.00001 | up |
| ENSMUSG00000026921 | Egfl7 | -1.18159 | <0.00001 | down |
| ENSMUSG00000026785 | Pkn3 | -2.56628 | <0.00001 | down |
| ENSMUSG00000020427 | Igfbp3 | -1.3981 | <0.00001 | down |
| ENSMUSG00000032959 | Pebp1 | 1.03167 | <0.00001 | up |
| ENSMUSG00000027276 | Jag1 | -1.54291 | <0.00001 | down |
| ENSMUSG00000020570 | Sypl | 1.307834 | <0.00001 | up |
| ENSMUSG00000106992 | Gm43167 | -3.428 | <0.00001 | down |
| ENSMUSG00000036181 | H1f2 | 1.706317 | <0.00001 | up |
| ENSMUSG00000022210 | Dhrs4 | 1.047733 | <0.00001 | up |
| ENSMUSG00000006307 | Kmt2b | -1.4113 | <0.00001 | down |
| ENSMUSG00000002486 | Tchp | -1.89616 | <0.00001 | down |
| ENSMUSG00000021458 | Aopep | -1.0672 | <0.00001 | down |
| ENSMUSG00000072772 | Grcc10 | 1.256016 | <0.00001 | up |
| ENSMUSG00000107225 | Gm43637 | -2.96615 | <0.00001 | down |
| ENSMUSG00000051586 | Mical3 | -1.00639 | <0.00001 | down |
| ENSMUSG00000032744 | Heyl | -1.70169 | <0.00001 | down |
| ENSMUSG00000101462 | Gm3052 | -2.91262 | <0.00001 | down |
| ENSMUSG00000022587 | Ly6e | 1.192282 | <0.00001 | up |
| ENSMUSG00000031353 | Rbbp7 | 1.113099 | <0.00001 | up |
| ENSMUSG00000037797 | Adh4 | 1.027056 | <0.00001 | up |
| ENSMUSG00000015090 | Ptgds | -2.62681 | <0.00001 | down |
| ENSMUSG00000018821 | Avpi1 | 1.41346 | <0.00001 | up |
| ENSMUSG00000030815 | Phkg2 | -1.15358 | <0.00001 | down |
| ENSMUSG00000039234 | Sec24d | 1.094033 | <0.00001 | up |
| ENSMUSG00000098055 | Gm26947 | -2.73702 | <0.00001 | down |
| ENSMUSG00000016382 | Pls3 | 1.044567 | <0.00001 | up |
| ENSMUSG00000029656 | C8b | 1.348807 | <0.00001 | up |
| ENSMUSG00000012640 | Zfp715 | -1.27265 | <0.00001 | down |
| ENSMUSG00000059939 | 9430015G10Rik | -1.94802 | <0.00001 | down |
| ENSMUSG00000085180 | AI838599 | -3.88238 | <0.00001 | down |
| ENSMUSG00000031967 | Afg3l1 | 1.042759 | <0.00001 | up |
| ENSMUSG00000046079 | Lrrc8d | 1.258216 | <0.00001 | up |
| ENSMUSG00000059824 | Dbp | 3.574642 | <0.00001 | up |
| ENSMUSG00000024990 | Rbp4 | 1.408783 | <0.00001 | up |
| ENSMUSG00000092471 | Cyp21a2-ps | -1.75174 | <0.00001 | down |
| ENSMUSG00000025314 | Ptprj | 1.024148 | <0.00001 | up |
| ENSMUSG00000001062 | Vps9d1 | -1.15468 | <0.00001 | down |
| ENSMUSG00000042894 | Olfr1260 | -2.39078 | <0.00001 | down |
| ENSMUSG00000112734 | Gm47644 | -4.0661 | <0.00001 | down |
| ENSMUSG00000014867 | Surf4 | 1.00807 | <0.00001 | up |
| ENSMUSG00000024747 | Aldh1a7 | 1.284974 | <0.00001 | up |
| ENSMUSG00000050777 | Tmem37 | 1.281197 | <0.00001 | up |
| ENSMUSG00000030298 | Sec13 | 1.161192 | <0.00001 | up |
| ENSMUSG00000031060 | Rbm10 | -1.15779 | <0.00001 | down |
| ENSMUSG00000106838 | 1810017P11Rik | -2.56209 | <0.00001 | down |
| ENSMUSG00000112110 | Gm15608 | -1.84422 | <0.00001 | down |
| ENSMUSG00000003604 | Aven | 1.407178 | <0.00001 | up |
| ENSMUSG00000041479 | Syt15 | -2.77504 | <0.00001 | down |
| ENSMUSG00000082762 | Gm12366 | -6.214 | <0.00001 | down |
| ENSMUSG00000118215 | Vmn1r55 | -3.02641 | <0.00001 | down |
| ENSMUSG00000113788 | Gm47904 | -5.04448 | <0.00001 | down |
| ENSMUSG00000041445 | Mmrn2 | -1.34689 | <0.00001 | down |
| ENSMUSG00000002767 | Mrpl2 | 1.093352 | <0.00001 | up |
| ENSMUSG00000025089 | Gfra1 | 1.392614 | <0.00001 | up |
| ENSMUSG00000015575 | Atp6v0e | 1.459735 | <0.00001 | up |
| ENSMUSG00000040283 | Btnl9 | -1.62194 | <0.00001 | down |
| ENSMUSG00000029859 | Epha1 | 1.094169 | <0.00001 | up |
| ENSMUSG00000007836 | Hnrnpa0 | 1.548163 | <0.00001 | up |
| ENSMUSG00000059708 | Akap17b | -1.41076 | <0.00001 | down |
| ENSMUSG00000056492 | Adgrf5 | -1.21351 | <0.00001 | down |
| ENSMUSG00000039623 | Ap5z1 | -1.39069 | <0.00001 | down |
| ENSMUSG00000000759 | Tubgcp3 | -1.14764 | <0.00001 | down |
| ENSMUSG00000028461 | Ccdc107 | -1.12571 | <0.00001 | down |
| ENSMUSG00000021660 | Btf3 | 1.127124 | <0.00001 | up |
| ENSMUSG00000078636 | Gm7336 | 1.776288 | <0.00001 | up |
| ENSMUSG00000027474 | Ccm2l | -1.56496 | <0.00001 | down |
| ENSMUSG00000105287 | Gm43577 | -2.56886 | <0.00001 | down |
| ENSMUSG00000024665 | Fads2 | 1.567144 | <0.00001 | up |
| ENSMUSG00000001247 | Lsr | 1.025489 | <0.00001 | up |
| ENSMUSG00000035992 | Fnip1 | -1.62984 | <0.00001 | down |
| ENSMUSG00000054757 | Akr1c20 | 1.532444 | <0.00001 | up |
| ENSMUSG00000022098 | Bmp1 | -1.08462 | <0.00001 | down |
| ENSMUSG00000015002 | Efr3a | -1.1738 | <0.00001 | down |
| ENSMUSG00000031556 | Tm2d2 | 1.401212 | <0.00001 | up |
| ENSMUSG00000022820 | Ndufb4 | -1.0229 | <0.00001 | down |
| ENSMUSG00000028639 | Ybx1 | 1.174671 | <0.00001 | up |
| ENSMUSG00000041736 | Tspo | 1.679983 | <0.00001 | up |
| ENSMUSG00000033209 | Ttc28 | -1.7117 | <0.00001 | down |
| ENSMUSG00000032348 | Gsta4 | 1.475113 | <0.00001 | up |
| ENSMUSG00000037787 | Coa8 | 1.153686 | <0.00001 | up |
| ENSMUSG00000031161 | Hdac6 | -1.184 | <0.00001 | down |
| ENSMUSG00000074862 | BC025920 | -2.4898 | <0.00001 | down |
| ENSMUSG00000042453 | Reln | -1.1898 | <0.00001 | down |
| ENSMUSG00000004040 | Stat3 | 1.234548 | <0.00001 | up |
| ENSMUSG00000032126 | Hmbs | 1.145046 | <0.00001 | up |
| ENSMUSG00000032014 | Oaf | 1.098274 | <0.00001 | up |
| ENSMUSG00000078919 | Dpm1 | 2.004935 | <0.00001 | up |
| ENSMUSG00000002064 | Sdf2 | 1.236849 | <0.00001 | up |
| ENSMUSG00000032383 | Ppib | 1.213216 | <0.00001 | up |
| ENSMUSG00000105868 | Gm43766 | -3.25009 | <0.00001 | down |
| ENSMUSG00000056724 | Nbeal2 | -1.39391 | <0.00001 | down |
| ENSMUSG00000027099 | Mtx2 | 1.180628 | <0.00001 | up |
| ENSMUSG00000002028 | Kmt2a | -1.79448 | <0.00001 | down |
| ENSMUSG00000071866 | Ppia | 1.159401 | <0.00001 | up |
| ENSMUSG00000118516 | Cyp2d13 | -1.07898 | <0.00001 | down |
| ENSMUSG00000105476 | Gm35439 | -2.30417 | <0.00001 | down |
| ENSMUSG00000026411 | Tmem9 | 1.46809 | <0.00001 | up |
| ENSMUSG00000040260 | Daam2 | -1.66092 | <0.00001 | down |
| ENSMUSG00000028328 | Tmod1 | -1.825 | <0.00001 | down |
| ENSMUSG00000040712 | Camta2 | -1.57673 | <0.00001 | down |
| ENSMUSG00000002846 | Timmdc1 | 1.396277 | <0.00001 | up |
| ENSMUSG00000045826 | Ptprcap | -3.31299 | <0.00001 | down |
| ENSMUSG00000038299 | Wdr36 | 1.186903 | <0.00001 | up |
| ENSMUSG00000054619 | Mettl7a1 | 1.221785 | <0.00001 | up |
| ENSMUSG00000026932 | Nacc2 | -1.0059 | <0.00001 | down |
| ENSMUSG00000015944 | Castor2 | -1.76578 | <0.00001 | down |
| ENSMUSG00000079334 | Naa80 | -1.17552 | <0.00001 | down |
| ENSMUSG00000020423 | Btg2 | 2.312181 | <0.00001 | up |
| ENSMUSG00000049280 | Olfr509 | -1.95268 | <0.00001 | down |
| ENSMUSG00000112542 | Gm47840 | -3.33745 | <0.00001 | down |
| ENSMUSG00000030879 | Mrpl17 | 1.098198 | <0.00001 | up |
| ENSMUSG00000027828 | Ssr3 | 1.136484 | <0.00001 | up |
| ENSMUSG00000053128 | Rnf26 | 1.441966 | <0.00001 | up |
| ENSMUSG00000047260 | Emc6 | 1.082226 | <0.00001 | up |
| ENSMUSG00000001918 | Slc1a5 | -1.71236 | <0.00001 | down |
| ENSMUSG00000029720 | Gm20605 | -2.32683 | <0.00001 | down |
| ENSMUSG00000011114 | Tbrg1 | 1.221404 | <0.00001 | up |
| ENSMUSG00000064068 | Mtx1 | 1.039321 | <0.00001 | up |
| ENSMUSG00000025220 | Oga | -1.06469 | <0.00001 | down |
| ENSMUSG00000042369 | Rbm45 | 2.461663 | <0.00001 | up |
| ENSMUSG00000019471 | Cdc37 | 1.152012 | <0.00001 | up |
| ENSMUSG00000024725 | Ostf1 | 1.373073 | <0.00001 | up |
| ENSMUSG00000020069 | Hnrnph3 | -1.36002 | <0.00001 | down |
| ENSMUSG00000037686 | Aspg | -1.3876 | <0.00001 | down |
| ENSMUSG00000022019 | Tdrd3 | -1.12342 | <0.00001 | down |
| ENSMUSG00000037344 | Slc12a9 | -1.95611 | <0.00001 | down |
| ENSMUSG00000024248 | Cox7a2l | 1.250896 | <0.00001 | up |
| ENSMUSG00000032224 | Fam81a | 2.733491 | <0.00001 | up |
| ENSMUSG00000052752 | Traf7 | -1.41525 | <0.00001 | down |
| ENSMUSG00000031153 | Gripap1 | -1.48214 | <0.00001 | down |
| ENSMUSG00000002320 | Tm9sf1 | 1.148816 | <0.00001 | up |
| ENSMUSG00000024359 | Hspa9 | 1.204497 | <0.00001 | up |
| ENSMUSG00000057375 | Yipf1 | 1.606346 | <0.00001 | up |
| ENSMUSG00000004633 | Chn2 | 1.063627 | <0.00001 | up |
| ENSMUSG00000019254 | Ppp1r12c | -1.06014 | <0.00001 | down |
| ENSMUSG00000031634 | Ufsp2 | 1.072541 | <0.00001 | up |
| ENSMUSG00000008200 | Fnbp4 | -1.71402 | <0.00001 | down |
| ENSMUSG00000008892 | Vdac3 | 1.323587 | <0.00001 | up |
| ENSMUSG00000106024 | A530083M17Rik | -5.33742 | <0.00001 | down |
| ENSMUSG00000039062 | Anpep | 1.089548 | <0.00001 | up |
| ENSMUSG00000030122 | Ptms | -1.06818 | <0.00001 | down |
| ENSMUSG00000091845 | Rpl36-ps12 | -1.91848 | <0.00001 | down |
| ENSMUSG00000032582 | Rbm6 | -1.39551 | <0.00001 | down |
| ENSMUSG00000029610 | Aimp2 | 1.903263 | <0.00001 | up |
| ENSMUSG00000051111 | Sv2c | -3.87394 | <0.00001 | down |
| ENSMUSG00000027304 | Rtf1 | -1.3608 | <0.00001 | down |
| ENSMUSG00000021094 | Dhrs7 | 1.563603 | <0.00001 | up |
| ENSMUSG00000022914 | Brwd1 | -1.01524 | <0.00001 | down |
| ENSMUSG00000057103 | Nat8f1 | 1.264024 | <0.00001 | up |
| ENSMUSG00000029153 | Ociad2 | 1.108844 | <0.00001 | up |
| ENSMUSG00000074639 | Rdh16f2 | 1.118091 | <0.00001 | up |
| ENSMUSG00000024193 | Phf1 | -1.08498 | <0.00001 | down |
| ENSMUSG00000055312 | Them7 | 1.274112 | <0.00001 | up |
| ENSMUSG00000008604 | Ubqln4 | -1.00768 | <0.00001 | down |
| ENSMUSG00000027195 | Hsd17b12 | 1.378111 | <0.00001 | up |
| ENSMUSG00000109628 | BC024386 | -1.40857 | <0.00001 | down |
| ENSMUSG00000001569 | Nom1 | -1.33165 | <0.00001 | down |
| ENSMUSG00000044285 | Ubb-ps | 1.989968 | <0.00001 | up |
| ENSMUSG00000031389 | Arhgap4 | -1.55456 | <0.00001 | down |
| ENSMUSG00000044068 | Zrsr1 | -1.13992 | <0.00001 | down |
| ENSMUSG00000013033 | Adgrl1 | -1.54463 | <0.00001 | down |
| ENSMUSG00000029152 | Ociad1 | 1.188323 | <0.00001 | up |
| ENSMUSG00000043241 | Upf2 | -1.77926 | <0.00001 | down |
| ENSMUSG00000114407 | Gm48765 | -1.52003 | <0.00001 | down |
| ENSMUSG00000019210 | Atp6v1e1 | 1.529702 | <0.00001 | up |
| ENSMUSG00000055302 | Mrfap1 | 1.196657 | <0.00001 | up |
| ENSMUSG00000024079 | Eif2ak2 | -1.88401 | <0.00001 | down |
| ENSMUSG00000028420 | Tmem38b | 1.062147 | <0.00001 | up |
| ENSMUSG00000005417 | Mprip | -1.37762 | <0.00001 | down |
| ENSMUSG00000063704 | Mapk15 | -1.8527 | <0.00001 | down |
| ENSMUSG00000031029 | Eif3f | 1.482121 | <0.00001 | up |
| ENSMUSG00000040234 | Tm7sf3 | 1.023794 | <0.00001 | up |
| ENSMUSG00000025372 | Baiap2 | -2.13972 | <0.00001 | down |
| ENSMUSG00000022537 | Tmem44 | -1.88256 | <0.00001 | down |
| ENSMUSG00000042599 | Kdm7a | -1.91973 | <0.00001 | down |
| ENSMUSG00000040681 | Hmgn1 | 1.575096 | <0.00001 | up |
| ENSMUSG00000031158 | Timm17b | 1.108655 | <0.00001 | up |
| ENSMUSG00000107096 | Gm43597 | -2.47982 | <0.00001 | down |
| ENSMUSG00000037434 | Slc30a1 | 1.08956 | <0.00001 | up |
| ENSMUSG00000021068 | Nin | -2.16368 | <0.00001 | down |
| ENSMUSG00000034974 | Dapk3 | 1.294309 | <0.00001 | up |
| ENSMUSG00000095937 | Gm12671 | 1.567257 | <0.00001 | up |
| ENSMUSG00000014778 | Fhod1 | -1.50524 | <0.00001 | down |
| ENSMUSG00000027249 | F2 | 1.303318 | <0.00001 | up |
| ENSMUSG00000031959 | Wdr59 | -1.18163 | <0.00001 | down |
| ENSMUSG00000098371 | Gm28037 | 10.82443 | <0.00001 | up |
| ENSMUSG00000097254 | C430042M11Rik | -3.2776 | <0.00001 | down |
| ENSMUSG00000022244 | Amacr | 1.025509 | <0.00001 | up |
| ENSMUSG00000025076 | Casp7 | 1.2759 | <0.00001 | up |
| ENSMUSG00000112972 | Gm48417 | -3.45273 | <0.00001 | down |
| ENSMUSG00000081406 | Rps6-ps4 | 2.155618 | <0.00001 | up |
| ENSMUSG00000078923 | Ube2v1 | 1.49311 | <0.00001 | up |
| ENSMUSG00000035171 | 1110059E24Rik | 1.370062 | <0.00001 | up |
| ENSMUSG00000026617 | Bpnt1 | 1.352181 | <0.00001 | up |
| ENSMUSG00000114148 | Gm47701 | -3.86145 | <0.00001 | down |
| ENSMUSG00000051786 | Tubgcp6 | -1.26187 | <0.00001 | down |
| ENSMUSG00000022799 | Arhgap31 | -1.25574 | <0.00001 | down |
| ENSMUSG00000104011 | Gm32391 | -2.00881 | <0.00001 | down |
| ENSMUSG00000047050 | Olfr914 | -5.73123 | <0.00001 | down |
| ENSMUSG00000062070 | Pgk1 | 1.476076 | <0.00001 | up |
| ENSMUSG00000045038 | Prkce | -2.04689 | <0.00001 | down |
| ENSMUSG00000003166 | Dgcr2 | 1.091633 | <0.00001 | up |
| ENSMUSG00000031119 | Gpc4 | 1.081828 | <0.00001 | up |
| ENSMUSG00000021248 | Tmed10 | 1.384814 | <0.00001 | up |
| ENSMUSG00000081648 | Gm13423 | -3.63775 | <0.00001 | down |
| ENSMUSG00000034528 | Hsd17b13 | 1.376777 | <0.00001 | up |
| ENSMUSG00000032125 | Robo4 | -1.65121 | <0.00001 | down |
| ENSMUSG00000075705 | Msrb1 | 1.272827 | <0.00001 | up |
| ENSMUSG00000097462 | 9530026P05Rik | -4.40713 | <0.00001 | down |
| ENSMUSG00000031755 | Bbs2 | -1.4633 | <0.00001 | down |
| ENSMUSG00000020954 | Strn3 | -1.38362 | <0.00001 | down |
| ENSMUSG00000026761 | Orc4 | -1.1269 | <0.00001 | down |
| ENSMUSG00000068749 | Psma5 | 1.465877 | <0.00001 | up |
| ENSMUSG00000032449 | Slc25a36 | -1.60347 | <0.00001 | down |
| ENSMUSG00000028179 | Cth | 1.331012 | <0.00001 | up |
| ENSMUSG00000032621 | Srek1 | -1.23712 | <0.00001 | down |
| ENSMUSG00000025867 | Cplx2 | -2.3491 | <0.00001 | down |
| ENSMUSG00000020674 | Pxdn | -1.21989 | <0.00001 | down |
| ENSMUSG00000040026 | Saa3 | 1.869497 | <0.00001 | up |
| ENSMUSG00000024067 | Dpy30 | 1.543493 | <0.00001 | up |
| ENSMUSG00000022094 | Slc39a14 | 1.065671 | <0.00001 | up |
| ENSMUSG00000037617 | Spag1 | -2.43676 | <0.00001 | down |
| ENSMUSG00000022466 | Rpap3 | 2.010434 | <0.00001 | up |
| ENSMUSG00000026153 | Fam135a | -1.1299 | <0.00001 | down |
| ENSMUSG00000011752 | Pgam1 | 1.373484 | <0.00001 | up |
| ENSMUSG00000051716 | Apon | 1.10195 | <0.00001 | up |
| ENSMUSG00000034424 | Gcsh | 1.177055 | <0.00001 | up |
| ENSMUSG00000038241 | Cep250 | -1.56236 | <0.00001 | down |
| ENSMUSG00000026614 | Slc30a10 | -2.09744 | <0.00001 | down |
| ENSMUSG00000022816 | Fstl1 | -1.77746 | <0.00001 | down |
| ENSMUSG00000030096 | Slc6a6 | 1.269152 | <0.00001 | up |
| ENSMUSG00000002265 | Peg3 | -1.69323 | <0.00001 | down |
| ENSMUSG00000027411 | Vps16 | -1.05621 | <0.00001 | down |
| ENSMUSG00000120012 | - | -2.25086 | <0.00001 | down |
| ENSMUSG00000051427 | Ccdc157 | -1.13214 | <0.00001 | down |
| ENSMUSG00000036438 | Calm2 | 1.254385 | <0.00001 | up |
| ENSMUSG00000030612 | Mrpl46 | 1.53318 | <0.00001 | up |
| ENSMUSG00000055240 | Zfp101 | -1.4858 | <0.00001 | down |
| ENSMUSG00000064220 | H2ac18 | 3.334177 | <0.00001 | up |
| ENSMUSG00000023960 | Enpp5 | 1.125934 | <0.00001 | up |
| ENSMUSG00000054728 | Phactr1 | -2.86864 | <0.00001 | down |
| ENSMUSG00000025934 | Gsta3 | 1.412016 | <0.00001 | up |
| ENSMUSG00000027931 | Npr1 | -1.35726 | <0.00001 | down |
| ENSMUSG00000028617 | Lrrc42 | 1.788654 | <0.00001 | up |
| ENSMUSG00000060441 | Trim5 | -2.48593 | <0.00001 | down |
| ENSMUSG00000043384 | Gprasp1 | -1.69765 | <0.00001 | down |
| ENSMUSG00000002107 | Celf2 | -1.67728 | <0.00001 | down |
| ENSMUSG00000096929 | A330023F24Rik | -1.997 | <0.00001 | down |
| ENSMUSG00000024292 | Cyp4f14 | 1.30858 | <0.00001 | up |
| ENSMUSG00000033253 | Szt2 | -1.33284 | <0.00001 | down |
| ENSMUSG00000058569 | Tmed9 | 1.119211 | <0.00001 | up |
| ENSMUSG00000059772 | Slx1b | -1.33894 | <0.00001 | down |
| ENSMUSG00000051671 | Coa6 | 1.190031 | <0.00001 | up |
| ENSMUSG00000009733 | Tfcp2 | -1.51779 | <0.00001 | down |
| ENSMUSG00000020486 | Septin4 | -1.28179 | <0.00001 | down |
| ENSMUSG00000095928 | Olfr204 | -2.46261 | <0.00001 | down |
| ENSMUSG00000021819 | Zswim8 | -1.09784 | <0.00001 | down |
| ENSMUSG00000024085 | Man2a1 | 1.054268 | <0.00001 | up |
| ENSMUSG00000057132 | Rpgrip1 | -1.23547 | <0.00001 | down |
| ENSMUSG00000090077 | Lime1 | -1.95182 | <0.00001 | down |
| ENSMUSG00000046982 | Tshz1 | -1.72591 | <0.00001 | down |
| ENSMUSG00000027365 | Trpm7 | -1.20453 | <0.00001 | down |
| ENSMUSG00000106702 | Gm42846 | -2.14735 | <0.00001 | down |
| ENSMUSG00000109196 | Gm44715 | -2.28935 | <0.00001 | down |
| ENSMUSG00000036281 | Snapc4 | -1.57311 | <0.00001 | down |
| ENSMUSG00000029344 | Tpst2 | 1.146327 | <0.00001 | up |
| ENSMUSG00000022351 | Sqle | 2.149659 | <0.00001 | up |
| ENSMUSG00000020580 | Rock2 | -1.27662 | <0.00001 | down |
| ENSMUSG00000020863 | Luc7l3 | -1.25238 | <0.00001 | down |
| ENSMUSG00000105373 | Gm42429 | -1.01668 | <0.00001 | down |
| ENSMUSG00000026987 | Baz2b | -1.26083 | <0.00001 | down |
| ENSMUSG00000025237 | Parp6 | -1.20447 | <0.00001 | down |
| ENSMUSG00000045948 | Mrps12 | 1.559254 | <0.00001 | up |
| ENSMUSG00000035202 | Lars2 | -2.07214 | <0.00001 | down |
| ENSMUSG00000107276 | Gm42858 | -3.51068 | <0.00001 | down |
| ENSMUSG00000025470 | Zfp511 | -1.23725 | <0.00001 | down |
| ENSMUSG00000026368 | F13b | 1.519642 | <0.00001 | up |
| ENSMUSG00000070605 | Zfp992 | -1.71338 | <0.00001 | down |
| ENSMUSG00000078937 | Cpt1b | -3.36785 | <0.00001 | down |
| ENSMUSG00000024421 | Lama3 | -1.69768 | <0.00001 | down |
| ENSMUSG00000117912 | Gm50383 | -6.59832 | <0.00001 | down |
| ENSMUSG00000032092 | Mpzl2 | 1.146671 | <0.00001 | up |
| ENSMUSG00000050332 | Amer1 | -2.28149 | <0.00001 | down |
| ENSMUSG00000010097 | Nxf1 | -1.51746 | <0.00001 | down |
| ENSMUSG00000047631 | Apof | 1.383584 | <0.00001 | up |
| ENSMUSG00000052562 | Slc22a30 | -1.23421 | <0.00001 | down |
| ENSMUSG00000071076 | Jund | 1.834539 | <0.00001 | up |
| ENSMUSG00000115837 | 9130002K18Rik | -2.75755 | <0.00001 | down |
| ENSMUSG00000054702 | Ap1s3 | -3.19643 | <0.00001 | down |
| ENSMUSG00000006395 | Hyi | -1.72715 | <0.00001 | down |
| ENSMUSG00000027074 | Slc43a3 | -1.10059 | <0.00001 | down |
| ENSMUSG00000068263 | Efcc1 | -2.35864 | <0.00001 | down |
| ENSMUSG00000019734 | Tmc4 | -2.24775 | <0.00001 | down |
| ENSMUSG00000036275 | 9530068E07Rik | 1.087889 | <0.00001 | up |
| ENSMUSG00000029817 | Tra2a | -1.21348 | <0.00001 | down |
| ENSMUSG00000027829 | Ccnl1 | -1.22951 | <0.00001 | down |
| ENSMUSG00000049600 | Zbtb45 | -2.34269 | <0.00001 | down |
| ENSMUSG00000095687 | Rnaset2a | 2.44835 | <0.00001 | up |
| ENSMUSG00000021025 | Nfkbia | -1.45363 | <0.00001 | down |
| ENSMUSG00000022559 | Fbxl6 | -1.26366 | <0.00001 | down |
| ENSMUSG00000027313 | Chac1 | 2.659561 | <0.00001 | up |
| ENSMUSG00000027620 | Rbm39 | -1.00556 | <0.00001 | down |
| ENSMUSG00000026970 | Rbms1 | -2.18391 | <0.00001 | down |
| ENSMUSG00000026208 | Des | -1.47041 | <0.00001 | down |
| ENSMUSG00000083306 | Gm13868 | -5.6869 | <0.00001 | down |
| ENSMUSG00000074457 | S100a16 | 1.294357 | <0.00001 | up |
| ENSMUSG00000028479 | Gne | 1.24839 | <0.00001 | up |
| ENSMUSG00000094103 | Fam177a2 | -8.95026 | <0.00001 | down |
| ENSMUSG00000033545 | Znrf1 | -1.6866 | <0.00001 | down |
| ENSMUSG00000029062 | Cdk11b | -1.23259 | <0.00001 | down |
| ENSMUSG00000116590 | Gm53028 | -3.88346 | <0.00001 | down |
| ENSMUSG00000027583 | Zbtb46 | -1.83858 | <0.00001 | down |
| ENSMUSG00000026248 | Mrpl44 | 1.551209 | <0.00001 | up |
| ENSMUSG00000113159 | Gm48771 | -3.13774 | <0.00001 | down |
| ENSMUSG00000017286 | Glod4 | 1.695276 | <0.00001 | up |
| ENSMUSG00000005899 | Smpd4 | -1.15563 | <0.00001 | down |
| ENSMUSG00000048076 | Arf1 | 1.084136 | <0.00001 | up |
| ENSMUSG00000113889 | Gm48501 | -1.76638 | <0.00001 | down |
| ENSMUSG00000111361 | Gm47445 | -3.68304 | <0.00001 | down |
| ENSMUSG00000102326 | Gm37788 | -2.64258 | <0.00001 | down |
| ENSMUSG00000038895 | Zfp653 | -2.02154 | <0.00001 | down |
| ENSMUSG00000053580 | Tanc2 | -1.69013 | <0.00001 | down |
| ENSMUSG00000028469 | Npr2 | -1.42932 | <0.00001 | down |
| ENSMUSG00000039117 | Taf4 | -1.11769 | <0.00001 | down |
| ENSMUSG00000063931 | Pepd | 1.342013 | <0.00001 | up |
| ENSMUSG00000035637 | Grhpr | 1.212441 | <0.00001 | up |
| ENSMUSG00000024830 | Rps6kb2 | -1.29974 | <0.00001 | down |
| ENSMUSG00000111928 | Gm48082 | -1.85198 | <0.00001 | down |
| ENSMUSG00000105691 | Gm42876 | -3.08579 | <0.00001 | down |
| ENSMUSG00000025507 | Pidd1 | -1.86785 | <0.00001 | down |
| ENSMUSG00000061894 | Zscan20 | -1.66716 | <0.00001 | down |
| ENSMUSG00000039542 | Ncam1 | -3.44552 | <0.00001 | down |
| ENSMUSG00000092526 | Gm17907 | -4.05069 | <0.00001 | down |
| ENSMUSG00000042606 | Hirip3 | -1.56382 | <0.00001 | down |
| ENSMUSG00000079563 | Pglyrp2 | 1.251317 | <0.00001 | up |
| ENSMUSG00000022228 | Zscan26 | -1.23444 | <0.00001 | down |
| ENSMUSG00000035769 | Xylb | -1.19291 | <0.00001 | down |
| ENSMUSG00000038705 | Gmeb2 | -1.18664 | <0.00001 | down |
| ENSMUSG00000029650 | Slc46a3 | 1.327145 | <0.00001 | up |
| ENSMUSG00000037965 | Zc3h7a | -1.33116 | <0.00001 | down |
| ENSMUSG00000034912 | Mdga2 | -4.4008 | <0.00001 | down |
| ENSMUSG00000037475 | Thoc2 | -1.24904 | <0.00001 | down |
| ENSMUSG00000106446 | Gm42970 | -4.83809 | <0.00001 | down |
| ENSMUSG00000020869 | Lrrc59 | 1.411248 | <0.00001 | up |
| ENSMUSG00000020402 | Vdac1 | 1.063536 | <0.00001 | up |
| ENSMUSG00000000346 | Dazap2 | 1.148683 | <0.00001 | up |
| ENSMUSG00000025377 | Tepsin | -1.42732 | <0.00001 | down |
| ENSMUSG00000021917 | Spcs1 | 2.250647 | <0.00001 | up |
| ENSMUSG00000104867 | Gm43728 | -6.63499 | <0.00001 | down |
| ENSMUSG00000038738 | Shank1 | -3.59933 | <0.00001 | down |
| ENSMUSG00000111544 | 4930534H03Rik | -3.24471 | <0.00001 | down |
| ENSMUSG00000037519 | Ppfia1 | -1.01419 | <0.00001 | down |
| ENSMUSG00000030814 | Bcl7c | -1.65597 | <0.00001 | down |
| ENSMUSG00000001415 | Smg5 | -1.11096 | <0.00001 | down |
| ENSMUSG00000061589 | Dot1l | -1.27233 | <0.00001 | down |
| ENSMUSG00000062822 | 4833420G17Rik | -1.39699 | <0.00001 | down |
| ENSMUSG00000070343 | Gm10288 | -10.4776 | <0.00001 | down |
| ENSMUSG00000048280 | Zfp738 | -2.24541 | <0.00001 | down |
| ENSMUSG00000029553 | Tfec | -2.09261 | <0.00001 | down |
| ENSMUSG00000022500 | Litaf | 1.294611 | <0.00001 | up |
| ENSMUSG00000103672 | Gm37621 | -2.17013 | <0.00001 | down |
| ENSMUSG00000057098 | Ebf1 | -2.71132 | <0.00001 | down |
| ENSMUSG00000095041 | - | -1.64604 | <0.00001 | down |
| ENSMUSG00000022508 | Bcl6 | -1.98316 | <0.00001 | down |
| ENSMUSG00000046312 | Myorg | 1.127192 | <0.00001 | up |
| ENSMUSG00000044950 | Pwwp2a | -1.836 | <0.00001 | down |
| ENSMUSG00000106526 | Gm42604 | 2.060614 | <0.00001 | up |
| ENSMUSG00000073758 | Sh3d21 | -2.09728 | <0.00001 | down |
| ENSMUSG00000001911 | Nfix | 1.003105 | <0.00001 | up |
| ENSMUSG00000032388 | Spg21 | 1.146287 | <0.00001 | up |
| ENSMUSG00000104291 | A130071D04Rik | -3.12024 | <0.00001 | down |
| ENSMUSG00000024165 | Jpt2 | 1.505628 | <0.00001 | up |
| ENSMUSG00000041355 | Ssr2 | 1.402488 | <0.00001 | up |
| ENSMUSG00000026933 | Camsap1 | -1.6545 | <0.00001 | down |
| ENSMUSG00000024378 | Stard4 | 1.125217 | <0.00001 | up |
| ENSMUSG00000035967 | Ints6l | -2.00393 | <0.00001 | down |
| ENSMUSG00000025479 | Cyp2e1 | 1.819111 | <0.00001 | up |
| ENSMUSG00000053644 | Aldh7a1 | 1.137853 | <0.00001 | up |
| ENSMUSG00000106874 | Gm20186 | -1.33102 | <0.00001 | down |
| ENSMUSG00000086844 | B230206H07Rik | -2.92725 | <0.00001 | down |
| ENSMUSG00000025381 | Cnpy2 | 1.065215 | <0.00001 | up |
| ENSMUSG00000104046 | Gm37567 | -1.67036 | <0.00001 | down |
| ENSMUSG00000040829 | Zmynd15 | -1.95197 | <0.00001 | down |
| ENSMUSG00000109881 | Gm45507 | -10.7879 | <0.00001 | down |
| ENSMUSG00000061559 | Wdr61 | 1.256877 | <0.00001 | up |
| ENSMUSG00000033439 | Trmt13 | -1.51198 | <0.00001 | down |
| ENSMUSG00000086825 | Gm15675 | -2.18796 | <0.00001 | down |
| ENSMUSG00000045466 | Zfp956 | -1.74604 | <0.00001 | down |
| ENSMUSG00000062646 | Ganc | -1.22998 | <0.00001 | down |
| ENSMUSG00000021431 | Snrnp48 | -1.22909 | <0.00001 | down |
| ENSMUSG00000075028 | Prdm11 | -2.53296 | <0.00001 | down |
| ENSMUSG00000039086 | Ss18l1 | -1.75027 | <0.00001 | down |
| ENSMUSG00000079084 | Ccdc82 | -1.14732 | <0.00001 | down |
| ENSMUSG00000112226 | Gm48786 | -2.57892 | <0.00001 | down |
| ENSMUSG00000009549 | Srp14 | 1.264476 | <0.00001 | up |
| ENSMUSG00000014418 | Hps5 | -1.40495 | <0.00001 | down |
| ENSMUSG00000006740 | Kif5b | -1.02901 | <0.00001 | down |
| ENSMUSG00000027879 | Sec22b | 1.261141 | <0.00001 | up |
| ENSMUSG00000019731 | Slc35e1 | 1.174048 | <0.00001 | up |
| ENSMUSG00000041096 | Tspyl2 | -1.65439 | <0.00001 | down |
| ENSMUSG00000029389 | Ddx55 | -1.53692 | <0.00001 | down |
| ENSMUSG00000067144 | Slc22a7 | 1.643154 | <0.00001 | up |
| ENSMUSG00000021687 | Scamp1 | 1.172499 | <0.00001 | up |
| ENSMUSG00000054889 | Dsp | 1.004503 | <0.00001 | up |
| ENSMUSG00000031450 | Grk1 | -4.03846 | <0.00001 | down |
| ENSMUSG00000030924 | Rexo5 | -2.18879 | <0.00001 | down |
| ENSMUSG00000074207 | Adh1 | 1.347375 | <0.00001 | up |
| ENSMUSG00000094520 | Olfr635 | -2.7814 | <0.00001 | down |
| ENSMUSG00000089715 | Cbx6 | -1.63021 | <0.00001 | down |
| ENSMUSG00000103318 | Gm38356 | -1.75207 | <0.00001 | down |
| ENSMUSG00000052738 | Suclg1 | 1.809752 | <0.00001 | up |
| ENSMUSG00000079426 | Arpc4 | 1.152354 | <0.00001 | up |
| ENSMUSG00000024012 | Mtch1 | 1.16043 | <0.00001 | up |
| ENSMUSG00000020829 | Slc46a1 | 1.448332 | <0.00001 | up |
| ENSMUSG00000037679 | Inf2 | 1.159304 | <0.00001 | up |
| ENSMUSG00000050994 | Adgb | -2.67823 | <0.00001 | down |
| ENSMUSG00000024436 | Mrps18b | 1.09898 | <0.00001 | up |
| ENSMUSG00000036896 | C1qc | 1.116238 | <0.00001 | up |
| ENSMUSG00000025393 | Atp5b | 1.474926 | <0.00001 | up |
| ENSMUSG00000091382 | Vmn1r18 | -2.65161 | <0.00001 | down |
| ENSMUSG00000058318 | Phf21a | -1.2579 | <0.00001 | down |
| ENSMUSG00000109089 | 4833411C07Rik | -2.20085 | <0.00001 | down |
| ENSMUSG00000047228 | A2ml1 | 1.509441 | <0.00001 | up |
| ENSMUSG00000070315 | 4930581F22Rik | -1.70174 | <0.00001 | down |
| ENSMUSG00000066760 | Psg16 | -1.45685 | <0.00001 | down |
| ENSMUSG00000028849 | Map7d1 | 1.034624 | <0.00001 | up |
| ENSMUSG00000026887 | Mrrf | 1.092246 | <0.00001 | up |
| ENSMUSG00000031845 | Bco1 | -2.02367 | <0.00001 | down |
| ENSMUSG00000024006 | Stk38 | -1.12461 | <0.00001 | down |
| ENSMUSG00000037254 | Itih2 | 1.389657 | <0.00001 | up |
| ENSMUSG00000091076 | Vmn2r115 | -3.00853 | <0.00001 | down |
| ENSMUSG00000097464 | Gm26736 | -3.66117 | <0.00001 | down |
| ENSMUSG00000121137 | - | -1.32183 | <0.00001 | down |
| ENSMUSG00000035960 | Apex1 | 1.296507 | <0.00001 | up |
| ENSMUSG00000030313 | Dennd5b | -1.32932 | <0.00001 | down |
| ENSMUSG00000000301 | Pemt | 1.534037 | <0.00001 | up |
| ENSMUSG00000118506 | Cfap141 | -2.9185 | <0.00001 | down |
| ENSMUSG00000031167 | Rbm3 | 1.182554 | <0.00001 | up |
| ENSMUSG00000070934 | Rraga | 1.703177 | <0.00001 | up |
| ENSMUSG00000025050 | Pcgf6 | -1.30424 | <0.00001 | down |
| ENSMUSG00000070493 | Chchd2 | 1.383632 | <0.00001 | up |
| ENSMUSG00000022437 | Samm50 | 1.259184 | <0.00001 | up |
| ENSMUSG00000040054 | Baz2a | -1.26044 | <0.00001 | down |
| ENSMUSG00000000555 | Itga5 | -1.21883 | <0.00001 | down |
| ENSMUSG00000026427 | Eif2d | 1.006219 | <0.00001 | up |
| ENSMUSG00000035597 | Prpf39 | -1.52921 | <0.00001 | down |
| ENSMUSG00000028419 | Chmp5 | 1.580818 | <0.00001 | up |
| ENSMUSG00000039671 | Zmynd8 | -1.23528 | <0.00001 | down |
| ENSMUSG00000038745 | Nlrp6 | -1.73154 | <0.00001 | down |
| ENSMUSG00000115708 | Gm49187 | -1.61925 | <0.00001 | down |
| ENSMUSG00000083563 | Gm13340 | -3.43375 | <0.00001 | down |
| ENSMUSG00000007739 | Cct4 | 1.102611 | <0.00001 | up |
| ENSMUSG00000029442 | Wdr66 | -2.27139 | <0.00001 | down |
| ENSMUSG00000068011 | Mkrn2os | 1.534472 | <0.00001 | up |
| ENSMUSG00000046567 | 4930430F08Rik | -1.50387 | <0.00001 | down |
| ENSMUSG00000020592 | Sdc1 | 1.67063 | <0.00001 | up |
| ENSMUSG00000052921 | Arhgef15 | -1.74331 | <0.00001 | down |
| ENSMUSG00000090655 | Vmn2r120 | -3.77846 | <0.00001 | down |
| ENSMUSG00000026043 | Col3a1 | -1.31374 | <0.00001 | down |
| ENSMUSG00000005779 | Psmb4 | 1.191878 | <0.00001 | up |
| ENSMUSG00000027405 | Nop56 | -1.47969 | <0.00001 | down |
| ENSMUSG00000030881 | Arfip2 | -1.12878 | <0.00001 | down |
| ENSMUSG00000028398 | Dmac1 | 1.488219 | <0.00001 | up |
| ENSMUSG00000029246 | Ppat | 1.347875 | <0.00001 | up |
| ENSMUSG00000062825 | Actg1 | 1.740264 | <0.00001 | up |
| ENSMUSG00000040274 | Cdk6 | -2.24404 | <0.00001 | down |
| ENSMUSG00000037580 | Gch1 | 1.600685 | <0.00001 | up |
| ENSMUSG00000020198 | Ap3d1 | -1.14467 | <0.00001 | down |
| ENSMUSG00000036632 | Alg5 | 1.019304 | <0.00001 | up |
| ENSMUSG00000036120 | Rfxank | -1.35332 | <0.00001 | down |
| ENSMUSG00000108803 | 4930533N22Rik | -1.56194 | <0.00001 | down |
| ENSMUSG00000038884 | Shfl | -1.78643 | <0.00001 | down |
| ENSMUSG00000042043 | Tbca | 1.422601 | <0.00001 | up |
| ENSMUSG00000029599 | Ddx54 | 1.419742 | <0.00001 | up |
| ENSMUSG00000121179 | - | -1.64537 | <0.00001 | down |
| ENSMUSG00000052512 | Nav2 | -1.93886 | <0.00001 | down |
| ENSMUSG00000078193 | Gm2000 | -9.19532 | <0.00001 | down |
| ENSMUSG00000026463 | Atp2b4 | -1.79356 | <0.00001 | down |
| ENSMUSG00000023010 | Tmbim6 | 1.360767 | <0.00001 | up |
| ENSMUSG00000068950 | Olfr338 | -2.10454 | <0.00001 | down |
| ENSMUSG00000029071 | Dvl1 | -1.4504 | <0.00001 | down |
| ENSMUSG00000068566 | Myadm | 1.11029 | <0.00001 | up |
| ENSMUSG00000045409 | Trim39 | -1.62742 | <0.00001 | down |
| ENSMUSG00000101609 | Kcnq1ot1 | -2.52476 | <0.00001 | down |
| ENSMUSG00000008035 | Mid1ip1 | 1.986372 | <0.00001 | up |
| ENSMUSG00000039202 | Abhd2 | 1.637304 | <0.00001 | up |
| ENSMUSG00000028798 | Eif3i | 1.293928 | <0.00001 | up |
| ENSMUSG00000029647 | Pan3 | -1.48876 | <0.00001 | down |
| ENSMUSG00000051790 | Nlgn2 | -2.18827 | <0.00001 | down |
| ENSMUSG00000032679 | Cd59a | -1.84511 | <0.00001 | down |
| ENSMUSG00000026273 | Mterf4 | -1.35401 | <0.00001 | down |
| ENSMUSG00000021537 | Cetn3 | 1.615708 | <0.00001 | up |
| ENSMUSG00000040904 | Gm21988 | 2.048329 | <0.00001 | up |
| ENSMUSG00000031782 | Coq9 | 1.127677 | <0.00001 | up |
| ENSMUSG00000057894 | Zfp329 | -1.9026 | <0.00001 | down |
| ENSMUSG00000040936 | Ulk4 | -4.78076 | <0.00001 | down |
| ENSMUSG00000068245 | Phf11d | -2.02733 | <0.00001 | down |
| ENSMUSG00000029499 | Pxmp2 | 1.642339 | <0.00001 | up |
| ENSMUSG00000025137 | Pcyt2 | -1.18503 | <0.00001 | down |
| ENSMUSG00000039233 | Tbce | -1.45011 | <0.00001 | down |
| ENSMUSG00000116238 | Gm49413 | -4.72264 | <0.00001 | down |
| ENSMUSG00000037234 | Hook3 | -1.11425 | <0.00001 | down |
| ENSMUSG00000023089 | Ndufa5 | -1.05067 | <0.00001 | down |
| ENSMUSG00000034459 | Ifit1 | -2.54272 | <0.00001 | down |
| ENSMUSG00000036292 | Gramd1c | -1.0029 | <0.00001 | down |
| ENSMUSG00000040564 | Apoc1 | -1.05778 | <0.00001 | down |
| ENSMUSG00000030382 | Slc27a5 | 1.290089 | <0.00001 | up |
| ENSMUSG00000098014 | Gm26967 | -2.13244 | <0.00001 | down |
| ENSMUSG00000055216 | 9430025C20Rik | -3.26504 | <0.00001 | down |
| ENSMUSG00000108092 | Gm44189 | -3.25544 | <0.00001 | down |
| ENSMUSG00000021190 | Lgmn | 1.13929 | <0.00001 | up |
| ENSMUSG00000079550 | Mpp4 | -1.64302 | <0.00001 | down |
| ENSMUSG00000112639 | A730063M14Rik | -2.31437 | <0.00001 | down |
| ENSMUSG00000025198 | Erlin1 | 1.374581 | <0.00001 | up |
| ENSMUSG00000108158 | Gm44002 | 6.5475 | <0.00001 | up |
| ENSMUSG00000082127 | Gm13577 | -6.87109 | <0.00001 | down |
| ENSMUSG00000039804 | Ncoa5 | -1.57619 | <0.00001 | down |
| ENSMUSG00000015597 | Zfp318 | -1.06947 | <0.00001 | down |
| ENSMUSG00000037710 | Cisd1 | 1.432329 | <0.00001 | up |
| ENSMUSG00000113019 | Gm47467 | -1.39537 | <0.00001 | down |
| ENSMUSG00000025004 | Cyp2c40 | -2.19485 | <0.00001 | down |
| ENSMUSG00000027523 | Gnas | -1.14641 | <0.00001 | down |
| ENSMUSG00000054545 | Ugt1a6a | 2.320163 | <0.00001 | up |
| ENSMUSG00000102153 | Gm37474 | -4.01787 | <0.00001 | down |
| ENSMUSG00000040385 | Ppp1ca | 1.278421 | <0.00001 | up |
| ENSMUSG00000053094 | Tmem248 | 1.235453 | <0.00001 | up |
| ENSMUSG00000071647 | Eml3 | -1.09317 | <0.00001 | down |
| ENSMUSG00000052707 | Tnrc6a | -2.21199 | <0.00001 | down |
| ENSMUSG00000074219 | Gm10644 | -2.01653 | <0.00001 | down |
| ENSMUSG00000032177 | Pde4a | -2.08332 | <0.00001 | down |
| ENSMUSG00000055204 | Ankrd17 | -1.38367 | <0.00001 | down |
| ENSMUSG00000099034 | 2810039B14Rik | -1.73307 | <0.00001 | down |
| ENSMUSG00000025421 | Hdhd2 | 1.40815 | <0.00001 | up |
| ENSMUSG00000037674 | Rfx7 | -1.17265 | <0.00001 | down |
| ENSMUSG00000062382 | Ftl1-ps1 | -1.56926 | <0.00001 | down |
| ENSMUSG00000019907 | Ppp1r12a | -1.04835 | <0.00001 | down |
| ENSMUSG00000017493 | Igfbp4 | 1.550764 | <0.00001 | up |
| ENSMUSG00000102275 | Gm37144 | -3.22561 | <0.00001 | down |
| ENSMUSG00000024197 | Plin3 | 1.23085 | <0.00001 | up |
| ENSMUSG00000025790 | Slco3a1 | -2.16462 | <0.00001 | down |
| ENSMUSG00000027942 | 4933434E20Rik | 1.540199 | <0.00001 | up |
| ENSMUSG00000030127 | Cops7a | 1.137315 | <0.00001 | up |
| ENSMUSG00000062270 | Morf4l1 | 1.395716 | <0.00001 | up |
| ENSMUSG00000113427 | Gm46378 | -3.28975 | <0.00001 | down |
| ENSMUSG00000025607 | Copg2 | 1.174053 | <0.00001 | up |
| ENSMUSG00000097911 | Gm26691 | -3.50853 | <0.00001 | down |
| ENSMUSG00000021835 | Bmp4 | -2.08209 | <0.00001 | down |
| ENSMUSG00000050390 | C77080 | 1.132009 | <0.00001 | up |
| ENSMUSG00000020053 | Igf1 | -1.53798 | <0.00001 | down |
| ENSMUSG00000040488 | Ltbp4 | -1.09125 | <0.00001 | down |
| ENSMUSG00000097893 | 1700034P13Rik | -2.32152 | <0.00001 | down |
| ENSMUSG00000048938 | Nr1h5 | -2.33082 | <0.00001 | down |
| ENSMUSG00000023829 | Slc22a1 | 1.312109 | <0.00001 | up |
| ENSMUSG00000002625 | Akap8l | -2.12341 | <0.00001 | down |
| ENSMUSG00000031906 | Smpd3 | 3.105385 | <0.00001 | up |
| ENSMUSG00000035561 | Aldh1b1 | 2.044011 | <0.00001 | up |
| ENSMUSG00000011148 | Adssl1 | 1.515997 | <0.00001 | up |
| ENSMUSG00000029642 | Polr1d | 1.153605 | <0.00001 | up |
| ENSMUSG00000031068 | Glrx3 | 1.066343 | <0.00001 | up |
| ENSMUSG00000037364 | Srrt | -1.30409 | <0.00001 | down |
| ENSMUSG00000051166 | Eml5 | -2.80992 | <0.00001 | down |
| ENSMUSG00000028073 | Pear1 | -1.66549 | <0.00001 | down |
| ENSMUSG00000022708 | Zbtb20 | -2.33737 | <0.00001 | down |
| ENSMUSG00000039617 | Gm7488 | 6.426567 | <0.00001 | up |
| ENSMUSG00000026048 | Ercc5 | -1.20476 | <0.00001 | down |
| ENSMUSG00000015467 | Egfl8 | -2.88797 | <0.00001 | down |
| ENSMUSG00000036572 | Upf3b | -1.43731 | <0.00001 | down |
| ENSMUSG00000025355 | Mmp19 | 1.649578 | <0.00001 | up |
| ENSMUSG00000066150 | Slc31a1 | 1.191322 | <0.00001 | up |
| ENSMUSG00000032845 | Alpk2 | -3.03292 | <0.00001 | down |
| ENSMUSG00000102562 | Gm37694 | -5.56293 | <0.00001 | down |
| ENSMUSG00000090778 | Gm3235 | -3.83784 | <0.00001 | down |
| ENSMUSG00000054808 | Actn4 | -2.23122 | <0.00001 | down |
| ENSMUSG00000034429 | Zfp707 | -1.27887 | <0.00001 | down |
| ENSMUSG00000024997 | Prdx3 | 1.261262 | <0.00001 | up |
| ENSMUSG00000031443 | F7 | 1.264718 | <0.00001 | up |
| ENSMUSG00000021928 | Ebpl | 1.424777 | <0.00001 | up |
| ENSMUSG00000028086 | Fbxw7 | -1.86952 | <0.00001 | down |
| ENSMUSG00000001750 | Tcirg1 | -1.1359 | <0.00001 | down |
| ENSMUSG00000038224 | Serpinf2 | 1.154647 | <0.00001 | up |
| ENSMUSG00000032815 | Fanca | -3.21149 | <0.00001 | down |
| ENSMUSG00000020364 | Zfp354a | -2.13275 | <0.00001 | down |
| ENSMUSG00000022610 | Mapk12 | -1.91262 | <0.00001 | down |
| ENSMUSG00000044600 | Smim7 | 1.164 | <0.00001 | up |
| ENSMUSG00000053985 | Zfp14 | -2.33987 | <0.00001 | down |
| ENSMUSG00000049858 | Suox | 1.60103 | <0.00001 | up |
| ENSMUSG00000006390 | Elovl1 | 1.048571 | <0.00001 | up |
| ENSMUSG00000037278 | Tmem97 | 1.809074 | <0.00001 | up |
| ENSMUSG00000026568 | Mpc2 | 1.535928 | <0.00001 | up |
| ENSMUSG00000027593 | Raly | 1.107847 | <0.00001 | up |
| ENSMUSG00000024109 | Nrxn1 | -2.41607 | <0.00001 | down |
| ENSMUSG00000038872 | Zfhx3 | -2.29973 | <0.00001 | down |
| ENSMUSG00000035875 | AI182371 | 1.341076 | <0.00001 | up |
| ENSMUSG00000026083 | Eif5b | -2.17659 | <0.00001 | down |
| ENSMUSG00000021124 | Vti1b | 1.425837 | <0.00001 | up |
| ENSMUSG00000103711 | Tstd1 | 1.379496 | <0.00001 | up |
| ENSMUSG00000015224 | Cyp2j9 | -2.22473 | <0.00001 | down |
| ENSMUSG00000000563 | Atp5pb | 1.687111 | <0.00001 | up |
| ENSMUSG00000005682 | Pan2 | -1.72619 | <0.00001 | down |
| ENSMUSG00000038009 | Dnajc22 | 1.360417 | <0.00001 | up |
| ENSMUSG00000021361 | Tmem14c | 1.626615 | <0.00001 | up |
| ENSMUSG00000033004 | Mycbp2 | -1.51158 | <0.00001 | down |
| ENSMUSG00000079003 | Samd1 | -1.78144 | <0.00001 | down |
| ENSMUSG00000033499 | Larp4b | -1.89651 | <0.00001 | down |
| ENSMUSG00000050240 | Hic2 | -2.30058 | <0.00001 | down |
| ENSMUSG00000038213 | Tapbpl | 1.325641 | <0.00001 | up |
| ENSMUSG00000007850 | Hnrnph1 | -1.09859 | <0.00001 | down |
| ENSMUSG00000025571 | Tnrc6c | -1.90152 | <0.00001 | down |
| ENSMUSG00000015806 | Qdpr | 1.687477 | <0.00001 | up |
| ENSMUSG00000041084 | Ostc | 1.904735 | <0.00001 | up |
| ENSMUSG00000020668 | Kif3c | -2.22932 | <0.00001 | down |
| ENSMUSG00000042308 | Setd1a | -1.69793 | <0.00001 | down |
| ENSMUSG00000021815 | Mss51 | -3.47367 | <0.00001 | down |
| ENSMUSG00000032399 | Rpl4 | 1.383679 | <0.00001 | up |
| ENSMUSG00000029727 | Cyp3a13 | 2.017754 | <0.00001 | up |
| ENSMUSG00000030541 | Idh2 | 1.546109 | <0.00001 | up |
| ENSMUSG00000032497 | Lrrfip2 | -1.35585 | <0.00001 | down |
| ENSMUSG00000028601 | Echdc2 | -1.69359 | <0.00001 | down |
| ENSMUSG00000022766 | Serpind1 | 1.297632 | <0.00001 | up |
| ENSMUSG00000027318 | Adam33 | -2.95058 | <0.00001 | down |
| ENSMUSG00000006304 | Arpc2 | 1.049604 | <0.00001 | up |
| ENSMUSG00000112794 | Gm48878 | -1.70737 | <0.00001 | down |
| ENSMUSG00000025504 | Eps8l2 | -1.2477 | <0.00001 | down |
| ENSMUSG00000020321 | Mdh1 | 1.783202 | <0.00001 | up |
| ENSMUSG00000066263 | Olfr639 | -2.29713 | <0.00001 | down |
| ENSMUSG00000019806 | Aig1 | 1.899643 | <0.00001 | up |
| ENSMUSG00000022706 | Mrpl40 | 1.113028 | <0.00001 | up |
| ENSMUSG00000037007 | Zfp113 | -1.95635 | <0.00001 | down |
| ENSMUSG00000029648 | Flt1 | -1.87492 | <0.00001 | down |
| ENSMUSG00000022110 | Sucla2 | 1.365957 | <0.00001 | up |
| ENSMUSG00000046210 | Olfr735 | -3.38711 | <0.00001 | down |
| ENSMUSG00000021492 | F12 | 1.037882 | <0.00001 | up |
| ENSMUSG00000090236 | Car15 | -4.08219 | <0.00001 | down |
| ENSMUSG00000095597 | Rps7-ps3 | -11.7436 | <0.00001 | down |
| ENSMUSG00000030287 | Itpr2 | -1.82658 | <0.00001 | down |
| ENSMUSG00000024799 | Tm7sf2 | 1.376151 | <0.00001 | up |
| ENSMUSG00000068290 | Ddrgk1 | 1.213992 | <0.00001 | up |
| ENSMUSG00000013495 | Tmem175 | -1.67562 | <0.00001 | down |
| ENSMUSG00000051095 | Olfr986 | -2.15458 | <0.00001 | down |
| ENSMUSG00000024948 | Map4k2 | -1.62415 | <0.00001 | down |
| ENSMUSG00000115018 | Ndor1 | -2.0236 | <0.00001 | down |
| ENSMUSG00000022194 | Pabpn1 | -3.08908 | <0.00001 | down |
| ENSMUSG00000073643 | Wdfy1 | -1.79347 | <0.00001 | down |
| ENSMUSG00000056917 | Sipa1 | -1.57443 | <0.00001 | down |
| ENSMUSG00000103932 | Gm36963 | -4.95129 | <0.00001 | down |
| ENSMUSG00000120368 | - | -3.8852 | <0.00001 | down |
| ENSMUSG00000025893 | Kbtbd3 | -1.79375 | <0.00001 | down |
| ENSMUSG00000010663 | Fads1 | 1.640743 | <0.00001 | up |
| ENSMUSG00000022894 | Adamts5 | -2.11209 | <0.00001 | down |
| ENSMUSG00000034771 | Tle2 | -1.82814 | <0.00001 | down |
| ENSMUSG00000032601 | Prkar2a | -1.89176 | <0.00001 | down |
| ENSMUSG00000079614 | Seh1l | 1.001253 | <0.00001 | up |
| ENSMUSG00000052915 | Msl1 | -1.82289 | <0.00001 | down |
| ENSMUSG00000017756 | Slc12a7 | -1.23279 | <0.00001 | down |
| ENSMUSG00000112013 | Gm47967 | -3.43087 | <0.00001 | down |
| ENSMUSG00000043991 | Pura | -1.208 | <0.00001 | down |
| ENSMUSG00000019370 | Calm3 | 1.110951 | <0.00001 | up |
| ENSMUSG00000042286 | Stab1 | -1.57453 | <0.00001 | down |
| ENSMUSG00000002885 | Adgre5 | -1.37209 | <0.00001 | down |
| ENSMUSG00000024430 | Cabyr | -2.50134 | <0.00001 | down |
| ENSMUSG00000042472 | Zfp410 | -1.29304 | <0.00001 | down |
| ENSMUSG00000052534 | Pbx1 | -2.17895 | <0.00001 | down |
| ENSMUSG00000018340 | Anxa6 | 1.068482 | <0.00001 | up |
| ENSMUSG00000072770 | Acrbp | -3.06911 | <0.00001 | down |
| ENSMUSG00000026154 | Sdhaf4 | 1.637156 | <0.00001 | up |
| ENSMUSG00000029767 | Calu | 1.356115 | <0.00001 | up |
| ENSMUSG00000021716 | Srek1ip1 | -2.46093 | <0.00001 | down |
| ENSMUSG00000026111 | Unc50 | 2.01071 | <0.00001 | up |
| ENSMUSG00000022365 | Derl1 | 1.148432 | <0.00001 | up |
| ENSMUSG00000029993 | Nfu1 | 1.791117 | <0.00001 | up |
| ENSMUSG00000116450 | Gm49534 | -2.92036 | <0.00001 | down |
| ENSMUSG00000022961 | Son | -1.88778 | <0.00001 | down |
| ENSMUSG00000024174 | Pot1b | -1.60806 | <0.00001 | down |
| ENSMUSG00000066037 | Hnrnpr | -1.99469 | <0.00001 | down |
| ENSMUSG00000027712 | Anxa5 | 1.032763 | <0.00001 | up |
| ENSMUSG00000001942 | Siae | 1.060236 | <0.00001 | up |
| ENSMUSG00000002769 | Gnmt | 2.203686 | <0.00001 | up |
| ENSMUSG00000035021 | Baz1a | -1.96692 | <0.00001 | down |
| ENSMUSG00000113069 | Gm48541 | -2.44115 | <0.00001 | down |
| ENSMUSG00000027884 | Clcc1 | 1.291942 | <0.00001 | up |
| ENSMUSG00000004069 | Dnaja3 | 1.143975 | <0.00001 | up |
| ENSMUSG00000052794 | 1700030K09Rik | -1.90733 | <0.00001 | down |
| ENSMUSG00000004187 | Kifc2 | -2.31338 | <0.00001 | down |
| ENSMUSG00000061244 | Exoc5 | -1.53594 | <0.00001 | down |
| ENSMUSG00000039438 | Ttc36 | 1.276084 | <0.00001 | up |
| ENSMUSG00000108216 | Gm44153 | -4.09631 | <0.00001 | down |
| ENSMUSG00000024045 | Akap8 | -1.494 | <0.00001 | down |
| ENSMUSG00000115431 | Gm3219 | -1.72318 | <0.00001 | down |
| ENSMUSG00000038692 | Hoxb4 | -2.93418 | <0.00001 | down |
| ENSMUSG00000035851 | Ythdc1 | -1.17548 | <0.00001 | down |
| ENSMUSG00000028138 | Adh5 | 1.431794 | <0.00001 | up |
| ENSMUSG00000009292 | Trpm2 | -2.13443 | <0.00001 | down |
| ENSMUSG00000067274 | Rplp0 | 1.03195 | <0.00001 | up |
| ENSMUSG00000038074 | Fkbp14 | -1.62618 | <0.00001 | down |
| ENSMUSG00000029390 | Tmed2 | 1.214431 | <0.00001 | up |
| ENSMUSG00000037933 | Bicd2 | -1.38056 | <0.00001 | down |
| ENSMUSG00000037523 | Mavs | 1.173546 | <0.00001 | up |
| ENSMUSG00000062054 | Iah1 | 1.562323 | <0.00001 | up |
| ENSMUSG00000086141 | 9030622O22Rik | -1.50782 | <0.00001 | down |
| ENSMUSG00000027706 | Sec62 | -2.35068 | <0.00001 | down |
| ENSMUSG00000030591 | Psmd8 | 1.111751 | <0.00001 | up |
| ENSMUSG00000075010 | AW112010 | 1.894154 | <0.00001 | up |
| ENSMUSG00000086370 | Ftx | -2.16291 | <0.00001 | down |
| ENSMUSG00000024163 | Mapk8ip3 | -1.62625 | <0.00001 | down |
| ENSMUSG00000121283 | - | -2.41574 | <0.00001 | down |
| ENSMUSG00000078713 | Tomm5 | 1.398991 | <0.00001 | up |
| ENSMUSG00000000693 | Loxl3 | -2.15959 | <0.00001 | down |
| ENSMUSG00000021413 | Prpf4b | -1.22068 | <0.00001 | down |
| ENSMUSG00000024290 | Rock1 | -1.23329 | <0.00001 | down |
| ENSMUSG00000005107 | Slc2a9 | 1.219801 | <0.00001 | up |
| ENSMUSG00000020074 | Ccar1 | -1.41204 | <0.00001 | down |
| ENSMUSG00000030002 | Dusp11 | -1.17877 | <0.00001 | down |
| ENSMUSG00000072115 | Ang | 1.576789 | <0.00001 | up |
| ENSMUSG00000004207 | Psap | 1.212135 | <0.00001 | up |
| ENSMUSG00000109157 | Gm44829 | -4.19706 | <0.00001 | down |
| ENSMUSG00000057074 | Ces1g | 1.244177 | <0.00001 | up |
| ENSMUSG00000037190 | Cyb561d2 | 1.581196 | <0.00001 | up |
| ENSMUSG00000096822 | Olfr344 | -2.8878 | <0.00001 | down |
| ENSMUSG00000022844 | Pdia5 | 1.327167 | <0.00001 | up |
| ENSMUSG00000028013 | Ppa2 | 1.508709 | <0.00001 | up |
| ENSMUSG00000094724 | Rnaset2b | 1.333095 | <0.00001 | up |
| ENSMUSG00000025127 | Gcgr | -1.20559 | <0.00001 | down |
| ENSMUSG00000028565 | Nfia | -1.40381 | <0.00001 | down |
| ENSMUSG00000019494 | Cops6 | 1.837582 | <0.00001 | up |
| ENSMUSG00000062410 | Hsd3b3 | 1.033141 | <0.00001 | up |
| ENSMUSG00000016541 | Atxn10 | 1.521376 | <0.00001 | up |
| ENSMUSG00000029427 | Zcchc8 | -1.17184 | <0.00001 | down |
| ENSMUSG00000025428 | Atp5a1 | 1.449997 | <0.00001 | up |
| ENSMUSG00000022868 | Ahsg | 1.827151 | <0.00001 | up |
| ENSMUSG00000030067 | Foxp1 | -2.5139 | <0.00001 | down |
| ENSMUSG00000021615 | Xrcc4 | 2.043553 | <0.00001 | up |
| ENSMUSG00000057649 | Brd9 | -1.7433 | <0.00001 | down |
| ENSMUSG00000030674 | Qprt | 1.57607 | <0.00001 | up |
| ENSMUSG00000045983 | Eif4g1 | 1.182497 | <0.00001 | up |
| ENSMUSG00000032198 | Dock6 | -1.86727 | <0.00001 | down |
| ENSMUSG00000111403 | Gm47544 | -8.21173 | <0.00001 | down |
| ENSMUSG00000068874 | Selenbp1 | 1.133955 | <0.00001 | up |
| ENSMUSG00000018761 | Mpdu1 | 1.35708 | <0.00001 | up |
| ENSMUSG00000015750 | Aph1a | 1.178722 | <0.00001 | up |
| ENSMUSG00000010911 | Apip | 1.178756 | <0.00001 | up |
| ENSMUSG00000068923 | Syt11 | -2.4111 | <0.00001 | down |
| ENSMUSG00000021999 | Cpb2 | 1.971556 | <0.00001 | up |
| ENSMUSG00000032359 | Ctsh | 1.753203 | <0.00001 | up |
| ENSMUSG00000109461 | Gm44848 | -4.28305 | <0.00001 | down |
| ENSMUSG00000030088 | Aldh1l1 | 1.195403 | <0.00001 | up |
| ENSMUSG00000022323 | Rida | -1.11001 | <0.00001 | down |
| ENSMUSG00000016319 | Slc25a5 | 1.732802 | <0.00001 | up |
| ENSMUSG00000052031 | Tagap1 | -1.81068 | <0.00001 | down |
| ENSMUSG00000021211 | Akr1c12 | 1.436864 | <0.00001 | up |
| ENSMUSG00000076441 | Ass1 | 1.714154 | <0.00001 | up |
| ENSMUSG00000001380 | Hars | 1.297702 | <0.00001 | up |
| ENSMUSG00000025791 | Pgm1 | 1.425118 | <0.00001 | up |
| ENSMUSG00000071176 | Arhgef10 | -1.8278 | <0.00001 | down |
| ENSMUSG00000033237 | Arid2 | -1.39544 | <0.00001 | down |
| ENSMUSG00000041650 | Pcca | 1.750467 | <0.00001 | up |
| ENSMUSG00000066621 | Tecpr1 | -1.99393 | <0.00001 | down |
| ENSMUSG00000073838 | Tufm | 1.645578 | <0.00001 | up |
| ENSMUSG00000059146 | Ntrk3 | -3.88114 | <0.00001 | down |
| ENSMUSG00000117148 | Vmn1r229 | -2.0961 | <0.00001 | down |
| ENSMUSG00000027642 | Rpn2 | 1.213717 | <0.00001 | up |
| ENSMUSG00000058952 | Cfi | 1.623633 | <0.00001 | up |
| ENSMUSG00000041235 | Chd7 | -2.28715 | <0.00001 | down |
| ENSMUSG00000028822 | Tmem50a | 1.475597 | <0.00001 | up |
| ENSMUSG00000059974 | Ntm | -2.5315 | <0.00001 | down |
| ENSMUSG00000000171 | Sdhd | 1.490396 | <0.00001 | up |
| ENSMUSG00000031380 | Vegfd | -2.74311 | <0.00001 | down |
| ENSMUSG00000000605 | Clcn4 | -2.19885 | <0.00001 | down |
| ENSMUSG00000024188 | Luc7l | -1.20557 | <0.00001 | down |
| ENSMUSG00000089960 | Ugt1a1 | 1.870482 | <0.00001 | up |
| ENSMUSG00000022515 | Anks3 | -1.20238 | <0.00001 | down |
| ENSMUSG00000040565 | Btaf1 | -1.25244 | <0.00001 | down |
| ENSMUSG00000078931 | Pdf | 2.138088 | <0.00001 | up |
| ENSMUSG00000021719 | Rgs7bp | -2.35571 | <0.00001 | down |
| ENSMUSG00000000340 | Dbt | -2.12236 | <0.00001 | down |
| ENSMUSG00000021495 | Fam193b | -2.13955 | <0.00001 | down |
| ENSMUSG00000063787 | Chchd1 | 1.57367 | <0.00001 | up |
| ENSMUSG00000022360 | Atad2 | -2.73372 | <0.00001 | down |
| ENSMUSG00000042082 | Arsb | 1.474824 | <0.00001 | up |
| ENSMUSG00000021149 | Gtpbp4 | -2.22286 | <0.00001 | down |
| ENSMUSG00000024451 | Arap3 | -1.75277 | <0.00001 | down |
| ENSMUSG00000078201 | Tmem203 | 1.377434 | <0.00001 | up |
| ENSMUSG00000009418 | Nav1 | -1.60526 | <0.00001 | down |
| ENSMUSG00000032370 | Lactb | 1.230986 | <0.00001 | up |
| ENSMUSG00000024870 | Rab1b | 1.145798 | <0.00001 | up |
| ENSMUSG00000026417 | Pigr | -2.01426 | <0.00001 | down |
| ENSMUSG00000060036 | Rpl3 | 1.313454 | <0.00001 | up |
| ENSMUSG00000039219 | Arid4b | -1.10814 | <0.00001 | down |
| ENSMUSG00000018501 | Ncor1 | -1.06107 | <0.00001 | down |
| ENSMUSG00000028478 | Clta | 1.526513 | <0.00001 | up |
| ENSMUSG00000024866 | Acy3 | 1.275239 | <0.00001 | up |
| ENSMUSG00000034543 | Morc2a | -1.37818 | <0.00001 | down |
| ENSMUSG00000026064 | Ptp4a1 | 4.363092 | <0.00001 | up |
| ENSMUSG00000021271 | Zfp839 | -1.65035 | <0.00001 | down |
| ENSMUSG00000086922 | Gm13835 | 8.940429 | <0.00001 | up |
| ENSMUSG00000034994 | Eef2 | 1.326935 | <0.00001 | up |
| ENSMUSG00000021969 | Zdhhc20 | -1.94525 | <0.00001 | down |
| ENSMUSG00000038538 | Ubn2 | -3.20786 | <0.00001 | down |
| ENSMUSG00000085028 | Slc2a4rg-ps | -2.66191 | <0.00001 | down |
| ENSMUSG00000054733 | Msra | 1.195008 | <0.00001 | up |
| ENSMUSG00000069805 | Fbp1 | 2.386347 | <0.00001 | up |
| ENSMUSG00000005054 | Cstb | 2.031044 | <0.00001 | up |
| ENSMUSG00000032554 | Trf | 1.716228 | <0.00001 | up |
| ENSMUSG00000115276 | 9930017N22Rik | -3.71081 | <0.00001 | down |
| ENSMUSG00000023175 | Bsg | 1.102174 | <0.00001 | up |
| ENSMUSG00000033713 | Foxn3 | -1.95776 | <0.00001 | down |
| ENSMUSG00000036764 | Dnajc12 | 3.535322 | <0.00001 | up |
| ENSMUSG00000010608 | Rbm25 | -1.51692 | <0.00001 | down |
| ENSMUSG00000035885 | Cox8a | 1.451703 | <0.00001 | up |
| ENSMUSG00000026872 | Zeb2 | -1.83513 | <0.00001 | down |
| ENSMUSG00000034037 | Fgd5 | -1.75639 | <0.00001 | down |
| ENSMUSG00000063358 | Mapk1 | 1.083958 | <0.00001 | up |
| ENSMUSG00000120336 | - | -3.56401 | <0.00001 | down |
| ENSMUSG00000097820 | E530011L22Rik | -1.42317 | <0.00001 | down |
| ENSMUSG00000067951 | Vmn1r227 | -3.14307 | <0.00001 | down |
| ENSMUSG00000035011 | Zbtb7a | -2.08311 | <0.00001 | down |
| ENSMUSG00000036932 | Aifm1 | 1.095278 | <0.00001 | up |
| ENSMUSG00000020561 | Polr1f | -1.50453 | <0.00001 | down |
| ENSMUSG00000033544 | Angptl1 | -4.50743 | <0.00001 | down |
| ENSMUSG00000038569 | Rad9b | -2.07462 | <0.00001 | down |
| ENSMUSG00000021646 | Mccc2 | 1.09334 | <0.00001 | up |
| ENSMUSG00000021268 | Meg3 | -2.79893 | <0.00001 | down |
| ENSMUSG00000031320 | Rps4x | 1.094251 | <0.00001 | up |
| ENSMUSG00000120390 | - | -1.57612 | <0.00001 | down |
| ENSMUSG00000062933 | Gm10123 | 2.401868 | <0.00001 | up |
| ENSMUSG00000038286 | Bphl | 1.648148 | <0.00001 | up |
| ENSMUSG00000059429 | Olfr365 | -2.28724 | <0.00001 | down |
| ENSMUSG00000046229 | Scand1 | 3.826129 | <0.00001 | up |
| ENSMUSG00000016024 | Lbp | 1.502444 | <0.00001 | up |
| ENSMUSG00000026199 | Ankzf1 | -1.748 | <0.00001 | down |
| ENSMUSG00000041775 | Mapk1ip1 | -2.09379 | <0.00001 | down |
| ENSMUSG00000031858 | Mau2 | -1.5799 | <0.00001 | down |
| ENSMUSG00000078695 | Cisd3 | -1.10885 | <0.00001 | down |
| ENSMUSG00000053754 | Chd8 | -1.42428 | <0.00001 | down |
| ENSMUSG00000024330 | Col11a2 | -4.01606 | <0.00001 | down |
| ENSMUSG00000048234 | Rnf149 | 2.355234 | <0.00001 | up |
| ENSMUSG00000019762 | Iyd | 1.753305 | <0.00001 | up |
| ENSMUSG00000024217 | Snrpc | 1.750356 | <0.00001 | up |
| ENSMUSG00000037994 | Slc9b2 | -3.12734 | <0.00001 | down |
| ENSMUSG00000059355 | Wdr83os | 1.668335 | <0.00001 | up |
| ENSMUSG00000090145 | Ugt1a6b | 1.523178 | <0.00001 | up |
| ENSMUSG00000022912 | Pros1 | 1.174736 | <0.00001 | up |
| ENSMUSG00000046811 | Gltpd2 | 1.63077 | <0.00001 | up |
| ENSMUSG00000033943 | Mga | -1.15278 | <0.00001 | down |
| ENSMUSG00000104388 | Gm37033 | -4.40787 | <0.00001 | down |
| ENSMUSG00000031508 | Ankrd10 | -1.69824 | <0.00001 | down |
| ENSMUSG00000002332 | Dhrs1 | 1.641344 | <0.00001 | up |
| ENSMUSG00000024958 | Gpr137 | -1.69997 | <0.00001 | down |
| ENSMUSG00000025724 | Sec11a | 1.306448 | <0.00001 | up |
| ENSMUSG00000031490 | Eif4ebp1 | 1.522822 | <0.00001 | up |
| ENSMUSG00000067212 | H2-T23 | 1.158102 | <0.00001 | up |
| ENSMUSG00000043923 | Ccdc84 | -1.76973 | <0.00001 | down |
| ENSMUSG00000023052 | Npff | -3.13259 | <0.00001 | down |
| ENSMUSG00000046814 | Gchfr | 1.554917 | <0.00001 | up |
| ENSMUSG00000000711 | Rab5b | 1.206309 | <0.00001 | up |
| ENSMUSG00000022877 | Hrg | 1.502093 | <0.00001 | up |
| ENSMUSG00000121093 | - | -1.53451 | <0.00001 | down |
| ENSMUSG00000014850 | Msh3 | -1.54867 | <0.00001 | down |
| ENSMUSG00000105810 | Gm43435 | -5.04123 | <0.00001 | down |
| ENSMUSG00000105247 | Gm42519 | -3.75028 | <0.00001 | down |
| ENSMUSG00000106636 | Gm43813 | -4.35485 | <0.00001 | down |
| ENSMUSG00000038010 | Ccdc138 | -2.29913 | <0.00001 | down |
| ENSMUSG00000121426 | - | -2.38991 | <0.00001 | down |
| ENSMUSG00000109807 | Gm45244 | -4.78657 | <0.00001 | down |
| ENSMUSG00000021048 | Mthfd1 | 1.079538 | <0.00001 | up |
| ENSMUSG00000035649 | Zcchc7 | -1.39815 | <0.00001 | down |
| ENSMUSG00000030842 | Lamtor1 | 1.373205 | <0.00001 | up |
| ENSMUSG00000017802 | Retreg3 | 1.086982 | <0.00001 | up |
| ENSMUSG00000042742 | Bmt2 | -1.56658 | <0.00001 | down |
| ENSMUSG00000028691 | Prdx1 | 1.689449 | <0.00001 | up |
| ENSMUSG00000030359 | Pzp | 1.730823 | <0.00001 | up |
| ENSMUSG00000032580 | Rbm5 | -2.10183 | <0.00001 | down |
| ENSMUSG00000032253 | Phip | -1.50054 | <0.00001 | down |
| ENSMUSG00000052557 | Gan | -2.35617 | <0.00001 | down |
| ENSMUSG00000025060 | Slk | -1.07728 | <0.00001 | down |
| ENSMUSG00000020530 | Ggnbp2 | -1.00789 | <0.00001 | down |
| ENSMUSG00000063229 | Ldha | 2.077186 | <0.00001 | up |
| ENSMUSG00000036813 | Entpd8 | -1.39959 | <0.00001 | down |
| ENSMUSG00000032743 | Katnip | -1.54864 | <0.00001 | down |
| ENSMUSG00000035107 | Dcbld2 | -2.77109 | <0.00001 | down |
| ENSMUSG00000105201 | Gm43362 | -5.47585 | <0.00001 | down |
| ENSMUSG00000097048 | 1600020E01Rik | -2.29085 | <0.00001 | down |
| ENSMUSG00000121473 | Adh6-ps1 | -2.6953 | <0.00001 | down |
| ENSMUSG00000092395 | Gm20463 | -2.4343 | <0.00001 | down |
| ENSMUSG00000048620 | Olfr1336 | -3.61327 | <0.00001 | down |
| ENSMUSG00000005373 | Mlxipl | -1.43892 | <0.00001 | down |
| ENSMUSG00000035697 | Arhgap45 | -1.93935 | <0.00001 | down |
| ENSMUSG00000032212 | Sltm | -1.3551 | <0.00001 | down |
| ENSMUSG00000079555 | Haus3 | -1.84021 | <0.00001 | down |
| ENSMUSG00000024477 | Pggt1b | -1.70089 | <0.00001 | down |
| ENSMUSG00000053477 | Tcf4 | -1.8363 | <0.00001 | down |
| ENSMUSG00000073888 | Ccl27a | -1.57601 | <0.00001 | down |
| ENSMUSG00000020571 | Pdia6 | 1.670965 | <0.00001 | up |
| ENSMUSG00000097392 | Thoc2l | -1.70753 | <0.00001 | down |
| ENSMUSG00000071337 | Tia1 | -1.82068 | <0.00001 | down |
| ENSMUSG00000046532 | Ar | -2.85635 | <0.00001 | down |
| ENSMUSG00000103432 | 6720464F23Rik | -7.15106 | <0.00001 | down |
| ENSMUSG00000032316 | Clk3 | -1.44455 | <0.00001 | down |
| ENSMUSG00000104377 | Gm37515 | -2.40569 | <0.00001 | down |
| ENSMUSG00000062580 | Timm17a | 1.669106 | <0.00001 | up |
| ENSMUSG00000028199 | Cryz | 1.925707 | <0.00001 | up |
| ENSMUSG00000101599 | Gm20342 | -2.80943 | <0.00001 | down |
| ENSMUSG00000078812 | Eif5a | 1.395163 | <0.00001 | up |
| ENSMUSG00000028161 | Ppp3ca | -2.28987 | <0.00001 | down |
| ENSMUSG00000103421 | Golt1a | 2.455669 | <0.00001 | up |
| ENSMUSG00000021820 | Camk2g | -1.47227 | <0.00001 | down |
| ENSMUSG00000021868 | Ppif | 1.344277 | <0.00001 | up |
| ENSMUSG00000043467 | Zbtb37 | -1.70662 | <0.00001 | down |
| ENSMUSG00000121141 | - | -2.72336 | <0.00001 | down |
| ENSMUSG00000029545 | Acads | 1.792627 | <0.00001 | up |
| ENSMUSG00000019876 | Pkib | -2.7149 | <0.00001 | down |
| ENSMUSG00000026102 | Inpp1 | -2.29569 | <0.00001 | down |
| ENSMUSG00000121069 | - | -1.92494 | <0.00001 | down |
| ENSMUSG00000021576 | Pdcd6 | 1.634842 | <0.00001 | up |
| ENSMUSG00000104149 | Gm37138 | -3.55248 | <0.00001 | down |
| ENSMUSG00000017721 | Pigt | 1.973575 | <0.00001 | up |
| ENSMUSG00000029198 | Grpel1 | 1.93201 | <0.00001 | up |
| ENSMUSG00000049382 | Krt8 | 1.614777 | <0.00001 | up |
| ENSMUSG00000101249 | Gm29216 | -3.31602 | <0.00001 | down |
| ENSMUSG00000053286 | Trmt1l | -1.48662 | <0.00001 | down |
| ENSMUSG00000020134 | Peli1 | -2.46448 | <0.00001 | down |
| ENSMUSG00000108173 | Gm44231 | -3.00571 | <0.00001 | down |
| ENSMUSG00000063406 | Tmed5 | -3.25256 | <0.00001 | down |
| ENSMUSG00000114470 | Gm49395 | -3.77084 | <0.00001 | down |
| ENSMUSG00000109162 | 2900027M19Rik | -2.85129 | <0.00001 | down |
| ENSMUSG00000059729 | Olfr1385 | -2.42958 | <0.00001 | down |
| ENSMUSG00000031595 | Pdgfrl | -4.36911 | <0.00001 | down |
| ENSMUSG00000056268 | Dennd1b | -2.20241 | <0.00001 | down |
| ENSMUSG00000022841 | Ap2m1 | 1.088576 | <0.00001 | up |
| ENSMUSG00000106577 | Gm31026 | -3.51586 | <0.00001 | down |
| ENSMUSG00000027665 | Pik3ca | -2.09026 | <0.00001 | down |
| ENSMUSG00000028760 | Eif4g3 | -2.15074 | <0.00001 | down |
| ENSMUSG00000035401 | Emsy | -2.70607 | <0.00001 | down |
| ENSMUSG00000019590 | Cyb561 | 3.483488 | <0.00001 | up |
| ENSMUSG00000029068 | Ccnl2 | -1.66639 | <0.00001 | down |
| ENSMUSG00000056763 | Cspp1 | -1.73094 | <0.00001 | down |
| ENSMUSG00000064356 | mt-Atp8 | -2.74712 | <0.00001 | down |
| ENSMUSG00000051748 | Wfdc21 | 2.334319 | <0.00001 | up |
| ENSMUSG00000030761 | Myo7a | -1.82122 | <0.00001 | down |
| ENSMUSG00000079165 | Sap25 | -3.81816 | <0.00001 | down |
| ENSMUSG00000022304 | Dpys | 1.659882 | <0.00001 | up |
| ENSMUSG00000026193 | Fn1 | 1.452226 | <0.00001 | up |
| ENSMUSG00000012117 | Dhdds | 1.105661 | <0.00001 | up |
| ENSMUSG00000085826 | Gm15638 | -3.06815 | <0.00001 | down |
| ENSMUSG00000013076 | Amotl1 | -2.78578 | <0.00001 | down |
| ENSMUSG00000114722 | Gm31392 | -3.67545 | <0.00001 | down |
| ENSMUSG00000000600 | Krit1 | -1.36262 | <0.00001 | down |
| ENSMUSG00000063856 | Gpx1 | 2.052106 | <0.00001 | up |
| ENSMUSG00000069045 | Ddx3y | -2.67686 | <0.00001 | down |
| ENSMUSG00000024299 | Adamts10 | -1.90263 | <0.00001 | down |
| ENSMUSG00000104211 | Gm37985 | -2.74785 | <0.00001 | down |
| ENSMUSG00000117599 | Gm49971 | -2.79231 | <0.00001 | down |
| ENSMUSG00000054408 | Spcs3 | 1.331245 | <0.00001 | up |
| ENSMUSG00000008575 | Nfib | -2.77987 | <0.00001 | down |
| ENSMUSG00000055436 | Srsf11 | -1.64587 | <0.00001 | down |
| ENSMUSG00000097336 | Fendrr | -2.84125 | <0.00001 | down |
| ENSMUSG00000028743 | Akr7a5 | 1.721992 | <0.00001 | up |
| ENSMUSG00000113961 | Gm48498 | -2.81141 | <0.00001 | down |
| ENSMUSG00000076431 | Sox4 | -2.71913 | <0.00001 | down |
| ENSMUSG00000001755 | Coasy | 1.485935 | <0.00001 | up |
| ENSMUSG00000024037 | Wdr4 | -1.49254 | <0.00001 | down |
| ENSMUSG00000026034 | Clk1 | -1.60354 | <0.00001 | down |
| ENSMUSG00000032042 | Srpr | 1.6092 | <0.00001 | up |
| ENSMUSG00000094747 | Olfr1307 | -2.88368 | <0.00001 | down |
| ENSMUSG00000040481 | Bptf | -2.20403 | <0.00001 | down |
| ENSMUSG00000080985 | Gm13559 | -5.20527 | <0.00001 | down |
| ENSMUSG00000047230 | Cldn2 | 2.174815 | <0.00001 | up |
| ENSMUSG00000043183 | Simc1 | -2.1816 | <0.00001 | down |
| ENSMUSG00000022974 | Paxbp1 | -2.21763 | <0.00001 | down |
| ENSMUSG00000020458 | Rtn4 | 1.778313 | <0.00001 | up |
| ENSMUSG00000029238 | Clock | -2.01256 | <0.00001 | down |
| ENSMUSG00000005732 | Ranbp1 | 1.24253 | <0.00001 | up |
| ENSMUSG00000033831 | Fgb | 2.130802 | <0.00001 | up |
| ENSMUSG00000072572 | Slc39a2 | -3.54311 | <0.00001 | down |
| ENSMUSG00000026750 | Psmb7 | 1.540947 | <0.00001 | up |
| ENSMUSG00000020089 | Ppa1 | 2.124879 | <0.00001 | up |
| ENSMUSG00000017390 | Aldoc | 2.568806 | <0.00001 | up |
| ENSMUSG00000070891 | Gm12689 | -5.39689 | <0.00001 | down |
| ENSMUSG00000037022 | Mmaa | -1.13213 | <0.00001 | down |
| ENSMUSG00000030967 | Zranb1 | -2.24843 | <0.00001 | down |
| ENSMUSG00000034911 | Ushbp1 | -1.92153 | <0.00001 | down |
| ENSMUSG00000106948 | Gm42785 | -2.99993 | <0.00001 | down |
| ENSMUSG00000066258 | Trim12a | -1.91989 | <0.00001 | down |
| ENSMUSG00000106408 | Gm43321 | -2.74565 | <0.00001 | down |
| ENSMUSG00000031232 | Magt1 | -2.34923 | <0.00001 | down |
| ENSMUSG00000019122 | Ccl9 | 1.907136 | <0.00001 | up |
| ENSMUSG00000025487 | Psmd13 | 2.066011 | <0.00001 | up |
| ENSMUSG00000040033 | Stat2 | -1.92078 | <0.00001 | down |
| ENSMUSG00000021286 | Zfyve21 | 1.614836 | <0.00001 | up |
| ENSMUSG00000116858 | Gm49797 | -2.42951 | <0.00001 | down |
| ENSMUSG00000024422 | Dhx16 | -1.56001 | <0.00001 | down |
| ENSMUSG00000025130 | P4hb | 1.406634 | <0.00001 | up |
| ENSMUSG00000037243 | Zfp692 | -2.38552 | <0.00001 | down |
| ENSMUSG00000029716 | Tfr2 | 1.023467 | <0.00001 | up |
| ENSMUSG00000085334 | Gm12940 | -3.25926 | <0.00001 | down |
| ENSMUSG00000021876 | Rnase4 | 1.705121 | <0.00001 | up |
| ENSMUSG00000025357 | Dgka | -1.56863 | <0.00001 | down |
| ENSMUSG00000002032 | Tmem25 | -2.05616 | <0.00001 | down |
| ENSMUSG00000038301 | Snx10 | 1.51829 | <0.00001 | up |
| ENSMUSG00000037966 | Ninj1 | 1.413972 | <0.00001 | up |
| ENSMUSG00000055435 | Maf | -2.48682 | <0.00001 | down |
| ENSMUSG00000091192 | Sardhos | -9.93622 | <0.00001 | down |
| ENSMUSG00000118017 | Gm29966 | -4.76296 | <0.00001 | down |
| ENSMUSG00000104394 | Gm37254 | -2.7246 | <0.00001 | down |
| ENSMUSG00000026542 | Apcs | 3.476417 | <0.00001 | up |
| ENSMUSG00000026342 | Slc35f5 | 2.42812 | <0.00001 | up |
| ENSMUSG00000105454 | Gm43830 | -4.25075 | <0.00001 | down |
| ENSMUSG00000054404 | Slfn5 | -2.04852 | <0.00001 | down |
| ENSMUSG00000117390 | Gm50080 | -5.36274 | <0.00001 | down |
| ENSMUSG00000026189 | Pecr | 1.825381 | <0.00001 | up |
| ENSMUSG00000042138 | Msantd2 | -2.09108 | <0.00001 | down |
| ENSMUSG00000017747 | Ghdc | -2.81298 | <0.00001 | down |
| ENSMUSG00000008140 | Emc10 | 1.867537 | <0.00001 | up |
| ENSMUSG00000075543 | Urad | 1.942674 | <0.00001 | up |
| ENSMUSG00000033161 | Atp1a1 | 1.675448 | <0.00001 | up |
| ENSMUSG00000111118 | Gm6545 | -4.53182 | <0.00001 | down |
| ENSMUSG00000060600 | Eno3 | -3.00407 | <0.00001 | down |
| ENSMUSG00000037958 | Nsrp1 | -2.3584 | <0.00001 | down |
| ENSMUSG00000006014 | Prg4 | 2.288389 | <0.00001 | up |
| ENSMUSG00000031447 | Lamp1 | 2.025741 | <0.00001 | up |
| ENSMUSG00000086583 | Gm15500 | 1.322714 | <0.00001 | up |
| ENSMUSG00000075081 | Olfr1247 | -2.26221 | <0.00001 | down |
| ENSMUSG00000074340 | Ovgp1 | -4.08541 | <0.00001 | down |
| ENSMUSG00000021270 | Hsp90aa1 | -2.18129 | <0.00001 | down |
| ENSMUSG00000101970 | Chaserr | -1.67211 | <0.00001 | down |
| ENSMUSG00000065947 | mt-Nd4l | -3.69593 | <0.00001 | down |
| ENSMUSG00000042797 | Aqp11 | 1.50245 | <0.00001 | up |
| ENSMUSG00000036620 | Mgat4b | 1.502303 | <0.00001 | up |
| ENSMUSG00000115801 | Gm55330 | -1.56678 | <0.00001 | down |
| ENSMUSG00000031133 | Arhgef6 | -1.93764 | <0.00001 | down |
| ENSMUSG00000112319 | Gm47221 | -2.36047 | <0.00001 | down |
| ENSMUSG00000024181 | Mrpl28 | 1.580327 | <0.00001 | up |
| ENSMUSG00000106706 | C530043K16Rik | -5.44981 | <0.00001 | down |
| ENSMUSG00000034168 | Irf2bpl | 1.472844 | <0.00001 | up |
| ENSMUSG00000020766 | Galk1 | 1.804529 | <0.00001 | up |
| ENSMUSG00000067736 | Gm10222 | -2.43529 | <0.00001 | down |
| ENSMUSG00000037072 | Selenof | 1.696409 | <0.00001 | up |
| ENSMUSG00000005803 | Sqor | 1.959805 | <0.00001 | up |
| ENSMUSG00000032369 | Plscr1 | 1.557867 | <0.00001 | up |
| ENSMUSG00000003402 | Prkcsh | 1.366742 | <0.00001 | up |
| ENSMUSG00000040612 | Ildr2 | 2.104498 | <0.00001 | up |
| ENSMUSG00000113523 | Gm48366 | -3.33921 | <0.00001 | down |
| ENSMUSG00000006315 | Tmem147 | 1.31292 | <0.00001 | up |
| ENSMUSG00000023073 | Slc10a2 | 1.951649 | <0.00001 | up |
| ENSMUSG00000025102 | 3110040N11Rik | 2.329966 | <0.00001 | up |
| ENSMUSG00000117238 | - | -4.0896 | <0.00001 | down |
| ENSMUSG00000060487 | Samd5 | -3.0584 | <0.00001 | down |
| ENSMUSG00000029119 | Man2b2 | 1.853724 | <0.00001 | up |
| ENSMUSG00000018411 | Mapt | -2.53459 | <0.00001 | down |
| ENSMUSG00000033735 | Spr | 1.986211 | <0.00001 | up |
| ENSMUSG00000052310 | Slc39a1 | 1.094274 | <0.00001 | up |
| ENSMUSG00000024981 | Acsl5 | 2.139864 | <0.00001 | up |
| ENSMUSG00000027801 | Tm4sf4 | 1.616371 | <0.00001 | up |
| ENSMUSG00000027993 | Trim2 | -2.8069 | <0.00001 | down |
| ENSMUSG00000024164 | C3 | 1.426924 | <0.00001 | up |
| ENSMUSG00000109179 | Gm35339 | -3.05357 | <0.00001 | down |
| ENSMUSG00000029701 | Rbm28 | -1.49117 | <0.00001 | down |
| ENSMUSG00000112788 | Gm47821 | -3.56017 | <0.00001 | down |
| ENSMUSG00000044139 | Prss53 | -2.43408 | <0.00001 | down |
| ENSMUSG00000016481 | Cr1l | 1.221515 | <0.00001 | up |
| ENSMUSG00000000088 | Cox5a | 2.167902 | <0.00001 | up |
| ENSMUSG00000027613 | Eif6 | 2.037394 | <0.00001 | up |
| ENSMUSG00000040963 | Asgr2 | 1.355599 | <0.00001 | up |
| ENSMUSG00000064246 | Chil1 | 6.467503 | <0.00001 | up |
| ENSMUSG00000044937 | Ttc41 | -2.45725 | <0.00001 | down |
| ENSMUSG00000034926 | Dhcr24 | 2.446662 | <0.00001 | up |
| ENSMUSG00000030341 | Tnfrsf1a | 1.486871 | <0.00001 | up |
| ENSMUSG00000045991 | Onecut2 | -2.22238 | <0.00001 | down |
| ENSMUSG00000007891 | Ctsd | 1.176708 | <0.00001 | up |
| ENSMUSG00000026223 | Itm2c | 2.52676 | <0.00001 | up |
| ENSMUSG00000031925 | Maml2 | -3.18266 | <0.00001 | down |
| ENSMUSG00000113517 | Gm47905 | -3.46674 | <0.00001 | down |
| ENSMUSG00000034449 | Dhrs11 | 1.472794 | <0.00001 | up |
| ENSMUSG00000029430 | Ran | 1.777052 | <0.00001 | up |
| ENSMUSG00000003053 | Cyp2c29 | 2.486254 | <0.00001 | up |
| ENSMUSG00000089940 | Gm4117 | -3.03896 | <0.00001 | down |
| ENSMUSG00000033684 | Qsox1 | 1.70778 | <0.00001 | up |
| ENSMUSG00000029038 | Ssu72 | 2.140618 | <0.00001 | up |
| ENSMUSG00000059481 | Plg | 1.391687 | <0.00001 | up |
| ENSMUSG00000023456 | Tpi1 | 1.77702 | <0.00001 | up |
| ENSMUSG00000052151 | Plpp2 | 1.526043 | <0.00001 | up |
| ENSMUSG00000025979 | Mob4 | -2.05227 | <0.00001 | down |
| ENSMUSG00000021952 | Xpo4 | -3.13092 | <0.00001 | down |
| ENSMUSG00000001424 | Snd1 | 1.224588 | <0.00001 | up |
| ENSMUSG00000028356 | Ambp | 1.93067 | <0.00001 | up |
| ENSMUSG00000036083 | Slc17a3 | -1.681 | <0.00001 | down |
| ENSMUSG00000021135 | Slc10a1 | 1.837387 | <0.00001 | up |
| ENSMUSG00000087412 | Gm15501 | 2.996597 | <0.00001 | up |
| ENSMUSG00000057729 | Prtn3 | 6.822511 | <0.00001 | up |
| ENSMUSG00000060961 | Slc4a4 | 1.464868 | <0.00001 | up |
| ENSMUSG00000038695 | Josd2 | -2.08488 | <0.00001 | down |
| ENSMUSG00000073411 | H2-D1 | 1.256175 | <0.00001 | up |
| ENSMUSG00000025511 | Tspan4 | 2.011021 | <0.00001 | up |
| ENSMUSG00000040128 | Pnrc1 | -1.51864 | <0.00001 | down |
| ENSMUSG00000032051 | Fdx1 | 1.495917 | <0.00001 | up |
| ENSMUSG00000041483 | Zfp281 | -3.07182 | <0.00001 | down |
| ENSMUSG00000001100 | Poldip2 | 1.358529 | <0.00001 | up |
| ENSMUSG00000118155 | Gm50136 | -4.72485 | <0.00001 | down |
| ENSMUSG00000021751 | Acox2 | 1.500562 | <0.00001 | up |
| ENSMUSG00000021213 | Akr1c13 | 2.000454 | <0.00001 | up |
| ENSMUSG00000078687 | Mup8 | -2.77409 | <0.00001 | down |
| ENSMUSG00000032265 | Tent5a | -2.76994 | <0.00001 | down |
| ENSMUSG00000045672 | Col27a1 | -4.15117 | <0.00001 | down |
| ENSMUSG00000007097 | Atp1a2 | -2.38035 | <0.00001 | down |
| ENSMUSG00000024943 | Smc5 | -1.80059 | <0.00001 | down |
| ENSMUSG00000025481 | Urah | 1.887349 | <0.00001 | up |
| ENSMUSG00000090386 | Mir99ahg | -3.4983 | <0.00001 | down |
| ENSMUSG00000029422 | Rsrc2 | -1.03647 | <0.00001 | down |
| ENSMUSG00000108857 | Gm44578 | -4.77912 | <0.00001 | down |
| ENSMUSG00000027430 | Dtd1 | 1.607908 | <0.00001 | up |
| ENSMUSG00000021091 | Serpina3n | 2.341803 | <0.00001 | up |
| ENSMUSG00000019647 | Sema6a | -2.19344 | <0.00001 | down |
| ENSMUSG00000091780 | Sco2 | -1.8987 | <0.00001 | down |
| ENSMUSG00000021210 | Akr1c6 | 1.998299 | <0.00001 | up |
| ENSMUSG00000018770 | Atp5g3 | 1.992227 | <0.00001 | up |
| ENSMUSG00000044636 | Csrnp2 | -3.22097 | <0.00001 | down |
| ENSMUSG00000072501 | Phf20l1 | -1.89056 | <0.00001 | down |
| ENSMUSG00000022617 | Chkb | -1.6449 | <0.00001 | down |
| ENSMUSG00000029096 | Htra3 | -2.55926 | <0.00001 | down |
| ENSMUSG00000004565 | Pnpla6 | -1.7987 | <0.00001 | down |
| ENSMUSG00000107529 | Gm44291 | -4.64653 | <0.00001 | down |
| ENSMUSG00000114828 | AI463229 | -3.15406 | <0.00001 | down |
| ENSMUSG00000033446 | Lpar6 | -1.37203 | <0.00001 | down |
| ENSMUSG00000022037 | Clu | 2.193031 | <0.00001 | up |
| ENSMUSG00000074212 | Dnajb14 | -1.75196 | <0.00001 | down |
| ENSMUSG00000090272 | Mndal | -2.13881 | <0.00001 | down |
| ENSMUSG00000097042 | Gm17491 | -2.63324 | <0.00001 | down |
| ENSMUSG00000121083 | - | -2.28337 | <0.00001 | down |
| ENSMUSG00000026405 | C4bp | 1.92014 | <0.00001 | up |
| ENSMUSG00000106030 | Gm43611 | -3.96457 | <0.00001 | down |
| ENSMUSG00000022546 | Gpt | 1.287545 | <0.00001 | up |
| ENSMUSG00000038188 | Scarf1 | -2.40722 | <0.00001 | down |
| ENSMUSG00000032080 | Apoa4 | 2.611617 | <0.00001 | up |
| ENSMUSG00000023224 | Serping1 | 1.900023 | <0.00001 | up |
| ENSMUSG00000053931 | Cnn3 | 1.015112 | <0.00001 | up |
| ENSMUSG00000046402 | Rbp1 | 1.900869 | <0.00001 | up |
| ENSMUSG00000029810 | Tmem176b | 1.927623 | <0.00001 | up |
| ENSMUSG00000060591 | Ifitm2 | 1.606348 | <0.00001 | up |
| ENSMUSG00000053317 | Sec61b | 1.76105 | <0.00001 | up |
| ENSMUSG00000037818 | Abhd18 | -1.74404 | <0.00001 | down |
| ENSMUSG00000096002 | Vmn2r53 | -2.63262 | <0.00001 | down |
| ENSMUSG00000112239 | Gm17823 | -4.46799 | <0.00001 | down |
| ENSMUSG00000067653 | Ankrd23 | -2.7585 | <0.00001 | down |
| ENSMUSG00000059136 | Olfr539 | -3.31641 | <0.00001 | down |
| ENSMUSG00000064373 | Selenop | 2.661765 | <0.00001 | up |
| ENSMUSG00000031848 | Lsm4 | 2.081843 | <0.00001 | up |
| ENSMUSG00000053964 | Lgals4 | -3.54525 | <0.00001 | down |
| ENSMUSG00000001376 | Vps50 | -1.89636 | <0.00001 | down |
| ENSMUSG00000025465 | Echs1 | 1.617981 | <0.00001 | up |
| ENSMUSG00000103693 | Gm37529 | -4.68732 | <0.00001 | down |
| ENSMUSG00000029009 | Mthfr | -2.3116 | <0.00001 | down |
| ENSMUSG00000112580 | Gm47673 | -3.28432 | <0.00001 | down |
| ENSMUSG00000117780 | Gm3734 | -3.11918 | <0.00001 | down |
| ENSMUSG00000101939 | Gm28438 | -2.4625 | <0.00001 | down |
| ENSMUSG00000024131 | Slc3a1 | 2.652615 | <0.00001 | up |
| ENSMUSG00000042770 | Hebp1 | 1.426542 | <0.00001 | up |
| ENSMUSG00000097589 | Dleu2 | -2.02386 | <0.00001 | down |
| ENSMUSG00000031722 | Hp | 1.804865 | <0.00001 | up |
| ENSMUSG00000113476 | Gm48309 | -3.24319 | <0.00001 | down |
| ENSMUSG00000021877 | Arf4 | 1.678742 | <0.00001 | up |
| ENSMUSG00000031958 | Ldhd | 1.219278 | <0.00001 | up |
| ENSMUSG00000085404 | Gm12909 | -3.1635 | <0.00001 | down |
| ENSMUSG00000091509 | Gm17066 | -3.48242 | <0.00001 | down |
| ENSMUSG00000108155 | Gm44443 | -4.83777 | <0.00001 | down |
| ENSMUSG00000112622 | Gm47164 | -2.95208 | <0.00001 | down |
| ENSMUSG00000024843 | Chka | -3.40526 | <0.00001 | down |
| ENSMUSG00000038880 | Mrps34 | 1.454503 | <0.00001 | up |
| ENSMUSG00000053329 | Gatd3a | 1.711539 | <0.00001 | up |
| ENSMUSG00000075701 | Selenos | 2.393531 | <0.00001 | up |
| ENSMUSG00000023176 | Cpn2 | 1.742358 | <0.00001 | up |
| ENSMUSG00000117098 | Gm49909 | 2.899671 | <0.00001 | up |
| ENSMUSG00000042073 | Abhd14b | 1.746507 | <0.00001 | up |
| ENSMUSG00000044037 | Als2cl | -2.11928 | <0.00001 | down |
| ENSMUSG00000061947 | Serpina10 | 2.119169 | <0.00001 | up |
| ENSMUSG00000031594 | Fgl1 | 2.392567 | <0.00001 | up |
| ENSMUSG00000038155 | Gstp2 | -4.85993 | <0.00001 | down |
| ENSMUSG00000036040 | Adamtsl2 | -1.90445 | <0.00001 | down |
| ENSMUSG00000033860 | Fgg | 2.612722 | <0.00001 | up |
| ENSMUSG00000058793 | Cds2 | 1.348272 | <0.00001 | up |
| ENSMUSG00000021477 | Ctsl | 2.327869 | <0.00001 | up |
| ENSMUSG00000020733 | Slc9a3r1 | 1.905167 | <0.00001 | up |
| ENSMUSG00000039997 | Ifi203 | -2.22432 | <0.00001 | down |
| ENSMUSG00000028001 | Fga | 3.02044 | <0.00001 | up |
| ENSMUSG00000015289 | Lage3 | 1.803904 | <0.00001 | up |
| ENSMUSG00000060703 | Cd302 | 2.354505 | <0.00001 | up |
| ENSMUSG00000012428 | Steap4 | 2.720233 | <0.00001 | up |
| ENSMUSG00000064368 | mt-Nd6 | -2.40258 | <0.00001 | down |
| ENSMUSG00000064354 | mt-Co2 | -3.40462 | <0.00001 | down |
| ENSMUSG00000037936 | Scarb1 | 1.430005 | <0.00001 | up |
| ENSMUSG00000037095 | Lrg1 | 2.826954 | <0.00001 | up |
| ENSMUSG00000058486 | Wdr91 | -2.26598 | <0.00001 | down |
| ENSMUSG00000064367 | mt-Nd5 | -2.66735 | <0.00001 | down |
| ENSMUSG00000064360 | mt-Nd3 | -3.6839 | <0.00001 | down |
| ENSMUSG00000039196 | Orm1 | 2.350518 | <0.00001 | up |
| ENSMUSG00000064358 | mt-Co3 | -3.74934 | <0.00001 | down |
| ENSMUSG00000100862 | Gm10925 | -3.03368 | <0.00001 | down |
| ENSMUSG00000064351 | mt-Co1 | -2.92501 | <0.00001 | down |
| ENSMUSG00000101111 | Gm28437 | -2.94072 | <0.00001 | down |
| ENSMUSG00000021922 | Itih4 | 2.56793 | <0.00001 | up |
| ENSMUSG00000064339 | mt-Rnr2 | -3.60152 | <0.00001 | down |
| ENSMUSG00000030895 | Hpx | 2.621133 | <0.00001 | up |
| ENSMUSG00000064337 | mt-Rnr1 | -3.41691 | <0.00001 | down |
| ENSMUSG00000064345 | mt-Nd2 | -2.85197 | <0.00001 | down |
| ENSMUSG00000064341 | mt-Nd1 | -2.98024 | <0.00001 | down |
| ENSMUSG00000064357 | mt-Atp6 | -3.50296 | <0.00001 | down |
| ENSMUSG00000030968 | Pdilt | -2.85912 | <0.00001 | down |
| ENSMUSG00000064370 | mt-Cytb | -3.06463 | <0.00001 | down |
| ENSMUSG00000102070 | Gm28661 | -3.04433 | <0.00001 | down |
| ENSMUSG00000064363 | mt-Nd4 | -3.22349 | <0.00001 | down |
| ENSMUSG00000061540 | Orm2 | 4.815266 | <0.00001 | up |
| ENSMUSG00000074115 | Saa1 | 7.446594 | <0.00001 | up |
| ENSMUSG00000057465 | Saa2 | 8.246726 | <0.00001 | up |
| ENSMUSG00000026822 | Lcn2 | 5.462367 | <0.00001 | up |
