# Supplementary material for: Multi-time point transcriptomics and metabolomics reveal key transcription and metabolic features of hepatic ischemia-reperfusion injury in mice
Source: Genes Dis. 2024 Nov 17;12(2):101465. doi: 10.1016/j.gendis.2024.101465 (PMC11697123; doi:10.1016/j.gendis.2024.101465)
Supplement: Multimedia component 4 [file mmc4.docx]

**Table S2A.** The GO terms of differentially expressed genes (DEGs) identified by Gene Ontology (GO) in the Sham and I1R12 groups.

| **GO ID** | **GO term description** | **Rich factor** | **P-value** |
| --- | --- | --- | --- |
| GO:0007613 | memory | 0.085271 | 0.04979 |
| GO:0009060 | aerobic respiration | 0.075676 | 0.04966 |
| GO:1903844 | regulation of cellular response to transforming growth factor beta stimulus | 0.088235 | 0.04930 |
| GO:0007272 | ensheathment of neurons | 0.088235 | 0.04930 |
| GO:0008366 | axon ensheathment | 0.088235 | 0.04930 |
| GO:1901987 | regulation of cell cycle phase transition | 0.065169 | 0.04912 |
| GO:1900181 | negative regulation of protein localization to nucleus | 0.129032 | 0.04866 |
| GO:0032885 | regulation of polysaccharide biosynthetic process | 0.129032 | 0.04866 |
| GO:0051899 | membrane depolarization | 0.129032 | 0.04866 |
| GO:0051444 | negative regulation of ubiquitin-protein transferase activity | 0.129032 | 0.04866 |
| GO:0031670 | cellular response to nutrient | 0.129032 | 0.04866 |
| GO:0009886 | post-embryonic animal morphogenesis | 0.129032 | 0.04866 |
| GO:1903715 | regulation of aerobic respiration | 0.129032 | 0.04866 |
| GO:0030316 | osteoclast differentiation | 0.129032 | 0.04866 |
| GO:0042168 | heme metabolic process | 0.129032 | 0.04866 |
| GO:0045429 | positive regulation of nitric oxide biosynthetic process | 0.129032 | 0.04866 |
| GO:0044305 | calyx of Held | 0.129032 | 0.04866 |
| GO:0050681 | nuclear androgen receptor binding | 0.129032 | 0.04866 |
| GO:0015026 | coreceptor activity | 0.129032 | 0.04866 |
| GO:0004112 | cyclic-nucleotide phosphodiesterase activity | 0.129032 | 0.04866 |
| GO:0051057 | positive regulation of small GTPase mediated signal transduction | 0.101695 | 0.04855 |
| GO:0097035 | regulation of membrane lipid distribution | 0.101695 | 0.04855 |
| GO:0019233 | sensory perception of pain | 0.101695 | 0.04855 |
| GO:0032652 | regulation of interleukin-1 production | 0.094595 | 0.04829 |
| GO:0006081 | cellular aldehyde metabolic process | 0.094595 | 0.04829 |
| GO:0022834 | ligand-gated channel activity | 0.07947 | 0.04783 |
| GO:0044272 | sulfur compound biosynthetic process | 0.089109 | 0.04764 |
| GO:0034762 | regulation of transmembrane transport | 0.062731 | 0.04739 |
| GO:0051272 | obsolete positive regulation of cellular component movement | 0.25 | 0.04714 |
| GO:0090286 | obsolete cytoskeletal anchoring at nuclear membrane | 0.25 | 0.04714 |
| GO:0045657 | positive regulation of monocyte differentiation | 0.25 | 0.04714 |
| GO:0046618 | xenobiotic export from cell | 0.25 | 0.04714 |
| GO:0071377 | cellular response to glucagon stimulus | 0.25 | 0.04714 |
| GO:0002455 | humoral immune response mediated by circulating immunoglobulin | 0.25 | 0.04714 |
| GO:0031953 | negative regulation of protein autophosphorylation | 0.25 | 0.04714 |
| GO:0005984 | disaccharide metabolic process | 0.25 | 0.04714 |
| GO:0030300 | regulation of intestinal cholesterol absorption | 0.25 | 0.04714 |
| GO:0010041 | response to iron(III) ion | 0.25 | 0.04714 |
| GO:0070445 | regulation of oligodendrocyte progenitor proliferation | 0.25 | 0.04714 |
| GO:0060346 | bone trabecula formation | 0.25 | 0.04714 |
| GO:0031642 | negative regulation of myelination | 0.25 | 0.04714 |
| GO:0033085 | negative regulation of T cell differentiation in thymus | 0.25 | 0.04714 |
| GO:0014060 | regulation of epinephrine secretion | 0.25 | 0.04714 |
| GO:0038089 | positive regulation of cell migration by vascular endothelial growth factor signaling pathway | 0.25 | 0.04714 |
| GO:0090685 | RNA localization to nucleus | 0.25 | 0.04714 |
| GO:1904153 | negative regulation of retrograde protein transport, ER to cytosol | 0.25 | 0.04714 |
| GO:0019388 | galactose catabolic process | 0.25 | 0.04714 |
| GO:0090666 | scaRNA localization to Cajal body | 0.25 | 0.04714 |
| GO:0048251 | elastic fiber assembly | 0.25 | 0.04714 |
| GO:0003207 | cardiac chamber formation | 0.25 | 0.04714 |
| GO:0070486 | leukocyte aggregation | 0.25 | 0.04714 |
| GO:1901142 | insulin metabolic process | 0.25 | 0.04714 |
| GO:0097104 | postsynaptic membrane assembly | 0.25 | 0.04714 |
| GO:0019367 | fatty acid elongation, saturated fatty acid | 0.25 | 0.04714 |
| GO:0072718 | response to cisplatin | 0.25 | 0.04714 |
| GO:1904862 | inhibitory synapse assembly | 0.25 | 0.04714 |
| GO:0010873 | positive regulation of cholesterol esterification | 0.25 | 0.04714 |
| GO:0035095 | behavioral response to nicotine | 0.25 | 0.04714 |
| GO:0042256 | mature ribosome assembly | 0.25 | 0.04714 |
| GO:0032835 | glomerulus development | 0.25 | 0.04714 |
| GO:0010832 | negative regulation of myotube differentiation | 0.25 | 0.04714 |
| GO:0051342 | regulation of cyclic-nucleotide phosphodiesterase activity | 0.25 | 0.04714 |
| GO:0032819 | positive regulation of natural killer cell proliferation | 0.25 | 0.04714 |
| GO:0055123 | digestive system development | 0.25 | 0.04714 |
| GO:1903632 | positive regulation of aminoacyl-tRNA ligase activity | 0.25 | 0.04714 |
| GO:0006567 | threonine catabolic process | 0.25 | 0.04714 |
| GO:0034372 | very-low-density lipoprotein particle remodeling | 0.25 | 0.04714 |
| GO:0090670 | RNA localization to Cajal body | 0.25 | 0.04714 |
| GO:0098856 | intestinal lipid absorption | 0.25 | 0.04714 |
| GO:1902525 | regulation of protein monoubiquitination | 0.25 | 0.04714 |
| GO:1901678 | iron coordination entity transport | 0.25 | 0.04714 |
| GO:0010940 | positive regulation of necrotic cell death | 0.25 | 0.04714 |
| GO:0034756 | regulation of iron ion transport | 0.25 | 0.04714 |
| GO:1902548 | negative regulation of cellular response to vascular endothelial growth factor stimulus | 0.25 | 0.04714 |
| GO:0042588 | zymogen granule | 0.25 | 0.04714 |
| GO:0090661 | box H/ACA telomerase RNP complex | 0.25 | 0.04714 |
| GO:0004427 | inorganic diphosphate phosphatase activity | 0.25 | 0.04714 |
| GO:0102756 | very-long-chain 3-ketoacyl-CoA synthase activity | 0.25 | 0.04714 |
| GO:0004769 | steroid delta-isomerase activity | 0.25 | 0.04714 |
| GO:0009922 | fatty acid elongase activity | 0.25 | 0.04714 |
| GO:0031994 | insulin-like growth factor I binding | 0.25 | 0.04714 |
| GO:0015450 | protein-transporting ATPase activity | 0.25 | 0.04714 |
| GO:0004169 | dolichyl-phosphate-mannose-protein mannosyltransferase activity | 0.25 | 0.04714 |
| GO:0030283 | testosterone dehydrogenase [NAD(P)] activity | 0.25 | 0.04714 |
| GO:0005384 | manganese ion transmembrane transporter activity | 0.25 | 0.04714 |
| GO:0003857 | 3-hydroxyacyl-CoA dehydrogenase activity | 0.25 | 0.04714 |
| GO:0015093 | ferrous iron transmembrane transporter activity | 0.25 | 0.04714 |
| GO:0015136 | sialic acid transmembrane transporter activity | 0.25 | 0.04714 |
| GO:0033673 | negative regulation of kinase activity | 0.074074 | 0.04656 |
| GO:0004518 | nuclease activity | 0.065116 | 0.04651 |
| GO:0009218 | pyrimidine ribonucleotide metabolic process | 0.113636 | 0.04637 |
| GO:0051209 | release of sequestered calcium ion into cytosol | 0.113636 | 0.04637 |
| GO:0003254 | regulation of membrane depolarization | 0.113636 | 0.04637 |
| GO:0006730 | one-carbon metabolic process | 0.113636 | 0.04637 |
| GO:2000379 | positive regulation of reactive oxygen species metabolic process | 0.113636 | 0.04637 |
| GO:1904666 | regulation of ubiquitin protein ligase activity | 0.113636 | 0.04637 |
| GO:0005246 | calcium channel regulator activity | 0.113636 | 0.04637 |
| GO:0006721 | terpenoid metabolic process | 0.09 | 0.04608 |
| GO:0048704 | embryonic skeletal system morphogenesis | 0.09589 | 0.04540 |
| GO:0000027 | ribosomal large subunit assembly | 0.09589 | 0.04540 |
| GO:0030672 | synaptic vesicle membrane | 0.09589 | 0.04540 |
| GO:0099501 | exocytic vesicle membrane | 0.09589 | 0.04540 |
| GO:0005903 | brush border | 0.09589 | 0.04540 |
| GO:0071944 | cell periphery | 0.09589 | 0.04540 |
| GO:0009142 | nucleoside triphosphate biosynthetic process | 0.077348 | 0.04536 |
| GO:0048639 | positive regulation of developmental growth | 0.077348 | 0.04536 |
| GO:0055117 | regulation of cardiac muscle contraction | 0.103448 | 0.04526 |
| GO:1903202 | negative regulation of oxidative stress-induced cell death | 0.103448 | 0.04526 |
| GO:0010677 | negative regulation of cellular carbohydrate metabolic process | 0.103448 | 0.04526 |
| GO:0006260 | DNA replication | 0.080537 | 0.04518 |
| GO:0051780 | behavioral response to nutrient | 1 | 0.04492 |
| GO:0006593 | ornithine catabolic process | 1 | 0.04492 |
| GO:0002299 | alpha-beta intraepithelial T cell differentiation | 1 | 0.04492 |
| GO:0002904 | positive regulation of B cell apoptotic process | 1 | 0.04492 |
| GO:0033242 | negative regulation of cellular amine catabolic process | 1 | 0.04492 |
| GO:0033241 | regulation of cellular amine catabolic process | 1 | 0.04492 |
| GO:0042092 | type 2 immune response | 1 | 0.04492 |
| GO:0090343 | obsolete positive regulation of cell aging | 1 | 0.04492 |
| GO:1902913 | positive regulation of neuroepithelial cell differentiation | 1 | 0.04492 |
| GO:0039656 | modulation by virus of host gene expression | 1 | 0.04492 |
| GO:0009108 | obsolete coenzyme biosynthetic process | 1 | 0.04492 |
| GO:0098736 | negative regulation of the force of heart contraction | 1 | 0.04492 |
| GO:0120078 | cell adhesion involved in sprouting angiogenesis | 1 | 0.04492 |
| GO:0019402 | galactitol metabolic process | 1 | 0.04492 |
| GO:0015774 | polysaccharide transport | 1 | 0.04492 |
| GO:0021644 | vagus nerve morphogenesis | 1 | 0.04492 |
| GO:0046074 | dTMP catabolic process | 1 | 0.04492 |
| GO:0030103 | vasopressin secretion | 1 | 0.04492 |
| GO:1902078 | positive regulation of lateral motor column neuron migration | 1 | 0.04492 |
| GO:1902073 | positive regulation of hypoxia-inducible factor-1alpha signaling pathway | 1 | 0.04492 |
| GO:1902076 | regulation of lateral motor column neuron migration | 1 | 0.04492 |
| GO:0021812 | neuronal-glial interaction involved in cerebral cortex radial glia guided migration | 1 | 0.04492 |
| GO:0002943 | tRNA dihydrouridine synthesis | 1 | 0.04492 |
| GO:0046449 | creatinine metabolic process | 1 | 0.04492 |
| GO:1900082 | negative regulation of arginine catabolic process | 1 | 0.04492 |
| GO:1903280 | negative regulation of calcium:sodium antiporter activity | 1 | 0.04492 |
| GO:0097477 | lateral motor column neuron migration | 1 | 0.04492 |
| GO:0001300 | obsolete chronological cell aging | 1 | 0.04492 |
| GO:0046057 | dADP catabolic process | 1 | 0.04492 |
| GO:0070150 | mitochondrial glycyl-tRNA aminoacylation | 1 | 0.04492 |
| GO:0010476 | gibberellin mediated signaling pathway | 1 | 0.04492 |
| GO:2000691 | negative regulation of cardiac muscle cell myoblast differentiation | 1 | 0.04492 |
| GO:0034441 | plasma lipoprotein particle oxidation | 1 | 0.04492 |
| GO:2000524 | negative regulation of T cell costimulation | 1 | 0.04492 |
| GO:0018272 | protein-pyridoxal-5-phosphate linkage via peptidyl-N6-pyridoxal phosphate-L-lysine | 1 | 0.04492 |
| GO:0001985 | negative regulation of heart rate involved in baroreceptor response to increased systemic arterial blood pressure | 1 | 0.04492 |
| GO:1901249 | regulation of lung goblet cell differentiation | 1 | 0.04492 |
| GO:0019056 | modulation by virus of host transcription | 1 | 0.04492 |
| GO:0038178 | complement component C5a signaling pathway | 1 | 0.04492 |
| GO:1904034 | positive regulation of t-SNARE clustering | 1 | 0.04492 |
| GO:0097252 | oligodendrocyte apoptotic process | 1 | 0.04492 |
| GO:1903249 | negative regulation of citrulline biosynthetic process | 1 | 0.04492 |
| GO:0009184 | purine deoxyribonucleoside diphosphate catabolic process | 1 | 0.04492 |
| GO:0009183 | purine deoxyribonucleoside diphosphate biosynthetic process | 1 | 0.04492 |
| GO:0061141 | lung ciliated cell differentiation | 1 | 0.04492 |
| GO:0099564 | modification of synaptic structure, modulating synaptic transmission | 1 | 0.04492 |
| GO:1904829 | regulation of aortic smooth muscle cell differentiation | 1 | 0.04492 |
| GO:1905355 | spine apparatus assembly | 1 | 0.04492 |
| GO:0071247 | cellular response to chromate | 1 | 0.04492 |
| GO:0038096 | Fc-gamma receptor signaling pathway involved in phagocytosis | 1 | 0.04492 |
| GO:0072377 | blood coagulation, common pathway | 1 | 0.04492 |
| GO:0021511 | spinal cord patterning | 1 | 0.04492 |
| GO:1905169 | regulation of protein localization to phagocytic vesicle | 1 | 0.04492 |
| GO:0031275 | obsolete regulation of lateral pseudopodium assembly | 1 | 0.04492 |
| GO:1901202 | negative regulation of extracellular matrix assembly | 1 | 0.04492 |
| GO:0070684 | seminal clot liquefaction | 1 | 0.04492 |
| GO:0044524 | protein sulfhydration | 1 | 0.04492 |
| GO:0001079 | nitrogen catabolite regulation of transcription from RNA polymerase II promoter | 1 | 0.04492 |
| GO:0106089 | negative regulation of cell adhesion involved in sprouting angiogenesis | 1 | 0.04492 |
| GO:1901250 | negative regulation of lung goblet cell differentiation | 1 | 0.04492 |
| GO:2000761 | positive regulation of N-terminal peptidyl-lysine acetylation | 1 | 0.04492 |
| GO:0006571 | tyrosine biosynthetic process | 1 | 0.04492 |
| GO:0039019 | pronephric nephron development | 1 | 0.04492 |
| GO:0046711 | GDP biosynthetic process | 1 | 0.04492 |
| GO:1905485 | positive regulation of motor neuron migration | 1 | 0.04492 |
| GO:1905483 | regulation of motor neuron migration | 1 | 0.04492 |
| GO:0106088 | regulation of cell adhesion involved in sprouting angiogenesis | 1 | 0.04492 |
| GO:0072277 | metanephric glomerular capillary formation | 1 | 0.04492 |
| GO:0018900 | dichloromethane metabolic process | 1 | 0.04492 |
| GO:0044601 | protein denucleotidylation | 1 | 0.04492 |
| GO:0044602 | protein deadenylylation | 1 | 0.04492 |
| GO:1990092 | calcium-dependent self proteolysis | 1 | 0.04492 |
| GO:1990091 | sodium-dependent self proteolysis | 1 | 0.04492 |
| GO:1905469 | negative regulation of clathrin-coated pit assembly | 1 | 0.04492 |
| GO:1905468 | regulation of clathrin-coated pit assembly | 1 | 0.04492 |
| GO:0036371 | protein localization to T-tubule | 1 | 0.04492 |
| GO:0097510 | base-excision repair, AP site formation via deaminated base removal | 1 | 0.04492 |
| GO:0006808 | regulation of nitrogen utilization | 1 | 0.04492 |
| GO:1905145 | cellular response to acetylcholine | 1 | 0.04492 |
| GO:0140820 | cytosol to Golgi apparatus transport | 1 | 0.04492 |
| GO:0099588 | positive regulation of postsynaptic cytosolic calcium concentration | 1 | 0.04492 |
| GO:0010650 | positive regulation of cell communication by electrical coupling | 1 | 0.04492 |
| GO:1903048 | regulation of acetylcholine-gated cation channel activity | 1 | 0.04492 |
| GO:0043990 | obsolete histone H2A-S1 phosphorylation | 1 | 0.04492 |
| GO:0061837 | neuropeptide processing | 1 | 0.04492 |
| GO:1905162 | regulation of phagosome maturation | 1 | 0.04492 |
| GO:1903392 | negative regulation of adherens junction organization | 1 | 0.04492 |
| GO:0031619 | homologous chromosome orientation involved in meiotic metaphase I plate congression | 1 | 0.04492 |
| GO:2000690 | regulation of cardiac muscle cell myoblast differentiation | 1 | 0.04492 |
| GO:0021730 | trigeminal sensory nucleus development | 1 | 0.04492 |
| GO:1900279 | regulation of CD4-positive, alpha-beta T cell costimulation | 1 | 0.04492 |
| GO:0006426 | glycyl-tRNA aminoacylation | 1 | 0.04492 |
| GO:0009087 | methionine catabolic process | 1 | 0.04492 |
| GO:0060217 | hemangioblast cell differentiation | 1 | 0.04492 |
| GO:1902748 | positive regulation of lens fiber cell differentiation | 1 | 0.04492 |
| GO:0021943 | formation of radial glial scaffolds | 1 | 0.04492 |
| GO:0021888 | hypothalamus gonadotrophin-releasing hormone neuron development | 1 | 0.04492 |
| GO:0051638 | barbed-end actin filament uncapping | 1 | 0.04492 |
| GO:1901331 | positive regulation of odontoblast differentiation | 1 | 0.04492 |
| GO:0033514 | L-lysine catabolic process to acetyl-CoA via L-pipecolate | 1 | 0.04492 |
| GO:2000283 | negative regulation of cellular amino acid biosynthetic process | 1 | 0.04492 |
| GO:0007630 | jump response | 1 | 0.04492 |
| GO:0006948 | induction by virus of host cell-cell fusion | 1 | 0.04492 |
| GO:0021572 | rhombomere 6 development | 1 | 0.04492 |
| GO:2000584 | negative regulation of platelet-derived growth factor receptor-alpha signaling pathway | 1 | 0.04492 |
| GO:0035397 | helper T cell enhancement of adaptive immune response | 1 | 0.04492 |
| GO:0097324 | melanocyte migration | 1 | 0.04492 |
| GO:0090295 | nitrogen catabolite repression of transcription | 1 | 0.04492 |
| GO:0090293 | nitrogen catabolite regulation of transcription | 1 | 0.04492 |
| GO:0006580 | ethanolamine metabolic process | 1 | 0.04492 |
| GO:1905065 | positive regulation of vascular associated smooth muscle cell differentiation | 1 | 0.04492 |
| GO:0060916 | mesenchymal cell proliferation involved in lung development | 1 | 0.04492 |
| GO:0072003 | kidney rudiment formation | 1 | 0.04492 |
| GO:1905271 | regulation of proton-transporting ATP synthase activity, rotational mechanism | 1 | 0.04492 |
| GO:1905273 | positive regulation of proton-transporting ATP synthase activity, rotational mechanism | 1 | 0.04492 |
| GO:0061984 | catabolite repression | 1 | 0.04492 |
| GO:1900081 | regulation of arginine catabolic process | 1 | 0.04492 |
| GO:0019341 | dibenzo-p-dioxin catabolic process | 1 | 0.04492 |
| GO:0032646 | regulation of hepatocyte growth factor production | 1 | 0.04492 |
| GO:2000795 | negative regulation of epithelial cell proliferation involved in lung morphogenesis | 1 | 0.04492 |
| GO:0046067 | dGDP catabolic process | 1 | 0.04492 |
| GO:0036394 | amylase secretion | 1 | 0.04492 |
| GO:1905590 | fibronectin fibril organization | 1 | 0.04492 |
| GO:0090410 | malonate catabolic process | 1 | 0.04492 |
| GO:1904716 | positive regulation of chaperone-mediated autophagy | 1 | 0.04492 |
| GO:0021793 | chemorepulsion of branchiomotor axon | 1 | 0.04492 |
| GO:0052026 | modulation by symbiont of host transcription | 1 | 0.04492 |
| GO:0072592 | oxygen metabolic process | 1 | 0.04492 |
| GO:0019605 | butyrate metabolic process | 1 | 0.04492 |
| GO:0061444 | endocardial cushion cell development | 1 | 0.04492 |
| GO:0072046 | establishment of planar polarity involved in nephron morphogenesis | 1 | 0.04492 |
| GO:0033488 | cholesterol biosynthetic process via 24,25-dihydrolanosterol | 1 | 0.04492 |
| GO:0019290 | siderophore biosynthetic process | 1 | 0.04492 |
| GO:1900280 | negative regulation of CD4-positive, alpha-beta T cell costimulation | 1 | 0.04492 |
| GO:1902283 | negative regulation of primary amine oxidase activity | 1 | 0.04492 |
| GO:0032690 | negative regulation of interleukin-1 alpha production | 1 | 0.04492 |
| GO:0001081 | nitrogen catabolite repression of transcription from RNA polymerase II promoter | 1 | 0.04492 |
| GO:0003142 | cardiogenic plate morphogenesis | 1 | 0.04492 |
| GO:0021633 | optic nerve structural organization | 1 | 0.04492 |
| GO:0021636 | trigeminal nerve morphogenesis | 1 | 0.04492 |
| GO:0051892 | negative regulation of cardioblast differentiation | 1 | 0.04492 |
| GO:0009740 | gibberellic acid mediated signaling pathway | 1 | 0.04492 |
| GO:0061373 | mammillary axonal complex development | 1 | 0.04492 |
| GO:0005993 | trehalose catabolic process | 1 | 0.04492 |
| GO:2001040 | positive regulation of cellular response to drug | 1 | 0.04492 |
| GO:0009192 | deoxyribonucleoside diphosphate catabolic process | 1 | 0.04492 |
| GO:0015783 | GDP-fucose transmembrane transport | 1 | 0.04492 |
| GO:1904205 | negative regulation of skeletal muscle hypertrophy | 1 | 0.04492 |
| GO:0009073 | aromatic amino acid family biosynthetic process | 1 | 0.04492 |
| GO:1900039 | positive regulation of cellular response to hypoxia | 1 | 0.04492 |
| GO:1904681 | response to 3-methylcholanthrene | 1 | 0.04492 |
| GO:0120117 | T cell meandering migration | 1 | 0.04492 |
| GO:2000777 | positive regulation of proteasomal ubiquitin-dependent protein catabolic process involved in cellular response to hypoxia | 1 | 0.04492 |
| GO:0002041 | intussusceptive angiogenesis | 1 | 0.04492 |
| GO:0032686 | negative regulation of hepatocyte growth factor production | 1 | 0.04492 |
| GO:0007576 | obsolete nucleolar fragmentation | 1 | 0.04492 |
| GO:1905492 | positive regulation of branching morphogenesis of a nerve | 1 | 0.04492 |
| GO:1905673 | positive regulation of lysosome organization | 1 | 0.04492 |
| GO:2000759 | regulation of N-terminal peptidyl-lysine acetylation | 1 | 0.04492 |
| GO:1904985 | negative regulation of quinolinate biosynthetic process | 1 | 0.04492 |
| GO:1904984 | regulation of quinolinate biosynthetic process | 1 | 0.04492 |
| GO:0035491 | positive regulation of leukotriene production involved in inflammatory response | 1 | 0.04492 |
| GO:0035490 | regulation of leukotriene production involved in inflammatory response | 1 | 0.04492 |
| GO:0035971 | peptidyl-histidine dephosphorylation | 1 | 0.04492 |
| GO:0009758 | carbohydrate utilization | 1 | 0.04492 |
| GO:0009237 | siderophore metabolic process | 1 | 0.04492 |
| GO:0036085 | GDP-fucose import into Golgi lumen | 1 | 0.04492 |
| GO:0002300 | CD8-positive, alpha-beta intraepithelial T cell differentiation | 1 | 0.04492 |
| GO:0040040 | thermosensory behavior | 1 | 0.04492 |
| GO:0060807 | obsolete regulation of transcription from RNA polymerase II promoter involved in definitive endodermal cell fate specification | 1 | 0.04492 |
| GO:1901639 | obsolete XDP catabolic process | 1 | 0.04492 |
| GO:0061044 | negative regulation of vascular wound healing | 1 | 0.04492 |
| GO:0071976 | cell gliding | 1 | 0.04492 |
| GO:0045751 | negative regulation of Toll signaling pathway | 1 | 0.04492 |
| GO:1900243 | negative regulation of synaptic vesicle endocytosis | 1 | 0.04492 |
| GO:0097501 | stress response to metal ion | 1 | 0.04492 |
| GO:0001970 | positive regulation of activation of membrane attack complex | 1 | 0.04492 |
| GO:1903382 | negative regulation of endoplasmic reticulum stress-induced neuron intrinsic apoptotic signaling pathway | 1 | 0.04492 |
| GO:1903381 | regulation of endoplasmic reticulum stress-induced neuron intrinsic apoptotic signaling pathway | 1 | 0.04492 |
| GO:1902389 | ceramide 1-phosphate transport | 1 | 0.04492 |
| GO:1905438 | non-canonical Wnt signaling pathway involved in midbrain dopaminergic neuron differentiation | 1 | 0.04492 |
| GO:0036324 | vascular endothelial growth factor receptor-2 signaling pathway | 1 | 0.04492 |
| GO:2000043 | regulation of cardiac cell fate specification | 1 | 0.04492 |
| GO:0021589 | cerebellum structural organization | 1 | 0.04492 |
| GO:2000282 | regulation of cellular amino acid biosynthetic process | 1 | 0.04492 |
| GO:1904831 | positive regulation of aortic smooth muscle cell differentiation | 1 | 0.04492 |
| GO:0035964 | COPI-coated vesicle budding | 1 | 0.04492 |
| GO:0009095 | aromatic amino acid family biosynthetic process, prephenate pathway | 1 | 0.04492 |
| GO:0045210 | FasL biosynthetic process | 1 | 0.04492 |
| GO:1905171 | positive regulation of protein localization to phagocytic vesicle | 1 | 0.04492 |
| GO:2000984 | negative regulation of ATP citrate synthase activity | 1 | 0.04492 |
| GO:2000983 | regulation of ATP citrate synthase activity | 1 | 0.04492 |
| GO:0033955 | obsolete mitochondrial DNA inheritance | 1 | 0.04492 |
| GO:0002433 | immune response-regulating cell surface receptor signaling pathway involved in phagocytosis | 1 | 0.04492 |
| GO:0097497 | blood vessel endothelial cell delamination | 1 | 0.04492 |
| GO:1900827 | positive regulation of membrane depolarization during cardiac muscle cell action potential | 1 | 0.04492 |
| GO:0035862 | dITP metabolic process | 1 | 0.04492 |
| GO:0035863 | dITP catabolic process | 1 | 0.04492 |
| GO:0048757 | pigment granule maturation | 1 | 0.04492 |
| GO:1903165 | response to polycyclic arene | 1 | 0.04492 |
| GO:0036494 | positive regulation of translation initiation in response to endoplasmic reticulum stress | 1 | 0.04492 |
| GO:0090480 | purine nucleotide-sugar transmembrane transport | 1 | 0.04492 |
| GO:0019310 | inositol catabolic process | 1 | 0.04492 |
| GO:0052047 | obsolete symbiotic process mediated by secreted substance | 1 | 0.04492 |
| GO:1904032 | regulation of t-SNARE clustering | 1 | 0.04492 |
| GO:0006185 | dGDP biosynthetic process | 1 | 0.04492 |
| GO:0021740 | principal sensory nucleus of trigeminal nerve development | 1 | 0.04492 |
| GO:0046352 | disaccharide catabolic process | 1 | 0.04492 |
| GO:0046359 | butyrate catabolic process | 1 | 0.04492 |
| GO:0060520 | activation of prostate induction by androgen receptor signaling pathway | 1 | 0.04492 |
| GO:1903248 | regulation of citrulline biosynthetic process | 1 | 0.04492 |
| GO:2000295 | regulation of hydrogen peroxide catabolic process | 1 | 0.04492 |
| GO:2000296 | negative regulation of hydrogen peroxide catabolic process | 1 | 0.04492 |
| GO:0061474 | phagolysosome membrane | 1 | 0.04492 |
| GO:0097229 | sperm end piece | 1 | 0.04492 |
| GO:0002144 | cytosolic tRNA wobble base thiouridylase complex | 1 | 0.04492 |
| GO:1990435 | upper tip-link density | 1 | 0.04492 |
| GO:0070369 | beta-catenin-TCF7L2 complex | 1 | 0.04492 |
| GO:0098595 | perivitelline space | 1 | 0.04492 |
| GO:0071752 | secretory dimeric IgA immunoglobulin complex | 1 | 0.04492 |
| GO:0071750 | dimeric IgA immunoglobulin complex | 1 | 0.04492 |
| GO:0071751 | secretory IgA immunoglobulin complex | 1 | 0.04492 |
| GO:0033593 | BRCA2-MAGE-D1 complex | 1 | 0.04492 |
| GO:0032996 | Bcl3-Bcl10 complex | 1 | 0.04492 |
| GO:0000329 | fungal-type vacuole membrane | 1 | 0.04492 |
| GO:1990427 | stereocilia tip-link density | 1 | 0.04492 |
| GO:0031477 | myosin VII complex | 1 | 0.04492 |
| GO:0005586 | collagen type III trimer | 1 | 0.04492 |
| GO:0071757 | hexameric IgM immunoglobulin complex | 1 | 0.04492 |
| GO:0099631 | postsynaptic endocytic zone cytoplasmic component | 1 | 0.04492 |
| GO:0043257 | laminin-8 complex | 1 | 0.04492 |
| GO:0033193 | Lsd1/2 complex | 1 | 0.04492 |
| GO:0005607 | laminin-2 complex | 1 | 0.04492 |
| GO:0071748 | monomeric IgA immunoglobulin complex | 1 | 0.04492 |
| GO:0035000 | oligosaccharyltransferase III complex | 1 | 0.04492 |
| GO:0097541 | axonemal basal plate | 1 | 0.04492 |
| GO:0071749 | polymeric IgA immunoglobulin complex | 1 | 0.04492 |
| GO:0071745 | IgA immunoglobulin complex | 1 | 0.04492 |
| GO:0071746 | IgA immunoglobulin complex, circulating | 1 | 0.04492 |
| GO:0061846 | dendritic spine cytoplasm | 1 | 0.04492 |
| GO:0061845 | neuron projection branch point | 1 | 0.04492 |
| GO:0031309 | integral component of nuclear outer membrane | 1 | 0.04492 |
| GO:0031308 | intrinsic component of nuclear outer membrane | 1 | 0.04492 |
| GO:0070274 | RES complex | 1 | 0.04492 |
| GO:0047127 | thiomorpholine-carboxylate dehydrogenase activity | 1 | 0.04492 |
| GO:0036458 | hepatocyte growth factor binding | 1 | 0.04492 |
| GO:0102340 | 3-oxo-behenoyl-CoA reductase activity | 1 | 0.04492 |
| GO:0044716 | 8-oxo-GDP phosphatase activity | 1 | 0.04492 |
| GO:0044717 | 8-hydroxy-dADP phosphatase activity | 1 | 0.04492 |
| GO:0005457 | GDP-fucose transmembrane transporter activity | 1 | 0.04492 |
| GO:0015067 | amidinotransferase activity | 1 | 0.04492 |
| GO:0102339 | 3-oxo-arachidoyl-CoA reductase activity | 1 | 0.04492 |
| GO:0090409 | malonyl-CoA synthetase activity | 1 | 0.04492 |
| GO:0042586 | peptide deformylase activity | 1 | 0.04492 |
| GO:0043754 | dihydrolipoyllysine-residue (2-methylpropanoyl)transferase activity | 1 | 0.04492 |
| GO:0047273 | galactosylgalactosylglucosylceramide beta-D-acetylgalactosaminyltransferase activity | 1 | 0.04492 |
| GO:0031405 | lipoic acid binding | 1 | 0.04492 |
| GO:0019912 | cyclin-dependent protein kinase activating kinase activity | 1 | 0.04492 |
| GO:0044540 | L-cystine L-cysteine-lyase (deaminating) | 1 | 0.04492 |
| GO:0042012 | interleukin-16 receptor activity | 1 | 0.04492 |
| GO:0004585 | ornithine carbamoyltransferase activity | 1 | 0.04492 |
| GO:0003912 | DNA nucleotidylexotransferase activity | 1 | 0.04492 |
| GO:0015068 | glycine amidinotransferase activity | 1 | 0.04492 |
| GO:0061751 | neutral sphingomyelin phosphodiesterase activity | 1 | 0.04492 |
| GO:0000246 | delta24(24-1) sterol reductase activity | 1 | 0.04492 |
| GO:0031714 | C5a anaphylatoxin chemotactic receptor binding | 1 | 0.04492 |
| GO:0031715 | C5L2 anaphylatoxin chemotactic receptor binding | 1 | 0.04492 |
| GO:0047936 | glucose 1-dehydrogenase [NAD(P)] activity | 1 | 0.04492 |
| GO:0016515 | interleukin-13 receptor activity | 1 | 0.04492 |
| GO:0106345 | glyoxylate reductase activity | 1 | 0.04492 |
| GO:0005006 | epidermal growth factor receptor activity | 1 | 0.04492 |
| GO:0004061 | arylformamidase activity | 1 | 0.04492 |
| GO:0047568 | 3-oxo-5-beta-steroid 4-dehydrogenase activity | 1 | 0.04492 |
| GO:0061710 | L-threonylcarbamoyladenylate synthase | 1 | 0.04492 |
| GO:0004085 | butyryl-CoA dehydrogenase activity | 1 | 0.04492 |
| GO:0050113 | inositol oxygenase activity | 1 | 0.04492 |
| GO:0044603 | protein adenylylhydrolase activity | 1 | 0.04492 |
| GO:0030267 | glyoxylate reductase (NADP+) activity | 1 | 0.04492 |
| GO:0004506 | squalene monooxygenase activity | 1 | 0.04492 |
| GO:0030748 | amine N-methyltransferase activity | 1 | 0.04492 |
| GO:0050459 | ethanolamine-phosphate phospho-lyase activity | 1 | 0.04492 |
| GO:0004121 | cystathionine beta-lyase activity | 1 | 0.04492 |
| GO:0004123 | cystathionine gamma-lyase activity | 1 | 0.04492 |
| GO:0016711 | flavonoid 3'-monooxygenase activity | 1 | 0.04492 |
| GO:1901641 | ITP binding | 1 | 0.04492 |
| GO:1901640 | XTP binding | 1 | 0.04492 |
| GO:0008963 | phospho-N-acetylmuramoyl-pentapeptide-transferase activity | 1 | 0.04492 |
| GO:0080146 | L-cysteine desulfhydrase activity | 1 | 0.04492 |
| GO:0015126 | canalicular bile acid transmembrane transporter activity | 1 | 0.04492 |
| GO:0019120 | hydrolase activity, acting on acid halide bonds, in C-halide compounds | 1 | 0.04492 |
| GO:0050683 | AF-1 domain binding | 1 | 0.04492 |
| GO:0072571 | mono-ADP-D-ribose binding | 1 | 0.04492 |
| GO:0008682 | 3-demethoxyubiquinol 3-hydroxylase activity | 1 | 0.04492 |
| GO:0019807 | aspartoacylase activity | 1 | 0.04492 |
| GO:0030412 | formimidoyltetrahydrofolate cyclodeaminase activity | 1 | 0.04492 |
| GO:0008112 | nicotinamide N-methyltransferase activity | 1 | 0.04492 |
| GO:0008115 | sarcosine oxidase activity | 1 | 0.04492 |
| GO:0005175 | CD27 receptor binding | 1 | 0.04492 |
| GO:0004371 | glycerone kinase activity | 1 | 0.04492 |
| GO:0015491 | obsolete cation:cation antiporter activity | 1 | 0.04492 |
| GO:0102707 | S-adenosyl-L-methionine:beta-alanine N-methyltransferase activity | 1 | 0.04492 |
| GO:0036487 | nitric-oxide synthase inhibitor activity | 1 | 0.04492 |
| GO:0005151 | interleukin-1, type II receptor binding | 1 | 0.04492 |
| GO:0004820 | glycine-tRNA ligase activity | 1 | 0.04492 |
| GO:0008398 | sterol 14-demethylase activity | 1 | 0.04492 |
| GO:0008775 | acetate CoA-transferase activity | 1 | 0.04492 |
| GO:0140566 | histone reader activity | 1 | 0.04492 |
| GO:1990447 | U2 snRNP binding | 1 | 0.04492 |
| GO:0016618 | hydroxypyruvate reductase activity | 1 | 0.04492 |
| GO:0004475 | mannose-1-phosphate guanylyltransferase (GTP) activity | 1 | 0.04492 |
| GO:0050614 | delta24-sterol reductase activity | 1 | 0.04492 |
| GO:0005460 | UDP-glucose transmembrane transporter activity | 1 | 0.04492 |
| GO:0001760 | aminocarboxymuconate-semialdehyde decarboxylase activity | 1 | 0.04492 |
| GO:0070287 | ferritin receptor activity | 1 | 0.04492 |
| GO:1904599 | advanced glycation end-product binding | 1 | 0.04492 |
| GO:0016824 | hydrolase activity, acting on acid halide bonds | 1 | 0.04492 |
| GO:0045353 | interleukin-1 type II receptor antagonist activity | 1 | 0.04492 |
| GO:0045352 | interleukin-1 type I receptor antagonist activity | 1 | 0.04492 |
| GO:0140463 | chromatin-protein adaptor activity | 1 | 0.04492 |
| GO:0034899 | trimethylamine monooxygenase activity | 1 | 0.04492 |
| GO:0001856 | complement component C5a binding | 1 | 0.04492 |
| GO:1904928 | coreceptor activity involved in canonical Wnt signaling pathway | 1 | 0.04492 |
| GO:0102341 | 3-oxo-lignoceroyl-CoA reductase activity | 1 | 0.04492 |
| GO:0102342 | 3-oxo-cerotoyl-CoA reductase activity | 1 | 0.04492 |
| GO:0050561 | glutamate-tRNA(Gln) ligase activity | 1 | 0.04492 |
| GO:0008903 | hydroxypyruvate isomerase activity | 1 | 0.04492 |
| GO:0050354 | triokinase activity | 1 | 0.04492 |
| GO:0051750 | delta(3,5)-delta(2,4)-dienoyl-CoA isomerase activity | 1 | 0.04492 |
| GO:0016524 | latrotoxin receptor activity | 1 | 0.04492 |
| GO:0017125 | deoxycytidyl transferase activity | 1 | 0.04492 |
| GO:0070089 | chloride-activated potassium channel activity | 1 | 0.04492 |
| GO:0047651 | alkylhalidase activity | 1 | 0.04492 |
| GO:0015052 | beta3-adrenergic receptor activity | 1 | 0.04492 |
| GO:0098615 | dimethyl selenide methyltransferase activity | 1 | 0.04492 |
| GO:0005010 | insulin-like growth factor receptor activity | 1 | 0.04492 |
| GO:0035538 | carbohydrate response element binding | 1 | 0.04492 |
| GO:0102264 | tRNA-dihydrouridine20 synthase activity | 1 | 0.04492 |
| GO:0034012 | FAD-AMP lyase (cyclizing) activity | 1 | 0.04492 |
| GO:0050031 | L-pipecolate oxidase activity | 1 | 0.04492 |
| GO:0004070 | aspartate carbamoyltransferase activity | 1 | 0.04492 |
| GO:0018601 | 4-nitrophenol 2-monooxygenase activity | 1 | 0.04492 |
| GO:0003974 | UDP-N-acetylglucosamine 4-epimerase activity | 1 | 0.04492 |
| GO:0102194 | protein-fructosamine 3-kinase activity | 1 | 0.04492 |
| GO:0004692 | cGMP-dependent protein kinase activity | 1 | 0.04492 |
| GO:0102522 | tRNA 4-demethylwyosine alpha-amino-alpha-carboxypropyltransferase activity | 1 | 0.04492 |
| GO:0036080 | purine nucleotide-sugar transmembrane transporter activity | 1 | 0.04492 |
| GO:0050129 | N-formylglutamate deformylase activity | 1 | 0.04492 |
| GO:0016153 | urocanate hydratase activity | 1 | 0.04492 |
| GO:0047395 | glycerophosphoinositol glycerophosphodiesterase activity | 1 | 0.04492 |
| GO:0003975 | UDP-N-acetylglucosamine-dolichyl-phosphate N-acetylglucosaminephosphotransferase activity | 1 | 0.04492 |
| GO:0003978 | UDP-glucose 4-epimerase activity | 1 | 0.04492 |
| GO:0055103 | ligase regulator activity | 1 | 0.04492 |
| GO:0070379 | high mobility group box 1 binding | 1 | 0.04492 |
| GO:0001792 | polymeric immunoglobulin receptor activity | 1 | 0.04492 |
| GO:0008465 | glycerate dehydrogenase activity | 1 | 0.04492 |
| GO:0047444 | N-acylneuraminate-9-phosphate synthase activity | 1 | 0.04492 |
| GO:0030791 | arsenite methyltransferase activity | 1 | 0.04492 |
| GO:0004344 | glucose dehydrogenase activity | 1 | 0.04492 |
| GO:0004151 | dihydroorotase activity | 1 | 0.04492 |
| GO:0033971 | hydroxyisourate hydrolase activity | 1 | 0.04492 |
| GO:0010348 | lithium:proton antiporter activity | 1 | 0.04492 |
| GO:0030409 | glutamate formimidoyltransferase activity | 1 | 0.04492 |
| GO:0004610 | phosphoacetylglucosamine mutase activity | 1 | 0.04492 |
| GO:0042011 | interleukin-16 binding | 1 | 0.04492 |
| GO:0004790 | thioether S-methyltransferase activity | 1 | 0.04492 |
| GO:0019782 | ISG15 activating enzyme activity | 1 | 0.04492 |
| GO:0047290 | (alpha-N-acetylneuraminyl-2,3-beta-galactosyl-1,3)-N-acetyl-galactosaminide 6-alpha-sialyltransferase activity | 1 | 0.04492 |
| GO:0070524 | 11-beta-hydroxysteroid dehydrogenase (NADP+) activity | 1 | 0.04492 |
| GO:0017015 | regulation of transforming growth factor beta receptor signaling pathway | 0.090909 | 0.04461 |
| GO:0002702 | positive regulation of production of molecular mediator of immune response | 0.090909 | 0.04461 |
| GO:0051354 | negative regulation of oxidoreductase activity | 0.166667 | 0.04458 |
| GO:0046386 | deoxyribose phosphate catabolic process | 0.166667 | 0.04458 |
| GO:0060766 | negative regulation of androgen receptor signaling pathway | 0.166667 | 0.04458 |
| GO:0042490 | mechanoreceptor differentiation | 0.166667 | 0.04458 |
| GO:0046348 | amino sugar catabolic process | 0.166667 | 0.04458 |
| GO:0009130 | pyrimidine nucleoside monophosphate biosynthetic process | 0.166667 | 0.04458 |
| GO:0051900 | regulation of mitochondrial depolarization | 0.166667 | 0.04458 |
| GO:0032354 | response to follicle-stimulating hormone | 0.166667 | 0.04458 |
| GO:0098581 | detection of external biotic stimulus | 0.166667 | 0.04458 |
| GO:0070314 | G1 to G0 transition | 0.166667 | 0.04458 |
| GO:2001256 | regulation of store-operated calcium entry | 0.166667 | 0.04458 |
| GO:0051156 | glucose 6-phosphate metabolic process | 0.166667 | 0.04458 |
| GO:2000479 | regulation of cAMP-dependent protein kinase activity | 0.166667 | 0.04458 |
| GO:0001892 | embryonic placenta development | 0.166667 | 0.04458 |
| GO:0043517 | positive regulation of DNA damage response, signal transduction by p53 class mediator | 0.166667 | 0.04458 |
| GO:1904816 | positive regulation of protein localization to chromosome, telomeric region | 0.166667 | 0.04458 |
| GO:0071295 | cellular response to vitamin | 0.166667 | 0.04458 |
| GO:0051004 | regulation of lipoprotein lipase activity | 0.166667 | 0.04458 |
| GO:0042612 | MHC class I protein complex | 0.166667 | 0.04458 |
| GO:0033010 | paranodal junction | 0.166667 | 0.04458 |
| GO:0002161 | aminoacyl-tRNA editing activity | 0.166667 | 0.04458 |
| GO:0017110 | nucleoside diphosphate phosphatase activity | 0.166667 | 0.04458 |
| GO:0004935 | adrenergic receptor activity | 0.166667 | 0.04458 |
| GO:0046916 | cellular transition metal ion homeostasis | 0.084746 | 0.04442 |
| GO:1901699 | cellular response to nitrogen compound | 0.062712 | 0.04440 |
| GO:0048729 | tissue morphogenesis | 0.067024 | 0.04406 |
| GO:0009887 | animal organ morphogenesis | 0.06403 | 0.04402 |
| GO:0042110 | T cell activation | 0.072581 | 0.04396 |
| GO:0003018 | vascular process in circulatory system | 0.081081 | 0.04394 |
| GO:0008360 | regulation of cell shape | 0.081081 | 0.04394 |
| GO:0061050 | regulation of cell growth involved in cardiac muscle cell development | 0.133333 | 0.04389 |
| GO:0009209 | pyrimidine ribonucleoside triphosphate biosynthetic process | 0.133333 | 0.04389 |
| GO:0098761 | cellular response to interleukin-7 | 0.133333 | 0.04389 |
| GO:0098760 | response to interleukin-7 | 0.133333 | 0.04389 |
| GO:0031683 | G-protein beta/gamma-subunit complex binding | 0.133333 | 0.04389 |
| GO:0016849 | phosphorus-oxygen lyase activity | 0.133333 | 0.04389 |
| GO:0004114 | 3',5'-cyclic-nucleotide phosphodiesterase activity | 0.133333 | 0.04389 |
| GO:0016645 | oxidoreductase activity, acting on the CH-NH group of donors | 0.133333 | 0.04389 |
| GO:0019218 | regulation of steroid metabolic process | 0.091837 | 0.04324 |
| GO:0032386 | regulation of intracellular transport | 0.069909 | 0.04315 |
| GO:0006915 | apoptotic process | 0.059081 | 0.04311 |
| GO:0009117 | nucleotide metabolic process | 0.058586 | 0.04304 |
| GO:0071805 | potassium ion transmembrane transport | 0.08547 | 0.04270 |
| GO:0019646 | aerobic electron transport chain | 0.08547 | 0.04270 |
| GO:2000058 | regulation of ubiquitin-dependent protein catabolic process | 0.078652 | 0.04269 |
| GO:0007399 | nervous system development | 0.078652 | 0.04269 |
| GO:0043621 | protein self-association | 0.097222 | 0.04263 |
| GO:0006123 | mitochondrial electron transport, cytochrome c to oxygen | 0.116279 | 0.04261 |
| GO:0070884 | regulation of calcineurin-NFAT signaling cascade | 0.116279 | 0.04261 |
| GO:0038127 | ERBB signaling pathway | 0.116279 | 0.04261 |
| GO:0106056 | regulation of calcineurin-mediated signaling | 0.116279 | 0.04261 |
| GO:1904352 | positive regulation of protein catabolic process in the vacuole | 0.116279 | 0.04261 |
| GO:0140358 | P-type transmembrane transporter activity | 0.116279 | 0.04261 |
| GO:0015662 | P-type ion transporter activity | 0.116279 | 0.04261 |
| GO:0030888 | regulation of B cell proliferation | 0.105263 | 0.04211 |
| GO:2000134 | negative regulation of G1/S transition of mitotic cell cycle | 0.105263 | 0.04211 |
| GO:0006919 | activation of cysteine-type endopeptidase activity involved in apoptotic process | 0.092784 | 0.04197 |
| GO:0006897 | endocytosis | 0.071174 | 0.04162 |
| GO:0006310 | DNA recombination | 0.06812 | 0.04135 |
| GO:0120036 | plasma membrane bounded cell projection organization | 0.059829 | 0.04088 |
| GO:0071495 | cellular response to endogenous stimulus | 0.059351 | 0.04085 |
| GO:0006022 | aminoglycan metabolic process | 0.09375 | 0.04079 |
| GO:0042770 | signal transduction in response to DNA damage | 0.082759 | 0.04061 |
| GO:0031175 | neuron projection development | 0.065502 | 0.04024 |
| GO:0005765 | lysosomal membrane | 0.07767 | 0.03996 |
| GO:0016879 | ligase activity, forming carbon-nitrogen bonds | 0.094737 | 0.03970 |
| GO:0002824 | positive regulation of adaptive immune response based on somatic recombination of immune receptors built from immunoglobulin superfamily domains | 0.086957 | 0.03949 |
| GO:0008652 | cellular amino acid biosynthetic process | 0.086957 | 0.03949 |
| GO:0071294 | cellular response to zinc ion | 0.137931 | 0.03939 |
| GO:0004602 | glutathione peroxidase activity | 0.137931 | 0.03939 |
| GO:0010721 | negative regulation of cell development | 0.080925 | 0.03931 |
| GO:0002889 | regulation of immunoglobulin mediated immune response | 0.107143 | 0.03911 |
| GO:0045620 | negative regulation of lymphocyte differentiation | 0.107143 | 0.03911 |
| GO:0051146 | striated muscle cell differentiation | 0.107143 | 0.03911 |
| GO:0032233 | positive regulation of actin filament bundle assembly | 0.107143 | 0.03911 |
| GO:0072523 | purine-containing compound catabolic process | 0.107143 | 0.03911 |
| GO:0002712 | regulation of B cell mediated immunity | 0.107143 | 0.03911 |
| GO:0035254 | glutamate receptor binding | 0.107143 | 0.03911 |
| GO:0018130 | heterocycle biosynthetic process | 0.05848 | 0.03908 |
| GO:0009147 | pyrimidine nucleoside triphosphate metabolic process | 0.119048 | 0.03905 |
| GO:0045954 | positive regulation of natural killer cell mediated cytotoxicity | 0.119048 | 0.03905 |
| GO:0000959 | mitochondrial RNA metabolic process | 0.119048 | 0.03905 |
| GO:1905167 | positive regulation of lysosomal protein catabolic process | 0.119048 | 0.03905 |
| GO:0006693 | prostaglandin metabolic process | 0.119048 | 0.03905 |
| GO:0006692 | prostanoid metabolic process | 0.119048 | 0.03905 |
| GO:0002717 | positive regulation of natural killer cell mediated immunity | 0.119048 | 0.03905 |
| GO:0032391 | photoreceptor connecting cilium | 0.119048 | 0.03905 |
| GO:0019840 | isoprenoid binding | 0.119048 | 0.03905 |
| GO:0010950 | positive regulation of endopeptidase activity | 0.081395 | 0.03879 |
| GO:1903708 | positive regulation of hemopoiesis | 0.083916 | 0.03870 |
| GO:0043524 | negative regulation of neuron apoptotic process | 0.083916 | 0.03870 |
| GO:1902107 | positive regulation of leukocyte differentiation | 0.083916 | 0.03870 |
| GO:0006978 | DNA damage response, signal transduction by p53 class mediator resulting in transcription of p21 class mediator | 0.176471 | 0.03840 |
| GO:0010755 | regulation of plasminogen activation | 0.176471 | 0.03840 |
| GO:0002293 | alpha-beta T cell differentiation involved in immune response | 0.176471 | 0.03840 |
| GO:0003208 | cardiac ventricle morphogenesis | 0.176471 | 0.03840 |
| GO:0009143 | nucleoside triphosphate catabolic process | 0.176471 | 0.03840 |
| GO:0032823 | regulation of natural killer cell differentiation | 0.176471 | 0.03840 |
| GO:0072359 | circulatory system development | 0.176471 | 0.03840 |
| GO:0060416 | response to growth hormone | 0.176471 | 0.03840 |
| GO:0014002 | astrocyte development | 0.176471 | 0.03840 |
| GO:0009264 | deoxyribonucleotide catabolic process | 0.176471 | 0.03840 |
| GO:0001829 | trophectodermal cell differentiation | 0.176471 | 0.03840 |
| GO:0006901 | vesicle coating | 0.176471 | 0.03840 |
| GO:0072677 | eosinophil migration | 0.176471 | 0.03840 |
| GO:0090330 | regulation of platelet aggregation | 0.176471 | 0.03840 |
| GO:0070203 | regulation of establishment of protein localization to telomere | 0.176471 | 0.03840 |
| GO:0042982 | amyloid precursor protein metabolic process | 0.176471 | 0.03840 |
| GO:2000114 | regulation of establishment of cell polarity | 0.176471 | 0.03840 |
| GO:0050820 | positive regulation of coagulation | 0.176471 | 0.03840 |
| GO:0034143 | regulation of toll-like receptor 4 signaling pathway | 0.176471 | 0.03840 |
| GO:0035089 | establishment of apical/basal cell polarity | 0.176471 | 0.03840 |
| GO:0042789 | mRNA transcription by RNA polymerase II | 0.176471 | 0.03840 |
| GO:0048266 | behavioral response to pain | 0.176471 | 0.03840 |
| GO:0030126 | COPI vesicle coat | 0.176471 | 0.03840 |
| GO:0000815 | ESCRT III complex | 0.176471 | 0.03840 |
| GO:0097449 | astrocyte projection | 0.176471 | 0.03840 |
| GO:0101020 | estrogen 16-alpha-hydroxylase activity | 0.176471 | 0.03840 |
| GO:0033549 | MAP kinase phosphatase activity | 0.176471 | 0.03840 |
| GO:0005381 | iron ion transmembrane transporter activity | 0.176471 | 0.03840 |
| GO:0070492 | oligosaccharide binding | 0.176471 | 0.03840 |
| GO:1901361 | organic cyclic compound catabolic process | 0.067332 | 0.03825 |
| GO:0000122 | negative regulation of transcription by RNA polymerase II | 0.059448 | 0.03808 |
| GO:0033574 | response to testosterone | 0.087719 | 0.03800 |
| GO:1902106 | negative regulation of leukocyte differentiation | 0.087719 | 0.03800 |
| GO:0050728 | negative regulation of inflammatory response | 0.087719 | 0.03800 |
| GO:0032680 | regulation of tumor necrosis factor production | 0.084507 | 0.03784 |
| GO:0003697 | single-stranded DNA binding | 0.084507 | 0.03784 |
| GO:0005901 | caveola | 0.096774 | 0.03782 |
| GO:0032501 | multicellular organismal process | 0.050799 | 0.03758 |
| GO:0031225 | anchored component of membrane | 0.074236 | 0.03743 |
| GO:1904035 | regulation of epithelial cell apoptotic process | 0.1 | 0.03743 |
| GO:0006836 | neurotransmitter transport | 0.1 | 0.03743 |
| GO:0055038 | recycling endosome membrane | 0.1 | 0.03743 |
| GO:0061136 | regulation of proteasomal protein catabolic process | 0.076923 | 0.03712 |
| GO:0006720 | isoprenoid metabolic process | 0.085106 | 0.03704 |
| GO:0009416 | response to light stimulus | 0.071197 | 0.03680 |
| GO:0044389 | ubiquitin-like protein ligase binding | 0.068182 | 0.03679 |
| GO:0009100 | glycoprotein metabolic process | 0.088496 | 0.03658 |
| GO:0060264 | regulation of respiratory burst involved in inflammatory response | 0.285714 | 0.03642 |
| GO:0060267 | positive regulation of respiratory burst | 0.285714 | 0.03642 |
| GO:0006621 | protein retention in ER lumen | 0.285714 | 0.03642 |
| GO:0009698 | phenylpropanoid metabolic process | 0.285714 | 0.03642 |
| GO:1901341 | positive regulation of store-operated calcium channel activity | 0.285714 | 0.03642 |
| GO:1904587 | response to glycoprotein | 0.285714 | 0.03642 |
| GO:0042659 | regulation of cell fate specification | 0.285714 | 0.03642 |
| GO:0031584 | activation of phospholipase D activity | 0.285714 | 0.03642 |
| GO:0070842 | aggresome assembly | 0.285714 | 0.03642 |
| GO:0032811 | negative regulation of epinephrine secretion | 0.285714 | 0.03642 |
| GO:0008655 | pyrimidine-containing compound salvage | 0.285714 | 0.03642 |
| GO:0010616 | negative regulation of cardiac muscle adaptation | 0.285714 | 0.03642 |
| GO:1900747 | negative regulation of vascular endothelial growth factor signaling pathway | 0.285714 | 0.03642 |
| GO:2000480 | negative regulation of cAMP-dependent protein kinase activity | 0.285714 | 0.03642 |
| GO:0048668 | collateral sprouting | 0.285714 | 0.03642 |
| GO:0070995 | NADPH oxidation | 0.285714 | 0.03642 |
| GO:0032262 | pyrimidine nucleotide salvage | 0.285714 | 0.03642 |
| GO:0051409 | response to nitrosative stress | 0.285714 | 0.03642 |
| GO:0045792 | negative regulation of cell size | 0.285714 | 0.03642 |
| GO:0072531 | pyrimidine-containing compound transmembrane transport | 0.285714 | 0.03642 |
| GO:0010138 | pyrimidine ribonucleotide salvage | 0.285714 | 0.03642 |
| GO:0048023 | positive regulation of melanin biosynthetic process | 0.285714 | 0.03642 |
| GO:0019368 | fatty acid elongation, unsaturated fatty acid | 0.285714 | 0.03642 |
| GO:1900378 | positive regulation of secondary metabolite biosynthetic process | 0.285714 | 0.03642 |
| GO:0021517 | ventral spinal cord development | 0.285714 | 0.03642 |
| GO:0003184 | pulmonary valve morphogenesis | 0.285714 | 0.03642 |
| GO:0009804 | coumarin metabolic process | 0.285714 | 0.03642 |
| GO:0003211 | cardiac ventricle formation | 0.285714 | 0.03642 |
| GO:0021534 | cell proliferation in hindbrain | 0.285714 | 0.03642 |
| GO:0046476 | glycosylceramide biosynthetic process | 0.285714 | 0.03642 |
| GO:0046629 | gamma-delta T cell activation | 0.285714 | 0.03642 |
| GO:0001955 | blood vessel maturation | 0.285714 | 0.03642 |
| GO:0009155 | purine deoxyribonucleotide catabolic process | 0.285714 | 0.03642 |
| GO:2000501 | regulation of natural killer cell chemotaxis | 0.285714 | 0.03642 |
| GO:0006198 | cAMP catabolic process | 0.285714 | 0.03642 |
| GO:1904874 | positive regulation of telomerase RNA localization to Cajal body | 0.285714 | 0.03642 |
| GO:0034625 | fatty acid elongation, monounsaturated fatty acid | 0.285714 | 0.03642 |
| GO:0034626 | fatty acid elongation, polyunsaturated fatty acid | 0.285714 | 0.03642 |
| GO:0014012 | peripheral nervous system axon regeneration | 0.285714 | 0.03642 |
| GO:0070841 | inclusion body assembly | 0.285714 | 0.03642 |
| GO:0034374 | low-density lipoprotein particle remodeling | 0.285714 | 0.03642 |
| GO:0014050 | negative regulation of glutamate secretion | 0.285714 | 0.03642 |
| GO:1900221 | regulation of amyloid-beta clearance | 0.285714 | 0.03642 |
| GO:0006069 | ethanol oxidation | 0.285714 | 0.03642 |
| GO:1902527 | positive regulation of protein monoubiquitination | 0.285714 | 0.03642 |
| GO:0044793 | negative regulation by host of viral process | 0.285714 | 0.03642 |
| GO:0018879 | biphenyl metabolic process | 0.285714 | 0.03642 |
| GO:0044206 | UMP salvage | 0.285714 | 0.03642 |
| GO:0044691 | tooth eruption | 0.285714 | 0.03642 |
| GO:0150094 | amyloid-beta clearance by cellular catabolic process | 0.285714 | 0.03642 |
| GO:0098698 | postsynaptic specialization assembly | 0.285714 | 0.03642 |
| GO:0044352 | pinosome | 0.285714 | 0.03642 |
| GO:0044354 | macropinosome | 0.285714 | 0.03642 |
| GO:0031730 | CCR5 chemokine receptor binding | 0.285714 | 0.03642 |
| GO:0102336 | 3-oxo-arachidoyl-CoA synthase activity | 0.285714 | 0.03642 |
| GO:0102338 | 3-oxo-lignoceronyl-CoA synthase activity | 0.285714 | 0.03642 |
| GO:0050733 | RS domain binding | 0.285714 | 0.03642 |
| GO:0001849 | complement component C1q complex binding | 0.285714 | 0.03642 |
| GO:0004024 | alcohol dehydrogenase activity, zinc-dependent | 0.285714 | 0.03642 |
| GO:0048406 | nerve growth factor binding | 0.285714 | 0.03642 |
| GO:0042289 | MHC class II protein binding | 0.285714 | 0.03642 |
| GO:0008499 | UDP-galactose:beta-N-acetylglucosamine beta-1,3-galactosyltransferase activity | 0.285714 | 0.03642 |
| GO:0019798 | procollagen-proline dioxygenase activity | 0.285714 | 0.03642 |
| GO:0102337 | 3-oxo-cerotoyl-CoA synthase activity | 0.285714 | 0.03642 |
| GO:0016151 | nickel cation binding | 0.285714 | 0.03642 |
| GO:1902388 | ceramide 1-phosphate transfer activity | 0.285714 | 0.03642 |
| GO:1902387 | ceramide 1-phosphate binding | 0.285714 | 0.03642 |
| GO:0010823 | negative regulation of mitochondrion organization | 0.109091 | 0.03624 |
| GO:0008286 | insulin receptor signaling pathway | 0.109091 | 0.03624 |
| GO:0035914 | skeletal muscle cell differentiation | 0.109091 | 0.03624 |
| GO:0099738 | cell cortex region | 0.109091 | 0.03624 |
| GO:0042113 | B cell activation | 0.07732 | 0.03619 |
| GO:0070374 | positive regulation of ERK1 and ERK2 cascade | 0.07732 | 0.03619 |
| GO:0009187 | cyclic nucleotide metabolic process | 0.121951 | 0.03567 |
| GO:0031648 | protein destabilization | 0.121951 | 0.03567 |
| GO:0034204 | lipid translocation | 0.121951 | 0.03567 |
| GO:0070227 | lymphocyte apoptotic process | 0.121951 | 0.03567 |
| GO:0006767 | water-soluble vitamin metabolic process | 0.121951 | 0.03567 |
| GO:0032330 | regulation of chondrocyte differentiation | 0.121951 | 0.03567 |
| GO:0051452 | intracellular pH reduction | 0.121951 | 0.03567 |
| GO:0045214 | sarcomere organization | 0.121951 | 0.03567 |
| GO:0098815 | modulation of excitatory postsynaptic potential | 0.121951 | 0.03567 |
| GO:0005751 | mitochondrial respiratory chain complex IV | 0.121951 | 0.03567 |
| GO:0140303 | intramembrane lipid transporter activity | 0.121951 | 0.03567 |
| GO:0046464 | acylglycerol catabolic process | 0.142857 | 0.03517 |
| GO:0046461 | neutral lipid catabolic process | 0.142857 | 0.03517 |
| GO:0008210 | estrogen metabolic process | 0.142857 | 0.03517 |
| GO:1904019 | epithelial cell apoptotic process | 0.142857 | 0.03517 |
| GO:1903205 | regulation of hydrogen peroxide-induced cell death | 0.142857 | 0.03517 |
| GO:0002861 | regulation of inflammatory response to antigenic stimulus | 0.142857 | 0.03517 |
| GO:0050908 | detection of light stimulus involved in visual perception | 0.142857 | 0.03517 |
| GO:0050962 | detection of light stimulus involved in sensory perception | 0.142857 | 0.03517 |
| GO:0051953 | negative regulation of amine transport | 0.142857 | 0.03517 |
| GO:0051968 | positive regulation of synaptic transmission, glutamatergic | 0.142857 | 0.03517 |
| GO:0090025 | regulation of monocyte chemotaxis | 0.142857 | 0.03517 |
| GO:0050996 | positive regulation of lipid catabolic process | 0.142857 | 0.03517 |
| GO:0002026 | regulation of the force of heart contraction | 0.142857 | 0.03517 |
| GO:0030574 | collagen catabolic process | 0.142857 | 0.03517 |
| GO:0046625 | sphingolipid binding | 0.142857 | 0.03517 |
| GO:0016641 | oxidoreductase activity, acting on the CH-NH2 group of donors, oxygen as acceptor | 0.142857 | 0.03517 |
| GO:0051092 | positive regulation of NF-kappaB transcription factor activity | 0.086957 | 0.03501 |
| GO:0042056 | chemoattractant activity | 0.101449 | 0.03499 |
| GO:0012501 | programmed cell death | 0.059184 | 0.03477 |
| GO:0010976 | positive regulation of neuron projection development | 0.078125 | 0.03446 |
| GO:0002687 | positive regulation of leukocyte migration | 0.087591 | 0.03446 |
| GO:0016791 | phosphatase activity | 0.070175 | 0.03435 |
| GO:0023061 | signal release | 0.08125 | 0.03416 |
| GO:0002832 | negative regulation of response to biotic stimulus | 0.09009 | 0.03395 |
| GO:0070925 | organelle assembly | 0.063574 | 0.03392 |
| GO:0051253 | negative regulation of RNA metabolic process | 0.056021 | 0.03391 |
| GO:0045596 | negative regulation of cell differentiation | 0.061438 | 0.03387 |
| GO:0033013 | tetrapyrrole metabolic process | 0.111111 | 0.03351 |
| GO:0016529 | sarcoplasmic reticulum | 0.111111 | 0.03351 |
| GO:0015020 | glucuronosyltransferase activity | 0.111111 | 0.03351 |
| GO:0017111 | ribonucleoside triphosphate phosphatase activity | 0.060606 | 0.03344 |
| GO:0051091 | positive regulation of DNA-binding transcription factor activity | 0.076233 | 0.03309 |
| GO:0021766 | hippocampus development | 0.096386 | 0.03289 |
| GO:0002294 | CD4-positive, alpha-beta T cell differentiation involved in immune response | 0.1875 | 0.03269 |
| GO:0042093 | T-helper cell differentiation | 0.1875 | 0.03269 |
| GO:1900048 | positive regulation of hemostasis | 0.1875 | 0.03269 |
| GO:0002011 | morphogenesis of an epithelial sheet | 0.1875 | 0.03269 |
| GO:0099151 | regulation of postsynaptic density assembly | 0.1875 | 0.03269 |
| GO:0048245 | eosinophil chemotaxis | 0.1875 | 0.03269 |
| GO:0046835 | carbohydrate phosphorylation | 0.1875 | 0.03269 |
| GO:0038084 | vascular endothelial growth factor signaling pathway | 0.1875 | 0.03269 |
| GO:0009074 | aromatic amino acid family catabolic process | 0.1875 | 0.03269 |
| GO:1903975 | regulation of glial cell migration | 0.1875 | 0.03269 |
| GO:0030194 | positive regulation of blood coagulation | 0.1875 | 0.03269 |
| GO:0032292 | peripheral nervous system axon ensheathment | 0.1875 | 0.03269 |
| GO:0071236 | cellular response to antibiotic | 0.1875 | 0.03269 |
| GO:0030259 | lipid glycosylation | 0.1875 | 0.03269 |
| GO:1904851 | positive regulation of establishment of protein localization to telomere | 0.1875 | 0.03269 |
| GO:0090153 | regulation of sphingolipid biosynthetic process | 0.1875 | 0.03269 |
| GO:0022011 | myelination in peripheral nervous system | 0.1875 | 0.03269 |
| GO:1905038 | regulation of membrane lipid metabolic process | 0.1875 | 0.03269 |
| GO:0034755 | iron ion transmembrane transport | 0.1875 | 0.03269 |
| GO:0030137 | COPI-coated vesicle | 0.1875 | 0.03269 |
| GO:0008540 | proteasome regulatory particle, base subcomplex | 0.1875 | 0.03269 |
| GO:0016671 | oxidoreductase activity, acting on a sulfur group of donors, disulfide as acceptor | 0.1875 | 0.03269 |
| GO:0016411 | acylglycerol O-acyltransferase activity | 0.1875 | 0.03269 |
| GO:0004303 | estradiol 17-beta-dehydrogenase activity | 0.1875 | 0.03269 |
| GO:0031402 | sodium ion binding | 0.1875 | 0.03269 |
| GO:0050865 | regulation of cell activation | 0.062229 | 0.03267 |
| GO:0002792 | negative regulation of peptide secretion | 0.102941 | 0.03266 |
| GO:0044843 | cell cycle G1/S phase transition | 0.102941 | 0.03266 |
| GO:0046626 | regulation of insulin receptor signaling pathway | 0.102941 | 0.03266 |
| GO:0090278 | negative regulation of peptide hormone secretion | 0.102941 | 0.03266 |
| GO:0035869 | ciliary transition zone | 0.102941 | 0.03266 |
| GO:0030544 | Hsp70 protein binding | 0.102941 | 0.03266 |
| GO:0045581 | negative regulation of T cell differentiation | 0.125 | 0.03248 |
| GO:0048662 | negative regulation of smooth muscle cell proliferation | 0.125 | 0.03248 |
| GO:0006778 | porphyrin-containing compound metabolic process | 0.125 | 0.03248 |
| GO:0045332 | phospholipid translocation | 0.125 | 0.03248 |
| GO:0004177 | aminopeptidase activity | 0.125 | 0.03248 |
| GO:0016628 | oxidoreductase activity, acting on the CH-CH group of donors, NAD or NADP as acceptor | 0.125 | 0.03248 |
| GO:0010675 | regulation of cellular carbohydrate metabolic process | 0.079365 | 0.03218 |
| GO:0009260 | ribonucleotide biosynthetic process | 0.072165 | 0.03196 |
| GO:0030003 | cellular cation homeostasis | 0.069333 | 0.03185 |
| GO:0048640 | negative regulation of developmental growth | 0.091743 | 0.03161 |
| GO:0098542 | defense response to other organism | 0.056962 | 0.03138 |
| GO:0000186 | obsolete activation of MAPKK activity | 0.148148 | 0.03122 |
| GO:0046823 | negative regulation of nucleocytoplasmic transport | 0.148148 | 0.03122 |
| GO:0045981 | positive regulation of nucleotide metabolic process | 0.148148 | 0.03122 |
| GO:1900544 | positive regulation of purine nucleotide metabolic process | 0.148148 | 0.03122 |
| GO:0019724 | B cell mediated immunity | 0.148148 | 0.03122 |
| GO:0045058 | T cell selection | 0.148148 | 0.03122 |
| GO:0000313 | organellar ribosome | 0.148148 | 0.03122 |
| GO:0005761 | mitochondrial ribosome | 0.148148 | 0.03122 |
| GO:0099604 | ligand-gated calcium channel activity | 0.148148 | 0.03122 |
| GO:0042288 | MHC class I protein binding | 0.148148 | 0.03122 |
| GO:0140326 | ATPase-coupled intramembrane lipid transporter activity | 0.148148 | 0.03122 |
| GO:0016722 | oxidoreductase activity, acting on metal ions | 0.148148 | 0.03122 |
| GO:0010824 | regulation of centrosome duplication | 0.113208 | 0.03092 |
| GO:0071383 | cellular response to steroid hormone stimulus | 0.097561 | 0.03089 |
| GO:1901607 | alpha-amino acid biosynthetic process | 0.092593 | 0.03055 |
| GO:0033177 | proton-transporting two-sector ATPase complex, proton-transporting domain | 0.092593 | 0.03055 |
| GO:0004896 | cytokine receptor activity | 0.092593 | 0.03055 |
| GO:0097553 | calcium ion transmembrane import into cytosol | 0.104478 | 0.03043 |
| GO:0000082 | G1/S transition of mitotic cell cycle | 0.104478 | 0.03043 |
| GO:0005518 | collagen binding | 0.104478 | 0.03043 |
| GO:0034654 | nucleobase-containing compound biosynthetic process | 0.06015 | 0.03028 |
| GO:0008150 | biological_process | 0.045937 | 0.03002 |
| GO:0000287 | magnesium ion binding | 0.073427 | 0.02983 |
| GO:0090257 | regulation of muscle system process | 0.078704 | 0.02968 |
| GO:0001910 | regulation of leukocyte mediated cytotoxicity | 0.093458 | 0.02956 |
| GO:0003725 | double-stranded RNA binding | 0.093458 | 0.02956 |
| GO:0009308 | amine metabolic process | 0.083871 | 0.02953 |
| GO:0046578 | regulation of Ras protein signal transduction | 0.076305 | 0.02950 |
| GO:0002456 | T cell mediated immunity | 0.128205 | 0.02947 |
| GO:0032757 | positive regulation of interleukin-8 production | 0.128205 | 0.02947 |
| GO:0009220 | pyrimidine ribonucleotide biosynthetic process | 0.128205 | 0.02947 |
| GO:0006636 | unsaturated fatty acid biosynthetic process | 0.128205 | 0.02947 |
| GO:2000279 | negative regulation of DNA biosynthetic process | 0.128205 | 0.02947 |
| GO:0055081 | anion homeostasis | 0.128205 | 0.02947 |
| GO:0072659 | protein localization to plasma membrane | 0.081522 | 0.02921 |
| GO:0051302 | regulation of cell division | 0.081522 | 0.02921 |
| GO:0044773 | mitotic DNA damage checkpoint signaling | 0.098765 | 0.02897 |
| GO:0045927 | positive regulation of growth | 0.074205 | 0.02888 |
| GO:1901564 | organonitrogen compound metabolic process | 0.049371 | 0.02872 |
| GO:0002698 | negative regulation of immune effector process | 0.09434 | 0.02863 |
| GO:0015909 | long-chain fatty acid transport | 0.115385 | 0.02847 |
| GO:0031099 | regeneration | 0.082418 | 0.02832 |
| GO:0000323 | lytic vacuole | 0.070028 | 0.02823 |
| GO:0005764 | lysosome | 0.070028 | 0.02823 |
| GO:0006974 | cellular response to DNA damage stimulus | 0.060639 | 0.02818 |
| GO:0033043 | regulation of organelle organization | 0.058382 | 0.02803 |
| GO:0019751 | polyol metabolic process | 0.084967 | 0.02801 |
| GO:0045934 | negative regulation of nucleobase-containing compound metabolic process | 0.055977 | 0.02759 |
| GO:0099560 | synaptic membrane adhesion | 0.153846 | 0.02755 |
| GO:2000463 | positive regulation of excitatory postsynaptic potential | 0.153846 | 0.02755 |
| GO:1903846 | positive regulation of cellular response to transforming growth factor beta stimulus | 0.153846 | 0.02755 |
| GO:0060765 | regulation of androgen receptor signaling pathway | 0.153846 | 0.02755 |
| GO:1900274 | regulation of phospholipase C activity | 0.153846 | 0.02755 |
| GO:0032094 | response to food | 0.153846 | 0.02755 |
| GO:0030511 | positive regulation of transforming growth factor beta receptor signaling pathway | 0.153846 | 0.02755 |
| GO:0006026 | aminoglycan catabolic process | 0.153846 | 0.02755 |
| GO:0009975 | cyclase activity | 0.153846 | 0.02755 |
| GO:1900750 | oligopeptide binding | 0.153846 | 0.02755 |
| GO:0030859 | polarized epithelial cell differentiation | 0.2 | 0.02746 |
| GO:0045019 | negative regulation of nitric oxide biosynthetic process | 0.2 | 0.02746 |
| GO:0032692 | negative regulation of interleukin-1 production | 0.2 | 0.02746 |
| GO:0051298 | centrosome duplication | 0.2 | 0.02746 |
| GO:0051769 | regulation of nitric-oxide synthase biosynthetic process | 0.2 | 0.02746 |
| GO:0003181 | atrioventricular valve morphogenesis | 0.2 | 0.02746 |
| GO:0002862 | negative regulation of inflammatory response to antigenic stimulus | 0.2 | 0.02746 |
| GO:0045760 | positive regulation of action potential | 0.2 | 0.02746 |
| GO:0042308 | negative regulation of protein import into nucleus | 0.2 | 0.02746 |
| GO:0060216 | definitive hemopoiesis | 0.2 | 0.02746 |
| GO:2000303 | regulation of ceramide biosynthetic process | 0.2 | 0.02746 |
| GO:0014733 | regulation of skeletal muscle adaptation | 0.2 | 0.02746 |
| GO:0009151 | purine deoxyribonucleotide metabolic process | 0.2 | 0.02746 |
| GO:0009312 | oligosaccharide biosynthetic process | 0.2 | 0.02746 |
| GO:0099084 | postsynaptic specialization organization | 0.2 | 0.02746 |
| GO:0006047 | UDP-N-acetylglucosamine metabolic process | 0.2 | 0.02746 |
| GO:1904406 | negative regulation of nitric oxide metabolic process | 0.2 | 0.02746 |
| GO:0032591 | dendritic spine membrane | 0.2 | 0.02746 |
| GO:0017017 | MAP kinase tyrosine/serine/threonine phosphatase activity | 0.2 | 0.02746 |
| GO:0016838 | carbon-oxygen lyase activity, acting on phosphates | 0.2 | 0.02746 |
| GO:0008179 | adenylate cyclase binding | 0.2 | 0.02746 |
| GO:0005324 | long-chain fatty acid transporter activity | 0.2 | 0.02746 |
| GO:0016840 | carbon-nitrogen lyase activity | 0.2 | 0.02746 |
| GO:0031901 | early endosome membrane | 0.08871 | 0.02744 |
| GO:0046434 | organophosphate catabolic process | 0.085526 | 0.02732 |
| GO:0019438 | aromatic compound biosynthetic process | 0.059281 | 0.02717 |
| GO:0051963 | regulation of synapse assembly | 0.096154 | 0.02699 |
| GO:0006682 | galactosylceramide biosynthetic process | 0.333333 | 0.02681 |
| GO:0033499 | galactose catabolic process via UDP-galactose | 0.333333 | 0.02681 |
| GO:0014744 | positive regulation of muscle adaptation | 0.333333 | 0.02681 |
| GO:1903243 | negative regulation of cardiac muscle hypertrophy in response to stress | 0.333333 | 0.02681 |
| GO:1900020 | positive regulation of protein kinase C activity | 0.333333 | 0.02681 |
| GO:0071223 | cellular response to lipoteichoic acid | 0.333333 | 0.02681 |
| GO:0061073 | ciliary body morphogenesis | 0.333333 | 0.02681 |
| GO:0140052 | cellular response to oxidised low-density lipoprotein particle stimulus | 0.333333 | 0.02681 |
| GO:0043568 | positive regulation of insulin-like growth factor receptor signaling pathway | 0.333333 | 0.02681 |
| GO:0019626 | short-chain fatty acid catabolic process | 0.333333 | 0.02681 |
| GO:0097187 | dentinogenesis | 0.333333 | 0.02681 |
| GO:0060008 | Sertoli cell differentiation | 0.333333 | 0.02681 |
| GO:0031848 | protection from non-homologous end joining at telomere | 0.333333 | 0.02681 |
| GO:0002467 | germinal center formation | 0.333333 | 0.02681 |
| GO:0001706 | endoderm formation | 0.333333 | 0.02681 |
| GO:0032237 | activation of store-operated calcium channel activity | 0.333333 | 0.02681 |
| GO:0003215 | cardiac right ventricle morphogenesis | 0.333333 | 0.02681 |
| GO:0006651 | diacylglycerol biosynthetic process | 0.333333 | 0.02681 |
| GO:0044351 | macropinocytosis | 0.333333 | 0.02681 |
| GO:0061687 | detoxification of inorganic compound | 0.333333 | 0.02681 |
| GO:0038033 | positive regulation of endothelial cell chemotaxis by VEGF-activated vascular endothelial growth factor receptor signaling pathway | 0.333333 | 0.02681 |
| GO:0010519 | negative regulation of phospholipase activity | 0.333333 | 0.02681 |
| GO:0097090 | presynaptic membrane organization | 0.333333 | 0.02681 |
| GO:0070127 | tRNA aminoacylation for mitochondrial protein translation | 0.333333 | 0.02681 |
| GO:0002578 | negative regulation of antigen processing and presentation | 0.333333 | 0.02681 |
| GO:0002501 | peptide antigen assembly with MHC protein complex | 0.333333 | 0.02681 |
| GO:0061370 | testosterone biosynthetic process | 0.333333 | 0.02681 |
| GO:0015780 | nucleotide-sugar transmembrane transport | 0.333333 | 0.02681 |
| GO:1900019 | regulation of protein kinase C activity | 0.333333 | 0.02681 |
| GO:0039533 | regulation of MDA-5 signaling pathway | 0.333333 | 0.02681 |
| GO:0070391 | response to lipoteichoic acid | 0.333333 | 0.02681 |
| GO:0003330 | regulation of extracellular matrix constituent secretion | 0.333333 | 0.02681 |
| GO:0019375 | galactolipid biosynthetic process | 0.333333 | 0.02681 |
| GO:0051005 | negative regulation of lipoprotein lipase activity | 0.333333 | 0.02681 |
| GO:0072557 | IPAF inflammasome complex | 0.333333 | 0.02681 |
| GO:0070419 | nonhomologous end joining complex | 0.333333 | 0.02681 |
| GO:0001730 | 2'-5'-oligoadenylate synthetase activity | 0.333333 | 0.02681 |
| GO:0008970 | phospholipase A1 activity | 0.333333 | 0.02681 |
| GO:0004645 | 1,4-alpha-oligoglucan phosphorylase activity | 0.333333 | 0.02681 |
| GO:0016812 | hydrolase activity, acting on carbon-nitrogen (but not peptide) bonds, in cyclic amides | 0.333333 | 0.02681 |
| GO:0030346 | protein phosphatase 2B binding | 0.333333 | 0.02681 |
| GO:0004936 | alpha-adrenergic receptor activity | 0.333333 | 0.02681 |
| GO:0010521 | telomerase inhibitor activity | 0.333333 | 0.02681 |
| GO:0016892 | endoribonuclease activity, producing 3'-phosphomonoesters | 0.333333 | 0.02681 |
| GO:0070401 | NADP+ binding | 0.333333 | 0.02681 |
| GO:0003997 | acyl-CoA oxidase activity | 0.333333 | 0.02681 |
| GO:0030942 | endoplasmic reticulum signal peptide binding | 0.333333 | 0.02681 |
| GO:0008390 | testosterone 16-alpha-hydroxylase activity | 0.333333 | 0.02681 |
| GO:0102158 | very-long-chain 3-hydroxyacyl-CoA dehydratase activity | 0.333333 | 0.02681 |
| GO:0102343 | 3-hydroxy-arachidoyl-CoA dehydratase activity | 0.333333 | 0.02681 |
| GO:0102344 | 3-hydroxy-behenoyl-CoA dehydratase activity | 0.333333 | 0.02681 |
| GO:0102345 | 3-hydroxy-lignoceroyl-CoA dehydratase activity | 0.333333 | 0.02681 |
| GO:0005035 | death receptor activity | 0.333333 | 0.02681 |
| GO:0034235 | GPI anchor binding | 0.333333 | 0.02681 |
| GO:0030235 | nitric-oxide synthase regulator activity | 0.333333 | 0.02681 |
| GO:0047035 | testosterone dehydrogenase (NAD+) activity | 0.333333 | 0.02681 |
| GO:0072595 | maintenance of protein localization in organelle | 0.131579 | 0.02665 |
| GO:0006541 | glutamine metabolic process | 0.131579 | 0.02665 |
| GO:0031902 | late endosome membrane | 0.089431 | 0.02636 |
| GO:0042645 | mitochondrial nucleoid | 0.107692 | 0.02629 |
| GO:0009295 | nucleoid | 0.107692 | 0.02629 |
| GO:0010769 | regulation of cell morphogenesis involved in differentiation | 0.097087 | 0.02628 |
| GO:0006873 | cellular ion homeostasis | 0.069231 | 0.02624 |
| GO:0050775 | positive regulation of dendrite morphogenesis | 0.117647 | 0.02614 |
| GO:0009112 | nucleobase metabolic process | 0.117647 | 0.02614 |
| GO:0010812 | negative regulation of cell-substrate adhesion | 0.117647 | 0.02614 |
| GO:0005801 | cis-Golgi network | 0.117647 | 0.02614 |
| GO:1903305 | regulation of regulated secretory pathway | 0.086667 | 0.02609 |
| GO:0030018 | Z disc | 0.086667 | 0.02609 |
| GO:0001952 | regulation of cell-matrix adhesion | 0.098039 | 0.02563 |
| GO:0070555 | response to interleukin-1 | 0.098039 | 0.02563 |
| GO:0006816 | calcium ion transport | 0.079208 | 0.02562 |
| GO:0045087 | innate immune response | 0.061652 | 0.02561 |
| GO:0005634 | nucleus | 0.049717 | 0.02540 |
| GO:1901292 | nucleoside phosphate catabolic process | 0.101266 | 0.02539 |
| GO:0006414 | translational elongation | 0.101266 | 0.02539 |
| GO:0070482 | response to oxygen levels | 0.069767 | 0.02536 |
| GO:0002761 | regulation of myeloid leukocyte differentiation | 0.090164 | 0.02532 |
| GO:0098739 | import across plasma membrane | 0.09901 | 0.02505 |
| GO:0043279 | response to alkaloid | 0.09901 | 0.02505 |
| GO:0004620 | phospholipase activity | 0.09901 | 0.02505 |
| GO:0006955 | immune response | 0.056295 | 0.02505 |
| GO:0006753 | nucleoside phosphate metabolic process | 0.06006 | 0.02463 |
| GO:1901136 | carbohydrate derivative catabolic process | 0.088435 | 0.02459 |
| GO:0002244 | hematopoietic progenitor cell differentiation | 0.088435 | 0.02459 |
| GO:0016324 | apical plasma membrane | 0.070496 | 0.02445 |
| GO:0045685 | regulation of glial cell differentiation | 0.109375 | 0.02437 |
| GO:1900076 | regulation of cellular response to insulin stimulus | 0.109375 | 0.02437 |
| GO:1903050 | regulation of proteolysis involved in protein catabolic process | 0.077586 | 0.02432 |
| GO:0051291 | protein heterooligomerization | 0.089041 | 0.02418 |
| GO:1904893 | negative regulation of receptor signaling pathway via STAT | 0.16 | 0.02415 |
| GO:0090208 | positive regulation of triglyceride metabolic process | 0.16 | 0.02415 |
| GO:0071867 | response to monoamine | 0.16 | 0.02415 |
| GO:0006071 | glycerol metabolic process | 0.16 | 0.02415 |
| GO:0055069 | zinc ion homeostasis | 0.16 | 0.02415 |
| GO:0090162 | establishment of epithelial cell polarity | 0.16 | 0.02415 |
| GO:0002097 | tRNA wobble base modification | 0.16 | 0.02415 |
| GO:0035025 | positive regulation of Rho protein signal transduction | 0.16 | 0.02415 |
| GO:1903792 | negative regulation of anion transport | 0.16 | 0.02415 |
| GO:0046426 | negative regulation of receptor signaling pathway via JAK-STAT | 0.16 | 0.02415 |
| GO:1902991 | regulation of amyloid precursor protein catabolic process | 0.16 | 0.02415 |
| GO:0048710 | regulation of astrocyte differentiation | 0.16 | 0.02415 |
| GO:0001968 | fibronectin binding | 0.16 | 0.02415 |
| GO:0050750 | low-density lipoprotein particle receptor binding | 0.16 | 0.02415 |
| GO:0016405 | CoA-ligase activity | 0.16 | 0.02415 |
| GO:1902476 | chloride transmembrane transport | 0.135135 | 0.02401 |
| GO:0060135 | maternal process involved in female pregnancy | 0.135135 | 0.02401 |
| GO:0009311 | oligosaccharide metabolic process | 0.135135 | 0.02401 |
| GO:0008180 | COP9 signalosome | 0.135135 | 0.02401 |
| GO:0043588 | skin development | 0.12 | 0.02395 |
| GO:0070098 | chemokine-mediated signaling pathway | 0.12 | 0.02395 |
| GO:0060078 | regulation of postsynaptic membrane potential | 0.12 | 0.02395 |
| GO:0045773 | positive regulation of axon extension | 0.12 | 0.02395 |
| GO:0030010 | establishment of cell polarity | 0.102564 | 0.02372 |
| GO:0015849 | organic acid transport | 0.078261 | 0.02344 |
| GO:0046390 | ribose phosphate biosynthetic process | 0.073826 | 0.02336 |
| GO:0046942 | carboxylic acid transport | 0.080808 | 0.02325 |
| GO:0003924 | GTPase activity | 0.067538 | 0.02317 |
| GO:0062013 | positive regulation of small molecule metabolic process | 0.083832 | 0.02286 |
| GO:0016818 | hydrolase activity, acting on acid anhydrides, in phosphorus-containing anhydrides | 0.060908 | 0.02279 |
| GO:0016817 | hydrolase activity, acting on acid anhydrides | 0.060908 | 0.02279 |
| GO:1901072 | glucosamine-containing compound catabolic process | 0.214286 | 0.02271 |
| GO:0010457 | centriole-centriole cohesion | 0.214286 | 0.02271 |
| GO:0070230 | positive regulation of lymphocyte apoptotic process | 0.214286 | 0.02271 |
| GO:0097284 | hepatocyte apoptotic process | 0.214286 | 0.02271 |
| GO:0009173 | pyrimidine ribonucleoside monophosphate metabolic process | 0.214286 | 0.02271 |
| GO:0046459 | short-chain fatty acid metabolic process | 0.214286 | 0.02271 |
| GO:0006020 | inositol metabolic process | 0.214286 | 0.02271 |
| GO:0003407 | neural retina development | 0.214286 | 0.02271 |
| GO:0039532 | negative regulation of viral-induced cytoplasmic pattern recognition receptor signaling pathway | 0.214286 | 0.02271 |
| GO:0031280 | negative regulation of cyclase activity | 0.214286 | 0.02271 |
| GO:0003414 | chondrocyte morphogenesis involved in endochondral bone morphogenesis | 0.214286 | 0.02271 |
| GO:0003422 | growth plate cartilage morphogenesis | 0.214286 | 0.02271 |
| GO:1905153 | regulation of membrane invagination | 0.214286 | 0.02271 |
| GO:0010896 | regulation of triglyceride catabolic process | 0.214286 | 0.02271 |
| GO:0075522 | IRES-dependent viral translational initiation | 0.214286 | 0.02271 |
| GO:0003429 | growth plate cartilage chondrocyte morphogenesis | 0.214286 | 0.02271 |
| GO:0090171 | chondrocyte morphogenesis | 0.214286 | 0.02271 |
| GO:0033270 | paranode region of axon | 0.214286 | 0.02271 |
| GO:0047023 | androsterone dehydrogenase activity | 0.214286 | 0.02271 |
| GO:0004312 | fatty acid synthase activity | 0.214286 | 0.02271 |
| GO:0032589 | neuron projection membrane | 0.111111 | 0.02255 |
| GO:0050772 | positive regulation of axonogenesis | 0.097826 | 0.02251 |
| GO:0032200 | telomere organization | 0.097826 | 0.02251 |
| GO:0000723 | telomere maintenance | 0.097826 | 0.02251 |
| GO:0090068 | positive regulation of cell cycle process | 0.076923 | 0.02241 |
| GO:0016462 | pyrophosphatase activity | 0.061179 | 0.02231 |
| GO:0006399 | tRNA metabolic process | 0.07722 | 0.02215 |
| GO:0051881 | regulation of mitochondrial membrane potential | 0.103896 | 0.02213 |
| GO:0072524 | pyridine-containing compound metabolic process | 0.103896 | 0.02213 |
| GO:1902680 | positive regulation of RNA biosynthetic process | 0.05741 | 0.02204 |
| GO:0045893 | positive regulation of DNA-templated transcription | 0.057448 | 0.02199 |
| GO:1903508 | positive regulation of nucleic acid-templated transcription | 0.057448 | 0.02199 |
| GO:0043113 | receptor clustering | 0.122449 | 0.02188 |
| GO:2000278 | regulation of DNA biosynthetic process | 0.09322 | 0.02173 |
| GO:0045940 | positive regulation of steroid metabolic process | 0.138889 | 0.02153 |
| GO:0019692 | deoxyribose phosphate metabolic process | 0.138889 | 0.02153 |
| GO:0042773 | ATP synthesis coupled electron transport | 0.138889 | 0.02153 |
| GO:0045744 | negative regulation of G protein-coupled receptor signaling pathway | 0.138889 | 0.02153 |
| GO:0008207 | C21-steroid hormone metabolic process | 0.138889 | 0.02153 |
| GO:0032590 | dendrite membrane | 0.138889 | 0.02153 |
| GO:0033017 | sarcoplasmic reticulum membrane | 0.138889 | 0.02153 |
| GO:0042742 | defense response to bacterium | 0.066937 | 0.02152 |
| GO:0043648 | dicarboxylic acid metabolic process | 0.084848 | 0.02152 |
| GO:0045944 | positive regulation of transcription by RNA polymerase II | 0.059432 | 0.02120 |
| GO:0005509 | calcium ion binding | 0.060158 | 0.02118 |
| GO:0043235 | receptor complex | 0.071429 | 0.02110 |
| GO:0032535 | regulation of cellular component size | 0.07362 | 0.02109 |
| GO:0018126 | protein hydroxylation | 0.166667 | 0.02101 |
| GO:0010863 | positive regulation of phospholipase C activity | 0.166667 | 0.02101 |
| GO:0071869 | response to catecholamine | 0.166667 | 0.02101 |
| GO:0010738 | regulation of protein kinase A signaling | 0.166667 | 0.02101 |
| GO:0009415 | response to water | 0.166667 | 0.02101 |
| GO:0097440 | apical dendrite | 0.166667 | 0.02101 |
| GO:0031233 | intrinsic component of external side of plasma membrane | 0.166667 | 0.02101 |
| GO:0005797 | Golgi medial cisterna | 0.166667 | 0.02101 |
| GO:0043295 | glutathione binding | 0.166667 | 0.02101 |
| GO:0002688 | regulation of leukocyte chemotaxis | 0.094017 | 0.02096 |
| GO:0042180 | cellular ketone metabolic process | 0.094017 | 0.02096 |
| GO:0031625 | ubiquitin protein ligase binding | 0.071625 | 0.02085 |
| GO:0045912 | negative regulation of carbohydrate metabolic process | 0.112903 | 0.02083 |
| GO:0048839 | inner ear development | 0.112903 | 0.02083 |
| GO:0098754 | detoxification | 0.112903 | 0.02083 |
| GO:0043280 | positive regulation of cysteine-type endopeptidase activity involved in apoptotic process | 0.088889 | 0.02079 |
| GO:0046677 | response to antibiotic | 0.105263 | 0.02061 |
| GO:0044291 | cell-cell contact zone | 0.105263 | 0.02061 |
| GO:0044282 | small molecule catabolic process | 0.064364 | 0.02059 |
| GO:1903707 | negative regulation of hemopoiesis | 0.094828 | 0.02024 |
| GO:0071407 | cellular response to organic cyclic compound | 0.067762 | 0.02015 |
| GO:0030308 | negative regulation of cell growth | 0.084211 | 0.02006 |
| GO:0140678 | molecular function inhibitor activity | 0.125 | 0.01993 |
| GO:1901618 | organic hydroxy compound transmembrane transporter activity | 0.125 | 0.01993 |
| GO:0005160 | transforming growth factor beta receptor binding | 0.125 | 0.01993 |
| GO:0002690 | positive regulation of leukocyte chemotaxis | 0.1 | 0.01979 |
| GO:0005125 | cytokine activity | 0.075269 | 0.01965 |
| GO:1903828 | negative regulation of protein localization | 0.079812 | 0.01959 |
| GO:0097746 | blood vessel diameter maintenance | 0.095652 | 0.01958 |
| GO:1903052 | positive regulation of proteolysis involved in protein catabolic process | 0.095652 | 0.01958 |
| GO:0035296 | regulation of tube diameter | 0.095652 | 0.01958 |
| GO:0007346 | regulation of mitotic cell cycle | 0.066667 | 0.01946 |
| GO:0007169 | transmembrane receptor protein tyrosine kinase signaling pathway | 0.073482 | 0.01942 |
| GO:0009148 | pyrimidine nucleoside triphosphate biosynthetic process | 0.142857 | 0.01924 |
| GO:0033144 | negative regulation of intracellular steroid hormone receptor signaling pathway | 0.142857 | 0.01924 |
| GO:0014742 | positive regulation of muscle hypertrophy | 0.142857 | 0.01924 |
| GO:0050687 | negative regulation of defense response to virus | 0.142857 | 0.01924 |
| GO:0021510 | spinal cord development | 0.142857 | 0.01924 |
| GO:0070566 | adenylyltransferase activity | 0.142857 | 0.01924 |
| GO:0016863 | intramolecular oxidoreductase activity, transposing C=C bonds | 0.142857 | 0.01924 |
| GO:0098552 | side of membrane | 0.064611 | 0.01914 |
| GO:0016810 | hydrolase activity, acting on carbon-nitrogen (but not peptide) bonds | 0.077869 | 0.01899 |
| GO:0050770 | regulation of axonogenesis | 0.0875 | 0.01879 |
| GO:1901653 | cellular response to peptide | 0.078189 | 0.01857 |
| GO:0005762 | mitochondrial large ribosomal subunit | 0.101124 | 0.01853 |
| GO:0000315 | organellar large ribosomal subunit | 0.101124 | 0.01853 |
| GO:0008144 | obsolete drug binding | 0.101124 | 0.01853 |
| GO:0060263 | regulation of respiratory burst | 0.230769 | 0.01845 |
| GO:0007194 | negative regulation of adenylate cyclase activity | 0.230769 | 0.01845 |
| GO:0050872 | white fat cell differentiation | 0.230769 | 0.01845 |
| GO:0051956 | negative regulation of amino acid transport | 0.230769 | 0.01845 |
| GO:0045198 | establishment of epithelial cell apical/basal polarity | 0.230769 | 0.01845 |
| GO:0048532 | anatomical structure arrangement | 0.230769 | 0.01845 |
| GO:0036336 | dendritic cell migration | 0.230769 | 0.01845 |
| GO:0046049 | UMP metabolic process | 0.230769 | 0.01845 |
| GO:0016093 | polyprenol metabolic process | 0.230769 | 0.01845 |
| GO:1990000 | amyloid fibril formation | 0.230769 | 0.01845 |
| GO:0009191 | ribonucleoside diphosphate catabolic process | 0.230769 | 0.01845 |
| GO:0071257 | cellular response to electrical stimulus | 0.230769 | 0.01845 |
| GO:1900016 | negative regulation of cytokine production involved in inflammatory response | 0.230769 | 0.01845 |
| GO:0042301 | phosphate ion binding | 0.230769 | 0.01845 |
| GO:0016679 | oxidoreductase activity, acting on diphenols and related substances as donors | 0.230769 | 0.01845 |
| GO:0032393 | MHC class I receptor activity | 0.230769 | 0.01845 |
| GO:0015271 | outward rectifier potassium channel activity | 0.230769 | 0.01845 |
| GO:0010757 | negative regulation of plasminogen activation | 0.4 | 0.01842 |
| GO:0032650 | regulation of interleukin-1 alpha production | 0.4 | 0.01842 |
| GO:0070291 | N-acylethanolamine metabolic process | 0.4 | 0.01842 |
| GO:0051918 | negative regulation of fibrinolysis | 0.4 | 0.01842 |
| GO:0060662 | salivary gland cavitation | 0.4 | 0.01842 |
| GO:1903207 | regulation of hydrogen peroxide-induced neuron death | 0.4 | 0.01842 |
| GO:1903208 | negative regulation of hydrogen peroxide-induced neuron death | 0.4 | 0.01842 |
| GO:0070327 | thyroid hormone transport | 0.4 | 0.01842 |
| GO:0060605 | tube lumen cavitation | 0.4 | 0.01842 |
| GO:0097049 | motor neuron apoptotic process | 0.4 | 0.01842 |
| GO:0051045 | negative regulation of membrane protein ectodomain proteolysis | 0.4 | 0.01842 |
| GO:0001554 | luteolysis | 0.4 | 0.01842 |
| GO:0060510 | type II pneumocyte differentiation | 0.4 | 0.01842 |
| GO:0099179 | regulation of synaptic membrane adhesion | 0.4 | 0.01842 |
| GO:0061622 | glycolytic process through glucose-1-phosphate | 0.4 | 0.01842 |
| GO:1905232 | cellular response to L-glutamate | 0.4 | 0.01842 |
| GO:0097105 | presynaptic membrane assembly | 0.4 | 0.01842 |
| GO:1901164 | negative regulation of trophoblast cell migration | 0.4 | 0.01842 |
| GO:0042088 | T-helper 1 type immune response | 0.4 | 0.01842 |
| GO:2000807 | regulation of synaptic vesicle clustering | 0.4 | 0.01842 |
| GO:0060136 | embryonic process involved in female pregnancy | 0.4 | 0.01842 |
| GO:0021532 | neural tube patterning | 0.4 | 0.01842 |
| GO:2000659 | regulation of interleukin-1-mediated signaling pathway | 0.4 | 0.01842 |
| GO:2000304 | positive regulation of ceramide biosynthetic process | 0.4 | 0.01842 |
| GO:2000503 | positive regulation of natural killer cell chemotaxis | 0.4 | 0.01842 |
| GO:0051152 | positive regulation of smooth muscle cell differentiation | 0.4 | 0.01842 |
| GO:0018057 | peptidyl-lysine oxidation | 0.4 | 0.01842 |
| GO:0008592 | regulation of Toll signaling pathway | 0.4 | 0.01842 |
| GO:0051343 | positive regulation of cyclic-nucleotide phosphodiesterase activity | 0.4 | 0.01842 |
| GO:0006548 | histidine catabolic process | 0.4 | 0.01842 |
| GO:0002583 | regulation of antigen processing and presentation of peptide antigen | 0.4 | 0.01842 |
| GO:0015942 | formate metabolic process | 0.4 | 0.01842 |
| GO:0090154 | positive regulation of sphingolipid biosynthetic process | 0.4 | 0.01842 |
| GO:0006083 | acetate metabolic process | 0.4 | 0.01842 |
| GO:0015677 | copper ion import | 0.4 | 0.01842 |
| GO:0031120 | snRNA pseudouridine synthesis | 0.4 | 0.01842 |
| GO:0097118 | neuroligin clustering involved in postsynaptic membrane assembly | 0.4 | 0.01842 |
| GO:0015739 | sialic acid transport | 0.4 | 0.01842 |
| GO:0005638 | lamin filament | 0.4 | 0.01842 |
| GO:0005588 | collagen type V trimer | 0.4 | 0.01842 |
| GO:0018812 | 3-hydroxyacyl-CoA dehydratase activity | 0.4 | 0.01842 |
| GO:0016623 | oxidoreductase activity, acting on the aldehyde or oxo group of donors, oxygen as acceptor | 0.4 | 0.01842 |
| GO:0033265 | choline binding | 0.4 | 0.01842 |
| GO:0004396 | hexokinase activity | 0.4 | 0.01842 |
| GO:0004720 | protein-lysine 6-oxidase activity | 0.4 | 0.01842 |
| GO:0048403 | brain-derived neurotrophic factor binding | 0.4 | 0.01842 |
| GO:0016841 | ammonia-lyase activity | 0.4 | 0.01842 |
| GO:1990239 | steroid hormone binding | 0.4 | 0.01842 |
| GO:0004031 | aldehyde oxidase activity | 0.4 | 0.01842 |
| GO:0005031 | tumor necrosis factor receptor activity | 0.4 | 0.01842 |
| GO:0071723 | lipopeptide binding | 0.4 | 0.01842 |
| GO:0019153 | protein-disulfide reductase (glutathione) activity | 0.4 | 0.01842 |
| GO:0023024 | MHC class I protein complex binding | 0.4 | 0.01842 |
| GO:0046822 | regulation of nucleocytoplasmic transport | 0.097345 | 0.01840 |
| GO:0006882 | cellular zinc ion homeostasis | 0.173913 | 0.01814 |
| GO:0009068 | aspartate family amino acid catabolic process | 0.173913 | 0.01814 |
| GO:0010464 | regulation of mesenchymal cell proliferation | 0.173913 | 0.01814 |
| GO:0032891 | negative regulation of organic acid transport | 0.173913 | 0.01814 |
| GO:1903580 | positive regulation of ATP metabolic process | 0.173913 | 0.01814 |
| GO:0002082 | regulation of oxidative phosphorylation | 0.173913 | 0.01814 |
| GO:0070861 | regulation of protein exit from endoplasmic reticulum | 0.173913 | 0.01814 |
| GO:0017156 | calcium-ion regulated exocytosis | 0.12766 | 0.01811 |
| GO:2001238 | positive regulation of extrinsic apoptotic signaling pathway | 0.12766 | 0.01811 |
| GO:0032814 | regulation of natural killer cell activation | 0.12766 | 0.01811 |
| GO:0051591 | response to cAMP | 0.098214 | 0.01788 |
| GO:0071347 | cellular response to interleukin-1 | 0.108108 | 0.01781 |
| GO:0098657 | import into cell | 0.091603 | 0.01755 |
| GO:0031644 | regulation of nervous system process | 0.089744 | 0.01725 |
| GO:0010613 | positive regulation of cardiac muscle hypertrophy | 0.147059 | 0.01710 |
| GO:1901031 | regulation of response to reactive oxygen species | 0.147059 | 0.01710 |
| GO:0032816 | positive regulation of natural killer cell activation | 0.147059 | 0.01710 |
| GO:0009394 | 2'-deoxyribonucleotide metabolic process | 0.147059 | 0.01710 |
| GO:0016248 | channel inhibitor activity | 0.147059 | 0.01710 |
| GO:0098852 | lytic vacuole membrane | 0.082126 | 0.01676 |
| GO:0001817 | regulation of cytokine production | 0.062429 | 0.01674 |
| GO:0097190 | apoptotic signaling pathway | 0.078067 | 0.01660 |
| GO:0032543 | mitochondrial translation | 0.109589 | 0.01651 |
| GO:0051965 | positive regulation of synapse assembly | 0.109589 | 0.01651 |
| GO:0031638 | zymogen activation | 0.109589 | 0.01651 |
| GO:0090317 | negative regulation of intracellular protein transport | 0.130435 | 0.01641 |
| GO:0051155 | positive regulation of striated muscle cell differentiation | 0.130435 | 0.01641 |
| GO:0030330 | DNA damage response, signal transduction by p53 class mediator | 0.130435 | 0.01641 |
| GO:0051602 | response to electrical stimulus | 0.130435 | 0.01641 |
| GO:0090288 | negative regulation of cellular response to growth factor stimulus | 0.103448 | 0.01617 |
| GO:0035556 | intracellular signal transduction | 0.056757 | 0.01581 |
| GO:0005543 | phospholipid binding | 0.068548 | 0.01579 |
| GO:0042445 | hormone metabolic process | 0.085714 | 0.01572 |
| GO:0005938 | cell cortex | 0.081545 | 0.01566 |
| GO:0030163 | protein catabolic process | 0.068687 | 0.01565 |
| GO:0010628 | positive regulation of gene expression | 0.058955 | 0.01563 |
| GO:0002708 | positive regulation of lymphocyte mediated immunity | 0.09375 | 0.01555 |
| GO:0055067 | monovalent inorganic cation homeostasis | 0.09375 | 0.01555 |
| GO:0007202 | activation of phospholipase C activity | 0.181818 | 0.01552 |
| GO:0042908 | xenobiotic transport | 0.181818 | 0.01552 |
| GO:0048641 | regulation of skeletal muscle tissue development | 0.181818 | 0.01552 |
| GO:0045686 | negative regulation of glial cell differentiation | 0.181818 | 0.01552 |
| GO:0055083 | monovalent inorganic anion homeostasis | 0.181818 | 0.01552 |
| GO:0009129 | pyrimidine nucleoside monophosphate metabolic process | 0.181818 | 0.01552 |
| GO:0048731 | system development | 0.06729 | 0.01551 |
| GO:0007599 | hemostasis | 0.111111 | 0.01529 |
| GO:0071219 | cellular response to molecule of bacterial origin | 0.075862 | 0.01524 |
| GO:0098857 | membrane microdomain | 0.071429 | 0.01521 |
| GO:1903555 | regulation of tumor necrosis factor superfamily cytokine production | 0.089655 | 0.01518 |
| GO:1904062 | regulation of cation transmembrane transport | 0.074074 | 0.01516 |
| GO:0032940 | secretion by cell | 0.073569 | 0.01516 |
| GO:0055075 | potassium ion homeostasis | 0.151515 | 0.01513 |
| GO:0099174 | regulation of presynapse organization | 0.151515 | 0.01513 |
| GO:0006110 | regulation of glycolytic process | 0.151515 | 0.01513 |
| GO:1901861 | regulation of muscle tissue development | 0.151515 | 0.01513 |
| GO:0003014 | renal system process | 0.104651 | 0.01508 |
| GO:0043679 | axon terminus | 0.094488 | 0.01496 |
| GO:0045121 | membrane raft | 0.071782 | 0.01488 |
| GO:0036293 | response to decreased oxygen levels | 0.074303 | 0.01486 |
| GO:0019229 | regulation of vasoconstriction | 0.12069 | 0.01484 |
| GO:0042306 | regulation of protein import into nucleus | 0.12069 | 0.01484 |
| GO:0009409 | response to cold | 0.12069 | 0.01484 |
| GO:0001837 | epithelial to mesenchymal transition | 0.133333 | 0.01481 |
| GO:0050679 | positive regulation of epithelial cell proliferation | 0.086705 | 0.01478 |
| GO:0010544 | negative regulation of platelet activation | 0.25 | 0.01467 |
| GO:0010269 | response to selenium ion | 0.25 | 0.01467 |
| GO:0090331 | negative regulation of platelet aggregation | 0.25 | 0.01467 |
| GO:0018158 | protein oxidation | 0.25 | 0.01467 |
| GO:0009174 | pyrimidine ribonucleoside monophosphate biosynthetic process | 0.25 | 0.01467 |
| GO:0046689 | response to mercury ion | 0.25 | 0.01467 |
| GO:0044065 | regulation of respiratory system process | 0.25 | 0.01467 |
| GO:0019682 | glyceraldehyde-3-phosphate metabolic process | 0.25 | 0.01467 |
| GO:1904181 | positive regulation of membrane depolarization | 0.25 | 0.01467 |
| GO:0043471 | regulation of cellular carbohydrate catabolic process | 0.25 | 0.01467 |
| GO:0006222 | UMP biosynthetic process | 0.25 | 0.01467 |
| GO:0071305 | cellular response to vitamin D | 0.25 | 0.01467 |
| GO:0033179 | proton-transporting V-type ATPase, V0 domain | 0.25 | 0.01467 |
| GO:0030915 | Smc5-Smc6 complex | 0.25 | 0.01467 |
| GO:0008195 | phosphatidate phosphatase activity | 0.25 | 0.01467 |
| GO:1990778 | protein localization to cell periphery | 0.080717 | 0.01460 |
| GO:0008134 | transcription factor binding | 0.063694 | 0.01451 |
| GO:0071356 | cellular response to tumor necrosis factor | 0.095238 | 0.01441 |
| GO:0051056 | regulation of small GTPase mediated signal transduction | 0.076655 | 0.01428 |
| GO:1903008 | organelle disassembly | 0.112676 | 0.01413 |
| GO:0033143 | regulation of intracellular steroid hormone receptor signaling pathway | 0.112676 | 0.01413 |
| GO:0051258 | protein polymerization | 0.112676 | 0.01413 |
| GO:0004364 | glutathione transferase activity | 0.112676 | 0.01413 |
| GO:0055092 | sterol homeostasis | 0.105882 | 0.01405 |
| GO:0010770 | positive regulation of cell morphogenesis involved in differentiation | 0.105882 | 0.01405 |
| GO:1901890 | positive regulation of cell junction assembly | 0.10101 | 0.01357 |
| GO:0032092 | positive regulation of protein binding | 0.10101 | 0.01357 |
| GO:0016782 | transferase activity, transferring sulphur-containing groups | 0.10101 | 0.01357 |
| GO:0046605 | regulation of centrosome cycle | 0.122807 | 0.01356 |
| GO:0043198 | dendritic shaft | 0.122807 | 0.01356 |
| GO:0071222 | cellular response to lipopolysaccharide | 0.077465 | 0.01346 |
| GO:1902105 | regulation of leukocyte differentiation | 0.077465 | 0.01346 |
| GO:0036296 | response to increased oxygen levels | 0.15625 | 0.01332 |
| GO:0001848 | complement binding | 0.15625 | 0.01332 |
| GO:0030855 | epithelial cell differentiation | 0.07571 | 0.01332 |
| GO:0005178 | integrin binding | 0.091549 | 0.01329 |
| GO:0005802 | trans-Golgi network | 0.084656 | 0.01323 |
| GO:0071868 | cellular response to monoamine stimulus | 0.190476 | 0.01316 |
| GO:0045723 | positive regulation of fatty acid biosynthetic process | 0.190476 | 0.01316 |
| GO:1905874 | regulation of postsynaptic density organization | 0.190476 | 0.01316 |
| GO:0009595 | detection of biotic stimulus | 0.190476 | 0.01316 |
| GO:0090312 | positive regulation of protein deacetylation | 0.190476 | 0.01316 |
| GO:0032461 | positive regulation of protein oligomerization | 0.190476 | 0.01316 |
| GO:0014048 | regulation of glutamate secretion | 0.190476 | 0.01316 |
| GO:0055064 | chloride ion homeostasis | 0.190476 | 0.01316 |
| GO:0046058 | cAMP metabolic process | 0.190476 | 0.01316 |
| GO:1904667 | negative regulation of ubiquitin protein ligase activity | 0.190476 | 0.01316 |
| GO:0060716 | labyrinthine layer blood vessel development | 0.190476 | 0.01316 |
| GO:0042632 | cholesterol homeostasis | 0.107143 | 0.01306 |
| GO:0050688 | regulation of defense response to virus | 0.107143 | 0.01306 |
| GO:2001237 | negative regulation of extrinsic apoptotic signaling pathway | 0.107143 | 0.01306 |
| GO:0050817 | coagulation | 0.114286 | 0.01304 |
| GO:0007596 | blood coagulation | 0.114286 | 0.01304 |
| GO:0044853 | plasma membrane raft | 0.097561 | 0.01297 |
| GO:0002695 | negative regulation of leukocyte activation | 0.089286 | 0.01290 |
| GO:0044237 | cellular metabolic process | 0.048909 | 0.01282 |
| GO:1901796 | regulation of signal transduction by p53 class mediator | 0.102041 | 0.01270 |
| GO:0002443 | leukocyte mediated immunity | 0.102041 | 0.01270 |
| GO:0062014 | negative regulation of small molecule metabolic process | 0.102041 | 0.01270 |
| GO:0071216 | cellular response to biotic stimulus | 0.076677 | 0.01257 |
| GO:0051147 | regulation of muscle cell differentiation | 0.098361 | 0.01256 |
| GO:0005096 | GTPase activator activity | 0.074928 | 0.01243 |
| GO:0032677 | regulation of interleukin-8 production | 0.125 | 0.01236 |
| GO:0098858 | actin-based cell projection | 0.085561 | 0.01232 |
| GO:0030545 | signaling receptor regulator activity | 0.065217 | 0.01225 |
| GO:0002573 | myeloid leukocyte differentiation | 0.108434 | 0.01213 |
| GO:0019217 | regulation of fatty acid metabolic process | 0.108434 | 0.01213 |
| GO:0017157 | regulation of exocytosis | 0.083333 | 0.01205 |
| GO:1902414 | protein localization to cell junction | 0.115942 | 0.01200 |
| GO:0070373 | negative regulation of ERK1 and ERK2 cascade | 0.115942 | 0.01200 |
| GO:0001917 | photoreceptor inner segment | 0.115942 | 0.01200 |
| GO:0019400 | alditol metabolic process | 0.139535 | 0.01196 |
| GO:0050873 | brown fat cell differentiation | 0.139535 | 0.01196 |
| GO:0001659 | temperature homeostasis | 0.139535 | 0.01196 |
| GO:0070207 | protein homotrimerization | 0.139535 | 0.01196 |
| GO:0009262 | deoxyribonucleotide metabolic process | 0.139535 | 0.01196 |
| GO:0097386 | glial cell projection | 0.139535 | 0.01196 |
| GO:2000060 | positive regulation of ubiquitin-dependent protein catabolic process | 0.103093 | 0.01187 |
| GO:0070069 | cytochrome complex | 0.103093 | 0.01187 |
| GO:0043281 | regulation of cysteine-type endopeptidase activity involved in apoptotic process | 0.083721 | 0.01177 |
| GO:2000116 | regulation of cysteine-type endopeptidase activity | 0.081967 | 0.01170 |
| GO:1901606 | alpha-amino acid catabolic process | 0.093525 | 0.01167 |
| GO:1905606 | regulation of presynapse assembly | 0.16129 | 0.01166 |
| GO:0034698 | response to gonadotropin | 0.16129 | 0.01166 |
| GO:0010830 | regulation of myotube differentiation | 0.16129 | 0.01166 |
| GO:0060674 | placenta blood vessel development | 0.16129 | 0.01166 |
| GO:0031210 | phosphatidylcholine binding | 0.16129 | 0.01166 |
| GO:0061024 | membrane organization | 0.065789 | 0.01161 |
| GO:0043152 | induction of bacterial agglutination | 0.5 | 0.01139 |
| GO:0046465 | dolichyl diphosphate metabolic process | 0.5 | 0.01139 |
| GO:0090071 | negative regulation of ribosome biogenesis | 0.5 | 0.01139 |
| GO:2001012 | mesenchymal cell differentiation involved in renal system development | 0.5 | 0.01139 |
| GO:0045343 | regulation of MHC class I biosynthetic process | 0.5 | 0.01139 |
| GO:0045345 | positive regulation of MHC class I biosynthetic process | 0.5 | 0.01139 |
| GO:0090289 | regulation of osteoclast proliferation | 0.5 | 0.01139 |
| GO:0009189 | deoxyribonucleoside diphosphate biosynthetic process | 0.5 | 0.01139 |
| GO:0018894 | dibenzo-p-dioxin metabolic process | 0.5 | 0.01139 |
| GO:0019557 | histidine catabolic process to glutamate and formate | 0.5 | 0.01139 |
| GO:0046710 | GDP metabolic process | 0.5 | 0.01139 |
| GO:0015917 | aminophospholipid transport | 0.5 | 0.01139 |
| GO:0009609 | response to symbiotic bacterium | 0.5 | 0.01139 |
| GO:0009608 | response to symbiont | 0.5 | 0.01139 |
| GO:0097466 | ubiquitin-dependent glycoprotein ERAD pathway | 0.5 | 0.01139 |
| GO:0072161 | mesenchymal cell differentiation involved in kidney development | 0.5 | 0.01139 |
| GO:0048205 | COPI coating of Golgi vesicle | 0.5 | 0.01139 |
| GO:2000809 | positive regulation of synaptic vesicle clustering | 0.5 | 0.01139 |
| GO:0006489 | dolichyl diphosphate biosynthetic process | 0.5 | 0.01139 |
| GO:0090069 | regulation of ribosome biogenesis | 0.5 | 0.01139 |
| GO:0003433 | chondrocyte development involved in endochondral bone morphogenesis | 0.5 | 0.01139 |
| GO:0070125 | mitochondrial translational elongation | 0.5 | 0.01139 |
| GO:0000454 | snoRNA guided rRNA pseudouridine synthesis | 0.5 | 0.01139 |
| GO:0002502 | peptide antigen assembly with MHC class I protein complex | 0.5 | 0.01139 |
| GO:0071461 | cellular response to redox state | 0.5 | 0.01139 |
| GO:0019556 | histidine catabolic process to glutamate and formamide | 0.5 | 0.01139 |
| GO:1904683 | regulation of metalloendopeptidase activity | 0.5 | 0.01139 |
| GO:0043606 | formamide metabolic process | 0.5 | 0.01139 |
| GO:0039534 | negative regulation of MDA-5 signaling pathway | 0.5 | 0.01139 |
| GO:0002584 | negative regulation of antigen processing and presentation of peptide antigen | 0.5 | 0.01139 |
| GO:0060018 | astrocyte fate commitment | 0.5 | 0.01139 |
| GO:0048200 | Golgi transport vesicle coating | 0.5 | 0.01139 |
| GO:0000103 | sulfate assimilation | 0.5 | 0.01139 |
| GO:0097116 | gephyrin clustering involved in postsynaptic density assembly | 0.5 | 0.01139 |
| GO:0005610 | laminin-5 complex | 0.5 | 0.01139 |
| GO:0005608 | laminin-3 complex | 0.5 | 0.01139 |
| GO:0097169 | AIM2 inflammasome complex | 0.5 | 0.01139 |
| GO:0005606 | laminin-1 complex | 0.5 | 0.01139 |
| GO:0071614 | linoleic acid epoxygenase activity | 0.5 | 0.01139 |
| GO:0000293 | ferric-chelate reductase activity | 0.5 | 0.01139 |
| GO:0102294 | cholesterol dehydrogenase activity | 0.5 | 0.01139 |
| GO:0046923 | ER retention sequence binding | 0.5 | 0.01139 |
| GO:0008865 | fructokinase activity | 0.5 | 0.01139 |
| GO:0005030 | neurotrophin receptor activity | 0.5 | 0.01139 |
| GO:0004340 | glucokinase activity | 0.5 | 0.01139 |
| GO:0019158 | mannokinase activity | 0.5 | 0.01139 |
| GO:0004366 | glycerol-3-phosphate O-acyltransferase activity | 0.5 | 0.01139 |
| GO:0102420 | sn-1-glycerol-3-phosphate C16:0-DCA-CoA acyl transferase activity | 0.5 | 0.01139 |
| GO:0014889 | muscle atrophy | 0.272727 | 0.01138 |
| GO:0008211 | glucocorticoid metabolic process | 0.272727 | 0.01138 |
| GO:0072376 | protein activation cascade | 0.272727 | 0.01138 |
| GO:0043247 | telomere maintenance in response to DNA damage | 0.272727 | 0.01138 |
| GO:1904152 | regulation of retrograde protein transport, ER to cytosol | 0.272727 | 0.01138 |
| GO:0006030 | chitin metabolic process | 0.272727 | 0.01138 |
| GO:0034145 | positive regulation of toll-like receptor 4 signaling pathway | 0.272727 | 0.01138 |
| GO:0071280 | cellular response to copper ion | 0.272727 | 0.01138 |
| GO:0051775 | response to redox state | 0.272727 | 0.01138 |
| GO:0097205 | renal filtration | 0.272727 | 0.01138 |
| GO:0032612 | interleukin-1 production | 0.272727 | 0.01138 |
| GO:0032611 | interleukin-1 beta production | 0.272727 | 0.01138 |
| GO:0048712 | negative regulation of astrocyte differentiation | 0.272727 | 0.01138 |
| GO:0106083 | nuclear membrane protein complex | 0.272727 | 0.01138 |
| GO:0002199 | zona pellucida receptor complex | 0.272727 | 0.01138 |
| GO:0106094 | nuclear membrane microtubule tethering complex | 0.272727 | 0.01138 |
| GO:0034993 | meiotic nuclear membrane microtubule tethering complex | 0.272727 | 0.01138 |
| GO:0008603 | cAMP-dependent protein kinase regulator activity | 0.272727 | 0.01138 |
| GO:0043121 | neurotrophin binding | 0.272727 | 0.01138 |
| GO:0019966 | interleukin-1 binding | 0.272727 | 0.01138 |
| GO:0015232 | heme transmembrane transporter activity | 0.272727 | 0.01138 |
| GO:0035325 | Toll-like receptor binding | 0.272727 | 0.01138 |
| GO:0071806 | protein transmembrane transport | 0.109756 | 0.01125 |
| GO:0065002 | intracellular protein transmembrane transport | 0.109756 | 0.01125 |
| GO:0001912 | positive regulation of leukocyte mediated cytotoxicity | 0.109756 | 0.01125 |
| GO:0072528 | pyrimidine-containing compound biosynthetic process | 0.127273 | 0.01124 |
| GO:0043531 | ADP binding | 0.127273 | 0.01124 |
| GO:0009914 | hormone transport | 0.104167 | 0.01108 |
| GO:0046931 | pore complex assembly | 0.2 | 0.01104 |
| GO:0070231 | T cell apoptotic process | 0.2 | 0.01104 |
| GO:0045662 | negative regulation of myoblast differentiation | 0.2 | 0.01104 |
| GO:0071870 | cellular response to catecholamine stimulus | 0.2 | 0.01104 |
| GO:0022624 | proteasome accessory complex | 0.2 | 0.01104 |
| GO:0034605 | cellular response to heat | 0.117647 | 0.01104 |
| GO:0032720 | negative regulation of tumor necrosis factor production | 0.142857 | 0.01068 |
| GO:2001056 | positive regulation of cysteine-type endopeptidase activity | 0.090909 | 0.01062 |
| GO:0001667 | ameboidal-type cell migration | 0.090909 | 0.01062 |
| GO:0051153 | regulation of striated muscle cell differentiation | 0.111111 | 0.01042 |
| GO:0032024 | positive regulation of insulin secretion | 0.111111 | 0.01042 |
| GO:1901605 | alpha-amino acid metabolic process | 0.077966 | 0.01034 |
| GO:0005911 | cell-cell junction | 0.068662 | 0.01027 |
| GO:0006040 | amino sugar metabolic process | 0.12963 | 0.01020 |
| GO:0060142 | regulation of syncytium formation by plasma membrane fusion | 0.166667 | 0.01015 |
| GO:0010614 | negative regulation of cardiac muscle hypertrophy | 0.166667 | 0.01015 |
| GO:0001953 | negative regulation of cell-matrix adhesion | 0.166667 | 0.01015 |
| GO:0016878 | acid-thiol ligase activity | 0.166667 | 0.01015 |
| GO:0050830 | defense response to Gram-positive bacterium | 0.091503 | 0.01014 |
| GO:1900542 | regulation of purine nucleotide metabolic process | 0.119403 | 0.01013 |
| GO:0006220 | pyrimidine nucleotide metabolic process | 0.119403 | 0.01013 |
| GO:0071692 | protein localization to extracellular region | 0.100917 | 0.00998 |
| GO:0035592 | establishment of protein localization to extracellular region | 0.100917 | 0.00998 |
| GO:0043178 | alcohol binding | 0.100917 | 0.00998 |
| GO:0098800 | inner mitochondrial membrane protein complex | 0.076687 | 0.00993 |
| GO:0030178 | negative regulation of Wnt signaling pathway | 0.096296 | 0.00992 |
| GO:0051961 | negative regulation of nervous system development | 0.096296 | 0.00992 |
| GO:0010952 | positive regulation of peptidase activity | 0.088889 | 0.00983 |
| GO:0001750 | photoreceptor outer segment | 0.1125 | 0.00964 |
| GO:0044089 | positive regulation of cellular component biogenesis | 0.070815 | 0.00955 |
| GO:1902652 | secondary alcohol metabolic process | 0.097015 | 0.00955 |
| GO:0090207 | regulation of triglyceride metabolic process | 0.146341 | 0.00951 |
| GO:0090311 | regulation of protein deacetylation | 0.146341 | 0.00951 |
| GO:0044275 | cellular carbohydrate catabolic process | 0.146341 | 0.00951 |
| GO:0001664 | G protein-coupled receptor binding | 0.077399 | 0.00949 |
| GO:0009306 | protein secretion | 0.101852 | 0.00934 |
| GO:0051651 | maintenance of location in cell | 0.101852 | 0.00934 |
| GO:0006650 | glycerophospholipid metabolic process | 0.079585 | 0.00928 |
| GO:0007157 | heterophilic cell-cell adhesion via plasma membrane cell adhesion molecules | 0.132075 | 0.00922 |
| GO:0005048 | signal sequence binding | 0.132075 | 0.00922 |
| GO:0015485 | cholesterol binding | 0.132075 | 0.00922 |
| GO:0050768 | negative regulation of neurogenesis | 0.097744 | 0.00920 |
| GO:1900273 | positive regulation of long-term synaptic potentiation | 0.210526 | 0.00915 |
| GO:0071625 | vocalization behavior | 0.210526 | 0.00915 |
| GO:1901863 | positive regulation of muscle tissue development | 0.210526 | 0.00915 |
| GO:0070402 | NADPH binding | 0.210526 | 0.00915 |
| GO:0046474 | glycerophospholipid biosynthetic process | 0.086294 | 0.00908 |
| GO:0009612 | response to mechanical stimulus | 0.086294 | 0.00908 |
| GO:0071840 | cellular component organization or biogenesis | 0.050595 | 0.00897 |
| GO:0006826 | iron ion transport | 0.107527 | 0.00894 |
| GO:0099175 | regulation of postsynapse organization | 0.107527 | 0.00894 |
| GO:0031343 | positive regulation of cell killing | 0.107527 | 0.00894 |
| GO:0008146 | sulfotransferase activity | 0.113924 | 0.00890 |
| GO:0005791 | rough endoplasmic reticulum | 0.098485 | 0.00889 |
| GO:0055076 | transition metal ion homeostasis | 0.093333 | 0.00885 |
| GO:1901888 | regulation of cell junction assembly | 0.090909 | 0.00884 |
| GO:0070328 | triglyceride homeostasis | 0.172414 | 0.00878 |
| GO:0043500 | muscle adaptation | 0.172414 | 0.00878 |
| GO:0055090 | acylglycerol homeostasis | 0.172414 | 0.00878 |
| GO:0014904 | myotube cell development | 0.172414 | 0.00878 |
| GO:0010837 | regulation of keratinocyte proliferation | 0.172414 | 0.00878 |
| GO:0033209 | tumor necrosis factor-mediated signaling pathway | 0.172414 | 0.00878 |
| GO:0070971 | endoplasmic reticulum exit site | 0.172414 | 0.00878 |
| GO:0001941 | postsynaptic membrane organization | 0.3 | 0.00856 |
| GO:0042268 | regulation of cytolysis | 0.3 | 0.00856 |
| GO:0014745 | negative regulation of muscle adaptation | 0.3 | 0.00856 |
| GO:0006703 | estrogen biosynthetic process | 0.3 | 0.00856 |
| GO:0003094 | glomerular filtration | 0.3 | 0.00856 |
| GO:0006032 | chitin catabolic process | 0.3 | 0.00856 |
| GO:0009642 | response to light intensity | 0.3 | 0.00856 |
| GO:0006924 | activation-induced cell death of T cells | 0.3 | 0.00856 |
| GO:0035437 | maintenance of protein localization in endoplasmic reticulum | 0.3 | 0.00856 |
| GO:0071871 | response to epinephrine | 0.3 | 0.00856 |
| GO:0045821 | positive regulation of glycolytic process | 0.3 | 0.00856 |
| GO:0047086 | ketosteroid monooxygenase activity | 0.3 | 0.00856 |
| GO:0008430 | selenium binding | 0.3 | 0.00856 |
| GO:0004499 | N,N-dimethylaniline monooxygenase activity | 0.3 | 0.00856 |
| GO:0050543 | icosatetraenoic acid binding | 0.3 | 0.00856 |
| GO:0004862 | cAMP-dependent protein kinase inhibitor activity | 0.3 | 0.00856 |
| GO:0023023 | MHC protein complex binding | 0.3 | 0.00856 |
| GO:0050433 | regulation of catecholamine secretion | 0.123077 | 0.00847 |
| GO:0002218 | activation of innate immune response | 0.123077 | 0.00847 |
| GO:0034612 | response to tumor necrosis factor | 0.09396 | 0.00846 |
| GO:0098803 | respiratory chain complex | 0.087179 | 0.00844 |
| GO:1903706 | regulation of hemopoiesis | 0.074468 | 0.00844 |
| GO:0010008 | endosome membrane | 0.074468 | 0.00844 |
| GO:1904036 | negative regulation of epithelial cell apoptotic process | 0.15 | 0.00843 |
| GO:0097384 | cellular lipid biosynthetic process | 0.15 | 0.00843 |
| GO:0051412 | response to corticosterone | 0.15 | 0.00843 |
| GO:1990573 | potassium ion import across plasma membrane | 0.15 | 0.00843 |
| GO:0030291 | protein serine/threonine kinase inhibitor activity | 0.15 | 0.00843 |
| GO:0030217 | T cell differentiation | 0.1 | 0.00833 |
| GO:0006221 | pyrimidine nucleotide biosynthetic process | 0.134615 | 0.00832 |
| GO:0032963 | collagen metabolic process | 0.134615 | 0.00832 |
| GO:0051385 | response to mineralocorticoid | 0.134615 | 0.00832 |
| GO:0008028 | monocarboxylic acid transmembrane transporter activity | 0.134615 | 0.00832 |
| GO:0016234 | inclusion body | 0.108696 | 0.00831 |
| GO:0005622 | intracellular anatomical structure | 0.108696 | 0.00831 |
| GO:0060348 | bone development | 0.115385 | 0.00820 |
| GO:1902806 | regulation of cell cycle G1/S phase transition | 0.094595 | 0.00810 |
| GO:0002705 | positive regulation of leukocyte mediated immunity | 0.094595 | 0.00810 |
| GO:0005575 | cellular_component | 0.045856 | 0.00803 |
| GO:0051082 | unfolded protein binding | 0.088083 | 0.00787 |
| GO:0051149 | positive regulation of muscle cell differentiation | 0.125 | 0.00773 |
| GO:0070206 | protein trimerization | 0.125 | 0.00773 |
| GO:0098563 | intrinsic component of synaptic vesicle membrane | 0.125 | 0.00773 |
| GO:0001786 | phosphatidylserine binding | 0.125 | 0.00773 |
| GO:1902882 | regulation of response to oxidative stress | 0.10989 | 0.00771 |
| GO:0090090 | negative regulation of canonical Wnt signaling pathway | 0.104762 | 0.00761 |
| GO:2000377 | regulation of reactive oxygen species metabolic process | 0.088542 | 0.00761 |
| GO:0050807 | regulation of synapse organization | 0.086758 | 0.00758 |
| GO:0071396 | cellular response to lipid | 0.068627 | 0.00757 |
| GO:0043410 | positive regulation of MAPK cascade | 0.074074 | 0.00756 |
| GO:0019915 | lipid storage | 0.178571 | 0.00754 |
| GO:0010866 | regulation of triglyceride biosynthetic process | 0.222222 | 0.00749 |
| GO:0019432 | triglyceride biosynthetic process | 0.222222 | 0.00749 |
| GO:0042772 | DNA damage response, signal transduction resulting in transcription | 0.222222 | 0.00749 |
| GO:0007588 | excretion | 0.222222 | 0.00749 |
| GO:0070202 | regulation of establishment of protein localization to chromosome | 0.222222 | 0.00749 |
| GO:2001026 | regulation of endothelial cell chemotaxis | 0.222222 | 0.00749 |
| GO:0006677 | glycosylceramide metabolic process | 0.222222 | 0.00749 |
| GO:0043252 | sodium-independent organic anion transport | 0.222222 | 0.00749 |
| GO:0043576 | regulation of respiratory gaseous exchange | 0.222222 | 0.00749 |
| GO:0031091 | platelet alpha granule | 0.222222 | 0.00749 |
| GO:0030660 | Golgi-associated vesicle membrane | 0.222222 | 0.00749 |
| GO:0061035 | regulation of cartilage development | 0.137255 | 0.00748 |
| GO:0035418 | protein localization to synapse | 0.137255 | 0.00748 |
| GO:0048678 | response to axon injury | 0.137255 | 0.00748 |
| GO:0033157 | regulation of intracellular protein transport | 0.087156 | 0.00745 |
| GO:0002931 | response to ischemia | 0.153846 | 0.00745 |
| GO:0048500 | signal recognition particle | 0.153846 | 0.00745 |
| GO:0016757 | glycosyltransferase activity | 0.075881 | 0.00742 |
| GO:0030246 | carbohydrate binding | 0.077612 | 0.00738 |
| GO:0097060 | synaptic membrane | 0.079734 | 0.00718 |
| GO:0034061 | DNA polymerase activity | 0.089474 | 0.00714 |
| GO:0044306 | neuron projection terminus | 0.096552 | 0.00712 |
| GO:0031397 | negative regulation of protein ubiquitination | 0.105769 | 0.00710 |
| GO:0032088 | negative regulation of NF-kappaB transcription factor activity | 0.118421 | 0.00694 |
| GO:0044283 | small molecule biosynthetic process | 0.065823 | 0.00692 |
| GO:0009314 | response to radiation | 0.07234 | 0.00680 |
| GO:0030695 | GTPase regulator activity | 0.06914 | 0.00673 |
| GO:0060589 | nucleoside-triphosphatase regulator activity | 0.06914 | 0.00673 |
| GO:0055065 | metal ion homeostasis | 0.075758 | 0.00673 |
| GO:0050878 | regulation of body fluid levels | 0.080808 | 0.00672 |
| GO:0031589 | cell-substrate adhesion | 0.086957 | 0.00670 |
| GO:0060828 | regulation of canonical Wnt signaling pathway | 0.086957 | 0.00670 |
| GO:1901800 | positive regulation of proteasomal protein catabolic process | 0.106796 | 0.00661 |
| GO:0050729 | positive regulation of inflammatory response | 0.106796 | 0.00661 |
| GO:0005774 | vacuolar membrane | 0.082707 | 0.00661 |
| GO:0034308 | primary alcohol metabolic process | 0.11236 | 0.00661 |
| GO:0043066 | negative regulation of apoptotic process | 0.061022 | 0.00660 |
| GO:0045202 | synapse | 0.061022 | 0.00660 |
| GO:1901503 | ether biosynthetic process | 0.157895 | 0.00654 |
| GO:0046504 | glycerol ether biosynthetic process | 0.157895 | 0.00654 |
| GO:2000249 | regulation of actin cytoskeleton reorganization | 0.157895 | 0.00654 |
| GO:0008611 | ether lipid biosynthetic process | 0.157895 | 0.00654 |
| GO:2000351 | regulation of endothelial cell apoptotic process | 0.157895 | 0.00654 |
| GO:0042287 | MHC protein binding | 0.157895 | 0.00654 |
| GO:0050997 | quaternary ammonium group binding | 0.157895 | 0.00654 |
| GO:0060143 | positive regulation of syncytium formation by plasma membrane fusion | 0.185185 | 0.00642 |
| GO:0031063 | regulation of histone deacetylation | 0.185185 | 0.00642 |
| GO:0070325 | lipoprotein particle receptor binding | 0.185185 | 0.00642 |
| GO:0006821 | chloride transport | 0.12 | 0.00636 |
| GO:0050678 | regulation of epithelial cell proliferation | 0.078616 | 0.00630 |
| GO:0006977 | DNA damage response, signal transduction by p53 class mediator resulting in cell cycle arrest | 0.333333 | 0.00620 |
| GO:0090399 | replicative senescence | 0.333333 | 0.00620 |
| GO:0061140 | lung secretory cell differentiation | 0.333333 | 0.00620 |
| GO:0042439 | ethanolamine-containing compound metabolic process | 0.333333 | 0.00620 |
| GO:0010838 | positive regulation of keratinocyte proliferation | 0.333333 | 0.00620 |
| GO:2001046 | positive regulation of integrin-mediated signaling pathway | 0.333333 | 0.00620 |
| GO:0071872 | cellular response to epinephrine stimulus | 0.333333 | 0.00620 |
| GO:0007023 | post-chaperonin tubulin folding pathway | 0.333333 | 0.00620 |
| GO:0060541 | respiratory system development | 0.333333 | 0.00620 |
| GO:0036513 | Derlin-1 retrotranslocation complex | 0.333333 | 0.00620 |
| GO:0050544 | arachidonic acid binding | 0.333333 | 0.00620 |
| GO:0050542 | icosanoid binding | 0.333333 | 0.00620 |
| GO:0015379 | potassium:chloride symporter activity | 0.333333 | 0.00620 |
| GO:0015293 | symporter activity | 0.09434 | 0.00616 |
| GO:0051952 | regulation of amine transport | 0.107843 | 0.00615 |
| GO:1901343 | negative regulation of vasculature development | 0.113636 | 0.00610 |
| GO:0016525 | negative regulation of angiogenesis | 0.113636 | 0.00610 |
| GO:2000181 | negative regulation of blood vessel morphogenesis | 0.113636 | 0.00610 |
| GO:0072527 | pyrimidine-containing compound metabolic process | 0.113636 | 0.00610 |
| GO:1903513 | endoplasmic reticulum to cytosol transport | 0.235294 | 0.00604 |
| GO:0014912 | negative regulation of smooth muscle cell migration | 0.235294 | 0.00604 |
| GO:0006825 | copper ion transport | 0.235294 | 0.00604 |
| GO:0030970 | retrograde protein transport, ER to cytosol | 0.235294 | 0.00604 |
| GO:0019433 | triglyceride catabolic process | 0.235294 | 0.00604 |
| GO:0035457 | cellular response to interferon-alpha | 0.235294 | 0.00604 |
| GO:0031065 | positive regulation of histone deacetylation | 0.235294 | 0.00604 |
| GO:0048643 | positive regulation of skeletal muscle tissue development | 0.235294 | 0.00604 |
| GO:0032026 | response to magnesium ion | 0.235294 | 0.00604 |
| GO:1902547 | regulation of cellular response to vascular endothelial growth factor stimulus | 0.235294 | 0.00604 |
| GO:0031527 | filopodium membrane | 0.235294 | 0.00604 |
| GO:0071256 | translocon complex | 0.235294 | 0.00604 |
| GO:0015347 | sodium-independent organic anion transmembrane transporter activity | 0.235294 | 0.00604 |
| GO:0035150 | regulation of tube size | 0.103448 | 0.00603 |
| GO:0042383 | sarcolemma | 0.103448 | 0.00603 |
| GO:0030332 | cyclin binding | 0.142857 | 0.00600 |
| GO:0006259 | DNA metabolic process | 0.062673 | 0.00593 |
| GO:0043161 | proteasome-mediated ubiquitin-dependent protein catabolic process | 0.077586 | 0.00591 |
| GO:0001969 | regulation of activation of membrane attack complex | 0.666667 | 0.00587 |
| GO:2000660 | negative regulation of interleukin-1-mediated signaling pathway | 0.666667 | 0.00587 |
| GO:0009757 | hexose mediated signaling | 0.666667 | 0.00587 |
| GO:0009182 | purine deoxyribonucleoside diphosphate metabolic process | 0.666667 | 0.00587 |
| GO:0071865 | regulation of apoptotic process in bone marrow cell | 0.666667 | 0.00587 |
| GO:0071866 | negative regulation of apoptotic process in bone marrow cell | 0.666667 | 0.00587 |
| GO:0019240 | citrulline biosynthetic process | 0.666667 | 0.00587 |
| GO:0061623 | glycolytic process from galactose | 0.666667 | 0.00587 |
| GO:0090290 | positive regulation of osteoclast proliferation | 0.666667 | 0.00587 |
| GO:0046066 | dGDP metabolic process | 0.666667 | 0.00587 |
| GO:1904684 | negative regulation of metalloendopeptidase activity | 0.666667 | 0.00587 |
| GO:1903895 | negative regulation of IRE1-mediated unfolded protein response | 0.666667 | 0.00587 |
| GO:0009756 | carbohydrate mediated signaling | 0.666667 | 0.00587 |
| GO:0043323 | positive regulation of natural killer cell degranulation | 0.666667 | 0.00587 |
| GO:0043321 | regulation of natural killer cell degranulation | 0.666667 | 0.00587 |
| GO:0010273 | detoxification of copper ion | 0.666667 | 0.00587 |
| GO:0010182 | sugar mediated signaling pathway | 0.666667 | 0.00587 |
| GO:2000233 | negative regulation of rRNA processing | 0.666667 | 0.00587 |
| GO:0002232 | leukocyte chemotaxis involved in inflammatory response | 0.666667 | 0.00587 |
| GO:0044752 | response to human chorionic gonadotropin | 0.666667 | 0.00587 |
| GO:0150099 | neuron-glial cell signaling | 0.666667 | 0.00587 |
| GO:0010255 | glucose mediated signaling pathway | 0.666667 | 0.00587 |
| GO:0140284 | endoplasmic reticulum-endosome membrane contact site | 0.666667 | 0.00587 |
| GO:0036502 | Derlin-1-VIMP complex | 0.666667 | 0.00587 |
| GO:0004937 | alpha1-adrenergic receptor activity | 0.666667 | 0.00587 |
| GO:0019862 | IgA binding | 0.666667 | 0.00587 |
| GO:0106137 | IkappaB kinase complex binding | 0.666667 | 0.00587 |
| GO:0043120 | tumor necrosis factor binding | 0.666667 | 0.00587 |
| GO:0070891 | lipoteichoic acid binding | 0.666667 | 0.00587 |
| GO:0031726 | CCR1 chemokine receptor binding | 0.666667 | 0.00587 |
| GO:0003960 | NADPH:quinone reductase activity | 0.666667 | 0.00587 |
| GO:0005046 | KDEL sequence binding | 0.666667 | 0.00587 |
| GO:0008593 | regulation of Notch signaling pathway | 0.121622 | 0.00583 |
| GO:0006140 | regulation of nucleotide metabolic process | 0.121622 | 0.00583 |
| GO:0045111 | intermediate filament cytoskeleton | 0.131148 | 0.00578 |
| GO:0006536 | glutamate metabolic process | 0.162162 | 0.00572 |
| GO:0015980 | energy derivation by oxidation of organic compounds | 0.08156 | 0.00567 |
| GO:0032436 | positive regulation of proteasomal ubiquitin-dependent protein catabolic process | 0.114943 | 0.00563 |
| GO:0001816 | cytokine production | 0.104348 | 0.00563 |
| GO:0045785 | positive regulation of cell adhesion | 0.07672 | 0.00556 |
| GO:0005126 | cytokine receptor binding | 0.07672 | 0.00556 |
| GO:2001234 | negative regulation of apoptotic signaling pathway | 0.084 | 0.00548 |
| GO:0048701 | embryonic cranial skeleton morphogenesis | 0.192308 | 0.00543 |
| GO:0006515 | protein quality control for misfolded or incompletely synthesized proteins | 0.192308 | 0.00543 |
| GO:0009226 | nucleotide-sugar biosynthetic process | 0.192308 | 0.00543 |
| GO:0032527 | protein exit from endoplasmic reticulum | 0.192308 | 0.00543 |
| GO:0048741 | skeletal muscle fiber development | 0.192308 | 0.00543 |
| GO:0006457 | protein folding | 0.078717 | 0.00539 |
| GO:0006520 | cellular amino acid metabolic process | 0.07561 | 0.00537 |
| GO:0002253 | activation of immune response | 0.07561 | 0.00537 |
| GO:0042181 | ketone biosynthetic process | 0.145833 | 0.00535 |
| GO:0043536 | positive regulation of blood vessel endothelial cell migration | 0.145833 | 0.00535 |
| GO:1903201 | regulation of oxidative stress-induced cell death | 0.123288 | 0.00533 |
| GO:0043535 | regulation of blood vessel endothelial cell migration | 0.123288 | 0.00533 |
| GO:0016597 | amino acid binding | 0.123288 | 0.00533 |
| GO:1903321 | negative regulation of protein modification by small protein conjugation or removal | 0.105263 | 0.00525 |
| GO:0010565 | regulation of cellular ketone metabolic process | 0.105263 | 0.00525 |
| GO:0061387 | regulation of extent of cell growth | 0.105263 | 0.00525 |
| GO:0097110 | scaffold protein binding | 0.133333 | 0.00523 |
| GO:0038024 | cargo receptor activity | 0.116279 | 0.00519 |
| GO:0097435 | supramolecular fiber organization | 0.071845 | 0.00515 |
| GO:0043413 | macromolecule glycosylation | 0.084677 | 0.00514 |
| GO:0006486 | protein glycosylation | 0.084677 | 0.00514 |
| GO:2001242 | regulation of intrinsic apoptotic signaling pathway | 0.090452 | 0.00503 |
| GO:0048536 | spleen development | 0.166667 | 0.00498 |
| GO:0045923 | positive regulation of fatty acid metabolic process | 0.166667 | 0.00498 |
| GO:0014014 | negative regulation of gliogenesis | 0.166667 | 0.00498 |
| GO:1900015 | regulation of cytokine production involved in inflammatory response | 0.166667 | 0.00498 |
| GO:0021955 | central nervous system neuron axonogenesis | 0.166667 | 0.00498 |
| GO:0005640 | nuclear outer membrane | 0.166667 | 0.00498 |
| GO:0008021 | synaptic vesicle | 0.097403 | 0.00493 |
| GO:0051350 | negative regulation of lyase activity | 0.25 | 0.00478 |
| GO:0034111 | negative regulation of homotypic cell-cell adhesion | 0.25 | 0.00478 |
| GO:1900746 | regulation of vascular endothelial growth factor signaling pathway | 0.25 | 0.00478 |
| GO:0099172 | presynapse organization | 0.25 | 0.00478 |
| GO:0031204 | post-translational protein targeting to membrane, translocation | 0.25 | 0.00478 |
| GO:2001044 | regulation of integrin-mediated signaling pathway | 0.25 | 0.00478 |
| GO:0008312 | 7S RNA binding | 0.25 | 0.00478 |
| GO:0015914 | phospholipid transport | 0.117647 | 0.00477 |
| GO:0048598 | embryonic morphogenesis | 0.076142 | 0.00474 |
| GO:0009063 | cellular amino acid catabolic process | 0.098039 | 0.00472 |
| GO:0050714 | positive regulation of protein secretion | 0.098039 | 0.00472 |
| GO:0050766 | positive regulation of phagocytosis | 0.135593 | 0.00471 |
| GO:0032934 | sterol binding | 0.135593 | 0.00471 |
| GO:0016780 | phosphotransferase activity, for other substituted phosphate groups | 0.135593 | 0.00471 |
| GO:0010605 | negative regulation of macromolecule metabolic process | 0.054617 | 0.00459 |
| GO:1903318 | negative regulation of protein maturation | 0.2 | 0.00455 |
| GO:0010955 | negative regulation of protein processing | 0.2 | 0.00455 |
| GO:0031341 | regulation of cell killing | 0.103175 | 0.00446 |
| GO:0090150 | establishment of protein localization to membrane | 0.092308 | 0.00446 |
| GO:0000902 | cell morphogenesis | 0.08642 | 0.00443 |
| GO:0050709 | negative regulation of protein secretion | 0.119048 | 0.00438 |
| GO:0051604 | protein maturation | 0.081911 | 0.00436 |
| GO:0045931 | positive regulation of mitotic cell cycle | 0.099338 | 0.00436 |
| GO:1903169 | regulation of calcium ion transmembrane transport | 0.099338 | 0.00436 |
| GO:0019903 | protein phosphatase binding | 0.099338 | 0.00436 |
| GO:0006643 | membrane lipid metabolic process | 0.092784 | 0.00435 |
| GO:0045919 | positive regulation of cytolysis | 0.375 | 0.00427 |
| GO:0051917 | regulation of fibrinolysis | 0.375 | 0.00427 |
| GO:0032790 | ribosome disassembly | 0.375 | 0.00427 |
| GO:0071286 | cellular response to magnesium ion | 0.375 | 0.00427 |
| GO:0006547 | histidine metabolic process | 0.375 | 0.00427 |
| GO:0009635 | response to herbicide | 0.375 | 0.00427 |
| GO:0021957 | corticospinal tract morphogenesis | 0.375 | 0.00427 |
| GO:0140268 | endoplasmic reticulum-plasma membrane contact site | 0.375 | 0.00427 |
| GO:0033018 | sarcoplasmic reticulum lumen | 0.375 | 0.00427 |
| GO:0004300 | enoyl-CoA hydratase activity | 0.375 | 0.00427 |
| GO:0004568 | chitinase activity | 0.375 | 0.00427 |
| GO:0042609 | CD4 receptor binding | 0.375 | 0.00427 |
| GO:1900180 | regulation of protein localization to nucleus | 0.108108 | 0.00424 |
| GO:0051289 | protein homotetramerization | 0.108108 | 0.00424 |
| GO:0010507 | negative regulation of autophagy | 0.137931 | 0.00424 |
| GO:0045277 | respiratory chain complex IV | 0.137931 | 0.00424 |
| GO:0044087 | regulation of cellular component biogenesis | 0.065195 | 0.00420 |
| GO:1901998 | toxin transport | 0.152174 | 0.00420 |
| GO:0045428 | regulation of nitric oxide biosynthetic process | 0.152174 | 0.00420 |
| GO:0016235 | aggresome | 0.152174 | 0.00420 |
| GO:0008106 | alcohol dehydrogenase (NADP+) activity | 0.152174 | 0.00420 |
| GO:0019955 | cytokine binding | 0.1 | 0.00419 |
| GO:0010498 | proteasomal protein catabolic process | 0.077519 | 0.00415 |
| GO:0097305 | response to alcohol | 0.079096 | 0.00413 |
| GO:0002699 | positive regulation of immune effector process | 0.0875 | 0.00411 |
| GO:0051348 | negative regulation of transferase activity | 0.084615 | 0.00408 |
| GO:0070085 | glycosylation | 0.084615 | 0.00408 |
| GO:0044262 | cellular carbohydrate metabolic process | 0.094241 | 0.00406 |
| GO:0019902 | phosphatase binding | 0.094241 | 0.00406 |
| GO:0032507 | maintenance of protein location in cell | 0.128571 | 0.00402 |
| GO:1901224 | positive regulation of NIK/NF-kappaB signaling | 0.128571 | 0.00402 |
| GO:0010631 | epithelial cell migration | 0.128571 | 0.00402 |
| GO:0051924 | regulation of calcium ion transport | 0.087866 | 0.00402 |
| GO:0031960 | response to corticosteroid | 0.094737 | 0.00398 |
| GO:0005524 | ATP binding | 0.057437 | 0.00395 |
| GO:0030546 | signaling receptor activator activity | 0.069552 | 0.00395 |
| GO:0010564 | regulation of cell cycle process | 0.067935 | 0.00393 |
| GO:0043204 | perikaryon | 0.096386 | 0.00391 |
| GO:0098685 | Schaffer collateral - CA1 synapse | 0.114583 | 0.00388 |
| GO:0010959 | regulation of metal ion transport | 0.078534 | 0.00387 |
| GO:0050890 | cognition | 0.081505 | 0.00386 |
| GO:0016874 | ligase activity | 0.083333 | 0.00380 |
| GO:0008009 | chemokine activity | 0.140351 | 0.00380 |
| GO:0034110 | regulation of homotypic cell-cell adhesion | 0.208333 | 0.00378 |
| GO:0004745 | NAD-retinol dehydrogenase activity | 0.208333 | 0.00378 |
| GO:0005158 | insulin receptor binding | 0.208333 | 0.00378 |
| GO:0043433 | negative regulation of DNA-binding transcription factor activity | 0.102041 | 0.00378 |
| GO:0048018 | receptor ligand activity | 0.070093 | 0.00374 |
| GO:0099503 | secretory vesicle | 0.073077 | 0.00374 |
| GO:0043069 | negative regulation of programmed cell death | 0.061794 | 0.00373 |
| GO:0002921 | negative regulation of humoral immune response | 0.266667 | 0.00372 |
| GO:0071285 | cellular response to lithium ion | 0.266667 | 0.00372 |
| GO:0030497 | fatty acid elongation | 0.266667 | 0.00372 |
| GO:0098691 | dopaminergic synapse | 0.266667 | 0.00372 |
| GO:0072562 | blood microparticle | 0.266667 | 0.00372 |
| GO:0035455 | response to interferon-alpha | 0.176471 | 0.00371 |
| GO:0044232 | organelle membrane contact site | 0.176471 | 0.00371 |
| GO:0004032 | alditol:NADP+ 1-oxidoreductase activity | 0.176471 | 0.00371 |
| GO:0050727 | regulation of inflammatory response | 0.083624 | 0.00371 |
| GO:0002920 | regulation of humoral immune response | 0.155556 | 0.00370 |
| GO:1903556 | negative regulation of tumor necrosis factor superfamily cytokine production | 0.155556 | 0.00370 |
| GO:0030120 | vesicle coat | 0.155556 | 0.00370 |
| GO:0008654 | phospholipid biosynthetic process | 0.085603 | 0.00369 |
| GO:1900407 | regulation of cellular response to oxidative stress | 0.121951 | 0.00368 |
| GO:0016627 | oxidoreductase activity, acting on the CH-CH group of donors | 0.121951 | 0.00368 |
| GO:0042379 | chemokine receptor binding | 0.121951 | 0.00368 |
| GO:0008284 | positive regulation of cell population proliferation | 0.063771 | 0.00367 |
| GO:0006109 | regulation of carbohydrate metabolic process | 0.090909 | 0.00366 |
| GO:0030324 | lung development | 0.110092 | 0.00365 |
| GO:0043065 | positive regulation of apoptotic process | 0.072072 | 0.00364 |
| GO:0009896 | positive regulation of catabolic process | 0.07431 | 0.00352 |
| GO:0031348 | negative regulation of defense response | 0.091787 | 0.00343 |
| GO:0002020 | protease binding | 0.09816 | 0.00341 |
| GO:1903578 | regulation of ATP metabolic process | 0.142857 | 0.00340 |
| GO:0008203 | cholesterol metabolic process | 0.106557 | 0.00338 |
| GO:0033273 | response to vitamin | 0.123457 | 0.00336 |
| GO:0019395 | fatty acid oxidation | 0.123457 | 0.00336 |
| GO:1901652 | response to peptide | 0.076037 | 0.00335 |
| GO:0016485 | protein processing | 0.092233 | 0.00332 |
| GO:0009611 | response to wounding | 0.092233 | 0.00332 |
| GO:0007268 | chemical synaptic transmission | 0.085714 | 0.00321 |
| GO:0098916 | anterograde trans-synaptic signaling | 0.085714 | 0.00321 |
| GO:0032990 | cell part morphogenesis | 0.081081 | 0.00315 |
| GO:0032989 | cellular component morphogenesis | 0.081081 | 0.00315 |
| GO:0015748 | organophosphate ester transport | 0.107438 | 0.00315 |
| GO:0006465 | signal peptide processing | 0.217391 | 0.00311 |
| GO:0015721 | bile acid and bile salt transport | 0.217391 | 0.00311 |
| GO:2000352 | negative regulation of endothelial cell apoptotic process | 0.217391 | 0.00311 |
| GO:0071813 | lipoprotein particle binding | 0.217391 | 0.00311 |
| GO:0071814 | protein-lipid complex binding | 0.217391 | 0.00311 |
| GO:0000502 | proteasome complex | 0.125 | 0.00307 |
| GO:0010557 | positive regulation of macromolecule biosynthetic process | 0.060081 | 0.00300 |
| GO:0051048 | negative regulation of secretion | 0.094059 | 0.00297 |
| GO:0046903 | secretion | 0.074747 | 0.00293 |
| GO:0098793 | presynapse | 0.083612 | 0.00290 |
| GO:0016209 | antioxidant activity | 0.113208 | 0.00290 |
| GO:2001235 | positive regulation of apoptotic signaling pathway | 0.104478 | 0.00289 |
| GO:0043021 | ribonucleoprotein complex binding | 0.090498 | 0.00288 |
| GO:0030336 | negative regulation of cell migration | 0.08871 | 0.00286 |
| GO:0070382 | exocytic vesicle | 0.096591 | 0.00285 |
| GO:0009165 | nucleotide biosynthetic process | 0.08078 | 0.00285 |
| GO:0030285 | integral component of synaptic vesicle membrane | 0.162791 | 0.00284 |
| GO:0006620 | post-translational protein targeting to endoplasmic reticulum membrane | 0.285714 | 0.00283 |
| GO:0090129 | positive regulation of synapse maturation | 0.285714 | 0.00283 |
| GO:0009134 | nucleoside diphosphate catabolic process | 0.285714 | 0.00283 |
| GO:2001028 | positive regulation of endothelial cell chemotaxis | 0.285714 | 0.00283 |
| GO:0060192 | negative regulation of lipase activity | 0.285714 | 0.00283 |
| GO:0000002 | mitochondrial genome maintenance | 0.285714 | 0.00283 |
| GO:0042761 | very long-chain fatty acid biosynthetic process | 0.285714 | 0.00283 |
| GO:0030169 | low-density lipoprotein particle binding | 0.285714 | 0.00283 |
| GO:0004771 | sterol esterase activity | 0.285714 | 0.00283 |
| GO:0009064 | glutamine family amino acid metabolic process | 0.119565 | 0.00278 |
| GO:0099699 | integral component of synaptic membrane | 0.095477 | 0.00278 |
| GO:0016323 | basolateral plasma membrane | 0.090909 | 0.00278 |
| GO:1903044 | protein localization to membrane raft | 0.428571 | 0.00276 |
| GO:0000052 | citrulline metabolic process | 0.428571 | 0.00276 |
| GO:0044320 | cellular response to leptin stimulus | 0.428571 | 0.00276 |
| GO:1903894 | regulation of IRE1-mediated unfolded protein response | 0.428571 | 0.00276 |
| GO:0005785 | signal recognition particle receptor complex | 0.428571 | 0.00276 |
| GO:0044316 | cone cell pedicle | 0.428571 | 0.00276 |
| GO:0004703 | G protein-coupled receptor kinase activity | 0.428571 | 0.00276 |
| GO:0035662 | Toll-like receptor 4 binding | 0.428571 | 0.00276 |
| GO:0051384 | response to glucocorticoid | 0.097143 | 0.00272 |
| GO:0019216 | regulation of lipid metabolic process | 0.082822 | 0.00271 |
| GO:0014741 | negative regulation of muscle hypertrophy | 0.1875 | 0.00270 |
| GO:0017169 | CDP-alcohol phosphatidyltransferase activity | 0.1875 | 0.00270 |
| GO:0046467 | membrane lipid biosynthetic process | 0.105263 | 0.00270 |
| GO:0097529 | myeloid leukocyte migration | 0.105263 | 0.00270 |
| GO:0099536 | synaptic signaling | 0.084459 | 0.00268 |
| GO:0099537 | trans-synaptic signaling | 0.084459 | 0.00268 |
| GO:0030111 | regulation of Wnt signaling pathway | 0.086466 | 0.00264 |
| GO:0022904 | respiratory electron transport chain | 0.097701 | 0.00259 |
| GO:0019209 | kinase activator activity | 0.102564 | 0.00259 |
| GO:0005085 | guanyl-nucleotide exchange factor activity | 0.083591 | 0.00259 |
| GO:0034764 | positive regulation of transmembrane transport | 0.091743 | 0.00258 |
| GO:0002449 | lymphocyte mediated immunity | 0.128205 | 0.00254 |
| GO:0043269 | regulation of ion transport | 0.070254 | 0.00253 |
| GO:0010634 | positive regulation of epithelial cell migration | 0.110169 | 0.00253 |
| GO:0043086 | negative regulation of catalytic activity | 0.067217 | 0.00252 |
| GO:0046463 | acylglycerol biosynthetic process | 0.227273 | 0.00252 |
| GO:0046460 | neutral lipid biosynthetic process | 0.227273 | 0.00252 |
| GO:0044819 | mitotic G1/S transition checkpoint signaling | 0.227273 | 0.00252 |
| GO:0055093 | response to hyperoxia | 0.227273 | 0.00252 |
| GO:0085029 | extracellular matrix assembly | 0.227273 | 0.00252 |
| GO:0031571 | mitotic G1 DNA damage checkpoint signaling | 0.227273 | 0.00252 |
| GO:0098798 | mitochondrial protein-containing complex | 0.073126 | 0.00250 |
| GO:0050777 | negative regulation of immune response | 0.103896 | 0.00243 |
| GO:0140352 | export from cell | 0.078431 | 0.00242 |
| GO:0045936 | negative regulation of phosphate metabolic process | 0.075789 | 0.00242 |
| GO:0010563 | negative regulation of phosphorus metabolic process | 0.075789 | 0.00242 |
| GO:0045995 | regulation of embryonic development | 0.138462 | 0.00241 |
| GO:0061512 | protein localization to cilium | 0.150943 | 0.00239 |
| GO:1903531 | negative regulation of secretion by cell | 0.098837 | 0.00236 |
| GO:0040013 | negative regulation of locomotion | 0.086207 | 0.00236 |
| GO:0015718 | monocarboxylic acid transport | 0.122222 | 0.00233 |
| GO:0005581 | collagen trimer | 0.122222 | 0.00233 |
| GO:1905369 | endopeptidase complex | 0.122222 | 0.00233 |
| GO:0030162 | regulation of proteolysis | 0.066381 | 0.00233 |
| GO:0048858 | cell projection morphogenesis | 0.086505 | 0.00232 |
| GO:0050778 | positive regulation of immune response | 0.070997 | 0.00231 |
| GO:0046873 | metal ion transmembrane transporter activity | 0.076271 | 0.00231 |
| GO:0016853 | isomerase activity | 0.080429 | 0.00229 |
| GO:0045017 | glycerolipid biosynthetic process | 0.093458 | 0.00226 |
| GO:0008201 | heparin binding | 0.093458 | 0.00226 |
| GO:0022604 | regulation of cell morphogenesis | 0.083871 | 0.00223 |
| GO:2000146 | negative regulation of cell motility | 0.088462 | 0.00220 |
| GO:0006996 | organelle organization | 0.056919 | 0.00218 |
| GO:0042304 | regulation of fatty acid biosynthetic process | 0.170732 | 0.00214 |
| GO:0014059 | regulation of dopamine secretion | 0.170732 | 0.00214 |
| GO:0004857 | enzyme inhibitor activity | 0.069577 | 0.00213 |
| GO:0005815 | microtubule organizing center | 0.066813 | 0.00210 |
| GO:1990266 | neutrophil migration | 0.117647 | 0.00210 |
| GO:0000188 | obsolete inactivation of MAPK activity | 0.307692 | 0.00209 |
| GO:0071712 | ER-associated misfolded protein catabolic process | 0.307692 | 0.00209 |
| GO:0008191 | metalloendopeptidase inhibitor activity | 0.307692 | 0.00209 |
| GO:0042834 | peptidoglycan binding | 0.307692 | 0.00209 |
| GO:0004115 | 3',5'-cyclic-AMP phosphodiesterase activity | 0.307692 | 0.00209 |
| GO:0022900 | electron transport chain | 0.096774 | 0.00209 |
| GO:0016758 | hexosyltransferase activity | 0.089147 | 0.00209 |
| GO:0016747 | acyltransferase activity, transferring groups other than amino-acyl groups | 0.086643 | 0.00203 |
| GO:0006665 | sphingolipid metabolic process | 0.108527 | 0.00203 |
| GO:0045655 | regulation of monocyte differentiation | 0.238095 | 0.00202 |
| GO:1903523 | negative regulation of blood circulation | 0.238095 | 0.00202 |
| GO:0034620 | cellular response to unfolded protein | 0.238095 | 0.00202 |
| GO:0045822 | negative regulation of heart contraction | 0.238095 | 0.00202 |
| GO:0071378 | cellular response to growth hormone stimulus | 1 | 0.00202 |
| GO:0001869 | negative regulation of complement activation, lectin pathway | 1 | 0.00202 |
| GO:0001868 | regulation of complement activation, lectin pathway | 1 | 0.00202 |
| GO:0010615 | positive regulation of cardiac muscle adaptation | 1 | 0.00202 |
| GO:0006864 | pyrimidine nucleotide transport | 1 | 0.00202 |
| GO:0070488 | neutrophil aggregation | 1 | 0.00202 |
| GO:2000790 | regulation of mesenchymal cell proliferation involved in lung development | 1 | 0.00202 |
| GO:2000791 | negative regulation of mesenchymal cell proliferation involved in lung development | 1 | 0.00202 |
| GO:0048792 | spontaneous exocytosis of neurotransmitter | 1 | 0.00202 |
| GO:0017143 | insecticide metabolic process | 1 | 0.00202 |
| GO:1990519 | pyrimidine nucleotide import into mitochondrion | 1 | 0.00202 |
| GO:0051599 | response to hydrostatic pressure | 1 | 0.00202 |
| GO:1903244 | positive regulation of cardiac muscle hypertrophy in response to stress | 1 | 0.00202 |
| GO:0071753 | IgM immunoglobulin complex | 1 | 0.00202 |
| GO:0071756 | pentameric IgM immunoglobulin complex | 1 | 0.00202 |
| GO:0071754 | IgM immunoglobulin complex, circulating | 1 | 0.00202 |
| GO:0050254 | rhodopsin kinase activity | 1 | 0.00202 |
| GO:0008503 | benzodiazepine receptor activity | 1 | 0.00202 |
| GO:0005497 | androgen binding | 1 | 0.00202 |
| GO:0015218 | pyrimidine nucleotide transmembrane transporter activity | 1 | 0.00202 |
| GO:0016743 | carboxyl- or carbamoyltransferase activity | 1 | 0.00202 |
| GO:0016401 | palmitoyl-CoA oxidase activity | 1 | 0.00202 |
| GO:0072341 | modified amino acid binding | 0.113043 | 0.00201 |
| GO:0023052 | signaling | 0.076923 | 0.00199 |
| GO:1901222 | regulation of NIK/NF-kappaB signaling | 0.118812 | 0.00193 |
| GO:0051117 | ATPase binding | 0.118812 | 0.00193 |
| GO:0034767 | positive regulation of ion transmembrane transport | 0.101796 | 0.00191 |
| GO:0006663 | platelet activating factor biosynthetic process | 0.2 | 0.00191 |
| GO:0001666 | response to hypoxia | 0.087273 | 0.00189 |
| GO:0042542 | response to hydrogen peroxide | 0.109375 | 0.00188 |
| GO:0045471 | response to ethanol | 0.10241 | 0.00184 |
| GO:0008219 | cell death | 0.065078 | 0.00184 |
| GO:0016043 | cellular component organization | 0.051914 | 0.00180 |
| GO:0006796 | phosphate-containing compound metabolic process | 0.058101 | 0.00180 |
| GO:0007584 | response to nutrient | 0.10303 | 0.00178 |
| GO:0043177 | organic acid binding | 0.10303 | 0.00178 |
| GO:0005507 | copper ion binding | 0.126437 | 0.00177 |
| GO:0030516 | regulation of axon extension | 0.12 | 0.00177 |
| GO:0055086 | nucleobase-containing small molecule metabolic process | 0.065378 | 0.00176 |
| GO:0030117 | membrane coat | 0.145161 | 0.00173 |
| GO:0030148 | sphingolipid biosynthetic process | 0.135135 | 0.00171 |
| GO:1901362 | organic cyclic compound biosynthetic process | 0.064407 | 0.00169 |
| GO:0051222 | positive regulation of protein transport | 0.087542 | 0.00164 |
| GO:1901681 | sulfur compound binding | 0.082857 | 0.00164 |
| GO:0009186 | deoxyribonucleoside diphosphate metabolic process | 0.5 | 0.00163 |
| GO:0048280 | vesicle fusion with Golgi apparatus | 0.5 | 0.00163 |
| GO:0034139 | regulation of toll-like receptor 3 signaling pathway | 0.5 | 0.00163 |
| GO:0021960 | anterior commissure morphogenesis | 0.5 | 0.00163 |
| GO:0097119 | postsynaptic density protein 95 clustering | 0.5 | 0.00163 |
| GO:0035723 | interleukin-15-mediated signaling pathway | 0.5 | 0.00163 |
| GO:0021540 | corpus callosum morphogenesis | 0.5 | 0.00163 |
| GO:0030663 | COPI-coated vesicle membrane | 0.5 | 0.00163 |
| GO:0030292 | protein tyrosine kinase inhibitor activity | 0.5 | 0.00163 |
| GO:1902533 | positive regulation of intracellular signal transduction | 0.06512 | 0.00163 |
| GO:0000904 | cell morphogenesis involved in differentiation | 0.107143 | 0.00163 |
| GO:0007611 | learning or memory | 0.088889 | 0.00161 |
| GO:0090128 | regulation of synapse maturation | 0.25 | 0.00160 |
| GO:0097242 | amyloid-beta clearance | 0.25 | 0.00160 |
| GO:0012507 | ER to Golgi transport vesicle membrane | 0.25 | 0.00160 |
| GO:0048037 | obsolete cofactor binding | 0.25 | 0.00160 |
| GO:0015125 | bile acid transmembrane transporter activity | 0.25 | 0.00160 |
| GO:0062012 | regulation of small molecule metabolic process | 0.083095 | 0.00160 |
| GO:1902042 | negative regulation of extrinsic apoptotic signaling pathway via death domain receptors | 0.206897 | 0.00159 |
| GO:0035967 | cellular response to topologically incorrect protein | 0.206897 | 0.00159 |
| GO:0004806 | triglyceride lipase activity | 0.206897 | 0.00159 |
| GO:0010518 | positive regulation of phospholipase activity | 0.179487 | 0.00159 |
| GO:0032196 | transposition | 0.179487 | 0.00159 |
| GO:0097530 | granulocyte migration | 0.116071 | 0.00158 |
| GO:1903532 | positive regulation of secretion by cell | 0.084906 | 0.00156 |
| GO:0051051 | negative regulation of transport | 0.077253 | 0.00156 |
| GO:0010810 | regulation of cell-substrate adhesion | 0.096939 | 0.00154 |
| GO:0099587 | inorganic ion import across plasma membrane | 0.136986 | 0.00154 |
| GO:0098659 | inorganic cation import across plasma membrane | 0.136986 | 0.00154 |
| GO:0005201 | extracellular matrix structural constituent | 0.136986 | 0.00154 |
| GO:0048762 | mesenchymal cell differentiation | 0.147541 | 0.00154 |
| GO:0050839 | cell adhesion molecule binding | 0.083573 | 0.00153 |
| GO:0035162 | embryonic hemopoiesis | 0.333333 | 0.00150 |
| GO:0019673 | GDP-mannose metabolic process | 0.333333 | 0.00150 |
| GO:0045916 | negative regulation of complement activation | 0.333333 | 0.00150 |
| GO:0050801 | ion homeostasis | 0.076459 | 0.00147 |
| GO:0015850 | organic hydroxy compound transport | 0.117117 | 0.00145 |
| GO:0048638 | regulation of developmental growth | 0.084302 | 0.00144 |
| GO:0043068 | positive regulation of programmed cell death | 0.074468 | 0.00142 |
| GO:0004033 | aldo-keto reductase (NADP) activity | 0.163265 | 0.00142 |
| GO:0120039 | plasma membrane bounded cell projection morphogenesis | 0.087719 | 0.00140 |
| GO:0044092 | negative regulation of molecular function | 0.06476 | 0.00137 |
| GO:1901293 | nucleoside phosphate biosynthetic process | 0.082418 | 0.00137 |
| GO:0030098 | lymphocyte differentiation | 0.091255 | 0.00135 |
| GO:0007626 | locomotory behavior | 0.088028 | 0.00135 |
| GO:0043547 | positive regulation of GTPase activity | 0.088028 | 0.00135 |
| GO:1901798 | positive regulation of signal transduction by p53 class mediator | 0.184211 | 0.00135 |
| GO:0032197 | transposition, RNA-mediated | 0.184211 | 0.00135 |
| GO:0099240 | intrinsic component of synaptic membrane | 0.096774 | 0.00135 |
| GO:0043022 | ribosome binding | 0.130952 | 0.00133 |
| GO:0010954 | positive regulation of protein processing | 0.214286 | 0.00131 |
| GO:0035987 | endodermal cell differentiation | 0.214286 | 0.00131 |
| GO:0033198 | response to ATP | 0.214286 | 0.00131 |
| GO:0021952 | central nervous system projection neuron axonogenesis | 0.214286 | 0.00131 |
| GO:0018995 | host cellular component | 0.214286 | 0.00131 |
| GO:0004142 | diacylglycerol cholinephosphotransferase activity | 0.214286 | 0.00131 |
| GO:1902532 | negative regulation of intracellular signal transduction | 0.076336 | 0.00131 |
| GO:0007041 | lysosomal transport | 0.10596 | 0.00130 |
| GO:0005525 | GTP binding | 0.073248 | 0.00127 |
| GO:0043270 | positive regulation of ion transport | 0.088652 | 0.00126 |
| GO:0001525 | angiogenesis | 0.088652 | 0.00126 |
| GO:0033762 | response to glucagon | 0.263158 | 0.00125 |
| GO:0045185 | maintenance of protein location | 0.125 | 0.00124 |
| GO:0080164 | regulation of nitric oxide metabolic process | 0.166667 | 0.00124 |
| GO:0018904 | ether metabolic process | 0.166667 | 0.00124 |
| GO:0031985 | Golgi cisterna | 0.166667 | 0.00124 |
| GO:0071326 | cellular response to monosaccharide stimulus | 0.140845 | 0.00124 |
| GO:0016667 | oxidoreductase activity, acting on a sulfur group of donors | 0.140845 | 0.00124 |
| GO:0090277 | positive regulation of peptide hormone secretion | 0.119266 | 0.00123 |
| GO:0006814 | sodium ion transport | 0.119266 | 0.00123 |
| GO:0033674 | positive regulation of kinase activity | 0.079646 | 0.00122 |
| GO:0140375 | immune receptor activity | 0.110294 | 0.00121 |
| GO:0003954 | NADH dehydrogenase activity | 0.152542 | 0.00121 |
| GO:0048812 | neuron projection morphogenesis | 0.089286 | 0.00118 |
| GO:0071704 | organic substance metabolic process | 0.049649 | 0.00118 |
| GO:0008285 | negative regulation of cell population proliferation | 0.074194 | 0.00115 |
| GO:1904018 | positive regulation of vasculature development | 0.107383 | 0.00113 |
| GO:0045766 | positive regulation of angiogenesis | 0.107383 | 0.00113 |
| GO:0070372 | regulation of ERK1 and ERK2 cascade | 0.089928 | 0.00111 |
| GO:0019001 | guanyl nucleotide binding | 0.073394 | 0.00111 |
| GO:0071331 | cellular response to hexose stimulus | 0.142857 | 0.00111 |
| GO:0051928 | positive regulation of calcium ion transport | 0.115702 | 0.00110 |
| GO:0034754 | cellular hormone metabolic process | 0.115702 | 0.00110 |
| GO:0032561 | guanyl ribonucleotide binding | 0.07362 | 0.00109 |
| GO:0034440 | lipid oxidation | 0.134146 | 0.00109 |
| GO:0030593 | neutrophil chemotaxis | 0.134146 | 0.00109 |
| GO:0002460 | adaptive immune response based on somatic recombination of immune receptors built from immunoglobulin superfamily domains | 0.134146 | 0.00109 |
| GO:0016779 | nucleotidyltransferase activity | 0.084507 | 0.00109 |
| GO:0050920 | regulation of chemotaxis | 0.097561 | 0.00108 |
| GO:0034364 | high-density lipoprotein particle | 0.222222 | 0.00107 |
| GO:0003955 | NAD(P)H dehydrogenase (quinone) activity | 0.155172 | 0.00106 |
| GO:0055088 | lipid homeostasis | 0.108108 | 0.00105 |
| GO:0030295 | protein kinase activator activity | 0.108108 | 0.00105 |
| GO:0044406 | adhesion of symbiont to host | 0.363636 | 0.00104 |
| GO:0016651 | oxidoreductase activity, acting on NAD(P)H | 0.121495 | 0.00103 |
| GO:0055080 | cation homeostasis | 0.078891 | 0.00102 |
| GO:0005886 | plasma membrane | 0.053506 | 0.00100 |
| GO:0071333 | cellular response to glucose stimulus | 0.144928 | 0.00099 |
| GO:1904950 | negative regulation of establishment of protein localization | 0.108844 | 0.00098 |
| GO:0016746 | acyltransferase activity | 0.086957 | 0.00098 |
| GO:0050769 | positive regulation of neurogenesis | 0.093117 | 0.00098 |
| GO:0033365 | protein localization to organelle | 0.072808 | 0.00097 |
| GO:0048568 | embryonic organ development | 0.112782 | 0.00097 |
| GO:0048010 | vascular endothelial growth factor receptor signaling pathway | 0.277778 | 0.00095 |
| GO:0031324 | negative regulation of cellular metabolic process | 0.05794 | 0.00094 |
| GO:0007160 | cell-matrix adhesion | 0.129032 | 0.00093 |
| GO:1902041 | regulation of extrinsic apoptotic signaling pathway via death domain receptors | 0.173913 | 0.00093 |
| GO:0030030 | cell projection organization | 0.066918 | 0.00092 |
| GO:0051262 | protein tetramerization | 0.102703 | 0.00091 |
| GO:0045089 | positive regulation of innate immune response | 0.10625 | 0.00091 |
| GO:0046649 | lymphocyte activation | 0.081395 | 0.00090 |
| GO:0006633 | fatty acid biosynthetic process | 0.118644 | 0.00086 |
| GO:0007199 | G protein-coupled receptor signaling pathway coupled to cGMP nucleotide second messenger | 0.6 | 0.00085 |
| GO:0072378 | blood coagulation, fibrin clot formation | 0.6 | 0.00085 |
| GO:0034141 | positive regulation of toll-like receptor 3 signaling pathway | 0.6 | 0.00085 |
| GO:0002933 | lipid hydroxylation | 0.6 | 0.00085 |
| GO:1905049 | negative regulation of metallopeptidase activity | 0.6 | 0.00085 |
| GO:0072201 | negative regulation of mesenchymal cell proliferation | 0.6 | 0.00085 |
| GO:0033227 | dsRNA transport | 0.6 | 0.00085 |
| GO:0042731 | PH domain binding | 0.6 | 0.00085 |
| GO:0046683 | response to organophosphorus | 0.110345 | 0.00084 |
| GO:0098771 | inorganic ion homeostasis | 0.078675 | 0.00084 |
| GO:0050866 | negative regulation of cell activation | 0.103825 | 0.00084 |
| GO:0048545 | response to steroid hormone | 0.09465 | 0.00084 |
| GO:0070330 | aromatase activity | 0.160714 | 0.00082 |
| GO:0015711 | organic anion transport | 0.092308 | 0.00082 |
| GO:0019221 | cytokine-mediated signaling pathway | 0.092308 | 0.00082 |
| GO:0072594 | establishment of protein localization to organelle | 0.084932 | 0.00081 |
| GO:0006793 | phosphorus metabolic process | 0.05899 | 0.00080 |
| GO:0006662 | glycerol ether metabolic process | 0.177778 | 0.00080 |
| GO:0035094 | response to nicotine | 0.177778 | 0.00080 |
| GO:0071322 | cellular response to carbohydrate stimulus | 0.139241 | 0.00079 |
| GO:1905368 | peptidase complex | 0.111111 | 0.00078 |
| GO:0043549 | regulation of kinase activity | 0.072702 | 0.00077 |
| GO:0051090 | regulation of DNA-binding transcription factor activity | 0.085399 | 0.00077 |
| GO:0005902 | microvillus | 0.131868 | 0.00077 |
| GO:0016125 | sterol metabolic process | 0.115385 | 0.00076 |
| GO:0004867 | serine-type endopeptidase inhibitor activity | 0.10828 | 0.00073 |
| GO:0045732 | positive regulation of protein catabolic process | 0.096234 | 0.00073 |
| GO:0051224 | negative regulation of protein transport | 0.111888 | 0.00073 |
| GO:0001676 | long-chain fatty acid metabolic process | 0.12069 | 0.00072 |
| GO:0015036 | disulfide oxidoreductase activity | 0.163636 | 0.00072 |
| GO:0050136 | NADH dehydrogenase (quinone) activity | 0.163636 | 0.00072 |
| GO:0008137 | NADH dehydrogenase (ubiquinone) activity | 0.163636 | 0.00072 |
| GO:0009895 | negative regulation of catabolic process | 0.089109 | 0.00072 |
| GO:0034114 | regulation of heterotypic cell-cell adhesion | 0.294118 | 0.00072 |
| GO:0033194 | response to hydroperoxide | 0.294118 | 0.00072 |
| GO:0042824 | MHC class I peptide loading complex | 0.294118 | 0.00072 |
| GO:0043023 | ribosomal large subunit binding | 0.294118 | 0.00072 |
| GO:0060548 | negative regulation of cell death | 0.064201 | 0.00071 |
| GO:0044419 | biological process involved in interspecies interaction between organisms | 0.061299 | 0.00071 |
| GO:0050921 | positive regulation of chemotaxis | 0.116279 | 0.00070 |
| GO:0006694 | steroid biosynthetic process | 0.116279 | 0.00070 |
| GO:0030176 | integral component of endoplasmic reticulum membrane | 0.116279 | 0.00070 |
| GO:0007265 | Ras protein signal transduction | 0.092199 | 0.00069 |
| GO:0030449 | regulation of complement activation | 0.24 | 0.00069 |
| GO:1902235 | regulation of endoplasmic reticulum stress-induced intrinsic apoptotic signaling pathway | 0.24 | 0.00069 |
| GO:0010612 | regulation of cardiac muscle adaptation | 0.4 | 0.00069 |
| GO:0034116 | positive regulation of heterotypic cell-cell adhesion | 0.4 | 0.00069 |
| GO:0006616 | SRP-dependent cotranslational protein targeting to membrane, translocation | 0.4 | 0.00069 |
| GO:1900102 | negative regulation of endoplasmic reticulum unfolded protein response | 0.4 | 0.00069 |
| GO:0043256 | laminin complex | 0.4 | 0.00069 |
| GO:0000038 | very long-chain fatty acid metabolic process | 0.205882 | 0.00067 |
| GO:0090066 | regulation of anatomical structure size | 0.081818 | 0.00067 |
| GO:0001933 | negative regulation of protein phosphorylation | 0.088146 | 0.00067 |
| GO:0016614 | oxidoreductase activity, acting on CH-OH group of donors | 0.089701 | 0.00067 |
| GO:0007030 | Golgi organization | 0.121739 | 0.00066 |
| GO:0000149 | SNARE binding | 0.121739 | 0.00066 |
| GO:0005539 | glycosaminoglycan binding | 0.09319 | 0.00065 |
| GO:0043087 | regulation of GTPase activity | 0.084656 | 0.00065 |
| GO:0042391 | regulation of membrane potential | 0.083538 | 0.00064 |
| GO:0019901 | protein kinase binding | 0.071795 | 0.00062 |
| GO:0060255 | regulation of macromolecule metabolic process | 0.0521 | 0.00060 |
| GO:0045860 | positive regulation of protein kinase activity | 0.084158 | 0.00060 |
| GO:0002697 | regulation of immune effector process | 0.085333 | 0.00060 |
| GO:0019838 | growth factor binding | 0.11039 | 0.00059 |
| GO:0031323 | regulation of cellular metabolic process | 0.052772 | 0.00058 |
| GO:0009225 | nucleotide-sugar metabolic process | 0.186047 | 0.00058 |
| GO:0046485 | ether lipid metabolic process | 0.186047 | 0.00058 |
| GO:0032994 | protein-lipid complex | 0.186047 | 0.00058 |
| GO:0003964 | RNA-directed DNA polymerase activity | 0.114286 | 0.00058 |
| GO:0019369 | arachidonic acid metabolic process | 0.144737 | 0.00057 |
| GO:0006605 | protein targeting | 0.095618 | 0.00057 |
| GO:0071621 | granulocyte chemotaxis | 0.136364 | 0.00057 |
| GO:0042562 | hormone binding | 0.136364 | 0.00057 |
| GO:0016358 | dendrite development | 0.212121 | 0.00056 |
| GO:0015291 | secondary active transmembrane transporter activity | 0.093284 | 0.00055 |
| GO:0042326 | negative regulation of phosphorylation | 0.086253 | 0.00055 |
| GO:0031301 | integral component of organelle membrane | 0.086253 | 0.00055 |
| GO:0030133 | transport vesicle | 0.096 | 0.00055 |
| GO:1903829 | positive regulation of protein localization | 0.081498 | 0.00055 |
| GO:0030315 | T-tubule | 0.169811 | 0.00054 |
| GO:0010611 | regulation of cardiac muscle hypertrophy | 0.15625 | 0.00054 |
| GO:0046887 | positive regulation of hormone secretion | 0.115108 | 0.00053 |
| GO:0031579 | membrane raft organization | 0.3125 | 0.00052 |
| GO:0008061 | chitin binding | 0.3125 | 0.00052 |
| GO:0016772 | transferase activity, transferring phosphorus-containing groups | 0.064094 | 0.00052 |
| GO:0071345 | cellular response to cytokine stimulus | 0.069286 | 0.00052 |
| GO:0030427 | site of polarized growth | 0.093985 | 0.00051 |
| GO:0030234 | enzyme regulator activity | 0.063015 | 0.00051 |
| GO:1903131 | mononuclear cell differentiation | 0.092466 | 0.00050 |
| GO:0031668 | cellular response to extracellular stimulus | 0.097166 | 0.00050 |
| GO:0045926 | negative regulation of growth | 0.097166 | 0.00050 |
| GO:0032496 | response to lipopolysaccharide | 0.083532 | 0.00049 |
| GO:0010720 | positive regulation of cell development | 0.090032 | 0.00049 |
| GO:0043470 | regulation of carbohydrate catabolic process | 0.190476 | 0.00049 |
| GO:0006656 | phosphatidylcholine biosynthetic process | 0.190476 | 0.00049 |
| GO:0031328 | positive regulation of cellular biosynthetic process | 0.062032 | 0.00048 |
| GO:0045321 | leukocyte activation | 0.078505 | 0.00048 |
| GO:0032559 | adenyl ribonucleotide binding | 0.059893 | 0.00048 |
| GO:0000302 | response to reactive oxygen species | 0.109091 | 0.00048 |
| GO:0022408 | negative regulation of cell-cell adhesion | 0.109091 | 0.00048 |
| GO:0001726 | ruffle | 0.109091 | 0.00048 |
| GO:0045834 | positive regulation of lipid metabolic process | 0.112583 | 0.00047 |
| GO:1904064 | positive regulation of cation transmembrane transport | 0.112583 | 0.00047 |
| GO:0060193 | positive regulation of lipase activity | 0.173077 | 0.00047 |
| GO:0071949 | FAD binding | 0.173077 | 0.00047 |
| GO:0051248 | negative regulation of protein metabolic process | 0.066451 | 0.00046 |
| GO:0120161 | regulation of cold-induced thermogenesis | 0.120968 | 0.00046 |
| GO:0120162 | positive regulation of cold-induced thermogenesis | 0.139535 | 0.00046 |
| GO:0071277 | cellular response to calcium ion | 0.139535 | 0.00046 |
| GO:0006829 | zinc ion transport | 0.21875 | 0.00046 |
| GO:0016298 | lipase activity | 0.116788 | 0.00045 |
| GO:0031330 | negative regulation of cellular catabolic process | 0.1 | 0.00045 |
| GO:0031329 | regulation of cellular catabolic process | 0.074963 | 0.00045 |
| GO:0042594 | response to starvation | 0.098765 | 0.00045 |
| GO:0071496 | cellular response to external stimulus | 0.09009 | 0.00043 |
| GO:0030426 | growth cone | 0.09542 | 0.00043 |
| GO:1903242 | regulation of cardiac muscle hypertrophy in response to stress | 0.444444 | 0.00043 |
| GO:1905048 | regulation of metallopeptidase activity | 0.444444 | 0.00043 |
| GO:0010226 | response to lithium ion | 0.26087 | 0.00042 |
| GO:0001891 | phagocytic cup | 0.26087 | 0.00042 |
| GO:0005385 | zinc ion transmembrane transporter activity | 0.26087 | 0.00042 |
| GO:0030054 | cell junction | 0.060674 | 0.00042 |
| GO:0006635 | fatty acid beta-oxidation | 0.16129 | 0.00042 |
| GO:0030554 | adenyl nucleotide binding | 0.05991 | 0.00042 |
| GO:0030551 | cyclic nucleotide binding | 0.195122 | 0.00041 |
| GO:0007264 | small GTPase mediated signal transduction | 0.086387 | 0.00041 |
| GO:0050764 | regulation of phagocytosis | 0.141176 | 0.00041 |
| GO:0033116 | endoplasmic reticulum-Golgi intermediate compartment membrane | 0.176471 | 0.00040 |
| GO:0008081 | phosphoric diester hydrolase activity | 0.134021 | 0.00040 |
| GO:0051726 | regulation of cell cycle | 0.067474 | 0.00040 |
| GO:0002831 | regulation of response to biotic stimulus | 0.086842 | 0.00040 |
| GO:0005887 | integral component of plasma membrane | 0.064935 | 0.00039 |
| GO:0051047 | positive regulation of secretion | 0.087533 | 0.00038 |
| GO:0031984 | organelle subcompartment | 0.094203 | 0.00037 |
| GO:0046469 | platelet activating factor metabolic process | 0.225806 | 0.00037 |
| GO:0007034 | vacuolar transport | 0.105528 | 0.00037 |
| GO:0052689 | carboxylic ester hydrolase activity | 0.105528 | 0.00037 |
| GO:0001775 | cell activation | 0.076797 | 0.00037 |
| GO:0046889 | positive regulation of lipid biosynthetic process | 0.142857 | 0.00037 |
| GO:1904427 | positive regulation of calcium ion transmembrane transport | 0.142857 | 0.00037 |
| GO:0043502 | regulation of muscle adaptation | 0.142857 | 0.00037 |
| GO:0060076 | excitatory synapse | 0.163934 | 0.00036 |
| GO:0007204 | positive regulation of cytosolic calcium ion concentration | 0.106061 | 0.00036 |
| GO:0031346 | positive regulation of cell projection organization | 0.085642 | 0.00035 |
| GO:0048646 | anatomical structure formation involved in morphogenesis | 0.073727 | 0.00034 |
| GO:1904951 | positive regulation of establishment of protein localization | 0.090909 | 0.00034 |
| GO:0007155 | cell adhesion | 0.070601 | 0.00033 |
| GO:0009719 | response to endogenous stimulus | 0.066929 | 0.00033 |
| GO:0099059 | integral component of presynaptic active zone membrane | 0.272727 | 0.00033 |
| GO:0033643 | host cell part | 0.272727 | 0.00033 |
| GO:0000041 | transition metal ion transport | 0.116438 | 0.00032 |
| GO:0098791 | Golgi apparatus subcompartment | 0.099206 | 0.00031 |
| GO:0042578 | phosphoric ester hydrolase activity | 0.08371 | 0.00030 |
| GO:1903319 | positive regulation of protein maturation | 0.233333 | 0.00030 |
| GO:0009891 | positive regulation of biosynthetic process | 0.0625 | 0.00030 |
| GO:0035023 | regulation of Rho protein signal transduction | 0.121212 | 0.00030 |
| GO:0016765 | transferase activity, transferring alkyl or aryl (other than methyl) groups | 0.121212 | 0.00030 |
| GO:0030662 | coated vesicle membrane | 0.183673 | 0.00029 |
| GO:1990777 | lipoprotein particle | 0.205128 | 0.00029 |
| GO:0034358 | plasma lipoprotein particle | 0.205128 | 0.00029 |
| GO:0031300 | intrinsic component of organelle membrane | 0.085575 | 0.00028 |
| GO:0051172 | negative regulation of nitrogen compound metabolic process | 0.059265 | 0.00027 |
| GO:0090287 | regulation of cellular response to growth factor stimulus | 0.099174 | 0.00027 |
| GO:0019210 | kinase inhibitor activity | 0.157143 | 0.00027 |
| GO:0046415 | urate metabolic process | 0.357143 | 0.00026 |
| GO:0044321 | response to leptin | 0.357143 | 0.00026 |
| GO:1902236 | negative regulation of endoplasmic reticulum stress-induced intrinsic apoptotic signaling pathway | 0.357143 | 0.00026 |
| GO:0045859 | regulation of protein kinase activity | 0.076805 | 0.00026 |
| GO:0022407 | regulation of cell-cell adhesion | 0.089385 | 0.00026 |
| GO:0002237 | response to molecule of bacterial origin | 0.085057 | 0.00025 |
| GO:0015035 | protein-disulfide reductase activity | 0.1875 | 0.00025 |
| GO:0019900 | kinase binding | 0.072414 | 0.00025 |
| GO:0071577 | zinc ion transmembrane transport | 0.285714 | 0.00025 |
| GO:0045088 | regulation of innate immune response | 0.1 | 0.00025 |
| GO:0005784 | Sec61 translocon complex | 0.5 | 0.00025 |
| GO:0005577 | fibrinogen complex | 0.5 | 0.00025 |
| GO:0005813 | centrosome | 0.076358 | 0.00024 |
| GO:0060627 | regulation of vesicle-mediated transport | 0.080292 | 0.00024 |
| GO:0046688 | response to copper ion | 0.241379 | 0.00024 |
| GO:1903573 | negative regulation of response to endoplasmic reticulum stress | 0.241379 | 0.00024 |
| GO:0006612 | protein targeting to membrane | 0.141304 | 0.00024 |
| GO:0098590 | plasma membrane region | 0.067675 | 0.00023 |
| GO:0005769 | early endosome | 0.091743 | 0.00023 |
| GO:0016616 | oxidoreductase activity, acting on the CH-OH group of donors, NAD or NADP as acceptor | 0.095745 | 0.00023 |
| GO:0042176 | regulation of protein catabolic process | 0.086047 | 0.00023 |
| GO:0009892 | negative regulation of metabolic process | 0.05704 | 0.00022 |
| GO:0010517 | regulation of phospholipase activity | 0.191489 | 0.00021 |
| GO:0046470 | phosphatidylcholine metabolic process | 0.161765 | 0.00021 |
| GO:0051347 | positive regulation of transferase activity | 0.079929 | 0.00020 |
| GO:0045746 | negative regulation of Notch signaling pathway | 0.216216 | 0.00020 |
| GO:0080135 | regulation of cellular response to stress | 0.07815 | 0.00020 |
| GO:0009636 | response to toxic substance | 0.137255 | 0.00019 |
| GO:0043408 | regulation of MAPK cascade | 0.0784 | 0.00019 |
| GO:0030552 | cAMP binding | 0.25 | 0.00019 |
| GO:0002833 | positive regulation of response to biotic stimulus | 0.108247 | 0.00019 |
| GO:0009267 | cellular response to starvation | 0.117647 | 0.00019 |
| GO:0050796 | regulation of insulin secretion | 0.111111 | 0.00019 |
| GO:0042277 | peptide binding | 0.089918 | 0.00018 |
| GO:0014743 | regulation of muscle hypertrophy | 0.164179 | 0.00018 |
| GO:0016655 | oxidoreductase activity, acting on NAD(P)H, quinone or similar compound as acceptor | 0.164179 | 0.00018 |
| GO:1900101 | regulation of endoplasmic reticulum unfolded protein response | 0.3 | 0.00018 |
| GO:0007165 | signal transduction | 0.054925 | 0.00018 |
| GO:0010942 | positive regulation of cell death | 0.078905 | 0.00018 |
| GO:0031669 | cellular response to nutrient levels | 0.105991 | 0.00018 |
| GO:0051707 | response to other organism | 0.064009 | 0.00018 |
| GO:0008250 | oligosaccharyltransferase complex | 0.384615 | 0.00017 |
| GO:1901137 | carbohydrate derivative biosynthetic process | 0.081227 | 0.00017 |
| GO:0006575 | cellular modified amino acid metabolic process | 0.109375 | 0.00016 |
| GO:0006644 | phospholipid metabolic process | 0.089005 | 0.00016 |
| GO:2001236 | regulation of extrinsic apoptotic signaling pathway | 0.123188 | 0.00016 |
| GO:0031349 | positive regulation of defense response | 0.099265 | 0.00016 |
| GO:0031966 | mitochondrial membrane | 0.073512 | 0.00016 |
| GO:0051235 | maintenance of location | 0.115854 | 0.00015 |
| GO:0043539 | protein serine/threonine kinase activator activity | 0.14 | 0.00015 |
| GO:0061041 | regulation of wound healing | 0.147727 | 0.00015 |
| GO:0002793 | positive regulation of peptide secretion | 0.133929 | 0.00015 |
| GO:0071709 | membrane assembly | 0.181818 | 0.00015 |
| GO:0098945 | intrinsic component of presynaptic active zone membrane | 0.259259 | 0.00015 |
| GO:0006690 | icosanoid metabolic process | 0.124088 | 0.00015 |
| GO:0031227 | intrinsic component of endoplasmic reticulum membrane | 0.124088 | 0.00015 |
| GO:0051962 | positive regulation of nervous system development | 0.095082 | 0.00015 |
| GO:0030658 | transport vesicle membrane | 0.129032 | 0.00014 |
| GO:0014074 | response to purine-containing compound | 0.116564 | 0.00014 |
| GO:0034330 | cell junction organization | 0.086854 | 0.00014 |
| GO:0007409 | axonogenesis | 0.110526 | 0.00014 |
| GO:0004860 | protein kinase inhibitor activity | 0.169231 | 0.00014 |
| GO:0002376 | immune system process | 0.061327 | 0.00014 |
| GO:0007032 | endosome organization | 0.135135 | 0.00014 |
| GO:0001818 | negative regulation of cytokine production | 0.105263 | 0.00013 |
| GO:0051093 | negative regulation of developmental process | 0.071573 | 0.00013 |
| GO:1901701 | cellular response to oxygen-containing compound | 0.069588 | 0.00013 |
| GO:0010632 | regulation of epithelial cell migration | 0.111111 | 0.00013 |
| GO:0044057 | regulation of system process | 0.082397 | 0.00013 |
| GO:0015990 | electron transport coupled proton transport | 0.571429 | 0.00013 |
| GO:0015988 | energy coupled proton transmembrane transport, against electrochemical gradient | 0.571429 | 0.00013 |
| GO:0031406 | carboxylic acid binding | 0.114286 | 0.00013 |
| GO:0051668 | localization within membrane | 0.083665 | 0.00012 |
| GO:0042596 | fear response | 0.142857 | 0.00012 |
| GO:0007267 | cell-cell signaling | 0.089286 | 0.00012 |
| GO:0031400 | negative regulation of protein modification process | 0.083333 | 0.00011 |
| GO:0005496 | steroid binding | 0.126866 | 0.00011 |
| GO:0005583 | fibrillar collagen trimer | 0.416667 | 0.00011 |
| GO:0044322 | endoplasmic reticulum quality control compartment | 0.416667 | 0.00011 |
| GO:0002209 | behavioral defense response | 0.14433 | 0.00011 |
| GO:0099056 | integral component of presynaptic membrane | 0.14433 | 0.00011 |
| GO:0045661 | regulation of myoblast differentiation | 0.188679 | 0.00011 |
| GO:0005044 | scavenger receptor activity | 0.188679 | 0.00011 |
| GO:0070613 | regulation of protein processing | 0.174603 | 0.00010 |
| GO:0030134 | COPII-coated ER to Golgi transport vesicle | 0.235294 | 0.00010 |
| GO:0055082 | cellular chemical homeostasis | 0.08502 | 0.00010 |
| GO:0019866 | organelle inner membrane | 0.079812 | 0.00010 |
| GO:0050660 | flavin adenine dinucleotide binding | 0.138889 | 0.00010 |
| GO:0033218 | amide binding | 0.086393 | 0.00010 |
| GO:0050900 | leukocyte migration | 0.110553 | 0.00010 |
| GO:0046890 | regulation of lipid biosynthetic process | 0.113514 | 0.00010 |
| GO:0036041 | long-chain fatty acid binding | 0.333333 | 0.00009 |
| GO:0071466 | cellular response to xenobiotic stimulus | 0.164384 | 0.00009 |
| GO:0051087 | chaperone binding | 0.128788 | 0.00009 |
| GO:0009743 | response to carbohydrate | 0.116959 | 0.00009 |
| GO:0033764 | steroid dehydrogenase activity, acting on the CH-OH group of donors, NAD or NADP as acceptor | 0.192308 | 0.00009 |
| GO:0006935 | chemotaxis | 0.091146 | 0.00009 |
| GO:0042330 | taxis | 0.091146 | 0.00009 |
| GO:0043686 | co-translational protein modification | 1 | 0.00009 |
| GO:0019748 | secondary metabolic process | 0.177419 | 0.00009 |
| GO:0072329 | monocarboxylic acid catabolic process | 0.140187 | 0.00009 |
| GO:0002521 | leukocyte differentiation | 0.094955 | 0.00009 |
| GO:0001662 | behavioral fear response | 0.147368 | 0.00009 |
| GO:0001562 | response to protozoan | 0.242424 | 0.00008 |
| GO:0010595 | positive regulation of endothelial cell migration | 0.156627 | 0.00008 |
| GO:0042981 | regulation of apoptotic process | 0.06405 | 0.00008 |
| GO:0007568 | aging | 0.101887 | 0.00008 |
| GO:0048513 | animal organ development | 0.066416 | 0.00008 |
| GO:0031974 | membrane-enclosed lumen | 0.076074 | 0.00007 |
| GO:0043233 | organelle lumen | 0.076074 | 0.00007 |
| GO:0070013 | intracellular organelle lumen | 0.076167 | 0.00007 |
| GO:1903322 | positive regulation of protein modification by small protein conjugation or removal | 0.119048 | 0.00007 |
| GO:0099572 | postsynaptic specialization | 0.090909 | 0.00007 |
| GO:1902600 | proton transmembrane transport | 0.169014 | 0.00007 |
| GO:0001678 | cellular glucose homeostasis | 0.150538 | 0.00007 |
| GO:0009404 | toxin metabolic process | 0.352941 | 0.00007 |
| GO:1903035 | negative regulation of response to wounding | 0.183333 | 0.00007 |
| GO:0009725 | response to hormone | 0.077023 | 0.00007 |
| GO:0014069 | postsynaptic density | 0.091603 | 0.00007 |
| GO:0006613 | cotranslational protein targeting to membrane | 0.291667 | 0.00006 |
| GO:0015078 | proton transmembrane transporter activity | 0.103053 | 0.00006 |
| GO:0051171 | regulation of nitrogen compound metabolic process | 0.054118 | 0.00006 |
| GO:0035249 | synaptic transmission, glutamatergic | 0.160494 | 0.00006 |
| GO:0001701 | in utero embryonic development | 0.096875 | 0.00006 |
| GO:1901565 | organonitrogen compound catabolic process | 0.075058 | 0.00006 |
| GO:0006749 | glutathione metabolic process | 0.171429 | 0.00006 |
| GO:1901135 | carbohydrate derivative metabolic process | 0.068433 | 0.00006 |
| GO:0031226 | intrinsic component of plasma membrane | 0.06701 | 0.00006 |
| GO:0045862 | positive regulation of proteolysis | 0.09621 | 0.00006 |
| GO:1903034 | regulation of response to wounding | 0.13913 | 0.00006 |
| GO:0048471 | perinuclear region of cytoplasm | 0.075581 | 0.00006 |
| GO:0050767 | regulation of neurogenesis | 0.093023 | 0.00006 |
| GO:0001558 | regulation of cell growth | 0.089655 | 0.00006 |
| GO:0006820 | anion transport | 0.094183 | 0.00005 |
| GO:0046883 | regulation of hormone secretion | 0.10219 | 0.00005 |
| GO:0071241 | cellular response to inorganic substance | 0.100671 | 0.00005 |
| GO:0005604 | basement membrane | 0.140351 | 0.00005 |
| GO:0009749 | response to glucose | 0.130435 | 0.00005 |
| GO:0008270 | zinc ion binding | 0.071181 | 0.00005 |
| GO:1901698 | response to nitrogen compound | 0.069539 | 0.00005 |
| GO:0006614 | SRP-dependent cotranslational protein targeting to membrane | 0.304348 | 0.00005 |
| GO:0009986 | cell surface | 0.078484 | 0.00005 |
| GO:0019887 | protein kinase regulator activity | 0.105469 | 0.00005 |
| GO:0055114 | obsolete oxidation-reduction process | 0.126667 | 0.00005 |
| GO:0120254 | olefinic compound metabolic process | 0.126667 | 0.00005 |
| GO:0050819 | negative regulation of coagulation | 0.230769 | 0.00005 |
| GO:0008152 | metabolic process | 0.050735 | 0.00004 |
| GO:0043009 | chordate embryonic development | 0.097264 | 0.00004 |
| GO:0043067 | regulation of programmed cell death | 0.064763 | 0.00004 |
| GO:0031398 | positive regulation of protein ubiquitination | 0.127517 | 0.00004 |
| GO:1905897 | regulation of response to endoplasmic reticulum stress | 0.192982 | 0.00004 |
| GO:1903317 | regulation of protein maturation | 0.179104 | 0.00004 |
| GO:0060191 | regulation of lipase activity | 0.179104 | 0.00004 |
| GO:0005787 | signal peptidase complex | 0.5 | 0.00004 |
| GO:0031325 | positive regulation of cellular metabolic process | 0.060567 | 0.00004 |
| GO:0060284 | regulation of cell development | 0.087041 | 0.00004 |
| GO:0035639 | purine ribonucleoside triphosphate binding | 0.060834 | 0.00004 |
| GO:0061045 | negative regulation of wound healing | 0.212766 | 0.00004 |
| GO:0046915 | transition metal ion transmembrane transporter activity | 0.212766 | 0.00004 |
| GO:1900047 | negative regulation of hemostasis | 0.236842 | 0.00004 |
| GO:0034284 | response to monosaccharide | 0.129252 | 0.00003 |
| GO:0010975 | regulation of neuron projection development | 0.088477 | 0.00003 |
| GO:0005793 | endoplasmic reticulum-Golgi intermediate compartment | 0.122093 | 0.00003 |
| GO:0044242 | cellular lipid catabolic process | 0.118919 | 0.00003 |
| GO:0045787 | positive regulation of cell cycle | 0.097633 | 0.00003 |
| GO:0046486 | glycerolipid metabolic process | 0.096419 | 0.00003 |
| GO:0005319 | lipid transporter activity | 0.122807 | 0.00003 |
| GO:0042832 | defense response to protozoan | 0.275862 | 0.00003 |
| GO:0098889 | intrinsic component of presynaptic membrane | 0.146789 | 0.00003 |
| GO:0030195 | negative regulation of blood coagulation | 0.243243 | 0.00003 |
| GO:0033993 | response to lipid | 0.073473 | 0.00003 |
| GO:0009062 | fatty acid catabolic process | 0.162791 | 0.00003 |
| GO:0006790 | sulfur compound metabolic process | 0.098214 | 0.00003 |
| GO:0019207 | kinase regulator activity | 0.102389 | 0.00003 |
| GO:0016601 | Rac protein signal transduction | 0.173333 | 0.00003 |
| GO:0072657 | protein localization to membrane | 0.091981 | 0.00003 |
| GO:0002684 | positive regulation of immune system process | 0.074447 | 0.00003 |
| GO:0019637 | organophosphate metabolic process | 0.069013 | 0.00003 |
| GO:0051336 | regulation of hydrolase activity | 0.071661 | 0.00002 |
| GO:0097494 | regulation of vesicle size | 0.1875 | 0.00002 |
| GO:0005576 | extracellular region | 0.072426 | 0.00002 |
| GO:0004089 | carbonate dehydratase activity | 0.333333 | 0.00002 |
| GO:0030595 | leukocyte chemotaxis | 0.137405 | 0.00002 |
| GO:0051338 | regulation of transferase activity | 0.076923 | 0.00002 |
| GO:0050818 | regulation of coagulation | 0.203704 | 0.00002 |
| GO:0002250 | adaptive immune response | 0.103448 | 0.00002 |
| GO:1901575 | organic substance catabolic process | 0.064208 | 0.00002 |
| GO:0015318 | inorganic molecular entity transmembrane transporter activity | 0.075612 | 0.00002 |
| GO:0019222 | regulation of metabolic process | 0.053628 | 0.00002 |
| GO:0001934 | positive regulation of protein phosphorylation | 0.080054 | 0.00002 |
| GO:0009790 | embryo development | 0.098266 | 0.00002 |
| GO:0009792 | embryo development ending in birth or egg hatching | 0.098266 | 0.00002 |
| GO:0045597 | positive regulation of cell differentiation | 0.077844 | 0.00002 |
| GO:0016229 | steroid dehydrogenase activity | 0.190476 | 0.00002 |
| GO:0030199 | collagen fibril organization | 0.227273 | 0.00002 |
| GO:0001765 | membrane raft assembly | 0.555556 | 0.00002 |
| GO:1900046 | regulation of hemostasis | 0.207547 | 0.00002 |
| GO:0016050 | vesicle organization | 0.107011 | 0.00002 |
| GO:0009746 | response to hexose | 0.134752 | 0.00002 |
| GO:0033500 | carbohydrate homeostasis | 0.112069 | 0.00002 |
| GO:0043112 | receptor metabolic process | 0.168675 | 0.00002 |
| GO:0120035 | regulation of plasma membrane bounded cell projection organization | 0.082335 | 0.00002 |
| GO:0016740 | transferase activity | 0.05945 | 0.00002 |
| GO:0002523 | leukocyte migration involved in inflammatory response | 0.428571 | 0.00002 |
| GO:0042593 | glucose homeostasis | 0.112554 | 0.00002 |
| GO:0098772 | molecular function regulator activity | 0.063113 | 0.00002 |
| GO:0023056 | positive regulation of signaling | 0.065789 | 0.00002 |
| GO:0040011 | locomotion | 0.094527 | 0.00002 |
| GO:0060326 | cell chemotaxis | 0.115207 | 0.00002 |
| GO:0007275 | multicellular organism development | 0.083728 | 0.00002 |
| GO:0005743 | mitochondrial inner membrane | 0.086505 | 0.00002 |
| GO:0033293 | monocarboxylic acid binding | 0.170732 | 0.00002 |
| GO:0030193 | regulation of blood coagulation | 0.211538 | 0.00002 |
| GO:0051036 | regulation of endosome size | 0.196721 | 0.00001 |
| GO:1901615 | organic hydroxy compound metabolic process | 0.088975 | 0.00001 |
| GO:0080090 | regulation of primary metabolic process | 0.055066 | 0.00001 |
| GO:0016675 | oxidoreductase activity, acting on a heme group of donors | 0.215686 | 0.00001 |
| GO:0010647 | positive regulation of cell communication | 0.066599 | 0.00001 |
| GO:0010594 | regulation of endothelial cell migration | 0.138686 | 0.00001 |
| GO:0009408 | response to heat | 0.144 | 0.00001 |
| GO:0022853 | active ion transmembrane transporter activity | 0.101796 | 0.00001 |
| GO:0002683 | negative regulation of immune system process | 0.096447 | 0.00001 |
| GO:0072330 | monocarboxylic acid biosynthetic process | 0.130435 | 0.00001 |
| GO:0030027 | lamellipodium | 0.115044 | 0.00001 |
| GO:0045177 | apical part of cell | 0.139706 | 0.00001 |
| GO:0009967 | positive regulation of signal transduction | 0.068735 | 0.00001 |
| GO:0030659 | cytoplasmic vesicle membrane | 0.081717 | 0.00001 |
| GO:0030258 | lipid modification | 0.13125 | 0.00001 |
| GO:0004129 | cytochrome-c oxidase activity | 0.22 | 0.00001 |
| GO:0010522 | regulation of calcium ion transport into cytosol | 0.461538 | 0.00001 |
| GO:0031639 | plasminogen activation | 0.461538 | 0.00001 |
| GO:0098794 | postsynapse | 0.103976 | 0.00001 |
| GO:0008392 | arachidonic acid epoxygenase activity | 0.243902 | 0.00001 |
| GO:0033555 | multicellular organismal response to stress | 0.146341 | 0.00001 |
| GO:0090407 | organophosphate biosynthetic process | 0.08309 | 0.00001 |
| GO:0012506 | vesicle membrane | 0.081879 | 0.00001 |
| GO:0008395 | steroid hydroxylase activity | 0.168539 | 0.00001 |
| GO:0031344 | regulation of cell projection organization | 0.083333 | 0.00001 |
| GO:0009987 | cellular process | 0.050162 | 0.00001 |
| GO:0043229 | intracellular organelle | 0.052843 | 0.00001 |
| GO:0005488 | binding | 0.052669 | 0.00001 |
| GO:0042127 | regulation of cell population proliferation | 0.06774 | 0.00001 |
| GO:0033559 | unsaturated fatty acid metabolic process | 0.141791 | 0.00001 |
| GO:0065007 | biological regulation | 0.057267 | 0.00001 |
| GO:0032102 | negative regulation of response to external stimulus | 0.101744 | 0.00001 |
| GO:0043226 | organelle | 0.05296 | 0.00001 |
| GO:0032880 | regulation of protein localization | 0.078212 | 0.00001 |
| GO:0043025 | neuronal cell body | 0.087248 | 0.00001 |
| GO:0110165 | cellular anatomical entity | 0.05164 | 0.00001 |
| GO:0050794 | regulation of cellular process | 0.056695 | 0.00001 |
| GO:0043231 | intracellular membrane-bounded organelle | 0.065426 | 0.00001 |
| GO:0003824 | catalytic activity | 0.069031 | 0.00001 |
| GO:0050789 | regulation of biological process | 0.05639 | 0.00001 |
| GO:0005515 | protein binding | 0.063514 | 0.00001 |
| GO:0016020 | membrane | 0.063048 | 0.00001 |
| GO:0043227 | membrane-bounded organelle | 0.065297 | 0.00001 |
| GO:0031224 | intrinsic component of membrane | 0.062114 | 0.00001 |
| GO:1901265 | nucleoside phosphate binding | 0.060372 | 0.00001 |
| GO:0000166 | nucleotide binding | 0.060372 | 0.00001 |
| GO:0016021 | integral component of membrane | 0.061616 | 0.00001 |
| GO:0003674 | molecular_function | 0.047664 | 0.00001 |
| GO:0051130 | positive regulation of cellular component organization | 0.075437 | 0.00001 |
| GO:0043167 | ion binding | 0.063685 | 0.00001 |
| GO:0050808 | synapse organization | 0.11588 | 0.00001 |
| GO:0032502 | developmental process | 0.064726 | 0.00001 |
| GO:0010941 | regulation of cell death | 0.066351 | 0.00001 |
| GO:0005504 | fatty acid binding | 0.210526 | 0.00001 |
| GO:0048518 | positive regulation of biological process | 0.066245 | 0.00001 |
| GO:0050708 | regulation of protein secretion | 0.109929 | 0.00001 |
| GO:0043207 | response to external biotic stimulus | 0.066764 | 0.00001 |
| GO:0009653 | anatomical structure morphogenesis | 0.070243 | 0.00001 |
| GO:0007610 | behavior | 0.083333 | 0.00001 |
| GO:0032553 | ribonucleotide binding | 0.063075 | 0.00001 |
| GO:0017076 | purine nucleotide binding | 0.062767 | 0.00001 |
| GO:0060341 | regulation of cellular localization | 0.076617 | 0.00001 |
| GO:0048522 | positive regulation of cellular process | 0.065717 | 0.00001 |
| GO:0048519 | negative regulation of biological process | 0.065091 | 0.00001 |
| GO:0050896 | response to stimulus | 0.073101 | 0.00001 |
| GO:0043169 | cation binding | 0.064328 | 0.00001 |
| GO:0032555 | purine ribonucleotide binding | 0.062831 | 0.00001 |
| GO:0090276 | regulation of peptide hormone secretion | 0.119266 | 0.00001 |
| GO:0005737 | cytoplasm | 0.06112 | 0.00001 |
| GO:0009607 | response to biotic stimulus | 0.066827 | 0.00001 |
| GO:0046872 | metal ion binding | 0.06347 | 0.00001 |
| GO:0048856 | anatomical structure development | 0.063846 | 0.00001 |
| GO:0048523 | negative regulation of cellular process | 0.067149 | 0.00001 |
| GO:0007166 | cell surface receptor signaling pathway | 0.068568 | 0.00001 |
| GO:0010035 | response to inorganic substance | 0.079861 | 0.00001 |
| GO:0036094 | small molecule binding | 0.061606 | 0.00001 |
| GO:0042327 | positive regulation of phosphorylation | 0.08125 | 0.00001 |
| GO:0005829 | cytosol | 0.066951 | 0.00001 |
| GO:0006066 | alcohol metabolic process | 0.100543 | 0.00001 |
| GO:0006890 | retrograde vesicle-mediated transport, Golgi to endoplasmic reticulum | 0.234043 | 0.00001 |
| GO:0043085 | positive regulation of catalytic activity | 0.075771 | 0.00001 |
| GO:0051179 | localization | 0.082098 | 0.00001 |
| GO:0051234 | establishment of localization | 0.083661 | 0.00001 |
| GO:0048583 | regulation of response to stimulus | 0.073871 | 0.00001 |
| GO:0051240 | positive regulation of multicellular organismal process | 0.070012 | 0.00001 |
| GO:0009893 | positive regulation of metabolic process | 0.064194 | 0.00001 |
| GO:0022890 | inorganic cation transmembrane transporter activity | 0.08377 | 0.00001 |
| GO:0009605 | response to external stimulus | 0.071429 | 0.00001 |
| GO:0042221 | response to chemical | 0.079612 | 0.00001 |
| GO:0031090 | organelle membrane | 0.082241 | 0.00001 |
| GO:0031347 | regulation of defense response | 0.08769 | 0.00001 |
| GO:0006810 | transport | 0.085935 | 0.00001 |
| GO:0065008 | regulation of biological quality | 0.073845 | 0.00001 |
| GO:0023051 | regulation of signaling | 0.072351 | 0.00001 |
| GO:0006950 | response to stress | 0.079128 | 0.00001 |
| GO:1903320 | regulation of protein modification by small protein conjugation or removal | 0.109589 | <0.00001 |
| GO:0010033 | response to organic substance | 0.077606 | <0.00001 |
| GO:0019373 | epoxygenase P450 pathway | 0.263158 | <0.00001 |
| GO:0010604 | positive regulation of macromolecule metabolic process | 0.062241 | <0.00001 |
| GO:0016053 | organic acid biosynthetic process | 0.107143 | <0.00001 |
| GO:0048869 | cellular developmental process | 0.063013 | <0.00001 |
| GO:0010646 | regulation of cell communication | 0.072722 | <0.00001 |
| GO:0006952 | defense response | 0.069945 | <0.00001 |
| GO:0065009 | regulation of molecular function | 0.069121 | <0.00001 |
| GO:0042802 | identical protein binding | 0.073868 | <0.00001 |
| GO:0008324 | cation transmembrane transporter activity | 0.081081 | <0.00001 |
| GO:0051239 | regulation of multicellular organismal process | 0.078895 | <0.00001 |
| GO:0009966 | regulation of signal transduction | 0.07508 | <0.00001 |
| GO:0016787 | hydrolase activity | 0.066206 | <0.00001 |
| GO:0019899 | enzyme binding | 0.070413 | <0.00001 |
| GO:0005102 | signaling receptor binding | 0.080042 | <0.00001 |
| GO:0050793 | regulation of developmental process | 0.074257 | <0.00001 |
| GO:0070727 | cellular macromolecule localization | 0.082431 | <0.00001 |
| GO:0051173 | positive regulation of nitrogen compound metabolic process | 0.065955 | <0.00001 |
| GO:0007162 | negative regulation of cell adhesion | 0.114173 | <0.00001 |
| GO:0043168 | anion binding | 0.068862 | <0.00001 |
| GO:0044248 | cellular catabolic process | 0.073915 | <0.00001 |
| GO:0051716 | cellular response to stimulus | 0.072989 | <0.00001 |
| GO:0051223 | regulation of protein transport | 0.093284 | <0.00001 |
| GO:0051128 | regulation of cellular component organization | 0.06688 | <0.00001 |
| GO:0046394 | carboxylic acid biosynthetic process | 0.107843 | <0.00001 |
| GO:0097367 | carbohydrate derivative binding | 0.067885 | <0.00001 |
| GO:0051641 | cellular localization | 0.077978 | <0.00001 |
| GO:0016477 | cell migration | 0.081683 | <0.00001 |
| GO:1902531 | regulation of intracellular signal transduction | 0.070231 | <0.00001 |
| GO:0007154 | cell communication | 0.085843 | <0.00001 |
| GO:2001233 | regulation of apoptotic signaling pathway | 0.100251 | <0.00001 |
| GO:0019752 | carboxylic acid metabolic process | 0.076341 | <0.00001 |
| GO:0070887 | cellular response to chemical stimulus | 0.072664 | <0.00001 |
| GO:0071702 | organic substance transport | 0.082714 | <0.00001 |
| GO:0015075 | ion transmembrane transporter activity | 0.078154 | <0.00001 |
| GO:0051246 | regulation of protein metabolic process | 0.070437 | <0.00001 |
| GO:0048585 | negative regulation of response to stimulus | 0.083703 | <0.00001 |
| GO:0050790 | regulation of catalytic activity | 0.070829 | <0.00001 |
| GO:0006082 | organic acid metabolic process | 0.078576 | <0.00001 |
| GO:0042995 | cell projection | 0.077033 | <0.00001 |
| GO:0009056 | catabolic process | 0.068885 | <0.00001 |
| GO:0044281 | small molecule metabolic process | 0.082045 | <0.00001 |
| GO:0071705 | nitrogen compound transport | 0.075808 | <0.00001 |
| GO:0031982 | vesicle | 0.082536 | <0.00001 |
| GO:0033036 | macromolecule localization | 0.083178 | <0.00001 |
| GO:0120025 | plasma membrane bounded cell projection | 0.078046 | <0.00001 |
| GO:0005768 | endosome | 0.083671 | <0.00001 |
| GO:0098588 | bounding membrane of organelle | 0.090062 | <0.00001 |
| GO:0033554 | cellular response to stress | 0.076561 | <0.00001 |
| GO:0030154 | cell differentiation | 0.073684 | <0.00001 |
| GO:0007528 | neuromuscular junction development | 0.172043 | <0.00001 |
| GO:0032787 | monocarboxylic acid metabolic process | 0.080628 | <0.00001 |
| GO:2000026 | regulation of multicellular organismal development | 0.088889 | <0.00001 |
| GO:0016491 | oxidoreductase activity | 0.076741 | <0.00001 |
| GO:0051049 | regulation of transport | 0.078057 | <0.00001 |
| GO:0046914 | transition metal ion binding | 0.080606 | <0.00001 |
| GO:0006886 | intracellular protein transport | 0.08168 | <0.00001 |
| GO:0045937 | positive regulation of phosphate metabolic process | 0.079955 | <0.00001 |
| GO:0010562 | positive regulation of phosphorus metabolic process | 0.079955 | <0.00001 |
| GO:0005615 | extracellular space | 0.091434 | <0.00001 |
| GO:0046983 | protein dimerization activity | 0.081976 | <0.00001 |
| GO:0040008 | regulation of growth | 0.086892 | <0.00001 |
| GO:0043436 | oxoacid metabolic process | 0.076766 | <0.00001 |
| GO:0048584 | positive regulation of response to stimulus | 0.072284 | <0.00001 |
| GO:0005789 | endoplasmic reticulum membrane | 0.126144 | <0.00001 |
| GO:0032879 | regulation of localization | 0.075437 | <0.00001 |
| GO:0006811 | ion transport | 0.093245 | <0.00001 |
| GO:0051247 | positive regulation of protein metabolic process | 0.08208 | <0.00001 |
| GO:0051094 | positive regulation of developmental process | 0.077217 | <0.00001 |
| GO:0008104 | protein localization | 0.082586 | <0.00001 |
| GO:0044093 | positive regulation of molecular function | 0.074646 | <0.00001 |
| GO:0009894 | regulation of catabolic process | 0.080169 | <0.00001 |
| GO:0008289 | lipid binding | 0.092795 | <0.00001 |
| GO:0022857 | transmembrane transporter activity | 0.084972 | <0.00001 |
| GO:1901700 | response to oxygen-containing compound | 0.079496 | <0.00001 |
| GO:0046907 | intracellular transport | 0.081133 | <0.00001 |
| GO:0044877 | protein-containing complex binding | 0.079202 | <0.00001 |
| GO:0050776 | regulation of immune response | 0.08086 | <0.00001 |
| GO:0043005 | neuron projection | 0.084015 | <0.00001 |
| GO:0051960 | regulation of nervous system development | 0.096436 | <0.00001 |
| GO:0034097 | response to cytokine | 0.076923 | <0.00001 |
| GO:0009991 | response to extracellular stimulus | 0.091075 | <0.00001 |
| GO:0005739 | mitochondrion | 0.080757 | <0.00001 |
| GO:0005794 | Golgi apparatus | 0.085944 | <0.00001 |
| GO:0001932 | regulation of protein phosphorylation | 0.082437 | <0.00001 |
| GO:0023057 | negative regulation of signaling | 0.085693 | <0.00001 |
| GO:0030425 | dendrite | 0.090177 | <0.00001 |
| GO:0051649 | establishment of localization in cell | 0.074114 | <0.00001 |
| GO:0019725 | cellular homeostasis | 0.090323 | <0.00001 |
| GO:0044297 | cell body | 0.086771 | <0.00001 |
| GO:0016192 | vesicle-mediated transport | 0.086773 | <0.00001 |
| GO:0010648 | negative regulation of cell communication | 0.085949 | <0.00001 |
| GO:0005215 | transporter activity | 0.087605 | <0.00001 |
| GO:0097708 | intracellular vesicle | 0.085504 | <0.00001 |
| GO:0042592 | homeostatic process | 0.078888 | <0.00001 |
| GO:0031410 | cytoplasmic vesicle | 0.085082 | <0.00001 |
| GO:0098655 | cation transmembrane transport | 0.104623 | <0.00001 |
| GO:0005783 | endoplasmic reticulum | 0.125177 | <0.00001 |
| GO:0010524 | positive regulation of calcium ion transport into cytosol | 0.545455 | <0.00001 |
| GO:0020005 | symbiont-containing vacuole membrane | 0.545455 | <0.00001 |
| GO:0071310 | cellular response to organic substance | 0.073082 | <0.00001 |
| GO:0001881 | receptor recycling | 0.209677 | <0.00001 |
| GO:0009266 | response to temperature stimulus | 0.126904 | <0.00001 |
| GO:0045184 | establishment of protein localization | 0.080483 | <0.00001 |
| GO:0031399 | regulation of protein modification process | 0.07963 | <0.00001 |
| GO:0018196 | peptidyl-asparagine modification | 0.363636 | <0.00001 |
| GO:0044255 | cellular lipid metabolic process | 0.105314 | <0.00001 |
| GO:0006629 | lipid metabolic process | 0.108515 | <0.00001 |
| GO:0005506 | iron ion binding | 0.106061 | <0.00001 |
| GO:0042325 | regulation of phosphorylation | 0.0824 | <0.00001 |
| GO:0015031 | protein transport | 0.079659 | <0.00001 |
| GO:0009968 | negative regulation of signal transduction | 0.087821 | <0.00001 |
| GO:0031667 | response to nutrient levels | 0.092664 | <0.00001 |
| GO:0048870 | cell motility | 0.080386 | <0.00001 |
| GO:0045595 | regulation of cell differentiation | 0.073024 | <0.00001 |
| GO:0098796 | membrane protein complex | 0.073295 | <0.00001 |
| GO:0009628 | response to abiotic stimulus | 0.078599 | <0.00001 |
| GO:0045454 | cell redox homeostasis | 0.197183 | <0.00001 |
| GO:0002682 | regulation of immune system process | 0.075893 | <0.00001 |
| GO:0014070 | response to organic cyclic compound | 0.086576 | <0.00001 |
| GO:0031396 | regulation of protein ubiquitination | 0.114943 | <0.00001 |
| GO:0051174 | regulation of phosphorus metabolic process | 0.078484 | <0.00001 |
| GO:0006869 | lipid transport | 0.113402 | <0.00001 |
| GO:0019220 | regulation of phosphate metabolic process | 0.078538 | <0.00001 |
| GO:0030155 | regulation of cell adhesion | 0.098746 | <0.00001 |
| GO:0009617 | response to bacterium | 0.088427 | <0.00001 |
| GO:0051050 | positive regulation of transport | 0.086911 | <0.00001 |
| GO:0032101 | regulation of response to external stimulus | 0.095185 | <0.00001 |
| GO:2000145 | regulation of cell motility | 0.096305 | <0.00001 |
| GO:0016788 | hydrolase activity, acting on ester bonds | 0.078313 | <0.00001 |
| GO:0051345 | positive regulation of hydrolase activity | 0.091071 | <0.00001 |
| GO:0010243 | response to organonitrogen compound | 0.084813 | <0.00001 |
| GO:0016054 | organic acid catabolic process | 0.113553 | <0.00001 |
| GO:0080134 | regulation of response to stress | 0.088538 | <0.00001 |
| GO:0034220 | ion transmembrane transport | 0.093357 | <0.00001 |
| GO:0000139 | Golgi membrane | 0.118492 | <0.00001 |
| GO:0098660 | inorganic ion transmembrane transport | 0.101655 | <0.00001 |
| GO:0022603 | regulation of anatomical structure morphogenesis | 0.08548 | <0.00001 |
| GO:0098662 | inorganic cation transmembrane transport | 0.10733 | <0.00001 |
| GO:0062023 | collagen-containing extracellular matrix | 0.157459 | <0.00001 |
| GO:2000147 | positive regulation of cell motility | 0.102362 | <0.00001 |
| GO:0030001 | metal ion transport | 0.097122 | <0.00001 |
| GO:0006954 | inflammatory response | 0.098969 | <0.00001 |
| GO:0051046 | regulation of secretion | 0.09375 | <0.00001 |
| GO:0051241 | negative regulation of multicellular organismal process | 0.107582 | <0.00001 |
| GO:0048878 | chemical homeostasis | 0.084615 | <0.00001 |
| GO:0006979 | response to oxidative stress | 0.114558 | <0.00001 |
| GO:0040012 | regulation of locomotion | 0.092455 | <0.00001 |
| GO:0055085 | transmembrane transport | 0.088832 | <0.00001 |
| GO:0031401 | positive regulation of protein modification process | 0.082772 | <0.00001 |
| GO:0042803 | protein homodimerization activity | 0.106833 | <0.00001 |
| GO:0030312 | external encapsulating structure | 0.139004 | <0.00001 |
| GO:0030334 | regulation of cell migration | 0.097156 | <0.00001 |
| GO:0031012 | extracellular matrix | 0.139293 | <0.00001 |
| GO:0032103 | positive regulation of response to external stimulus | 0.106538 | <0.00001 |
| GO:0035634 | response to stilbenoid | 0.380952 | <0.00001 |
| GO:0018279 | protein N-linked glycosylation via asparagine | 0.380952 | <0.00001 |
| GO:1901342 | regulation of vasculature development | 0.116732 | <0.00001 |
| GO:0051260 | protein homooligomerization | 0.105263 | <0.00001 |
| GO:0006638 | neutral lipid metabolic process | 0.171717 | <0.00001 |
| GO:0006631 | fatty acid metabolic process | 0.132626 | <0.00001 |
| GO:0016042 | lipid catabolic process | 0.115512 | <0.00001 |
| GO:0070201 | regulation of establishment of protein localization | 0.094571 | <0.00001 |
| GO:0046395 | carboxylic acid catabolic process | 0.114815 | <0.00001 |
| GO:0040017 | positive regulation of locomotion | 0.101145 | <0.00001 |
| GO:0030424 | axon | 0.097328 | <0.00001 |
| GO:0022804 | active transmembrane transporter activity | 0.110687 | <0.00001 |
| GO:1903530 | regulation of secretion by cell | 0.093851 | <0.00001 |
| GO:0006812 | cation transport | 0.095172 | <0.00001 |
| GO:0016705 | oxidoreductase activity, acting on paired donors, with incorporation or reduction of molecular oxygen | 0.136213 | <0.00001 |
| GO:0045765 | regulation of angiogenesis | 0.117188 | <0.00001 |
| GO:0051259 | protein complex oligomerization | 0.099688 | <0.00001 |
| GO:0003756 | protein disulfide isomerase activity | 0.466667 | <0.00001 |
| GO:0016864 | intramolecular oxidoreductase activity, transposing S-S bonds | 0.466667 | <0.00001 |
| GO:0020037 | heme binding | 0.120567 | <0.00001 |
| GO:0006639 | acylglycerol metabolic process | 0.173469 | <0.00001 |
| GO:0030947 | regulation of vascular endothelial growth factor receptor signaling pathway | 0.333333 | <0.00001 |
| GO:0016829 | lyase activity | 0.113208 | <0.00001 |
| GO:0043197 | dendritic spine | 0.125581 | <0.00001 |
| GO:0042470 | melanosome | 0.159664 | <0.00001 |
| GO:0048770 | pigment granule | 0.159664 | <0.00001 |
| GO:0008610 | lipid biosynthetic process | 0.103448 | <0.00001 |
| GO:0010817 | regulation of hormone levels | 0.097015 | <0.00001 |
| GO:0046906 | tetrapyrrole binding | 0.116041 | <0.00001 |
| GO:0072599 | establishment of protein localization to endoplasmic reticulum | 0.265306 | <0.00001 |
| GO:0010038 | response to metal ion | 0.124378 | <0.00001 |
| GO:0140534 | endoplasmic reticulum protein-containing complex | 0.251534 | <0.00001 |
| GO:0030335 | positive regulation of cell migration | 0.105372 | <0.00001 |
| GO:0034976 | response to endoplasmic reticulum stress | 0.170854 | <0.00001 |
| GO:0016709 | oxidoreductase activity, acting on paired donors, with incorporation or reduction of molecular oxygen, NAD(P)H as one donor, and incorporation of one atom of oxygen | 0.22807 | <0.00001 |
| GO:0043062 | extracellular structure organization | 0.125 | <0.00001 |
| GO:0004497 | monooxygenase activity | 0.160714 | <0.00001 |
| GO:0036503 | ERAD pathway | 0.211765 | <0.00001 |
| GO:0016836 | hydro-lyase activity | 0.2 | <0.00001 |
| GO:0002526 | acute inflammatory response | 0.24 | <0.00001 |
| GO:0015453 | oxidoreduction-driven active transmembrane transporter activity | 0.189655 | <0.00001 |
| GO:0006888 | endoplasmic reticulum to Golgi vesicle-mediated transport | 0.212598 | <0.00001 |
| GO:0045229 | external encapsulating structure organization | 0.125561 | <0.00001 |
| GO:0009055 | electron transfer activity | 0.151351 | <0.00001 |
| GO:0030948 | negative regulation of vascular endothelial growth factor receptor signaling pathway | 0.5 | <0.00001 |
| GO:0009410 | response to xenobiotic stimulus | 0.123839 | <0.00001 |
| GO:0090087 | regulation of peptide transport | 0.126126 | <0.00001 |
| GO:0030198 | extracellular matrix organization | 0.126126 | <0.00001 |
| GO:0048193 | Golgi vesicle transport | 0.135036 | <0.00001 |
| GO:0016712 | oxidoreductase activity, acting on paired donors, with incorporation or reduction of molecular oxygen, reduced flavin or flavoprotein as one donor, and incorporation of one atom of oxygen | 0.165217 | <0.00001 |
| GO:0002791 | regulation of peptide secretion | 0.126697 | <0.00001 |
| GO:0051787 | misfolded protein binding | 0.421053 | <0.00001 |
| GO:0016860 | intramolecular oxidoreductase activity | 0.204819 | <0.00001 |
| GO:0044309 | neuron spine | 0.127273 | <0.00001 |
| GO:0045444 | fat cell differentiation | 0.169355 | <0.00001 |
| GO:0051592 | response to calcium ion | 0.14557 | <0.00001 |
| GO:0005788 | endoplasmic reticulum lumen | 0.19863 | <0.00001 |
| GO:0006487 | protein N-linked glycosylation | 0.238095 | <0.00001 |
| GO:0008202 | steroid metabolic process | 0.136719 | <0.00001 |
| GO:0015399 | primary active transmembrane transporter activity | 0.128906 | <0.00001 |
| GO:0035456 | response to interferon-beta | 0.222222 | <0.00001 |
| GO:0006953 | acute-phase response | 0.394737 | <0.00001 |
| GO:0071248 | cellular response to metal ion | 0.137255 | <0.00001 |
| GO:0070972 | protein localization to endoplasmic reticulum | 0.225806 | <0.00001 |
| GO:0042730 | fibrinolysis | 0.538462 | <0.00001 |
| GO:0030968 | endoplasmic reticulum unfolded protein response | 0.288889 | <0.00001 |
| GO:0045047 | protein targeting to ER | 0.288889 | <0.00001 |
| GO:0008391 | arachidonic acid monooxygenase activity | 0.266667 | <0.00001 |
| GO:0006641 | triglyceride metabolic process | 0.228571 | <0.00001 |
| GO:0030433 | ubiquitin-dependent ERAD pathway | 0.214286 | <0.00001 |
| GO:0016835 | carbon-oxygen lyase activity | 0.175 | <0.00001 |
| GO:0006805 | xenobiotic metabolic process | 0.20915 | <0.00001 |
| GO:0035458 | cellular response to interferon-beta | 0.25 | <0.00001 |
| GO:0042178 | xenobiotic catabolic process | 0.24359 | <0.00001 |
| GO:0034663 | endoplasmic reticulum chaperone complex | 0.529412 | <0.00001 |
| GO:0035966 | response to topologically incorrect protein | 0.237288 | <0.00001 |
| GO:0006986 | response to unfolded protein | 0.26 | <0.00001 |
| GO:0005790 | smooth endoplasmic reticulum | 0.357143 | <0.00001 |
| GO:0004579 | dolichyl-diphosphooligosaccharide-protein glycotransferase activity | 1 | <0.00001 |
| GO:0004576 | oligosaccharyl transferase activity | 1 | <0.00001 |

Rich factor: The ratio of the number of genes belonging to this Term in the target gene set to the number of all genes belonging to this Term in the background gene set.
