# Supplementary material for: Multi-time point transcriptomics and metabolomics reveal key transcription and metabolic features of hepatic ischemia-reperfusion injury in mice
Source: Genes Dis. 2024 Nov 17;12(2):101465. doi: 10.1016/j.gendis.2024.101465 (PMC11697123; doi:10.1016/j.gendis.2024.101465)
Supplement: Multimedia component 5 [file mmc5.docx]

**Table S2B.** The GO terms of differentially expressed genes (DEGs) identified by Gene Ontology (GO) in the Sham and I1R24 groups.

| **GO ID** | **GO term description** | **Rich factor** | **P-value** |
| --- | --- | --- | --- |
| GO:1902105 | regulation of leukocyte differentiation | 0.073944 | 0.04987 |
| GO:0005262 | calcium channel activity | 0.090909 | 0.04984 |
| GO:0043470 | regulation of carbohydrate catabolic process | 0.119048 | 0.04980 |
| GO:0006687 | glycosphingolipid metabolic process | 0.119048 | 0.04980 |
| GO:1905167 | positive regulation of lysosomal protein catabolic process | 0.119048 | 0.04980 |
| GO:0048844 | artery morphogenesis | 0.119048 | 0.04980 |
| GO:0019840 | isoprenoid binding | 0.119048 | 0.04980 |
| GO:0002366 | leukocyte activation involved in immune response | 0.080925 | 0.04950 |
| GO:0010811 | positive regulation of cell-substrate adhesion | 0.091667 | 0.04900 |
| GO:0032680 | regulation of tumor necrosis factor production | 0.084507 | 0.04889 |
| GO:0030239 | myofibril assembly | 0.137931 | 0.04854 |
| GO:0030149 | sphingolipid catabolic process | 0.137931 | 0.04854 |
| GO:0045663 | positive regulation of myoblast differentiation | 0.137931 | 0.04854 |
| GO:0090049 | regulation of cell migration involved in sprouting angiogenesis | 0.137931 | 0.04854 |
| GO:0051154 | negative regulation of striated muscle cell differentiation | 0.137931 | 0.04854 |
| GO:0060079 | excitatory postsynaptic potential | 0.137931 | 0.04854 |
| GO:0043491 | protein kinase B signaling | 0.137931 | 0.04854 |
| GO:0031430 | M band | 0.137931 | 0.04854 |
| GO:0005775 | vacuolar lumen | 0.137931 | 0.04854 |
| GO:0016896 | exoribonuclease activity, producing 5'-phosphomonoesters | 0.137931 | 0.04854 |
| GO:0019825 | oxygen binding | 0.137931 | 0.04854 |
| GO:0099186 | structural constituent of postsynapse | 0.137931 | 0.04854 |
| GO:0030099 | myeloid cell differentiation | 0.081395 | 0.04826 |
| GO:1900166 | regulation of glial cell-derived neurotrophic factor production | 1 | 0.04807 |
| GO:0048389 | intermediate mesoderm development | 1 | 0.04807 |
| GO:1900168 | positive regulation of glial cell-derived neurotrophic factor production | 1 | 0.04807 |
| GO:0033242 | negative regulation of cellular amine catabolic process | 1 | 0.04807 |
| GO:0033241 | regulation of cellular amine catabolic process | 1 | 0.04807 |
| GO:1902910 | positive regulation of melanosome transport | 1 | 0.04807 |
| GO:0039656 | modulation by virus of host gene expression | 1 | 0.04807 |
| GO:0019428 | allantoin biosynthetic process | 1 | 0.04807 |
| GO:0019429 | fluorene catabolic process | 1 | 0.04807 |
| GO:0060901 | regulation of hair cycle by canonical Wnt signaling pathway | 1 | 0.04807 |
| GO:0071505 | response to mycophenolic acid | 1 | 0.04807 |
| GO:0003193 | pulmonary valve formation | 1 | 0.04807 |
| GO:0120078 | cell adhesion involved in sprouting angiogenesis | 1 | 0.04807 |
| GO:2001031 | positive regulation of cellular glucuronidation | 1 | 0.04807 |
| GO:0007403 | glial cell fate determination | 1 | 0.04807 |
| GO:0019402 | galactitol metabolic process | 1 | 0.04807 |
| GO:0015774 | polysaccharide transport | 1 | 0.04807 |
| GO:0021644 | vagus nerve morphogenesis | 1 | 0.04807 |
| GO:0072194 | kidney smooth muscle tissue development | 1 | 0.04807 |
| GO:0046074 | dTMP catabolic process | 1 | 0.04807 |
| GO:0030103 | vasopressin secretion | 1 | 0.04807 |
| GO:1902078 | positive regulation of lateral motor column neuron migration | 1 | 0.04807 |
| GO:1902076 | regulation of lateral motor column neuron migration | 1 | 0.04807 |
| GO:0021812 | neuronal-glial interaction involved in cerebral cortex radial glia guided migration | 1 | 0.04807 |
| GO:0071802 | negative regulation of podosome assembly | 1 | 0.04807 |
| GO:0072592 | oxygen metabolic process | 1 | 0.04807 |
| GO:0046449 | creatinine metabolic process | 1 | 0.04807 |
| GO:1900082 | negative regulation of arginine catabolic process | 1 | 0.04807 |
| GO:1903280 | negative regulation of calcium:sodium antiporter activity | 1 | 0.04807 |
| GO:0097291 | renal phosphate ion absorption | 1 | 0.04807 |
| GO:0060490 | lateral sprouting involved in lung morphogenesis | 1 | 0.04807 |
| GO:0003226 | right ventricular compact myocardium morphogenesis | 1 | 0.04807 |
| GO:0001572 | lactosylceramide biosynthetic process | 1 | 0.04807 |
| GO:0001905 | activation of membrane attack complex | 1 | 0.04807 |
| GO:0051892 | negative regulation of cardioblast differentiation | 1 | 0.04807 |
| GO:2000691 | negative regulation of cardiac muscle cell myoblast differentiation | 1 | 0.04807 |
| GO:2000524 | negative regulation of T cell costimulation | 1 | 0.04807 |
| GO:1901076 | positive regulation of engulfment of apoptotic cell | 1 | 0.04807 |
| GO:1903926 | cellular response to bisphenol A | 1 | 0.04807 |
| GO:1903925 | response to bisphenol A | 1 | 0.04807 |
| GO:0001980 | regulation of systemic arterial blood pressure by ischemic conditions | 1 | 0.04807 |
| GO:0019056 | modulation by virus of host transcription | 1 | 0.04807 |
| GO:0003175 | tricuspid valve development | 1 | 0.04807 |
| GO:1904034 | positive regulation of t-SNARE clustering | 1 | 0.04807 |
| GO:1903249 | negative regulation of citrulline biosynthetic process | 1 | 0.04807 |
| GO:1903248 | regulation of citrulline biosynthetic process | 1 | 0.04807 |
| GO:0009183 | purine deoxyribonucleoside diphosphate biosynthetic process | 1 | 0.04807 |
| GO:0099564 | modification of synaptic structure, modulating synaptic transmission | 1 | 0.04807 |
| GO:0072183 | negative regulation of nephron tubule epithelial cell differentiation | 1 | 0.04807 |
| GO:0072184 | renal vesicle progenitor cell differentiation | 1 | 0.04807 |
| GO:0038096 | Fc-gamma receptor signaling pathway involved in phagocytosis | 1 | 0.04807 |
| GO:1903906 | regulation of plasma membrane raft polarization | 1 | 0.04807 |
| GO:0072377 | blood coagulation, common pathway | 1 | 0.04807 |
| GO:0021511 | spinal cord patterning | 1 | 0.04807 |
| GO:0036076 | ligamentous ossification | 1 | 0.04807 |
| GO:0071247 | cellular response to chromate | 1 | 0.04807 |
| GO:1905438 | non-canonical Wnt signaling pathway involved in midbrain dopaminergic neuron differentiation | 1 | 0.04807 |
| GO:1904464 | regulation of matrix metallopeptidase secretion | 1 | 0.04807 |
| GO:1904465 | negative regulation of matrix metallopeptidase secretion | 1 | 0.04807 |
| GO:1905169 | regulation of protein localization to phagocytic vesicle | 1 | 0.04807 |
| GO:0072239 | metanephric glomerulus vasculature development | 1 | 0.04807 |
| GO:0052213 | obsolete interaction with symbiont via secreted substance | 1 | 0.04807 |
| GO:0031275 | obsolete regulation of lateral pseudopodium assembly | 1 | 0.04807 |
| GO:1905787 | regulation of detection of mechanical stimulus involved in sensory perception of touch | 1 | 0.04807 |
| GO:1905788 | negative regulation of detection of mechanical stimulus involved in sensory perception of touch | 1 | 0.04807 |
| GO:0032771 | regulation of tyrosinase activity | 1 | 0.04807 |
| GO:0032773 | positive regulation of tyrosinase activity | 1 | 0.04807 |
| GO:1901202 | negative regulation of extracellular matrix assembly | 1 | 0.04807 |
| GO:0106088 | regulation of cell adhesion involved in sprouting angiogenesis | 1 | 0.04807 |
| GO:0098923 | retrograde trans-synaptic signaling by soluble gas | 1 | 0.04807 |
| GO:0098924 | retrograde trans-synaptic signaling by nitric oxide | 1 | 0.04807 |
| GO:0098925 | retrograde trans-synaptic signaling by nitric oxide, modulating synaptic transmission | 1 | 0.04807 |
| GO:1901250 | negative regulation of lung goblet cell differentiation | 1 | 0.04807 |
| GO:0010585 | glutamine secretion | 1 | 0.04807 |
| GO:2000761 | positive regulation of N-terminal peptidyl-lysine acetylation | 1 | 0.04807 |
| GO:0072254 | metanephric glomerular mesangial cell differentiation | 1 | 0.04807 |
| GO:0072259 | metanephric interstitial fibroblast development | 1 | 0.04807 |
| GO:0039019 | pronephric nephron development | 1 | 0.04807 |
| GO:1901229 | regulation of non-canonical Wnt signaling pathway via JNK cascade | 1 | 0.04807 |
| GO:0046711 | GDP biosynthetic process | 1 | 0.04807 |
| GO:1905483 | regulation of motor neuron migration | 1 | 0.04807 |
| GO:1902866 | regulation of retina development in camera-type eye | 1 | 0.04807 |
| GO:1904425 | negative regulation of GTP binding | 1 | 0.04807 |
| GO:0072277 | metanephric glomerular capillary formation | 1 | 0.04807 |
| GO:0002034 | maintenance of blood vessel diameter homeostasis by renin-angiotensin | 1 | 0.04807 |
| GO:0002032 | desensitization of G protein-coupled receptor signaling pathway by arrestin | 1 | 0.04807 |
| GO:1905469 | negative regulation of clathrin-coated pit assembly | 1 | 0.04807 |
| GO:1905468 | regulation of clathrin-coated pit assembly | 1 | 0.04807 |
| GO:0036371 | protein localization to T-tubule | 1 | 0.04807 |
| GO:0097510 | base-excision repair, AP site formation via deaminated base removal | 1 | 0.04807 |
| GO:0090094 | metanephric cap mesenchymal cell proliferation involved in metanephros development | 1 | 0.04807 |
| GO:1903457 | lactate catabolic process | 1 | 0.04807 |
| GO:1905145 | cellular response to acetylcholine | 1 | 0.04807 |
| GO:0097188 | dentin mineralization | 1 | 0.04807 |
| GO:1903048 | regulation of acetylcholine-gated cation channel activity | 1 | 0.04807 |
| GO:2000690 | regulation of cardiac muscle cell myoblast differentiation | 1 | 0.04807 |
| GO:1900275 | negative regulation of phospholipase C activity | 1 | 0.04807 |
| GO:1900279 | regulation of CD4-positive, alpha-beta T cell costimulation | 1 | 0.04807 |
| GO:0061837 | neuropeptide processing | 1 | 0.04807 |
| GO:1905162 | regulation of phagosome maturation | 1 | 0.04807 |
| GO:1904613 | cellular response to 2,3,7,8-tetrachlorodibenzodioxine | 1 | 0.04807 |
| GO:0007146 | meiotic recombination nodule assembly | 1 | 0.04807 |
| GO:0021730 | trigeminal sensory nucleus development | 1 | 0.04807 |
| GO:1900276 | regulation of proteinase activated receptor activity | 1 | 0.04807 |
| GO:0061227 | pattern specification involved in mesonephros development | 1 | 0.04807 |
| GO:0060577 | pulmonary vein morphogenesis | 1 | 0.04807 |
| GO:1904808 | positive regulation of protein oxidation | 1 | 0.04807 |
| GO:1904806 | regulation of protein oxidation | 1 | 0.04807 |
| GO:0099158 | regulation of recycling endosome localization within postsynapse | 1 | 0.04807 |
| GO:0070487 | monocyte aggregation | 1 | 0.04807 |
| GO:0035922 | foramen ovale closure | 1 | 0.04807 |
| GO:1903803 | L-glutamine import across plasma membrane | 1 | 0.04807 |
| GO:0060217 | hemangioblast cell differentiation | 1 | 0.04807 |
| GO:0072167 | specification of mesonephric tubule identity | 1 | 0.04807 |
| GO:0072168 | specification of anterior mesonephric tubule identity | 1 | 0.04807 |
| GO:0097324 | melanocyte migration | 1 | 0.04807 |
| GO:0106089 | negative regulation of cell adhesion involved in sprouting angiogenesis | 1 | 0.04807 |
| GO:1990384 | hyaloid vascular plexus regression | 1 | 0.04807 |
| GO:0051612 | negative regulation of serotonin uptake | 1 | 0.04807 |
| GO:1902748 | positive regulation of lens fiber cell differentiation | 1 | 0.04807 |
| GO:0051581 | negative regulation of neurotransmitter uptake | 1 | 0.04807 |
| GO:0000707 | meiotic DNA recombinase assembly | 1 | 0.04807 |
| GO:1905025 | negative regulation of membrane repolarization during ventricular cardiac muscle cell action potential | 1 | 0.04807 |
| GO:0021553 | olfactory nerve development | 1 | 0.04807 |
| GO:0046186 | acetaldehyde biosynthetic process | 1 | 0.04807 |
| GO:0098759 | cellular response to interleukin-8 | 1 | 0.04807 |
| GO:0098758 | response to interleukin-8 | 1 | 0.04807 |
| GO:0014876 | response to injury involved in regulation of muscle adaptation | 1 | 0.04807 |
| GO:2000283 | negative regulation of cellular amino acid biosynthetic process | 1 | 0.04807 |
| GO:0051796 | negative regulation of timing of catagen | 1 | 0.04807 |
| GO:0007630 | jump response | 1 | 0.04807 |
| GO:0002280 | monocyte activation involved in immune response | 1 | 0.04807 |
| GO:0006948 | induction by virus of host cell-cell fusion | 1 | 0.04807 |
| GO:2000349 | negative regulation of CD40 signaling pathway | 1 | 0.04807 |
| GO:1903118 | urate homeostasis | 1 | 0.04807 |
| GO:0035397 | helper T cell enhancement of adaptive immune response | 1 | 0.04807 |
| GO:0098736 | negative regulation of the force of heart contraction | 1 | 0.04807 |
| GO:0003271 | smoothened signaling pathway involved in regulation of secondary heart field cardioblast proliferation | 1 | 0.04807 |
| GO:0006580 | ethanolamine metabolic process | 1 | 0.04807 |
| GO:1990227 | paranodal junction maintenance | 1 | 0.04807 |
| GO:1901810 | beta-carotene metabolic process | 1 | 0.04807 |
| GO:0060601 | lateral sprouting from an epithelium | 1 | 0.04807 |
| GO:0072008 | glomerular mesangial cell differentiation | 1 | 0.04807 |
| GO:0072003 | kidney rudiment formation | 1 | 0.04807 |
| GO:0072007 | mesangial cell differentiation | 1 | 0.04807 |
| GO:1900081 | regulation of arginine catabolic process | 1 | 0.04807 |
| GO:0019341 | dibenzo-p-dioxin catabolic process | 1 | 0.04807 |
| GO:2000795 | negative regulation of epithelial cell proliferation involved in lung morphogenesis | 1 | 0.04807 |
| GO:1905590 | fibronectin fibril organization | 1 | 0.04807 |
| GO:0061141 | lung ciliated cell differentiation | 1 | 0.04807 |
| GO:0090410 | malonate catabolic process | 1 | 0.04807 |
| GO:0001970 | positive regulation of activation of membrane attack complex | 1 | 0.04807 |
| GO:0021793 | chemorepulsion of branchiomotor axon | 1 | 0.04807 |
| GO:0097477 | lateral motor column neuron migration | 1 | 0.04807 |
| GO:0014736 | negative regulation of muscle atrophy | 1 | 0.04807 |
| GO:0046478 | lactosylceramide metabolic process | 1 | 0.04807 |
| GO:1903072 | regulation of death-inducing signaling complex assembly | 1 | 0.04807 |
| GO:1903073 | negative regulation of death-inducing signaling complex assembly | 1 | 0.04807 |
| GO:0090251 | protein localization involved in establishment of planar polarity | 1 | 0.04807 |
| GO:0060488 | orthogonal dichotomous subdivision of terminal units involved in lung branching morphogenesis | 1 | 0.04807 |
| GO:0060489 | planar dichotomous subdivision of terminal units involved in lung branching morphogenesis | 1 | 0.04807 |
| GO:2000844 | negative regulation of testosterone secretion | 1 | 0.04807 |
| GO:1900402 | obsolete regulation of carbohydrate metabolic process by regulation of transcription from RNA polymerase II promoter | 1 | 0.04807 |
| GO:0019605 | butyrate metabolic process | 1 | 0.04807 |
| GO:0021694 | cerebellar Purkinje cell layer formation | 1 | 0.04807 |
| GO:0051838 | cytolysis by host of symbiont cells | 1 | 0.04807 |
| GO:1990401 | embryonic lung development | 1 | 0.04807 |
| GO:0072046 | establishment of planar polarity involved in nephron morphogenesis | 1 | 0.04807 |
| GO:0048749 | compound eye development | 1 | 0.04807 |
| GO:0003025 | regulation of systemic arterial blood pressure by baroreceptor feedback | 1 | 0.04807 |
| GO:0033488 | cholesterol biosynthetic process via 24,25-dihydrolanosterol | 1 | 0.04807 |
| GO:1900280 | negative regulation of CD4-positive, alpha-beta T cell costimulation | 1 | 0.04807 |
| GO:0003142 | cardiogenic plate morphogenesis | 1 | 0.04807 |
| GO:1905485 | positive regulation of motor neuron migration | 1 | 0.04807 |
| GO:1903621 | protein localization to photoreceptor connecting cilium | 1 | 0.04807 |
| GO:0021636 | trigeminal nerve morphogenesis | 1 | 0.04807 |
| GO:0061373 | mammillary axonal complex development | 1 | 0.04807 |
| GO:0009740 | gibberellic acid mediated signaling pathway | 1 | 0.04807 |
| GO:0005993 | trehalose catabolic process | 1 | 0.04807 |
| GO:0072190 | ureter urothelium development | 1 | 0.04807 |
| GO:0002362 | CD4-positive, CD25-positive, alpha-beta regulatory T cell lineage commitment | 1 | 0.04807 |
| GO:0072209 | metanephric mesangial cell differentiation | 1 | 0.04807 |
| GO:0072208 | metanephric smooth muscle tissue development | 1 | 0.04807 |
| GO:1900039 | positive regulation of cellular response to hypoxia | 1 | 0.04807 |
| GO:1900035 | negative regulation of cellular response to heat | 1 | 0.04807 |
| GO:0003072 | renal control of peripheral vascular resistance involved in regulation of systemic arterial blood pressure | 1 | 0.04807 |
| GO:0010813 | neuropeptide catabolic process | 1 | 0.04807 |
| GO:1904681 | response to 3-methylcholanthrene | 1 | 0.04807 |
| GO:1990983 | tRNA demethylation | 1 | 0.04807 |
| GO:0071449 | cellular response to lipid hydroperoxide | 1 | 0.04807 |
| GO:0043041 | amino acid activation for nonribosomal peptide biosynthetic process | 1 | 0.04807 |
| GO:0033484 | nitric oxide homeostasis | 1 | 0.04807 |
| GO:1901231 | positive regulation of non-canonical Wnt signaling pathway via JNK cascade | 1 | 0.04807 |
| GO:1901232 | regulation of convergent extension involved in axis elongation | 1 | 0.04807 |
| GO:1901233 | negative regulation of convergent extension involved in axis elongation | 1 | 0.04807 |
| GO:1904049 | negative regulation of spontaneous neurotransmitter secretion | 1 | 0.04807 |
| GO:0046724 | oxalic acid secretion | 1 | 0.04807 |
| GO:0007576 | obsolete nucleolar fragmentation | 1 | 0.04807 |
| GO:1905673 | positive regulation of lysosome organization | 1 | 0.04807 |
| GO:2000759 | regulation of N-terminal peptidyl-lysine acetylation | 1 | 0.04807 |
| GO:0072301 | regulation of metanephric glomerular mesangial cell proliferation | 1 | 0.04807 |
| GO:0072303 | positive regulation of glomerular metanephric mesangial cell proliferation | 1 | 0.04807 |
| GO:0097017 | renal protein absorption | 1 | 0.04807 |
| GO:1905802 | regulation of cellular response to manganese ion | 1 | 0.04807 |
| GO:0072166 | posterior mesonephric tubule development | 1 | 0.04807 |
| GO:0072169 | specification of posterior mesonephric tubule identity | 1 | 0.04807 |
| GO:0042140 | late meiotic recombination nodule assembly | 1 | 0.04807 |
| GO:0035709 | memory T cell activation | 1 | 0.04807 |
| GO:1904205 | negative regulation of skeletal muscle hypertrophy | 1 | 0.04807 |
| GO:0040040 | thermosensory behavior | 1 | 0.04807 |
| GO:0060807 | obsolete regulation of transcription from RNA polymerase II promoter involved in definitive endodermal cell fate specification | 1 | 0.04807 |
| GO:0052182 | obsolete modification by host of symbiont morphology or physiology via secreted substance | 1 | 0.04807 |
| GO:0072110 | glomerular mesangial cell proliferation | 1 | 0.04807 |
| GO:0071976 | cell gliding | 1 | 0.04807 |
| GO:0071971 | extracellular exosome assembly | 1 | 0.04807 |
| GO:1900737 | negative regulation of phospholipase C-activating G protein-coupled receptor signaling pathway | 1 | 0.04807 |
| GO:0070377 | negative regulation of ERK5 cascade | 1 | 0.04807 |
| GO:1900243 | negative regulation of synaptic vesicle endocytosis | 1 | 0.04807 |
| GO:0072141 | renal interstitial fibroblast development | 1 | 0.04807 |
| GO:0010476 | gibberellin mediated signaling pathway | 1 | 0.04807 |
| GO:0052163 | symbiont defense to host-produced nitric oxide | 1 | 0.04807 |
| GO:0072133 | metanephric mesenchyme morphogenesis | 1 | 0.04807 |
| GO:0072131 | kidney mesenchyme morphogenesis | 1 | 0.04807 |
| GO:0010272 | response to silver ion | 1 | 0.04807 |
| GO:0036324 | vascular endothelial growth factor receptor-2 signaling pathway | 1 | 0.04807 |
| GO:2000043 | regulation of cardiac cell fate specification | 1 | 0.04807 |
| GO:2000282 | regulation of cellular amino acid biosynthetic process | 1 | 0.04807 |
| GO:0035964 | COPI-coated vesicle budding | 1 | 0.04807 |
| GO:0072098 | anterior/posterior pattern specification involved in kidney development | 1 | 0.04807 |
| GO:1905174 | regulation of vascular associated smooth muscle cell dedifferentiation | 1 | 0.04807 |
| GO:1905171 | positive regulation of protein localization to phagocytic vesicle | 1 | 0.04807 |
| GO:2000984 | negative regulation of ATP citrate synthase activity | 1 | 0.04807 |
| GO:2000983 | regulation of ATP citrate synthase activity | 1 | 0.04807 |
| GO:0050894 | determination of affect | 1 | 0.04807 |
| GO:0050893 | sensory processing | 1 | 0.04807 |
| GO:0036306 | embryonic heart tube elongation | 1 | 0.04807 |
| GO:0033955 | obsolete mitochondrial DNA inheritance | 1 | 0.04807 |
| GO:0002433 | immune response-regulating cell surface receptor signaling pathway involved in phagocytosis | 1 | 0.04807 |
| GO:0097497 | blood vessel endothelial cell delamination | 1 | 0.04807 |
| GO:2000439 | positive regulation of monocyte extravasation | 1 | 0.04807 |
| GO:0052026 | modulation by symbiont of host transcription | 1 | 0.04807 |
| GO:1903165 | response to polycyclic arene | 1 | 0.04807 |
| GO:1905032 | negative regulation of membrane repolarization during cardiac muscle cell action potential | 1 | 0.04807 |
| GO:1901249 | regulation of lung goblet cell differentiation | 1 | 0.04807 |
| GO:0034021 | response to silicon dioxide | 1 | 0.04807 |
| GO:0019310 | inositol catabolic process | 1 | 0.04807 |
| GO:0071506 | cellular response to mycophenolic acid | 1 | 0.04807 |
| GO:0035971 | peptidyl-histidine dephosphorylation | 1 | 0.04807 |
| GO:0052047 | obsolete symbiotic process mediated by secreted substance | 1 | 0.04807 |
| GO:0120117 | T cell meandering migration | 1 | 0.04807 |
| GO:1904032 | regulation of t-SNARE clustering | 1 | 0.04807 |
| GO:0006185 | dGDP biosynthetic process | 1 | 0.04807 |
| GO:0021564 | vagus nerve development | 1 | 0.04807 |
| GO:0021563 | glossopharyngeal nerve development | 1 | 0.04807 |
| GO:0021740 | principal sensory nucleus of trigeminal nerve development | 1 | 0.04807 |
| GO:0046352 | disaccharide catabolic process | 1 | 0.04807 |
| GO:0046359 | butyrate catabolic process | 1 | 0.04807 |
| GO:0060520 | activation of prostate induction by androgen receptor signaling pathway | 1 | 0.04807 |
| GO:1902994 | regulation of phospholipid efflux | 1 | 0.04807 |
| GO:1902995 | positive regulation of phospholipid efflux | 1 | 0.04807 |
| GO:2000295 | regulation of hydrogen peroxide catabolic process | 1 | 0.04807 |
| GO:2000296 | negative regulation of hydrogen peroxide catabolic process | 1 | 0.04807 |
| GO:0036457 | keratohyalin granule | 1 | 0.04807 |
| GO:0030128 | clathrin coat of endocytic vesicle | 1 | 0.04807 |
| GO:0061474 | phagolysosome membrane | 1 | 0.04807 |
| GO:1990435 | upper tip-link density | 1 | 0.04807 |
| GO:0005586 | collagen type III trimer | 1 | 0.04807 |
| GO:0070369 | beta-catenin-TCF7L2 complex | 1 | 0.04807 |
| GO:0098595 | perivitelline space | 1 | 0.04807 |
| GO:0036053 | glomerular endothelium fenestra | 1 | 0.04807 |
| GO:0033648 | host intracellular membrane-bounded organelle | 1 | 0.04807 |
| GO:0033647 | host intracellular organelle | 1 | 0.04807 |
| GO:0005943 | phosphatidylinositol 3-kinase complex, class IA | 1 | 0.04807 |
| GO:0071752 | secretory dimeric IgA immunoglobulin complex | 1 | 0.04807 |
| GO:0071750 | dimeric IgA immunoglobulin complex | 1 | 0.04807 |
| GO:0071751 | secretory IgA immunoglobulin complex | 1 | 0.04807 |
| GO:0071757 | hexameric IgM immunoglobulin complex | 1 | 0.04807 |
| GO:0032996 | Bcl3-Bcl10 complex | 1 | 0.04807 |
| GO:0000329 | fungal-type vacuole membrane | 1 | 0.04807 |
| GO:0072517 | host cell viral assembly compartment | 1 | 0.04807 |
| GO:0009841 | mitochondrial endopeptidase Clp complex | 1 | 0.04807 |
| GO:0031477 | myosin VII complex | 1 | 0.04807 |
| GO:0099631 | postsynaptic endocytic zone cytoplasmic component | 1 | 0.04807 |
| GO:0043257 | laminin-8 complex | 1 | 0.04807 |
| GO:0097229 | sperm end piece | 1 | 0.04807 |
| GO:1990008 | neurosecretory vesicle | 1 | 0.04807 |
| GO:0033193 | Lsd1/2 complex | 1 | 0.04807 |
| GO:0005607 | laminin-2 complex | 1 | 0.04807 |
| GO:0071748 | monomeric IgA immunoglobulin complex | 1 | 0.04807 |
| GO:0071749 | polymeric IgA immunoglobulin complex | 1 | 0.04807 |
| GO:0071745 | IgA immunoglobulin complex | 1 | 0.04807 |
| GO:0071746 | IgA immunoglobulin complex, circulating | 1 | 0.04807 |
| GO:0061846 | dendritic spine cytoplasm | 1 | 0.04807 |
| GO:0061845 | neuron projection branch point | 1 | 0.04807 |
| GO:0098998 | extrinsic component of postsynaptic early endosome membrane | 1 | 0.04807 |
| GO:1990427 | stereocilia tip-link density | 1 | 0.04807 |
| GO:0070274 | RES complex | 1 | 0.04807 |
| GO:0039713 | viral factory | 1 | 0.04807 |
| GO:0039714 | cytoplasmic viral factory | 1 | 0.04807 |
| GO:0047127 | thiomorpholine-carboxylate dehydrogenase activity | 1 | 0.04807 |
| GO:0051871 | dihydrofolic acid binding | 1 | 0.04807 |
| GO:0000773 | phosphatidyl-N-methylethanolamine N-methyltransferase activity | 1 | 0.04807 |
| GO:0030108 | HLA-A specific activating MHC class I receptor activity | 1 | 0.04807 |
| GO:0090409 | malonyl-CoA synthetase activity | 1 | 0.04807 |
| GO:0042586 | peptide deformylase activity | 1 | 0.04807 |
| GO:0043754 | dihydrolipoyllysine-residue (2-methylpropanoyl)transferase activity | 1 | 0.04807 |
| GO:0004398 | histidine decarboxylase activity | 1 | 0.04807 |
| GO:1904768 | all-trans-retinol binding | 1 | 0.04807 |
| GO:0086057 | voltage-gated calcium channel activity involved in bundle of His cell action potential | 1 | 0.04807 |
| GO:0019912 | cyclin-dependent protein kinase activating kinase activity | 1 | 0.04807 |
| GO:0031714 | C5a anaphylatoxin chemotactic receptor binding | 1 | 0.04807 |
| GO:0031715 | C5L2 anaphylatoxin chemotactic receptor binding | 1 | 0.04807 |
| GO:0080101 | phosphatidyl-N-dimethylethanolamine N-methyltransferase activity | 1 | 0.04807 |
| GO:0042012 | interleukin-16 receptor activity | 1 | 0.04807 |
| GO:0005183 | gonadotropin hormone-releasing hormone activity | 1 | 0.04807 |
| GO:0004760 | serine-pyruvate transaminase activity | 1 | 0.04807 |
| GO:0061751 | neutral sphingomyelin phosphodiesterase activity | 1 | 0.04807 |
| GO:0000246 | delta24(24-1) sterol reductase activity | 1 | 0.04807 |
| GO:0050683 | AF-1 domain binding | 1 | 0.04807 |
| GO:0016515 | interleukin-13 receptor activity | 1 | 0.04807 |
| GO:0010348 | lithium:proton antiporter activity | 1 | 0.04807 |
| GO:0005006 | epidermal growth factor receptor activity | 1 | 0.04807 |
| GO:0018601 | 4-nitrophenol 2-monooxygenase activity | 1 | 0.04807 |
| GO:0061710 | L-threonylcarbamoyladenylate synthase | 1 | 0.04807 |
| GO:0035716 | chemokine (C-C motif) ligand 12 binding | 1 | 0.04807 |
| GO:0004085 | butyryl-CoA dehydrogenase activity | 1 | 0.04807 |
| GO:0050113 | inositol oxygenase activity | 1 | 0.04807 |
| GO:0004506 | squalene monooxygenase activity | 1 | 0.04807 |
| GO:0050459 | ethanolamine-phosphate phospho-lyase activity | 1 | 0.04807 |
| GO:0016711 | flavonoid 3'-monooxygenase activity | 1 | 0.04807 |
| GO:0004873 | asialoglycoprotein receptor activity | 1 | 0.04807 |
| GO:0015362 | high-affinity sodium:dicarboxylate symporter activity | 1 | 0.04807 |
| GO:0005018 | platelet-derived growth factor alpha-receptor activity | 1 | 0.04807 |
| GO:0072571 | mono-ADP-D-ribose binding | 1 | 0.04807 |
| GO:0008682 | 3-demethoxyubiquinol 3-hydroxylase activity | 1 | 0.04807 |
| GO:0030412 | formimidoyltetrahydrofolate cyclodeaminase activity | 1 | 0.04807 |
| GO:0008112 | nicotinamide N-methyltransferase activity | 1 | 0.04807 |
| GO:0005175 | CD27 receptor binding | 1 | 0.04807 |
| GO:0070856 | myosin VI light chain binding | 1 | 0.04807 |
| GO:0036487 | nitric-oxide synthase inhibitor activity | 1 | 0.04807 |
| GO:0005151 | interleukin-1, type II receptor binding | 1 | 0.04807 |
| GO:0008398 | sterol 14-demethylase activity | 1 | 0.04807 |
| GO:0018585 | fluorene oxygenase activity | 1 | 0.04807 |
| GO:0097160 | polychlorinated biphenyl binding | 1 | 0.04807 |
| GO:0008720 | D-lactate dehydrogenase activity | 1 | 0.04807 |
| GO:0031530 | gonadotropin-releasing hormone receptor binding | 1 | 0.04807 |
| GO:0001681 | sialate O-acetylesterase activity | 1 | 0.04807 |
| GO:0050614 | delta24-sterol reductase activity | 1 | 0.04807 |
| GO:0047805 | cytidylate cyclase activity | 1 | 0.04807 |
| GO:0008761 | UDP-N-acetylglucosamine 2-epimerase activity | 1 | 0.04807 |
| GO:0060175 | brain-derived neurotrophic factor receptor activity | 1 | 0.04807 |
| GO:0004751 | ribose-5-phosphate isomerase activity | 1 | 0.04807 |
| GO:0045353 | interleukin-1 type II receptor antagonist activity | 1 | 0.04807 |
| GO:0045352 | interleukin-1 type I receptor antagonist activity | 1 | 0.04807 |
| GO:0047058 | vitamin-K-epoxide reductase (warfarin-insensitive) activity | 1 | 0.04807 |
| GO:0004595 | pantetheine-phosphate adenylyltransferase activity | 1 | 0.04807 |
| GO:1904928 | coreceptor activity involved in canonical Wnt signaling pathway | 1 | 0.04807 |
| GO:0008903 | hydroxypyruvate isomerase activity | 1 | 0.04807 |
| GO:0047012 | sterol-4-alpha-carboxylate 3-dehydrogenase (decarboxylating) activity | 1 | 0.04807 |
| GO:0016784 | 3-mercaptopyruvate sulfurtransferase activity | 1 | 0.04807 |
| GO:0051750 | delta(3,5)-delta(2,4)-dienoyl-CoA isomerase activity | 1 | 0.04807 |
| GO:0016524 | latrotoxin receptor activity | 1 | 0.04807 |
| GO:0103066 | 4alpha-carboxy-4beta-methyl-5alpha-cholesta-8-en-3beta-ol:NAD(P)+ 3-oxidoreductase (decarboxylating) activity | 1 | 0.04807 |
| GO:0103067 | 4alpha-carboxy-5alpha-cholesta-8-en-3beta-ol:NAD(P)+ 3-dehydrogenase (decarboxylating) activity | 1 | 0.04807 |
| GO:0005010 | insulin-like growth factor receptor activity | 1 | 0.04807 |
| GO:0005019 | platelet-derived growth factor beta-receptor activity | 1 | 0.04807 |
| GO:0035538 | carbohydrate response element binding | 1 | 0.04807 |
| GO:0070089 | chloride-activated potassium channel activity | 1 | 0.04807 |
| GO:0051978 | lysophospholipid:sodium symporter activity | 1 | 0.04807 |
| GO:0015091 | ferric iron transmembrane transporter activity | 1 | 0.04807 |
| GO:0047390 | glycerophosphocholine cholinephosphodiesterase activity | 1 | 0.04807 |
| GO:0102522 | tRNA 4-demethylwyosine alpha-amino-alpha-carboxypropyltransferase activity | 1 | 0.04807 |
| GO:0015390 | purine-specific nucleoside:sodium symporter activity | 1 | 0.04807 |
| GO:0050129 | N-formylglutamate deformylase activity | 1 | 0.04807 |
| GO:0001567 | cholesterol 25-hydroxylase activity | 1 | 0.04807 |
| GO:0031405 | lipoic acid binding | 1 | 0.04807 |
| GO:0001792 | polymeric immunoglobulin receptor activity | 1 | 0.04807 |
| GO:0008127 | quercetin 2,3-dioxygenase activity | 1 | 0.04807 |
| GO:0008126 | acetylesterase activity | 1 | 0.04807 |
| GO:0033971 | hydroxyisourate hydrolase activity | 1 | 0.04807 |
| GO:0030409 | glutamate formimidoyltransferase activity | 1 | 0.04807 |
| GO:0042011 | interleukin-16 binding | 1 | 0.04807 |
| GO:0031877 | somatostatin receptor binding | 1 | 0.04807 |
| GO:0004608 | phosphatidylethanolamine N-methyltransferase activity | 1 | 0.04807 |
| GO:0052692 | raffinose alpha-galactosidase activity | 1 | 0.04807 |
| GO:0070042 | rRNA (uridine-N3-)-methyltransferase activity | 1 | 0.04807 |
| GO:0047290 | (alpha-N-acetylneuraminyl-2,3-beta-galactosyl-1,3)-N-acetyl-galactosaminide 6-alpha-sialyltransferase activity | 1 | 0.04807 |
| GO:1903532 | positive regulation of secretion by cell | 0.072327 | 0.04795 |
| GO:0008286 | insulin receptor signaling pathway | 0.109091 | 0.04779 |
| GO:0071900 | regulation of protein serine/threonine kinase activity | 0.075 | 0.04761 |
| GO:0016773 | phosphotransferase activity, alcohol group as acceptor | 0.062048 | 0.04755 |
| GO:1902414 | protein localization to cell junction | 0.101449 | 0.04754 |
| GO:0001917 | photoreceptor inner segment | 0.101449 | 0.04754 |
| GO:1901293 | nucleoside phosphate biosynthetic process | 0.071429 | 0.04744 |
| GO:0007188 | adenylate cyclase-modulating G protein-coupled receptor signaling pathway | 0.078818 | 0.04738 |
| GO:0031334 | positive regulation of protein-containing complex assembly | 0.081871 | 0.04708 |
| GO:1903829 | positive regulation of protein localization | 0.068282 | 0.04701 |
| GO:0051225 | spindle assembly | 0.095745 | 0.04668 |
| GO:0048863 | stem cell differentiation | 0.095745 | 0.04668 |
| GO:0070469 | respirasome | 0.095745 | 0.04668 |
| GO:0006820 | anion transport | 0.072022 | 0.04639 |
| GO:0012506 | vesicle membrane | 0.06443 | 0.04618 |
| GO:1901135 | carbohydrate derivative metabolic process | 0.059603 | 0.04582 |
| GO:0090311 | regulation of protein deacetylation | 0.121951 | 0.04561 |
| GO:0044275 | cellular carbohydrate catabolic process | 0.121951 | 0.04561 |
| GO:0048675 | axon extension | 0.121951 | 0.04561 |
| GO:0030551 | cyclic nucleotide binding | 0.121951 | 0.04561 |
| GO:0005547 | phosphatidylinositol-3,4,5-trisphosphate binding | 0.121951 | 0.04561 |
| GO:0030554 | adenyl nucleotide binding | 0.056627 | 0.04560 |
| GO:0030852 | regulation of granulocyte differentiation | 0.176471 | 0.04553 |
| GO:0002293 | alpha-beta T cell differentiation involved in immune response | 0.176471 | 0.04553 |
| GO:0055003 | cardiac myofibril assembly | 0.176471 | 0.04553 |
| GO:0006978 | DNA damage response, signal transduction by p53 class mediator resulting in transcription of p21 class mediator | 0.176471 | 0.04553 |
| GO:0007340 | acrosome reaction | 0.176471 | 0.04553 |
| GO:1903513 | endoplasmic reticulum to cytosol transport | 0.176471 | 0.04553 |
| GO:0061326 | renal tubule development | 0.176471 | 0.04553 |
| GO:0048643 | positive regulation of skeletal muscle tissue development | 0.176471 | 0.04553 |
| GO:0001778 | plasma membrane repair | 0.176471 | 0.04553 |
| GO:0072359 | circulatory system development | 0.176471 | 0.04553 |
| GO:0001895 | retina homeostasis | 0.176471 | 0.04553 |
| GO:0006825 | copper ion transport | 0.176471 | 0.04553 |
| GO:0034695 | response to prostaglandin E | 0.176471 | 0.04553 |
| GO:0035336 | long-chain fatty-acyl-CoA metabolic process | 0.176471 | 0.04553 |
| GO:0009404 | toxin metabolic process | 0.176471 | 0.04553 |
| GO:0003208 | cardiac ventricle morphogenesis | 0.176471 | 0.04553 |
| GO:0030970 | retrograde protein transport, ER to cytosol | 0.176471 | 0.04553 |
| GO:0042574 | retinal metabolic process | 0.176471 | 0.04553 |
| GO:0034143 | regulation of toll-like receptor 4 signaling pathway | 0.176471 | 0.04553 |
| GO:0010669 | epithelial structure maintenance | 0.176471 | 0.04553 |
| GO:0002367 | cytokine production involved in immune response | 0.176471 | 0.04553 |
| GO:0032332 | positive regulation of chondrocyte differentiation | 0.176471 | 0.04553 |
| GO:1900038 | negative regulation of cellular response to hypoxia | 0.176471 | 0.04553 |
| GO:0010575 | positive regulation of vascular endothelial growth factor production | 0.176471 | 0.04553 |
| GO:0042982 | amyloid precursor protein metabolic process | 0.176471 | 0.04553 |
| GO:0097449 | astrocyte projection | 0.176471 | 0.04553 |
| GO:0005542 | folic acid binding | 0.176471 | 0.04553 |
| GO:0001654 | eye development | 0.09009 | 0.04553 |
| GO:0032412 | regulation of ion transmembrane transporter activity | 0.076923 | 0.04547 |
| GO:0032526 | response to retinoic acid | 0.096774 | 0.04505 |
| GO:0033365 | protein localization to organelle | 0.065379 | 0.04488 |
| GO:0007507 | heart development | 0.077253 | 0.04464 |
| GO:1901214 | regulation of neuron death | 0.073718 | 0.04459 |
| GO:0046887 | positive regulation of hormone secretion | 0.086331 | 0.04456 |
| GO:0018108 | peptidyl-tyrosine phosphorylation | 0.102941 | 0.04448 |
| GO:0019362 | pyridine nucleotide metabolic process | 0.102941 | 0.04448 |
| GO:0044843 | cell cycle G1/S phase transition | 0.102941 | 0.04448 |
| GO:0046496 | nicotinamide nucleotide metabolic process | 0.102941 | 0.04448 |
| GO:0046626 | regulation of insulin receptor signaling pathway | 0.102941 | 0.04448 |
| GO:0015908 | fatty acid transport | 0.102941 | 0.04448 |
| GO:0030301 | cholesterol transport | 0.111111 | 0.04430 |
| GO:0051931 | regulation of sensory perception | 0.111111 | 0.04430 |
| GO:0061245 | establishment or maintenance of bipolar cell polarity | 0.111111 | 0.04430 |
| GO:0050922 | negative regulation of chemotaxis | 0.111111 | 0.04430 |
| GO:0015918 | sterol transport | 0.111111 | 0.04430 |
| GO:0035088 | establishment or maintenance of apical/basal cell polarity | 0.111111 | 0.04430 |
| GO:0062208 | positive regulation of pattern recognition receptor signaling pathway | 0.111111 | 0.04430 |
| GO:0034109 | homotypic cell-cell adhesion | 0.111111 | 0.04430 |
| GO:0042509 | regulation of tyrosine phosphorylation of STAT protein | 0.111111 | 0.04430 |
| GO:0006040 | amino sugar metabolic process | 0.111111 | 0.04430 |
| GO:0015020 | glucuronosyltransferase activity | 0.111111 | 0.04430 |
| GO:0003779 | actin binding | 0.067151 | 0.04407 |
| GO:1903322 | positive regulation of protein modification by small protein conjugation or removal | 0.083333 | 0.04391 |
| GO:0055074 | calcium ion homeostasis | 0.080402 | 0.04377 |
| GO:1901987 | regulation of cell cycle phase transition | 0.069663 | 0.04368 |
| GO:0031032 | actomyosin structure organization | 0.090909 | 0.04364 |
| GO:0055065 | metal ion homeostasis | 0.070707 | 0.04359 |
| GO:0043161 | proteasome-mediated ubiquitin-dependent protein catabolic process | 0.071839 | 0.04347 |
| GO:0043171 | peptide catabolic process | 0.142857 | 0.04344 |
| GO:0008210 | estrogen metabolic process | 0.142857 | 0.04344 |
| GO:0001709 | cell fate determination | 0.142857 | 0.04344 |
| GO:0035640 | exploration behavior | 0.142857 | 0.04344 |
| GO:0033198 | response to ATP | 0.142857 | 0.04344 |
| GO:0002026 | regulation of the force of heart contraction | 0.142857 | 0.04344 |
| GO:0008535 | respiratory chain complex IV assembly | 0.142857 | 0.04344 |
| GO:0000175 | 3'-5'-exoribonuclease activity | 0.142857 | 0.04344 |
| GO:0016641 | oxidoreductase activity, acting on the CH-NH2 group of donors, oxygen as acceptor | 0.142857 | 0.04344 |
| GO:0009749 | response to glucose | 0.086957 | 0.04326 |
| GO:2001236 | regulation of extrinsic apoptotic signaling pathway | 0.086957 | 0.04326 |
| GO:0016247 | channel regulator activity | 0.086957 | 0.04326 |
| GO:0015085 | calcium ion transmembrane transporter activity | 0.086957 | 0.04326 |
| GO:0051494 | negative regulation of cytoskeleton organization | 0.083832 | 0.04298 |
| GO:0009266 | response to temperature stimulus | 0.081218 | 0.04228 |
| GO:1902882 | regulation of response to oxidative stress | 0.098901 | 0.04214 |
| GO:0005881 | cytoplasmic microtubule | 0.098901 | 0.04214 |
| GO:0031227 | intrinsic component of endoplasmic reticulum membrane | 0.087591 | 0.04203 |
| GO:0016298 | lipase activity | 0.087591 | 0.04203 |
| GO:0061134 | peptidase regulator activity | 0.067164 | 0.04193 |
| GO:0071692 | protein localization to extracellular region | 0.091743 | 0.04183 |
| GO:0035592 | establishment of protein localization to extracellular region | 0.091743 | 0.04183 |
| GO:0098889 | intrinsic component of presynaptic membrane | 0.091743 | 0.04183 |
| GO:0042531 | positive regulation of tyrosine phosphorylation of STAT protein | 0.125 | 0.04163 |
| GO:0060706 | cell differentiation involved in embryonic placenta development | 0.125 | 0.04163 |
| GO:0046596 | regulation of viral entry into host cell | 0.125 | 0.04163 |
| GO:1901021 | positive regulation of calcium ion transmembrane transporter activity | 0.125 | 0.04163 |
| GO:0030890 | positive regulation of B cell proliferation | 0.125 | 0.04163 |
| GO:0051495 | positive regulation of cytoskeleton organization | 0.081633 | 0.04161 |
| GO:0016197 | endosomal transport | 0.076046 | 0.04161 |
| GO:1900542 | regulation of purine nucleotide metabolic process | 0.104478 | 0.04156 |
| GO:0000082 | G1/S transition of mitotic cell cycle | 0.104478 | 0.04156 |
| GO:0005518 | collagen binding | 0.104478 | 0.04156 |
| GO:0060264 | regulation of respiratory burst involved in inflammatory response | 0.285714 | 0.04127 |
| GO:0060267 | positive regulation of respiratory burst | 0.285714 | 0.04127 |
| GO:2000811 | negative regulation of anoikis | 0.285714 | 0.04127 |
| GO:0003229 | ventricular cardiac muscle tissue development | 0.285714 | 0.04127 |
| GO:0034115 | negative regulation of heterotypic cell-cell adhesion | 0.285714 | 0.04127 |
| GO:0042659 | regulation of cell fate specification | 0.285714 | 0.04127 |
| GO:0072180 | mesonephric duct morphogenesis | 0.285714 | 0.04127 |
| GO:0070587 | regulation of cell-cell adhesion involved in gastrulation | 0.285714 | 0.04127 |
| GO:0034638 | phosphatidylcholine catabolic process | 0.285714 | 0.04127 |
| GO:0035743 | CD4-positive, alpha-beta T cell cytokine production | 0.285714 | 0.04127 |
| GO:0003096 | renal sodium ion transport | 0.285714 | 0.04127 |
| GO:0010616 | negative regulation of cardiac muscle adaptation | 0.285714 | 0.04127 |
| GO:0071694 | maintenance of protein location in extracellular region | 0.285714 | 0.04127 |
| GO:1900747 | negative regulation of vascular endothelial growth factor signaling pathway | 0.285714 | 0.04127 |
| GO:2000480 | negative regulation of cAMP-dependent protein kinase activity | 0.285714 | 0.04127 |
| GO:0048668 | collateral sprouting | 0.285714 | 0.04127 |
| GO:0043569 | negative regulation of insulin-like growth factor receptor signaling pathway | 0.285714 | 0.04127 |
| GO:0070995 | NADPH oxidation | 0.285714 | 0.04127 |
| GO:0030241 | skeletal muscle myosin thick filament assembly | 0.285714 | 0.04127 |
| GO:0072124 | regulation of glomerular mesangial cell proliferation | 0.285714 | 0.04127 |
| GO:0072126 | positive regulation of glomerular mesangial cell proliferation | 0.285714 | 0.04127 |
| GO:0051823 | regulation of synapse structural plasticity | 0.285714 | 0.04127 |
| GO:0030949 | positive regulation of vascular endothelial growth factor receptor signaling pathway | 0.285714 | 0.04127 |
| GO:1902947 | regulation of tau-protein kinase activity | 0.285714 | 0.04127 |
| GO:2000821 | regulation of grooming behavior | 0.285714 | 0.04127 |
| GO:0072531 | pyrimidine-containing compound transmembrane transport | 0.285714 | 0.04127 |
| GO:0048023 | positive regulation of melanin biosynthetic process | 0.285714 | 0.04127 |
| GO:0019368 | fatty acid elongation, unsaturated fatty acid | 0.285714 | 0.04127 |
| GO:1900378 | positive regulation of secondary metabolite biosynthetic process | 0.285714 | 0.04127 |
| GO:0021517 | ventral spinal cord development | 0.285714 | 0.04127 |
| GO:1905274 | regulation of modification of postsynaptic actin cytoskeleton | 0.285714 | 0.04127 |
| GO:0019695 | choline metabolic process | 0.285714 | 0.04127 |
| GO:0071688 | striated muscle myosin thick filament assembly | 0.285714 | 0.04127 |
| GO:0001955 | blood vessel maturation | 0.285714 | 0.04127 |
| GO:0006521 | regulation of cellular amino acid metabolic process | 0.285714 | 0.04127 |
| GO:0071447 | cellular response to hydroperoxide | 0.285714 | 0.04127 |
| GO:0030643 | cellular phosphate ion homeostasis | 0.285714 | 0.04127 |
| GO:0034625 | fatty acid elongation, monounsaturated fatty acid | 0.285714 | 0.04127 |
| GO:0034626 | fatty acid elongation, polyunsaturated fatty acid | 0.285714 | 0.04127 |
| GO:0014012 | peripheral nervous system axon regeneration | 0.285714 | 0.04127 |
| GO:2000002 | negative regulation of DNA damage checkpoint | 0.285714 | 0.04127 |
| GO:1900221 | regulation of amyloid-beta clearance | 0.285714 | 0.04127 |
| GO:0018879 | biphenyl metabolic process | 0.285714 | 0.04127 |
| GO:0072502 | cellular trivalent inorganic anion homeostasis | 0.285714 | 0.04127 |
| GO:0097688 | glutamate receptor clustering | 0.285714 | 0.04127 |
| GO:0048699 | generation of neurons | 0.285714 | 0.04127 |
| GO:0097113 | AMPA glutamate receptor clustering | 0.285714 | 0.04127 |
| GO:0031232 | extrinsic component of external side of plasma membrane | 0.285714 | 0.04127 |
| GO:0060091 | kinocilium | 0.285714 | 0.04127 |
| GO:0097732 | 9+2 non-motile cilium | 0.285714 | 0.04127 |
| GO:0102337 | 3-oxo-cerotoyl-CoA synthase activity | 0.285714 | 0.04127 |
| GO:0102336 | 3-oxo-arachidoyl-CoA synthase activity | 0.285714 | 0.04127 |
| GO:0102338 | 3-oxo-lignoceronyl-CoA synthase activity | 0.285714 | 0.04127 |
| GO:0050733 | RS domain binding | 0.285714 | 0.04127 |
| GO:0008093 | cytoskeletal anchor activity | 0.285714 | 0.04127 |
| GO:0004931 | extracellularly ATP-gated cation channel activity | 0.285714 | 0.04127 |
| GO:0001849 | complement component C1q complex binding | 0.285714 | 0.04127 |
| GO:0004703 | G protein-coupled receptor kinase activity | 0.285714 | 0.04127 |
| GO:0015142 | tricarboxylic acid transmembrane transporter activity | 0.285714 | 0.04127 |
| GO:0015137 | citrate transmembrane transporter activity | 0.285714 | 0.04127 |
| GO:0019798 | procollagen-proline dioxygenase activity | 0.285714 | 0.04127 |
| GO:0035473 | lipase binding | 0.285714 | 0.04127 |
| GO:0035662 | Toll-like receptor 4 binding | 0.285714 | 0.04127 |
| GO:0043878 | glyceraldehyde-3-phosphate dehydrogenase (NAD+) (non-phosphorylating) activity | 0.285714 | 0.04127 |
| GO:0032027 | myosin light chain binding | 0.285714 | 0.04127 |
| GO:0050291 | sphingosine N-acyltransferase activity | 0.285714 | 0.04127 |
| GO:0006672 | ceramide metabolic process | 0.1 | 0.04084 |
| GO:0042176 | regulation of protein catabolic process | 0.069767 | 0.04075 |
| GO:0004518 | nuclease activity | 0.069767 | 0.04075 |
| GO:1901575 | organic substance catabolic process | 0.057377 | 0.04045 |
| GO:0006643 | membrane lipid metabolic process | 0.082474 | 0.04043 |
| GO:0002831 | regulation of response to biotic stimulus | 0.071053 | 0.04032 |
| GO:0009306 | protein secretion | 0.092593 | 0.04011 |
| GO:0033177 | proton-transporting two-sector ATPase complex, proton-transporting domain | 0.092593 | 0.04011 |
| GO:0050660 | flavin adenine dinucleotide binding | 0.092593 | 0.04011 |
| GO:0003714 | transcription corepressor activity | 0.079646 | 0.04009 |
| GO:0045936 | negative regulation of phosphate metabolic process | 0.069474 | 0.03929 |
| GO:0010563 | negative regulation of phosphorus metabolic process | 0.069474 | 0.03929 |
| GO:0016301 | kinase activity | 0.061372 | 0.03904 |
| GO:0051047 | positive regulation of secretion | 0.071618 | 0.03891 |
| GO:0002294 | CD4-positive, alpha-beta T cell differentiation involved in immune response | 0.1875 | 0.03885 |
| GO:0042093 | T-helper cell differentiation | 0.1875 | 0.03885 |
| GO:0051350 | negative regulation of lyase activity | 0.1875 | 0.03885 |
| GO:1902036 | regulation of hematopoietic stem cell differentiation | 0.1875 | 0.03885 |
| GO:0002864 | regulation of acute inflammatory response to antigenic stimulus | 0.1875 | 0.03885 |
| GO:0072080 | nephron tubule development | 0.1875 | 0.03885 |
| GO:0060211 | regulation of nuclear-transcribed mRNA poly(A) tail shortening | 0.1875 | 0.03885 |
| GO:0031579 | membrane raft organization | 0.1875 | 0.03885 |
| GO:0061333 | renal tubule morphogenesis | 0.1875 | 0.03885 |
| GO:0034310 | primary alcohol catabolic process | 0.1875 | 0.03885 |
| GO:2001044 | regulation of integrin-mediated signaling pathway | 0.1875 | 0.03885 |
| GO:0038084 | vascular endothelial growth factor signaling pathway | 0.1875 | 0.03885 |
| GO:0016894 | endonuclease activity, active with either ribo- or deoxyribonucleic acids and producing 3'-phosphomonoesters | 0.1875 | 0.03885 |
| GO:0016671 | oxidoreductase activity, acting on a sulfur group of donors, disulfide as acceptor | 0.1875 | 0.03885 |
| GO:0022841 | potassium ion leak channel activity | 0.1875 | 0.03885 |
| GO:0004303 | estradiol 17-beta-dehydrogenase activity | 0.1875 | 0.03885 |
| GO:0001851 | complement component C3b binding | 0.1875 | 0.03885 |
| GO:0033559 | unsaturated fatty acid metabolic process | 0.089552 | 0.03877 |
| GO:0090596 | sensory organ morphogenesis | 0.106061 | 0.03876 |
| GO:0030545 | signaling receptor regulator activity | 0.065217 | 0.03872 |
| GO:0060143 | positive regulation of syncytium formation by plasma membrane fusion | 0.148148 | 0.03866 |
| GO:0019585 | glucuronate metabolic process | 0.148148 | 0.03866 |
| GO:0060251 | regulation of glial cell proliferation | 0.148148 | 0.03866 |
| GO:0051491 | positive regulation of filopodium assembly | 0.148148 | 0.03866 |
| GO:0019724 | B cell mediated immunity | 0.148148 | 0.03866 |
| GO:0006063 | uronic acid metabolic process | 0.148148 | 0.03866 |
| GO:0010664 | negative regulation of striated muscle cell apoptotic process | 0.148148 | 0.03866 |
| GO:0098945 | intrinsic component of presynaptic active zone membrane | 0.148148 | 0.03866 |
| GO:0005343 | organic acid:sodium symporter activity | 0.148148 | 0.03866 |
| GO:0031690 | adrenergic receptor binding | 0.148148 | 0.03866 |
| GO:0072330 | monocarboxylic acid biosynthetic process | 0.086957 | 0.03864 |
| GO:0007389 | pattern specification process | 0.071809 | 0.03849 |
| GO:0018130 | heterocycle biosynthetic process | 0.062378 | 0.03815 |
| GO:0060548 | negative regulation of cell death | 0.060014 | 0.03792 |
| GO:0002931 | response to ischemia | 0.128205 | 0.03787 |
| GO:0140115 | export across plasma membrane | 0.128205 | 0.03787 |
| GO:0055081 | anion homeostasis | 0.128205 | 0.03787 |
| GO:0060193 | positive regulation of lipase activity | 0.115385 | 0.03782 |
| GO:0033764 | steroid dehydrogenase activity, acting on the CH-OH group of donors, NAD or NADP as acceptor | 0.115385 | 0.03782 |
| GO:0009260 | ribonucleotide biosynthetic process | 0.075601 | 0.03750 |
| GO:0042326 | negative regulation of phosphorylation | 0.072776 | 0.03679 |
| GO:0050433 | regulation of catecholamine secretion | 0.107692 | 0.03609 |
| GO:0045995 | regulation of embryonic development | 0.107692 | 0.03609 |
| GO:0042645 | mitochondrial nucleoid | 0.107692 | 0.03609 |
| GO:0009295 | nucleoid | 0.107692 | 0.03609 |
| GO:0030139 | endocytic vesicle | 0.086093 | 0.03564 |
| GO:0006164 | purine nucleotide biosynthetic process | 0.076923 | 0.03559 |
| GO:0071396 | cellular response to lipid | 0.066993 | 0.03550 |
| GO:0090090 | negative regulation of canonical Wnt signaling pathway | 0.095238 | 0.03544 |
| GO:0000785 | chromatin | 0.067103 | 0.03532 |
| GO:0030659 | cytoplasmic vesicle membrane | 0.065097 | 0.03505 |
| GO:0099601 | regulation of neurotransmitter receptor activity | 0.117647 | 0.03482 |
| GO:0043949 | regulation of cAMP-mediated signaling | 0.117647 | 0.03482 |
| GO:0061138 | morphogenesis of a branching epithelium | 0.093023 | 0.03476 |
| GO:1901503 | ether biosynthetic process | 0.131579 | 0.03433 |
| GO:0046504 | glycerol ether biosynthetic process | 0.131579 | 0.03433 |
| GO:0006801 | superoxide metabolic process | 0.131579 | 0.03433 |
| GO:0042116 | macrophage activation | 0.131579 | 0.03433 |
| GO:0070059 | intrinsic apoptotic signaling pathway in response to endoplasmic reticulum stress | 0.131579 | 0.03433 |
| GO:0010939 | regulation of necrotic cell death | 0.131579 | 0.03433 |
| GO:0001974 | blood vessel remodeling | 0.131579 | 0.03433 |
| GO:1900077 | negative regulation of cellular response to insulin stimulus | 0.131579 | 0.03433 |
| GO:0008611 | ether lipid biosynthetic process | 0.131579 | 0.03433 |
| GO:0007498 | mesoderm development | 0.131579 | 0.03433 |
| GO:0070527 | platelet aggregation | 0.131579 | 0.03433 |
| GO:0005501 | retinoid binding | 0.131579 | 0.03433 |
| GO:0007035 | vacuolar acidification | 0.153846 | 0.03419 |
| GO:0060765 | regulation of androgen receptor signaling pathway | 0.153846 | 0.03419 |
| GO:0090102 | cochlea development | 0.153846 | 0.03419 |
| GO:1900037 | regulation of cellular response to hypoxia | 0.153846 | 0.03419 |
| GO:0055067 | monovalent inorganic cation homeostasis | 0.09375 | 0.03417 |
| GO:0022402 | cell cycle process | 0.063666 | 0.03403 |
| GO:0043010 | camera-type eye development | 0.102564 | 0.03362 |
| GO:1900076 | regulation of cellular response to insulin stimulus | 0.109375 | 0.03354 |
| GO:0098563 | intrinsic component of synaptic vesicle membrane | 0.109375 | 0.03354 |
| GO:0001786 | phosphatidylserine binding | 0.109375 | 0.03354 |
| GO:0051100 | negative regulation of binding | 0.087248 | 0.03334 |
| GO:0030546 | signaling receptor activator activity | 0.066461 | 0.03277 |
| GO:0005634 | nucleus | 0.052808 | 0.03274 |
| GO:0048050 | post-embryonic eye morphogenesis | 0.2 | 0.03271 |
| GO:0002921 | negative regulation of humoral immune response | 0.2 | 0.03271 |
| GO:0072078 | nephron tubule morphogenesis | 0.2 | 0.03271 |
| GO:0090050 | positive regulation of cell migration involved in sprouting angiogenesis | 0.2 | 0.03271 |
| GO:0071285 | cellular response to lithium ion | 0.2 | 0.03271 |
| GO:0072088 | nephron epithelium morphogenesis | 0.2 | 0.03271 |
| GO:0060252 | positive regulation of glial cell proliferation | 0.2 | 0.03271 |
| GO:0014733 | regulation of skeletal muscle adaptation | 0.2 | 0.03271 |
| GO:1902306 | negative regulation of sodium ion transmembrane transport | 0.2 | 0.03271 |
| GO:0030497 | fatty acid elongation | 0.2 | 0.03271 |
| GO:1903019 | negative regulation of glycoprotein metabolic process | 0.2 | 0.03271 |
| GO:2000291 | regulation of myoblast proliferation | 0.2 | 0.03271 |
| GO:0032421 | stereocilium bundle | 0.2 | 0.03271 |
| GO:0098691 | dopaminergic synapse | 0.2 | 0.03271 |
| GO:0015643 | toxic substance binding | 0.2 | 0.03271 |
| GO:0048019 | receptor antagonist activity | 0.2 | 0.03271 |
| GO:0003012 | muscle system process | 0.081731 | 0.03259 |
| GO:0090092 | regulation of transmembrane receptor protein serine/threonine kinase signaling pathway | 0.081731 | 0.03259 |
| GO:0045596 | negative regulation of cell differentiation | 0.065359 | 0.03239 |
| GO:0033135 | regulation of peptidyl-serine phosphorylation | 0.087838 | 0.03228 |
| GO:0002263 | cell activation involved in immune response | 0.084746 | 0.03222 |
| GO:0045732 | positive regulation of protein catabolic process | 0.079498 | 0.03198 |
| GO:0045333 | cellular respiration | 0.082126 | 0.03198 |
| GO:0043903 | regulation of biological process involved in symbiotic interaction | 0.12 | 0.03198 |
| GO:0043457 | regulation of cellular respiration | 0.12 | 0.03198 |
| GO:0031349 | positive regulation of defense response | 0.077206 | 0.03176 |
| GO:0035639 | purine ribonucleoside triphosphate binding | 0.056391 | 0.03171 |
| GO:1903844 | regulation of cellular response to transforming growth factor beta stimulus | 0.098039 | 0.03153 |
| GO:0003013 | circulatory system process | 0.085227 | 0.03148 |
| GO:0006874 | cellular calcium ion homeostasis | 0.085227 | 0.03148 |
| GO:0051881 | regulation of mitochondrial membrane potential | 0.103896 | 0.03144 |
| GO:0005765 | lysosomal membrane | 0.082524 | 0.03140 |
| GO:0043433 | negative regulation of DNA-binding transcription factor activity | 0.088435 | 0.03127 |
| GO:1901136 | carbohydrate derivative catabolic process | 0.088435 | 0.03127 |
| GO:0032675 | regulation of interleukin-6 production | 0.092437 | 0.03125 |
| GO:0001503 | ossification | 0.092437 | 0.03125 |
| GO:0014910 | regulation of smooth muscle cell migration | 0.111111 | 0.03111 |
| GO:0015031 | protein transport | 0.060455 | 0.03101 |
| GO:0016339 | calcium-dependent cell-cell adhesion via plasma membrane cell adhesion molecules | 0.135135 | 0.03099 |
| GO:0021675 | nerve development | 0.135135 | 0.03099 |
| GO:0009311 | oligosaccharide metabolic process | 0.135135 | 0.03099 |
| GO:0042445 | hormone metabolic process | 0.085714 | 0.03079 |
| GO:0031406 | carboxylic acid binding | 0.085714 | 0.03079 |
| GO:0050839 | cell adhesion molecule binding | 0.074928 | 0.03076 |
| GO:2000109 | regulation of macrophage apoptotic process | 0.333333 | 0.03044 |
| GO:0015712 | hexose phosphate transport | 0.333333 | 0.03044 |
| GO:0150065 | regulation of deacetylase activity | 0.333333 | 0.03044 |
| GO:1990314 | cellular response to insulin-like growth factor stimulus | 0.333333 | 0.03044 |
| GO:0014744 | positive regulation of muscle adaptation | 0.333333 | 0.03044 |
| GO:0097250 | mitochondrial respirasome assembly | 0.333333 | 0.03044 |
| GO:1903243 | negative regulation of cardiac muscle hypertrophy in response to stress | 0.333333 | 0.03044 |
| GO:0009186 | deoxyribonucleoside diphosphate metabolic process | 0.333333 | 0.03044 |
| GO:0044857 | plasma membrane raft organization | 0.333333 | 0.03044 |
| GO:1900020 | positive regulation of protein kinase C activity | 0.333333 | 0.03044 |
| GO:0002865 | negative regulation of acute inflammatory response to antigenic stimulus | 0.333333 | 0.03044 |
| GO:0120252 | hydrocarbon metabolic process | 0.333333 | 0.03044 |
| GO:1900748 | positive regulation of vascular endothelial growth factor signaling pathway | 0.333333 | 0.03044 |
| GO:0033292 | T-tubule organization | 0.333333 | 0.03044 |
| GO:0003198 | epithelial to mesenchymal transition involved in endocardial cushion formation | 0.333333 | 0.03044 |
| GO:0038033 | positive regulation of endothelial cell chemotaxis by VEGF-activated vascular endothelial growth factor receptor signaling pathway | 0.333333 | 0.03044 |
| GO:0060008 | Sertoli cell differentiation | 0.333333 | 0.03044 |
| GO:0050942 | positive regulation of pigment cell differentiation | 0.333333 | 0.03044 |
| GO:0002315 | marginal zone B cell differentiation | 0.333333 | 0.03044 |
| GO:0061687 | detoxification of inorganic compound | 0.333333 | 0.03044 |
| GO:0002467 | germinal center formation | 0.333333 | 0.03044 |
| GO:0003330 | regulation of extracellular matrix constituent secretion | 0.333333 | 0.03044 |
| GO:0015886 | heme transport | 0.333333 | 0.03044 |
| GO:0003215 | cardiac right ventricle morphogenesis | 0.333333 | 0.03044 |
| GO:0015760 | glucose-6-phosphate transport | 0.333333 | 0.03044 |
| GO:0048087 | positive regulation of developmental pigmentation | 0.333333 | 0.03044 |
| GO:1904778 | positive regulation of protein localization to cell cortex | 0.333333 | 0.03044 |
| GO:0010519 | negative regulation of phospholipase activity | 0.333333 | 0.03044 |
| GO:0015744 | succinate transport | 0.333333 | 0.03044 |
| GO:0010990 | regulation of SMAD protein complex assembly | 0.333333 | 0.03044 |
| GO:0000820 | regulation of glutamine family amino acid metabolic process | 0.333333 | 0.03044 |
| GO:1900019 | regulation of protein kinase C activity | 0.333333 | 0.03044 |
| GO:0046487 | glyoxylate metabolic process | 0.333333 | 0.03044 |
| GO:0061009 | common bile duct development | 0.333333 | 0.03044 |
| GO:0090324 | negative regulation of oxidative phosphorylation | 0.333333 | 0.03044 |
| GO:0051601 | exocyst localization | 0.333333 | 0.03044 |
| GO:0045634 | regulation of melanocyte differentiation | 0.333333 | 0.03044 |
| GO:0051001 | negative regulation of nitric-oxide synthase activity | 0.333333 | 0.03044 |
| GO:0099031 | anchored component of postsynaptic density membrane | 0.333333 | 0.03044 |
| GO:0072557 | IPAF inflammasome complex | 0.333333 | 0.03044 |
| GO:0030663 | COPI-coated vesicle membrane | 0.333333 | 0.03044 |
| GO:0099030 | anchored component of postsynaptic specialization membrane | 0.333333 | 0.03044 |
| GO:0097208 | alveolar lamellar body | 0.333333 | 0.03044 |
| GO:0004645 | 1,4-alpha-oligoglucan phosphorylase activity | 0.333333 | 0.03044 |
| GO:0016892 | endoribonuclease activity, producing 3'-phosphomonoesters | 0.333333 | 0.03044 |
| GO:0070095 | fructose-6-phosphate binding | 0.333333 | 0.03044 |
| GO:0005004 | GPI-linked ephrin receptor activity | 0.333333 | 0.03044 |
| GO:0005035 | death receptor activity | 0.333333 | 0.03044 |
| GO:0030235 | nitric-oxide synthase regulator activity | 0.333333 | 0.03044 |
| GO:1903522 | regulation of blood circulation | 0.083333 | 0.03037 |
| GO:2001257 | regulation of cation channel activity | 0.089041 | 0.03032 |
| GO:0030832 | regulation of actin filament length | 0.086207 | 0.03015 |
| GO:0070160 | tight junction | 0.086207 | 0.03015 |
| GO:0090208 | positive regulation of triglyceride metabolic process | 0.16 | 0.03004 |
| GO:0030325 | adrenal gland development | 0.16 | 0.03004 |
| GO:0014065 | phosphatidylinositol 3-kinase signaling | 0.16 | 0.03004 |
| GO:0032689 | negative regulation of interferon-gamma production | 0.16 | 0.03004 |
| GO:0090162 | establishment of epithelial cell polarity | 0.16 | 0.03004 |
| GO:0035025 | positive regulation of Rho protein signal transduction | 0.16 | 0.03004 |
| GO:0048710 | regulation of astrocyte differentiation | 0.16 | 0.03004 |
| GO:0050750 | low-density lipoprotein particle receptor binding | 0.16 | 0.03004 |
| GO:0072507 | divalent inorganic cation homeostasis | 0.081197 | 0.02971 |
| GO:0007051 | spindle organization | 0.086705 | 0.02956 |
| GO:0004857 | enzyme inhibitor activity | 0.065484 | 0.02945 |
| GO:0016684 | oxidoreductase activity, acting on peroxide as acceptor | 0.105263 | 0.02937 |
| GO:0001954 | positive regulation of cell-matrix adhesion | 0.122449 | 0.02929 |
| GO:0046513 | ceramide biosynthetic process | 0.122449 | 0.02929 |
| GO:0030864 | cortical actin cytoskeleton | 0.122449 | 0.02929 |
| GO:0030016 | myofibril | 0.122449 | 0.02929 |
| GO:0097060 | synaptic membrane | 0.076412 | 0.02919 |
| GO:0008064 | regulation of actin polymerization or depolymerization | 0.087209 | 0.02901 |
| GO:0046631 | alpha-beta T cell activation | 0.112903 | 0.02881 |
| GO:0045912 | negative regulation of carbohydrate metabolic process | 0.112903 | 0.02881 |
| GO:0014068 | positive regulation of phosphatidylinositol 3-kinase signaling | 0.112903 | 0.02881 |
| GO:0009743 | response to carbohydrate | 0.087719 | 0.02850 |
| GO:0005319 | lipid transporter activity | 0.087719 | 0.02850 |
| GO:0005126 | cytokine receptor binding | 0.074074 | 0.02846 |
| GO:0017015 | regulation of transforming growth factor beta receptor signaling pathway | 0.10101 | 0.02834 |
| GO:1901890 | positive regulation of cell junction assembly | 0.10101 | 0.02834 |
| GO:0008344 | adult locomotory behavior | 0.10101 | 0.02834 |
| GO:0042773 | ATP synthesis coupled electron transport | 0.138889 | 0.02787 |
| GO:1900271 | regulation of long-term synaptic potentiation | 0.138889 | 0.02787 |
| GO:0045940 | positive regulation of steroid metabolic process | 0.138889 | 0.02787 |
| GO:1903670 | regulation of sprouting angiogenesis | 0.138889 | 0.02787 |
| GO:0098609 | cell-cell adhesion | 0.069583 | 0.02750 |
| GO:0030004 | cellular monovalent inorganic cation homeostasis | 0.102041 | 0.02744 |
| GO:0001539 | cilium or flagellum-dependent cell motility | 0.102041 | 0.02744 |
| GO:0060285 | cilium-dependent cell motility | 0.102041 | 0.02744 |
| GO:0004601 | peroxidase activity | 0.106667 | 0.02739 |
| GO:0030141 | secretory granule | 0.07337 | 0.02739 |
| GO:0035150 | regulation of tube size | 0.094828 | 0.02737 |
| GO:0001676 | long-chain fatty acid metabolic process | 0.094828 | 0.02737 |
| GO:0033138 | positive regulation of peptidyl-serine phosphorylation | 0.094828 | 0.02737 |
| GO:0007162 | negative regulation of cell adhesion | 0.07874 | 0.02717 |
| GO:0006620 | post-translational protein targeting to endoplasmic reticulum membrane | 0.214286 | 0.02711 |
| GO:0000289 | nuclear-transcribed mRNA poly(A) tail shortening | 0.214286 | 0.02711 |
| GO:0050667 | homocysteine metabolic process | 0.214286 | 0.02711 |
| GO:1901072 | glucosamine-containing compound catabolic process | 0.214286 | 0.02711 |
| GO:0032793 | positive regulation of CREB transcription factor activity | 0.214286 | 0.02711 |
| GO:0030539 | male genitalia development | 0.214286 | 0.02711 |
| GO:0001655 | urogenital system development | 0.214286 | 0.02711 |
| GO:2000209 | regulation of anoikis | 0.214286 | 0.02711 |
| GO:0030225 | macrophage differentiation | 0.214286 | 0.02711 |
| GO:0060213 | positive regulation of nuclear-transcribed mRNA poly(A) tail shortening | 0.214286 | 0.02711 |
| GO:2001028 | positive regulation of endothelial cell chemotaxis | 0.214286 | 0.02711 |
| GO:0046341 | CDP-diacylglycerol metabolic process | 0.214286 | 0.02711 |
| GO:0035641 | locomotory exploration behavior | 0.214286 | 0.02711 |
| GO:0002523 | leukocyte migration involved in inflammatory response | 0.214286 | 0.02711 |
| GO:0006020 | inositol metabolic process | 0.214286 | 0.02711 |
| GO:0003407 | neural retina development | 0.214286 | 0.02711 |
| GO:0060055 | angiogenesis involved in wound healing | 0.214286 | 0.02711 |
| GO:0042761 | very long-chain fatty acid biosynthetic process | 0.214286 | 0.02711 |
| GO:0010896 | regulation of triglyceride catabolic process | 0.214286 | 0.02711 |
| GO:0072393 | microtubule anchoring at microtubule organizing center | 0.214286 | 0.02711 |
| GO:0042627 | chylomicron | 0.214286 | 0.02711 |
| GO:0001527 | microfibril | 0.214286 | 0.02711 |
| GO:0098871 | postsynaptic actin cytoskeleton | 0.214286 | 0.02711 |
| GO:0047023 | androsterone dehydrogenase activity | 0.214286 | 0.02711 |
| GO:0042910 | xenobiotic transmembrane transporter activity | 0.214286 | 0.02711 |
| GO:0019902 | phosphatase binding | 0.08377 | 0.02708 |
| GO:0018904 | ether metabolic process | 0.125 | 0.02675 |
| GO:0051496 | positive regulation of stress fiber assembly | 0.125 | 0.02675 |
| GO:0030017 | sarcomere | 0.125 | 0.02675 |
| GO:0140678 | molecular function inhibitor activity | 0.125 | 0.02675 |
| GO:0003707 | nuclear steroid receptor activity | 0.125 | 0.02675 |
| GO:0055008 | cardiac muscle tissue morphogenesis | 0.114754 | 0.02662 |
| GO:0046676 | negative regulation of insulin secretion | 0.114754 | 0.02662 |
| GO:0045184 | establishment of protein localization | 0.060362 | 0.02653 |
| GO:0009746 | response to hexose | 0.092199 | 0.02639 |
| GO:0031960 | response to corticosteroid | 0.084211 | 0.02631 |
| GO:0072594 | establishment of protein localization to organelle | 0.073973 | 0.02624 |
| GO:0042886 | amide transport | 0.095652 | 0.02620 |
| GO:0014888 | striated muscle adaptation | 0.166667 | 0.02620 |
| GO:0033627 | cell adhesion mediated by integrin | 0.166667 | 0.02620 |
| GO:0035278 | miRNA-mediated gene silencing by inhibition of translation | 0.166667 | 0.02620 |
| GO:0010667 | negative regulation of cardiac muscle cell apoptotic process | 0.166667 | 0.02620 |
| GO:0050780 | dopamine receptor binding | 0.166667 | 0.02620 |
| GO:0010468 | regulation of gene expression | 0.05355 | 0.02602 |
| GO:0090263 | positive regulation of canonical Wnt signaling pathway | 0.104167 | 0.02587 |
| GO:0098685 | Schaffer collateral - CA1 synapse | 0.104167 | 0.02587 |
| GO:0010675 | regulation of cellular carbohydrate metabolic process | 0.084656 | 0.02558 |
| GO:0071479 | cellular response to ionizing radiation | 0.108108 | 0.02550 |
| GO:0006081 | cellular aldehyde metabolic process | 0.108108 | 0.02550 |
| GO:0017022 | myosin binding | 0.108108 | 0.02550 |
| GO:0001817 | regulation of cytokine production | 0.064699 | 0.02544 |
| GO:0097193 | intrinsic apoptotic signaling pathway | 0.08805 | 0.02508 |
| GO:0033144 | negative regulation of intracellular steroid hormone receptor signaling pathway | 0.142857 | 0.02496 |
| GO:0046627 | negative regulation of insulin receptor signaling pathway | 0.142857 | 0.02496 |
| GO:0002062 | chondrocyte differentiation | 0.142857 | 0.02496 |
| GO:1901565 | organonitrogen compound catabolic process | 0.064665 | 0.02452 |
| GO:2001222 | regulation of neuron migration | 0.12766 | 0.02437 |
| GO:0044839 | cell cycle G2/M phase transition | 0.12766 | 0.02437 |
| GO:0045197 | establishment or maintenance of epithelial cell apical/basal polarity | 0.12766 | 0.02437 |
| GO:0032813 | tumor necrosis factor receptor superfamily binding | 0.12766 | 0.02437 |
| GO:0022407 | regulation of cell-cell adhesion | 0.075419 | 0.02427 |
| GO:0060415 | muscle tissue morphogenesis | 0.109589 | 0.02371 |
| GO:0032543 | mitochondrial translation | 0.109589 | 0.02371 |
| GO:0000987 | cis-regulatory region sequence-specific DNA binding | 0.065491 | 0.02371 |
| GO:0031668 | cellular response to extracellular stimulus | 0.080972 | 0.02363 |
| GO:0005525 | GTP binding | 0.068471 | 0.02345 |
| GO:0043523 | regulation of neuron apoptotic process | 0.083721 | 0.02327 |
| GO:0051262 | protein tetramerization | 0.086486 | 0.02304 |
| GO:0000981 | DNA-binding transcription factor activity, RNA polymerase II-specific | 0.063401 | 0.02281 |
| GO:0010720 | positive regulation of cell development | 0.07717 | 0.02270 |
| GO:0006875 | cellular metal ion homeostasis | 0.07717 | 0.02270 |
| GO:1901570 | fatty acid derivative biosynthetic process | 0.173913 | 0.02268 |
| GO:0002082 | regulation of oxidative phosphorylation | 0.173913 | 0.02268 |
| GO:0019200 | carbohydrate kinase activity | 0.173913 | 0.02268 |
| GO:0043395 | heparan sulfate proteoglycan binding | 0.173913 | 0.02268 |
| GO:0098797 | plasma membrane protein complex | 0.068182 | 0.02260 |
| GO:0001570 | vasculogenesis | 0.118644 | 0.02259 |
| GO:0014015 | positive regulation of gliogenesis | 0.118644 | 0.02259 |
| GO:0002275 | myeloid cell activation involved in immune response | 0.118644 | 0.02259 |
| GO:2000116 | regulation of cysteine-type endopeptidase activity | 0.081967 | 0.02253 |
| GO:0032271 | regulation of protein polymerization | 0.084507 | 0.02245 |
| GO:1903828 | negative regulation of protein localization | 0.084507 | 0.02245 |
| GO:0009062 | fatty acid catabolic process | 0.104651 | 0.02236 |
| GO:1901616 | organic hydroxy compound catabolic process | 0.104651 | 0.02236 |
| GO:0048029 | monosaccharide binding | 0.104651 | 0.02236 |
| GO:1901031 | regulation of response to reactive oxygen species | 0.147059 | 0.02225 |
| GO:0072163 | mesonephric epithelium development | 0.147059 | 0.02225 |
| GO:0016248 | channel inhibitor activity | 0.147059 | 0.02225 |
| GO:0004032 | alditol:NADP+ 1-oxidoreductase activity | 0.147059 | 0.02225 |
| GO:0051289 | protein homotetramerization | 0.099099 | 0.02216 |
| GO:0051294 | establishment of spindle orientation | 0.130435 | 0.02213 |
| GO:1902041 | regulation of extrinsic apoptotic signaling pathway via death domain receptors | 0.130435 | 0.02213 |
| GO:0030330 | DNA damage response, signal transduction by p53 class mediator | 0.130435 | 0.02213 |
| GO:0010662 | regulation of striated muscle cell apoptotic process | 0.130435 | 0.02213 |
| GO:0008106 | alcohol dehydrogenase (NADP+) activity | 0.130435 | 0.02213 |
| GO:0060263 | regulation of respiratory burst | 0.230769 | 0.02208 |
| GO:0010752 | regulation of cGMP-mediated signaling | 0.230769 | 0.02208 |
| GO:0061365 | positive regulation of triglyceride lipase activity | 0.230769 | 0.02208 |
| GO:0071257 | cellular response to electrical stimulus | 0.230769 | 0.02208 |
| GO:0016102 | diterpenoid biosynthetic process | 0.230769 | 0.02208 |
| GO:0036336 | dendritic cell migration | 0.230769 | 0.02208 |
| GO:0060099 | regulation of phagocytosis, engulfment | 0.230769 | 0.02208 |
| GO:0044130 | obsolete negative regulation of growth of symbiont in host | 0.230769 | 0.02208 |
| GO:0002281 | macrophage activation involved in immune response | 0.230769 | 0.02208 |
| GO:0002138 | retinoic acid biosynthetic process | 0.230769 | 0.02208 |
| GO:2001212 | regulation of vasculogenesis | 0.230769 | 0.02208 |
| GO:0032096 | negative regulation of response to food | 0.230769 | 0.02208 |
| GO:2000650 | negative regulation of sodium ion transmembrane transporter activity | 0.230769 | 0.02208 |
| GO:0016024 | CDP-diacylglycerol biosynthetic process | 0.230769 | 0.02208 |
| GO:0032105 | negative regulation of response to extracellular stimulus | 0.230769 | 0.02208 |
| GO:0032108 | negative regulation of response to nutrient levels | 0.230769 | 0.02208 |
| GO:1904478 | regulation of intestinal absorption | 0.230769 | 0.02208 |
| GO:1900016 | negative regulation of cytokine production involved in inflammatory response | 0.230769 | 0.02208 |
| GO:0006068 | ethanol catabolic process | 0.230769 | 0.02208 |
| GO:0052697 | xenobiotic glucuronidation | 0.230769 | 0.02208 |
| GO:0032393 | MHC class I receptor activity | 0.230769 | 0.02208 |
| GO:0008191 | metalloendopeptidase inhibitor activity | 0.230769 | 0.02208 |
| GO:0042301 | phosphate ion binding | 0.230769 | 0.02208 |
| GO:0004535 | poly(A)-specific ribonuclease activity | 0.230769 | 0.02208 |
| GO:0070161 | anchoring junction | 0.065554 | 0.02206 |
| GO:0001505 | regulation of neurotransmitter levels | 0.087432 | 0.02199 |
| GO:0022607 | cellular component assembly | 0.055597 | 0.02198 |
| GO:0009152 | purine ribonucleotide biosynthetic process | 0.080292 | 0.02179 |
| GO:0045862 | positive regulation of proteolysis | 0.075802 | 0.02178 |
| GO:0030183 | B cell differentiation | 0.094488 | 0.02143 |
| GO:0050731 | positive regulation of peptidyl-tyrosine phosphorylation | 0.090909 | 0.02120 |
| GO:0008021 | synaptic vesicle | 0.090909 | 0.02120 |
| GO:0010757 | negative regulation of plasminogen activation | 0.4 | 0.02096 |
| GO:0007199 | G protein-coupled receptor signaling pathway coupled to cGMP nucleotide second messenger | 0.4 | 0.02096 |
| GO:0003158 | endothelium development | 0.4 | 0.02096 |
| GO:0003176 | aortic valve development | 0.4 | 0.02096 |
| GO:0120253 | hydrocarbon catabolic process | 0.4 | 0.02096 |
| GO:0042117 | monocyte activation | 0.4 | 0.02096 |
| GO:1990961 | xenobiotic detoxification by transmembrane export across the plasma membrane | 0.4 | 0.02096 |
| GO:0060510 | type II pneumocyte differentiation | 0.4 | 0.02096 |
| GO:0042088 | T-helper 1 type immune response | 0.4 | 0.02096 |
| GO:0060136 | embryonic process involved in female pregnancy | 0.4 | 0.02096 |
| GO:1905049 | negative regulation of metallopeptidase activity | 0.4 | 0.02096 |
| GO:0007253 | cytoplasmic sequestering of NF-kappaB | 0.4 | 0.02096 |
| GO:1901725 | regulation of histone deacetylase activity | 0.4 | 0.02096 |
| GO:1905314 | semi-lunar valve development | 0.4 | 0.02096 |
| GO:1900449 | regulation of glutamate receptor signaling pathway | 0.4 | 0.02096 |
| GO:0010991 | negative regulation of SMAD protein complex assembly | 0.4 | 0.02096 |
| GO:0090027 | negative regulation of monocyte chemotaxis | 0.4 | 0.02096 |
| GO:0010815 | bradykinin catabolic process | 0.4 | 0.02096 |
| GO:0006548 | histidine catabolic process | 0.4 | 0.02096 |
| GO:0035754 | B cell chemotaxis | 0.4 | 0.02096 |
| GO:1900736 | regulation of phospholipase C-activating G protein-coupled receptor signaling pathway | 0.4 | 0.02096 |
| GO:0031427 | response to methotrexate | 0.4 | 0.02096 |
| GO:0072719 | cellular response to cisplatin | 0.4 | 0.02096 |
| GO:0015677 | copper ion import | 0.4 | 0.02096 |
| GO:1902109 | negative regulation of mitochondrial membrane permeability involved in apoptotic process | 0.4 | 0.02096 |
| GO:0033227 | dsRNA transport | 0.4 | 0.02096 |
| GO:1901842 | negative regulation of high voltage-gated calcium channel activity | 0.4 | 0.02096 |
| GO:0044107 | cellular alcohol metabolic process | 0.4 | 0.02096 |
| GO:0044108 | cellular alcohol biosynthetic process | 0.4 | 0.02096 |
| GO:0015739 | sialic acid transport | 0.4 | 0.02096 |
| GO:0000214 | tRNA-intron endonuclease complex | 0.4 | 0.02096 |
| GO:0044326 | dendritic spine neck | 0.4 | 0.02096 |
| GO:0008508 | bile acid:sodium symporter activity | 0.4 | 0.02096 |
| GO:0033265 | choline binding | 0.4 | 0.02096 |
| GO:0030160 | synaptic receptor adaptor activity | 0.4 | 0.02096 |
| GO:0042731 | PH domain binding | 0.4 | 0.02096 |
| GO:0017153 | sodium:dicarboxylate symporter activity | 0.4 | 0.02096 |
| GO:0016716 | oxidoreductase activity, acting on paired donors, with incorporation or reduction of molecular oxygen, another compound as one donor, and incorporation of one atom of oxygen | 0.4 | 0.02096 |
| GO:0060230 | lipoprotein lipase activator activity | 0.4 | 0.02096 |
| GO:0031721 | hemoglobin alpha binding | 0.4 | 0.02096 |
| GO:0016936 | galactoside binding | 0.4 | 0.02096 |
| GO:0019153 | protein-disulfide reductase (glutathione) activity | 0.4 | 0.02096 |
| GO:0023024 | MHC class I protein complex binding | 0.4 | 0.02096 |
| GO:0051129 | negative regulation of cellular component organization | 0.067606 | 0.02090 |
| GO:0097722 | sperm motility | 0.105882 | 0.02088 |
| GO:0015914 | phospholipid transport | 0.105882 | 0.02088 |
| GO:0022008 | neurogenesis | 0.105882 | 0.02088 |
| GO:0051492 | regulation of stress fiber assembly | 0.105882 | 0.02088 |
| GO:0098862 | cluster of actin-based cell projections | 0.105882 | 0.02088 |
| GO:0016836 | hydro-lyase activity | 0.105882 | 0.02088 |
| GO:0055117 | regulation of cardiac muscle contraction | 0.12069 | 0.02075 |
| GO:0050805 | negative regulation of synaptic transmission | 0.12069 | 0.02075 |
| GO:0048017 | inositol lipid-mediated signaling | 0.12069 | 0.02075 |
| GO:0002697 | regulation of immune effector process | 0.074667 | 0.02070 |
| GO:0010952 | positive regulation of peptidase activity | 0.088889 | 0.02069 |
| GO:0019751 | polyol metabolic process | 0.091503 | 0.02055 |
| GO:0007601 | visual perception | 0.091503 | 0.02055 |
| GO:0090277 | positive regulation of peptide hormone secretion | 0.100917 | 0.02050 |
| GO:0006814 | sodium ion transport | 0.100917 | 0.02050 |
| GO:0006662 | glycerol ether metabolic process | 0.133333 | 0.02003 |
| GO:0014911 | positive regulation of smooth muscle cell migration | 0.133333 | 0.02003 |
| GO:0040001 | establishment of mitotic spindle localization | 0.133333 | 0.02003 |
| GO:0043394 | proteoglycan binding | 0.133333 | 0.02003 |
| GO:0015081 | sodium ion transmembrane transporter activity | 0.092105 | 0.01994 |
| GO:0019904 | protein domain specific binding | 0.06391 | 0.01989 |
| GO:0015849 | organic acid transport | 0.082609 | 0.01977 |
| GO:0051651 | maintenance of location in cell | 0.101852 | 0.01976 |
| GO:0046466 | membrane lipid catabolic process | 0.151515 | 0.01974 |
| GO:0032715 | negative regulation of interleukin-6 production | 0.151515 | 0.01974 |
| GO:0061564 | axon development | 0.151515 | 0.01974 |
| GO:0072164 | mesonephric tubule development | 0.151515 | 0.01974 |
| GO:0060840 | artery development | 0.151515 | 0.01974 |
| GO:1901861 | regulation of muscle tissue development | 0.151515 | 0.01974 |
| GO:0010001 | glial cell differentiation | 0.107143 | 0.01947 |
| GO:0046889 | positive regulation of lipid biosynthetic process | 0.107143 | 0.01947 |
| GO:0030317 | flagellated sperm motility | 0.107143 | 0.01947 |
| GO:0051604 | protein maturation | 0.078498 | 0.01946 |
| GO:0043248 | proteasome assembly | 0.181818 | 0.01946 |
| GO:0055093 | response to hyperoxia | 0.181818 | 0.01946 |
| GO:0042908 | xenobiotic transport | 0.181818 | 0.01946 |
| GO:0060536 | cartilage morphogenesis | 0.181818 | 0.01946 |
| GO:0002063 | chondrocyte development | 0.181818 | 0.01946 |
| GO:0016064 | immunoglobulin mediated immune response | 0.181818 | 0.01946 |
| GO:0003206 | cardiac chamber morphogenesis | 0.181818 | 0.01946 |
| GO:0052695 | cellular glucuronidation | 0.181818 | 0.01946 |
| GO:0016327 | apicolateral plasma membrane | 0.181818 | 0.01946 |
| GO:0016502 | nucleotide receptor activity | 0.181818 | 0.01946 |
| GO:0001614 | purinergic nucleotide receptor activity | 0.181818 | 0.01946 |
| GO:2001242 | regulation of intrinsic apoptotic signaling pathway | 0.085427 | 0.01939 |
| GO:0072329 | monocarboxylic acid catabolic process | 0.102804 | 0.01908 |
| GO:0032410 | negative regulation of transporter activity | 0.114286 | 0.01887 |
| GO:0006836 | neurotransmitter transport | 0.114286 | 0.01887 |
| GO:0046165 | alcohol biosynthetic process | 0.093333 | 0.01885 |
| GO:0045121 | membrane raft | 0.074257 | 0.01877 |
| GO:0002698 | negative regulation of immune effector process | 0.103774 | 0.01845 |
| GO:0031398 | positive regulation of protein ubiquitination | 0.09396 | 0.01837 |
| GO:0005814 | centriole | 0.089286 | 0.01825 |
| GO:0060606 | tube closure | 0.108434 | 0.01813 |
| GO:0005246 | calcium channel regulator activity | 0.136364 | 0.01808 |
| GO:0055088 | lipid homeostasis | 0.094595 | 0.01793 |
| GO:0008277 | regulation of G protein-coupled receptor signaling pathway | 0.104762 | 0.01788 |
| GO:0044853 | plasma membrane raft | 0.097561 | 0.01784 |
| GO:0097178 | ruffle assembly | 0.25 | 0.01760 |
| GO:0045656 | negative regulation of monocyte differentiation | 0.25 | 0.01760 |
| GO:0019673 | GDP-mannose metabolic process | 0.25 | 0.01760 |
| GO:0071636 | positive regulation of transforming growth factor beta production | 0.25 | 0.01760 |
| GO:0045916 | negative regulation of complement activation | 0.25 | 0.01760 |
| GO:1901386 | negative regulation of voltage-gated calcium channel activity | 0.25 | 0.01760 |
| GO:0007263 | nitric oxide mediated signal transduction | 0.25 | 0.01760 |
| GO:0040037 | negative regulation of fibroblast growth factor receptor signaling pathway | 0.25 | 0.01760 |
| GO:0061053 | somite development | 0.25 | 0.01760 |
| GO:0010269 | response to selenium ion | 0.25 | 0.01760 |
| GO:0072578 | neurotransmitter-gated ion channel clustering | 0.25 | 0.01760 |
| GO:0097106 | postsynaptic density organization | 0.25 | 0.01760 |
| GO:0071305 | cellular response to vitamin D | 0.25 | 0.01760 |
| GO:0046949 | fatty-acyl-CoA biosynthetic process | 0.25 | 0.01760 |
| GO:0032099 | negative regulation of appetite | 0.25 | 0.01760 |
| GO:0006957 | complement activation, alternative pathway | 0.25 | 0.01760 |
| GO:0044065 | regulation of respiratory system process | 0.25 | 0.01760 |
| GO:0060670 | branching involved in labyrinthine layer morphogenesis | 0.25 | 0.01760 |
| GO:0007168 | receptor guanylyl cyclase signaling pathway | 0.25 | 0.01760 |
| GO:0097011 | cellular response to granulocyte macrophage colony-stimulating factor stimulus | 0.25 | 0.01760 |
| GO:0097012 | response to granulocyte macrophage colony-stimulating factor | 0.25 | 0.01760 |
| GO:0030388 | fructose 1,6-bisphosphate metabolic process | 0.25 | 0.01760 |
| GO:0010898 | positive regulation of triglyceride catabolic process | 0.25 | 0.01760 |
| GO:0072710 | response to hydroxyurea | 0.25 | 0.01760 |
| GO:0051006 | positive regulation of lipoprotein lipase activity | 0.25 | 0.01760 |
| GO:0042589 | zymogen granule membrane | 0.25 | 0.01760 |
| GO:0099026 | anchored component of presynaptic membrane | 0.25 | 0.01760 |
| GO:0033179 | proton-transporting V-type ATPase, V0 domain | 0.25 | 0.01760 |
| GO:0030915 | Smc5-Smc6 complex | 0.25 | 0.01760 |
| GO:0044322 | endoplasmic reticulum quality control compartment | 0.25 | 0.01760 |
| GO:0004016 | adenylate cyclase activity | 0.25 | 0.01760 |
| GO:0005923 | bicellular tight junction | 0.08982 | 0.01759 |
| GO:0034284 | response to monosaccharide | 0.095238 | 0.01752 |
| GO:0042056 | chemoattractant activity | 0.115942 | 0.01742 |
| GO:0001848 | complement binding | 0.15625 | 0.01742 |
| GO:0005164 | tumor necrosis factor receptor binding | 0.15625 | 0.01742 |
| GO:0017169 | CDP-alcohol phosphatidyltransferase activity | 0.15625 | 0.01742 |
| GO:0051926 | negative regulation of calcium ion transport | 0.125 | 0.01737 |
| GO:0051897 | positive regulation of protein kinase B signaling | 0.105769 | 0.01737 |
| GO:0051963 | regulation of synapse assembly | 0.105769 | 0.01737 |
| GO:0002761 | regulation of myeloid leukocyte differentiation | 0.098361 | 0.01706 |
| GO:1900407 | regulation of cellular response to oxidative stress | 0.109756 | 0.01686 |
| GO:0046683 | response to organophosphorus | 0.096552 | 0.01683 |
| GO:1902850 | microtubule cytoskeleton organization involved in mitosis | 0.096552 | 0.01683 |
| GO:2000311 | regulation of AMPA receptor activity | 0.190476 | 0.01653 |
| GO:0009595 | detection of biotic stimulus | 0.190476 | 0.01653 |
| GO:0007076 | mitotic chromosome condensation | 0.190476 | 0.01653 |
| GO:0090312 | positive regulation of protein deacetylation | 0.190476 | 0.01653 |
| GO:0065005 | protein-lipid complex assembly | 0.190476 | 0.01653 |
| GO:0034620 | cellular response to unfolded protein | 0.190476 | 0.01653 |
| GO:0034385 | triglyceride-rich plasma lipoprotein particle | 0.190476 | 0.01653 |
| GO:0034361 | very-low-density lipoprotein particle | 0.190476 | 0.01653 |
| GO:0004089 | carbonate dehydratase activity | 0.190476 | 0.01653 |
| GO:0006909 | phagocytosis | 0.099174 | 0.01633 |
| GO:0034754 | cellular hormone metabolic process | 0.099174 | 0.01633 |
| GO:0019001 | guanyl nucleotide binding | 0.068807 | 0.01632 |
| GO:0007159 | leukocyte cell-cell adhesion | 0.139535 | 0.01625 |
| GO:0009225 | nucleotide-sugar metabolic process | 0.139535 | 0.01625 |
| GO:0070207 | protein homotrimerization | 0.139535 | 0.01625 |
| GO:0046485 | ether lipid metabolic process | 0.139535 | 0.01625 |
| GO:0140110 | transcription regulator activity | 0.060175 | 0.01618 |
| GO:0006935 | chemotaxis | 0.075521 | 0.01607 |
| GO:0042330 | taxis | 0.075521 | 0.01607 |
| GO:0002792 | negative regulation of peptide secretion | 0.117647 | 0.01606 |
| GO:0046164 | alcohol catabolic process | 0.117647 | 0.01606 |
| GO:0090278 | negative regulation of peptide hormone secretion | 0.117647 | 0.01606 |
| GO:0034605 | cellular response to heat | 0.117647 | 0.01606 |
| GO:0001649 | osteoblast differentiation | 0.117647 | 0.01606 |
| GO:0032561 | guanyl ribonucleotide binding | 0.069018 | 0.01605 |
| GO:0008092 | cytoskeletal protein binding | 0.061966 | 0.01586 |
| GO:0021782 | glial cell development | 0.127273 | 0.01584 |
| GO:1901264 | carbohydrate derivative transport | 0.127273 | 0.01584 |
| GO:0001216 | DNA-binding transcription activator activity | 0.073276 | 0.01574 |
| GO:0042060 | wound healing | 0.111111 | 0.01566 |
| GO:0048771 | tissue remodeling | 0.111111 | 0.01566 |
| GO:0016835 | carbon-oxygen lyase activity | 0.1 | 0.01564 |
| GO:0001228 | DNA-binding transcription activator activity, RNA polymerase II-specific | 0.073593 | 0.01543 |
| GO:0001763 | morphogenesis of a branching structure | 0.095588 | 0.01540 |
| GO:0140375 | immune receptor activity | 0.095588 | 0.01540 |
| GO:0046469 | platelet activating factor metabolic process | 0.16129 | 0.01529 |
| GO:0010830 | regulation of myotube differentiation | 0.16129 | 0.01529 |
| GO:0051899 | membrane depolarization | 0.16129 | 0.01529 |
| GO:0009886 | post-embryonic animal morphogenesis | 0.16129 | 0.01529 |
| GO:1903715 | regulation of aerobic respiration | 0.16129 | 0.01529 |
| GO:0032232 | negative regulation of actin filament bundle assembly | 0.16129 | 0.01529 |
| GO:0006949 | syncytium formation | 0.16129 | 0.01529 |
| GO:0004112 | cyclic-nucleotide phosphodiesterase activity | 0.16129 | 0.01529 |
| GO:0014074 | response to purine-containing compound | 0.092025 | 0.01528 |
| GO:0048638 | regulation of developmental growth | 0.078488 | 0.01516 |
| GO:0120032 | regulation of plasma membrane bounded cell projection assembly | 0.089947 | 0.01501 |
| GO:0042470 | melanosome | 0.10084 | 0.01500 |
| GO:0048770 | pigment granule | 0.10084 | 0.01500 |
| GO:0060294 | cilium movement involved in cell motility | 0.106383 | 0.01491 |
| GO:0045785 | positive regulation of cell adhesion | 0.07672 | 0.01483 |
| GO:0060191 | regulation of lipase activity | 0.119403 | 0.01477 |
| GO:0062197 | cellular response to chemical stress | 0.083682 | 0.01472 |
| GO:0043280 | positive regulation of cysteine-type endopeptidase activity involved in apoptotic process | 0.096296 | 0.01467 |
| GO:0001843 | neural tube closure | 0.1125 | 0.01452 |
| GO:0051726 | regulation of cell cycle | 0.064014 | 0.01446 |
| GO:0048015 | phosphatidylinositol-mediated signaling | 0.12963 | 0.01440 |
| GO:0015629 | actin cytoskeleton | 0.084034 | 0.01433 |
| GO:0030002 | cellular anion homeostasis | 0.2 | 0.01390 |
| GO:0010766 | negative regulation of sodium ion transport | 0.2 | 0.01390 |
| GO:1901223 | negative regulation of NIK/NF-kappaB signaling | 0.2 | 0.01390 |
| GO:0005901 | caveola | 0.107527 | 0.01390 |
| GO:0098852 | lytic vacuole membrane | 0.086957 | 0.01384 |
| GO:1905244 | regulation of modification of synaptic structure | 0.272727 | 0.01368 |
| GO:0051764 | actin crosslink formation | 0.272727 | 0.01368 |
| GO:0072376 | protein activation cascade | 0.272727 | 0.01368 |
| GO:0006030 | chitin metabolic process | 0.272727 | 0.01368 |
| GO:0006740 | NADPH regeneration | 0.272727 | 0.01368 |
| GO:0034145 | positive regulation of toll-like receptor 4 signaling pathway | 0.272727 | 0.01368 |
| GO:0098868 | bone growth | 0.272727 | 0.01368 |
| GO:0035338 | long-chain fatty-acyl-CoA biosynthetic process | 0.272727 | 0.01368 |
| GO:0071280 | cellular response to copper ion | 0.272727 | 0.01368 |
| GO:0006098 | pentose-phosphate shunt | 0.272727 | 0.01368 |
| GO:0009133 | nucleoside diphosphate biosynthetic process | 0.272727 | 0.01368 |
| GO:1904776 | regulation of protein localization to cell cortex | 0.272727 | 0.01368 |
| GO:0014889 | muscle atrophy | 0.272727 | 0.01368 |
| GO:0099550 | trans-synaptic signaling, modulating synaptic transmission | 0.272727 | 0.01368 |
| GO:0097205 | renal filtration | 0.272727 | 0.01368 |
| GO:1900225 | regulation of NLRP3 inflammasome complex assembly | 0.272727 | 0.01368 |
| GO:0050930 | induction of positive chemotaxis | 0.272727 | 0.01368 |
| GO:0072711 | cellular response to hydroxyurea | 0.272727 | 0.01368 |
| GO:0052696 | flavonoid glucuronidation | 0.272727 | 0.01368 |
| GO:0048712 | negative regulation of astrocyte differentiation | 0.272727 | 0.01368 |
| GO:0008603 | cAMP-dependent protein kinase regulator activity | 0.272727 | 0.01368 |
| GO:0018455 | alcohol dehydrogenase [NAD(P)+] activity | 0.272727 | 0.01368 |
| GO:0039706 | co-receptor binding | 0.272727 | 0.01368 |
| GO:0098879 | structural constituent of postsynaptic specialization | 0.272727 | 0.01368 |
| GO:0098857 | membrane microdomain | 0.076355 | 0.01355 |
| GO:2000027 | regulation of animal organ morphogenesis | 0.113924 | 0.01344 |
| GO:0016485 | protein processing | 0.087379 | 0.01342 |
| GO:0051090 | regulation of DNA-binding transcription factor activity | 0.077135 | 0.01335 |
| GO:0006663 | platelet activating factor biosynthetic process | 0.166667 | 0.01334 |
| GO:0010614 | negative regulation of cardiac muscle hypertrophy | 0.166667 | 0.01334 |
| GO:0048240 | sperm capacitation | 0.166667 | 0.01334 |
| GO:0016878 | acid-thiol ligase activity | 0.166667 | 0.01334 |
| GO:0004114 | 3',5'-cyclic-nucleotide phosphodiesterase activity | 0.166667 | 0.01334 |
| GO:0042383 | sarcolemma | 0.103448 | 0.01333 |
| GO:0050778 | positive regulation of immune response | 0.069486 | 0.01309 |
| GO:0030315 | T-tubule | 0.132075 | 0.01306 |
| GO:0090207 | regulation of triglyceride metabolic process | 0.146341 | 0.01300 |
| GO:0031648 | protein destabilization | 0.146341 | 0.01300 |
| GO:0000132 | establishment of mitotic spindle orientation | 0.146341 | 0.01300 |
| GO:0032663 | regulation of interleukin-2 production | 0.146341 | 0.01300 |
| GO:0032330 | regulation of chondrocyte differentiation | 0.146341 | 0.01300 |
| GO:0045214 | sarcomere organization | 0.146341 | 0.01300 |
| GO:0051452 | intracellular pH reduction | 0.146341 | 0.01300 |
| GO:0005751 | mitochondrial respiratory chain complex IV | 0.146341 | 0.01300 |
| GO:0006975 | DNA damage induced protein phosphorylation | 0.5 | 0.01299 |
| GO:0043152 | induction of bacterial agglutination | 0.5 | 0.01299 |
| GO:0072014 | proximal tubule development | 0.5 | 0.01299 |
| GO:0072012 | glomerulus vasculature development | 0.5 | 0.01299 |
| GO:0009189 | deoxyribonucleoside diphosphate biosynthetic process | 0.5 | 0.01299 |
| GO:0097114 | NMDA glutamate receptor clustering | 0.5 | 0.01299 |
| GO:1903416 | response to glycoside | 0.5 | 0.01299 |
| GO:0072104 | glomerular capillary formation | 0.5 | 0.01299 |
| GO:0015917 | aminophospholipid transport | 0.5 | 0.01299 |
| GO:0010193 | response to ozone | 0.5 | 0.01299 |
| GO:2000427 | positive regulation of apoptotic cell clearance | 0.5 | 0.01299 |
| GO:0009609 | response to symbiotic bacterium | 0.5 | 0.01299 |
| GO:0009608 | response to symbiont | 0.5 | 0.01299 |
| GO:1902949 | positive regulation of tau-protein kinase activity | 0.5 | 0.01299 |
| GO:0035582 | sequestering of BMP in extracellular matrix | 0.5 | 0.01299 |
| GO:2000348 | regulation of CD40 signaling pathway | 0.5 | 0.01299 |
| GO:2000110 | negative regulation of macrophage apoptotic process | 0.5 | 0.01299 |
| GO:0061440 | kidney vasculature development | 0.5 | 0.01299 |
| GO:0003433 | chondrocyte development involved in endochondral bone morphogenesis | 0.5 | 0.01299 |
| GO:0042471 | ear morphogenesis | 0.5 | 0.01299 |
| GO:0034105 | positive regulation of tissue remodeling | 0.5 | 0.01299 |
| GO:1901256 | regulation of macrophage colony-stimulating factor production | 0.5 | 0.01299 |
| GO:0051964 | negative regulation of synapse assembly | 0.5 | 0.01299 |
| GO:0071461 | cellular response to redox state | 0.5 | 0.01299 |
| GO:0070318 | positive regulation of G0 to G1 transition | 0.5 | 0.01299 |
| GO:0034436 | glycoprotein transport | 0.5 | 0.01299 |
| GO:0008612 | peptidyl-lysine modification to peptidyl-hypusine | 0.5 | 0.01299 |
| GO:0000379 | tRNA-type intron splice site recognition and cleavage | 0.5 | 0.01299 |
| GO:0060018 | astrocyte fate commitment | 0.5 | 0.01299 |
| GO:0045636 | positive regulation of melanocyte differentiation | 0.5 | 0.01299 |
| GO:0097116 | gephyrin clustering involved in postsynaptic density assembly | 0.5 | 0.01299 |
| GO:0061437 | renal system vasculature development | 0.5 | 0.01299 |
| GO:0005610 | laminin-5 complex | 0.5 | 0.01299 |
| GO:0033063 | Rad51B-Rad51C-Rad51D-XRCC2 complex | 0.5 | 0.01299 |
| GO:0000293 | ferric-chelate reductase activity | 0.5 | 0.01299 |
| GO:0030984 | kininogen binding | 0.5 | 0.01299 |
| GO:0050309 | sugar-terminal-phosphatase activity | 0.5 | 0.01299 |
| GO:0031014 | troponin T binding | 0.5 | 0.01299 |
| GO:0086007 | voltage-gated calcium channel activity involved in cardiac muscle cell action potential | 0.5 | 0.01299 |
| GO:0031750 | D3 dopamine receptor binding | 0.5 | 0.01299 |
| GO:0000213 | tRNA-intron endonuclease activity | 0.5 | 0.01299 |
| GO:0034338 | short-chain carboxylesterase activity | 0.5 | 0.01299 |
| GO:0005030 | neurotrophin receptor activity | 0.5 | 0.01299 |
| GO:0004305 | ethanolamine kinase activity | 0.5 | 0.01299 |
| GO:0019115 | benzaldehyde dehydrogenase [NAD(P)+] activity | 0.5 | 0.01299 |
| GO:0004346 | glucose-6-phosphatase activity | 0.5 | 0.01299 |
| GO:0004366 | glycerol-3-phosphate O-acyltransferase activity | 0.5 | 0.01299 |
| GO:0102420 | sn-1-glycerol-3-phosphate C16:0-DCA-CoA acyl transferase activity | 0.5 | 0.01299 |
| GO:0034097 | response to cytokine | 0.064401 | 0.01295 |
| GO:0048732 | gland development | 0.081356 | 0.01292 |
| GO:1901888 | regulation of cell junction assembly | 0.090909 | 0.01279 |
| GO:0070382 | exocytic vesicle | 0.090909 | 0.01279 |
| GO:0050953 | sensory perception of light stimulus | 0.095541 | 0.01277 |
| GO:0005791 | rough endoplasmic reticulum | 0.098485 | 0.01272 |
| GO:0051087 | chaperone binding | 0.098485 | 0.01272 |
| GO:0099080 | supramolecular complex | 0.062816 | 0.01258 |
| GO:0001822 | kidney development | 0.096154 | 0.01245 |
| GO:0051015 | actin filament binding | 0.086207 | 0.01244 |
| GO:0007224 | smoothened signaling pathway | 0.115385 | 0.01243 |
| GO:1903321 | negative regulation of protein modification by small protein conjugation or removal | 0.105263 | 0.01242 |
| GO:0006869 | lipid transport | 0.082474 | 0.01221 |
| GO:0010977 | negative regulation of neuron projection development | 0.099237 | 0.01214 |
| GO:0019722 | calcium-mediated signaling | 0.099237 | 0.01214 |
| GO:0006885 | regulation of pH | 0.10989 | 0.01205 |
| GO:0110020 | regulation of actomyosin structure organization | 0.10989 | 0.01205 |
| GO:0005902 | microvillus | 0.10989 | 0.01205 |
| GO:0043065 | positive regulation of apoptotic process | 0.072072 | 0.01204 |
| GO:0050777 | negative regulation of immune response | 0.097403 | 0.01191 |
| GO:0001667 | ameboidal-type cell migration | 0.097403 | 0.01191 |
| GO:0031304 | intrinsic component of mitochondrial inner membrane | 0.134615 | 0.01182 |
| GO:1901265 | nucleoside phosphate binding | 0.056502 | 0.01178 |
| GO:0000166 | nucleotide binding | 0.056502 | 0.01178 |
| GO:0050730 | regulation of peptidyl-tyrosine phosphorylation | 0.087336 | 0.01176 |
| GO:0097305 | response to alcohol | 0.079096 | 0.01171 |
| GO:0051896 | regulation of protein kinase B signaling | 0.098039 | 0.01168 |
| GO:0002793 | positive regulation of peptide secretion | 0.107143 | 0.01167 |
| GO:0051282 | regulation of sequestering of calcium ion | 0.107143 | 0.01167 |
| GO:0030217 | T cell differentiation | 0.1 | 0.01160 |
| GO:0043500 | muscle adaptation | 0.172414 | 0.01156 |
| GO:0070328 | triglyceride homeostasis | 0.172414 | 0.01156 |
| GO:0055090 | acylglycerol homeostasis | 0.172414 | 0.01156 |
| GO:0140253 | cell-cell fusion | 0.172414 | 0.01156 |
| GO:0035967 | cellular response to topologically incorrect protein | 0.172414 | 0.01156 |
| GO:1902042 | negative regulation of extrinsic apoptotic signaling pathway via death domain receptors | 0.172414 | 0.01156 |
| GO:0042573 | retinoic acid metabolic process | 0.172414 | 0.01156 |
| GO:0000768 | syncytium formation by plasma membrane fusion | 0.172414 | 0.01156 |
| GO:0014904 | myotube cell development | 0.172414 | 0.01156 |
| GO:0071294 | cellular response to zinc ion | 0.172414 | 0.01156 |
| GO:0032965 | regulation of collagen biosynthetic process | 0.172414 | 0.01156 |
| GO:0007520 | myoblast fusion | 0.210526 | 0.01155 |
| GO:1900273 | positive regulation of long-term synaptic potentiation | 0.210526 | 0.01155 |
| GO:0048563 | post-embryonic animal organ morphogenesis | 0.210526 | 0.01155 |
| GO:0090322 | regulation of superoxide metabolic process | 0.210526 | 0.01155 |
| GO:1904036 | negative regulation of epithelial cell apoptotic process | 0.15 | 0.01155 |
| GO:0043618 | regulation of transcription from RNA polymerase II promoter in response to stress | 0.15 | 0.01155 |
| GO:0048662 | negative regulation of smooth muscle cell proliferation | 0.15 | 0.01155 |
| GO:0030641 | regulation of cellular pH | 0.116883 | 0.01147 |
| GO:0031345 | negative regulation of cell projection organization | 0.092486 | 0.01143 |
| GO:0070482 | response to oxygen levels | 0.077519 | 0.01143 |
| GO:0072503 | cellular divalent inorganic cation homeostasis | 0.09 | 0.01138 |
| GO:0032413 | negative regulation of ion transmembrane transporter activity | 0.125 | 0.01137 |
| GO:0030512 | negative regulation of transforming growth factor beta receptor signaling pathway | 0.125 | 0.01137 |
| GO:0045685 | regulation of glial cell differentiation | 0.125 | 0.01137 |
| GO:0015850 | organic hydroxy compound transport | 0.108108 | 0.01136 |
| GO:0090101 | negative regulation of transmembrane receptor protein serine/threonine kinase signaling pathway | 0.108108 | 0.01136 |
| GO:0048705 | skeletal system morphogenesis | 0.108108 | 0.01136 |
| GO:1905369 | endopeptidase complex | 0.111111 | 0.01119 |
| GO:0007034 | vacuolar transport | 0.090452 | 0.01112 |
| GO:0050921 | positive regulation of chemotaxis | 0.100775 | 0.01109 |
| GO:0016324 | apical plasma membrane | 0.078329 | 0.01098 |
| GO:0000977 | RNA polymerase II transcription regulatory region sequence-specific DNA binding | 0.065737 | 0.01095 |
| GO:0032553 | ribonucleotide binding | 0.057819 | 0.01079 |
| GO:0017076 | purine nucleotide binding | 0.057838 | 0.01077 |
| GO:0061035 | regulation of cartilage development | 0.137255 | 0.01066 |
| GO:0035418 | protein localization to synapse | 0.137255 | 0.01066 |
| GO:0005801 | cis-Golgi network | 0.137255 | 0.01066 |
| GO:0033116 | endoplasmic reticulum-Golgi intermediate compartment membrane | 0.137255 | 0.01066 |
| GO:0031305 | integral component of mitochondrial inner membrane | 0.137255 | 0.01066 |
| GO:0048514 | blood vessel morphogenesis | 0.118421 | 0.01057 |
| GO:0032555 | purine ribonucleotide binding | 0.05787 | 0.01056 |
| GO:1905368 | peptidase complex | 0.097222 | 0.01055 |
| GO:0008285 | negative regulation of cell population proliferation | 0.070968 | 0.01039 |
| GO:0046888 | negative regulation of hormone secretion | 0.11236 | 0.01038 |
| GO:0000315 | organellar large ribosomal subunit | 0.11236 | 0.01038 |
| GO:0005762 | mitochondrial large ribosomal subunit | 0.11236 | 0.01038 |
| GO:0008144 | obsolete drug binding | 0.11236 | 0.01038 |
| GO:0006487 | protein N-linked glycosylation | 0.126984 | 0.01037 |
| GO:0043292 | contractile fiber | 0.126984 | 0.01037 |
| GO:0016229 | steroid dehydrogenase activity | 0.126984 | 0.01037 |
| GO:0001941 | postsynaptic membrane organization | 0.3 | 0.01032 |
| GO:1905155 | positive regulation of membrane invagination | 0.3 | 0.01032 |
| GO:0060100 | positive regulation of phagocytosis, engulfment | 0.3 | 0.01032 |
| GO:0072132 | mesenchyme morphogenesis | 0.3 | 0.01032 |
| GO:0003094 | glomerular filtration | 0.3 | 0.01032 |
| GO:0006032 | chitin catabolic process | 0.3 | 0.01032 |
| GO:1900116 | extracellular negative regulation of signal transduction | 0.3 | 0.01032 |
| GO:1900115 | extracellular regulation of signal transduction | 0.3 | 0.01032 |
| GO:0006616 | SRP-dependent cotranslational protein targeting to membrane, translocation | 0.3 | 0.01032 |
| GO:0009642 | response to light intensity | 0.3 | 0.01032 |
| GO:0072178 | nephric duct morphogenesis | 0.3 | 0.01032 |
| GO:0072202 | cell differentiation involved in metanephros development | 0.3 | 0.01032 |
| GO:0044327 | dendritic spine head | 0.3 | 0.01032 |
| GO:0005787 | signal peptidase complex | 0.3 | 0.01032 |
| GO:0031313 | extrinsic component of endosome membrane | 0.3 | 0.01032 |
| GO:0043256 | laminin complex | 0.3 | 0.01032 |
| GO:0120103 | centriolar subdistal appendage | 0.3 | 0.01032 |
| GO:0097539 | ciliary transition fiber | 0.3 | 0.01032 |
| GO:0004022 | alcohol dehydrogenase (NAD+) activity | 0.3 | 0.01032 |
| GO:0004862 | cAMP-dependent protein kinase inhibitor activity | 0.3 | 0.01032 |
| GO:0047086 | ketosteroid monooxygenase activity | 0.3 | 0.01032 |
| GO:0008430 | selenium binding | 0.3 | 0.01032 |
| GO:0098803 | respiratory chain complex | 0.092308 | 0.01027 |
| GO:0002456 | T cell mediated immunity | 0.153846 | 0.01023 |
| GO:0120034 | positive regulation of plasma membrane bounded cell projection assembly | 0.107843 | 0.00997 |
| GO:0009636 | response to toxic substance | 0.107843 | 0.00997 |
| GO:0044770 | cell cycle phase transition | 0.094675 | 0.00996 |
| GO:0046464 | acylglycerol catabolic process | 0.178571 | 0.00996 |
| GO:0046461 | neutral lipid catabolic process | 0.178571 | 0.00996 |
| GO:1901020 | negative regulation of calcium ion transmembrane transporter activity | 0.178571 | 0.00996 |
| GO:0051968 | positive regulation of synaptic transmission, glutamatergic | 0.178571 | 0.00996 |
| GO:0050996 | positive regulation of lipid catabolic process | 0.178571 | 0.00996 |
| GO:0043367 | CD4-positive, alpha-beta T cell differentiation | 0.178571 | 0.00996 |
| GO:0002861 | regulation of inflammatory response to antigenic stimulus | 0.178571 | 0.00996 |
| GO:0030552 | cAMP binding | 0.178571 | 0.00996 |
| GO:0004142 | diacylglycerol cholinephosphotransferase activity | 0.178571 | 0.00996 |
| GO:0008219 | cell death | 0.065078 | 0.00981 |
| GO:0031253 | cell projection membrane | 0.080357 | 0.00972 |
| GO:0060562 | epithelial tube morphogenesis | 0.12 | 0.00972 |
| GO:0048545 | response to steroid hormone | 0.08642 | 0.00959 |
| GO:0060078 | regulation of postsynaptic membrane potential | 0.14 | 0.00958 |
| GO:0043068 | positive regulation of programmed cell death | 0.072695 | 0.00956 |
| GO:0002009 | morphogenesis of an epithelium | 0.084559 | 0.00949 |
| GO:0001892 | embryonic placenta development | 0.222222 | 0.00948 |
| GO:0019432 | triglyceride biosynthetic process | 0.222222 | 0.00948 |
| GO:2001026 | regulation of endothelial cell chemotaxis | 0.222222 | 0.00948 |
| GO:0106057 | negative regulation of calcineurin-mediated signaling | 0.222222 | 0.00948 |
| GO:0032703 | negative regulation of interleukin-2 production | 0.222222 | 0.00948 |
| GO:0043576 | regulation of respiratory gaseous exchange | 0.222222 | 0.00948 |
| GO:0070885 | negative regulation of calcineurin-NFAT signaling cascade | 0.222222 | 0.00948 |
| GO:0099144 | anchored component of synaptic membrane | 0.222222 | 0.00948 |
| GO:0036041 | long-chain fatty acid binding | 0.222222 | 0.00948 |
| GO:0048839 | inner ear development | 0.129032 | 0.00944 |
| GO:0010628 | positive regulation of gene expression | 0.063433 | 0.00941 |
| GO:0009408 | response to heat | 0.104 | 0.00935 |
| GO:0051117 | ATPase binding | 0.108911 | 0.00928 |
| GO:0046907 | intracellular transport | 0.06246 | 0.00926 |
| GO:0007015 | actin filament organization | 0.083333 | 0.00915 |
| GO:2001233 | regulation of apoptotic signaling pathway | 0.077694 | 0.00915 |
| GO:0006720 | isoprenoid metabolic process | 0.099291 | 0.00910 |
| GO:0090303 | positive regulation of wound healing | 0.157895 | 0.00901 |
| GO:2000351 | regulation of endothelial cell apoptotic process | 0.157895 | 0.00901 |
| GO:1901019 | regulation of calcium ion transmembrane transporter activity | 0.121622 | 0.00892 |
| GO:0015291 | secondary active transmembrane transporter activity | 0.085821 | 0.00890 |
| GO:0050848 | regulation of calcium-mediated signaling | 0.114943 | 0.00890 |
| GO:0048469 | cell maturation | 0.09697 | 0.00886 |
| GO:0043177 | organic acid binding | 0.09697 | 0.00886 |
| GO:0003964 | RNA-directed DNA polymerase activity | 0.1 | 0.00867 |
| GO:0034763 | negative regulation of transmembrane transport | 0.105691 | 0.00867 |
| GO:0098798 | mitochondrial protein-containing complex | 0.073126 | 0.00865 |
| GO:0048598 | embryonic morphogenesis | 0.07868 | 0.00859 |
| GO:0043113 | receptor clustering | 0.142857 | 0.00859 |
| GO:0004033 | aldo-keto reductase (NADP) activity | 0.142857 | 0.00859 |
| GO:0071634 | regulation of transforming growth factor beta production | 0.185185 | 0.00851 |
| GO:0001656 | metanephros development | 0.185185 | 0.00851 |
| GO:0031063 | regulation of histone deacetylation | 0.185185 | 0.00851 |
| GO:0070325 | lipoprotein particle receptor binding | 0.185185 | 0.00851 |
| GO:1901617 | organic hydroxy compound biosynthetic process | 0.090476 | 0.00849 |
| GO:0033218 | amide binding | 0.075594 | 0.00841 |
| GO:0034599 | cellular response to oxidative stress | 0.093407 | 0.00827 |
| GO:0006109 | regulation of carbohydrate metabolic process | 0.090909 | 0.00827 |
| GO:0003014 | renal system process | 0.116279 | 0.00822 |
| GO:0071277 | cellular response to calcium ion | 0.116279 | 0.00822 |
| GO:0071944 | cell periphery | 0.123288 | 0.00818 |
| GO:0009725 | response to hormone | 0.069191 | 0.00810 |
| GO:0015748 | organophosphate ester transport | 0.107438 | 0.00810 |
| GO:0043254 | regulation of protein-containing complex assembly | 0.078014 | 0.00804 |
| GO:0031348 | negative regulation of defense response | 0.091787 | 0.00788 |
| GO:0030177 | positive regulation of Wnt signaling pathway | 0.108333 | 0.00785 |
| GO:0035458 | cellular response to interferon-beta | 0.133333 | 0.00777 |
| GO:0070293 | renal absorption | 0.235294 | 0.00766 |
| GO:0034377 | plasma lipoprotein particle assembly | 0.235294 | 0.00766 |
| GO:2000050 | regulation of non-canonical Wnt signaling pathway | 0.235294 | 0.00766 |
| GO:0019433 | triglyceride catabolic process | 0.235294 | 0.00766 |
| GO:0031065 | positive regulation of histone deacetylation | 0.235294 | 0.00766 |
| GO:0061005 | cell differentiation involved in kidney development | 0.235294 | 0.00766 |
| GO:0005344 | oxygen carrier activity | 0.235294 | 0.00766 |
| GO:0070492 | oligosaccharide binding | 0.235294 | 0.00766 |
| GO:0009719 | response to endogenous stimulus | 0.064567 | 0.00759 |
| GO:0006977 | DNA damage response, signal transduction by p53 class mediator resulting in cell cycle arrest | 0.333333 | 0.00749 |
| GO:1904729 | regulation of intestinal lipid absorption | 0.333333 | 0.00749 |
| GO:0061140 | lung secretory cell differentiation | 0.333333 | 0.00749 |
| GO:1905048 | regulation of metallopeptidase activity | 0.333333 | 0.00749 |
| GO:0002369 | T cell cytokine production | 0.333333 | 0.00749 |
| GO:0034370 | triglyceride-rich lipoprotein particle remodeling | 0.333333 | 0.00749 |
| GO:0060541 | respiratory system development | 0.333333 | 0.00749 |
| GO:0009313 | oligosaccharide catabolic process | 0.333333 | 0.00749 |
| GO:1990454 | L-type voltage-gated calcium channel complex | 0.333333 | 0.00749 |
| GO:0030492 | hemoglobin binding | 0.333333 | 0.00749 |
| GO:0098919 | structural constituent of postsynaptic density | 0.333333 | 0.00749 |
| GO:0051279 | regulation of release of sequestered calcium ion into cytosol | 0.125 | 0.00748 |
| GO:0035456 | response to interferon-beta | 0.125 | 0.00748 |
| GO:0051453 | regulation of intracellular pH | 0.125 | 0.00748 |
| GO:0044419 | biological process involved in interspecies interaction between organisms | 0.061299 | 0.00748 |
| GO:0019218 | regulation of steroid metabolic process | 0.112245 | 0.00745 |
| GO:0043087 | regulation of GTPase activity | 0.079365 | 0.00742 |
| GO:0006796 | phosphate-containing compound metabolic process | 0.059738 | 0.00736 |
| GO:0000978 | RNA polymerase II cis-regulatory region sequence-specific DNA binding | 0.069799 | 0.00728 |
| GO:0008022 | protein C-terminus binding | 0.086957 | 0.00726 |
| GO:0002253 | activation of immune response | 0.078049 | 0.00726 |
| GO:1902532 | negative regulation of intracellular signal transduction | 0.074427 | 0.00725 |
| GO:0004713 | protein tyrosine kinase activity | 0.098684 | 0.00723 |
| GO:0048741 | skeletal muscle fiber development | 0.192308 | 0.00721 |
| GO:0006026 | aminoglycan catabolic process | 0.192308 | 0.00721 |
| GO:0035904 | aorta development | 0.192308 | 0.00721 |
| GO:0050766 | positive regulation of phagocytosis | 0.135593 | 0.00702 |
| GO:0009894 | regulation of catabolic process | 0.067511 | 0.00702 |
| GO:0016101 | diterpenoid metabolic process | 0.119048 | 0.00698 |
| GO:0055001 | muscle cell development | 0.113402 | 0.00691 |
| GO:0032231 | regulation of actin filament bundle assembly | 0.113402 | 0.00691 |
| GO:0070069 | cytochrome complex | 0.113402 | 0.00691 |
| GO:0045744 | negative regulation of G protein-coupled receptor signaling pathway | 0.166667 | 0.00690 |
| GO:0070888 | E-box binding | 0.166667 | 0.00690 |
| GO:0051489 | regulation of filopodium assembly | 0.148936 | 0.00683 |
| GO:0046503 | glycerolipid catabolic process | 0.148936 | 0.00683 |
| GO:0031526 | brush border membrane | 0.126761 | 0.00683 |
| GO:0016667 | oxidoreductase activity, acting on a sulfur group of donors | 0.126761 | 0.00683 |
| GO:0005575 | cellular_component | 0.049053 | 0.00680 |
| GO:0042113 | B cell activation | 0.092784 | 0.00677 |
| GO:0070374 | positive regulation of ERK1 and ERK2 cascade | 0.092784 | 0.00677 |
| GO:0043410 | positive regulation of MAPK cascade | 0.079012 | 0.00674 |
| GO:0010901 | regulation of very-low-density lipoprotein particle remodeling | 0.666667 | 0.00671 |
| GO:0072010 | glomerular epithelium development | 0.666667 | 0.00671 |
| GO:0072011 | glomerular endothelium development | 0.666667 | 0.00671 |
| GO:2000121 | regulation of removal of superoxide radicals | 0.666667 | 0.00671 |
| GO:0070447 | positive regulation of oligodendrocyte progenitor proliferation | 0.666667 | 0.00671 |
| GO:0051124 | synaptic assembly at neuromuscular junction | 0.666667 | 0.00671 |
| GO:0016128 | phytosteroid metabolic process | 0.666667 | 0.00671 |
| GO:0016129 | phytosteroid biosynthetic process | 0.666667 | 0.00671 |
| GO:0089700 | protein kinase D signaling | 0.666667 | 0.00671 |
| GO:0006982 | response to lipid hydroperoxide | 0.666667 | 0.00671 |
| GO:2000843 | regulation of testosterone secretion | 0.666667 | 0.00671 |
| GO:0071603 | endothelial cell-cell adhesion | 0.666667 | 0.00671 |
| GO:0090045 | positive regulation of deacetylase activity | 0.666667 | 0.00671 |
| GO:0019628 | urate catabolic process | 0.666667 | 0.00671 |
| GO:0000821 | regulation of arginine metabolic process | 0.666667 | 0.00671 |
| GO:0044028 | DNA hypomethylation | 0.666667 | 0.00671 |
| GO:0044029 | hypomethylation of CpG island | 0.666667 | 0.00671 |
| GO:0072111 | cell proliferation involved in kidney development | 0.666667 | 0.00671 |
| GO:0043323 | positive regulation of natural killer cell degranulation | 0.666667 | 0.00671 |
| GO:0043321 | regulation of natural killer cell degranulation | 0.666667 | 0.00671 |
| GO:0010273 | detoxification of copper ion | 0.666667 | 0.00671 |
| GO:0002232 | leukocyte chemotaxis involved in inflammatory response | 0.666667 | 0.00671 |
| GO:0061577 | calcium ion transmembrane transport via high voltage-gated calcium channel | 0.666667 | 0.00671 |
| GO:0042567 | insulin-like growth factor ternary complex | 0.666667 | 0.00671 |
| GO:0036502 | Derlin-1-VIMP complex | 0.666667 | 0.00671 |
| GO:1990584 | cardiac Troponin complex | 0.666667 | 0.00671 |
| GO:0097059 | CNTFR-CLCF1 complex | 0.666667 | 0.00671 |
| GO:0071953 | elastic fiber | 0.666667 | 0.00671 |
| GO:0035976 | transcription factor AP-1 complex | 0.666667 | 0.00671 |
| GO:0043259 | laminin-10 complex | 0.666667 | 0.00671 |
| GO:0019862 | IgA binding | 0.666667 | 0.00671 |
| GO:0043120 | tumor necrosis factor binding | 0.666667 | 0.00671 |
| GO:0016941 | natriuretic peptide receptor activity | 0.666667 | 0.00671 |
| GO:0003960 | NADPH:quinone reductase activity | 0.666667 | 0.00671 |
| GO:0004699 | calcium-independent protein kinase C activity | 0.666667 | 0.00671 |
| GO:0045934 | negative regulation of nucleobase-containing compound metabolic process | 0.062391 | 0.00668 |
| GO:0046578 | regulation of Ras protein signal transduction | 0.088353 | 0.00659 |
| GO:0120254 | olefinic compound metabolic process | 0.1 | 0.00653 |
| GO:0019955 | cytokine binding | 0.1 | 0.00653 |
| GO:0017124 | SH3 domain binding | 0.1 | 0.00653 |
| GO:0040011 | locomotion | 0.079602 | 0.00651 |
| GO:0050769 | positive regulation of neurogenesis | 0.089069 | 0.00632 |
| GO:0005773 | vacuole | 0.078341 | 0.00630 |
| GO:0060627 | regulation of vesicle-mediated transport | 0.074818 | 0.00628 |
| GO:0055080 | cation homeostasis | 0.076759 | 0.00627 |
| GO:1904035 | regulation of epithelial cell apoptotic process | 0.128571 | 0.00622 |
| GO:0032507 | maintenance of protein location in cell | 0.128571 | 0.00622 |
| GO:0042048 | olfactory behavior | 0.25 | 0.00609 |
| GO:0005978 | glycogen biosynthetic process | 0.25 | 0.00609 |
| GO:1903975 | regulation of glial cell migration | 0.25 | 0.00609 |
| GO:0009250 | glucan biosynthetic process | 0.25 | 0.00609 |
| GO:0050849 | negative regulation of calcium-mediated signaling | 0.2 | 0.00606 |
| GO:0042698 | ovulation cycle | 0.2 | 0.00606 |
| GO:0099524 | postsynaptic cytosol | 0.2 | 0.00606 |
| GO:0045428 | regulation of nitric oxide biosynthetic process | 0.152174 | 0.00606 |
| GO:0034764 | positive regulation of transmembrane transport | 0.091743 | 0.00601 |
| GO:0060491 | regulation of cell projection assembly | 0.094241 | 0.00599 |
| GO:0044262 | cellular carbohydrate metabolic process | 0.094241 | 0.00599 |
| GO:0046839 | phospholipid dephosphorylation | 0.171429 | 0.00599 |
| GO:0099522 | cytosolic region | 0.171429 | 0.00599 |
| GO:0019216 | regulation of lipid metabolic process | 0.082822 | 0.00595 |
| GO:0002376 | immune system process | 0.060075 | 0.00591 |
| GO:0071806 | protein transmembrane transport | 0.121951 | 0.00590 |
| GO:0065002 | intracellular protein transmembrane transport | 0.121951 | 0.00590 |
| GO:0033293 | monocarboxylic acid binding | 0.121951 | 0.00590 |
| GO:0035148 | tube formation | 0.10687 | 0.00585 |
| GO:0030595 | leukocyte chemotaxis | 0.10687 | 0.00585 |
| GO:0001501 | skeletal system development | 0.10687 | 0.00585 |
| GO:0042826 | histone deacetylase binding | 0.10687 | 0.00585 |
| GO:0110053 | regulation of actin filament organization | 0.086792 | 0.00585 |
| GO:0090257 | regulation of muscle system process | 0.092593 | 0.00570 |
| GO:1905897 | regulation of response to endoplasmic reticulum stress | 0.140351 | 0.00569 |
| GO:0043198 | dendritic shaft | 0.140351 | 0.00569 |
| GO:0005543 | phospholipid binding | 0.076613 | 0.00566 |
| GO:0010494 | cytoplasmic stress granule | 0.107692 | 0.00564 |
| GO:0046903 | secretion | 0.076768 | 0.00561 |
| GO:0043281 | regulation of cysteine-type endopeptidase activity involved in apoptotic process | 0.093023 | 0.00557 |
| GO:0051707 | response to other organism | 0.062325 | 0.00551 |
| GO:0006665 | sphingolipid metabolic process | 0.108527 | 0.00546 |
| GO:0002684 | positive regulation of immune system process | 0.067404 | 0.00545 |
| GO:0031400 | negative regulation of protein modification process | 0.075758 | 0.00536 |
| GO:0000041 | transition metal ion transport | 0.10274 | 0.00536 |
| GO:0002920 | regulation of humoral immune response | 0.155556 | 0.00536 |
| GO:0043620 | regulation of DNA-templated transcription in response to stress | 0.155556 | 0.00536 |
| GO:0031267 | small GTPase binding | 0.079518 | 0.00523 |
| GO:2001198 | regulation of dendritic cell differentiation | 0.375 | 0.00518 |
| GO:0051917 | regulation of fibrinolysis | 0.375 | 0.00518 |
| GO:0030300 | regulation of intestinal cholesterol absorption | 0.375 | 0.00518 |
| GO:0048251 | elastic fiber assembly | 0.375 | 0.00518 |
| GO:0032835 | glomerulus development | 0.375 | 0.00518 |
| GO:1905809 | negative regulation of synapse organization | 0.375 | 0.00518 |
| GO:0034372 | very-low-density lipoprotein particle remodeling | 0.375 | 0.00518 |
| GO:0010832 | negative regulation of myotube differentiation | 0.375 | 0.00518 |
| GO:1901678 | iron coordination entity transport | 0.375 | 0.00518 |
| GO:0072718 | response to cisplatin | 0.375 | 0.00518 |
| GO:0021957 | corticospinal tract morphogenesis | 0.375 | 0.00518 |
| GO:0004568 | chitinase activity | 0.375 | 0.00518 |
| GO:0042609 | CD4 receptor binding | 0.375 | 0.00518 |
| GO:0010712 | regulation of collagen metabolic process | 0.176471 | 0.00517 |
| GO:0030134 | COPII-coated ER to Golgi transport vesicle | 0.176471 | 0.00517 |
| GO:0043679 | axon terminus | 0.110236 | 0.00514 |
| GO:0035869 | ciliary transition zone | 0.132353 | 0.00513 |
| GO:0070330 | aromatase activity | 0.142857 | 0.00510 |
| GO:0006613 | cotranslational protein targeting to membrane | 0.208333 | 0.00505 |
| GO:0005797 | Golgi medial cisterna | 0.208333 | 0.00505 |
| GO:0004745 | NAD-retinol dehydrogenase activity | 0.208333 | 0.00505 |
| GO:0001846 | opsonin binding | 0.208333 | 0.00505 |
| GO:0005887 | integral component of plasma membrane | 0.064252 | 0.00503 |
| GO:0099175 | regulation of postsynapse organization | 0.11828 | 0.00503 |
| GO:0000139 | Golgi membrane | 0.075404 | 0.00493 |
| GO:0033043 | regulation of organelle organization | 0.065888 | 0.00491 |
| GO:0031974 | membrane-enclosed lumen | 0.069939 | 0.00483 |
| GO:0043233 | organelle lumen | 0.069939 | 0.00483 |
| GO:0070013 | intracellular organelle lumen | 0.070025 | 0.00479 |
| GO:0045019 | negative regulation of nitric oxide biosynthetic process | 0.266667 | 0.00474 |
| GO:0009812 | flavonoid metabolic process | 0.266667 | 0.00474 |
| GO:0002862 | negative regulation of inflammatory response to antigenic stimulus | 0.266667 | 0.00474 |
| GO:0060317 | cardiac epithelial to mesenchymal transition | 0.266667 | 0.00474 |
| GO:0099084 | postsynaptic specialization organization | 0.266667 | 0.00474 |
| GO:1904406 | negative regulation of nitric oxide metabolic process | 0.266667 | 0.00474 |
| GO:0005614 | interstitial matrix | 0.266667 | 0.00474 |
| GO:0072562 | blood microparticle | 0.266667 | 0.00474 |
| GO:0008654 | phospholipid biosynthetic process | 0.089494 | 0.00473 |
| GO:0042743 | hydrogen peroxide metabolic process | 0.159091 | 0.00471 |
| GO:0048471 | perinuclear region of cytoplasm | 0.069767 | 0.00464 |
| GO:0006612 | protein targeting to membrane | 0.119565 | 0.00463 |
| GO:0080135 | regulation of cellular response to stress | 0.073365 | 0.00463 |
| GO:0035914 | skeletal muscle cell differentiation | 0.145455 | 0.00456 |
| GO:0061515 | myeloid cell development | 0.145455 | 0.00456 |
| GO:0099738 | cell cortex region | 0.145455 | 0.00456 |
| GO:0042277 | peptide binding | 0.081744 | 0.00456 |
| GO:0001523 | retinoid metabolic process | 0.126582 | 0.00452 |
| GO:0030175 | filopodium | 0.126582 | 0.00452 |
| GO:0015370 | solute:sodium symporter activity | 0.126582 | 0.00452 |
| GO:0005178 | integrin binding | 0.105634 | 0.00446 |
| GO:0044458 | motile cilium assembly | 0.181818 | 0.00443 |
| GO:0043235 | receptor complex | 0.082418 | 0.00428 |
| GO:0031397 | negative regulation of protein ubiquitination | 0.115385 | 0.00426 |
| GO:0048661 | positive regulation of smooth muscle cell proliferation | 0.120879 | 0.00426 |
| GO:0034766 | negative regulation of ion transmembrane transport | 0.120879 | 0.00426 |
| GO:0019199 | transmembrane receptor protein kinase activity | 0.120879 | 0.00426 |
| GO:0052689 | carboxylic ester hydrolase activity | 0.095477 | 0.00417 |
| GO:0010224 | response to UV-B | 0.217391 | 0.00416 |
| GO:0006614 | SRP-dependent cotranslational protein targeting to membrane | 0.217391 | 0.00416 |
| GO:2000251 | positive regulation of actin cytoskeleton reorganization | 0.217391 | 0.00416 |
| GO:2000352 | negative regulation of endothelial cell apoptotic process | 0.217391 | 0.00416 |
| GO:0032426 | stereocilium tip | 0.217391 | 0.00416 |
| GO:0005385 | zinc ion transmembrane transporter activity | 0.217391 | 0.00416 |
| GO:0016772 | transferase activity, transferring phosphorus-containing groups | 0.064094 | 0.00415 |
| GO:0030285 | integral component of synaptic vesicle membrane | 0.162791 | 0.00413 |
| GO:1990748 | cellular detoxification | 0.148148 | 0.00407 |
| GO:1902903 | regulation of supramolecular fiber organization | 0.083565 | 0.00393 |
| GO:0009612 | response to mechanical stimulus | 0.096447 | 0.00385 |
| GO:0000323 | lytic vacuole | 0.084034 | 0.00382 |
| GO:0005764 | lysosome | 0.084034 | 0.00382 |
| GO:0005769 | early endosome | 0.085627 | 0.00381 |
| GO:0019900 | kinase binding | 0.070115 | 0.00379 |
| GO:0014741 | negative regulation of muscle hypertrophy | 0.1875 | 0.00378 |
| GO:0040018 | positive regulation of multicellular organism growth | 0.1875 | 0.00378 |
| GO:0035710 | CD4-positive, alpha-beta T cell activation | 0.1875 | 0.00378 |
| GO:0097190 | apoptotic signaling pathway | 0.089219 | 0.00374 |
| GO:0003700 | DNA-binding transcription factor activity | 0.065393 | 0.00368 |
| GO:0006793 | phosphorus metabolic process | 0.060606 | 0.00365 |
| GO:0045661 | regulation of myoblast differentiation | 0.150943 | 0.00362 |
| GO:0061512 | protein localization to cilium | 0.150943 | 0.00362 |
| GO:0090129 | positive regulation of synapse maturation | 0.285714 | 0.00361 |
| GO:0030948 | negative regulation of vascular endothelial growth factor receptor signaling pathway | 0.285714 | 0.00361 |
| GO:0003414 | chondrocyte morphogenesis involved in endochondral bone morphogenesis | 0.285714 | 0.00361 |
| GO:0000002 | mitochondrial genome maintenance | 0.285714 | 0.00361 |
| GO:0003422 | growth plate cartilage morphogenesis | 0.285714 | 0.00361 |
| GO:1905153 | regulation of membrane invagination | 0.285714 | 0.00361 |
| GO:0003429 | growth plate cartilage chondrocyte morphogenesis | 0.285714 | 0.00361 |
| GO:0090171 | chondrocyte morphogenesis | 0.285714 | 0.00361 |
| GO:0005833 | hemoglobin complex | 0.285714 | 0.00361 |
| GO:0002252 | immune effector process | 0.078512 | 0.00361 |
| GO:0032391 | photoreceptor connecting cilium | 0.166667 | 0.00361 |
| GO:0016712 | oxidoreductase activity, acting on paired donors, with incorporation or reduction of molecular oxygen, reduced flavin or flavoprotein as one donor, and incorporation of one atom of oxygen | 0.113043 | 0.00359 |
| GO:0061178 | regulation of insulin secretion involved in cellular response to glucose stimulus | 0.123596 | 0.00359 |
| GO:0008395 | steroid hydroxylase activity | 0.123596 | 0.00359 |
| GO:0060249 | anatomical structure homeostasis | 0.097436 | 0.00357 |
| GO:0045177 | apical part of cell | 0.110294 | 0.00356 |
| GO:0010611 | regulation of cardiac muscle hypertrophy | 0.140625 | 0.00340 |
| GO:0046463 | acylglycerol biosynthetic process | 0.227273 | 0.00339 |
| GO:0046460 | neutral lipid biosynthetic process | 0.227273 | 0.00339 |
| GO:0019934 | cGMP-mediated signaling | 0.227273 | 0.00339 |
| GO:0045686 | negative regulation of glial cell differentiation | 0.227273 | 0.00339 |
| GO:0018196 | peptidyl-asparagine modification | 0.227273 | 0.00339 |
| GO:0010749 | regulation of nitric oxide mediated signal transduction | 0.428571 | 0.00335 |
| GO:0006198 | cAMP catabolic process | 0.428571 | 0.00335 |
| GO:0044691 | tooth eruption | 0.428571 | 0.00335 |
| GO:0006069 | ethanol oxidation | 0.428571 | 0.00335 |
| GO:0150094 | amyloid-beta clearance by cellular catabolic process | 0.428571 | 0.00335 |
| GO:0005785 | signal recognition particle receptor complex | 0.428571 | 0.00335 |
| GO:0004024 | alcohol dehydrogenase activity, zinc-dependent | 0.428571 | 0.00335 |
| GO:0048406 | nerve growth factor binding | 0.428571 | 0.00335 |
| GO:1901222 | regulation of NIK/NF-kappaB signaling | 0.118812 | 0.00335 |
| GO:0050728 | negative regulation of inflammatory response | 0.114035 | 0.00333 |
| GO:0010976 | positive regulation of neuron projection development | 0.098958 | 0.00322 |
| GO:0071949 | FAD binding | 0.153846 | 0.00320 |
| GO:0001658 | branching involved in ureteric bud morphogenesis | 0.193548 | 0.00320 |
| GO:0030316 | osteoclast differentiation | 0.193548 | 0.00320 |
| GO:0060674 | placenta blood vessel development | 0.193548 | 0.00320 |
| GO:0045834 | positive regulation of lipid metabolic process | 0.10596 | 0.00316 |
| GO:0002237 | response to molecule of bacterial origin | 0.08046 | 0.00314 |
| GO:0014059 | regulation of dopamine secretion | 0.170732 | 0.00313 |
| GO:0015980 | energy derivation by oxidation of organic compounds | 0.088652 | 0.00310 |
| GO:0006721 | terpenoid metabolic process | 0.12 | 0.00309 |
| GO:0005768 | endosome | 0.072874 | 0.00306 |
| GO:0070613 | regulation of protein processing | 0.142857 | 0.00305 |
| GO:0030308 | negative regulation of cell growth | 0.1 | 0.00303 |
| GO:0055114 | obsolete oxidation-reduction process | 0.106667 | 0.00302 |
| GO:0050801 | ion homeostasis | 0.078471 | 0.00293 |
| GO:0032940 | secretion by cell | 0.084469 | 0.00290 |
| GO:0060828 | regulation of canonical Wnt signaling pathway | 0.096618 | 0.00290 |
| GO:0071695 | anatomical structure maturation | 0.101064 | 0.00287 |
| GO:0010558 | negative regulation of macromolecule biosynthetic process | 0.062933 | 0.00286 |
| GO:0033500 | carbohydrate homeostasis | 0.094828 | 0.00284 |
| GO:0034614 | cellular response to reactive oxygen species | 0.121212 | 0.00284 |
| GO:0043542 | endothelial cell migration | 0.156863 | 0.00283 |
| GO:0048678 | response to axon injury | 0.156863 | 0.00283 |
| GO:0098858 | actin-based cell projection | 0.101604 | 0.00280 |
| GO:0030148 | sphingolipid biosynthetic process | 0.135135 | 0.00280 |
| GO:0008360 | regulation of cell shape | 0.108108 | 0.00278 |
| GO:0042593 | glucose homeostasis | 0.095238 | 0.00277 |
| GO:0031327 | negative regulation of cellular biosynthetic process | 0.062951 | 0.00275 |
| GO:0022900 | electron transport chain | 0.102151 | 0.00274 |
| GO:0070972 | protein localization to endoplasmic reticulum | 0.145161 | 0.00273 |
| GO:0071577 | zinc ion transmembrane transport | 0.238095 | 0.00272 |
| GO:0035634 | response to stilbenoid | 0.238095 | 0.00272 |
| GO:0010714 | positive regulation of collagen metabolic process | 0.238095 | 0.00272 |
| GO:0046058 | cAMP metabolic process | 0.238095 | 0.00272 |
| GO:0018279 | protein N-linked glycosylation via asparagine | 0.238095 | 0.00272 |
| GO:0045723 | positive regulation of fatty acid biosynthetic process | 0.238095 | 0.00272 |
| GO:0050926 | regulation of positive chemotaxis | 0.238095 | 0.00272 |
| GO:0060716 | labyrinthine layer blood vessel development | 0.238095 | 0.00272 |
| GO:0097384 | cellular lipid biosynthetic process | 0.175 | 0.00271 |
| GO:0140694 | non-membrane-bounded organelle assembly | 0.095652 | 0.00271 |
| GO:0051962 | positive regulation of nervous system development | 0.088525 | 0.00269 |
| GO:0036464 | cytoplasmic ribonucleoprotein granule | 0.088525 | 0.00269 |
| GO:0050872 | white fat cell differentiation | 0.307692 | 0.00268 |
| GO:0033700 | phospholipid efflux | 0.307692 | 0.00268 |
| GO:0031639 | plasminogen activation | 0.307692 | 0.00268 |
| GO:0042834 | peptidoglycan binding | 0.307692 | 0.00268 |
| GO:0050920 | regulation of chemotaxis | 0.097561 | 0.00267 |
| GO:0002443 | leukocyte mediated immunity | 0.122449 | 0.00260 |
| GO:0045892 | negative regulation of DNA-templated transcription | 0.065369 | 0.00259 |
| GO:0048704 | embryonic skeletal system morphogenesis | 0.136986 | 0.00252 |
| GO:0030672 | synaptic vesicle membrane | 0.136986 | 0.00252 |
| GO:0099501 | exocytic vesicle membrane | 0.136986 | 0.00252 |
| GO:0036293 | response to decreased oxygen levels | 0.086687 | 0.00250 |
| GO:0050764 | regulation of phagocytosis | 0.129412 | 0.00249 |
| GO:0044306 | neuron projection terminus | 0.110345 | 0.00248 |
| GO:0060076 | excitatory synapse | 0.147541 | 0.00244 |
| GO:0008081 | phosphoric diester hydrolase activity | 0.123711 | 0.00239 |
| GO:1903170 | negative regulation of calcium ion transmembrane transport | 0.179487 | 0.00233 |
| GO:0016050 | vesicle organization | 0.092251 | 0.00232 |
| GO:0010902 | positive regulation of very-low-density lipoprotein particle remodeling | 1 | 0.00231 |
| GO:0033127 | obsolete regulation of histone phosphorylation | 1 | 0.00231 |
| GO:0099548 | trans-synaptic signaling by nitric oxide | 1 | 0.00231 |
| GO:0099543 | trans-synaptic signaling by soluble gas | 1 | 0.00231 |
| GO:1903244 | positive regulation of cardiac muscle hypertrophy in response to stress | 1 | 0.00231 |
| GO:0001869 | negative regulation of complement activation, lectin pathway | 1 | 0.00231 |
| GO:0001868 | regulation of complement activation, lectin pathway | 1 | 0.00231 |
| GO:0010615 | positive regulation of cardiac muscle adaptation | 1 | 0.00231 |
| GO:0006864 | pyrimidine nucleotide transport | 1 | 0.00231 |
| GO:0072162 | metanephric mesenchymal cell differentiation | 1 | 0.00231 |
| GO:1901727 | positive regulation of histone deacetylase activity | 1 | 0.00231 |
| GO:0099554 | trans-synaptic signaling by soluble gas, modulating synaptic transmission | 1 | 0.00231 |
| GO:0099555 | trans-synaptic signaling by nitric oxide, modulating synaptic transmission | 1 | 0.00231 |
| GO:0008204 | ergosterol metabolic process | 1 | 0.00231 |
| GO:0060448 | dichotomous subdivision of terminal units involved in lung branching | 1 | 0.00231 |
| GO:0006696 | ergosterol biosynthetic process | 1 | 0.00231 |
| GO:0090673 | endothelial cell-matrix adhesion | 1 | 0.00231 |
| GO:1990519 | pyrimidine nucleotide import into mitochondrion | 1 | 0.00231 |
| GO:0051599 | response to hydrostatic pressure | 1 | 0.00231 |
| GO:0099163 | synaptic signaling by nitric oxide | 1 | 0.00231 |
| GO:0042568 | insulin-like growth factor binary complex | 1 | 0.00231 |
| GO:0008074 | guanylate cyclase complex, soluble | 1 | 0.00231 |
| GO:0071753 | IgM immunoglobulin complex | 1 | 0.00231 |
| GO:0071756 | pentameric IgM immunoglobulin complex | 1 | 0.00231 |
| GO:0071754 | IgM immunoglobulin complex, circulating | 1 | 0.00231 |
| GO:0008503 | benzodiazepine receptor activity | 1 | 0.00231 |
| GO:0005497 | androgen binding | 1 | 0.00231 |
| GO:0004103 | choline kinase activity | 1 | 0.00231 |
| GO:0032397 | activating MHC class I receptor activity | 1 | 0.00231 |
| GO:0031722 | hemoglobin beta binding | 1 | 0.00231 |
| GO:0000254 | C-4 methylsterol oxidase activity | 1 | 0.00231 |
| GO:0005017 | platelet-derived growth factor receptor activity | 1 | 0.00231 |
| GO:0015218 | pyrimidine nucleotide transmembrane transporter activity | 1 | 0.00231 |
| GO:0019135 | deoxyhypusine monooxygenase activity | 1 | 0.00231 |
| GO:0031692 | alpha-1B adrenergic receptor binding | 1 | 0.00231 |
| GO:0031691 | alpha-1A adrenergic receptor binding | 1 | 0.00231 |
| GO:0051649 | establishment of localization in cell | 0.063373 | 0.00229 |
| GO:0062012 | regulation of small molecule metabolic process | 0.08596 | 0.00228 |
| GO:0043621 | protein self-association | 0.138889 | 0.00227 |
| GO:0071674 | mononuclear cell migration | 0.130952 | 0.00227 |
| GO:0050673 | epithelial cell proliferation | 0.130952 | 0.00227 |
| GO:0031300 | intrinsic component of organelle membrane | 0.08313 | 0.00226 |
| GO:0030258 | lipid modification | 0.10625 | 0.00225 |
| GO:0010837 | regulation of keratinocyte proliferation | 0.206897 | 0.00224 |
| GO:0007635 | chemosensory behavior | 0.206897 | 0.00224 |
| GO:1903573 | negative regulation of response to endoplasmic reticulum stress | 0.206897 | 0.00224 |
| GO:0015294 | solute:cation symporter activity | 0.119266 | 0.00224 |
| GO:0035770 | ribonucleoprotein granule | 0.087774 | 0.00223 |
| GO:0002250 | adaptive immune response | 0.089655 | 0.00222 |
| GO:0060326 | cell chemotaxis | 0.096774 | 0.00219 |
| GO:0021700 | developmental maturation | 0.096774 | 0.00219 |
| GO:0005096 | GTPase activator activity | 0.086455 | 0.00218 |
| GO:1903036 | positive regulation of response to wounding | 0.163265 | 0.00218 |
| GO:0097110 | scaffold protein binding | 0.15 | 0.00217 |
| GO:0050927 | positive regulation of positive chemotaxis | 0.25 | 0.00216 |
| GO:0090128 | regulation of synapse maturation | 0.25 | 0.00216 |
| GO:0048037 | obsolete cofactor binding | 0.25 | 0.00216 |
| GO:0030003 | cellular cation homeostasis | 0.085333 | 0.00214 |
| GO:0090407 | organophosphate biosynthetic process | 0.074344 | 0.00214 |
| GO:0001227 | DNA-binding transcription repressor activity, RNA polymerase II-specific | 0.090278 | 0.00208 |
| GO:0001217 | DNA-binding transcription repressor activity | 0.090278 | 0.00208 |
| GO:0022904 | respiratory electron transport chain | 0.103448 | 0.00208 |
| GO:0005911 | cell-cell junction | 0.077465 | 0.00206 |
| GO:0019217 | regulation of fatty acid metabolic process | 0.13253 | 0.00206 |
| GO:0001894 | tissue homeostasis | 0.107595 | 0.00205 |
| GO:0051258 | protein polymerization | 0.140845 | 0.00205 |
| GO:0050727 | regulation of inflammatory response | 0.090592 | 0.00202 |
| GO:0050997 | quaternary ammonium group binding | 0.184211 | 0.00199 |
| GO:0034139 | regulation of toll-like receptor 3 signaling pathway | 0.5 | 0.00199 |
| GO:0051562 | negative regulation of mitochondrial calcium ion concentration | 0.5 | 0.00199 |
| GO:0021960 | anterior commissure morphogenesis | 0.5 | 0.00199 |
| GO:0035723 | interleukin-15-mediated signaling pathway | 0.5 | 0.00199 |
| GO:0021540 | corpus callosum morphogenesis | 0.5 | 0.00199 |
| GO:0097119 | postsynaptic density protein 95 clustering | 0.5 | 0.00199 |
| GO:0007169 | transmembrane receptor protein tyrosine kinase signaling pathway | 0.089457 | 0.00195 |
| GO:0009214 | cyclic nucleotide catabolic process | 0.333333 | 0.00193 |
| GO:0018158 | protein oxidation | 0.333333 | 0.00193 |
| GO:0010872 | regulation of cholesterol esterification | 0.333333 | 0.00193 |
| GO:0019203 | carbohydrate phosphatase activity | 0.333333 | 0.00193 |
| GO:0003954 | NADH dehydrogenase activity | 0.152542 | 0.00192 |
| GO:0006357 | regulation of transcription by RNA polymerase II | 0.061958 | 0.00191 |
| GO:0080164 | regulation of nitric oxide metabolic process | 0.166667 | 0.00190 |
| GO:0006695 | cholesterol biosynthetic process | 0.166667 | 0.00190 |
| GO:0031985 | Golgi cisterna | 0.166667 | 0.00190 |
| GO:0099503 | secretory vesicle | 0.078846 | 0.00190 |
| GO:0019209 | kinase activator activity | 0.108974 | 0.00188 |
| GO:0005793 | endoplasmic reticulum-Golgi intermediate compartment | 0.104651 | 0.00187 |
| GO:0035239 | tube morphogenesis | 0.097046 | 0.00187 |
| GO:0035987 | endodermal cell differentiation | 0.214286 | 0.00185 |
| GO:0021952 | central nervous system projection neuron axonogenesis | 0.214286 | 0.00185 |
| GO:0005790 | smooth endoplasmic reticulum | 0.214286 | 0.00185 |
| GO:0019887 | protein kinase regulator activity | 0.09375 | 0.00180 |
| GO:0043270 | positive regulation of ion transport | 0.092199 | 0.00176 |
| GO:0098771 | inorganic ion homeostasis | 0.080745 | 0.00175 |
| GO:0009890 | negative regulation of biosynthetic process | 0.063387 | 0.00174 |
| GO:0032496 | response to lipopolysaccharide | 0.083532 | 0.00173 |
| GO:0006897 | endocytosis | 0.092527 | 0.00172 |
| GO:0034765 | regulation of ion transmembrane transport | 0.082405 | 0.00170 |
| GO:0006739 | NADP metabolic process | 0.189189 | 0.00170 |
| GO:0033273 | response to vitamin | 0.135802 | 0.00169 |
| GO:1904063 | negative regulation of cation transmembrane transport | 0.135802 | 0.00169 |
| GO:0072009 | nephron epithelium development | 0.263158 | 0.00169 |
| GO:0071625 | vocalization behavior | 0.263158 | 0.00169 |
| GO:0032967 | positive regulation of collagen biosynthetic process | 0.263158 | 0.00169 |
| GO:0000421 | autophagosome membrane | 0.170213 | 0.00165 |
| GO:0046915 | transition metal ion transmembrane transporter activity | 0.170213 | 0.00165 |
| GO:0051493 | regulation of cytoskeleton organization | 0.078899 | 0.00164 |
| GO:0098793 | presynapse | 0.090301 | 0.00163 |
| GO:0007167 | enzyme-linked receptor protein signaling pathway | 0.08159 | 0.00163 |
| GO:0031226 | intrinsic component of plasma membrane | 0.065722 | 0.00163 |
| GO:0043271 | negative regulation of ion transport | 0.111842 | 0.00162 |
| GO:0045935 | positive regulation of nucleobase-containing compound metabolic process | 0.06391 | 0.00158 |
| GO:1904064 | positive regulation of cation transmembrane transport | 0.112583 | 0.00157 |
| GO:0007041 | lysosomal transport | 0.112583 | 0.00157 |
| GO:0050878 | regulation of body fluid levels | 0.090909 | 0.00153 |
| GO:0016540 | protein autoprocessing | 0.222222 | 0.00152 |
| GO:0005504 | fatty acid binding | 0.157895 | 0.00150 |
| GO:0030133 | transport vesicle | 0.096 | 0.00148 |
| GO:0009056 | catabolic process | 0.061533 | 0.00147 |
| GO:0051248 | negative regulation of protein metabolic process | 0.068071 | 0.00146 |
| GO:0001664 | G protein-coupled receptor binding | 0.089783 | 0.00146 |
| GO:0034767 | positive regulation of ion transmembrane transport | 0.107784 | 0.00145 |
| GO:1901362 | organic cyclic compound biosynthetic process | 0.068644 | 0.00145 |
| GO:0051020 | GTPase binding | 0.081897 | 0.00144 |
| GO:0001942 | hair follicle development | 0.194444 | 0.00143 |
| GO:0045923 | positive regulation of fatty acid metabolic process | 0.194444 | 0.00143 |
| GO:0014014 | negative regulation of gliogenesis | 0.194444 | 0.00143 |
| GO:0034446 | substrate adhesion-dependent cell spreading | 0.173913 | 0.00143 |
| GO:0051602 | response to electrical stimulus | 0.173913 | 0.00143 |
| GO:0048589 | developmental growth | 0.083333 | 0.00142 |
| GO:0040007 | growth | 0.083333 | 0.00142 |
| GO:1902679 | negative regulation of RNA biosynthetic process | 0.066528 | 0.00138 |
| GO:1903507 | negative regulation of nucleic acid-templated transcription | 0.066574 | 0.00137 |
| GO:0019207 | kinase regulator activity | 0.09215 | 0.00136 |
| GO:0044342 | type B pancreatic cell proliferation | 0.363636 | 0.00134 |
| GO:1903671 | negative regulation of sprouting angiogenesis | 0.363636 | 0.00134 |
| GO:0050308 | sugar-phosphatase activity | 0.363636 | 0.00134 |
| GO:0015232 | heme transmembrane transporter activity | 0.363636 | 0.00134 |
| GO:0050678 | regulation of epithelial cell proliferation | 0.091195 | 0.00133 |
| GO:0000302 | response to reactive oxygen species | 0.109091 | 0.00133 |
| GO:1903320 | regulation of protein modification by small protein conjugation or removal | 0.092466 | 0.00132 |
| GO:0014743 | regulation of muscle hypertrophy | 0.149254 | 0.00131 |
| GO:1903317 | regulation of protein maturation | 0.149254 | 0.00131 |
| GO:0044849 | estrous cycle | 0.277778 | 0.00129 |
| GO:0051481 | negative regulation of cytosolic calcium ion concentration | 0.277778 | 0.00129 |
| GO:0051156 | glucose 6-phosphate metabolic process | 0.277778 | 0.00129 |
| GO:0048010 | vascular endothelial growth factor receptor signaling pathway | 0.277778 | 0.00129 |
| GO:0031091 | platelet alpha granule | 0.277778 | 0.00129 |
| GO:0031175 | neuron projection development | 0.082969 | 0.00129 |
| GO:0071705 | nitrogen compound transport | 0.06466 | 0.00128 |
| GO:0042542 | response to hydrogen peroxide | 0.117188 | 0.00128 |
| GO:0051235 | maintenance of location | 0.109756 | 0.00127 |
| GO:0010942 | positive regulation of cell death | 0.077295 | 0.00126 |
| GO:0035094 | response to nicotine | 0.177778 | 0.00123 |
| GO:0001837 | epithelial to mesenchymal transition | 0.177778 | 0.00123 |
| GO:0042178 | xenobiotic catabolic process | 0.141026 | 0.00123 |
| GO:0002449 | lymphocyte mediated immunity | 0.141026 | 0.00123 |
| GO:0048701 | embryonic cranial skeleton morphogenesis | 0.230769 | 0.00123 |
| GO:0072657 | protein localization to membrane | 0.084906 | 0.00122 |
| GO:0030098 | lymphocyte differentiation | 0.095057 | 0.00122 |
| GO:0002683 | negative regulation of immune system process | 0.086294 | 0.00121 |
| GO:0098660 | inorganic ion transmembrane transport | 0.085106 | 0.00121 |
| GO:0005759 | mitochondrial matrix | 0.090909 | 0.00120 |
| GO:0050807 | regulation of synapse organization | 0.100457 | 0.00118 |
| GO:0051056 | regulation of small GTPase mediated signal transduction | 0.094077 | 0.00118 |
| GO:0050136 | NADH dehydrogenase (quinone) activity | 0.163636 | 0.00116 |
| GO:0015036 | disulfide oxidoreductase activity | 0.163636 | 0.00116 |
| GO:0008137 | NADH dehydrogenase (ubiquinone) activity | 0.163636 | 0.00116 |
| GO:0007399 | nervous system development | 0.106742 | 0.00114 |
| GO:2000146 | negative regulation of cell motility | 0.096154 | 0.00110 |
| GO:0051253 | negative regulation of RNA metabolic process | 0.066323 | 0.00107 |
| GO:2000379 | positive regulation of reactive oxygen species metabolic process | 0.181818 | 0.00106 |
| GO:0045321 | leukocyte activation | 0.080374 | 0.00105 |
| GO:0030534 | adult behavior | 0.107955 | 0.00103 |
| GO:0051918 | negative regulation of fibrinolysis | 0.6 | 0.00103 |
| GO:0072378 | blood coagulation, fibrin clot formation | 0.6 | 0.00103 |
| GO:0034141 | positive regulation of toll-like receptor 3 signaling pathway | 0.6 | 0.00103 |
| GO:0002933 | lipid hydroxylation | 0.6 | 0.00103 |
| GO:0018057 | peptidyl-lysine oxidation | 0.6 | 0.00103 |
| GO:0004576 | oligosaccharyl transferase activity | 0.6 | 0.00103 |
| GO:0004579 | dolichyl-diphosphooligosaccharide-protein glycotransferase activity | 0.6 | 0.00103 |
| GO:0004720 | protein-lysine 6-oxidase activity | 0.6 | 0.00103 |
| GO:0048403 | brain-derived neurotrophic factor binding | 0.6 | 0.00103 |
| GO:0060228 | phosphatidylcholine-sterol O-acyltransferase activator activity | 0.6 | 0.00103 |
| GO:0043539 | protein serine/threonine kinase activator activity | 0.13 | 0.00101 |
| GO:0001666 | response to hypoxia | 0.094545 | 0.00100 |
| GO:0070301 | cellular response to hydrogen peroxide | 0.144737 | 0.00099 |
| GO:0004714 | transmembrane receptor protein tyrosine kinase activity | 0.144737 | 0.00099 |
| GO:0030449 | regulation of complement activation | 0.24 | 0.00098 |
| GO:0033344 | cholesterol efflux | 0.24 | 0.00098 |
| GO:1903318 | negative regulation of protein maturation | 0.24 | 0.00098 |
| GO:0010955 | negative regulation of protein processing | 0.24 | 0.00098 |
| GO:0002040 | sprouting angiogenesis | 0.24 | 0.00098 |
| GO:0042744 | hydrogen peroxide catabolic process | 0.24 | 0.00098 |
| GO:1902235 | regulation of endoplasmic reticulum stress-induced intrinsic apoptotic signaling pathway | 0.24 | 0.00098 |
| GO:0001968 | fibronectin binding | 0.24 | 0.00098 |
| GO:0009887 | animal organ morphogenesis | 0.080979 | 0.00098 |
| GO:0014002 | astrocyte development | 0.294118 | 0.00097 |
| GO:1902547 | regulation of cellular response to vascular endothelial growth factor stimulus | 0.294118 | 0.00097 |
| GO:0006067 | ethanol metabolic process | 0.294118 | 0.00097 |
| GO:0101020 | estrogen 16-alpha-hydroxylase activity | 0.294118 | 0.00097 |
| GO:0001558 | regulation of cell growth | 0.085057 | 0.00095 |
| GO:0045444 | fat cell differentiation | 0.120968 | 0.00092 |
| GO:0030658 | transport vesicle membrane | 0.120968 | 0.00092 |
| GO:0060998 | regulation of dendritic spine development | 0.15625 | 0.00091 |
| GO:0010721 | negative regulation of cell development | 0.109827 | 0.00090 |
| GO:0000122 | negative regulation of transcription by RNA polymerase II | 0.072187 | 0.00090 |
| GO:0048562 | embryonic organ morphogenesis | 0.126126 | 0.00089 |
| GO:0014745 | negative regulation of muscle adaptation | 0.4 | 0.00088 |
| GO:0010612 | regulation of cardiac muscle adaptation | 0.4 | 0.00088 |
| GO:0034116 | positive regulation of heterotypic cell-cell adhesion | 0.4 | 0.00088 |
| GO:0060999 | positive regulation of dendritic spine development | 0.169811 | 0.00088 |
| GO:0015485 | cholesterol binding | 0.169811 | 0.00088 |
| GO:1903531 | negative regulation of secretion by cell | 0.110465 | 0.00086 |
| GO:0044782 | cilium organization | 0.095941 | 0.00086 |
| GO:0031301 | integral component of organelle membrane | 0.088949 | 0.00084 |
| GO:1902533 | positive regulation of intracellular signal transduction | 0.070473 | 0.00084 |
| GO:0042596 | fear response | 0.132653 | 0.00084 |
| GO:0019865 | immunoglobulin binding | 0.212121 | 0.00083 |
| GO:0016791 | phosphatase activity | 0.090643 | 0.00081 |
| GO:0048660 | regulation of smooth muscle cell proliferation | 0.118519 | 0.00079 |
| GO:0030178 | negative regulation of Wnt signaling pathway | 0.118519 | 0.00079 |
| GO:0060429 | epithelium development | 0.114865 | 0.00079 |
| GO:0030295 | protein kinase activator activity | 0.114865 | 0.00079 |
| GO:0005509 | calcium ion binding | 0.071992 | 0.00078 |
| GO:0040036 | regulation of fibroblast growth factor receptor signaling pathway | 0.25 | 0.00078 |
| GO:0034762 | regulation of transmembrane transport | 0.081181 | 0.00078 |
| GO:0032963 | collagen metabolic process | 0.173077 | 0.00076 |
| GO:0002209 | behavioral defense response | 0.134021 | 0.00076 |
| GO:0036503 | ERAD pathway | 0.141176 | 0.00075 |
| GO:0003727 | single-stranded RNA binding | 0.113095 | 0.00074 |
| GO:0046890 | regulation of lipid biosynthetic process | 0.108108 | 0.00074 |
| GO:1904950 | negative regulation of establishment of protein localization | 0.115646 | 0.00073 |
| GO:0070201 | regulation of establishment of protein localization | 0.08056 | 0.00073 |
| GO:0005774 | vacuolar membrane | 0.097744 | 0.00073 |
| GO:0019637 | organophosphate metabolic process | 0.067633 | 0.00072 |
| GO:1900048 | positive regulation of hemostasis | 0.3125 | 0.00071 |
| GO:1900746 | regulation of vascular endothelial growth factor signaling pathway | 0.3125 | 0.00071 |
| GO:0031204 | post-translational protein targeting to membrane, translocation | 0.3125 | 0.00071 |
| GO:0030194 | positive regulation of blood coagulation | 0.3125 | 0.00071 |
| GO:0008061 | chitin binding | 0.3125 | 0.00071 |
| GO:0098754 | detoxification | 0.16129 | 0.00070 |
| GO:0043535 | regulation of blood vessel endothelial cell migration | 0.150685 | 0.00070 |
| GO:0030246 | carbohydrate binding | 0.092537 | 0.00070 |
| GO:0010557 | positive regulation of macromolecule biosynthetic process | 0.065858 | 0.00070 |
| GO:0009628 | response to abiotic stimulus | 0.069261 | 0.00070 |
| GO:0050900 | leukocyte migration | 0.105528 | 0.00070 |
| GO:0046873 | metal ion transmembrane transporter activity | 0.084746 | 0.00069 |
| GO:0006829 | zinc ion transport | 0.21875 | 0.00068 |
| GO:1901385 | regulation of voltage-gated calcium channel activity | 0.21875 | 0.00068 |
| GO:0042169 | SH2 domain binding | 0.21875 | 0.00068 |
| GO:0008047 | enzyme activator activity | 0.080268 | 0.00067 |
| GO:0050709 | negative regulation of protein secretion | 0.142857 | 0.00067 |
| GO:0014066 | regulation of phosphatidylinositol 3-kinase signaling | 0.142857 | 0.00067 |
| GO:0043502 | regulation of muscle adaptation | 0.142857 | 0.00067 |
| GO:1901698 | response to nitrogen compound | 0.068783 | 0.00066 |
| GO:0005794 | Golgi apparatus | 0.070725 | 0.00065 |
| GO:0031396 | regulation of protein ubiquitination | 0.099617 | 0.00065 |
| GO:0032880 | regulation of protein localization | 0.073743 | 0.00065 |
| GO:0031099 | regeneration | 0.10989 | 0.00064 |
| GO:0140352 | export from cell | 0.088235 | 0.00063 |
| GO:0046474 | glycerophospholipid biosynthetic process | 0.106599 | 0.00062 |
| GO:0046649 | lymphocyte activation | 0.086047 | 0.00062 |
| GO:0035023 | regulation of Rho protein signal transduction | 0.121212 | 0.00062 |
| GO:0001662 | behavioral fear response | 0.136842 | 0.00062 |
| GO:0001891 | phagocytic cup | 0.26087 | 0.00061 |
| GO:0042981 | regulation of apoptotic process | 0.065083 | 0.00060 |
| GO:0009410 | response to xenobiotic stimulus | 0.092879 | 0.00059 |
| GO:0050796 | regulation of insulin secretion | 0.111111 | 0.00058 |
| GO:0070372 | regulation of ERK1 and ERK2 cascade | 0.097122 | 0.00058 |
| GO:0004867 | serine-type endopeptidase inhibitor activity | 0.11465 | 0.00058 |
| GO:0006986 | response to unfolded protein | 0.18 | 0.00056 |
| GO:0016209 | antioxidant activity | 0.132075 | 0.00056 |
| GO:0031210 | phosphatidylcholine binding | 0.225806 | 0.00056 |
| GO:0071493 | cellular response to UV-B | 0.444444 | 0.00055 |
| GO:1903242 | regulation of cardiac muscle hypertrophy in response to stress | 0.444444 | 0.00055 |
| GO:0010838 | positive regulation of keratinocyte proliferation | 0.444444 | 0.00055 |
| GO:0099025 | anchored component of postsynaptic membrane | 0.444444 | 0.00055 |
| GO:0002460 | adaptive immune response based on somatic recombination of immune receptors built from immunoglobulin superfamily domains | 0.146341 | 0.00054 |
| GO:0051224 | negative regulation of protein transport | 0.118881 | 0.00053 |
| GO:0030031 | cell projection assembly | 0.092486 | 0.00053 |
| GO:0043067 | regulation of programmed cell death | 0.065273 | 0.00053 |
| GO:0005938 | cell cortex | 0.103004 | 0.00052 |
| GO:0071840 | cellular component organization or biogenesis | 0.055955 | 0.00051 |
| GO:1901841 | regulation of high voltage-gated calcium channel activity | 0.333333 | 0.00051 |
| GO:0004697 | protein kinase C activity | 0.333333 | 0.00051 |
| GO:1903131 | mononuclear cell differentiation | 0.09589 | 0.00051 |
| GO:0008134 | transcription factor binding | 0.076433 | 0.00051 |
| GO:0031324 | negative regulation of cellular metabolic process | 0.062232 | 0.00050 |
| GO:0033554 | cellular response to stress | 0.066549 | 0.00050 |
| GO:0036094 | small molecule binding | 0.059068 | 0.00050 |
| GO:0016829 | lyase activity | 0.09434 | 0.00050 |
| GO:0045202 | synapse | 0.069413 | 0.00049 |
| GO:0010631 | epithelial cell migration | 0.157143 | 0.00049 |
| GO:0030433 | ubiquitin-dependent ERAD pathway | 0.157143 | 0.00049 |
| GO:0072073 | kidney epithelium development | 0.183673 | 0.00048 |
| GO:1902653 | secondary alcohol biosynthetic process | 0.183673 | 0.00048 |
| GO:0072599 | establishment of protein localization to endoplasmic reticulum | 0.183673 | 0.00048 |
| GO:0030336 | negative regulation of cell migration | 0.100806 | 0.00048 |
| GO:0044248 | cellular catabolic process | 0.065881 | 0.00048 |
| GO:0040013 | negative regulation of locomotion | 0.096552 | 0.00047 |
| GO:0085029 | extracellular matrix assembly | 0.272727 | 0.00047 |
| GO:0035966 | response to topologically incorrect protein | 0.169492 | 0.00047 |
| GO:0032934 | sterol binding | 0.169492 | 0.00047 |
| GO:0006873 | cellular ion homeostasis | 0.089744 | 0.00047 |
| GO:0045926 | negative regulation of growth | 0.101215 | 0.00046 |
| GO:0030695 | GTPase regulator activity | 0.080944 | 0.00046 |
| GO:0060589 | nucleoside-triphosphatase regulator activity | 0.080944 | 0.00046 |
| GO:0014013 | regulation of gliogenesis | 0.141304 | 0.00045 |
| GO:0051345 | positive regulation of hydrolase activity | 0.082143 | 0.00045 |
| GO:0042391 | regulation of membrane potential | 0.088452 | 0.00043 |
| GO:0044089 | positive regulation of cellular component biogenesis | 0.085837 | 0.00043 |
| GO:2001199 | negative regulation of dendritic cell differentiation | 0.75 | 0.00043 |
| GO:2001012 | mesenchymal cell differentiation involved in renal system development | 0.75 | 0.00043 |
| GO:0098903 | regulation of membrane repolarization during action potential | 0.75 | 0.00043 |
| GO:0072161 | mesenchymal cell differentiation involved in kidney development | 0.75 | 0.00043 |
| GO:0097107 | postsynaptic density assembly | 0.75 | 0.00043 |
| GO:1901843 | positive regulation of high voltage-gated calcium channel activity | 0.75 | 0.00043 |
| GO:0036454 | growth factor complex | 0.75 | 0.00043 |
| GO:0016942 | insulin-like growth factor binding protein complex | 0.75 | 0.00043 |
| GO:0035276 | ethanol binding | 0.75 | 0.00043 |
| GO:0034220 | ion transmembrane transport | 0.082585 | 0.00043 |
| GO:0045471 | response to ethanol | 0.114458 | 0.00042 |
| GO:0001936 | regulation of endothelial cell proliferation | 0.130435 | 0.00041 |
| GO:0001816 | cytokine production | 0.130435 | 0.00041 |
| GO:0072341 | modified amino acid binding | 0.130435 | 0.00041 |
| GO:0043536 | positive regulation of blood vessel endothelial cell migration | 0.1875 | 0.00041 |
| GO:0015035 | protein-disulfide reductase activity | 0.1875 | 0.00041 |
| GO:0045277 | respiratory chain complex IV | 0.172414 | 0.00041 |
| GO:0003955 | NAD(P)H dehydrogenase (quinone) activity | 0.172414 | 0.00041 |
| GO:0005506 | iron ion binding | 0.093939 | 0.00040 |
| GO:0000902 | cell morphogenesis | 0.102881 | 0.00039 |
| GO:2000249 | regulation of actin cytoskeleton reorganization | 0.210526 | 0.00038 |
| GO:0051048 | negative regulation of secretion | 0.108911 | 0.00036 |
| GO:0046688 | response to copper ion | 0.241379 | 0.00036 |
| GO:0045655 | regulation of monocyte differentiation | 0.285714 | 0.00036 |
| GO:0046415 | urate metabolic process | 0.357143 | 0.00035 |
| GO:1902236 | negative regulation of endoplasmic reticulum stress-induced intrinsic apoptotic signaling pathway | 0.357143 | 0.00035 |
| GO:0016709 | oxidoreductase activity, acting on paired donors, with incorporation or reduction of molecular oxygen, NAD(P)H as one donor, and incorporation of one atom of oxygen | 0.175439 | 0.00035 |
| GO:0007268 | chemical synaptic transmission | 0.1 | 0.00034 |
| GO:0098916 | anterograde trans-synaptic signaling | 0.1 | 0.00034 |
| GO:0031346 | positive regulation of cell projection organization | 0.09068 | 0.00033 |
| GO:0051223 | regulation of protein transport | 0.083955 | 0.00033 |
| GO:0044092 | negative regulation of molecular function | 0.071489 | 0.00033 |
| GO:0034380 | high-density lipoprotein particle assembly | 0.5 | 0.00032 |
| GO:0010873 | positive regulation of cholesterol esterification | 0.5 | 0.00032 |
| GO:0005784 | Sec61 translocon complex | 0.5 | 0.00032 |
| GO:0051254 | positive regulation of RNA metabolic process | 0.067807 | 0.00031 |
| GO:0006996 | organelle organization | 0.062611 | 0.00031 |
| GO:0042572 | retinol metabolic process | 0.216216 | 0.00031 |
| GO:0051668 | localization within membrane | 0.085657 | 0.00031 |
| GO:0050890 | cognition | 0.097179 | 0.00030 |
| GO:0045597 | positive regulation of cell differentiation | 0.076647 | 0.00030 |
| GO:0042562 | hormone binding | 0.147727 | 0.00029 |
| GO:0042246 | tissue regeneration | 0.166667 | 0.00029 |
| GO:0007032 | endosome organization | 0.135135 | 0.00028 |
| GO:0006952 | defense response | 0.066667 | 0.00028 |
| GO:0001775 | cell activation | 0.081699 | 0.00027 |
| GO:0051961 | negative regulation of nervous system development | 0.125926 | 0.00027 |
| GO:0098662 | inorganic cation transmembrane transport | 0.091623 | 0.00027 |
| GO:0097242 | amyloid-beta clearance | 0.3 | 0.00026 |
| GO:0050776 | regulation of immune response | 0.074719 | 0.00026 |
| GO:0090288 | negative regulation of cellular response to growth factor stimulus | 0.149425 | 0.00026 |
| GO:0005507 | copper ion binding | 0.149425 | 0.00026 |
| GO:0120031 | plasma membrane bounded cell projection assembly | 0.095522 | 0.00026 |
| GO:0021955 | central nervous system neuron axonogenesis | 0.222222 | 0.00025 |
| GO:0008203 | cholesterol metabolic process | 0.131148 | 0.00025 |
| GO:0006954 | inflammatory response | 0.086598 | 0.00025 |
| GO:0045047 | protein targeting to ER | 0.2 | 0.00025 |
| GO:1903556 | negative regulation of tumor necrosis factor superfamily cytokine production | 0.2 | 0.00025 |
| GO:0043014 | alpha-tubulin binding | 0.2 | 0.00025 |
| GO:0015293 | symporter activity | 0.119497 | 0.00024 |
| GO:0007568 | aging | 0.101887 | 0.00024 |
| GO:0006650 | glycerophospholipid metabolic process | 0.100346 | 0.00024 |
| GO:0051336 | regulation of hydrolase activity | 0.071661 | 0.00024 |
| GO:0060271 | cilium assembly | 0.104839 | 0.00024 |
| GO:0008250 | oligosaccharyltransferase complex | 0.384615 | 0.00024 |
| GO:0004115 | 3',5'-cyclic-AMP phosphodiesterase activity | 0.384615 | 0.00024 |
| GO:0045860 | positive regulation of protein kinase activity | 0.091584 | 0.00023 |
| GO:0007219 | Notch signaling pathway | 0.132231 | 0.00023 |
| GO:0051928 | positive regulation of calcium ion transport | 0.132231 | 0.00023 |
| GO:0030324 | lung development | 0.137615 | 0.00023 |
| GO:0006631 | fatty acid metabolic process | 0.092838 | 0.00023 |
| GO:2000377 | regulation of reactive oxygen species metabolic process | 0.114583 | 0.00023 |
| GO:0048568 | embryonic organ development | 0.12782 | 0.00023 |
| GO:0050768 | negative regulation of neurogenesis | 0.12782 | 0.00023 |
| GO:1901701 | cellular response to oxygen-containing compound | 0.072165 | 0.00022 |
| GO:0070206 | protein trimerization | 0.171875 | 0.00022 |
| GO:1902680 | positive regulation of RNA biosynthetic process | 0.069426 | 0.00021 |
| GO:0045893 | positive regulation of DNA-templated transcription | 0.069472 | 0.00021 |
| GO:1903508 | positive regulation of nucleic acid-templated transcription | 0.069472 | 0.00021 |
| GO:0031589 | cell-substrate adhesion | 0.111111 | 0.00021 |
| GO:0048729 | tissue morphogenesis | 0.093834 | 0.00021 |
| GO:0016042 | lipid catabolic process | 0.09901 | 0.00020 |
| GO:0009891 | positive regulation of biosynthetic process | 0.066667 | 0.00020 |
| GO:0045185 | maintenance of protein location | 0.145833 | 0.00020 |
| GO:0051787 | misfolded protein binding | 0.315789 | 0.00019 |
| GO:0032956 | regulation of actin cytoskeleton organization | 0.095652 | 0.00019 |
| GO:0031644 | regulation of nervous system process | 0.121795 | 0.00019 |
| GO:0022404 | molting cycle process | 0.174603 | 0.00019 |
| GO:0022405 | hair cycle process | 0.174603 | 0.00019 |
| GO:0055082 | cellular chemical homeostasis | 0.087045 | 0.00019 |
| GO:0060341 | regulation of cellular localization | 0.074627 | 0.00018 |
| GO:0005201 | extracellular matrix structural constituent | 0.164384 | 0.00018 |
| GO:0033993 | response to lipid | 0.074427 | 0.00018 |
| GO:0071241 | cellular response to inorganic substance | 0.100671 | 0.00017 |
| GO:0031323 | regulation of cellular metabolic process | 0.056982 | 0.00017 |
| GO:0098869 | cellular oxidant detoxification | 0.269231 | 0.00017 |
| GO:2000463 | positive regulation of excitatory postsynaptic potential | 0.269231 | 0.00017 |
| GO:0009975 | cyclase activity | 0.269231 | 0.00017 |
| GO:0032994 | protein-lipid complex | 0.209302 | 0.00017 |
| GO:0010634 | positive regulation of epithelial cell migration | 0.135593 | 0.00017 |
| GO:0032103 | positive regulation of response to external stimulus | 0.09201 | 0.00017 |
| GO:0051338 | regulation of transferase activity | 0.076923 | 0.00017 |
| GO:0015990 | electron transport coupled proton transport | 0.571429 | 0.00017 |
| GO:0098698 | postsynaptic specialization assembly | 0.571429 | 0.00017 |
| GO:0015988 | energy coupled proton transmembrane transport, against electrochemical gradient | 0.571429 | 0.00017 |
| GO:0031995 | insulin-like growth factor II binding | 0.571429 | 0.00017 |
| GO:0098655 | cation transmembrane transport | 0.092457 | 0.00016 |
| GO:0031328 | positive regulation of cellular biosynthetic process | 0.06738 | 0.00016 |
| GO:0009617 | response to bacterium | 0.079324 | 0.00016 |
| GO:0002573 | myeloid leukocyte differentiation | 0.156627 | 0.00016 |
| GO:0016860 | intramolecular oxidoreductase activity | 0.156627 | 0.00016 |
| GO:0006694 | steroid biosynthetic process | 0.131783 | 0.00016 |
| GO:0010975 | regulation of neuron projection development | 0.088477 | 0.00016 |
| GO:0007599 | hemostasis | 0.166667 | 0.00015 |
| GO:0032970 | regulation of actin filament-based process | 0.093995 | 0.00015 |
| GO:0006182 | cGMP biosynthetic process | 0.416667 | 0.00015 |
| GO:1901387 | positive regulation of voltage-gated calcium channel activity | 0.416667 | 0.00015 |
| GO:0005583 | fibrillar collagen trimer | 0.416667 | 0.00015 |
| GO:0008195 | phosphatidate phosphatase activity | 0.416667 | 0.00015 |
| GO:0034976 | response to endoplasmic reticulum stress | 0.115578 | 0.00015 |
| GO:0009607 | response to biotic stimulus | 0.066346 | 0.00015 |
| GO:0007611 | learning or memory | 0.103704 | 0.00015 |
| GO:0006656 | phosphatidylcholine biosynthetic process | 0.214286 | 0.00014 |
| GO:0032720 | negative regulation of tumor necrosis factor production | 0.214286 | 0.00014 |
| GO:0051347 | positive regulation of transferase activity | 0.085258 | 0.00014 |
| GO:0000904 | cell morphogenesis involved in differentiation | 0.128571 | 0.00014 |
| GO:0034330 | cell junction organization | 0.091549 | 0.00014 |
| GO:0046068 | cGMP metabolic process | 0.333333 | 0.00014 |
| GO:0033674 | positive regulation of kinase activity | 0.090708 | 0.00014 |
| GO:0005886 | plasma membrane | 0.058402 | 0.00014 |
| GO:0072593 | reactive oxygen species metabolic process | 0.144231 | 0.00014 |
| GO:0045017 | glycerolipid biosynthetic process | 0.11215 | 0.00014 |
| GO:1902600 | proton transmembrane transport | 0.169014 | 0.00013 |
| GO:0045454 | cell redox homeostasis | 0.169014 | 0.00013 |
| GO:0045859 | regulation of protein kinase activity | 0.082949 | 0.00013 |
| GO:0009190 | cyclic nucleotide biosynthetic process | 0.28 | 0.00013 |
| GO:0052652 | cyclic purine nucleotide metabolic process | 0.28 | 0.00013 |
| GO:0001818 | negative regulation of cytokine production | 0.109649 | 0.00013 |
| GO:0030001 | metal ion transport | 0.086331 | 0.00012 |
| GO:0035249 | synaptic transmission, glutamatergic | 0.160494 | 0.00012 |
| GO:0030427 | site of polarized growth | 0.105263 | 0.00012 |
| GO:0051093 | negative regulation of developmental process | 0.075605 | 0.00012 |
| GO:0051050 | positive regulation of transport | 0.07644 | 0.00012 |
| GO:0050817 | coagulation | 0.171429 | 0.00012 |
| GO:0007596 | blood coagulation | 0.171429 | 0.00012 |
| GO:0042304 | regulation of fatty acid biosynthetic process | 0.219512 | 0.00012 |
| GO:0098815 | modulation of excitatory postsynaptic potential | 0.219512 | 0.00012 |
| GO:0010565 | regulation of cellular ketone metabolic process | 0.140351 | 0.00011 |
| GO:0048812 | neuron projection morphogenesis | 0.103571 | 0.00011 |
| GO:0043207 | response to external biotic stimulus | 0.066764 | 0.00011 |
| GO:0043549 | regulation of kinase activity | 0.080933 | 0.00011 |
| GO:0051260 | protein homooligomerization | 0.091533 | 0.00011 |
| GO:0030426 | growth cone | 0.10687 | 0.00011 |
| GO:0010605 | negative regulation of macromolecule metabolic process | 0.061937 | 0.00010 |
| GO:0009611 | response to wounding | 0.116505 | 0.00010 |
| GO:0050820 | positive regulation of coagulation | 0.352941 | 0.00010 |
| GO:0034114 | regulation of heterotypic cell-cell adhesion | 0.352941 | 0.00010 |
| GO:0033194 | response to hydroperoxide | 0.352941 | 0.00010 |
| GO:0034663 | endoplasmic reticulum chaperone complex | 0.352941 | 0.00010 |
| GO:0071256 | translocon complex | 0.352941 | 0.00010 |
| GO:0010524 | positive regulation of calcium ion transport into cytosol | 0.454545 | 0.00009 |
| GO:0004383 | guanylate cyclase activity | 0.454545 | 0.00009 |
| GO:0048407 | platelet-derived growth factor binding | 0.454545 | 0.00009 |
| GO:0043121 | neurotrophin binding | 0.454545 | 0.00009 |
| GO:0045944 | positive regulation of transcription by RNA polymerase II | 0.074074 | 0.00009 |
| GO:0044242 | cellular lipid catabolic process | 0.118919 | 0.00009 |
| GO:0098796 | membrane protein complex | 0.070108 | 0.00009 |
| GO:0033555 | multicellular organismal response to stress | 0.138211 | 0.00009 |
| GO:0046883 | regulation of hormone secretion | 0.105839 | 0.00008 |
| GO:0048858 | cell projection morphogenesis | 0.103806 | 0.00008 |
| GO:1902652 | secondary alcohol metabolic process | 0.134328 | 0.00008 |
| GO:1990777 | lipoprotein particle | 0.230769 | 0.00008 |
| GO:0034358 | plasma lipoprotein particle | 0.230769 | 0.00008 |
| GO:1901343 | negative regulation of vasculature development | 0.159091 | 0.00008 |
| GO:0016525 | negative regulation of angiogenesis | 0.159091 | 0.00008 |
| GO:2000181 | negative regulation of blood vessel morphogenesis | 0.159091 | 0.00008 |
| GO:0051592 | response to calcium ion | 0.126582 | 0.00008 |
| GO:0043269 | regulation of ion transport | 0.083707 | 0.00008 |
| GO:0043085 | positive regulation of catalytic activity | 0.07489 | 0.00007 |
| GO:0050808 | synapse organization | 0.111588 | 0.00007 |
| GO:0071813 | lipoprotein particle binding | 0.304348 | 0.00007 |
| GO:0071814 | protein-lipid complex binding | 0.304348 | 0.00007 |
| GO:0060255 | regulation of macromolecule metabolic process | 0.056726 | 0.00007 |
| GO:0043178 | alcohol binding | 0.146789 | 0.00007 |
| GO:0120039 | plasma membrane bounded cell projection morphogenesis | 0.105263 | 0.00007 |
| GO:0031347 | regulation of defense response | 0.086003 | 0.00006 |
| GO:0071310 | cellular response to organic substance | 0.066727 | 0.00006 |
| GO:0016849 | phosphorus-oxygen lyase activity | 0.266667 | 0.00006 |
| GO:0030335 | positive regulation of cell migration | 0.090909 | 0.00006 |
| GO:0005085 | guanyl-nucleotide exchange factor activity | 0.102167 | 0.00006 |
| GO:0051641 | cellular localization | 0.065439 | 0.00006 |
| GO:0001568 | blood vessel development | 0.132867 | 0.00006 |
| GO:2000147 | positive regulation of cell motility | 0.090551 | 0.00006 |
| GO:0050708 | regulation of protein secretion | 0.106383 | 0.00006 |
| GO:0009986 | cell surface | 0.082544 | 0.00006 |
| GO:0016651 | oxidoreductase activity, acting on NAD(P)H | 0.149533 | 0.00005 |
| GO:0031838 | haptoglobin-hemoglobin complex | 0.5 | 0.00005 |
| GO:0099536 | synaptic signaling | 0.10473 | 0.00005 |
| GO:0099537 | trans-synaptic signaling | 0.10473 | 0.00005 |
| GO:0008284 | positive regulation of cell population proliferation | 0.075786 | 0.00005 |
| GO:0001938 | positive regulation of endothelial cell proliferation | 0.164706 | 0.00005 |
| GO:0040017 | positive regulation of locomotion | 0.089695 | 0.00005 |
| GO:0006805 | xenobiotic metabolic process | 0.130719 | 0.00005 |
| GO:0097494 | regulation of vesicle size | 0.1875 | 0.00005 |
| GO:0016043 | cellular component organization | 0.057637 | 0.00005 |
| GO:1904427 | positive regulation of calcium ion transmembrane transport | 0.166667 | 0.00005 |
| GO:0032990 | cell part morphogenesis | 0.102102 | 0.00004 |
| GO:0032989 | cellular component morphogenesis | 0.102102 | 0.00004 |
| GO:0030968 | endoplasmic reticulum unfolded protein response | 0.222222 | 0.00004 |
| GO:0010632 | regulation of epithelial cell migration | 0.121693 | 0.00004 |
| GO:0060284 | regulation of cell development | 0.090909 | 0.00004 |
| GO:0003756 | protein disulfide isomerase activity | 0.4 | 0.00004 |
| GO:0016864 | intramolecular oxidoreductase activity, transposing S-S bonds | 0.4 | 0.00004 |
| GO:0043408 | regulation of MAPK cascade | 0.0864 | 0.00004 |
| GO:0048878 | chemical homeostasis | 0.079121 | 0.00004 |
| GO:0009987 | cellular process | 0.051643 | 0.00004 |
| GO:0030111 | regulation of Wnt signaling pathway | 0.109023 | 0.00004 |
| GO:0010595 | positive regulation of endothelial cell migration | 0.168675 | 0.00004 |
| GO:0032535 | regulation of cellular component size | 0.104294 | 0.00004 |
| GO:0009892 | negative regulation of metabolic process | 0.062179 | 0.00004 |
| GO:0007160 | cell-matrix adhesion | 0.16129 | 0.00004 |
| GO:0010941 | regulation of cell death | 0.067773 | 0.00004 |
| GO:0008270 | zinc ion binding | 0.075521 | 0.00004 |
| GO:0023052 | signaling | 0.094505 | 0.00003 |
| GO:0055085 | transmembrane transport | 0.082487 | 0.00003 |
| GO:0050679 | positive regulation of epithelial cell proliferation | 0.127168 | 0.00003 |
| GO:0007154 | cell communication | 0.085843 | 0.00003 |
| GO:0044087 | regulation of cellular component biogenesis | 0.078864 | 0.00003 |
| GO:0008289 | lipid binding | 0.079694 | 0.00003 |
| GO:0048762 | mesenchymal cell differentiation | 0.196721 | 0.00003 |
| GO:0051036 | regulation of endosome size | 0.196721 | 0.00003 |
| GO:0005813 | centrosome | 0.085169 | 0.00003 |
| GO:0016491 | oxidoreductase activity | 0.070838 | 0.00003 |
| GO:0030947 | regulation of vascular endothelial growth factor receptor signaling pathway | 0.296296 | 0.00003 |
| GO:0034364 | high-density lipoprotein particle | 0.296296 | 0.00003 |
| GO:0040008 | regulation of growth | 0.08542 | 0.00003 |
| GO:0030169 | low-density lipoprotein particle binding | 0.428571 | 0.00003 |
| GO:0099572 | postsynaptic specialization | 0.098485 | 0.00003 |
| GO:0033036 | macromolecule localization | 0.068224 | 0.00003 |
| GO:0010035 | response to inorganic substance | 0.081019 | 0.00003 |
| GO:1903035 | negative regulation of response to wounding | 0.2 | 0.00002 |
| GO:0070727 | cellular macromolecule localization | 0.0683 | 0.00002 |
| GO:0014069 | postsynaptic density | 0.099237 | 0.00002 |
| GO:0008104 | protein localization | 0.068429 | 0.00002 |
| GO:0120035 | regulation of plasma membrane bounded cell projection organization | 0.086826 | 0.00002 |
| GO:0030155 | regulation of cell adhesion | 0.087774 | 0.00002 |
| GO:0034308 | primary alcohol metabolic process | 0.168539 | 0.00002 |
| GO:0071702 | organic substance transport | 0.068309 | 0.00002 |
| GO:0051172 | negative regulation of nitrogen compound metabolic process | 0.065691 | 0.00002 |
| GO:0050767 | regulation of neurogenesis | 0.100775 | 0.00002 |
| GO:0003674 | molecular_function | 0.050351 | 0.00002 |
| GO:0030234 | enzyme regulator activity | 0.07126 | 0.00002 |
| GO:0098590 | plasma membrane region | 0.075637 | 0.00002 |
| GO:0014070 | response to organic cyclic compound | 0.078794 | 0.00002 |
| GO:0007409 | axonogenesis | 0.126316 | 0.00002 |
| GO:0016705 | oxidoreductase activity, acting on paired donors, with incorporation or reduction of molecular oxygen | 0.109635 | 0.00002 |
| GO:0016655 | oxidoreductase activity, acting on NAD(P)H, quinone or similar compound as acceptor | 0.19403 | 0.00002 |
| GO:0010522 | regulation of calcium ion transport into cytosol | 0.461538 | 0.00002 |
| GO:0009888 | tissue development | 0.089983 | 0.00002 |
| GO:0016125 | sterol metabolic process | 0.146154 | 0.00002 |
| GO:0042578 | phosphoric ester hydrolase activity | 0.097285 | 0.00002 |
| GO:0007613 | memory | 0.147287 | 0.00001 |
| GO:0015078 | proton transmembrane transporter activity | 0.114504 | 0.00001 |
| GO:0007267 | cell-cell signaling | 0.102041 | 0.00001 |
| GO:0009605 | response to external stimulus | 0.066403 | 0.00001 |
| GO:0005577 | fibrinogen complex | 0.625 | 0.00001 |
| GO:0031994 | insulin-like growth factor I binding | 0.625 | 0.00001 |
| GO:0019222 | regulation of metabolic process | 0.057458 | 0.00001 |
| GO:1901681 | sulfur compound binding | 0.105714 | 0.00001 |
| GO:0002020 | protease binding | 0.134969 | 0.00001 |
| GO:0031344 | regulation of cell projection organization | 0.087719 | 0.00001 |
| GO:0051960 | regulation of nervous system development | 0.096436 | 0.00001 |
| GO:0043229 | intracellular organelle | 0.054105 | 0.00001 |
| GO:0004497 | monooxygenase activity | 0.120536 | 0.00001 |
| GO:0032196 | transposition | 0.25641 | 0.00001 |
| GO:0005829 | cytosol | 0.061016 | 0.00001 |
| GO:0080090 | regulation of primary metabolic process | 0.058999 | 0.00001 |
| GO:0051240 | positive regulation of multicellular organismal process | 0.072345 | 0.00001 |
| GO:0061045 | negative regulation of wound healing | 0.234043 | 0.00001 |
| GO:0019725 | cellular homeostasis | 0.090323 | 0.00001 |
| GO:0007264 | small GTPase mediated signal transduction | 0.102094 | 0.00001 |
| GO:0002682 | regulation of immune system process | 0.073342 | 0.00001 |
| GO:0120036 | plasma membrane bounded cell projection organization | 0.084249 | 0.00001 |
| GO:0043226 | organelle | 0.054701 | 0.00001 |
| GO:0010243 | response to organonitrogen compound | 0.079882 | 0.00001 |
| GO:0051171 | regulation of nitrogen compound metabolic process | 0.059153 | 0.00001 |
| GO:0097435 | supramolecular fiber organization | 0.095146 | 0.00001 |
| GO:1904018 | positive regulation of vasculature development | 0.14094 | 0.00001 |
| GO:0045766 | positive regulation of angiogenesis | 0.14094 | 0.00001 |
| GO:1904062 | regulation of cation transmembrane transport | 0.108025 | 0.00001 |
| GO:0005488 | binding | 0.055905 | 0.00001 |
| GO:0023056 | positive regulation of signaling | 0.07085 | 0.00001 |
| GO:0044093 | positive regulation of molecular function | 0.074003 | 0.00001 |
| GO:0016192 | vesicle-mediated transport | 0.077506 | 0.00001 |
| GO:0050789 | regulation of biological process | 0.062292 | 0.00001 |
| GO:0110165 | cellular anatomical entity | 0.055172 | 0.00001 |
| GO:0065007 | biological regulation | 0.062584 | 0.00001 |
| GO:0048731 | system development | 0.093458 | 0.00001 |
| GO:0043168 | anion binding | 0.064235 | 0.00001 |
| GO:0043112 | receptor metabolic process | 0.180723 | 0.00001 |
| GO:0032197 | transposition, RNA-mediated | 0.263158 | 0.00001 |
| GO:0043227 | membrane-bounded organelle | 0.065757 | 0.00001 |
| GO:0050794 | regulation of cellular process | 0.062552 | 0.00001 |
| GO:0005515 | protein binding | 0.066086 | 0.00001 |
| GO:0016020 | membrane | 0.065868 | 0.00001 |
| GO:0043231 | intracellular membrane-bounded organelle | 0.065426 | 0.00001 |
| GO:0031224 | intrinsic component of membrane | 0.064135 | 0.00001 |
| GO:0016126 | sterol biosynthetic process | 0.222222 | 0.00001 |
| GO:0098588 | bounding membrane of organelle | 0.073913 | 0.00001 |
| GO:0016021 | integral component of membrane | 0.063466 | 0.00001 |
| GO:0003824 | catalytic activity | 0.066287 | 0.00001 |
| GO:0051716 | cellular response to stimulus | 0.064251 | 0.00001 |
| GO:0051130 | positive regulation of cellular component organization | 0.080037 | 0.00001 |
| GO:0043167 | ion binding | 0.068169 | 0.00001 |
| GO:0030424 | axon | 0.09542 | 0.00001 |
| GO:0006811 | ion transport | 0.079924 | 0.00001 |
| GO:0050896 | response to stimulus | 0.069017 | 0.00001 |
| GO:0030054 | cell junction | 0.070112 | 0.00001 |
| GO:0090276 | regulation of peptide hormone secretion | 0.123853 | 0.00001 |
| GO:0048519 | negative regulation of biological process | 0.06985 | 0.00001 |
| GO:0010647 | positive regulation of cell communication | 0.071174 | 0.00001 |
| GO:0005737 | cytoplasm | 0.06362 | 0.00001 |
| GO:0048518 | positive regulation of biological process | 0.068264 | 0.00001 |
| GO:0070887 | cellular response to chemical stimulus | 0.068435 | 0.00001 |
| GO:0043169 | cation binding | 0.07237 | 0.00001 |
| GO:1903530 | regulation of secretion by cell | 0.090615 | 0.00001 |
| GO:0032502 | developmental process | 0.076435 | 0.00001 |
| GO:0046872 | metal ion binding | 0.07184 | 0.00001 |
| GO:0048583 | regulation of response to stimulus | 0.081395 | 0.00001 |
| GO:0048523 | negative regulation of cellular process | 0.072217 | 0.00001 |
| GO:0031325 | positive regulation of cellular metabolic process | 0.06773 | 0.00001 |
| GO:0048522 | positive regulation of cellular process | 0.067402 | 0.00001 |
| GO:0071248 | cellular response to metal ion | 0.127451 | 0.00001 |
| GO:0051259 | protein complex oligomerization | 0.0919 | 0.00001 |
| GO:0032501 | multicellular organismal process | 0.066383 | 0.00001 |
| GO:0097367 | carbohydrate derivative binding | 0.065564 | 0.00001 |
| GO:0006950 | response to stress | 0.071773 | 0.00001 |
| GO:0015075 | ion transmembrane transporter activity | 0.080979 | 0.00001 |
| GO:0051179 | localization | 0.07479 | 0.00001 |
| GO:0023051 | regulation of signaling | 0.082302 | 0.00001 |
| GO:0007165 | signal transduction | 0.06627 | 0.00001 |
| GO:0098772 | molecular function regulator activity | 0.070169 | 0.00001 |
| GO:0010646 | regulation of cell communication | 0.082455 | 0.00001 |
| GO:0051234 | establishment of localization | 0.074311 | 0.00001 |
| GO:0051051 | negative regulation of transport | 0.098712 | 0.00001 |
| GO:0048869 | cellular developmental process | 0.074736 | 0.00001 |
| GO:0006644 | phospholipid metabolic process | 0.104712 | 0.00001 |
| GO:0010959 | regulation of metal ion transport | 0.104712 | 0.00001 |
| GO:0016787 | hydrolase activity | 0.067645 | 0.00001 |
| GO:0009893 | positive regulation of metabolic process | 0.067047 | 0.00001 |
| GO:0006641 | triglyceride metabolic process | 0.2 | 0.00001 |
| GO:0042221 | response to chemical | 0.074515 | 0.00001 |
| GO:0051239 | regulation of multicellular organismal process | 0.084887 | 0.00001 |
| GO:0009966 | regulation of signal transduction | 0.08722 | 0.00001 |
| GO:0065009 | regulation of molecular function | 0.075026 | 0.00001 |
| GO:0019899 | enzyme binding | 0.071835 | <0.00001 |
| GO:0050790 | regulation of catalytic activity | 0.071761 | <0.00001 |
| GO:0051173 | positive regulation of nitrogen compound metabolic process | 0.071608 | <0.00001 |
| GO:0031090 | organelle membrane | 0.073042 | <0.00001 |
| GO:0006810 | transport | 0.074839 | <0.00001 |
| GO:0065008 | regulation of biological quality | 0.075394 | <0.00001 |
| GO:0048856 | anatomical structure development | 0.078034 | <0.00001 |
| GO:0042995 | cell projection | 0.084689 | <0.00001 |
| GO:0042127 | regulation of cell population proliferation | 0.073293 | <0.00001 |
| GO:0005815 | microtubule organizing center | 0.083242 | <0.00001 |
| GO:0001726 | ruffle | 0.139394 | <0.00001 |
| GO:0031982 | vesicle | 0.082033 | <0.00001 |
| GO:0051775 | response to redox state | 0.545455 | <0.00001 |
| GO:0010604 | positive regulation of macromolecule metabolic process | 0.067168 | <0.00001 |
| GO:0120025 | plasma membrane bounded cell projection | 0.086103 | <0.00001 |
| GO:0048584 | positive regulation of response to stimulus | 0.074681 | <0.00001 |
| GO:0010033 | response to organic substance | 0.072738 | <0.00001 |
| GO:0043025 | neuronal cell body | 0.09396 | <0.00001 |
| GO:0051246 | regulation of protein metabolic process | 0.074074 | <0.00001 |
| GO:0032879 | regulation of localization | 0.077737 | <0.00001 |
| GO:0051049 | regulation of transport | 0.081332 | <0.00001 |
| GO:0005739 | mitochondrion | 0.075219 | <0.00001 |
| GO:0046983 | protein dimerization activity | 0.074116 | <0.00001 |
| GO:0061041 | regulation of wound healing | 0.181818 | <0.00001 |
| GO:1901700 | response to oxygen-containing compound | 0.082237 | <0.00001 |
| GO:0009967 | positive regulation of signal transduction | 0.077251 | <0.00001 |
| GO:0006812 | cation transport | 0.089655 | <0.00001 |
| GO:0002521 | leukocyte differentiation | 0.109792 | <0.00001 |
| GO:0044877 | protein-containing complex binding | 0.075982 | <0.00001 |
| GO:1902531 | regulation of intracellular signal transduction | 0.081237 | <0.00001 |
| GO:0044281 | small molecule metabolic process | 0.07174 | <0.00001 |
| GO:0016788 | hydrolase activity, acting on ester bonds | 0.080034 | <0.00001 |
| GO:0042802 | identical protein binding | 0.071485 | <0.00001 |
| GO:0031410 | cytoplasmic vesicle | 0.085082 | <0.00001 |
| GO:0035556 | intracellular signal transduction | 0.077838 | <0.00001 |
| GO:0051094 | positive regulation of developmental process | 0.081804 | <0.00001 |
| GO:0050793 | regulation of developmental process | 0.081874 | <0.00001 |
| GO:0022857 | transmembrane transporter activity | 0.086376 | <0.00001 |
| GO:0023057 | negative regulation of signaling | 0.088674 | <0.00001 |
| GO:0022853 | active ion transmembrane transporter activity | 0.113772 | <0.00001 |
| GO:0005215 | transporter activity | 0.086308 | <0.00001 |
| GO:0046470 | phosphatidylcholine metabolic process | 0.205882 | <0.00001 |
| GO:0030154 | cell differentiation | 0.08238 | <0.00001 |
| GO:0048585 | negative regulation of response to stimulus | 0.088776 | <0.00001 |
| GO:0010648 | negative regulation of cell communication | 0.088939 | <0.00001 |
| GO:0051128 | regulation of cellular component organization | 0.076492 | <0.00001 |
| GO:0031399 | regulation of protein modification process | 0.083333 | <0.00001 |
| GO:0005789 | endoplasmic reticulum membrane | 0.101729 | <0.00001 |
| GO:0005615 | extracellular space | 0.097209 | <0.00001 |
| GO:0045937 | positive regulation of phosphate metabolic process | 0.087838 | <0.00001 |
| GO:0010562 | positive regulation of phosphorus metabolic process | 0.087838 | <0.00001 |
| GO:0007265 | Ras protein signal transduction | 0.117021 | <0.00001 |
| GO:0031966 | mitochondrial membrane | 0.087515 | <0.00001 |
| GO:0007155 | cell adhesion | 0.085353 | <0.00001 |
| GO:0042592 | homeostatic process | 0.079639 | <0.00001 |
| GO:0090087 | regulation of peptide transport | 0.126126 | <0.00001 |
| GO:0045595 | regulation of cell differentiation | 0.079662 | <0.00001 |
| GO:0005102 | signaling receptor binding | 0.080561 | <0.00001 |
| GO:0009653 | anatomical structure morphogenesis | 0.08493 | <0.00001 |
| GO:0031401 | positive regulation of protein modification process | 0.085659 | <0.00001 |
| GO:0046914 | transition metal ion binding | 0.078182 | <0.00001 |
| GO:0042325 | regulation of phosphorylation | 0.0896 | <0.00001 |
| GO:0007166 | cell surface receptor signaling pathway | 0.078991 | <0.00001 |
| GO:0022890 | inorganic cation transmembrane transporter activity | 0.09555 | <0.00001 |
| GO:0015318 | inorganic molecular entity transmembrane transporter activity | 0.086262 | <0.00001 |
| GO:0051174 | regulation of phosphorus metabolic process | 0.083221 | <0.00001 |
| GO:0019220 | regulation of phosphate metabolic process | 0.083277 | <0.00001 |
| GO:0002791 | regulation of peptide secretion | 0.126697 | <0.00001 |
| GO:0038024 | cargo receptor activity | 0.186047 | <0.00001 |
| GO:2000026 | regulation of multicellular organismal development | 0.094624 | <0.00001 |
| GO:0009968 | negative regulation of signal transduction | 0.087821 | <0.00001 |
| GO:0048513 | animal organ development | 0.083333 | <0.00001 |
| GO:0051247 | positive regulation of protein metabolic process | 0.08208 | <0.00001 |
| GO:0010038 | response to metal ion | 0.106965 | <0.00001 |
| GO:0051046 | regulation of secretion | 0.09233 | <0.00001 |
| GO:0097708 | intracellular vesicle | 0.085504 | <0.00001 |
| GO:0044297 | cell body | 0.096728 | <0.00001 |
| GO:0030030 | cell projection organization | 0.086711 | <0.00001 |
| GO:0048870 | cell motility | 0.092176 | <0.00001 |
| GO:0043005 | neuron projection | 0.088476 | <0.00001 |
| GO:0030425 | dendrite | 0.098229 | <0.00001 |
| GO:0046906 | tetrapyrrole binding | 0.116041 | <0.00001 |
| GO:0032102 | negative regulation of response to external stimulus | 0.116279 | <0.00001 |
| GO:0090066 | regulation of anatomical structure size | 0.102273 | <0.00001 |
| GO:0016477 | cell migration | 0.091584 | <0.00001 |
| GO:0080134 | regulation of response to stress | 0.086957 | <0.00001 |
| GO:0010594 | regulation of endothelial cell migration | 0.153285 | <0.00001 |
| GO:0009187 | cyclic nucleotide metabolic process | 0.268293 | <0.00001 |
| GO:0031012 | extracellular matrix | 0.16632 | <0.00001 |
| GO:0005783 | endoplasmic reticulum | 0.112447 | <0.00001 |
| GO:0032101 | regulation of response to external stimulus | 0.098544 | <0.00001 |
| GO:2000145 | regulation of cell motility | 0.092945 | <0.00001 |
| GO:0022804 | active transmembrane transporter activity | 0.10687 | <0.00001 |
| GO:1903034 | regulation of response to wounding | 0.165217 | <0.00001 |
| GO:0001932 | regulation of protein phosphorylation | 0.09319 | <0.00001 |
| GO:0016601 | Rac protein signal transduction | 0.2 | <0.00001 |
| GO:0048646 | anatomical structure formation involved in morphogenesis | 0.11126 | <0.00001 |
| GO:0042327 | positive regulation of phosphorylation | 0.09 | <0.00001 |
| GO:0006629 | lipid metabolic process | 0.095705 | <0.00001 |
| GO:0005576 | extracellular region | 0.091623 | <0.00001 |
| GO:0022603 | regulation of anatomical structure morphogenesis | 0.096019 | <0.00001 |
| GO:0019866 | organelle inner membrane | 0.093897 | <0.00001 |
| GO:0051241 | negative regulation of multicellular organismal process | 0.109631 | <0.00001 |
| GO:0090287 | regulation of cellular response to growth factor stimulus | 0.128099 | <0.00001 |
| GO:0042803 | protein homodimerization activity | 0.090472 | <0.00001 |
| GO:1901615 | organic hydroxy compound metabolic process | 0.112186 | <0.00001 |
| GO:0005604 | basement membrane | 0.166667 | <0.00001 |
| GO:0070925 | organelle assembly | 0.103093 | <0.00001 |
| GO:0008610 | lipid biosynthetic process | 0.103448 | <0.00001 |
| GO:0040012 | regulation of locomotion | 0.091392 | <0.00001 |
| GO:0001934 | positive regulation of protein phosphorylation | 0.092266 | <0.00001 |
| GO:0044255 | cellular lipid metabolic process | 0.097585 | <0.00001 |
| GO:0007275 | multicellular organism development | 0.104265 | <0.00001 |
| GO:0010817 | regulation of hormone levels | 0.098881 | <0.00001 |
| GO:0007528 | neuromuscular junction development | 0.182796 | <0.00001 |
| GO:0030334 | regulation of cell migration | 0.094787 | <0.00001 |
| GO:0007626 | locomotory behavior | 0.126761 | <0.00001 |
| GO:0044057 | regulation of system process | 0.104869 | <0.00001 |
| GO:0008324 | cation transmembrane transporter activity | 0.094595 | <0.00001 |
| GO:0005743 | mitochondrial inner membrane | 0.098616 | <0.00001 |
| GO:0001525 | angiogenesis | 0.141844 | <0.00001 |
| GO:0020037 | heme binding | 0.120567 | <0.00001 |
| GO:0006066 | alcohol metabolic process | 0.130435 | <0.00001 |
| GO:0043197 | dendritic spine | 0.130233 | <0.00001 |
| GO:0051924 | regulation of calcium ion transport | 0.125523 | <0.00001 |
| GO:0008201 | heparin binding | 0.135514 | <0.00001 |
| GO:0030027 | lamellipodium | 0.128319 | <0.00001 |
| GO:0030312 | external encapsulating structure | 0.165975 | <0.00001 |
| GO:0098794 | postsynapse | 0.119266 | <0.00001 |
| GO:0009790 | embryo development | 0.121387 | <0.00001 |
| GO:0009792 | embryo development ending in birth or egg hatching | 0.121387 | <0.00001 |
| GO:0007610 | behavior | 0.098611 | <0.00001 |
| GO:0050819 | negative regulation of coagulation | 0.282051 | <0.00001 |
| GO:0043568 | positive regulation of insulin-like growth factor receptor signaling pathway | 0.833333 | <0.00001 |
| GO:0006979 | response to oxidative stress | 0.114558 | <0.00001 |
| GO:0046486 | glycerolipid metabolic process | 0.112948 | <0.00001 |
| GO:0062023 | collagen-containing extracellular matrix | 0.190608 | <0.00001 |
| GO:0043062 | extracellular structure organization | 0.160714 | <0.00001 |
| GO:0045229 | external encapsulating structure organization | 0.161435 | <0.00001 |
| GO:0005581 | collagen trimer | 0.188889 | <0.00001 |
| GO:1900047 | negative regulation of hemostasis | 0.289474 | <0.00001 |
| GO:0006953 | acute-phase response | 0.447368 | <0.00001 |
| GO:0001881 | receptor recycling | 0.225806 | <0.00001 |
| GO:0030198 | extracellular matrix organization | 0.162162 | <0.00001 |
| GO:0140534 | endoplasmic reticulum protein-containing complex | 0.190184 | <0.00001 |
| GO:0006638 | neutral lipid metabolic process | 0.181818 | <0.00001 |
| GO:0001701 | in utero embryonic development | 0.128125 | <0.00001 |
| GO:1903169 | regulation of calcium ion transmembrane transport | 0.15894 | <0.00001 |
| GO:1900046 | regulation of hemostasis | 0.283019 | <0.00001 |
| GO:0005044 | scavenger receptor activity | 0.245283 | <0.00001 |
| GO:0009055 | electron transfer activity | 0.156757 | <0.00001 |
| GO:1901342 | regulation of vasculature development | 0.143969 | <0.00001 |
| GO:0044309 | neuron spine | 0.131818 | <0.00001 |
| GO:0006639 | acylglycerol metabolic process | 0.183673 | <0.00001 |
| GO:0045765 | regulation of angiogenesis | 0.144531 | <0.00001 |
| GO:0008202 | steroid metabolic process | 0.125 | <0.00001 |
| GO:0015399 | primary active transmembrane transporter activity | 0.128906 | <0.00001 |
| GO:0030195 | negative regulation of blood coagulation | 0.297297 | <0.00001 |
| GO:0043567 | regulation of insulin-like growth factor receptor signaling pathway | 0.444444 | <0.00001 |
| GO:0042730 | fibrinolysis | 0.615385 | <0.00001 |
| GO:0030193 | regulation of blood coagulation | 0.288462 | <0.00001 |
| GO:0030199 | collagen fibril organization | 0.340909 | <0.00001 |
| GO:0043009 | chordate embryonic development | 0.12462 | <0.00001 |
| GO:0005539 | glycosaminoglycan binding | 0.132616 | <0.00001 |
| GO:0015453 | oxidoreduction-driven active transmembrane transporter activity | 0.206897 | <0.00001 |
| GO:0016675 | oxidoreductase activity, acting on a heme group of donors | 0.27451 | <0.00001 |
| GO:0005520 | insulin-like growth factor binding | 0.391304 | <0.00001 |
| GO:0005788 | endoplasmic reticulum lumen | 0.157534 | <0.00001 |
| GO:0004129 | cytochrome-c oxidase activity | 0.28 | <0.00001 |
| GO:0002526 | acute inflammatory response | 0.253333 | <0.00001 |
| GO:0019838 | growth factor binding | 0.175325 | <0.00001 |
| GO:0050818 | regulation of coagulation | 0.277778 | <0.00001 |

Rich factor: The ratio of the number of genes belonging to this Term in the target gene set to the number of all genes belonging to this Term in the background gene set.
