# Supplementary material for: Multi-time point transcriptomics and metabolomics reveal key transcription and metabolic features of hepatic ischemia-reperfusion injury in mice
Source: Genes Dis. 2024 Nov 17;12(2):101465. doi: 10.1016/j.gendis.2024.101465 (PMC11697123; doi:10.1016/j.gendis.2024.101465)
Supplement: Multimedia component 6 [file mmc6.docx]

**Table S2C.** The GO terms of differentially expressed genes (DEGs) identified by Gene Ontology (GO) in the Sham and I1R48 groups.

| **GO ID** | **GO term description** | **Rich factor** | **P-value** |
| --- | --- | --- | --- |
| GO:0120161 | regulation of cold-induced thermogenesis | 0.137097 | 0.04924 |
| GO:0007340 | acrosome reaction | 0.235294 | 0.04923 |
| GO:0035999 | tetrahydrofolate interconversion | 0.235294 | 0.04923 |
| GO:0070293 | renal absorption | 0.235294 | 0.04923 |
| GO:0061326 | renal tubule development | 0.235294 | 0.04923 |
| GO:0072359 | circulatory system development | 0.235294 | 0.04923 |
| GO:0060416 | response to growth hormone | 0.235294 | 0.04923 |
| GO:0014002 | astrocyte development | 0.235294 | 0.04923 |
| GO:0002053 | positive regulation of mesenchymal cell proliferation | 0.235294 | 0.04923 |
| GO:0006825 | copper ion transport | 0.235294 | 0.04923 |
| GO:0034143 | regulation of toll-like receptor 4 signaling pathway | 0.235294 | 0.04923 |
| GO:0060996 | dendritic spine development | 0.235294 | 0.04923 |
| GO:0009404 | toxin metabolic process | 0.235294 | 0.04923 |
| GO:0003208 | cardiac ventricle morphogenesis | 0.235294 | 0.04923 |
| GO:0042982 | amyloid precursor protein metabolic process | 0.235294 | 0.04923 |
| GO:1903055 | positive regulation of extracellular matrix organization | 0.235294 | 0.04923 |
| GO:0034472 | snRNA 3'-end processing | 0.235294 | 0.04923 |
| GO:0017121 | plasma membrane phospholipid scrambling | 0.235294 | 0.04923 |
| GO:0048499 | synaptic vesicle membrane organization | 0.235294 | 0.04923 |
| GO:0048643 | positive regulation of skeletal muscle tissue development | 0.235294 | 0.04923 |
| GO:0014829 | vascular associated smooth muscle contraction | 0.235294 | 0.04923 |
| GO:0090533 | cation-transporting ATPase complex | 0.235294 | 0.04923 |
| GO:0016717 | oxidoreductase activity, acting on paired donors, with oxidation of a pair of donors resulting in the reduction of molecular oxygen to two molecules of water | 0.235294 | 0.04923 |
| GO:0043023 | ribosomal large subunit binding | 0.235294 | 0.04923 |
| GO:0005381 | iron ion transmembrane transporter activity | 0.235294 | 0.04923 |
| GO:0061061 | muscle structure development | 0.149425 | 0.04922 |
| GO:0005507 | copper ion binding | 0.149425 | 0.04922 |
| GO:0009897 | external side of plasma membrane | 0.107639 | 0.04910 |
| GO:0018105 | peptidyl-serine phosphorylation | 0.131944 | 0.04902 |
| GO:0044262 | cellular carbohydrate metabolic process | 0.125654 | 0.04877 |
| GO:0051180 | vitamin transport | 0.1875 | 0.04824 |
| GO:0014741 | negative regulation of muscle hypertrophy | 0.1875 | 0.04824 |
| GO:0045721 | negative regulation of gluconeogenesis | 0.1875 | 0.04824 |
| GO:1902475 | L-alpha-amino acid transmembrane transport | 0.1875 | 0.04824 |
| GO:1904407 | positive regulation of nitric oxide metabolic process | 0.1875 | 0.04824 |
| GO:0048873 | homeostasis of number of cells within a tissue | 0.1875 | 0.04824 |
| GO:0046320 | regulation of fatty acid oxidation | 0.1875 | 0.04824 |
| GO:0032839 | dendrite cytoplasm | 0.1875 | 0.04824 |
| GO:0051019 | mitogen-activated protein kinase binding | 0.1875 | 0.04824 |
| GO:0005261 | cation channel activity | 0.113514 | 0.04786 |
| GO:0010631 | epithelial cell migration | 0.157143 | 0.04731 |
| GO:0032507 | maintenance of protein location in cell | 0.157143 | 0.04731 |
| GO:0008154 | actin polymerization or depolymerization | 0.157143 | 0.04731 |
| GO:0034704 | calcium channel complex | 0.157143 | 0.04731 |
| GO:1901264 | carbohydrate derivative transport | 0.163636 | 0.04710 |
| GO:0031113 | regulation of microtubule polymerization | 0.163636 | 0.04710 |
| GO:0044790 | suppression of viral release by host | 0.163636 | 0.04710 |
| GO:0042162 | telomeric DNA binding | 0.163636 | 0.04710 |
| GO:1903900 | regulation of viral life cycle | 0.128834 | 0.04708 |
| GO:0014074 | response to purine-containing compound | 0.128834 | 0.04708 |
| GO:0099568 | cytoplasmic region | 0.128834 | 0.04708 |
| GO:0001101 | response to acid chemical | 0.120833 | 0.04696 |
| GO:0002699 | positive regulation of immune effector process | 0.120833 | 0.04696 |
| GO:0071869 | response to catecholamine | 0.208333 | 0.04657 |
| GO:0040036 | regulation of fibroblast growth factor receptor signaling pathway | 0.208333 | 0.04657 |
| GO:0042753 | positive regulation of circadian rhythm | 0.208333 | 0.04657 |
| GO:0046856 | phosphatidylinositol dephosphorylation | 0.208333 | 0.04657 |
| GO:0031061 | negative regulation of histone methylation | 0.208333 | 0.04657 |
| GO:0010667 | negative regulation of cardiac muscle cell apoptotic process | 0.208333 | 0.04657 |
| GO:0043534 | blood vessel endothelial cell migration | 0.208333 | 0.04657 |
| GO:0034706 | sodium channel complex | 0.208333 | 0.04657 |
| GO:0031233 | intrinsic component of external side of plasma membrane | 0.208333 | 0.04657 |
| GO:0015296 | anion:cation symporter activity | 0.208333 | 0.04657 |
| GO:0008344 | adult locomotory behavior | 0.141414 | 0.04632 |
| GO:0015291 | secondary active transmembrane transporter activity | 0.119403 | 0.04610 |
| GO:0099641 | anterograde axonal protein transport | 0.3 | 0.04570 |
| GO:0150172 | regulation of phosphatidylcholine metabolic process | 0.3 | 0.04570 |
| GO:0021819 | layer formation in cerebral cortex | 0.3 | 0.04570 |
| GO:0042268 | regulation of cytolysis | 0.3 | 0.04570 |
| GO:0060100 | positive regulation of phagocytosis, engulfment | 0.3 | 0.04570 |
| GO:0014745 | negative regulation of muscle adaptation | 0.3 | 0.04570 |
| GO:0006558 | L-phenylalanine metabolic process | 0.3 | 0.04570 |
| GO:0060315 | negative regulation of ryanodine-sensitive calcium-release channel activity | 0.3 | 0.04570 |
| GO:0098598 | learned vocalization behavior or vocal learning | 0.3 | 0.04570 |
| GO:0048485 | sympathetic nervous system development | 0.3 | 0.04570 |
| GO:0006032 | chitin catabolic process | 0.3 | 0.04570 |
| GO:1903624 | regulation of DNA catabolic process | 0.3 | 0.04570 |
| GO:0045876 | positive regulation of sister chromatid cohesion | 0.3 | 0.04570 |
| GO:0060049 | regulation of protein glycosylation | 0.3 | 0.04570 |
| GO:0060081 | membrane hyperpolarization | 0.3 | 0.04570 |
| GO:0010171 | body morphogenesis | 0.3 | 0.04570 |
| GO:1900116 | extracellular negative regulation of signal transduction | 0.3 | 0.04570 |
| GO:1900115 | extracellular regulation of signal transduction | 0.3 | 0.04570 |
| GO:0043485 | endosome to pigment granule transport | 0.3 | 0.04570 |
| GO:1902221 | erythrose 4-phosphate/phosphoenolpyruvate family amino acid metabolic process | 0.3 | 0.04570 |
| GO:0045602 | negative regulation of endothelial cell differentiation | 0.3 | 0.04570 |
| GO:0006857 | oligopeptide transport | 0.3 | 0.04570 |
| GO:0001941 | postsynaptic membrane organization | 0.3 | 0.04570 |
| GO:0072178 | nephric duct morphogenesis | 0.3 | 0.04570 |
| GO:0006782 | protoporphyrinogen IX biosynthetic process | 0.3 | 0.04570 |
| GO:0010875 | positive regulation of cholesterol efflux | 0.3 | 0.04570 |
| GO:0048745 | smooth muscle tissue development | 0.3 | 0.04570 |
| GO:0035646 | endosome to melanosome transport | 0.3 | 0.04570 |
| GO:0032000 | positive regulation of fatty acid beta-oxidation | 0.3 | 0.04570 |
| GO:0097756 | obsolete negative regulation of blood vessel diameter | 0.3 | 0.04570 |
| GO:1901029 | negative regulation of mitochondrial outer membrane permeabilization involved in apoptotic signaling pathway | 0.3 | 0.04570 |
| GO:0019511 | peptidyl-proline hydroxylation | 0.3 | 0.04570 |
| GO:0030277 | maintenance of gastrointestinal epithelium | 0.3 | 0.04570 |
| GO:0090385 | phagosome-lysosome fusion | 0.3 | 0.04570 |
| GO:1905155 | positive regulation of membrane invagination | 0.3 | 0.04570 |
| GO:1905383 | protein localization to presynapse | 0.3 | 0.04570 |
| GO:0045821 | positive regulation of glycolytic process | 0.3 | 0.04570 |
| GO:1900102 | negative regulation of endoplasmic reticulum unfolded protein response | 0.3 | 0.04570 |
| GO:1902931 | negative regulation of alcohol biosynthetic process | 0.3 | 0.04570 |
| GO:0017059 | serine C-palmitoyltransferase complex | 0.3 | 0.04570 |
| GO:0031211 | endoplasmic reticulum palmitoyltransferase complex | 0.3 | 0.04570 |
| GO:0005787 | signal peptidase complex | 0.3 | 0.04570 |
| GO:0033646 | host intracellular part | 0.3 | 0.04570 |
| GO:0019869 | chloride channel inhibitor activity | 0.3 | 0.04570 |
| GO:0016670 | oxidoreductase activity, acting on a sulfur group of donors, oxygen as acceptor | 0.3 | 0.04570 |
| GO:0000700 | mismatch base pair DNA N-glycosylase activity | 0.3 | 0.04570 |
| GO:0034452 | dynactin binding | 0.3 | 0.04570 |
| GO:0016215 | acyl-CoA desaturase activity | 0.3 | 0.04570 |
| GO:0048495 | Roundabout binding | 0.3 | 0.04570 |
| GO:0042979 | ornithine decarboxylase regulator activity | 0.3 | 0.04570 |
| GO:0071333 | cellular response to glucose stimulus | 0.15942 | 0.04569 |
| GO:0097729 | 9+2 motile cilium | 0.15942 | 0.04569 |
| GO:0022607 | cellular component assembly | 0.092662 | 0.04528 |
| GO:0043255 | regulation of carbohydrate biosynthetic process | 0.135714 | 0.04520 |
| GO:0048306 | calcium-dependent protein binding | 0.148148 | 0.04509 |
| GO:0052547 | regulation of peptidase activity | 0.107203 | 0.04508 |
| GO:0008033 | tRNA processing | 0.130435 | 0.04488 |
| GO:0006473 | protein acetylation | 0.130435 | 0.04488 |
| GO:0030545 | signaling receptor regulator activity | 0.105797 | 0.04483 |
| GO:0099177 | regulation of trans-synaptic signaling | 0.107563 | 0.04445 |
| GO:0045833 | negative regulation of lipid metabolic process | 0.142857 | 0.04419 |
| GO:0048015 | phosphatidylinositol-mediated signaling | 0.166667 | 0.04396 |
| GO:0014704 | intercalated disc | 0.166667 | 0.04396 |
| GO:0120013 | lipid transfer activity | 0.166667 | 0.04396 |
| GO:0003012 | muscle system process | 0.125 | 0.04390 |
| GO:0034702 | ion channel complex | 0.117647 | 0.04359 |
| GO:0097193 | intrinsic apoptotic signaling pathway | 0.132075 | 0.04317 |
| GO:0001817 | regulation of cytokine production | 0.103292 | 0.04304 |
| GO:0036064 | ciliary basal body | 0.127778 | 0.04268 |
| GO:0030036 | actin cytoskeleton organization | 0.113695 | 0.04257 |
| GO:0015267 | channel activity | 0.109057 | 0.04243 |
| GO:0022803 | passive transmembrane transporter activity | 0.109057 | 0.04243 |
| GO:0042886 | amide transport | 0.13913 | 0.04226 |
| GO:0008652 | cellular amino acid biosynthetic process | 0.13913 | 0.04226 |
| GO:0070279 | vitamin B6 binding | 0.14433 | 0.04224 |
| GO:0033628 | regulation of cell adhesion mediated by integrin | 0.179487 | 0.04222 |
| GO:0006084 | acetyl-CoA metabolic process | 0.179487 | 0.04222 |
| GO:0048500 | signal recognition particle | 0.179487 | 0.04222 |
| GO:0098799 | outer mitochondrial membrane protein complex | 0.179487 | 0.04222 |
| GO:0007492 | endoderm development | 0.193548 | 0.04209 |
| GO:1905606 | regulation of presynapse assembly | 0.193548 | 0.04209 |
| GO:0009065 | glutamine family amino acid catabolic process | 0.193548 | 0.04209 |
| GO:0009886 | post-embryonic animal morphogenesis | 0.193548 | 0.04209 |
| GO:0030316 | osteoclast differentiation | 0.193548 | 0.04209 |
| GO:0031670 | cellular response to nutrient | 0.193548 | 0.04209 |
| GO:0002230 | positive regulation of defense response to virus by host | 0.193548 | 0.04209 |
| GO:0045429 | positive regulation of nitric oxide biosynthetic process | 0.193548 | 0.04209 |
| GO:0044305 | calyx of Held | 0.193548 | 0.04209 |
| GO:0050681 | nuclear androgen receptor binding | 0.193548 | 0.04209 |
| GO:0030855 | epithelial cell differentiation | 0.116719 | 0.04196 |
| GO:0003682 | chromatin binding | 0.10585 | 0.04174 |
| GO:0005096 | GTPase activator activity | 0.115274 | 0.04129 |
| GO:0045661 | regulation of myoblast differentiation | 0.169811 | 0.04115 |
| GO:0005048 | signal sequence binding | 0.169811 | 0.04115 |
| GO:0046467 | membrane lipid biosynthetic process | 0.135338 | 0.04107 |
| GO:0044325 | transmembrane transporter binding | 0.135338 | 0.04107 |
| GO:2001244 | positive regulation of intrinsic apoptotic signaling pathway | 0.15873 | 0.04093 |
| GO:0032482 | Rab protein signal transduction | 0.15873 | 0.04093 |
| GO:0008483 | transaminase activity | 0.15873 | 0.04093 |
| GO:0071478 | cellular response to radiation | 0.129213 | 0.04092 |
| GO:2000058 | regulation of ubiquitin-dependent protein catabolic process | 0.129213 | 0.04092 |
| GO:0007399 | nervous system development | 0.129213 | 0.04092 |
| GO:0004521 | endoribonuclease activity | 0.129213 | 0.04092 |
| GO:1902106 | negative regulation of leukocyte differentiation | 0.140351 | 0.04075 |
| GO:0050728 | negative regulation of inflammatory response | 0.140351 | 0.04075 |
| GO:0061387 | regulation of extent of cell growth | 0.140351 | 0.04075 |
| GO:0045666 | positive regulation of neuron differentiation | 0.145833 | 0.04048 |
| GO:0009791 | post-embryonic development | 0.145833 | 0.04048 |
| GO:0004713 | protein tyrosine kinase activity | 0.131579 | 0.04048 |
| GO:0007010 | cytoskeleton organization | 0.10091 | 0.04035 |
| GO:0006684 | sphingomyelin metabolic process | 0.25 | 0.04025 |
| GO:0034111 | negative regulation of homotypic cell-cell adhesion | 0.25 | 0.04025 |
| GO:1902036 | regulation of hematopoietic stem cell differentiation | 0.25 | 0.04025 |
| GO:1903909 | regulation of receptor clustering | 0.25 | 0.04025 |
| GO:0002864 | regulation of acute inflammatory response to antigenic stimulus | 0.25 | 0.04025 |
| GO:0038084 | vascular endothelial growth factor signaling pathway | 0.25 | 0.04025 |
| GO:0033189 | response to vitamin A | 0.25 | 0.04025 |
| GO:0002011 | morphogenesis of an epithelial sheet | 0.25 | 0.04025 |
| GO:0072080 | nephron tubule development | 0.25 | 0.04025 |
| GO:0060211 | regulation of nuclear-transcribed mRNA poly(A) tail shortening | 0.25 | 0.04025 |
| GO:0042048 | olfactory behavior | 0.25 | 0.04025 |
| GO:0035864 | response to potassium ion | 0.25 | 0.04025 |
| GO:0034755 | iron ion transmembrane transport | 0.25 | 0.04025 |
| GO:0002710 | negative regulation of T cell mediated immunity | 0.25 | 0.04025 |
| GO:0030137 | COPI-coated vesicle | 0.25 | 0.04025 |
| GO:0031314 | extrinsic component of mitochondrial inner membrane | 0.25 | 0.04025 |
| GO:0003951 | NAD+ kinase activity | 0.25 | 0.04025 |
| GO:0016411 | acylglycerol O-acyltransferase activity | 0.25 | 0.04025 |
| GO:0001851 | complement component C3b binding | 0.25 | 0.04025 |
| GO:0008061 | chitin binding | 0.25 | 0.04025 |
| GO:0030175 | filopodium | 0.151899 | 0.04024 |
| GO:0007389 | pattern specification process | 0.114362 | 0.03983 |
| GO:0005791 | rough endoplasmic reticulum | 0.136364 | 0.03976 |
| GO:0030534 | adult behavior | 0.130682 | 0.03958 |
| GO:0006874 | cellular calcium ion homeostasis | 0.130682 | 0.03958 |
| GO:0006308 | DNA catabolic process | 0.217391 | 0.03952 |
| GO:0070861 | regulation of protein exit from endoplasmic reticulum | 0.217391 | 0.03952 |
| GO:0006882 | cellular zinc ion homeostasis | 0.217391 | 0.03952 |
| GO:2000269 | regulation of fibroblast apoptotic process | 0.217391 | 0.03952 |
| GO:0030647 | aminoglycoside antibiotic metabolic process | 0.217391 | 0.03952 |
| GO:0010880 | regulation of release of sequestered calcium ion into cytosol by sarcoplasmic reticulum | 0.217391 | 0.03952 |
| GO:0015721 | bile acid and bile salt transport | 0.217391 | 0.03952 |
| GO:0097428 | protein maturation by iron-sulfur cluster transfer | 0.217391 | 0.03952 |
| GO:0010226 | response to lithium ion | 0.217391 | 0.03952 |
| GO:2000251 | positive regulation of actin cytoskeleton reorganization | 0.217391 | 0.03952 |
| GO:0045589 | regulation of regulatory T cell differentiation | 0.217391 | 0.03952 |
| GO:0005740 | mitochondrial envelope | 0.217391 | 0.03952 |
| GO:0005112 | Notch binding | 0.217391 | 0.03952 |
| GO:0035014 | phosphatidylinositol 3-kinase regulator activity | 0.217391 | 0.03952 |
| GO:0007204 | positive regulation of cytosolic calcium ion concentration | 0.126263 | 0.03945 |
| GO:0016879 | ligase activity, forming carbon-nitrogen bonds | 0.147368 | 0.03891 |
| GO:0030170 | pyridoxal phosphate binding | 0.147368 | 0.03891 |
| GO:0032963 | collagen metabolic process | 0.173077 | 0.03867 |
| GO:0008028 | monocarboxylic acid transmembrane transporter activity | 0.173077 | 0.03867 |
| GO:0009612 | response to mechanical stimulus | 0.126904 | 0.03861 |
| GO:0035148 | tube formation | 0.137405 | 0.03859 |
| GO:0030595 | leukocyte chemotaxis | 0.137405 | 0.03859 |
| GO:0061698 | protein deglutarylation | 0.153846 | 0.03814 |
| GO:0036046 | protein demalonylation | 0.153846 | 0.03814 |
| GO:0036047 | peptidyl-lysine demalonylation | 0.153846 | 0.03814 |
| GO:0061697 | protein-glutaryllysine deglutarylase activity | 0.153846 | 0.03814 |
| GO:0036054 | protein-malonyllysine demalonylase activity | 0.153846 | 0.03814 |
| GO:0036055 | protein-succinyllysine desuccinylase activity | 0.153846 | 0.03814 |
| GO:1903305 | regulation of regulated secretory pathway | 0.133333 | 0.03805 |
| GO:0017124 | SH3 domain binding | 0.133333 | 0.03805 |
| GO:0045650 | negative regulation of macrophage differentiation | 0.5 | 0.03794 |
| GO:0070508 | cholesterol import | 0.5 | 0.03794 |
| GO:0090071 | negative regulation of ribosome biogenesis | 0.5 | 0.03794 |
| GO:1902396 | protein localization to bicellular tight junction | 0.5 | 0.03794 |
| GO:2000669 | negative regulation of dendritic cell apoptotic process | 0.5 | 0.03794 |
| GO:0071499 | cellular response to laminar fluid shear stress | 0.5 | 0.03794 |
| GO:0032696 | negative regulation of interleukin-13 production | 0.5 | 0.03794 |
| GO:0002143 | tRNA wobble position uridine thiolation | 0.5 | 0.03794 |
| GO:0043152 | induction of bacterial agglutination | 0.5 | 0.03794 |
| GO:1905749 | regulation of endosome to plasma membrane protein transport | 0.5 | 0.03794 |
| GO:0097114 | NMDA glutamate receptor clustering | 0.5 | 0.03794 |
| GO:0034635 | glutathione transport | 0.5 | 0.03794 |
| GO:0099502 | calcium-dependent activation of synaptic vesicle fusion | 0.5 | 0.03794 |
| GO:1902177 | positive regulation of oxidative stress-induced intrinsic apoptotic signaling pathway | 0.5 | 0.03794 |
| GO:2000566 | positive regulation of CD8-positive, alpha-beta T cell proliferation | 0.5 | 0.03794 |
| GO:0060087 | relaxation of vascular associated smooth muscle | 0.5 | 0.03794 |
| GO:0006578 | amino-acid betaine biosynthetic process | 0.5 | 0.03794 |
| GO:0043137 | DNA replication, removal of RNA primer | 0.5 | 0.03794 |
| GO:0033087 | negative regulation of immature T cell proliferation | 0.5 | 0.03794 |
| GO:0032447 | protein urmylation | 0.5 | 0.03794 |
| GO:0072176 | nephric duct development | 0.5 | 0.03794 |
| GO:1901529 | positive regulation of anion channel activity | 0.5 | 0.03794 |
| GO:0060831 | smoothened signaling pathway involved in dorsal/ventral neural tube patterning | 0.5 | 0.03794 |
| GO:0033602 | negative regulation of dopamine secretion | 0.5 | 0.03794 |
| GO:0035509 | negative regulation of myosin-light-chain-phosphatase activity | 0.5 | 0.03794 |
| GO:0015917 | aminophospholipid transport | 0.5 | 0.03794 |
| GO:0010193 | response to ozone | 0.5 | 0.03794 |
| GO:2000427 | positive regulation of apoptotic cell clearance | 0.5 | 0.03794 |
| GO:0090494 | dopamine uptake | 0.5 | 0.03794 |
| GO:0090493 | catecholamine uptake | 0.5 | 0.03794 |
| GO:0002408 | myeloid dendritic cell chemotaxis | 0.5 | 0.03794 |
| GO:0042471 | ear morphogenesis | 0.5 | 0.03794 |
| GO:0009609 | response to symbiotic bacterium | 0.5 | 0.03794 |
| GO:0009608 | response to symbiont | 0.5 | 0.03794 |
| GO:0032971 | regulation of muscle filament sliding | 0.5 | 0.03794 |
| GO:2000348 | regulation of CD40 signaling pathway | 0.5 | 0.03794 |
| GO:0010745 | negative regulation of macrophage derived foam cell differentiation | 0.5 | 0.03794 |
| GO:2000809 | positive regulation of synaptic vesicle clustering | 0.5 | 0.03794 |
| GO:0048752 | semicircular canal morphogenesis | 0.5 | 0.03794 |
| GO:2000110 | negative regulation of macrophage apoptotic process | 0.5 | 0.03794 |
| GO:0010727 | negative regulation of hydrogen peroxide metabolic process | 0.5 | 0.03794 |
| GO:0061668 | mitochondrial ribosome assembly | 0.5 | 0.03794 |
| GO:0090069 | regulation of ribosome biogenesis | 0.5 | 0.03794 |
| GO:0032687 | negative regulation of interferon-alpha production | 0.5 | 0.03794 |
| GO:0044557 | relaxation of smooth muscle | 0.5 | 0.03794 |
| GO:1901256 | regulation of macrophage colony-stimulating factor production | 0.5 | 0.03794 |
| GO:0031946 | regulation of glucocorticoid biosynthetic process | 0.5 | 0.03794 |
| GO:0051964 | negative regulation of synapse assembly | 0.5 | 0.03794 |
| GO:0051771 | negative regulation of nitric-oxide synthase biosynthetic process | 0.5 | 0.03794 |
| GO:0071461 | cellular response to redox state | 0.5 | 0.03794 |
| GO:1902309 | negative regulation of peptidyl-serine dephosphorylation | 0.5 | 0.03794 |
| GO:1905653 | positive regulation of artery morphogenesis | 0.5 | 0.03794 |
| GO:1905651 | regulation of artery morphogenesis | 0.5 | 0.03794 |
| GO:0010571 | positive regulation of nuclear cell cycle DNA replication | 0.5 | 0.03794 |
| GO:0070318 | positive regulation of G0 to G1 transition | 0.5 | 0.03794 |
| GO:1905671 | regulation of lysosome organization | 0.5 | 0.03794 |
| GO:0021692 | cerebellar Purkinje cell layer morphogenesis | 0.5 | 0.03794 |
| GO:0045843 | negative regulation of striated muscle tissue development | 0.5 | 0.03794 |
| GO:0008612 | peptidyl-lysine modification to peptidyl-hypusine | 0.5 | 0.03794 |
| GO:0014053 | negative regulation of gamma-aminobutyric acid secretion | 0.5 | 0.03794 |
| GO:0031632 | positive regulation of synaptic vesicle fusion to presynaptic active zone membrane | 0.5 | 0.03794 |
| GO:1900222 | negative regulation of amyloid-beta clearance | 0.5 | 0.03794 |
| GO:1901634 | positive regulation of synaptic vesicle membrane organization | 0.5 | 0.03794 |
| GO:0000379 | tRNA-type intron splice site recognition and cleavage | 0.5 | 0.03794 |
| GO:0042939 | tripeptide transport | 0.5 | 0.03794 |
| GO:0072733 | response to staurosporine | 0.5 | 0.03794 |
| GO:0072734 | cellular response to staurosporine | 0.5 | 0.03794 |
| GO:0045829 | negative regulation of isotype switching | 0.5 | 0.03794 |
| GO:0046959 | habituation | 0.5 | 0.03794 |
| GO:1900108 | negative regulation of nodal signaling pathway | 0.5 | 0.03794 |
| GO:0097680 | double-strand break repair via classical nonhomologous end joining | 0.5 | 0.03794 |
| GO:0072177 | mesonephric duct development | 0.5 | 0.03794 |
| GO:0042998 | positive regulation of Golgi to plasma membrane protein transport | 0.5 | 0.03794 |
| GO:0097116 | gephyrin clustering involved in postsynaptic density assembly | 0.5 | 0.03794 |
| GO:0009449 | gamma-aminobutyric acid biosynthetic process | 0.5 | 0.03794 |
| GO:1901843 | positive regulation of high voltage-gated calcium channel activity | 0.5 | 0.03794 |
| GO:0051919 | positive regulation of fibrinolysis | 0.5 | 0.03794 |
| GO:0019376 | galactolipid catabolic process | 0.5 | 0.03794 |
| GO:0008275 | gamma-tubulin small complex | 0.5 | 0.03794 |
| GO:0005610 | laminin-5 complex | 0.5 | 0.03794 |
| GO:0033063 | Rad51B-Rad51C-Rad51D-XRCC2 complex | 0.5 | 0.03794 |
| GO:0072558 | NLRP1 inflammasome complex | 0.5 | 0.03794 |
| GO:0070033 | synaptobrevin 2-SNAP-25-syntaxin-1a-complexin II complex | 0.5 | 0.03794 |
| GO:0036488 | CHOP-C/EBP complex | 0.5 | 0.03794 |
| GO:0045180 | basal cortex | 0.5 | 0.03794 |
| GO:0097169 | AIM2 inflammasome complex | 0.5 | 0.03794 |
| GO:0042709 | succinate-CoA ligase complex | 0.5 | 0.03794 |
| GO:0035859 | Seh1-associated complex | 0.5 | 0.03794 |
| GO:0035867 | alphav-beta3 integrin-IGF-1-IGF1R complex | 0.5 | 0.03794 |
| GO:0004446 | inositol-hexakisphosphate phosphatase activity | 0.5 | 0.03794 |
| GO:0098519 | obsolete nucleotide phosphatase activity, acting on free nucleotides | 0.5 | 0.03794 |
| GO:0000293 | ferric-chelate reductase activity | 0.5 | 0.03794 |
| GO:0034417 | bisphosphoglycerate 3-phosphatase activity | 0.5 | 0.03794 |
| GO:0030984 | kininogen binding | 0.5 | 0.03794 |
| GO:0035594 | ganglioside binding | 0.5 | 0.03794 |
| GO:0032139 | dinucleotide insertion or deletion binding | 0.5 | 0.03794 |
| GO:0031014 | troponin T binding | 0.5 | 0.03794 |
| GO:0032422 | purine-rich negative regulatory element binding | 0.5 | 0.03794 |
| GO:0004656 | procollagen-proline 4-dioxygenase activity | 0.5 | 0.03794 |
| GO:0004774 | succinate-CoA ligase activity | 0.5 | 0.03794 |
| GO:0034338 | short-chain carboxylesterase activity | 0.5 | 0.03794 |
| GO:0016882 | cyclo-ligase activity | 0.5 | 0.03794 |
| GO:0004530 | deoxyribonuclease I activity | 0.5 | 0.03794 |
| GO:0052658 | inositol-1,4,5-trisphosphate 5-phosphatase activity | 0.5 | 0.03794 |
| GO:0016300 | tRNA (uracil) methyltransferase activity | 0.5 | 0.03794 |
| GO:0004305 | ethanolamine kinase activity | 0.5 | 0.03794 |
| GO:0051430 | corticotropin-releasing hormone receptor 1 binding | 0.5 | 0.03794 |
| GO:0043532 | angiostatin binding | 0.5 | 0.03794 |
| GO:0004487 | methylenetetrahydrofolate dehydrogenase (NAD+) activity | 0.5 | 0.03794 |
| GO:0052826 | inositol hexakisphosphate 2-phosphatase activity | 0.5 | 0.03794 |
| GO:0004366 | glycerol-3-phosphate O-acyltransferase activity | 0.5 | 0.03794 |
| GO:0102420 | sn-1-glycerol-3-phosphate C16:0-DCA-CoA acyl transferase activity | 0.5 | 0.03794 |
| GO:0000213 | tRNA-intron endonuclease activity | 0.5 | 0.03794 |
| GO:0046631 | alpha-beta T cell activation | 0.16129 | 0.03786 |
| GO:0048839 | inner ear development | 0.16129 | 0.03786 |
| GO:0016706 | 2-oxoglutarate-dependent dioxygenase activity | 0.16129 | 0.03786 |
| GO:0006508 | proteolysis | 0.099927 | 0.03783 |
| GO:0070585 | protein localization to mitochondrion | 0.148936 | 0.03752 |
| GO:0033119 | negative regulation of RNA splicing | 0.184211 | 0.03726 |
| GO:0006611 | protein export from nucleus | 0.184211 | 0.03726 |
| GO:0010939 | regulation of necrotic cell death | 0.184211 | 0.03726 |
| GO:0007044 | cell-substrate junction assembly | 0.184211 | 0.03726 |
| GO:0150115 | cell-substrate junction organization | 0.184211 | 0.03726 |
| GO:0005742 | mitochondrial outer membrane translocase complex | 0.184211 | 0.03726 |
| GO:0005501 | retinoid binding | 0.184211 | 0.03726 |
| GO:0070003 | threonine-type peptidase activity | 0.184211 | 0.03726 |
| GO:1900180 | regulation of protein localization to nucleus | 0.144144 | 0.03717 |
| GO:0048705 | skeletal system morphogenesis | 0.144144 | 0.03717 |
| GO:0030135 | coated vesicle | 0.134228 | 0.03701 |
| GO:0044770 | cell cycle phase transition | 0.130178 | 0.03697 |
| GO:0009117 | nucleotide metabolic process | 0.10303 | 0.03693 |
| GO:0061138 | morphogenesis of a branching epithelium | 0.139535 | 0.03663 |
| GO:0019213 | deacetylase activity | 0.139535 | 0.03663 |
| GO:0046579 | positive regulation of Ras protein signal transduction | 0.176471 | 0.03653 |
| GO:0031062 | positive regulation of histone methylation | 0.176471 | 0.03653 |
| GO:0005801 | cis-Golgi network | 0.176471 | 0.03653 |
| GO:0060142 | regulation of syncytium formation by plasma membrane fusion | 0.2 | 0.03645 |
| GO:0032369 | negative regulation of lipid transport | 0.2 | 0.03645 |
| GO:0010614 | negative regulation of cardiac muscle hypertrophy | 0.2 | 0.03645 |
| GO:0031294 | lymphocyte costimulation | 0.2 | 0.03645 |
| GO:0090314 | positive regulation of protein targeting to membrane | 0.2 | 0.03645 |
| GO:0007616 | long-term memory | 0.2 | 0.03645 |
| GO:0048240 | sperm capacitation | 0.2 | 0.03645 |
| GO:0098760 | response to interleukin-7 | 0.2 | 0.03645 |
| GO:0098761 | cellular response to interleukin-7 | 0.2 | 0.03645 |
| GO:0004532 | exoribonuclease activity | 0.2 | 0.03645 |
| GO:0030371 | translation repressor activity | 0.2 | 0.03645 |
| GO:0017157 | regulation of exocytosis | 0.125 | 0.03632 |
| GO:0001678 | cellular glucose homeostasis | 0.150538 | 0.03632 |
| GO:0032526 | response to retinoic acid | 0.150538 | 0.03632 |
| GO:0032587 | ruffle membrane | 0.150538 | 0.03632 |
| GO:0005901 | caveola | 0.150538 | 0.03632 |
| GO:0000049 | tRNA binding | 0.155844 | 0.03626 |
| GO:0060429 | epithelium development | 0.135135 | 0.03609 |
| GO:0048589 | developmental growth | 0.113426 | 0.03604 |
| GO:0040007 | growth | 0.113426 | 0.03604 |
| GO:0002695 | negative regulation of leukocyte activation | 0.130952 | 0.03594 |
| GO:0009064 | glutamine family amino acid metabolic process | 0.152174 | 0.03530 |
| GO:0032200 | telomere organization | 0.152174 | 0.03530 |
| GO:0006612 | protein targeting to membrane | 0.152174 | 0.03530 |
| GO:0000723 | telomere maintenance | 0.152174 | 0.03530 |
| GO:2001252 | positive regulation of chromosome organization | 0.152174 | 0.03530 |
| GO:0005342 | organic acid transmembrane transporter activity | 0.136054 | 0.03528 |
| GO:0008643 | carbohydrate transport | 0.163934 | 0.03504 |
| GO:0045111 | intermediate filament cytoskeleton | 0.163934 | 0.03504 |
| GO:0016773 | phosphotransferase activity, alcohol group as acceptor | 0.103413 | 0.03481 |
| GO:0070098 | chemokine-mediated signaling pathway | 0.18 | 0.03473 |
| GO:0032374 | regulation of cholesterol transport | 0.18 | 0.03473 |
| GO:0032371 | regulation of sterol transport | 0.18 | 0.03473 |
| GO:0016409 | palmitoyltransferase activity | 0.18 | 0.03473 |
| GO:0048514 | blood vessel morphogenesis | 0.157895 | 0.03460 |
| GO:0031312 | extrinsic component of organelle membrane | 0.157895 | 0.03460 |
| GO:0004714 | transmembrane receptor protein tyrosine kinase activity | 0.157895 | 0.03460 |
| GO:0006101 | citrate metabolic process | 0.333333 | 0.03409 |
| GO:0098703 | calcium ion import across plasma membrane | 0.333333 | 0.03409 |
| GO:0021781 | glial cell fate commitment | 0.333333 | 0.03409 |
| GO:1904729 | regulation of intestinal lipid absorption | 0.333333 | 0.03409 |
| GO:0014883 | transition between fast and slow fiber | 0.333333 | 0.03409 |
| GO:0032471 | negative regulation of endoplasmic reticulum calcium ion concentration | 0.333333 | 0.03409 |
| GO:0007023 | post-chaperonin tubulin folding pathway | 0.333333 | 0.03409 |
| GO:0021604 | cranial nerve structural organization | 0.333333 | 0.03409 |
| GO:1902033 | regulation of hematopoietic stem cell proliferation | 0.333333 | 0.03409 |
| GO:0006707 | cholesterol catabolic process | 0.333333 | 0.03409 |
| GO:0097212 | lysosomal membrane organization | 0.333333 | 0.03409 |
| GO:0015872 | dopamine transport | 0.333333 | 0.03409 |
| GO:0016127 | sterol catabolic process | 0.333333 | 0.03409 |
| GO:0033622 | integrin activation | 0.333333 | 0.03409 |
| GO:1902510 | regulation of apoptotic DNA fragmentation | 0.333333 | 0.03409 |
| GO:1990416 | cellular response to brain-derived neurotrophic factor stimulus | 0.333333 | 0.03409 |
| GO:1905048 | regulation of metallopeptidase activity | 0.333333 | 0.03409 |
| GO:0002369 | T cell cytokine production | 0.333333 | 0.03409 |
| GO:0007270 | neuron-neuron synaptic transmission | 0.333333 | 0.03409 |
| GO:0009313 | oligosaccharide catabolic process | 0.333333 | 0.03409 |
| GO:0051580 | regulation of neurotransmitter uptake | 0.333333 | 0.03409 |
| GO:0002507 | tolerance induction | 0.333333 | 0.03409 |
| GO:1904251 | regulation of bile acid metabolic process | 0.333333 | 0.03409 |
| GO:0071225 | cellular response to muramyl dipeptide | 0.333333 | 0.03409 |
| GO:0032351 | negative regulation of hormone metabolic process | 0.333333 | 0.03409 |
| GO:0034370 | triglyceride-rich lipoprotein particle remodeling | 0.333333 | 0.03409 |
| GO:0043983 | histone H4-K12 acetylation | 0.333333 | 0.03409 |
| GO:0009650 | UV protection | 0.333333 | 0.03409 |
| GO:0036513 | Derlin-1 retrotranslocation complex | 0.333333 | 0.03409 |
| GO:0017136 | NAD-dependent histone deacetylase activity | 0.333333 | 0.03409 |
| GO:0034595 | phosphatidylinositol phosphate 5-phosphatase activity | 0.333333 | 0.03409 |
| GO:0016634 | oxidoreductase activity, acting on the CH-CH group of donors, oxygen as acceptor | 0.333333 | 0.03409 |
| GO:0035256 | G protein-coupled glutamate receptor binding | 0.333333 | 0.03409 |
| GO:0016453 | C-acetyltransferase activity | 0.333333 | 0.03409 |
| GO:0098919 | structural constituent of postsynaptic density | 0.333333 | 0.03409 |
| GO:0008443 | phosphofructokinase activity | 0.333333 | 0.03409 |
| GO:0031698 | beta-2 adrenergic receptor binding | 0.333333 | 0.03409 |
| GO:0046912 | acyltransferase activity, acyl groups converted into alkyl on transfer | 0.333333 | 0.03409 |
| GO:0010906 | regulation of glucose metabolic process | 0.137931 | 0.03400 |
| GO:0072521 | purine-containing compound metabolic process | 0.104213 | 0.03375 |
| GO:0072593 | reactive oxygen species metabolic process | 0.144231 | 0.03371 |
| GO:0048469 | cell maturation | 0.133333 | 0.03346 |
| GO:0097150 | neuronal stem cell population maintenance | 0.227273 | 0.03316 |
| GO:0046460 | neutral lipid biosynthetic process | 0.227273 | 0.03316 |
| GO:0044597 | daunorubicin metabolic process | 0.227273 | 0.03316 |
| GO:0046697 | decidualization | 0.227273 | 0.03316 |
| GO:0014003 | oligodendrocyte development | 0.227273 | 0.03316 |
| GO:0060479 | lung cell differentiation | 0.227273 | 0.03316 |
| GO:0048854 | brain morphogenesis | 0.227273 | 0.03316 |
| GO:0046463 | acylglycerol biosynthetic process | 0.227273 | 0.03316 |
| GO:0030638 | polyketide metabolic process | 0.227273 | 0.03316 |
| GO:0071731 | response to nitric oxide | 0.227273 | 0.03316 |
| GO:0003206 | cardiac chamber morphogenesis | 0.227273 | 0.03316 |
| GO:0014812 | muscle cell migration | 0.227273 | 0.03316 |
| GO:0085029 | extracellular matrix assembly | 0.227273 | 0.03316 |
| GO:0060487 | lung epithelial cell differentiation | 0.227273 | 0.03316 |
| GO:0032957 | inositol trisphosphate metabolic process | 0.227273 | 0.03316 |
| GO:0002063 | chondrocyte development | 0.227273 | 0.03316 |
| GO:1901028 | regulation of mitochondrial outer membrane permeabilization involved in apoptotic signaling pathway | 0.227273 | 0.03316 |
| GO:0055083 | monovalent inorganic anion homeostasis | 0.227273 | 0.03316 |
| GO:0042311 | vasodilation | 0.227273 | 0.03316 |
| GO:0044598 | doxorubicin metabolic process | 0.227273 | 0.03316 |
| GO:0015377 | cation:chloride symporter activity | 0.227273 | 0.03316 |
| GO:0051248 | negative regulation of protein metabolic process | 0.101297 | 0.03286 |
| GO:0009060 | aerobic respiration | 0.12973 | 0.03282 |
| GO:0060135 | maternal process involved in female pregnancy | 0.189189 | 0.03269 |
| GO:0021675 | nerve development | 0.189189 | 0.03269 |
| GO:0008180 | COP9 signalosome | 0.189189 | 0.03269 |
| GO:0005154 | epidermal growth factor receptor binding | 0.189189 | 0.03269 |
| GO:0021987 | cerebral cortex development | 0.166667 | 0.03249 |
| GO:0050678 | regulation of epithelial cell proliferation | 0.119497 | 0.03246 |
| GO:1904406 | negative regulation of nitric oxide metabolic process | 0.266667 | 0.03228 |
| GO:0045019 | negative regulation of nitric oxide biosynthetic process | 0.266667 | 0.03228 |
| GO:0002921 | negative regulation of humoral immune response | 0.266667 | 0.03228 |
| GO:0006349 | regulation of gene expression by genomic imprinting | 0.266667 | 0.03228 |
| GO:0006517 | protein deglycosylation | 0.266667 | 0.03228 |
| GO:0006531 | aspartate metabolic process | 0.266667 | 0.03228 |
| GO:0030007 | cellular potassium ion homeostasis | 0.266667 | 0.03228 |
| GO:0060997 | dendritic spine morphogenesis | 0.266667 | 0.03228 |
| GO:0071285 | cellular response to lithium ion | 0.266667 | 0.03228 |
| GO:0060216 | definitive hemopoiesis | 0.266667 | 0.03228 |
| GO:0060330 | regulation of response to interferon-gamma | 0.266667 | 0.03228 |
| GO:0060334 | regulation of interferon-gamma-mediated signaling pathway | 0.266667 | 0.03228 |
| GO:2000678 | negative regulation of transcription regulatory region DNA binding | 0.266667 | 0.03228 |
| GO:0009151 | purine deoxyribonucleotide metabolic process | 0.266667 | 0.03228 |
| GO:0009312 | oligosaccharide biosynthetic process | 0.266667 | 0.03228 |
| GO:1903019 | negative regulation of glycoprotein metabolic process | 0.266667 | 0.03228 |
| GO:0051284 | positive regulation of sequestering of calcium ion | 0.266667 | 0.03228 |
| GO:0000291 | nuclear-transcribed mRNA catabolic process, exonucleolytic | 0.266667 | 0.03228 |
| GO:0030497 | fatty acid elongation | 0.266667 | 0.03228 |
| GO:0015693 | magnesium ion transport | 0.266667 | 0.03228 |
| GO:0007638 | mechanosensory behavior | 0.266667 | 0.03228 |
| GO:0048019 | receptor antagonist activity | 0.266667 | 0.03228 |
| GO:0042610 | CD8 receptor binding | 0.266667 | 0.03228 |
| GO:0008179 | adenylate cyclase binding | 0.266667 | 0.03228 |
| GO:0004697 | protein kinase C activity | 0.266667 | 0.03228 |
| GO:0016840 | carbon-nitrogen lyase activity | 0.266667 | 0.03228 |
| GO:0032432 | actin filament bundle | 0.151163 | 0.03220 |
| GO:0006081 | cellular aldehyde metabolic process | 0.162162 | 0.03193 |
| GO:0010506 | regulation of autophagy | 0.125541 | 0.03147 |
| GO:0070328 | triglyceride homeostasis | 0.206897 | 0.03132 |
| GO:0055090 | acylglycerol homeostasis | 0.206897 | 0.03132 |
| GO:0007635 | chemosensory behavior | 0.206897 | 0.03132 |
| GO:0033238 | regulation of cellular amine metabolic process | 0.206897 | 0.03132 |
| GO:0014904 | myotube cell development | 0.206897 | 0.03132 |
| GO:0010837 | regulation of keratinocyte proliferation | 0.206897 | 0.03132 |
| GO:0032965 | regulation of collagen biosynthetic process | 0.206897 | 0.03132 |
| GO:0016896 | exoribonuclease activity, producing 5'-phosphomonoesters | 0.206897 | 0.03132 |
| GO:0045069 | regulation of viral genome replication | 0.141667 | 0.03109 |
| GO:1903201 | regulation of oxidative stress-induced cell death | 0.164384 | 0.03091 |
| GO:0070664 | negative regulation of leukocyte proliferation | 0.164384 | 0.03091 |
| GO:0099587 | inorganic ion import across plasma membrane | 0.164384 | 0.03091 |
| GO:0098659 | inorganic cation import across plasma membrane | 0.164384 | 0.03091 |
| GO:0043535 | regulation of blood vessel endothelial cell migration | 0.164384 | 0.03091 |
| GO:0005201 | extracellular matrix structural constituent | 0.164384 | 0.03091 |
| GO:0043207 | response to external biotic stimulus | 0.097466 | 0.03085 |
| GO:0009749 | response to glucose | 0.137681 | 0.03063 |
| GO:0001952 | regulation of cell-matrix adhesion | 0.147059 | 0.03057 |
| GO:0001938 | positive regulation of endothelial cell proliferation | 0.152941 | 0.03029 |
| GO:0030667 | secretory granule membrane | 0.152941 | 0.03029 |
| GO:0016836 | hydro-lyase activity | 0.152941 | 0.03029 |
| GO:0010043 | response to zinc ion | 0.169492 | 0.03018 |
| GO:0001570 | vasculogenesis | 0.169492 | 0.03018 |
| GO:0043647 | inositol phosphate metabolic process | 0.169492 | 0.03018 |
| GO:0030173 | integral component of Golgi membrane | 0.169492 | 0.03018 |
| GO:0006605 | protein targeting | 0.123506 | 0.02991 |
| GO:0098948 | intrinsic component of postsynaptic specialization membrane | 0.148515 | 0.02922 |
| GO:0051117 | ATPase binding | 0.148515 | 0.02922 |
| GO:0030029 | actin filament-based process | 0.113953 | 0.02909 |
| GO:0006730 | one-carbon metabolic process | 0.181818 | 0.02889 |
| GO:0044390 | ubiquitin-like protein conjugating enzyme binding | 0.181818 | 0.02889 |
| GO:0010634 | positive regulation of epithelial cell migration | 0.144068 | 0.02887 |
| GO:0001763 | morphogenesis of a branching structure | 0.139706 | 0.02868 |
| GO:0046626 | regulation of insulin receptor signaling pathway | 0.161765 | 0.02863 |
| GO:0044843 | cell cycle G1/S phase transition | 0.161765 | 0.02863 |
| GO:0007155 | cell adhesion | 0.10432 | 0.02858 |
| GO:0045940 | positive regulation of steroid metabolic process | 0.194444 | 0.02851 |
| GO:0019692 | deoxyribose phosphate metabolic process | 0.194444 | 0.02851 |
| GO:0042773 | ATP synthesis coupled electron transport | 0.194444 | 0.02851 |
| GO:0051568 | histone H3-K4 methylation | 0.194444 | 0.02851 |
| GO:0006858 | extracellular transport | 0.194444 | 0.02851 |
| GO:0014014 | negative regulation of gliogenesis | 0.194444 | 0.02851 |
| GO:2000273 | positive regulation of signaling receptor activity | 0.194444 | 0.02851 |
| GO:0033017 | sarcoplasmic reticulum membrane | 0.194444 | 0.02851 |
| GO:0055074 | calcium ion homeostasis | 0.130653 | 0.02829 |
| GO:0031256 | leading edge membrane | 0.132184 | 0.02815 |
| GO:0030516 | regulation of axon extension | 0.15 | 0.02801 |
| GO:0046942 | carboxylic acid transport | 0.131313 | 0.02791 |
| GO:0001667 | ameboidal-type cell migration | 0.136364 | 0.02783 |
| GO:1902074 | response to salt | 0.238095 | 0.02747 |
| GO:1904031 | positive regulation of cyclin-dependent protein kinase activity | 0.238095 | 0.02747 |
| GO:0071868 | cellular response to monoamine stimulus | 0.238095 | 0.02747 |
| GO:0072350 | tricarboxylic acid metabolic process | 0.238095 | 0.02747 |
| GO:0045723 | positive regulation of fatty acid biosynthetic process | 0.238095 | 0.02747 |
| GO:0009086 | methionine biosynthetic process | 0.238095 | 0.02747 |
| GO:0007076 | mitotic chromosome condensation | 0.238095 | 0.02747 |
| GO:0090312 | positive regulation of protein deacetylation | 0.238095 | 0.02747 |
| GO:0045980 | negative regulation of nucleotide metabolic process | 0.238095 | 0.02747 |
| GO:0010874 | regulation of cholesterol efflux | 0.238095 | 0.02747 |
| GO:0032461 | positive regulation of protein oligomerization | 0.238095 | 0.02747 |
| GO:0055064 | chloride ion homeostasis | 0.238095 | 0.02747 |
| GO:0010499 | proteasomal ubiquitin-independent protein catabolic process | 0.238095 | 0.02747 |
| GO:0061629 | RNA polymerase II-specific DNA-binding transcription factor binding | 0.119266 | 0.02741 |
| GO:0010821 | regulation of mitochondrion organization | 0.141791 | 0.02715 |
| GO:0032411 | positive regulation of transporter activity | 0.146552 | 0.02714 |
| GO:0030833 | regulation of actin filament polymerization | 0.137255 | 0.02711 |
| GO:0009267 | cellular response to starvation | 0.137255 | 0.02711 |
| GO:1901879 | regulation of protein depolymerization | 0.151515 | 0.02695 |
| GO:0002702 | positive regulation of production of molecular mediator of immune response | 0.151515 | 0.02695 |
| GO:0042552 | myelination | 0.151515 | 0.02695 |
| GO:0004540 | ribonuclease activity | 0.125 | 0.02673 |
| GO:0008210 | estrogen metabolic process | 0.214286 | 0.02669 |
| GO:0031295 | T cell costimulation | 0.214286 | 0.02669 |
| GO:0051569 | regulation of histone H3-K4 methylation | 0.214286 | 0.02669 |
| GO:0051953 | negative regulation of amine transport | 0.214286 | 0.02669 |
| GO:0008045 | motor neuron axon guidance | 0.214286 | 0.02669 |
| GO:0035640 | exploration behavior | 0.214286 | 0.02669 |
| GO:0002026 | regulation of the force of heart contraction | 0.214286 | 0.02669 |
| GO:1904646 | cellular response to amyloid-beta | 0.214286 | 0.02669 |
| GO:0030552 | cAMP binding | 0.214286 | 0.02669 |
| GO:0000175 | 3'-5'-exoribonuclease activity | 0.214286 | 0.02669 |
| GO:0015081 | sodium ion transmembrane transporter activity | 0.138158 | 0.02647 |
| GO:0051588 | regulation of neurotransmitter transport | 0.147826 | 0.02646 |
| GO:0045638 | negative regulation of myeloid cell differentiation | 0.147826 | 0.02646 |
| GO:0008238 | exopeptidase activity | 0.147826 | 0.02646 |
| GO:0014743 | regulation of muscle hypertrophy | 0.164179 | 0.02638 |
| GO:0000082 | G1/S transition of mitotic cell cycle | 0.164179 | 0.02638 |
| GO:0034968 | histone lysine methylation | 0.164179 | 0.02638 |
| GO:0051966 | regulation of synaptic transmission, glutamatergic | 0.175439 | 0.02632 |
| GO:0048278 | vesicle docking | 0.175439 | 0.02632 |
| GO:0060326 | cell chemotaxis | 0.129032 | 0.02623 |
| GO:0060548 | negative regulation of cell death | 0.100488 | 0.02611 |
| GO:0009607 | response to biotic stimulus | 0.097596 | 0.02611 |
| GO:0140098 | catalytic activity, acting on RNA | 0.110368 | 0.02578 |
| GO:0098552 | side of membrane | 0.108664 | 0.02575 |
| GO:0002376 | immune system process | 0.096788 | 0.02557 |
| GO:0071383 | cellular response to steroid hormone stimulus | 0.158537 | 0.02555 |
| GO:0005884 | actin filament | 0.158537 | 0.02555 |
| GO:0006123 | mitochondrial electron transport, cytochrome c to oxygen | 0.186047 | 0.02546 |
| GO:0070207 | protein homotrimerization | 0.186047 | 0.02546 |
| GO:0002639 | positive regulation of immunoglobulin production | 0.186047 | 0.02546 |
| GO:0010665 | regulation of cardiac muscle cell apoptotic process | 0.186047 | 0.02546 |
| GO:0140358 | P-type transmembrane transporter activity | 0.186047 | 0.02546 |
| GO:0015662 | P-type ion transporter activity | 0.186047 | 0.02546 |
| GO:0006620 | post-translational protein targeting to endoplasmic reticulum membrane | 0.285714 | 0.02532 |
| GO:0000289 | nuclear-transcribed mRNA poly(A) tail shortening | 0.285714 | 0.02532 |
| GO:2000209 | regulation of anoikis | 0.285714 | 0.02532 |
| GO:1901077 | regulation of relaxation of muscle | 0.285714 | 0.02532 |
| GO:0032793 | positive regulation of CREB transcription factor activity | 0.285714 | 0.02532 |
| GO:1904424 | regulation of GTP binding | 0.285714 | 0.02532 |
| GO:0060213 | positive regulation of nuclear-transcribed mRNA poly(A) tail shortening | 0.285714 | 0.02532 |
| GO:0046341 | CDP-diacylglycerol metabolic process | 0.285714 | 0.02532 |
| GO:0046415 | urate metabolic process | 0.285714 | 0.02532 |
| GO:0071362 | cellular response to ether | 0.285714 | 0.02532 |
| GO:0035641 | locomotory exploration behavior | 0.285714 | 0.02532 |
| GO:0051280 | negative regulation of release of sequestered calcium ion into cytosol | 0.285714 | 0.02532 |
| GO:0060055 | angiogenesis involved in wound healing | 0.285714 | 0.02532 |
| GO:0045820 | negative regulation of glycolytic process | 0.285714 | 0.02532 |
| GO:1905207 | regulation of cardiocyte differentiation | 0.285714 | 0.02532 |
| GO:0042627 | chylomicron | 0.285714 | 0.02532 |
| GO:0002178 | palmitoyltransferase complex | 0.285714 | 0.02532 |
| GO:0047023 | androsterone dehydrogenase activity | 0.285714 | 0.02532 |
| GO:0004312 | fatty acid synthase activity | 0.285714 | 0.02532 |
| GO:0030228 | lipoprotein particle receptor activity | 0.285714 | 0.02532 |
| GO:0031434 | mitogen-activated protein kinase kinase binding | 0.285714 | 0.02532 |
| GO:0004143 | diacylglycerol kinase activity | 0.285714 | 0.02532 |
| GO:0004771 | sterol esterase activity | 0.285714 | 0.02532 |
| GO:0017081 | chloride channel regulator activity | 0.285714 | 0.02532 |
| GO:0006919 | activation of cysteine-type endopeptidase activity involved in apoptotic process | 0.154639 | 0.02525 |
| GO:1903076 | regulation of protein localization to plasma membrane | 0.154639 | 0.02525 |
| GO:0070069 | cytochrome complex | 0.154639 | 0.02525 |
| GO:0099056 | integral component of presynaptic membrane | 0.154639 | 0.02525 |
| GO:0032922 | circadian regulation of gene expression | 0.178571 | 0.02476 |
| GO:0002889 | regulation of immunoglobulin mediated immune response | 0.178571 | 0.02476 |
| GO:0032732 | positive regulation of interleukin-1 production | 0.178571 | 0.02476 |
| GO:0050885 | neuromuscular process controlling balance | 0.178571 | 0.02476 |
| GO:0002712 | regulation of B cell mediated immunity | 0.178571 | 0.02476 |
| GO:0033144 | negative regulation of intracellular steroid hormone receptor signaling pathway | 0.2 | 0.02471 |
| GO:0042310 | vasoconstriction | 0.2 | 0.02471 |
| GO:0035255 | ionotropic glutamate receptor binding | 0.2 | 0.02471 |
| GO:0006753 | nucleoside phosphate metabolic process | 0.104104 | 0.02457 |
| GO:0000287 | magnesium ion binding | 0.122378 | 0.02451 |
| GO:0003727 | single-stranded RNA binding | 0.136905 | 0.02445 |
| GO:0042246 | tissue regeneration | 0.166667 | 0.02432 |
| GO:0006105 | succinate metabolic process | 0.375 | 0.02422 |
| GO:2001198 | regulation of dendritic cell differentiation | 0.375 | 0.02422 |
| GO:0006309 | apoptotic DNA fragmentation | 0.375 | 0.02422 |
| GO:0045919 | positive regulation of cytolysis | 0.375 | 0.02422 |
| GO:0071380 | cellular response to prostaglandin E stimulus | 0.375 | 0.02422 |
| GO:0030300 | regulation of intestinal cholesterol absorption | 0.375 | 0.02422 |
| GO:0098596 | imitative learning | 0.375 | 0.02422 |
| GO:0098597 | observational learning | 0.375 | 0.02422 |
| GO:2000425 | regulation of apoptotic cell clearance | 0.375 | 0.02422 |
| GO:1902884 | positive regulation of response to oxidative stress | 0.375 | 0.02422 |
| GO:1901856 | negative regulation of cellular respiration | 0.375 | 0.02422 |
| GO:0019367 | fatty acid elongation, saturated fatty acid | 0.375 | 0.02422 |
| GO:1904717 | regulation of AMPA glutamate receptor clustering | 0.375 | 0.02422 |
| GO:0010873 | positive regulation of cholesterol esterification | 0.375 | 0.02422 |
| GO:0070857 | regulation of bile acid biosynthetic process | 0.375 | 0.02422 |
| GO:0032782 | bile acid secretion | 0.375 | 0.02422 |
| GO:0051342 | regulation of cyclic-nucleotide phosphodiesterase activity | 0.375 | 0.02422 |
| GO:0038089 | positive regulation of cell migration by vascular endothelial growth factor signaling pathway | 0.375 | 0.02422 |
| GO:0032819 | positive regulation of natural killer cell proliferation | 0.375 | 0.02422 |
| GO:2000969 | positive regulation of AMPA receptor activity | 0.375 | 0.02422 |
| GO:0006547 | histidine metabolic process | 0.375 | 0.02422 |
| GO:0034372 | very-low-density lipoprotein particle remodeling | 0.375 | 0.02422 |
| GO:0071218 | cellular response to misfolded protein | 0.375 | 0.02422 |
| GO:0003231 | cardiac ventricle development | 0.375 | 0.02422 |
| GO:0042297 | vocal learning | 0.375 | 0.02422 |
| GO:0106104 | regulation of glutamate receptor clustering | 0.375 | 0.02422 |
| GO:0072718 | response to cisplatin | 0.375 | 0.02422 |
| GO:0035814 | negative regulation of renal sodium excretion | 0.375 | 0.02422 |
| GO:0021957 | corticospinal tract morphogenesis | 0.375 | 0.02422 |
| GO:0005861 | troponin complex | 0.375 | 0.02422 |
| GO:0005784 | Sec61 translocon complex | 0.375 | 0.02422 |
| GO:0140268 | endoplasmic reticulum-plasma membrane contact site | 0.375 | 0.02422 |
| GO:0035253 | ciliary rootlet | 0.375 | 0.02422 |
| GO:0004445 | inositol-polyphosphate 5-phosphatase activity | 0.375 | 0.02422 |
| GO:0004300 | enoyl-CoA hydratase activity | 0.375 | 0.02422 |
| GO:0004767 | sphingomyelin phosphodiesterase activity | 0.375 | 0.02422 |
| GO:0004568 | chitinase activity | 0.375 | 0.02422 |
| GO:0009922 | fatty acid elongase activity | 0.375 | 0.02422 |
| GO:0102756 | very-long-chain 3-ketoacyl-CoA synthase activity | 0.375 | 0.02422 |
| GO:0004439 | phosphatidylinositol-4,5-bisphosphate 5-phosphatase activity | 0.375 | 0.02422 |
| GO:0015925 | galactosidase activity | 0.375 | 0.02422 |
| GO:0016071 | mRNA metabolic process | 0.107713 | 0.02406 |
| GO:0043414 | macromolecule methylation | 0.124514 | 0.02403 |
| GO:0016791 | phosphatase activity | 0.119883 | 0.02389 |
| GO:0071356 | cellular response to tumor necrosis factor | 0.142857 | 0.02380 |
| GO:0099072 | regulation of postsynaptic membrane neurotransmitter receptor levels | 0.181818 | 0.02343 |
| GO:0010823 | negative regulation of mitochondrion organization | 0.181818 | 0.02343 |
| GO:0031903 | microbody membrane | 0.181818 | 0.02343 |
| GO:0005778 | peroxisomal membrane | 0.181818 | 0.02343 |
| GO:0031589 | cell-substrate adhesion | 0.130435 | 0.02302 |
| GO:0031348 | negative regulation of defense response | 0.130435 | 0.02302 |
| GO:0043412 | macromolecule modification | 0.094683 | 0.02260 |
| GO:0000186 | obsolete activation of MAPKK activity | 0.222222 | 0.02253 |
| GO:0060143 | positive regulation of syncytium formation by plasma membrane fusion | 0.222222 | 0.02253 |
| GO:0097066 | response to thyroid hormone | 0.222222 | 0.02253 |
| GO:0031338 | regulation of vesicle fusion | 0.222222 | 0.02253 |
| GO:0016180 | snRNA processing | 0.222222 | 0.02253 |
| GO:0015800 | acidic amino acid transport | 0.222222 | 0.02253 |
| GO:0046337 | phosphatidylethanolamine metabolic process | 0.222222 | 0.02253 |
| GO:0010664 | negative regulation of striated muscle cell apoptotic process | 0.222222 | 0.02253 |
| GO:0070325 | lipoprotein particle receptor binding | 0.222222 | 0.02253 |
| GO:0005537 | mannose binding | 0.222222 | 0.02253 |
| GO:0002218 | activation of innate immune response | 0.169231 | 0.02246 |
| GO:0090128 | regulation of synapse maturation | 0.25 | 0.02245 |
| GO:0045939 | negative regulation of steroid metabolic process | 0.25 | 0.02245 |
| GO:1901223 | negative regulation of NIK/NF-kappaB signaling | 0.25 | 0.02245 |
| GO:1902473 | regulation of protein localization to synapse | 0.25 | 0.02245 |
| GO:0002098 | tRNA wobble uridine modification | 0.25 | 0.02245 |
| GO:1900543 | negative regulation of purine nucleotide metabolic process | 0.25 | 0.02245 |
| GO:0071870 | cellular response to catecholamine stimulus | 0.25 | 0.02245 |
| GO:0010894 | negative regulation of steroid biosynthetic process | 0.25 | 0.02245 |
| GO:0000737 | DNA catabolic process, endonucleolytic | 0.25 | 0.02245 |
| GO:0031331 | positive regulation of cellular catabolic process | 0.12012 | 0.02237 |
| GO:0051353 | positive regulation of oxidoreductase activity | 0.190476 | 0.02232 |
| GO:0031646 | positive regulation of nervous system process | 0.190476 | 0.02232 |
| GO:0071322 | cellular response to carbohydrate stimulus | 0.164557 | 0.02230 |
| GO:0046330 | positive regulation of JNK cascade | 0.164557 | 0.02230 |
| GO:0071704 | organic substance metabolic process | 0.088711 | 0.02211 |
| GO:0009057 | macromolecule catabolic process | 0.104128 | 0.02189 |
| GO:1903522 | regulation of blood circulation | 0.132353 | 0.02177 |
| GO:0006112 | energy reserve metabolic process | 0.155556 | 0.02154 |
| GO:0005581 | collagen trimer | 0.155556 | 0.02154 |
| GO:0060348 | bone development | 0.166667 | 0.02153 |
| GO:1901880 | negative regulation of protein depolymerization | 0.166667 | 0.02153 |
| GO:0045089 | positive regulation of innate immune response | 0.1375 | 0.02150 |
| GO:0010712 | regulation of collagen metabolic process | 0.205882 | 0.02127 |
| GO:0016137 | glycoside metabolic process | 0.205882 | 0.02127 |
| GO:1904645 | response to amyloid-beta | 0.205882 | 0.02127 |
| GO:0035455 | response to interferon-alpha | 0.205882 | 0.02127 |
| GO:0061462 | protein localization to lysosome | 0.205882 | 0.02127 |
| GO:0007257 | obsolete activation of JUN kinase activity | 0.205882 | 0.02127 |
| GO:0031929 | TOR signaling | 0.205882 | 0.02127 |
| GO:0044232 | organelle membrane contact site | 0.205882 | 0.02127 |
| GO:0140323 | solute:anion antiporter activity | 0.205882 | 0.02127 |
| GO:0071889 | 14-3-3 protein binding | 0.205882 | 0.02127 |
| GO:0006937 | regulation of muscle contraction | 0.141844 | 0.02124 |
| GO:0008213 | protein alkylation | 0.141844 | 0.02124 |
| GO:0009746 | response to hexose | 0.141844 | 0.02124 |
| GO:0006479 | protein methylation | 0.141844 | 0.02124 |
| GO:0070588 | calcium ion transmembrane transport | 0.146341 | 0.02108 |
| GO:0031902 | late endosome membrane | 0.146341 | 0.02108 |
| GO:0010611 | regulation of cardiac muscle hypertrophy | 0.171875 | 0.02078 |
| GO:0070206 | protein trimerization | 0.171875 | 0.02078 |
| GO:1900076 | regulation of cellular response to insulin stimulus | 0.171875 | 0.02078 |
| GO:0001786 | phosphatidylserine binding | 0.171875 | 0.02078 |
| GO:1902495 | transmembrane transporter complex | 0.116071 | 0.02048 |
| GO:1903037 | regulation of leukocyte cell-cell adhesion | 0.125461 | 0.02046 |
| GO:0007033 | vacuole organization | 0.147541 | 0.02037 |
| GO:1904375 | regulation of protein localization to cell periphery | 0.147541 | 0.02037 |
| GO:0001894 | tissue homeostasis | 0.139241 | 0.02029 |
| GO:0098936 | intrinsic component of postsynaptic membrane | 0.139241 | 0.02029 |
| GO:0090090 | negative regulation of canonical Wnt signaling pathway | 0.152381 | 0.02012 |
| GO:0071579 | regulation of zinc ion transport | 0.666667 | 0.02009 |
| GO:2000121 | regulation of removal of superoxide radicals | 0.666667 | 0.02009 |
| GO:0006933 | negative regulation of cell adhesion involved in substrate-bound cell migration | 0.666667 | 0.02009 |
| GO:0019287 | isopentenyl diphosphate biosynthetic process, mevalonate pathway | 0.666667 | 0.02009 |
| GO:0006683 | galactosylceramide catabolic process | 0.666667 | 0.02009 |
| GO:0090461 | glutamate homeostasis | 0.666667 | 0.02009 |
| GO:0098942 | retrograde trans-synaptic signaling by trans-synaptic protein complex | 0.666667 | 0.02009 |
| GO:0034444 | regulation of plasma lipoprotein oxidation | 0.666667 | 0.02009 |
| GO:2000547 | regulation of dendritic cell dendrite assembly | 0.666667 | 0.02009 |
| GO:1904261 | positive regulation of basement membrane assembly involved in embryonic body morphogenesis | 0.666667 | 0.02009 |
| GO:0010046 | response to mycotoxin | 0.666667 | 0.02009 |
| GO:0071865 | regulation of apoptotic process in bone marrow cell | 0.666667 | 0.02009 |
| GO:0071866 | negative regulation of apoptotic process in bone marrow cell | 0.666667 | 0.02009 |
| GO:0071242 | cellular response to ammonium ion | 0.666667 | 0.02009 |
| GO:0010025 | wax biosynthetic process | 0.666667 | 0.02009 |
| GO:0051124 | synaptic assembly at neuromuscular junction | 0.666667 | 0.02009 |
| GO:0034445 | negative regulation of plasma lipoprotein oxidation | 0.666667 | 0.02009 |
| GO:1904426 | positive regulation of GTP binding | 0.666667 | 0.02009 |
| GO:0018125 | peptidyl-cysteine methylation | 0.666667 | 0.02009 |
| GO:0001969 | regulation of activation of membrane attack complex | 0.666667 | 0.02009 |
| GO:0072156 | distal tubule morphogenesis | 0.666667 | 0.02009 |
| GO:0010182 | sugar mediated signaling pathway | 0.666667 | 0.02009 |
| GO:0032714 | negative regulation of interleukin-5 production | 0.666667 | 0.02009 |
| GO:0016128 | phytosteroid metabolic process | 0.666667 | 0.02009 |
| GO:0016129 | phytosteroid biosynthetic process | 0.666667 | 0.02009 |
| GO:1903847 | regulation of aorta morphogenesis | 0.666667 | 0.02009 |
| GO:1903849 | positive regulation of aorta morphogenesis | 0.666667 | 0.02009 |
| GO:0070814 | hydrogen sulfide biosynthetic process | 0.666667 | 0.02009 |
| GO:1904612 | response to 2,3,7,8-tetrachlorodibenzodioxine | 0.666667 | 0.02009 |
| GO:1903895 | negative regulation of IRE1-mediated unfolded protein response | 0.666667 | 0.02009 |
| GO:0071400 | cellular response to oleic acid | 0.666667 | 0.02009 |
| GO:0002765 | immune response-inhibiting signal transduction | 0.666667 | 0.02009 |
| GO:0089700 | protein kinase D signaling | 0.666667 | 0.02009 |
| GO:0099152 | regulation of neurotransmitter receptor transport, endosome to postsynaptic membrane | 0.666667 | 0.02009 |
| GO:1901401 | regulation of tetrapyrrole metabolic process | 0.666667 | 0.02009 |
| GO:1900138 | negative regulation of phospholipase A2 activity | 0.666667 | 0.02009 |
| GO:1900150 | regulation of defense response to fungus | 0.666667 | 0.02009 |
| GO:0060486 | club cell differentiation | 0.666667 | 0.02009 |
| GO:2001029 | regulation of cellular glucuronidation | 0.666667 | 0.02009 |
| GO:0055017 | cardiac muscle tissue growth | 0.666667 | 0.02009 |
| GO:2001025 | positive regulation of response to drug | 0.666667 | 0.02009 |
| GO:0046452 | dihydrofolate metabolic process | 0.666667 | 0.02009 |
| GO:0071603 | endothelial cell-cell adhesion | 0.666667 | 0.02009 |
| GO:0090045 | positive regulation of deacetylase activity | 0.666667 | 0.02009 |
| GO:0019628 | urate catabolic process | 0.666667 | 0.02009 |
| GO:0031947 | negative regulation of glucocorticoid biosynthetic process | 0.666667 | 0.02009 |
| GO:0031944 | negative regulation of glucocorticoid metabolic process | 0.666667 | 0.02009 |
| GO:1903378 | positive regulation of oxidative stress-induced neuron intrinsic apoptotic signaling pathway | 0.666667 | 0.02009 |
| GO:1904259 | regulation of basement membrane assembly involved in embryonic body morphogenesis | 0.666667 | 0.02009 |
| GO:0097029 | mature conventional dendritic cell differentiation | 0.666667 | 0.02009 |
| GO:0009757 | hexose mediated signaling | 0.666667 | 0.02009 |
| GO:0044028 | DNA hypomethylation | 0.666667 | 0.02009 |
| GO:0009756 | carbohydrate mediated signaling | 0.666667 | 0.02009 |
| GO:0075206 | obsolete positive regulation by host of symbiont cAMP-mediated signal transduction | 0.666667 | 0.02009 |
| GO:0044029 | hypomethylation of CpG island | 0.666667 | 0.02009 |
| GO:1904431 | positive regulation of t-circle formation | 0.666667 | 0.02009 |
| GO:0140014 | mitotic nuclear division | 0.666667 | 0.02009 |
| GO:0071585 | detoxification of cadmium ion | 0.666667 | 0.02009 |
| GO:1901656 | glycoside transport | 0.666667 | 0.02009 |
| GO:0043323 | positive regulation of natural killer cell degranulation | 0.666667 | 0.02009 |
| GO:0043321 | regulation of natural killer cell degranulation | 0.666667 | 0.02009 |
| GO:0010899 | regulation of phosphatidylcholine catabolic process | 0.666667 | 0.02009 |
| GO:0010273 | detoxification of copper ion | 0.666667 | 0.02009 |
| GO:0070949 | regulation of neutrophil mediated killing of symbiont cell | 0.666667 | 0.02009 |
| GO:2000233 | negative regulation of rRNA processing | 0.666667 | 0.02009 |
| GO:0046951 | ketone body biosynthetic process | 0.666667 | 0.02009 |
| GO:0000105 | histidine biosynthetic process | 0.666667 | 0.02009 |
| GO:0070829 | obsolete heterochromatin maintenance | 0.666667 | 0.02009 |
| GO:0061577 | calcium ion transmembrane transport via high voltage-gated calcium channel | 0.666667 | 0.02009 |
| GO:1903575 | cornified envelope assembly | 0.666667 | 0.02009 |
| GO:0070278 | extracellular matrix constituent secretion | 0.666667 | 0.02009 |
| GO:0010255 | glucose mediated signaling pathway | 0.666667 | 0.02009 |
| GO:0010166 | wax metabolic process | 0.666667 | 0.02009 |
| GO:2000297 | negative regulation of synapse maturation | 0.666667 | 0.02009 |
| GO:0043564 | Ku70:Ku80 complex | 0.666667 | 0.02009 |
| GO:0097451 | glial limiting end-foot | 0.666667 | 0.02009 |
| GO:0036502 | Derlin-1-VIMP complex | 0.666667 | 0.02009 |
| GO:0071735 | IgG immunoglobulin complex | 0.666667 | 0.02009 |
| GO:0033093 | Weibel-Palade body | 0.666667 | 0.02009 |
| GO:0031084 | BLOC-2 complex | 0.666667 | 0.02009 |
| GO:0097059 | CNTFR-CLCF1 complex | 0.666667 | 0.02009 |
| GO:0071953 | elastic fiber | 0.666667 | 0.02009 |
| GO:0035976 | transcription factor AP-1 complex | 0.666667 | 0.02009 |
| GO:0051870 | methotrexate binding | 0.666667 | 0.02009 |
| GO:0050816 | phosphothreonine residue binding | 0.666667 | 0.02009 |
| GO:0031997 | N-terminal myristoylation domain binding | 0.666667 | 0.02009 |
| GO:0004689 | phosphorylase kinase activity | 0.666667 | 0.02009 |
| GO:0048101 | calmodulin-activated 3',5'-cyclic-GMP phosphodiesterase activity | 0.666667 | 0.02009 |
| GO:0015143 | urate transmembrane transporter activity | 0.666667 | 0.02009 |
| GO:0019862 | IgA binding | 0.666667 | 0.02009 |
| GO:0018479 | benzaldehyde dehydrogenase (NAD+) activity | 0.666667 | 0.02009 |
| GO:0000701 | purine-specific mismatch base pair DNA N-glycosylase activity | 0.666667 | 0.02009 |
| GO:0031800 | type 3 metabotropic glutamate receptor binding | 0.666667 | 0.02009 |
| GO:0120160 | intraciliary transport particle A binding | 0.666667 | 0.02009 |
| GO:0016941 | natriuretic peptide receptor activity | 0.666667 | 0.02009 |
| GO:0003960 | NADPH:quinone reductase activity | 0.666667 | 0.02009 |
| GO:0030172 | troponin C binding | 0.666667 | 0.02009 |
| GO:1901702 | salt transmembrane transporter activity | 0.666667 | 0.02009 |
| GO:0004775 | succinate-CoA ligase (ADP-forming) activity | 0.666667 | 0.02009 |
| GO:0031208 | POZ domain binding | 0.666667 | 0.02009 |
| GO:0004967 | glucagon receptor activity | 0.666667 | 0.02009 |
| GO:0004117 | calmodulin-activated dual specificity 3',5'-cyclic-GMP, 3',5'-cyclic-AMP phosphodiesterase activity | 0.666667 | 0.02009 |
| GO:0004174 | electron-transferring-flavoprotein dehydrogenase activity | 0.666667 | 0.02009 |
| GO:0031852 | mu-type opioid receptor binding | 0.666667 | 0.02009 |
| GO:0002831 | regulation of response to biotic stimulus | 0.118421 | 0.02007 |
| GO:0072073 | kidney epithelium development | 0.183673 | 0.01987 |
| GO:0001954 | positive regulation of cell-matrix adhesion | 0.183673 | 0.01987 |
| GO:0030864 | cortical actin cytoskeleton | 0.183673 | 0.01987 |
| GO:0004033 | aldo-keto reductase (NADP) activity | 0.183673 | 0.01987 |
| GO:0015748 | organophosphate ester transport | 0.14876 | 0.01976 |
| GO:0050778 | positive regulation of immune response | 0.110272 | 0.01967 |
| GO:0046777 | protein autophosphorylation | 0.136364 | 0.01961 |
| GO:0003013 | circulatory system process | 0.136364 | 0.01961 |
| GO:0050691 | regulation of defense response to virus by host | 0.195122 | 0.01946 |
| GO:0005751 | mitochondrial respiratory chain complex IV | 0.195122 | 0.01946 |
| GO:0050863 | regulation of T cell activation | 0.123711 | 0.01942 |
| GO:0050872 | white fat cell differentiation | 0.307692 | 0.01935 |
| GO:0097194 | execution phase of apoptosis | 0.307692 | 0.01935 |
| GO:0036336 | dendritic cell migration | 0.307692 | 0.01935 |
| GO:0023058 | adaptation of signaling pathway | 0.307692 | 0.01935 |
| GO:0033700 | phospholipid efflux | 0.307692 | 0.01935 |
| GO:0021670 | lateral ventricle development | 0.307692 | 0.01935 |
| GO:0046834 | lipid phosphorylation | 0.307692 | 0.01935 |
| GO:0016024 | CDP-diacylglycerol biosynthetic process | 0.307692 | 0.01935 |
| GO:0071257 | cellular response to electrical stimulus | 0.307692 | 0.01935 |
| GO:1904478 | regulation of intestinal absorption | 0.307692 | 0.01935 |
| GO:0060099 | regulation of phagocytosis, engulfment | 0.307692 | 0.01935 |
| GO:0048857 | neural nucleus development | 0.307692 | 0.01935 |
| GO:0034362 | low-density lipoprotein particle | 0.307692 | 0.01935 |
| GO:0000346 | transcription export complex | 0.307692 | 0.01935 |
| GO:0005523 | tropomyosin binding | 0.307692 | 0.01935 |
| GO:0042171 | lysophosphatidic acid acyltransferase activity | 0.307692 | 0.01935 |
| GO:0005159 | insulin-like growth factor receptor binding | 0.307692 | 0.01935 |
| GO:0031748 | D1 dopamine receptor binding | 0.307692 | 0.01935 |
| GO:0004535 | poly(A)-specific ribonuclease activity | 0.307692 | 0.01935 |
| GO:0000724 | double-strand break repair via homologous recombination | 0.153846 | 0.01931 |
| GO:0014910 | regulation of smooth muscle cell migration | 0.174603 | 0.01929 |
| GO:0006487 | protein N-linked glycosylation | 0.174603 | 0.01929 |
| GO:0016835 | carbon-oxygen lyase activity | 0.15 | 0.01924 |
| GO:0006515 | protein quality control for misfolded or incompletely synthesized proteins | 0.230769 | 0.01883 |
| GO:0046209 | nitric oxide metabolic process | 0.230769 | 0.01883 |
| GO:0007035 | vacuolar acidification | 0.230769 | 0.01883 |
| GO:0032527 | protein exit from endoplasmic reticulum | 0.230769 | 0.01883 |
| GO:1901663 | quinone biosynthetic process | 0.230769 | 0.01883 |
| GO:0048741 | skeletal muscle fiber development | 0.230769 | 0.01883 |
| GO:0090102 | cochlea development | 0.230769 | 0.01883 |
| GO:0030832 | regulation of actin filament length | 0.137931 | 0.01877 |
| GO:0098802 | plasma membrane signaling receptor complex | 0.137931 | 0.01877 |
| GO:0005516 | calmodulin binding | 0.134021 | 0.01871 |
| GO:0062197 | cellular response to chemical stress | 0.129707 | 0.01859 |
| GO:2001233 | regulation of apoptotic signaling pathway | 0.117794 | 0.01845 |
| GO:0055076 | transition metal ion homeostasis | 0.14 | 0.01832 |
| GO:0030018 | Z disc | 0.14 | 0.01832 |
| GO:0051087 | chaperone binding | 0.143939 | 0.01830 |
| GO:0035384 | thioester biosynthetic process | 0.212121 | 0.01818 |
| GO:0016226 | iron-sulfur cluster assembly | 0.212121 | 0.01818 |
| GO:0071616 | acyl-CoA biosynthetic process | 0.212121 | 0.01818 |
| GO:0061564 | axon development | 0.212121 | 0.01818 |
| GO:0042559 | pteridine-containing compound biosynthetic process | 0.212121 | 0.01818 |
| GO:0033619 | membrane protein proteolysis | 0.212121 | 0.01818 |
| GO:0031163 | metallo-sulfur cluster assembly | 0.212121 | 0.01818 |
| GO:0016701 | oxidoreductase activity, acting on single donors with incorporation of molecular oxygen | 0.212121 | 0.01818 |
| GO:0019865 | immunoglobulin binding | 0.212121 | 0.01818 |
| GO:0008175 | tRNA methyltransferase activity | 0.212121 | 0.01818 |
| GO:0006107 | oxaloacetate metabolic process | 0.263158 | 0.01806 |
| GO:0030728 | ovulation | 0.263158 | 0.01806 |
| GO:0050435 | amyloid-beta metabolic process | 0.263158 | 0.01806 |
| GO:0045472 | response to ether | 0.263158 | 0.01806 |
| GO:0072009 | nephron epithelium development | 0.263158 | 0.01806 |
| GO:0006509 | membrane protein ectodomain proteolysis | 0.263158 | 0.01806 |
| GO:0045737 | positive regulation of cyclin-dependent protein serine/threonine kinase activity | 0.263158 | 0.01806 |
| GO:1903579 | negative regulation of ATP metabolic process | 0.263158 | 0.01806 |
| GO:0004467 | long-chain fatty acid-CoA ligase activity | 0.263158 | 0.01806 |
| GO:0070555 | response to interleukin-1 | 0.156863 | 0.01802 |
| GO:0007272 | ensheathment of neurons | 0.156863 | 0.01802 |
| GO:0008366 | axon ensheathment | 0.156863 | 0.01802 |
| GO:0032436 | positive regulation of proteasomal ubiquitin-dependent protein catabolic process | 0.16092 | 0.01800 |
| GO:0098754 | detoxification | 0.177419 | 0.01798 |
| GO:0019748 | secondary metabolic process | 0.177419 | 0.01798 |
| GO:0031228 | intrinsic component of Golgi membrane | 0.177419 | 0.01798 |
| GO:0005938 | cell cortex | 0.128755 | 0.01790 |
| GO:0045596 | negative regulation of cell differentiation | 0.108497 | 0.01790 |
| GO:0006399 | tRNA metabolic process | 0.127413 | 0.01773 |
| GO:0004553 | hydrolase activity, hydrolyzing O-glycosyl compounds | 0.149123 | 0.01772 |
| GO:0005741 | mitochondrial outer membrane | 0.136126 | 0.01763 |
| GO:0043536 | positive regulation of blood vessel endothelial cell migration | 0.1875 | 0.01749 |
| GO:0042181 | ketone biosynthetic process | 0.1875 | 0.01749 |
| GO:0030017 | sarcomere | 0.1875 | 0.01749 |
| GO:1901618 | organic hydroxy compound transmembrane transporter activity | 0.1875 | 0.01749 |
| GO:0051539 | 4 iron, 4 sulfur cluster binding | 0.1875 | 0.01749 |
| GO:0010977 | negative regulation of neuron projection development | 0.145038 | 0.01745 |
| GO:0055037 | recycling endosome | 0.145038 | 0.01745 |
| GO:0008047 | enzyme activator activity | 0.11204 | 0.01742 |
| GO:0048598 | embryonic morphogenesis | 0.119289 | 0.01732 |
| GO:0030246 | carbohydrate binding | 0.122388 | 0.01719 |
| GO:0044265 | cellular macromolecule catabolic process | 0.106982 | 0.01709 |
| GO:0018022 | peptidyl-lysine methylation | 0.162791 | 0.01707 |
| GO:1901616 | organic hydroxy compound catabolic process | 0.162791 | 0.01707 |
| GO:1901215 | negative regulation of neuron death | 0.133333 | 0.01697 |
| GO:0033143 | regulation of intracellular steroid hormone receptor signaling pathway | 0.169014 | 0.01692 |
| GO:0071326 | cellular response to monosaccharide stimulus | 0.169014 | 0.01692 |
| GO:0009261 | ribonucleotide catabolic process | 0.2 | 0.01688 |
| GO:0060076 | excitatory synapse | 0.180328 | 0.01684 |
| GO:2000378 | negative regulation of reactive oxygen species metabolic process | 0.150442 | 0.01677 |
| GO:0140535 | intracellular protein-containing complex | 0.103741 | 0.01658 |
| GO:0090263 | positive regulation of canonical Wnt signaling pathway | 0.15625 | 0.01638 |
| GO:1905477 | positive regulation of protein localization to membrane | 0.15625 | 0.01638 |
| GO:0043434 | response to peptide hormone | 0.120787 | 0.01632 |
| GO:0097722 | sperm motility | 0.164706 | 0.01626 |
| GO:0036503 | ERAD pathway | 0.164706 | 0.01626 |
| GO:0031060 | regulation of histone methylation | 0.164706 | 0.01626 |
| GO:0051492 | regulation of stress fiber assembly | 0.164706 | 0.01626 |
| GO:0050764 | regulation of phagocytosis | 0.164706 | 0.01626 |
| GO:1901136 | carbohydrate derivative catabolic process | 0.142857 | 0.01624 |
| GO:0034284 | response to monosaccharide | 0.142857 | 0.01624 |
| GO:0006685 | sphingomyelin catabolic process | 0.428571 | 0.01614 |
| GO:0034115 | negative regulation of heterotypic cell-cell adhesion | 0.428571 | 0.01614 |
| GO:0070587 | regulation of cell-cell adhesion involved in gastrulation | 0.428571 | 0.01614 |
| GO:0034638 | phosphatidylcholine catabolic process | 0.428571 | 0.01614 |
| GO:0006559 | L-phenylalanine catabolic process | 0.428571 | 0.01614 |
| GO:0010616 | negative regulation of cardiac muscle adaptation | 0.428571 | 0.01614 |
| GO:0051694 | pointed-end actin filament capping | 0.428571 | 0.01614 |
| GO:0048668 | collateral sprouting | 0.428571 | 0.01614 |
| GO:0051823 | regulation of synapse structural plasticity | 0.428571 | 0.01614 |
| GO:1902222 | erythrose 4-phosphate/phosphoenolpyruvate family amino acid catabolic process | 0.428571 | 0.01614 |
| GO:0002727 | regulation of natural killer cell cytokine production | 0.428571 | 0.01614 |
| GO:0019368 | fatty acid elongation, unsaturated fatty acid | 0.428571 | 0.01614 |
| GO:1902267 | regulation of polyamine transmembrane transport | 0.428571 | 0.01614 |
| GO:1905063 | regulation of vascular associated smooth muscle cell differentiation | 0.428571 | 0.01614 |
| GO:0009155 | purine deoxyribonucleotide catabolic process | 0.428571 | 0.01614 |
| GO:0034625 | fatty acid elongation, monounsaturated fatty acid | 0.428571 | 0.01614 |
| GO:0034626 | fatty acid elongation, polyunsaturated fatty acid | 0.428571 | 0.01614 |
| GO:1903894 | regulation of IRE1-mediated unfolded protein response | 0.428571 | 0.01614 |
| GO:0032353 | negative regulation of hormone biosynthetic process | 0.428571 | 0.01614 |
| GO:0044691 | tooth eruption | 0.428571 | 0.01614 |
| GO:0009256 | 10-formyltetrahydrofolate metabolic process | 0.428571 | 0.01614 |
| GO:0006003 | fructose 2,6-bisphosphate metabolic process | 0.428571 | 0.01614 |
| GO:0018401 | peptidyl-proline hydroxylation to 4-hydroxy-L-proline | 0.428571 | 0.01614 |
| GO:0051415 | microtubule nucleation by interphase microtubule organizing center | 0.428571 | 0.01614 |
| GO:0032490 | detection of molecule of bacterial origin | 0.428571 | 0.01614 |
| GO:0070841 | inclusion body assembly | 0.428571 | 0.01614 |
| GO:0070842 | aggresome assembly | 0.428571 | 0.01614 |
| GO:0070782 | phosphatidylserine exposure on apoptotic cell surface | 0.428571 | 0.01614 |
| GO:0042587 | glycogen granule | 0.428571 | 0.01614 |
| GO:0005785 | signal recognition particle receptor complex | 0.428571 | 0.01614 |
| GO:0102337 | 3-oxo-cerotoyl-CoA synthase activity | 0.428571 | 0.01614 |
| GO:0102336 | 3-oxo-arachidoyl-CoA synthase activity | 0.428571 | 0.01614 |
| GO:0102338 | 3-oxo-lignoceronyl-CoA synthase activity | 0.428571 | 0.01614 |
| GO:0050733 | RS domain binding | 0.428571 | 0.01614 |
| GO:0008093 | cytoskeletal anchor activity | 0.428571 | 0.01614 |
| GO:0031545 | peptidyl-proline 4-dioxygenase activity | 0.428571 | 0.01614 |
| GO:0008073 | ornithine decarboxylase inhibitor activity | 0.428571 | 0.01614 |
| GO:0043878 | glyceraldehyde-3-phosphate dehydrogenase (NAD+) (non-phosphorylating) activity | 0.428571 | 0.01614 |
| GO:0098800 | inner mitochondrial membrane protein complex | 0.122699 | 0.01596 |
| GO:0005930 | axoneme | 0.151786 | 0.01590 |
| GO:0046686 | response to cadmium ion | 0.183333 | 0.01587 |
| GO:0016055 | Wnt signaling pathway | 0.128514 | 0.01584 |
| GO:0006281 | DNA repair | 0.112013 | 0.01569 |
| GO:0051291 | protein heterooligomerization | 0.143836 | 0.01568 |
| GO:0019867 | outer membrane | 0.132159 | 0.01567 |
| GO:0031968 | organelle outer membrane | 0.132159 | 0.01567 |
| GO:0050866 | negative regulation of cell activation | 0.136612 | 0.01563 |
| GO:0050858 | negative regulation of antigen receptor-mediated signaling pathway | 0.24 | 0.01557 |
| GO:1902991 | regulation of amyloid precursor protein catabolic process | 0.24 | 0.01557 |
| GO:0120178 | steroid hormone biosynthetic process | 0.24 | 0.01557 |
| GO:0034694 | response to prostaglandin | 0.24 | 0.01557 |
| GO:1903318 | negative regulation of protein maturation | 0.24 | 0.01557 |
| GO:0006744 | ubiquinone biosynthetic process | 0.24 | 0.01557 |
| GO:0010955 | negative regulation of protein processing | 0.24 | 0.01557 |
| GO:0035025 | positive regulation of Rho protein signal transduction | 0.24 | 0.01557 |
| GO:0042744 | hydrogen peroxide catabolic process | 0.24 | 0.01557 |
| GO:1902235 | regulation of endoplasmic reticulum stress-induced intrinsic apoptotic signaling pathway | 0.24 | 0.01557 |
| GO:0043242 | negative regulation of protein-containing complex disassembly | 0.166667 | 0.01556 |
| GO:0043502 | regulation of muscle adaptation | 0.166667 | 0.01556 |
| GO:0030317 | flagellated sperm motility | 0.166667 | 0.01556 |
| GO:0032410 | negative regulation of transporter activity | 0.171429 | 0.01556 |
| GO:0071331 | cellular response to hexose stimulus | 0.171429 | 0.01556 |
| GO:0030433 | ubiquitin-dependent ERAD pathway | 0.171429 | 0.01556 |
| GO:0040018 | positive regulation of multicellular organism growth | 0.21875 | 0.01541 |
| GO:2000785 | regulation of autophagosome assembly | 0.21875 | 0.01541 |
| GO:0070269 | pyroptosis | 0.21875 | 0.01541 |
| GO:1901385 | regulation of voltage-gated calcium channel activity | 0.21875 | 0.01541 |
| GO:0071312 | cellular response to alkaloid | 0.21875 | 0.01541 |
| GO:0001848 | complement binding | 0.21875 | 0.01541 |
| GO:0016702 | oxidoreductase activity, acting on single donors with incorporation of molecular oxygen, incorporation of two atoms of oxygen | 0.21875 | 0.01541 |
| GO:0080171 | lytic vacuole organization | 0.191489 | 0.01533 |
| GO:2000772 | regulation of cellular senescence | 0.191489 | 0.01533 |
| GO:0007040 | lysosome organization | 0.191489 | 0.01533 |
| GO:0031503 | protein-containing complex localization | 0.144828 | 0.01519 |
| GO:0010595 | positive regulation of endothelial cell migration | 0.168675 | 0.01499 |
| GO:0016860 | intramolecular oxidoreductase activity | 0.168675 | 0.01499 |
| GO:0032388 | positive regulation of intracellular transport | 0.138122 | 0.01477 |
| GO:0140115 | export across plasma membrane | 0.205128 | 0.01455 |
| GO:0012501 | programmed cell death | 0.106122 | 0.01452 |
| GO:0050905 | neuromuscular process | 0.154545 | 0.01443 |
| GO:0051213 | dioxygenase activity | 0.154545 | 0.01443 |
| GO:0001568 | blood vessel development | 0.146853 | 0.01441 |
| GO:1990351 | transporter complex | 0.116883 | 0.01436 |
| GO:0050672 | negative regulation of lymphocyte proliferation | 0.173913 | 0.01435 |
| GO:0032945 | negative regulation of mononuclear cell proliferation | 0.173913 | 0.01435 |
| GO:0008235 | metalloexopeptidase activity | 0.173913 | 0.01435 |
| GO:0031341 | regulation of cell killing | 0.150794 | 0.01434 |
| GO:0035162 | embryonic hemopoiesis | 0.333333 | 0.01434 |
| GO:0045656 | negative regulation of monocyte differentiation | 0.333333 | 0.01434 |
| GO:0045916 | negative regulation of complement activation | 0.333333 | 0.01434 |
| GO:0000050 | urea cycle | 0.333333 | 0.01434 |
| GO:0097106 | postsynaptic density organization | 0.333333 | 0.01434 |
| GO:0015701 | bicarbonate transport | 0.333333 | 0.01434 |
| GO:0098693 | regulation of synaptic vesicle cycle | 0.333333 | 0.01434 |
| GO:0010872 | regulation of cholesterol esterification | 0.333333 | 0.01434 |
| GO:0006699 | bile acid biosynthetic process | 0.333333 | 0.01434 |
| GO:0051151 | negative regulation of smooth muscle cell differentiation | 0.333333 | 0.01434 |
| GO:0019627 | urea metabolic process | 0.333333 | 0.01434 |
| GO:0046689 | response to mercury ion | 0.333333 | 0.01434 |
| GO:0031223 | auditory behavior | 0.333333 | 0.01434 |
| GO:0002638 | negative regulation of immunoglobulin production | 0.333333 | 0.01434 |
| GO:0006171 | cAMP biosynthetic process | 0.333333 | 0.01434 |
| GO:0099590 | neurotransmitter receptor internalization | 0.333333 | 0.01434 |
| GO:0006957 | complement activation, alternative pathway | 0.333333 | 0.01434 |
| GO:0072710 | response to hydroxyurea | 0.333333 | 0.01434 |
| GO:0099026 | anchored component of presynaptic membrane | 0.333333 | 0.01434 |
| GO:0030915 | Smc5-Smc6 complex | 0.333333 | 0.01434 |
| GO:0005583 | fibrillar collagen trimer | 0.333333 | 0.01434 |
| GO:0048039 | ubiquinone binding | 0.333333 | 0.01434 |
| GO:0016783 | sulfurtransferase activity | 0.333333 | 0.01434 |
| GO:0003680 | minor groove of adenine-thymine-rich DNA binding | 0.333333 | 0.01434 |
| GO:0003691 | double-stranded telomeric DNA binding | 0.333333 | 0.01434 |
| GO:0060294 | cilium movement involved in cell motility | 0.159574 | 0.01433 |
| GO:0006646 | phosphatidylethanolamine biosynthetic process | 0.277778 | 0.01428 |
| GO:0035751 | regulation of lysosomal lumen pH | 0.277778 | 0.01428 |
| GO:0001892 | embryonic placenta development | 0.277778 | 0.01428 |
| GO:0051481 | negative regulation of cytosolic calcium ion concentration | 0.277778 | 0.01428 |
| GO:0046386 | deoxyribose phosphate catabolic process | 0.277778 | 0.01428 |
| GO:0031998 | regulation of fatty acid beta-oxidation | 0.277778 | 0.01428 |
| GO:0044849 | estrous cycle | 0.277778 | 0.01428 |
| GO:0032354 | response to follicle-stimulating hormone | 0.277778 | 0.01428 |
| GO:0006896 | Golgi to vacuole transport | 0.277778 | 0.01428 |
| GO:2001256 | regulation of store-operated calcium entry | 0.277778 | 0.01428 |
| GO:0032703 | negative regulation of interleukin-2 production | 0.277778 | 0.01428 |
| GO:0050860 | negative regulation of T cell receptor signaling pathway | 0.277778 | 0.01428 |
| GO:0031091 | platelet alpha granule | 0.277778 | 0.01428 |
| GO:0099144 | anchored component of synaptic membrane | 0.277778 | 0.01428 |
| GO:0050998 | nitric-oxide synthase binding | 0.277778 | 0.01428 |
| GO:0099699 | integral component of synaptic membrane | 0.135678 | 0.01421 |
| GO:0052689 | carboxylic ester hydrolase activity | 0.135678 | 0.01421 |
| GO:0034703 | cation channel complex | 0.133028 | 0.01387 |
| GO:0006401 | RNA catabolic process | 0.137056 | 0.01361 |
| GO:0098573 | intrinsic component of mitochondrial membrane | 0.16129 | 0.01345 |
| GO:0009888 | tissue development | 0.113752 | 0.01342 |
| GO:0048024 | regulation of mRNA splicing, via spliceosome | 0.145985 | 0.01341 |
| GO:0031111 | negative regulation of microtubule polymerization or depolymerization | 0.195652 | 0.01337 |
| GO:0051283 | negative regulation of sequestering of calcium ion | 0.195652 | 0.01337 |
| GO:0010662 | regulation of striated muscle cell apoptotic process | 0.195652 | 0.01337 |
| GO:0008106 | alcohol dehydrogenase (NADP+) activity | 0.195652 | 0.01337 |
| GO:0018108 | peptidyl-tyrosine phosphorylation | 0.176471 | 0.01327 |
| GO:0009166 | nucleotide catabolic process | 0.176471 | 0.01327 |
| GO:0022604 | regulation of cell morphogenesis | 0.125806 | 0.01320 |
| GO:0061179 | negative regulation of insulin secretion involved in cellular response to glucose stimulus | 0.225806 | 0.01296 |
| GO:0034698 | response to gonadotropin | 0.225806 | 0.01296 |
| GO:0001658 | branching involved in ureteric bud morphogenesis | 0.225806 | 0.01296 |
| GO:0001838 | embryonic epithelial tube formation | 0.225806 | 0.01296 |
| GO:0042168 | heme metabolic process | 0.225806 | 0.01296 |
| GO:0032232 | negative regulation of actin filament bundle assembly | 0.225806 | 0.01296 |
| GO:0031210 | phosphatidylcholine binding | 0.225806 | 0.01296 |
| GO:0004112 | cyclic-nucleotide phosphodiesterase activity | 0.225806 | 0.01296 |
| GO:0008064 | regulation of actin polymerization or depolymerization | 0.139535 | 0.01291 |
| GO:0030099 | myeloid cell differentiation | 0.139535 | 0.01291 |
| GO:0032675 | regulation of interleukin-6 production | 0.151261 | 0.01281 |
| GO:0070663 | regulation of leukocyte proliferation | 0.135514 | 0.01275 |
| GO:0002828 | regulation of type 2 immune response | 0.25 | 0.01272 |
| GO:0033627 | cell adhesion mediated by integrin | 0.25 | 0.01272 |
| GO:0000097 | sulfur amino acid biosynthetic process | 0.25 | 0.01272 |
| GO:0006613 | cotranslational protein targeting to membrane | 0.25 | 0.01272 |
| GO:0001846 | opsonin binding | 0.25 | 0.01272 |
| GO:0005158 | insulin receptor binding | 0.25 | 0.01272 |
| GO:0072507 | divalent inorganic cation homeostasis | 0.132479 | 0.01250 |
| GO:0032006 | regulation of TOR signaling | 0.160377 | 0.01247 |
| GO:0000725 | recombinational repair | 0.160377 | 0.01247 |
| GO:0050868 | negative regulation of T cell activation | 0.160377 | 0.01247 |
| GO:2000249 | regulation of actin cytoskeleton reorganization | 0.210526 | 0.01246 |
| GO:0070059 | intrinsic apoptotic signaling pathway in response to endoplasmic reticulum stress | 0.210526 | 0.01246 |
| GO:0002673 | regulation of acute inflammatory response | 0.210526 | 0.01246 |
| GO:0009154 | purine ribonucleotide catabolic process | 0.210526 | 0.01246 |
| GO:2000351 | regulation of endothelial cell apoptotic process | 0.210526 | 0.01246 |
| GO:0019373 | epoxygenase P450 pathway | 0.210526 | 0.01246 |
| GO:0070527 | platelet aggregation | 0.210526 | 0.01246 |
| GO:0048660 | regulation of smooth muscle cell proliferation | 0.148148 | 0.01216 |
| GO:0046916 | cellular transition metal ion homeostasis | 0.152542 | 0.01209 |
| GO:0110020 | regulation of actomyosin structure organization | 0.164835 | 0.01198 |
| GO:1902882 | regulation of response to oxidative stress | 0.164835 | 0.01198 |
| GO:0034766 | negative regulation of ion transmembrane transport | 0.164835 | 0.01198 |
| GO:0005902 | microvillus | 0.164835 | 0.01198 |
| GO:0032592 | integral component of mitochondrial membrane | 0.164835 | 0.01198 |
| GO:0000987 | cis-regulatory region sequence-specific DNA binding | 0.109572 | 0.01179 |
| GO:0045333 | cellular respiration | 0.135266 | 0.01174 |
| GO:2001235 | positive regulation of apoptotic signaling pathway | 0.149254 | 0.01163 |
| GO:0005496 | steroid binding | 0.149254 | 0.01163 |
| GO:0033866 | nucleoside bisphosphate biosynthetic process | 0.2 | 0.01160 |
| GO:0034030 | ribonucleoside bisphosphate biosynthetic process | 0.2 | 0.01160 |
| GO:0034033 | purine nucleoside bisphosphate biosynthetic process | 0.2 | 0.01160 |
| GO:0061001 | regulation of dendritic spine morphogenesis | 0.2 | 0.01160 |
| GO:0043014 | alpha-tubulin binding | 0.2 | 0.01160 |
| GO:0043394 | proteoglycan binding | 0.2 | 0.01160 |
| GO:0051047 | positive regulation of secretion | 0.122016 | 0.01155 |
| GO:1905114 | cell surface receptor signaling pathway involved in cell-cell signaling | 0.131474 | 0.01151 |
| GO:0099512 | supramolecular fiber | 0.10718 | 0.01150 |
| GO:0099081 | supramolecular polymer | 0.10718 | 0.01150 |
| GO:0008233 | peptidase activity | 0.10718 | 0.01150 |
| GO:0019646 | aerobic electron transport chain | 0.153846 | 0.01144 |
| GO:1903038 | negative regulation of leukocyte cell-cell adhesion | 0.153846 | 0.01144 |
| GO:0006898 | receptor-mediated endocytosis | 0.153846 | 0.01144 |
| GO:0098858 | actin-based cell projection | 0.139037 | 0.01140 |
| GO:0047485 | protein N-terminus binding | 0.150376 | 0.01116 |
| GO:0010755 | regulation of plasminogen activation | 0.294118 | 0.01107 |
| GO:1903513 | endoplasmic reticulum to cytosol transport | 0.294118 | 0.01107 |
| GO:0051938 | L-glutamate import | 0.294118 | 0.01107 |
| GO:0031643 | positive regulation of myelination | 0.294118 | 0.01107 |
| GO:0009264 | deoxyribonucleotide catabolic process | 0.294118 | 0.01107 |
| GO:0055070 | copper ion homeostasis | 0.294118 | 0.01107 |
| GO:0042574 | retinal metabolic process | 0.294118 | 0.01107 |
| GO:0033194 | response to hydroperoxide | 0.294118 | 0.01107 |
| GO:0031065 | positive regulation of histone deacetylation | 0.294118 | 0.01107 |
| GO:0030970 | retrograde protein transport, ER to cytosol | 0.294118 | 0.01107 |
| GO:0097449 | astrocyte projection | 0.294118 | 0.01107 |
| GO:0071256 | translocon complex | 0.294118 | 0.01107 |
| GO:0071617 | lysophospholipid acyltransferase activity | 0.294118 | 0.01107 |
| GO:0101020 | estrogen 16-alpha-hydroxylase activity | 0.294118 | 0.01107 |
| GO:0005542 | folic acid binding | 0.294118 | 0.01107 |
| GO:1902532 | negative regulation of intracellular signal transduction | 0.116412 | 0.01105 |
| GO:1905952 | regulation of lipid localization | 0.143713 | 0.01095 |
| GO:0045637 | regulation of myeloid cell differentiation | 0.133929 | 0.01088 |
| GO:0031346 | positive regulation of cell projection organization | 0.120907 | 0.01087 |
| GO:0005762 | mitochondrial large ribosomal subunit | 0.168539 | 0.01086 |
| GO:0000315 | organellar large ribosomal subunit | 0.168539 | 0.01086 |
| GO:1903707 | negative regulation of hemopoiesis | 0.155172 | 0.01085 |
| GO:0050684 | regulation of mRNA processing | 0.140541 | 0.01082 |
| GO:0051781 | positive regulation of cell division | 0.173333 | 0.01081 |
| GO:0002701 | negative regulation of production of molecular mediator of immune response | 0.233333 | 0.01080 |
| GO:0032469 | endoplasmic reticulum calcium ion homeostasis | 0.233333 | 0.01080 |
| GO:0004114 | 3',5'-cyclic-nucleotide phosphodiesterase activity | 0.233333 | 0.01080 |
| GO:0050433 | regulation of catecholamine secretion | 0.184615 | 0.01078 |
| GO:0010564 | regulation of cell cycle process | 0.111413 | 0.01077 |
| GO:0000902 | cell morphogenesis | 0.131687 | 0.01063 |
| GO:0009311 | oligosaccharide metabolic process | 0.216216 | 0.01060 |
| GO:0048038 | quinone binding | 0.216216 | 0.01060 |
| GO:0005272 | sodium channel activity | 0.216216 | 0.01060 |
| GO:1990778 | protein localization to cell periphery | 0.134529 | 0.01058 |
| GO:0022409 | positive regulation of cell-cell adhesion | 0.134529 | 0.01058 |
| GO:0006099 | tricarboxylic acid cycle | 0.192308 | 0.01058 |
| GO:0051385 | response to mineralocorticoid | 0.192308 | 0.01058 |
| GO:0033764 | steroid dehydrogenase activity, acting on the CH-OH group of donors, NAD or NADP as acceptor | 0.192308 | 0.01058 |
| GO:0003018 | vascular process in circulatory system | 0.148649 | 0.01057 |
| GO:0071495 | cellular response to endogenous stimulus | 0.108623 | 0.01043 |
| GO:0043467 | regulation of generation of precursor metabolites and energy | 0.152672 | 0.01039 |
| GO:0019722 | calcium-mediated signaling | 0.152672 | 0.01039 |
| GO:0042826 | histone deacetylase binding | 0.152672 | 0.01039 |
| GO:0001505 | regulation of neurotransmitter levels | 0.142077 | 0.01039 |
| GO:0033558 | protein lysine deacetylase activity | 0.156522 | 0.01033 |
| GO:0050670 | regulation of lymphocyte proliferation | 0.138614 | 0.01029 |
| GO:0006555 | methionine metabolic process | 0.26087 | 0.01026 |
| GO:0006614 | SRP-dependent cotranslational protein targeting to membrane | 0.26087 | 0.01026 |
| GO:2000352 | negative regulation of endothelial cell apoptotic process | 0.26087 | 0.01026 |
| GO:0120015 | sterol transfer activity | 0.26087 | 0.01026 |
| GO:0120020 | cholesterol transfer activity | 0.26087 | 0.01026 |
| GO:0031435 | mitogen-activated protein kinase kinase kinase binding | 0.26087 | 0.01026 |
| GO:0043395 | heparan sulfate proteoglycan binding | 0.26087 | 0.01026 |
| GO:0099054 | presynapse assembly | 0.363636 | 0.01023 |
| GO:0010524 | positive regulation of calcium ion transport into cytosol | 0.363636 | 0.01023 |
| GO:1903025 | regulation of RNA polymerase II regulatory region sequence-specific DNA binding | 0.363636 | 0.01023 |
| GO:0072376 | protein activation cascade | 0.363636 | 0.01023 |
| GO:0006012 | galactose metabolic process | 0.363636 | 0.01023 |
| GO:0034145 | positive regulation of toll-like receptor 4 signaling pathway | 0.363636 | 0.01023 |
| GO:0097205 | renal filtration | 0.363636 | 0.01023 |
| GO:0002890 | negative regulation of immunoglobulin mediated immune response | 0.363636 | 0.01023 |
| GO:0072711 | cellular response to hydroxyurea | 0.363636 | 0.01023 |
| GO:0006855 | xenobiotic transmembrane transport | 0.363636 | 0.01023 |
| GO:0002713 | negative regulation of B cell mediated immunity | 0.363636 | 0.01023 |
| GO:0097512 | cardiac myofibril | 0.363636 | 0.01023 |
| GO:0061700 | GATOR2 complex | 0.363636 | 0.01023 |
| GO:0048787 | presynaptic active zone membrane | 0.363636 | 0.01023 |
| GO:0050308 | sugar-phosphatase activity | 0.363636 | 0.01023 |
| GO:0043121 | neurotrophin binding | 0.363636 | 0.01023 |
| GO:0003841 | 1-acylglycerol-3-phosphate O-acyltransferase activity | 0.363636 | 0.01023 |
| GO:0034637 | cellular carbohydrate biosynthetic process | 0.1875 | 0.01019 |
| GO:0030512 | negative regulation of transforming growth factor beta receptor signaling pathway | 0.1875 | 0.01019 |
| GO:0002637 | regulation of immunoglobulin production | 0.1875 | 0.01019 |
| GO:0032092 | positive regulation of protein binding | 0.161616 | 0.01015 |
| GO:0071396 | cellular response to lipid | 0.114379 | 0.01007 |
| GO:0061024 | membrane organization | 0.112573 | 0.01004 |
| GO:0140096 | catalytic activity, acting on a protein | 0.095666 | 0.00996 |
| GO:0071347 | cellular response to interleukin-1 | 0.175676 | 0.00993 |
| GO:1901355 | response to rapamycin | 0.5 | 0.00983 |
| GO:0098943 | neurotransmitter receptor transport, postsynaptic endosome to lysosome | 0.5 | 0.00983 |
| GO:0043568 | positive regulation of insulin-like growth factor receptor signaling pathway | 0.5 | 0.00983 |
| GO:0031293 | membrane protein intracellular domain proteolysis | 0.5 | 0.00983 |
| GO:0038033 | positive regulation of endothelial cell chemotaxis by VEGF-activated vascular endothelial growth factor receptor signaling pathway | 0.5 | 0.00983 |
| GO:0034201 | response to oleic acid | 0.5 | 0.00983 |
| GO:0002467 | germinal center formation | 0.5 | 0.00983 |
| GO:0002729 | positive regulation of natural killer cell cytokine production | 0.5 | 0.00983 |
| GO:0021960 | anterior commissure morphogenesis | 0.5 | 0.00983 |
| GO:0150146 | cell junction disassembly | 0.5 | 0.00983 |
| GO:0015886 | heme transport | 0.5 | 0.00983 |
| GO:0070142 | synaptic vesicle budding | 0.5 | 0.00983 |
| GO:2000672 | negative regulation of motor neuron apoptotic process | 0.5 | 0.00983 |
| GO:0010519 | negative regulation of phospholipase activity | 0.5 | 0.00983 |
| GO:0046487 | glyoxylate metabolic process | 0.5 | 0.00983 |
| GO:0090324 | negative regulation of oxidative phosphorylation | 0.5 | 0.00983 |
| GO:0097119 | postsynaptic density protein 95 clustering | 0.5 | 0.00983 |
| GO:0072557 | IPAF inflammasome complex | 0.5 | 0.00983 |
| GO:0070419 | nonhomologous end joining complex | 0.5 | 0.00983 |
| GO:0033655 | host cell cytoplasm part | 0.5 | 0.00983 |
| GO:0000923 | equatorial microtubule organizing center | 0.5 | 0.00983 |
| GO:0016892 | endoribonuclease activity, producing 3'-phosphomonoesters | 0.5 | 0.00983 |
| GO:0008821 | crossover junction endodeoxyribonuclease activity | 0.5 | 0.00983 |
| GO:0005004 | GPI-linked ephrin receptor activity | 0.5 | 0.00983 |
| GO:0010856 | adenylate cyclase activator activity | 0.5 | 0.00983 |
| GO:0016406 | carnitine O-acyltransferase activity | 0.5 | 0.00983 |
| GO:0045088 | regulation of innate immune response | 0.133333 | 0.00981 |
| GO:0003697 | single-stranded DNA binding | 0.147887 | 0.00980 |
| GO:0005178 | integrin binding | 0.147887 | 0.00980 |
| GO:0015711 | organic anion transport | 0.130769 | 0.00974 |
| GO:0090068 | positive regulation of cell cycle process | 0.130769 | 0.00974 |
| GO:1902115 | regulation of organelle assembly | 0.136986 | 0.00971 |
| GO:0043393 | regulation of protein binding | 0.136986 | 0.00971 |
| GO:0006915 | apoptotic process | 0.108315 | 0.00963 |
| GO:0007623 | circadian rhythm | 0.144654 | 0.00959 |
| GO:0071496 | cellular response to external stimulus | 0.126126 | 0.00957 |
| GO:0031058 | positive regulation of histone modification | 0.163265 | 0.00950 |
| GO:0002443 | leukocyte mediated immunity | 0.163265 | 0.00950 |
| GO:0061136 | regulation of proteasomal protein catabolic process | 0.138462 | 0.00948 |
| GO:0098803 | respiratory chain complex | 0.138462 | 0.00948 |
| GO:1901657 | glycosyl compound metabolic process | 0.159292 | 0.00947 |
| GO:0015629 | actin cytoskeleton | 0.134454 | 0.00940 |
| GO:0009112 | nucleobase metabolic process | 0.196078 | 0.00922 |
| GO:0055086 | nucleobase-containing small molecule metabolic process | 0.106814 | 0.00919 |
| GO:0048704 | embryonic skeletal system morphogenesis | 0.178082 | 0.00915 |
| GO:0042734 | presynaptic membrane | 0.178082 | 0.00915 |
| GO:0016597 | amino acid binding | 0.178082 | 0.00915 |
| GO:0002833 | positive regulation of response to biotic stimulus | 0.139175 | 0.00913 |
| GO:0071806 | protein transmembrane transport | 0.170732 | 0.00901 |
| GO:0065002 | intracellular protein transmembrane transport | 0.170732 | 0.00901 |
| GO:1900407 | regulation of cellular response to oxidative stress | 0.170732 | 0.00901 |
| GO:0045923 | positive regulation of fatty acid metabolic process | 0.222222 | 0.00896 |
| GO:2000060 | positive regulation of ubiquitin-dependent protein catabolic process | 0.164948 | 0.00892 |
| GO:0042573 | retinoic acid metabolic process | 0.241379 | 0.00892 |
| GO:0090279 | regulation of calcium ion import | 0.241379 | 0.00892 |
| GO:0060079 | excitatory postsynaptic potential | 0.241379 | 0.00892 |
| GO:0061702 | inflammasome complex | 0.241379 | 0.00892 |
| GO:0004806 | triglyceride lipase activity | 0.241379 | 0.00892 |
| GO:0000904 | cell morphogenesis involved in differentiation | 0.15 | 0.00886 |
| GO:0042626 | ATPase-coupled transmembrane transporter activity | 0.15 | 0.00886 |
| GO:0048562 | embryonic organ morphogenesis | 0.162162 | 0.00885 |
| GO:0051289 | protein homotetramerization | 0.162162 | 0.00885 |
| GO:0044853 | plasma membrane raft | 0.154472 | 0.00871 |
| GO:0034654 | nucleobase-containing compound biosynthetic process | 0.108485 | 0.00864 |
| GO:0031514 | motile cilium | 0.143678 | 0.00861 |
| GO:0008156 | negative regulation of DNA replication | 0.209302 | 0.00859 |
| GO:0030261 | chromosome condensation | 0.209302 | 0.00859 |
| GO:1904352 | positive regulation of protein catabolic process in the vacuole | 0.209302 | 0.00859 |
| GO:0035591 | signaling adaptor activity | 0.209302 | 0.00859 |
| GO:0003700 | DNA-binding transcription factor activity | 0.104335 | 0.00851 |
| GO:0005874 | microtubule | 0.116364 | 0.00848 |
| GO:0006942 | regulation of striated muscle contraction | 0.180556 | 0.00846 |
| GO:0043621 | protein self-association | 0.180556 | 0.00846 |
| GO:0080182 | histone H3-K4 trimethylation | 0.3125 | 0.00839 |
| GO:0001841 | neural tube formation | 0.3125 | 0.00839 |
| GO:0015813 | L-glutamate transmembrane transport | 0.3125 | 0.00839 |
| GO:0010560 | positive regulation of glycoprotein biosynthetic process | 0.3125 | 0.00839 |
| GO:0015936 | coenzyme A metabolic process | 0.3125 | 0.00839 |
| GO:0099172 | presynapse organization | 0.3125 | 0.00839 |
| GO:0031579 | membrane raft organization | 0.3125 | 0.00839 |
| GO:0031204 | post-translational protein targeting to membrane, translocation | 0.3125 | 0.00839 |
| GO:0034310 | primary alcohol catabolic process | 0.3125 | 0.00839 |
| GO:0009074 | aromatic amino acid family catabolic process | 0.3125 | 0.00839 |
| GO:0051571 | positive regulation of histone H3-K4 methylation | 0.3125 | 0.00839 |
| GO:0032495 | response to muramyl dipeptide | 0.3125 | 0.00839 |
| GO:0019377 | glycolipid catabolic process | 0.3125 | 0.00839 |
| GO:0004303 | estradiol 17-beta-dehydrogenase activity | 0.3125 | 0.00839 |
| GO:0055117 | regulation of cardiac muscle contraction | 0.189655 | 0.00837 |
| GO:0008299 | isoprenoid biosynthetic process | 0.189655 | 0.00837 |
| GO:0042558 | pteridine-containing compound metabolic process | 0.189655 | 0.00837 |
| GO:0050805 | negative regulation of synaptic transmission | 0.189655 | 0.00837 |
| GO:0050679 | positive regulation of epithelial cell proliferation | 0.144509 | 0.00834 |
| GO:1904063 | negative regulation of cation transmembrane transport | 0.17284 | 0.00823 |
| GO:0019934 | cGMP-mediated signaling | 0.272727 | 0.00815 |
| GO:0042908 | xenobiotic transport | 0.272727 | 0.00815 |
| GO:0035337 | fatty-acyl-CoA metabolic process | 0.272727 | 0.00815 |
| GO:0060536 | cartilage morphogenesis | 0.272727 | 0.00815 |
| GO:0018196 | peptidyl-asparagine modification | 0.272727 | 0.00815 |
| GO:0043649 | dicarboxylic acid catabolic process | 0.272727 | 0.00815 |
| GO:0033643 | host cell part | 0.272727 | 0.00815 |
| GO:0010950 | positive regulation of endopeptidase activity | 0.145349 | 0.00811 |
| GO:0002009 | morphogenesis of an epithelium | 0.132353 | 0.00807 |
| GO:1901654 | response to ketone | 0.124661 | 0.00805 |
| GO:0042440 | pigment metabolic process | 0.2 | 0.00801 |
| GO:0060078 | regulation of postsynaptic membrane potential | 0.2 | 0.00801 |
| GO:0043457 | regulation of cellular respiration | 0.2 | 0.00801 |
| GO:0042129 | regulation of T cell proliferation | 0.149351 | 0.00794 |
| GO:0090092 | regulation of transmembrane receptor protein serine/threonine kinase signaling pathway | 0.139423 | 0.00793 |
| GO:0009743 | response to carbohydrate | 0.146199 | 0.00792 |
| GO:0120032 | regulation of plasma membrane bounded cell projection assembly | 0.142857 | 0.00791 |
| GO:0010632 | regulation of epithelial cell migration | 0.142857 | 0.00791 |
| GO:0031526 | brush border membrane | 0.183099 | 0.00786 |
| GO:0043209 | myelin sheath | 0.126471 | 0.00779 |
| GO:0010594 | regulation of endothelial cell migration | 0.153285 | 0.00778 |
| GO:0060828 | regulation of canonical Wnt signaling pathway | 0.140097 | 0.00776 |
| GO:0016922 | nuclear receptor binding | 0.150327 | 0.00773 |
| GO:0051101 | regulation of DNA binding | 0.161905 | 0.00772 |
| GO:1901363 | heterocyclic compound binding | 0.090169 | 0.00767 |
| GO:0009611 | response to wounding | 0.140777 | 0.00762 |
| GO:0000502 | proteasome complex | 0.175 | 0.00752 |
| GO:0004527 | exonuclease activity | 0.175 | 0.00752 |
| GO:0106027 | neuron projection organization | 0.228571 | 0.00751 |
| GO:0021510 | spinal cord development | 0.228571 | 0.00751 |
| GO:0072175 | epithelial tube formation | 0.228571 | 0.00751 |
| GO:0099173 | postsynapse organization | 0.228571 | 0.00751 |
| GO:0046627 | negative regulation of insulin receptor signaling pathway | 0.228571 | 0.00751 |
| GO:0046839 | phospholipid dephosphorylation | 0.228571 | 0.00751 |
| GO:0099522 | cytosolic region | 0.228571 | 0.00751 |
| GO:0005929 | cilium | 0.122892 | 0.00741 |
| GO:0030177 | positive regulation of Wnt signaling pathway | 0.158333 | 0.00738 |
| GO:0010811 | positive regulation of cell-substrate adhesion | 0.158333 | 0.00738 |
| GO:0050810 | regulation of steroid biosynthetic process | 0.185714 | 0.00735 |
| GO:1905953 | negative regulation of lipid localization | 0.192982 | 0.00733 |
| GO:0050994 | regulation of lipid catabolic process | 0.192982 | 0.00733 |
| GO:0006687 | glycosphingolipid metabolic process | 0.214286 | 0.00733 |
| GO:1905167 | positive regulation of lysosomal protein catabolic process | 0.214286 | 0.00733 |
| GO:0006195 | purine nucleotide catabolic process | 0.214286 | 0.00733 |
| GO:0009165 | nucleotide biosynthetic process | 0.125348 | 0.00730 |
| GO:0002861 | regulation of inflammatory response to antigenic stimulus | 0.25 | 0.00729 |
| GO:0097061 | dendritic spine organization | 0.25 | 0.00729 |
| GO:0006900 | vesicle budding from membrane | 0.25 | 0.00729 |
| GO:0051968 | positive regulation of synaptic transmission, glutamatergic | 0.25 | 0.00729 |
| GO:0050996 | positive regulation of lipid catabolic process | 0.25 | 0.00729 |
| GO:1901020 | negative regulation of calcium ion transmembrane transporter activity | 0.25 | 0.00729 |
| GO:0018995 | host cellular component | 0.25 | 0.00729 |
| GO:0043280 | positive regulation of cysteine-type endopeptidase activity involved in apoptotic process | 0.155556 | 0.00728 |
| GO:0099175 | regulation of postsynapse organization | 0.172043 | 0.00726 |
| GO:1901653 | cellular response to peptide | 0.135802 | 0.00719 |
| GO:0034599 | cellular response to oxidative stress | 0.142857 | 0.00715 |
| GO:0072503 | cellular divalent inorganic cation homeostasis | 0.14 | 0.00711 |
| GO:0010751 | negative regulation of nitric oxide mediated signal transduction | 1 | 0.00709 |
| GO:0010750 | positive regulation of nitric oxide mediated signal transduction | 1 | 0.00709 |
| GO:0070961 | positive regulation of neutrophil mediated killing of symbiont cell | 1 | 0.00709 |
| GO:0033127 | obsolete regulation of histone phosphorylation | 1 | 0.00709 |
| GO:0099548 | trans-synaptic signaling by nitric oxide | 1 | 0.00709 |
| GO:0099543 | trans-synaptic signaling by soluble gas | 1 | 0.00709 |
| GO:1901074 | regulation of engulfment of apoptotic cell | 1 | 0.00709 |
| GO:1903244 | positive regulation of cardiac muscle hypertrophy in response to stress | 1 | 0.00709 |
| GO:0018003 | peptidyl-lysine N6-acetylation | 1 | 0.00709 |
| GO:0010615 | positive regulation of cardiac muscle adaptation | 1 | 0.00709 |
| GO:0097026 | dendritic cell dendrite assembly | 1 | 0.00709 |
| GO:0035759 | mesangial cell-matrix adhesion | 1 | 0.00709 |
| GO:0098582 | innate vocalization behavior | 1 | 0.00709 |
| GO:0003335 | corneocyte development | 1 | 0.00709 |
| GO:0070953 | regulation of neutrophil mediated killing of fungus | 1 | 0.00709 |
| GO:1990927 | calcium ion regulated lysosome exocytosis | 1 | 0.00709 |
| GO:0032972 | regulation of muscle filament sliding speed | 1 | 0.00709 |
| GO:2000790 | regulation of mesenchymal cell proliferation involved in lung development | 1 | 0.00709 |
| GO:2000791 | negative regulation of mesenchymal cell proliferation involved in lung development | 1 | 0.00709 |
| GO:0019417 | sulfur oxidation | 1 | 0.00709 |
| GO:1901727 | positive regulation of histone deacetylase activity | 1 | 0.00709 |
| GO:0099554 | trans-synaptic signaling by soluble gas, modulating synaptic transmission | 1 | 0.00709 |
| GO:0099555 | trans-synaptic signaling by nitric oxide, modulating synaptic transmission | 1 | 0.00709 |
| GO:0008204 | ergosterol metabolic process | 1 | 0.00709 |
| GO:0060177 | regulation of angiotensin metabolic process | 1 | 0.00709 |
| GO:0048294 | negative regulation of isotype switching to IgE isotypes | 1 | 0.00709 |
| GO:0032290 | peripheral nervous system myelin formation | 1 | 0.00709 |
| GO:0006696 | ergosterol biosynthetic process | 1 | 0.00709 |
| GO:1900223 | positive regulation of amyloid-beta clearance | 1 | 0.00709 |
| GO:0006478 | peptidyl-tyrosine sulfation | 1 | 0.00709 |
| GO:0070960 | positive regulation of neutrophil mediated cytotoxicity | 1 | 0.00709 |
| GO:0070965 | positive regulation of neutrophil mediated killing of fungus | 1 | 0.00709 |
| GO:0016340 | calcium-dependent cell-matrix adhesion | 1 | 0.00709 |
| GO:0051599 | response to hydrostatic pressure | 1 | 0.00709 |
| GO:0015920 | lipopolysaccharide transport | 1 | 0.00709 |
| GO:1905203 | regulation of connective tissue replacement | 1 | 0.00709 |
| GO:1905205 | positive regulation of connective tissue replacement | 1 | 0.00709 |
| GO:0099163 | synaptic signaling by nitric oxide | 1 | 0.00709 |
| GO:0016222 | procollagen-proline 4-dioxygenase complex | 1 | 0.00709 |
| GO:0008074 | guanylate cyclase complex, soluble | 1 | 0.00709 |
| GO:0031251 | PAN complex | 1 | 0.00709 |
| GO:0033557 | Slx1-Slx4 complex | 1 | 0.00709 |
| GO:0072492 | host cell mitochondrial intermembrane space | 1 | 0.00709 |
| GO:0098999 | extrinsic component of postsynaptic endosome membrane | 1 | 0.00709 |
| GO:0031933 | obsolete telomeric heterochromatin | 1 | 0.00709 |
| GO:0005918 | septate junction | 1 | 0.00709 |
| GO:0004441 | inositol-1,4-bisphosphate 1-phosphatase activity | 1 | 0.00709 |
| GO:0008503 | benzodiazepine receptor activity | 1 | 0.00709 |
| GO:0050254 | rhodopsin kinase activity | 1 | 0.00709 |
| GO:0005497 | androgen binding | 1 | 0.00709 |
| GO:0004335 | galactokinase activity | 1 | 0.00709 |
| GO:0004103 | choline kinase activity | 1 | 0.00709 |
| GO:0032397 | activating MHC class I receptor activity | 1 | 0.00709 |
| GO:0008476 | protein-tyrosine sulfotransferase activity | 1 | 0.00709 |
| GO:0016213 | linoleoyl-CoA desaturase activity | 1 | 0.00709 |
| GO:0052596 | phenethylamine:oxygen oxidoreductase (deaminating) activity | 1 | 0.00709 |
| GO:0051903 | S-(hydroxymethyl)glutathione dehydrogenase activity | 1 | 0.00709 |
| GO:0019135 | deoxyhypusine monooxygenase activity | 1 | 0.00709 |
| GO:0052593 | tryptamine:oxygen oxidoreductase (deaminating) activity | 1 | 0.00709 |
| GO:0052595 | aliphatic amine oxidase activity | 1 | 0.00709 |
| GO:0052594 | aminoacetone:oxygen oxidoreductase(deaminating) activity | 1 | 0.00709 |
| GO:0031056 | regulation of histone modification | 0.146341 | 0.00706 |
| GO:0006534 | cysteine metabolic process | 0.4 | 0.00697 |
| GO:0003094 | glomerular filtration | 0.4 | 0.00697 |
| GO:1902065 | response to L-glutamate | 0.4 | 0.00697 |
| GO:0003205 | cardiac chamber development | 0.4 | 0.00697 |
| GO:0006616 | SRP-dependent cotranslational protein targeting to membrane, translocation | 0.4 | 0.00697 |
| GO:0032429 | regulation of phospholipase A2 activity | 0.4 | 0.00697 |
| GO:2000671 | regulation of motor neuron apoptotic process | 0.4 | 0.00697 |
| GO:1904491 | protein localization to ciliary transition zone | 0.4 | 0.00697 |
| GO:0070316 | regulation of G0 to G1 transition | 0.4 | 0.00697 |
| GO:0022401 | negative adaptation of signaling pathway | 0.4 | 0.00697 |
| GO:0031630 | regulation of synaptic vesicle fusion to presynaptic active zone membrane | 0.4 | 0.00697 |
| GO:0002029 | desensitization of G protein-coupled receptor signaling pathway | 0.4 | 0.00697 |
| GO:1901632 | regulation of synaptic vesicle membrane organization | 0.4 | 0.00697 |
| GO:0051418 | microtubule nucleation by microtubule organizing center | 0.4 | 0.00697 |
| GO:0000796 | condensin complex | 0.4 | 0.00697 |
| GO:0120103 | centriolar subdistal appendage | 0.4 | 0.00697 |
| GO:0097539 | ciliary transition fiber | 0.4 | 0.00697 |
| GO:0044327 | dendritic spine head | 0.4 | 0.00697 |
| GO:0050662 | obsolete coenzyme binding | 0.4 | 0.00697 |
| GO:0070567 | cytidylyltransferase activity | 0.4 | 0.00697 |
| GO:0008430 | selenium binding | 0.4 | 0.00697 |
| GO:0047086 | ketosteroid monooxygenase activity | 0.4 | 0.00697 |
| GO:0016889 | endodeoxyribonuclease activity, producing 3'-phosphomonoesters | 0.4 | 0.00697 |
| GO:0048256 | flap endonuclease activity | 0.4 | 0.00697 |
| GO:0015078 | proton transmembrane transporter activity | 0.133588 | 0.00696 |
| GO:1901292 | nucleoside phosphate catabolic process | 0.177215 | 0.00689 |
| GO:0006633 | fatty acid biosynthetic process | 0.161017 | 0.00674 |
| GO:0050729 | positive regulation of inflammatory response | 0.165049 | 0.00671 |
| GO:0050921 | positive regulation of chemotaxis | 0.155039 | 0.00667 |
| GO:0032355 | response to estradiol | 0.155039 | 0.00667 |
| GO:0032259 | methylation | 0.129568 | 0.00664 |
| GO:0051099 | positive regulation of binding | 0.141414 | 0.00660 |
| GO:0010008 | endosome membrane | 0.125 | 0.00660 |
| GO:0046164 | alcohol catabolic process | 0.191176 | 0.00656 |
| GO:0035869 | ciliary transition zone | 0.191176 | 0.00656 |
| GO:0007015 | actin filament organization | 0.13 | 0.00646 |
| GO:0009266 | response to temperature stimulus | 0.142132 | 0.00639 |
| GO:2000311 | regulation of AMPA receptor activity | 0.285714 | 0.00638 |
| GO:1904263 | positive regulation of TORC1 signaling | 0.285714 | 0.00638 |
| GO:0018279 | protein N-linked glycosylation via asparagine | 0.285714 | 0.00638 |
| GO:1903523 | negative regulation of blood circulation | 0.285714 | 0.00638 |
| GO:0071577 | zinc ion transmembrane transport | 0.285714 | 0.00638 |
| GO:0034367 | protein-containing complex remodeling | 0.285714 | 0.00638 |
| GO:0050926 | regulation of positive chemotaxis | 0.285714 | 0.00638 |
| GO:0036315 | cellular response to sterol | 0.285714 | 0.00638 |
| GO:0001833 | inner cell mass cell proliferation | 0.285714 | 0.00638 |
| GO:0046514 | ceramide catabolic process | 0.285714 | 0.00638 |
| GO:0045822 | negative regulation of heart contraction | 0.285714 | 0.00638 |
| GO:0004707 | MAP kinase activity | 0.285714 | 0.00638 |
| GO:0070577 | lysine-acetylated histone binding | 0.285714 | 0.00638 |
| GO:0140033 | acetylation-dependent protein binding | 0.285714 | 0.00638 |
| GO:0017111 | ribonucleoside triphosphate phosphatase activity | 0.111515 | 0.00637 |
| GO:0002449 | lymphocyte mediated immunity | 0.179487 | 0.00632 |
| GO:0090288 | negative regulation of cellular response to growth factor stimulus | 0.172414 | 0.00632 |
| GO:0034979 | NAD-dependent protein deacetylase activity | 0.172414 | 0.00632 |
| GO:0008013 | beta-catenin binding | 0.172414 | 0.00632 |
| GO:0016607 | nuclear speck | 0.119141 | 0.00632 |
| GO:0043281 | regulation of cysteine-type endopeptidase activity involved in apoptotic process | 0.139535 | 0.00629 |
| GO:0000978 | RNA polymerase II cis-regulatory region sequence-specific DNA binding | 0.112752 | 0.00629 |
| GO:0051952 | regulation of amine transport | 0.166667 | 0.00628 |
| GO:0120034 | positive regulation of plasma membrane bounded cell projection assembly | 0.166667 | 0.00628 |
| GO:1903844 | regulation of cellular response to transforming growth factor beta stimulus | 0.166667 | 0.00628 |
| GO:1901135 | carbohydrate derivative metabolic process | 0.105224 | 0.00628 |
| GO:0018023 | peptidyl-lysine trimethylation | 0.235294 | 0.00625 |
| GO:1903115 | regulation of actin filament-based movement | 0.235294 | 0.00625 |
| GO:0030134 | COPII-coated ER to Golgi transport vesicle | 0.235294 | 0.00625 |
| GO:0004032 | alditol:NADP+ 1-oxidoreductase activity | 0.235294 | 0.00625 |
| GO:0090207 | regulation of triglyceride metabolic process | 0.219512 | 0.00621 |
| GO:0031648 | protein destabilization | 0.219512 | 0.00621 |
| GO:0030282 | bone mineralization | 0.219512 | 0.00621 |
| GO:0014059 | regulation of dopamine secretion | 0.219512 | 0.00621 |
| GO:0051452 | intracellular pH reduction | 0.219512 | 0.00621 |
| GO:0005763 | mitochondrial small ribosomal subunit | 0.219512 | 0.00621 |
| GO:0000314 | organellar small ribosomal subunit | 0.219512 | 0.00621 |
| GO:0008392 | arachidonic acid epoxygenase activity | 0.219512 | 0.00621 |
| GO:0010810 | regulation of cell-substrate adhesion | 0.142857 | 0.00620 |
| GO:0002862 | negative regulation of inflammatory response to antigenic stimulus | 0.333333 | 0.00619 |
| GO:0099637 | neurotransmitter receptor transport | 0.333333 | 0.00619 |
| GO:0009812 | flavonoid metabolic process | 0.333333 | 0.00619 |
| GO:0099084 | postsynaptic specialization organization | 0.333333 | 0.00619 |
| GO:0006878 | cellular copper ion homeostasis | 0.333333 | 0.00619 |
| GO:1901841 | regulation of high voltage-gated calcium channel activity | 0.333333 | 0.00619 |
| GO:0072562 | blood microparticle | 0.333333 | 0.00619 |
| GO:0005452 | solute:inorganic anion antiporter activity | 0.333333 | 0.00619 |
| GO:0003756 | protein disulfide isomerase activity | 0.333333 | 0.00619 |
| GO:0016864 | intramolecular oxidoreductase activity, transposing S-S bonds | 0.333333 | 0.00619 |
| GO:0046390 | ribose phosphate biosynthetic process | 0.130872 | 0.00617 |
| GO:0072522 | purine-containing compound biosynthetic process | 0.130872 | 0.00617 |
| GO:0016301 | kinase activity | 0.107401 | 0.00612 |
| GO:0007507 | heart development | 0.137339 | 0.00611 |
| GO:1903532 | positive regulation of secretion by cell | 0.128931 | 0.00604 |
| GO:0034765 | regulation of ion transmembrane transport | 0.122494 | 0.00603 |
| GO:0032271 | regulation of protein polymerization | 0.140845 | 0.00596 |
| GO:1903050 | regulation of proteolysis involved in protein catabolic process | 0.137931 | 0.00595 |
| GO:0033500 | carbohydrate homeostasis | 0.137931 | 0.00595 |
| GO:0032731 | positive regulation of interleukin-1 beta production | 0.208333 | 0.00594 |
| GO:0006835 | dicarboxylic acid transport | 0.208333 | 0.00594 |
| GO:0031985 | Golgi cisterna | 0.208333 | 0.00594 |
| GO:0140297 | DNA-binding transcription factor binding | 0.120507 | 0.00594 |
| GO:0043279 | response to alkaloid | 0.168317 | 0.00590 |
| GO:0016242 | negative regulation of macroautophagy | 0.259259 | 0.00588 |
| GO:0034453 | microtubule anchoring | 0.259259 | 0.00588 |
| GO:0016540 | protein autoprocessing | 0.259259 | 0.00588 |
| GO:0048732 | gland development | 0.132203 | 0.00585 |
| GO:0045211 | postsynaptic membrane | 0.147727 | 0.00584 |
| GO:0042593 | glucose homeostasis | 0.138528 | 0.00581 |
| GO:0005623 | obsolete cell | 0.174419 | 0.00575 |
| GO:0008016 | regulation of heart contraction | 0.151899 | 0.00564 |
| GO:0032680 | regulation of tumor necrosis factor production | 0.15493 | 0.00562 |
| GO:0046578 | regulation of Ras protein signal transduction | 0.136546 | 0.00559 |
| GO:0021782 | glial cell development | 0.2 | 0.00555 |
| GO:0008284 | positive regulation of cell population proliferation | 0.108133 | 0.00551 |
| GO:0006820 | anion transport | 0.127424 | 0.00549 |
| GO:0070507 | regulation of microtubule cytoskeleton organization | 0.147059 | 0.00545 |
| GO:0002700 | regulation of production of molecular mediator of immune response | 0.156028 | 0.00541 |
| GO:0031668 | cellular response to extracellular stimulus | 0.137652 | 0.00538 |
| GO:0017015 | regulation of transforming growth factor beta receptor signaling pathway | 0.171717 | 0.00529 |
| GO:0071417 | cellular response to organonitrogen compound | 0.118397 | 0.00527 |
| GO:0009408 | response to heat | 0.16 | 0.00527 |
| GO:0051918 | negative regulation of fibrinolysis | 0.6 | 0.00524 |
| GO:1903026 | negative regulation of RNA polymerase II regulatory region sequence-specific DNA binding | 0.6 | 0.00524 |
| GO:0072378 | blood coagulation, fibrin clot formation | 0.6 | 0.00524 |
| GO:0070948 | regulation of neutrophil mediated cytotoxicity | 0.6 | 0.00524 |
| GO:0070813 | hydrogen sulfide metabolic process | 0.6 | 0.00524 |
| GO:0060510 | type II pneumocyte differentiation | 0.6 | 0.00524 |
| GO:1902268 | negative regulation of polyamine transmembrane transport | 0.6 | 0.00524 |
| GO:0060136 | embryonic process involved in female pregnancy | 0.6 | 0.00524 |
| GO:0007253 | cytoplasmic sequestering of NF-kappaB | 0.6 | 0.00524 |
| GO:0051152 | positive regulation of smooth muscle cell differentiation | 0.6 | 0.00524 |
| GO:0051343 | positive regulation of cyclic-nucleotide phosphodiesterase activity | 0.6 | 0.00524 |
| GO:0010815 | bradykinin catabolic process | 0.6 | 0.00524 |
| GO:0042126 | nitrate metabolic process | 0.6 | 0.00524 |
| GO:0042584 | chromaffin granule membrane | 0.6 | 0.00524 |
| GO:0032983 | kainate selective glutamate receptor complex | 0.6 | 0.00524 |
| GO:0044326 | dendritic spine neck | 0.6 | 0.00524 |
| GO:0004579 | dolichyl-diphosphooligosaccharide-protein glycotransferase activity | 0.6 | 0.00524 |
| GO:0004576 | oligosaccharyl transferase activity | 0.6 | 0.00524 |
| GO:0005534 | galactose binding | 0.6 | 0.00524 |
| GO:0015914 | phospholipid transport | 0.176471 | 0.00524 |
| GO:1904036 | negative regulation of epithelial cell apoptotic process | 0.225 | 0.00523 |
| GO:0002823 | negative regulation of adaptive immune response based on somatic recombination of immune receptors built from immunoglobulin superfamily domains | 0.225 | 0.00523 |
| GO:0009067 | aspartate family amino acid biosynthetic process | 0.225 | 0.00523 |
| GO:0006778 | porphyrin-containing compound metabolic process | 0.225 | 0.00523 |
| GO:0051281 | positive regulation of release of sequestered calcium ion into cytosol | 0.225 | 0.00523 |
| GO:0003924 | GTPase activity | 0.122004 | 0.00515 |
| GO:0010559 | regulation of glycoprotein biosynthetic process | 0.242424 | 0.00515 |
| GO:0048488 | synaptic vesicle endocytosis | 0.242424 | 0.00515 |
| GO:0140238 | presynaptic endocytosis | 0.242424 | 0.00515 |
| GO:0055075 | potassium ion homeostasis | 0.242424 | 0.00515 |
| GO:0015248 | sterol transporter activity | 0.242424 | 0.00515 |
| GO:0032944 | regulation of mononuclear cell proliferation | 0.142157 | 0.00511 |
| GO:1901606 | alpha-amino acid catabolic process | 0.158273 | 0.00510 |
| GO:0071398 | cellular response to fatty acid | 0.212766 | 0.00508 |
| GO:1904350 | regulation of protein catabolic process in the vacuole | 0.212766 | 0.00508 |
| GO:0010639 | negative regulation of organelle organization | 0.126649 | 0.00506 |
| GO:0001539 | cilium or flagellum-dependent cell motility | 0.173469 | 0.00505 |
| GO:0060285 | cilium-dependent cell motility | 0.173469 | 0.00505 |
| GO:0019218 | regulation of steroid metabolic process | 0.173469 | 0.00505 |
| GO:0043462 | regulation of ATP-dependent activity | 0.186667 | 0.00502 |
| GO:0032580 | Golgi cisterna membrane | 0.186667 | 0.00502 |
| GO:0043547 | positive regulation of GTPase activity | 0.133803 | 0.00496 |
| GO:1902105 | regulation of leukocyte differentiation | 0.133803 | 0.00496 |
| GO:0007188 | adenylate cyclase-modulating G protein-coupled receptor signaling pathway | 0.142857 | 0.00492 |
| GO:0050927 | positive regulation of positive chemotaxis | 0.3 | 0.00490 |
| GO:0051220 | cytoplasmic sequestering of protein | 0.3 | 0.00490 |
| GO:1902003 | regulation of amyloid-beta formation | 0.3 | 0.00490 |
| GO:0015645 | fatty acid ligase activity | 0.3 | 0.00490 |
| GO:0015125 | bile acid transmembrane transporter activity | 0.3 | 0.00490 |
| GO:0032231 | regulation of actin filament bundle assembly | 0.175258 | 0.00486 |
| GO:0016604 | nuclear body | 0.109453 | 0.00481 |
| GO:0007632 | visual behavior | 0.203704 | 0.00480 |
| GO:0034109 | homotypic cell-cell adhesion | 0.203704 | 0.00480 |
| GO:0034763 | negative regulation of transmembrane transport | 0.162602 | 0.00479 |
| GO:0007167 | enzyme-linked receptor protein signaling pathway | 0.121339 | 0.00479 |
| GO:0040014 | regulation of multicellular organism growth | 0.178571 | 0.00479 |
| GO:0048525 | negative regulation of viral process | 0.166667 | 0.00474 |
| GO:0062013 | positive regulation of small molecule metabolic process | 0.149701 | 0.00473 |
| GO:0072659 | protein localization to plasma membrane | 0.146739 | 0.00471 |
| GO:0030148 | sphingolipid biosynthetic process | 0.189189 | 0.00471 |
| GO:0006140 | regulation of nucleotide metabolic process | 0.189189 | 0.00471 |
| GO:0017022 | myosin binding | 0.189189 | 0.00471 |
| GO:0048701 | embryonic cranial skeleton morphogenesis | 0.269231 | 0.00470 |
| GO:0006743 | ubiquinone metabolic process | 0.269231 | 0.00470 |
| GO:0060765 | regulation of androgen receptor signaling pathway | 0.269231 | 0.00470 |
| GO:0006779 | porphyrin-containing compound biosynthetic process | 0.269231 | 0.00470 |
| GO:0048731 | system development | 0.119626 | 0.00466 |
| GO:0002684 | positive regulation of immune system process | 0.109658 | 0.00460 |
| GO:0016614 | oxidoreductase activity, acting on CH-OH group of donors | 0.13289 | 0.00460 |
| GO:0008168 | methyltransferase activity | 0.13289 | 0.00460 |
| GO:0051147 | regulation of muscle cell differentiation | 0.163934 | 0.00460 |
| GO:0002761 | regulation of myeloid leukocyte differentiation | 0.163934 | 0.00460 |
| GO:0043204 | perikaryon | 0.150602 | 0.00454 |
| GO:0051788 | response to misfolded protein | 0.444444 | 0.00448 |
| GO:1903242 | regulation of cardiac muscle hypertrophy in response to stress | 0.444444 | 0.00448 |
| GO:0038202 | TORC1 signaling | 0.444444 | 0.00448 |
| GO:0002407 | dendritic cell chemotaxis | 0.444444 | 0.00448 |
| GO:0061140 | lung secretory cell differentiation | 0.444444 | 0.00448 |
| GO:0032817 | regulation of natural killer cell proliferation | 0.444444 | 0.00448 |
| GO:0060445 | branching involved in salivary gland morphogenesis | 0.444444 | 0.00448 |
| GO:1903659 | regulation of complement-dependent cytotoxicity | 0.444444 | 0.00448 |
| GO:1990454 | L-type voltage-gated calcium channel complex | 0.444444 | 0.00448 |
| GO:0099025 | anchored component of postsynaptic membrane | 0.444444 | 0.00448 |
| GO:0001401 | SAM complex | 0.444444 | 0.00448 |
| GO:0046030 | inositol trisphosphate phosphatase activity | 0.444444 | 0.00448 |
| GO:0019238 | cyclohydrolase activity | 0.444444 | 0.00448 |
| GO:0015379 | potassium:chloride symporter activity | 0.444444 | 0.00448 |
| GO:0019887 | protein kinase regulator activity | 0.136719 | 0.00447 |
| GO:0032543 | mitochondrial translation | 0.191781 | 0.00445 |
| GO:0051928 | positive regulation of calcium ion transport | 0.165289 | 0.00444 |
| GO:0005262 | calcium channel activity | 0.165289 | 0.00444 |
| GO:0006400 | tRNA modification | 0.173913 | 0.00444 |
| GO:0044321 | response to leptin | 0.357143 | 0.00443 |
| GO:0030948 | negative regulation of vascular endothelial growth factor receptor signaling pathway | 0.357143 | 0.00443 |
| GO:0003414 | chondrocyte morphogenesis involved in endochondral bone morphogenesis | 0.357143 | 0.00443 |
| GO:2001028 | positive regulation of endothelial cell chemotaxis | 0.357143 | 0.00443 |
| GO:0046479 | glycosphingolipid catabolic process | 0.357143 | 0.00443 |
| GO:0003422 | growth plate cartilage morphogenesis | 0.357143 | 0.00443 |
| GO:1905153 | regulation of membrane invagination | 0.357143 | 0.00443 |
| GO:0072393 | microtubule anchoring at microtubule organizing center | 0.357143 | 0.00443 |
| GO:0003429 | growth plate cartilage chondrocyte morphogenesis | 0.357143 | 0.00443 |
| GO:0090171 | chondrocyte morphogenesis | 0.357143 | 0.00443 |
| GO:0042910 | xenobiotic transmembrane transporter activity | 0.357143 | 0.00443 |
| GO:0031099 | regeneration | 0.148352 | 0.00442 |
| GO:0002573 | myeloid leukocyte differentiation | 0.180723 | 0.00438 |
| GO:0002456 | T cell mediated immunity | 0.230769 | 0.00438 |
| GO:1903170 | negative regulation of calcium ion transmembrane transport | 0.230769 | 0.00438 |
| GO:0006636 | unsaturated fatty acid biosynthetic process | 0.230769 | 0.00438 |
| GO:0000271 | polysaccharide biosynthetic process | 0.230769 | 0.00438 |
| GO:0055081 | anion homeostasis | 0.230769 | 0.00438 |
| GO:0031669 | cellular response to nutrient levels | 0.142857 | 0.00437 |
| GO:1901998 | toxin transport | 0.217391 | 0.00432 |
| GO:1905165 | regulation of lysosomal protein catabolic process | 0.217391 | 0.00432 |
| GO:0051602 | response to electrical stimulus | 0.217391 | 0.00432 |
| GO:0045428 | regulation of nitric oxide biosynthetic process | 0.217391 | 0.00432 |
| GO:0016811 | hydrolase activity, acting on carbon-nitrogen (but not peptide) bonds, in linear amides | 0.149171 | 0.00430 |
| GO:0016462 | pyrophosphatase activity | 0.111235 | 0.00430 |
| GO:0005667 | transcription regulator complex | 0.121212 | 0.00429 |
| GO:0009152 | purine ribonucleotide biosynthetic process | 0.135036 | 0.00429 |
| GO:0051235 | maintenance of location | 0.152439 | 0.00423 |
| GO:2000677 | regulation of transcription regulatory region DNA binding | 0.25 | 0.00421 |
| GO:0008200 | ion channel inhibitor activity | 0.25 | 0.00421 |
| GO:0042169 | SH2 domain binding | 0.25 | 0.00421 |
| GO:0060999 | positive regulation of dendritic spine development | 0.207547 | 0.00413 |
| GO:0061512 | protein localization to cilium | 0.207547 | 0.00413 |
| GO:0030315 | T-tubule | 0.207547 | 0.00413 |
| GO:0016798 | hydrolase activity, acting on glycosyl bonds | 0.156463 | 0.00405 |
| GO:0016627 | oxidoreductase activity, acting on the CH-CH group of donors | 0.182927 | 0.00402 |
| GO:0033293 | monocarboxylic acid binding | 0.182927 | 0.00402 |
| GO:0042379 | chemokine receptor binding | 0.182927 | 0.00402 |
| GO:0009260 | ribonucleotide biosynthetic process | 0.134021 | 0.00399 |
| GO:0031253 | cell projection membrane | 0.130952 | 0.00398 |
| GO:2000377 | regulation of reactive oxygen species metabolic process | 0.145833 | 0.00393 |
| GO:0008277 | regulation of G protein-coupled receptor signaling pathway | 0.171429 | 0.00390 |
| GO:1903052 | positive regulation of proteolysis involved in protein catabolic process | 0.165217 | 0.00389 |
| GO:0035296 | regulation of tube diameter | 0.165217 | 0.00389 |
| GO:0001816 | cytokine production | 0.165217 | 0.00389 |
| GO:0097746 | blood vessel diameter maintenance | 0.165217 | 0.00389 |
| GO:1903555 | regulation of tumor necrosis factor superfamily cytokine production | 0.158621 | 0.00377 |
| GO:0060491 | regulation of cell projection assembly | 0.146597 | 0.00376 |
| GO:0043484 | regulation of RNA splicing | 0.146597 | 0.00376 |
| GO:0032386 | regulation of intracellular transport | 0.130699 | 0.00375 |
| GO:0030449 | regulation of complement activation | 0.28 | 0.00370 |
| GO:0002097 | tRNA wobble base modification | 0.28 | 0.00370 |
| GO:1903792 | negative regulation of anion transport | 0.28 | 0.00370 |
| GO:0099524 | postsynaptic cytosol | 0.28 | 0.00370 |
| GO:0070006 | metalloaminopeptidase activity | 0.28 | 0.00370 |
| GO:0051056 | regulation of small GTPase mediated signal transduction | 0.135889 | 0.00369 |
| GO:0040015 | negative regulation of multicellular organism growth | 0.315789 | 0.00369 |
| GO:0034368 | protein-lipid complex remodeling | 0.315789 | 0.00369 |
| GO:0034369 | plasma lipoprotein particle remodeling | 0.315789 | 0.00369 |
| GO:0046885 | regulation of hormone biosynthetic process | 0.315789 | 0.00369 |
| GO:0071625 | vocalization behavior | 0.315789 | 0.00369 |
| GO:0051787 | misfolded protein binding | 0.315789 | 0.00369 |
| GO:1905369 | endopeptidase complex | 0.177778 | 0.00366 |
| GO:0031641 | regulation of myelination | 0.222222 | 0.00365 |
| GO:0035094 | response to nicotine | 0.222222 | 0.00365 |
| GO:0030120 | vesicle coat | 0.222222 | 0.00365 |
| GO:0008391 | arachidonic acid monooxygenase activity | 0.222222 | 0.00365 |
| GO:0002697 | regulation of immune effector process | 0.128 | 0.00364 |
| GO:1900077 | negative regulation of cellular response to insulin stimulus | 0.236842 | 0.00363 |
| GO:0090303 | positive regulation of wound healing | 0.236842 | 0.00363 |
| GO:0033692 | cellular polysaccharide biosynthetic process | 0.236842 | 0.00363 |
| GO:0050997 | quaternary ammonium group binding | 0.236842 | 0.00363 |
| GO:0043565 | sequence-specific DNA binding | 0.105229 | 0.00362 |
| GO:0031345 | negative regulation of cell projection organization | 0.150289 | 0.00362 |
| GO:0007009 | plasma membrane organization | 0.166667 | 0.00360 |
| GO:0005604 | basement membrane | 0.166667 | 0.00360 |
| GO:2000116 | regulation of cysteine-type endopeptidase activity | 0.139344 | 0.00360 |
| GO:0016818 | hydrolase activity, acting on acid anhydrides, in phosphorus-containing anhydrides | 0.111849 | 0.00360 |
| GO:0016817 | hydrolase activity, acting on acid anhydrides | 0.111849 | 0.00360 |
| GO:0043254 | regulation of protein-containing complex assembly | 0.125296 | 0.00358 |
| GO:0097553 | calcium ion transmembrane import into cytosol | 0.19403 | 0.00355 |
| GO:0060191 | regulation of lipase activity | 0.19403 | 0.00355 |
| GO:0031304 | intrinsic component of mitochondrial inner membrane | 0.211538 | 0.00353 |
| GO:1901800 | positive regulation of proteasomal protein catabolic process | 0.174757 | 0.00352 |
| GO:0005793 | endoplasmic reticulum-Golgi intermediate compartment | 0.151163 | 0.00346 |
| GO:0043062 | extracellular structure organization | 0.142857 | 0.00344 |
| GO:0060090 | molecular adaptor activity | 0.130058 | 0.00343 |
| GO:2001057 | reactive nitrogen species metabolic process | 0.258065 | 0.00340 |
| GO:0030041 | actin filament polymerization | 0.258065 | 0.00340 |
| GO:0036314 | response to sterol | 0.258065 | 0.00340 |
| GO:1903715 | regulation of aerobic respiration | 0.258065 | 0.00340 |
| GO:0032496 | response to lipopolysaccharide | 0.126492 | 0.00335 |
| GO:0045229 | external encapsulating structure organization | 0.143498 | 0.00335 |
| GO:0042130 | negative regulation of T cell proliferation | 0.20339 | 0.00334 |
| GO:0051057 | positive regulation of small GTPase mediated signal transduction | 0.20339 | 0.00334 |
| GO:0016780 | phosphotransferase activity, for other substituted phosphate groups | 0.20339 | 0.00334 |
| GO:0003954 | NADH dehydrogenase activity | 0.20339 | 0.00334 |
| GO:0046888 | negative regulation of hormone secretion | 0.179775 | 0.00333 |
| GO:0048638 | regulation of developmental growth | 0.130814 | 0.00327 |
| GO:0030198 | extracellular matrix organization | 0.144144 | 0.00327 |
| GO:0015085 | calcium ion transmembrane transporter activity | 0.15942 | 0.00323 |
| GO:1901293 | nucleoside phosphate biosynthetic process | 0.129121 | 0.00321 |
| GO:0019838 | growth factor binding | 0.155844 | 0.00321 |
| GO:0003677 | DNA binding | 0.098115 | 0.00318 |
| GO:0051090 | regulation of DNA-binding transcription factor activity | 0.129477 | 0.00313 |
| GO:0016747 | acyltransferase activity, transferring groups other than amino-acyl groups | 0.137184 | 0.00312 |
| GO:0045807 | positive regulation of endocytosis | 0.169643 | 0.00311 |
| GO:0051726 | regulation of cell cycle | 0.108997 | 0.00310 |
| GO:0002253 | activation of immune response | 0.126829 | 0.00307 |
| GO:0051956 | negative regulation of amino acid transport | 0.384615 | 0.00306 |
| GO:0010522 | regulation of calcium ion transport into cytosol | 0.384615 | 0.00306 |
| GO:0071941 | nitrogen cycle metabolic process | 0.384615 | 0.00306 |
| GO:0031639 | plasminogen activation | 0.384615 | 0.00306 |
| GO:0006068 | ethanol catabolic process | 0.384615 | 0.00306 |
| GO:0072546 | EMC complex | 0.384615 | 0.00306 |
| GO:0008191 | metalloendopeptidase inhibitor activity | 0.384615 | 0.00306 |
| GO:0050700 | CARD domain binding | 0.384615 | 0.00306 |
| GO:0009063 | cellular amino acid catabolic process | 0.156863 | 0.00305 |
| GO:0099003 | vesicle-mediated transport in synapse | 0.181818 | 0.00304 |
| GO:0042562 | hormone binding | 0.181818 | 0.00304 |
| GO:0071407 | cellular response to organic cyclic compound | 0.123203 | 0.00303 |
| GO:0016298 | lipase activity | 0.160584 | 0.00302 |
| GO:0048678 | response to axon injury | 0.215686 | 0.00301 |
| GO:0008542 | visual learning | 0.215686 | 0.00301 |
| GO:0031305 | integral component of mitochondrial inner membrane | 0.215686 | 0.00301 |
| GO:0002237 | response to molecule of bacterial origin | 0.126437 | 0.00301 |
| GO:0097159 | organic cyclic compound binding | 0.090763 | 0.00300 |
| GO:0042572 | retinol metabolic process | 0.243243 | 0.00299 |
| GO:0046434 | organophosphate catabolic process | 0.157895 | 0.00292 |
| GO:0090101 | negative regulation of transmembrane receptor protein serine/threonine kinase signaling pathway | 0.171171 | 0.00291 |
| GO:0010638 | positive regulation of organelle organization | 0.123967 | 0.00290 |
| GO:0002704 | negative regulation of leukocyte mediated immunity | 0.206897 | 0.00287 |
| GO:0045277 | respiratory chain complex IV | 0.206897 | 0.00287 |
| GO:0019207 | kinase regulator activity | 0.136519 | 0.00287 |
| GO:0032780 | negative regulation of ATP-dependent activity | 0.291667 | 0.00287 |
| GO:0005797 | Golgi medial cisterna | 0.291667 | 0.00287 |
| GO:0050780 | dopamine receptor binding | 0.291667 | 0.00287 |
| GO:0097190 | apoptotic signaling pathway | 0.137546 | 0.00284 |
| GO:0098685 | Schaffer collateral - CA1 synapse | 0.177083 | 0.00282 |
| GO:0043087 | regulation of GTPase activity | 0.12963 | 0.00274 |
| GO:0009072 | aromatic amino acid family metabolic process | 0.266667 | 0.00272 |
| GO:0031683 | G-protein beta/gamma-subunit complex binding | 0.266667 | 0.00272 |
| GO:1903828 | negative regulation of protein localization | 0.14554 | 0.00272 |
| GO:0001067 | transcription regulatory region nucleic acid binding | 0.108534 | 0.00272 |
| GO:0071397 | cellular response to cholesterol | 0.333333 | 0.00272 |
| GO:1903020 | positive regulation of glycoprotein metabolic process | 0.333333 | 0.00272 |
| GO:2001026 | regulation of endothelial cell chemotaxis | 0.333333 | 0.00272 |
| GO:0046068 | cGMP metabolic process | 0.333333 | 0.00272 |
| GO:0098581 | detection of external biotic stimulus | 0.333333 | 0.00272 |
| GO:0048010 | vascular endothelial growth factor receptor signaling pathway | 0.333333 | 0.00272 |
| GO:0016888 | endodeoxyribonuclease activity, producing 5'-phosphomonoesters | 0.333333 | 0.00272 |
| GO:0006355 | regulation of DNA-templated transcription | 0.096645 | 0.00270 |
| GO:0051341 | regulation of oxidoreductase activity | 0.2 | 0.00269 |
| GO:0009295 | nucleoid | 0.2 | 0.00269 |
| GO:0042645 | mitochondrial nucleoid | 0.2 | 0.00269 |
| GO:0005080 | protein kinase C binding | 0.2 | 0.00269 |
| GO:0010628 | positive regulation of gene expression | 0.107463 | 0.00268 |
| GO:0051961 | negative regulation of nervous system development | 0.162963 | 0.00268 |
| GO:0034380 | high-density lipoprotein particle assembly | 0.5 | 0.00267 |
| GO:0090281 | negative regulation of calcium ion import | 0.5 | 0.00267 |
| GO:0046654 | tetrahydrofolate biosynthetic process | 0.5 | 0.00267 |
| GO:0046618 | xenobiotic export from cell | 0.5 | 0.00267 |
| GO:0051917 | regulation of fibrinolysis | 0.5 | 0.00267 |
| GO:0019388 | galactose catabolic process | 0.5 | 0.00267 |
| GO:1905809 | negative regulation of synapse organization | 0.5 | 0.00267 |
| GO:0009635 | response to herbicide | 0.5 | 0.00267 |
| GO:1901678 | iron coordination entity transport | 0.5 | 0.00267 |
| GO:0017108 | 5'-flap endonuclease activity | 0.5 | 0.00267 |
| GO:0097199 | cysteine-type endopeptidase activity involved in apoptotic signaling pathway | 0.5 | 0.00267 |
| GO:1903706 | regulation of hemopoiesis | 0.130319 | 0.00266 |
| GO:0006164 | purine nucleotide biosynthetic process | 0.136364 | 0.00266 |
| GO:0098978 | glutamatergic synapse | 0.11782 | 0.00265 |
| GO:0015849 | organic acid transport | 0.143478 | 0.00265 |
| GO:0034097 | response to cytokine | 0.110018 | 0.00264 |
| GO:0000976 | transcription cis-regulatory region binding | 0.108985 | 0.00263 |
| GO:1904018 | positive regulation of vasculature development | 0.161074 | 0.00262 |
| GO:0034612 | response to tumor necrosis factor | 0.161074 | 0.00262 |
| GO:0045766 | positive regulation of angiogenesis | 0.161074 | 0.00262 |
| GO:0048640 | negative regulation of developmental growth | 0.174312 | 0.00258 |
| GO:0098889 | intrinsic component of presynaptic membrane | 0.174312 | 0.00258 |
| GO:0098878 | neurotransmitter receptor complex | 0.232558 | 0.00256 |
| GO:0003014 | renal system process | 0.186047 | 0.00256 |
| GO:0071277 | cellular response to calcium ion | 0.186047 | 0.00256 |
| GO:0034198 | cellular response to amino acid starvation | 0.22 | 0.00255 |
| GO:0070374 | positive regulation of ERK1 and ERK2 cascade | 0.149485 | 0.00255 |
| GO:0005887 | integral component of plasma membrane | 0.10663 | 0.00252 |
| GO:0018193 | peptidyl-amino acid modification | 0.109244 | 0.00251 |
| GO:1905897 | regulation of response to endoplasmic reticulum stress | 0.210526 | 0.00247 |
| GO:0043198 | dendritic shaft | 0.210526 | 0.00247 |
| GO:0005504 | fatty acid binding | 0.210526 | 0.00247 |
| GO:0008009 | chemokine activity | 0.210526 | 0.00247 |
| GO:0050660 | flavin adenine dinucleotide binding | 0.175926 | 0.00245 |
| GO:0045744 | negative regulation of G protein-coupled receptor signaling pathway | 0.25 | 0.00245 |
| GO:0070888 | E-box binding | 0.25 | 0.00245 |
| GO:0050768 | negative regulation of neurogenesis | 0.165414 | 0.00241 |
| GO:0030098 | lymphocyte differentiation | 0.140684 | 0.00239 |
| GO:0006310 | DNA recombination | 0.13079 | 0.00239 |
| GO:0016616 | oxidoreductase activity, acting on the CH-OH group of donors, NAD or NADP as acceptor | 0.138298 | 0.00237 |
| GO:0050767 | regulation of neurogenesis | 0.129199 | 0.00234 |
| GO:0060998 | regulation of dendritic spine development | 0.203125 | 0.00233 |
| GO:0072594 | establishment of protein localization to organelle | 0.131507 | 0.00229 |
| GO:0000977 | RNA polymerase II transcription regulatory region sequence-specific DNA binding | 0.111554 | 0.00228 |
| GO:0072014 | proximal tubule development | 0.75 | 0.00224 |
| GO:2001199 | negative regulation of dendritic cell differentiation | 0.75 | 0.00224 |
| GO:0006121 | mitochondrial electron transport, succinate to ubiquinone | 0.75 | 0.00224 |
| GO:0090032 | negative regulation of steroid hormone biosynthetic process | 0.75 | 0.00224 |
| GO:0098903 | regulation of membrane repolarization during action potential | 0.75 | 0.00224 |
| GO:1903223 | positive regulation of oxidative stress-induced neuron death | 0.75 | 0.00224 |
| GO:0019346 | transsulfuration | 0.75 | 0.00224 |
| GO:0097107 | postsynaptic density assembly | 0.75 | 0.00224 |
| GO:0048293 | regulation of isotype switching to IgE isotypes | 0.75 | 0.00224 |
| GO:0003433 | chondrocyte development involved in endochondral bone morphogenesis | 0.75 | 0.00224 |
| GO:0034105 | positive regulation of tissue remodeling | 0.75 | 0.00224 |
| GO:0070125 | mitochondrial translational elongation | 0.75 | 0.00224 |
| GO:0060018 | astrocyte fate commitment | 0.75 | 0.00224 |
| GO:0009092 | homoserine metabolic process | 0.75 | 0.00224 |
| GO:0045283 | fumarate reductase complex | 0.75 | 0.00224 |
| GO:0045281 | succinate dehydrogenase complex | 0.75 | 0.00224 |
| GO:0005749 | mitochondrial respiratory chain complex II, succinate dehydrogenase complex (ubiquinone) | 0.75 | 0.00224 |
| GO:0045257 | succinate dehydrogenase complex (ubiquinone) | 0.75 | 0.00224 |
| GO:0004792 | thiosulfate sulfurtransferase activity | 0.75 | 0.00224 |
| GO:0016416 | O-palmitoyltransferase activity | 0.75 | 0.00224 |
| GO:0035276 | ethanol binding | 0.75 | 0.00224 |
| GO:0005030 | neurotrophin receptor activity | 0.75 | 0.00224 |
| GO:0015277 | kainate selective glutamate receptor activity | 0.75 | 0.00224 |
| GO:0004095 | carnitine O-palmitoyltransferase activity | 0.75 | 0.00224 |
| GO:0019115 | benzaldehyde dehydrogenase [NAD(P)+] activity | 0.75 | 0.00224 |
| GO:0010464 | regulation of mesenchymal cell proliferation | 0.304348 | 0.00219 |
| GO:0032891 | negative regulation of organic acid transport | 0.304348 | 0.00219 |
| GO:0002082 | regulation of oxidative phosphorylation | 0.304348 | 0.00219 |
| GO:0032426 | stereocilium tip | 0.304348 | 0.00219 |
| GO:0071813 | lipoprotein particle binding | 0.304348 | 0.00219 |
| GO:0071814 | protein-lipid complex binding | 0.304348 | 0.00219 |
| GO:0005385 | zinc ion transmembrane transporter activity | 0.304348 | 0.00219 |
| GO:0010721 | negative regulation of cell development | 0.156069 | 0.00216 |
| GO:0046653 | tetrahydrofolate metabolic process | 0.275862 | 0.00215 |
| GO:0033014 | tetrapyrrole biosynthetic process | 0.275862 | 0.00215 |
| GO:1903573 | negative regulation of response to endoplasmic reticulum stress | 0.275862 | 0.00215 |
| GO:0002707 | negative regulation of lymphocyte mediated immunity | 0.22449 | 0.00215 |
| GO:0072599 | establishment of protein localization to endoplasmic reticulum | 0.22449 | 0.00215 |
| GO:1903036 | positive regulation of response to wounding | 0.22449 | 0.00215 |
| GO:0046513 | ceramide biosynthetic process | 0.22449 | 0.00215 |
| GO:0030016 | myofibril | 0.22449 | 0.00215 |
| GO:1903432 | regulation of TORC1 signaling | 0.238095 | 0.00212 |
| GO:0032720 | negative regulation of tumor necrosis factor production | 0.238095 | 0.00212 |
| GO:0008328 | ionotropic glutamate receptor complex | 0.238095 | 0.00212 |
| GO:0007420 | brain development | 0.14346 | 0.00212 |
| GO:0072523 | purine-containing compound catabolic process | 0.214286 | 0.00211 |
| GO:0035254 | glutamate receptor binding | 0.214286 | 0.00211 |
| GO:0004867 | serine-type endopeptidase inhibitor activity | 0.159236 | 0.00210 |
| GO:1903320 | regulation of protein modification by small protein conjugation or removal | 0.136986 | 0.00209 |
| GO:0035150 | regulation of tube size | 0.172414 | 0.00204 |
| GO:0090030 | regulation of steroid hormone biosynthetic process | 0.416667 | 0.00202 |
| GO:0006570 | tyrosine metabolic process | 0.416667 | 0.00202 |
| GO:0046949 | fatty-acyl-CoA biosynthetic process | 0.416667 | 0.00202 |
| GO:0009214 | cyclic nucleotide catabolic process | 0.416667 | 0.00202 |
| GO:0006182 | cGMP biosynthetic process | 0.416667 | 0.00202 |
| GO:0019203 | carbohydrate phosphatase activity | 0.416667 | 0.00202 |
| GO:0004016 | adenylate cyclase activity | 0.416667 | 0.00202 |
| GO:0033157 | regulation of intracellular protein transport | 0.146789 | 0.00202 |
| GO:0031349 | positive regulation of defense response | 0.139706 | 0.00201 |
| GO:0051055 | negative regulation of lipid biosynthetic process | 0.206349 | 0.00201 |
| GO:0016229 | steroid dehydrogenase activity | 0.206349 | 0.00201 |
| GO:0044272 | sulfur compound biosynthetic process | 0.178218 | 0.00198 |
| GO:0006760 | folic acid-containing compound metabolic process | 0.257143 | 0.00198 |
| GO:0044088 | regulation of vacuole organization | 0.257143 | 0.00198 |
| GO:0005689 | U12-type spliceosomal complex | 0.257143 | 0.00198 |
| GO:0034695 | response to prostaglandin E | 0.352941 | 0.00195 |
| GO:0035336 | long-chain fatty-acyl-CoA metabolic process | 0.352941 | 0.00195 |
| GO:0004029 | aldehyde dehydrogenase (NAD+) activity | 0.352941 | 0.00195 |
| GO:0021700 | developmental maturation | 0.147465 | 0.00194 |
| GO:0099240 | intrinsic component of synaptic membrane | 0.147465 | 0.00194 |
| GO:0045071 | negative regulation of viral genome replication | 0.2 | 0.00188 |
| GO:0070542 | response to fatty acid | 0.2 | 0.00188 |
| GO:1904035 | regulation of epithelial cell apoptotic process | 0.2 | 0.00188 |
| GO:0018212 | peptidyl-tyrosine modification | 0.2 | 0.00188 |
| GO:0016877 | ligase activity, forming carbon-sulfur bonds | 0.2 | 0.00188 |
| GO:0030100 | regulation of endocytosis | 0.148148 | 0.00188 |
| GO:2001056 | positive regulation of cysteine-type endopeptidase activity | 0.162338 | 0.00188 |
| GO:0080164 | regulation of nitric oxide metabolic process | 0.229167 | 0.00181 |
| GO:0140678 | molecular function inhibitor activity | 0.229167 | 0.00181 |
| GO:0015035 | protein-disulfide reductase activity | 0.229167 | 0.00181 |
| GO:0046887 | positive regulation of hormone secretion | 0.165468 | 0.00180 |
| GO:0050136 | NADH dehydrogenase (quinone) activity | 0.218182 | 0.00179 |
| GO:0015036 | disulfide oxidoreductase activity | 0.218182 | 0.00179 |
| GO:0008137 | NADH dehydrogenase (ubiquinone) activity | 0.218182 | 0.00179 |
| GO:0006885 | regulation of pH | 0.186813 | 0.00177 |
| GO:1903311 | regulation of mRNA metabolic process | 0.135385 | 0.00176 |
| GO:0010975 | regulation of neuron projection development | 0.125514 | 0.00173 |
| GO:0070972 | protein localization to endoplasmic reticulum | 0.209677 | 0.00172 |
| GO:1901661 | quinone metabolic process | 0.209677 | 0.00172 |
| GO:0030117 | membrane coat | 0.209677 | 0.00172 |
| GO:0019904 | protein domain specific binding | 0.111842 | 0.00171 |
| GO:0070723 | response to cholesterol | 0.285714 | 0.00168 |
| GO:0010952 | positive regulation of peptidase activity | 0.155556 | 0.00168 |
| GO:0010558 | negative regulation of macromolecule biosynthetic process | 0.104533 | 0.00164 |
| GO:0032770 | positive regulation of monooxygenase activity | 0.318182 | 0.00164 |
| GO:0016741 | transferase activity, transferring one-carbon groups | 0.136646 | 0.00164 |
| GO:0006690 | icosanoid metabolic process | 0.167883 | 0.00163 |
| GO:0070403 | NAD+ binding | 0.188889 | 0.00163 |
| GO:1990837 | sequence-specific double-stranded DNA binding | 0.109338 | 0.00163 |
| GO:0034614 | cellular response to reactive oxygen species | 0.181818 | 0.00163 |
| GO:0016248 | channel inhibitor activity | 0.264706 | 0.00159 |
| GO:0034061 | DNA polymerase activity | 0.152632 | 0.00157 |
| GO:0090316 | positive regulation of intracellular protein transport | 0.169118 | 0.00157 |
| GO:0043433 | negative regulation of DNA-binding transcription factor activity | 0.163265 | 0.00155 |
| GO:1904950 | negative regulation of establishment of protein localization | 0.163265 | 0.00155 |
| GO:0030301 | cholesterol transport | 0.222222 | 0.00151 |
| GO:0015918 | sterol transport | 0.222222 | 0.00151 |
| GO:0033013 | tetrapyrrole metabolic process | 0.222222 | 0.00151 |
| GO:0008374 | O-acyltransferase activity | 0.222222 | 0.00151 |
| GO:2001141 | regulation of RNA biosynthetic process | 0.097406 | 0.00151 |
| GO:0061178 | regulation of insulin secretion involved in cellular response to glucose stimulus | 0.191011 | 0.00151 |
| GO:0016485 | protein processing | 0.150485 | 0.00150 |
| GO:0097485 | neuron projection guidance | 0.150485 | 0.00150 |
| GO:0046676 | negative regulation of insulin secretion | 0.213115 | 0.00147 |
| GO:0019438 | aromatic compound biosynthetic process | 0.112731 | 0.00143 |
| GO:2000811 | negative regulation of anoikis | 0.571429 | 0.00143 |
| GO:0015990 | electron transport coupled proton transport | 0.571429 | 0.00143 |
| GO:0046477 | glycosylceramide catabolic process | 0.571429 | 0.00143 |
| GO:0098698 | postsynaptic specialization assembly | 0.571429 | 0.00143 |
| GO:0006198 | cAMP catabolic process | 0.571429 | 0.00143 |
| GO:0098917 | retrograde trans-synaptic signaling | 0.571429 | 0.00143 |
| GO:0015988 | energy coupled proton transmembrane transport, against electrochemical gradient | 0.571429 | 0.00143 |
| GO:1900221 | regulation of amyloid-beta clearance | 0.571429 | 0.00143 |
| GO:0006069 | ethanol oxidation | 0.571429 | 0.00143 |
| GO:0016635 | oxidoreductase activity, acting on the CH-CH group of donors, quinone or related compound as acceptor | 0.571429 | 0.00143 |
| GO:0004024 | alcohol dehydrogenase activity, zinc-dependent | 0.571429 | 0.00143 |
| GO:0004703 | G protein-coupled receptor kinase activity | 0.571429 | 0.00143 |
| GO:0031995 | insulin-like growth factor II binding | 0.571429 | 0.00143 |
| GO:0004177 | aminopeptidase activity | 0.25 | 0.00143 |
| GO:0008152 | metabolic process | 0.090234 | 0.00143 |
| GO:1901343 | negative regulation of vasculature development | 0.193182 | 0.00141 |
| GO:0016525 | negative regulation of angiogenesis | 0.193182 | 0.00141 |
| GO:2000181 | negative regulation of blood vessel morphogenesis | 0.193182 | 0.00141 |
| GO:0002792 | negative regulation of peptide secretion | 0.205882 | 0.00140 |
| GO:0090278 | negative regulation of peptide hormone secretion | 0.205882 | 0.00140 |
| GO:0070304 | positive regulation of stress-activated protein kinase signaling cascade | 0.179245 | 0.00139 |
| GO:0002698 | negative regulation of immune effector process | 0.179245 | 0.00139 |
| GO:0016209 | antioxidant activity | 0.179245 | 0.00139 |
| GO:1903506 | regulation of nucleic acid-templated transcription | 0.097475 | 0.00136 |
| GO:0032292 | peripheral nervous system axon ensheathment | 0.375 | 0.00136 |
| GO:0022011 | myelination in peripheral nervous system | 0.375 | 0.00136 |
| GO:0051000 | positive regulation of nitric-oxide synthase activity | 0.375 | 0.00136 |
| GO:0008306 | associative learning | 0.185567 | 0.00134 |
| GO:0006974 | cellular response to DNA damage stimulus | 0.114664 | 0.00134 |
| GO:0007411 | axon guidance | 0.152709 | 0.00132 |
| GO:0002250 | adaptive immune response | 0.141379 | 0.00131 |
| GO:0050807 | regulation of synapse organization | 0.150685 | 0.00131 |
| GO:0045597 | positive regulation of cell differentiation | 0.116168 | 0.00130 |
| GO:0030947 | regulation of vascular endothelial growth factor receptor signaling pathway | 0.296296 | 0.00130 |
| GO:0003712 | transcription coregulator activity | 0.123894 | 0.00129 |
| GO:1905368 | peptidase complex | 0.166667 | 0.00128 |
| GO:0006816 | calcium ion transport | 0.153465 | 0.00127 |
| GO:0021549 | cerebellum development | 0.226415 | 0.00127 |
| GO:0019842 | vitamin binding | 0.155914 | 0.00127 |
| GO:0035338 | long-chain fatty-acyl-CoA biosynthetic process | 0.454545 | 0.00127 |
| GO:0009396 | folic acid-containing compound biosynthetic process | 0.454545 | 0.00127 |
| GO:0009437 | carnitine metabolic process | 0.454545 | 0.00127 |
| GO:0032612 | interleukin-1 production | 0.454545 | 0.00127 |
| GO:0032611 | interleukin-1 beta production | 0.454545 | 0.00127 |
| GO:0039706 | co-receptor binding | 0.454545 | 0.00127 |
| GO:0015232 | heme transmembrane transporter activity | 0.454545 | 0.00127 |
| GO:0098797 | plasma membrane protein complex | 0.121753 | 0.00127 |
| GO:0032874 | positive regulation of stress-activated MAPK cascade | 0.180952 | 0.00126 |
| GO:0097110 | scaffold protein binding | 0.216667 | 0.00126 |
| GO:0045321 | leukocyte activation | 0.125234 | 0.00125 |
| GO:1901568 | fatty acid derivative metabolic process | 0.23913 | 0.00125 |
| GO:0045185 | maintenance of protein location | 0.1875 | 0.00123 |
| GO:0043524 | negative regulation of neuron apoptotic process | 0.167832 | 0.00121 |
| GO:0051224 | negative regulation of protein transport | 0.167832 | 0.00121 |
| GO:1900542 | regulation of purine nucleotide metabolic process | 0.208955 | 0.00121 |
| GO:0006360 | transcription by RNA polymerase I | 0.333333 | 0.00121 |
| GO:0045655 | regulation of monocyte differentiation | 0.333333 | 0.00121 |
| GO:0060716 | labyrinthine layer blood vessel development | 0.333333 | 0.00121 |
| GO:0009595 | detection of biotic stimulus | 0.333333 | 0.00121 |
| GO:0065005 | protein-lipid complex assembly | 0.333333 | 0.00121 |
| GO:0034620 | cellular response to unfolded protein | 0.333333 | 0.00121 |
| GO:0034385 | triglyceride-rich plasma lipoprotein particle | 0.333333 | 0.00121 |
| GO:0034361 | very-low-density lipoprotein particle | 0.333333 | 0.00121 |
| GO:0001775 | cell activation | 0.122549 | 0.00120 |
| GO:0051240 | positive regulation of multicellular organismal process | 0.106184 | 0.00119 |
| GO:0051960 | regulation of nervous system development | 0.127883 | 0.00115 |
| GO:0003690 | double-stranded DNA binding | 0.109025 | 0.00115 |
| GO:0043270 | positive regulation of ion transport | 0.141844 | 0.00114 |
| GO:0008593 | regulation of Notch signaling pathway | 0.202703 | 0.00114 |
| GO:1903322 | positive regulation of protein modification by small protein conjugation or removal | 0.160714 | 0.00114 |
| GO:0042180 | cellular ketone metabolic process | 0.179487 | 0.00113 |
| GO:0001818 | negative regulation of cytokine production | 0.149123 | 0.00110 |
| GO:0030183 | B cell differentiation | 0.173228 | 0.00109 |
| GO:0005543 | phospholipid binding | 0.127016 | 0.00109 |
| GO:0042383 | sarcolemma | 0.181034 | 0.00108 |
| GO:0042113 | B cell activation | 0.154639 | 0.00108 |
| GO:0032024 | positive regulation of insulin secretion | 0.197531 | 0.00106 |
| GO:0031396 | regulation of protein ubiquitination | 0.145594 | 0.00104 |
| GO:1904064 | positive regulation of cation transmembrane transport | 0.165563 | 0.00103 |
| GO:0002820 | negative regulation of adaptive immune response | 0.244444 | 0.00103 |
| GO:0030968 | endoplasmic reticulum unfolded protein response | 0.244444 | 0.00103 |
| GO:0045047 | protein targeting to ER | 0.244444 | 0.00103 |
| GO:1903556 | negative regulation of tumor necrosis factor superfamily cytokine production | 0.244444 | 0.00103 |
| GO:0042594 | response to starvation | 0.148148 | 0.00102 |
| GO:0070372 | regulation of ERK1 and ERK2 cascade | 0.143885 | 0.00102 |
| GO:0043648 | dicarboxylic acid metabolic process | 0.163636 | 0.00099 |
| GO:0009066 | aspartate family amino acid metabolic process | 0.205479 | 0.00098 |
| GO:1901699 | cellular response to nitrogen compound | 0.123729 | 0.00098 |
| GO:0010556 | regulation of macromolecule biosynthetic process | 0.096588 | 0.00097 |
| GO:0007169 | transmembrane receptor protein tyrosine kinase signaling pathway | 0.140575 | 0.00097 |
| GO:0007160 | cell-matrix adhesion | 0.193548 | 0.00096 |
| GO:0031267 | small GTPase binding | 0.13253 | 0.00094 |
| GO:1903018 | regulation of glycoprotein metabolic process | 0.263158 | 0.00093 |
| GO:0042445 | hormone metabolic process | 0.16 | 0.00092 |
| GO:0006952 | defense response | 0.106011 | 0.00092 |
| GO:0097153 | cysteine-type endopeptidase activity involved in apoptotic process | 0.4 | 0.00091 |
| GO:0051100 | negative regulation of binding | 0.167785 | 0.00091 |
| GO:0031398 | positive regulation of protein ubiquitination | 0.167785 | 0.00091 |
| GO:1902494 | catalytic complex | 0.10374 | 0.00090 |
| GO:0051540 | metal cluster binding | 0.195652 | 0.00090 |
| GO:0051536 | iron-sulfur cluster binding | 0.195652 | 0.00090 |
| GO:0010677 | negative regulation of cellular carbohydrate metabolic process | 0.224138 | 0.00090 |
| GO:0016874 | ligase activity | 0.142361 | 0.00089 |
| GO:0050920 | regulation of chemotaxis | 0.156098 | 0.00088 |
| GO:0048037 | obsolete cofactor binding | 0.35 | 0.00087 |
| GO:0004030 | aldehyde dehydrogenase [NAD(P)+] activity | 0.35 | 0.00087 |
| GO:1904062 | regulation of cation transmembrane transport | 0.138889 | 0.00086 |
| GO:0007005 | mitochondrion organization | 0.136612 | 0.00085 |
| GO:0098739 | import across plasma membrane | 0.188119 | 0.00085 |
| GO:0005246 | calcium channel regulator activity | 0.25 | 0.00084 |
| GO:0071695 | anatomical structure maturation | 0.159574 | 0.00082 |
| GO:0099503 | secretory vesicle | 0.126923 | 0.00082 |
| GO:0001523 | retinoid metabolic process | 0.202532 | 0.00080 |
| GO:0000041 | transition metal ion transport | 0.171233 | 0.00078 |
| GO:0051015 | actin filament binding | 0.150862 | 0.00078 |
| GO:0035383 | thioester metabolic process | 0.19 | 0.00078 |
| GO:0006637 | acyl-CoA metabolic process | 0.19 | 0.00078 |
| GO:0032886 | regulation of microtubule-based process | 0.146617 | 0.00078 |
| GO:0110053 | regulation of actin filament organization | 0.14717 | 0.00076 |
| GO:0007219 | Notch signaling pathway | 0.181818 | 0.00075 |
| GO:0034754 | cellular hormone metabolic process | 0.181818 | 0.00075 |
| GO:0015980 | energy derivation by oxidation of organic compounds | 0.14539 | 0.00075 |
| GO:0048029 | monosaccharide binding | 0.197674 | 0.00075 |
| GO:0006703 | estrogen biosynthetic process | 0.5 | 0.00074 |
| GO:1903209 | positive regulation of oxidative stress-induced cell death | 0.5 | 0.00074 |
| GO:0010612 | regulation of cardiac muscle adaptation | 0.5 | 0.00074 |
| GO:0042994 | cytoplasmic sequestering of transcription factor | 0.5 | 0.00074 |
| GO:1902992 | negative regulation of amyloid precursor protein catabolic process | 0.5 | 0.00074 |
| GO:0031313 | extrinsic component of endosome membrane | 0.5 | 0.00074 |
| GO:0004022 | alcohol dehydrogenase (NAD+) activity | 0.5 | 0.00074 |
| GO:1902904 | negative regulation of supramolecular fiber organization | 0.166667 | 0.00074 |
| GO:0019209 | kinase activator activity | 0.166667 | 0.00074 |
| GO:1903421 | regulation of synaptic vesicle recycling | 0.32 | 0.00074 |
| GO:0016405 | CoA-ligase activity | 0.32 | 0.00074 |
| GO:0008219 | cell death | 0.113657 | 0.00073 |
| GO:0043588 | skin development | 0.24 | 0.00073 |
| GO:0051258 | protein polymerization | 0.211268 | 0.00073 |
| GO:0022402 | cell cycle process | 0.116356 | 0.00072 |
| GO:0042178 | xenobiotic catabolic process | 0.205128 | 0.00069 |
| GO:0097386 | glial cell projection | 0.255814 | 0.00068 |
| GO:0010494 | cytoplasmic stress granule | 0.176923 | 0.00066 |
| GO:0035507 | regulation of myosin-light-chain-phosphatase activity | 0.666667 | 0.00066 |
| GO:0061687 | detoxification of inorganic compound | 0.666667 | 0.00066 |
| GO:0034139 | regulation of toll-like receptor 3 signaling pathway | 0.666667 | 0.00066 |
| GO:1990130 | GATOR1 complex | 0.666667 | 0.00066 |
| GO:0031732 | CCR7 chemokine receptor binding | 0.666667 | 0.00066 |
| GO:0030235 | nitric-oxide synthase regulator activity | 0.666667 | 0.00066 |
| GO:0008177 | succinate dehydrogenase (ubiquinone) activity | 0.666667 | 0.00066 |
| GO:0000104 | succinate dehydrogenase activity | 0.666667 | 0.00066 |
| GO:0050777 | negative regulation of immune response | 0.168831 | 0.00065 |
| GO:0005525 | GTP binding | 0.124204 | 0.00063 |
| GO:0006520 | cellular amino acid metabolic process | 0.134146 | 0.00063 |
| GO:1903578 | regulation of ATP metabolic process | 0.232143 | 0.00063 |
| GO:0070330 | aromatase activity | 0.232143 | 0.00063 |
| GO:0046649 | lymphocyte activation | 0.132558 | 0.00063 |
| GO:0006749 | glutathione metabolic process | 0.214286 | 0.00062 |
| GO:0008081 | phosphoric diester hydrolase activity | 0.195876 | 0.00062 |
| GO:0007613 | memory | 0.178295 | 0.00062 |
| GO:1900242 | regulation of synaptic vesicle endocytosis | 0.368421 | 0.00061 |
| GO:0070402 | NADPH binding | 0.368421 | 0.00061 |
| GO:0070859 | positive regulation of bile acid biosynthetic process | 1 | 0.00060 |
| GO:1904253 | positive regulation of bile acid metabolic process | 1 | 0.00060 |
| GO:0002232 | leukocyte chemotaxis involved in inflammatory response | 1 | 0.00060 |
| GO:1990584 | cardiac Troponin complex | 1 | 0.00060 |
| GO:0045892 | negative regulation of DNA-templated transcription | 0.109875 | 0.00060 |
| GO:0030641 | regulation of cellular pH | 0.207792 | 0.00060 |
| GO:0042391 | regulation of membrane potential | 0.135135 | 0.00059 |
| GO:0050667 | homocysteine metabolic process | 0.428571 | 0.00059 |
| GO:0060192 | negative regulation of lipase activity | 0.428571 | 0.00059 |
| GO:0002523 | leukocyte migration involved in inflammatory response | 0.428571 | 0.00059 |
| GO:1902236 | negative regulation of endoplasmic reticulum stress-induced intrinsic apoptotic signaling pathway | 0.428571 | 0.00059 |
| GO:0030169 | low-density lipoprotein particle binding | 0.428571 | 0.00059 |
| GO:0032768 | regulation of monooxygenase activity | 0.3 | 0.00059 |
| GO:0051338 | regulation of transferase activity | 0.117647 | 0.00059 |
| GO:0034762 | regulation of transmembrane transport | 0.127306 | 0.00059 |
| GO:0048286 | lung alveolus development | 0.277778 | 0.00058 |
| GO:0035239 | tube morphogenesis | 0.151899 | 0.00057 |
| GO:1904427 | positive regulation of calcium ion transmembrane transport | 0.202381 | 0.00056 |
| GO:0031330 | negative regulation of cellular catabolic process | 0.154545 | 0.00056 |
| GO:0042398 | cellular modified amino acid biosynthetic process | 0.261905 | 0.00055 |
| GO:0032391 | photoreceptor connecting cilium | 0.261905 | 0.00055 |
| GO:0006888 | endoplasmic reticulum to Golgi vesicle-mediated transport | 0.181102 | 0.00055 |
| GO:0046328 | regulation of JNK cascade | 0.181102 | 0.00055 |
| GO:0045912 | negative regulation of carbohydrate metabolic process | 0.225806 | 0.00053 |
| GO:0006635 | fatty acid beta-oxidation | 0.225806 | 0.00053 |
| GO:1990928 | response to amino acid starvation | 0.236364 | 0.00052 |
| GO:0009887 | animal organ morphogenesis | 0.12806 | 0.00052 |
| GO:0034764 | positive regulation of transmembrane transport | 0.155963 | 0.00051 |
| GO:0019369 | arachidonic acid metabolic process | 0.210526 | 0.00051 |
| GO:0018904 | ether metabolic process | 0.25 | 0.00049 |
| GO:0019217 | regulation of fatty acid metabolic process | 0.204819 | 0.00049 |
| GO:0140110 | transcription regulator activity | 0.107221 | 0.00048 |
| GO:0090257 | regulation of muscle system process | 0.157407 | 0.00048 |
| GO:0070302 | regulation of stress-activated protein kinase signaling cascade | 0.162162 | 0.00048 |
| GO:0051262 | protein tetramerization | 0.162162 | 0.00048 |
| GO:1902533 | positive regulation of intracellular signal transduction | 0.114184 | 0.00047 |
| GO:0032561 | guanyl ribonucleotide binding | 0.124233 | 0.00047 |
| GO:0030674 | protein-macromolecule adaptor activity | 0.151639 | 0.00046 |
| GO:0016810 | hydrolase activity, acting on carbon-nitrogen (but not peptide) bonds | 0.151639 | 0.00046 |
| GO:0005856 | cytoskeleton | 0.113271 | 0.00045 |
| GO:0030149 | sphingolipid catabolic process | 0.310345 | 0.00045 |
| GO:0008285 | negative regulation of cell population proliferation | 0.125806 | 0.00044 |
| GO:0042304 | regulation of fatty acid biosynthetic process | 0.268293 | 0.00044 |
| GO:0005814 | centriole | 0.166667 | 0.00044 |
| GO:0032940 | secretion by cell | 0.138965 | 0.00043 |
| GO:0042277 | peptide binding | 0.138965 | 0.00043 |
| GO:0006875 | cellular metal ion homeostasis | 0.144695 | 0.00043 |
| GO:0003714 | transcription corepressor activity | 0.154867 | 0.00043 |
| GO:0060284 | regulation of cell development | 0.129594 | 0.00043 |
| GO:0009314 | response to radiation | 0.131915 | 0.00043 |
| GO:0016477 | cell migration | 0.12005 | 0.00042 |
| GO:0032872 | regulation of stress-activated MAPK cascade | 0.163934 | 0.00042 |
| GO:0034440 | lipid oxidation | 0.207317 | 0.00042 |
| GO:0002460 | adaptive immune response based on somatic recombination of immune receptors built from immunoglobulin superfamily domains | 0.207317 | 0.00042 |
| GO:1903131 | mononuclear cell differentiation | 0.14726 | 0.00042 |
| GO:0006677 | glycosylceramide metabolic process | 0.388889 | 0.00041 |
| GO:0048193 | Golgi vesicle transport | 0.149635 | 0.00041 |
| GO:0048568 | embryonic organ development | 0.180451 | 0.00040 |
| GO:0034767 | positive regulation of ion transmembrane transport | 0.167665 | 0.00040 |
| GO:0051494 | negative regulation of cytoskeleton organization | 0.167665 | 0.00040 |
| GO:0044282 | small molecule catabolic process | 0.125589 | 0.00040 |
| GO:0046915 | transition metal ion transmembrane transporter activity | 0.255319 | 0.00040 |
| GO:1902430 | negative regulation of amyloid-beta formation | 0.555556 | 0.00040 |
| GO:0031957 | very long-chain fatty acid-CoA ligase activity | 0.555556 | 0.00040 |
| GO:0045202 | synapse | 0.112128 | 0.00040 |
| GO:0016746 | acyltransferase activity | 0.142857 | 0.00039 |
| GO:0050999 | regulation of nitric-oxide synthase activity | 0.347826 | 0.00039 |
| GO:0052745 | inositol phosphate phosphatase activity | 0.347826 | 0.00039 |
| GO:0019200 | carbohydrate kinase activity | 0.347826 | 0.00039 |
| GO:0001822 | kidney development | 0.173077 | 0.00039 |
| GO:0005777 | peroxisome | 0.173077 | 0.00039 |
| GO:0035023 | regulation of Rho protein signal transduction | 0.181818 | 0.00038 |
| GO:0006721 | terpenoid metabolic process | 0.2 | 0.00038 |
| GO:0043539 | protein serine/threonine kinase activator activity | 0.2 | 0.00038 |
| GO:0045471 | response to ethanol | 0.168675 | 0.00037 |
| GO:0007612 | learning | 0.174194 | 0.00037 |
| GO:0008250 | oligosaccharyltransferase complex | 0.461538 | 0.00036 |
| GO:0019855 | calcium channel inhibitor activity | 0.461538 | 0.00036 |
| GO:0045787 | positive regulation of cell cycle | 0.142012 | 0.00036 |
| GO:0033273 | response to vitamin | 0.209877 | 0.00036 |
| GO:0019395 | fatty acid oxidation | 0.209877 | 0.00036 |
| GO:0000122 | negative regulation of transcription by RNA polymerase II | 0.117834 | 0.00036 |
| GO:1902679 | negative regulation of RNA biosynthetic process | 0.11088 | 0.00035 |
| GO:0071825 | protein-lipid complex subunit organization | 0.294118 | 0.00035 |
| GO:1903507 | negative regulation of nucleic acid-templated transcription | 0.110957 | 0.00035 |
| GO:0035770 | ribonucleoprotein granule | 0.144201 | 0.00035 |
| GO:1901652 | response to peptide | 0.135945 | 0.00033 |
| GO:0010954 | positive regulation of protein processing | 0.321429 | 0.00033 |
| GO:0021952 | central nervous system projection neuron axonogenesis | 0.321429 | 0.00033 |
| GO:0006897 | endocytosis | 0.149466 | 0.00032 |
| GO:0071466 | cellular response to xenobiotic stimulus | 0.219178 | 0.00032 |
| GO:0007268 | chemical synaptic transmission | 0.15 | 0.00031 |
| GO:0098916 | anterograde trans-synaptic signaling | 0.15 | 0.00031 |
| GO:0048471 | perinuclear region of cytoplasm | 0.119767 | 0.00031 |
| GO:0055088 | lipid homeostasis | 0.175676 | 0.00030 |
| GO:0030295 | protein kinase activator activity | 0.175676 | 0.00030 |
| GO:0098857 | membrane microdomain | 0.137931 | 0.00029 |
| GO:0051129 | negative regulation of cellular component organization | 0.123944 | 0.00029 |
| GO:0051020 | GTPase binding | 0.133621 | 0.00029 |
| GO:0019001 | guanyl nucleotide binding | 0.125382 | 0.00029 |
| GO:0045121 | membrane raft | 0.138614 | 0.00028 |
| GO:0006577 | amino-acid betaine metabolic process | 0.411765 | 0.00027 |
| GO:0019433 | triglyceride catabolic process | 0.411765 | 0.00027 |
| GO:0034377 | plasma lipoprotein particle assembly | 0.411765 | 0.00027 |
| GO:0006067 | ethanol metabolic process | 0.411765 | 0.00027 |
| GO:0034663 | endoplasmic reticulum chaperone complex | 0.411765 | 0.00027 |
| GO:0042578 | phosphoric ester hydrolase activity | 0.135747 | 0.00027 |
| GO:0006110 | regulation of glycolytic process | 0.30303 | 0.00027 |
| GO:0051279 | regulation of release of sequestered calcium ion into cytosol | 0.222222 | 0.00027 |
| GO:0051453 | regulation of intracellular pH | 0.222222 | 0.00027 |
| GO:0043549 | regulation of kinase activity | 0.123457 | 0.00027 |
| GO:0042981 | regulation of apoptotic process | 0.107438 | 0.00027 |
| GO:0030162 | regulation of proteolysis | 0.118844 | 0.00027 |
| GO:0018130 | heterocycle biosynthetic process | 0.116959 | 0.00026 |
| GO:0006662 | glycerol ether metabolic process | 0.266667 | 0.00026 |
| GO:0009925 | basal plasma membrane | 0.266667 | 0.00026 |
| GO:0010507 | negative regulation of autophagy | 0.241379 | 0.00026 |
| GO:0003955 | NAD(P)H dehydrogenase (quinone) activity | 0.241379 | 0.00026 |
| GO:0045785 | positive regulation of cell adhesion | 0.140212 | 0.00026 |
| GO:0036464 | cytoplasmic ribonucleoprotein granule | 0.147541 | 0.00025 |
| GO:0034364 | high-density lipoprotein particle | 0.333333 | 0.00024 |
| GO:0030178 | negative regulation of Wnt signaling pathway | 0.185185 | 0.00024 |
| GO:0035418 | protein localization to synapse | 0.254902 | 0.00024 |
| GO:0033116 | endoplasmic reticulum-Golgi intermediate compartment membrane | 0.254902 | 0.00024 |
| GO:0008022 | protein C-terminus binding | 0.15415 | 0.00024 |
| GO:0034141 | positive regulation of toll-like receptor 3 signaling pathway | 0.8 | 0.00023 |
| GO:1990961 | xenobiotic detoxification by transmembrane export across the plasma membrane | 0.8 | 0.00023 |
| GO:0016936 | galactoside binding | 0.8 | 0.00023 |
| GO:0051347 | positive regulation of transferase activity | 0.129663 | 0.00023 |
| GO:0006643 | membrane lipid metabolic process | 0.164948 | 0.00023 |
| GO:0031226 | intrinsic component of plasma membrane | 0.110825 | 0.00023 |
| GO:0031327 | negative regulation of cellular biosynthetic process | 0.107843 | 0.00023 |
| GO:0098563 | intrinsic component of synaptic vesicle membrane | 0.234375 | 0.00022 |
| GO:0004536 | deoxyribonuclease activity | 0.245614 | 0.00021 |
| GO:1901503 | ether biosynthetic process | 0.289474 | 0.00021 |
| GO:0046504 | glycerol ether biosynthetic process | 0.289474 | 0.00021 |
| GO:0008611 | ether lipid biosynthetic process | 0.289474 | 0.00021 |
| GO:0050714 | positive regulation of protein secretion | 0.176471 | 0.00021 |
| GO:0006829 | zinc ion transport | 0.3125 | 0.00020 |
| GO:1901605 | alpha-amino acid metabolic process | 0.149153 | 0.00020 |
| GO:0019901 | protein kinase binding | 0.123077 | 0.00020 |
| GO:0030176 | integral component of endoplasmic reticulum membrane | 0.186047 | 0.00020 |
| GO:0006986 | response to unfolded protein | 0.26 | 0.00019 |
| GO:0004129 | cytochrome-c oxidase activity | 0.26 | 0.00019 |
| GO:0071377 | cellular response to glucagon stimulus | 0.625 | 0.00019 |
| GO:0005577 | fibrinogen complex | 0.625 | 0.00019 |
| GO:0031994 | insulin-like growth factor I binding | 0.625 | 0.00019 |
| GO:0042609 | CD4 receptor binding | 0.625 | 0.00019 |
| GO:0043235 | receptor complex | 0.142857 | 0.00019 |
| GO:0046058 | cAMP metabolic process | 0.380952 | 0.00019 |
| GO:0050709 | negative regulation of protein secretion | 0.214286 | 0.00019 |
| GO:0046889 | positive regulation of lipid biosynthetic process | 0.214286 | 0.00019 |
| GO:0016101 | diterpenoid metabolic process | 0.214286 | 0.00019 |
| GO:0034655 | nucleobase-containing compound catabolic process | 0.148387 | 0.00019 |
| GO:0022408 | negative regulation of cell-cell adhesion | 0.175758 | 0.00018 |
| GO:0043177 | organic acid binding | 0.175758 | 0.00018 |
| GO:0043067 | regulation of programmed cell death | 0.108108 | 0.00018 |
| GO:0030234 | enzyme regulator activity | 0.11013 | 0.00018 |
| GO:0051384 | response to glucocorticoid | 0.171429 | 0.00018 |
| GO:0010675 | regulation of cellular carbohydrate metabolic process | 0.169312 | 0.00017 |
| GO:0006869 | lipid transport | 0.151203 | 0.00017 |
| GO:0016894 | endonuclease activity, active with either ribo- or deoxyribonucleic acids and producing 3'-phosphomonoesters | 0.4375 | 0.00017 |
| GO:0051926 | negative regulation of calcium ion transport | 0.25 | 0.00017 |
| GO:0030004 | cellular monovalent inorganic cation homeostasis | 0.204082 | 0.00017 |
| GO:0062014 | negative regulation of small molecule metabolic process | 0.204082 | 0.00017 |
| GO:0003964 | RNA-directed DNA polymerase activity | 0.185714 | 0.00017 |
| GO:0048729 | tissue morphogenesis | 0.142091 | 0.00017 |
| GO:0048858 | cell projection morphogenesis | 0.152249 | 0.00016 |
| GO:0045746 | negative regulation of Notch signaling pathway | 0.297297 | 0.00016 |
| GO:0000096 | sulfur amino acid metabolic process | 0.297297 | 0.00016 |
| GO:0046485 | ether lipid metabolic process | 0.27907 | 0.00016 |
| GO:0032994 | protein-lipid complex | 0.27907 | 0.00016 |
| GO:0030285 | integral component of synaptic vesicle membrane | 0.27907 | 0.00016 |
| GO:0070301 | cellular response to hydrogen peroxide | 0.223684 | 0.00016 |
| GO:0033674 | positive regulation of kinase activity | 0.137168 | 0.00016 |
| GO:1902414 | protein localization to cell junction | 0.231884 | 0.00016 |
| GO:0097435 | supramolecular fiber organization | 0.133981 | 0.00016 |
| GO:0045859 | regulation of protein kinase activity | 0.127496 | 0.00015 |
| GO:0042579 | microbody | 0.17341 | 0.00015 |
| GO:0043113 | receptor clustering | 0.265306 | 0.00015 |
| GO:0060674 | placenta blood vessel development | 0.322581 | 0.00015 |
| GO:0034330 | cell junction organization | 0.138498 | 0.00015 |
| GO:0018205 | peptidyl-lysine modification | 0.140049 | 0.00015 |
| GO:0022853 | active ion transmembrane transporter activity | 0.146707 | 0.00015 |
| GO:0098793 | presynapse | 0.150502 | 0.00014 |
| GO:0032990 | cell part morphogenesis | 0.147147 | 0.00014 |
| GO:0032989 | cellular component morphogenesis | 0.147147 | 0.00014 |
| GO:0043410 | positive regulation of MAPK cascade | 0.140741 | 0.00014 |
| GO:0071241 | cellular response to inorganic substance | 0.151007 | 0.00014 |
| GO:0005319 | lipid transporter activity | 0.175439 | 0.00014 |
| GO:0008395 | steroid hydroxylase activity | 0.213483 | 0.00013 |
| GO:0048812 | neuron projection morphogenesis | 0.153571 | 0.00013 |
| GO:0044283 | small molecule biosynthetic process | 0.124051 | 0.00013 |
| GO:0043470 | regulation of carbohydrate catabolic process | 0.285714 | 0.00013 |
| GO:0016197 | endosomal transport | 0.155894 | 0.00013 |
| GO:0016646 | oxidoreductase activity, acting on the CH-NH group of donors, NAD or NADP as acceptor | 0.4 | 0.00013 |
| GO:1905475 | regulation of protein localization to membrane | 0.172222 | 0.00013 |
| GO:0033344 | cholesterol efflux | 0.36 | 0.00012 |
| GO:0009190 | cyclic nucleotide biosynthetic process | 0.36 | 0.00012 |
| GO:0052652 | cyclic purine nucleotide metabolic process | 0.36 | 0.00012 |
| GO:0032787 | monocarboxylic acid metabolic process | 0.120419 | 0.00012 |
| GO:0072341 | modified amino acid binding | 0.2 | 0.00012 |
| GO:0000149 | SNARE binding | 0.2 | 0.00012 |
| GO:0045444 | fat cell differentiation | 0.193548 | 0.00012 |
| GO:0018455 | alcohol dehydrogenase [NAD(P)+] activity | 0.545455 | 0.00011 |
| GO:0004383 | guanylate cyclase activity | 0.545455 | 0.00011 |
| GO:0051098 | regulation of binding | 0.142494 | 0.00011 |
| GO:0071827 | plasma lipoprotein particle organization | 0.333333 | 0.00011 |
| GO:0016645 | oxidoreductase activity, acting on the CH-NH group of donors | 0.333333 | 0.00011 |
| GO:0050727 | regulation of inflammatory response | 0.15331 | 0.00010 |
| GO:2000147 | positive regulation of cell motility | 0.135827 | 0.00010 |
| GO:0009895 | negative regulation of catabolic process | 0.151815 | 0.00010 |
| GO:0099080 | supramolecular complex | 0.114079 | 0.00010 |
| GO:0098657 | import into cell | 0.19084 | 0.00010 |
| GO:0090287 | regulation of cellular response to growth factor stimulus | 0.161157 | 0.00010 |
| GO:0006357 | regulation of transcription by RNA polymerase II | 0.107019 | 0.00010 |
| GO:0090277 | positive regulation of peptide hormone secretion | 0.201835 | 0.00010 |
| GO:0033218 | amide binding | 0.138229 | 0.00010 |
| GO:0120039 | plasma membrane bounded cell projection morphogenesis | 0.154386 | 0.00010 |
| GO:0005044 | scavenger receptor activity | 0.264151 | 0.00009 |
| GO:0043271 | negative regulation of ion transport | 0.184211 | 0.00009 |
| GO:0009605 | response to external stimulus | 0.10481 | 0.00009 |
| GO:0050776 | regulation of immune response | 0.120778 | 0.00009 |
| GO:0072330 | monocarboxylic acid biosynthetic process | 0.180124 | 0.00009 |
| GO:0022904 | respiratory electron transport chain | 0.178161 | 0.00009 |
| GO:0044092 | negative regulation of molecular function | 0.116905 | 0.00009 |
| GO:0001525 | angiogenesis | 0.156028 | 0.00009 |
| GO:0045834 | positive regulation of lipid metabolic process | 0.18543 | 0.00008 |
| GO:1903169 | regulation of calcium ion transmembrane transport | 0.18543 | 0.00008 |
| GO:0030111 | regulation of Wnt signaling pathway | 0.157895 | 0.00008 |
| GO:0032934 | sterol binding | 0.254237 | 0.00008 |
| GO:0046903 | secretion | 0.137374 | 0.00008 |
| GO:0033865 | nucleoside bisphosphate metabolic process | 0.2 | 0.00008 |
| GO:0033875 | ribonucleoside bisphosphate metabolic process | 0.2 | 0.00008 |
| GO:0034032 | purine nucleoside bisphosphate metabolic process | 0.2 | 0.00008 |
| GO:0033365 | protein localization to organelle | 0.129272 | 0.00008 |
| GO:0005769 | early endosome | 0.149847 | 0.00008 |
| GO:0033762 | response to glucagon | 0.421053 | 0.00008 |
| GO:0009062 | fatty acid catabolic process | 0.22093 | 0.00008 |
| GO:0006665 | sphingolipid metabolic process | 0.193798 | 0.00008 |
| GO:0035967 | cellular response to topologically incorrect protein | 0.344828 | 0.00008 |
| GO:0046165 | alcohol biosynthetic process | 0.186667 | 0.00008 |
| GO:0055065 | metal ion homeostasis | 0.143939 | 0.00008 |
| GO:0009416 | response to light stimulus | 0.152104 | 0.00008 |
| GO:0050808 | synapse organization | 0.16309 | 0.00008 |
| GO:0010749 | regulation of nitric oxide mediated signal transduction | 0.714286 | 0.00008 |
| GO:0001955 | blood vessel maturation | 0.714286 | 0.00008 |
| GO:1902903 | regulation of supramolecular fiber organization | 0.147632 | 0.00008 |
| GO:0009986 | cell surface | 0.127199 | 0.00007 |
| GO:0030054 | cell junction | 0.107865 | 0.00007 |
| GO:0072329 | monocarboxylic acid catabolic process | 0.205607 | 0.00007 |
| GO:0055067 | monovalent inorganic cation homeostasis | 0.195313 | 0.00007 |
| GO:0009410 | response to xenobiotic stimulus | 0.151703 | 0.00007 |
| GO:0040017 | positive regulation of locomotion | 0.135496 | 0.00007 |
| GO:0030335 | positive regulation of cell migration | 0.13843 | 0.00007 |
| GO:0043679 | axon terminus | 0.19685 | 0.00007 |
| GO:1902600 | proton transmembrane transport | 0.239437 | 0.00007 |
| GO:0016667 | oxidoreductase activity, acting on a sulfur group of donors | 0.239437 | 0.00007 |
| GO:0001227 | DNA-binding transcription repressor activity, RNA polymerase II-specific | 0.15625 | 0.00006 |
| GO:0001217 | DNA-binding transcription repressor activity | 0.15625 | 0.00006 |
| GO:0048870 | cell motility | 0.122186 | 0.00006 |
| GO:0044782 | cilium organization | 0.158672 | 0.00006 |
| GO:0051253 | negative regulation of RNA metabolic process | 0.113329 | 0.00006 |
| GO:0012506 | vesicle membrane | 0.127517 | 0.00006 |
| GO:0005520 | insulin-like growth factor binding | 0.391304 | 0.00006 |
| GO:0007264 | small GTPase mediated signal transduction | 0.146597 | 0.00006 |
| GO:0010959 | regulation of metal ion transport | 0.146597 | 0.00006 |
| GO:0016675 | oxidoreductase activity, acting on a heme group of donors | 0.27451 | 0.00006 |
| GO:0032196 | transposition | 0.307692 | 0.00006 |
| GO:1990777 | lipoprotein particle | 0.307692 | 0.00006 |
| GO:0034358 | plasma lipoprotein particle | 0.307692 | 0.00006 |
| GO:0046464 | acylglycerol catabolic process | 0.357143 | 0.00006 |
| GO:0046461 | neutral lipid catabolic process | 0.357143 | 0.00006 |
| GO:0034116 | positive regulation of heterotypic cell-cell adhesion | 0.6 | 0.00006 |
| GO:0009890 | negative regulation of biosynthetic process | 0.110041 | 0.00006 |
| GO:0016709 | oxidoreductase activity, acting on paired donors, with incorporation or reduction of molecular oxygen, NAD(P)H as one donor, and incorporation of one atom of oxygen | 0.263158 | 0.00005 |
| GO:0009896 | positive regulation of catabolic process | 0.140127 | 0.00005 |
| GO:0002793 | positive regulation of peptide secretion | 0.205357 | 0.00005 |
| GO:0005539 | glycosaminoglycan binding | 0.157706 | 0.00005 |
| GO:0043567 | regulation of insulin-like growth factor receptor signaling pathway | 0.444444 | 0.00005 |
| GO:0051150 | regulation of smooth muscle cell differentiation | 0.444444 | 0.00005 |
| GO:0046466 | membrane lipid catabolic process | 0.333333 | 0.00005 |
| GO:0070613 | regulation of protein processing | 0.253968 | 0.00005 |
| GO:0043292 | contractile fiber | 0.253968 | 0.00005 |
| GO:0006672 | ceramide metabolic process | 0.222222 | 0.00005 |
| GO:0043112 | receptor metabolic process | 0.228916 | 0.00005 |
| GO:1903531 | negative regulation of secretion by cell | 0.180233 | 0.00005 |
| GO:0022900 | electron transport chain | 0.177419 | 0.00005 |
| GO:0140352 | export from cell | 0.144608 | 0.00005 |
| GO:0051604 | protein maturation | 0.156997 | 0.00005 |
| GO:0016779 | nucleotidyltransferase activity | 0.149296 | 0.00005 |
| GO:0004519 | endonuclease activity | 0.149296 | 0.00005 |
| GO:0051252 | regulation of RNA metabolic process | 0.100501 | 0.00005 |
| GO:0030199 | collagen fibril organization | 0.295455 | 0.00005 |
| GO:0015850 | organic hydroxy compound transport | 0.207207 | 0.00004 |
| GO:0002521 | leukocyte differentiation | 0.151335 | 0.00004 |
| GO:0045860 | positive regulation of protein kinase activity | 0.14604 | 0.00004 |
| GO:0032197 | transposition, RNA-mediated | 0.315789 | 0.00004 |
| GO:0004520 | endodeoxyribonuclease activity | 0.315789 | 0.00004 |
| GO:0005524 | ATP binding | 0.108015 | 0.00004 |
| GO:0043525 | positive regulation of neuron apoptotic process | 0.258065 | 0.00004 |
| GO:0030742 | GTP-dependent protein binding | 0.37037 | 0.00004 |
| GO:0097060 | synaptic membrane | 0.156146 | 0.00004 |
| GO:0098772 | molecular function regulator activity | 0.107017 | 0.00004 |
| GO:0030695 | GTPase regulator activity | 0.134907 | 0.00004 |
| GO:0060589 | nucleoside-triphosphatase regulator activity | 0.134907 | 0.00004 |
| GO:0017169 | CDP-alcohol phosphatidyltransferase activity | 0.34375 | 0.00004 |
| GO:1901701 | cellular response to oxygen-containing compound | 0.119416 | 0.00004 |
| GO:0098798 | mitochondrial protein-containing complex | 0.137112 | 0.00003 |
| GO:0042176 | regulation of protein catabolic process | 0.144186 | 0.00003 |
| GO:0005788 | endoplasmic reticulum lumen | 0.191781 | 0.00003 |
| GO:0009636 | response to toxic substance | 0.215686 | 0.00003 |
| GO:0044089 | positive regulation of cellular component biogenesis | 0.141631 | 0.00003 |
| GO:0030324 | lung development | 0.211009 | 0.00003 |
| GO:0043178 | alcohol binding | 0.211009 | 0.00003 |
| GO:0004115 | 3',5'-cyclic-AMP phosphodiesterase activity | 0.538462 | 0.00003 |
| GO:0051036 | regulation of endosome size | 0.262295 | 0.00003 |
| GO:0031227 | intrinsic component of endoplasmic reticulum membrane | 0.19708 | 0.00003 |
| GO:0016053 | organic acid biosynthetic process | 0.155844 | 0.00003 |
| GO:0001676 | long-chain fatty acid metabolic process | 0.206897 | 0.00003 |
| GO:0030308 | negative regulation of cell growth | 0.178947 | 0.00003 |
| GO:0031960 | response to corticosteroid | 0.178947 | 0.00003 |
| GO:0007568 | aging | 0.162264 | 0.00003 |
| GO:0120035 | regulation of plasma membrane bounded cell projection organization | 0.131737 | 0.00003 |
| GO:0050820 | positive regulation of coagulation | 0.470588 | 0.00003 |
| GO:0031667 | response to nutrient levels | 0.138996 | 0.00003 |
| GO:0045934 | negative regulation of nucleobase-containing compound metabolic process | 0.11312 | 0.00003 |
| GO:0051093 | negative regulation of developmental process | 0.122984 | 0.00003 |
| GO:0097305 | response to alcohol | 0.152542 | 0.00003 |
| GO:0046394 | carboxylic acid biosynthetic process | 0.156863 | 0.00003 |
| GO:0031406 | carboxylic acid binding | 0.182857 | 0.00003 |
| GO:0031344 | regulation of cell projection organization | 0.131579 | 0.00003 |
| GO:0016712 | oxidoreductase activity, acting on paired donors, with incorporation or reduction of molecular oxygen, reduced flavin or flavoprotein as one donor, and incorporation of one atom of oxygen | 0.208696 | 0.00003 |
| GO:0006695 | cholesterol biosynthetic process | 0.291667 | 0.00003 |
| GO:0010605 | negative regulation of macromolecule metabolic process | 0.103885 | 0.00003 |
| GO:2000463 | positive regulation of excitatory postsynaptic potential | 0.384615 | 0.00003 |
| GO:0046873 | metal ion transmembrane transporter activity | 0.141949 | 0.00003 |
| GO:0046469 | platelet activating factor metabolic process | 0.354839 | 0.00003 |
| GO:0030554 | adenyl nucleotide binding | 0.10833 | 0.00003 |
| GO:0035458 | cellular response to interferon-beta | 0.266667 | 0.00003 |
| GO:0030659 | cytoplasmic vesicle membrane | 0.130194 | 0.00003 |
| GO:0033559 | unsaturated fatty acid metabolic process | 0.201493 | 0.00002 |
| GO:0032559 | adenyl ribonucleotide binding | 0.108633 | 0.00002 |
| GO:0009725 | response to hormone | 0.129243 | 0.00002 |
| GO:0002020 | protease binding | 0.190184 | 0.00002 |
| GO:0021540 | corpus callosum morphogenesis | 0.833333 | 0.00002 |
| GO:0032103 | positive regulation of response to external stimulus | 0.1477 | 0.00002 |
| GO:0021955 | central nervous system neuron axonogenesis | 0.333333 | 0.00002 |
| GO:0007611 | learning or memory | 0.162963 | 0.00002 |
| GO:0032956 | regulation of actin cytoskeleton organization | 0.153623 | 0.00002 |
| GO:1901214 | regulation of neuron death | 0.157051 | 0.00002 |
| GO:0015485 | cholesterol binding | 0.283019 | 0.00002 |
| GO:0046503 | glycerolipid catabolic process | 0.297872 | 0.00002 |
| GO:0007265 | Ras protein signal transduction | 0.163121 | 0.00002 |
| GO:0019900 | kinase binding | 0.126437 | 0.00002 |
| GO:0009991 | response to extracellular stimulus | 0.138434 | 0.00002 |
| GO:0001664 | G protein-coupled receptor binding | 0.157895 | 0.00002 |
| GO:0031974 | membrane-enclosed lumen | 0.128834 | 0.00002 |
| GO:0043233 | organelle lumen | 0.128834 | 0.00002 |
| GO:0051924 | regulation of calcium ion transport | 0.171548 | 0.00002 |
| GO:0045732 | positive regulation of protein catabolic process | 0.171548 | 0.00002 |
| GO:0006663 | platelet activating factor biosynthetic process | 0.366667 | 0.00002 |
| GO:1903319 | positive regulation of protein maturation | 0.366667 | 0.00002 |
| GO:0016878 | acid-thiol ligase activity | 0.366667 | 0.00002 |
| GO:0051336 | regulation of hydrolase activity | 0.119707 | 0.00002 |
| GO:0060627 | regulation of vesicle-mediated transport | 0.138686 | 0.00002 |
| GO:1900048 | positive regulation of hemostasis | 0.5 | 0.00002 |
| GO:0005978 | glycogen biosynthetic process | 0.5 | 0.00002 |
| GO:0030194 | positive regulation of blood coagulation | 0.5 | 0.00002 |
| GO:0009250 | glucan biosynthetic process | 0.5 | 0.00002 |
| GO:0051282 | regulation of sequestering of calcium ion | 0.214286 | 0.00002 |
| GO:0042470 | melanosome | 0.210084 | 0.00002 |
| GO:0048770 | pigment granule | 0.210084 | 0.00002 |
| GO:0031326 | regulation of cellular biosynthetic process | 0.100748 | 0.00002 |
| GO:0071949 | FAD binding | 0.288462 | 0.00002 |
| GO:0004518 | nuclease activity | 0.146512 | 0.00002 |
| GO:0060271 | cilium assembly | 0.169355 | 0.00002 |
| GO:0097384 | cellular lipid biosynthetic process | 0.325 | 0.00001 |
| GO:0005654 | nucleoplasm | 0.105051 | 0.00001 |
| GO:0006641 | triglyceride metabolic process | 0.257143 | 0.00001 |
| GO:0022407 | regulation of cell-cell adhesion | 0.153631 | 0.00001 |
| GO:0070013 | intracellular organelle lumen | 0.128993 | 0.00001 |
| GO:0031175 | neuron projection development | 0.146288 | 0.00001 |
| GO:0005488 | binding | 0.099929 | 0.00001 |
| GO:0019219 | regulation of nucleobase-containing compound metabolic process | 0.101499 | 0.00001 |
| GO:0008092 | cytoskeletal protein binding | 0.118234 | 0.00001 |
| GO:0003779 | actin binding | 0.139746 | 0.00001 |
| GO:0043229 | intracellular organelle | 0.102014 | 0.00001 |
| GO:0044306 | neuron projection terminus | 0.2 | 0.00001 |
| GO:0009987 | cellular process | 0.091008 | 0.00001 |
| GO:0050789 | regulation of biological process | 0.108866 | 0.00001 |
| GO:0005886 | plasma membrane | 0.100367 | 0.00001 |
| GO:0009719 | response to endogenous stimulus | 0.119685 | 0.00001 |
| GO:0099536 | synaptic signaling | 0.162162 | 0.00001 |
| GO:0099537 | trans-synaptic signaling | 0.162162 | 0.00001 |
| GO:0065007 | biological regulation | 0.109461 | 0.00001 |
| GO:0001558 | regulation of cell growth | 0.147126 | 0.00001 |
| GO:0043226 | organelle | 0.102718 | 0.00001 |
| GO:0009889 | regulation of biosynthetic process | 0.101056 | 0.00001 |
| GO:0050794 | regulation of cellular process | 0.109484 | 0.00001 |
| GO:0043227 | membrane-bounded organelle | 0.12362 | 0.00001 |
| GO:0031329 | regulation of cellular catabolic process | 0.134933 | 0.00001 |
| GO:0007162 | negative regulation of cell adhesion | 0.169291 | 0.00001 |
| GO:0010468 | regulation of gene expression | 0.099602 | 0.00001 |
| GO:0016020 | membrane | 0.112096 | 0.00001 |
| GO:0043231 | intracellular membrane-bounded organelle | 0.123286 | 0.00001 |
| GO:0031224 | intrinsic component of membrane | 0.104871 | 0.00001 |
| GO:0110165 | cellular anatomical entity | 0.095527 | 0.00001 |
| GO:0008201 | heparin binding | 0.17757 | 0.00001 |
| GO:1901681 | sulfur compound binding | 0.157143 | 0.00001 |
| GO:0016601 | Rac protein signal transduction | 0.253333 | 0.00001 |
| GO:0060255 | regulation of macromolecule metabolic process | 0.103348 | 0.00001 |
| GO:0003824 | catalytic activity | 0.123601 | 0.00001 |
| GO:0071248 | cellular response to metal ion | 0.181373 | 0.00001 |
| GO:0005515 | protein binding | 0.116442 | 0.00001 |
| GO:0080090 | regulation of primary metabolic process | 0.109047 | 0.00001 |
| GO:0005634 | nucleus | 0.104843 | 0.00001 |
| GO:0016021 | integral component of membrane | 0.104289 | 0.00001 |
| GO:0007409 | axonogenesis | 0.184211 | 0.00001 |
| GO:0040013 | negative regulation of locomotion | 0.165517 | 0.00001 |
| GO:0007041 | lysosomal transport | 0.198675 | 0.00001 |
| GO:0003674 | molecular_function | 0.088338 | 0.00001 |
| GO:0071840 | cellular component organization or biogenesis | 0.099359 | 0.00001 |
| GO:0043167 | ion binding | 0.122543 | 0.00001 |
| GO:0050896 | response to stimulus | 0.11639 | 0.00001 |
| GO:0030027 | lamellipodium | 0.176991 | 0.00001 |
| GO:0006720 | isoprenoid metabolic process | 0.205674 | 0.00001 |
| GO:0001881 | receptor recycling | 0.274194 | 0.00001 |
| GO:0016043 | cellular component organization | 0.102466 | 0.00001 |
| GO:0031347 | regulation of defense response | 0.13828 | 0.00001 |
| GO:0016829 | lyase activity | 0.160377 | 0.00001 |
| GO:0048518 | positive regulation of biological process | 0.120102 | 0.00001 |
| GO:0031323 | regulation of cellular metabolic process | 0.106667 | 0.00001 |
| GO:0019222 | regulation of metabolic process | 0.107481 | 0.00001 |
| GO:0051048 | negative regulation of secretion | 0.183168 | 0.00001 |
| GO:0008134 | transcription factor binding | 0.132484 | 0.00001 |
| GO:0005737 | cytoplasm | 0.113766 | 0.00001 |
| GO:1901698 | response to nitrogen compound | 0.120181 | 0.00001 |
| GO:0007267 | cell-cell signaling | 0.153061 | 0.00001 |
| GO:0030336 | negative regulation of cell migration | 0.173387 | 0.00001 |
| GO:0043269 | regulation of ion transport | 0.136024 | 0.00001 |
| GO:2000146 | negative regulation of cell motility | 0.169231 | 0.00001 |
| GO:0048522 | positive regulation of cellular process | 0.1178 | 0.00001 |
| GO:0051171 | regulation of nitrogen compound metabolic process | 0.107677 | 0.00001 |
| GO:0007165 | signal transduction | 0.103187 | 0.00001 |
| GO:0048519 | negative regulation of biological process | 0.12097 | 0.00001 |
| GO:0080135 | regulation of cellular response to stress | 0.137161 | 0.00001 |
| GO:0043169 | cation binding | 0.127288 | 0.00001 |
| GO:0002682 | regulation of immune system process | 0.117985 | 0.00001 |
| GO:0004142 | diacylglycerol cholinephosphotransferase activity | 0.392857 | 0.00001 |
| GO:0048523 | negative regulation of cellular process | 0.123077 | 0.00001 |
| GO:0032502 | developmental process | 0.122134 | 0.00001 |
| GO:0045944 | positive regulation of transcription by RNA polymerase II | 0.124031 | 0.00001 |
| GO:0051094 | positive regulation of developmental process | 0.120795 | 0.00001 |
| GO:0046872 | metal ion binding | 0.127637 | 0.00001 |
| GO:0010941 | regulation of cell death | 0.11327 | 0.00001 |
| GO:0036094 | small molecule binding | 0.114675 | 0.00001 |
| GO:0051179 | localization | 0.128734 | 0.00001 |
| GO:0009893 | positive regulation of metabolic process | 0.120304 | 0.00001 |
| GO:0008289 | lipid binding | 0.128821 | 0.00001 |
| GO:0051172 | negative regulation of nitrogen compound metabolic process | 0.109604 | 0.00001 |
| GO:0015318 | inorganic molecular entity transmembrane transporter activity | 0.127796 | 0.00001 |
| GO:0042127 | regulation of cell population proliferation | 0.116047 | 0.00001 |
| GO:0043168 | anion binding | 0.122754 | 0.00001 |
| GO:0048583 | regulation of response to stimulus | 0.129275 | 0.00001 |
| GO:0016651 | oxidoreductase activity, acting on NAD(P)H | 0.224299 | 0.00001 |
| GO:0010565 | regulation of cellular ketone metabolic process | 0.219298 | 0.00001 |
| GO:0048545 | response to steroid hormone | 0.17284 | 0.00001 |
| GO:0051716 | cellular response to stimulus | 0.117965 | 0.00001 |
| GO:0023051 | regulation of signaling | 0.129371 | 0.00001 |
| GO:0005829 | cytosol | 0.118694 | 0.00001 |
| GO:0051234 | establishment of localization | 0.128445 | 0.00001 |
| GO:1902653 | secondary alcohol biosynthetic process | 0.306122 | 0.00001 |
| GO:0006950 | response to stress | 0.124524 | 0.00001 |
| GO:1903317 | regulation of protein maturation | 0.268657 | 0.00001 |
| GO:0016655 | oxidoreductase activity, acting on NAD(P)H, quinone or similar compound as acceptor | 0.268657 | 0.00001 |
| GO:1901342 | regulation of vasculature development | 0.171206 | 0.00001 |
| GO:1901570 | fatty acid derivative biosynthetic process | 0.434783 | 0.00001 |
| GO:0035639 | purine ribonucleoside triphosphate binding | 0.110731 | 0.00001 |
| GO:0006109 | regulation of carbohydrate metabolic process | 0.181818 | 0.00001 |
| GO:1901265 | nucleoside phosphate binding | 0.108875 | 0.00001 |
| GO:0000166 | nucleotide binding | 0.108875 | 0.00001 |
| GO:0006810 | transport | 0.130839 | 0.00001 |
| GO:0065008 | regulation of biological quality | 0.12755 | 0.00001 |
| GO:0032501 | multicellular organismal process | 0.105849 | 0.00001 |
| GO:0031090 | organelle membrane | 0.139392 | 0.00001 |
| GO:0045765 | regulation of angiogenesis | 0.171875 | 0.00001 |
| GO:0071310 | cellular response to organic substance | 0.112574 | 0.00001 |
| GO:0010604 | positive regulation of macromolecule metabolic process | 0.118776 | 0.00001 |
| GO:0048869 | cellular developmental process | 0.112837 | 0.00001 |
| GO:0006259 | DNA metabolic process | 0.125346 | 0.00001 |
| GO:0016740 | transferase activity | 0.110073 | 0.00001 |
| GO:0140640 | catalytic activity, acting on a nucleic acid | 0.127812 | 0.00001 |
| GO:0009628 | response to abiotic stimulus | 0.121401 | 0.00001 |
| GO:0099572 | postsynaptic specialization | 0.15404 | 0.00001 |
| GO:0097367 | carbohydrate derivative binding | 0.116623 | 0.00001 |
| GO:0009892 | negative regulation of metabolic process | 0.109455 | 0.00001 |
| GO:0048856 | anatomical structure development | 0.125412 | 0.00001 |
| GO:0031324 | negative regulation of cellular metabolic process | 0.110515 | 0.00001 |
| GO:0051173 | positive regulation of nitrogen compound metabolic process | 0.128141 | 0.00001 |
| GO:0050790 | regulation of catalytic activity | 0.116962 | 0.00001 |
| GO:0042221 | response to chemical | 0.122816 | 0.00001 |
| GO:0009966 | regulation of signal transduction | 0.131949 | 0.00001 |
| GO:0010033 | response to organic substance | 0.117984 | 0.00001 |
| GO:0006793 | phosphorus metabolic process | 0.112727 | 0.00001 |
| GO:0051246 | regulation of protein metabolic process | 0.121362 | 0.00001 |
| GO:0032555 | purine ribonucleotide binding | 0.111111 | 0.00001 |
| GO:0065009 | regulation of molecular function | 0.120528 | 0.00001 |
| GO:0031325 | positive regulation of cellular metabolic process | 0.120156 | 0.00001 |
| GO:1902531 | regulation of intracellular signal transduction | 0.117925 | 0.00001 |
| GO:0070887 | cellular response to chemical stimulus | 0.118032 | 0.00001 |
| GO:0010646 | regulation of cell communication | 0.129765 | 0.00001 |
| GO:0042327 | positive regulation of phosphorylation | 0.1325 | 0.00001 |
| GO:0051641 | cellular localization | 0.130486 | 0.00001 |
| GO:0019439 | aromatic compound catabolic process | 0.156915 | 0.00001 |
| GO:0016787 | hydrolase activity | 0.123777 | 0.00001 |
| GO:0023056 | positive regulation of signaling | 0.115891 | 0.00001 |
| GO:0051239 | regulation of multicellular organismal process | 0.13016 | 0.00001 |
| GO:0009056 | catabolic process | 0.120356 | 0.00001 |
| GO:0019899 | enzyme binding | 0.124111 | 0.00001 |
| GO:0051128 | regulation of cellular component organization | 0.132559 | 0.00001 |
| GO:0006996 | organelle organization | 0.120953 | 0.00001 |
| GO:0050793 | regulation of developmental process | 0.125286 | 0.00001 |
| GO:0070727 | cellular macromolecule localization | 0.134244 | 0.00001 |
| GO:0030031 | cell projection assembly | 0.15896 | 0.00001 |
| GO:0009967 | positive regulation of signal transduction | 0.118613 | 0.00001 |
| GO:0010647 | positive regulation of cell communication | 0.116421 | 0.00001 |
| GO:0008104 | protein localization | 0.134497 | 0.00001 |
| GO:0032553 | ribonucleotide binding | 0.111367 | 0.00001 |
| GO:0017076 | purine nucleotide binding | 0.111075 | 0.00001 |
| GO:0098660 | inorganic ion transmembrane transport | 0.153664 | 0.00001 |
| GO:0005615 | extracellular space | 0.12897 | 0.00001 |
| GO:0043068 | positive regulation of programmed cell death | 0.141844 | 0.00001 |
| GO:1901575 | organic substance catabolic process | 0.118852 | 0.00001 |
| GO:0006796 | phosphate-containing compound metabolic process | 0.11252 | 0.00001 |
| GO:0005102 | signaling receptor binding | 0.126819 | 0.00001 |
| GO:0023052 | signaling | 0.149451 | 0.00001 |
| GO:0035456 | response to interferon-beta | 0.263889 | 0.00001 |
| GO:0043523 | regulation of neuron apoptotic process | 0.181395 | 0.00001 |
| GO:0071702 | organic substance transport | 0.131041 | 0.00001 |
| GO:0044281 | small molecule metabolic process | 0.136742 | 0.00001 |
| GO:0098796 | membrane protein complex | 0.128744 | 0.00001 |
| GO:0015075 | ion transmembrane transporter activity | 0.126177 | 0.00001 |
| GO:0032102 | negative regulation of response to external stimulus | 0.162791 | 0.00001 |
| GO:0030154 | cell differentiation | 0.120824 | 0.00001 |
| GO:0042802 | identical protein binding | 0.126688 | 0.00001 |
| GO:0042596 | fear response | 0.234694 | 0.00001 |
| GO:0031982 | vesicle | 0.140413 | 0.00001 |
| GO:0033554 | cellular response to stress | 0.127797 | 0.00001 |
| GO:0045862 | positive regulation of proteolysis | 0.16035 | 0.00001 |
| GO:1902680 | positive regulation of RNA biosynthetic process | 0.124166 | 0.00001 |
| GO:0120025 | plasma membrane bounded cell projection | 0.153575 | 0.00001 |
| GO:0033036 | macromolecule localization | 0.134112 | 0.00001 |
| GO:0045893 | positive regulation of DNA-templated transcription | 0.124248 | 0.00001 |
| GO:1903508 | positive regulation of nucleic acid-templated transcription | 0.124248 | 0.00001 |
| GO:0010557 | positive regulation of macromolecule biosynthetic process | 0.12305 | 0.00001 |
| GO:0016772 | transferase activity, transferring phosphorus-containing groups | 0.119686 | 0.00001 |
| GO:0022890 | inorganic cation transmembrane transporter activity | 0.136126 | 0.00001 |
| GO:0031410 | cytoplasmic vesicle | 0.145774 | 0.00001 |
| GO:0032879 | regulation of localization | 0.140754 | 0.00001 |
| GO:0006954 | inflammatory response | 0.148454 | 0.00001 |
| GO:0031328 | positive regulation of cellular biosynthetic process | 0.126738 | 0.00001 |
| GO:0044248 | cellular catabolic process | 0.133905 | <0.00001 |
| GO:0010648 | negative regulation of cell communication | 0.14574 | <0.00001 |
| GO:0048584 | positive regulation of response to stimulus | 0.122604 | <0.00001 |
| GO:0051049 | regulation of transport | 0.141921 | <0.00001 |
| GO:0071705 | nitrogen compound transport | 0.128763 | <0.00001 |
| GO:0045595 | regulation of cell differentiation | 0.11889 | <0.00001 |
| GO:0042995 | cell projection | 0.151675 | <0.00001 |
| GO:0044093 | positive regulation of molecular function | 0.125483 | <0.00001 |
| GO:0031399 | regulation of protein modification process | 0.125926 | <0.00001 |
| GO:0044877 | protein-containing complex binding | 0.123632 | <0.00001 |
| GO:0005739 | mitochondrion | 0.155515 | <0.00001 |
| GO:2000026 | regulation of multicellular organismal development | 0.13405 | <0.00001 |
| GO:1901700 | response to oxygen-containing compound | 0.135965 | <0.00001 |
| GO:0045935 | positive regulation of nucleobase-containing compound metabolic process | 0.119764 | <0.00001 |
| GO:0051649 | establishment of localization in cell | 0.126745 | <0.00001 |
| GO:0022857 | transmembrane transporter activity | 0.129916 | <0.00001 |
| GO:0046914 | transition metal ion binding | 0.133939 | <0.00001 |
| GO:0051051 | negative regulation of transport | 0.150215 | <0.00001 |
| GO:0007166 | cell surface receptor signaling pathway | 0.123971 | <0.00001 |
| GO:0019752 | carboxylic acid metabolic process | 0.126547 | <0.00001 |
| GO:0043065 | positive regulation of apoptotic process | 0.144144 | <0.00001 |
| GO:0032535 | regulation of cellular component size | 0.162577 | <0.00001 |
| GO:0001934 | positive regulation of protein phosphorylation | 0.135685 | <0.00001 |
| GO:0035966 | response to topologically incorrect protein | 0.288136 | <0.00001 |
| GO:0098588 | bounding membrane of organelle | 0.147826 | <0.00001 |
| GO:0006629 | lipid metabolic process | 0.183873 | <0.00001 |
| GO:0046983 | protein dimerization activity | 0.129702 | <0.00001 |
| GO:0008270 | zinc ion binding | 0.126736 | <0.00001 |
| GO:0048585 | negative regulation of response to stimulus | 0.145212 | <0.00001 |
| GO:0051174 | regulation of phosphorus metabolic process | 0.126522 | <0.00001 |
| GO:0005215 | transporter activity | 0.13303 | <0.00001 |
| GO:0035556 | intracellular signal transduction | 0.12 | <0.00001 |
| GO:0019220 | regulation of phosphate metabolic process | 0.126608 | <0.00001 |
| GO:0080134 | regulation of response to stress | 0.145455 | <0.00001 |
| GO:0009968 | negative regulation of signal transduction | 0.144159 | <0.00001 |
| GO:0005783 | endoplasmic reticulum | 0.169731 | <0.00001 |
| GO:0009891 | positive regulation of biosynthetic process | 0.126042 | <0.00001 |
| GO:0045454 | cell redox homeostasis | 0.267606 | <0.00001 |
| GO:0051254 | positive regulation of RNA metabolic process | 0.121564 | <0.00001 |
| GO:0002209 | behavioral defense response | 0.237113 | <0.00001 |
| GO:0044270 | cellular nitrogen compound catabolic process | 0.161932 | <0.00001 |
| GO:0120254 | olefinic compound metabolic process | 0.206667 | <0.00001 |
| GO:0043436 | oxoacid metabolic process | 0.126359 | <0.00001 |
| GO:1901565 | organonitrogen compound catabolic process | 0.132794 | <0.00001 |
| GO:0009653 | anatomical structure morphogenesis | 0.132184 | <0.00001 |
| GO:0005576 | extracellular region | 0.128272 | <0.00001 |
| GO:0120036 | plasma membrane bounded cell projection organization | 0.150183 | <0.00001 |
| GO:0005815 | microtubule organizing center | 0.146769 | <0.00001 |
| GO:0031401 | positive regulation of protein modification process | 0.14052 | <0.00001 |
| GO:0042803 | protein homodimerization activity | 0.156882 | <0.00001 |
| GO:0015031 | protein transport | 0.130156 | <0.00001 |
| GO:1901617 | organic hydroxy compound biosynthetic process | 0.185714 | <0.00001 |
| GO:0045937 | positive regulation of phosphate metabolic process | 0.131757 | <0.00001 |
| GO:0010562 | positive regulation of phosphorus metabolic process | 0.131757 | <0.00001 |
| GO:0048513 | animal organ development | 0.134085 | <0.00001 |
| GO:0051247 | positive regulation of protein metabolic process | 0.143484 | <0.00001 |
| GO:0043005 | neuron projection | 0.158364 | <0.00001 |
| GO:0005794 | Golgi apparatus | 0.140555 | <0.00001 |
| GO:0097708 | intracellular vesicle | 0.146093 | <0.00001 |
| GO:0046700 | heterocycle catabolic process | 0.159544 | <0.00001 |
| GO:0098590 | plasma membrane region | 0.130573 | <0.00001 |
| GO:0001932 | regulation of protein phosphorylation | 0.128136 | <0.00001 |
| GO:0008324 | cation transmembrane transporter activity | 0.135135 | <0.00001 |
| GO:0043408 | regulation of MAPK cascade | 0.1408 | <0.00001 |
| GO:0140097 | catalytic activity, acting on DNA | 0.153285 | <0.00001 |
| GO:0016491 | oxidoreductase activity | 0.128099 | <0.00001 |
| GO:0023057 | negative regulation of signaling | 0.145306 | <0.00001 |
| GO:0120031 | plasma membrane bounded cell projection assembly | 0.161194 | <0.00001 |
| GO:0051130 | positive regulation of cellular component organization | 0.129715 | <0.00001 |
| GO:0050890 | cognition | 0.166144 | <0.00001 |
| GO:0002683 | negative regulation of immune system process | 0.15736 | <0.00001 |
| GO:0042325 | regulation of phosphorylation | 0.128 | <0.00001 |
| GO:0045184 | establishment of protein localization | 0.128773 | <0.00001 |
| GO:0030658 | transport vesicle membrane | 0.217742 | <0.00001 |
| GO:0046907 | intracellular transport | 0.132646 | <0.00001 |
| GO:0006082 | organic acid metabolic process | 0.126931 | <0.00001 |
| GO:0014069 | postsynaptic density | 0.155216 | <0.00001 |
| GO:0072657 | protein localization to membrane | 0.15566 | <0.00001 |
| GO:0016788 | hydrolase activity, acting on ester bonds | 0.138554 | <0.00001 |
| GO:0006886 | intracellular protein transport | 0.13769 | <0.00001 |
| GO:0031966 | mitochondrial membrane | 0.154026 | <0.00001 |
| GO:0043085 | positive regulation of catalytic activity | 0.129515 | <0.00001 |
| GO:0016192 | vesicle-mediated transport | 0.139848 | <0.00001 |
| GO:0055085 | transmembrane transport | 0.149746 | <0.00001 |
| GO:0006811 | ion transport | 0.134158 | <0.00001 |
| GO:0051241 | negative regulation of multicellular organismal process | 0.161885 | <0.00001 |
| GO:0014070 | response to organic cyclic compound | 0.134241 | <0.00001 |
| GO:0042592 | homeostatic process | 0.132983 | <0.00001 |
| GO:0044087 | regulation of cellular component biogenesis | 0.13775 | <0.00001 |
| GO:0022603 | regulation of anatomical structure morphogenesis | 0.139344 | <0.00001 |
| GO:0097494 | regulation of vesicle size | 0.28125 | <0.00001 |
| GO:0019637 | organophosphate metabolic process | 0.126294 | <0.00001 |
| GO:0051260 | protein homooligomerization | 0.153318 | <0.00001 |
| GO:0022804 | active transmembrane transporter activity | 0.150763 | <0.00001 |
| GO:0005768 | endosome | 0.14305 | <0.00001 |
| GO:0009894 | regulation of catabolic process | 0.140295 | <0.00001 |
| GO:0051345 | positive regulation of hydrolase activity | 0.144643 | <0.00001 |
| GO:0033993 | response to lipid | 0.137405 | <0.00001 |
| GO:0030427 | site of polarized growth | 0.176692 | <0.00001 |
| GO:0009975 | cyclase activity | 0.423077 | <0.00001 |
| GO:0009187 | cyclic nucleotide metabolic process | 0.341463 | <0.00001 |
| GO:0098815 | modulation of excitatory postsynaptic potential | 0.341463 | <0.00001 |
| GO:0030003 | cellular cation homeostasis | 0.16 | <0.00001 |
| GO:1901362 | organic cyclic compound biosynthetic process | 0.129661 | <0.00001 |
| GO:0032880 | regulation of protein localization | 0.157542 | <0.00001 |
| GO:0006873 | cellular ion homeostasis | 0.158974 | <0.00001 |
| GO:0055080 | cation homeostasis | 0.153518 | <0.00001 |
| GO:0034220 | ion transmembrane transport | 0.147217 | <0.00001 |
| GO:0000139 | Golgi membrane | 0.156194 | <0.00001 |
| GO:0040012 | regulation of locomotion | 0.149841 | <0.00001 |
| GO:0032101 | regulation of response to external stimulus | 0.153415 | <0.00001 |
| GO:2000145 | regulation of cell motility | 0.148936 | <0.00001 |
| GO:0005506 | iron ion binding | 0.166667 | <0.00001 |
| GO:0030001 | metal ion transport | 0.154676 | <0.00001 |
| GO:0051668 | localization within membrane | 0.155378 | <0.00001 |
| GO:0010243 | response to organonitrogen compound | 0.138067 | <0.00001 |
| GO:1901361 | organic cyclic compound catabolic process | 0.159601 | <0.00001 |
| GO:0043009 | chordate embryonic development | 0.170213 | <0.00001 |
| GO:0030334 | regulation of cell migration | 0.150474 | <0.00001 |
| GO:0033043 | regulation of organelle organization | 0.137615 | <0.00001 |
| GO:0005773 | vacuole | 0.156682 | <0.00001 |
| GO:0098771 | inorganic ion homeostasis | 0.153209 | <0.00001 |
| GO:0031644 | regulation of nervous system process | 0.205128 | <0.00001 |
| GO:0010035 | response to inorganic substance | 0.143519 | <0.00001 |
| GO:0030426 | growth cone | 0.179389 | <0.00001 |
| GO:0044255 | cellular lipid metabolic process | 0.188406 | <0.00001 |
| GO:0030030 | cell projection organization | 0.147974 | <0.00001 |
| GO:0031301 | integral component of organelle membrane | 0.175202 | <0.00001 |
| GO:0000302 | response to reactive oxygen species | 0.2 | <0.00001 |
| GO:0008610 | lipid biosynthetic process | 0.183908 | <0.00001 |
| GO:0048878 | chemical homeostasis | 0.142857 | <0.00001 |
| GO:0005789 | endoplasmic reticulum membrane | 0.179044 | <0.00001 |
| GO:0090407 | organophosphate biosynthetic process | 0.14723 | <0.00001 |
| GO:0001662 | behavioral fear response | 0.242105 | <0.00001 |
| GO:0032970 | regulation of actin filament-based process | 0.16188 | <0.00001 |
| GO:0060341 | regulation of cellular localization | 0.151244 | <0.00001 |
| GO:0051050 | positive regulation of transport | 0.13822 | <0.00001 |
| GO:0051046 | regulation of secretion | 0.15767 | <0.00001 |
| GO:0006812 | cation transport | 0.151724 | <0.00001 |
| GO:0045017 | glycerolipid biosynthetic process | 0.191589 | <0.00001 |
| GO:0007626 | locomotory behavior | 0.176056 | <0.00001 |
| GO:0007154 | cell communication | 0.141566 | <0.00001 |
| GO:0008021 | synaptic vesicle | 0.207792 | <0.00001 |
| GO:0016054 | organic acid catabolic process | 0.175824 | <0.00001 |
| GO:0051259 | protein complex oligomerization | 0.154206 | <0.00001 |
| GO:0044297 | cell body | 0.16643 | <0.00001 |
| GO:0048646 | anatomical structure formation involved in morphogenesis | 0.140751 | <0.00001 |
| GO:0034114 | regulation of heterotypic cell-cell adhesion | 0.529412 | <0.00001 |
| GO:0010942 | positive regulation of cell death | 0.149758 | <0.00001 |
| GO:0030425 | dendrite | 0.172303 | <0.00001 |
| GO:1901216 | positive regulation of neuron death | 0.25 | <0.00001 |
| GO:0055082 | cellular chemical homeostasis | 0.157895 | <0.00001 |
| GO:0019866 | organelle inner membrane | 0.15493 | <0.00001 |
| GO:0005813 | centrosome | 0.14978 | <0.00001 |
| GO:0019725 | cellular homeostasis | 0.159677 | <0.00001 |
| GO:0050878 | regulation of body fluid levels | 0.16835 | <0.00001 |
| GO:0051222 | positive regulation of protein transport | 0.175084 | <0.00001 |
| GO:0098655 | cation transmembrane transport | 0.163017 | <0.00001 |
| GO:0007610 | behavior | 0.15 | <0.00001 |
| GO:0040008 | regulation of growth | 0.14433 | <0.00001 |
| GO:0070925 | organelle assembly | 0.147766 | <0.00001 |
| GO:0030155 | regulation of cell adhesion | 0.161442 | <0.00001 |
| GO:0062012 | regulation of small molecule metabolic process | 0.166189 | <0.00001 |
| GO:0016849 | phosphorus-oxygen lyase activity | 0.4 | <0.00001 |
| GO:0051493 | regulation of cytoskeleton organization | 0.152294 | <0.00001 |
| GO:0005759 | mitochondrial matrix | 0.172078 | <0.00001 |
| GO:0008654 | phospholipid biosynthetic process | 0.178988 | <0.00001 |
| GO:0045926 | negative regulation of growth | 0.186235 | <0.00001 |
| GO:1903530 | regulation of secretion by cell | 0.160194 | <0.00001 |
| GO:0016050 | vesicle organization | 0.180812 | <0.00001 |
| GO:0006790 | sulfur compound metabolic process | 0.184524 | <0.00001 |
| GO:0090066 | regulation of anatomical structure size | 0.165909 | <0.00001 |
| GO:0030424 | axon | 0.179389 | <0.00001 |
| GO:0046486 | glycerolipid metabolic process | 0.195592 | <0.00001 |
| GO:0005743 | mitochondrial inner membrane | 0.1609 | <0.00001 |
| GO:0046395 | carboxylic acid catabolic process | 0.177778 | <0.00001 |
| GO:0043025 | neuronal cell body | 0.169463 | <0.00001 |
| GO:0007275 | multicellular organism development | 0.161137 | <0.00001 |
| GO:0062023 | collagen-containing extracellular matrix | 0.220994 | <0.00001 |
| GO:0006631 | fatty acid metabolic process | 0.196286 | <0.00001 |
| GO:0001701 | in utero embryonic development | 0.175 | <0.00001 |
| GO:1903829 | positive regulation of protein localization | 0.15859 | <0.00001 |
| GO:0009790 | embryo development | 0.17052 | <0.00001 |
| GO:0009792 | embryo development ending in birth or egg hatching | 0.17052 | <0.00001 |
| GO:0090087 | regulation of peptide transport | 0.216216 | <0.00001 |
| GO:0046906 | tetrapyrrole binding | 0.201365 | <0.00001 |
| GO:0051223 | regulation of protein transport | 0.173507 | <0.00001 |
| GO:0010817 | regulation of hormone levels | 0.171642 | <0.00001 |
| GO:1904951 | positive regulation of establishment of protein localization | 0.172414 | <0.00001 |
| GO:0005774 | vacuolar membrane | 0.184211 | <0.00001 |
| GO:0002791 | regulation of peptide secretion | 0.217195 | <0.00001 |
| GO:0070201 | regulation of establishment of protein localization | 0.171629 | <0.00001 |
| GO:1901615 | organic hydroxy compound metabolic process | 0.179884 | <0.00001 |
| GO:0006979 | response to oxidative stress | 0.188544 | <0.00001 |
| GO:0030312 | external encapsulating structure | 0.188797 | <0.00001 |
| GO:0044309 | neuron spine | 0.204545 | <0.00001 |
| GO:0044057 | regulation of system process | 0.162921 | <0.00001 |
| GO:0007528 | neuromuscular junction development | 0.247312 | <0.00001 |
| GO:0016042 | lipid catabolic process | 0.20132 | <0.00001 |
| GO:0030258 | lipid modification | 0.20625 | <0.00001 |
| GO:0007034 | vacuolar transport | 0.190955 | <0.00001 |
| GO:0034976 | response to endoplasmic reticulum stress | 0.19598 | <0.00001 |
| GO:0006650 | glycerophospholipid metabolic process | 0.179931 | <0.00001 |
| GO:0031012 | extracellular matrix | 0.189189 | <0.00001 |
| GO:0010038 | response to metal ion | 0.199005 | <0.00001 |
| GO:0000323 | lytic vacuole | 0.168067 | <0.00001 |
| GO:0005764 | lysosome | 0.168067 | <0.00001 |
| GO:0090276 | regulation of peptide hormone secretion | 0.215596 | <0.00001 |
| GO:0050801 | ion homeostasis | 0.15493 | <0.00001 |
| GO:0098852 | lytic vacuole membrane | 0.193237 | <0.00001 |
| GO:0098794 | postsynapse | 0.195719 | <0.00001 |
| GO:0051592 | response to calcium ion | 0.234177 | <0.00001 |
| GO:0038024 | cargo receptor activity | 0.255814 | <0.00001 |
| GO:0006638 | neutral lipid metabolic process | 0.252525 | <0.00001 |
| GO:0016705 | oxidoreductase activity, acting on paired donors, with incorporation or reduction of molecular oxygen | 0.202658 | <0.00001 |
| GO:0043197 | dendritic spine | 0.2 | <0.00001 |
| GO:0006066 | alcohol metabolic process | 0.206522 | <0.00001 |
| GO:0046474 | glycerophospholipid biosynthetic process | 0.192893 | <0.00001 |
| GO:0019216 | regulation of lipid metabolic process | 0.171779 | <0.00001 |
| GO:0005765 | lysosomal membrane | 0.18932 | <0.00001 |
| GO:0070382 | exocytic vesicle | 0.204545 | <0.00001 |
| GO:0006644 | phospholipid metabolic process | 0.183246 | <0.00001 |
| GO:0098662 | inorganic cation transmembrane transport | 0.164921 | <0.00001 |
| GO:0030672 | synaptic vesicle membrane | 0.273973 | <0.00001 |
| GO:0099501 | exocytic vesicle membrane | 0.273973 | <0.00001 |
| GO:0046883 | regulation of hormone secretion | 0.189781 | <0.00001 |
| GO:0030133 | transport vesicle | 0.196 | <0.00001 |
| GO:0031300 | intrinsic component of organelle membrane | 0.176039 | <0.00001 |
| GO:0005085 | guanyl-nucleotide exchange factor activity | 0.173375 | <0.00001 |
| GO:0006639 | acylglycerol metabolic process | 0.255102 | <0.00001 |
| GO:0006575 | cellular modified amino acid metabolic process | 0.208333 | <0.00001 |
| GO:0001889 | liver development | 0.224 | <0.00001 |
| GO:0007584 | response to nutrient | 0.212121 | <0.00001 |
| GO:0001726 | ruffle | 0.236364 | <0.00001 |
| GO:0007032 | endosome organization | 0.243243 | <0.00001 |
| GO:0004497 | monooxygenase activity | 0.200893 | <0.00001 |
| GO:0050708 | regulation of protein secretion | 0.195035 | <0.00001 |
| GO:0020037 | heme binding | 0.205674 | <0.00001 |
| GO:0006805 | xenobiotic metabolic process | 0.215686 | <0.00001 |
| GO:0007599 | hemostasis | 0.291667 | <0.00001 |
| GO:1903035 | negative regulation of response to wounding | 0.3 | <0.00001 |
| GO:0140534 | endoplasmic reticulum protein-containing complex | 0.257669 | <0.00001 |
| GO:0050818 | regulation of coagulation | 0.388889 | <0.00001 |
| GO:0016126 | sterol biosynthetic process | 0.314815 | <0.00001 |
| GO:0008202 | steroid metabolic process | 0.191406 | <0.00001 |
| GO:0015399 | primary active transmembrane transporter activity | 0.1875 | <0.00001 |
| GO:0050796 | regulation of insulin secretion | 0.211111 | <0.00001 |
| GO:0016125 | sterol metabolic process | 0.246154 | <0.00001 |
| GO:0033555 | multicellular organismal response to stress | 0.227642 | <0.00001 |
| GO:0015453 | oxidoreduction-driven active transmembrane transporter activity | 0.232759 | <0.00001 |
| GO:1900046 | regulation of hemostasis | 0.396226 | <0.00001 |
| GO:0034308 | primary alcohol metabolic process | 0.258427 | <0.00001 |
| GO:0006694 | steroid biosynthetic process | 0.24031 | <0.00001 |
| GO:0008203 | cholesterol metabolic process | 0.237705 | <0.00001 |
| GO:0030195 | negative regulation of blood coagulation | 0.432432 | <0.00001 |
| GO:0055114 | obsolete oxidation-reduction process | 0.22 | <0.00001 |
| GO:1903034 | regulation of response to wounding | 0.234783 | <0.00001 |
| GO:0006656 | phosphatidylcholine biosynthetic process | 0.380952 | <0.00001 |
| GO:0050817 | coagulation | 0.3 | <0.00001 |
| GO:0007596 | blood coagulation | 0.3 | <0.00001 |
| GO:0061045 | negative regulation of wound healing | 0.361702 | <0.00001 |
| GO:0044242 | cellular lipid catabolic process | 0.243243 | <0.00001 |
| GO:0046890 | regulation of lipid biosynthetic process | 0.205405 | <0.00001 |
| GO:0009055 | electron transfer activity | 0.221622 | <0.00001 |
| GO:0042542 | response to hydrogen peroxide | 0.226563 | <0.00001 |
| GO:0061041 | regulation of wound healing | 0.272727 | <0.00001 |
| GO:0030193 | regulation of blood coagulation | 0.403846 | <0.00001 |
| GO:0002526 | acute inflammatory response | 0.36 | <0.00001 |
| GO:1902652 | secondary alcohol metabolic process | 0.238806 | <0.00001 |
| GO:0035249 | synaptic transmission, glutamatergic | 0.271605 | <0.00001 |
| GO:0046470 | phosphatidylcholine metabolic process | 0.323529 | <0.00001 |
| GO:0051775 | response to redox state | 0.727273 | <0.00001 |
| GO:0050819 | negative regulation of coagulation | 0.410256 | <0.00001 |
| GO:1900047 | negative regulation of hemostasis | 0.421053 | <0.00001 |
| GO:0006953 | acute-phase response | 0.552632 | <0.00001 |
| GO:0042730 | fibrinolysis | 0.692308 | <0.00001 |

Rich factor: The ratio of the number of genes belonging to this Term in the target gene set to the number of all genes belonging to this Term in the background gene set.
