# Supplementary material for: Multi-time point transcriptomics and metabolomics reveal key transcription and metabolic features of hepatic ischemia-reperfusion injury in mice
Source: Genes Dis. 2024 Nov 17;12(2):101465. doi: 10.1016/j.gendis.2024.101465 (PMC11697123; doi:10.1016/j.gendis.2024.101465)
Supplement: Multimedia component 7 [file mmc7.docx]

**Table S3A.** The KEGG pathway of differentially expressed genes (DEGs) identified by Kyoto Encyclopedia of Genes and Genomes (KEGG) database in the Sham and I1R12 groups.

| **Pathway ID** | **pathway description** | **Rich factor** | **P-value** |
| --- | --- | --- | --- |
| mmu04740 | Olfactory transduction | 0.021661 | 0.99999 |
| mmu05034 | Alcoholism | 0.023529 | 0.99975 |
| mmu04666 | Fc gamma R-mediated phagocytosis | 0.010753 | 0.99971 |
| mmu05226 | Gastric cancer | 0.027397 | 0.99865 |
| mmu05211 | Renal cell carcinoma | 0.014706 | 0.99741 |
| mmu04650 | Natural killer cell mediated cytotoxicity | 0.027027 | 0.99629 |
| mmu04080 | Neuroactive ligand-receptor interaction | 0.05 | 0.99608 |
| mmu04014 | Ras signaling pathway | 0.043103 | 0.99491 |
| mmu05213 | Endometrial cancer | 0.017241 | 0.99378 |
| mmu04390 | Hippo signaling pathway | 0.03871 | 0.99159 |
| mmu04070 | Phosphatidylinositol signaling system | 0.03125 | 0.98933 |
| mmu05220 | Chronic myeloid leukemia | 0.026667 | 0.98897 |
| mmu05214 | Glioma | 0.027397 | 0.98716 |
| mmu04072 | Phospholipase D signaling pathway | 0.040816 | 0.98668 |
| mmu05210 | Colorectal cancer | 0.033708 | 0.98275 |
| mmu04664 | Fc epsilon RI signaling pathway | 0.029851 | 0.97982 |
| mmu04722 | Neurotrophin signaling pathway | 0.041667 | 0.97654 |
| mmu04012 | ErbB signaling pathway | 0.035714 | 0.97583 |
| mmu05231 | Choline metabolism in cancer | 0.040816 | 0.96887 |
| mmu04662 | B cell receptor signaling pathway | 0.037975 | 0.96633 |
| mmu05224 | Breast cancer | 0.047945 | 0.96600 |
| mmu05216 | Thyroid cancer | 0.027027 | 0.96078 |
| mmu04340 | Hedgehog signaling pathway | 0.035088 | 0.95771 |
| mmu04614 | Renin-angiotensin system | 0.027778 | 0.95718 |
| mmu05410 | Hypertrophic cardiomyopathy | 0.044444 | 0.94984 |
| mmu05144 | Malaria | 0.037037 | 0.94742 |
| mmu05223 | Non-small cell lung cancer | 0.041667 | 0.94699 |
| mmu05218 | Melanoma | 0.041667 | 0.94699 |
| mmu04360 | Axon guidance | 0.054945 | 0.94613 |
| mmu04660 | T cell receptor signaling pathway | 0.048077 | 0.94267 |
| mmu04916 | Melanogenesis | 0.049505 | 0.93273 |
| mmu05031 | Amphetamine addiction | 0.044118 | 0.93171 |
| mmu04728 | Dopaminergic synapse | 0.053435 | 0.92956 |
| mmu05322 | Systemic lupus erythematosus | 0.052174 | 0.92702 |
| mmu04720 | Long-term potentiation | 0.045455 | 0.92263 |
| mmu03013 | Nucleocytoplasmic transport | 0.053097 | 0.91954 |
| mmu05030 | Cocaine addiction | 0.041667 | 0.91926 |
| mmu05132 | Salmonella infection | 0.061728 | 0.91876 |
| mmu04380 | Osteoclast differentiation | 0.055118 | 0.91530 |
| mmu04150 | mTOR signaling pathway | 0.057692 | 0.91360 |
| mmu04810 | Regulation of actin cytoskeleton | 0.061674 | 0.91294 |
| mmu03015 | mRNA surveillance pathway | 0.052632 | 0.90813 |
| mmu05217 | Basal cell carcinoma | 0.047619 | 0.90693 |
| mmu04623 | Cytosolic DNA-sensing pathway | 0.048387 | 0.90109 |
| mmu05100 | Bacterial invasion of epithelial cells | 0.052632 | 0.88950 |
| mmu05212 | Pancreatic cancer | 0.052632 | 0.88950 |
| mmu05310 | Asthma | 0.04 | 0.88778 |
| mmu00514 | Other types of O-glycan biosynthesis | 0.046512 | 0.88548 |
| mmu04919 | Thyroid hormone signaling pathway | 0.058333 | 0.88434 |
| mmu05150 | Staphylococcus aureus infection | 0.059322 | 0.87392 |
| mmu03040 | Spliceosome | 0.060606 | 0.87208 |
| mmu05320 | Autoimmune thyroid disease | 0.056338 | 0.85587 |
| mmu00562 | Inositol phosphate metabolism | 0.056338 | 0.85587 |
| mmu04926 | Relaxin signaling pathway | 0.062016 | 0.85574 |
| mmu04540 | Gap junction | 0.058824 | 0.84963 |
| mmu05140 | Leishmaniasis | 0.057143 | 0.84819 |
| mmu04960 | Aldosterone-regulated sodium reabsorption | 0.052632 | 0.83900 |
| mmu04725 | Cholinergic synapse | 0.0625 | 0.83793 |
| mmu04625 | C-type lectin receptor signaling pathway | 0.0625 | 0.83793 |
| mmu04140 | Autophagy - animal | 0.064748 | 0.83239 |
| mmu04110 | Cell cycle | 0.064 | 0.83141 |
| mmu04530 | Tight junction | 0.066265 | 0.83023 |
| mmu00532 | Glycosaminoglycan biosynthesis - chondroitin sulfate / dermatan sulfate | 0.05 | 0.82614 |
| mmu00770 | Pantothenate and CoA biosynthesis | 0.05 | 0.82614 |
| mmu04024 | cAMP signaling pathway | 0.068493 | 0.82613 |
| mmu05161 | Hepatitis B | 0.066667 | 0.82447 |
| mmu04350 | TGF-beta signaling pathway | 0.063158 | 0.81711 |
| mmu05143 | African trypanosomiasis | 0.055556 | 0.81602 |
| mmu04210 | Apoptosis | 0.066176 | 0.81323 |
| mmu02010 | ABC transporters | 0.058824 | 0.81170 |
| mmu03018 | RNA degradation | 0.0625 | 0.81029 |
| mmu04929 | GnRH secretion | 0.061538 | 0.80441 |
| mmu05414 | Dilated cardiomyopathy | 0.064516 | 0.80105 |
| mmu04071 | Sphingolipid signaling pathway | 0.066667 | 0.79661 |
| mmu04914 | Progesterone-mediated oocyte maturation | 0.065217 | 0.79262 |
| mmu04710 | Circadian rhythm | 0.058824 | 0.79015 |
| mmu00030 | Pentose phosphate pathway | 0.060606 | 0.77604 |
| mmu04120 | Ubiquitin mediated proteolysis | 0.069444 | 0.77500 |
| mmu04940 | Type I diabetes mellitus | 0.064516 | 0.77354 |
| mmu05412 | Arrhythmogenic right ventricular cardiomyopathy | 0.065789 | 0.77324 |
| mmu04211 | Longevity regulating pathway | 0.067416 | 0.76570 |
| mmu04213 | Longevity regulating pathway - multiple species | 0.065574 | 0.76243 |
| mmu00512 | Mucin type O-glycan biosynthesis | 0.0625 | 0.76112 |
| mmu00020 | Citrate cycle (TCA cycle) | 0.0625 | 0.76112 |
| mmu00603 | Glycosphingolipid biosynthesis - globo and isoglobo series | 0.0625 | 0.75326 |
| mmu04730 | Long-term depression | 0.066667 | 0.75090 |
| mmu04136 | Autophagy - other | 0.064516 | 0.74534 |
| mmu04620 | Toll-like receptor signaling pathway | 0.070707 | 0.73257 |
| mmu04520 | Adherens junction | 0.069444 | 0.73090 |
| mmu00604 | Glycosphingolipid biosynthesis - ganglio series | 0.066667 | 0.73070 |
| mmu04370 | VEGF signaling pathway | 0.068966 | 0.72657 |
| mmu04713 | Circadian entrainment | 0.071429 | 0.72281 |
| mmu05160 | Hepatitis C | 0.07362 | 0.72062 |
| mmu04015 | Rap1 signaling pathway | 0.074766 | 0.71832 |
| mmu04371 | Apelin signaling pathway | 0.073529 | 0.71128 |
| mmu05135 | Yersinia infection | 0.073529 | 0.71128 |
| mmu03420 | Nucleotide excision repair | 0.069767 | 0.70989 |
| mmu04622 | RIG-I-like receptor signaling pathway | 0.071429 | 0.70768 |
| mmu04137 | Mitophagy - animal | 0.072464 | 0.69556 |
| mmu05330 | Allograft rejection | 0.072727 | 0.68682 |
| mmu05332 | Graft-versus-host disease | 0.072727 | 0.68682 |
| mmu05416 | Viral myocarditis | 0.074074 | 0.68158 |
| mmu05167 | Kaposi sarcoma-associated herpesvirus infection | 0.077626 | 0.66388 |
| mmu00601 | Glycosphingolipid biosynthesis - lacto and neolacto series | 0.076923 | 0.65255 |
| mmu05168 | Herpes simplex virus 1 infection | 0.080092 | 0.63468 |
| mmu01521 | EGFR tyrosine kinase inhibitor resistance | 0.077922 | 0.63285 |
| mmu04020 | Calcium signaling pathway | 0.079498 | 0.62788 |
| mmu04921 | Oxytocin signaling pathway | 0.078947 | 0.62711 |
| mmu04144 | Endocytosis | 0.080153 | 0.61580 |
| mmu05202 | Transcriptional misregulation in cancer | 0.08 | 0.61299 |
| mmu00900 | Terpenoid backbone biosynthesis | 0.083333 | 0.60850 |
| mmu04977 | Vitamin digestion and absorption | 0.083333 | 0.60850 |
| mmu04218 | Cellular senescence | 0.08046 | 0.60189 |
| mmu05321 | Inflammatory bowel disease | 0.080645 | 0.60136 |
| mmu04721 | Synaptic vesicle cycle | 0.081081 | 0.59359 |
| mmu05170 | Human immunodeficiency virus 1 infection | 0.081197 | 0.59028 |
| mmu04961 | Endocrine and other factor-regulated calcium reabsorption | 0.081967 | 0.58663 |
| mmu05164 | Influenza A | 0.081395 | 0.58414 |
| mmu00130 | Ubiquinone and other terpenoid-quinone biosynthesis | 0.1 | 0.58291 |
| mmu05215 | Prostate cancer | 0.081633 | 0.58274 |
| mmu05225 | Hepatocellular carcinoma | 0.081871 | 0.57515 |
| mmu04010 | MAPK signaling pathway | 0.082192 | 0.56882 |
| mmu04142 | Lysosome | 0.082707 | 0.56234 |
| mmu04915 | Estrogen signaling pathway | 0.082707 | 0.56234 |
| mmu05162 | Measles | 0.082759 | 0.56029 |
| mmu04151 | PI3K-Akt signaling pathway | 0.082621 | 0.55823 |
| mmu03410 | Base excision repair | 0.088235 | 0.55038 |
| mmu03030 | DNA replication | 0.088235 | 0.55038 |
| mmu04068 | FoxO signaling pathway | 0.083969 | 0.54156 |
| mmu05221 | Acute myeloid leukemia | 0.085714 | 0.53800 |
| mmu05152 | Tuberculosis | 0.083799 | 0.53767 |
| mmu04270 | Vascular smooth muscle contraction | 0.084507 | 0.53030 |
| mmu04215 | Apoptosis - multiple species | 0.090909 | 0.52975 |
| mmu04064 | NF-kappa B signaling pathway | 0.085714 | 0.52128 |
| mmu05165 | Human papillomavirus infection | 0.084058 | 0.51931 |
| mmu05163 | Human cytomegalovirus infection | 0.084337 | 0.51918 |
| mmu04912 | GnRH signaling pathway | 0.086957 | 0.50930 |
| mmu04742 | Taste transduction | 0.086957 | 0.50930 |
| mmu04962 | Vasopressin-regulated water reabsorption | 0.090909 | 0.50876 |
| mmu00360 | Phenylalanine metabolism | 0.1 | 0.50806 |
| mmu04122 | Sulfur relay system | 0.125 | 0.50318 |
| mmu00400 | Phenylalanine, tyrosine and tryptophan biosynthesis | 0.125 | 0.50318 |
| mmu04550 | Signaling pathways regulating pluripotency of stem cells | 0.086331 | 0.49969 |
| mmu04672 | Intestinal immune network for IgA production | 0.093023 | 0.49038 |
| mmu04130 | SNARE interactions in vesicular transport | 0.096774 | 0.48696 |
| mmu00670 | One carbon pool by folate | 0.105263 | 0.48044 |
| mmu00330 | Arginine and proline metabolism | 0.092593 | 0.47617 |
| mmu04724 | Glutamatergic synapse | 0.088496 | 0.47561 |
| mmu04613 | Neutrophil extracellular trap formation | 0.087209 | 0.47364 |
| mmu03440 | Homologous recombination | 0.097561 | 0.45281 |
| mmu00511 | Other glycan degradation | 0.111111 | 0.45189 |
| mmu00120 | Primary bile acid biosynthesis | 0.111111 | 0.45189 |
| mmu00061 | Fatty acid biosynthesis | 0.111111 | 0.45189 |
| mmu04924 | Renin secretion | 0.093333 | 0.44023 |
| mmu04216 | Ferroptosis | 0.1 | 0.43368 |
| mmu05033 | Nicotine addiction | 0.1 | 0.43368 |
| mmu04146 | Peroxisome | 0.093023 | 0.43217 |
| mmu03460 | Fanconi anemia pathway | 0.098039 | 0.42586 |
| mmu04114 | Oocyte meiosis | 0.091667 | 0.42273 |
| mmu05205 | Proteoglycans in cancer | 0.089552 | 0.41817 |
| mmu04726 | Serotonergic synapse | 0.091603 | 0.41632 |
| mmu00350 | Tyrosine metabolism | 0.102564 | 0.41437 |
| mmu00470 | D-Amino acid metabolism | 0.166667 | 0.40821 |
| mmu00650 | Butanoate metabolism | 0.111111 | 0.39634 |
| mmu04670 | Leukocyte transendothelial migration | 0.094017 | 0.38981 |
| mmu04920 | Adipocytokine signaling pathway | 0.098592 | 0.38402 |
| mmu04659 | Th17 cell differentiation | 0.095238 | 0.38340 |
| mmu04934 | Cushing syndrome | 0.092593 | 0.38067 |
| mmu05222 | Small cell lung cancer | 0.096774 | 0.37589 |
| mmu00790 | Folate biosynthesis | 0.115385 | 0.37293 |
| mmu00563 | Glycosylphosphatidylinositol (GPI)-anchor biosynthesis | 0.115385 | 0.37293 |
| mmu04966 | Collecting duct acid secretion | 0.115385 | 0.37293 |
| mmu05203 | Viral carcinogenesis | 0.091787 | 0.37175 |
| mmu05206 | MicroRNAs in cancer | 0.093168 | 0.37144 |
| mmu04935 | Growth hormone synthesis, secretion and action | 0.095652 | 0.36795 |
| mmu01522 | Endocrine resistance | 0.097826 | 0.36370 |
| mmu04261 | Adrenergic signaling in cardiomyocytes | 0.094595 | 0.35668 |
| mmu04514 | Cell adhesion molecules | 0.094118 | 0.34959 |
| mmu04933 | AGE-RAGE signaling pathway in diabetic complications | 0.09901 | 0.33760 |
| mmu05230 | Central carbon metabolism in cancer | 0.104478 | 0.32803 |
| mmu04727 | GABAergic synapse | 0.102273 | 0.31553 |
| mmu04260 | Cardiac muscle contraction | 0.102273 | 0.31553 |
| mmu05235 | PD-L1 expression and PD-1 checkpoint pathway in cancer | 0.102273 | 0.31553 |
| mmu05133 | Pertussis | 0.103896 | 0.31534 |
| mmu03008 | Ribosome biogenesis in eukaryotes | 0.105263 | 0.30267 |
| mmu05200 | Pathways in cancer | 0.090573 | 0.30052 |
| mmu00290 | Valine, leucine and isoleucine biosynthesis | 0.25 | 0.29511 |
| mmu05323 | Rheumatoid arthritis | 0.104651 | 0.29197 |
| mmu00600 | Sphingolipid metabolism | 0.113208 | 0.28168 |
| mmu03430 | Mismatch repair | 0.136364 | 0.27840 |
| mmu05017 | Spinocerebellar ataxia | 0.100719 | 0.27368 |
| mmu04022 | cGMP-PKG signaling pathway | 0.098837 | 0.27199 |
| mmu00310 | Lysine degradation | 0.112903 | 0.26022 |
| mmu00760 | Nicotinate and nicotinamide metabolism | 0.121951 | 0.25674 |
| mmu05219 | Bladder cancer | 0.121951 | 0.25674 |
| mmu04917 | Prolactin signaling pathway | 0.111111 | 0.25329 |
| mmu04657 | IL-17 signaling pathway | 0.107527 | 0.24998 |
| mmu00920 | Sulfur metabolism | 0.181818 | 0.23356 |
| mmu04630 | JAK-STAT signaling pathway | 0.101796 | 0.23347 |
| mmu05142 | Chagas disease | 0.107843 | 0.23298 |
| mmu00220 | Arginine biosynthesis | 0.15 | 0.23187 |
| mmu05032 | Morphine addiction | 0.10989 | 0.22938 |
| mmu04152 | AMPK signaling pathway | 0.105691 | 0.22892 |
| mmu04062 | Chemokine signaling pathway | 0.101064 | 0.22692 |
| mmu04510 | Focal adhesion | 0.10101 | 0.22035 |
| mmu03250 | Viral life cycle - HIV-1 | 0.12069 | 0.20928 |
| mmu00250 | Alanine, aspartate and glutamate metabolism | 0.131579 | 0.20918 |
| mmu05145 | Toxoplasmosis | 0.109091 | 0.20879 |
| mmu04923 | Regulation of lipolysis in adipocytes | 0.122807 | 0.19717 |
| mmu04310 | Wnt signaling pathway | 0.105263 | 0.18567 |
| mmu04971 | Gastric acid secretion | 0.12 | 0.17361 |
| mmu04950 | Maturity onset diabetes of the young | 0.153846 | 0.16856 |
| mmu04744 | Phototransduction | 0.153846 | 0.16856 |
| mmu00340 | Histidine metabolism | 0.153846 | 0.16856 |
| mmu04217 | Necroptosis | 0.107143 | 0.16670 |
| mmu00450 | Selenocompound metabolism | 0.176471 | 0.16551 |
| mmu04611 | Platelet activation | 0.112 | 0.16142 |
| mmu00261 | Monobactam biosynthesis | 0.5 | 0.16041 |
| mmu05166 | Human T-cell leukemia virus 1 infection | 0.103734 | 0.15510 |
| mmu04922 | Glucagon signaling pathway | 0.116505 | 0.15186 |
| mmu04978 | Mineral absorption | 0.132075 | 0.15177 |
| mmu00620 | Pyruvate metabolism | 0.139535 | 0.14687 |
| mmu04115 | p53 signaling pathway | 0.125 | 0.14590 |
| mmu00230 | Purine metabolism | 0.112782 | 0.14529 |
| mmu05417 | Lipid and atherosclerosis | 0.105991 | 0.14288 |
| mmu04972 | Pancreatic secretion | 0.118182 | 0.12949 |
| mmu00410 | beta-Alanine metabolism | 0.15625 | 0.12498 |
| mmu00500 | Starch and sucrose metabolism | 0.15625 | 0.12498 |
| mmu04621 | NOD-like receptor signaling pathway | 0.109453 | 0.11765 |
| mmu00564 | Glycerophospholipid metabolism | 0.122449 | 0.11688 |
| mmu00630 | Glyoxylate and dicarboxylate metabolism | 0.16129 | 0.11278 |
| mmu04928 | Parathyroid hormone synthesis, secretion and action | 0.121495 | 0.11073 |
| mmu00430 | Taurine and hypotaurine metabolism | 0.181818 | 0.10672 |
| mmu04964 | Proximal tubule bicarbonate reclamation | 0.181818 | 0.10672 |
| mmu00513 | Various types of N-glycan biosynthesis | 0.153846 | 0.10353 |
| mmu04512 | ECM-receptor interaction | 0.127907 | 0.10314 |
| mmu04911 | Insulin secretion | 0.127907 | 0.10314 |
| mmu00565 | Ether lipid metabolism | 0.145833 | 0.10299 |
| mmu04930 | Type II diabetes mellitus | 0.145833 | 0.10299 |
| mmu05171 | Coronavirus disease - COVID-19 | 0.109705 | 0.09385 |
| mmu04723 | Retrograde endocannabinoid signaling | 0.118056 | 0.09371 |
| mmu00280 | Valine, leucine and isoleucine degradation | 0.142857 | 0.09320 |
| mmu04932 | Non-alcoholic fatty liver disease | 0.116883 | 0.09304 |
| mmu04640 | Hematopoietic cell lineage | 0.12766 | 0.09260 |
| mmu04925 | Aldosterone synthesis and secretion | 0.126214 | 0.08839 |
| mmu04060 | Cytokine-cytokine receptor interaction | 0.107639 | 0.08827 |
| mmu04973 | Carbohydrate digestion and absorption | 0.152174 | 0.08626 |
| mmu04066 | HIF-1 signaling pathway | 0.125 | 0.08428 |
| mmu00100 | Steroid biosynthesis | 0.2 | 0.08054 |
| mmu00062 | Fatty acid elongation | 0.178571 | 0.07991 |
| mmu03050 | Proteasome | 0.155556 | 0.07851 |
| mmu04913 | Ovarian steroidogenesis | 0.142857 | 0.07793 |
| mmu05340 | Primary immunodeficiency | 0.166667 | 0.07614 |
| mmu00561 | Glycerolipid metabolism | 0.145161 | 0.07187 |
| mmu00970 | Aminoacyl-tRNA biosynthesis | 0.159091 | 0.07119 |
| mmu00051 | Fructose and mannose metabolism | 0.171429 | 0.06804 |
| mmu05134 | Legionellosis | 0.147541 | 0.06611 |
| mmu00071 | Fatty acid degradation | 0.153846 | 0.06590 |
| mmu04658 | Th1 and Th2 cell differentiation | 0.136364 | 0.06248 |
| mmu04330 | Notch signaling pathway | 0.15 | 0.06065 |
| mmu00524 | Neomycin, kanamycin and gentamicin biosynthesis | 0.4 | 0.05903 |
| mmu00380 | Tryptophan metabolism | 0.16 | 0.05436 |
| mmu00592 | alpha-Linolenic acid metabolism | 0.2 | 0.05301 |
| mmu05146 | Amoebiasis | 0.134615 | 0.05129 |
| mmu04970 | Salivary secretion | 0.144578 | 0.04299 |
| mmu04061 | Viral protein interaction with cytokine and cytokine receptor | 0.141304 | 0.04244 |
| mmu00240 | Pyrimidine metabolism | 0.160714 | 0.04173 |
| mmu00052 | Galactose metabolism | 0.193548 | 0.04089 |
| mmu04931 | Insulin resistance | 0.138889 | 0.03531 |
| mmu05169 | Epstein-Barr virus infection | 0.120536 | 0.03481 |
| mmu03320 | PPAR signaling pathway | 0.146067 | 0.03361 |
| mmu04750 | Inflammatory mediator regulation of TRP channels | 0.134921 | 0.03361 |
| mmu04612 | Antigen processing and presentation | 0.15 | 0.03358 |
| mmu05022 | Pathways of neurodegeneration - multiple diseases | 0.108742 | 0.03197 |
| mmu00270 | Cysteine and methionine metabolism | 0.169811 | 0.03049 |
| mmu04936 | Alcoholic liver disease | 0.134752 | 0.02606 |
| mmu04668 | TNF signaling pathway | 0.141593 | 0.02593 |
| mmu04145 | Phagosome | 0.130178 | 0.02521 |
| mmu04975 | Fat digestion and absorption | 0.186047 | 0.02450 |
| mmu00510 | N-Glycan biosynthesis | 0.18 | 0.02155 |
| mmu00040 | Pentose and glucuronate interconversions | 0.205882 | 0.02053 |
| mmu04918 | Thyroid hormone synthesis | 0.162162 | 0.01932 |
| mmu04910 | Insulin signaling pathway | 0.139706 | 0.01852 |
| mmu05016 | Huntington disease | 0.120401 | 0.01713 |
| mmu01524 | Platinum drug resistance | 0.160494 | 0.01658 |
| mmu04927 | Cortisol synthesis and secretion | 0.166667 | 0.01577 |
| mmu03450 | Non-homologous end-joining | 0.333333 | 0.01401 |
| mmu04974 | Protein digestion and absorption | 0.152381 | 0.01367 |
| mmu00590 | Arachidonic acid metabolism | 0.164706 | 0.01060 |
| mmu00640 | Propanoate metabolism | 0.233333 | 0.01040 |
| mmu05418 | Fluid shear stress and atherosclerosis | 0.143836 | 0.01007 |
| mmu00053 | Ascorbate and aldarate metabolism | 0.241379 | 0.00858 |
| mmu03010 | Ribosome | 0.150376 | 0.00733 |
| mmu00260 | Glycine, serine and threonine metabolism | 0.214286 | 0.00698 |
| mmu00520 | Amino sugar and nucleotide sugar metabolism | 0.204082 | 0.00656 |
| mmu00010 | Glycolysis / Gluconeogenesis | 0.1875 | 0.00624 |
| mmu00860 | Porphyrin metabolism | 0.219512 | 0.00592 |
| mmu05014 | Amyotrophic lateral sclerosis | 0.123626 | 0.00529 |
| mmu01040 | Biosynthesis of unsaturated fatty acids | 0.242424 | 0.00492 |
| mmu00750 | Vitamin B6 metabolism | 0.444444 | 0.00437 |
| mmu05010 | Alzheimer disease | 0.124011 | 0.00418 |
| mmu04610 | Complement and coagulation cascades | 0.175824 | 0.00341 |
| mmu05415 | Diabetic cardiomyopathy | 0.142157 | 0.00338 |
| mmu05207 | Chemical carcinogenesis - receptor activation | 0.144231 | 0.00233 |
| mmu04714 | Thermogenesis | 0.141593 | 0.00229 |
| mmu05020 | Prion disease | 0.143939 | 0.00069 |
| mmu04979 | Cholesterol metabolism | 0.24 | 0.00068 |
| mmu00910 | Nitrogen metabolism | 0.411765 | 0.00026 |
| mmu00591 | Linoleic acid metabolism | 0.26 | 0.00018 |
| mmu05208 | Chemical carcinogenesis - reactive oxygen species | 0.163636 | 0.00007 |
| mmu00480 | Glutathione metabolism | 0.239437 | 0.00006 |
| mmu00190 | Oxidative phosphorylation | 0.19403 | 0.00004 |
| mmu04976 | Bile secretion | 0.22449 | 0.00002 |
| mmu05012 | Parkinson disease | 0.168582 | 0.00001 |
| mmu00983 | Drug metabolism - other enzymes | 0.247191 | <0.00001 |
| mmu00980 | Metabolism of xenobiotics by cytochrome P450 | 0.28169 | <0.00001 |
| mmu00830 | Retinol metabolism | 0.252632 | <0.00001 |
| mmu00982 | Drug metabolism - cytochrome P450 | 0.304348 | <0.00001 |
| mmu00140 | Steroid hormone biosynthesis | 0.280899 | <0.00001 |
| mmu04141 | Protein processing in endoplasmic reticulum | 0.236686 | <0.00001 |
| mmu05204 | Chemical carcinogenesis - DNA adducts | 0.333333 | <0.00001 |
| mmu03060 | Protein export | 0.571429 | <0.00001 |

Rich factor: The ratio of the number of genes belonging to this pathway in the target gene set to the number of all genes belonging to this pathway in the background gene set.

**Table S3B.** The KEGG pathway of differentially expressed genes (DEGs) identified by Kyoto Encyclopedia of Genes and Genomes (KEGG) database in the Sham and I1R24 groups.

| **Pathway ID** | **Pathway description** | **Rich factor** | **P-value** |
| --- | --- | --- | --- |
| mmu04740 | Olfactory transduction | 0.038809 | 0.99999 |
| mmu04080 | Neuroactive ligand-receptor interaction | 0.047368 | 0.99883 |
| mmu05320 | Autoimmune thyroid disease | 0.014085 | 0.99837 |
| mmu04940 | Type I diabetes mellitus | 0.016129 | 0.99631 |
| mmu04120 | Ubiquitin mediated proteolysis | 0.034722 | 0.99581 |
| mmu05330 | Allograft rejection | 0.018182 | 0.99304 |
| mmu05332 | Graft-versus-host disease | 0.018182 | 0.99304 |
| mmu03013 | Nucleocytoplasmic transport | 0.035398 | 0.99023 |
| mmu02010 | ABC transporters | 0.019608 | 0.99001 |
| mmu04650 | Natural killer cell mediated cytotoxicity | 0.036036 | 0.98884 |
| mmu03022 | Basal transcription factors | 0.022727 | 0.98118 |
| mmu04620 | Toll-like receptor signaling pathway | 0.040404 | 0.97569 |
| mmu05322 | Systemic lupus erythematosus | 0.043478 | 0.97446 |
| mmu05226 | Gastric cancer | 0.047945 | 0.97290 |
| mmu03250 | Viral life cycle - HIV-1 | 0.034483 | 0.96555 |
| mmu04390 | Hippo signaling pathway | 0.051613 | 0.96252 |
| mmu04742 | Taste transduction | 0.043478 | 0.96231 |
| mmu03030 | DNA replication | 0.029412 | 0.95352 |
| mmu00330 | Arginine and proline metabolism | 0.037037 | 0.95342 |
| mmu05167 | Kaposi sarcoma-associated herpesvirus infection | 0.059361 | 0.94556 |
| mmu00512 | Mucin type O-glycan biosynthesis | 0.03125 | 0.94431 |
| mmu05224 | Breast cancer | 0.054795 | 0.94223 |
| mmu05211 | Renal cell carcinoma | 0.044118 | 0.94015 |
| mmu03020 | RNA polymerase | 0.032258 | 0.93905 |
| mmu04012 | ErbB signaling pathway | 0.047619 | 0.93881 |
| mmu05132 | Salmonella infection | 0.061728 | 0.93714 |
| mmu03015 | mRNA surveillance pathway | 0.052632 | 0.92089 |
| mmu05170 | Human immunodeficiency virus 1 infection | 0.064103 | 0.91329 |
| mmu04392 | Hippo signaling pathway - multiple species | 0.037037 | 0.91252 |
| mmu04623 | Cytosolic DNA-sensing pathway | 0.048387 | 0.91195 |
| mmu04950 | Maturity onset diabetes of the young | 0.038462 | 0.90425 |
| mmu00563 | Glycosylphosphatidylinositol (GPI)-anchor biosynthesis | 0.038462 | 0.90425 |
| mmu00601 | Glycosphingolipid biosynthesis - lacto and neolacto series | 0.038462 | 0.90425 |
| mmu04962 | Vasopressin-regulated water reabsorption | 0.045455 | 0.90280 |
| mmu05210 | Colorectal cancer | 0.05618 | 0.89159 |
| mmu00260 | Glycine, serine and threonine metabolism | 0.047619 | 0.88781 |
| mmu05213 | Endometrial cancer | 0.051724 | 0.88693 |
| mmu00900 | Terpenoid backbone biosynthesis | 0.041667 | 0.88530 |
| mmu04150 | mTOR signaling pathway | 0.064103 | 0.87438 |
| mmu04622 | RIG-I-like receptor signaling pathway | 0.057143 | 0.86423 |
| mmu05140 | Leishmaniasis | 0.057143 | 0.86423 |
| mmu00430 | Taurine and hypotaurine metabolism | 0.045455 | 0.86260 |
| mmu00350 | Tyrosine metabolism | 0.051282 | 0.86128 |
| mmu05168 | Herpes simplex virus 1 infection | 0.073227 | 0.85805 |
| mmu04137 | Mitophagy - animal | 0.057971 | 0.85669 |
| mmu05161 | Hepatitis B | 0.066667 | 0.85181 |
| mmu00250 | Alanine, aspartate and glutamate metabolism | 0.052632 | 0.85123 |
| mmu04960 | Aldosterone-regulated sodium reabsorption | 0.052632 | 0.85123 |
| mmu04664 | Fc epsilon RI signaling pathway | 0.059701 | 0.84054 |
| mmu05135 | Yersinia infection | 0.066176 | 0.83916 |
| mmu05416 | Viral myocarditis | 0.061728 | 0.83836 |
| mmu00532 | Glycosaminoglycan biosynthesis - chondroitin sulfate / dermatan sulfate | 0.05 | 0.83541 |
| mmu00770 | Pantothenate and CoA biosynthesis | 0.05 | 0.83541 |
| mmu04720 | Long-term potentiation | 0.060606 | 0.83191 |
| mmu04614 | Renin-angiotensin system | 0.055556 | 0.82911 |
| mmu04979 | Cholesterol metabolism | 0.06 | 0.81724 |
| mmu00380 | Tryptophan metabolism | 0.06 | 0.81724 |
| mmu05163 | Human cytomegalovirus infection | 0.072289 | 0.81539 |
| mmu05225 | Hepatocellular carcinoma | 0.070175 | 0.81092 |
| mmu05150 | Staphylococcus aureus infection | 0.067797 | 0.80812 |
| mmu05034 | Alcoholism | 0.070588 | 0.80472 |
| mmu05217 | Basal cell carcinoma | 0.063492 | 0.80365 |
| mmu04660 | T cell receptor signaling pathway | 0.067308 | 0.80325 |
| mmu00120 | Primary bile acid biosynthesis | 0.055556 | 0.80284 |
| mmu05100 | Bacterial invasion of epithelial cells | 0.065789 | 0.79529 |
| mmu04930 | Type II diabetes mellitus | 0.0625 | 0.79492 |
| mmu05321 | Inflammatory bowel disease | 0.064516 | 0.79341 |
| mmu04024 | cAMP signaling pathway | 0.073059 | 0.79080 |
| mmu05142 | Chagas disease | 0.068627 | 0.78686 |
| mmu05220 | Chronic myeloid leukemia | 0.066667 | 0.78567 |
| mmu04961 | Endocrine and other factor-regulated calcium reabsorption | 0.065574 | 0.78275 |
| mmu05235 | PD-L1 expression and PD-1 checkpoint pathway in cancer | 0.068182 | 0.78100 |
| mmu05152 | Tuberculosis | 0.072626 | 0.77980 |
| mmu04916 | Melanogenesis | 0.069307 | 0.77830 |
| mmu04550 | Signaling pathways regulating pluripotency of stem cells | 0.071942 | 0.76911 |
| mmu00603 | Glycosphingolipid biosynthesis - globo and isoglobo series | 0.0625 | 0.76383 |
| mmu04146 | Peroxisome | 0.069767 | 0.76200 |
| mmu04110 | Cell cycle | 0.072 | 0.76039 |
| mmu04625 | C-type lectin receptor signaling pathway | 0.071429 | 0.76000 |
| mmu04520 | Adherens junction | 0.069444 | 0.75472 |
| mmu04917 | Prolactin signaling pathway | 0.069444 | 0.75472 |
| mmu04144 | Endocytosis | 0.076336 | 0.74840 |
| mmu04370 | VEGF signaling pathway | 0.068966 | 0.74814 |
| mmu00970 | Aminoacyl-tRNA biosynthesis | 0.068182 | 0.74346 |
| mmu00604 | Glycosphingolipid biosynthesis - ganglio series | 0.066667 | 0.74153 |
| mmu04340 | Hedgehog signaling pathway | 0.070175 | 0.73570 |
| mmu05221 | Acute myeloid leukemia | 0.071429 | 0.73230 |
| mmu04672 | Intestinal immune network for IgA production | 0.069767 | 0.72909 |
| mmu00514 | Other types of O-glycan biosynthesis | 0.069767 | 0.72909 |
| mmu00240 | Pyrimidine metabolism | 0.071429 | 0.72281 |
| mmu00533 | Glycosaminoglycan biosynthesis - keratan sulfate | 0.071429 | 0.71712 |
| mmu04514 | Cell adhesion molecules | 0.076471 | 0.71485 |
| mmu04360 | Axon guidance | 0.076923 | 0.71143 |
| mmu04640 | Hematopoietic cell lineage | 0.074468 | 0.71121 |
| mmu05219 | Bladder cancer | 0.073171 | 0.69846 |
| mmu00760 | Nicotinate and nicotinamide metabolism | 0.073171 | 0.69846 |
| mmu00650 | Butanoate metabolism | 0.074074 | 0.68927 |
| mmu04530 | Tight junction | 0.078313 | 0.68247 |
| mmu04216 | Ferroptosis | 0.075 | 0.68219 |
| mmu05033 | Nicotine addiction | 0.075 | 0.68219 |
| mmu04060 | Cytokine-cytokine receptor interaction | 0.079861 | 0.68145 |
| mmu00270 | Cysteine and methionine metabolism | 0.075472 | 0.68135 |
| mmu04020 | Calcium signaling pathway | 0.079498 | 0.67888 |
| mmu00790 | Folate biosynthesis | 0.076923 | 0.66897 |
| mmu05133 | Pertussis | 0.077922 | 0.66173 |
| mmu05412 | Arrhythmogenic right ventricular cardiomyopathy | 0.078947 | 0.64912 |
| mmu05203 | Viral carcinogenesis | 0.082126 | 0.61986 |
| mmu04713 | Circadian entrainment | 0.081633 | 0.61691 |
| mmu05134 | Legionellosis | 0.081967 | 0.61355 |
| mmu05166 | Human T-cell leukemia virus 1 infection | 0.082988 | 0.60511 |
| mmu04142 | Lysosome | 0.082707 | 0.60270 |
| mmu04070 | Phosphatidylinositol signaling system | 0.083333 | 0.59319 |
| mmu03040 | Spliceosome | 0.083333 | 0.59245 |
| mmu04919 | Thyroid hormone signaling pathway | 0.083333 | 0.59243 |
| mmu04722 | Neurotrophin signaling pathway | 0.083333 | 0.59243 |
| mmu04810 | Regulation of actin cytoskeleton | 0.0837 | 0.58882 |
| mmu04726 | Serotonergic synapse | 0.083969 | 0.58209 |
| mmu04350 | TGF-beta signaling pathway | 0.084211 | 0.58107 |
| mmu04964 | Proximal tubule bicarbonate reclamation | 0.090909 | 0.57698 |
| mmu03410 | Base excision repair | 0.088235 | 0.57108 |
| mmu05205 | Proteoglycans in cancer | 0.084577 | 0.57004 |
| mmu04064 | NF-kappa B signaling pathway | 0.085714 | 0.55790 |
| mmu01524 | Platinum drug resistance | 0.08642 | 0.55479 |
| mmu04914 | Progesterone-mediated oocyte maturation | 0.086957 | 0.54374 |
| mmu04140 | Autophagy - animal | 0.086331 | 0.54209 |
| mmu04612 | Antigen processing and presentation | 0.0875 | 0.54129 |
| mmu05031 | Amphetamine addiction | 0.088235 | 0.53867 |
| mmu04934 | Cushing syndrome | 0.08642 | 0.53714 |
| mmu00410 | beta-Alanine metabolism | 0.09375 | 0.52910 |
| mmu00020 | Citrate cycle (TCA cycle) | 0.09375 | 0.52910 |
| mmu04122 | Sulfur relay system | 0.125 | 0.51394 |
| mmu04136 | Autophagy - other | 0.096774 | 0.50726 |
| mmu00630 | Glyoxylate and dicarboxylate metabolism | 0.096774 | 0.50726 |
| mmu04211 | Longevity regulating pathway | 0.089888 | 0.50515 |
| mmu04727 | GABAergic synapse | 0.090909 | 0.49206 |
| mmu05212 | Pancreatic cancer | 0.092105 | 0.48573 |
| mmu03008 | Ribosome biogenesis in eukaryotes | 0.092105 | 0.48573 |
| mmu04915 | Estrogen signaling pathway | 0.090226 | 0.47886 |
| mmu03440 | Homologous recombination | 0.097561 | 0.47612 |
| mmu00511 | Other glycan degradation | 0.111111 | 0.46762 |
| mmu00061 | Fatty acid biosynthesis | 0.111111 | 0.46762 |
| mmu04911 | Insulin secretion | 0.093023 | 0.46561 |
| mmu04114 | Oocyte meiosis | 0.091667 | 0.46211 |
| mmu01523 | Antifolate resistance | 0.103448 | 0.46208 |
| mmu00590 | Arachidonic acid metabolism | 0.094118 | 0.45227 |
| mmu03460 | Fanconi anemia pathway | 0.098039 | 0.45169 |
| mmu04014 | Ras signaling pathway | 0.090517 | 0.44088 |
| mmu05160 | Hepatitis C | 0.092025 | 0.43529 |
| mmu04213 | Longevity regulating pathway - multiple species | 0.098361 | 0.43140 |
| mmu05223 | Non-small cell lung cancer | 0.097222 | 0.42841 |
| mmu04970 | Salivary secretion | 0.096386 | 0.42545 |
| mmu05017 | Spinocerebellar ataxia | 0.093525 | 0.42191 |
| mmu00562 | Inositol phosphate metabolism | 0.098592 | 0.41393 |
| mmu04666 | Fc gamma R-mediated phagocytosis | 0.096774 | 0.40991 |
| mmu01522 | Endocrine resistance | 0.097826 | 0.39728 |
| mmu05216 | Thyroid cancer | 0.108108 | 0.39705 |
| mmu04371 | Apelin signaling pathway | 0.095588 | 0.39087 |
| mmu04210 | Apoptosis | 0.095588 | 0.39087 |
| mmu05410 | Hypertrophic cardiomyopathy | 0.1 | 0.37208 |
| mmu04725 | Cholinergic synapse | 0.098214 | 0.37170 |
| mmu04630 | JAK-STAT signaling pathway | 0.095808 | 0.36715 |
| mmu00592 | alpha-Linolenic acid metabolism | 0.12 | 0.36705 |
| mmu04973 | Carbohydrate digestion and absorption | 0.108696 | 0.36399 |
| mmu05202 | Transcriptional misregulation in cancer | 0.095 | 0.36341 |
| mmu04062 | Chemokine signaling pathway | 0.095745 | 0.35613 |
| mmu00280 | Valine, leucine and isoleucine degradation | 0.107143 | 0.35226 |
| mmu04658 | Th1 and Th2 cell differentiation | 0.102273 | 0.34702 |
| mmu04977 | Vitamin digestion and absorption | 0.125 | 0.34269 |
| mmu04270 | Vascular smooth muscle contraction | 0.098592 | 0.33999 |
| mmu04071 | Sphingolipid signaling pathway | 0.1 | 0.33914 |
| mmu05323 | Rheumatoid arthritis | 0.104651 | 0.32223 |
| mmu05144 | Malaria | 0.111111 | 0.32081 |
| mmu04926 | Relaxin signaling pathway | 0.100775 | 0.31962 |
| mmu04215 | Apoptosis - multiple species | 0.121212 | 0.31627 |
| mmu05164 | Influenza A | 0.098837 | 0.31362 |
| mmu04613 | Neutrophil extracellular trap formation | 0.098837 | 0.31362 |
| mmu04721 | Synaptic vesicle cycle | 0.108108 | 0.30523 |
| mmu04659 | Th17 cell differentiation | 0.104762 | 0.29449 |
| mmu04261 | Adrenergic signaling in cardiomyocytes | 0.101351 | 0.29410 |
| mmu05214 | Glioma | 0.109589 | 0.29226 |
| mmu00310 | Lysine degradation | 0.112903 | 0.28480 |
| mmu05146 | Amoebiasis | 0.105769 | 0.28381 |
| mmu04910 | Insulin signaling pathway | 0.102941 | 0.28302 |
| mmu04217 | Necroptosis | 0.10119 | 0.27997 |
| mmu05414 | Dilated cardiomyopathy | 0.107527 | 0.27944 |
| mmu04927 | Cortisol synthesis and secretion | 0.111111 | 0.27942 |
| mmu05218 | Melanoma | 0.111111 | 0.27942 |
| mmu03450 | Non-homologous end-joining | 0.166667 | 0.27728 |
| mmu05200 | Pathways in cancer | 0.09427 | 0.26945 |
| mmu04061 | Viral protein interaction with cytokine and cytokine receptor | 0.108696 | 0.26833 |
| mmu05162 | Measles | 0.103448 | 0.26751 |
| mmu04668 | TNF signaling pathway | 0.106195 | 0.26725 |
| mmu04730 | Long-term depression | 0.116667 | 0.25734 |
| mmu03018 | RNA degradation | 0.1125 | 0.25055 |
| mmu00520 | Amino sugar and nucleotide sugar metabolism | 0.122449 | 0.24463 |
| mmu00920 | Sulfur metabolism | 0.181818 | 0.24408 |
| mmu00513 | Various types of N-glycan biosynthesis | 0.128205 | 0.24308 |
| mmu05417 | Lipid and atherosclerosis | 0.101382 | 0.24267 |
| mmu04218 | Cellular senescence | 0.103448 | 0.24235 |
| mmu04662 | B cell receptor signaling pathway | 0.113924 | 0.23915 |
| mmu04728 | Dopaminergic synapse | 0.10687 | 0.23815 |
| mmu04972 | Pancreatic secretion | 0.109091 | 0.23811 |
| mmu05145 | Toxoplasmosis | 0.109091 | 0.23811 |
| mmu00983 | Drug metabolism - other enzymes | 0.11236 | 0.23591 |
| mmu05030 | Cocaine addiction | 0.125 | 0.23005 |
| mmu04022 | cGMP-PKG signaling pathway | 0.104651 | 0.22741 |
| mmu00670 | One carbon pool by folate | 0.157895 | 0.22187 |
| mmu04310 | Wnt signaling pathway | 0.105263 | 0.22009 |
| mmu05169 | Epstein-Barr virus infection | 0.102679 | 0.21820 |
| mmu01521 | EGFR tyrosine kinase inhibitor resistance | 0.116883 | 0.21691 |
| mmu04621 | NOD-like receptor signaling pathway | 0.104478 | 0.20757 |
| mmu04670 | Leukocyte transendothelial migration | 0.111111 | 0.20747 |
| mmu05022 | Pathways of neurodegeneration - multiple diseases | 0.098081 | 0.19687 |
| mmu05143 | African trypanosomiasis | 0.138889 | 0.19494 |
| mmu05340 | Primary immunodeficiency | 0.138889 | 0.19494 |
| mmu04929 | GnRH secretion | 0.123077 | 0.19488 |
| mmu04935 | Growth hormone synthesis, secretion and action | 0.113043 | 0.19068 |
| mmu04744 | Phototransduction | 0.153846 | 0.18159 |
| mmu00340 | Histidine metabolism | 0.153846 | 0.18159 |
| mmu04966 | Collecting duct acid secretion | 0.153846 | 0.18159 |
| mmu00051 | Fructose and mannose metabolism | 0.142857 | 0.17969 |
| mmu04723 | Retrograde endocannabinoid signaling | 0.111111 | 0.17665 |
| mmu04657 | IL-17 signaling pathway | 0.11828 | 0.17614 |
| mmu04724 | Glutamatergic synapse | 0.115044 | 0.17455 |
| mmu04152 | AMPK signaling pathway | 0.113821 | 0.17318 |
| mmu04912 | GnRH signaling pathway | 0.119565 | 0.16745 |
| mmu00040 | Pentose and glucuronate interconversions | 0.147059 | 0.16491 |
| mmu04975 | Fat digestion and absorption | 0.139535 | 0.16205 |
| mmu00620 | Pyruvate metabolism | 0.139535 | 0.16205 |
| mmu04068 | FoxO signaling pathway | 0.114504 | 0.15751 |
| mmu00071 | Fatty acid degradation | 0.134615 | 0.15749 |
| mmu04936 | Alcoholic liver disease | 0.113475 | 0.15629 |
| mmu00480 | Glutathione metabolism | 0.126761 | 0.15573 |
| mmu01040 | Biosynthesis of unsaturated fatty acids | 0.151515 | 0.15065 |
| mmu04330 | Notch signaling pathway | 0.133333 | 0.14223 |
| mmu04145 | Phagosome | 0.112426 | 0.14021 |
| mmu00860 | Porphyrin metabolism | 0.146341 | 0.13764 |
| mmu00500 | Starch and sucrose metabolism | 0.15625 | 0.13693 |
| mmu05215 | Prostate cancer | 0.122449 | 0.13650 |
| mmu00510 | N-Glycan biosynthesis | 0.14 | 0.13582 |
| mmu05165 | Human papillomavirus infection | 0.104348 | 0.13308 |
| mmu04380 | Osteoclast differentiation | 0.11811 | 0.13137 |
| mmu04072 | Phospholipase D signaling pathway | 0.115646 | 0.13080 |
| mmu05014 | Amyotrophic lateral sclerosis | 0.104396 | 0.12516 |
| mmu04923 | Regulation of lipolysis in adipocytes | 0.140351 | 0.11453 |
| mmu04540 | Gap junction | 0.129412 | 0.11299 |
| mmu04015 | Rap1 signaling pathway | 0.11215 | 0.11034 |
| mmu04924 | Renin secretion | 0.133333 | 0.10941 |
| mmu04971 | Gastric acid secretion | 0.133333 | 0.10941 |
| mmu04918 | Thyroid hormone synthesis | 0.135135 | 0.10227 |
| mmu04010 | MAPK signaling pathway | 0.109589 | 0.09451 |
| mmu04933 | AGE-RAGE signaling pathway in diabetic complications | 0.128713 | 0.09352 |
| mmu05032 | Morphine addiction | 0.131868 | 0.09076 |
| mmu04913 | Ovarian steroidogenesis | 0.142857 | 0.08990 |
| mmu03050 | Proteasome | 0.155556 | 0.08875 |
| mmu00062 | Fatty acid elongation | 0.178571 | 0.08822 |
| mmu04978 | Mineral absorption | 0.150943 | 0.08262 |
| mmu03320 | PPAR signaling pathway | 0.134831 | 0.07970 |
| mmu05231 | Choline metabolism in cancer | 0.132653 | 0.07777 |
| mmu04976 | Bile secretion | 0.132653 | 0.07777 |
| mmu04750 | Inflammatory mediator regulation of TRP channels | 0.126984 | 0.07524 |
| mmu04932 | Non-alcoholic fatty liver disease | 0.123377 | 0.07113 |
| mmu05206 | MicroRNAs in cancer | 0.124224 | 0.06182 |
| mmu05230 | Central carbon metabolism in cancer | 0.149254 | 0.06015 |
| mmu00030 | Pentose phosphate pathway | 0.181818 | 0.06011 |
| mmu04922 | Glucagon signaling pathway | 0.135922 | 0.05859 |
| mmu04925 | Aldosterone synthesis and secretion | 0.135922 | 0.05859 |
| mmu05207 | Chemical carcinogenesis - receptor activation | 0.120192 | 0.05620 |
| mmu05222 | Small cell lung cancer | 0.139785 | 0.05551 |
| mmu05171 | Coronavirus disease - COVID-19 | 0.118143 | 0.05440 |
| mmu00910 | Nitrogen metabolism | 0.235294 | 0.05281 |
| mmu00130 | Ubiquinone and other terpenoid-quinone biosynthesis | 0.3 | 0.04843 |
| mmu00052 | Galactose metabolism | 0.193548 | 0.04620 |
| mmu04931 | Insulin resistance | 0.138889 | 0.04397 |
| mmu04115 | p53 signaling pathway | 0.152778 | 0.04350 |
| mmu00640 | Propanoate metabolism | 0.2 | 0.04007 |
| mmu04920 | Adipocytokine signaling pathway | 0.15493 | 0.03983 |
| mmu05016 | Huntington disease | 0.117057 | 0.03911 |
| mmu00561 | Glycerolipid metabolism | 0.16129 | 0.03829 |
| mmu00230 | Purine metabolism | 0.135338 | 0.03679 |
| mmu00600 | Sphingolipid metabolism | 0.169811 | 0.03592 |
| mmu04066 | HIF-1 signaling pathway | 0.142857 | 0.03057 |
| mmu04151 | PI3K-Akt signaling pathway | 0.116809 | 0.02824 |
| mmu05010 | Alzheimer disease | 0.116095 | 0.02602 |
| mmu05418 | Fluid shear stress and atherosclerosis | 0.136986 | 0.02576 |
| mmu00591 | Linoleic acid metabolism | 0.18 | 0.02555 |
| mmu00565 | Ether lipid metabolism | 0.1875 | 0.01995 |
| mmu03010 | Ribosome | 0.142857 | 0.01970 |
| mmu04260 | Cardiac muscle contraction | 0.159091 | 0.01798 |
| mmu00982 | Drug metabolism - cytochrome P450 | 0.173913 | 0.01416 |
| mmu04921 | Oxytocin signaling pathway | 0.144737 | 0.01107 |
| mmu00053 | Ascorbate and aldarate metabolism | 0.241379 | 0.01003 |
| mmu00140 | Steroid hormone biosynthesis | 0.168539 | 0.00876 |
| mmu00010 | Glycolysis / Gluconeogenesis | 0.1875 | 0.00784 |
| mmu04710 | Circadian rhythm | 0.235294 | 0.00713 |
| mmu04510 | Focal adhesion | 0.141414 | 0.00630 |
| mmu04714 | Thermogenesis | 0.141593 | 0.00361 |
| mmu00830 | Retinol metabolism | 0.178947 | 0.00291 |
| mmu04611 | Platelet activation | 0.168 | 0.00226 |
| mmu04928 | Parathyroid hormone synthesis, secretion and action | 0.17757 | 0.00187 |
| mmu00564 | Glycerophospholipid metabolism | 0.183673 | 0.00164 |
| mmu05204 | Chemical carcinogenesis - DNA adducts | 0.197531 | 0.00133 |
| mmu05208 | Chemical carcinogenesis - reactive oxygen species | 0.15 | 0.00121 |
| mmu05020 | Prion disease | 0.143939 | 0.00119 |
| mmu00100 | Steroid biosynthesis | 0.35 | 0.00099 |
| mmu04512 | ECM-receptor interaction | 0.197674 | 0.00094 |
| mmu00980 | Metabolism of xenobiotics by cytochrome P450 | 0.211268 | 0.00090 |
| mmu04974 | Protein digestion and absorption | 0.190476 | 0.00058 |
| mmu05012 | Parkinson disease | 0.149425 | 0.00050 |
| mmu05415 | Diabetic cardiomyopathy | 0.161765 | 0.00031 |
| mmu04141 | Protein processing in endoplasmic reticulum | 0.171598 | 0.00026 |
| mmu04610 | Complement and coagulation cascades | 0.21978 | 0.00008 |
| mmu03060 | Protein export | 0.357143 | 0.00007 |
| mmu00190 | Oxidative phosphorylation | 0.201493 | 0.00002 |

Rich factor: The ratio of the number of genes belonging to this pathway in the target gene set to the number of all genes belonging to this pathway in the background gene set.

**Table S4C.** The KEGG pathway of differentially expressed genes (DEGs) identified by Kyoto Encyclopedia of Genes and Genomes (KEGG) database in the Sham and I1R12 groups.

| **Pathway ID** | **Pathway description** | **Rich factor** | **P-value** |
| --- | --- | --- | --- |
| mmu04740 | Olfactory transduction | 0.063177 | 0.99999 |
| mmu04080 | Neuroactive ligand-receptor interaction | 0.073684 | 0.99999 |
| mmu04137 | Mitophagy - animal | 0.028986 | 0.99984 |
| mmu04650 | Natural killer cell mediated cytotoxicity | 0.054054 | 0.99963 |
| mmu05211 | Renal cell carcinoma | 0.044118 | 0.99878 |
| mmu04120 | Ubiquitin mediated proteolysis | 0.083333 | 0.99482 |
| mmu00512 | Mucin type O-glycan biosynthesis | 0.03125 | 0.99473 |
| mmu04110 | Cell cycle | 0.08 | 0.99426 |
| mmu05320 | Autoimmune thyroid disease | 0.070423 | 0.98775 |
| mmu04950 | Maturity onset diabetes of the young | 0.038462 | 0.98590 |
| mmu05322 | Systemic lupus erythematosus | 0.086957 | 0.98553 |
| mmu05310 | Asthma | 0.04 | 0.98338 |
| mmu05220 | Chronic myeloid leukemia | 0.08 | 0.97808 |
| mmu05150 | Staphylococcus aureus infection | 0.09322 | 0.97656 |
| mmu05144 | Malaria | 0.074074 | 0.97194 |
| mmu04962 | Vasopressin-regulated water reabsorption | 0.068182 | 0.97136 |
| mmu04940 | Type I diabetes mellitus | 0.080645 | 0.96736 |
| mmu00532 | Glycosaminoglycan biosynthesis - chondroitin sulfate / dermatan sulfate | 0.05 | 0.96227 |
| mmu04060 | Cytokine-cytokine receptor interaction | 0.118056 | 0.95532 |
| mmu05033 | Nicotine addiction | 0.075 | 0.95328 |
| mmu05166 | Human T-cell leukemia virus 1 infection | 0.116183 | 0.95073 |
| mmu04390 | Hippo signaling pathway | 0.109677 | 0.94548 |
| mmu04620 | Toll-like receptor signaling pathway | 0.10101 | 0.94423 |
| mmu05034 | Alcoholism | 0.111765 | 0.94369 |
| mmu04514 | Cell adhesion molecules | 0.111765 | 0.94369 |
| mmu05226 | Gastric cancer | 0.109589 | 0.94107 |
| mmu05216 | Thyroid cancer | 0.081081 | 0.93322 |
| mmu05330 | Allograft rejection | 0.090909 | 0.93321 |
| mmu05332 | Graft-versus-host disease | 0.090909 | 0.93321 |
| mmu04392 | Hippo signaling pathway - multiple species | 0.074074 | 0.93041 |
| mmu05217 | Basal cell carcinoma | 0.095238 | 0.92933 |
| mmu05170 | Human immunodeficiency virus 1 infection | 0.119658 | 0.92909 |
| mmu04614 | Renin-angiotensin system | 0.083333 | 0.92494 |
| mmu00601 | Glycosphingolipid biosynthesis - lacto and neolacto series | 0.076923 | 0.92052 |
| mmu04640 | Hematopoietic cell lineage | 0.106383 | 0.91818 |
| mmu00730 | Thiamine metabolism | 0.066667 | 0.91435 |
| mmu00514 | Other types of O-glycan biosynthesis | 0.093023 | 0.90691 |
| mmu04672 | Intestinal immune network for IgA production | 0.093023 | 0.90691 |
| mmu03420 | Nucleotide excision repair | 0.093023 | 0.90691 |
| mmu04742 | Taste transduction | 0.108696 | 0.90516 |
| mmu00533 | Glycosaminoglycan biosynthesis - keratan sulfate | 0.071429 | 0.89909 |
| mmu00515 | Mannose type O-glycan biosynthesis | 0.083333 | 0.89666 |
| mmu04014 | Ras signaling pathway | 0.125 | 0.88875 |
| mmu05161 | Hepatitis B | 0.121212 | 0.88444 |
| mmu04211 | Longevity regulating pathway | 0.11236 | 0.88244 |
| mmu05202 | Transcriptional misregulation in cancer | 0.125 | 0.87341 |
| mmu04136 | Autophagy - other | 0.096774 | 0.86768 |
| mmu00430 | Taurine and hypotaurine metabolism | 0.090909 | 0.86625 |
| mmu03430 | Mismatch repair | 0.090909 | 0.86625 |
| mmu04550 | Signaling pathways regulating pluripotency of stem cells | 0.122302 | 0.85883 |
| mmu05167 | Kaposi sarcoma-associated herpesvirus infection | 0.127854 | 0.85631 |
| mmu04622 | RIG-I-like receptor signaling pathway | 0.114286 | 0.84914 |
| mmu05321 | Inflammatory bowel disease | 0.112903 | 0.84665 |
| mmu05169 | Epstein-Barr virus infection | 0.129464 | 0.84215 |
| mmu05132 | Salmonella infection | 0.131687 | 0.82579 |
| mmu03022 | Basal transcription factors | 0.113636 | 0.81512 |
| mmu04664 | Fc epsilon RI signaling pathway | 0.119403 | 0.81293 |
| mmu04660 | T cell receptor signaling pathway | 0.125 | 0.80923 |
| mmu05210 | Colorectal cancer | 0.123596 | 0.80677 |
| mmu02010 | ABC transporters | 0.117647 | 0.80284 |
| mmu03040 | Spliceosome | 0.128788 | 0.79706 |
| mmu04140 | Autophagy - animal | 0.129496 | 0.79486 |
| mmu04350 | TGF-beta signaling pathway | 0.126316 | 0.79047 |
| mmu04630 | JAK-STAT signaling pathway | 0.131737 | 0.78836 |
| mmu05223 | Non-small cell lung cancer | 0.125 | 0.77893 |
| mmu03030 | DNA replication | 0.117647 | 0.77679 |
| mmu00563 | Glycosylphosphatidylinositol (GPI)-anchor biosynthesis | 0.115385 | 0.77493 |
| mmu00750 | Vitamin B6 metabolism | 0.111111 | 0.77103 |
| mmu05323 | Rheumatoid arthritis | 0.127907 | 0.76926 |
| mmu05222 | Small cell lung cancer | 0.129032 | 0.76558 |
| mmu00760 | Nicotinate and nicotinamide metabolism | 0.121951 | 0.76267 |
| mmu00240 | Pyrimidine metabolism | 0.125 | 0.76154 |
| mmu04144 | Endocytosis | 0.137405 | 0.75773 |
| mmu00565 | Ether lipid metabolism | 0.125 | 0.75232 |
| mmu04930 | Type II diabetes mellitus | 0.125 | 0.75232 |
| mmu00592 | alpha-Linolenic acid metabolism | 0.12 | 0.75100 |
| mmu05221 | Acute myeloid leukemia | 0.128571 | 0.74943 |
| mmu04722 | Neurotrophin signaling pathway | 0.133333 | 0.74389 |
| mmu04012 | ErbB signaling pathway | 0.130952 | 0.74159 |
| mmu04623 | Cytosolic DNA-sensing pathway | 0.129032 | 0.73844 |
| mmu04659 | Th17 cell differentiation | 0.133333 | 0.73447 |
| mmu04625 | C-type lectin receptor signaling pathway | 0.133929 | 0.73291 |
| mmu05100 | Bacterial invasion of epithelial cells | 0.131579 | 0.72994 |
| mmu03010 | Ribosome | 0.135338 | 0.72986 |
| mmu04360 | Axon guidance | 0.137363 | 0.72836 |
| mmu00330 | Arginine and proline metabolism | 0.12963 | 0.72659 |
| mmu04213 | Longevity regulating pathway - multiple species | 0.131148 | 0.72134 |
| mmu00603 | Glycosphingolipid biosynthesis - globo and isoglobo series | 0.125 | 0.72010 |
| mmu05224 | Breast cancer | 0.136986 | 0.71739 |
| mmu04670 | Leukocyte transendothelial migration | 0.136752 | 0.70610 |
| mmu04727 | GABAergic synapse | 0.136364 | 0.69480 |
| mmu04370 | VEGF signaling pathway | 0.137931 | 0.66570 |
| mmu05213 | Endometrial cancer | 0.137931 | 0.66570 |
| mmu04929 | GnRH secretion | 0.138462 | 0.66433 |
| mmu05218 | Melanoma | 0.138889 | 0.66355 |
| mmu04666 | Fc gamma R-mediated phagocytosis | 0.139785 | 0.66341 |
| mmu04662 | B cell receptor signaling pathway | 0.139241 | 0.66320 |
| mmu04380 | Osteoclast differentiation | 0.141732 | 0.65343 |
| mmu05143 | African trypanosomiasis | 0.138889 | 0.65174 |
| mmu03013 | Nucleocytoplasmic transport | 0.141593 | 0.65087 |
| mmu04217 | Necroptosis | 0.142857 | 0.64999 |
| mmu04914 | Progesterone-mediated oocyte maturation | 0.141304 | 0.64757 |
| mmu04725 | Cholinergic synapse | 0.142857 | 0.63626 |
| mmu04064 | NF-kappa B signaling pathway | 0.142857 | 0.63458 |
| mmu05203 | Viral carcinogenesis | 0.144928 | 0.62754 |
| mmu05140 | Leishmaniasis | 0.142857 | 0.62707 |
| mmu04530 | Tight junction | 0.144578 | 0.62573 |
| mmu05162 | Measles | 0.144828 | 0.61879 |
| mmu05152 | Tuberculosis | 0.145251 | 0.61827 |
| mmu04068 | FoxO signaling pathway | 0.145038 | 0.61391 |
| mmu00531 | Glycosaminoglycan degradation | 0.15 | 0.60030 |
| mmu00770 | Pantothenate and CoA biosynthesis | 0.15 | 0.60030 |
| mmu05135 | Yersinia infection | 0.147059 | 0.58878 |
| mmu05031 | Amphetamine addiction | 0.147059 | 0.58864 |
| mmu05160 | Hepatitis C | 0.147239 | 0.58802 |
| mmu04150 | mTOR signaling pathway | 0.147436 | 0.58486 |
| mmu05416 | Viral myocarditis | 0.148148 | 0.57680 |
| mmu04215 | Apoptosis - multiple species | 0.151515 | 0.57127 |
| mmu00030 | Pentose phosphate pathway | 0.151515 | 0.57127 |
| mmu05163 | Human cytomegalovirus infection | 0.148594 | 0.57088 |
| mmu00524 | Neomycin, kanamycin and gentamicin biosynthesis | 0.2 | 0.55907 |
| mmu05205 | Proteoglycans in cancer | 0.149254 | 0.55902 |
| mmu04612 | Antigen processing and presentation | 0.15 | 0.55845 |
| mmu05214 | Glioma | 0.150685 | 0.55366 |
| mmu04915 | Estrogen signaling pathway | 0.150376 | 0.54604 |
| mmu04010 | MAPK signaling pathway | 0.150685 | 0.53247 |
| mmu04960 | Aldosterone-regulated sodium reabsorption | 0.157895 | 0.52195 |
| mmu04726 | Serotonergic synapse | 0.152672 | 0.51681 |
| mmu04130 | SNARE interactions in vesicular transport | 0.16129 | 0.51262 |
| mmu05200 | Pathways in cancer | 0.151571 | 0.50525 |
| mmu00970 | Aminoacyl-tRNA biosynthesis | 0.159091 | 0.50468 |
| mmu04972 | Pancreatic secretion | 0.154545 | 0.49994 |
| mmu04371 | Apelin signaling pathway | 0.154412 | 0.49302 |
| mmu00450 | Selenocompound metabolism | 0.176471 | 0.48523 |
| mmu05212 | Pancreatic cancer | 0.157895 | 0.48246 |
| mmu00290 | Valine, leucine and isoleucine biosynthesis | 0.25 | 0.48059 |
| mmu00785 | Lipoic acid metabolism | 0.25 | 0.48059 |
| mmu00520 | Amino sugar and nucleotide sugar metabolism | 0.163265 | 0.46564 |
| mmu05235 | PD-L1 expression and PD-1 checkpoint pathway in cancer | 0.159091 | 0.46157 |
| mmu04024 | cAMP signaling pathway | 0.155251 | 0.45964 |
| mmu04961 | Endocrine and other factor-regulated calcium reabsorption | 0.163934 | 0.44287 |
| mmu05030 | Cocaine addiction | 0.166667 | 0.44139 |
| mmu05171 | Coronavirus disease - COVID-19 | 0.156118 | 0.44094 |
| mmu04657 | IL-17 signaling pathway | 0.16129 | 0.43473 |
| mmu05225 | Hepatocellular carcinoma | 0.157895 | 0.43329 |
| mmu03440 | Homologous recombination | 0.170732 | 0.42695 |
| mmu05219 | Bladder cancer | 0.170732 | 0.42695 |
| mmu04720 | Long-term potentiation | 0.166667 | 0.41243 |
| mmu03410 | Base excision repair | 0.176471 | 0.40946 |
| mmu04710 | Circadian rhythm | 0.176471 | 0.40946 |
| mmu04917 | Prolactin signaling pathway | 0.166667 | 0.40423 |
| mmu00604 | Glycosphingolipid biosynthesis - ganglio series | 0.2 | 0.40036 |
| mmu04910 | Insulin signaling pathway | 0.161765 | 0.39888 |
| mmu04920 | Adipocytokine signaling pathway | 0.169014 | 0.38466 |
| mmu03250 | Viral life cycle - HIV-1 | 0.172414 | 0.37801 |
| mmu01521 | EGFR tyrosine kinase inhibitor resistance | 0.168831 | 0.37786 |
| mmu05164 | Influenza A | 0.162791 | 0.36427 |
| mmu03015 | mRNA surveillance pathway | 0.168421 | 0.35948 |
| mmu04340 | Hedgehog signaling pathway | 0.175439 | 0.35651 |
| mmu04744 | Phototransduction | 0.192308 | 0.35525 |
| mmu00340 | Histidine metabolism | 0.192308 | 0.35525 |
| mmu04916 | Melanogenesis | 0.168317 | 0.35390 |
| mmu04919 | Thyroid hormone signaling pathway | 0.166667 | 0.35301 |
| mmu00500 | Starch and sucrose metabolism | 0.1875 | 0.35189 |
| mmu04151 | PI3K-Akt signaling pathway | 0.159544 | 0.34879 |
| mmu04668 | TNF signaling pathway | 0.168142 | 0.34341 |
| mmu04810 | Regulation of actin cytoskeleton | 0.162996 | 0.33344 |
| mmu04728 | Dopaminergic synapse | 0.167939 | 0.32904 |
| mmu04145 | Phagosome | 0.16568 | 0.32776 |
| mmu03020 | RNA polymerase | 0.193548 | 0.32330 |
| mmu04721 | Synaptic vesicle cycle | 0.175676 | 0.32254 |
| mmu04911 | Insulin secretion | 0.174419 | 0.31448 |
| mmu04218 | Cellular senescence | 0.166667 | 0.31178 |
| mmu04152 | AMPK signaling pathway | 0.170732 | 0.30587 |
| mmu05340 | Primary immunodeficiency | 0.194444 | 0.29619 |
| mmu05417 | Lipid and atherosclerosis | 0.165899 | 0.29617 |
| mmu04977 | Vitamin digestion and absorption | 0.208333 | 0.29168 |
| mmu04613 | Neutrophil extracellular trap formation | 0.168605 | 0.28887 |
| mmu04520 | Adherens junction | 0.180556 | 0.28680 |
| mmu04310 | Wnt signaling pathway | 0.169591 | 0.27765 |
| mmu05142 | Chagas disease | 0.176471 | 0.27371 |
| mmu00860 | Porphyrin metabolism | 0.195122 | 0.27270 |
| mmu00051 | Fructose and mannose metabolism | 0.2 | 0.27095 |
| mmu00562 | Inositol phosphate metabolism | 0.183099 | 0.26940 |
| mmu04015 | Rap1 signaling pathway | 0.168224 | 0.26630 |
| mmu04071 | Sphingolipid signaling pathway | 0.175 | 0.26565 |
| mmu04114 | Oocyte meiosis | 0.175 | 0.26565 |
| mmu04020 | Calcium signaling pathway | 0.167364 | 0.26386 |
| mmu04973 | Carbohydrate digestion and absorption | 0.195652 | 0.25204 |
| mmu03008 | Ribosome biogenesis in eukaryotes | 0.184211 | 0.25137 |
| mmu04216 | Ferroptosis | 0.2 | 0.24995 |
| mmu04658 | Th1 and Th2 cell differentiation | 0.181818 | 0.24872 |
| mmu00910 | Nitrogen metabolism | 0.235294 | 0.24839 |
| mmu04923 | Regulation of lipolysis in adipocytes | 0.192982 | 0.23499 |
| mmu04210 | Apoptosis | 0.176471 | 0.23423 |
| mmu03460 | Fanconi anemia pathway | 0.196078 | 0.23366 |
| mmu03050 | Proteasome | 0.2 | 0.23140 |
| mmu04964 | Proximal tubule bicarbonate reclamation | 0.227273 | 0.23046 |
| mmu00513 | Various types of N-glycan biosynthesis | 0.205128 | 0.22785 |
| mmu00440 | Phosphonate and phosphinate metabolism | 0.333333 | 0.22596 |
| mmu04072 | Phospholipase D signaling pathway | 0.176871 | 0.21946 |
| mmu04061 | Viral protein interaction with cytokine and cytokine receptor | 0.184783 | 0.21924 |
| mmu05231 | Choline metabolism in cancer | 0.183673 | 0.21853 |
| mmu05215 | Prostate cancer | 0.183673 | 0.21853 |
| mmu05145 | Toxoplasmosis | 0.181818 | 0.21665 |
| mmu00510 | N-Glycan biosynthesis | 0.2 | 0.21485 |
| mmu00380 | Tryptophan metabolism | 0.2 | 0.21485 |
| mmu05165 | Human papillomavirus infection | 0.168116 | 0.20468 |
| mmu04621 | NOD-like receptor signaling pathway | 0.174129 | 0.20376 |
| mmu05017 | Spinocerebellar ataxia | 0.179856 | 0.19975 |
| mmu04062 | Chemokine signaling pathway | 0.175532 | 0.19824 |
| mmu04931 | Insulin resistance | 0.185185 | 0.19267 |
| mmu04975 | Fat digestion and absorption | 0.209302 | 0.19217 |
| mmu04115 | p53 signaling pathway | 0.194444 | 0.19042 |
| mmu00790 | Folate biosynthesis | 0.230769 | 0.18909 |
| mmu04966 | Collecting duct acid secretion | 0.230769 | 0.18909 |
| mmu04330 | Notch signaling pathway | 0.2 | 0.18644 |
| mmu04934 | Cushing syndrome | 0.179012 | 0.18547 |
| mmu00130 | Ubiquinone and other terpenoid-quinone biosynthesis | 0.3 | 0.18247 |
| mmu04933 | AGE-RAGE signaling pathway in diabetic complications | 0.188119 | 0.18110 |
| mmu05133 | Pertussis | 0.194805 | 0.17809 |
| mmu00360 | Phenylalanine metabolism | 0.25 | 0.17363 |
| mmu04926 | Relaxin signaling pathway | 0.186047 | 0.16037 |
| mmu04974 | Protein digestion and absorption | 0.190476 | 0.15936 |
| mmu05168 | Herpes simplex virus 1 infection | 0.169336 | 0.15501 |
| mmu01524 | Platinum drug resistance | 0.197531 | 0.15465 |
| mmu05146 | Amoebiasis | 0.192308 | 0.14900 |
| mmu00900 | Terpenoid backbone biosynthesis | 0.25 | 0.14274 |
| mmu04935 | Growth hormone synthesis, secretion and action | 0.191304 | 0.14089 |
| mmu04918 | Thyroid hormone synthesis | 0.202703 | 0.14057 |
| mmu00053 | Ascorbate and aldarate metabolism | 0.241379 | 0.13673 |
| mmu01523 | Antifolate resistance | 0.241379 | 0.13673 |
| mmu00590 | Arachidonic acid metabolism | 0.2 | 0.13424 |
| mmu00040 | Pentose and glucuronate interconversions | 0.235294 | 0.13027 |
| mmu04070 | Phosphatidylinositol signaling system | 0.197917 | 0.12794 |
| mmu05206 | MicroRNAs in cancer | 0.186335 | 0.12712 |
| mmu05410 | Hypertrophic cardiomyopathy | 0.2 | 0.12610 |
| mmu00511 | Other glycan degradation | 0.277778 | 0.12320 |
| mmu00061 | Fatty acid biosynthesis | 0.277778 | 0.12320 |
| mmu04928 | Parathyroid hormone synthesis, secretion and action | 0.196262 | 0.12177 |
| mmu04936 | Alcoholic liver disease | 0.191489 | 0.11163 |
| mmu00400 | Phenylalanine, tyrosine and tryptophan biosynthesis | 0.375 | 0.10692 |
| mmu04122 | Sulfur relay system | 0.375 | 0.10692 |
| mmu05412 | Arrhythmogenic right ventricular cardiomyopathy | 0.210526 | 0.10168 |
| mmu04713 | Circadian entrainment | 0.204082 | 0.09521 |
| mmu00564 | Glycerophospholipid metabolism | 0.204082 | 0.09521 |
| mmu04978 | Mineral absorption | 0.226415 | 0.09405 |
| mmu04723 | Retrograde endocannabinoid signaling | 0.194444 | 0.09239 |
| mmu03018 | RNA degradation | 0.2125 | 0.08748 |
| mmu05032 | Morphine addiction | 0.208791 | 0.08530 |
| mmu04540 | Gap junction | 0.211765 | 0.08262 |
| mmu04066 | HIF-1 signaling pathway | 0.205357 | 0.07369 |
| mmu05230 | Central carbon metabolism in cancer | 0.223881 | 0.07225 |
| mmu00220 | Arginine biosynthesis | 0.3 | 0.06910 |
| mmu04979 | Cholesterol metabolism | 0.24 | 0.06528 |
| mmu00350 | Tyrosine metabolism | 0.25641 | 0.05999 |
| mmu04912 | GnRH signaling pathway | 0.217391 | 0.05583 |
| mmu04922 | Glucagon signaling pathway | 0.213592 | 0.05517 |
| mmu04971 | Gastric acid secretion | 0.226667 | 0.05275 |
| mmu00250 | Alanine, aspartate and glutamate metabolism | 0.263158 | 0.05128 |
| mmu01040 | Biosynthesis of unsaturated fatty acids | 0.272727 | 0.05120 |
| mmu00600 | Sphingolipid metabolism | 0.245283 | 0.04844 |
| mmu00270 | Cysteine and methionine metabolism | 0.245283 | 0.04844 |
| mmu05418 | Fluid shear stress and atherosclerosis | 0.205479 | 0.04600 |
| mmu04913 | Ovarian steroidogenesis | 0.238095 | 0.04539 |
| mmu00120 | Primary bile acid biosynthesis | 0.333333 | 0.04307 |
| mmu00410 | beta-Alanine metabolism | 0.28125 | 0.04276 |
| mmu05207 | Chemical carcinogenesis - receptor activation | 0.197115 | 0.04183 |
| mmu00983 | Drug metabolism - other enzymes | 0.224719 | 0.04121 |
| mmu00650 | Butanoate metabolism | 0.296296 | 0.04109 |
| mmu00230 | Purine metabolism | 0.210526 | 0.04005 |
| mmu00561 | Glycerolipid metabolism | 0.241935 | 0.03996 |
| mmu05022 | Pathways of neurodegeneration - multiple diseases | 0.181237 | 0.03920 |
| mmu04970 | Salivary secretion | 0.228916 | 0.03867 |
| mmu04927 | Cortisol synthesis and secretion | 0.236111 | 0.03732 |
| mmu04260 | Cardiac muscle contraction | 0.227273 | 0.03702 |
| mmu05414 | Dilated cardiomyopathy | 0.225806 | 0.03540 |
| mmu00052 | Galactose metabolism | 0.290323 | 0.03531 |
| mmu00630 | Glyoxylate and dicarboxylate metabolism | 0.290323 | 0.03531 |
| mmu04932 | Non-alcoholic fatty liver disease | 0.207792 | 0.03518 |
| mmu05134 | Legionellosis | 0.245902 | 0.03501 |
| mmu00480 | Glutathione metabolism | 0.239437 | 0.03299 |
| mmu01522 | Endocrine resistance | 0.228261 | 0.03178 |
| mmu00591 | Linoleic acid metabolism | 0.26 | 0.03140 |
| mmu04730 | Long-term depression | 0.25 | 0.03052 |
| mmu04724 | Glutamatergic synapse | 0.221239 | 0.02941 |
| mmu00982 | Drug metabolism - cytochrome P450 | 0.246377 | 0.02546 |
| mmu03450 | Non-homologous end-joining | 0.416667 | 0.02455 |
| mmu03320 | PPAR signaling pathway | 0.235955 | 0.02256 |
| mmu00140 | Steroid hormone biosynthesis | 0.235955 | 0.02256 |
| mmu00620 | Pyruvate metabolism | 0.27907 | 0.02233 |
| mmu04261 | Adrenergic signaling in cardiomyocytes | 0.216216 | 0.02097 |
| mmu04270 | Vascular smooth muscle contraction | 0.21831 | 0.02007 |
| mmu00310 | Lysine degradation | 0.258065 | 0.01951 |
| mmu05014 | Amyotrophic lateral sclerosis | 0.192308 | 0.01796 |
| mmu00670 | One carbon pool by folate | 0.368421 | 0.01687 |
| mmu00280 | Valine, leucine and isoleucine degradation | 0.267857 | 0.01669 |
| mmu00920 | Sulfur metabolism | 0.454545 | 0.01632 |
| mmu00020 | Citrate cycle (TCA cycle) | 0.3125 | 0.01627 |
| mmu04512 | ECM-receptor interaction | 0.244186 | 0.01556 |
| mmu04924 | Renin secretion | 0.253333 | 0.01413 |
| mmu04141 | Protein processing in endoplasmic reticulum | 0.218935 | 0.01142 |
| mmu04022 | cGMP-PKG signaling pathway | 0.22093 | 0.00903 |
| mmu04142 | Lysosome | 0.233083 | 0.00795 |
| mmu04510 | Focal adhesion | 0.217172 | 0.00787 |
| mmu04146 | Peroxisome | 0.255814 | 0.00774 |
| mmu00260 | Glycine, serine and threonine metabolism | 0.309524 | 0.00718 |
| mmu00062 | Fatty acid elongation | 0.357143 | 0.00585 |
| mmu00010 | Glycolysis / Gluconeogenesis | 0.28125 | 0.00540 |
| mmu04925 | Aldosterone synthesis and secretion | 0.252427 | 0.00486 |
| mmu05204 | Chemical carcinogenesis - DNA adducts | 0.271605 | 0.00359 |
| mmu00980 | Metabolism of xenobiotics by cytochrome P450 | 0.28169 | 0.00340 |
| mmu05016 | Huntington disease | 0.210702 | 0.00327 |
| mmu00640 | Propanoate metabolism | 0.366667 | 0.00308 |
| mmu05010 | Alzheimer disease | 0.205805 | 0.00229 |
| mmu04750 | Inflammatory mediator regulation of TRP channels | 0.253968 | 0.00173 |
| mmu00830 | Retinol metabolism | 0.273684 | 0.00145 |
| mmu00100 | Steroid biosynthesis | 0.45 | 0.00139 |
| mmu00071 | Fatty acid degradation | 0.326923 | 0.00116 |
| mmu04976 | Bile secretion | 0.27551 | 0.00106 |
| mmu03060 | Protein export | 0.428571 | 0.00039 |
| mmu05012 | Parkinson disease | 0.233716 | 0.00025 |
| mmu04611 | Platelet activation | 0.28 | 0.00015 |
| mmu04610 | Complement and coagulation cascades | 0.307692 | 0.00012 |
| mmu05020 | Prion disease | 0.238636 | 0.00011 |
| mmu04921 | Oxytocin signaling pathway | 0.269737 | 0.00011 |
| mmu05415 | Diabetic cardiomyopathy | 0.254902 | 0.00007 |
| mmu04714 | Thermogenesis | 0.252212 | 0.00005 |
| mmu00190 | Oxidative phosphorylation | 0.291045 | 0.00003 |
| mmu05208 | Chemical carcinogenesis - reactive oxygen species | 0.272727 | <0.00001 |

Rich factor: The ratio of the number of genes belonging to this pathway in the target gene set to the number of all genes belonging to this pathway in the background gene set.
