# Supplementary material for: Multi-time point transcriptomics and metabolomics reveal key transcription and metabolic features of hepatic ischemia-reperfusion injury in mice
Source: Genes Dis. 2024 Nov 17;12(2):101465. doi: 10.1016/j.gendis.2024.101465 (PMC11697123; doi:10.1016/j.gendis.2024.101465)
Supplement: Multimedia component 8 [file mmc8.docx]

**Table S4A.** Identified differentially expressed metabolites in sham and I1R12 groups.

| **Metabolite** | **Library ID** | **VIP** | **P-value** | **Regulate** |
| --- | --- | --- | --- | --- |
| 4alpha-carboxy-5alpha-cholesta-8,24-dien-3beta-ol | LMST01010152 | 1.3783 | 0.04992 | up |
| Risbitin | HMDB0302980 | 1.1722 | 0.04985 | up |
| 9-Aminocamptothecin | HMDB0247579 | 1.1221 | 0.04943 | up |
| 3alpha,7alpha,12alpha-trihydroxy-5beta-cholestanate | HMDB0062207 | 1.4532 | 0.04793 | up |
| Geranate | HMDB0304369 | 1.7466 | 0.04415 | down |
| 2-Undecen-1-ol | HMDB0034856 | 1.227 | 0.04273 | down |
| 12-oxo-PDA | LMFA0201000 | 1.6121 | 0.04195 | down |
| 6-Hydroxyoctanoylcarnitine | HMDB0241741 | 1.4774 | 0.04022 | up |
| N-Palmitoyl Aspartic acid | HMDB0241922 | 1.0394 | 0.03921 | down |
| Antimycin A | HMDB0248488 | 2.8398 | 0.03903 | up |
| Altanserin | HMDB0248260 | 1.1163 | 0.03854 | up |
| Cyclopentanecarboxylic acid | HMDB0250667 | 1.9083 | 0.03746 | up |
| Arecoline | HMDB0030353 | 1.4998 | 0.03608 | up |
| 5-Hydroxyheptanoylcarnitine | HMDB0241678 | 1.3285 | 0.03599 | up |
| L-Proline, 1-(1-L-leucyl-L-prolyl)- | HMDB0253022 | 1.3889 | 0.03591 | up |
| Fenofibric acid | HMDB0250346 | 1.5132 | 0.03519 | up |
| LysoPE(18:0/0:0) | HMDB0011130 | 1.1282 | 0.03456 | up |
| 7-[(1R,2R,3R)-3-Hydroxy-2-[(3S)-3-hydroxyoctyl]-5-oxocyclopentyl]heptanoylcarnitine | HMDB0241874 | 1.3393 | 0.0339 | up |
| Ala-phe | HMDB0028694 | 1.1021 | 0.03306 | up |
| Lorlatinib | HMDB0254164 | 1.4793 | 0.03196 | up |
| (S)-Oleuropeic acid | HMDB0036998 | 1.3849 | 0.03119 | down |
| Valylisoleucine | HMDB0029130 | 1.0361 | 0.03112 | up |
| 4-Vinylphenol sulfate | HMDB0062775 | 1.4828 | 0.03031 | up |
| Propionylcarnitine | HMDB0000824 | 1.31 | 0.02994 | down |
| (2-oxo-2,3-Dihydro-1H-Indol-3-yl)acetic Acid | - | 1.5825 | 0.02975 | up |
| Prenalterol | HMDB0256762 | 1.2341 | 0.02953 | up |
| Hypoglycin B | HMDB0029428 | 1.4782 | 0.02909 | up |
| Dephospho-CoA | PW_C001061 | 1.1125 | 0.02903 | up |
| Bendiocarb | HMDB0248947 | 1.8127 | 0.02812 | up |
| Ineketone | HMDB0036698 | 1.0041 | 0.0281 | down |
| 5'-Guanylic Acid | HMDB0001397 | 1.3596 | 0.02797 | up |
| 3-ketosphingosine | LMSP01010002 | 1.2984 | 0.02786 | down |
| Uric Acid | HMDB0000289 | 0.9803 | 0.02755 | down |
| N-Acetylmuramoyl-Ala | HMDB0060494 | 1.3118 | 0.02749 | up |
| 2-Lysophosphatidylcholine | HMDB0258493 | 1.0315 | 0.02703 | up |
| Nonadecanoic acid | HMDB0000772 | 1.7499 | 0.02681 | up |
| Phe Thr | - | 1.275 | 0.02674 | up |
| Indole-3-carboxilic acid-O-sulphate | HMDB0060002 | 1.5274 | 0.02661 | up |
| Loganoside | HMDB0247827 | 1.1613 | 0.02652 | up |
| Glucosyl (2E,6E,10x)-10,11-dihydroxy-2,6-farnesadienoate | HMDB0037823 | 1.3922 | 0.02643 | down |
| Indole carboxylic acid sulfate | HMDB0304912 | 1.9789 | 0.02602 | up |
| N-Acetyl-L-Tyrosine | HMDB0000866 | 1.0162 | 0.02547 | up |
| 3-Sulfopropyl methacrylate | HMDB0245985 | 1.6728 | 0.02545 | down |
| OXYQUINOLINE | - | 1.3311 | 0.02488 | down |
| Gomisin D | HMDB0252911 | 1.2506 | 0.02452 | down |
| Propyl 2,4-decadienoate | LMFA07011001 | 1.6199 | 0.02395 | down |
| Phenylacetylglutamine | HMDB0006344 | 1.0588 | 0.02385 | up |
| 8,11-eicosadiynoic acid | LMFA01030688 | 1.0683 | 0.02384 | up |
| Taurallocholic acid | HMDB0000922 | 1.7362 | 0.02335 | down |
| Versalide | HMDB0259795 | 1.3941 | 0.02327 | down |
| Octhilinone | - | 1.84 | 0.02306 | down |
| Panthenol | HMDB0004231 | 1.2141 | 0.02265 | up |
| Adipic acid | LMFA01170048 | 1.0392 | 0.02248 | down |
| Heptadecanoic acid | LMFA01010017 | 1.5916 | 0.02181 | up |
| 2,8-Quinolinediol | HMDB0240311 | 1.8862 | 0.02145 | up |
| 12-HETE | HMDB0006111 | 1.4253 | 0.02133 | down |
| Norhygrine | HMDB0302903 | 1.2692 | 0.02087 | up |
| LysoPA(20:2(11Z,14Z)/0:0) | HMDB0114758 | 1.1581 | 0.0206 | up |
| 3-Hydroxyhexanedioylcarnitine | HMDB0241674 | 1.4215 | 0.01998 | down |
| Betamethasone 17,21-dipropionate | HMDB0249160 | 1.121 | 0.01835 | down |
| Liquoric acid | HMDB0034512 | 1.1694 | 0.01828 | up |
| (3S,4R,5R)-1,3,4,5,6-Pentahydroxy-1-morpholin-4-ylhexan-2-one | HMDB0243864 | 1.7367 | 0.01818 | down |
| Histidylvaline | HMDB0028898 | 2.0675 | 0.01807 | up |
| 18-HEPE | HMDB0012611 | 1.7299 | 0.01759 | down |
| Gly Leu | - | 1.4385 | 0.0175 | up |
| 2,6-Dimethylnaphthalene | HMDB0059764 | 1.6993 | 0.01745 | down |
| Hypotaurine | HMDB0000965 | 1.4254 | 0.01707 | up |
| Ribose 1-phosphate | HMDB0001489 | 1.0473 | 0.01694 | down |
| 17-AAG | HMDB0244762 | 1.0819 | 0.0164 | up |
| PI(20:5(5Z,8Z,11Z,14Z,17Z)/0:0) | HMDB0256524 | 1.0326 | 0.01635 | up |
| (8S,9S,10S,11S,13S,14S,17R)-11,17-Dihydroxy-17-(2-hydroxyacetyl)-10,13-dimethyl-9-nonyl-1,2,6,7,8,11,12,14,15,16-decahydrocyclopenta[a]phenanthren-3-one | HMDB0260197 | 1.3522 | 0.01611 | down |
| Equol 4'-O-glucuronide | HMDB0041731 | 1.4832 | 0.01604 | up |
| 3-(naphthalen-1-ylmethyl)-1h-pyrazolo[3,4-d]pyrimidin-4-Amine | - | 2.0072 | 0.01549 | down |
| 1-Acetamidocyclopentanecarboxylic acid | - | 1.0263 | 0.01537 | down |
| Furanodienone | HMDB0036768 | 1.3158 | 0.01517 | up |
| Ribalinium | HMDB0033346 | 1.1031 | 0.01511 | down |
| Hexanoylglycine | HMDB0000701 | 1.8605 | 0.01505 | down |
| N2-Acetylornithine | HMDB0003357 | 0.9968 | 0.0148 | up |
| Palmitoyl Ethanolamide | HMDB0002100 | 1.0518 | 0.0146 | up |
| N6-phenylisopropyladenosine | HMDB0255302 | 1.3962 | 0.01436 | up |
| Biochanin A | LMPK12050229 | 1.2137 | 0.01427 | up |
| S-Lactoylglutathione | HMDB0001066 | 1.8812 | 0.01397 | up |
| 3-Phosphoglycerate | HMDB0000807 | 1.106 | 0.01378 | down |
| Isocolumbin | HMDB0036837 | 1.7751 | 0.01366 | up |
| Ser-Leu | HMDB0258242 | 1.4667 | 0.01357 | up |
| Acetyl-DL-Leucine | HMDB0011756 | 1.1309 | 0.01345 | up |
| 2-Phenylethanol glucuronide | HMDB0010350 | 2.0022 | 0.0133 | up |
| 15-keto-PGE1 | LMFA03010146 | 2.6878 | 0.01324 | up |
| Leucylhydroxyproline | HMDB0028930 | 1.105 | 0.01315 | up |
| PE(18:0/0:0) | LMGP02050001 | 1.0256 | 0.01313 | up |
| N-Acetyl-L-phenylalanine | HMDB0000512 | 1.1939 | 0.01311 | up |
| Gamma-L-Glutamyl-gamma-L-glutamyl-L-methionine | HMDB0038674 | 0.9917 | 0.01297 | up |
| Gly Val Leu | - | 1.6812 | 0.01271 | up |
| Dodecanedioic Acid | LMFA01170009 | 1.1694 | 0.01267 | up |
| 19(S)-HETE | PW_C006775 | 1.6611 | 0.01191 | up |
| 5-Hydroxy-L-tryptophan | HMDB0000472 | 1.4462 | 0.01187 | up |
| 1,3-Propanediol, 2,2-diethyl-, dicarbamate | HMDB0254691 | 1.349 | 0.01159 | up |
| LysoPC(20:4(5Z,8Z,11Z,14Z)/0:0) | HMDB0010395 | 1.5348 | 0.01128 | up |
| Gabazine | HMDB0252568 | 1.4361 | 0.0111 | up |
| Camphorsulfonic Acid | HMDB0243496 | 1.9518 | 0.011 | down |
| O-Desmethyltramadol glucuronide | PW_C040591 | 1.8734 | 0.01079 | down |
| Nifekalant | HMDB0255595 | 1.8545 | 0.01062 | down |
| 16-B1-phytoprostane | HMDB0304634 | 1.3724 | 0.01058 | up |
| Clomoxir | HMDB0250351 | 1.6591 | 0.01055 | down |
| Stercobilin | HMDB0240259 | 3.0424 | 0.01044 | down |
| Adenosine monophosphate | PW_C000032 | 1.2582 | 0.01039 | down |
| 3-[[(2S)-2,4-Dihydroxy-3,3-dimethylbutanoyl]amino]propanoic acid | HMDB0250782 | 1.685 | 0.01029 | up |
| 8(R)-Hydroperoxylinoleic acid | HMDB0004706 | 1.5187 | 0.01022 | down |
| 2-Hydroxyquinoline | - | 1.6136 | 0.01008 | up |
| 4-Guanidinobutanoic acid | HMDB0031842 | 2.1379 | 0.01006 | up |
| 21-Deoxycortisol | LMST02030195 | 2.2078 | 0.01004 | down |
| Glutarate semialdehyde | HMDB0012233 | 1.3615 | 0.009889 | up |
| Avocadene 2-acetate | LMFA05000641 | 1.0323 | 0.009323 | up |
| (1'R)-Nepetalic acid | HMDB0036117 | 1.9024 | 0.009125 | up |
| Phe Ser | - | 1.4823 | 0.00896 | up |
| Tetrahydrocortisol | LMST02030143 | 1.6006 | 0.008851 | down |
| Ecgonine | HMDB0006548 | 1.9236 | 0.008741 | down |
| PGB2 | LMFA03010018 | 1.2584 | 0.008457 | up |
| Ginsenoyne J | HMDB0040373 | 1.5607 | 0.008327 | down |
| Dihomo-alpha-linolenic acid | HMDB0060039 | 0.9817 | 0.008301 | up |
| Deoxycholic acid 3-glucuronide | HMDB0002596 | 2.3527 | 0.008133 | down |
| N-alpha-Acetyl-L-citrulline | HMDB0000856 | 2.4299 | 0.00777 | up |
| 5-Hydroxylysine | HMDB0000450 | 1.8737 | 0.007641 | up |
| Pilocarpine | HMDB0015217 | 1.9563 | 0.007598 | up |
| N6-(1,2-Dicarboxyethyl)-AMP | - | 2.0535 | 0.007581 | down |
| Cinncassiol D3 | HMDB0036854 | 1.9125 | 0.007511 | up |
| N-alpha-Acetyl-L-lysine | HMDB0000446 | 1.2953 | 0.007507 | up |
| 15H-11,12-EETA | PW_C002389 | 1.1442 | 0.007463 | up |
| 2-Hydroxypentanoic Acid | HMDB0001863 | 0.9851 | 0.007409 | down |
| Adenylosuccinate | HMDB0000536 | 1.6854 | 0.007355 | down |
| Hexyl benzoate | HMDB0040431 | 1.733 | 0.00735 | down |
| Phe Gly | - | 1.6893 | 0.007341 | up |
| 11-Hydroxydodecanoylcarnitine | HMDB0241302 | 2.2662 | 0.007318 | down |
| NH-DVal(NMe)-Val-OMe | - | 1.3434 | 0.00719 | up |
| [3-[2,3-Dihydroxypropoxy(hydroxy)phosphoryl]oxy-2-hydroxypropyl] hexadecanoate | HMDB0242118 | 1.1977 | 0.006718 | up |
| LysoPC(20:4(8Z,11Z,14Z,17Z)/0:0) | HMDB0010396 | 1.0029 | 0.006642 | up |
| Isopentenyladenine | HMDB0245646 | 0.9998 | 0.006424 | up |
| PS(22:4(7Z,10Z,13Z,16Z)/PGJ2) | HMDB0283220 | 1.622 | 0.006402 | down |
| Retinol | HMDB0006216 | 1.33 | 0.006363 | up |
| Caprylic acid | HMDB0000482 | 1.0878 | 0.006345 | up |
| Butyryl-L-carnitine | HMDB0002013 | 2.2792 | 0.006282 | down |
| Dodemorph | - | 1.1834 | 0.006212 | down |
| Geranic acid | HMDB0304369 | 1.4609 | 0.006145 | down |
| 7-Aminoheptanoic acid | HMDB0247233 | 0.9678 | 0.005875 | up |
| Docosahexaenoic Acid | HMDB0002183 | 1.0078 | 0.005813 | up |
| Taurine | PW_C000171 | 0.9578 | 0.005771 | down |
| Glycyl-leucine | HMDB0000759 | 0.9865 | 0.005709 | up |
| P-Cresol glucuronide | HMDB0011686 | 1.7785 | 0.005669 | down |
| Gly-Ile | HMDB0028844 | 1.0905 | 0.005574 | up |
| D-Glucamine | HMDB0246693 | 1.3962 | 0.00554 | up |
| Phosphoenolpyruvic Acid | PW_C000180 | 1.4148 | 0.005467 | down |
| Antroquinonol | HMDB0248495 | 1.3479 | 0.005347 | down |
| 1,2,10-Trihydroxydihydro-trans-linalyl oxide 7-O-beta-D-glucopyranoside | HMDB0033237 | 1.2266 | 0.005181 | up |
| 3'-Hydroxy-T2-triol | HMDB0034575 | 1.4942 | 0.004955 | up |
| Threonyllysine | HMDB0029066 | 0.9693 | 0.004955 | up |
| Cis-Quinceoxepane | HMDB0038108 | 0.9815 | 0.004929 | down |
| LysoPC(17:0/0:0) | HMDB0012108 | 1.0657 | 0.00481 | up |
| Butyl (S)-3-hydroxybutyrate glucoside | HMDB0031694 | 1.533 | 0.004755 | up |
| 4-Hydroxyretinoic acid | HMDB0006254 | 1.7386 | 0.004753 | down |
| N-Jasmonoylisoleucine | HMDB0029391 | 1.5238 | 0.004643 | up |
| PE(18:1(9Z)/0:0) | LMGP02050004 | 1.3423 | 0.004577 | up |
| 1-[(2R,3S,5R)-3,4-Dihydroxy-5-(hydroxymethyl)oxolan-2-yl]pyrimidine-2,4-dione | HMDB0246131 | 1.097 | 0.004274 | down |
| (3beta,5alpha,6alpha,7alpha,22E,24R)-5,6-Epoxyergosta-8,14,22-triene-3,7-diol | HMDB0036304 | 1.6903 | 0.004184 | up |
| LysoPA(0:0/18:1(9Z)) | HMDB0007851 | 1.3936 | 0.003915 | up |
| Isofebrifugine | HMDB0253633 | 1.2042 | 0.003856 | up |
| PC(20:5(5Z,8Z,11Z,14Z,17Z)/16:0) | LMGP01011932 | 1.1706 | 0.003775 | down |
| 7-Hydroxyoctanoylcarnitine | HMDB0241695 | 1.9191 | 0.003767 | up |
| N-lactoyl-phenylalanine | HMDB0062175 | 1.0755 | 0.003766 | up |
| Tyrosyl-Proline | HMDB0029113 | 1.0324 | 0.003555 | up |
| Val Asp Ile | - | 1.6061 | 0.003529 | up |
| Myristoylcarnitine | HMDB0254979 | 1.9574 | 0.0035 | down |
| Subaphylline | HMDB0033463 | 2.1203 | 0.003459 | up |
| 3-Acetylphenol sulfate | HMDB0304930 | 1.8686 | 0.003394 | up |
| Ethyl-4-hydroxymethyl-3(2H)-Furanone | - | 1.2224 | 0.003334 | down |
| 8-Hydroxyoctadecanoylcarnitine | HMDB0241524 | 1.6978 | 0.003294 | down |
| Dethiobiotin | HMDB0003581 | 1.1442 | 0.003245 | up |
| Ile-Glu-Thr-Asp-fluoromethyl ketone | HMDB0253396 | 1.695 | 0.003235 | up |
| 2-Hydroxyethanesulfonate | HMDB0003903 | 1.3671 | 0.003231 | down |
| PC(17:2(9Z,12Z)/0:0) | LMGP01050127 | 1.2222 | 0.003024 | up |
| Oxymesterone | HMDB0006027 | 2.1849 | 0.002985 | down |
| Vomifoliol | HMDB0303570 | 1.528 | 0.002714 | up |
| Oxymetazoline | HMDB0015070 | 1.4543 | 0.00268 | up |
| P-Tolyl Sulfate | - | 2.2312 | 0.002648 | down |
| LysoPE(20:5(5Z,8Z,11Z,14Z,17Z)/0:0) | HMDB0011519 | 1.2185 | 0.002635 | up |
| Hydroxyprolyl-Valine | HMDB0028876 | 1.1073 | 0.002581 | up |
| Gamma-L-Glutamyl-L-pipecolic acid | HMDB0038614 | 1.1547 | 0.002567 | up |
| 5'-Methylthioadenosine | PW_C000910 | 1.4931 | 0.002515 | down |
| N5-formyl-N5-hydroxy-L-ornithine | HMDB0304431 | 1.2057 | 0.002458 | up |
| 2'-Hydroxynicotine | HMDB0001329 | 2.4861 | 0.002413 | up |
| Gly-Pro-Arg-Pro-Lys | HMDB0252830 | 1.6407 | 0.002377 | up |
| Eltoprazine | HMDB0251757 | 1.5684 | 0.002326 | up |
| Dihydrokainic acid | HMDB0251319 | 2.2287 | 0.002306 | down |
| LysoPC(0:0/18:0) | HMDB0011128 | 1.0708 | 0.002298 | up |
| PE(15:0/0:0) | LMGP02050031 | 1.3494 | 0.002295 | up |
| PE(16:1/0:0) | - | 1.4996 | 0.002292 | up |
| Pro-Pro-Pro | HMDB0256689 | 1.1261 | 0.002227 | up |
| L-Acetylcarnitine | HMDB0000201 | 1.2158 | 0.002212 | up |
| PE(20:0/PGJ2) | HMDB0261850 | 1.2771 | 0.002204 | down |
| SM(d17:2(4E,8Z)/TXB2) | HMDB0290419 | 1.763 | 0.002197 | up |
| Leucopelargonidin | HMDB0032322 | 1.5642 | 0.002163 | up |
| LysoPC(20:1(11Z)/0:0) | HMDB0010391 | 1.8335 | 0.002117 | up |
| 2-(S-Glutathionyl)acetyl glutathione | HMDB0060343 | 1.2668 | 0.002081 | down |
| LysoPE(0:0/20:0) | HMDB0011481 | 1.8858 | 0.002014 | up |
| 1-Phenyl-2-(pyridin-2-yl)ethanamine | HMDB0247142 | 2.3334 | 0.002001 | down |
| LysoPC(20:2(11Z,14Z)/0:0) | HMDB0010392 | 1.41 | 0.001986 | up |
| Sphinganine | HMDB0242634 | 1.3744 | 0.00198 | down |
| 3-Deoxyestrone | HMDB0245855 | 2.5455 | 0.001905 | down |
| (3beta,5alpha,9alpha,22E,24R)-5,9-Epidioxy-3-hydroxyergosta-7,22-dien-6-one | HMDB0032666 | 1.6267 | 0.001905 | up |
| (8R,9R,10S,13R,14S)-1,2,3,4,5,6,7,8,9,10,11,12,14,15,16,17-Hexadecahydrocyclopenta[a]phenanthrene-13-carbaldehyde | HMDB0260168 | 1.0969 | 0.001861 | down |
| LysoPC(18:1(11Z)/0:0) | HMDB0010385 | 1.4011 | 0.001824 | up |
| Cortisol | LMST02030001 | 2.1528 | 0.001823 | down |
| LysoPC(22:4(7Z,10Z,13Z,16Z)/0:0) | HMDB0010401 | 1.546 | 0.001788 | up |
| 2-[(Tetrahydrofurfuryl)oxy]ethanol | HMDB0252869 | 1.3353 | 0.001741 | up |
| Rocuronium | HMDB0014866 | 1.2154 | 0.001631 | up |
| Afzelechin-(4alpha->8)-pelargonidin 3-O-beta-glucopyranoside | HMDB0302009 | 1.9845 | 0.001549 | down |
| 14,15-DiHETrE | HMDB0002265 | 1.092 | 0.001535 | up |
| L-phenylalanyl-L-proline | HMDB0011177 | 1.1459 | 0.001514 | up |
| Gemeprost | HMDB0252671 | 1.1833 | 0.00151 | up |
| Arginyl-prolyl-proline | HMDB0249363 | 1.6304 | 0.001486 | up |
| Candicine | HMDB0303388 | 1.995 | 0.001481 | down |
| N-lactoyl-Methionine | HMDB0062182 | 1.0995 | 0.001463 | up |
| Leu-Gly-Gly | - | 1.2867 | 0.001459 | up |
| Gly Gly Leu | - | 1.3611 | 0.001458 | up |
| Milbemycin beta1 | HMDB0254721 | 1.5417 | 0.00145 | up |
| Parylene C | HMDB0256138 | 1.5826 | 0.001441 | up |
| LysoPA(20:4(8Z,11Z,14Z,17Z)/0:0) | HMDB0114742 | 1.1519 | 0.001438 | up |
| 3alpha-Hydroxynorethynodrel | HMDB0246253 | 2.1505 | 0.001356 | up |
| Buprenorphine | PW_C009368 | 1.5227 | 0.001346 | up |
| LysoPC(18:3(6Z,9Z,12Z)/0:0) | HMDB0010387 | 1.3971 | 0.00134 | up |
| Yohimbine | HMDB0015464 | 2.3944 | 0.00133 | down |
| 3-Hydroxyanthranilic Acid | PW_C001137 | 1.9376 | 0.001305 | down |
| Undecanoic Acid | HMDB0000947 | 1.6626 | 0.001248 | up |
| LysoPC(16:1(9Z)/0:0) | HMDB0010383 | 1.4262 | 0.001218 | up |
| Pro Phe | - | 1.2043 | 0.001171 | up |
| Castasterone | LMST01030129 | 1.9345 | 0.001136 | up |
| Cycloheximide | HMDB0250657 | 1.2236 | 0.00113 | up |
| Sulfonamidoethanol | HMDB0258596 | 1.2257 | 0.001125 | down |
| GPCho(14:1/20:1) | HMDB0007912 | 1.3134 | 0.001112 | down |
| N-Lactoylphenylalanine | HMDB0062175 | 1.3491 | 0.001099 | up |
| Pc(18:0/0:0) | LMGP01050026 | 1.6708 | 0.001051 | up |
| 3-Methylglutarylcarnitine | HMDB0000552 | 2.8693 | 0.001023 | down |
| Benzoin | HMDB0032039 | 1.1018 | 0.0009705 | down |
| Choline Phosphate | HMDB0001565 | 1.0211 | 0.0009567 | up |
| PC(16:0/18:2(9Z,12Z)) | LMGP01010594 | 1.3585 | 0.000914 | down |
| Valylproline | HMDB0029135 | 1.1812 | 0.0009137 | up |
| Eicosapentaenoic Acid | PW_C001337 | 1.3199 | 0.0008979 | up |
| Isovalerylglycine | HMDB0000678 | 1.407 | 0.0008933 | up |
| Tetradecanedioic acid | HMDB0000872 | 1.0873 | 0.0008552 | up |
| Levomefolic acid | HMDB0254053 | 1.5238 | 0.000814 | down |
| Mesobilirubinogen | HMDB0001898 | 2.8374 | 0.0007979 | down |
| Streptidine | HMDB0258506 | 1.2623 | 0.0007918 | down |
| Adenosine 3'-monophosphate | HMDB0003540 | 1.3885 | 0.0007713 | down |
| 5(6)-Epoxy Prostaglandin E1 | HMDB0012110 | 1.4846 | 0.0007402 | up |
| Trigonelline | HMDB0000875 | 1.188 | 0.0006909 | up |
| Strophanthidin | HMDB0258516 | 2.2359 | 0.0006789 | up |
| LysoPI(18:1(9Z)/0:0) | HMDB0061693 | 1.4158 | 0.0006716 | up |
| Valeric acid | LMFA01010005 | 2.0254 | 0.0006604 | down |
| Contignasterol | HMDB0250439 | 1.591 | 0.0006537 | up |
| 8-Amino-7-oxononanoic acid | HMDB0240687 | 1.1753 | 0.0006499 | up |
| Xanthosine | PW_C000203 | 1.1389 | 0.0006074 | up |
| Glycylprolylarginine | HMDB0252828 | 1.7354 | 0.0005858 | down |
| LysoPC(0:0/16:0) | HMDB0240262 | 1.2277 | 0.0005818 | up |
| Haloperidol | HMDB0014645 | 1.6865 | 0.0005743 | down |
| Allysine | HMDB0303320 | 1.6538 | 0.0005728 | up |
| 1-Carboxyethyltyrosine | HMDB0242159 | 1.2306 | 0.0005582 | up |
| Valtrate | HMDB0034493 | 1.2011 | 0.000557 | up |
| 1,8-Heptadecadiene-4,6-diyne-3,10-diol | LMFA05000584 | 2.4626 | 0.0005536 | up |
| Phenylacetic Acid | HMDB0000209 | 1.3486 | 0.0005293 | up |
| 3-(4-Hydroxyphenyl)lactate | HMDB0000755 | 1.3532 | 0.0005119 | up |
| 2-Phenyl-1,3-propanediol monocarbamate | PW_C040528 | 1.9332 | 0.0005088 | up |
| PC(18:1(9Z)e/2:0) | HMDB0011148 | 1.8482 | 0.0005087 | up |
| Amastatin | HMDB0248277 | 1.326 | 0.0004891 | up |
| Tenuazonic acid | HMDB0036074 | 2.6697 | 0.0004714 | down |
| Deoxynivalenol | HMDB0036156 | 2.0219 | 0.000445 | down |
| PC(17:0/0:0) | HMDB0012108 | 1.4528 | 0.0004438 | up |
| LysoPC(18:4(6Z,9Z,12Z,15Z)/0:0) | HMDB0010389 | 2.4963 | 0.0004417 | up |
| PC(22:6/0:0) | LMGP01050056 | 1.1515 | 0.0004274 | up |
| Ophthalmic Acid | HMDB0005765 | 1.9981 | 0.0004072 | down |
| Dithizone | HMDB0251496 | 3.0704 | 0.0004011 | down |
| Kahweol | HMDB0035602 | 2.3356 | 0.000401 | down |
| 4-Methoxyestrone | HMDB0060088 | 2.6702 | 0.000399 | down |
| Gamma-Glutamylacetamide | HMDB0252618 | 1.8649 | 0.0003808 | down |
| L-Carnitine | PW_C000044 | 1.3524 | 0.0003126 | down |
| L-Theanine | HMDB0034365 | 1.4261 | 0.0003085 | up |
| Miroprofen | HMDB0254753 | 2.5402 | 0.0002955 | up |
| Valproic acid glucuronide | HMDB0000901 | 2.1854 | 0.0002841 | down |
| Heptanoic acid | LMFA01010007 | 2.1014 | 0.0002753 | up |
| Menthyl pyrrolidone carboxylate | HMDB0032368 | 2.882 | 0.0002687 | up |
| Sorbitan laurate | LMFA07011017 | 2.317 | 0.0002665 | up |
| D-Ribose 1-phosphate | HMDB0001489 | 1.5588 | 0.0002661 | down |
| METHACHOLINE | HMDB0015654 | 1.2946 | 0.0002624 | down |
| L-Palmitoylcarnitine | LMFA07070004 | 2.1551 | 0.0002578 | down |
| LysoPE(0:0/22:5(7Z,10Z,13Z,16Z,19Z)) | HMDB0011495 | 1.3617 | 0.0002525 | up |
| Gamma-Glutamylthreonine | HMDB0029159 | 2.247 | 0.0002318 | down |
| PC(15:0/0:0) | LMGP01050016 | 1.4857 | 0.0002197 | up |
| Prostaglandin F1a | HMDB0002685 | 1.4931 | 0.0002166 | up |
| Vulgarole | HMDB0035085 | 1.0468 | 0.0001962 | down |
| Dihydrozeatin-O-glucoside | HMDB0012214 | 2.739 | 0.000195 | up |
| Cropropamide | HMDB0250543 | 1.3546 | 0.0001939 | up |
| Xi-7-Hydroxyhexadecanedioic acid | HMDB0037830 | 2.4848 | 0.0001876 | up |
| Zanamivir | HMDB0014698 | 1.9211 | 0.0001872 | up |
| Pyroglutamyl-glutamyl-proline amide | HMDB0247524 | 2.8535 | 0.0001764 | up |
| Glycylprolylhydroxyproline | HMDB0002171 | 2.0701 | 0.0001639 | down |
| LysoPC(14:0/0:0) | HMDB0010379 | 1.7342 | 0.000157 | up |
| Taurocholic acid 3-sulfate | LMST05020031 | 1.2406 | 0.0001552 | down |
| Cellobioside | HMDB0249793 | 2.9006 | 0.0001521 | up |
| Pro Val | - | 1.1737 | 0.0001403 | up |
| (6Z)-Oct-6-enedioylcarnitine | HMDB0241707 | 1.9372 | 0.0001342 | down |
| N-lactoyl-Tryptophan | HMDB0062178 | 1.3893 | 0.0001299 | up |
| (9S,10S)-9,10-dihydroxyoctadecanoate | HMDB0059633 | 1.3802 | 0.0001201 | up |
| Esculentic acid (Phytolacca) | HMDB0034639 | 1.8604 | 0.0001122 | up |
| Falcarindiol | HMDB0244257 | 2.1141 | 0.0001062 | up |
| Palmitoylcarnitine | HMDB0240783 | 1.9665 | 0.0001046 | down |
| 2,3-dihydroxy-3-methylvalerate | HMDB0304038 | 1.458 | <0.0001 | up |
| Hex-3-enedioylcarnitine | HMDB0241671 | 1.9295 | <0.0001 | down |
| Glycerophosphocholine | HMDB0008787 | 1.8061 | <0.0001 | up |
| PC(17:1/0:0) | - | 1.8371 | <0.0001 | up |
| (12Z)-10-Hydroxyoctadecenoylcarnitine | HMDB0241535 | 1.9616 | <0.0001 | down |
| Glabric acid | HMDB0034689 | 2.7409 | <0.0001 | up |
| (2S,3R)-3-hydroxy-2-methylpentanedioylcarnitine | HMDB0241035 | 2.524 | <0.0001 | up |
| Saxitoxin | HMDB0029368 | 1.757 | <0.0001 | down |
| 3-Hydroxypropyl methacrylate | HMDB0245905 | 2.1747 | <0.0001 | down |
| 1-Isopropyl-N-((6-methyl-2-oxo-4-propyl-1,2-dihydropyridin-3-yl)methyl)-6-(2-(4-methylpiperazin-1-yl)pyridin-4-yl)-1H-indazole-4-carboxamide | HMDB0252956 | 1.8359 | <0.0001 | up |
| Palmitoyl-L-carnitine | HMDB0000222 | 2.5037 | <0.0001 | down |
| Alprostadil | HMDB0001442 | 1.6996 | <0.0001 | up |
| Cotinine glucuronide | HMDB0001013 | 2.598 | <0.0001 | down |
| 1-Heptadecanoylglycerophosphoethanolamine | HMDB0061691 | 1.9333 | <0.0001 | up |
| 3,5,6-Trihydroxy-5-(hydroxymethyl)-2-methoxy-2-cyclohexen-1-one | HMDB0041031 | 1.3931 | <0.0001 | up |
| (24E)-3alpha,15alpha-Diacetoxy-23-oxo-7,9(11),24-lanostatrien-26-oic acid | HMDB0035388 | 1.9092 | <0.0001 | up |
| Dihydrotestosterone | PW_C001745 | 1.324 | <0.0001 | up |
| 4-{[(3S)-3-{[(7-Methoxynaphthalen-2-yl)sulfonyl](methyl)amino}-2-oxopyrrolidin-1-yl]methyl}thiophene-2-carboximidamide | HMDB0257330 | 2.3452 | <0.0001 | down |
| Guanosine diphosphate adenosine | HMDB0001501 | 1.9436 | <0.0001 | down |
| LysoPE(20:0/0:0) | HMDB0011511 | 1.6507 | <0.0001 | up |
| LysoPA(16:0/0:0) | HMDB0007853 | 1.6553 | <0.0001 | down |
| 3'-Adenylic Acid | HMDB0258140 | 1.7927 | <0.0001 | down |
| 3,7,8,15-Scirpenetetrol | HMDB0037560 | 2.6229 | <0.0001 | down |
| 3-methylheptanedioylcarnitine | HMDB0241046 | 2.6839 | <0.0001 | down |
| Caryophyllen-beta | HMDB0304653 | 2.9606 | <0.0001 | down |
| Ruscogenin | HMDB0257364 | 1.7081 | <0.0001 | down |
| Hydroxyprolyl-Proline | HMDB0028871 | 1.8613 | <0.0001 | down |
| Glucosyl (E)-2,6-Dimethyl-2,5-heptadienoate | HMDB0035136 | 3.2785 | <0.0001 | down |
| Arginyl-Gamma-glutamate | HMDB0028723 | 3.073 | <0.0001 | down |
| LysoPC(20:5(5Z,8Z,11Z,14Z,17Z)/0:0) | HMDB0010397 | 1.9607 | <0.0001 | up |
| Prednisolone | HMDB0014998 | 3.0297 | <0.0001 | down |
| N-(2-Hydroxyethyl)-2-(1-isoquinolinylmethylene)hydrazinecarbothioamide | HMDB0247709 | 3.3176 | <0.0001 | down |
| Tryptophyl-Gamma-glutamate | HMDB0029097 | 2.7587 | <0.0001 | down |
| (2S)-2-[[(2R,3S,4R,5R)-5-(6-Aminopurin-9-yl)-3,4-dihydroxyoxolan-2-yl]methylamino]-4-sulfanylbutanoic acid | HMDB0258158 | 1.5193 | <0.0001 | up |
| L-beta-aspartyl-L-glutamic acid | HMDB0011164 | 1.1833 | <0.0001 | down |
| Taxiphyllin | HMDB0030704 | 2.7182 | <0.0001 | down |
| 3,4,5-Trihydroxypentanoylcarnitine | HMDB0241652 | 3.1124 | <0.0001 | up |
| Deca-2,5,8-trienedioylcarnitine | HMDB0241128 | 2.944 | <0.0001 | down |
| UDP-D-Galactose | HMDB0000302 | 3.5463 | <0.0001 | down |
| Uracil | HMDB0000300 | 1.0636 | <0.0001 | down |
| Non-5-enedioylcarnitine | HMDB0241756 | 2.2222 | <0.0001 | down |
| DL-Ethionine | HMDB0251518 | 1.7523 | <0.0001 | up |
| LysoPC(18:0/0:0) | HMDB0010384 | 2.0864 | <0.0001 | up |
| Octanoylcarnitine | LMFA07070095 | 3.8776 | <0.0001 | down |
| (2Z)-5-Hydroxydec-2-enedioylcarnitine | HMDB0241092 | 2.5079 | <0.0001 | down |

VIP: variable importance on projection.

**Table S4B.** Identified differentially expressed metabolites in sham and I1R24 groups.

| **Metabolite** | **Library ID** | **VIP** | **P-value** | **Regulate** |
| --- | --- | --- | --- | --- |
| 2-Tiglylcarnitine | HMDB0241658 | 0.9853 | 0.04958 | down |
| Mesobilirubinogen | HMDB0001898 | 1.8543 | 0.04706 | down |
| Oxymetazoline | HMDB0015070 | 1.1447 | 0.04698 | up |
| Cinncassiol D3 | HMDB0036854 | 1.3844 | 0.04595 | up |
| Altanserin | HMDB0248260 | 1.091 | 0.04583 | up |
| 1-Nitro-7-glutathionyl-8-hydroxy-7,8-dihydronaphthalene | HMDB0060329 | 1.0829 | 0.04571 | up |
| Fructosyl valine | HMDB0252496 | 1.3058 | 0.04529 | up |
| Naringenin Chalcone | HMDB0029631 | 1.7182 | 0.04495 | down |
| 3-(naphthalen-1-ylmethyl)-1h-pyrazolo[3,4-d]pyrimidin-4-Amine | - | 1.6414 | 0.04137 | down |
| N-Arachidonoyl-L-Serine | - | 1.3898 | 0.04096 | up |
| LysoPE(P-18:0/0:0) | HMDB0240598 | 1.3618 | 0.03734 | up |
| 1-Phenyl-2-(pyridin-2-yl)ethanamine | HMDB0247142 | 1.7605 | 0.03716 | down |
| S-(Formylmethyl)glutathione | HMDB0060507 | 1.4413 | 0.03682 | up |
| Pregnanediol | HMDB0004025 | 1.2998 | 0.03618 | up |
| Adenylosuccinate | HMDB0000536 | 1.3547 | 0.03538 | down |
| N6-(1,2-Dicarboxyethyl)-AMP | - | 1.5991 | 0.03475 | down |
| Domoic acid | HMDB0033939 | 1.2141 | 0.03464 | up |
| Oxymesterone | HMDB0006027 | 1.614 | 0.03438 | down |
| O6-Carboxymethyl-2'-deoxy-guanosine | HMDB0255879 | 1.1421 | 0.03382 | up |
| 2-Phenyl-1,3-propanediol monocarbamate | PW_C040528 | 1.3734 | 0.03328 | up |
| Cortolone | LMST02030227 | 1.4149 | 0.03324 | down |
| N-alpha-Acetyl-L-citrulline | HMDB0000856 | 2.1576 | 0.03282 | up |
| Phenol sulphate | PW_C040399 | 1.037 | 0.03266 | up |
| Epirubicin glucuronide | HMDB0251861 | 1.3015 | 0.03186 | down |
| 4-Heptylphenol | HMDB0246435 | 1.6843 | 0.03128 | up |
| Ergosine | HMDB0251913 | 1.2174 | 0.0301 | down |
| O-Phosphoethanolamine | HMDB0000224 | 1.1527 | 0.02985 | down |
| Lumichrome | HMDB0254199 | 1.0151 | 0.02962 | down |
| 7-[(1R,2R,3R)-3-Hydroxy-2-[(3S)-3-hydroxyoctyl]-5-oxocyclopentyl]heptanoylcarnitine | HMDB0241874 | 1.2639 | 0.0291 | up |
| Thr Phe | - | 1.7104 | 0.02851 | up |
| N-Lauroylglycine | HMDB0013272 | 1.3597 | 0.02821 | down |
| 2-Undecen-1-ol | HMDB0034856 | 1.4309 | 0.02819 | down |
| LysoPC(20:2(11Z,14Z)/0:0) | HMDB0010392 | 1.1202 | 0.02801 | up |
| LysoPA(0:0/18:0) | HMDB0007850 | 1.143 | 0.02697 | up |
| D-Erythrose-4-Phosphate | HMDB0001321 | 1.8509 | 0.026 | down |
| Diisononyl phthalate | HMDB0251352 | 1.4545 | 0.02586 | down |
| 3-ketosphingosine | LMSP01010002 | 1.2624 | 0.02581 | down |
| S-(2-Carboxyethyl)-L-cysteine | HMDB0246645 | 1.5196 | 0.02557 | down |
| D-(+)-dihydrocarvone | HMDB0302236 | 1.0585 | 0.02541 | down |
| 6-Hydroxyoctanoylcarnitine | HMDB0241741 | 1.478 | 0.02517 | up |
| Biochanin A | LMPK12050229 | 1.0458 | 0.02504 | up |
| Isocolumbin | HMDB0036837 | 1.6983 | 0.02403 | up |
| 1-Nitro-5-glutathionyl-6-hydroxy-5,6-dihydronaphthalene | HMDB0060326 | 1.0836 | 0.02357 | up |
| Gamma-Glutamyltyrosine | HMDB0011741 | 1.2322 | 0.02312 | up |
| Arecoline | HMDB0030353 | 1.4001 | 0.02272 | up |
| Glutarate semialdehyde | HMDB0012233 | 1.1463 | 0.02243 | up |
| Histidylvaline | HMDB0028898 | 1.5423 | 0.02219 | up |
| Pilocarpine | HMDB0015217 | 1.6065 | 0.0218 | up |
| Equol 4'-O-glucuronide | HMDB0041731 | 1.2999 | 0.02116 | up |
| PS(6 keto-PGF1alpha/14:1(9Z)) | HMDB0280931 | 1.0969 | 0.02083 | up |
| Hypotaurine | HMDB0000965 | 1.5462 | 0.01976 | up |
| Clomoxir | HMDB0250351 | 1.6879 | 0.01956 | down |
| Cortisol | LMST02030001 | 1.4774 | 0.01925 | down |
| PI(20:5(5Z,8Z,11Z,14Z,17Z)/0:0) | HMDB0256524 | 1.0644 | 0.01923 | up |
| 12-oxo-PDA | LMFA02010001 | 1.9461 | 0.01912 | down |
| Uric Acid | HMDB0000289 | 1.0319 | 0.01907 | down |
| LysoPC(20:4(5Z,8Z,11Z,14Z)/0:0) | HMDB0010395 | 1.3331 | 0.019 | up |
| 9(S)-HOTrE | HMDB0247705 | 1.6978 | 0.01891 | down |
| LysoPA(20:2(11Z,14Z)/0:0) | HMDB0114758 | 1.0625 | 0.01881 | up |
| Psychosine sulfate | HMDB0013046 | 1.1448 | 0.01856 | up |
| Glabric acid | HMDB0034689 | 1.8936 | 0.01845 | up |
| Butyryl-L-carnitine | HMDB0002013 | 1.9878 | 0.01776 | down |
| 4-Hydroxyretinoic acid | HMDB0006254 | 1.4269 | 0.01751 | down |
| 12(R)-HETE | PW_C040453 | 1.1001 | 0.01748 | down |
| 3'-Hydroxy-T2-triol | HMDB0034575 | 1.2253 | 0.01704 | up |
| SAICAR | HMDB0240295 | 1.8826 | 0.01652 | up |
| 4-Hydroxy Triamterene | HMDB0246440 | 1.5133 | 0.01647 | down |
| Gomisin D | HMDB0252911 | 1.2106 | 0.01645 | down |
| Arginyl-Gamma-glutamate | HMDB0028723 | 1.7621 | 0.01594 | down |
| 8(R)-Hydroperoxylinoleic acid | HMDB0004706 | 1.4594 | 0.01577 | down |
| N-Acetyl-L-phenylalanine | HMDB0000512 | 1.1399 | 0.01533 | up |
| Panthenol | HMDB0004231 | 1.087 | 0.01529 | up |
| P-Tolyl Sulfate | - | 1.8276 | 0.0145 | down |
| LysoPE(20:4(8Z,11Z,14Z,17Z)/0:0) | HMDB0011518 | 1.0244 | 0.01386 | up |
| Adenosine monophosphate | PW_C000032 | 1.2077 | 0.01345 | down |
| LysoPI(18:1(9Z)/0:0) | HMDB0061693 | 1.0431 | 0.01337 | up |
| 1-[(2R,3S,5R)-3,4-Dihydroxy-5-(hydroxymethyl)oxolan-2-yl]pyrimidine-2,4-dione | HMDB0246131 | 0.9812 | 0.01297 | down |
| LysoPC(0:0/18:2(9Z,12Z)) | HMDB0061700 | 1.1555 | 0.01195 | up |
| Ascorbic Acid | HMDB0000044 | 2.6552 | 0.01105 | down |
| 3alpha,7alpha,12alpha-trihydroxy-5beta-cholestanate | HMDB0062207 | 1.6897 | 0.011 | up |
| 2-Propylglutaric acid | PW_C040548 | 1.3505 | 0.0109 | up |
| Prenalterol | HMDB0256762 | 1.3327 | 0.0107 | up |
| 7-Hydroxyoctanoylcarnitine | HMDB0241695 | 1.7589 | 0.01046 | up |
| Deoxyguanylic Acid | HMDB0001044 | 0.965 | 0.009942 | down |
| Parylene C | HMDB0256138 | 1.2615 | 0.00958 | up |
| Cytidine 5'-monophosphate-N-acetylneuraminic acid | HMDB0001176 | 2.2377 | 0.009575 | down |
| 2-Hydroxyethanesulfonate | HMDB0003903 | 1.2317 | 0.009316 | down |
| Tenuazonic acid | HMDB0036074 | 1.8454 | 0.009254 | down |
| Corticosterone | LMST02030186 | 2.0578 | 0.009135 | down |
| 6''-O-Acetylgenistin | HMDB0029528 | 1.2065 | 0.008866 | down |
| Jasmine ketolactone | HMDB0030150 | 1.0821 | 0.008564 | down |
| Arginyl-prolyl-proline | HMDB0249363 | 1.111 | 0.008432 | up |
| Retaspimycin | HMDB0257169 | 1.1983 | 0.007836 | down |
| Retinyl ester | PW_C001935 | 1.3079 | 0.007592 | up |
| L-Serine | PW_C000120 | 1.2008 | 0.007084 | down |
| Dithizone | HMDB0251496 | 2.5094 | 0.006756 | down |
| Taurallocholic acid | HMDB0000922 | 1.576 | 0.00675 | down |
| Ribose 1-phosphate | HMDB0001489 | 1.2167 | 0.006696 | down |
| Falcarindiol | HMDB0244257 | 1.4916 | 0.006667 | up |
| Gamma-Glutamylthreonine | HMDB0029159 | 1.6759 | 0.006322 | down |
| 16-B1-phytoprostane | HMDB0304634 | 1.5889 | 0.006286 | up |
| 8,11-eicosadiynoic acid | LMFA01030688 | 1.1738 | 0.006265 | up |
| 17-AAG | HMDB0244762 | 1.0409 | 0.006244 | up |
| Gamma-Glutamylmethionine | HMDB0034367 | 1.3292 | 0.006225 | down |
| All-trans-18-Hydroxyretinoic acid | HMDB0012452 | 2.0896 | 0.006189 | down |
| D-Glucamine | HMDB0246693 | 1.3724 | 0.006091 | up |
| 13(S)-HpODE | HMDB0003871 | 1.1872 | 0.005928 | up |
| Dodecanedioic Acid | LMFA01170009 | 1.3946 | 0.005924 | up |
| LysoPC(20:1(11Z)/0:0) | HMDB0010391 | 1.8708 | 0.005903 | up |
| 3-Methylglutarylcarnitine | HMDB0000552 | 2.1904 | 0.005683 | down |
| Versalide | HMDB0259795 | 1.6443 | 0.005675 | down |
| Ophthalmic Acid | HMDB0005765 | 1.6352 | 0.005113 | down |
| 3-Methyladipic Acid | HMDB0000555 | 1.2405 | 0.005104 | down |
| Norhygrine | HMDB0302903 | 1.4199 | 0.004809 | up |
| LysoPE(0:0/20:0) | HMDB0011481 | 1.6016 | 0.004787 | up |
| Propionylcarnitine | HMDB0000824 | 1.7055 | 0.004711 | down |
| Resolvin D5 | LMFA04030003 | 1.6944 | 0.004404 | down |
| Medroxyprogesterone | HMDB0001939 | 1.279 | 0.004376 | down |
| N-(2-Hydroxyethyl)-2-(1-isoquinolinylmethylene)hydrazinecarbothioamide | HMDB0247709 | 2.3488 | 0.004368 | down |
| 15H-11,12-EETA | PW_C002389 | 1.2232 | 0.004319 | up |
| Pro Phe | - | 1.0584 | 0.004309 | up |
| Eltoprazine | HMDB0251757 | 1.471 | 0.004297 | up |
| 2'-Hydroxynicotine | HMDB0001329 | 2.3922 | 0.004133 | up |
| Isovalerylglycine | HMDB0000678 | 1.2395 | 0.00408 | up |
| Candicine | HMDB0303388 | 1.9363 | 0.004078 | down |
| Heptanoic acid | LMFA01010007 | 1.6919 | 0.004021 | up |
| Esculentic acid (Phytolacca) | HMDB0034639 | 1.4862 | 0.003763 | up |
| Citric Acid | PW_C000063 | 1.627 | 0.003658 | down |
| ACEXAMIC ACID | HMDB0247031 | 1.5445 | 0.0036 | up |
| [3-[2,3-Dihydroxypropoxy(hydroxy)phosphoryl]oxy-2-hydroxypropyl] hexadecanoate | HMDB0242118 | 1.2733 | 0.003576 | up |
| 5-Methyltetrahydrofolic acid | PW_C001079 | 1.2488 | 0.003539 | down |
| LysoPA(20:4(8Z,11Z,14Z,17Z)/0:0) | HMDB0114742 | 1.0826 | 0.003524 | up |
| 1,2,10-Trihydroxydihydro-trans-linalyl oxide 7-O-beta-D-glucopyranoside | HMDB0033237 | 1.2727 | 0.003456 | up |
| LysoPE(20:5(5Z,8Z,11Z,14Z,17Z)/0:0) | HMDB0011519 | 1.1117 | 0.003369 | up |
| 5'-Methylthioadenosine | PW_C000910 | 1.5872 | 0.003213 | down |
| NH-DVal(NMe)-Val-OMe | - | 1.333 | 0.003193 | up |
| Rutinose | HMDB0302439 | 1.4674 | 0.003147 | down |
| LysoPA(0:0/18:1(9Z)) | HMDB0007851 | 1.34 | 0.003058 | up |
| Aceglutamide | HMDB0006029 | 1.1441 | 0.003042 | down |
| (3beta,5alpha,6alpha,7alpha,22E,24R)-5,6-Epoxyergosta-8,14,22-triene-3,7-diol | HMDB0036304 | 1.6851 | 0.003031 | up |
| 3-hydroxytridecanoic acid | HMDB0061655 | 1.0836 | 0.002958 | down |
| PE(18:0/0:0) | LMGP02050001 | 1.0725 | 0.00288 | up |
| 2beta-Hydroxytestosterone | HMDB0012654 | 1.3552 | 0.002739 | up |
| Vomifoliol | HMDB0303570 | 1.3747 | 0.002682 | up |
| Blennin D | HMDB0031902 | 1.4499 | 0.002662 | down |
| 2-[(Tetrahydrofurfuryl)oxy]ethanol | HMDB0252869 | 1.4021 | 0.002598 | up |
| PE(15:0/0:0) | LMGP02050031 | 1.4263 | 0.002578 | up |
| Rocuronium | HMDB0014866 | 1.1093 | 0.002573 | up |
| LysoPE(0:0/22:5(7Z,10Z,13Z,16Z,19Z)) | HMDB0011495 | 1.4389 | 0.002561 | up |
| (8R,9R,10S,13R,14S)-1,2,3,4,5,6,7,8,9,10,11,12,14,15,16,17-Hexadecahydrocyclopenta[a]phenanthrene-13-carbaldehyde | HMDB0260168 | 1.0239 | 0.002555 | down |
| 3-Deoxyestrone | HMDB0245855 | 2.2268 | 0.002263 | down |
| LysoPE(18:0/0:0) | HMDB0011130 | 1.4448 | 0.002145 | up |
| Naepaine | HMDB0255417 | 1.2659 | 0.00213 | up |
| (1'R)-Nepetalic acid | HMDB0036117 | 2.1861 | 0.002124 | up |
| Hygromycin B | HMDB0034229 | 2.5917 | 0.002056 | down |
| Dihomo-alpha-linolenic acid | HMDB0060039 | 1.1189 | 0.002027 | up |
| Stercobilin | HMDB0240259 | 3.299 | 0.002019 | down |
| Levomefolic acid | HMDB0254053 | 1.4852 | 0.002009 | down |
| 2-Amino-4-oxo-6-(1',2'-dioxoprolyl)-7,8-dihydroxypteridine | HMDB0001410 | 1.3108 | 0.001911 | down |
| 4-Methoxyestrone | HMDB0060088 | 2.1526 | 0.001835 | down |
| Gemeprost | HMDB0252671 | 1.2205 | 0.00183 | up |
| Leucopelargonidin | HMDB0032322 | 1.5714 | 0.00182 | up |
| PE(18:2/0:0) | - | 1.1633 | 0.001792 | up |
| Trans-zeatin riboside | HMDB0030388 | 1.2452 | 0.001779 | down |
| (8S,9S,10S,11S,13S,14S,17R)-11,17-Dihydroxy-17-(2-hydroxyacetyl)-10,13-dimethyl-9-nonyl-1,2,6,7,8,11,12,14,15,16-decahydrocyclopenta[a]phenanthren-3-one | HMDB0260197 | 1.6912 | 0.001757 | down |
| Xanthosine | PW_C000203 | 1.0029 | 0.001745 | up |
| Betamethasone 17,21-dipropionate | HMDB0249160 | 1.5034 | 0.001743 | down |
| N-[1'-(Aminocarbonyl)-2',2'-dimethylpropyl]-1-(cyclohexylmethyl)-1h-indazole-3-carboxamide | HMDB0255030 | 1.1492 | 0.001712 | down |
| O-Glutarylcarnitine | LMFA07070091 | 1.5948 | 0.001641 | down |
| 3,5,6-Trihydroxy-5-(hydroxymethyl)-2-methoxy-2-cyclohexen-1-one | HMDB0041031 | 1.1003 | 0.001641 | up |
| PC(18:1(9Z)e/2:0) | HMDB0011148 | 1.7365 | 0.001639 | up |
| 1-Hydroxy-2,2,5,5-tetramethylpyrrolidine-3-carboxamide | HMDB0243894 | 1.1897 | 0.001613 | up |
| 3beta-Hydroxy-17-(1H-imidazol-1-yl)androsta-5,16-diene | HMDB0249948 | 2.5597 | 0.001578 | up |
| Allantoin | HMDB0000462 | 1.2956 | 0.001533 | down |
| Tetraphyllin B | HMDB0029914 | 1.4335 | 0.001525 | up |
| Valylproline | HMDB0029135 | 1.2334 | 0.001512 | up |
| Phenylpropionylglycine | HMDB0000860 | 1.9444 | 0.001488 | up |
| Haloperidol | HMDB0014645 | 1.5716 | 0.001465 | down |
| Afzelechin-(4alpha->8)-pelargonidin 3-O-beta-glucopyranoside | HMDB0302009 | 2.2206 | 0.00146 | down |
| (9S,10S)-9,10-dihydroxyoctadecanoate | HMDB0059633 | 1.2745 | 0.001458 | up |
| Glucosyl (E)-2,6-Dimethyl-2,5-heptadienoate | HMDB0035136 | 2.3527 | 0.001435 | down |
| Bemegride | HMDB0248936 | 1.088 | 0.001424 | up |
| PC(22:6/0:0) | LMGP01050056 | 1.1163 | 0.001288 | up |
| 3,4,5-Trihydroxypentanoylcarnitine | HMDB0241652 | 2.418 | 0.001282 | up |
| 8-Hydroxyoctadecanoylcarnitine | HMDB0241524 | 1.4956 | 0.001233 | down |
| Undecanoic Acid | HMDB0000947 | 1.5076 | 0.001176 | up |
| Acetolein | HMDB0303184 | 1.1521 | 0.001167 | down |
| 9,10-DHOME | HMDB0004704 | 1.4443 | 0.001157 | up |
| Phosphate | HMDB0001429 | 1.7096 | 0.001152 | up |
| LysoPC(18:1(11Z)/0:0) | HMDB0010385 | 1.4558 | 0.00115 | up |
| N-Stearoyl Arginine | HMDB0241937 | 1.0513 | 0.001076 | down |
| Adipic acid | LMFA01170048 | 1.096 | 0.001061 | down |
| LysoPA(16:0/0:0) | HMDB0007853 | 1.0938 | 0.001028 | down |
| Tyrosyl-Proline | HMDB0029113 | 1.1127 | 0.001026 | up |
| LysoPC(22:4(7Z,10Z,13Z,16Z)/0:0) | HMDB0010401 | 1.7151 | 0.000986 | up |
| LysoPE(22:6(4Z,7Z,10Z,13Z,16Z,19Z)/0:0) | HMDB0011526 | 1.0142 | 0.000978 | up |
| 1-Isopropyl-N-((6-methyl-2-oxo-4-propyl-1,2-dihydropyridin-3-yl)methyl)-6-(2-(4-methylpiperazin-1-yl)pyridin-4-yl)-1H-indazole-4-carboxamide | HMDB0252956 | 1.579 | 0.000967 | up |
| Fenofibric acid | HMDB0250346 | 2.6486 | 0.000932 | up |
| Glycerophosphocholine | HMDB0008787 | 1.5633 | 0.000913 | up |
| PC(16:0/18:3(6Z,9Z,12Z)) | PW_C004023 | 1.067 | 0.0009 | up |
| Risbitin | HMDB0302980 | 1.773 | 0.000876 | up |
| Avocadene 2-acetate | LMFA05000641 | 1.414 | 0.000862 | up |
| 8-Amino-7-oxononanoic acid | HMDB0240687 | 1.1125 | 0.000859 | up |
| 14,15-DiHETrE | HMDB0002265 | 1.0774 | 0.000847 | up |
| Mevastatin | HMDB0254692 | 1.9041 | 0.00081 | down |
| AB-Chminaca | HMDB0247738 | 1.162 | 0.0008 | down |
| Allysine | HMDB0303320 | 1.5851 | 0.000793 | up |
| (24E)-3alpha,15alpha-Diacetoxy-23-oxo-7,9(11),24-lanostatrien-26-oic acid | HMDB0035388 | 1.6188 | 0.000769 | up |
| Glycylprolylarginine | HMDB0252828 | 1.8277 | 0.000743 | down |
| 5-Hydroxylysine | HMDB0000450 | 2.2395 | 0.000685 | up |
| Docosahexaenoic Acid | HMDB0002183 | 1.0849 | 0.000663 | up |
| Choline Phosphate | HMDB0001565 | 1.0754 | 0.000621 | up |
| Buprenorphine | PW_C009368 | 1.6701 | 0.000579 | up |
| PE(18:1(9Z)/0:0) | LMGP02050004 | 1.4904 | 0.00056 | up |
| Phosphoenolpyruvic Acid | PW_C000180 | 1.7274 | 0.000552 | down |
| Vulgarole | HMDB0035085 | 1.0759 | 0.000532 | down |
| LysoPI(18:2(9Z,12Z)/0:0) | HMDB0240597 | 1.4045 | 0.000527 | up |
| 2-(S-Glutathionyl)acetyl glutathione | HMDB0060343 | 1.6697 | 0.000513 | down |
| 3-[[(2S)-2,4-Dihydroxy-3,3-dimethylbutanoyl]amino]propanoic acid | HMDB0250782 | 1.9769 | 0.00049 | up |
| SM(d17:2(4E,8Z)/TXB2) | HMDB0290419 | 1.8147 | 0.000488 | up |
| N-lactoyl-Tryptophan | HMDB0062178 | 1.2136 | 0.000486 | up |
| Caprylic acid | HMDB0000482 | 1.111 | 0.000468 | up |
| PC(17:0/0:0) | HMDB0012108 | 1.4795 | 0.000433 | up |
| Valtrate | HMDB0034493 | 1.3068 | 0.000428 | up |
| Isofebrifugine | HMDB0253633 | 1.6356 | 0.000404 | up |
| 2-Hydroxymyristic Acid | HMDB0002261 | 0.997 | 0.0004 | down |
| 7-Aminoheptanoic acid | HMDB0247233 | 1.175 | 0.000388 | up |
| LysoPI(16:0/0:0) | HMDB0061695 | 1.0801 | 0.000387 | up |
| PE(22:6/0:0) | - | 1.1884 | 0.000367 | up |
| Tetradecanedioic acid | HMDB0000872 | 1.2783 | 0.000354 | up |
| LysoPA(18:1(9Z)/0:0) | HMDB0007855 | 1.697 | 0.000334 | up |
| L-Carnitine | PW_C000044 | 1.53 | 0.000291 | down |
| LysoPA(18:2(9Z,12Z)/0:0) | HMDB0007856 | 1.796 | 0.000285 | up |
| 3-Hydroxyanthranilic Acid | PW_C001137 | 2.2558 | 0.000279 | down |
| Benzoin | HMDB0032039 | 1.1998 | 0.000258 | down |
| PE(18:3/0:0) | - | 1.6008 | 0.000256 | up |
| LysoPE(20:3(5Z,8Z,11Z)/0:0) | HMDB0011515 | 1.304 | 0.000246 | up |
| L-Palmitoylcarnitine | LMFA07070004 | 1.9664 | 0.000231 | down |
| Valproic acid glucuronide | HMDB0000901 | 2.4573 | 0.000221 | down |
| Gly Leu | - | 1.6322 | 0.000221 | up |
| 3-Phosphoglycerate | HMDB0000807 | 1.5373 | 0.000214 | down |
| PC(17:2(9Z,12Z)/0:0) | LMGP01050127 | 1.5444 | 0.000202 | up |
| Eplerenone | HMDB0014838 | 1.3273 | 0.000191 | down |
| Pimelic Acid | LMFA01170051 | 1.6013 | 0.000171 | down |
| Myristoleic acid | LMFA01030051 | 1.0208 | 0.000164 | up |
| L-Acetylcarnitine | HMDB0000201 | 1.1786 | 0.00015 | up |
| 3,7,8,15-Scirpenetetrol | HMDB0037560 | 2.2153 | 0.000146 | down |
| Adenosine 3'-monophosphate | HMDB0003540 | 1.4776 | 0.000145 | down |
| Sorbitan laurate | LMFA07011017 | 2.3426 | 0.000144 | up |
| Contignasterol | HMDB0250439 | 1.7043 | 0.000137 | up |
| 3-Oxoadipic acid | HMDB0000398 | 1.0748 | 0.000135 | down |
| 3'-Adenylic Acid | HMDB0258140 | 1.5803 | 0.000134 | down |
| LysoPC(20:5(5Z,8Z,11Z,14Z,17Z)/0:0) | HMDB0010397 | 1.7887 | 0.000131 | up |
| 8-Hydroxyquinoline | - | 1.1608 | 0.000124 | up |
| Hexadecanedioic acid | HMDB0000672 | 1.1052 | 0.000117 | up |
| L-phenylalanyl-L-proline | HMDB0011177 | 1.3137 | 0.000116 | up |
| N-Jasmonoylisoleucine | HMDB0029391 | 1.8489 | 0.000115 | up |
| Streptidine | HMDB0258506 | 1.7664 | 0.000113 | down |
| 13-Hode | HMDB0242324 | 1.3804 | 0.000105 | up |
| Hexanoylglycine | HMDB0000701 | 2.7227 | <0.0001 | down |
| Non-5-enedioylcarnitine | HMDB0241756 | 2.0884 | <0.0001 | down |
| Hydroxyprolyl-Valine | HMDB0028876 | 1.3193 | <0.0001 | up |
| D-Galactaric acid | HMDB0000639 | 1.3197 | <0.0001 | down |
| 2,3-dihydroxy-3-methylvalerate | HMDB0304038 | 1.2814 | <0.0001 | up |
| 13(S)-HODE | - | 1.2466 | <0.0001 | down |
| [(2R,3R,4R)-3,4,5-Trihydroxy-1-oxopentan-2-yl] (2R)-2-amino-3-sulfanylpropanoate | HMDB0260216 | 1.0401 | <0.0001 | up |
| LysoPC(18:0/0:0) | HMDB0010384 | 1.609 | <0.0001 | up |
| Milbemycin beta1 | HMDB0254721 | 1.8562 | <0.0001 | up |
| Glycylprolylhydroxyproline | HMDB0002171 | 2.2348 | <0.0001 | down |
| 9(S)-HpODE | HMDB0006940 | 1.9932 | <0.0001 | up |
| Gamma-Glutamylacetamide | HMDB0252618 | 2.1899 | <0.0001 | down |
| Pro-Pro-Pro | HMDB0256689 | 1.1101 | <0.0001 | up |
| Zanamivir | HMDB0014698 | 2.3862 | <0.0001 | up |
| 4-Guanidinobutanoic acid | HMDB0031842 | 3.0331 | <0.0001 | up |
| PE(16:1/0:0) | - | 1.8089 | <0.0001 | up |
| LysoPI(0:0/18:0) | HMDB0061704 | 1.3534 | <0.0001 | up |
| N-lactoyl-Methionine | HMDB0062182 | 1.2393 | <0.0001 | up |
| Valeric acid | LMFA01010005 | 1.945 | <0.0001 | down |
| (3beta,5alpha,9alpha,22E,24R)-5,9-Epidioxy-3-hydroxyergosta-7,22-dien-6-one | HMDB0032666 | 1.9546 | <0.0001 | up |
| Dihydrozeatin-O-glucoside | HMDB0012214 | 2.1848 | <0.0001 | up |
| Prostaglandin F1a | HMDB0002685 | 1.5522 | <0.0001 | up |
| Hydroxyprolyl-Proline | HMDB0028871 | 1.7619 | <0.0001 | down |
| 4-{[(3S)-3-{[(7-Methoxynaphthalen-2-yl)sulfonyl](methyl)amino}-2-oxopyrrolidin-1-yl]methyl}thiophene-2-carboximidamide | HMDB0257330 | 2.3634 | <0.0001 | down |
| Miroprofen | HMDB0254753 | 2.9252 | <0.0001 | up |
| Caryophyllen-beta | HMDB0304653 | 2.677 | <0.0001 | down |
| LysoPC(16:1(9Z)/0:0) | HMDB0010383 | 1.7363 | <0.0001 | up |
| Pyroglutamyl-glutamyl-proline amide | HMDB0247524 | 1.8977 | <0.0001 | up |
| Val Asp Ile | - | 1.9957 | <0.0001 | up |
| Xi-7-Hydroxyhexadecanedioic acid | HMDB0037830 | 2.9328 | <0.0001 | up |
| PC(15:0/0:0) | LMGP01050016 | 1.7452 | <0.0001 | up |
| Cycloheximide | HMDB0250657 | 1.2894 | <0.0001 | up |
| 3-methylheptanedioylcarnitine | HMDB0241046 | 2.3356 | <0.0001 | down |
| Menthyl pyrrolidone carboxylate | HMDB0032368 | 3.1345 | <0.0001 | up |
| LysoPC(18:3(6Z,9Z,12Z)/0:0) | HMDB0010387 | 1.7216 | <0.0001 | up |
| Ineketone | HMDB0036698 | 1.831 | <0.0001 | down |
| 1,8-Heptadecadiene-4,6-diyne-3,10-diol | LMFA05000584 | 2.9956 | <0.0001 | up |
| Cotinine glucuronide | HMDB0001013 | 2.5824 | <0.0001 | down |
| Cyclohexanecarboxylic acid | HMDB0031342 | 1.4986 | <0.0001 | up |
| Allopregnanolone | HMDB0001449 | 1.4305 | <0.0001 | up |
| Dihydrotestosterone | PW_C001745 | 1.337 | <0.0001 | up |
| D-Ribose 1-phosphate | HMDB0001489 | 1.7518 | <0.0001 | down |
| Ruscogenin | HMDB0257364 | 1.7285 | <0.0001 | down |
| LysoPE(20:0/0:0) | HMDB0011511 | 1.5708 | <0.0001 | up |
| 3-Hydroxyhexanedioylcarnitine | HMDB0241674 | 2.6433 | <0.0001 | down |
| Gly Val Leu | - | 2.1581 | <0.0001 | up |
| 1-Heptadecanoylglycerophosphoethanolamine | HMDB0061691 | 1.8109 | <0.0001 | up |
| N-Lactoylphenylalanine | HMDB0062175 | 1.7426 | <0.0001 | up |
| Taxiphyllin | HMDB0030704 | 2.3161 | <0.0001 | down |
| PC(17:1/0:0) | - | 2.027 | <0.0001 | up |
| 3-Hydroxypropyl methacrylate | HMDB0245905 | 1.9249 | <0.0001 | down |
| LysoPC(18:4(6Z,9Z,12Z,15Z)/0:0) | HMDB0010389 | 2.9763 | <0.0001 | up |
| (2Z)-5-Hydroxydec-2-enedioylcarnitine | HMDB0241092 | 2.3614 | <0.0001 | down |
| Amastatin | HMDB0248277 | 1.6547 | <0.0001 | up |
| Gly-Pro-Arg-Pro-Lys | HMDB0252830 | 2.4646 | <0.0001 | up |
| 3-Mercaptohexyl hexanoate | HMDB0037766 | 1.1143 | <0.0001 | down |
| Guanosine diphosphate adenosine | HMDB0001501 | 2.2554 | <0.0001 | down |
| Xi-8-Hydroxyhexadecanedioic acid | HMDB0037831 | 1.4522 | <0.0001 | up |
| Pyridine-3,4-diol | HMDB0256965 | 1.0401 | <0.0001 | down |
| LysoPC(14:0/0:0) | HMDB0010379 | 1.9708 | <0.0001 | up |
| Cellobioside | HMDB0249793 | 2.0483 | <0.0001 | up |
| LysoPC(0:0/16:0) | HMDB0240262 | 1.6135 | <0.0001 | up |
| Alprostadil | HMDB0001442 | 1.8098 | <0.0001 | up |
| (12Z)-10-Hydroxyoctadecenoylcarnitine | HMDB0241535 | 1.8485 | <0.0001 | down |
| Gly Gly Leu | - | 1.6594 | <0.0001 | up |
| Leu-Gly-Gly | - | 1.5312 | <0.0001 | up |
| Deca-2,5,8-trienedioylcarnitine | HMDB0241128 | 2.8276 | <0.0001 | down |
| (2S)-2-[[(2R,3S,4R,5R)-5-(6-Aminopurin-9-yl)-3,4-dihydroxyoxolan-2-yl]methylamino]-4-sulfanylbutanoic acid | HMDB0258158 | 1.464 | <0.0001 | up |
| (6Z)-Oct-6-enedioylcarnitine | HMDB0241707 | 2.4855 | <0.0001 | down |
| 2',3'-Dideoxyadenosine | HMDB0245544 | 1.1078 | <0.0001 | up |
| DL-Ethionine | HMDB0251518 | 2.012 | <0.0001 | up |
| Palmitoyl-L-carnitine | HMDB0000222 | 2.6634 | <0.0001 | down |
| Palmitoylcarnitine | HMDB0240783 | 1.9822 | <0.0001 | down |
| METHACHOLINE | HMDB0015654 | 1.8699 | <0.0001 | down |
| Saxitoxin | HMDB0029368 | 2.1636 | <0.0001 | down |
| Tryptophyl-Gamma-glutamate | HMDB0029097 | 2.9449 | <0.0001 | down |
| Deoxynivalenol | HMDB0036156 | 2.9017 | <0.0001 | down |
| Octanoylcarnitine | LMFA07070095 | 3.8984 | <0.0001 | down |
| Ethyl-4-hydroxymethyl-3(2H)-Furanone | - | 2.4643 | <0.0001 | down |
| Hex-3-enedioylcarnitine | HMDB0241671 | 2.8102 | <0.0001 | down |

VIP: variable importance on projection.

**Table S4C.** Identified differentially expressed metabolites in sham and I1R48 groups.

| **Metabolite** | **Library ID** | **VIP** | **P-value** | **Regulate** |
| --- | --- | --- | --- | --- |
| Luvangetin | - | 1.4847 | 0.04871 | up |
| Imidazoleacetic acid ribotide | HMDB0006032 | 0.9856 | 0.04617 | up |
| Fenvalerate | HMDB0031791 | 1.0483 | 0.04559 | down |
| 19-Nor-5-androstenediol | LMST02010050 | 1.3639 | 0.04457 | down |
| S-Hydroxymethylglutathione | HMDB0004662 | 1.3157 | 0.04455 | down |
| Norhygrine | HMDB0302903 | 1.0131 | 0.04427 | up |
| Diisononyl phthalate | HMDB0251352 | 1.2673 | 0.04422 | down |
| Cucurbic acid | HMDB0029388 | 1.0209 | 0.04364 | up |
| Phosphoenolpyruvic Acid | PW_C000180 | 1.3224 | 0.04262 | down |
| Adenylosuccinate | HMDB0000536 | 1.3357 | 0.04204 | down |
| Glucosyl (2E,6E,10x)-10,11-dihydroxy-2,6-farnesadienoate | HMDB0037823 | 1.4273 | 0.042 | up |
| (+/-)10-HDoHE | - | 1.6385 | 0.04157 | up |
| Ubrogepant | HMDB0304882 | 1.3149 | 0.04096 | up |
| Propyl 2,4-decadienoate | LMFA07011001 | 1.4586 | 0.0387 | up |
| Phe Gly | - | 1.2021 | 0.03777 | up |
| 2,3-dinor Prostaglandin E1 | - | 1.3013 | 0.03694 | up |
| Galactaric acid | LMFA01170107 | 1.0973 | 0.03679 | down |
| L-Proline, 1-(1-L-leucyl-L-prolyl)- | HMDB0253022 | 1.1595 | 0.03537 | up |
| N1-(5-Phospho-a-D-ribosyl)-5,6-dimethylbenzimidazole | HMDB0003882 | 1.3844 | 0.03491 | down |
| Antimycin A | HMDB0248488 | 2.6162 | 0.03355 | up |
| 2-Hydroxyphenylacetic Acid | HMDB0000669 | 0.9863 | 0.03218 | up |
| 4-Vinylphenol sulfate | HMDB0062775 | 1.3702 | 0.03153 | up |
| Heptanoic acid | LMFA01010007 | 1.3495 | 0.03127 | up |
| Valylisoleucine | HMDB0029130 | 1.0979 | 0.02971 | up |
| Furanodienone | HMDB0036768 | 1.1696 | 0.02951 | up |
| 3'-Hydroxy-T2-triol | HMDB0034575 | 1.1918 | 0.02796 | up |
| Methylsuccinic acid | LMFA01170119 | 1.8579 | 0.02713 | down |
| 1,3-Propanediol, 2,2-diethyl-, dicarbamate | HMDB0254691 | 1.0993 | 0.02607 | up |
| 20-Hydroxyeicosatetraenoic acid | PW_C002614 | 1.2245 | 0.02557 | down |
| Prostaglandin F1a | HMDB0002685 | 1.5527 | 0.02544 | up |
| 3,4-Dihydroxy-2-methoxy-4-methyl-3-[2-methyl-3-(3-methyl-but-2-enyl)-oxiranyl]-cyclohexanone | HMDB0246031 | 1.3486 | 0.02522 | up |
| Arginyl-prolyl-proline | HMDB0249363 | 1.1838 | 0.02464 | up |
| Gamma-Glutamylthreonine | HMDB0029159 | 1.0838 | 0.02433 | down |
| Glycylprolylhydroxyproline | HMDB0002171 | 1.2088 | 0.02402 | down |
| Allysine | HMDB0303320 | 1.1191 | 0.02312 | up |
| 12,13-DHOME | HMDB0004705 | 1.1465 | 0.02301 | down |
| 14,15-DiHETrE | HMDB0002265 | 0.9575 | 0.02293 | up |
| 9(S)-HODE | HMDB0004670 | 1.4079 | 0.02231 | up |
| Folinic acid | HMDB0247218 | 1.6774 | 0.02229 | up |
| LysoPI(16:0/0:0) | HMDB0061695 | 0.9876 | 0.02221 | up |
| 4-Hydroxy Triamterene | HMDB0246440 | 1.3935 | 0.02184 | down |
| Cortolone | LMST02030227 | 1.6283 | 0.02157 | down |
| Undecanoic Acid | HMDB0000947 | 1.4578 | 0.02133 | up |
| N-Palmitoyl Glycine | LMFA08020079 | 1.1897 | 0.02119 | up |
| Dhesn | HMDB0251109 | 1.2764 | 0.02084 | down |
| 2-[[3-Cyclohexyl-1-[2-[3-(diaminomethylideneamino)propylcarbamoyl]piperidin-1-yl]-1-oxopropan-2-yl]amino]acetic acid | HMDB0247862 | 1.3598 | 0.02081 | up |
| 15-keto-PGE1 | LMFA03010146 | 2.3123 | 0.02058 | up |
| N-Acetyl-L-Tyrosine | HMDB0000866 | 1.0397 | 0.02058 | up |
| 12-oxo-PDA | LMFA02010001 | 1.7549 | 0.02041 | down |
| 6,8-Dihydroxypurine | HMDB0001182 | 0.9293 | 0.02025 | up |
| (3Z)-2-Propylpent-3-enoic acid | HMDB0013903 | 1.2495 | 0.01999 | up |
| Cysteine-glutathione disulfide | HMDB0000656 | 1.1653 | 0.01999 | up |
| Citric Acid | PW_C000063 | 1.3496 | 0.01989 | down |
| Ala-phe | HMDB0028694 | 1.2455 | 0.01911 | up |
| LysoPI(18:0/0:0) | HMDB0240261 | 0.966 | 0.01878 | up |
| Octanoylcarnitine | LMFA07070095 | 1.8658 | 0.01871 | down |
| Taurallocholic acid | HMDB0000922 | 1.7408 | 0.01855 | down |
| (9S,10S)-9,10-dihydroxyoctadecanoate | HMDB0059633 | 1.1204 | 0.01814 | up |
| Yohimbine | HMDB0015464 | 2.6924 | 0.01743 | up |
| Taxiphyllin | HMDB0030704 | 1.08 | 0.01729 | down |
| 3,5,6-Trihydroxy-5-(hydroxymethyl)-2-methoxy-2-cyclohexen-1-one | HMDB0041031 | 1.0691 | 0.01653 | up |
| 6''-O-Acetylgenistin | HMDB0029528 | 1.0276 | 0.01631 | down |
| N-Palmitoyl Valine | LMFA08020120 | 1.5793 | 0.01618 | up |
| Jasmolone | HMDB0030039 | 1.2805 | 0.01579 | down |
| (3beta,5alpha,9alpha,22E,24R)-5,9-Epidioxy-3-hydroxyergosta-7,22-dien-6-one | HMDB0032666 | 1.2768 | 0.01502 | up |
| EMycin E | HMDB0251782 | 0.9886 | 0.01479 | up |
| 6-(2-Hydroxyethoxy)-6-oxohexanoic acid | HMDB0061681 | 1.0144 | 0.0147 | up |
| (6R)-5,10-Methylenetetrahydrofolate | HMDB0001533 | 1.408 | 0.01462 | up |
| Deoxycholic Acid | PW_C000487 | 1.3592 | 0.01462 | up |
| UDP-D-Galactose | HMDB0000302 | 1.9229 | 0.01446 | down |
| Octhilinone | - | 1.8655 | 0.01417 | down |
| Bisorganyltrisulfane | HMDB0304276 | 1.6404 | 0.01412 | up |
| 3-[[(2S)-2,4-Dihydroxy-3,3-dimethylbutanoyl]amino]propanoic acid | HMDB0250782 | 1.3395 | 0.01405 | up |
| Kynurenine | HMDB0000684 | 1.6159 | 0.01392 | up |
| Alanine lactate pyruvate | HMDB0248105 | 1.0113 | 0.01377 | up |
| 3beta-Hydroxy-17-(1H-imidazol-1-yl)androsta-5,16-diene | HMDB0249948 | 1.7629 | 0.01365 | up |
| Hyodeoxycholic acid | HMDB0000811 | 1.1814 | 0.01356 | up |
| Alprostadil | HMDB0001442 | 1.7278 | 0.01355 | up |
| Naepaine | HMDB0255417 | 1.1504 | 0.01344 | up |
| 15H-11,12-EETA | PW_C002389 | 1.5313 | 0.01333 | up |
| Indicaxanthin | HMDB0304729 | 0.9754 | 0.01328 | up |
| Avocadene 2-acetate | LMFA05000641 | 1.1001 | 0.01323 | up |
| Allopregnanolone | HMDB0001449 | 1.1388 | 0.01262 | up |
| 4-Hydroxynonenal | HMDB0245269 | 1.234 | 0.01239 | up |
| Leucylhydroxyproline | HMDB0028930 | 0.9621 | 0.01213 | up |
| Dodecanedioic Acid | LMFA01170009 | 1.5717 | 0.01198 | up |
| 1-(11Z-eicosenoyl)-glycero-3-phosphate | LMGP10050026 | 0.9863 | 0.0115 | up |
| NH-DVal(NMe)-Val-OMe | - | 1.3037 | 0.0114 | up |
| Domoic acid | HMDB0033939 | 1.1647 | 0.01124 | up |
| Gabazine | HMDB0252568 | 1.0768 | 0.01119 | up |
| Melleolide | HMDB0035689 | 1.2062 | 0.01115 | up |
| (+/-)-Pelletierine | HMDB0030325 | 1.0262 | 0.01085 | up |
| 1-arachidonoyl-2-hydroxy-sn-glycero-3-phosphate | - | 1.0197 | 0.01017 | up |
| N-Palmitoyl Threonine | HMDB0241933 | 1.3389 | 0.01016 | up |
| N6-(1,2-Dicarboxyethyl)-AMP | - | 1.7034 | 0.00961 | down |
| LysoPE(P-18:1(9Z)/0:0) | HMDB0240599 | 1.791 | 0.009591 | up |
| Hydroxyprolyl-Proline | HMDB0028871 | 1.1412 | 0.00956 | down |
| Linolenic Acid | HMDB0001388 | 1.2266 | 0.00948 | down |
| L-Palmitoylcarnitine | LMFA07070004 | 1.3074 | 0.009463 | down |
| LysoPC(16:0/0:0) | HMDB0010382 | 1.0484 | 0.008922 | up |
| 9,10,13-TriHOME | HMDB0004710 | 1.6422 | 0.008847 | up |
| 3-Hydroxyanthranilic Acid | PW_C001137 | 1.4485 | 0.0085 | down |
| Bemegride | HMDB0248936 | 1.0839 | 0.008244 | up |
| Hexadecanedioic acid | HMDB0000672 | 1.0215 | 0.008222 | up |
| Cholic acid glucuronide | HMDB0002577 | 0.9987 | 0.008045 | up |
| Gamma-Glutamyltyrosine | HMDB0011741 | 1.4985 | 0.007996 | up |
| Risbitin | HMDB0302980 | 1.6182 | 0.007933 | up |
| N-Docosahexaenoyl Methionine | HMDB0242019 | 1.467 | 0.007932 | up |
| 4-Octylphenol | HMDB0246557 | 1.3155 | 0.007915 | down |
| Demethylphylloquinone | HMDB0004649 | 1.1322 | 0.00766 | up |
| Pimelic Acid | LMFA01170051 | 1.1392 | 0.007575 | down |
| Butyryl-L-carnitine | HMDB0002013 | 1.9446 | 0.007542 | down |
| Xanthosine | PW_C000203 | 0.9283 | 0.007196 | up |
| Ascorbic Acid | HMDB0000044 | 2.4803 | 0.00712 | down |
| Blennin D | HMDB0031902 | 1.3138 | 0.007103 | down |
| Alfaprostolum | HMDB0248132 | 2.0205 | 0.00689 | up |
| (6Z)-Oct-6-enedioylcarnitine | HMDB0241707 | 1.2623 | 0.006603 | down |
| Palmitic Acid ethyl ester | HMDB0029811 | 1.3036 | 0.006483 | up |
| 1,8-Heptadecadiene-4,6-diyne-3,10-diol | LMFA05000584 | 1.9184 | 0.006292 | up |
| Linoelaidic acid | HMDB0247706 | 0.9964 | 0.006244 | down |
| (Z)-Farnesol | HMDB0059849 | 1.2918 | 0.006197 | up |
| Adenosine monophosphate | PW_C000032 | 1.2168 | 0.006051 | down |
| (12Z)-10-Hydroxyoctadecenoylcarnitine | HMDB0241535 | 1.0656 | 0.006034 | down |
| D-Erythrose-4-Phosphate | HMDB0001321 | 2.1599 | 0.005833 | down |
| Amastatin | HMDB0248277 | 1.0734 | 0.005638 | up |
| LysoPS(18:0/0:0) | HMDB0240606 | 1.1213 | 0.005516 | up |
| Cyclohexanecarboxylic acid | HMDB0031342 | 1.4301 | 0.005296 | up |
| ACEXAMIC ACID | HMDB0247031 | 1.5534 | 0.005284 | up |
| LysoPA(20:5(5Z,8Z,11Z,14Z,17Z)/0:0) | HMDB0114748 | 1.5083 | 0.005251 | up |
| Gamma-Glutamylacetamide | HMDB0252618 | 1.3649 | 0.005235 | down |
| 2-Phenyl-1,3-propanediol monocarbamate | PW_C040528 | 1.607 | 0.00519 | up |
| LysoPC(17:0/0:0) | HMDB0012108 | 1.2402 | 0.005139 | up |
| (6E,8R,10Z)-8-hydroxy-3-oxohexadecadienoic acid | HMDB0062365 | 1.3588 | 0.005091 | up |
| Deoxynivalenol | HMDB0036156 | 1.5331 | 0.005023 | down |
| Tetradecanedioic acid | HMDB0000872 | 1.203 | 0.004966 | up |
| N-Palmitoyl Leucine | LMFA08020115 | 1.8101 | 0.004898 | up |
| PI(20:5(5Z,8Z,11Z,14Z,17Z)/0:0) | HMDB0256524 | 1.3172 | 0.004724 | up |
| (2S)-2-[[(2R,3S,4R,5R)-5-(6-Aminopurin-9-yl)-3,4-dihydroxyoxolan-2-yl]methylamino]-4-sulfanylbutanoic acid | HMDB0258158 | 0.9728 | 0.004664 | up |
| PA(8:0/20:4(5Z,8Z,11Z,14Z)-OH(19S)) | HMDB0266595 | 1.0772 | 0.004619 | up |
| Salmeterol | HMDB0015073 | 1.4251 | 0.004589 | up |
| Linoleoyl ethanolamide | HMDB0012252 | 1.4606 | 0.004583 | up |
| 5'-Guanylic Acid | HMDB0001397 | 1.4469 | 0.004519 | up |
| 9(S)-HpOTrE | - | 1.0551 | 0.004465 | up |
| 9,10-Epoxy-18-hydroxy-octadecanoic acid | HMDB0302107 | 1.337 | 0.004201 | up |
| LysoPC(0:0/18:0) | HMDB0011128 | 1.2417 | 0.004178 | up |
| Ethyl butylacetylaminopropionate | HMDB0246904 | 1.3189 | 0.004128 | down |
| 9(S)-HOTrE | HMDB0247705 | 1.8801 | 0.004019 | down |
| 2-Undecen-1-ol | HMDB0034856 | 1.4747 | 0.00397 | down |
| 1-Hydroxy-2,2,5,5-tetramethylpyrrolidine-3-carboxamide | HMDB0243894 | 1.2059 | 0.003964 | up |
| Hygromycin B | HMDB0034229 | 2.654 | 0.003874 | down |
| 9,10-DHOME | HMDB0004704 | 1.8242 | 0.003859 | up |
| Eicosapentaenoic Acid | PW_C001337 | 1.2697 | 0.003807 | up |
| CE(PGE2) | HMDB0290213 | 1.2992 | 0.003429 | down |
| 4-Guanidinobutanoic acid | HMDB0031842 | 2.1577 | 0.003408 | up |
| SAICAR | HMDB0240295 | 2.0416 | 0.003365 | up |
| Notoginsenoside R1 | HMDB0035363 | 1.37 | 0.00335 | up |
| 13(S)-HpODE | HMDB0003871 | 1.4332 | 0.003321 | up |
| Beta-Ionone | HMDB0036565 | 1.5782 | 0.003138 | down |
| (4E,7E,10Z,13E,16E,19E)-docosa-4,7,10,13,16,19-hexaenoic acid | HMDB0251558 | 1.4216 | 0.002918 | up |
| 1-Nonanol | LMFA05000092 | 1.3053 | 0.002864 | down |
| N-lactoyl-phenylalanine | HMDB0062175 | 1.0615 | 0.002851 | up |
| LysoPC(20:4(8Z,11Z,14Z,17Z)/0:0) | HMDB0010396 | 1.201 | 0.002553 | up |
| L-Carnitine | PW_C000044 | 1.025 | 0.002526 | down |
| Cropropamide | HMDB0250543 | 0.908 | 0.002524 | up |
| LysoPI(18:1(9Z)/0:0) | HMDB0061693 | 1.399 | 0.002478 | up |
| Palmitoylglycine | HMDB0013034 | 1.6527 | 0.002349 | up |
| Panthenol | HMDB0004231 | 1.5024 | 0.002292 | up |
| PS(6 keto-PGF1alpha/14:1(9Z)) | HMDB0280931 | 1.5627 | 0.00217 | up |
| Dethiobiotin | HMDB0003581 | 1.0776 | 0.002121 | up |
| Phenylacetylglutamine | HMDB0006344 | 1.0833 | 0.002081 | up |
| 1-Isopropyl-N-((6-methyl-2-oxo-4-propyl-1,2-dihydropyridin-3-yl)methyl)-6-(2-(4-methylpiperazin-1-yl)pyridin-4-yl)-1H-indazole-4-carboxamide | HMDB0252956 | 1.502 | 0.001968 | up |
| Indole-3-acetaldehyde | HMDB0001190 | 1.0455 | 0.00196 | down |
| LysoPE(P-18:0/0:0) | HMDB0240598 | 1.9259 | 0.001929 | up |
| (2Z)-5-Hydroxydec-2-enedioylcarnitine | HMDB0241092 | 1.3204 | 0.001877 | down |
| 3-Ethoxypropanoic Acid | - | 0.9431 | 0.001799 | up |
| 2,3-dihydroxy-3-methylvalerate | HMDB0304038 | 0.993 | 0.001734 | up |
| Isovalerylglycine | HMDB0000678 | 1.3412 | 0.001731 | up |
| N-Acetyl-L-phenylalanine | HMDB0000512 | 1.2862 | 0.0017 | up |
| Tetraphyllin B | HMDB0029914 | 1.4633 | 0.001637 | up |
| (24E)-3alpha,15alpha-Diacetoxy-23-oxo-7,9(11),24-lanostatrien-26-oic acid | HMDB0035388 | 1.5905 | 0.001557 | up |
| 13-Hode | HMDB0242324 | 1.4226 | 0.001514 | up |
| PE(20:4/0:0) | LMGP02050009 | 1.2671 | 0.001509 | up |
| N-Eicosapentaenoyl Serine | HMDB0242079 | 1.741 | 0.00149 | up |
| N-Arachidonoyl-L-Serine | - | 1.6611 | 0.001445 | up |
| Menthyl pyrrolidone carboxylate | HMDB0032368 | 2.3447 | 0.001431 | up |
| 9(S)-HpODE | HMDB0006940 | 2.1255 | 0.001375 | up |
| Eltoprazine | HMDB0251757 | 1.9264 | 0.001275 | up |
| Alpha-Ionone | HMDB0059883 | 1.3697 | 0.001198 | down |
| Xi-8-Hydroxyhexadecanedioic acid | HMDB0037831 | 1.2618 | 0.001178 | up |
| PI(20:0/PGE1) | HMDB0277055 | 1.9732 | 0.00107 | up |
| Bufadienolide | HMDB0249427 | 1.639 | 0.00107 | up |
| 3'-Adenylic Acid | HMDB0258140 | 1.3302 | 0.001035 | down |
| D-manno-2-Heptulose | HMDB0029935 | 1.0075 | 0.001027 | down |
| L-Acetylcarnitine | HMDB0000201 | 1.0231 | 0.001023 | up |
| Subaphylline | HMDB0033463 | 2.0216 | 0.001017 | up |
| Sorbitan laurate | LMFA07011017 | 2.1322 | 0.00101 | up |
| 2,5-Dimethylbenzaldehyde | HMDB0032014 | 1.2062 | 0.000958 | down |
| N-Eicosapentaenoyl Valine | HMDB0242083 | 1.9798 | 0.000847 | up |
| P-Menthane-3,8-diol | HMDB0036145 | 1.4306 | 0.000803 | down |
| 1-(4Z,7Z,10Z,13Z,16Z,19Z-docosahexaenoyl)-glycero-3-phosphate | LMGP10050019 | 1.2698 | 0.000784 | up |
| Arecoline | HMDB0030353 | 2.1896 | 0.000738 | up |
| D-Ribose 1-phosphate | HMDB0001489 | 1.327 | 0.000685 | down |
| Ginnalin B | HMDB0252723 | 1.3762 | 0.000676 | up |
| Palmitoyl-L-carnitine | HMDB0000222 | 1.7749 | 0.000674 | down |
| Arachidonic acid | PW_C000821 | 1.2725 | 0.000666 | up |
| L-phenylalanyl-L-proline | HMDB0011177 | 1.0762 | 0.000653 | up |
| Phosphate | HMDB0001429 | 1.7676 | 0.000632 | up |
| Betamethasone 17,21-dipropionate | HMDB0249160 | 1.5605 | 0.000621 | down |
| 1-Carboxyethyltyrosine | HMDB0242159 | 1.153 | 0.00061 | up |
| Retinyl ester | PW_C001935 | 1.6857 | 0.000583 | up |
| Alpha-Linolenoyl ethanolamide | HMDB0013624 | 1.7939 | 0.000564 | up |
| Oxyphencyclimine | HMDB0014527 | 1.0592 | 0.000561 | up |
| Biochanin A | LMPK12050229 | 1.4336 | 0.000555 | up |
| Clomoxir | HMDB0250351 | 2.101 | 0.000543 | down |
| Versalide | HMDB0259795 | 1.8524 | 0.000532 | down |
| Guanosine diphosphate adenosine | HMDB0001501 | 1.8209 | 0.00053 | down |
| Palmitoylcarnitine | HMDB0240783 | 1.3463 | 0.000527 | down |
| Prolyl-Alanine | HMDB0029010 | 1.2562 | 0.000505 | down |
| LysoPC(P-18:0/0:0) | HMDB0013122 | 1.3085 | 0.000499 | up |
| Sn-glycero-3-Phosphoethanolamine | HMDB0000114 | 1.3514 | 0.000497 | up |
| Ruscogenin | HMDB0257364 | 1.1142 | 0.00049 | down |
| Taurocholic acid 3-sulfate | LMST05020031 | 1.3368 | 0.000486 | down |
| 1-Nonadecanoyl-glycero-3-phosphoethanolamine | HMDB0243971 | 1.4961 | 0.000476 | up |
| Ile-Glu-Thr-Asp-fluoromethyl ketone | HMDB0253396 | 1.584 | 0.000473 | up |
| 12-Hydroxydodecanoylcarnitine | HMDB0241208 | 2.0938 | 0.000455 | up |
| PS(22:4(7Z,10Z,13Z,16Z)/PGJ2) | HMDB0283220 | 1.8747 | 0.000433 | down |
| Gly Leu | - | 1.8831 | 0.000431 | up |
| N-Palmitoyl Aspartic acid | HMDB0241922 | 1.8265 | 0.000409 | down |
| D-(+)-dihydrocarvone | HMDB0302236 | 1.424 | 0.000406 | down |
| Milbemycin beta1 | HMDB0254721 | 1.7593 | 0.000393 | up |
| Xi-7-Hydroxyhexadecanedioic acid | HMDB0037830 | 2.5134 | 0.000385 | up |
| 8,11-eicosadiynoic acid | LMFA01030688 | 1.5685 | 0.000374 | up |
| Chrysin-7-O-Glucuronide | - | 2.156 | 0.000358 | down |
| LysoPE(20:5(5Z,8Z,11Z,14Z,17Z)/0:0) | HMDB0011519 | 1.5782 | 0.000357 | up |
| LysoPC(20:5(5Z,8Z,11Z,14Z,17Z)/0:0) | HMDB0010397 | 1.4833 | 0.000354 | up |
| 2beta-Hydroxytestosterone | HMDB0012654 | 1.6967 | 0.000353 | up |
| Cis-Quinceoxepane | HMDB0038108 | 1.1673 | 0.000352 | down |
| Sphingosine | LMSP01010001 | 1.0987 | 0.000349 | down |
| (2S)-2-Hydroxyhexadecanoylcarnitine | HMDB0241459 | 1.4108 | 0.00033 | up |
| Gly Gly Leu | - | 1.6235 | 0.000325 | up |
| Vomifoliol | HMDB0303570 | 1.4559 | 0.000314 | up |
| 8(R)-Hydroperoxylinoleic acid | HMDB0004706 | 1.8595 | 0.000289 | down |
| 3-ketosphingosine | LMSP01010002 | 1.7409 | 0.000283 | down |
| Dihomolinoleic acid | LMFA01031043 | 0.9954 | 0.000283 | up |
| 5'-Methylthioadenosine | PW_C000910 | 1.6121 | 0.000268 | down |
| Leucopelargonidin | HMDB0032322 | 1.8869 | 0.000265 | up |
| PE(16:0/0:0) | HMDB0011503 | 1.0187 | 0.000263 | up |
| PA(10:0/20:5(7Z,9Z,11E,13E,17Z)-3OH(5,6,15)) | HMDB0262713 | 1.2821 | 0.000259 | up |
| 1-Stearoylglycerophosphoglycerol | HMDB0061697 | 2.4573 | 0.000253 | up |
| PI(20:4(5Z,8Z,11Z,14Z)/0:0) | HMDB0061690 | 1.4062 | 0.000251 | up |
| Linoleic Acid | HMDB0247706 | 1.369 | 0.00023 | up |
| Rocuronium | HMDB0014866 | 1.4868 | 0.000227 | up |
| METENEPROST | HMDB0254509 | 1.4361 | 0.000219 | up |
| PC(16:0/0:0) | LMGP01050018 | 1.1174 | 0.000195 | up |
| LysoPE(20:0/0:0) | HMDB0011511 | 1.8043 | 0.000193 | up |
| Isofebrifugine | HMDB0253633 | 1.4503 | 0.000174 | up |
| PE(18:0/0:0) | LMGP02050001 | 1.6184 | 0.000173 | up |
| LysoPI(18:2(9Z,12Z)/0:0) | HMDB0240597 | 1.7805 | 0.000156 | up |
| S-(2-Carboxyethyl)-L-cysteine | HMDB0246645 | 2.3083 | 0.000152 | down |
| (3beta,5alpha,6beta,9alpha,22E,24R)-23-Methylergosta-7,22-diene-3,5,6,9-tetrol | HMDB0033634 | 1.2031 | 0.000152 | up |
| 1,2,10-Trihydroxydihydro-trans-linalyl oxide 7-O-beta-D-glucopyranoside | HMDB0033237 | 1.6105 | 0.000145 | up |
| (2S,3R)-3-hydroxy-2-methylpentanedioylcarnitine | HMDB0241035 | 2.1424 | 0.000143 | down |
| Dihomo-gamma-linolenic acid | HMDB0002925 | 2.356 | 0.000141 | up |
| Penitrem D | HMDB0256240 | 1.4198 | 0.000137 | up |
| LysoPE(20:4(8Z,11Z,14Z,17Z)/0:0) | HMDB0011518 | 1.5952 | 0.000136 | up |
| 1-Heptadecanoylglycerophosphoethanolamine | HMDB0061691 | 1.9145 | 0.000121 | up |
| Jasmine ketolactone | HMDB0030150 | 1.3362 | 0.000104 | down |
| Eriojaposide B | HMDB0038029 | 1.893 | <0.0001 | up |
| PE(15:0/0:0) | LMGP02050031 | 1.6918 | <0.0001 | up |
| Acetolein | HMDB0303184 | 1.2938 | <0.0001 | down |
| LysoPI(0:0/18:0) | HMDB0061704 | 1.3342 | <0.0001 | up |
| Gly-Pro-Arg-Pro-Lys | HMDB0252830 | 2.6111 | <0.0001 | up |
| LysoPC(18:3(6Z,9Z,12Z)/0:0) | HMDB0010387 | 1.5884 | <0.0001 | up |
| Tryptophyl-Gamma-glutamate | HMDB0029097 | 2.0482 | <0.0001 | down |
| Esmolol | HMDB0014333 | 1.4741 | <0.0001 | up |
| 3-Hydroxyhexanedioylcarnitine | HMDB0241674 | 2.1338 | <0.0001 | down |
| N-lactoyl-Tryptophan | HMDB0062178 | 1.2775 | <0.0001 | up |
| PC(22:6(4Z,7Z,10Z,13Z,16Z,19Z)/18:0) | PW_C004775 | 1.4582 | <0.0001 | down |
| LysoPE(0:0/22:5(7Z,10Z,13Z,16Z,19Z)) | HMDB0011495 | 1.6962 | <0.0001 | up |
| Pantothenic Acid | PW_C000136 | 1.0469 | <0.0001 | up |
| Pc(18:0/0:0) | LMGP01050026 | 1.8787 | <0.0001 | up |
| PC(LTE4/22:1(13Z)) | HMDB0287902 | 1.6729 | <0.0001 | up |
| LysoPE(0:0/20:4(5Z,8Z,11Z,14Z)) | HMDB0011487 | 1.0043 | <0.0001 | up |
| LysoPC(20:4(5Z,8Z,11Z,14Z)/0:0) | HMDB0010395 | 2.1055 | <0.0001 | up |
| Gemeprost | HMDB0252671 | 1.5859 | <0.0001 | up |
| PE(18:2/0:0) | - | 1.7141 | <0.0001 | up |
| Streptidine | HMDB0258506 | 1.5383 | <0.0001 | down |
| LysoPA(20:4(8Z,11Z,14Z,17Z)/0:0) | HMDB0114742 | 1.4666 | <0.0001 | up |
| LysoPC(15:0/0:0) | HMDB0010375 | 1.4001 | <0.0001 | up |
| [3-[2,3-Dihydroxypropoxy(hydroxy)phosphoryl]oxy-2-hydroxypropyl] hexadecanoate | HMDB0242118 | 1.6401 | <0.0001 | up |
| 7-[(1R,2R,3R)-3-Hydroxy-2-[(3S)-3-hydroxyoctyl]-5-oxocyclopentyl]heptanoylcarnitine | HMDB0241874 | 2.22 | <0.0001 | up |
| PE(22:6/0:0) | - | 1.6172 | <0.0001 | up |
| Docosahexaenoic Acid | HMDB0002183 | 1.5666 | <0.0001 | up |
| (3E,5E)-1,3,5-octatriene | HMDB0303832 | 1.2167 | <0.0001 | down |
| LysoPE(20:3(5Z,8Z,11Z)/0:0) | HMDB0011515 | 2.2632 | <0.0001 | up |
| N-Lactoylphenylalanine | HMDB0062175 | 1.929 | <0.0001 | up |
| (8R,9R,10S,13R,14S)-1,2,3,4,5,6,7,8,9,10,11,12,14,15,16,17-Hexadecahydrocyclopenta[a]phenanthrene-13-carbaldehyde | HMDB0260168 | 1.3892 | <0.0001 | down |
| PE(18:3/0:0) | - | 1.792 | <0.0001 | up |
| (2-Hydroxy-3-phosphonooxypropyl) octadec-9-enoate | HMDB0242470 | 1.3676 | <0.0001 | up |
| Saxitoxin | HMDB0029368 | 1.6781 | <0.0001 | down |
| Psychosine sulfate | HMDB0013046 | 2.12 | <0.0001 | up |
| Cinobufagin | HMDB0250266 | 1.4802 | <0.0001 | down |
| Cycloheximide | HMDB0250657 | 1.5249 | <0.0001 | up |
| Polygonal | HMDB0256674 | 1.0704 | <0.0001 | down |
| LysoPA(18:2(9Z,12Z)/0:0) | HMDB0007856 | 1.9449 | <0.0001 | up |
| Prenalterol | HMDB0256762 | 1.931 | <0.0001 | up |
| O-Glutarylcarnitine | LMFA07070091 | 1.5189 | <0.0001 | down |
| LysoPC(18:4(6Z,9Z,12Z,15Z)/0:0) | HMDB0010389 | 2.7895 | <0.0001 | up |
| Dihomo-alpha-linolenic acid | HMDB0060039 | 1.3982 | <0.0001 | up |
| PI(PGF2alpha/22:2(13Z,16Z)) | HMDB0277722 | 1.7606 | <0.0001 | up |
| Val Asp Ile | - | 2.4369 | <0.0001 | up |
| LysoPA(0:0/18:1(9Z)) | HMDB0007851 | 1.7871 | <0.0001 | up |
| 1-Arachidonoylglycerol | HMDB0243830 | 1.1496 | <0.0001 | up |
| 2-Lysophosphatidylcholine | HMDB0258493 | 1.743 | <0.0001 | up |
| Gly Val Leu | - | 2.5386 | <0.0001 | up |
| 8-Hydroxyquinoline | - | 1.2393 | <0.0001 | up |
| L-Serine | PW_C000120 | 1.554 | <0.0001 | down |
| Hydroxyprolyl-Valine | HMDB0028876 | 1.3135 | <0.0001 | up |
| 1-Palmitoylphosphatidylcholine | HMDB0256091 | 1.2871 | <0.0001 | up |
| Valtrate | HMDB0034493 | 1.2954 | <0.0001 | up |
| LysoPC(20:2(11Z,14Z)/0:0) | HMDB0010392 | 1.9291 | <0.0001 | up |
| LysoPC(0:0/18:2(9Z,12Z)) | HMDB0061700 | 1.8652 | <0.0001 | up |
| Leu-Gly-Gly | - | 1.6767 | <0.0001 | up |
| Flumethasone | HMDB0252336 | 1.1762 | <0.0001 | up |
| PE(16:1/0:0) | - | 2.0215 | <0.0001 | up |
| N-Jasmonoylisoleucine | HMDB0029391 | 2.4691 | <0.0001 | up |
| LysoPA(20:2(11Z,14Z)/0:0) | HMDB0114758 | 1.8511 | <0.0001 | up |
| LysoPC(18:0/0:0) | HMDB0010384 | 1.4862 | <0.0001 | up |
| N-lactoyl-Methionine | HMDB0062182 | 1.2414 | <0.0001 | up |
| Falcarindiol | HMDB0244257 | 2.2583 | <0.0001 | up |
| Caryophyllen-beta | HMDB0304653 | 2.3081 | <0.0001 | down |
| 7-Aminoheptanoic acid | HMDB0247233 | 1.2916 | <0.0001 | up |
| DL-Ethionine | HMDB0251518 | 1.5979 | <0.0001 | up |
| LysoPC(18:2(9Z,12Z)/0:0) | HMDB0010386 | 1.4069 | <0.0001 | up |
| LysoPA(0:0/18:0) | HMDB0007850 | 2.0755 | <0.0001 | up |
| 2-[(Tetrahydrofurfuryl)oxy]ethanol | HMDB0252869 | 1.9259 | <0.0001 | up |
| LysoPE(18:0/0:0) | HMDB0011130 | 2.4731 | <0.0001 | up |
| PC(17:0/0:0) | HMDB0012108 | 1.6692 | <0.0001 | up |
| LysoPC(16:1(9Z)/0:0) | HMDB0010383 | 1.8898 | <0.0001 | up |
| LysoPC(22:4(7Z,10Z,13Z,16Z)/0:0) | HMDB0010401 | 1.7585 | <0.0001 | up |
| Hexanoylglycine | HMDB0000701 | 2.9521 | <0.0001 | down |
| Pyroglutamic Acid | PW_C000182 | 1.127 | <0.0001 | down |
| Drostanolone | HMDB0014996 | 1.3006 | <0.0001 | up |
| 2-Hydroxy-p-mentha-1,8-dien-6-one | HMDB0037012 | 0.9815 | <0.0001 | down |
| Buprenorphine | PW_C009368 | 1.9403 | <0.0001 | up |
| LysoPC(18:1(11Z)/0:0) | HMDB0010385 | 1.8578 | <0.0001 | up |
| Threonyllysine | HMDB0029066 | 1.4274 | <0.0001 | up |
| PE(18:1(9Z)/0:0) | LMGP02050004 | 1.9483 | <0.0001 | up |
| Pro-Pro-Pro | HMDB0256689 | 1.4038 | <0.0001 | up |
| Vulgarole | HMDB0035085 | 1.3288 | <0.0001 | down |
| Hex-3-enedioylcarnitine | HMDB0241671 | 2.1335 | <0.0001 | down |
| PC(18:1(9Z)e/2:0) | HMDB0011148 | 2.4791 | <0.0001 | up |
| 17-AAG | HMDB0244762 | 1.6716 | <0.0001 | up |
| PC(17:1/0:0) | - | 2.0149 | <0.0001 | up |
| Contignasterol | HMDB0250439 | 2.0952 | <0.0001 | up |
| LysoPC(20:1(11Z)/0:0) | HMDB0010391 | 2.9981 | <0.0001 | up |
| LysoPC(14:0/0:0) | HMDB0010379 | 1.9715 | <0.0001 | up |
| Choline Phosphate | HMDB0001565 | 1.4029 | <0.0001 | up |
| LysoPE(22:6(4Z,7Z,10Z,13Z,16Z,19Z)/0:0) | HMDB0011526 | 1.3861 | <0.0001 | up |
| LysoPE(0:0/20:0) | HMDB0011481 | 3.0428 | <0.0001 | up |
| LysoPC(0:0/16:0) | HMDB0240262 | 1.8025 | <0.0001 | up |
| LysoPA(18:1(9Z)/0:0) | HMDB0007855 | 2.8142 | <0.0001 | up |
| PC(15:0/0:0) | LMGP01050016 | 1.8666 | <0.0001 | up |
| PC(17:2(9Z,12Z)/0:0) | LMGP01050127 | 2.3608 | <0.0001 | up |
| PC(22:6/0:0) | LMGP01050056 | 1.4831 | <0.0001 | up |
| Ethyl-4-hydroxymethyl-3(2H)-Furanone | - | 2.1929 | <0.0001 | down |
| Benzoin | HMDB0032039 | 1.539 | <0.0001 | down |

VIP: variable importance on projection.
