# Supplementary material for: Multi-time point transcriptomics and metabolomics reveal key transcription and metabolic features of hepatic ischemia-reperfusion injury in mice
Source: Genes Dis. 2024 Nov 17;12(2):101465. doi: 10.1016/j.gendis.2024.101465 (PMC11697123; doi:10.1016/j.gendis.2024.101465)
Supplement: Multimedia component 9 [file mmc9.docx]

**Table S5A.** The KEGG pathway of differentially expressed metabolites (DEMs) identified by Kyoto Encyclopedia of Genes and Genomes (KEGG) in the Sham and I1R12 groups.

| **Pathway ID** | **Pathway description** | **P-value** |
| --- | --- | --- |
| map04150 | mTOR signaling pathway | 0.0648 |
| map04151 | PI3K-Akt signaling pathway | 0.0648 |
| map04068 | FoxO signaling pathway | 0.0803 |
| map04071 | Sphingolipid signaling pathway | 0.2223 |
| map04152 | AMPK signaling pathway | 0.3086 |
| map04024 | cAMP signaling pathway | 0.3426 |
| map04022 | cGMP-PKG signaling pathway | 0.0112 |
| map02010 | ABC transporters | 0.6741 |
| map04080 | Neuroactive ligand-receptor interaction | 0.0104 |
| map05310 | Asthma | 0.0803 |
| map05032 | Morphine addiction | 0.1254 |
| map04931 | Insulin resistance | 0.2728 |
| map05012 | Parkinson disease | 0.3536 |
| map05022 | Pathways of neurodegeneration - multiple diseases | 0.4157 |
| map05415 | Diabetic cardiomyopathy | 0.4808 |
| map05215 | Prostate cancer | 0.0136 |
| map04934 | Cushing syndrome | 0.0188 |
| map05200 | Pathways in cancer | 0.0928 |
| map05230 | Central carbon metabolism in cancer | 0.1249 |
| map01523 | Antifolate resistance | 0.0025 |
| map05231 | Choline metabolism in cancer | 0 |
| map00670 | One carbon pool by folate | 0.1399 |
| map00785 | Lipoic acid metabolism | 0.1957 |
| map00020 | Citrate cycle (TCA cycle) | 0.285 |
| map00232 | Caffeine metabolism | 0.3086 |
| map00600 | Sphingolipid metabolism | 0.3644 |
| map00920 | Sulfur metabolism | 0.4255 |
| map00410 | beta-Alanine metabolism | 0.4157 |
| map00400 | Phenylalanine, tyrosine and tryptophan biosynthesis | 0.4351 |
| map00561 | Glycerolipid metabolism | 0.472 |
| map00052 | Galactose metabolism | 0.5387 |
| map00260 | Glycine, serine and threonine metabolism | 0.5541 |
| map00061 | Fatty acid biosynthesis | 0.6235 |
| map00630 | Glyoxylate and dicarboxylate metabolism | 0.6482 |
| map00100 | Steroid biosynthesis | 0.6171 |
| map00440 | Phosphonate and phosphinate metabolism | 0.6106 |
| map00240 | Pyrimidine metabolism | 0.66 |
| map00982 | Drug metabolism - cytochrome P450 | 0.7702 |
| map00980 | Metabolism of xenobiotics by cytochrome P450 | 0.8717 |
| map00220 | Arginine biosynthesis | 0.0549 |
| map00780 | Biotin metabolism | 0.0827 |
| map00620 | Pyruvate metabolism | 0.098 |
| map00830 | Retinol metabolism | 0.0638 |
| map00010 | Glycolysis / Gluconeogenesis | 0.0928 |
| map00120 | Primary bile acid biosynthesis | 0.1829 |
| map00270 | Cysteine and methionine metabolism | 0.2997 |
| map00330 | Arginine and proline metabolism | 0.3182 |
| map00250 | Alanine, aspartate and glutamate metabolism | 0.375 |
| map00380 | Tryptophan metabolism | 0.4026 |
| map00520 | Amino sugar and nucleotide sugar metabolism | 0.5883 |
| map00040 | Pentose and glucuronate interconversions | 0.6235 |
| map00053 | Ascorbate and aldarate metabolism | 0.6171 |
| map00860 | Porphyrin metabolism | 0.7113 |
| map01250 | Biosynthesis of nucleotide sugars | 0.8524 |
| map00565 | Ether lipid metabolism | 0.0078 |
| map00430 | Taurine and hypotaurine metabolism | 0.0069 |
| map00770 | Pantothenate and CoA biosynthesis | 0.0129 |
| map00310 | Lysine degradation | 0.0493 |
| map00360 | Phenylalanine metabolism | 0.0469 |
| map00030 | Pentose phosphate pathway | 0.1139 |
| map01040 | Biosynthesis of unsaturated fatty acids | 0.1237 |
| map00071 | Fatty acid degradation | 0.5689 |
| map00140 | Steroid hormone biosynthesis | 0.081 |
| map00591 | Linoleic acid metabolism | 0.0778 |
| map00592 | alpha-Linolenic acid metabolism | 0.165 |
| map01232 | Nucleotide metabolism | 0.0025 |
| map00590 | Arachidonic acid metabolism | 0.0014 |
| map00230 | Purine metabolism | 0.0013 |
| map01240 | Biosynthesis of cofactors | 0.0182 |
| map00564 | Glycerophospholipid metabolism | 0.0134 |
| map04960 | Aldosterone-regulated sodium reabsorption | 0.1254 |
| map04744 | Phototransduction | 0.1254 |
| map04211 | Longevity regulating pathway | 0.1254 |
| map04928 | Parathyroid hormone synthesis, secretion and action | 0.1542 |
| map04923 | Regulation of lipolysis in adipocytes | 0.2091 |
| map04924 | Renin secretion | 0.248 |
| map04964 | Proximal tubule bicarbonate reclamation | 0.248 |
| map04714 | Thermogenesis | 0.3201 |
| map04913 | Ovarian steroidogenesis | 0.3315 |
| map04977 | Vitamin digestion and absorption | 0.4808 |
| map04740 | Olfactory transduction | 0.0071 |
| map04927 | Cortisol synthesis and secretion | 0.0161 |
| map04925 | Aldosterone synthesis and secretion | 0.0507 |
| map04922 | Glucagon signaling pathway | 0.0683 |
| map04742 | Taste transduction | 0.098 |
| map04726 | Serotonergic synapse | 0.0317 |
| map04723 | Retrograde endocannabinoid signaling | 0.2728 |
| map04976 | Bile secretion | 0.0764 |

**Table S5B.** The KEGG pathway of differentially expressed metabolites (DEMs) identified by Kyoto Encyclopedia of Genes and Genomes (KEGG) in the Sham and I1R24 groups.

| **Pathway ID** | **Pathway description** | **P-value** |
| --- | --- | --- |
| map04217 | Necroptosis | 0.1717 |
| map04151 | PI3K-Akt signaling pathway | 0.0725 |
| map04150 | mTOR signaling pathway | 0.0725 |
| map04068 | FoxO signaling pathway | 0.0898 |
| map04022 | cGMP-PKG signaling pathway | 0.1717 |
| map04152 | AMPK signaling pathway | 0.3396 |
| map04024 | cAMP signaling pathway | 0.3761 |
| map04080 | Neuroactive ligand-receptor interaction | 0.2527 |
| map04071 | Sphingolipid signaling pathway | 0.0024 |
| map02010 | ABC transporters | 0.4782 |
| map00970 | Aminoacyl-tRNA biosynthesis | 0.6263 |
| map05310 | Asthma | 0.0898 |
| map05020 | Prion disease | 0.0898 |
| map05032 | Morphine addiction | 0.1399 |
| map04931 | Insulin resistance | 0.3011 |
| map05415 | Diabetic cardiomyopathy | 0.5215 |
| map05215 | Prostate cancer | 0.0169 |
| map04934 | Cushing syndrome | 0.0234 |
| map05012 | Parkinson disease | 0.0836 |
| map05200 | Pathways in cancer | 0.1128 |
| map05022 | Pathways of neurodegeneration - multiple diseases | 0.119 |
| map01523 | Antifolate resistance | 0.0389 |
| map05230 | Central carbon metabolism in cancer | 0.0046 |
| map05231 | Choline metabolism in cancer | 0 |
| map00563 | Glycosylphosphatidylinositol (GPI)-anchor biosynthesis | 0.1068 |
| map00785 | Lipoic acid metabolism | 0.2173 |
| map00190 | Oxidative phosphorylation | 0.2604 |
| map00232 | Caffeine metabolism | 0.3396 |
| map00220 | Arginine biosynthesis | 0.352 |
| map00740 | Riboflavin metabolism | 0.3642 |
| map00750 | Vitamin B6 metabolism | 0.4216 |
| map00770 | Pantothenate and CoA biosynthesis | 0.4324 |
| map00620 | Pyruvate metabolism | 0.4535 |
| map00920 | Sulfur metabolism | 0.4638 |
| map00561 | Glycerolipid metabolism | 0.5123 |
| map00120 | Primary bile acid biosynthesis | 0.589 |
| map00360 | Phenylalanine metabolism | 0.6043 |
| map00440 | Phosphonate and phosphinate metabolism | 0.6537 |
| map00061 | Fatty acid biosynthesis | 0.6666 |
| map00470 | D-Amino acid metabolism | 0.7139 |
| map00330 | Arginine and proline metabolism | 0.7298 |
| map00380 | Tryptophan metabolism | 0.7933 |
| map00020 | Citrate cycle (TCA cycle) | 0.0525 |
| map00430 | Taurine and hypotaurine metabolism | 0.0727 |
| map00780 | Biotin metabolism | 0.1009 |
| map00010 | Glycolysis / Gluconeogenesis | 0.1128 |
| map00400 | Phenylalanine, tyrosine and tryptophan biosynthesis | 0.1315 |
| map00670 | One carbon pool by folate | 0.1559 |
| map00260 | Glycine, serine and threonine metabolism | 0.225 |
| map00053 | Ascorbate and aldarate metabolism | 0.2875 |
| map01040 | Biosynthesis of unsaturated fatty acids | 0.4033 |
| map00982 | Drug metabolism - cytochrome P450 | 0.4859 |
| map00520 | Amino sugar and nucleotide sugar metabolism | 0.652 |
| map00860 | Porphyrin metabolism | 0.7699 |
| map01250 | Biosynthesis of nucleotide sugars | 0.8942 |
| map00600 | Sphingolipid metabolism | 0.0132 |
| map00830 | Retinol metabolism | 0.0107 |
| map00565 | Ether lipid metabolism | 0.0107 |
| map00592 | alpha-Linolenic acid metabolism | 0.0478 |
| map00310 | Lysine degradation | 0.0655 |
| map00250 | Alanine, aspartate and glutamate metabolism | 0.095 |
| map00630 | Glyoxylate and dicarboxylate metabolism | 0.1081 |
| map00270 | Cysteine and methionine metabolism | 0.1242 |
| map00071 | Fatty acid degradation | 0.6118 |
| map00030 | Pentose phosphate pathway | 0.0266 |
| map00590 | Arachidonic acid metabolism | 0.0503 |
| map00980 | Metabolism of xenobiotics by cytochrome P450 | 0.1881 |
| map01232 | Nucleotide metabolism | 0.0222 |
| map00140 | Steroid hormone biosynthesis | 0.0098 |
| map00591 | Linoleic acid metabolism | 0 |
| map00230 | Purine metabolism | 0.0005 |
| map01240 | Biosynthesis of cofactors | 0.0394 |
| map00564 | Glycerophospholipid metabolism | 0.0005 |
| map04960 | Aldosterone-regulated sodium reabsorption | 0.1399 |
| map04740 | Olfactory transduction | 0.1399 |
| map04211 | Longevity regulating pathway | 0.1399 |
| map04924 | Renin secretion | 0.2742 |
| map04964 | Proximal tubule bicarbonate reclamation | 0.2742 |
| map04723 | Retrograde endocannabinoid signaling | 0.3011 |
| map04714 | Thermogenesis | 0.352 |
| map04977 | Vitamin digestion and absorption | 0.5215 |
| map04726 | Serotonergic synapse | 0.548 |
| map04974 | Protein digestion and absorption | 0.589 |
| map04928 | Parathyroid hormone synthesis, secretion and action | 0.014 |
| map04927 | Cortisol synthesis and secretion | 0.0201 |
| map04923 | Regulation of lipolysis in adipocytes | 0.027 |
| map04978 | Mineral absorption | 0.1009 |
| map04742 | Taste transduction | 0.119 |
| map03320 | PPAR signaling pathway | 0.0898 |
| map04925 | Aldosterone synthesis and secretion | 0.0074 |
| map04922 | Glucagon signaling pathway | 0.0119 |
| map04976 | Bile secretion | 0.2704 |

**Table S5C.** The KEGG pathway of differentially expressed metabolites (DEMs) identified by Kyoto Encyclopedia of Genes and Genomes (KEGG) in the Sham and I1R48 groups.

| **Pathway ID** | **Pathway description** | **P-value** |
| --- | --- | --- |
| map04210 | Apoptosis | 0.0717 |
| map04216 | Ferroptosis | 0.4177 |
| map04217 | Necroptosis | 0.0007 |
| map04150 | mTOR signaling pathway | 0.0717 |
| map04151 | PI3K-Akt signaling pathway | 0.0717 |
| map04068 | FoxO signaling pathway | 0.0888 |
| map04152 | AMPK signaling pathway | 0.3363 |
| map04024 | cAMP signaling pathway | 0.3724 |
| map04080 | Neuroactive ligand-receptor interaction | 0.6217 |
| map04022 | cGMP-PKG signaling pathway | 0.0137 |
| map04071 | Sphingolipid signaling pathway | 0.0023 |
| map02010 | ABC transporters | 0.4701 |
| map00970 | Aminoacyl-tRNA biosynthesis | 0.6217 |
| map05310 | Asthma | 0.0888 |
| map05140 | Leishmaniasis | 0.1056 |
| map05143 | African trypanosomiasis | 0.1383 |
| map05032 | Morphine addiction | 0.1383 |
| map05146 | Amoebiasis | 0.2149 |
| map04934 | Cushing syndrome | 0.2149 |
| map05225 | Hepatocellular carcinoma | 0.2294 |
| map04931 | Insulin resistance | 0.298 |
| map05200 | Pathways in cancer | 0.4391 |
| map05415 | Diabetic cardiomyopathy | 0.5171 |
| map05012 | Parkinson disease | 0.0819 |
| map05022 | Pathways of neurodegeneration - multiple diseases | 0.1166 |
| map01523 | Antifolate resistance | 0.0034 |
| map05230 | Central carbon metabolism in cancer | 0.0298 |
| map05231 | Choline metabolism in cancer | 0.0009 |
| map00190 | Oxidative phosphorylation | 0.2576 |
| map00232 | Caffeine metabolism | 0.3363 |
| map00830 | Retinol metabolism | 0.3724 |
| map00750 | Vitamin B6 metabolism | 0.4177 |
| map00920 | Sulfur metabolism | 0.4597 |
| map00410 | beta-Alanine metabolism | 0.4495 |
| map00620 | Pyruvate metabolism | 0.4495 |
| map00010 | Glycolysis / Gluconeogenesis | 0.4391 |
| map00480 | Glutathione metabolism | 0.508 |
| map00052 | Galactose metabolism | 0.5766 |
| map00340 | Histidine metabolism | 0.5844 |
| map00310 | Lysine degradation | 0.6072 |
| map00440 | Phosphonate and phosphinate metabolism | 0.6491 |
| map00470 | D-Amino acid metabolism | 0.7094 |
| map00130 | Ubiquinone and other terpenoid-quinone biosynthesis | 0.7356 |
| map00520 | Amino sugar and nucleotide sugar metabolism | 0.8917 |
| map00860 | Porphyrin metabolism | 0.9391 |
| map01250 | Biosynthesis of nucleotide sugars | 0.9777 |
| map00670 | One carbon pool by folate | 0.0111 |
| map00020 | Citrate cycle (TCA cycle) | 0.0514 |
| map00780 | Biotin metabolism | 0.0988 |
| map00770 | Pantothenate and CoA biosynthesis | 0.1046 |
| map00400 | Phenylalanine, tyrosine and tryptophan biosynthesis | 0.1289 |
| map00030 | Pentose phosphate pathway | 0.1351 |
| map00260 | Glycine, serine and threonine metabolism | 0.2209 |
| map00053 | Ascorbate and aldarate metabolism | 0.2827 |
| map00630 | Glyoxylate and dicarboxylate metabolism | 0.317 |
| map00270 | Cysteine and methionine metabolism | 0.3441 |
| map00330 | Arginine and proline metabolism | 0.3643 |
| map00982 | Drug metabolism - cytochrome P450 | 0.4794 |
| map00140 | Steroid hormone biosynthesis | 0.5489 |
| map00565 | Ether lipid metabolism | 0.0103 |
| map00600 | Sphingolipid metabolism | 0.0128 |
| map00360 | Phenylalanine metabolism | 0.0605 |
| map00250 | Alanine, aspartate and glutamate metabolism | 0.0931 |
| map00380 | Tryptophan metabolism | 0.196 |
| map00071 | Fatty acid degradation | 0.6072 |
| map00592 | alpha-Linolenic acid metabolism | 0.0012 |
| map00590 | Arachidonic acid metabolism | 0.0118 |
| map01232 | Nucleotide metabolism | 0.0213 |
| map00230 | Purine metabolism | 0.0024 |
| map01040 | Biosynthesis of unsaturated fatty acids | 0.0001 |
| map00591 | Linoleic acid metabolism | 0 |
| map01240 | Biosynthesis of cofactors | 0.0023 |
| map00564 | Glycerophospholipid metabolism | 0.0034 |
| map04912 | GnRH signaling pathway | 0.1056 |
| map04744 | Phototransduction | 0.1383 |
| map04666 | Fc gamma R-mediated phagocytosis | 0.1383 |
| map04211 | Longevity regulating pathway | 0.1383 |
| map04730 | Long-term depression | 0.1542 |
| map04664 | Fc epsilon RI signaling pathway | 0.1851 |
| map04921 | Oxytocin signaling pathway | 0.2001 |
| map04927 | Cortisol synthesis and secretion | 0.2001 |
| map04611 | Platelet activation | 0.2294 |
| map04924 | Renin secretion | 0.2713 |
| map04964 | Proximal tubule bicarbonate reclamation | 0.2713 |
| map04714 | Thermogenesis | 0.3485 |
| map04913 | Ovarian steroidogenesis | 0.3606 |
| map04750 | Inflammatory mediator regulation of TRP channels | 0.4795 |
| map04974 | Protein digestion and absorption | 0.5844 |
| map03320 | PPAR signaling pathway | 0.0032 |
| map04740 | Olfactory transduction | 0.0087 |
| map04928 | Parathyroid hormone synthesis, secretion and action | 0.0137 |
| map04923 | Regulation of lipolysis in adipocytes | 0.0264 |
| map04270 | Vascular smooth muscle contraction | 0.034 |
| map04723 | Retrograde endocannabinoid signaling | 0.0468 |
| map04925 | Aldosterone synthesis and secretion | 0.061 |
| map04922 | Glucagon signaling pathway | 0.0819 |
| map04978 | Mineral absorption | 0.0988 |
| map04977 | Vitamin digestion and absorption | 0.1607 |
| map04726 | Serotonergic synapse | 0.1805 |
| map04976 | Bile secretion | 0.5378 |
| map04742 | Taste transduction | 0.0203 |
